# AmpC β-lactamases: A key to antibiotic resistance in ESKAPE pathogens

**DOI:** 10.1016/j.tcsw.2025.100154

**Published:** 2025-09-22

**Authors:** Deeksha Pandey, Isha Gupta, Dinesh Gupta

**Affiliations:** a Translational Bioinformatics Group, International Centre for Genetic Engineering and Biotechnology (ICGEB), New Delhi, India; b Current Affiliation: Department of Biotechnology, Center of Excellence in Emerging Diseases, Jaypee Institute of Information Technology, Sector 62, Noida, Uttar Pradesh, India

**Keywords:** Plasmid, Chromosome, Cephalosporinase, Nosocomial infections, Antimicrobial resistance

## Abstract

**Background:**

AmpC β-lactamases (*blaAmpC*) are essential drivers of antimicrobial resistance (AMR) in ESKAPE pathogens, bacteria that cause hospital-acquired infections. Understanding AmpC enzymes is essential for uncovering resistance mechanisms and guiding antimicrobial strategies. We analyzed *blaAmpC* presence, genomic location, copy number, sequence variability, and evolutionary traits in ESKAPE pathogens.

**Results:**

We identified 1790 AmpC enzymes in 4713 complete genomes, classified into nine enzyme groups. Consistent with known taxonomic profiles, no class C β-lactamases were detected in Gram-positive bacteria (*Staphylococcus aureus* and *Enterococcus faecium*). *Acinetobacter baumannii* exhibited the highest occurrence of class C β-lactamases, with *Enterobacter* spp. showing the second highest prevalence, followed by *Pseudomonas aeruginosa* and *Klebsiella pneumoniae*. The largest enzyme group, ADC was restricted to *A. baumannii*; similarly, ACC, ACT, CMH, and MIR to *Enterobacter* spp.; and PDC and PIB to *P. aeruginosa.* Phylogenetic analysis showed divergence among some groups and closer evolutionary relationships in others. Functional Motif analysis revealed conserved catalytic residues across all groups except PIB. Instead of the canonical YXN and KTG motifs, PIB contains YST and AQG variants, respectively. Because of these variations, PIB's ability to bind cephalosporins decreases while enhancing their activity against carbapenems.

**Conclusions:**

We identified 1790 AmpC enzymes in nine distinct groups across ESKAPE pathogens, with species-specific distribution patterns and notable absence in Gram-positive bacteria. The PIB enzyme group demonstrated unique motif variants (YST/AQG) conferring carbapenem resistance, while other groups maintained conserved catalytic motifs. Phylogenetic analysis revealed evolutionary divergence and horizontal gene transfer potential, emphasizing the need for targeted therapeutic approaches against AmpC-mediated resistance.

## Highlights

• Presence, diversity, & evolution of AmpC β-lactamases in ESKAPE pathogens.
• 1790 AmpC enzymes found in 4713 ESKAPE genomes, classified into 9 groups.
• AmpC is absent in Gram-positive ESKAPE; highly prevalent in Gram-negative pathogens.
• Conserved motifs SXSK, YXN, KTG in 8 AmpC groups; PIB had variants SXXK, YST, AQG.
• Mutations in conserved AmpC motifs are deleterious, critical for enzyme function.

## 1 Background

The World Health Organization identifies Antimicrobial Resistance (AMR) as one of the top ten global public health emergencies. The growing challenge of multidrug-resistant (MDR) and extensively drug-resistant (XDR) strains of Enterobacteriaceae and Acinetobacter species is a serious problem for humans and animals. The World Health Organization identified ESKAPE pathogens (*Enterococcus faecium*, *Staphylococcus aureus*, *Klebsiella pneumoniae*, *Acinetobacter baumannii*, *Pseudomonas aeruginosa*, and *Enterobacter* spp.) as the most challenging antibiotic-resistant nosocomial pathogens (World Health Organization, 2024; Chen et al., 2025; Antimicrobial Resistance Collaborators, 2022). These infections carry a high disease burden, increased in-hospital mortality, and dwindling treatment options. In 2019, AMR caused approximately 4.95 million deaths worldwide, of which ESKAPE pathogens accounted for over 1.2 million (World Health Organization, 2024). Many different genetic factors drive the resistance exhibited by ESKAPE organisms, such as point mutations, gene amplification, and horizontal gene transfer (Sommer et al., 2017). These pathogens resist antibiotics through enzymatic degradation, efflux pumps, target modification, and biofilm formation (Pandey et al., 2021).

β-lactamase production is the predominant resistance mechanism against β-lactam antibiotics (penicillins, cephalosporins, monobactams, and carbapenems), the foundation of modern antimicrobial therapy (Bush and Jacoby, 2010). Bacterial β-lactamase production enables antibiotic evasion, posing a significant threat to global public health. Some bacteria carry β-lactamases on plasmids as mobile genetic elements, while chromosomes encode others. β-lactam antibiotics have been essential for treating bacterial infections (Williams, 1999). However, bacteria produce β-lactamases that inactivate these antibiotics (Fernandes et al., 2013). Based on the Ambler classification system, β-lactamases are divided into four molecular classes: Class A (e.g., TEM, SHV, KPC), Class B (metallo-β-lactamases such as NDM), Class C (AmpC β-lactamases), and Class D (OXA-type enzymes, including narrow-spectrum enzymes like OXA-1 and carbapenemases such as OXA-48) (Bush and Jacoby, 2010). AmpC β-lactamases are particularly concerning because they hydrolyze cephalosporins and penicillins and resist most inhibitors (Ronni Mol et al., 2022). Inducible expression and horizontal gene transfer contribute to resistance mechanisms in clinical isolates. Either plasmids or chromosomes encode these enzymes (Anitha et al., 2015). This study aims to comprehensively characterize AmpC β-lactamase diversity in ESKAPE pathogens through genomic analysis. Specific objectives include: (1) identifying and classifying AmpC enzymes into homologous groups based on sequence similarity, (2) analyzing phylogenetic relationships and evolutionary divergence among enzyme groups, (3) investigating chromosomal versus plasmid distribution patterns across pathogen species, (4) examining structural variations and conserved catalytic motifs, and (5) predicting functional impacts of sequence variants to understand resistance mechanisms and inform targeted therapeutic strategies.

## 2 Methods

### 2.1 Data collection

We downloaded ESKAPE pathogen genomes (*E. faecium*, *S. aureus*, *K. pneumoniae*, *A. baumannii*, *P. aeruginosa*, and Enterobacter spp.) from the NCBI genome database (September 21, 2023). We retrieved 3460, 15,739, 17,608, 8037, 8221, and 4970 genomes for *E. faecium*, *S. aureus*, *K. pneumoniae*, *A. baumannii*, *P. aeruginosa*, and *Enterobacter* spp., respectively. We excluded incomplete genome assemblies to ensure high-quality, contiguous sequences preserving complete *blaAmpC* genes with surrounding genomic context, including regulatory elements and mobile genetic components. Draft or fragmented assemblies often contain gaps or mis-assemblies that can hinder accurate detection, localization, and classification of AmpC enzyme groups. Table 1**a & b** provide basic statistics of the genomic and replicon datasets used in the study. Depending on the nature of the replicons in the genome, we classified each genome into two categories: (a) those with only chromosomes and (b) those with both chromosomes and plasmids. Subsequently, each replicon was searched for *blaAmpC*.

**Table 1:** Genomic Data Statistics and replicon-wise distribution of *blaAmpC*  in ESKAPE Pathogens.

a) Genomic Data distribution among ESKAPE Pathogens.						
Organism	Total no. of genomes (NCBI)	No. of complete genomes	Replicons			
			No. of chromosomal genomes	Both Chromosome & Plasmid		
				No. of genomes containing both chromosomes & plasmids	No. of chromosomes	No. of plasmids
*E. faecium*	3260	318	14	304	304	1420
*S. aureus*	15,739	1022	454	568	568	841
*K. pneumoniae*	17,608	1661	110	1551	1551	5594
*A. baumannii*	8037	579	122	457	457	923
*P. aeruginosa*	8221	624	497	127	127	188
*Enterobacter* spp.	4970	509	98	411	411	1295
**Total**	**57,835**	**4713**	**1295**	**3418**	**3418**	**10,261**


b) Statistics of replicon-wise *blaAmpC* Gene distribution across ESKAPE Pathogens.					
Organism	No. of *blaAmpC* present in complete genomes	Replicons			
		No. of *blaAmpC* present in chromosomal genomes	Both Chromosome & Plasmid		
			No. of *blaAmpC* present in both chromosomes & plasmids	No. of *blaAmpC* present in chromosomes	No. of *blaAmpC* present in plasmids
*K. pneumoniae*	175	1	11	11	152
*A. baumannii*	755	97	330	327	1
*P. aeruginosa*	401	250	75	75	1
*Enterobacter* spp.	459	19	207	233	0
**Total**	**1790**	**367**	**623**	**646**	**154**

### 2.2 Searching for *blaAmpC* in ESKAPE sequences

The β-LacFampred tool retrieved the profile Hidden Markov Models (pHMM) of *blaAmpC*, which uses family-specific profile HMMs trained on >8000 curated β-lactamase sequences from CBMAR, BLDB, CARD, UniProtKB, and NCBI NR (96 families, ≥5 sequences each), built with MUSCLE alignments and HMMER3 with LOOCV validation, achieving ≥98 % precision and recall (Pandey et al., 2022). Each replicon was searched against the pHMM of *blaAmpC* using the NHMMSCAN program of the HMMER package (version 3.1) (Finn et al., 2011) with an *E*-value threshold of 1e-06. The NHMMSCAN search results were validated through manual curation using GenBank annotations of the corresponding genomes to retain only the verified *blaAmpC* and eliminate non-*blaAmpC* sequences. Each identified sequence was individually examined to confirm its annotation as an AmpC β-lactamase, and borderline hits near the *E*-value threshold (1e-06) were manually reviewed to ensure accurate classification. Afterwards, the protein sequences of verified AmpC were retrieved from the NCBI protein database.

### 2.3 Distribution of AmpC into enzyme groups

We obtained Class C β-lactamase protein sequences from BLDB (Beta-Lactamase DataBase), a comprehensive, manually curated resource (Naas et al., 2017). A comprehensive BLAST search identified AmpC sequences across ESKAPE pathogens. We retained only sequences with 100% identity to ensure data reliability and focus on well-characterized AmpC variants, avoiding non-AmpC sequences or those with ambiguous classifications. This stringent criterion allowed for a precise analysis of known enzyme groups present in the dataset.

### 2.4 Multiple sequence alignment and phylogenetic analysis

Multiple Sequence Alignment (MSA) was done using the MAFFT version 7 (Katoh and Standley, 2013) and visualized using Jalview Version 2 (Waterhouse et al., 2009). We used a Neighbor-Joining (NJ) method followed by 1000 bootstrap values for robust tree construction. We visualized the phylogenetic tree using Microreact (Argimón et al., 2016), a web-based application to investigate the evolutionary patterns of AmpC across ESKAPE pathogens.

### 2.5 Assessment of pairwise sequence identity in AmpC enzyme group representatives and construction of phylogenetic trees

To compare amino acid sequences and construct the phylogenetic tree we selected a representative sequence for each enzyme group as the first sequence in the MSA after sorting sequences by completeness and annotation quality. The pairwise sequence identity of the nine AmpC enzyme group representatives was calculated to determine their evolutionary relationships. Phylogenetic analysis of representative AmpC enzyme sequences was conducted using MEGA12 version 12.0.11 (Kumar et al., 2024). The sequences were aligned with the MUSCLE algorithm. A NJtree was inferred under the Poisson substitution model with uniform rates among sites, using pairwise deletion for gaps. Tree topology support was assessed by 1000 bootstrap replicates. RelTime-ML was applied in MEGA12 to estimate divergence times, using PIB as an outgroup. Calibration constraints from TimeTree (http://www.timetree.org/) (Kumar et al., 2022) were imported as uniform priors. Analytical variance estimation was used for node age confidence.

### 2.6 *In-silico* prediction of functional effects of variants

An *In-silico* analysis using PROVEAN was performed to evaluate the possible functional impact of sequence variations. From each enzyme group, we chose one representative sequence and determined the variations present within the corresponding enzyme intra-group. The software calculates the effects of certain changes on protein functionality based on a scoring system where −2.5 is used as a threshold, above −2.5 as neutral, and scores below or equal to −2.5 as deleterious (damaging).

### 2.7 3D structural variations in the AmpC enzyme groups

The structural analysis of AmpC enzyme groups was performed using experimentally determined 3D protein structures retrieved from the PDB. For each of the eight groups (ADC, ACC, PDC, DHA, CMY, CMH, ACT, and MIR), a representative structure was selected based on high sequence identity (>60 %) and query coverage (>92 %) to the respective enzyme group members. The protein name, PDB ID, query coverage, and group members' identities are shown in Table 2. The superimposition of these protein models was performed using PyMOL Schrödinger. The primary quantitative parameter to assess structural similarity was the root mean square deviation (RMSD), which measures the average distance between the backbone atoms of superimposed structures.

**Table 2:** PDB IDs of the structural representatives of the nine AmpC enzyme groups with query coverage and identity percentage.

S. No.	Protein Name	PDB ID	Query Coverage (%)	Identity (%)
1	ADC	8FQV	93 %	99.72 %
2	ACC	6K8X	93 %	100.00 %
3	PDC	6S1S	100 %	100.00 %
4	DHA	5GGW	92 %	61.25 %
5	CMY	1ZC2	94 %	99.72 %
6	CMH	6LC7	94 %	97.51 %
7	ACT	7TI1	100 %	86.09 %
8	MIR	2ZC7	94 %	93.59 %

## 3 Results and discussion

### 3.1 Data analysis and ESKAPE pathogen classification

The NCBI genome database contained 57,835 total entries across six ESKAPE pathogens. Of these, 318, 1022, 1661, 579, 624, and 509 entries corresponded to complete genomes of ESKAPE pathogens*,* respectively (Table 1**a**). Genome composition varied significantly among species, with most containing chromosomal and plasmid elements (Table 1**b**).

The quantity of plasmids found in the ESKAPE pathogens showed a notable pattern. Plasmid counts ranged from one per genome in *S. aureus*/*P. aeruginosa* to four in *Enterobacter* spp., reflecting inter-species variability, presented in Table 3. Botelho et al. *(*Botelho et al., *2023**)* also showed the same pattern that ESKAPE pathogens have a considerable fraction of plasmids encoding a significant percent of AMR, where over 35 % of plasmids across the different ESKAPE species had at least one AMR gene.

**Table 3:** Summary of AmpC Distribution Pattern in ESKAPE Pathogens.

Organism	Avg. Plasmids/Genome	AmpC Distribution Pattern	Predominant Location
*E. faecium*	∼4	None detected	N/A
*S. aureus*	∼1	None detected	N/A
*K. pneumoniae*	∼4	93.3 % plasmid, 6.7 % chromosome	Plasmid
*A. baumannii*	∼2	99.9 % chromosome, 0.1 % plasmid	Chromosome (∼1.3 copies/genome)
*P. aeruginosa*	∼1	99.2 % chromosome, 0.8 % plasmid	Chromosome (∼0.6 copies/genome)
*Enterobacter* spp.	∼4	100 % chromosome, 0 % plasmid	Chromosome

### 3.2 Identification and filtration of AmpC sequences in ESKAPE pathogens

A profile HMM (pHMM) of *blaAmpC* was retrieved from the β-lacFampred tool, previously developed by us (Pandey et al., 2022). pHMM profile of *blaAmpC* was searched against each of the six pathogens using the NHMMSCAN tool of the HMMER package (Finn et al., 2011). Subsequent re-validation of the NHMMSCAN outputs against the GenBank annotations for the corresponding genomes did not identify any unannotated or additional *blaAmpC* sequences, indicating that the pHMM of *blaAmpC* showed 100 % specificity for the AmpC family of class C β-lactamases. Using the Beta-Lactamase DataBase (BLDB), a specialized repository developed by Naas et al. (Naas et al., 2017), 3781 AmpC protein sequences of Class C β-lactamases were extracted for annotation of *blaAmpC* genes present in ESKAPE pathogens. A comprehensive BLAST search was performed across ESKAPE pathogen against AmpC sequences. We applied a 100 % identity threshold to remove redundancy and retain only high-confidence, full-length AmpC sequences. This approach ensured accurate classification and comparison of canonical variants while avoiding ambiguity from partial or highly divergent sequences. This filtering step was particularly critical for downstream phylogenetic and functional analyses at the variant level. After removing duplicates and sequences with less than 100 % identity, the final dataset comprised 1790 unique AmpC sequences.

### 3.3 Dissemination and localization of AmpC in ESKAPE pathogens

We identified 1790 AmpC proteins in 4713 assembled ESKAPE pathogen genomes **(**Table 1**)**. The absence of *blaAmpC* in *E. faecium* and *S. aureus* reflects that *blaAmpC* reservoirs are typically Gram-negative species (El Shamy et al., 2021; Bush and Bradford, 2020).

*Enterobacter* spp. and *P. aeruginosa* genomes contained 459 and 401 AmpC sequences, respectively. *blaAmpC* was found on both chromosomes and plasmids in 75 *P. aeruginosa* and 207 *Enterobacter* spp. The average number of plasmids per genome varied among pathogens, with *K. pneumoniae* having the highest count, containing about 152 plasmids carrying AmpC sequences. *P. aeruginosa* and *Enterobacter* spp. naturally harbor chromosomal *blaAmpC* genes, and therefore, their detection in these organisms is expected. We intended to report the presence and diversity of *blaAmpC* genes across species, including those where chromosomal carriage is typical.

Particular ESKAPE species, such as *Enterobacter species*, *P. aeruginosa*, and *A. baumannii*, are known to be natural reservoirs of chromosomal AmpC enzymes. These enzymes are usually produced at low levels and do not provide clinical resistance until subjected to overexpression. (Davin-Regli et al., 2019; Howard et al., 2012; Mirsalehian et al., 2014). Acquired *blaAmpC* genes, which are often located on plasmids, are more regulated and pose a clinical threat because of their association with multidrug resistance and horizontal gene transfer. Therefore, distinguishing the identified enzyme groups of AmpC into natural or acquired variants (**Table S1**) aids understanding of their clinical significance.

**Fig. 1:**
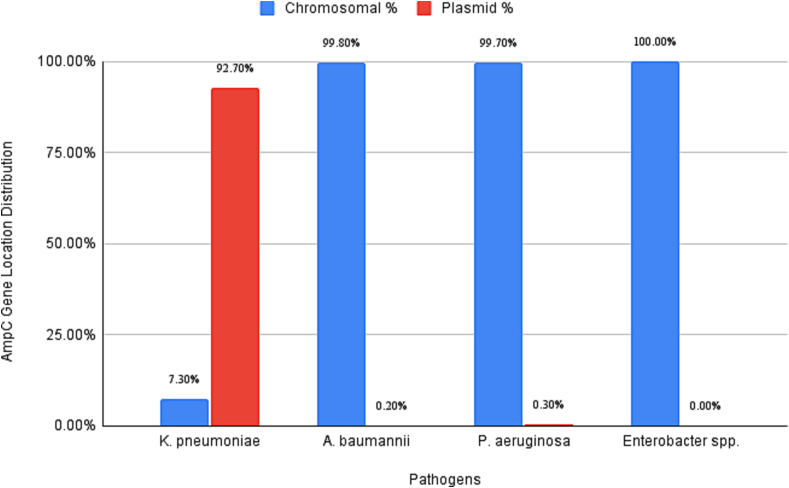
Distribution of *blaAmpC * Genes on Chromosomes and Plasmids Across ESKAPE Pathogens.

In *K. pneumoniae*, *blaAmpC* was detected predominantly on plasmids (152/163, 93.3 %) compared to chromosomes (11/163, 6.7 %), consistent with horizontal gene transfer as the primary acquisition mechanism in this species, as represented in Fig. 1.

In *A. baumannii*, 97 *blaAmpC* sequences were observed in 122 chromosomal genome assemblies. Furthermore, 327 *blaAmpC* were found on chromosomes, and 1 on plasmids in 457 genomes containing both chromosomes and plasmids, indicating a higher prevalence of *blaAmpC* on chromosomes (16 %) compared to plasmids (0.1 %), and more than one copy of *blaAmpC* might be present on chromosomes of *A. baumannii* strains.

For *P. aeruginosa*, 250 *blaAmpC* sequences were observed in 497 complete chromosomal genome assemblies, whereas 75 *blaAmpC* were found on chromosomes and 1 on plasmids across 127 genomes containing both chromosomes and plasmids. This indicates that *blaAmpC* is predominantly localized on chromosomes (61 %) rather than plasmids (0.8 %) in *P. aeruginosa*.

*Enterobacter* spp. revealed the presence of *blaAmpC* sequences in 19 out of 98 chromosomal genomes. In addition, 411 genomes with both chromosomes and plasmids showed 233 *blaAmpC* on the chromosomes and none on the plasmids. This clearly showed that *blaAmpC* was more prevalent on chromosomes (10 %) than on plasmids (0 %) in *Enterobacter* spp.

AmpC distribution and localization varied across ESKAPE pathogens. *blaAmpC* was absent in the *E. faecium* and *S. aureus* genomes but present in *Enterobacter* spp. genomes in lower frequency. *blaAmpC* showed chromosomal prevalence in *A. baumannii* (∼1.3 copies per genome) and *P. aeruginosa* (∼0.6 copies per genome), with lower but notable presence in *K. pneumoniae* and *Enterobacter* spp. Among ESKAPE pathogens, *A. baumannii* had the highest AmpC prevalence with approximately 1.3 copies per genome, while *P. aeruginosa* and *K. pneumoniae* showed moderate levels. Our findings mirror those of Segal et al. (2004), who demonstrated chromosomal ampC carriage and its transcriptional regulation in *A. baumannii* clinical isolates; Bonomo and Szabo (2006), who reviewed the high prevalence and chromosomal localization of *blaAmpC* in both *A. baumannii* and *P. aeruginosa*; and Kong et al. (2005), who characterized a chromosomally encoded oxacillinase (poxB) in *P. aeruginosa*, underscoring the role of chromosomal β-lactamases in nosocomial resistance (Kong et al., 2005; Bonomo and Szabo, 2006; Segal et al., 2004).

### 3.4 Classification of AmpC into different enzyme groups

1790 AmpC proteins in this study formed nine clusters; hence, the AmpC proteins discerned in ESKAPE pathogens were further classified into nine enzyme groups based on their sequence homology and information provided in the BLDB database (Naas et al., 2017). The comprehensive details about the identified enzyme groups, related variations, and the organisms with their gene location that contain these enzyme groups among ESKAPE pathogens are provided in **Tables S2 & S3**.

ADC was the largest enzyme group (755 proteins), followed by PDC (397 proteins), ACT (377 proteins), DHA enzyme group (135 proteins), CMY enzyme group (54 proteins), MIR enzyme group (40 proteins), CMH enzyme group (16 proteins), ACC enzyme group (13 proteins), and PIB enzyme group (3 proteins). ADC, the largest enzyme group, was exclusively present in *A. baumannii* strains on plasmids and chromosomes (**Table S3**). Therefore, ADC enzymes represent a ubiquitous chromosomal feature of *A. baumannii*. The ADC β-lactamase is regarded as “universally present in the *A. baumannii* chromosome”   (Colquhoun et al., 2023), emphasizing how widespread and conserved it is within this particular species. Our exclusive detection of ADC in *A. baumannii* aligns with Liu and Liu (2015), who found ADC-type AmpC β-lactamases in 72 % of clinical *A. baumannii* isolates from the Xuzhou region, demonstrating ADC's widespread distribution in this species (Liu and Liu, 2015). This finding is further supported by Colquhoun et al. (2023), who characterized ADC-7 β-lactamase expression in *A. baumannii*, confirming ADC as an intrinsic chromosomal feature (Colquhoun et al., 2023). These concordant results across different geographic regions and periods reinforce ADC as a conserved, species-specific resistance determinant in *A. baumannii*. The ACT enzyme group (377 proteins), CMH enzyme group (16 proteins), MIR enzyme group (40 proteins), and ACC enzyme group (13 proteins) were present exclusively in *Enterobacter* spp.*,* as seen in previous studies (Reisbig and Hanson, 2002; Ku et al., 2018; Nepal et al., 2018; Crichlow et al., 1999). PDC enzyme group (397 proteins) and PIB enzyme group (3 proteins) were exclusively present in *P. aeruginosa*. This was also reported in the previous studies (Fajardo et al., 2014; Medrano et al., 2024). The remaining two enzyme groups, such as CMY (54 proteins), were present in *K. pneumoniae* and *P. aeruginosa (*Xu et al., *2022**;* Lahiri et al., *2015**),* while DHA (135 proteins) were present in *K. pneumoniae* and *Enterobacter* spp. (Fortineau et al., 2001; Barnaud et al., 2006).

### 3.5 Evolutionary relatedness among AmpC enzyme groups

**Fig. 2:**
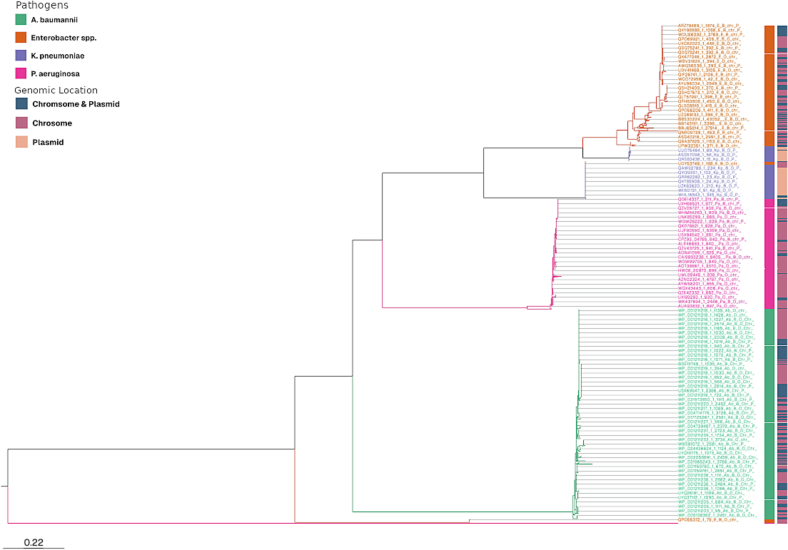
**Phylogram illustrating the evolutionary relationships of 1790 Ambler Class C Beta-lactamase (*blaAmpC*) sequences in the ESKAPE pathogens.** The tree shows the diversity and relationships among these ESKAPE pathogens. Sequences are color-coded to indicate the source pathogen, with each species represented by a distinct color. Additionally, the genetic location of the *blaAmpC* gene (chromosome, plasmid, or both) is distinguished by a separate color scheme. An interactive version of this phylogram is available for a more detailed exploration of specific branches and clades at: https://microreact.org/project/4pADHsN8W8uymhEpJwMHDS-pathogen-n-location.

**Supplementary Figure S1:**
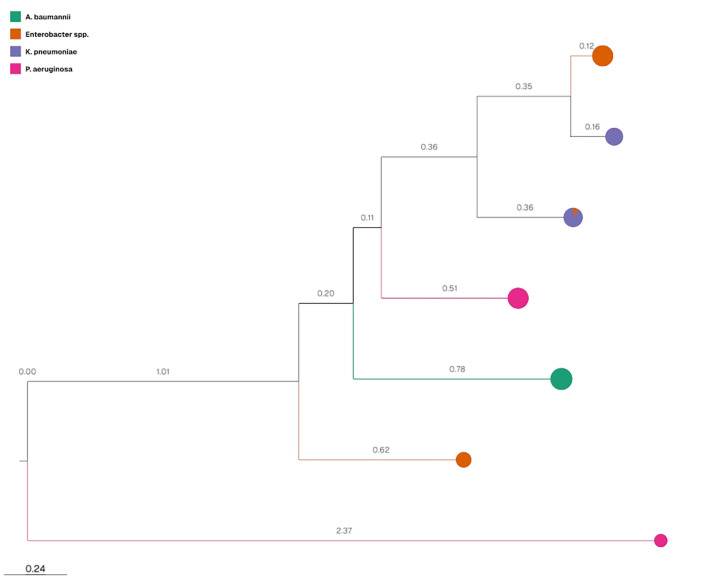
Phylogram of AmpC Beta-lactamase Sequences from ESKAPE Pathogens. This figure represents a condensed view of the full phylogram (Fig. 2), with branches collapsed to show the major evolutionary distance (branch length) between AmpC sequences found in *Acinetobacter baumannii*, *Enterobacter* spp., *Klebsiella pneumoniae*, and *Pseudomonas aeruginosa*. The color of each circle corresponds to the host pathogen as indicated in the legend. The phylogenetic tree can be explored in more detail through the following shared session: https://microreact.org/project/eZxEhaFTqS4KstKqFUNKQ7-pathogenrectclade. The session allows for interactive zooming and scrolling (up, down, left, and right) to facilitate the examination of specific branches and clades of each enzyme group and their corresponding sequences.

Phylogenetic analysis of the 1790 AmpC sequences revealed that AmpC enzyme groups of *A. baumannii, P. aeruginosa, K. pneumoniae, and Enterobacter* spp. formed four distinct groups, each suggesting conserved evolutionary paths within our groups **(**Fig. 2**)**. To comprehensively visualize the evolutionary relationships and diversity of the AmpC enzymes, Fig. S1 **is provided, showing** the major clades with branch length, a condensed phylogram with collapsed nodes, and a detailed, uncollapsed version of this phylogram, which includes all individual sequences and their bootstrap values, is provided as Fig. S2.

**Supplementary Figure S3:**
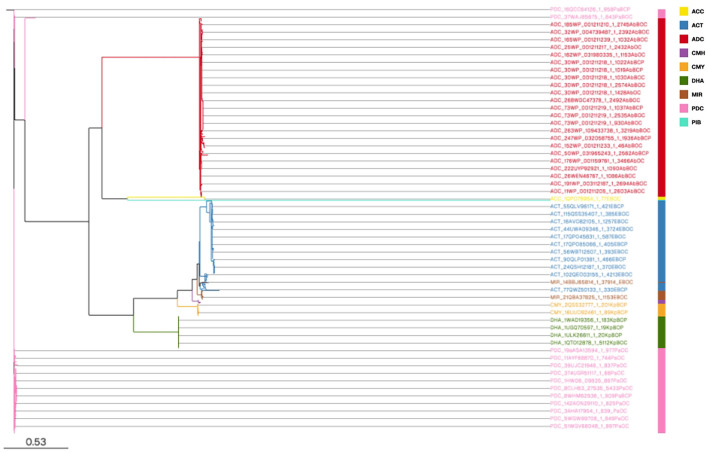
Phylogram of AmpC Enzyme Groups. This phylogram is a detailed representation of the evolutionary relationships among the 1790 AmpC sequences. The tree nodes and tips are color-coded to indicate the specific AmpC enzyme group. The phylogenetic tree can be explored in more detail through the following shared session: https://microreact.org/project/okssuk9PwZ7tUmZwsMp2f6-familycirc. The session allows for interactive zooming and scrolling (up, down, left, and right) to facilitate the examination of specific branches and clades of each enzyme group and their corresponding sequences.

The AmpC sequences of *P. aeruginosa* and *A. baumannii* formed distinct and well-defined groupings, indicating significant conservation. *K. pneumoniae*, on the other hand, had mixed clustering with *P. aeruginosa* and *Enterobacter* species. Additionally, *Enterobacter* spp. and *P. aeruginosa* had mixed clustering, suggesting that their *blaAmpC* share evolutionary origins unique to these genera. The phylogram of AmpC enzyme groups further highlights that these evolutionary patterns are independent of the genetic location (chromosomal/plasmid) among all the AmpC enzyme groups **(**Fig. S3**)**. The AmpC enzyme groups ACC, ACT, ADC, CMH, CMY, DHA, MIR, PDC, and PIB, which were present in all four pathogens, formed separate clusters; however, these clusters could be sub-grouped based on the pathogen. However, AmpC enzyme groups ADC, ACC, PDC, ACT, MIR, CMH, and PIB are present only in a single pathogen and form independent clusters, respectively.

### 3.6 Phylogenetic insights into AmpC enzyme groups

Amino acid variation analysis within the nine AmpC enzyme groups revealed that the amino acid sequences of the ADC enzyme group formed a separate cluster, which is exclusively conserved in *A. baumannii*. The PDC conserved in *P. aeruginosa* forms its cluster. Since DHA is present in both *Enterobacter* spp. and *K. pneumoniae*, it is possible that these pathogens shared a genetic ancestor or an evolutionary pathway. *P. aeruginosa* and *K. pneumoniae* share the CMY enzyme group, which may indicate horizontal gene transfer. Enzyme groups restricted to *Enterobacter* spp. form unique clusters, like ACC, ACT, MIR, and CMH, which show diversification within their enzyme groups. PIB is present exclusively in *P. aeruginosa*, forming a unique branch with a branch length of 3.3812, which likely reflects its significant evolutionary rate and divergence from the other eight AmpC enzyme groups. Notably, the largest enzyme group, ADC, showed the most critical degree of variation, followed by ACT and PDC, indicating a notable degree of diversity within these groups. On the other hand, the ACC and PIB enzyme groups in *Enterobacter* spp. showed no variations, indicating potential conservation of function within these enzyme groups **(Table S3)**.

### 3.7 Pairwise identity between the enzyme groups

Given the diversity of AmpC among different pathogens, we conducted pairwise comparisons of the representative sequences of nine enzyme groups to examine their relatedness. Pairwise sequence identity among the nine enzyme group representatives ranged from 18.84 % to 100 % **(**Table 4**)**. For instance, the greatest sequence identities were noticed in the groups CMH, ACT, and MIR and were 89.76 % (ACT-MIR), 89.50 % (CMH-MIR), and 87.40 % (CMH-ACT). Strong similarities were also noted between CMY and other groups, such as CMH (76.38 %), MIR (75.85 %), and ACT (74.02 %). However, the lowest observed identities of ACC and PIB (18.84 %) and PDC and PIB (18.84 %) indicate a higher degree of divergence, or they might be distant homologs. These findings demonstrate the diversity through percentage identity among the various entities, with some pairs showing close alignments and others showing significant divergence.

**Table 4:**
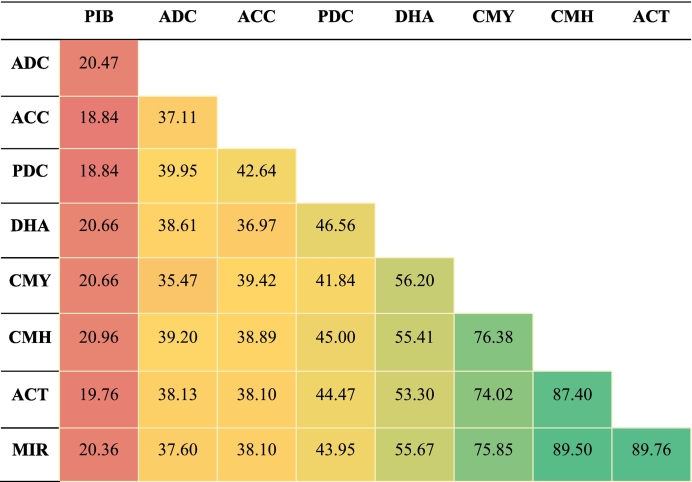
Pairwise amino acid identities (%) between AmpC enzyme groups.

### 3.8 Amino acid analysis of representatives of the AmpC enzyme groups and their phylogenetic study

The AmpC enzymes are a heterogeneous group, with diverse sequences and functional properties. All enzyme groups of AmpC belong to Ambler Class C; however, we found low sequence identity within the class. This means that although these enzymes have similar functions (i.e., β-lactamase activity), their amino acid sequences differ significantly. Hence, to better understand the common characteristics that might be shared among the various AmpC enzyme groups, the amino acid sequences of representative AmpC enzymes of the nine AmpC enzyme groups were analyzed. The analysis aims to identify conserved motifs, structural features, or functional domains that explain enzyme properties like substrate specificity, stability, and antibiotic resistance profiles by aligning representative sequences.

**Fig. 3:**
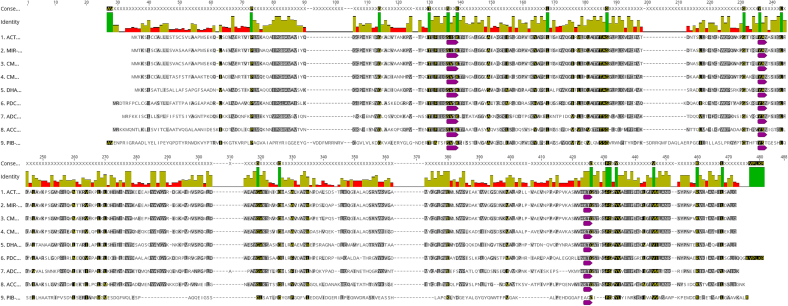
**Multiple Sequence Alignment and Amino Acid Conservation of Representative AmpC Beta-lactamase Sequences.** The corresponding conservation plot highlights identical amino acid residues across the sequences in dark green, indicating conserved regions within the enzyme groups. Key functional motifs are highlighted in purple circles, including the ‘SXXK,’ ‘YXN,’ and ‘KTG’ motifs crucial for catalytic activity. Notably, the PIB enzyme group shows unique motif variants (YST and AQG) instead of the canonical YXN and KTG motifs. (For interpretation of the references to color in this figure legend, the reader is referred to the web version of this article.)

Green bars in the identity plot of Fig. 3 highlight highly conserved regions observed across all the representative sequences; these sequences suggest functional and structural importance, as evolutionary pressures preserve these residues to maintain the essential role. Notably, all three significant conserved motifs, ‘SXSK’, ‘YXN’, and ‘KTG’ of class C β-lactamase were conserved in all eight enzyme groups, excluding the PIB enzyme group. However, PIB retains the SXXK motif, which is essential for its catalytic activity, but lacks the typical YXN and KTG motifs that are present in the other eight enzyme groups (e.g., ACT, MIR, CMY, DHA, PDC, ADC, ACC). Instead of the YXN and KTG motifs, it possesses the YST and AQG motifs   (Philippon et al., 2022). Despite being categorized as an AmpC Class C β-lactamase, the enzyme group showed a completely different pattern from the other eight. This was also confirmed in the study, which indicates that PIB does not bind to cephalosporins while enhancing its activity against carbapenems (Philippon et al., 2022) because of the difference in conserved motifs.

The motif variations in PIB fundamentally alter its substrate profile compared to other Class C β-lactamases (Table 5) (Medrano et al., 2024; Philippon et al., 2022). While the SXXK catalytic motif remains conserved across all enzyme groups, the YXN, YST substitution (asparagine to threonine) in PIB reduces its affinity for cephalosporins, and the KTG, AQG change (lysine to alanine, threonine to glutamine) enhances carbapenem binding specificity. These structural modifications explain PIB's unique resistance profile and its clinical significance as a carbapenem-hydrolyzing enzyme (Medrano et al., 2024).

**Table 5:** Implications of Motif Variants in the PIB enzyme group.

Motif	PIB Variant	Structural Implication
SX SK	SXXK	Retains nucleophilic serine for acylation; the catalytic mechanism is preserved across all class C enzymes.
YXN	YST	Asparagine to Threonine reduces hydrogen bonding with β-lactam C4 carboxylate, lowering cephalosporin affinity.
KTG	AQG	Lysine to Alanine and Threonine to Glutamine disrupt ionic stabilization of the β-lactam ring, favoring carbapenem binding by altering active-site loop conformation.

**Fig. 4:**
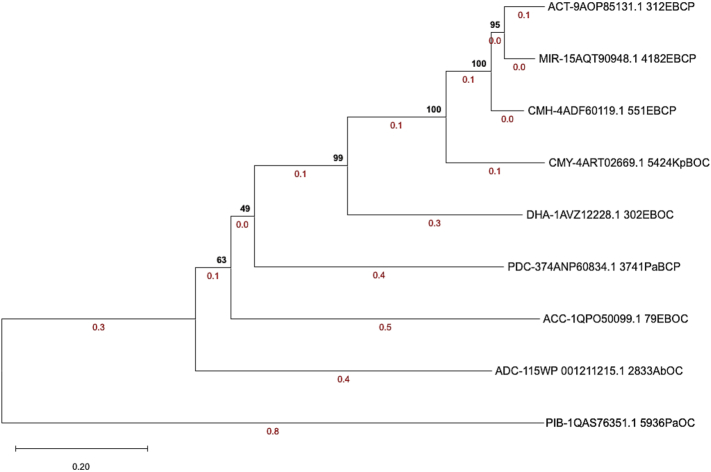
**Phylogenetic Relationships Among Representative AmpC Enzyme Groups.** This Neighbor-Joining tree with bootstrap values (1000 replicates) is shown at the nodes to indicate statistical support. Branch lengths, marked by the red numbers, represent the evolutionary distance and illustrate the evolutionary divergence of the nine AmpC enzyme groups. (For interpretation of the references to color in this figure legend, the reader is referred to the web version of this article.)

Phylogenetic relationships among the nine representative AmpC sequences inferred by NJ in MEGA12 **(**Fig. 4**)** reveal tight clustering of *Enterobacter* spp. associated enzymes: ACT and MIR form a robust clade (bootstrap 0.955; branch lengths 0.061 and 0.046), with CMH joining this group at full support (1.000). CMY branches next (1.000; 0.149), followed by DHA at deep divergence (0.994; 0.265). PDC occupies an outlying position relative to the *Enterobacter* spp. cluster (0.496; 0.378), consistent with its *P. aeruginosa* origin. ACC groups subsequently (0.636; 0.468), while ADC and PIB represent the most divergent lineages, with PIB's long branch (1.117) defining it as the outgroup and ADC branching at 0.449. High bootstrap values across internal nodes confirm genus-specific lineages and broader divergence among AmpC classes.

**Fig. 5:**
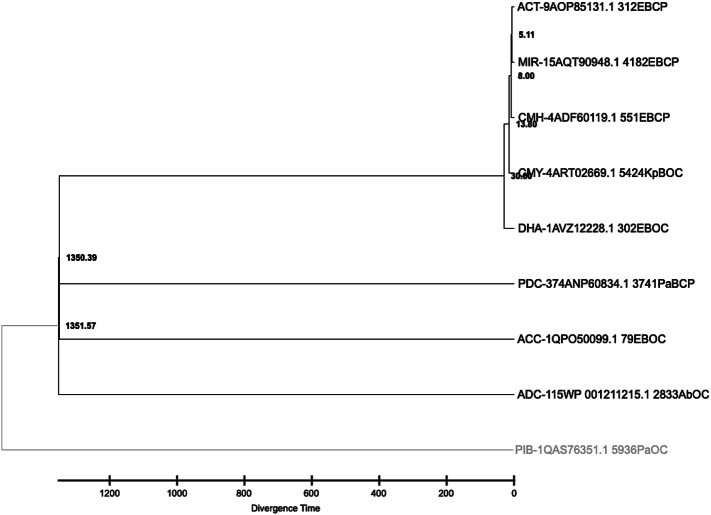
**Time-Calibrated Phylogenetic Tree of Representative AmpC Enzyme Groups.** This phylogram was generated using RelTime-ML to estimate the divergence times of the nine representative AmpC enzyme groups. The horizontal axis represents divergence time in millions of years ago (mya).

A time-calibrated phylogenetic tree of the nine representative AmpC enzyme sequences was constructed using RelTime-ML with calibration priors obtained from known divergence times **(**Fig. 5**)**. This analysis reveals that the split between *Enterobacter* spp. associated AmpC groups (ACT, MIR, CMH, CMY, DHA) and *P. aeruginosa* ones (PDC, PIB) occurred approximately 1350–1351 million years ago (mya), consistent with the divergence of their bacterial hosts. The *Enterobacter* spp. lineage sequences clustered tightly, with ACT and MIR showing the most recent common ancestry, diverging roughly within the past 10–20 mya, supported by high bootstrap values and short branch lengths. The *P. aeruginosa-*specific PIB exhibits the greatest branch length and represents the basal outgroup in the tree, demonstrating its high evolutionary rate and early divergence from other AmpC groups, and also has different motifs, enhancing carbapenem binding specificity. Therefore, PIB is excluded from further analysis as this study is focused on cephalosporins. ADC and ACC, corresponding to *A. baumannii* and distinct *Enterobacter* spp. lineages, respectively, diverged near 1350 mya but form separate clades consistent with their species specificity and functional uniqueness. This temporal framework aligns with known host divergence, reinforcing that AmpC enzyme evolution is tightly linked to its bacterial host phylogeny. Overall, the tree supports the hypothesis that AmpC enzymes co-evolved with their bacterial species over hundreds of millions of years, with diversification events corresponding to host speciation and lateral gene transfer events reflected by subgroup clustering within *Enterobacter* spp. and *P. aeruginosa* branches. Consistent with previous comprehensive studies on AmpC β-lactamases (Jacoby, 2009; Hall and Barlow, 2004), our phylogenetic analysis supports the ancient origin and deep evolutionary divergence of AmpC enzymes, which have co-evolved with their bacterial hosts over hundreds of millions to billions of years (Hall and Barlow, 2004; Jacoby, n.d.). These enzymes have diversified their sequence and functional properties while maintaining conserved catalytic motifs, reflecting long-term evolutionary pressures and recent adaptations to clinical antibiotic use.

This is also supported by the pairwise identity between MIR and ACT, MIR and CMH, ACT and CMH, CMH and CMY, MIR and CMY, and ACT and CMY enzyme groups, which were 89.76 %, 89.50 %, 87.40 %, 76.38 %, 75.85 %, and 74.02 % respectively. Their sequence identities also demonstrate these enzyme groups' branch lengths and the time elapsed since their divergence from a common ancestor. The sequence identities indicate a recent divergence or a close relationship between them. Overall, this suggested that the higher the pairwise sequence identity between two sequences, the smaller the divergence between them and the shorter the period since their divergence.

### 3.9 PROVEAN analysis reveals functionally significant variants in SXXK, YXN, and KTG motifs of class C β-lactamases

**Fig. 6:**
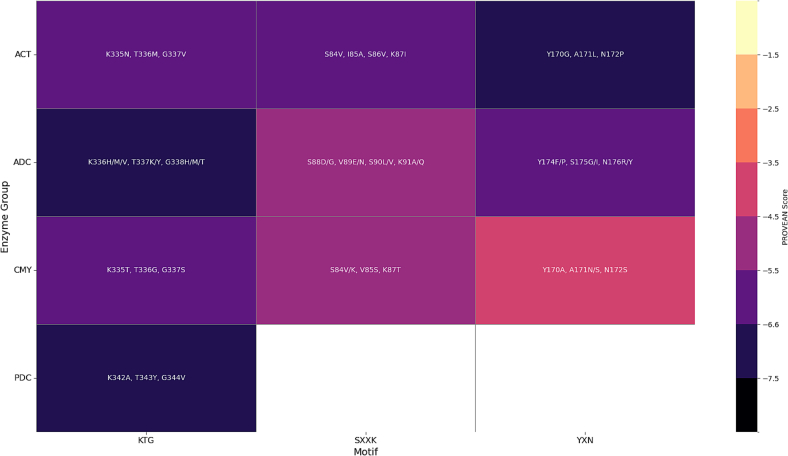
**PROVEAN Analysis of Functionally Significant Amino Acid Variants within Conserved AmpC Motifs.** This heatmap visually represents the predicted functional impact of amino acid substitutions within the SXXK, YXN, and KTG catalytic motifs of four major AmpC enzyme groups (ACT, ADC, CMY, and PDC). The color scale on the right indicates the PROVEAN score, where darker colors signify more deleterious (damaging) effects on protein function. Lighter colors correspond to less damaging substitutions.

PROVEAN analysis identified deleterious substitutions within the three conserved motifs (SXXK, YXN, and KTG), reinforcing their structural and catalytic importance, shown in Fig. 6 and **Table S4**.

**ACT:** Mutations S84V, S86V, and K87I within the SXXK motif were described with a strong positive bias to being harmful (scores < −2.5), suggesting that there would be some disruption to active-site serine nucleophilicity as well as catalytic conformation.

Concerning the YXN motif, changes such as Y170G and N172P were also found to be damaging (scores < −2.5), likely affecting substrate positioning. The KTG motif variants K335N, T336M, and G337V have shown sharply negative PROVEAN scores, consistent with loss-of-function destabilization.

**PDC:** Substitutions K342A, T343Y, and G344V within the KTG motif were all deleterious (scores < −2.5), suggesting impairment in β-lactam ring stabilization or hydrolysis.

**ADC:** Several deleterious substitutions were clustered within the SXXK motif: S88D/G, K91A/Q, and the flanking positions (V89E/N, S90L/V). In the YXN motif, Y174F/P, N176R/Y, and S175I/G were predicted to be deleterious, indicating possible effects on substrate affinity. KTG motif mutations (K336V/M/H, T337K/Y, and G338H/M/T) were uniformly deleterious (scores −5.9 ≤ −8.9).

**CMY:** Similar deleterious variants in conserved motifs were observed: S84V/K, V85S, and K87T, highlighting preservation of the SXXK catalytic site. The YXN motif also exhibited deleterious substitutions (Y170A, N172S) with negative scores, while A171N/S appeared neutral. KTG mutations again proved damaging: K335T, T336G, and G337S all exceeded the deleterious thresholds.

### 3.10 Structural variations in 3D protein models of AmpC

**Fig. 7:**
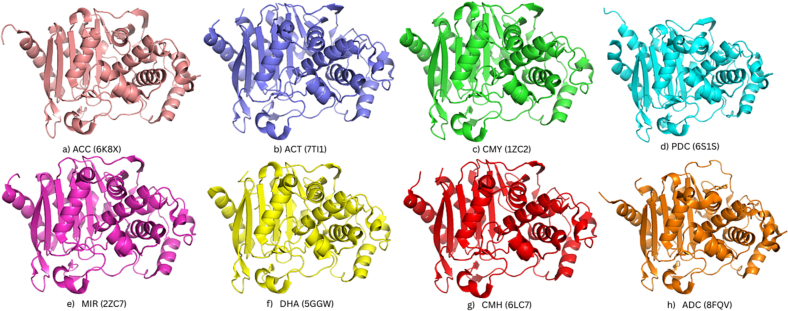
**Three-Dimensional Structures of Representative AmpC Beta-Lactamase Enzyme Groups.** This figure presents ribbon diagrams of the crystal structures for eight major AmpC enzyme groups (ACC, ACT, CMY, PDC, MIR, DHA, CMH, and ADC). Each panel displays a representative structure, with its corresponding PDB ID listed in parentheses.

The three-dimensional structures of representative AmpC enzymes were analyzed to correlate their unique features with their host organisms and resistance profiles. Individual structures for eight enzyme groups (ADC, ACC, PDC, DHA, CMY, CMH, ACT, and MIR) are presented as separate panels in Fig. 7. The PIB group was excluded from this structural analysis due to its significant sequence and motif divergence, which confers unique carbapenem-hydrolyzing activity rather than the cephalosporinase activity that is the focus of this study.

**Fig. 8:**
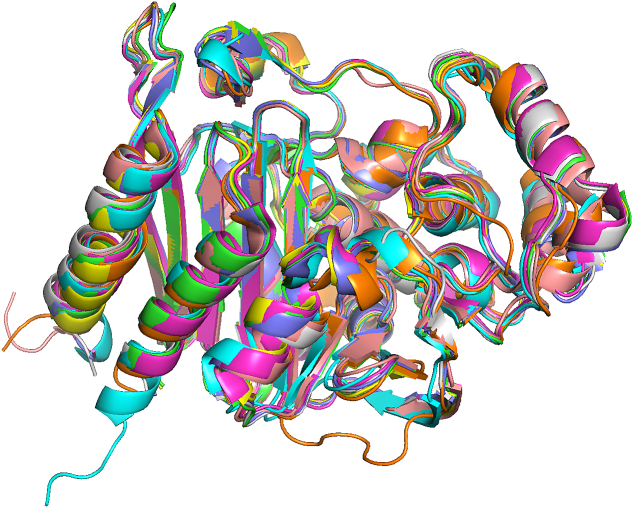
**Superimposed 3D protein models of representatives of the nine AmpC enzyme groups.** (Color Key, Orange: 8FQV(ADC), Light Pink: 6K8X(ACC), Cyan: 6S1S(PDC), Yellow: 5GGW(DHA), Green: 1ZC2(CMY), Grey: 6LC7(CMH), Purple: 7TI1(ACT), Magenta: 2ZC7(MIR). (For interpretation of the references to color in this figure legend, the reader is referred to the web version of this article.)

**Supplementary Figure S5:**
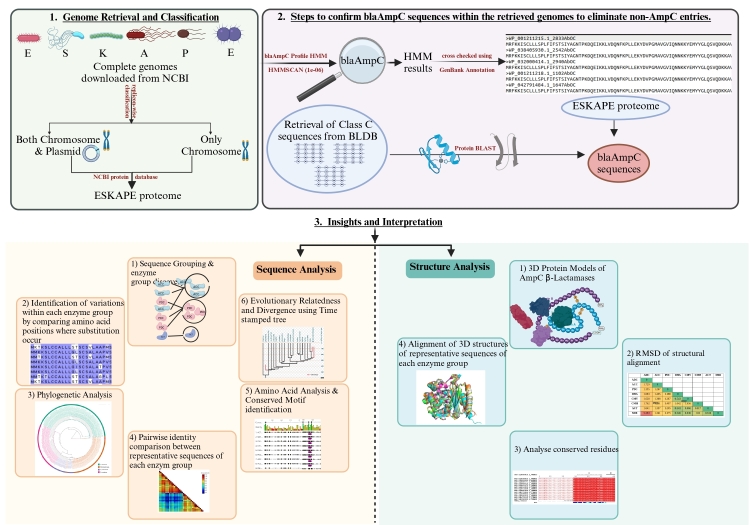
The comprehensive methodological approach employed for the investigation of *blaAmpC* in ESKAPE pathogens.

To quantify structural relationships, we performed a pairwise superimposition of the eight protein models **(**Fig. 8**)** and calculated the Root Mean Square Deviation (RMSD) values **(**Table 6**)**. The resulting RMSD values directly correlated with structural similarity, phylogenetic relationships, and the host organism's resistance patterns. The multiple sequence alignment of the AmpC sequences demonstrated that the primary and secondary amino acid sequences were significantly conserved within a subfamily (Fig. S4). Fig. S5 depicts the overall workflow adopted in this study.

**Table 6:**
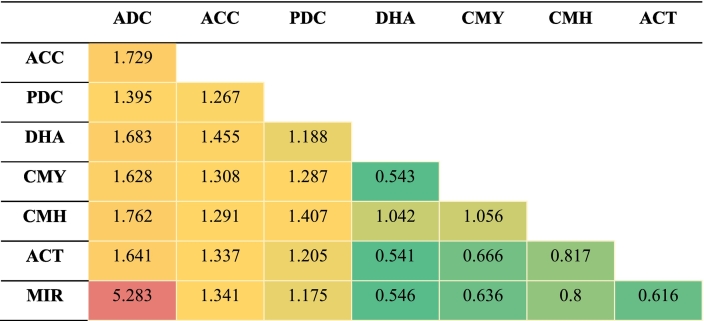
Root Mean Square Deviation values of pairwise structure alignments of 3D protein models of the nine AmpC enzyme groups (The color scale is from highest (red) to lowest (green)).

The ADC enzyme showed the highest structural divergence, evidenced by the large RMSD values (MIR group is 5.283 Å, the highest value). This structural uniqueness reflects its phylogenetic isolation and role as a key intrinsic chromosomal enzyme found exclusively in *A. baumannii.* Similarly, PDC has a low pairwise identity with PIB (18.84 %), similar to ACC. Both PDC and ACC are structurally divergent from the other groups, as shown by their high RMSD values with most enzymes. This distinction is expected because PDC is a chromosomally encoded enzyme unique to *P. aeruginosa,* and ACC has its own unique evolutionary path, consistent with its exclusive presence in *Enterobacter* spp.

Conversely, the AmpC enzymes predominantly found in *Enterobacter* spp. and *K. pneumoniae* (CMY, DHA, ACT, CMH, and MIR) exhibited high structural similarity, with low RMSD values among them (e.g., CMY vs. DHA: 0.543 Å; MIR vs. CMY: 0.636 Å). This structural conservation strongly suggests a common evolutionary origin and a high potential for horizontal gene transfer among these related pathogens, a major driver of acquired resistance. The structural analysis confirms that a low RMSD value in pairwise alignment directly correlates with a high pairwise sequence identity and low evolutionary divergence, reinforcing the phylogenetic and sequence-based findings. This suggests that the AmpC enzyme groups' structural variations correlate with the host organism and the specific resistance mechanism. Intrinsic chromosomal enzymes, such as ADC and PDC, have evolved distinct structures, while enzymes frequently carried on plasmids (like CMY and DHA) share a common structural scaffold, facilitating their rapid spread and contributing to widespread multidrug resistance.

## 4 Conclusion

This comprehensive genomic analysis reveals that AmpC enzymes are highly diverse and contribute significantly to antimicrobial resistance among ESKAPE pathogens, with a notable absence in *E. faecium* and *S. aureus*. Identifying nine distinct AmpC groups, each with unique occurrence patterns and varying degrees of sequence diversity, underscores these resistance determinants' evolutionary complexity and adaptability. Phylogenetic and structural analyses further highlight these enzymes' close relationships and potential for horizontal gene transfer. Our results highlight AmpC enzymes contributing to the escalating problem of antimicrobial resistance among strains in the nosocomial environment. These findings emphasize the urgent need for ongoing surveillance, experimental validation, and the development of targeted therapeutic strategies to address the growing threat of AmpC-mediated resistance in clinical settings. However, the present study has some limitations that warrant future investigation. Notably, the functional impact predictions are based solely on computational methods and lack experimental validation. Therefore, future studies should prioritize experimental validation of these variants through phenotypic assays, enzymatic activity measurements, and clinical correlation to better understand their impact on antimicrobial resistance. Incorporating such experimental data will significantly enhance the translational relevance and robustness of insights into the role of AmpC mutations in resistance evolution. Our study re-emphasizes the responsible use of antibiotics, particularly in hospital settings such as Intensive Care Units (ICUs), which raise serious concerns about limited treatment options, to ensure more effective treatments and better patient outcomes in the fight against AMR.

The following are the supplementary data related to this article.

Supplementary Figure S2 Neighbor-Joining Phylogram of 1790 AmpC Beta-Lactamase Sequences. This tree illustrates the detailed evolutionary relationships and diversity among all 1790 AmpC sequences from *Acinetobacter baumannii*, *Enterobacter* spp., *Klebsiella pneumoniae*, and *Pseudomonas aeruginosa*. The branches are color-coded according to the pathogen, revealing distinct clades and bootstrap values. Supplementary Figure S2

Supplementary material 1 Supplementary Figure S4: Structure-based multiple sequence alignment of the AmpC enzyme groups in ESKAPE Pathogens. Supplementary material 1

Supplementary Table 1 Origin (Natural/Acquired) of Ambler Class C Beta-lactamases. Supplementary Table 1

Supplementary Table 2 Distribution of AmpC enzyme groups in ESKAPE pathogens. Supplementary Table 2

Supplementary Table 3 List of variations within each enzyme group by comparing amino acid positions where substitution occurs. Supplementary Table 3

Supplementary Table 4 Variant Analysis of Conserved Motifs in Class C β-Lactamases Using PROVEAN. Supplementary Table 4

## Author's Contribution

DP conceived the idea, collected and organized the data. DP and IG performed the analysis, finalized the results and drafted the original draft. DG supervised the work and revised the manuscript. All authors reviewed and finalized the manuscript.

## CRediT authorship contribution statement

**Deeksha Pandey:** Writing – review & editing, Validation, Supervision, Funding acquisition, Formal analysis, Data curation. **Isha Gupta:** Writing – review & editing, Writing – original draft, Visualization, Software, Resources, Methodology, Investigation. **Dinesh Gupta:** Writing – review & editing, Supervision, Investigation, Funding acquisition, Formal analysis, Conceptualization.

## Consent for publication

Not applicable.

## Ethics approval and consent to participate

Not applicable.

## Funding

The work was carried out using the resources funded by the Department of Biotechnology (DBT), Ministry of Science and Technology, Government of India (Grant No: BT/PR40151/BTIS/137/5/2021 and BT/PR40180/BTIS/137/59/2023).

## Declaration of competing interest

The authors declare that they have no known competing financial interests or personal relationships that could have appeared to influence the work reported in this paper.

## Data Availability

The dataset used for this study can be found in GitHub repository: https://github.com/tbgicgeb/AmpC-Beta-lactamases GithubAmpC-Beta-lactamases (Original data)
