# Supplementary material for: AmpC β-lactamases: A key to antibiotic resistance in ESKAPE pathogens
Source: Cell Surf. 2025 Sep 22;14:100154. doi: 10.1016/j.tcsw.2025.100154 (PMC12528871; doi:10.1016/j.tcsw.2025.100154)
Supplement: Supplementary material 1 — Supplementary Figure S4: Structure-based multiple sequence alignment of the AmpC enzyme groups in ESKAPE Pathogens. [file mmc4.pdf]

ACC enzyme group

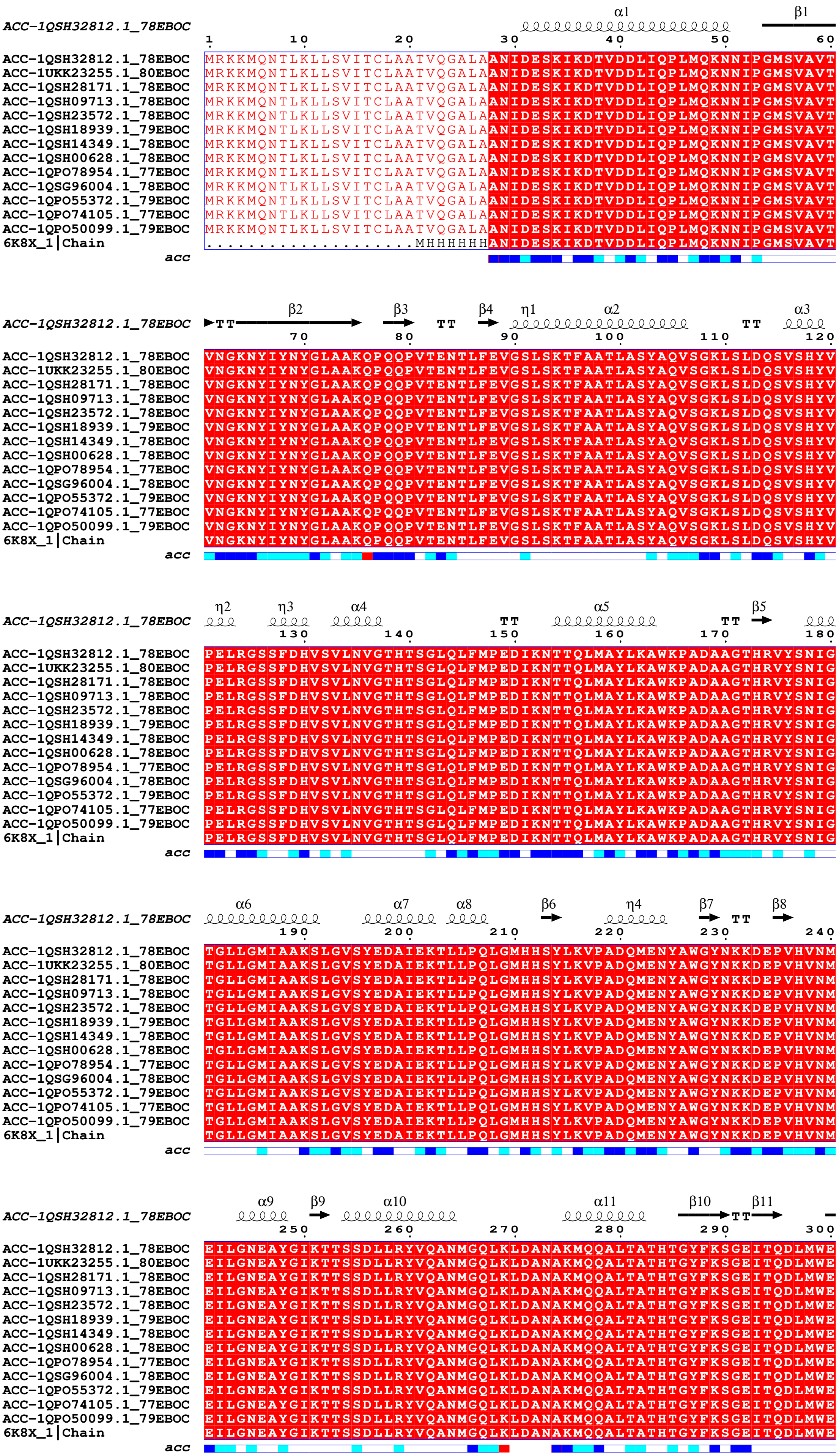

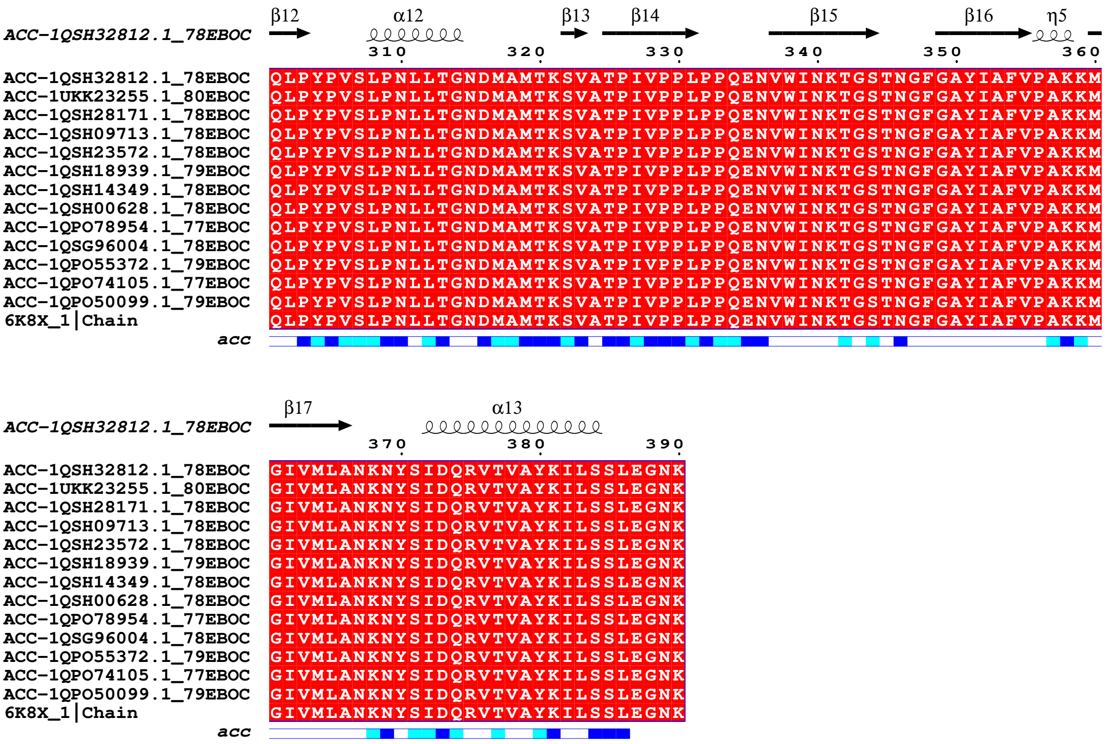
β12
α12
β13
β14
β15
β16
η5
ACC-1QSH32812.1_78EBOC
310
320
330
340
350
360
ACC-1QSH32812.1_78EBOC QLPYPVSLPNLLTGNDMAMTKSVATPIVPPLPPQENVWINKTGSTNGFGAYIAFVPAKKM
ACC-1UKK23255.1_80EBOC QLPYPVSLPNLLTGNDMAMTKSVATPIVPPLPPQENVWINKTGSTNGFGAYIAFVPAKKM
ACC-1QSH28171.1_78EBOC QLPYPVSLPNLLTGNDMAMTKSVATPIVPPLPPQENVWINKTGSTNGFGAYIAFVPAKKM
ACC-1QSH09713.1_78EBOC QLPYPVSLPNLLTGNDMAMTKSVATPIVPPLPPQENVWINKTGSTNGFGAYIAFVPAKKM
ACC-1QSH23572.1_78EBOC QLPYPVSLPNLLTGNDMAMTKSVATPIVPPLPPQENVWINKTGSTNGFGAYIAFVPAKKM
ACC-1QSH18939.1_79EBOC QLPYPVSLPNLLTGNDMAMTKSVATPIVPPLPPQENVWINKTGSTNGFGAYIAFVPAKKM
ACC-1QSH14349.1_78EBOC QLPYPVSLPNLLTGNDMAMTKSVATPIVPPLPPQENVWINKTGSTNGFGAYIAFVPAKKM
ACC-1QSH00628.1_78EBOC QLPYPVSLPNLLTGNDMAMTKSVATPIVPPLPPQENVWINKTGSTNGFGAYIAFVPAKKM
ACC-1QPO78954.1_77EBOC QLPYPVSLPNLLTGNDMAMTKSVATPIVPPLPPQENVWINKTGSTNGFGAYIAFVPAKKM
ACC-1QSG96004.1_78EBOC QLPYPVSLPNLLTGNDMAMTKSVATPIVPPLPPQENVWINKTGSTNGFGAYIAFVPAKKM
ACC-1QPO55372.1_79EBOC QLPYPVSLPNLLTGNDMAMTKSVATPIVPPLPPQENVWINKTGSTNGFGAYIAFVPAKKM
ACC-1QPO74105.1_77EBOC QLPYPVSLPNLLTGNDMAMTKSVATPIVPPLPPQENVWINKTGSTNGFGAYIAFVPAKKM
ACC-1QPO50099.1_79EBOC QLPYPVSLPNLLTGNDMAMTKSVATPIVPPLPPQENVWINKTGSTNGFGAYIAFVPAKKM
6K8X_1|Chain QLPYPVSLPNLLTGNDMAMTKSVATPIVPPLPPQENVWINKTGSTNGFGAYIAFVPAKKM
acc
β17
α13
ACC-1QSH32812.1_78EBOC
370
380
390
ACC-1QSH32812.1_78EBOC GIVMLANKNYSIDQRVTVAYKILSSLEGNK
ACC-1UKK23255.1_80EBOC GIVMLANKNYSIDQRVTVAYKILSSLEGNK
ACC-1QSH28171.1_78EBOC GIVMLANKNYSIDQRVTVAYKILSSLEGNK
ACC-1QSH09713.1_78EBOC GIVMLANKNYSIDQRVTVAYKILSSLEGNK
ACC-1QSH23572.1_78EBOC GIVMLANKNYSIDQRVTVAYKILSSLEGNK
ACC-1QSH18939.1_79EBOC GIVMLANKNYSIDQRVTVAYKILSSLEGNK
ACC-1QSH14349.1_78EBOC GIVMLANKNYSIDQRVTVAYKILSSLEGNK
ACC-1QSH00628.1_78EBOC GIVMLANKNYSIDQRVTVAYKILSSLEGNK
ACC-1QPO78954.1_77EBOC GIVMLANKNYSIDQRVTVAYKILSSLEGNK
ACC-1QSG96004.1_78EBOC GIVMLANKNYSIDQRVTVAYKILSSLEGNK
ACC-1QPO55372.1_79EBOC GIVMLANKNYSIDQRVTVAYKILSSLEGNK
ACC-1QPO74105.1_77EBOC GIVMLANKNYSIDQRVTVAYKILSSLEGNK
ACC-1QPO50099.1_79EBOC GIVMLANKNYSIDQRVTVAYKILSSLEGNK
6K8X_1|Chain GIVMLANKNYSIDQRVTVAYKILSSLEGNK
acc

ACT-17BBW34093.1_49417]EBCP

```
                               1        10        20        30        40        50        60
ACT-17BBW34093.1_49417]EBCP    MLNLTVCQALSNPTDYACLTGPDIPLTRYYGRLLMMKKSLCCALLLGISCSALATPVSEK
ACT-17BBW34093.1_49417]EBOC    MLNLTVCQALSNPTDYACLTGPDIPLTRYYGRLLMMKKSLCCALLLGISCSALATPVSEK
7TI1_1|Chain                   ..................................MMRKSLCCALLLGISCSALATPVSEK
ACT-37QPO56206.1_411EBCP       ..................................MMKKSFCCALLLAISGAALAAPVSEK
ACT-37QPO65286.1_410EBCP       ..................................MMKKSFCCALLLAISGAALAAPVSEK
ACT-37UKK25917.1_410EBCP       ..................................MMKKSFCCALLLAISGAALAAPVSEK
ACT-37UKK30553.1_410EBCP       ..................................MMKKSFCCALLLAISGAALAAPVSEK
ACT-37QPO56206.1_411EBOC       ..................................MMKKSFCCALLLAISGAALAAPVSEK
ACT-37QPO65286.1_410EBOC       ..................................MMKKSFCCALLLAISGAALAAPVSEK
ACT-37UKK25917.1_410EBOC       ..................................MMKKSFCCALLLAISGAALAAPVSEK
ACT-37UKK30553.1_410EBOC       ..................................MMKKSFCCALLLAISGAALAAPVSEK
ACT-65AXO42196.1_4001EBCP      ..................................MMKKSLCCALLLGISCSALATPVSEK
ACT-65AXO42196.1_4001EBOC      ..................................MMKKSLCCALLLGISCSALATPVSEK
ACT-65WBV31824.1_394EOC        ..................................MMKKSLCCALLLGISCSALATPVSEK
ACT-17ARZ78489.1_1874EBCP      ..................................MMKKSLCCALLLGISCSALATPVSEK
ACT-17ASQ75348.1_386EBCP       ..................................MMKKSLCCALLLGISCSALATPVSEK
ACT-17AWV77932.1_4320EBCP      ..................................MMKKSLCCALLLGISCSALATPVSEK
ACT-17QEQ46013.1_2619EBCP      ..................................MMKKSLCCALLLGISCSALATPVSEK
ACT-17QFI20602.1_494EBCP       ..................................MMKKSLCCALLLGISCSALATPVSEK
ACT-17QIE65424.1_3050EBCP      ..................................MMKKSLCCALLLGISCSALATPVSEK
ACT-17QPD71520.1_416EBCP       ..................................MMKKSLCCALLLGISCSALATPVSEK
ACT-17QPO45831.1_587EBCP       ..................................MMKKSLCCALLLGISCSALATPVSEK
ACT-17QPO51098.1_586EBCP       ..................................MMKKSLCCALLLGISCSALATPVSEK
ACT-17QPO69921.1_405EBCP       ..................................MMKKSLCCALLLGISCSALATPVSEK
ACT-17QPO74759.1_405EBCP       ..................................MMKKSLCCALLLGISCSALATPVSEK
ACT-17QPO85066.1_405EBCP       ..................................MMKKSLCCALLLGISCSALATPVSEK
ACT-17QSB73424.1_1103EBCP      ..................................MMKKSLCCALLLGISCSALATPVSEK
ACT-17QXY98988.1_1068EBCP      ..................................MMKKSLCCALLLGISCSALATPVSEK
ACT-17UDV73425.1_430EBCP       ..................................MMKKSLCCALLLGISCSALATPVSEK
ACT-17UDV69018.1_894EBCP       ..................................MMKKSLCCALLLGISCSALATPVSEK
ACT-17UKK21116.1_588EBCP       ..................................MMKKSLCCALLLGISCSALATPVSEK
ACT-17UOY44701.1_2566EBCP      ..................................MMKKSLCCALLLGISCSALATPVSEK
ACT-17URL69109.1_2030EBCP      ..................................MMKKSLCCALLLGISCSALATPVSEK
ACT-17UUQ70490.1_426EBCP       ..................................MMKKSLCCALLLGISCSALATPVSEK
ACT-17UWA70738.1_427EBCP       ..................................MMKKSLCCALLLGISCSALATPVSEK
ACT-17UWC98141.1_545EBCP       ..................................MMKKSLCCALLLGISCSALATPVSEK
ACT-17UXC72711.1_448EBCP       ..................................MMKKSLCCALLLGISCSALATPVSEK
ACT-17UXC77377.1_448EBCP       ..................................MMKKSLCCALLLGISCSALATPVSEK
ACT-17UXC82023.1_448EBCP       ..................................MMKKSLCCALLLGISCSALATPVSEK
ACT-17UZE41799.1_406EBCP       ..................................MMKKSLCCALLLGISCSALATPVSEK
ACT-17UZH49391.1_396EBCP       ..................................MMKKSLCCALLLGISCSALATPVSEK
ACT-17WAZ63377.1_405EBCP       ..................................MMKKSLCCALLLGISCSALATPVSEK
ACT-17WDU86092.1_3769EBCP      ..................................MMKKSLCCALLLGISCSALATPVSEK
ACT-17WDW12975.1_3764EBCP      ..................................MMKKSLCCALLLGISCSALATPVSEK
ACT-17WGA71117.1_396EBCP       ..................................MMKKSLCCALLLGISCSALATPVSEK
ACT-17WGA75603.1_396EBCP       ..................................MMKKSLCCALLLGISCSALATPVSEK
ACT-17WGZ57121.1_2667EBCP      ..................................MMKKSLCCALLLGISCSALATPVSEK
ACT-17ARZ78489.1_1874EBOC      ..................................MMKKSLCCALLLGISCSALATPVSEK
ACT-17ASQ75348.1_386EBOC       ..................................MMKKSLCCALLLGISCSALATPVSEK
ACT-17AWV77932.1_4320EBOC      ..................................MMKKSLCCALLLGISCSALATPVSEK
ACT-17QEQ46013.1_2619EBOC      ..................................MMKKSLCCALLLGISCSALATPVSEK
ACT-17QFI20602.1_494EBOC       ..................................MMKKSLCCALLLGISCSALATPVSEK
ACT-17QIE65424.1_3050EBOC      ..................................MMKKSLCCALLLGISCSALATPVSEK
ACT-17QPD71520.1_416EBOC       ..................................MMKKSLCCALLLGISCSALATPVSEK
ACT-17QPO45831.1_587EBOC       ..................................MMKKSLCCALLLGISCSALATPVSEK
ACT-17QPO51098.1_586EBOC       ..................................MMKKSLCCALLLGISCSALATPVSEK
ACT-17QPO69921.1_405EBOC       ..................................MMKKSLCCALLLGISCSALATPVSEK
ACT-17QPO74759.1_405EBOC       ..................................MMKKSLCCALLLGISCSALATPVSEK
ACT-17QPO85066.1_405EBOC       ..................................MMKKSLCCALLLGISCSALATPVSEK
ACT-17QSB73424.1_1103EBOC      ..................................MMKKSLCCALLLGISCSALATPVSEK
ACT-17QXY98988.1_1068EBOC      ..................................MMKKSLCCALLLGISCSALATPVSEK
ACT-17UDV73425.1_430EBOC       ..................................MMKKSLCCALLLGISCSALATPVSEK
ACT-17UDV69018.1_894EBOC       ..................................MMKKSLCCALLLGISCSALATPVSEK
ACT-17UKK21116.1_588EBOC       ..................................MMKKSLCCALLLGISCSALATPVSEK
ACT-17UOY44701.1_2566EBOC      ..................................MMKKSLCCALLLGISCSALATPVSEK
ACT-17URL69109.1_2030EBOC      ..................................MMKKSLCCALLLGISCSALATPVSEK
ACT-17UUQ70490.1_426EBOC       ..................................MMKKSLCCALLLGISCSALATPVSEK
ACT-17UWA70738.1_427EBOC       ..................................MMKKSLCCALLLGISCSALATPVSEK
ACT-17UWC98141.1_545EBOC       ..................................MMKKSLCCALLLGISCSALATPVSEK
ACT-17UXC72711.1_448EBOC       ..................................MMKKSLCCALLLGISCSALATPVSEK
ACT-17UXC77377.1_448EBOC       ..................................MMKKSLCCALLLGISCSALATPVSEK
ACT-17UXC82023.1_448EBOC       ..................................MMKKSLCCALLLGISCSALATPVSEK
ACT-17UZE41799.1_406EBOC       ..................................MMKKSLCCALLLGISCSALATPVSEK
ACT-17UZH49391.1_396EBOC       ..................................MMKKSLCCALLLGISCSALATPVSEK
ACT-17WAZ63377.1_405EBOC       ..................................MMKKSLCCALLLGISCSALATPVSEK
ACT-17WDU86092.1_3769EBOC      ..................................MMKKSLCCALLLGISCSALATPVSEK
ACT-17WDW12975.1_3764EBOC      ..................................MMKKSLCCALLLGISCSALATPVSEK
ACT-17WGA71117.1_396EBOC       ..................................MMKKSLCCALLLGISCSALATPVSEK
ACT-17WGA75603.1_396EBOC       ..................................MMKKSLCCALLLGISCSALATPVSEK
ACT-17WGZ57121.1_2667EBOC      ..................................MMKKSLCCALLLGISCSALATPVSEK
ACT-17QEI60759.1_389EOC        ..................................MMKKSLCCALLLGISCSALATPVSEK
ACT-17QEI70010.1_389EOC        ..................................MMKKSLCCALLLGISCSALATPVSEK
ACT-17QEU13386.1_612EOC        ..................................MMKKSLCCALLLGISCSALATPVSEK
ACT-17QHI59365.1_3674EOC       ..................................MMKKSLCCALLLGISCSALATPVSEK
ACT-17UZQ58984.1_410EOC        ..................................MMKKSLCCALLLGISCSALATPVSEK
ACT-40AWS79793.1_3058EBCP      ..................................MMKKSLCCALLLGISCSALATPVSEK
ACT-40AXO44745.1_1607EBCP      ..................................MMKKSLCCALLLGISCSALATPVSEK
ACT-40UXI63958.1_1746EBCP      ..................................MMKKSLCCALLLGISCSALATPVSEK
ACT-40WDC52451.1_4284EBCP      ..................................MMKKSLCCALLLGISCSALATPVSEK
ACT-40AWS79793.1_3058EBOC      ..................................MMKKSLCCALLLGISCSALATPVSEK
ACT-40AXO44745.1_1607EBOC      ..................................MMKKSLCCALLLGISCSALATPVSEK
ACT-40UXI63958.1_1746EBOC      ..................................MMKKSLCCALLLGISCSALATPVSEK
ACT-40WDC52451.1_4284EBOC      ..................................MMKKSLCCALLLGISCSALATPVSEK
ACT-44UWA09346.1_3724EBCP      ..................................MMKKSLCCALLLGISCSALATPVSEK
ACT-44UWA09346.1_3724EBOC      ..................................MMKKSLCCALLLGISCSALATPVSEK
ACT-90QFH83806.1_450EBCP       ..................................MMKKSLCCALLLGISCSALAAPVSEK
ACT-90QLO92194.1_500EBCP       ..................................MMKKSLCCALLLGISCSALAAPVSEK
ACT-90QLP01381.1_466EBCP       ..................................MMKKSLCCALLLGISCSALAAPVSEK
ACT-90QFH83806.1_450EBOC       ..................................MMKKSLCCALLLGISCSALAAPVSEK
ACT-90QLO92194.1_500EBOC       ..................................MMKKSLCCALLLGISCSALAAPVSEK
ACT-90QLP01381.1_466EBOC       ..................................MMKKSLCCALLLGISCSALAAPVSEK
ACT-24ASB73507.1_1185EBCP      ..................................MMKKSLCCALLLGISCSALAAPVSEK
ACT-24AVU18445.1_378EBCP       ..................................MMKKSLCCALLLGISCSALAAPVSEK
```

```
ACT-24QHC75881.1_374EBCP       ...........................................MMKKSLCCALLLGISCSALAAPVSEK
ACT-24QLP84500.1_370EBCP       ...........................................MMKKSLCCALLLGISCSALAAPVSEK
ACT-24QSH03071.1_370EBCP       ...........................................MMKKSLCCALLLGISCSALAAPVSEK
ACT-24QSG93844.1_370EBCP       ...........................................MMKKSLCCALLLGISCSALAAPVSEK
ACT-24QSG98466.1_370EBCP       ...........................................MMKKSLCCALLLGISCSALAAPVSEK
ACT-24QSH12187.1_370EBCP       ...........................................MMKKSLCCALLLGISCSALAAPVSEK
ACT-24QSH16801.1_370EBCP       ...........................................MMKKSLCCALLLGISCSALAAPVSEK
ACT-24QSH07573.1_370EBCP       ...........................................MMKKSLCCALLLGISCSALAAPVSEK
ACT-24QSH21409.1_370EBCP       ...........................................MMKKSLCCALLLGISCSALAAPVSEK
ACT-24QSH26023.1_370EBCP       ...........................................MMKKSLCCALLLGISCSALAAPVSEK
ACT-24QSH30639.1_370EBCP       ...........................................MMKKSLCCALLLGISCSALAAPVSEK
ACT-24UIL48372.1_372EBCP       ...........................................MMKKSLCCALLLGISCSALAAPVSEK
ACT-24UIM14687.1_372EBCP       ...........................................MMKKSLCCALLLGISCSALAAPVSEK
ACT-24UXA74146.1_371EBCP       ...........................................MMKKSLCCALLLGISCSALAAPVSEK
ACT-24WDY48721.1_372EBCP       ...........................................MMKKSLCCALLLGISCSALAAPVSEK
ACT-24ASB73507.1_1185EBOC      ...........................................MMKKSLCCALLLGISCSALAAPVSEK
ACT-24AVU18445.1_378EBOC       ...........................................MMKKSLCCALLLGISCSALAAPVSEK
ACT-24QHC75881.1_374EBOC       ...........................................MMKKSLCCALLLGISCSALAAPVSEK
ACT-24QLP84500.1_370EBOC       ...........................................MMKKSLCCALLLGISCSALAAPVSEK
ACT-24QSH03071.1_370EBOC       ...........................................MMKKSLCCALLLGISCSALAAPVSEK
ACT-24QSG93844.1_370EBOC       ...........................................MMKKSLCCALLLGISCSALAAPVSEK
ACT-24QSG98466.1_370EBOC       ...........................................MMKKSLCCALLLGISCSALAAPVSEK
ACT-24QSH12187.1_370EBOC       ...........................................MMKKSLCCALLLGISCSALAAPVSEK
ACT-24QSH16801.1_370EBOC       ...........................................MMKKSLCCALLLGISCSALAAPVSEK
ACT-24QSH07573.1_370EBOC       ...........................................MMKKSLCCALLLGISCSALAAPVSEK
ACT-24QSH21409.1_370EBOC       ...........................................MMKKSLCCALLLGISCSALAAPVSEK
ACT-24QSH26023.1_370EBOC       ...........................................MMKKSLCCALLLGISCSALAAPVSEK
ACT-24QSH30639.1_370EBOC       ...........................................MMKKSLCCALLLGISCSALAAPVSEK
ACT-24UIL48372.1_372EBOC       ...........................................MMKKSLCCALLLGISCSALAAPVSEK
ACT-24UIM14687.1_372EBOC       ...........................................MMKKSLCCALLLGISCSALAAPVSEK
ACT-24UXA74146.1_371EBOC       ...........................................MMKKSLCCALLLGISCSALAAPVSEK
ACT-24WDY48721.1_372EBOC       ...........................................MMKKSLCCALLLGISCSALAAPVSEK
ACT-89AVE73204.1_2453EBCP      ...........................................MMRKSLCCALLLGISCSALATPVSEK
ACT-89AVE73204.1_2453EBOC      ...........................................MMRKSLCCALLLGISCSALATPVSEK
ACT-56ASO99603.1_1270EBCP      ...........................................MMKKSLCCALLLGISCSALATPVSEK
ACT-56QDQ75241.1_392EBCP       ...........................................MMKKSLCCALLLGISCSALATPVSEK
ACT-56QFH98918.1_395EBCP       ...........................................MMKKSLCCALLLGISCSALATPVSEK
ACT-56QFH34334.1_395EBCP       ...........................................MMKKSLCCALLLGISCSALATPVSEK
ACT-56QFI32707.1_2233EBCP      ...........................................MMKKSLCCALLLGISCSALATPVSEK
ACT-56QMI65633.1_4184EBCP      ...........................................MMKKSLCCALLLGISCSALATPVSEK
ACT-56QMI71192.1_422EBCP       ...........................................MMKKSLCCALLLGISCSALATPVSEK
ACT-56UDV17856.1_8EBCP         ...........................................MMKKSLCCALLLGISCSALATPVSEK
ACT-56UDV39132.1_1376EBCP      ...........................................MMKKSLCCALLLGISCSALATPVSEK
ACT-56UWB10427.1_430EBCP       ...........................................MMKKSLCCALLLGISCSALATPVSEK
ACT-56UWC18315.1_415EBCP       ...........................................MMKKSLCCALLLGISCSALATPVSEK
ACT-56UWC23093.1_417EBCP       ...........................................MMKKSLCCALLLGISCSALATPVSEK
ACT-56WBT12607.1_393EBCP       ...........................................MMKKSLCCALLLGISCSALATPVSEK
ACT-56WBT28831.1_393EBCP       ...........................................MMKKSLCCALLLGISCSALATPVSEK
ACT-56WGB34617.1_392EBCP       ...........................................MMKKSLCCALLLGISCSALATPVSEK
ACT-56WGU03466.1_393EBCP       ...........................................MMKKSLCCALLLGISCSALATPVSEK
ACT-56ASO99603.1_1270EBOC      ...........................................MMKKSLCCALLLGISCSALATPVSEK
ACT-56QDQ75241.1_392EBOC       ...........................................MMKKSLCCALLLGISCSALATPVSEK
ACT-56QFH98918.1_395EBOC       ...........................................MMKKSLCCALLLGISCSALATPVSEK
ACT-56QFH34334.1_395EBOC       ...........................................MMKKSLCCALLLGISCSALATPVSEK
ACT-56QFI32707.1_2233EBOC      ...........................................MMKKSLCCALLLGISCSALATPVSEK
ACT-56QMI65633.1_4184EBOC      ...........................................MMKKSLCCALLLGISCSALATPVSEK
ACT-56QMI71192.1_422EBOC       ...........................................MMKKSLCCALLLGISCSALATPVSEK
ACT-56UDV17856.1_8EBOC         ...........................................MMKKSLCCALLLGISCSALATPVSEK
ACT-56UDV39132.1_1376EBOC      ...........................................MMKKSLCCALLLGISCSALATPVSEK
ACT-56UWB10427.1_430EBOC       ...........................................MMKKSLCCALLLGISCSALATPVSEK
ACT-56UWC18315.1_415EBOC       ...........................................MMKKSLCCALLLGISCSALATPVSEK
ACT-56UWC23093.1_417EBOC       ...........................................MMKKSLCCALLLGISCSALATPVSEK
ACT-56WBT12607.1_393EBOC       ...........................................MMKKSLCCALLLGISCSALATPVSEK
ACT-56WBT28831.1_393EBOC       ...........................................MMKKSLCCALLLGISCSALATPVSEK
ACT-56WGB34617.1_392EBOC       ...........................................MMKKSLCCALLLGISCSALATPVSEK
ACT-56WGU03466.1_393EBOC       ...........................................MMKKSLCCALLLGISCSALATPVSEK
ACT-56AOP76464.1_392EOC        ...........................................MMKKSLCCALLLGISCSALATPVSEK
ACT-56QXA77046.1_2672EOC       ...........................................MMKKSLCCALLLGISCSALATPVSEK
ACT-56WFP29817.1_397EOC        ...........................................MMKKSLCCALLLGISCSALATPVSEK
ACT-27AXO49795.1_1610EBCP      ...........................................MMKKSLCCALLLGISCSALAAPVSEK
ACT-27QHO79915.1_457EBCP       ...........................................MMKKSLCCALLLGISCSALAAPVSEK
ACT-27QHO97012.1_458EBCP       ...........................................MMKKSLCCALLLGISCSALAAPVSEK
ACT-27QQH03712.1_3802EBCP      ...........................................MMKKSLCCALLLGISCSALAAPVSEK
ACT-27QQH08784.1_3802EBCP      ...........................................MMKKSLCCALLLGISCSALAAPVSEK
ACT-27UVZ91365.1_388EBCP       ...........................................MMKKSLCCALLLGISCSALAAPVSEK
ACT-27AXO49795.1_1610EBOC      ...........................................MMKKSLCCALLLGISCSALAAPVSEK
ACT-27QHO79915.1_457EBOC       ...........................................MMKKSLCCALLLGISCSALAAPVSEK
ACT-27QHO97012.1_458EBOC       ...........................................MMKKSLCCALLLGISCSALAAPVSEK
ACT-27QQH03712.1_3802EBOC      ...........................................MMKKSLCCALLLGISCSALAAPVSEK
ACT-27QQH08784.1_3802EBOC      ...........................................MMKKSLCCALLLGISCSALAAPVSEK
ACT-27UVZ91365.1_388EBOC       ...........................................MMKKSLCCALLLGISCSALAAPVSEK
ACT-102QEO03155.1_4213EBCP    ...........................................MKTKSLCCALLLSTSCSVLAAPMSEK
ACT-102BBV79841.1_51928]EBCP  ...........................................MKTKSLCCALLLSTSCSVLAAPMSEK
ACT-102QZS47739.1_401EBCP     ...........................................MKTKSLCCALLLSTSCSVLAAPMSEK
ACT-102QEO03155.1_4213EBOC    ...........................................MKTKSLCCALLLSTSCSVLAAPMSEK
ACT-102BBV79841.1_51928]EBOC  ...........................................MKTKSLCCALLLSTSCSVLAAPMSEK
ACT-102QZS47739.1_401EBOC     ...........................................MKTKSLCCALLLSTSCSVLAAPMSEK
ACT-55QLP71015.1_413EBCP       ...........................................MMKKSLCCALLLGISCSALAAPVSEK
ACT-55QLP80034.1_414EBCP       ...........................................MMKKSLCCALLLGISCSALAAPVSEK
ACT-55QLS08818.1_415EBCP       ...........................................MMKKSLCCALLLGISCSALAAPVSEK
ACT-55QLT25377.1_3621EBCP      ...........................................MMKKSLCCALLLGISCSALAAPVSEK
ACT-55QLV12510.1_4006EBCP      ...........................................MMKKSLCCALLLGISCSALAAPVSEK
ACT-55QLW04180.1_4182EBCP      ...........................................MMKKSLCCALLLGISCSALAAPVSEK
ACT-55QLV08026.1_4006EBCP      ...........................................MMKKSLCCALLLGISCSALAAPVSEK
ACT-55QLV96171.1_421EBCP       ...........................................MMKKSLCCALLLGISCSALAAPVSEK
ACT-55QLW44293.1_405EBCP       ...........................................MMKKSLCCALLLGISCSALAAPVSEK
ACT-55UBH32480.1_675EBCP       ...........................................MMKKSLCCALLLGISCSALAAPVSEK
ACT-55UDV61223.1_2251EBCP      ...........................................MMKKSLCCALLLGISCSALAAPVSEK
ACT-55QLP71015.1_413EBOC       ...........................................MMKKSLCCALLLGISCSALAAPVSEK
ACT-55QLP80034.1_414EBOC       ...........................................MMKKSLCCALLLGISCSALAAPVSEK
ACT-55QLS08818.1_415EBOC       ...........................................MMKKSLCCALLLGISCSALAAPVSEK
ACT-55QLT25377.1_3621EBOC      ...........................................MMKKSLCCALLLGISCSALAAPVSEK
ACT-55QLV12510.1_4006EBOC      ...........................................MMKKSLCCALLLGISCSALAAPVSEK
```

```
ACT-55QLW04180.1_4182EBOC     ........................................MMKKSLCCALLLGISCSALAAPVSEK
ACT-55QLV08026.1_4006EBOC     ........................................MMKKSLCCALLLGISCSALAAPVSEK
ACT-55QLV96171.1_421EBOC      ........................................MMKKSLCCALLLGISCSALAAPVSEK
ACT-55QLW44293.1_405EBOC      ........................................MMKKSLCCALLLGISCSALAAPVSEK
ACT-55UBH32480.1_675EBOC      ........................................MMKKSLCCALLLGISCSALAAPVSEK
ACT-55UDV61223.1_2251EBOC     ........................................MMKKSLCCALLLGISCSALAAPVSEK
ACT-55AOP80857.1_409EOC       ........................................MMKKSLCCALLLGISCSALAAPVSEK
ACT-51BBW20048.1_38351]EBCP   ........................................MKTKSLCCALMLSTSCSVLAAPMSEK
ACT-51BBW20048.1_38351]EBOC   ........................................MKTKSLCCALMLSTSCSVLAAPMSEK
ACT-28URE95606.1_351EBCP      ........................................MKTKSLCCALLLSTSCSVLAAPMSEK
ACT-28URE95606.1_351EBOC      ........................................MKTKSLCCALLLSTSCSVLAAPMSEK
ACT-87AYL04658.1_1249EBCP     ........................................MKTKSLCCALLLSTSCSVLAAPMSEK
ACT-87QIP18425.1_352EBCP      ........................................MKTKSLCCALLLSTSCSVLAAPMSEK
ACT-87AYL04658.1_1249EBOC     ........................................MKTKSLCCALLLSTSCSVLAAPMSEK
ACT-87QIP18425.1_352EBOC      ........................................MKTKSLCCALLLSTSCSVLAAPMSEK
ACT-75AWX01586.1_1447EBCP     ........................................MMKKSLCCALLLGLSCSALAAPVSEK
ACT-75AWX01586.1_1447EBOC     ........................................MMKKSLCCALLLGLSCSALAAPVSEK
ACT-103URL27837.1_342EOC      ........................................MKTKSLFCALLLSTSCSVLAAPMSEK
ACT-99UXJ64669.1_346EBCP      ........................................MKTKSLCSALLLSTSCSVLAAPMSEK
ACT-99UXJ64669.1_346EBOC      ........................................MKTKSLCSALLLSTSCSVLAAPMSEK
ACT-95QIB80518.1_348EBCP      ........................................MKTKSLCCALLLSTSCSVLAAPMSEK
ACT-95BBV69349.1_35128]EBCP   ........................................MKTKSLCCALLLSTSCSVLAAPMSEK
ACT-95UZQ68133.1_388EBCP      ........................................MKTKSLCCALLLSTSCSVLAAPMSEK
ACT-95QIB80518.1_348EBOC      ........................................MKTKSLCCALLLSTSCSVLAAPMSEK
ACT-95BBV69349.1_35128]EBOC   ........................................MKTKSLCCALLLSTSCSVLAAPMSEK
ACT-95UZQ68133.1_388EBOC      ........................................MKTKSLCCALLLSTSCSVLAAPMSEK
ACT-52QFH88560.1_350EBCP      ........................................MKTKSLCCALLLSTSCSVLAAPMSEK
ACT-52BBS30224.1_43052]EBCP   ........................................MKTKSLCCALLLSTSCSVLAAPMSEK
ACT-52WBN86316.1_4083EBCP     ........................................MKTKSLCCALLLSTSCSVLAAPMSEK
ACT-52QFH88560.1_350EBOC      ........................................MKTKSLCCALLLSTSCSVLAAPMSEK
ACT-52BBS30224.1_43052]EBOC   ........................................MKTKSLCCALLLSTSCSVLAAPMSEK
ACT-52WBN86316.1_4083EBOC     ........................................MKTKSLCCALLLSTSCSVLAAPMSEK
ACT-4QLR27288.1_395EBCP       ........................................MMTKSLCCALLLSTSCSVLAAPMSEK
ACT-4QPS68257.1_413EBCP       ........................................MMTKSLCCALLLSTSCSVLAAPMSEK
ACT-4QLR27288.1_395EBOC       ........................................MMTKSLCCALLLSTSCSVLAAPMSEK
ACT-4QPS68257.1_413EBOC       ........................................MMTKSLCCALLLSTSCSVLAAPMSEK
ACT-3UOY53510.1_358EBCP       ........................................MMTKSLCCALLLSTSCSVLATPMSEK
ACT-3UOY63785.1_4171EBCP      ........................................MMTKSLCCALLLSTSCSVLATPMSEK
ACT-3UOY53510.1_358EBOC       ........................................MMTKSLCCALLLSTSCSVLATPMSEK
ACT-3UOY63785.1_4171EBOC      ........................................MMTKSLCCALLLSTSCSVLATPMSEK
ACT-1QLV61731.1_472EBCP       ........................................MMTKSLCCALLLSTSCSVLATPMSEK
ACT-1UWA77567.1_382EBCP       ........................................MMTKSLCCALLLSTSCSVLATPMSEK
ACT-1QLV61731.1_472EBOC       ........................................MMTKSLCCALLLSTSCSVLATPMSEK
ACT-1UWA77567.1_382EBOC       ........................................MMTKSLCCALLLSTSCSVLATPMSEK
ACT-77QWZ50133.1_330EBCP      ........................................MMKKFLCCALLLSTSCSVLAAPMSEK
ACT-77UBH39796.1_3999EBCP     ........................................MMKKFLCCALLLSTSCSVLAAPMSEK
ACT-77UBH98103.1_3842EBCP     ........................................MMKKFLCCALLLSTSCSVLAAPMSEK
ACT-77UBH94108.1_193EBCP      ........................................MMKKFLCCALLLSTSCSVLAAPMSEK
ACT-77QWZ50133.1_330EBOC      ........................................MMKKFLCCALLLSTSCSVLAAPMSEK
ACT-77UBH39796.1_3999EBOC     ........................................MMKKFLCCALLLSTSCSVLAAPMSEK
ACT-77UBH98103.1_3842EBOC     ........................................MMKKFLCCALLLSTSCSVLAAPMSEK
ACT-77UBH94108.1_193EBOC      ........................................MMKKFLCCALLLSTSCSVLAAPMSEK
ACT-53QEL38380.1_485EBCP      ........................................MMTKTLCCALLLSTSCSALAGPLSEK
ACT-53QEL38380.1_485EBOC      ........................................MMTKTLCCALLLSTSCSALAGPLSEK
ACT-57QBB07838.1_4703EBCP     ........................................MMTKSLCCALLLSTSCSVLAAPMSEK
ACT-57QYD27335.1_499EBCP      ........................................MMTKSLCCALLLSTSCSVLAAPMSEK
ACT-57UBM17197.1_2672EBCP     ........................................MMTKSLCCALLLSTSCSVLAAPMSEK
ACT-57UKB55021.1_380EBCP      ........................................MMTKSLCCALLLSTSCSVLAAPMSEK
ACT-57QBB07838.1_4703EBOC     ........................................MMTKSLCCALLLSTSCSVLAAPMSEK
ACT-57QYD27335.1_499EBOC      ........................................MMTKSLCCALLLSTSCSVLAAPMSEK
ACT-57UBM17197.1_2672EBOC     ........................................MMTKSLCCALLLSTSCSVLAAPMSEK
ACT-57UKB55021.1_380EBOC      ........................................MMTKSLCCALLLSTSCSVLAAPMSEK
ACT-93ALL15984.1_396EOC       ........................................MMTKSLCCALLLSTSCSVLAAPMSEK
ACT-9AOP85131.1_312EBCP       ........................................MKTKSLCCALLLSTSCSVLAAPMSEK
ACT-9UOY32628.1_343EBCP       ........................................MKTKSLCCALLLSTSCSVLAAPMSEK
ACT-9AOP85131.1_312EBOC       ........................................MKTKSLCCALLLSTSCSVLAAPMSEK
ACT-9UOY32628.1_343EBOC       ........................................MKTKSLCCALLLSTSCSVLAAPMSEK
ACT-9AMZ75637.1_173EOC        ........................................MKTKSLCCALLLSTSCSVLAAPMSEK
ACT-6BBT43181.1_3396]EBCP     ........................................MMTKSLCCALLLSTSCSVLAAPMSEK
ACT-6BBT43181.1_3396]EBOC     ........................................MMTKSLCCALLLSTSCSVLAAPMSEK
ACT-46QUP91302.1_387EBCP      ........................................MMKKSLCCALLLGLSCSALAAPVSEK
ACT-46QUP91302.1_387EBOC      ........................................MMKKSLCCALLLGLSCSALAAPVSEK
ACT-67QLP16520.1_400EBCP      ........................................MMKKSLCCALLLGISCSALAAPVSEK
ACT-67QLP16520.1_400EBOC      ........................................MMKKSLCCALLLGISCSALAAPVSEK
ACT-84QOR49295.1_413EBCP      ........................................MMKKSLCCALLLGLSCSALAAPVSEK
ACT-84QOR49295.1_413EBOC      ........................................MMKKSLCCALLLGLSCSALAAPVSEK
ACT-74QOX68946.1_496EBCP      ........................................MMKKSLCCALLLGISCSALATPVSEK
ACT-74UIZ04332.1_460EBCP      ........................................MMKKSLCCALLLGISCSALATPVSEK
ACT-74QOX68946.1_496EBOC      ........................................MMKKSLCCALLLGISCSALATPVSEK
ACT-74UIZ04332.1_460EBOC      ........................................MMKKSLCCALLLGISCSALATPVSEK
ACT-74QQT40108.1_4574EOC      ........................................MMKKSLCCALLLGISCSALATPVSEK
ACT-16ATW90451.1_311EBCP      ........................................MMKKSLCCALLLGLSCSALAAPVSEK
ACT-16AVO82105.1_1257EBCP     ........................................MMKKSLCCALLLGLSCSALAAPVSEK
ACT-16AWQ41825.1_393EBCP      ........................................MMKKSLCCALLLGLSCSALAAPVSEK
ACT-16AWQ56038.1_393EBCP      ........................................MMKKSLCCALLLGLSCSALAAPVSEK
ACT-16QFH53420.1_474EBCP      ........................................MMKKSLCCALLLGLSCSALAAPVSEK
ACT-16QPO90952.1_396EBCP      ........................................MMKKSLCCALLLGLSCSALAAPVSEK
ACT-16QQA67679.1_472EBCP      ........................................MMKKSLCCALLLGLSCSALAAPVSEK
ACT-16UDV14809.1_2173EBCP     ........................................MMKKSLCCALLLGLSCSALAAPVSEK
ACT-16UDV41468.1_3105EBCP     ........................................MMKKSLCCALLLGLSCSALAAPVSEK
ACT-16UIZ08994.1_395EBCP      ........................................MMKKSLCCALLLGLSCSALAAPVSEK
ACT-16WJJ28417.1_386EBCP      ........................................MMKKSLCCALLLGLSCSALAAPVSEK
ACT-16ATW90451.1_311EBOC      ........................................MMKKSLCCALLLGLSCSALAAPVSEK
ACT-16AVO82105.1_1257EBOC     ........................................MMKKSLCCALLLGLSCSALAAPVSEK
ACT-16AWQ41825.1_393EBOC      ........................................MMKKSLCCALLLGLSCSALAAPVSEK
ACT-16AWQ56038.1_393EBOC      ........................................MMKKSLCCALLLGLSCSALAAPVSEK
ACT-16QFH53420.1_474EBOC      ........................................MMKKSLCCALLLGLSCSALAAPVSEK
ACT-16QPO90952.1_396EBOC      ........................................MMKKSLCCALLLGLSCSALAAPVSEK
ACT-16QQA67679.1_472EBOC      ........................................MMKKSLCCALLLGLSCSALAAPVSEK
ACT-16UDV14809.1_2173EBOC     ........................................MMKKSLCCALLLGLSCSALAAPVSEK
ACT-16UDV41468.1_3105EBOC     ........................................MMKKSLCCALLLGLSCSALAAPVSEK
ACT-16UIZ08994.1_395EBOC      ........................................MMKKSLCCALLLGLSCSALAAPVSEK
ACT-16WJJ28417.1_386EBOC      ........................................MMKKSLCCALLLGLSCSALAAPVSEK
```

```
ACT-23QLP51667.1_388EBCP      .......................................MMKKSLCCALLLGISCSALAAPVSEK
ACT-23QLP61663.1_388EBCP      .......................................MMKKSLCCALLLGISCSALAAPVSEK
ACT-23QLP66427.1_430EBCP      .......................................MMKKSLCCALLLGISCSALAAPVSEK
ACT-23QLU80799.1_388EBCP      .......................................MMKKSLCCALLLGISCSALAAPVSEK
ACT-23QLT97825.1_367EBCP      .......................................MMKKSLCCALLLGISCSALAAPVSEK
ACT-23QLU89187.1_4307EBCP     .......................................MMKKSLCCALLLGISCSALAAPVSEK
ACT-23QLV76440.1_389EBCP      .......................................MMKKSLCCALLLGISCSALAAPVSEK
ACT-23QLT57991.1_396EBCP      .......................................MMKKSLCCALLLGISCSALAAPVSEK
ACT-23QLV44058.1_369EBCP      .......................................MMKKSLCCALLLGISCSALAAPVSEK
ACT-23QLP51667.1_388EBOC      .......................................MMKKSLCCALLLGISCSALAAPVSEK
ACT-23QLP61663.1_388EBOC      .......................................MMKKSLCCALLLGISCSALAAPVSEK
ACT-23QLP66427.1_430EBOC      .......................................MMKKSLCCALLLGISCSALAAPVSEK
ACT-23QLU80799.1_388EBOC      .......................................MMKKSLCCALLLGISCSALAAPVSEK
ACT-23QLT97825.1_367EBOC      .......................................MMKKSLCCALLLGISCSALAAPVSEK
ACT-23QLU89187.1_4307EBOC     .......................................MMKKSLCCALLLGISCSALAAPVSEK
ACT-23QLV76440.1_389EBOC      .......................................MMKKSLCCALLLGISCSALAAPVSEK
ACT-23QLT57991.1_396EBOC      .......................................MMKKSLCCALLLGISCSALAAPVSEK
ACT-23QLV44058.1_369EBOC      .......................................MMKKSLCCALLLGISCSALAAPVSEK
ACT-115AXQ35263.1_2972EBCP    .......................................MMKKSLCCALLLGLSCSALAAPVSEK
ACT-115QGR11187.1_384EBCP     .......................................MMKKSLCCALLLGLSCSALAAPVSEK
ACT-115QIF26741.1_2106EBCP    .......................................MMKKSLCCALLLGLSCSALAAPVSEK
ACT-115QIF32246.1_3030EBCP    .......................................MMKKSLCCALLLGLSCSALAAPVSEK
ACT-115QOD35429.1_385EBCP     .......................................MMKKSLCCALLLGLSCSALAAPVSEK
ACT-115QPD66621.1_385EBCP     .......................................MMKKSLCCALLLGLSCSALAAPVSEK
ACT-115QSS35407.1_385EBCP     .......................................MMKKSLCCALLLGLSCSALAAPVSEK
ACT-115URE88756.1_385EBCP     .......................................MMKKSLCCALLLGLSCSALAAPVSEK
ACT-115USM81982.1_384EBCP     .......................................MMKKSLCCALLLGLSCSALAAPVSEK
ACT-115AXQ35263.1_2972EBOC    .......................................MMKKSLCCALLLGLSCSALAAPVSEK
ACT-115QGR11187.1_384EBOC     .......................................MMKKSLCCALLLGLSCSALAAPVSEK
ACT-115QIF26741.1_2106EBOC    .......................................MMKKSLCCALLLGLSCSALAAPVSEK
ACT-115QIF32246.1_3030EBOC    .......................................MMKKSLCCALLLGLSCSALAAPVSEK
ACT-115QOD35429.1_385EBOC     .......................................MMKKSLCCALLLGLSCSALAAPVSEK
ACT-115QPD66621.1_385EBOC     .......................................MMKKSLCCALLLGLSCSALAAPVSEK
ACT-115QSS35407.1_385EBOC     .......................................MMKKSLCCALLLGLSCSALAAPVSEK
ACT-115URE88756.1_385EBOC     .......................................MMKKSLCCALLLGLSCSALAAPVSEK
ACT-115USM81982.1_384EBOC     .......................................MMKKSLCCALLLGLSCSALAAPVSEK
ACT-25AVU48988.1_427EBCP      .......................................MMKKSLCCALLLGLSCSALAAPVSEK
ACT-25UQQ46893.1_379EBCP      .......................................MMKKSLCCALLLGLSCSALAAPVSEK
ACT-25WDO17280.1_310EBCP      .......................................MMKKSLCCALLLGLSCSALAAPVSEK
ACT-25AVU48988.1_427EBOC      .......................................MMKKSLCCALLLGLSCSALAAPVSEK
ACT-25UQQ46893.1_379EBOC      .......................................MMKKSLCCALLLGLSCSALAAPVSEK
ACT-25WDO17280.1_310EBOC      .......................................MMKKSLCCALLLGLSCSALAAPVSEK
ACT-25AOP89724.1_377EOC       .......................................MMKKSLCCALLLGLSCSALAAPVSEK
ACT-45APR43249.1_2902EBCP     .......................................MMKKSLCCALLLGLSCSALAAPVSEK
ACT-45ASA05885.1_3853EBCP     .......................................MMKKSLCCALLLGLSCSALAAPVSEK
ACT-45ASB85359.1_3592EBCP     .......................................MMKKSLCCALLLGLSCSALAAPVSEK
ACT-45AWR70714.1_4154EBCP     .......................................MMKKSLCCALLLGLSCSALAAPVSEK
ACT-45AXM01511.1_4138EBCP     .......................................MMKKSLCCALLLGLSCSALAAPVSEK
ACT-45AYU96034.1_2649EBCP     .......................................MMKKSLCCALLLGLSCSALAAPVSEK
ACT-45QBH65043.1_3960EBCP     .......................................MMKKSLCCALLLGLSCSALAAPVSEK
ACT-45QEL31100.1_1855EBCP     .......................................MMKKSLCCALLLGLSCSALAAPVSEK
ACT-45ULQ22907.1_427EBCP      .......................................MMKKSLCCALLLGLSCSALAAPVSEK
ACT-45ULQ25748.1_3478EBCP     .......................................MMKKSLCCALLLGLSCSALAAPVSEK
ACT-45WCO72956.1_42EBCP       .......................................MMKKSLCCALLLGLSCSALAAPVSEK
ACT-45APR43249.1_2902EBOC     .......................................MMKKSLCCALLLGLSCSALAAPVSEK
ACT-45ASA05885.1_3853EBOC     .......................................MMKKSLCCALLLGLSCSALAAPVSEK
ACT-45ASB85359.1_3592EBOC     .......................................MMKKSLCCALLLGLSCSALAAPVSEK
ACT-45AWR70714.1_4154EBOC     .......................................MMKKSLCCALLLGLSCSALAAPVSEK
ACT-45AXM01511.1_4138EBOC     .......................................MMKKSLCCALLLGLSCSALAAPVSEK
ACT-45AYU96034.1_2649EBOC     .......................................MMKKSLCCALLLGLSCSALAAPVSEK
ACT-45QBH65043.1_3960EBOC     .......................................MMKKSLCCALLLGLSCSALAAPVSEK
ACT-45QEL31100.1_1855EBOC     .......................................MMKKSLCCALLLGLSCSALAAPVSEK
ACT-45ULQ22907.1_427EBOC      .......................................MMKKSLCCALLLGLSCSALAAPVSEK
ACT-45ULQ25748.1_3478EBOC     .......................................MMKKSLCCALLLGLSCSALAAPVSEK
ACT-45WCO72956.1_42EBOC       .......................................MMKKSLCCALLLGLSCSALAAPVSEK
ACT-C197QLA02472.1_2022EOC    .......................................MMKKSLCCALLLGISCSALAAPVSEK
ACT-64QMT06026.1_4255EBCP     .......................................MKTKSLCCALLLSTSCSVLAAPMSEK
ACT-64QMT06026.1_4255EBOC     .......................................MKTKSLCCALLLSTSCSVLAAPMSEK
ACT-C198QXA51435.1_2071EOC    .......................................MKKKLVFCALLLGVSCSALASSLSEK
                         acc
```

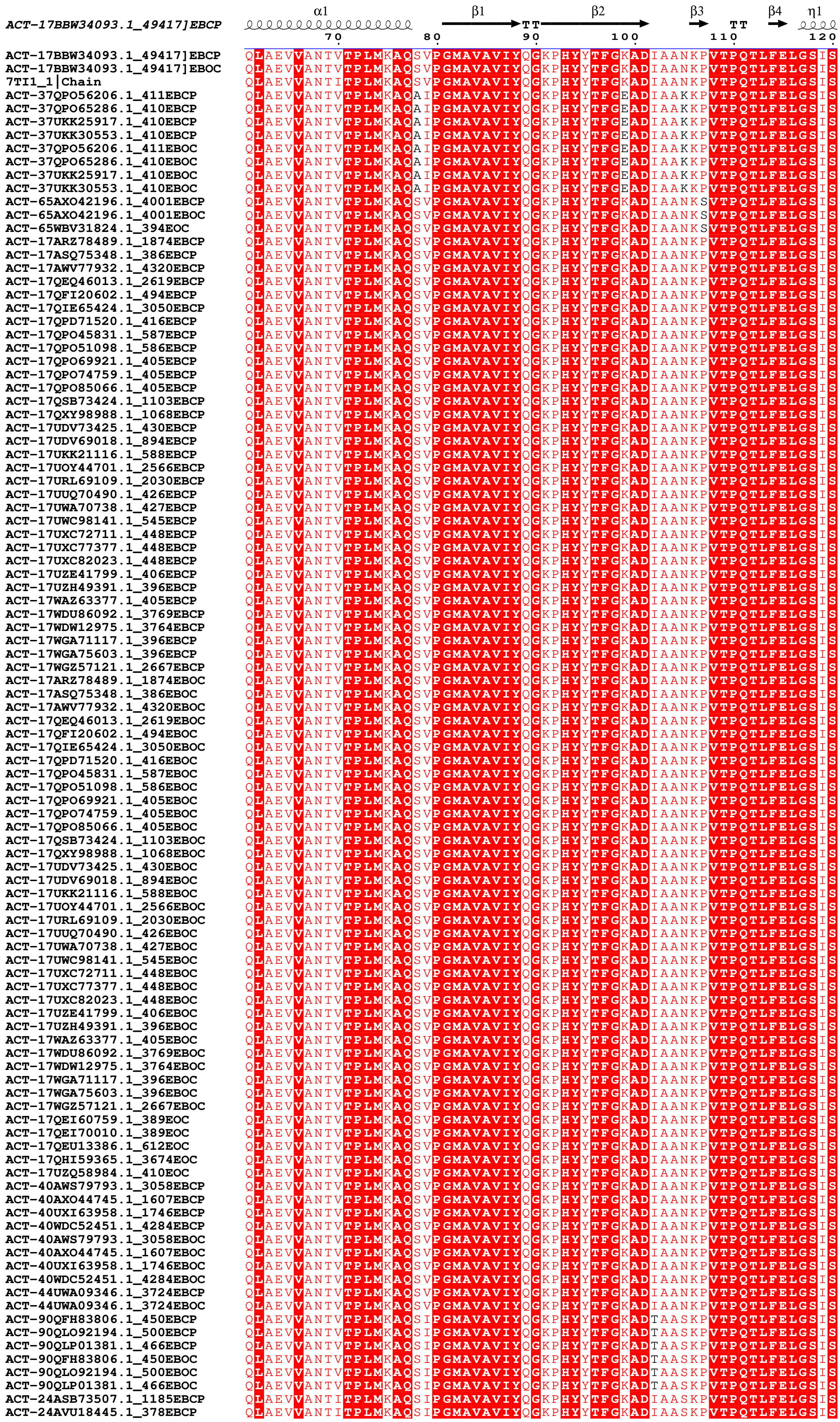
ACT-17BBW34093.1_49417]EBCP
α1 β1 β2 β3 β4 η1
TT TT
70 80 90 100 110 120
ACT-17BBW34093.1_49417]EBCP QLAEVVANTVTPLMKAQSVPGMAVAVIYQGKPHYYTFGKADIAANKPVTPQTLFELGSIS
ACT-17BBW34093.1_49417]EBOC QLAEVVANTVTPLMKAQSVPGMAVAVIYQGKPHYYTFGKADIAANKPVTPQTLFELGSIS
7TI1_1|Chain QLAEVVANTITPLMKAQSVPGMAVAVIYQGKPHYYTFGKADIAANKPVTPQTLFELGSIS
ACT-37QPO56206.1_411EBCP QLAEVVANTVTPLMKAQAIPGMAVAVIYQGKPHYYTFGEADIAAKKPVTPQTLFELGSIS
ACT-37QPO65286.1_410EBCP QLAEVVANTVTPLMKAQAIPGMAVAVIYQGKPHYYTFGEADIAAKKPVTPQTLFELGSIS
ACT-37UKK25917.1_410EBCP QLAEVVANTVTPLMKAQAIPGMAVAVIYQGKPHYYTFGEADIAAKKPVTPQTLFELGSIS
ACT-37UKK30553.1_410EBCP QLAEVVANTVTPLMKAQAIPGMAVAVIYQGKPHYYTFGEADIAAKKPVTPQTLFELGSIS
ACT-37QPO56206.1_411EBOC QLAEVVANTVTPLMKAQAIPGMAVAVIYQGKPHYYTFGEADIAAKKPVTPQTLFELGSIS
ACT-37QPO65286.1_410EBOC QLAEVVANTVTPLMKAQAIPGMAVAVIYQGKPHYYTFGEADIAAKKPVTPQTLFELGSIS
ACT-37UKK25917.1_410EBOC QLAEVVANTVTPLMKAQAIPGMAVAVIYQGKPHYYTFGEADIAAKKPVTPQTLFELGSIS
ACT-37UKK30553.1_410EBOC QLAEVVANTVTPLMKAQAIPGMAVAVIYQGKPHYYTFGEADIAAKKPVTPQTLFELGSIS
ACT-65AXO42196.1_4001EBCP QLAEVVANTVTPLMKAQSVPGMAVAVIYQGKPHYYTFGKADIAANKSVTPQTLFELGSIS
ACT-65AXO42196.1_4001EBOC QLAEVVANTVTPLMKAQSVPGMAVAVIYQGKPHYYTFGKADIAANKSVTPQTLFELGSIS
ACT-65WBV31824.1_394EOC QLAEVVANTVTPLMKAQSVPGMAVAVIYQGKPHYYTFGKADIAANKSVTPQTLFELGSIS
ACT-17ARZ78489.1_1874EBCP QLAEVVANTVTPLMKAQSVPGMAVAVIYQGKPHYYTFGKADIAANKPVTPQTLFELGSIS
ACT-17ASQ75348.1_386EBCP QLAEVVANTVTPLMKAQSVPGMAVAVIYQGKPHYYTFGKADIAANKPVTPQTLFELGSIS
ACT-17AWV77932.1_4320EBCP QLAEVVANTVTPLMKAQSVPGMAVAVIYQGKPHYYTFGKADIAANKPVTPQTLFELGSIS
ACT-17QEQ46013.1_2619EBCP QLAEVVANTVTPLMKAQSVPGMAVAVIYQGKPHYYTFGKADIAANKPVTPQTLFELGSIS
ACT-17QFI20602.1_494EBCP QLAEVVANTVTPLMKAQSVPGMAVAVIYQGKPHYYTFGKADIAANKPVTPQTLFELGSIS
ACT-17QIE65424.1_3050EBCP QLAEVVANTVTPLMKAQSVPGMAVAVIYQGKPHYYTFGKADIAANKPVTPQTLFELGSIS
ACT-17QPD71520.1_416EBCP QLAEVVANTVTPLMKAQSVPGMAVAVIYQGKPHYYTFGKADIAANKPVTPQTLFELGSIS
ACT-17QPO45831.1_587EBCP QLAEVVANTVTPLMKAQSVPGMAVAVIYQGKPHYYTFGKADIAANKPVTPQTLFELGSIS
ACT-17QPO51098.1_586EBCP QLAEVVANTVTPLMKAQSVPGMAVAVIYQGKPHYYTFGKADIAANKPVTPQTLFELGSIS
ACT-17QPO69921.1_405EBCP QLAEVVANTVTPLMKAQSVPGMAVAVIYQGKPHYYTFGKADIAANKPVTPQTLFELGSIS
ACT-17QPO74759.1_405EBCP QLAEVVANTVTPLMKAQSVPGMAVAVIYQGKPHYYTFGKADIAANKPVTPQTLFELGSIS
ACT-17QPO85066.1_405EBCP QLAEVVANTVTPLMKAQSVPGMAVAVIYQGKPHYYTFGKADIAANKPVTPQTLFELGSIS
ACT-17QSB73424.1_1103EBCP QLAEVVANTVTPLMKAQSVPGMAVAVIYQGKPHYYTFGKADIAANKPVTPQTLFELGSIS
ACT-17QXY98988.1_1068EBCP QLAEVVANTVTPLMKAQSVPGMAVAVIYQGKPHYYTFGKADIAANKPVTPQTLFELGSIS
ACT-17UDV73425.1_430EBCP QLAEVVANTVTPLMKAQSVPGMAVAVIYQGKPHYYTFGKADIAANKPVTPQTLFELGSIS
ACT-17UDV69018.1_894EBCP QLAEVVANTVTPLMKAQSVPGMAVAVIYQGKPHYYTFGKADIAANKPVTPQTLFELGSIS
ACT-17UKK21116.1_588EBCP QLAEVVANTVTPLMKAQSVPGMAVAVIYQGKPHYYTFGKADIAANKPVTPQTLFELGSIS
ACT-17UOY44701.1_2566EBCP QLAEVVANTVTPLMKAQSVPGMAVAVIYQGKPHYYTFGKADIAANKPVTPQTLFELGSIS
ACT-17URL69109.1_2030EBCP QLAEVVANTVTPLMKAQSVPGMAVAVIYQGKPHYYTFGKADIAANKPVTPQTLFELGSIS
ACT-17UUQ70490.1_426EBCP QLAEVVANTVTPLMKAQSVPGMAVAVIYQGKPHYYTFGKADIAANKPVTPQTLFELGSIS
ACT-17UWA70738.1_427EBCP QLAEVVANTVTPLMKAQSVPGMAVAVIYQGKPHYYTFGKADIAANKPVTPQTLFELGSIS
ACT-17UWC98141.1_545EBCP QLAEVVANTVTPLMKAQSVPGMAVAVIYQGKPHYYTFGKADIAANKPVTPQTLFELGSIS
ACT-17UXC72711.1_448EBCP QLAEVVANTVTPLMKAQSVPGMAVAVIYQGKPHYYTFGKADIAANKPVTPQTLFELGSIS
ACT-17UXC77377.1_448EBCP QLAEVVANTVTPLMKAQSVPGMAVAVIYQGKPHYYTFGKADIAANKPVTPQTLFELGSIS
ACT-17UXC82023.1_448EBCP QLAEVVANTVTPLMKAQSVPGMAVAVIYQGKPHYYTFGKADIAANKPVTPQTLFELGSIS
ACT-17UZE41799.1_406EBCP QLAEVVANTVTPLMKAQSVPGMAVAVIYQGKPHYYTFGKADIAANKPVTPQTLFELGSIS
ACT-17UZH49391.1_396EBCP QLAEVVANTVTPLMKAQSVPGMAVAVIYQGKPHYYTFGKADIAANKPVTPQTLFELGSIS
ACT-17WAZ63377.1_405EBCP QLAEVVANTVTPLMKAQSVPGMAVAVIYQGKPHYYTFGKADIAANKPVTPQTLFELGSIS
ACT-17WDU86092.1_3769EBCP QLAEVVANTVTPLMKAQSVPGMAVAVIYQGKPHYYTFGKADIAANKPVTPQTLFELGSIS
ACT-17WDW12975.1_3764EBCP QLAEVVANTVTPLMKAQSVPGMAVAVIYQGKPHYYTFGKADIAANKPVTPQTLFELGSIS
ACT-17WGA71117.1_396EBCP QLAEVVANTVTPLMKAQSVPGMAVAVIYQGKPHYYTFGKADIAANKPVTPQTLFELGSIS
ACT-17WGA75603.1_396EBCP QLAEVVANTVTPLMKAQSVPGMAVAVIYQGKPHYYTFGKADIAANKPVTPQTLFELGSIS
ACT-17WGZ57121.1_2667EBCP QLAEVVANTVTPLMKAQSVPGMAVAVIYQGKPHYYTFGKADIAANKPVTPQTLFELGSIS
ACT-17ARZ78489.1_1874EBOC QLAEVVANTVTPLMKAQSVPGMAVAVIYQGKPHYYTFGKADIAANKPVTPQTLFELGSIS
ACT-17ASQ75348.1_386EBOC QLAEVVANTVTPLMKAQSVPGMAVAVIYQGKPHYYTFGKADIAANKPVTPQTLFELGSIS
ACT-17AWV77932.1_4320EBOC QLAEVVANTVTPLMKAQSVPGMAVAVIYQGKPHYYTFGKADIAANKPVTPQTLFELGSIS
ACT-17QEQ46013.1_2619EBOC QLAEVVANTVTPLMKAQSVPGMAVAVIYQGKPHYYTFGKADIAANKPVTPQTLFELGSIS
ACT-17QFI20602.1_494EBOC QLAEVVANTVTPLMKAQSVPGMAVAVIYQGKPHYYTFGKADIAANKPVTPQTLFELGSIS
ACT-17QIE65424.1_3050EBOC QLAEVVANTVTPLMKAQSVPGMAVAVIYQGKPHYYTFGKADIAANKPVTPQTLFELGSIS
ACT-17QPD71520.1_416EBOC QLAEVVANTVTPLMKAQSVPGMAVAVIYQGKPHYYTFGKADIAANKPVTPQTLFELGSIS
ACT-17QPO45831.1_587EBOC QLAEVVANTVTPLMKAQSVPGMAVAVIYQGKPHYYTFGKADIAANKPVTPQTLFELGSIS
ACT-17QPO51098.1_586EBOC QLAEVVANTVTPLMKAQSVPGMAVAVIYQGKPHYYTFGKADIAANKPVTPQTLFELGSIS
ACT-17QPO69921.1_405EBOC QLAEVVANTVTPLMKAQSVPGMAVAVIYQGKPHYYTFGKADIAANKPVTPQTLFELGSIS
ACT-17QPO74759.1_405EBOC QLAEVVANTVTPLMKAQSVPGMAVAVIYQGKPHYYTFGKADIAANKPVTPQTLFELGSIS
ACT-17QPO85066.1_405EBOC QLAEVVANTVTPLMKAQSVPGMAVAVIYQGKPHYYTFGKADIAANKPVTPQTLFELGSIS
ACT-17QSB73424.1_1103EBOC QLAEVVANTVTPLMKAQSVPGMAVAVIYQGKPHYYTFGKADIAANKPVTPQTLFELGSIS
ACT-17QXY98988.1_1068EBOC QLAEVVANTVTPLMKAQSVPGMAVAVIYQGKPHYYTFGKADIAANKPVTPQTLFELGSIS
ACT-17UDV73425.1_430EBOC QLAEVVANTVTPLMKAQSVPGMAVAVIYQGKPHYYTFGKADIAANKPVTPQTLFELGSIS
ACT-17UDV69018.1_894EBOC QLAEVVANTVTPLMKAQSVPGMAVAVIYQGKPHYYTFGKADIAANKPVTPQTLFELGSIS
ACT-17UKK21116.1_588EBOC QLAEVVANTVTPLMKAQSVPGMAVAVIYQGKPHYYTFGKADIAANKPVTPQTLFELGSIS
ACT-17UOY44701.1_2566EBOC QLAEVVANTVTPLMKAQSVPGMAVAVIYQGKPHYYTFGKADIAANKPVTPQTLFELGSIS
ACT-17URL69109.1_2030EBOC QLAEVVANTVTPLMKAQSVPGMAVAVIYQGKPHYYTFGKADIAANKPVTPQTLFELGSIS
ACT-17UUQ70490.1_426EBOC QLAEVVANTVTPLMKAQSVPGMAVAVIYQGKPHYYTFGKADIAANKPVTPQTLFELGSIS
ACT-17UWA70738.1_427EBOC QLAEVVANTVTPLMKAQSVPGMAVAVIYQGKPHYYTFGKADIAANKPVTPQTLFELGSIS
ACT-17UWC98141.1_545EBOC QLAEVVANTVTPLMKAQSVPGMAVAVIYQGKPHYYTFGKADIAANKPVTPQTLFELGSIS
ACT-17UXC72711.1_448EBOC QLAEVVANTVTPLMKAQSVPGMAVAVIYQGKPHYYTFGKADIAANKPVTPQTLFELGSIS
ACT-17UXC77377.1_448EBOC QLAEVVANTVTPLMKAQSVPGMAVAVIYQGKPHYYTFGKADIAANKPVTPQTLFELGSIS
ACT-17UXC82023.1_448EBOC QLAEVVANTVTPLMKAQSVPGMAVAVIYQGKPHYYTFGKADIAANKPVTPQTLFELGSIS
ACT-17UZE41799.1_406EBOC QLAEVVANTVTPLMKAQSVPGMAVAVIYQGKPHYYTFGKADIAANKPVTPQTLFELGSIS
ACT-17UZH49391.1_396EBOC QLAEVVANTVTPLMKAQSVPGMAVAVIYQGKPHYYTFGKADIAANKPVTPQTLFELGSIS
ACT-17WAZ63377.1_405EBOC QLAEVVANTVTPLMKAQSVPGMAVAVIYQGKPHYYTFGKADIAANKPVTPQTLFELGSIS
ACT-17WDU86092.1_3769EBOC QLAEVVANTVTPLMKAQSVPGMAVAVIYQGKPHYYTFGKADIAANKPVTPQTLFELGSIS
ACT-17WDW12975.1_3764EBOC QLAEVVANTVTPLMKAQSVPGMAVAVIYQGKPHYYTFGKADIAANKPVTPQTLFELGSIS
ACT-17WGA71117.1_396EBOC QLAEVVANTVTPLMKAQSVPGMAVAVIYQGKPHYYTFGKADIAANKPVTPQTLFELGSIS
ACT-17WGA75603.1_396EBOC QLAEVVANTVTPLMKAQSVPGMAVAVIYQGKPHYYTFGKADIAANKPVTPQTLFELGSIS
ACT-17WGZ57121.1_2667EBOC QLAEVVANTVTPLMKAQSVPGMAVAVIYQGKPHYYTFGKADIAANKPVTPQTLFELGSIS
ACT-17QEI60759.1_389EOC QLAEVVANTVTPLMKAQSVPGMAVAVIYQGKPHYYTFGKADIAANKPVTPQTLFELGSIS
ACT-17QEI70010.1_389EOC QLAEVVANTVTPLMKAQSVPGMAVAVIYQGKPHYYTFGKADIAANKPVTPQTLFELGSIS
ACT-17QEU13386.1_612EOC QLAEVVANTVTPLMKAQSVPGMAVAVIYQGKPHYYTFGKADIAANKPVTPQTLFELGSIS
ACT-17QHI59365.1_3674EOC QLAEVVANTVTPLMKAQSVPGMAVAVIYQGKPHYYTFGKADIAANKPVTPQTLFELGSIS
ACT-17UZQ58984.1_410EOC QLAEVVANTVTPLMKAQSVPGMAVAVIYQGKPHYYTFGKADIAANKPVTPQTLFELGSIS
ACT-40AWS79793.1_3058EBCP QLAEVVANTVTPLMKAQSVPGMAVAVIYQGKPHYYTFGKADIAANKPVTPQTLFELGSIS
ACT-40AXO44745.1_1607EBCP QLAEVVANTVTPLMKAQSVPGMAVAVIYQGKPHYYTFGKADIAANKPVTPQTLFELGSIS
ACT-40UXI63958.1_1746EBCP QLAEVVANTVTPLMKAQSVPGMAVAVIYQGKPHYYTFGKADIAANKPVTPQTLFELGSIS
ACT-40WDC52451.1_4284EBCP QLAEVVANTVTPLMKAQSVPGMAVAVIYQGKPHYYTFGKADIAANKPVTPQTLFELGSIS
ACT-40AWS79793.1_3058EBOC QLAEVVANTVTPLMKAQSVPGMAVAVIYQGKPHYYTFGKADIAANKPVTPQTLFELGSIS
ACT-40AXO44745.1_1607EBOC QLAEVVANTVTPLMKAQSVPGMAVAVIYQGKPHYYTFGKADIAANKPVTPQTLFELGSIS
ACT-40UXI63958.1_1746EBOC QLAEVVANTVTPLMKAQSVPGMAVAVIYQGKPHYYTFGKADIAANKPVTPQTLFELGSIS
ACT-40WDC52451.1_4284EBOC QLAEVVANTVTPLMKAQSVPGMAVAVIYQGKPHYYTFGKADIAANKPVTPQTLFELGSIS
ACT-44UWA09346.1_3724EBCP QLAEVVANTVTPLMKAQSVPGMAVAVIYQGKPHYYTFGKADIAANKPVTPQTLFELGSIS
ACT-44UWA09346.1_3724EBOC QLAEVVANTVTPLMKAQSVPGMAVAVIYQGKPHYYTFGKADIAANKPVTPQTLFELGSIS
ACT-90QFH83806.1_450EBCP QLAEVVANTVTPLMKAQSIPGMAVAVIYQGKPHYYTFGKADTAASKPVTPQTLFELGSIS
ACT-90QLO92194.1_500EBCP QLAEVVANTVTPLMKAQSIPGMAVAVIYQGKPHYYTFGKADTAASKPVTPQTLFELGSIS
ACT-90QLP01381.1_466EBCP QLAEVVANTVTPLMKAQSIPGMAVAVIYQGKPHYYTFGKADTAASKPVTPQTLFELGSIS
ACT-90QFH83806.1_450EBOC QLAEVVANTVTPLMKAQSIPGMAVAVIYQGKPHYYTFGKADTAASKPVTPQTLFELGSIS
ACT-90QLO92194.1_500EBOC QLAEVVANTVTPLMKAQSIPGMAVAVIYQGKPHYYTFGKADTAASKPVTPQTLFELGSIS
ACT-90QLP01381.1_466EBOC QLAEVVANTVTPLMKAQSIPGMAVAVIYQGKPHYYTFGKADTAASKPVTPQTLFELGSIS
ACT-24ASB73507.1_1185EBCP QLAEVVANTITPLMKAQSIPGMAVAVIYQGKPHYYTFGKADIAASKPVTPQTLFELGSIS
ACT-24AVU18445.1_378EBCP QLAEVVANTITPLMKAQSIPGMAVAVIYQGKPHYYTFGKADIAASKPVTPQTLFELGSIS

ACT-24QHC75881.1_374EBCP	QLAEVVANTITPLMKAQSIPGMAVAVIYQGKPHYYTFGKADIAASKPVTPQTLFELGSIS
ACT-24QLP84500.1_370EBCP	QLAEVVANTITPLMKAQSIPGMAVAVIYQGKPHYYTFGKADIAASKPVTPQTLFELGSIS
ACT-24QSH03071.1_370EBCP	QLAEVVANTITPLMKAQSIPGMAVAVIYQGKPHYYTFGKADIAASKPVTPQTLFELGSIS
ACT-24QSG93844.1_370EBCP	QLAEVVANTITPLMKAQSIPGMAVAVIYQGKPHYYTFGKADIAASKPVTPQTLFELGSIS
ACT-24QSG98466.1_370EBCP	QLAEVVANTITPLMKAQSIPGMAVAVIYQGKPHYYTFGKADIAASKPVTPQTLFELGSIS
ACT-24QSH12187.1_370EBCP	QLAEVVANTITPLMKAQSIPGMAVAVIYQGKPHYYTFGKADIAASKPVTPQTLFELGSIS
ACT-24QSH16801.1_370EBCP	QLAEVVANTITPLMKAQSIPGMAVAVIYQGKPHYYTFGKADIAASKPVTPQTLFELGSIS
ACT-24QSH07573.1_370EBCP	QLAEVVANTITPLMKAQSIPGMAVAVIYQGKPHYYTFGKADIAASKPVTPQTLFELGSIS
ACT-24QSH21409.1_370EBCP	QLAEVVANTITPLMKAQSIPGMAVAVIYQGKPHYYTFGKADIAASKPVTPQTLFELGSIS
ACT-24QSH26023.1_370EBCP	QLAEVVANTITPLMKAQSIPGMAVAVIYQGKPHYYTFGKADIAASKPVTPQTLFELGSIS
ACT-24QSH30639.1_370EBCP	QLAEVVANTITPLMKAQSIPGMAVAVIYQGKPHYYTFGKADIAASKPVTPQTLFELGSIS
ACT-24UIL48372.1_372EBCP	QLAEVVANTITPLMKAQSIPGMAVAVIYQGKPHYYTFGKADIAASKPVTPQTLFELGSIS
ACT-24UIM14687.1_372EBCP	QLAEVVANTITPLMKAQSIPGMAVAVIYQGKPHYYTFGKADIAASKPVTPQTLFELGSIS
ACT-24UXA74146.1_371EBCP	QLAEVVANTITPLMKAQSIPGMAVAVIYQGKPHYYTFGKADIAASKPVTPQTLFELGSIS
ACT-24WDY48721.1_372EBCP	QLAEVVANTITPLMKAQSIPGMAVAVIYQGKPHYYTFGKADIAASKPVTPQTLFELGSIS
ACT-24ASB73507.1_1185EBOC	QLAEVVANTITPLMKAQSIPGMAVAVIYQGKPHYYTFGKADIAASKPVTPQTLFELGSIS
ACT-24AVU18445.1_378EBOC	QLAEVVANTITPLMKAQSIPGMAVAVIYQGKPHYYTFGKADIAASKPVTPQTLFELGSIS
ACT-24QHC75881.1_374EBOC	QLAEVVANTITPLMKAQSIPGMAVAVIYQGKPHYYTFGKADIAASKPVTPQTLFELGSIS
ACT-24QLP84500.1_370EBOC	QLAEVVANTITPLMKAQSIPGMAVAVIYQGKPHYYTFGKADIAASKPVTPQTLFELGSIS
ACT-24QSH03071.1_370EBOC	QLAEVVANTITPLMKAQSIPGMAVAVIYQGKPHYYTFGKADIAASKPVTPQTLFELGSIS
ACT-24QSG93844.1_370EBOC	QLAEVVANTITPLMKAQSIPGMAVAVIYQGKPHYYTFGKADIAASKPVTPQTLFELGSIS
ACT-24QSG98466.1_370EBOC	QLAEVVANTITPLMKAQSIPGMAVAVIYQGKPHYYTFGKADIAASKPVTPQTLFELGSIS
ACT-24QSH12187.1_370EBOC	QLAEVVANTITPLMKAQSIPGMAVAVIYQGKPHYYTFGKADIAASKPVTPQTLFELGSIS
ACT-24QSH16801.1_370EBOC	QLAEVVANTITPLMKAQSIPGMAVAVIYQGKPHYYTFGKADIAASKPVTPQTLFELGSIS
ACT-24QSH07573.1_370EBOC	QLAEVVANTITPLMKAQSIPGMAVAVIYQGKPHYYTFGKADIAASKPVTPQTLFELGSIS
ACT-24QSH21409.1_370EBOC	QLAEVVANTITPLMKAQSIPGMAVAVIYQGKPHYYTFGKADIAASKPVTPQTLFELGSIS
ACT-24QSH26023.1_370EBOC	QLAEVVANTITPLMKAQSIPGMAVAVIYQGKPHYYTFGKADIAASKPVTPQTLFELGSIS
ACT-24QSH30639.1_370EBOC	QLAEVVANTITPLMKAQSIPGMAVAVIYQGKPHYYTFGKADIAASKPVTPQTLFELGSIS
ACT-24UIL48372.1_372EBOC	QLAEVVANTITPLMKAQSIPGMAVAVIYQGKPHYYTFGKADIAASKPVTPQTLFELGSIS
ACT-24UIM14687.1_372EBOC	QLAEVVANTITPLMKAQSIPGMAVAVIYQGKPHYYTFGKADIAASKPVTPQTLFELGSIS
ACT-24UXA74146.1_371EBOC	QLAEVVANTITPLMKAQSIPGMAVAVIYQGKPHYYTFGKADIAASKPVTPQTLFELGSIS
ACT-24WDY48721.1_372EBOC	QLAEVVANTITPLMKAQSIPGMAVAVIYQGKPHYYTFGKADIAASKPVTPQTLFELGSIS
ACT-89AVE73204.1_2453EBCP	QLAEVVANTITPLMKAQSVPGMAVAVIYQGKPHYYTFGKADIAANKPVTPQTLFELGSIS
ACT-89AVE73204.1_2453EBOC	QLAEVVANTITPLMKAQSVPGMAVAVIYQGKPHYYTFGKADIAANKPVTPQTLFELGSIS
ACT-56ASO99603.1_1270EBCP	QLAEVVANTVTPLMKAQSVPGMAVAVIYQGKPHYYTFGKADIAANKPVTPQTLFELGSIS
ACT-56QDQ75241.1_392EBCP	QLAEVVANTVTPLMKAQSVPGMAVAVIYQGKPHYYTFGKADIAANKPVTPQTLFELGSIS
ACT-56QFH98918.1_395EBCP	QLAEVVANTVTPLMKAQSVPGMAVAVIYQGKPHYYTFGKADIAANKPVTPQTLFELGSIS
ACT-56QFH34334.1_395EBCP	QLAEVVANTVTPLMKAQSVPGMAVAVIYQGKPHYYTFGKADIAANKPVTPQTLFELGSIS
ACT-56QFI32707.1_2233EBCP	QLAEVVANTVTPLMKAQSVPGMAVAVIYQGKPHYYTFGKADIAANKPVTPQTLFELGSIS
ACT-56QMI65633.1_4184EBCP	QLAEVVANTVTPLMKAQSVPGMAVAVIYQGKPHYYTFGKADIAANKPVTPQTLFELGSIS
ACT-56QMI71192.1_422EBCP	QLAEVVANTVTPLMKAQSVPGMAVAVIYQGKPHYYTFGKADIAANKPVTPQTLFELGSIS
ACT-56UDV17856.1_8EBCP	QLAEVVANTVTPLMKAQSVPGMAVAVIYQGKPHYYTFGKADIAANKPVTPQTLFELGSIS
ACT-56UDV39132.1_1376EBCP	QLAEVVANTVTPLMKAQSVPGMAVAVIYQGKPHYYTFGKADIAANKPVTPQTLFELGSIS
ACT-56UWB10427.1_430EBCP	QLAEVVANTVTPLMKAQSVPGMAVAVIYQGKPHYYTFGKADIAANKPVTPQTLFELGSIS
ACT-56UWC18315.1_415EBCP	QLAEVVANTVTPLMKAQSVPGMAVAVIYQGKPHYYTFGKADIAANKPVTPQTLFELGSIS
ACT-56UWC23093.1_417EBCP	QLAEVVANTVTPLMKAQSVPGMAVAVIYQGKPHYYTFGKADIAANKPVTPQTLFELGSIS
ACT-56WBT12607.1_393EBCP	QLAEVVANTVTPLMKAQSVPGMAVAVIYQGKPHYYTFGKADIAANKPVTPQTLFELGSIS
ACT-56WBT28831.1_393EBCP	QLAEVVANTVTPLMKAQSVPGMAVAVIYQGKPHYYTFGKADIAANKPVTPQTLFELGSIS
ACT-56WGB34617.1_392EBCP	QLAEVVANTVTPLMKAQSVPGMAVAVIYQGKPHYYTFGKADIAANKPVTPQTLFELGSIS
ACT-56WGU03466.1_393EBCP	QLAEVVANTVTPLMKAQSVPGMAVAVIYQGKPHYYTFGKADIAANKPVTPQTLFELGSIS
ACT-56ASO99603.1_1270EBOC	QLAEVVANTVTPLMKAQSVPGMAVAVIYQGKPHYYTFGKADIAANKPVTPQTLFELGSIS
ACT-56QDQ75241.1_392EBOC	QLAEVVANTVTPLMKAQSVPGMAVAVIYQGKPHYYTFGKADIAANKPVTPQTLFELGSIS
ACT-56QFH98918.1_395EBOC	QLAEVVANTVTPLMKAQSVPGMAVAVIYQGKPHYYTFGKADIAANKPVTPQTLFELGSIS
ACT-56QFH34334.1_395EBOC	QLAEVVANTVTPLMKAQSVPGMAVAVIYQGKPHYYTFGKADIAANKPVTPQTLFELGSIS
ACT-56QFI32707.1_2233EBOC	QLAEVVANTVTPLMKAQSVPGMAVAVIYQGKPHYYTFGKADIAANKPVTPQTLFELGSIS
ACT-56QMI65633.1_4184EBOC	QLAEVVANTVTPLMKAQSVPGMAVAVIYQGKPHYYTFGKADIAANKPVTPQTLFELGSIS
ACT-56QMI71192.1_422EBOC	QLAEVVANTVTPLMKAQSVPGMAVAVIYQGKPHYYTFGKADIAANKPVTPQTLFELGSIS
ACT-56UDV17856.1_8EBOC	QLAEVVANTVTPLMKAQSVPGMAVAVIYQGKPHYYTFGKADIAANKPVTPQTLFELGSIS
ACT-56UDV39132.1_1376EBOC	QLAEVVANTVTPLMKAQSVPGMAVAVIYQGKPHYYTFGKADIAANKPVTPQTLFELGSIS
ACT-56UWB10427.1_430EBOC	QLAEVVANTVTPLMKAQSVPGMAVAVIYQGKPHYYTFGKADIAANKPVTPQTLFELGSIS
ACT-56UWC18315.1_415EBOC	QLAEVVANTVTPLMKAQSVPGMAVAVIYQGKPHYYTFGKADIAANKPVTPQTLFELGSIS
ACT-56UWC23093.1_417EBOC	QLAEVVANTVTPLMKAQSVPGMAVAVIYQGKPHYYTFGKADIAANKPVTPQTLFELGSIS
ACT-56WBT12607.1_393EBOC	QLAEVVANTVTPLMKAQSVPGMAVAVIYQGKPHYYTFGKADIAANKPVTPQTLFELGSIS
ACT-56WBT28831.1_393EBOC	QLAEVVANTVTPLMKAQSVPGMAVAVIYQGKPHYYTFGKADIAANKPVTPQTLFELGSIS
ACT-56WGB34617.1_392EBOC	QLAEVVANTVTPLMKAQSVPGMAVAVIYQGKPHYYTFGKADIAANKPVTPQTLFELGSIS
ACT-56WGU03466.1_393EBOC	QLAEVVANTVTPLMKAQSVPGMAVAVIYQGKPHYYTFGKADIAANKPVTPQTLFELGSIS
ACT-56AOP76464.1_392EOC	QLAEVVANTVTPLMKAQSVPGMAVAVIYQGKPHYYTFGKADIAANKPVTPQTLFELGSIS
ACT-56QXA77046.1_2672EOC	QLAEVVANTVTPLMKAQSVPGMAVAVIYQGKPHYYTFGKADIAANKPVTPQTLFELGSIS
ACT-56WFP29817.1_397EOC	QLAEVVANTVTPLMKAQSVPGMAVAVIYQGKPHYYTFGKADIAANKPVTPQTLFELGSIS
ACT-27AXO49795.1_1610EBCP	QLAEVVANTVTPLMKAQSVPGMAVAVIYQGKPHYYTFGKADIAANKPVTPQTLFELGSIS
ACT-27QHO79915.1_457EBCP	QLAEVVANTVTPLMKAQSVPGMAVAVIYQGKPHYYTFGKADIAANKPVTPQTLFELGSIS
ACT-27QHO97012.1_458EBCP	QLAEVVANTVTPLMKAQSVPGMAVAVIYQGKPHYYTFGKADIAANKPVTPQTLFELGSIS
ACT-27QQH03712.1_3802EBCP	QLAEVVANTVTPLMKAQSVPGMAVAVIYQGKPHYYTFGKADIAANKPVTPQTLFELGSIS
ACT-27QQH08784.1_3802EBCP	QLAEVVANTVTPLMKAQSVPGMAVAVIYQGKPHYYTFGKADIAANKPVTPQTLFELGSIS
ACT-27UVZ91365.1_388EBCP	QLAEVVANTVTPLMKAQSVPGMAVAVIYQGKPHYYTFGKADIAANKPVTPQTLFELGSIS
ACT-27AXO49795.1_1610EBOC	QLAEVVANTVTPLMKAQSVPGMAVAVIYQGKPHYYTFGKADIAANKPVTPQTLFELGSIS
ACT-27QHO79915.1_457EBOC	QLAEVVANTVTPLMKAQSVPGMAVAVIYQGKPHYYTFGKADIAANKPVTPQTLFELGSIS
ACT-27QHO97012.1_458EBOC	QLAEVVANTVTPLMKAQSVPGMAVAVIYQGKPHYYTFGKADIAANKPVTPQTLFELGSIS
ACT-27QQH03712.1_3802EBOC	QLAEVVANTVTPLMKAQSVPGMAVAVIYQGKPHYYTFGKADIAANKPVTPQTLFELGSIS
ACT-27QQH08784.1_3802EBOC	QLAEVVANTVTPLMKAQSVPGMAVAVIYQGKPHYYTFGKADIAANKPVTPQTLFELGSIS
ACT-27UVZ91365.1_388EBOC	QLAEVVANTVTPLMKAQSVPGMAVAVIYQGKPHYYTFGKADIAANKPVTPQTLFELGSIS
ACT-102QEO03155.1_4213EBCP	QLSDVVERTVTPLMKAQAIPGMAVAVIYQGHPHYFTFGKADVAANKPVTPQTLFELGSIS
ACT-102BBV79841.1_51928]EBCP	QLSDVVERTVTPLMKAQAIPGMAVAVIYQGHPHYFTFGKADVAANKPVTPQTLFELGSIS
ACT-102QZS47739.1_401EBCP	QLSDVVERTVTPLMKAQAIPGMAVAVIYQGHPHYFTFGKADVAANKPVTPQTLFELGSIS
ACT-102QEO03155.1_4213EBOC	QLSDVVERTVTPLMKAQAIPGMAVAVIYQGHPHYFTFGKADVAANKPVTPQTLFELGSIS
ACT-102BBV79841.1_51928]EBOC	QLSDVVERTVTPLMKAQAIPGMAVAVIYQGHPHYFTFGKADVAANKPVTPQTLFELGSIS
ACT-102QZS47739.1_401EBOC	QLSDVVERTVTPLMKAQAIPGMAVAVIYQGHPHYFTFGKADVAANKPVTPQTLFELGSIS
ACT-55QLP71015.1_413EBCP	QLAEVVANTVTPLMKAQSVPGMAVAVIYQGKPHYYTFGKADIAANKPVTPQTLFELGSIS
ACT-55QLP80034.1_414EBCP	QLAEVVANTVTPLMKAQSVPGMAVAVIYQGKPHYYTFGKADIAANKPVTPQTLFELGSIS
ACT-55QLS08818.1_415EBCP	QLAEVVANTVTPLMKAQSVPGMAVAVIYQGKPHYYTFGKADIAANKPVTPQTLFELGSIS
ACT-55QLT25377.1_3621EBCP	QLAEVVANTVTPLMKAQSVPGMAVAVIYQGKPHYYTFGKADIAANKPVTPQTLFELGSIS
ACT-55QLV12510.1_4006EBCP	QLAEVVANTVTPLMKAQSVPGMAVAVIYQGKPHYYTFGKADIAANKPVTPQTLFELGSIS
ACT-55QLW04180.1_4182EBCP	QLAEVVANTVTPLMKAQSVPGMAVAVIYQGKPHYYTFGKADIAANKPVTPQTLFELGSIS
ACT-55QLV08026.1_4006EBCP	QLAEVVANTVTPLMKAQSVPGMAVAVIYQGKPHYYTFGKADIAANKPVTPQTLFELGSIS
ACT-55QLV96171.1_421EBCP	QLAEVVANTVTPLMKAQSVPGMAVAVIYQGKPHYYTFGKADIAANKPVTPQTLFELGSIS
ACT-55QLW44293.1_405EBCP	QLAEVVANTVTPLMKAQSVPGMAVAVIYQGKPHYYTFGKADIAANKPVTPQTLFELGSIS
ACT-55UBH32480.1_675EBCP	QLAEVVANTVTPLMKAQSVPGMAVAVIYQGKPHYYTFGKADIAANKPVTPQTLFELGSIS
ACT-55UDV61223.1_2251EBCP	QLAEVVANTVTPLMKAQSVPGMAVAVIYQGKPHYYTFGKADIAANKPVTPQTLFELGSIS
ACT-55QLP71015.1_413EBOC	QLAEVVANTVTPLMKAQSVPGMAVAVIYQGKPHYYTFGKADIAANKPVTPQTLFELGSIS
ACT-55QLP80034.1_414EBOC	QLAEVVANTVTPLMKAQSVPGMAVAVIYQGKPHYYTFGKADIAANKPVTPQTLFELGSIS
ACT-55QLS08818.1_415EBOC	QLAEVVANTVTPLMKAQSVPGMAVAVIYQGKPHYYTFGKADIAANKPVTPQTLFELGSIS
ACT-55QLT25377.1_3621EBOC	QLAEVVANTVTPLMKAQSVPGMAVAVIYQGKPHYYTFGKADIAANKPVTPQTLFELGSIS
ACT-55QLV12510.1_4006EBOC	QLAEVVANTVTPLMKAQSVPGMAVAVIYQGKPHYYTFGKADIAANKPVTPQTLFELGSIS

```
ACT-55QLW04180.1_4182EBOC      QLAEVVANTVTPLMKAQSVPGMAVAVIYQGKPHYYTFGKADIAANKPVTPQTLFELGSIS
ACT-55QLV08026.1_4006EBOC      QLAEVVANTVTPLMKAQSVPGMAVAVIYQGKPHYYTFGKADIAANKPVTPQTLFELGSIS
ACT-55QLV96171.1_421EBOC       QLAEVVANTVTPLMKAQSVPGMAVAVIYQGKPHYYTFGKADIAANKPVTPQTLFELGSIS
ACT-55QLW44293.1_405EBOC       QLAEVVANTVTPLMKAQSVPGMAVAVIYQGKPHYYTFGKADIAANKPVTPQTLFELGSIS
ACT-55UBH32480.1_675EBOC       QLAEVVANTVTPLMKAQSVPGMAVAVIYQGKPHYYTFGKADIAANKPVTPQTLFELGSIS
ACT-55UDV61223.1_2251EBOC      QLAEVVANTVTPLMKAQSVPGMAVAVIYQGKPHYYTFGKADIAANKPVTPQTLFELGSIS
ACT-55AOP80857.1_409EOC        QLAEVVANTVTPLMKAQSVPGMAVAVIYQGKPHYYTFGKADIAANKPVTPQTLFELGSIS
ACT-51BBW20048.1_38351]EBCP    QLSDVVERTVTPLMKAQAIPGMAVAVIYQGQPHYFTFGKADVAANKPVTPQTLFELGSIS
ACT-51BBW20048.1_38351]EBOC    QLSDVVERTVTPLMKAQAIPGMAVAVIYQGQPHYFTFGKADVAANKPVTPQTLFELGSIS
ACT-28URE95606.1_351EBCP       QLSDVVERTVTPLMKAQAIPGMAVAVIYQGQPHYFTFGKADVAANKPVTPQTLFELGSIS
ACT-28URE95606.1_351EBOC       QLSDVVERTVTPLMKAQAIPGMAVAVIYQGQPHYFTFGKADVAANKPVTPQTLFELGSIS
ACT-87AYL04658.1_1249EBCP      QLSDVVERTVTPLMKAQAIPGMAVAVIYQGQPHYFTFGKADVAANKPVTPQTLFELGSIS
ACT-87QIP18425.1_352EBCP       QLSDVVERTVTPLMKAQAIPGMAVAVIYQGQPHYFTFGKADVAANKPVTPQTLFELGSIS
ACT-87AYL04658.1_1249EBOC      QLSDVVERTVTPLMKAQAIPGMAVAVIYQGQPHYFTFGKADVAANKPVTPQTLFELGSIS
ACT-87QIP18425.1_352EBOC       QLSDVVERTVTPLMKAQAIPGMAVAVIYQGQPHYFTFGKADVAANKPVTPQTLFELGSIS
ACT-75AWX01586.1_1447EBCP      QLAEVVANTVTPLMIAQSVPGMAVAVIYQGKSHYYTFGKADIAANKPVTPQTLFELGSIS
ACT-75AWX01586.1_1447EBOC      QLAEVVANTVTPLMIAQSVPGMAVAVIYQGKSHYYTFGKADIAANKPVTPQTLFELGSIS
ACT-103URL27837.1_342EOC       QLSDVVERTVTPLMKAQAIPGMAVAVIYQGQPHYFTFGKADVAANKPVTPQTLFELGSIS
ACT-99UXJ64669.1_346EBCP       QLSDVVERTVTPLMKAQAIPGMAVAVIYQGQPHYFTFGKADVAANKPVTPQTLFELGSIS
ACT-99UXJ64669.1_346EBOC       QLSDVVERTVTPLMKAQAIPGMAVAVIYQGQPHYFTFGKADVAANKPVTPQTLFELGSIS
ACT-95QIB80518.1_348EBCP       QLSDVVERTVTPLMKAQAIPGMAVAVIYQGQPHYFTFGKADVAANKPVTPQTLFELGSIS
ACT-95BBV69349.1_35128]EBCP    QLSDVVERTVTPLMKAQAIPGMAVAVIYQGQPHYFTFGKADVAANKPVTPQTLFELGSIS
ACT-95UZQ68133.1_388EBCP       QLSDVVERTVTPLMKAQAIPGMAVAVIYQGQPHYFTFGKADVAANKPVTPQTLFELGSIS
ACT-95QIB80518.1_348EBOC       QLSDVVERTVTPLMKAQAIPGMAVAVIYQGQPHYFTFGKADVAANKPVTPQTLFELGSIS
ACT-95BBV69349.1_35128]EBOC    QLSDVVERTVTPLMKAQAIPGMAVAVIYQGQPHYFTFGKADVAANKPVTPQTLFELGSIS
ACT-95UZQ68133.1_388EBOC       QLSDVVERTVTPLMKAQAIPGMAVAVIYQGQPHYFTFGKADVAANKPVTPQTLFELGSIS
ACT-52QFH88560.1_350EBCP       QLSDVVERTVTPLMKAQAIPGMAVAVIYQGQPHYFTFGKADVAANKPVTPQTLFELGSIS
ACT-52BBS30224.1_43052]EBCP    QLSDVVERTVTPLMKAQAIPGMAVAVIYQGQPHYFTFGKADVAANKPVTPQTLFELGSIS
ACT-52WBN86316.1_4083EBCP      QLSDVVERTVTPLMKAQAIPGMAVAVIYQGQPHYFTFGKADVAANKPVTPQTLFELGSIS
ACT-52QFH88560.1_350EBOC       QLSDVVERTVTPLMKAQAIPGMAVAVIYQGQPHYFTFGKADVAANKPVTPQTLFELGSIS
ACT-52BBS30224.1_43052]EBOC    QLSDVVERTVTPLMKAQAIPGMAVAVIYQGQPHYFTFGKADVAANKPVTPQTLFELGSIS
ACT-52WBN86316.1_4083EBOC      QLSDVVERTVTPLMKAQAIPGMAVAVIYQGQPHYFTFGKADVAANKPVTPQTLFELGSIS
ACT-4QLR27288.1_395EBCP        QLAEVVERTVTPLMKAQAIPGMAVAVIYQGQPHYFTFGKADVAANKPVTPQTLFELGSIS
ACT-4QPS68257.1_413EBCP        QLAEVVERTVTPLMKAQAIPGMAVAVIYQGQPHYFTFGKADVAANKPVTPQTLFELGSIS
ACT-4QLR27288.1_395EBOC        QLAEVVERTVTPLMKAQAIPGMAVAVIYQGQPHYFTFGKADVAANKPVTPQTLFELGSIS
ACT-4QPS68257.1_413EBOC        QLAEVVERTVTPLMKAQAIPGMAVAVIYQGQPHYFTFGKADVAANKPVTPQTLFELGSIS
ACT-3UOY53510.1_358EBCP        QLAEVVERTVTPLMKAQAIPGMAVAVIYEGQPHYFTFGKADVAANKPVTPQTLFELGSIS
ACT-3UOY63785.1_4171EBCP       QLAEVVERTVTPLMKAQAIPGMAVAVIYEGQPHYFTFGKADVAANKPVTPQTLFELGSIS
ACT-3UOY53510.1_358EBOC        QLAEVVERTVTPLMKAQAIPGMAVAVIYEGQPHYFTFGKADVAANKPVTPQTLFELGSIS
ACT-3UOY63785.1_4171EBOC       QLAEVVERTVTPLMKAQAIPGMAVAVIYEGQPHYFTFGKADVAANKPVTPQTLFELGSIS
ACT-1QLV61731.1_472EBCP        QLAEVVERTVTPLMKAQAIPGMAVAVIYEGQPHYFTFGKADVAANKPVTPQTLFELGSIS
ACT-1UWA77567.1_382EBCP        QLAEVVERTVTPLMKAQAIPGMAVAVIYEGQPHYFTFGKADVAANKPVTPQTLFELGSIS
ACT-1QLV61731.1_472EBOC        QLAEVVERTVTPLMKAQAIPGMAVAVIYEGQPHYFTFGKADVAANKPVTPQTLFELGSIS
ACT-1UWA77567.1_382EBOC        QLAEVVERTVTPLMKAQAIPGMAVAVIYEGQPHYFTFGKADVAANKPVTPQTLFELGSIS
ACT-77QWZ50133.1_330EBCP       QLADVVERNVTPLMKAQGIPGMAVAVIYQGQPHYFTFGKADIAANKPVTPQTLFELGSIS
ACT-77UBH39796.1_3999EBCP      QLADVVERNVTPLMKAQGIPGMAVAVIYQGQPHYFTFGKADIAANKPVTPQTLFELGSIS
ACT-77UBH98103.1_3842EBCP      QLADVVERNVTPLMKAQGIPGMAVAVIYQGQPHYFTFGKADIAANKPVTPQTLFELGSIS
ACT-77UBH94108.1_193EBCP       QLADVVERNVTPLMKAQGIPGMAVAVIYQGQPHYFTFGKADIAANKPVTPQTLFELGSIS
ACT-77QWZ50133.1_330EBOC       QLADVVERNVTPLMKAQGIPGMAVAVIYQGQPHYFTFGKADIAANKPVTPQTLFELGSIS
ACT-77UBH39796.1_3999EBOC      QLADVVERNVTPLMKAQGIPGMAVAVIYQGQPHYFTFGKADIAANKPVTPQTLFELGSIS
ACT-77UBH98103.1_3842EBOC      QLADVVERNVTPLMKAQGIPGMAVAVIYQGQPHYFTFGKADIAANKPVTPQTLFELGSIS
ACT-77UBH94108.1_193EBOC       QLADVVERNVTPLMKAQGIPGMAVAVIYQGQPHYFTFGKADIAANKPVTPQTLFELGSIS
ACT-53QEL38380.1_485EBCP       QLAEVVEQTVTPLMKAQAIPGMAVAVIYEGQPHYFTFGKADVAKNKPVTPQTLFELGSIS
ACT-53QEL38380.1_485EBOC       QLAEVVEQTVTPLMKAQAIPGMAVAVIYEGQPHYFTFGKADVAKNKPVTPQTLFELGSIS
ACT-57QBB07838.1_4703EBCP      QLAEMVERTVTPLMKAQAIPGMAVAVIYQGQPHYFTFGKADVAANKPVTPQTLFELGSIS
ACT-57QYD27335.1_499EBCP       QLAEMVERTVTPLMKAQAIPGMAVAVIYQGQPHYFTFGKADVAANKPVTPQTLFELGSIS
ACT-57UBM17197.1_2672EBCP      QLAEMVERTVTPLMKAQAIPGMAVAVIYQGQPHYFTFGKADVAANKPVTPQTLFELGSIS
ACT-57UKB55021.1_380EBCP       QLAEMVERTVTPLMKAQAIPGMAVAVIYQGQPHYFTFGKADVAANKPVTPQTLFELGSIS
ACT-57QBB07838.1_4703EBOC      QLAEMVERTVTPLMKAQAIPGMAVAVIYQGQPHYFTFGKADVAANKPVTPQTLFELGSIS
ACT-57QYD27335.1_499EBOC       QLAEMVERTVTPLMKAQAIPGMAVAVIYQGQPHYFTFGKADVAANKPVTPQTLFELGSIS
ACT-57UBM17197.1_2672EBOC      QLAEMVERTVTPLMKAQAIPGMAVAVIYQGQPHYFTFGKADVAANKPVTPQTLFELGSIS
ACT-57UKB55021.1_380EBOC       QLAEMVERTVTPLMKAQAIPGMAVAVIYQGQPHYFTFGKADVAANKPVTPQTLFELGSIS
ACT-93ALL15984.1_396EOC        QLAEVVERTVTPLMKAQVIPGMAVAVIYQGQPHYFTFGKADVAANKPVTPQTLFELGSIS
ACT-9AOP85131.1_312EBCP        QLSDVVERTVTPLMKAQAIPGMAVAVIYQGQPHYFTFGKADVTANKPVTPQTLFELGSIS
ACT-9UOY32628.1_343EBCP        QLSDVVERTVTPLMKAQAIPGMAVAVIYQGQPHYFTFGKADVTANKPVTPQTLFELGSIS
ACT-9AOP85131.1_312EBOC        QLSDVVERTVTPLMKAQAIPGMAVAVIYQGQPHYFTFGKADVTANKPVTPQTLFELGSIS
ACT-9UOY32628.1_343EBOC        QLSDVVERTVTPLMKAQAIPGMAVAVIYQGQPHYFTFGKADVTANKPVTPQTLFELGSIS
ACT-9AMZ75637.1_173EOC         QLSDVVERTVTPLMKAQAIPGMAVAVIYQGQPHYFTFGKADVTANKPVTPQTLFELGSIS
ACT-6BBT43181.1_3396]EBCP      QLAEVVERTVTPLMKAQAIPGMAVAVIYQGQPHYFTFGKADVAANKPVTPQTLFELGSIS
ACT-6BBT43181.1_3396]EBOC      QLAEVVERTVTPLMKAQAIPGMAVAVIYQGQPHYFTFGKADVAANKPVTPQTLFELGSIS
ACT-46QUP91302.1_387EBCP       QLAEVVANTVTPLMIAQSVPGMAVAVIYQGKSHYYTFGKADIAANKPVTPQTLFELGSIS
ACT-46QUP91302.1_387EBOC       QLAEVVANTVTPLMIAQSVPGMAVAVIYQGKSHYYTFGKADIAANKPVTPQTLFELGSIS
ACT-67QLP16520.1_400EBCP       QLAEVVANTVTPLMKAQSIPGMAVAVIYQGKPHYYTFGKADIAASKPVTPQTLFELGSIS
ACT-67QLP16520.1_400EBOC       QLAEVVANTVTPLMKAQSIPGMAVAVIYQGKPHYYTFGKADIAASKPVTPQTLFELGSIS
ACT-84QOR49295.1_413EBCP       QLAEVVANTVTPLMKAQSVPGMAVAVIYQGKPHYYTFGKADIAANKPVTPQTLFELGSIS
ACT-84QOR49295.1_413EBOC       QLAEVVANTVTPLMKAQSVPGMAVAVIYQGKPHYYTFGKADIAANKPVTPQTLFELGSIS
ACT-74QOX68946.1_496EBCP       QLAEVVANTVTPLMKAQSVPGMAVAVIYQGKPHYYTFGKADIAANKPVTPQTLFELGSIS
ACT-74UIZ04332.1_460EBCP       QLAEVVANTVTPLMKAQSVPGMAVAVIYQGKPHYYTFGKADIAANKPVTPQTLFELGSIS
ACT-74QOX68946.1_496EBOC       QLAEVVANTVTPLMKAQSVPGMAVAVIYQGKPHYYTFGKADIAANKPVTPQTLFELGSIS
ACT-74UIZ04332.1_460EBOC       QLAEVVANTVTPLMKAQSVPGMAVAVIYQGKPHYYTFGKADIAANKPVTPQTLFELGSIS
ACT-74QQT40108.1_4574EOC       QLAEVVANTVTPLMKAQSVPGMAVAVIYQGKPHYYTFGKADIAANKPVTPQTLFELGSIS
ACT-16ATW90451.1_311EBCP       QLAEVVANTVTPLMKAQSVPGMAVAVIYQGKSHYYTFGKADIAANKPVTPQTLFELGSIS
ACT-16AVO82105.1_1257EBCP      QLAEVVANTVTPLMKAQSVPGMAVAVIYQGKSHYYTFGKADIAANKPVTPQTLFELGSIS
ACT-16AWQ41825.1_393EBCP       QLAEVVANTVTPLMKAQSVPGMAVAVIYQGKSHYYTFGKADIAANKPVTPQTLFELGSIS
ACT-16AWQ56038.1_393EBCP       QLAEVVANTVTPLMKAQSVPGMAVAVIYQGKSHYYTFGKADIAANKPVTPQTLFELGSIS
ACT-16QFH53420.1_474EBCP       QLAEVVANTVTPLMKAQSVPGMAVAVIYQGKSHYYTFGKADIAANKPVTPQTLFELGSIS
ACT-16QPO90952.1_396EBCP       QLAEVVANTVTPLMKAQSVPGMAVAVIYQGKSHYYTFGKADIAANKPVTPQTLFELGSIS
ACT-16QQA67679.1_472EBCP       QLAEVVANTVTPLMKAQSVPGMAVAVIYQGKSHYYTFGKADIAANKPVTPQTLFELGSIS
ACT-16UDV14809.1_2173EBCP      QLAEVVANTVTPLMKAQSVPGMAVAVIYQGKSHYYTFGKADIAANKPVTPQTLFELGSIS
ACT-16UDV41468.1_3105EBCP      QLAEVVANTVTPLMKAQSVPGMAVAVIYQGKSHYYTFGKADIAANKPVTPQTLFELGSIS
ACT-16UIZ08994.1_395EBCP       QLAEVVANTVTPLMKAQSVPGMAVAVIYQGKSHYYTFGKADIAANKPVTPQTLFELGSIS
ACT-16WJJ28417.1_386EBCP       QLAEVVANTVTPLMKAQSVPGMAVAVIYQGKSHYYTFGKADIAANKPVTPQTLFELGSIS
ACT-16ATW90451.1_311EBOC       QLAEVVANTVTPLMKAQSVPGMAVAVIYQGKSHYYTFGKADIAANKPVTPQTLFELGSIS
ACT-16AVO82105.1_1257EBOC      QLAEVVANTVTPLMKAQSVPGMAVAVIYQGKSHYYTFGKADIAANKPVTPQTLFELGSIS
ACT-16AWQ41825.1_393EBOC       QLAEVVANTVTPLMKAQSVPGMAVAVIYQGKSHYYTFGKADIAANKPVTPQTLFELGSIS
ACT-16AWQ56038.1_393EBOC       QLAEVVANTVTPLMKAQSVPGMAVAVIYQGKSHYYTFGKADIAANKPVTPQTLFELGSIS
ACT-16QFH53420.1_474EBOC       QLAEVVANTVTPLMKAQSVPGMAVAVIYQGKSHYYTFGKADIAANKPVTPQTLFELGSIS
ACT-16QPO90952.1_396EBOC       QLAEVVANTVTPLMKAQSVPGMAVAVIYQGKSHYYTFGKADIAANKPVTPQTLFELGSIS
ACT-16QQA67679.1_472EBOC       QLAEVVANTVTPLMKAQSVPGMAVAVIYQGKSHYYTFGKADIAANKPVTPQTLFELGSIS
ACT-16UDV14809.1_2173EBOC      QLAEVVANTVTPLMKAQSVPGMAVAVIYQGKSHYYTFGKADIAANKPVTPQTLFELGSIS
ACT-16UDV41468.1_3105EBOC      QLAEVVANTVTPLMKAQSVPGMAVAVIYQGKSHYYTFGKADIAANKPVTPQTLFELGSIS
ACT-16UIZ08994.1_395EBOC       QLAEVVANTVTPLMKAQSVPGMAVAVIYQGKSHYYTFGKADIAANKPVTPQTLFELGSIS
ACT-16WJJ28417.1_386EBOC       QLAEVVANTVTPLMKAQSVPGMAVAVIYQGKSHYYTFGKADIAANKPVTPQTLFELGSIS
```

```
ACT-23QLP51667.1_388EBCP        QLAEVVANTVTPLMKAQSIPGMAVAVIYQGKPHYYTFGKADIAASKPVTPQTLFELGSIS
ACT-23QLP61663.1_388EBCP        QLAEVVANTVTPLMKAQSIPGMAVAVIYQGKPHYYTFGKADIAASKPVTPQTLFELGSIS
ACT-23QLP66427.1_430EBCP        QLAEVVANTVTPLMKAQSIPGMAVAVIYQGKPHYYTFGKADIAASKPVTPQTLFELGSIS
ACT-23QLU80799.1_388EBCP        QLAEVVANTVTPLMKAQSIPGMAVAVIYQGKPHYYTFGKADIAASKPVTPQTLFELGSIS
ACT-23QLT97825.1_367EBCP        QLAEVVANTVTPLMKAQSIPGMAVAVIYQGKPHYYTFGKADIAASKPVTPQTLFELGSIS
ACT-23QLU89187.1_4307EBCP       QLAEVVANTVTPLMKAQSIPGMAVAVIYQGKPHYYTFGKADIAASKPVTPQTLFELGSIS
ACT-23QLV76440.1_389EBCP        QLAEVVANTVTPLMKAQSIPGMAVAVIYQGKPHYYTFGKADIAASKPVTPQTLFELGSIS
ACT-23QLT57991.1_396EBCP        QLAEVVANTVTPLMKAQSIPGMAVAVIYQGKPHYYTFGKADIAASKPVTPQTLFELGSIS
ACT-23QLV44058.1_369EBCP        QLAEVVANTVTPLMKAQSIPGMAVAVIYQGKPHYYTFGKADIAASKPVTPQTLFELGSIS
ACT-23QLP51667.1_388EBOC        QLAEVVANTVTPLMKAQSIPGMAVAVIYQGKPHYYTFGKADIAASKPVTPQTLFELGSIS
ACT-23QLP61663.1_388EBOC        QLAEVVANTVTPLMKAQSIPGMAVAVIYQGKPHYYTFGKADIAASKPVTPQTLFELGSIS
ACT-23QLP66427.1_430EBOC        QLAEVVANTVTPLMKAQSIPGMAVAVIYQGKPHYYTFGKADIAASKPVTPQTLFELGSIS
ACT-23QLU80799.1_388EBOC        QLAEVVANTVTPLMKAQSIPGMAVAVIYQGKPHYYTFGKADIAASKPVTPQTLFELGSIS
ACT-23QLT97825.1_367EBOC        QLAEVVANTVTPLMKAQSIPGMAVAVIYQGKPHYYTFGKADIAASKPVTPQTLFELGSIS
ACT-23QLU89187.1_4307EBOC       QLAEVVANTVTPLMKAQSIPGMAVAVIYQGKPHYYTFGKADIAASKPVTPQTLFELGSIS
ACT-23QLV76440.1_389EBOC        QLAEVVANTVTPLMKAQSIPGMAVAVIYQGKPHYYTFGKADIAASKPVTPQTLFELGSIS
ACT-23QLT57991.1_396EBOC        QLAEVVANTVTPLMKAQSIPGMAVAVIYQGKPHYYTFGKADIAASKPVTPQTLFELGSIS
ACT-23QLV44058.1_369EBOC        QLAEVVANTVTPLMKAQSIPGMAVAVIYQGKPHYYTFGKADIAASKPVTPQTLFELGSIS
ACT-115AXQ35263.1_2972EBCP      QLAEVVANTVTPLMKAQSVPGMAVAVIYQGKPHYYTFGKADIAANKPVTPQTLFELGSIS
ACT-115QGR11187.1_384EBCP       QLAEVVANTVTPLMKAQSVPGMAVAVIYQGKPHYYTFGKADIAANKPVTPQTLFELGSIS
ACT-115QIF26741.1_2106EBCP      QLAEVVANTVTPLMKAQSVPGMAVAVIYQGKPHYYTFGKADIAANKPVTPQTLFELGSIS
ACT-115QIF32246.1_3030EBCP      QLAEVVANTVTPLMKAQSVPGMAVAVIYQGKPHYYTFGKADIAANKPVTPQTLFELGSIS
ACT-115QOD35429.1_385EBCP       QLAEVVANTVTPLMKAQSVPGMAVAVIYQGKPHYYTFGKADIAANKPVTPQTLFELGSIS
ACT-115QPD66621.1_385EBCP       QLAEVVANTVTPLMKAQSVPGMAVAVIYQGKPHYYTFGKADIAANKPVTPQTLFELGSIS
ACT-115QSS35407.1_385EBCP       QLAEVVANTVTPLMKAQSVPGMAVAVIYQGKPHYYTFGKADIAANKPVTPQTLFELGSIS
ACT-115URE88756.1_385EBCP       QLAEVVANTVTPLMKAQSVPGMAVAVIYQGKPHYYTFGKADIAANKPVTPQTLFELGSIS
ACT-115USM81982.1_384EBCP       QLAEVVANTVTPLMKAQSVPGMAVAVIYQGKPHYYTFGKADIAANKPVTPQTLFELGSIS
ACT-115AXQ35263.1_2972EBOC      QLAEVVANTVTPLMKAQSVPGMAVAVIYQGKPHYYTFGKADIAANKPVTPQTLFELGSIS
ACT-115QGR11187.1_384EBOC       QLAEVVANTVTPLMKAQSVPGMAVAVIYQGKPHYYTFGKADIAANKPVTPQTLFELGSIS
ACT-115QIF26741.1_2106EBOC      QLAEVVANTVTPLMKAQSVPGMAVAVIYQGKPHYYTFGKADIAANKPVTPQTLFELGSIS
ACT-115QIF32246.1_3030EBOC      QLAEVVANTVTPLMKAQSVPGMAVAVIYQGKPHYYTFGKADIAANKPVTPQTLFELGSIS
ACT-115QOD35429.1_385EBOC       QLAEVVANTVTPLMKAQSVPGMAVAVIYQGKPHYYTFGKADIAANKPVTPQTLFELGSIS
ACT-115QPD66621.1_385EBOC       QLAEVVANTVTPLMKAQSVPGMAVAVIYQGKPHYYTFGKADIAANKPVTPQTLFELGSIS
ACT-115QSS35407.1_385EBOC       QLAEVVANTVTPLMKAQSVPGMAVAVIYQGKPHYYTFGKADIAANKPVTPQTLFELGSIS
ACT-115URE88756.1_385EBOC       QLAEVVANTVTPLMKAQSVPGMAVAVIYQGKPHYYTFGKADIAANKPVTPQTLFELGSIS
ACT-115USM81982.1_384EBOC       QLAEVVANTVTPLMKAQSVPGMAVAVIYQGKPHYYTFGKADIAANKPVTPQTLFELGSIS
ACT-25AVU48988.1_427EBCP        QLAEVVANTVTPLMKAQSVPGMAVAVIYQGKSHYYTFGKADIAANKPVTPQTLFELGSIS
ACT-25UQQ46893.1_379EBCP        QLAEVVANTVTPLMKAQSVPGMAVAVIYQGKSHYYTFGKADIAANKPVTPQTLFELGSIS
ACT-25WDO17280.1_310EBCP        QLAEVVANTVTPLMKAQSVPGMAVAVIYQGKSHYYTFGKADIAANKPVTPQTLFELGSIS
ACT-25AVU48988.1_427EBOC        QLAEVVANTVTPLMKAQSVPGMAVAVIYQGKSHYYTFGKADIAANKPVTPQTLFELGSIS
ACT-25UQQ46893.1_379EBOC        QLAEVVANTVTPLMKAQSVPGMAVAVIYQGKSHYYTFGKADIAANKPVTPQTLFELGSIS
ACT-25WDO17280.1_310EBOC        QLAEVVANTVTPLMKAQSVPGMAVAVIYQGKSHYYTFGKADIAANKPVTPQTLFELGSIS
ACT-25AOP89724.1_377EOC         QLAEVVANTVTPLMKAQSVPGMAVAVIYQGKSHYYTFGKADIAANKPVTPQTLFELGSIS
ACT-45APR43249.1_2902EBCP       QLAEVVANTVTPLMKAQSVPGMAVAVIYQGKPHYYTFGKADIAANKPVTPQTLFELGSIS
ACT-45ASA05885.1_3853EBCP       QLAEVVANTVTPLMKAQSVPGMAVAVIYQGKPHYYTFGKADIAANKPVTPQTLFELGSIS
ACT-45ASB85359.1_3592EBCP       QLAEVVANTVTPLMKAQSVPGMAVAVIYQGKPHYYTFGKADIAANKPVTPQTLFELGSIS
ACT-45AWR70714.1_4154EBCP       QLAEVVANTVTPLMKAQSVPGMAVAVIYQGKPHYYTFGKADIAANKPVTPQTLFELGSIS
ACT-45AXM01511.1_4138EBCP       QLAEVVANTVTPLMKAQSVPGMAVAVIYQGKPHYYTFGKADIAANKPVTPQTLFELGSIS
ACT-45AYU96034.1_2649EBCP       QLAEVVANTVTPLMKAQSVPGMAVAVIYQGKPHYYTFGKADIAANKPVTPQTLFELGSIS
ACT-45QBH65043.1_3960EBCP       QLAEVVANTVTPLMKAQSVPGMAVAVIYQGKPHYYTFGKADIAANKPVTPQTLFELGSIS
ACT-45QEL31100.1_1855EBCP       QLAEVVANTVTPLMKAQSVPGMAVAVIYQGKPHYYTFGKADIAANKPVTPQTLFELGSIS
ACT-45ULQ22907.1_427EBCP        QLAEVVANTVTPLMKAQSVPGMAVAVIYQGKPHYYTFGKADIAANKPVTPQTLFELGSIS
ACT-45ULQ25748.1_3478EBCP       QLAEVVANTVTPLMKAQSVPGMAVAVIYQGKPHYYTFGKADIAANKPVTPQTLFELGSIS
ACT-45WCO72956.1_42EBCP         QLAEVVANTVTPLMKAQSVPGMAVAVIYQGKPHYYTFGKADIAANKPVTPQTLFELGSIS
ACT-45APR43249.1_2902EBOC       QLAEVVANTVTPLMKAQSVPGMAVAVIYQGKPHYYTFGKADIAANKPVTPQTLFELGSIS
ACT-45ASA05885.1_3853EBOC       QLAEVVANTVTPLMKAQSVPGMAVAVIYQGKPHYYTFGKADIAANKPVTPQTLFELGSIS
ACT-45ASB85359.1_3592EBOC       QLAEVVANTVTPLMKAQSVPGMAVAVIYQGKPHYYTFGKADIAANKPVTPQTLFELGSIS
ACT-45AWR70714.1_4154EBOC       QLAEVVANTVTPLMKAQSVPGMAVAVIYQGKPHYYTFGKADIAANKPVTPQTLFELGSIS
ACT-45AXM01511.1_4138EBOC       QLAEVVANTVTPLMKAQSVPGMAVAVIYQGKPHYYTFGKADIAANKPVTPQTLFELGSIS
ACT-45AYU96034.1_2649EBOC       QLAEVVANTVTPLMKAQSVPGMAVAVIYQGKPHYYTFGKADIAANKPVTPQTLFELGSIS
ACT-45QBH65043.1_3960EBOC       QLAEVVANTVTPLMKAQSVPGMAVAVIYQGKPHYYTFGKADIAANKPVTPQTLFELGSIS
ACT-45QEL31100.1_1855EBOC       QLAEVVANTVTPLMKAQSVPGMAVAVIYQGKPHYYTFGKADIAANKPVTPQTLFELGSIS
ACT-45ULQ22907.1_427EBOC        QLAEVVANTVTPLMKAQSVPGMAVAVIYQGKPHYYTFGKADIAANKPVTPQTLFELGSIS
ACT-45ULQ25748.1_3478EBOC       QLAEVVANTVTPLMKAQSVPGMAVAVIYQGKPHYYTFGKADIAANKPVTPQTLFELGSIS
ACT-45WCO72956.1_42EBOC         QLAEVVANTVTPLMKAQSVPGMAVAVIYQGKPHYYTFGKADIAANKPVTPQTLFELGSIS
ACT-C197QLA02472.1_2022EOC      QLAEVVANTVTPLMKAQSIPGMAVAVIYQGKPHYYTFGKADIAANKPVTPQTLFELGSIS
ACT-64QMT06026.1_4255EBCP       QLSDVVERTVTPLMKAQAIPGMAVAVIYQGHPHYFTFGKADVAANKPVTPQTLFELGSIS
ACT-64QMT06026.1_4255EBOC       QLSDVVERTVTPLMKAQAIPGMAVAVIYQGHPHYFTFGKADVAANKPVTPQTLFELGSIS
ACT-C198QXA51435.1_2071EOC      ELAKVVKRTVTPLMKAQSIPGMAVAVIYQGQPHYFTFGKADVAANTPVTPQTLFELGSVS
                          acc
```

```
                                   α2                     η2      η3       α3
ACT-17BBW34093.1_49417]EBCP    ℓℓℓℓℓℓℓℓℓℓℓℓℓℓ    TT     ℓℓℓ     ℓℓℓ     ℓℓℓℓℓℓ            TT
                                   130       140       150       160       170       180

ACT-17BBW34093.1_49417]EBCP    KTFTGVLGGDAIARGEISLDDPVTRYWPQLTGKQWQGIRMLDLATYTAGGLPLQVPDEVT
ACT-17BBW34093.1_49417]EBOC    KTFTGVLGGDAIARGEISLDDPVTRYWPQLTGKQWQGIRMLDLATYTAGGLPLQVPDEVT
7TI1_1|Chain                   KTFTGVLGGDAIARGEISLDDAVTRYWPQLTGKQWQGIRMLDLATYTAGGLPLQVPDEVT
ACT-37QPO56206.1_411EBCP       KTFTGVLGGDAIARGEISLDDPVIKYWPELTGKQWQGIRMLDLATYTAGGLPLQVPEEVT
ACT-37QPO65286.1_410EBCP       KTFTGVLGGDAIARGEISLDDPVIKYWPELTGKQWQGIRMLDLATYTAGGLPLQVPEEVT
ACT-37UKK25917.1_410EBCP       KTFTGVLGGDAIARGEISLDDPVIKYWPELTGKQWQGIRMLDLATYTAGGLPLQVPEEVT
ACT-37UKK30553.1_410EBCP       KTFTGVLGGDAIARGEISLDDPVIKYWPELTGKQWQGIRMLDLATYTAGGLPLQVPEEVT
ACT-37QPO56206.1_411EBOC       KTFTGVLGGDAIARGEISLDDPVIKYWPELTGKQWQGIRMLDLATYTAGGLPLQVPEEVT
ACT-37QPO65286.1_410EBOC       KTFTGVLGGDAIARGEISLDDPVIKYWPELTGKQWQGIRMLDLATYTAGGLPLQVPEEVT
ACT-37UKK25917.1_410EBOC       KTFTGVLGGDAIARGEISLDDPVIKYWPELTGKQWQGIRMLDLATYTAGGLPLQVPEEVT
ACT-37UKK30553.1_410EBOC       KTFTGVLGGDAIARGEISLDDPVIKYWPELTGKQWQGIRMLDLATYTAGGLPLQVPEEVT
ACT-65AXO42196.1_4001EBCP      KTFTGVLGGDAIARGEISLDDPVTRYWPQLTGKQWQGIRMLDLATYTAGGLPLQVPDEVT
ACT-65AXO42196.1_4001EBOC      KTFTGVLGGDAIARGEISLDDPVTRYWPQLTGKQWQGIRMLDLATYTAGGLPLQVPDEVT
ACT-65WBV31824.1_394EOC        KTFTGVLGGDAIARGEISLDDPVTRYWPQLTGKQWQGIRMLDLATYTAGGLPLQVPDEVT
ACT-17ARZ78489.1_1874EBCP      KTFTGVLGGDAIARGEISLDDPVTRYWPQLTGKQWQGIRMLDLATYTAGGLPLQVPDEVT
ACT-17ASQ75348.1_386EBCP       KTFTGVLGGDAIARGEISLDDPVTRYWPQLTGKQWQGIRMLDLATYTAGGLPLQVPDEVT
ACT-17AWV77932.1_4320EBCP      KTFTGVLGGDAIARGEISLDDPVTRYWPQLTGKQWQGIRMLDLATYTAGGLPLQVPDEVT
ACT-17QEQ46013.1_2619EBCP      KTFTGVLGGDAIARGEISLDDPVTRYWPQLTGKQWQGIRMLDLATYTAGGLPLQVPDEVT
ACT-17QFI20602.1_494EBCP       KTFTGVLGGDAIARGEISLDDPVTRYWPQLTGKQWQGIRMLDLATYTAGGLPLQVPDEVT
ACT-17QIE65424.1_3050EBCP      KTFTGVLGGDAIARGEISLDDPVTRYWPQLTGKQWQGIRMLDLATYTAGGLPLQVPDEVT
ACT-17QPD71520.1_416EBCP       KTFTGVLGGDAIARGEISLDDPVTRYWPQLTGKQWQGIRMLDLATYTAGGLPLQVPDEVT
ACT-17QPO45831.1_587EBCP       KTFTGVLGGDAIARGEISLDDPVTRYWPQLTGKQWQGIRMLDLATYTAGGLPLQVPDEVT
ACT-17QPO51098.1_586EBCP       KTFTGVLGGDAIARGEISLDDPVTRYWPQLTGKQWQGIRMLDLATYTAGGLPLQVPDEVT
ACT-17QPO69921.1_405EBCP       KTFTGVLGGDAIARGEISLDDPVTRYWPQLTGKQWQGIRMLDLATYTAGGLPLQVPDEVT
ACT-17QPO74759.1_405EBCP       KTFTGVLGGDAIARGEISLDDPVTRYWPQLTGKQWQGIRMLDLATYTAGGLPLQVPDEVT
ACT-17QPO85066.1_405EBCP       KTFTGVLGGDAIARGEISLDDPVTRYWPQLTGKQWQGIRMLDLATYTAGGLPLQVPDEVT
ACT-17QSB73424.1_1103EBCP      KTFTGVLGGDAIARGEISLDDPVTRYWPQLTGKQWQGIRMLDLATYTAGGLPLQVPDEVT
ACT-17QXY98988.1_1068EBCP      KTFTGVLGGDAIARGEISLDDPVTRYWPQLTGKQWQGIRMLDLATYTAGGLPLQVPDEVT
ACT-17UDV73425.1_430EBCP       KTFTGVLGGDAIARGEISLDDPVTRYWPQLTGKQWQGIRMLDLATYTAGGLPLQVPDEVT
ACT-17UDV69018.1_894EBCP       KTFTGVLGGDAIARGEISLDDPVTRYWPQLTGKQWQGIRMLDLATYTAGGLPLQVPDEVT
ACT-17UKK21116.1_588EBCP       KTFTGVLGGDAIARGEISLDDPVTRYWPQLTGKQWQGIRMLDLATYTAGGLPLQVPDEVT
ACT-17UOY44701.1_2566EBCP      KTFTGVLGGDAIARGEISLDDPVTRYWPQLTGKQWQGIRMLDLATYTAGGLPLQVPDEVT
ACT-17URL69109.1_2030EBCP      KTFTGVLGGDAIARGEISLDDPVTRYWPQLTGKQWQGIRMLDLATYTAGGLPLQVPDEVT
ACT-17UUQ70490.1_426EBCP       KTFTGVLGGDAIARGEISLDDPVTRYWPQLTGKQWQGIRMLDLATYTAGGLPLQVPDEVT
ACT-17UWA70738.1_427EBCP       KTFTGVLGGDAIARGEISLDDPVTRYWPQLTGKQWQGIRMLDLATYTAGGLPLQVPDEVT
ACT-17UWC98141.1_545EBCP       KTFTGVLGGDAIARGEISLDDPVTRYWPQLTGKQWQGIRMLDLATYTAGGLPLQVPDEVT
ACT-17UXC72711.1_448EBCP       KTFTGVLGGDAIARGEISLDDPVTRYWPQLTGKQWQGIRMLDLATYTAGGLPLQVPDEVT
ACT-17UXC77377.1_448EBCP       KTFTGVLGGDAIARGEISLDDPVTRYWPQLTGKQWQGIRMLDLATYTAGGLPLQVPDEVT
ACT-17UXC82023.1_448EBCP       KTFTGVLGGDAIARGEISLDDPVTRYWPQLTGKQWQGIRMLDLATYTAGGLPLQVPDEVT
ACT-17UZE41799.1_406EBCP       KTFTGVLGGDAIARGEISLDDPVTRYWPQLTGKQWQGIRMLDLATYTAGGLPLQVPDEVT
ACT-17UZH49391.1_396EBCP       KTFTGVLGGDAIARGEISLDDPVTRYWPQLTGKQWQGIRMLDLATYTAGGLPLQVPDEVT
ACT-17WAZ63377.1_405EBCP       KTFTGVLGGDAIARGEISLDDPVTRYWPQLTGKQWQGIRMLDLATYTAGGLPLQVPDEVT
ACT-17WDU86092.1_3769EBCP      KTFTGVLGGDAIARGEISLDDPVTRYWPQLTGKQWQGIRMLDLATYTAGGLPLQVPDEVT
ACT-17WDW12975.1_3764EBCP      KTFTGVLGGDAIARGEISLDDPVTRYWPQLTGKQWQGIRMLDLATYTAGGLPLQVPDEVT
ACT-17WGA71117.1_396EBCP       KTFTGVLGGDAIARGEISLDDPVTRYWPQLTGKQWQGIRMLDLATYTAGGLPLQVPDEVT
ACT-17WGA75603.1_396EBCP       KTFTGVLGGDAIARGEISLDDPVTRYWPQLTGKQWQGIRMLDLATYTAGGLPLQVPDEVT
ACT-17WGZ57121.1_2667EBCP      KTFTGVLGGDAIARGEISLDDPVTRYWPQLTGKQWQGIRMLDLATYTAGGLPLQVPDEVT
ACT-17ARZ78489.1_1874EBOC      KTFTGVLGGDAIARGEISLDDPVTRYWPQLTGKQWQGIRMLDLATYTAGGLPLQVPDEVT
ACT-17ASQ75348.1_386EBOC       KTFTGVLGGDAIARGEISLDDPVTRYWPQLTGKQWQGIRMLDLATYTAGGLPLQVPDEVT
ACT-17AWV77932.1_4320EBOC      KTFTGVLGGDAIARGEISLDDPVTRYWPQLTGKQWQGIRMLDLATYTAGGLPLQVPDEVT
ACT-17QEQ46013.1_2619EBOC      KTFTGVLGGDAIARGEISLDDPVTRYWPQLTGKQWQGIRMLDLATYTAGGLPLQVPDEVT
ACT-17QFI20602.1_494EBOC       KTFTGVLGGDAIARGEISLDDPVTRYWPQLTGKQWQGIRMLDLATYTAGGLPLQVPDEVT
ACT-17QIE65424.1_3050EBOC      KTFTGVLGGDAIARGEISLDDPVTRYWPQLTGKQWQGIRMLDLATYTAGGLPLQVPDEVT
ACT-17QPD71520.1_416EBOC       KTFTGVLGGDAIARGEISLDDPVTRYWPQLTGKQWQGIRMLDLATYTAGGLPLQVPDEVT
ACT-17QPO45831.1_587EBOC       KTFTGVLGGDAIARGEISLDDPVTRYWPQLTGKQWQGIRMLDLATYTAGGLPLQVPDEVT
ACT-17QPO51098.1_586EBOC       KTFTGVLGGDAIARGEISLDDPVTRYWPQLTGKQWQGIRMLDLATYTAGGLPLQVPDEVT
ACT-17QPO69921.1_405EBOC       KTFTGVLGGDAIARGEISLDDPVTRYWPQLTGKQWQGIRMLDLATYTAGGLPLQVPDEVT
ACT-17QPO74759.1_405EBOC       KTFTGVLGGDAIARGEISLDDPVTRYWPQLTGKQWQGIRMLDLATYTAGGLPLQVPDEVT
ACT-17QPO85066.1_405EBOC       KTFTGVLGGDAIARGEISLDDPVTRYWPQLTGKQWQGIRMLDLATYTAGGLPLQVPDEVT
ACT-17QSB73424.1_1103EBOC      KTFTGVLGGDAIARGEISLDDPVTRYWPQLTGKQWQGIRMLDLATYTAGGLPLQVPDEVT
ACT-17QXY98988.1_1068EBOC      KTFTGVLGGDAIARGEISLDDPVTRYWPQLTGKQWQGIRMLDLATYTAGGLPLQVPDEVT
ACT-17UDV73425.1_430EBOC       KTFTGVLGGDAIARGEISLDDPVTRYWPQLTGKQWQGIRMLDLATYTAGGLPLQVPDEVT
ACT-17UDV69018.1_894EBOC       KTFTGVLGGDAIARGEISLDDPVTRYWPQLTGKQWQGIRMLDLATYTAGGLPLQVPDEVT
ACT-17UKK21116.1_588EBOC       KTFTGVLGGDAIARGEISLDDPVTRYWPQLTGKQWQGIRMLDLATYTAGGLPLQVPDEVT
ACT-17UOY44701.1_2566EBOC      KTFTGVLGGDAIARGEISLDDPVTRYWPQLTGKQWQGIRMLDLATYTAGGLPLQVPDEVT
ACT-17URL69109.1_2030EBOC      KTFTGVLGGDAIARGEISLDDPVTRYWPQLTGKQWQGIRMLDLATYTAGGLPLQVPDEVT
ACT-17UUQ70490.1_426EBOC       KTFTGVLGGDAIARGEISLDDPVTRYWPQLTGKQWQGIRMLDLATYTAGGLPLQVPDEVT
ACT-17UWA70738.1_427EBOC       KTFTGVLGGDAIARGEISLDDPVTRYWPQLTGKQWQGIRMLDLATYTAGGLPLQVPDEVT
ACT-17UWC98141.1_545EBOC       KTFTGVLGGDAIARGEISLDDPVTRYWPQLTGKQWQGIRMLDLATYTAGGLPLQVPDEVT
ACT-17UXC72711.1_448EBOC       KTFTGVLGGDAIARGEISLDDPVTRYWPQLTGKQWQGIRMLDLATYTAGGLPLQVPDEVT
ACT-17UXC77377.1_448EBOC       KTFTGVLGGDAIARGEISLDDPVTRYWPQLTGKQWQGIRMLDLATYTAGGLPLQVPDEVT
ACT-17UXC82023.1_448EBOC       KTFTGVLGGDAIARGEISLDDPVTRYWPQLTGKQWQGIRMLDLATYTAGGLPLQVPDEVT
ACT-17UZE41799.1_406EBOC       KTFTGVLGGDAIARGEISLDDPVTRYWPQLTGKQWQGIRMLDLATYTAGGLPLQVPDEVT
ACT-17UZH49391.1_396EBOC       KTFTGVLGGDAIARGEISLDDPVTRYWPQLTGKQWQGIRMLDLATYTAGGLPLQVPDEVT
ACT-17WAZ63377.1_405EBOC       KTFTGVLGGDAIARGEISLDDPVTRYWPQLTGKQWQGIRMLDLATYTAGGLPLQVPDEVT
ACT-17WDU86092.1_3769EBOC      KTFTGVLGGDAIARGEISLDDPVTRYWPQLTGKQWQGIRMLDLATYTAGGLPLQVPDEVT
ACT-17WDW12975.1_3764EBOC      KTFTGVLGGDAIARGEISLDDPVTRYWPQLTGKQWQGIRMLDLATYTAGGLPLQVPDEVT
ACT-17WGA71117.1_396EBOC       KTFTGVLGGDAIARGEISLDDPVTRYWPQLTGKQWQGIRMLDLATYTAGGLPLQVPDEVT
ACT-17WGA75603.1_396EBOC       KTFTGVLGGDAIARGEISLDDPVTRYWPQLTGKQWQGIRMLDLATYTAGGLPLQVPDEVT
ACT-17WGZ57121.1_2667EBOC      KTFTGVLGGDAIARGEISLDDPVTRYWPQLTGKQWQGIRMLDLATYTAGGLPLQVPDEVT
ACT-17QEI60759.1_389EOC        KTFTGVLGGDAIARGEISLDDPVTRYWPQLTGKQWQGIRMLDLATYTAGGLPLQVPDEVT
ACT-17QEI70010.1_389EOC        KTFTGVLGGDAIARGEISLDDPVTRYWPQLTGKQWQGIRMLDLATYTAGGLPLQVPDEVT
ACT-17QEU13386.1_612EOC        KTFTGVLGGDAIARGEISLDDPVTRYWPQLTGKQWQGIRMLDLATYTAGGLPLQVPDEVT
ACT-17QHI59365.1_3674EOC       KTFTGVLGGDAIARGEISLDDPVTRYWPQLTGKQWQGIRMLDLATYTAGGLPLQVPDEVT
ACT-17UZQ58984.1_410EOC        KTFTGVLGGDAIARGEISLDDPVTRYWPQLTGKQWQGIRMLDLATYTAGGLPLQVPDEVT
ACT-40AWS79793.1_3058EBCP      KTFTAVLGGDAIARGEISLDDPVTRYWPQLTGKQWQGIRMLDLATYTAGGLPLQVPDEVT
ACT-40AXO44745.1_1607EBCP      KTFTAVLGGDAIARGEISLDDPVTRYWPQLTGKQWQGIRMLDLATYTAGGLPLQVPDEVT
ACT-40UXI63958.1_1746EBCP      KTFTAVLGGDAIARGEISLDDPVTRYWPQLTGKQWQGIRMLDLATYTAGGLPLQVPDEVT
ACT-40WDC52451.1_4284EBCP      KTFTAVLGGDAIARGEISLDDPVTRYWPQLTGKQWQGIRMLDLATYTAGGLPLQVPDEVT
ACT-40AWS79793.1_3058EBOC      KTFTAVLGGDAIARGEISLDDPVTRYWPQLTGKQWQGIRMLDLATYTAGGLPLQVPDEVT
ACT-40AXO44745.1_1607EBOC      KTFTAVLGGDAIARGEISLDDPVTRYWPQLTGKQWQGIRMLDLATYTAGGLPLQVPDEVT
ACT-40UXI63958.1_1746EBOC      KTFTAVLGGDAIARGEISLDDPVTRYWPQLTGKQWQGIRMLDLATYTAGGLPLQVPDEVT
ACT-40WDC52451.1_4284EBOC      KTFTAVLGGDAIARGEISLDDPVTRYWPQLTGKQWQGIRMLDLATYTAGGLPLQVPDEVT
ACT-44UWA09346.1_3724EBCP      KTFTGVLGGDAIARGEISLDDPVTRYWPQLTGKQWQGIRMLDLATYTAGGLPLQVPDEVT
ACT-44UWA09346.1_3724EBOC      KTFTGVLGGDAIARGEISLDDPVTRYWPQLTGKQWQGIRMLDLATYTAGGLPLQVPDEVT
ACT-90QFH83806.1_450EBCP       KTFTGVLGGDAIARGEISLDDPVTRYWPQLTGKQWQGIRMLDLATYTAGGLPLQVPDEVT
ACT-90QLO92194.1_500EBCP       KTFTGVLGGDAIARGEISLDDPVTRYWPQLTGKQWQGIRMLDLATYTAGGLPLQVPDEVT
ACT-90QLP01381.1_466EBCP       KTFTGVLGGDAIARGEISLDDPVTRYWPQLTGKQWQGIRMLDLATYTAGGLPLQVPDEVT
ACT-90QFH83806.1_450EBOC       KTFTGVLGGDAIARGEISLDDPVTRYWPQLTGKQWQGIRMLDLATYTAGGLPLQVPDEVT
ACT-90QLO92194.1_500EBOC       KTFTGVLGGDAIARGEISLDDPVTRYWPQLTGKQWQGIRMLDLATYTAGGLPLQVPDEVT
ACT-90QLP01381.1_466EBOC       KTFTGVLGGDAIARGEISLDDPVTRYWPQLTGKQWQGIRMLDLATYTAGGLPLQVPDEVT
ACT-24ASB73507.1_1185EBCP      KTFTGVLGGDAIARGEISLDDPVTRYWPQLTGKQWQGIRMLDLATYTAGGLPLQVPDEVT
ACT-24AVU18445.1_378EBCP       KTFTGVLGGDAIARGEISLDDPVTRYWPQLTGKQWQGIRMLDLATYTAGGLPLQVPDEVT
```

```
ACT-24QHC75881.1_374EBCP      KTFTGVLGGDAIARGEISLDDPVTRYWPQLTGKQWQGIRMLDLATYTAGGLPLQVPDEVT
ACT-24QLP84500.1_370EBCP      KTFTGVLGGDAIARGEISLDDPVTRYWPQLTGKQWQGIRMLDLATYTAGGLPLQVPDEVT
ACT-24QSH03071.1_370EBCP      KTFTGVLGGDAIARGEISLDDPVTRYWPQLTGKQWQGIRMLDLATYTAGGLPLQVPDEVT
ACT-24QSG93844.1_370EBCP      KTFTGVLGGDAIARGEISLDDPVTRYWPQLTGKQWQGIRMLDLATYTAGGLPLQVPDEVT
ACT-24QSG98466.1_370EBCP      KTFTGVLGGDAIARGEISLDDPVTRYWPQLTGKQWQGIRMLDLATYTAGGLPLQVPDEVT
ACT-24QSH12187.1_370EBCP      KTFTGVLGGDAIARGEISLDDPVTRYWPQLTGKQWQGIRMLDLATYTAGGLPLQVPDEVT
ACT-24QSH16801.1_370EBCP      KTFTGVLGGDAIARGEISLDDPVTRYWPQLTGKQWQGIRMLDLATYTAGGLPLQVPDEVT
ACT-24QSH07573.1_370EBCP      KTFTGVLGGDAIARGEISLDDPVTRYWPQLTGKQWQGIRMLDLATYTAGGLPLQVPDEVT
ACT-24QSH21409.1_370EBCP      KTFTGVLGGDAIARGEISLDDPVTRYWPQLTGKQWQGIRMLDLATYTAGGLPLQVPDEVT
ACT-24QSH26023.1_370EBCP      KTFTGVLGGDAIARGEISLDDPVTRYWPQLTGKQWQGIRMLDLATYTAGGLPLQVPDEVT
ACT-24QSH30639.1_370EBCP      KTFTGVLGGDAIARGEISLDDPVTRYWPQLTGKQWQGIRMLDLATYTAGGLPLQVPDEVT
ACT-24UIL48372.1_372EBCP      KTFTGVLGGDAIARGEISLDDPVTRYWPQLTGKQWQGIRMLDLATYTAGGLPLQVPDEVT
ACT-24UIM14687.1_372EBCP      KTFTGVLGGDAIARGEISLDDPVTRYWPQLTGKQWQGIRMLDLATYTAGGLPLQVPDEVT
ACT-24UXA74146.1_371EBCP      KTFTGVLGGDAIARGEISLDDPVTRYWPQLTGKQWQGIRMLDLATYTAGGLPLQVPDEVT
ACT-24WDY48721.1_372EBCP      KTFTGVLGGDAIARGEISLDDPVTRYWPQLTGKQWQGIRMLDLATYTAGGLPLQVPDEVT
ACT-24ASB73507.1_1185EBOC     KTFTGVLGGDAIARGEISLDDPVTRYWPQLTGKQWQGIRMLDLATYTAGGLPLQVPDEVT
ACT-24AVU18445.1_378EBOC      KTFTGVLGGDAIARGEISLDDPVTRYWPQLTGKQWQGIRMLDLATYTAGGLPLQVPDEVT
ACT-24QHC75881.1_374EBOC      KTFTGVLGGDAIARGEISLDDPVTRYWPQLTGKQWQGIRMLDLATYTAGGLPLQVPDEVT
ACT-24QLP84500.1_370EBOC      KTFTGVLGGDAIARGEISLDDPVTRYWPQLTGKQWQGIRMLDLATYTAGGLPLQVPDEVT
ACT-24QSH03071.1_370EBOC      KTFTGVLGGDAIARGEISLDDPVTRYWPQLTGKQWQGIRMLDLATYTAGGLPLQVPDEVT
ACT-24QSG93844.1_370EBOC      KTFTGVLGGDAIARGEISLDDPVTRYWPQLTGKQWQGIRMLDLATYTAGGLPLQVPDEVT
ACT-24QSG98466.1_370EBOC      KTFTGVLGGDAIARGEISLDDPVTRYWPQLTGKQWQGIRMLDLATYTAGGLPLQVPDEVT
ACT-24QSH12187.1_370EBOC      KTFTGVLGGDAIARGEISLDDPVTRYWPQLTGKQWQGIRMLDLATYTAGGLPLQVPDEVT
ACT-24QSH16801.1_370EBOC      KTFTGVLGGDAIARGEISLDDPVTRYWPQLTGKQWQGIRMLDLATYTAGGLPLQVPDEVT
ACT-24QSH07573.1_370EBOC      KTFTGVLGGDAIARGEISLDDPVTRYWPQLTGKQWQGIRMLDLATYTAGGLPLQVPDEVT
ACT-24QSH21409.1_370EBOC      KTFTGVLGGDAIARGEISLDDPVTRYWPQLTGKQWQGIRMLDLATYTAGGLPLQVPDEVT
ACT-24QSH26023.1_370EBOC      KTFTGVLGGDAIARGEISLDDPVTRYWPQLTGKQWQGIRMLDLATYTAGGLPLQVPDEVT
ACT-24QSH30639.1_370EBOC      KTFTGVLGGDAIARGEISLDDPVTRYWPQLTGKQWQGIRMLDLATYTAGGLPLQVPDEVT
ACT-24UIL48372.1_372EBOC      KTFTGVLGGDAIARGEISLDDPVTRYWPQLTGKQWQGIRMLDLATYTAGGLPLQVPDEVT
ACT-24UIM14687.1_372EBOC      KTFTGVLGGDAIARGEISLDDPVTRYWPQLTGKQWQGIRMLDLATYTAGGLPLQVPDEVT
ACT-24UXA74146.1_371EBOC      KTFTGVLGGDAIARGEISLDDPVTRYWPQLTGKQWQGIRMLDLATYTAGGLPLQVPDEVT
ACT-24WDY48721.1_372EBOC      KTFTGVLGGDAIARGEISLDDPVTRYWPQLTGKQWQGIRMLDLATYTAGGLPLQVPDEVT
ACT-89AVE73204.1_2453EBCP     KTFTGVLGGDAIARGEISLDDAVTRYWPQLTGKQWQGIRMLDLATYTAGGLPLQVPDEVT
ACT-89AVE73204.1_2453EBOC     KTFTGVLGGDAIARGEISLDDAVTRYWPQLTGKQWQGIRMLDLATYTAGGLPLQVPDEVT
ACT-56ASO99603.1_1270EBCP     KTFTGVLGGDAIARGEISLDDPVTRYWPQLTGKQWQGIRMLDLATYTAGGLPLQVPDEVT
ACT-56QDQ75241.1_392EBCP      KTFTGVLGGDAIARGEISLDDPVTRYWPQLTGKQWQGIRMLDLATYTAGGLPLQVPDEVT
ACT-56QFH98918.1_395EBCP      KTFTGVLGGDAIARGEISLDDPVTRYWPQLTGKQWQGIRMLDLATYTAGGLPLQVPDEVT
ACT-56QFH34334.1_395EBCP      KTFTGVLGGDAIARGEISLDDPVTRYWPQLTGKQWQGIRMLDLATYTAGGLPLQVPDEVT
ACT-56QFI32707.1_2233EBCP     KTFTGVLGGDAIARGEISLDDPVTRYWPQLTGKQWQGIRMLDLATYTAGGLPLQVPDEVT
ACT-56QMI65633.1_4184EBCP     KTFTGVLGGDAIARGEISLDDPVTRYWPQLTGKQWQGIRMLDLATYTAGGLPLQVPDEVT
ACT-56QMI71192.1_422EBCP      KTFTGVLGGDAIARGEISLDDPVTRYWPQLTGKQWQGIRMLDLATYTAGGLPLQVPDEVT
ACT-56UDV17856.1_8EBCP        KTFTGVLGGDAIARGEISLDDPVTRYWPQLTGKQWQGIRMLDLATYTAGGLPLQVPDEVT
ACT-56UDV39132.1_1376EBCP     KTFTGVLGGDAIARGEISLDDPVTRYWPQLTGKQWQGIRMLDLATYTAGGLPLQVPDEVT
ACT-56UWB10427.1_430EBCP      KTFTGVLGGDAIARGEISLDDPVTRYWPQLTGKQWQGIRMLDLATYTAGGLPLQVPDEVT
ACT-56UWC18315.1_415EBCP      KTFTGVLGGDAIARGEISLDDPVTRYWPQLTGKQWQGIRMLDLATYTAGGLPLQVPDEVT
ACT-56UWC23093.1_417EBCP      KTFTGVLGGDAIARGEISLDDPVTRYWPQLTGKQWQGIRMLDLATYTAGGLPLQVPDEVT
ACT-56WBT12607.1_393EBCP      KTFTGVLGGDAIARGEISLDDPVTRYWPQLTGKQWQGIRMLDLATYTAGGLPLQVPDEVT
ACT-56WBT28831.1_393EBCP      KTFTGVLGGDAIARGEISLDDPVTRYWPQLTGKQWQGIRMLDLATYTAGGLPLQVPDEVT
ACT-56WGB34617.1_392EBCP      KTFTGVLGGDAIARGEISLDDPVTRYWPQLTGKQWQGIRMLDLATYTAGGLPLQVPDEVT
ACT-56WGU03466.1_393EBCP      KTFTGVLGGDAIARGEISLDDPVTRYWPQLTGKQWQGIRMLDLATYTAGGLPLQVPDEVT
ACT-56ASO99603.1_1270EBOC     KTFTGVLGGDAIARGEISLDDPVTRYWPQLTGKQWQGIRMLDLATYTAGGLPLQVPDEVT
ACT-56QDQ75241.1_392EBOC      KTFTGVLGGDAIARGEISLDDPVTRYWPQLTGKQWQGIRMLDLATYTAGGLPLQVPDEVT
ACT-56QFH98918.1_395EBOC      KTFTGVLGGDAIARGEISLDDPVTRYWPQLTGKQWQGIRMLDLATYTAGGLPLQVPDEVT
ACT-56QFH34334.1_395EBOC      KTFTGVLGGDAIARGEISLDDPVTRYWPQLTGKQWQGIRMLDLATYTAGGLPLQVPDEVT
ACT-56QFI32707.1_2233EBOC     KTFTGVLGGDAIARGEISLDDPVTRYWPQLTGKQWQGIRMLDLATYTAGGLPLQVPDEVT
ACT-56QMI65633.1_4184EBOC     KTFTGVLGGDAIARGEISLDDPVTRYWPQLTGKQWQGIRMLDLATYTAGGLPLQVPDEVT
ACT-56QMI71192.1_422EBOC      KTFTGVLGGDAIARGEISLDDPVTRYWPQLTGKQWQGIRMLDLATYTAGGLPLQVPDEVT
ACT-56UDV17856.1_8EBOC        KTFTGVLGGDAIARGEISLDDPVTRYWPQLTGKQWQGIRMLDLATYTAGGLPLQVPDEVT
ACT-56UDV39132.1_1376EBOC     KTFTGVLGGDAIARGEISLDDPVTRYWPQLTGKQWQGIRMLDLATYTAGGLPLQVPDEVT
ACT-56UWB10427.1_430EBOC      KTFTGVLGGDAIARGEISLDDPVTRYWPQLTGKQWQGIRMLDLATYTAGGLPLQVPDEVT
ACT-56UWC18315.1_415EBOC      KTFTGVLGGDAIARGEISLDDPVTRYWPQLTGKQWQGIRMLDLATYTAGGLPLQVPDEVT
ACT-56UWC23093.1_417EBOC      KTFTGVLGGDAIARGEISLDDPVTRYWPQLTGKQWQGIRMLDLATYTAGGLPLQVPDEVT
ACT-56WBT12607.1_393EBOC      KTFTGVLGGDAIARGEISLDDPVTRYWPQLTGKQWQGIRMLDLATYTAGGLPLQVPDEVT
ACT-56WBT28831.1_393EBOC      KTFTGVLGGDAIARGEISLDDPVTRYWPQLTGKQWQGIRMLDLATYTAGGLPLQVPDEVT
ACT-56WGB34617.1_392EBOC      KTFTGVLGGDAIARGEISLDDPVTRYWPQLTGKQWQGIRMLDLATYTAGGLPLQVPDEVT
ACT-56WGU03466.1_393EBOC      KTFTGVLGGDAIARGEISLDDPVTRYWPQLTGKQWQGIRMLDLATYTAGGLPLQVPDEVT
ACT-56AOP76464.1_392EOC       KTFTGVLGGDAIARGEISLDDPVTRYWPQLTGKQWQGIRMLDLATYTAGGLPLQVPDEVT
ACT-56QXA77046.1_2672EOC      KTFTGVLGGDAIARGEISLDDPVTRYWPQLTGKQWQGIRMLDLATYTAGGLPLQVPDEVT
ACT-56WFP29817.1_397EOC       KTFTGVLGGDAIARGEISLDDPVTRYWPQLTGKQWQGIRMLDLATYTAGGLPLQVPDEVT
ACT-27AXO49795.1_1610EBCP     KTFTGVLGGDAIARGEISLDDPVTRYWPELTGKQWQGIRMLDLATYTAGGLPLQVPDEVT
ACT-27QHO79915.1_457EBCP      KTFTGVLGGDAIARGEISLDDPVTRYWPELTGKQWQGIRMLDLATYTAGGLPLQVPDEVT
ACT-27QHO97012.1_458EBCP      KTFTGVLGGDAIARGEISLDDPVTRYWPELTGKQWQGIRMLDLATYTAGGLPLQVPDEVT
ACT-27QQH03712.1_3802EBCP     KTFTGVLGGDAIARGEISLDDPVTRYWPELTGKQWQGIRMLDLATYTAGGLPLQVPDEVT
ACT-27QQH08784.1_3802EBCP     KTFTGVLGGDAIARGEISLDDPVTRYWPELTGKQWQGIRMLDLATYTAGGLPLQVPDEVT
ACT-27UVZ91365.1_388EBCP      KTFTGVLGGDAIARGEISLDDPVTRYWPELTGKQWQGIRMLDLATYTAGGLPLQVPDEVT
ACT-27AXO49795.1_1610EBOC     KTFTGVLGGDAIARGEISLDDPVTRYWPELTGKQWQGIRMLDLATYTAGGLPLQVPDEVT
ACT-27QHO79915.1_457EBOC      KTFTGVLGGDAIARGEISLDDPVTRYWPELTGKQWQGIRMLDLATYTAGGLPLQVPDEVT
ACT-27QHO97012.1_458EBOC      KTFTGVLGGDAIARGEISLDDPVTRYWPELTGKQWQGIRMLDLATYTAGGLPLQVPDEVT
ACT-27QQH03712.1_3802EBOC     KTFTGVLGGDAIARGEISLDDPVTRYWPELTGKQWQGIRMLDLATYTAGGLPLQVPDEVT
ACT-27QQH08784.1_3802EBOC     KTFTGVLGGDAIARGEISLDDPVTRYWPELTGKQWQGIRMLDLATYTAGGLPLQVPDEVT
ACT-27UVZ91365.1_388EBOC      KTFTGVLGGDAIARGEISLDDPVTRYWPELTGKQWQGIRMLDLATYTAGGLPLQVPDEVT
ACT-102QEO03155.1_4213EBCP    KTFTGVLGGDAIARGEISLGDPVTKYWPELTGKQWQGIRMLDLATYTAGGLPLQVPDEVT
ACT-102BBV79841.1_51928]EBCP  KTFTGVLGGDAIARGEISLGDPVTKYWPELTGKQWQGIRMLDLATYTAGGLPLQVPDEVT
ACT-102QZS47739.1_401EBCP     KTFTGVLGGDAIARGEISLGDPVTKYWPELTGKQWQGIRMLDLATYTAGGLPLQVPDEVT
ACT-102QEO03155.1_4213EBOC    KTFTGVLGGDAIARGEISLGDPVTKYWPELTGKQWQGIRMLDLATYTAGGLPLQVPDEVT
ACT-102BBV79841.1_51928]EBOC  KTFTGVLGGDAIARGEISLGDPVTKYWPELTGKQWQGIRMLDLATYTAGGLPLQVPDEVT
ACT-102QZS47739.1_401EBOC     KTFTGVLGGDAIARGEISLGDPVTKYWPELTGKQWQGIRMLDLATYTAGGLPLQVPDEVT
ACT-55QLP71015.1_413EBCP      KTFTGVLGGDAIARGEISLDDPVTRYWPQLTGKQWQGIRMLDLATYTAGGLPLQVPDEIT
ACT-55QLP80034.1_414EBCP      KTFTGVLGGDAIARGEISLDDPVTRYWPQLTGKQWQGIRMLDLATYTAGGLPLQVPDEIT
ACT-55QLS08818.1_415EBCP      KTFTGVLGGDAIARGEISLDDPVTRYWPQLTGKQWQGIRMLDLATYTAGGLPLQVPDEIT
ACT-55QLT25377.1_3621EBCP     KTFTGVLGGDAIARGEISLDDPVTRYWPQLTGKQWQGIRMLDLATYTAGGLPLQVPDEIT
ACT-55QLV12510.1_4006EBCP     KTFTGVLGGDAIARGEISLDDPVTRYWPQLTGKQWQGIRMLDLATYTAGGLPLQVPDEIT
ACT-55QLW04180.1_4182EBCP     KTFTGVLGGDAIARGEISLDDPVTRYWPQLTGKQWQGIRMLDLATYTAGGLPLQVPDEIT
ACT-55QLV08026.1_4006EBCP     KTFTGVLGGDAIARGEISLDDPVTRYWPQLTGKQWQGIRMLDLATYTAGGLPLQVPDEIT
ACT-55QLV96171.1_421EBCP      KTFTGVLGGDAIARGEISLDDPVTRYWPQLTGKQWQGIRMLDLATYTAGGLPLQVPDEIT
ACT-55QLW44293.1_405EBCP      KTFTGVLGGDAIARGEISLDDPVTRYWPQLTGKQWQGIRMLDLATYTAGGLPLQVPDEIT
ACT-55UBH32480.1_675EBCP      KTFTGVLGGDAIARGEISLDDPVTRYWPQLTGKQWQGIRMLDLATYTAGGLPLQVPDEIT
ACT-55UDV61223.1_2251EBCP     KTFTGVLGGDAIARGEISLDDPVTRYWPQLTGKQWQGIRMLDLATYTAGGLPLQVPDEIT
ACT-55QLP71015.1_413EBOC      KTFTGVLGGDAIARGEISLDDPVTRYWPQLTGKQWQGIRMLDLATYTAGGLPLQVPDEIT
ACT-55QLP80034.1_414EBOC      KTFTGVLGGDAIARGEISLDDPVTRYWPQLTGKQWQGIRMLDLATYTAGGLPLQVPDEIT
ACT-55QLS08818.1_415EBOC      KTFTGVLGGDAIARGEISLDDPVTRYWPQLTGKQWQGIRMLDLATYTAGGLPLQVPDEIT
ACT-55QLT25377.1_3621EBOC     KTFTGVLGGDAIARGEISLDDPVTRYWPQLTGKQWQGIRMLDLATYTAGGLPLQVPDEIT
ACT-55QLV12510.1_4006EBOC     KTFTGVLGGDAIARGEISLDDPVTRYWPQLTGKQWQGIRMLDLATYTAGGLPLQVPDEIT
```

ACT-55QLW04180.1_4182EBOC	KTFTGVLGGDAIARGEISLDDPVTRYWPQLTGKQWQGIRMLDLATYTAGGLPLQVPDEIT
ACT-55QLV08026.1_4006EBOC	KTFTGVLGGDAIARGEISLDDPVTRYWPQLTGKQWQGIRMLDLATYTAGGLPLQVPDEIT
ACT-55QLV96171.1_421EBOC	KTFTGVLGGDAIARGEISLDDPVTRYWPQLTGKQWQGIRMLDLATYTAGGLPLQVPDEIT
ACT-55QLW44293.1_405EBOC	KTFTGVLGGDAIARGEISLDDPVTRYWPQLTGKQWQGIRMLDLATYTAGGLPLQVPDEIT
ACT-55UBH32480.1_675EBOC	KTFTGVLGGDAIARGEISLDDPVTRYWPQLTGKQWQGIRMLDLATYTAGGLPLQVPDEIT
ACT-55UDV61223.1_2251EBOC	KTFTGVLGGDAIARGEISLDDPVTRYWPQLTGKQWQGIRMLDLATYTAGGLPLQVPDEIT
ACT-55AOP80857.1_409EOC	KTFTGVLGGDAIARGEISLDDPVTRYWPQLTGKQWQGIRMLDLATYTAGGLPLQVPDEIT
ACT-51BBW20048.1_38351]EBCP	KTFTGVLGGDAIARGEISLGDPVTKYWPELTGKQWQGIRMLDLATYTAGGLPLQVPDEVT
ACT-51BBW20048.1_38351]EBOC	KTFTGVLGGDAIARGEISLGDPVTKYWPELTGKQWQGIRMLDLATYTAGGLPLQVPDEVT
ACT-28URE95606.1_351EBCP	KTFTGVLGGDAIARGEISLGDPVTKYWPELTGKQWQGIRMLDLATYTAGGLPLQVPDEVT
ACT-28URE95606.1_351EBOC	KTFTGVLGGDAIARGEISLGDPVTKYWPELTGKQWQGIRMLDLATYTAGGLPLQVPDEVT
ACT-87AYL04658.1_1249EBCP	KTFTGVLGGDAIARGEISLGDPVTKYWPELTGKQWQGISMLDLATYTAGGLPLQVPDEVT
ACT-87QIP18425.1_352EBCP	KTFTGVLGGDAIARGEISLGDPVTKYWPELTGKQWQGISMLDLATYTAGGLPLQVPDEVT
ACT-87AYL04658.1_1249EBOC	KTFTGVLGGDAIARGEISLGDPVTKYWPELTGKQWQGISMLDLATYTAGGLPLQVPDEVT
ACT-87QIP18425.1_352EBOC	KTFTGVLGGDAIARGEISLGDPVTKYWPELTGKQWQGISMLDLATYTAGGLPLQVPDEVT
ACT-75AWX01586.1_1447EBCP	KTFTGVLGGDAIARGEISLDDPVTRYWPQLTGKQWQGIRMLDLATYTAGGLPLQVPDEVT
ACT-75AWX01586.1_1447EBOC	KTFTGVLGGDAIARGEISLDDPVTRYWPQLTGKQWQGIRMLDLATYTAGGLPLQVPDEVT
ACT-103URL27837.1_342EOC	KTFTGVLGGDAIARGEISLGDPVTKYWPELTGKQWQGIRMLDLATYTAGGLPLQVPDEVT
ACT-99UXJ64669.1_346EBCP	KTFTGVLGGDAIARGEISLGDPVAKYWPELTGKQWQGIRMLDLATYTAGGLPLQVPDEVT
ACT-99UXJ64669.1_346EBOC	KTFTGVLGGDAIARGEISLGDPVAKYWPELTGKQWQGIRMLDLATYTAGGLPLQVPDEVT
ACT-95QIB80518.1_348EBCP	KTFTGVLGGDAIARGEISLGDPVTKYWPELTGKQWQGIRMLDLATYTAGGLPLQVPDEVT
ACT-95BBV69349.1_35128]EBCP	KTFTGVLGGDAIARGEISLGDPVTKYWPELTGKQWQGIRMLDLATYTAGGLPLQVPDEVT
ACT-95UZQ68133.1_388EBCP	KTFTGVLGGDAIARGEISLGDPVTKYWPELTGKQWQGIRMLDLATYTAGGLPLQVPDEVT
ACT-95QIB80518.1_348EBOC	KTFTGVLGGDAIARGEISLGDPVTKYWPELTGKQWQGIRMLDLATYTAGGLPLQVPDEVT
ACT-95BBV69349.1_35128]EBOC	KTFTGVLGGDAIARGEISLGDPVTKYWPELTGKQWQGIRMLDLATYTAGGLPLQVPDEVT
ACT-95UZQ68133.1_388EBOC	KTFTGVLGGDAIARGEISLGDPVTKYWPELTGKQWQGIRMLDLATYTAGGLPLQVPDEVT
ACT-52QFH88560.1_350EBCP	KTFTGVLGGDAIARGEISLGDPVTKYWPELTGKQWQGIRMLDLATYTAGGLPLQVPDEVT
ACT-52BBS30224.1_43052]EBCP	KTFTGVLGGDAIARGEISLGDPVTKYWPELTGKQWQGIRMLDLATYTAGGLPLQVPDEVT
ACT-52WBN86316.1_4083EBCP	KTFTGVLGGDAIARGEISLGDPVTKYWPELTGKQWQGIRMLDLATYTAGGLPLQVPDEVT
ACT-52QFH88560.1_350EBOC	KTFTGVLGGDAIARGEISLGDPVTKYWPELTGKQWQGIRMLDLATYTAGGLPLQVPDEVT
ACT-52BBS30224.1_43052]EBOC	KTFTGVLGGDAIARGEISLGDPVTKYWPELTGKQWQGIRMLDLATYTAGGLPLQVPDEVT
ACT-52WBN86316.1_4083EBOC	KTFTGVLGGDAIARGEISLGDPVTKYWPELTGKQWQGIRMLDLATYTAGGLPLQVPDEVT
ACT-4QLR27288.1_395EBCP	KTFTGVLGGDAIARGEISLGDPVTKYWPELTGKQWQGIRMLDLATYTAGGLPLQVPDEVA
ACT-4QPS68257.1_413EBCP	KTFTGVLGGDAIARGEISLGDPVTKYWPELTGKQWQGIRMLDLATYTAGGLPLQVPDEVA
ACT-4QLR27288.1_395EBOC	KTFTGVLGGDAIARGEISLGDPVTKYWPELTGKQWQGIRMLDLATYTAGGLPLQVPDEVA
ACT-4QPS68257.1_413EBOC	KTFTGVLGGDAIARGEISLGDPVTKYWPELTGKQWQGIRMLDLATYTAGGLPLQVPDEVA
ACT-3UOY53510.1_358EBCP	KTFTGVLGGDAIARGEISLGDPVTKYWPELTGKQWQGIRMLDLATYTAGGLPLQVPDEVT
ACT-3UOY63785.1_4171EBCP	KTFTGVLGGDAIARGEISLGDPVTKYWPELTGKQWQGIRMLDLATYTAGGLPLQVPDEVT
ACT-3UOY53510.1_358EBOC	KTFTGVLGGDAIARGEISLGDPVTKYWPELTGKQWQGIRMLDLATYTAGGLPLQVPDEVT
ACT-3UOY63785.1_4171EBOC	KTFTGVLGGDAIARGEISLGDPVTKYWPELTGKQWQGIRMLDLATYTAGGLPLQVPDEVT
ACT-1QLV61731.1_472EBCP	KTFTGVLGGDAIARGEISLGDPVTKYWPELTGKQWQGIRMLDLATYTAGGLPLQVPDEVK
ACT-1UWA77567.1_382EBCP	KTFTGVLGGDAIARGEISLGDPVTKYWPELTGKQWQGIRMLDLATYTAGGLPLQVPDEVK
ACT-1QLV61731.1_472EBOC	KTFTGVLGGDAIARGEISLGDPVTKYWPELTGKQWQGIRMLDLATYTAGGLPLQVPDEVK
ACT-1UWA77567.1_382EBOC	KTFTGVLGGDAIARGEISLGDPVTKYWPELTGKQWQGIRMLDLATYTAGGLPLQVPDEVK
ACT-77QWZ50133.1_330EBCP	KTFTGVLGGDAIARGEISLGDPVTKYWPDLTGKQWQGIRMLDLATYTAGGLPLQVPDEVT
ACT-77UBH39796.1_3999EBCP	KTFTGVLGGDAIARGEISLGDPVTKYWPDLTGKQWQGIRMLDLATYTAGGLPLQVPDEVT
ACT-77UBH98103.1_3842EBCP	KTFTGVLGGDAIARGEISLGDPVTKYWPDLTGKQWQGIRMLDLATYTAGGLPLQVPDEVT
ACT-77UBH94108.1_193EBCP	KTFTGVLGGDAIARGEISLGDPVTKYWPDLTGKQWQGIRMLDLATYTAGGLPLQVPDEVT
ACT-77QWZ50133.1_330EBOC	KTFTGVLGGDAIARGEISLGDPVTKYWPDLTGKQWQGIRMLDLATYTAGGLPLQVPDEVT
ACT-77UBH39796.1_3999EBOC	KTFTGVLGGDAIARGEISLGDPVTKYWPDLTGKQWQGIRMLDLATYTAGGLPLQVPDEVT
ACT-77UBH98103.1_3842EBOC	KTFTGVLGGDAIARGEISLGDPVTKYWPDLTGKQWQGIRMLDLATYTAGGLPLQVPDEVT
ACT-77UBH94108.1_193EBOC	KTFTGVLGGDAIARGEISLGDPVTKYWPDLTGKQWQGIRMLDLATYTAGGLPLQVPDEVT
ACT-53QEL38380.1_485EBCP	KTFTGVLGGDAVARGEISLGDPVTKYWPALTGKQWQGIRMLDLATYTAGGLPLQVPDDVT
ACT-53QEL38380.1_485EBOC	KTFTGVLGGDAVARGEISLGDPVTKYWPALTGKQWQGIRMLDLATYTAGGLPLQVPDDVT
ACT-57QBB07838.1_4703EBCP	KTFTGVLGGDAIARGEISLGDPVTKYWPELTGKQWQGIRMLDLATYTAGGLPLQVPDEVT
ACT-57QYD27335.1_499EBCP	KTFTGVLGGDAIARGEISLGDPVTKYWPELTGKQWQGIRMLDLATYTAGGLPLQVPDEVT
ACT-57UBM17197.1_2672EBCP	KTFTGVLGGDAIARGEISLGDPVTKYWPELTGKQWQGIRMLDLATYTAGGLPLQVPDEVT
ACT-57UKB55021.1_380EBCP	KTFTGVLGGDAIARGEISLGDPVTKYWPELTGKQWQGIRMLDLATYTAGGLPLQVPDEVT
ACT-57QBB07838.1_4703EBOC	KTFTGVLGGDAIARGEISLGDPVTKYWPELTGKQWQGIRMLDLATYTAGGLPLQVPDEVT
ACT-57QYD27335.1_499EBOC	KTFTGVLGGDAIARGEISLGDPVTKYWPELTGKQWQGIRMLDLATYTAGGLPLQVPDEVT
ACT-57UBM17197.1_2672EBOC	KTFTGVLGGDAIARGEISLGDPVTKYWPELTGKQWQGIRMLDLATYTAGGLPLQVPDEVT
ACT-57UKB55021.1_380EBOC	KTFTGVLGGDAIARGEISLGDPVTKYWPELTGKQWQGIRMLDLATYTAGGLPLQVPDEVT
ACT-93ALL15984.1_396EOC	KTFTGVLGGDAIARGEISLGDPVTKYWPELTGKQWQGIRMLDLATYTAGGLPLQVPDEVT
ACT-9AOP85131.1_312EBCP	KTFTGVLGGDAIARGEISLGDPVTKYWPELTGKQWQGIRMLDLATYTAGGLPLQVPDEVT
ACT-9UOY32628.1_343EBCP	KTFTGVLGGDAIARGEISLGDPVTKYWPELTGKQWQGIRMLDLATYTAGGLPLQVPDEVT
ACT-9AOP85131.1_312EBOC	KTFTGVLGGDAIARGEISLGDPVTKYWPELTGKQWQGIRMLDLATYTAGGLPLQVPDEVT
ACT-9UOY32628.1_343EBOC	KTFTGVLGGDAIARGEISLGDPVTKYWPELTGKQWQGIRMLDLATYTAGGLPLQVPDEVT
ACT-9AMZ75637.1_173EOC	KTFTGVLGGDAIARGEISLGDPVTKYWPELTGKQWQGIRMLDLATYTAGGLPLQVPDEVT
ACT-6BBT43181.1_3396]EBCP	KTFTGVLGGDAIARGEISLGDPVTKYWPELTGKQWQGIRMLDLATYTAGGLPLQVPDEVT
ACT-6BBT43181.1_3396]EBOC	KTFTGVLGGDAIARGEISLGDPVTKYWPELTGKQWQGIRMLDLATYTAGGLPLQVPDEVT
ACT-46QUP91302.1_387EBCP	KTFTGVLGGDAIARGEISLDDPVTRYWPQLTGKQWQGIRMLDLATYTAGGLPLQVPDEVT
ACT-46QUP91302.1_387EBOC	KTFTGVLGGDAIARGEISLDDPVTRYWPQLTGKQWQGIRMLDLATYTAGGLPLQVPDEVT
ACT-67QLP16520.1_400EBCP	KTFTGVLGGDAIARGEISLDDPVTRYWPQLTGKQWQGIRMLDLATYTAGGLPLQVPDEVT
ACT-67QLP16520.1_400EBOC	KTFTGVLGGDAIARGEISLDDPVTRYWPQLTGKQWQGIRMLDLATYTAGGLPLQVPDEVT
ACT-84QOR49295.1_413EBCP	KTFTGVLGGDAIARGEISLDDPVTRYWPELTGKQWQGIRMLDLATYTAGGLPLQVPDEVT
ACT-84QOR49295.1_413EBOC	KTFTGVLGGDAIARGEISLDDPVTRYWPELTGKQWQGIRMLDLATYTAGGLPLQVPDEVT
ACT-74QOX68946.1_496EBCP	KTFTGVLGGDAIARGEISLDDPVTRYWPQLTGKQWQGIRMLDLATYTAGGLPLQVPDEVT
ACT-74UIZ04332.1_460EBCP	KTFTGVLGGDAIARGEISLDDPVTRYWPQLTGKQWQGIRMLDLATYTAGGLPLQVPDEVT
ACT-74QOX68946.1_496EBOC	KTFTGVLGGDAIARGEISLDDPVTRYWPQLTGKQWQGIRMLDLATYTAGGLPLQVPDEVT
ACT-74UIZ04332.1_460EBOC	KTFTGVLGGDAIARGEISLDDPVTRYWPQLTGKQWQGIRMLDLATYTAGGLPLQVPDEVT
ACT-74QQT40108.1_4574EOC	KTFTGVLGGDAIARGEISLDDPVTRYWPQLTGKQWQGIRMLDLATYTAGGLPLQVPDEVT
ACT-16ATW90451.1_311EBCP	KTFTGVLGGDAIARGEISLDDPVTRYWPQLTGKQWQGIRMLDLATYTAGGLPLQVPDEVT
ACT-16AVO82105.1_1257EBCP	KTFTGVLGGDAIARGEISLDDPVTRYWPQLTGKQWQGIRMLDLATYTAGGLPLQVPDEVT
ACT-16AWQ41825.1_393EBCP	KTFTGVLGGDAIARGEISLDDPVTRYWPQLTGKQWQGIRMLDLATYTAGGLPLQVPDEVT
ACT-16AWQ56038.1_393EBCP	KTFTGVLGGDAIARGEISLDDPVTRYWPQLTGKQWQGIRMLDLATYTAGGLPLQVPDEVT
ACT-16QFH53420.1_474EBCP	KTFTGVLGGDAIARGEISLDDPVTRYWPQLTGKQWQGIRMLDLATYTAGGLPLQVPDEVT
ACT-16QPO90952.1_396EBCP	KTFTGVLGGDAIARGEISLDDPVTRYWPQLTGKQWQGIRMLDLATYTAGGLPLQVPDEVT
ACT-16QQA67679.1_472EBCP	KTFTGVLGGDAIARGEISLDDPVTRYWPQLTGKQWQGIRMLDLATYTAGGLPLQVPDEVT
ACT-16UDV14809.1_2173EBCP	KTFTGVLGGDAIARGEISLDDPVTRYWPQLTGKQWQGIRMLDLATYTAGGLPLQVPDEVT
ACT-16UDV41468.1_3105EBCP	KTFTGVLGGDAIARGEISLDDPVTRYWPQLTGKQWQGIRMLDLATYTAGGLPLQVPDEVT
ACT-16UIZ08994.1_395EBCP	KTFTGVLGGDAIARGEISLDDPVTRYWPQLTGKQWQGIRMLDLATYTAGGLPLQVPDEVT
ACT-16WJJ28417.1_386EBCP	KTFTGVLGGDAIARGEISLDDPVTRYWPQLTGKQWQGIRMLDLATYTAGGLPLQVPDEVT
ACT-16ATW90451.1_311EBOC	KTFTGVLGGDAIARGEISLDDPVTRYWPQLTGKQWQGIRMLDLATYTAGGLPLQVPDEVT
ACT-16AVO82105.1_1257EBOC	KTFTGVLGGDAIARGEISLDDPVTRYWPQLTGKQWQGIRMLDLATYTAGGLPLQVPDEVT
ACT-16AWQ41825.1_393EBOC	KTFTGVLGGDAIARGEISLDDPVTRYWPQLTGKQWQGIRMLDLATYTAGGLPLQVPDEVT
ACT-16AWQ56038.1_393EBOC	KTFTGVLGGDAIARGEISLDDPVTRYWPQLTGKQWQGIRMLDLATYTAGGLPLQVPDEVT
ACT-16QFH53420.1_474EBOC	KTFTGVLGGDAIARGEISLDDPVTRYWPQLTGKQWQGIRMLDLATYTAGGLPLQVPDEVT
ACT-16QPO90952.1_396EBOC	KTFTGVLGGDAIARGEISLDDPVTRYWPQLTGKQWQGIRMLDLATYTAGGLPLQVPDEVT
ACT-16QQA67679.1_472EBOC	KTFTGVLGGDAIARGEISLDDPVTRYWPQLTGKQWQGIRMLDLATYTAGGLPLQVPDEVT
ACT-16UDV14809.1_2173EBOC	KTFTGVLGGDAIARGEISLDDPVTRYWPQLTGKQWQGIRMLDLATYTAGGLPLQVPDEVT
ACT-16UDV41468.1_3105EBOC	KTFTGVLGGDAIARGEISLDDPVTRYWPQLTGKQWQGIRMLDLATYTAGGLPLQVPDEVT
ACT-16UIZ08994.1_395EBOC	KTFTGVLGGDAIARGEISLDDPVTRYWPQLTGKQWQGIRMLDLATYTAGGLPLQVPDEVT
ACT-16WJJ28417.1_386EBOC	KTFTGVLGGDAIARGEISLDDPVTRYWPQLTGKQWQGIRMLDLATYTAGGLPLQVPDEVT

```
ACT-23QLP51667.1_388EBCP      KTFTGVLGGDAIARGEISLDDPVTRYWPQLTGKQWQGIRMLDLATYTAGGLPLQVPDEVT
ACT-23QLP61663.1_388EBCP      KTFTGVLGGDAIARGEISLDDPVTRYWPQLTGKQWQGIRMLDLATYTAGGLPLQVPDEVT
ACT-23QLP66427.1_430EBCP      KTFTGVLGGDAIARGEISLDDPVTRYWPQLTGKQWQGIRMLDLATYTAGGLPLQVPDEVT
ACT-23QLU80799.1_388EBCP      KTFTGVLGGDAIARGEISLDDPVTRYWPQLTGKQWQGIRMLDLATYTAGGLPLQVPDEVT
ACT-23QLT97825.1_367EBCP      KTFTGVLGGDAIARGEISLDDPVTRYWPQLTGKQWQGIRMLDLATYTAGGLPLQVPDEVT
ACT-23QLU89187.1_4307EBCP     KTFTGVLGGDAIARGEISLDDPVTRYWPQLTGKQWQGIRMLDLATYTAGGLPLQVPDEVT
ACT-23QLV76440.1_389EBCP      KTFTGVLGGDAIARGEISLDDPVTRYWPQLTGKQWQGIRMLDLATYTAGGLPLQVPDEVT
ACT-23QLT57991.1_396EBCP      KTFTGVLGGDAIARGEISLDDPVTRYWPQLTGKQWQGIRMLDLATYTAGGLPLQVPDEVT
ACT-23QLV44058.1_369EBCP      KTFTGVLGGDAIARGEISLDDPVTRYWPQLTGKQWQGIRMLDLATYTAGGLPLQVPDEVT
ACT-23QLP51667.1_388EBOC      KTFTGVLGGDAIARGEISLDDPVTRYWPQLTGKQWQGIRMLDLATYTAGGLPLQVPDEVT
ACT-23QLP61663.1_388EBOC      KTFTGVLGGDAIARGEISLDDPVTRYWPQLTGKQWQGIRMLDLATYTAGGLPLQVPDEVT
ACT-23QLP66427.1_430EBOC      KTFTGVLGGDAIARGEISLDDPVTRYWPQLTGKQWQGIRMLDLATYTAGGLPLQVPDEVT
ACT-23QLU80799.1_388EBOC      KTFTGVLGGDAIARGEISLDDPVTRYWPQLTGKQWQGIRMLDLATYTAGGLPLQVPDEVT
ACT-23QLT97825.1_367EBOC      KTFTGVLGGDAIARGEISLDDPVTRYWPQLTGKQWQGIRMLDLATYTAGGLPLQVPDEVT
ACT-23QLU89187.1_4307EBOC     KTFTGVLGGDAIARGEISLDDPVTRYWPQLTGKQWQGIRMLDLATYTAGGLPLQVPDEVT
ACT-23QLV76440.1_389EBOC      KTFTGVLGGDAIARGEISLDDPVTRYWPQLTGKQWQGIRMLDLATYTAGGLPLQVPDEVT
ACT-23QLT57991.1_396EBOC      KTFTGVLGGDAIARGEISLDDPVTRYWPQLTGKQWQGIRMLDLATYTAGGLPLQVPDEVT
ACT-23QLV44058.1_369EBOC      KTFTGVLGGDAIARGEISLDDPVTRYWPQLTGKQWQGIRMLDLATYTAGGLPLQVPDEVT
ACT-115AXQ35263.1_2972EBCP    KTFTGVLGGDAIARGEISLDDPVTRYWPQLTGKQWQGIRMLDLATYTAGGLPLQVPDEVT
ACT-115QGR11187.1_384EBCP     KTFTGVLGGDAIARGEISLDDPVTRYWPQLTGKQWQGIRMLDLATYTAGGLPLQVPDEVT
ACT-115QIF26741.1_2106EBCP    KTFTGVLGGDAIARGEISLDDPVTRYWPQLTGKQWQGIRMLDLATYTAGGLPLQVPDEVT
ACT-115QIF32246.1_3030EBCP    KTFTGVLGGDAIARGEISLDDPVTRYWPQLTGKQWQGIRMLDLATYTAGGLPLQVPDEVT
ACT-115QOD35429.1_385EBCP     KTFTGVLGGDAIARGEISLDDPVTRYWPQLTGKQWQGIRMLDLATYTAGGLPLQVPDEVT
ACT-115QPD66621.1_385EBCP     KTFTGVLGGDAIARGEISLDDPVTRYWPQLTGKQWQGIRMLDLATYTAGGLPLQVPDEVT
ACT-115QSS35407.1_385EBCP     KTFTGVLGGDAIARGEISLDDPVTRYWPQLTGKQWQGIRMLDLATYTAGGLPLQVPDEVT
ACT-115URE88756.1_385EBCP     KTFTGVLGGDAIARGEISLDDPVTRYWPQLTGKQWQGIRMLDLATYTAGGLPLQVPDEVT
ACT-115USM81982.1_384EBCP     KTFTGVLGGDAIARGEISLDDPVTRYWPQLTGKQWQGIRMLDLATYTAGGLPLQVPDEVT
ACT-115AXQ35263.1_2972EBOC    KTFTGVLGGDAIARGEISLDDPVTRYWPQLTGKQWQGIRMLDLATYTAGGLPLQVPDEVT
ACT-115QGR11187.1_384EBOC     KTFTGVLGGDAIARGEISLDDPVTRYWPQLTGKQWQGIRMLDLATYTAGGLPLQVPDEVT
ACT-115QIF26741.1_2106EBOC    KTFTGVLGGDAIARGEISLDDPVTRYWPQLTGKQWQGIRMLDLATYTAGGLPLQVPDEVT
ACT-115QIF32246.1_3030EBOC    KTFTGVLGGDAIARGEISLDDPVTRYWPQLTGKQWQGIRMLDLATYTAGGLPLQVPDEVT
ACT-115QOD35429.1_385EBOC     KTFTGVLGGDAIARGEISLDDPVTRYWPQLTGKQWQGIRMLDLATYTAGGLPLQVPDEVT
ACT-115QPD66621.1_385EBOC     KTFTGVLGGDAIARGEISLDDPVTRYWPQLTGKQWQGIRMLDLATYTAGGLPLQVPDEVT
ACT-115QSS35407.1_385EBOC     KTFTGVLGGDAIARGEISLDDPVTRYWPQLTGKQWQGIRMLDLATYTAGGLPLQVPDEVT
ACT-115URE88756.1_385EBOC     KTFTGVLGGDAIARGEISLDDPVTRYWPQLTGKQWQGIRMLDLATYTAGGLPLQVPDEVT
ACT-115USM81982.1_384EBOC     KTFTGVLGGDAIARGEISLDDPVTRYWPQLTGKQWQGIRMLDLATYTAGGLPLQVPDEVT
ACT-25AVU48988.1_427EBCP      KTFTGVLGGDAIARGEISLDDPVTRYWPQLTGKQWQGIRMLDLATYTAGGLPLQVPDEVT
ACT-25UQQ46893.1_379EBCP      KTFTGVLGGDAIARGEISLDDPVTRYWPQLTGKQWQGIRMLDLATYTAGGLPLQVPDEVT
ACT-25WDO17280.1_310EBCP      KTFTGVLGGDAIARGEISLDDPVTRYWPQLTGKQWQGIRMLDLATYTAGGLPLQVPDEVT
ACT-25AVU48988.1_427EBOC      KTFTGVLGGDAIARGEISLDDPVTRYWPQLTGKQWQGIRMLDLATYTAGGLPLQVPDEVT
ACT-25UQQ46893.1_379EBOC      KTFTGVLGGDAIARGEISLDDPVTRYWPQLTGKQWQGIRMLDLATYTAGGLPLQVPDEVT
ACT-25WDO17280.1_310EBOC      KTFTGVLGGDAIARGEISLDDPVTRYWPQLTGKQWQGIRMLDLATYTAGGLPLQVPDEVT
ACT-25AOP89724.1_377EOC       KTFTGVLGGDAIARGEISLDDPVTRYWPQLTGKQWQGIRMLDLATYTAGGLPLQVPDEVT
ACT-45APR43249.1_2902EBCP     KTFTGVLGGDAIARGEISLDDPVTRYWPQLTGKQWQGIRMLDLATYTAGGLPLQVPDEVT
ACT-45ASA05885.1_3853EBCP     KTFTGVLGGDAIARGEISLDDPVTRYWPQLTGKQWQGIRMLDLATYTAGGLPLQVPDEVT
ACT-45ASB85359.1_3592EBCP     KTFTGVLGGDAIARGEISLDDPVTRYWPQLTGKQWQGIRMLDLATYTAGGLPLQVPDEVT
ACT-45AWR70714.1_4154EBCP     KTFTGVLGGDAIARGEISLDDPVTRYWPQLTGKQWQGIRMLDLATYTAGGLPLQVPDEVT
ACT-45AXM01511.1_4138EBCP     KTFTGVLGGDAIARGEISLDDPVTRYWPQLTGKQWQGIRMLDLATYTAGGLPLQVPDEVT
ACT-45AYU96034.1_2649EBCP     KTFTGVLGGDAIARGEISLDDPVTRYWPQLTGKQWQGIRMLDLATYTAGGLPLQVPDEVT
ACT-45QBH65043.1_3960EBCP     KTFTGVLGGDAIARGEISLDDPVTRYWPQLTGKQWQGIRMLDLATYTAGGLPLQVPDEVT
ACT-45QEL31100.1_1855EBCP     KTFTGVLGGDAIARGEISLDDPVTRYWPQLTGKQWQGIRMLDLATYTAGGLPLQVPDEVT
ACT-45ULQ22907.1_427EBCP      KTFTGVLGGDAIARGEISLDDPVTRYWPQLTGKQWQGIRMLDLATYTAGGLPLQVPDEVT
ACT-45ULQ25748.1_3478EBCP     KTFTGVLGGDAIARGEISLDDPVTRYWPQLTGKQWQGIRMLDLATYTAGGLPLQVPDEVT
ACT-45WCO72956.1_42EBCP       KTFTGVLGGDAIARGEISLDDPVTRYWPQLTGKQWQGIRMLDLATYTAGGLPLQVPDEVT
ACT-45APR43249.1_2902EBOC     KTFTGVLGGDAIARGEISLDDPVTRYWPQLTGKQWQGIRMLDLATYTAGGLPLQVPDEVT
ACT-45ASA05885.1_3853EBOC     KTFTGVLGGDAIARGEISLDDPVTRYWPQLTGKQWQGIRMLDLATYTAGGLPLQVPDEVT
ACT-45ASB85359.1_3592EBOC     KTFTGVLGGDAIARGEISLDDPVTRYWPQLTGKQWQGIRMLDLATYTAGGLPLQVPDEVT
ACT-45AWR70714.1_4154EBOC     KTFTGVLGGDAIARGEISLDDPVTRYWPQLTGKQWQGIRMLDLATYTAGGLPLQVPDEVT
ACT-45AXM01511.1_4138EBOC     KTFTGVLGGDAIARGEISLDDPVTRYWPQLTGKQWQGIRMLDLATYTAGGLPLQVPDEVT
ACT-45AYU96034.1_2649EBOC     KTFTGVLGGDAIARGEISLDDPVTRYWPQLTGKQWQGIRMLDLATYTAGGLPLQVPDEVT
ACT-45QBH65043.1_3960EBOC     KTFTGVLGGDAIARGEISLDDPVTRYWPQLTGKQWQGIRMLDLATYTAGGLPLQVPDEVT
ACT-45QEL31100.1_1855EBOC     KTFTGVLGGDAIARGEISLDDPVTRYWPQLTGKQWQGIRMLDLATYTAGGLPLQVPDEVT
ACT-45ULQ22907.1_427EBOC      KTFTGVLGGDAIARGEISLDDPVTRYWPQLTGKQWQGIRMLDLATYTAGGLPLQVPDEVT
ACT-45ULQ25748.1_3478EBOC     KTFTGVLGGDAIARGEISLDDPVTRYWPQLTGKQWQGIRMLDLATYTAGGLPLQVPDEVT
ACT-45WCO72956.1_42EBOC       KTFTGVLGGDAIARGEISLDDPVTRYWPQLTGKQWQGIRMLDLATYTAGGLPLQVPDEVT
ACT-C197QLA02472.1_2022EOC    KTFTGVLGGDAIARGEISLDDPVTRYWPQLTGKQWQGIRMLDLATYTAGGLPLQVPDEVT
ACT-64QMT06026.1_4255EBCP     KTFTGVLGGDAIARGEISLGDPVTKYWPELTGKQWQGIRMLDLATYTAGGLPLQVPDEVT
ACT-64QMT06026.1_4255EBOC     KTFTGVLGGDAIARGEISLGDPVTKYWPELTGKQWQGIRMLDLATYTAGGLPLQVPDEVT
ACT-C198QXA51435.1_2071EOC    KTFTGVLGGDAIARGEISLNDPVTRYWPELTGKQWQGIRLLDLATYTAGGLPLQVPDNVT
                         acc
```

ACT-17BBW34093.1_49417]EBCP α4 β5 α5 η4 α6

TT

190 200 210 220 230 240

ACT-17BBW34093.1_49417]EBCP	DNASLLRFYQNWQPQWKPGTTRLYANASIGLFGALAVKPSGMPYEQAMTTRVLKPLKLDH
ACT-17BBW34093.1_49417]EBOC	DNASLLRFYQNWQPQWKPGTTRLYANASIGLFGALAVKPSGMPYEQAMTTRVLKPLKLDH
7TI1_1|Chain	DNASLLRFYQNWQPQWKPGTTRLYANASIGLFGALAVKPSGMPYEQAMTTRVLKPLKLDH
ACT-37QPO56206.1_411EBCP	DNASLLRFYQHWQPQWKPGTTRLYANASIGLFGALAVKPSGMRYEQAMTKRVFKPLRLNH
ACT-37QPO65286.1_410EBCP	DNASLLRFYQHWQPQWKPGTTRLYANASIGLFGALAVKPSGMRYEQAMTKRVFKPLRLNH
ACT-37UKK25917.1_410EBCP	DNASLLRFYQHWQPQWKPGTTRLYANASIGLFGALAVKPSGMRYEQAMTKRVFKPLRLNH
ACT-37UKK30553.1_410EBCP	DNASLLRFYQHWQPQWKPGTTRLYANASIGLFGALAVKPSGMRYEQAMTKRVFKPLRLNH
ACT-37QPO56206.1_411EBOC	DNASLLRFYQHWQPQWKPGTTRLYANASIGLFGALAVKPSGMRYEQAMTKRVFKPLRLNH
ACT-37QPO65286.1_410EBOC	DNASLLRFYQHWQPQWKPGTTRLYANASIGLFGALAVKPSGMRYEQAMTKRVFKPLRLNH
ACT-37UKK25917.1_410EBOC	DNASLLRFYQHWQPQWKPGTTRLYANASIGLFGALAVKPSGMRYEQAMTKRVFKPLRLNH
ACT-37UKK30553.1_410EBOC	DNASLLRFYQHWQPQWKPGTTRLYANASIGLFGALAVKPSGMRYEQAMTKRVFKPLRLNH
ACT-65AXO42196.1_4001EBCP	DNASLLRFYQNWQPQWKPGTTRLYANASIGLFGALAVKPSGMPYEQAMTTRVLKPLKLDH
ACT-65AXO42196.1_4001EBOC	DNASLLRFYQNWQPQWKPGTTRLYANASIGLFGALAVKPSGMPYEQAMTTRVLKPLKLDH
ACT-65WBV31824.1_394EOC	DNASLLRFYQNWQPQWKPGTTRLYANASIGLFGALAVKPSGMPYEQAMTTRVLKPLKLDH
ACT-17ARZ78489.1_1874EBCP	DNASLLRFYQNWQPQWKPGTTRLYANASIGLFGALAVKPSGMPYEQAMTTRVLKPLKLDH
ACT-17ASQ75348.1_386EBCP	DNASLLRFYQNWQPQWKPGTTRLYANASIGLFGALAVKPSGMPYEQAMTTRVLKPLKLDH
ACT-17AWV77932.1_4320EBCP	DNASLLRFYQNWQPQWKPGTTRLYANASIGLFGALAVKPSGMPYEQAMTTRVLKPLKLDH
ACT-17QEQ46013.1_2619EBCP	DNASLLRFYQNWQPQWKPGTTRLYANASIGLFGALAVKPSGMPYEQAMTTRVLKPLKLDH
ACT-17QFI20602.1_494EBCP	DNASLLRFYQNWQPQWKPGTTRLYANASIGLFGALAVKPSGMPYEQAMTTRVLKPLKLDH
ACT-17QIE65424.1_3050EBCP	DNASLLRFYQNWQPQWKPGTTRLYANASIGLFGALAVKPSGMPYEQAMTTRVLKPLKLDH
ACT-17QPD71520.1_416EBCP	DNASLLRFYQNWQPQWKPGTTRLYANASIGLFGALAVKPSGMPYEQAMTTRVLKPLKLDH
ACT-17QPO45831.1_587EBCP	DNASLLRFYQNWQPQWKPGTTRLYANASIGLFGALAVKPSGMPYEQAMTTRVLKPLKLDH
ACT-17QPO51098.1_586EBCP	DNASLLRFYQNWQPQWKPGTTRLYANASIGLFGALAVKPSGMPYEQAMTTRVLKPLKLDH
ACT-17QPO69921.1_405EBCP	DNASLLRFYQNWQPQWKPGTTRLYANASIGLFGALAVKPSGMPYEQAMTTRVLKPLKLDH
ACT-17QPO74759.1_405EBCP	DNASLLRFYQNWQPQWKPGTTRLYANASIGLFGALAVKPSGMPYEQAMTTRVLKPLKLDH
ACT-17QPO85066.1_405EBCP	DNASLLRFYQNWQPQWKPGTTRLYANASIGLFGALAVKPSGMPYEQAMTTRVLKPLKLDH
ACT-17QSB73424.1_1103EBCP	DNASLLRFYQNWQPQWKPGTTRLYANASIGLFGALAVKPSGMPYEQAMTTRVLKPLKLDH
ACT-17QXY98988.1_1068EBCP	DNASLLRFYQNWQPQWKPGTTRLYANASIGLFGALAVKPSGMPYEQAMTTRVLKPLKLDH
ACT-17UDV73425.1_430EBCP	DNASLLRFYQNWQPQWKPGTTRLYANASIGLFGALAVKPSGMPYEQAMTTRVLKPLKLDH
ACT-17UDV69018.1_894EBCP	DNASLLRFYQNWQPQWKPGTTRLYANASIGLFGALAVKPSGMPYEQAMTTRVLKPLKLDH
ACT-17UKK21116.1_588EBCP	DNASLLRFYQNWQPQWKPGTTRLYANASIGLFGALAVKPSGMPYEQAMTTRVLKPLKLDH
ACT-17UOY44701.1_2566EBCP	DNASLLRFYQNWQPQWKPGTTRLYANASIGLFGALAVKPSGMPYEQAMTTRVLKPLKLDH
ACT-17URL69109.1_2030EBCP	DNASLLRFYQNWQPQWKPGTTRLYANASIGLFGALAVKPSGMPYEQAMTTRVLKPLKLDH
ACT-17UUQ70490.1_426EBCP	DNASLLRFYQNWQPQWKPGTTRLYANASIGLFGALAVKPSGMPYEQAMTTRVLKPLKLDH
ACT-17UWA70738.1_427EBCP	DNASLLRFYQNWQPQWKPGTTRLYANASIGLFGALAVKPSGMPYEQAMTTRVLKPLKLDH
ACT-17UWC98141.1_545EBCP	DNASLLRFYQNWQPQWKPGTTRLYANASIGLFGALAVKPSGMPYEQAMTTRVLKPLKLDH
ACT-17UXC72711.1_448EBCP	DNASLLRFYQNWQPQWKPGTTRLYANASIGLFGALAVKPSGMPYEQAMTTRVLKPLKLDH
ACT-17UXC77377.1_448EBCP	DNASLLRFYQNWQPQWKPGTTRLYANASIGLFGALAVKPSGMPYEQAMTTRVLKPLKLDH
ACT-17UXC82023.1_448EBCP	DNASLLRFYQNWQPQWKPGTTRLYANASIGLFGALAVKPSGMPYEQAMTTRVLKPLKLDH
ACT-17UZE41799.1_406EBCP	DNASLLRFYQNWQPQWKPGTTRLYANASIGLFGALAVKPSGMPYEQAMTTRVLKPLKLDH
ACT-17UZH49391.1_396EBCP	DNASLLRFYQNWQPQWKPGTTRLYANASIGLFGALAVKPSGMPYEQAMTTRVLKPLKLDH
ACT-17WAZ63377.1_405EBCP	DNASLLRFYQNWQPQWKPGTTRLYANASIGLFGALAVKPSGMPYEQAMTTRVLKPLKLDH
ACT-17WDU86092.1_3769EBCP	DNASLLRFYQNWQPQWKPGTTRLYANASIGLFGALAVKPSGMPYEQAMTTRVLKPLKLDH
ACT-17WDW12975.1_3764EBCP	DNASLLRFYQNWQPQWKPGTTRLYANASIGLFGALAVKPSGMPYEQAMTTRVLKPLKLDH
ACT-17WGA71117.1_396EBCP	DNASLLRFYQNWQPQWKPGTTRLYANASIGLFGALAVKPSGMPYEQAMTTRVLKPLKLDH
ACT-17WGA75603.1_396EBCP	DNASLLRFYQNWQPQWKPGTTRLYANASIGLFGALAVKPSGMPYEQAMTTRVLKPLKLDH
ACT-17WGZ57121.1_2667EBCP	DNASLLRFYQNWQPQWKPGTTRLYANASIGLFGALAVKPSGMPYEQAMTTRVLKPLKLDH
ACT-17ARZ78489.1_1874EBOC	DNASLLRFYQNWQPQWKPGTTRLYANASIGLFGALAVKPSGMPYEQAMTTRVLKPLKLDH
ACT-17ASQ75348.1_386EBOC	DNASLLRFYQNWQPQWKPGTTRLYANASIGLFGALAVKPSGMPYEQAMTTRVLKPLKLDH
ACT-17AWV77932.1_4320EBOC	DNASLLRFYQNWQPQWKPGTTRLYANASIGLFGALAVKPSGMPYEQAMTTRVLKPLKLDH
ACT-17QEQ46013.1_2619EBOC	DNASLLRFYQNWQPQWKPGTTRLYANASIGLFGALAVKPSGMPYEQAMTTRVLKPLKLDH
ACT-17QFI20602.1_494EBOC	DNASLLRFYQNWQPQWKPGTTRLYANASIGLFGALAVKPSGMPYEQAMTTRVLKPLKLDH
ACT-17QIE65424.1_3050EBOC	DNASLLRFYQNWQPQWKPGTTRLYANASIGLFGALAVKPSGMPYEQAMTTRVLKPLKLDH
ACT-17QPD71520.1_416EBOC	DNASLLRFYQNWQPQWKPGTTRLYANASIGLFGALAVKPSGMPYEQAMTTRVLKPLKLDH
ACT-17QPO45831.1_587EBOC	DNASLLRFYQNWQPQWKPGTTRLYANASIGLFGALAVKPSGMPYEQAMTTRVLKPLKLDH
ACT-17QPO51098.1_586EBOC	DNASLLRFYQNWQPQWKPGTTRLYANASIGLFGALAVKPSGMPYEQAMTTRVLKPLKLDH
ACT-17QPO69921.1_405EBOC	DNASLLRFYQNWQPQWKPGTTRLYANASIGLFGALAVKPSGMPYEQAMTTRVLKPLKLDH
ACT-17QPO74759.1_405EBOC	DNASLLRFYQNWQPQWKPGTTRLYANASIGLFGALAVKPSGMPYEQAMTTRVLKPLKLDH
ACT-17QPO85066.1_405EBOC	DNASLLRFYQNWQPQWKPGTTRLYANASIGLFGALAVKPSGMPYEQAMTTRVLKPLKLDH
ACT-17QSB73424.1_1103EBOC	DNASLLRFYQNWQPQWKPGTTRLYANASIGLFGALAVKPSGMPYEQAMTTRVLKPLKLDH
ACT-17QXY98988.1_1068EBOC	DNASLLRFYQNWQPQWKPGTTRLYANASIGLFGALAVKPSGMPYEQAMTTRVLKPLKLDH
ACT-17UDV73425.1_430EBOC	DNASLLRFYQNWQPQWKPGTTRLYANASIGLFGALAVKPSGMPYEQAMTTRVLKPLKLDH
ACT-17UDV69018.1_894EBOC	DNASLLRFYQNWQPQWKPGTTRLYANASIGLFGALAVKPSGMPYEQAMTTRVLKPLKLDH
ACT-17UKK21116.1_588EBOC	DNASLLRFYQNWQPQWKPGTTRLYANASIGLFGALAVKPSGMPYEQAMTTRVLKPLKLDH
ACT-17UOY44701.1_2566EBOC	DNASLLRFYQNWQPQWKPGTTRLYANASIGLFGALAVKPSGMPYEQAMTTRVLKPLKLDH
ACT-17URL69109.1_2030EBOC	DNASLLRFYQNWQPQWKPGTTRLYANASIGLFGALAVKPSGMPYEQAMTTRVLKPLKLDH
ACT-17UUQ70490.1_426EBOC	DNASLLRFYQNWQPQWKPGTTRLYANASIGLFGALAVKPSGMPYEQAMTTRVLKPLKLDH
ACT-17UWA70738.1_427EBOC	DNASLLRFYQNWQPQWKPGTTRLYANASIGLFGALAVKPSGMPYEQAMTTRVLKPLKLDH
ACT-17UWC98141.1_545EBOC	DNASLLRFYQNWQPQWKPGTTRLYANASIGLFGALAVKPSGMPYEQAMTTRVLKPLKLDH
ACT-17UXC72711.1_448EBOC	DNASLLRFYQNWQPQWKPGTTRLYANASIGLFGALAVKPSGMPYEQAMTTRVLKPLKLDH
ACT-17UXC77377.1_448EBOC	DNASLLRFYQNWQPQWKPGTTRLYANASIGLFGALAVKPSGMPYEQAMTTRVLKPLKLDH
ACT-17UXC82023.1_448EBOC	DNASLLRFYQNWQPQWKPGTTRLYANASIGLFGALAVKPSGMPYEQAMTTRVLKPLKLDH
ACT-17UZE41799.1_406EBOC	DNASLLRFYQNWQPQWKPGTTRLYANASIGLFGALAVKPSGMPYEQAMTTRVLKPLKLDH
ACT-17UZH49391.1_396EBOC	DNASLLRFYQNWQPQWKPGTTRLYANASIGLFGALAVKPSGMPYEQAMTTRVLKPLKLDH
ACT-17WAZ63377.1_405EBOC	DNASLLRFYQNWQPQWKPGTTRLYANASIGLFGALAVKPSGMPYEQAMTTRVLKPLKLDH
ACT-17WDU86092.1_3769EBOC	DNASLLRFYQNWQPQWKPGTTRLYANASIGLFGALAVKPSGMPYEQAMTTRVLKPLKLDH
ACT-17WDW12975.1_3764EBOC	DNASLLRFYQNWQPQWKPGTTRLYANASIGLFGALAVKPSGMPYEQAMTTRVLKPLKLDH
ACT-17WGA71117.1_396EBOC	DNASLLRFYQNWQPQWKPGTTRLYANASIGLFGALAVKPSGMPYEQAMTTRVLKPLKLDH
ACT-17WGA75603.1_396EBOC	DNASLLRFYQNWQPQWKPGTTRLYANASIGLFGALAVKPSGMPYEQAMTTRVLKPLKLDH
ACT-17WGZ57121.1_2667EBOC	DNASLLRFYQNWQPQWKPGTTRLYANASIGLFGALAVKPSGMPYEQAMTTRVLKPLKLDH
ACT-17QEI60759.1_389EOC	DNASLLRFYQNWQPQWKPGTTRLYANASIGLFGALAVKPSGMPYEQAMTTRVLKPLKLDH
ACT-17QEI70010.1_389EOC	DNASLLRFYQNWQPQWKPGTTRLYANASIGLFGALAVKPSGMPYEQAMTTRVLKPLKLDH
ACT-17QEU13386.1_612EOC	DNASLLRFYQNWQPQWKPGTTRLYANASIGLFGALAVKPSGMPYEQAMTTRVLKPLKLDH
ACT-17QHI59365.1_3674EOC	DNASLLRFYQNWQPQWKPGTTRLYANASIGLFGALAVKPSGMPYEQAMTTRVLKPLKLDH
ACT-17UZQ58984.1_410EOC	DNASLLRFYQNWQPQWKPGTTRLYANASIGLFGALAVKPSGMPYEQAMTTRVLKPLKLDH
ACT-40AWS79793.1_3058EBCP	DNASLLRFYQNWQPQWKPGTTRLYANASIGLFGALAVKPSGMPYEQAMTTRVLKPLKLDH
ACT-40AXO44745.1_1607EBCP	DNASLLRFYQNWQPQWKPGTTRLYANASIGLFGALAVKPSGMPYEQAMTTRVLKPLKLDH
ACT-40UXI63958.1_1746EBCP	DNASLLRFYQNWQPQWKPGTTRLYANASIGLFGALAVKPSGMPYEQAMTTRVLKPLKLDH
ACT-40WDC52451.1_4284EBCP	DNASLLRFYQNWQPQWKPGTTRLYANASIGLFGALAVKPSGMPYEQAMTTRVLKPLKLDH
ACT-40AWS79793.1_3058EBOC	DNASLLRFYQNWQPQWKPGTTRLYANASIGLFGALAVKPSGMPYEQAMTTRVLKPLKLDH
ACT-40AXO44745.1_1607EBOC	DNASLLRFYQNWQPQWKPGTTRLYANASIGLFGALAVKPSGMPYEQAMTTRVLKPLKLDH
ACT-40UXI63958.1_1746EBOC	DNASLLRFYQNWQPQWKPGTTRLYANASIGLFGALAVKPSGMPYEQAMTTRVLKPLKLDH
ACT-40WDC52451.1_4284EBOC	DNASLLRFYQNWQPQWKPGTTRLYANASIGLFGALAVKPSGMPYEQAMTTRVLKPLKLDH
ACT-44UWA09346.1_3724EBCP	DNASLLRFYQNWQPQWKPGTTRLYANASIGLFGALAVKPSGMPYEQAMTTRVLKPLKLDH
ACT-44UWA09346.1_3724EBOC	DNASLLRFYQNWQPQWKPGTTRLYANASIGLFGALAVKPSGMPYEQAMTTRVLKPLKLDH
ACT-90QFH83806.1_450EBCP	DNAALLRFYQNWQPQWKPGTTRLYANASIGLFGALAVKPSGMGYEQAMTTRVLKPLKLDH
ACT-90QLO92194.1_500EBCP	DNAALLRFYQNWQPQWKPGTTRLYANASIGLFGALAVKPSGMGYEQAMTTRVLKPLKLDH
ACT-90QLP01381.1_466EBCP	DNAALLRFYQNWQPQWKPGTTRLYANASIGLFGALAVKPSGMGYEQAMTTRVLKPLKLDH
ACT-90QFH83806.1_450EBOC	DNAALLRFYQNWQPQWKPGTTRLYANASIGLFGALAVKPSGMGYEQAMTTRVLKPLKLDH
ACT-90QLO92194.1_500EBOC	DNAALLRFYQNWQPQWKPGTTRLYANASIGLFGALAVKPSGMGYEQAMTTRVLKPLKLDH
ACT-90QLP01381.1_466EBOC	DNAALLRFYQNWQPQWKPGTTRLYANASIGLFGALAVKPSGMGYEQAMTTRVLKPLKLDH
ACT-24ASB73507.1_1185EBCP	DNAALLRFYQNWQPQWKPGTTRLYANASIGLFGALAVKPSGMGYEQAMTTRVLKPLKLDH
ACT-24AVU18445.1_378EBCP	DNAALLRFYQNWQPQWKPGTTRLYANASIGLFGALAVKPSGMGYEQAMTTRVLKPLKLDH

```
ACT-24QHC75881.1_374EBCP       DNAALLRFYQNWQPQWKPGTTRLYANASIGLFGALAVKPSGMGYEQAMTTRVLKPLKLDH
ACT-24QLP84500.1_370EBCP       DNAALLRFYQNWQPQWKPGTTRLYANASIGLFGALAVKPSGMGYEQAMTTRVLKPLKLDH
ACT-24QSH03071.1_370EBCP       DNAALLRFYQNWQPQWKPGTTRLYANASIGLFGALAVKPSGMGYEQAMTTRVLKPLKLDH
ACT-24QSG93844.1_370EBCP       DNAALLRFYQNWQPQWKPGTTRLYANASIGLFGALAVKPSGMGYEQAMTTRVLKPLKLDH
ACT-24QSG98466.1_370EBCP       DNAALLRFYQNWQPQWKPGTTRLYANASIGLFGALAVKPSGMGYEQAMTTRVLKPLKLDH
ACT-24QSH12187.1_370EBCP       DNAALLRFYQNWQPQWKPGTTRLYANASIGLFGALAVKPSGMGYEQAMTTRVLKPLKLDH
ACT-24QSH16801.1_370EBCP       DNAALLRFYQNWQPQWKPGTTRLYANASIGLFGALAVKPSGMGYEQAMTTRVLKPLKLDH
ACT-24QSH07573.1_370EBCP       DNAALLRFYQNWQPQWKPGTTRLYANASIGLFGALAVKPSGMGYEQAMTTRVLKPLKLDH
ACT-24QSH21409.1_370EBCP       DNAALLRFYQNWQPQWKPGTTRLYANASIGLFGALAVKPSGMGYEQAMTTRVLKPLKLDH
ACT-24QSH26023.1_370EBCP       DNAALLRFYQNWQPQWKPGTTRLYANASIGLFGALAVKPSGMGYEQAMTTRVLKPLKLDH
ACT-24QSH30639.1_370EBCP       DNAALLRFYQNWQPQWKPGTTRLYANASIGLFGALAVKPSGMGYEQAMTTRVLKPLKLDH
ACT-24UIL48372.1_372EBCP       DNAALLRFYQNWQPQWKPGTTRLYANASIGLFGALAVKPSGMGYEQAMTTRVLKPLKLDH
ACT-24UIM14687.1_372EBCP       DNAALLRFYQNWQPQWKPGTTRLYANASIGLFGALAVKPSGMGYEQAMTTRVLKPLKLDH
ACT-24UXA74146.1_371EBCP       DNAALLRFYQNWQPQWKPGTTRLYANASIGLFGALAVKPSGMGYEQAMTTRVLKPLKLDH
ACT-24WDY48721.1_372EBCP       DNAALLRFYQNWQPQWKPGTTRLYANASIGLFGALAVKPSGMGYEQAMTTRVLKPLKLDH
ACT-24ASB73507.1_1185EBOC      DNAALLRFYQNWQPQWKPGTTRLYANASIGLFGALAVKPSGMGYEQAMTTRVLKPLKLDH
ACT-24AVU18445.1_378EBOC       DNAALLRFYQNWQPQWKPGTTRLYANASIGLFGALAVKPSGMGYEQAMTTRVLKPLKLDH
ACT-24QHC75881.1_374EBOC       DNAALLRFYQNWQPQWKPGTTRLYANASIGLFGALAVKPSGMGYEQAMTTRVLKPLKLDH
ACT-24QLP84500.1_370EBOC       DNAALLRFYQNWQPQWKPGTTRLYANASIGLFGALAVKPSGMGYEQAMTTRVLKPLKLDH
ACT-24QSH03071.1_370EBOC       DNAALLRFYQNWQPQWKPGTTRLYANASIGLFGALAVKPSGMGYEQAMTTRVLKPLKLDH
ACT-24QSG93844.1_370EBOC       DNAALLRFYQNWQPQWKPGTTRLYANASIGLFGALAVKPSGMGYEQAMTTRVLKPLKLDH
ACT-24QSG98466.1_370EBOC       DNAALLRFYQNWQPQWKPGTTRLYANASIGLFGALAVKPSGMGYEQAMTTRVLKPLKLDH
ACT-24QSH12187.1_370EBOC       DNAALLRFYQNWQPQWKPGTTRLYANASIGLFGALAVKPSGMGYEQAMTTRVLKPLKLDH
ACT-24QSH16801.1_370EBOC       DNAALLRFYQNWQPQWKPGTTRLYANASIGLFGALAVKPSGMGYEQAMTTRVLKPLKLDH
ACT-24QSH07573.1_370EBOC       DNAALLRFYQNWQPQWKPGTTRLYANASIGLFGALAVKPSGMGYEQAMTTRVLKPLKLDH
ACT-24QSH21409.1_370EBOC       DNAALLRFYQNWQPQWKPGTTRLYANASIGLFGALAVKPSGMGYEQAMTTRVLKPLKLDH
ACT-24QSH26023.1_370EBOC       DNAALLRFYQNWQPQWKPGTTRLYANASIGLFGALAVKPSGMGYEQAMTTRVLKPLKLDH
ACT-24QSH30639.1_370EBOC       DNAALLRFYQNWQPQWKPGTTRLYANASIGLFGALAVKPSGMGYEQAMTTRVLKPLKLDH
ACT-24UIL48372.1_372EBOC       DNAALLRFYQNWQPQWKPGTTRLYANASIGLFGALAVKPSGMGYEQAMTTRVLKPLKLDH
ACT-24UIM14687.1_372EBOC       DNAALLRFYQNWQPQWKPGTTRLYANASIGLFGALAVKPSGMGYEQAMTTRVLKPLKLDH
ACT-24UXA74146.1_371EBOC       DNAALLRFYQNWQPQWKPGTTRLYANASIGLFGALAVKPSGMGYEQAMTTRVLKPLKLDH
ACT-24WDY48721.1_372EBOC       DNAALLRFYQNWQPQWKPGTTRLYANASIGLFGALAVKPSGMGYEQAMTTRVLKPLKLDH
ACT-89AVE73204.1_2453EBCP      DNASLLRFYQNWQPQWKPGTTRLYANASIGLFGALAVKPSGMPYEQAMTTRVLKPLKLDH
ACT-89AVE73204.1_2453EBOC      DNASLLRFYQNWQPQWKPGTTRLYANASIGLFGALAVKPSGMPYEQAMTTRVLKPLKLDH
ACT-56ASO99603.1_1270EBCP      DNASLLRFYQNWQPQWKPGTTRLYANASIGLFGALAVKPSGMPYEQAMTTRVLKPLKLDH
ACT-56QDQ75241.1_392EBCP       DNASLLRFYQNWQPQWKPGTTRLYANASIGLFGALAVKPSGMPYEQAMTTRVLKPLKLDH
ACT-56QFH98918.1_395EBCP       DNASLLRFYQNWQPQWKPGTTRLYANASIGLFGALAVKPSGMPYEQAMTTRVLKPLKLDH
ACT-56QFH34334.1_395EBCP       DNASLLRFYQNWQPQWKPGTTRLYANASIGLFGALAVKPSGMPYEQAMTTRVLKPLKLDH
ACT-56QFI32707.1_2233EBCP      DNASLLRFYQNWQPQWKPGTTRLYANASIGLFGALAVKPSGMPYEQAMTTRVLKPLKLDH
ACT-56QMI65633.1_4184EBCP      DNASLLRFYQNWQPQWKPGTTRLYANASIGLFGALAVKPSGMPYEQAMTTRVLKPLKLDH
ACT-56QMI71192.1_422EBCP       DNASLLRFYQNWQPQWKPGTTRLYANASIGLFGALAVKPSGMPYEQAMTTRVLKPLKLDH
ACT-56UDV17856.1_8EBCP         DNASLLRFYQNWQPQWKPGTTRLYANASIGLFGALAVKPSGMPYEQAMTTRVLKPLKLDH
ACT-56UDV39132.1_1376EBCP      DNASLLRFYQNWQPQWKPGTTRLYANASIGLFGALAVKPSGMPYEQAMTTRVLKPLKLDH
ACT-56UWB10427.1_430EBCP       DNASLLRFYQNWQPQWKPGTTRLYANASIGLFGALAVKPSGMPYEQAMTTRVLKPLKLDH
ACT-56UWC18315.1_415EBCP       DNASLLRFYQNWQPQWKPGTTRLYANASIGLFGALAVKPSGMPYEQAMTTRVLKPLKLDH
ACT-56UWC23093.1_417EBCP       DNASLLRFYQNWQPQWKPGTTRLYANASIGLFGALAVKPSGMPYEQAMTTRVLKPLKLDH
ACT-56WBT12607.1_393EBCP       DNASLLRFYQNWQPQWKPGTTRLYANASIGLFGALAVKPSGMPYEQAMTTRVLKPLKLDH
ACT-56WBT28831.1_393EBCP       DNASLLRFYQNWQPQWKPGTTRLYANASIGLFGALAVKPSGMPYEQAMTTRVLKPLKLDH
ACT-56WGB34617.1_392EBCP       DNASLLRFYQNWQPQWKPGTTRLYANASIGLFGALAVKPSGMPYEQAMTTRVLKPLKLDH
ACT-56WGU03466.1_393EBCP       DNASLLRFYQNWQPQWKPGTTRLYANASIGLFGALAVKPSGMPYEQAMTTRVLKPLKLDH
ACT-56ASO99603.1_1270EBOC      DNASLLRFYQNWQPQWKPGTTRLYANASIGLFGALAVKPSGMPYEQAMTTRVLKPLKLDH
ACT-56QDQ75241.1_392EBOC       DNASLLRFYQNWQPQWKPGTTRLYANASIGLFGALAVKPSGMPYEQAMTTRVLKPLKLDH
ACT-56QFH98918.1_395EBOC       DNASLLRFYQNWQPQWKPGTTRLYANASIGLFGALAVKPSGMPYEQAMTTRVLKPLKLDH
ACT-56QFH34334.1_395EBOC       DNASLLRFYQNWQPQWKPGTTRLYANASIGLFGALAVKPSGMPYEQAMTTRVLKPLKLDH
ACT-56QFI32707.1_2233EBOC      DNASLLRFYQNWQPQWKPGTTRLYANASIGLFGALAVKPSGMPYEQAMTTRVLKPLKLDH
ACT-56QMI65633.1_4184EBOC      DNASLLRFYQNWQPQWKPGTTRLYANASIGLFGALAVKPSGMPYEQAMTTRVLKPLKLDH
ACT-56QMI71192.1_422EBOC       DNASLLRFYQNWQPQWKPGTTRLYANASIGLFGALAVKPSGMPYEQAMTTRVLKPLKLDH
ACT-56UDV17856.1_8EBOC         DNASLLRFYQNWQPQWKPGTTRLYANASIGLFGALAVKPSGMPYEQAMTTRVLKPLKLDH
ACT-56UDV39132.1_1376EBOC      DNASLLRFYQNWQPQWKPGTTRLYANASIGLFGALAVKPSGMPYEQAMTTRVLKPLKLDH
ACT-56UWB10427.1_430EBOC       DNASLLRFYQNWQPQWKPGTTRLYANASIGLFGALAVKPSGMPYEQAMTTRVLKPLKLDH
ACT-56UWC18315.1_415EBOC       DNASLLRFYQNWQPQWKPGTTRLYANASIGLFGALAVKPSGMPYEQAMTTRVLKPLKLDH
ACT-56UWC23093.1_417EBOC       DNASLLRFYQNWQPQWKPGTTRLYANASIGLFGALAVKPSGMPYEQAMTTRVLKPLKLDH
ACT-56WBT12607.1_393EBOC       DNASLLRFYQNWQPQWKPGTTRLYANASIGLFGALAVKPSGMPYEQAMTTRVLKPLKLDH
ACT-56WBT28831.1_393EBOC       DNASLLRFYQNWQPQWKPGTTRLYANASIGLFGALAVKPSGMPYEQAMTTRVLKPLKLDH
ACT-56WGB34617.1_392EBOC       DNASLLRFYQNWQPQWKPGTTRLYANASIGLFGALAVKPSGMPYEQAMTTRVLKPLKLDH
ACT-56WGU03466.1_393EBOC       DNASLLRFYQNWQPQWKPGTTRLYANASIGLFGALAVKPSGMPYEQAMTTRVLKPLKLDH
ACT-56AOP76464.1_392EOC        DNASLLRFYQNWQPQWKPGTTRLYANASIGLFGALAVKPSGMPYEQAMTTRVLKPLKLDH
ACT-56QXA77046.1_2672EOC       DNASLLRFYQNWQPQWKPGTTRLYANASIGLFGALAVKPSGMPYEQAMTTRVLKPLKLDH
ACT-56WFP29817.1_397EOC        DNASLLRFYQNWQPQWKPGTTRLYANASIGLFGALAVKPSGMPYEQAMTTRVLKPLKLDH
ACT-27AXO49795.1_1610EBCP      DNASLLRFYQNWQPQWKPGTTRLYANASIGLFGALAVKPSGMPYEQAMTTRVLKPLKLDH
ACT-27QHO79915.1_457EBCP       DNASLLRFYQNWQPQWKPGTTRLYANASIGLFGALAVKPSGMPYEQAMTTRVLKPLKLDH
ACT-27QHO97012.1_458EBCP       DNASLLRFYQNWQPQWKPGTTRLYANASIGLFGALAVKPSGMPYEQAMTTRVLKPLKLDH
ACT-27QQH03712.1_3802EBCP      DNASLLRFYQNWQPQWKPGTTRLYANASIGLFGALAVKPSGMPYEQAMTTRVLKPLKLDH
ACT-27QQH08784.1_3802EBCP      DNASLLRFYQNWQPQWKPGTTRLYANASIGLFGALAVKPSGMPYEQAMTTRVLKPLKLDH
ACT-27UVZ91365.1_388EBCP       DNASLLRFYQNWQPQWKPGTTRLYANASIGLFGALAVKPSGMPYEQAMTTRVLKPLKLDH
ACT-27AXO49795.1_1610EBOC      DNASLLRFYQNWQPQWKPGTTRLYANASIGLFGALAVKPSGMPYEQAMTTRVLKPLKLDH
ACT-27QHO79915.1_457EBOC       DNASLLRFYQNWQPQWKPGTTRLYANASIGLFGALAVKPSGMPYEQAMTTRVLKPLKLDH
ACT-27QHO97012.1_458EBOC       DNASLLRFYQNWQPQWKPGTTRLYANASIGLFGALAVKPSGMPYEQAMTTRVLKPLKLDH
ACT-27QQH03712.1_3802EBOC      DNASLLRFYQNWQPQWKPGTTRLYANASIGLFGALAVKPSGMPYEQAMTTRVLKPLKLDH
ACT-27QQH08784.1_3802EBOC      DNASLLRFYQNWQPQWKPGTTRLYANASIGLFGALAVKPSGMPYEQAMTTRVLKPLKLDH
ACT-27UVZ91365.1_388EBOC       DNASLLRFYQNWQPQWKPGTTRLYANASIGLFGALAVKPSGMPYEQAMTTRVLKPLKLDH
ACT-102QEO03155.1_4213EBCP     DNASLLRFYQHWQPQWKPGATRLYANASIGLFGALAVKPSGMSFEQAMTKRVFKPLKLDH
ACT-102BBV79841.1_51928]EBCP   DNASLLRFYQHWQPQWKPGATRLYANASIGLFGALAVKPSGMSFEQAMTKRVFKPLKLDH
ACT-102QZS47739.1_401EBCP      DNASLLRFYQHWQPQWKPGATRLYANASIGLFGALAVKPSGMSFEQAMTKRVFKPLKLDH
ACT-102QEO03155.1_4213EBOC     DNASLLRFYQHWQPQWKPGATRLYANASIGLFGALAVKPSGMSFEQAMTKRVFKPLKLDH
ACT-102BBV79841.1_51928]EBOC   DNASLLRFYQHWQPQWKPGATRLYANASIGLFGALAVKPSGMSFEQAMTKRVFKPLKLDH
ACT-102QZS47739.1_401EBOC      DNASLLRFYQHWQPQWKPGATRLYANASIGLFGALAVKPSGMSFEQAMTKRVFKPLKLDH
ACT-55QLP71015.1_413EBCP       DNASLLRFYQNWQPQWKPGTTRLYANASIGLFGALAVKPSGMSYEQAMTTRVLKPLKLDH
ACT-55QLP80034.1_414EBCP       DNASLLRFYQNWQPQWKPGTTRLYANASIGLFGALAVKPSGMSYEQAMTTRVLKPLKLDH
ACT-55QLS08818.1_415EBCP       DNASLLRFYQNWQPQWKPGTTRLYANASIGLFGALAVKPSGMSYEQAMTTRVLKPLKLDH
ACT-55QLT25377.1_3621EBCP      DNASLLRFYQNWQPQWKPGTTRLYANASIGLFGALAVKPSGMSYEQAMTTRVLKPLKLDH
ACT-55QLV12510.1_4006EBCP      DNASLLRFYQNWQPQWKPGTTRLYANASIGLFGALAVKPSGMSYEQAMTTRVLKPLKLDH
ACT-55QLW04180.1_4182EBCP      DNASLLRFYQNWQPQWKPGTTRLYANASIGLFGALAVKPSGMSYEQAMTTRVLKPLKLDH
ACT-55QLV08026.1_4006EBCP      DNASLLRFYQNWQPQWKPGTTRLYANASIGLFGALAVKPSGMSYEQAMTTRVLKPLKLDH
ACT-55QLV96171.1_421EBCP       DNASLLRFYQNWQPQWKPGTTRLYANASIGLFGALAVKPSGMSYEQAMTTRVLKPLKLDH
ACT-55QLW44293.1_405EBCP       DNASLLRFYQNWQPQWKPGTTRLYANASIGLFGALAVKPSGMSYEQAMTTRVLKPLKLDH
ACT-55UBH32480.1_675EBCP       DNASLLRFYQNWQPQWKPGTTRLYANASIGLFGALAVKPSGMSYEQAMTTRVLKPLKLDH
ACT-55UDV61223.1_2251EBCP      DNASLLRFYQNWQPQWKPGTTRLYANASIGLFGALAVKPSGMSYEQAMTTRVLKPLKLDH
ACT-55QLP71015.1_413EBOC       DNASLLRFYQNWQPQWKPGTTRLYANASIGLFGALAVKPSGMSYEQAMTTRVLKPLKLDH
ACT-55QLP80034.1_414EBOC       DNASLLRFYQNWQPQWKPGTTRLYANASIGLFGALAVKPSGMSYEQAMTTRVLKPLKLDH
ACT-55QLS08818.1_415EBOC       DNASLLRFYQNWQPQWKPGTTRLYANASIGLFGALAVKPSGMSYEQAMTTRVLKPLKLDH
ACT-55QLT25377.1_3621EBOC      DNASLLRFYQNWQPQWKPGTTRLYANASIGLFGALAVKPSGMSYEQAMTTRVLKPLKLDH
ACT-55QLV12510.1_4006EBOC      DNASLLRFYQNWQPQWKPGTTRLYANASIGLFGALAVKPSGMSYEQAMTTRVLKPLKLDH
```

```
ACT-55QLW04180.1_4182EBOC     DNASLLRFYQNWQPQWKPGTTRLYANASIGLFGALAVKPSGMSYEQAMTTRVLKPLKLDH
ACT-55QLV08026.1_4006EBOC     DNASLLRFYQNWQPQWKPGTTRLYANASIGLFGALAVKPSGMSYEQAMTTRVLKPLKLDH
ACT-55QLV96171.1_421EBOC      DNASLLRFYQNWQPQWKPGTTRLYANASIGLFGALAVKPSGMSYEQAMTTRVLKPLKLDH
ACT-55QLW44293.1_405EBOC      DNASLLRFYQNWQPQWKPGTTRLYANASIGLFGALAVKPSGMSYEQAMTTRVLKPLKLDH
ACT-55UBH32480.1_675EBOC      DNASLLRFYQNWQPQWKPGTTRLYANASIGLFGALAVKPSGMSYEQAMTTRVLKPLKLDH
ACT-55UDV61223.1_2251EBOC     DNASLLRFYQNWQPQWKPGTTRLYANASIGLFGALAVKPSGMSYEQAMTTRVLKPLKLDH
ACT-55AOP80857.1_409EOC       DNASLLRFYQNWQPQWKPGTTRLYANASIGLFGALAVKPSGMSYEQAMTTRVLKPLKLDH
ACT-51BBW20048.1_38351]EBCP   DNASLLRFYQHWQPQWKPGTTRLYANASIGLFGALAVKPSGMSFEQAMTKRVFKPLKLDH
ACT-51BBW20048.1_38351]EBOC   DNASLLRFYQHWQPQWKPGTTRLYANASIGLFGALAVKPSGMSFEQAMTKRVFKPLKLDH
ACT-28URE95606.1_351EBCP      DNASLLRFYQHWQPQWKPGATRLYANASIGLFGALAVKPSGMSFEQAMTKRVFKPLKLDH
ACT-28URE95606.1_351EBOC      DNASLLRFYQHWQPQWKPGATRLYANASIGLFGALAVKPSGMSFEQAMTKRVFKPLKLDH
ACT-87AYL04658.1_1249EBCP     DNASLLRFYQYWQPQWKPGATRLYANASIGLFGALAVKPSGMSFEQAMTKRVFKPLKLDH
ACT-87QIP18425.1_352EBCP      DNASLLRFYQYWQPQWKPGATRLYANASIGLFGALAVKPSGMSFEQAMTKRVFKPLKLDH
ACT-87AYL04658.1_1249EBOC     DNASLLRFYQYWQPQWKPGATRLYANASIGLFGALAVKPSGMSFEQAMTKRVFKPLKLDH
ACT-87QIP18425.1_352EBOC      DNASLLRFYQYWQPQWKPGATRLYANASIGLFGALAVKPSGMSFEQAMTKRVFKPLKLDH
ACT-75AWX01586.1_1447EBCP     DNASLLRFYQNWQPQWKPGTTRLYANASIGLFGALAVKPSGMPYEQAMTTRVLKPLKLDH
ACT-75AWX01586.1_1447EBOC     DNASLLRFYQNWQPQWKPGTTRLYANASIGLFGALAVKPSGMPYEQAMTTRVLKPLKLDH
ACT-103URL27837.1_342EOC      DNASLLRFYQHWQPQWKPGTTRLYANASIGLFGALAVKPSGMSFEQAMTKRVFKPLKLDH
ACT-99UXJ64669.1_346EBCP      DNASLLRFYQHWQPQWKPGTTRLYANASIGLFGALAVKPSGMSFEQAMTKRVFKPLKLDH
ACT-99UXJ64669.1_346EBOC      DNASLLRFYQHWQPQWKPGTTRLYANASIGLFGALAVKPSGMSFEQAMTKRVFKPLKLDH
ACT-95QIB80518.1_348EBCP      DNASLLRFYQHWQPQWKPGTTRLYANASIGLFGALAVKPSGMSFEQAMTKRVFKPLKLDH
ACT-95BBV69349.1_35128]EBCP   DNASLLRFYQHWQPQWKPGTTRLYANASIGLFGALAVKPSGMSFEQAMTKRVFKPLKLDH
ACT-95UZQ68133.1_388EBCP      DNASLLRFYQHWQPQWKPGTTRLYANASIGLFGALAVKPSGMSFEQAMTKRVFKPLKLDH
ACT-95QIB80518.1_348EBOC      DNASLLRFYQHWQPQWKPGTTRLYANASIGLFGALAVKPSGMSFEQAMTKRVFKPLKLDH
ACT-95BBV69349.1_35128]EBOC   DNASLLRFYQHWQPQWKPGTTRLYANASIGLFGALAVKPSGMSFEQAMTKRVFKPLKLDH
ACT-95UZQ68133.1_388EBOC      DNASLLRFYQHWQPQWKPGTTRLYANASIGLFGALAVKPSGMSFEQAMTKRVFKPLKLDH
ACT-52QFH88560.1_350EBCP      DNASLLRFYQHWQPQWKPGATRLYANASIGLFGALAVKPSGMSFEQAMTKRVFKPLKLDH
ACT-52BBS30224.1_43052]EBCP   DNASLLRFYQHWQPQWKPGATRLYANASIGLFGALAVKPSGMSFEQAMTKRVFKPLKLDH
ACT-52WBN86316.1_4083EBCP     DNASLLRFYQHWQPQWKPGATRLYANASIGLFGALAVKPSGMSFEQAMTKRVFKPLKLDH
ACT-52QFH88560.1_350EBOC      DNASLLRFYQHWQPQWKPGATRLYANASIGLFGALAVKPSGMSFEQAMTKRVFKPLKLDH
ACT-52BBS30224.1_43052]EBOC   DNASLLRFYQHWQPQWKPGATRLYANASIGLFGALAVKPSGMSFEQAMTKRVFKPLKLDH
ACT-52WBN86316.1_4083EBOC     DNASLLRFYQHWQPQWKPGATRLYANASIGLFGALAVKPSGMSFEQAMTKRVFKPLKLDH
ACT-4QLR27288.1_395EBCP       DNASLLRFYQNWQPQWKPGTTRLYANTSIGLFGALAVKPSGMSYEQAITTRVFKPLKLDH
ACT-4QPS68257.1_413EBCP       DNASLLRFYQNWQPQWKPGTTRLYANTSIGLFGALAVKPSGMSYEQAITTRVFKPLKLDH
ACT-4QLR27288.1_395EBOC       DNASLLRFYQNWQPQWKPGTTRLYANTSIGLFGALAVKPSGMSYEQAITTRVFKPLKLDH
ACT-4QPS68257.1_413EBOC       DNASLLRFYQNWQPQWKPGTTRLYANTSIGLFGALAVKPSGMSYEQAITTRVFKPLKLDH
ACT-3UOY53510.1_358EBCP       DNASLLRFYQNWQPQWKPGTTRLYANASIGLFGALAVKPSGMSYEQAITTRVFKPLKLDH
ACT-3UOY63785.1_4171EBCP      DNASLLRFYQNWQPQWKPGTTRLYANASIGLFGALAVKPSGMSYEQAITTRVFKPLKLDH
ACT-3UOY53510.1_358EBOC       DNASLLRFYQNWQPQWKPGTTRLYANASIGLFGALAVKPSGMSYEQAITTRVFKPLKLDH
ACT-3UOY63785.1_4171EBOC      DNASLLRFYQNWQPQWKPGTTRLYANASIGLFGALAVKPSGMSYEQAITTRVFKPLKLDH
ACT-1QLV61731.1_472EBCP       DNASLLRFYQNWQPQWKPGTTRLYANASIGLFGALAVKPSGMSYEQAITTRVFKPLKLDH
ACT-1UWA77567.1_382EBCP       DNASLLRFYQNWQPQWKPGTTRLYANASIGLFGALAVKPSGMSYEQAITTRVFKPLKLDH
ACT-1QLV61731.1_472EBOC       DNASLLRFYQNWQPQWKPGTTRLYANASIGLFGALAVKPSGMSYEQAITTRVFKPLKLDH
ACT-1UWA77567.1_382EBOC       DNASLLRFYQNWQPQWKPGTTRLYANASIGLFGALAVKPSGMSYEQAITTRVFKPLKLDH
ACT-77QWZ50133.1_330EBCP      DNASLLRFYQNWQPQWKPGTTRLYANSSIGLFGALAVKPSGMSYEQAMTTRVFKPLKLDH
ACT-77UBH39796.1_3999EBCP     DNASLLRFYQNWQPQWKPGTTRLYANSSIGLFGALAVKPSGMSYEQAMTTRVFKPLKLDH
ACT-77UBH98103.1_3842EBCP     DNASLLRFYQNWQPQWKPGTTRLYANSSIGLFGALAVKPSGMSYEQAMTTRVFKPLKLDH
ACT-77UBH94108.1_193EBCP      DNASLLRFYQNWQPQWKPGTTRLYANSSIGLFGALAVKPSGMSYEQAMTTRVFKPLKLDH
ACT-77QWZ50133.1_330EBOC      DNASLLRFYQNWQPQWKPGTTRLYANSSIGLFGALAVKPSGMSYEQAMTTRVFKPLKLDH
ACT-77UBH39796.1_3999EBOC     DNASLLRFYQNWQPQWKPGTTRLYANSSIGLFGALAVKPSGMSYEQAMTTRVFKPLKLDH
ACT-77UBH98103.1_3842EBOC     DNASLLRFYQNWQPQWKPGTTRLYANSSIGLFGALAVKPSGMSYEQAMTTRVFKPLKLDH
ACT-77UBH94108.1_193EBOC      DNASLLRFYQNWQPQWKPGTTRLYANSSIGLFGALAVKPSGMSYEQAMTTRVFKPLKLDH
ACT-53QEL38380.1_485EBCP      DNASLLRFYQNWQPQWKPGTTRLYANASIGLFGALAVQPSGMSYEQAITTRVLKPLKLDH
ACT-53QEL38380.1_485EBOC      DNASLLRFYQNWQPQWKPGTTRLYANASIGLFGALAVQPSGMSYEQAITTRVLKPLKLDH
ACT-57QBB07838.1_4703EBCP     DNASLLRFYQNWQPQWKPGTTRLYANASIGLFGALAVKPSGMSYEQAITTRVFKRLKLDH
ACT-57QYD27335.1_499EBCP      DNASLLRFYQNWQPQWKPGTTRLYANASIGLFGALAVKPSGMSYEQAITTRVFKRLKLDH
ACT-57UBM17197.1_2672EBCP     DNASLLRFYQNWQPQWKPGTTRLYANASIGLFGALAVKPSGMSYEQAITTRVFKRLKLDH
ACT-57UKB55021.1_380EBCP      DNASLLRFYQNWQPQWKPGTTRLYANASIGLFGALAVKPSGMSYEQAITTRVFKRLKLDH
ACT-57QBB07838.1_4703EBOC     DNASLLRFYQNWQPQWKPGTTRLYANASIGLFGALAVKPSGMSYEQAITTRVFKRLKLDH
ACT-57QYD27335.1_499EBOC      DNASLLRFYQNWQPQWKPGTTRLYANASIGLFGALAVKPSGMSYEQAITTRVFKRLKLDH
ACT-57UBM17197.1_2672EBOC     DNASLLRFYQNWQPQWKPGTTRLYANASIGLFGALAVKPSGMSYEQAITTRVFKRLKLDH
ACT-57UKB55021.1_380EBOC      DNASLLRFYQNWQPQWKPGTTRLYANASIGLFGALAVKPSGMSYEQAITTRVFKRLKLDH
ACT-93ALL15984.1_396EOC       DNASLLRFYQNWQPQWKPGTTRLYANASIGLFGALAVKPSGMSYEQTITTRVFKPLKLDH
ACT-9AOP85131.1_312EBCP       DNTSLLRFYQHWQPQWKPGTTRLYANASIGLFGALAVKPSGMNFEQAMTKRVFKPLKLDH
ACT-9UOY32628.1_343EBCP       DNTSLLRFYQHWQPQWKPGTTRLYANASIGLFGALAVKPSGMNFEQAMTKRVFKPLKLDH
ACT-9AOP85131.1_312EBOC       DNTSLLRFYQHWQPQWKPGTTRLYANASIGLFGALAVKPSGMNFEQAMTKRVFKPLKLDH
ACT-9UOY32628.1_343EBOC       DNTSLLRFYQHWQPQWKPGTTRLYANASIGLFGALAVKPSGMNFEQAMTKRVFKPLKLDH
ACT-9AMZ75637.1_173EOC        DNTSLLRFYQHWQPQWKPGTTRLYANASIGLFGALAVKPSGMNFEQAMTKRVFKPLKLDH
ACT-6BBT43181.1_3396]EBCP     DNASLLRFYQNWQPQWKPGTTRLYANASIGLFGALAVKPSGMSYEQAITTRVFKPLKLDH
ACT-6BBT43181.1_3396]EBOC     DNASLLRFYQNWQPQWKPGTTRLYANASIGLFGALAVKPSGMSYEQAITTRVFKPLKLDH
ACT-46QUP91302.1_387EBCP      DNASLLRFYQNWQPQWKPGTTRLYANASIGLFGALAVKPSGMPYEQAMTTRVLKPLKLDH
ACT-46QUP91302.1_387EBOC      DNASLLRFYQNWQPQWKPGTTRLYANASIGLFGALAVKPSGMPYEQAMTTRVLKPLKLDH
ACT-67QLP16520.1_400EBCP      DNAALLRFYQNWQPQWKPGTTRLYANASIGLFGALAVKPSGMGYERAMTTRVLKPLKLDH
ACT-67QLP16520.1_400EBOC      DNAALLRFYQNWQPQWKPGTTRLYANASIGLFGALAVKPSGMGYERAMTTRVLKPLKLDH
ACT-84QOR49295.1_413EBCP      DDASLLRFYQHWQPQWKPGTTRLYANASIGLFGALAVKPSGMRYEQAMTERVFKPLALHH
ACT-84QOR49295.1_413EBOC      DDASLLRFYQHWQPQWKPGTTRLYANASIGLFGALAVKPSGMRYEQAMTERVFKPLALHH
ACT-74QOX68946.1_496EBCP      DNASLLRFYQNWQPQWKPGTTRLYANASIGLFGALAVKPSGMPYEQAMTTRVLKPLKLDH
ACT-74UIZ04332.1_460EBCP      DNASLLRFYQNWQPQWKPGTTRLYANASIGLFGALAVKPSGMPYEQAMTTRVLKPLKLDH
ACT-74QOX68946.1_496EBOC      DNASLLRFYQNWQPQWKPGTTRLYANASIGLFGALAVKPSGMPYEQAMTTRVLKPLKLDH
ACT-74UIZ04332.1_460EBOC      DNASLLRFYQNWQPQWKPGTTRLYANASIGLFGALAVKPSGMPYEQAMTTRVLKPLKLDH
ACT-74QQT40108.1_4574EOC      DNASLLRFYQNWQPQWKPGTTRLYANASIGLFGALAVKPSGMPYEQAMTTRVLKPLKLDH
ACT-16ATW90451.1_311EBCP      DNASLLRFYQNWQPQWKPGTTRLYANASIGLFGALAVKPSGMPYEQAMTTRVLKPLKLDH
ACT-16AVO82105.1_1257EBCP     DNASLLRFYQNWQPQWKPGTTRLYANASIGLFGALAVKPSGMPYEQAMTTRVLKPLKLDH
ACT-16AWQ41825.1_393EBCP      DNASLLRFYQNWQPQWKPGTTRLYANASIGLFGALAVKPSGMPYEQAMTTRVLKPLKLDH
ACT-16AWQ56038.1_393EBCP      DNASLLRFYQNWQPQWKPGTTRLYANASIGLFGALAVKPSGMPYEQAMTTRVLKPLKLDH
ACT-16QFH53420.1_474EBCP      DNASLLRFYQNWQPQWKPGTTRLYANASIGLFGALAVKPSGMPYEQAMTTRVLKPLKLDH
ACT-16QPO90952.1_396EBCP      DNASLLRFYQNWQPQWKPGTTRLYANASIGLFGALAVKPSGMPYEQAMTTRVLKPLKLDH
ACT-16QQA67679.1_472EBCP      DNASLLRFYQNWQPQWKPGTTRLYANASIGLFGALAVKPSGMPYEQAMTTRVLKPLKLDH
ACT-16UDV14809.1_2173EBCP     DNASLLRFYQNWQPQWKPGTTRLYANASIGLFGALAVKPSGMPYEQAMTTRVLKPLKLDH
ACT-16UDV41468.1_3105EBCP     DNASLLRFYQNWQPQWKPGTTRLYANASIGLFGALAVKPSGMPYEQAMTTRVLKPLKLDH
ACT-16UIZ08994.1_395EBCP      DNASLLRFYQNWQPQWKPGTTRLYANASIGLFGALAVKPSGMPYEQAMTTRVLKPLKLDH
ACT-16WJJ28417.1_386EBCP      DNASLLRFYQNWQPQWKPGTTRLYANASIGLFGALAVKPSGMPYEQAMTTRVLKPLKLDH
ACT-16ATW90451.1_311EBOC      DNASLLRFYQNWQPQWKPGTTRLYANASIGLFGALAVKPSGMPYEQAMTTRVLKPLKLDH
ACT-16AVO82105.1_1257EBOC     DNASLLRFYQNWQPQWKPGTTRLYANASIGLFGALAVKPSGMPYEQAMTTRVLKPLKLDH
ACT-16AWQ41825.1_393EBOC      DNASLLRFYQNWQPQWKPGTTRLYANASIGLFGALAVKPSGMPYEQAMTTRVLKPLKLDH
ACT-16AWQ56038.1_393EBOC      DNASLLRFYQNWQPQWKPGTTRLYANASIGLFGALAVKPSGMPYEQAMTTRVLKPLKLDH
ACT-16QFH53420.1_474EBOC      DNASLLRFYQNWQPQWKPGTTRLYANASIGLFGALAVKPSGMPYEQAMTTRVLKPLKLDH
ACT-16QPO90952.1_396EBOC      DNASLLRFYQNWQPQWKPGTTRLYANASIGLFGALAVKPSGMPYEQAMTTRVLKPLKLDH
ACT-16QQA67679.1_472EBOC      DNASLLRFYQNWQPQWKPGTTRLYANASIGLFGALAVKPSGMPYEQAMTTRVLKPLKLDH
ACT-16UDV14809.1_2173EBOC     DNASLLRFYQNWQPQWKPGTTRLYANASIGLFGALAVKPSGMPYEQAMTTRVLKPLKLDH
ACT-16UDV41468.1_3105EBOC     DNASLLRFYQNWQPQWKPGTTRLYANASIGLFGALAVKPSGMPYEQAMTTRVLKPLKLDH
ACT-16UIZ08994.1_395EBOC      DNASLLRFYQNWQPQWKPGTTRLYANASIGLFGALAVKPSGMPYEQAMTTRVLKPLKLDH
ACT-16WJJ28417.1_386EBOC      DNASLLRFYQNWQPQWKPGTTRLYANASIGLFGALAVKPSGMPYEQAMTTRVLKPLKLDH
```

```
ACT-23QLP51667.1_388EBCP     DNAALLRFYQNWQPQWKPGTTRLYANASIGLFGALAVKPSGMGYEQAMTTRVLKPLKLDH
ACT-23QLP61663.1_388EBCP     DNAALLRFYQNWQPQWKPGTTRLYANASIGLFGALAVKPSGMGYEQAMTTRVLKPLKLDH
ACT-23QLP66427.1_430EBCP     DNAALLRFYQNWQPQWKPGTTRLYANASIGLFGALAVKPSGMGYEQAMTTRVLKPLKLDH
ACT-23QLU80799.1_388EBCP     DNAALLRFYQNWQPQWKPGTTRLYANASIGLFGALAVKPSGMGYEQAMTTRVLKPLKLDH
ACT-23QLT97825.1_367EBCP     DNAALLRFYQNWQPQWKPGTTRLYANASIGLFGALAVKPSGMGYEQAMTTRVLKPLKLDH
ACT-23QLU89187.1_4307EBCP    DNAALLRFYQNWQPQWKPGTTRLYANASIGLFGALAVKPSGMGYEQAMTTRVLKPLKLDH
ACT-23QLV76440.1_389EBCP     DNAALLRFYQNWQPQWKPGTTRLYANASIGLFGALAVKPSGMGYEQAMTTRVLKPLKLDH
ACT-23QLT57991.1_396EBCP     DNAALLRFYQNWQPQWKPGTTRLYANASIGLFGALAVKPSGMGYEQAMTTRVLKPLKLDH
ACT-23QLV44058.1_369EBCP     DNAALLRFYQNWQPQWKPGTTRLYANASIGLFGALAVKPSGMGYEQAMTTRVLKPLKLDH
ACT-23QLP51667.1_388EBOC     DNAALLRFYQNWQPQWKPGTTRLYANASIGLFGALAVKPSGMGYEQAMTTRVLKPLKLDH
ACT-23QLP61663.1_388EBOC     DNAALLRFYQNWQPQWKPGTTRLYANASIGLFGALAVKPSGMGYEQAMTTRVLKPLKLDH
ACT-23QLP66427.1_430EBOC     DNAALLRFYQNWQPQWKPGTTRLYANASIGLFGALAVKPSGMGYEQAMTTRVLKPLKLDH
ACT-23QLU80799.1_388EBOC     DNAALLRFYQNWQPQWKPGTTRLYANASIGLFGALAVKPSGMGYEQAMTTRVLKPLKLDH
ACT-23QLT97825.1_367EBOC     DNAALLRFYQNWQPQWKPGTTRLYANASIGLFGALAVKPSGMGYEQAMTTRVLKPLKLDH
ACT-23QLU89187.1_4307EBOC    DNAALLRFYQNWQPQWKPGTTRLYANASIGLFGALAVKPSGMGYEQAMTTRVLKPLKLDH
ACT-23QLV76440.1_389EBOC     DNAALLRFYQNWQPQWKPGTTRLYANASIGLFGALAVKPSGMGYEQAMTTRVLKPLKLDH
ACT-23QLT57991.1_396EBOC     DNAALLRFYQNWQPQWKPGTTRLYANASIGLFGALAVKPSGMGYEQAMTTRVLKPLKLDH
ACT-23QLV44058.1_369EBOC     DNAALLRFYQNWQPQWKPGTTRLYANASIGLFGALAVKPSGMGYEQAMTTRVLKPLKLDH
ACT-115AXQ35263.1_2972EBCP   DNASLLRFYQNWQPQWKPGTTRLYANASIGLFGALAVKPSGMPYEQAMTTRVLKPLKLDH
ACT-115QGR11187.1_384EBCP    DNASLLRFYQNWQPQWKPGTTRLYANASIGLFGALAVKPSGMPYEQAMTTRVLKPLKLDH
ACT-115QIF26741.1_2106EBCP   DNASLLRFYQNWQPQWKPGTTRLYANASIGLFGALAVKPSGMPYEQAMTTRVLKPLKLDH
ACT-115QIF32246.1_3030EBCP   DNASLLRFYQNWQPQWKPGTTRLYANASIGLFGALAVKPSGMPYEQAMTTRVLKPLKLDH
ACT-115QOD35429.1_385EBCP    DNASLLRFYQNWQPQWKPGTTRLYANASIGLFGALAVKPSGMPYEQAMTTRVLKPLKLDH
ACT-115QPD66621.1_385EBCP    DNASLLRFYQNWQPQWKPGTTRLYANASIGLFGALAVKPSGMPYEQAMTTRVLKPLKLDH
ACT-115QSS35407.1_385EBCP    DNASLLRFYQNWQPQWKPGTTRLYANASIGLFGALAVKPSGMPYEQAMTTRVLKPLKLDH
ACT-115URE88756.1_385EBCP    DNASLLRFYQNWQPQWKPGTTRLYANASIGLFGALAVKPSGMPYEQAMTTRVLKPLKLDH
ACT-115USM81982.1_384EBCP    DNASLLRFYQNWQPQWKPGTTRLYANASIGLFGALAVKPSGMPYEQAMTTRVLKPLKLDH
ACT-115AXQ35263.1_2972EBOC   DNASLLRFYQNWQPQWKPGTTRLYANASIGLFGALAVKPSGMPYEQAMTTRVLKPLKLDH
ACT-115QGR11187.1_384EBOC    DNASLLRFYQNWQPQWKPGTTRLYANASIGLFGALAVKPSGMPYEQAMTTRVLKPLKLDH
ACT-115QIF26741.1_2106EBOC   DNASLLRFYQNWQPQWKPGTTRLYANASIGLFGALAVKPSGMPYEQAMTTRVLKPLKLDH
ACT-115QIF32246.1_3030EBOC   DNASLLRFYQNWQPQWKPGTTRLYANASIGLFGALAVKPSGMPYEQAMTTRVLKPLKLDH
ACT-115QOD35429.1_385EBOC    DNASLLRFYQNWQPQWKPGTTRLYANASIGLFGALAVKPSGMPYEQAMTTRVLKPLKLDH
ACT-115QPD66621.1_385EBOC    DNASLLRFYQNWQPQWKPGTTRLYANASIGLFGALAVKPSGMPYEQAMTTRVLKPLKLDH
ACT-115QSS35407.1_385EBOC    DNASLLRFYQNWQPQWKPGTTRLYANASIGLFGALAVKPSGMPYEQAMTTRVLKPLKLDH
ACT-115URE88756.1_385EBOC    DNASLLRFYQNWQPQWKPGTTRLYANASIGLFGALAVKPSGMPYEQAMTTRVLKPLKLDH
ACT-115USM81982.1_384EBOC    DNASLLRFYQNWQPQWKPGTTRLYANASIGLFGALAVKPSGMPYEQAMTTRVLKPLKLDH
ACT-25AVU48988.1_427EBCP     DNASLLRFYQNWQPQWKPGTTRLYANASIGLFGALAVKPSGMPYEQAMTTRVLKPLKLDH
ACT-25UQQ46893.1_379EBCP     DNASLLRFYQNWQPQWKPGTTRLYANASIGLFGALAVKPSGMPYEQAMTTRVLKPLKLDH
ACT-25WDO17280.1_310EBCP     DNASLLRFYQNWQPQWKPGTTRLYANASIGLFGALAVKPSGMPYEQAMTTRVLKPLKLDH
ACT-25AVU48988.1_427EBOC     DNASLLRFYQNWQPQWKPGTTRLYANASIGLFGALAVKPSGMPYEQAMTTRVLKPLKLDH
ACT-25UQQ46893.1_379EBOC     DNASLLRFYQNWQPQWKPGTTRLYANASIGLFGALAVKPSGMPYEQAMTTRVLKPLKLDH
ACT-25WDO17280.1_310EBOC     DNASLLRFYQNWQPQWKPGTTRLYANASIGLFGALAVKPSGMPYEQAMTTRVLKPLKLDH
ACT-25AOP89724.1_377EOC      DNASLLRFYQNWQPQWKPGTTRLYANASIGLFGALAVKPSGMPYEQAMTTRVLKPLKLDH
ACT-45APR43249.1_2902EBCP    DNASLLRFYQNWQPQWKPGTTRLYANASIGLFGALAVKPSGMPYEQAMTTRVLKPLKLDH
ACT-45ASA05885.1_3853EBCP    DNASLLRFYQNWQPQWKPGTTRLYANASIGLFGALAVKPSGMPYEQAMTTRVLKPLKLDH
ACT-45ASB85359.1_3592EBCP    DNASLLRFYQNWQPQWKPGTTRLYANASIGLFGALAVKPSGMPYEQAMTTRVLKPLKLDH
ACT-45AWR70714.1_4154EBCP    DNASLLRFYQNWQPQWKPGTTRLYANASIGLFGALAVKPSGMPYEQAMTTRVLKPLKLDH
ACT-45AXM01511.1_4138EBCP    DNASLLRFYQNWQPQWKPGTTRLYANASIGLFGALAVKPSGMPYEQAMTTRVLKPLKLDH
ACT-45AYU96034.1_2649EBCP    DNASLLRFYQNWQPQWKPGTTRLYANASIGLFGALAVKPSGMPYEQAMTTRVLKPLKLDH
ACT-45QBH65043.1_3960EBCP    DNASLLRFYQNWQPQWKPGTTRLYANASIGLFGALAVKPSGMPYEQAMTTRVLKPLKLDH
ACT-45QEL31100.1_1855EBCP    DNASLLRFYQNWQPQWKPGTTRLYANASIGLFGALAVKPSGMPYEQAMTTRVLKPLKLDH
ACT-45ULQ22907.1_427EBCP     DNASLLRFYQNWQPQWKPGTTRLYANASIGLFGALAVKPSGMPYEQAMTTRVLKPLKLDH
ACT-45ULQ25748.1_3478EBCP    DNASLLRFYQNWQPQWKPGTTRLYANASIGLFGALAVKPSGMPYEQAMTTRVLKPLKLDH
ACT-45WCO72956.1_42EBCP      DNASLLRFYQNWQPQWKPGTTRLYANASIGLFGALAVKPSGMPYEQAMTTRVLKPLKLDH
ACT-45APR43249.1_2902EBOC    DNASLLRFYQNWQPQWKPGTTRLYANASIGLFGALAVKPSGMPYEQAMTTRVLKPLKLDH
ACT-45ASA05885.1_3853EBOC    DNASLLRFYQNWQPQWKPGTTRLYANASIGLFGALAVKPSGMPYEQAMTTRVLKPLKLDH
ACT-45ASB85359.1_3592EBOC    DNASLLRFYQNWQPQWKPGTTRLYANASIGLFGALAVKPSGMPYEQAMTTRVLKPLKLDH
ACT-45AWR70714.1_4154EBOC    DNASLLRFYQNWQPQWKPGTTRLYANASIGLFGALAVKPSGMPYEQAMTTRVLKPLKLDH
ACT-45AXM01511.1_4138EBOC    DNASLLRFYQNWQPQWKPGTTRLYANASIGLFGALAVKPSGMPYEQAMTTRVLKPLKLDH
ACT-45AYU96034.1_2649EBOC    DNASLLRFYQNWQPQWKPGTTRLYANASIGLFGALAVKPSGMPYEQAMTTRVLKPLKLDH
ACT-45QBH65043.1_3960EBOC    DNASLLRFYQNWQPQWKPGTTRLYANASIGLFGALAVKPSGMPYEQAMTTRVLKPLKLDH
ACT-45QEL31100.1_1855EBOC    DNASLLRFYQNWQPQWKPGTTRLYANASIGLFGALAVKPSGMPYEQAMTTRVLKPLKLDH
ACT-45ULQ22907.1_427EBOC     DNASLLRFYQNWQPQWKPGTTRLYANASIGLFGALAVKPSGMPYEQAMTTRVLKPLKLDH
ACT-45ULQ25748.1_3478EBOC    DNASLLRFYQNWQPQWKPGTTRLYANASIGLFGALAVKPSGMPYEQAMTTRVLKPLKLDH
ACT-45WCO72956.1_42EBOC      DNASLLRFYQNWQPQWKPGTTRLYANASIGLFGALAVKPSGMPYEQAMTTRVLKPLKLDH
ACT-C197QLA02472.1_2022EOC   DNAALLRFYQNWQPQWKPGTTRLYANASIGLFGALAVKPSGMGYEQAMTTRVLKPLKLDH
ACT-64QMT06026.1_4255EBCP    DNASLLRFYQDWQPQWKPGTTRLYANASIGLFGALAVKPSGMSFEQAMTKRVFKPLKLDH
ACT-64QMT06026.1_4255EBOC    DNASLLRFYQDWQPQWKPGTTRLYANASIGLFGALAVKPSGMSFEQAMTKRVFKPLKLDH
ACT-C198QXA51435.1_2071EOC   DPAALLNFYQTWQPQWKPGTTRLYANSSIGLFGALAVKPSGMGFEQAMTTRVLKPLKLDH
                        acc
```

```
                                      η5        β6    β7           α7      β8        α8          η6
ACT-17BBW34093.1_49417]EBCP                                          TT
                                      250       260          270         280          290         300

ACT-17BBW34093.1_49417]EBCP   TWINVPKAEEAHYAWGYRDGKAVRVSPGMLDAQAYGVKTNVQDMANWVMANMAPENVADA
ACT-17BBW34093.1_49417]EBOC   TWINVPKAEEAHYAWGYRDGKAVRVSPGMLDAQAYGVKTNVQDMANWVMANMAPENVADA
7TI1_1|Chain                  TWINVPKAEEAHYAWGYRDGKAVRVSPGMLDAQAYGVKTNVQDMANWVMANMAPENVADA
ACT-37QPO56206.1_411EBCP      TWINVPKAEAAHYAWGYRDGKAVHVSPGMLDAEAYGVKTNVQDMANWVMANMAPENVADA
ACT-37QPO65286.1_410EBCP      TWINVPKAEAAHYAWGYRDGKAVHVSPGMLDAEAYGVKTNVQDMANWVMANMAPENVADA
ACT-37UKK25917.1_410EBCP      TWINVPKAEAAHYAWGYRDGKAVHVSPGMLDAEAYGVKTNVQDMANWVMANMAPENVADA
ACT-37UKK30553.1_410EBCP      TWINVPKAEAAHYAWGYRDGKAVHVSPGMLDAEAYGVKTNVQDMANWVMANMAPENVADA
ACT-37QPO56206.1_411EBOC      TWINVPKAEAAHYAWGYRDGKAVHVSPGMLDAEAYGVKTNVQDMANWVMANMAPENVADA
ACT-37QPO65286.1_410EBOC      TWINVPKAEAAHYAWGYRDGKAVHVSPGMLDAEAYGVKTNVQDMANWVMANMAPENVADA
ACT-37UKK25917.1_410EBOC      TWINVPKAEAAHYAWGYRDGKAVHVSPGMLDAEAYGVKTNVQDMANWVMANMAPENVADA
ACT-37UKK30553.1_410EBOC      TWINVPKAEAAHYAWGYRDGKAVHVSPGMLDAEAYGVKTNVQDMANWVMANMAPENVADA
ACT-65AXO42196.1_4001EBCP     TWINVPKAEEAHYAWGYRDGKAVRVSPGMLDAQAYGVKTNVQDMANWVMANMAPENVADA
ACT-65AXO42196.1_4001EBOC     TWINVPKAEEAHYAWGYRDGKAVRVSPGMLDAQAYGVKTNVQDMANWVMANMAPENVADA
ACT-65WBV31824.1_394EOC       TWINVPKAEEAHYAWGYRDGKAVRVSPGMLDAQAYGVKTNVQDMANWVMANMAPENVADA
ACT-17ARZ78489.1_1874EBCP     TWINVPKAEEAHYAWGYRDGKAVRVSPGMLDAQAYGVKTNVQDMANWVMANMAPENVADA
ACT-17ASQ75348.1_386EBCP      TWINVPKAEEAHYAWGYRDGKAVRVSPGMLDAQAYGVKTNVQDMANWVMANMAPENVADA
ACT-17AWV77932.1_4320EBCP     TWINVPKAEEAHYAWGYRDGKAVRVSPGMLDAQAYGVKTNVQDMANWVMANMAPENVADA
ACT-17QEQ46013.1_2619EBCP     TWINVPKAEEAHYAWGYRDGKAVRVSPGMLDAQAYGVKTNVQDMANWVMANMAPENVADA
ACT-17QFI20602.1_494EBCP      TWINVPKAEEAHYAWGYRDGKAVRVSPGMLDAQAYGVKTNVQDMANWVMANMAPENVADA
ACT-17QIE65424.1_3050EBCP     TWINVPKAEEAHYAWGYRDGKAVRVSPGMLDAQAYGVKTNVQDMANWVMANMAPENVADA
ACT-17QPD71520.1_416EBCP      TWINVPKAEEAHYAWGYRDGKAVRVSPGMLDAQAYGVKTNVQDMANWVMANMAPENVADA
ACT-17QPO45831.1_587EBCP      TWINVPKAEEAHYAWGYRDGKAVRVSPGMLDAQAYGVKTNVQDMANWVMANMAPENVADA
ACT-17QPO51098.1_586EBCP      TWINVPKAEEAHYAWGYRDGKAVRVSPGMLDAQAYGVKTNVQDMANWVMANMAPENVADA
ACT-17QPO69921.1_405EBCP      TWINVPKAEEAHYAWGYRDGKAVRVSPGMLDAQAYGVKTNVQDMANWVMANMAPENVADA
ACT-17QPO74759.1_405EBCP      TWINVPKAEEAHYAWGYRDGKAVRVSPGMLDAQAYGVKTNVQDMANWVMANMAPENVADA
ACT-17QPO85066.1_405EBCP      TWINVPKAEEAHYAWGYRDGKAVRVSPGMLDAQAYGVKTNVQDMANWVMANMAPENVADA
ACT-17QSB73424.1_1103EBCP     TWINVPKAEEAHYAWGYRDGKAVRVSPGMLDAQAYGVKTNVQDMANWVMANMAPENVADA
ACT-17QXY98988.1_1068EBCP     TWINVPKAEEAHYAWGYRDGKAVRVSPGMLDAQAYGVKTNVQDMANWVMANMAPENVADA
ACT-17UDV73425.1_430EBCP      TWINVPKAEEAHYAWGYRDGKAVRVSPGMLDAQAYGVKTNVQDMANWVMANMAPENVADA
ACT-17UDV69018.1_894EBCP      TWINVPKAEEAHYAWGYRDGKAVRVSPGMLDAQAYGVKTNVQDMANWVMANMAPENVADA
ACT-17UKK21116.1_588EBCP      TWINVPKAEEAHYAWGYRDGKAVRVSPGMLDAQAYGVKTNVQDMANWVMANMAPENVADA
ACT-17UOY44701.1_2566EBCP     TWINVPKAEEAHYAWGYRDGKAVRVSPGMLDAQAYGVKTNVQDMANWVMANMAPENVADA
ACT-17URL69109.1_2030EBCP     TWINVPKAEEAHYAWGYRDGKAVRVSPGMLDAQAYGVKTNVQDMANWVMANMAPENVADA
ACT-17UUQ70490.1_426EBCP      TWINVPKAEEAHYAWGYRDGKAVRVSPGMLDAQAYGVKTNVQDMANWVMANMAPENVADA
ACT-17UWA70738.1_427EBCP      TWINVPKAEEAHYAWGYRDGKAVRVSPGMLDAQAYGVKTNVQDMANWVMANMAPENVADA
ACT-17UWC98141.1_545EBCP      TWINVPKAEEAHYAWGYRDGKAVRVSPGMLDAQAYGVKTNVQDMANWVMANMAPENVADA
ACT-17UXC72711.1_448EBCP      TWINVPKAEEAHYAWGYRDGKAVRVSPGMLDAQAYGVKTNVQDMANWVMANMAPENVADA
ACT-17UXC77377.1_448EBCP      TWINVPKAEEAHYAWGYRDGKAVRVSPGMLDAQAYGVKTNVQDMANWVMANMAPENVADA
ACT-17UXC82023.1_448EBCP      TWINVPKAEEAHYAWGYRDGKAVRVSPGMLDAQAYGVKTNVQDMANWVMANMAPENVADA
ACT-17UZE41799.1_406EBCP      TWINVPKAEEAHYAWGYRDGKAVRVSPGMLDAQAYGVKTNVQDMANWVMANMAPENVADA
ACT-17UZH49391.1_396EBCP      TWINVPKAEEAHYAWGYRDGKAVRVSPGMLDAQAYGVKTNVQDMANWVMANMAPENVADA
ACT-17WAZ63377.1_405EBCP      TWINVPKAEEAHYAWGYRDGKAVRVSPGMLDAQAYGVKTNVQDMANWVMANMAPENVADA
ACT-17WDU86092.1_3769EBCP     TWINVPKAEEAHYAWGYRDGKAVRVSPGMLDAQAYGVKTNVQDMANWVMANMAPENVADA
ACT-17WDW12975.1_3764EBCP     TWINVPKAEEAHYAWGYRDGKAVRVSPGMLDAQAYGVKTNVQDMANWVMANMAPENVADA
ACT-17WGA71117.1_396EBCP      TWINVPKAEEAHYAWGYRDGKAVRVSPGMLDAQAYGVKTNVQDMANWVMANMAPENVADA
ACT-17WGA75603.1_396EBCP      TWINVPKAEEAHYAWGYRDGKAVRVSPGMLDAQAYGVKTNVQDMANWVMANMAPENVADA
ACT-17WGZ57121.1_2667EBCP     TWINVPKAEEAHYAWGYRDGKAVRVSPGMLDAQAYGVKTNVQDMANWVMANMAPENVADA
ACT-17ARZ78489.1_1874EBOC     TWINVPKAEEAHYAWGYRDGKAVRVSPGMLDAQAYGVKTNVQDMANWVMANMAPENVADA
ACT-17ASQ75348.1_386EBOC      TWINVPKAEEAHYAWGYRDGKAVRVSPGMLDAQAYGVKTNVQDMANWVMANMAPENVADA
ACT-17AWV77932.1_4320EBOC     TWINVPKAEEAHYAWGYRDGKAVRVSPGMLDAQAYGVKTNVQDMANWVMANMAPENVADA
ACT-17QEQ46013.1_2619EBOC     TWINVPKAEEAHYAWGYRDGKAVRVSPGMLDAQAYGVKTNVQDMANWVMANMAPENVADA
ACT-17QFI20602.1_494EBOC      TWINVPKAEEAHYAWGYRDGKAVRVSPGMLDAQAYGVKTNVQDMANWVMANMAPENVADA
ACT-17QIE65424.1_3050EBOC     TWINVPKAEEAHYAWGYRDGKAVRVSPGMLDAQAYGVKTNVQDMANWVMANMAPENVADA
ACT-17QPD71520.1_416EBOC      TWINVPKAEEAHYAWGYRDGKAVRVSPGMLDAQAYGVKTNVQDMANWVMANMAPENVADA
ACT-17QPO45831.1_587EBOC      TWINVPKAEEAHYAWGYRDGKAVRVSPGMLDAQAYGVKTNVQDMANWVMANMAPENVADA
ACT-17QPO51098.1_586EBOC      TWINVPKAEEAHYAWGYRDGKAVRVSPGMLDAQAYGVKTNVQDMANWVMANMAPENVADA
ACT-17QPO69921.1_405EBOC      TWINVPKAEEAHYAWGYRDGKAVRVSPGMLDAQAYGVKTNVQDMANWVMANMAPENVADA
ACT-17QPO74759.1_405EBOC      TWINVPKAEEAHYAWGYRDGKAVRVSPGMLDAQAYGVKTNVQDMANWVMANMAPENVADA
ACT-17QPO85066.1_405EBOC      TWINVPKAEEAHYAWGYRDGKAVRVSPGMLDAQAYGVKTNVQDMANWVMANMAPENVADA
ACT-17QSB73424.1_1103EBOC     TWINVPKAEEAHYAWGYRDGKAVRVSPGMLDAQAYGVKTNVQDMANWVMANMAPENVADA
ACT-17QXY98988.1_1068EBOC     TWINVPKAEEAHYAWGYRDGKAVRVSPGMLDAQAYGVKTNVQDMANWVMANMAPENVADA
ACT-17UDV73425.1_430EBOC      TWINVPKAEEAHYAWGYRDGKAVRVSPGMLDAQAYGVKTNVQDMANWVMANMAPENVADA
ACT-17UDV69018.1_894EBOC      TWINVPKAEEAHYAWGYRDGKAVRVSPGMLDAQAYGVKTNVQDMANWVMANMAPENVADA
ACT-17UKK21116.1_588EBOC      TWINVPKAEEAHYAWGYRDGKAVRVSPGMLDAQAYGVKTNVQDMANWVMANMAPENVADA
ACT-17UOY44701.1_2566EBOC     TWINVPKAEEAHYAWGYRDGKAVRVSPGMLDAQAYGVKTNVQDMANWVMANMAPENVADA
ACT-17URL69109.1_2030EBOC     TWINVPKAEEAHYAWGYRDGKAVRVSPGMLDAQAYGVKTNVQDMANWVMANMAPENVADA
ACT-17UUQ70490.1_426EBOC      TWINVPKAEEAHYAWGYRDGKAVRVSPGMLDAQAYGVKTNVQDMANWVMANMAPENVADA
ACT-17UWA70738.1_427EBOC      TWINVPKAEEAHYAWGYRDGKAVRVSPGMLDAQAYGVKTNVQDMANWVMANMAPENVADA
ACT-17UWC98141.1_545EBOC      TWINVPKAEEAHYAWGYRDGKAVRVSPGMLDAQAYGVKTNVQDMANWVMANMAPENVADA
ACT-17UXC72711.1_448EBOC      TWINVPKAEEAHYAWGYRDGKAVRVSPGMLDAQAYGVKTNVQDMANWVMANMAPENVADA
ACT-17UXC77377.1_448EBOC      TWINVPKAEEAHYAWGYRDGKAVRVSPGMLDAQAYGVKTNVQDMANWVMANMAPENVADA
ACT-17UXC82023.1_448EBOC      TWINVPKAEEAHYAWGYRDGKAVRVSPGMLDAQAYGVKTNVQDMANWVMANMAPENVADA
ACT-17UZE41799.1_406EBOC      TWINVPKAEEAHYAWGYRDGKAVRVSPGMLDAQAYGVKTNVQDMANWVMANMAPENVADA
ACT-17UZH49391.1_396EBOC      TWINVPKAEEAHYAWGYRDGKAVRVSPGMLDAQAYGVKTNVQDMANWVMANMAPENVADA
ACT-17WAZ63377.1_405EBOC      TWINVPKAEEAHYAWGYRDGKAVRVSPGMLDAQAYGVKTNVQDMANWVMANMAPENVADA
ACT-17WDU86092.1_3769EBOC     TWINVPKAEEAHYAWGYRDGKAVRVSPGMLDAQAYGVKTNVQDMANWVMANMAPENVADA
ACT-17WDW12975.1_3764EBOC     TWINVPKAEEAHYAWGYRDGKAVRVSPGMLDAQAYGVKTNVQDMANWVMANMAPENVADA
ACT-17WGA71117.1_396EBOC      TWINVPKAEEAHYAWGYRDGKAVRVSPGMLDAQAYGVKTNVQDMANWVMANMAPENVADA
ACT-17WGA75603.1_396EBOC      TWINVPKAEEAHYAWGYRDGKAVRVSPGMLDAQAYGVKTNVQDMANWVMANMAPENVADA
ACT-17WGZ57121.1_2667EBOC     TWINVPKAEEAHYAWGYRDGKAVRVSPGMLDAQAYGVKTNVQDMANWVMANMAPENVADA
ACT-17QEI60759.1_389EOC       TWINVPKAEEAHYAWGYRDGKAVRVSPGMLDAQAYGVKTNVQDMANWVMANMAPENVADA
ACT-17QEI70010.1_389EOC       TWINVPKAEEAHYAWGYRDGKAVRVSPGMLDAQAYGVKTNVQDMANWVMANMAPENVADA
ACT-17QEU13386.1_612EOC       TWINVPKAEEAHYAWGYRDGKAVRVSPGMLDAQAYGVKTNVQDMANWVMANMAPENVADA
ACT-17QHI59365.1_3674EOC      TWINVPKAEEAHYAWGYRDGKAVRVSPGMLDAQAYGVKTNVQDMANWVMANMAPENVADA
ACT-17UZQ58984.1_410EOC       TWINVPKAEEAHYAWGYRDGKAVRVSPGMLDAQAYGVKTNVQDMANWVMANMAPENVADA
ACT-40AWS79793.1_3058EBCP     TWINVPKAEEAHYAWGYRDGKAVRVSPGMLDAQAYGVKTNVQDMANWVMANMAPENVADA
ACT-40AXO44745.1_1607EBCP     TWINVPKAEEAHYAWGYRDGKAVRVSPGMLDAQAYGVKTNVQDMANWVMANMAPENVADA
ACT-40UXI63958.1_1746EBCP     TWINVPKAEEAHYAWGYRDGKAVRVSPGMLDAQAYGVKTNVQDMANWVMANMAPENVADA
ACT-40WDC52451.1_4284EBCP     TWINVPKAEEAHYAWGYRDGKAVRVSPGMLDAQAYGVKTNVQDMANWVMANMAPENVADA
ACT-40AWS79793.1_3058EBOC     TWINVPKAEEAHYAWGYRDGKAVRVSPGMLDAQAYGVKTNVQDMANWVMANMAPENVADA
ACT-40AXO44745.1_1607EBOC     TWINVPKAEEAHYAWGYRDGKAVRVSPGMLDAQAYGVKTNVQDMANWVMANMAPENVADA
ACT-40UXI63958.1_1746EBOC     TWINVPKAEEAHYAWGYRDGKAVRVSPGMLDAQAYGVKTNVQDMANWVMANMAPENVADA
ACT-40WDC52451.1_4284EBOC     TWINVPKAEEAHYAWGYRDGKAVRVSPGMLDAQAYGVKTNVQDMANWVMANMAPENVADA
ACT-44UWA09346.1_3724EBCP     TWINVPKAEEAHYAWGYRDGKAVRVSPGMLDAQAYGVKTNVQDMANWVMANMAPENVADA
ACT-44UWA09346.1_3724EBOC     TWINVPKAEEAHYAWGYRDGKAVRVSPGMLDAQAYGVKTNVQDMANWVMANMAPENVADA
ACT-90QFH83806.1_450EBCP      TWINVPKAEEAHYAWGYRDGKAVRVSPGMLDAQAYGVKTNVQDMANWVMANMAPENVADA
ACT-90QLO92194.1_500EBCP      TWINVPKAEEAHYAWGYRDGKAVRVSPGMLDAQAYGVKTNVQDMANWVMANMAPENVADA
ACT-90QLP01381.1_466EBCP      TWINVPKAEEAHYAWGYRDGKAVRVSPGMLDAQAYGVKTNVQDMANWVMANMAPENVADA
ACT-90QFH83806.1_450EBOC      TWINVPKAEEAHYAWGYRDGKAVRVSPGMLDAQAYGVKTNVQDMANWVMANMAPENVADA
ACT-90QLO92194.1_500EBOC      TWINVPKAEEAHYAWGYRDGKAVRVSPGMLDAQAYGVKTNVQDMANWVMANMAPENVADA
ACT-90QLP01381.1_466EBOC      TWINVPKAEEAHYAWGYRDGKAVRVSPGMLDAQAYGVKTNVQDMANWVMANMAPENVADA
ACT-24ASB73507.1_1185EBCP     TWINVPKAEEAHYAWGYRDGKAVRVSPGMLDAQAYGVKTNVQDMANWVMANMAPENVADA
ACT-24AVU18445.1_378EBCP      TWINVPKAEEAHYAWGYRDGKAVRVSPGMLDAQAYGVKTNVQDMANWVMANMAPENVADA
```

ACT-24QHC75881.1_374EBCP	TWINVPKAEEAHYAWGYRDGKAVRVSPGMLDAQAYGVKTNVQDMANWVMANMAPENVADA
ACT-24QLP84500.1_370EBCP	TWINVPKAEEAHYAWGYRDGKAVRVSPGMLDAQAYGVKTNVQDMANWVMANMAPENVADA
ACT-24QSH03071.1_370EBCP	TWINVPKAEEAHYAWGYRDGKAVRVSPGMLDAQAYGVKTNVQDMANWVMANMAPENVADA
ACT-24QSG93844.1_370EBCP	TWINVPKAEEAHYAWGYRDGKAVRVSPGMLDAQAYGVKTNVQDMANWVMANMAPENVADA
ACT-24QSG98466.1_370EBCP	TWINVPKAEEAHYAWGYRDGKAVRVSPGMLDAQAYGVKTNVQDMANWVMANMAPENVADA
ACT-24QSH12187.1_370EBCP	TWINVPKAEEAHYAWGYRDGKAVRVSPGMLDAQAYGVKTNVQDMANWVMANMAPENVADA
ACT-24QSH16801.1_370EBCP	TWINVPKAEEAHYAWGYRDGKAVRVSPGMLDAQAYGVKTNVQDMANWVMANMAPENVADA
ACT-24QSH07573.1_370EBCP	TWINVPKAEEAHYAWGYRDGKAVRVSPGMLDAQAYGVKTNVQDMANWVMANMAPENVADA
ACT-24QSH21409.1_370EBCP	TWINVPKAEEAHYAWGYRDGKAVRVSPGMLDAQAYGVKTNVQDMANWVMANMAPENVADA
ACT-24QSH26023.1_370EBCP	TWINVPKAEEAHYAWGYRDGKAVRVSPGMLDAQAYGVKTNVQDMANWVMANMAPENVADA
ACT-24QSH30639.1_370EBCP	TWINVPKAEEAHYAWGYRDGKAVRVSPGMLDAQAYGVKTNVQDMANWVMANMAPENVADA
ACT-24UIL48372.1_372EBCP	TWINVPKAEEAHYAWGYRDGKAVRVSPGMLDAQAYGVKTNVQDMANWVMANMAPENVADA
ACT-24UIM14687.1_372EBCP	TWINVPKAEEAHYAWGYRDGKAVRVSPGMLDAQAYGVKTNVQDMANWVMANMAPENVADA
ACT-24UXA74146.1_371EBCP	TWINVPKAEEAHYAWGYRDGKAVRVSPGMLDAQAYGVKTNVQDMANWVMANMAPENVADA
ACT-24WDY48721.1_372EBCP	TWINVPKAEEAHYAWGYRDGKAVRVSPGMLDAQAYGVKTNVQDMANWVMANMAPENVADA
ACT-24ASB73507.1_1185EBOC	TWINVPKAEEAHYAWGYRDGKAVRVSPGMLDAQAYGVKTNVQDMANWVMANMAPENVADA
ACT-24AVU18445.1_378EBOC	TWINVPKAEEAHYAWGYRDGKAVRVSPGMLDAQAYGVKTNVQDMANWVMANMAPENVADA
ACT-24QHC75881.1_374EBOC	TWINVPKAEEAHYAWGYRDGKAVRVSPGMLDAQAYGVKTNVQDMANWVMANMAPENVADA
ACT-24QLP84500.1_370EBOC	TWINVPKAEEAHYAWGYRDGKAVRVSPGMLDAQAYGVKTNVQDMANWVMANMAPENVADA
ACT-24QSH03071.1_370EBOC	TWINVPKAEEAHYAWGYRDGKAVRVSPGMLDAQAYGVKTNVQDMANWVMANMAPENVADA
ACT-24QSG93844.1_370EBOC	TWINVPKAEEAHYAWGYRDGKAVRVSPGMLDAQAYGVKTNVQDMANWVMANMAPENVADA
ACT-24QSG98466.1_370EBOC	TWINVPKAEEAHYAWGYRDGKAVRVSPGMLDAQAYGVKTNVQDMANWVMANMAPENVADA
ACT-24QSH12187.1_370EBOC	TWINVPKAEEAHYAWGYRDGKAVRVSPGMLDAQAYGVKTNVQDMANWVMANMAPENVADA
ACT-24QSH16801.1_370EBOC	TWINVPKAEEAHYAWGYRDGKAVRVSPGMLDAQAYGVKTNVQDMANWVMANMAPENVADA
ACT-24QSH07573.1_370EBOC	TWINVPKAEEAHYAWGYRDGKAVRVSPGMLDAQAYGVKTNVQDMANWVMANMAPENVADA
ACT-24QSH21409.1_370EBOC	TWINVPKAEEAHYAWGYRDGKAVRVSPGMLDAQAYGVKTNVQDMANWVMANMAPENVADA
ACT-24QSH26023.1_370EBOC	TWINVPKAEEAHYAWGYRDGKAVRVSPGMLDAQAYGVKTNVQDMANWVMANMAPENVADA
ACT-24QSH30639.1_370EBOC	TWINVPKAEEAHYAWGYRDGKAVRVSPGMLDAQAYGVKTNVQDMANWVMANMAPENVADA
ACT-24UIL48372.1_372EBOC	TWINVPKAEEAHYAWGYRDGKAVRVSPGMLDAQAYGVKTNVQDMANWVMANMAPENVADA
ACT-24UIM14687.1_372EBOC	TWINVPKAEEAHYAWGYRDGKAVRVSPGMLDAQAYGVKTNVQDMANWVMANMAPENVADA
ACT-24UXA74146.1_371EBOC	TWINVPKAEEAHYAWGYRDGKAVRVSPGMLDAQAYGVKTNVQDMANWVMANMAPENVADA
ACT-24WDY48721.1_372EBOC	TWINVPKAEEAHYAWGYRDGKAVRVSPGMLDAQAYGVKTNVQDMANWVMANMAPENVADA
ACT-89AVE73204.1_2453EBCP	TWINVPKAEEAHYAWGYRDGKAVRVSPGMLDAQAYGVKTNVQDMANWVMANMAPENVADA
ACT-89AVE73204.1_2453EBOC	TWINVPKAEEAHYAWGYRDGKAVRVSPGMLDAQAYGVKTNVQDMANWVMANMAPENVADA
ACT-56ASO99603.1_1270EBCP	TWINVPKAEEAHYAWGYRDGKAVRVSPGMLDAQAYGVKTNVQDMANWVMANMAPENVADA
ACT-56QDQ75241.1_392EBCP	TWINVPKAEEAHYAWGYRDGKAVRVSPGMLDAQAYGVKTNVQDMANWVMANMAPENVADA
ACT-56QFH98918.1_395EBCP	TWINVPKAEEAHYAWGYRDGKAVRVSPGMLDAQAYGVKTNVQDMANWVMANMAPENVADA
ACT-56QFH34334.1_395EBCP	TWINVPKAEEAHYAWGYRDGKAVRVSPGMLDAQAYGVKTNVQDMANWVMANMAPENVADA
ACT-56QFI32707.1_2233EBCP	TWINVPKAEEAHYAWGYRDGKAVRVSPGMLDAQAYGVKTNVQDMANWVMANMAPENVADA
ACT-56QMI65633.1_4184EBCP	TWINVPKAEEAHYAWGYRDGKAVRVSPGMLDAQAYGVKTNVQDMANWVMANMAPENVADA
ACT-56QMI71192.1_422EBCP	TWINVPKAEEAHYAWGYRDGKAVRVSPGMLDAQAYGVKTNVQDMANWVMANMAPENVADA
ACT-56UDV17856.1_8EBCP	TWINVPKAEEAHYAWGYRDGKAVRVSPGMLDAQAYGVKTNVQDMANWVMANMAPENVADA
ACT-56UDV39132.1_1376EBCP	TWINVPKAEEAHYAWGYRDGKAVRVSPGMLDAQAYGVKTNVQDMANWVMANMAPENVADA
ACT-56UWB10427.1_430EBCP	TWINVPKAEEAHYAWGYRDGKAVRVSPGMLDAQAYGVKTNVQDMANWVMANMAPENVADA
ACT-56UWC18315.1_415EBCP	TWINVPKAEEAHYAWGYRDGKAVRVSPGMLDAQAYGVKTNVQDMANWVMANMAPENVADA
ACT-56UWC23093.1_417EBCP	TWINVPKAEEAHYAWGYRDGKAVRVSPGMLDAQAYGVKTNVQDMANWVMANMAPENVADA
ACT-56WBT12607.1_393EBCP	TWINVPKAEEAHYAWGYRDGKAVRVSPGMLDAQAYGVKTNVQDMANWVMANMAPENVADA
ACT-56WBT28831.1_393EBCP	TWINVPKAEEAHYAWGYRDGKAVRVSPGMLDAQAYGVKTNVQDMANWVMANMAPENVADA
ACT-56WGB34617.1_392EBCP	TWINVPKAEEAHYAWGYRDGKAVRVSPGMLDAQAYGVKTNVQDMANWVMANMAPENVADA
ACT-56WGU03466.1_393EBCP	TWINVPKAEEAHYAWGYRDGKAVRVSPGMLDAQAYGVKTNVQDMANWVMANMAPENVADA
ACT-56ASO99603.1_1270EBOC	TWINVPKAEEAHYAWGYRDGKAVRVSPGMLDAQAYGVKTNVQDMANWVMANMAPENVADA
ACT-56QDQ75241.1_392EBOC	TWINVPKAEEAHYAWGYRDGKAVRVSPGMLDAQAYGVKTNVQDMANWVMANMAPENVADA
ACT-56QFH98918.1_395EBOC	TWINVPKAEEAHYAWGYRDGKAVRVSPGMLDAQAYGVKTNVQDMANWVMANMAPENVADA
ACT-56QFH34334.1_395EBOC	TWINVPKAEEAHYAWGYRDGKAVRVSPGMLDAQAYGVKTNVQDMANWVMANMAPENVADA
ACT-56QFI32707.1_2233EBOC	TWINVPKAEEAHYAWGYRDGKAVRVSPGMLDAQAYGVKTNVQDMANWVMANMAPENVADA
ACT-56QMI65633.1_4184EBOC	TWINVPKAEEAHYAWGYRDGKAVRVSPGMLDAQAYGVKTNVQDMANWVMANMAPENVADA
ACT-56QMI71192.1_422EBOC	TWINVPKAEEAHYAWGYRDGKAVRVSPGMLDAQAYGVKTNVQDMANWVMANMAPENVADA
ACT-56UDV17856.1_8EBOC	TWINVPKAEEAHYAWGYRDGKAVRVSPGMLDAQAYGVKTNVQDMANWVMANMAPENVADA
ACT-56UDV39132.1_1376EBOC	TWINVPKAEEAHYAWGYRDGKAVRVSPGMLDAQAYGVKTNVQDMANWVMANMAPENVADA
ACT-56UWB10427.1_430EBOC	TWINVPKAEEAHYAWGYRDGKAVRVSPGMLDAQAYGVKTNVQDMANWVMANMAPENVADA
ACT-56UWC18315.1_415EBOC	TWINVPKAEEAHYAWGYRDGKAVRVSPGMLDAQAYGVKTNVQDMANWVMANMAPENVADA
ACT-56UWC23093.1_417EBOC	TWINVPKAEEAHYAWGYRDGKAVRVSPGMLDAQAYGVKTNVQDMANWVMANMAPENVADA
ACT-56WBT12607.1_393EBOC	TWINVPKAEEAHYAWGYRDGKAVRVSPGMLDAQAYGVKTNVQDMANWVMANMAPENVADA
ACT-56WBT28831.1_393EBOC	TWINVPKAEEAHYAWGYRDGKAVRVSPGMLDAQAYGVKTNVQDMANWVMANMAPENVADA
ACT-56WGB34617.1_392EBOC	TWINVPKAEEAHYAWGYRDGKAVRVSPGMLDAQAYGVKTNVQDMANWVMANMAPENVADA
ACT-56WGU03466.1_393EBOC	TWINVPKAEEAHYAWGYRDGKAVRVSPGMLDAQAYGVKTNVQDMANWVMANMAPENVADA
ACT-56AOP76464.1_392EOC	TWINVPKAEEAHYAWGYRDGKAVRVSPGMLDAQAYGVKTNVQDMANWVMANMAPENVADA
ACT-56QXA77046.1_2672EOC	TWINVPKAEEAHYAWGYRDGKAVRVSPGMLDAQAYGVKTNVQDMANWVMANMAPENVADA
ACT-56WFP29817.1_397EOC	TWINVPKAEEAHYAWGYRDGKAVRVSPGMLDAQAYGVKTNVQDMANWVMANMAPENVADA
ACT-27AXO49795.1_1610EBCP	TWINVPKAEEAHYAWGYRDGKAVRVSPGMLDAQAYGVKTNVQDMANWVMANMAPENVADA
ACT-27QHO79915.1_457EBCP	TWINVPKAEEAHYAWGYRDGKAVRVSPGMLDAQAYGVKTNVQDMANWVMANMAPENVADA
ACT-27QHO97012.1_458EBCP	TWINVPKAEEAHYAWGYRDGKAVRVSPGMLDAQAYGVKTNVQDMANWVMANMAPENVADA
ACT-27QQH03712.1_3802EBCP	TWINVPKAEEAHYAWGYRDGKAVRVSPGMLDAQAYGVKTNVQDMANWVMANMAPENVADA
ACT-27QQH08784.1_3802EBCP	TWINVPKAEEAHYAWGYRDGKAVRVSPGMLDAQAYGVKTNVQDMANWVMANMAPENVADA
ACT-27UVZ91365.1_388EBCP	TWINVPKAEEAHYAWGYRDGKAVRVSPGMLDAQAYGVKTNVQDMANWVMANMAPENVADA
ACT-27AXO49795.1_1610EBOC	TWINVPKAEEAHYAWGYRDGKAVRVSPGMLDAQAYGVKTNVQDMANWVMANMAPENVADA
ACT-27QHO79915.1_457EBOC	TWINVPKAEEAHYAWGYRDGKAVRVSPGMLDAQAYGVKTNVQDMANWVMANMAPENVADA
ACT-27QHO97012.1_458EBOC	TWINVPKAEEAHYAWGYRDGKAVRVSPGMLDAQAYGVKTNVQDMANWVMANMAPENVADA
ACT-27QQH03712.1_3802EBOC	TWINVPKAEEAHYAWGYRDGKAVRVSPGMLDAQAYGVKTNVQDMANWVMANMAPENVADA
ACT-27QQH08784.1_3802EBOC	TWINVPKAEEAHYAWGYRDGKAVRVSPGMLDAQAYGVKTNVQDMANWVMANMAPENVADA
ACT-27UVZ91365.1_388EBOC	TWINVPKAEEAHYAWGYRDGKAVRVSPGMLDAQAYGVKTNVQDMANWVMANMAPENVADA
ACT-102QEO03155.1_4213EBCP	TWINVPKEEEAHYAWGYRDGKAIHVSPGMLDAEAYGVKTNIQDMASWVKANMNPDALPNS
ACT-102BBV79841.1_51928]EBCP	TWINVPKEEEAHYAWGYRDGKAIHVSPGMLDAEAYGVKTNIQDMASWVKANMNPDALPNS
ACT-102QZS47739.1_401EBCP	TWINVPKEEEAHYAWGYRDGKAIHVSPGMLDAEAYGVKTNIQDMASWVKANMNPDALPNS
ACT-102QEO03155.1_4213EBOC	TWINVPKEEEAHYAWGYRDGKAIHVSPGMLDAEAYGVKTNIQDMASWVKANMNPDALPNS
ACT-102BBV79841.1_51928]EBOC	TWINVPKEEEAHYAWGYRDGKAIHVSPGMLDAEAYGVKTNIQDMASWVKANMNPDALPNS
ACT-102QZS47739.1_401EBOC	TWINVPKEEEAHYAWGYRDGKAIHVSPGMLDAEAYGVKTNIQDMASWVKANMNPDALPNS
ACT-55QLP71015.1_413EBCP	TWINVPKAEEAHYAWGYRDGKAVRVSPGMLDAQAYGVKTNVQDMANWVMANMAPENVADA
ACT-55QLP80034.1_414EBCP	TWINVPKAEEAHYAWGYRDGKAVRVSPGMLDAQAYGVKTNVQDMANWVMANMAPENVADA
ACT-55QLS08818.1_415EBCP	TWINVPKAEEAHYAWGYRDGKAVRVSPGMLDAQAYGVKTNVQDMANWVMANMAPENVADA
ACT-55QLT25377.1_3621EBCP	TWINVPKAEEAHYAWGYRDGKAVRVSPGMLDAQAYGVKTNVQDMANWVMANMAPENVADA
ACT-55QLV12510.1_4006EBCP	TWINVPKAEEAHYAWGYRDGKAVRVSPGMLDAQAYGVKTNVQDMANWVMANMAPENVADA
ACT-55QLW04180.1_4182EBCP	TWINVPKAEEAHYAWGYRDGKAVRVSPGMLDAQAYGVKTNVQDMANWVMANMAPENVADA
ACT-55QLV08026.1_4006EBCP	TWINVPKAEEAHYAWGYRDGKAVRVSPGMLDAQAYGVKTNVQDMANWVMANMAPENVADA
ACT-55QLV96171.1_421EBCP	TWINVPKAEEAHYAWGYRDGKAVRVSPGMLDAQAYGVKTNVQDMANWVMANMAPENVADA
ACT-55QLW44293.1_405EBCP	TWINVPKAEEAHYAWGYRDGKAVRVSPGMLDAQAYGVKTNVQDMANWVMANMAPENVADA
ACT-55UBH32480.1_675EBCP	TWINVPKAEEAHYAWGYRDGKAVRVSPGMLDAQAYGVKTNVQDMANWVMANMAPENVADA
ACT-55UDV61223.1_2251EBCP	TWINVPKAEEAHYAWGYRDGKAVRVSPGMLDAQAYGVKTNVQDMANWVMANMAPENVADA
ACT-55QLP71015.1_413EBOC	TWINVPKAEEAHYAWGYRDGKAVRVSPGMLDAQAYGVKTNVQDMANWVMANMAPENVADA
ACT-55QLP80034.1_414EBOC	TWINVPKAEEAHYAWGYRDGKAVRVSPGMLDAQAYGVKTNVQDMANWVMANMAPENVADA
ACT-55QLS08818.1_415EBOC	TWINVPKAEEAHYAWGYRDGKAVRVSPGMLDAQAYGVKTNVQDMANWVMANMAPENVADA
ACT-55QLT25377.1_3621EBOC	TWINVPKAEEAHYAWGYRDGKAVRVSPGMLDAQAYGVKTNVQDMANWVMANMAPENVADA
ACT-55QLV12510.1_4006EBOC	TWINVPKAEEAHYAWGYRDGKAVRVSPGMLDAQAYGVKTNVQDMANWVMANMAPENVADA

```
ACT-55QLW04180.1_4182EBOC      TWINVPKAEEAHYAWGYRDGKAVRVSPGMLDAQAYGVKTNVQDMANWVMANMAPENVADA
ACT-55QLV08026.1_4006EBOC      TWINVPKAEEAHYAWGYRDGKAVRVSPGMLDAQAYGVKTNVQDMANWVMANMAPENVADA
ACT-55QLV96171.1_421EBOC       TWINVPKAEEAHYAWGYRDGKAVRVSPGMLDAQAYGVKTNVQDMANWVMANMAPENVADA
ACT-55QLW44293.1_405EBOC       TWINVPKAEEAHYAWGYRDGKAVRVSPGMLDAQAYGVKTNVQDMANWVMANMAPENVADA
ACT-55UBH32480.1_675EBOC       TWINVPKAEEAHYAWGYRDGKAVRVSPGMLDAQAYGVKTNVQDMANWVMANMAPENVADA
ACT-55UDV61223.1_2251EBOC      TWINVPKAEEAHYAWGYRDGKAVRVSPGMLDAQAYGVKTNVQDMANWVMANMAPENVADA
ACT-55AOP80857.1_409EOC        TWINVPKAEEAHYAWGYRDGKAVRVSPGMLDAQAYGVKTNVQDMANWVMANMAPENVADA
ACT-51BBW20048.1_38351]EBCP    TWINVPKEEEAHYAWGYRDGKATHVSPGMLDAEAYGVKTNVQDMASWVKANMNPDALPDS
ACT-51BBW20048.1_38351]EBOC    TWINVPKEEEAHYAWGYRDGKATHVSPGMLDAEAYGVKTNVQDMASWVKANMNPDALPDS
ACT-28URE95606.1_351EBCP       TWINVPKEEEAHYAWGYRDGKAIHVSPGMLDAEAYGVKTNIQDMASWLKANMNPDALPDS
ACT-28URE95606.1_351EBOC       TWINVPKEEEAHYAWGYRDGKAIHVSPGMLDAEAYGVKTNIQDMASWLKANMNPDALPDS
ACT-87AYL04658.1_1249EBCP      TWINVPKEEEAHYAWGYRDGKAIHVSPGMLDTEAYGVKTNIQDMASWLKANMNPDALSDS
ACT-87QIP18425.1_352EBCP       TWINVPKEEEAHYAWGYRDGKAIHVSPGMLDTEAYGVKTNIQDMASWLKANMNPDALSDS
ACT-87AYL04658.1_1249EBOC      TWINVPKEEEAHYAWGYRDGKAIHVSPGMLDTEAYGVKTNIQDMASWLKANMNPDALSDS
ACT-87QIP18425.1_352EBOC       TWINVPKEEEAHYAWGYRDGKAIHVSPGMLDTEAYGVKTNIQDMASWLKANMNPDALSDS
ACT-75AWX01586.1_1447EBCP      TWINVPKAEEAHYARGYRDGKAVRVSPGMLDAQAYGVKTNVQDMANWVMANMAPEKVADA
ACT-75AWX01586.1_1447EBOC      TWINVPKAEEAHYARGYRDGKAVRVSPGMLDAQAYGVKTNVQDMANWVMANMAPEKVADA
ACT-103URL27837.1_342EOC       TWINVPKEEEAHYAWGYRDGKAIHVSPGMLDAEAYGVKTNIQDMASWLKANMNPDALPDS
ACT-99UXJ64669.1_346EBCP       TWINVPKEEEAYYAWGYRDGKAVHVSPGMLDAEAYGVKTNVQDMASWVKANMNPAALPDS
ACT-99UXJ64669.1_346EBOC       TWINVPKEEEAYYAWGYRDGKAVHVSPGMLDAEAYGVKTNVQDMASWVKANMNPAALPDS
ACT-95QIB80518.1_348EBCP       TWINVPKEEEAHYAWGYRDGKAIHVSPGMLDAEAYGVKTNIQDMASWLKANMNPDALSDS
ACT-95BBV69349.1_35128]EBCP    TWINVPKEEEAHYAWGYRDGKAIHVSPGMLDAEAYGVKTNIQDMASWLKANMNPDALSDS
ACT-95UZQ68133.1_388EBCP       TWINVPKEEEAHYAWGYRDGKAIHVSPGMLDAEAYGVKTNIQDMASWLKANMNPDALSDS
ACT-95QIB80518.1_348EBOC       TWINVPKEEEAHYAWGYRDGKAIHVSPGMLDAEAYGVKTNIQDMASWLKANMNPDALSDS
ACT-95BBV69349.1_35128]EBOC    TWINVPKEEEAHYAWGYRDGKAIHVSPGMLDAEAYGVKTNIQDMASWLKANMNPDALSDS
ACT-95UZQ68133.1_388EBOC       TWINVPKEEEAHYAWGYRDGKAIHVSPGMLDAEAYGVKTNIQDMASWLKANMNPDALSDS
ACT-52QFH88560.1_350EBCP       TWINVPKEEEAHYAWGYRDGKAVHVSPGMLDAEAYGVKTNVQDISSWVKANMNPAALPDS
ACT-52BBS30224.1_43052]EBCP    TWINVPKEEEAHYAWGYRDGKAVHVSPGMLDAEAYGVKTNVQDISSWVKANMNPAALPDS
ACT-52WBN86316.1_4083EBCP      TWINVPKEEEAHYAWGYRDGKAVHVSPGMLDAEAYGVKTNVQDISSWVKANMNPAALPDS
ACT-52QFH88560.1_350EBOC       TWINVPKEEEAHYAWGYRDGKAVHVSPGMLDAEAYGVKTNVQDISSWVKANMNPAALPDS
ACT-52BBS30224.1_43052]EBOC    TWINVPKEEEAHYAWGYRDGKAVHVSPGMLDAEAYGVKTNVQDISSWVKANMNPAALPDS
ACT-52WBN86316.1_4083EBOC      TWINVPKEEEAHYAWGYRDGKAVHVSPGMLDAEAYGVKTNVQDISSWVKANMNPAALPDS
ACT-4QLR27288.1_395EBCP        TWINVPKAEEAHYAWGYRDGKAVHVSPGMLDAEAYGVKTNVQDMASWVMVNMKPDSLQDN
ACT-4QPS68257.1_413EBCP        TWINVPKAEEAHYAWGYRDGKAVHVSPGMLDAEAYGVKTNVQDMASWVMVNMKPDSLQDN
ACT-4QLR27288.1_395EBOC        TWINVPKAEEAHYAWGYRDGKAVHVSPGMLDAEAYGVKTNVQDMASWVMVNMKPDSLQDN
ACT-4QPS68257.1_413EBOC        TWINVPKAEEAHYAWGYRDGKAVHVSPGMLDAEAYGVKTNVQDMASWVMVNMKPDSLQDN
ACT-3UOY53510.1_358EBCP        TWINVPKAEEAHYAWGYRDGKAIHVSPGMLDAEAYGVKTNVQDMASWVMVNMKPDSLQDN
ACT-3UOY63785.1_4171EBCP       TWINVPKAEEAHYAWGYRDGKAIHVSPGMLDAEAYGVKTNVQDMASWVMVNMKPDSLQDN
ACT-3UOY53510.1_358EBOC        TWINVPKAEEAHYAWGYRDGKAIHVSPGMLDAEAYGVKTNVQDMASWVMVNMKPDSLQDN
ACT-3UOY63785.1_4171EBOC       TWINVPKAEEAHYAWGYRDGKAIHVSPGMLDAEAYGVKTNVQDMASWVMVNMKPDSLQDN
ACT-1QLV61731.1_472EBCP        TWINVPKAEEAHYAWGYRDGKAVHVSPGMLDAEAYGVKTNVQDMASWVMVNMKPDSLQDN
ACT-1UWA77567.1_382EBCP        TWINVPKAEEAHYAWGYRDGKAVHVSPGMLDAEAYGVKTNVQDMASWVMVNMKPDSLQDN
ACT-1QLV61731.1_472EBOC        TWINVPKAEEAHYAWGYRDGKAVHVSPGMLDAEAYGVKTNVQDMASWVMVNMKPDSLQDN
ACT-1UWA77567.1_382EBOC        TWINVPKAEEAHYAWGYRDGKAVHVSPGMLDAEAYGVKTNVQDMASWVMVNMKPDSLQDN
ACT-77QWZ50133.1_330EBCP       TWINVPKAEETHYAWGYRDGKAVHVSPGMLDAEAYGVKTNVQDMASWVMANMAPDALQDT
ACT-77UBH39796.1_3999EBCP      TWINVPKAEETHYAWGYRDGKAVHVSPGMLDAEAYGVKTNVQDMASWVMANMAPDALQDT
ACT-77UBH98103.1_3842EBCP      TWINVPKAEETHYAWGYRDGKAVHVSPGMLDAEAYGVKTNVQDMASWVMANMAPDALQDT
ACT-77UBH94108.1_193EBCP       TWINVPKAEETHYAWGYRDGKAVHVSPGMLDAEAYGVKTNVQDMASWVMANMAPDALQDT
ACT-77QWZ50133.1_330EBOC       TWINVPKAEETHYAWGYRDGKAVHVSPGMLDAEAYGVKTNVQDMASWVMANMAPDALQDT
ACT-77UBH39796.1_3999EBOC      TWINVPKAEETHYAWGYRDGKAVHVSPGMLDAEAYGVKTNVQDMASWVMANMAPDALQDT
ACT-77UBH98103.1_3842EBOC      TWINVPKAEETHYAWGYRDGKAVHVSPGMLDAEAYGVKTNVQDMASWVMANMAPDALQDT
ACT-77UBH94108.1_193EBOC       TWINVPKAEETHYAWGYRDGKAVHVSPGMLDAEAYGVKTNVQDMASWVMANMAPDALQDT
ACT-53QEL38380.1_485EBCP       TWINVPKAEEAHYAWGYRDGKAVHVSPGMLDAEAYGVKTNVQDMASWVMVNMKPEALQDT
ACT-53QEL38380.1_485EBOC       TWINVPKAEEAHYAWGYRDGKAVHVSPGMLDAEAYGVKTNVQDMASWVMVNMKPEALQDT
ACT-57QBB07838.1_4703EBCP      TWINVPKAEEAHYAWGYRDGKAVHVSPGMLDAEAYGVKTNVKDMANWVMVNMKPDSLEDS
ACT-57QYD27335.1_499EBCP       TWINVPKAEEAHYAWGYRDGKAVHVSPGMLDAEAYGVKTNVKDMANWVMVNMKPDSLEDS
ACT-57UBM17197.1_2672EBCP      TWINVPKAEEAHYAWGYRDGKAVHVSPGMLDAEAYGVKTNVKDMANWVMVNMKPDSLEDS
ACT-57UKB55021.1_380EBCP       TWINVPKAEEAHYAWGYRDGKAVHVSPGMLDAEAYGVKTNVKDMANWVMVNMKPDSLEDS
ACT-57QBB07838.1_4703EBOC      TWINVPKAEEAHYAWGYRDGKAVHVSPGMLDAEAYGVKTNVKDMANWVMVNMKPDSLEDS
ACT-57QYD27335.1_499EBOC       TWINVPKAEEAHYAWGYRDGKAVHVSPGMLDAEAYGVKTNVKDMANWVMVNMKPDSLEDS
ACT-57UBM17197.1_2672EBOC      TWINVPKAEEAHYAWGYRDGKAVHVSPGMLDAEAYGVKTNVKDMANWVMVNMKPDSLEDS
ACT-57UKB55021.1_380EBOC       TWINVPKAEEAHYAWGYRDGKAVHVSPGMLDAEAYGVKTNVKDMANWVMVNMKPDSLEDS
ACT-93ALL15984.1_396EOC        TWINVPKAEEAHYAWGYRDGKAVHVSPGMLDAEAYGVKTNVQDMASWVMVNMKPDSLQDS
ACT-9AOP85131.1_312EBCP        TWINVPKEEEAHYAWGYRDGKAIHVSPGMLDAEAYGVKTNIQDMASWLKANMNPDALSDS
ACT-9UOY32628.1_343EBCP        TWINVPKEEEAHYAWGYRDGKAIHVSPGMLDAEAYGVKTNIQDMASWLKANMNPDALSDS
ACT-9AOP85131.1_312EBOC        TWINVPKEEEAHYAWGYRDGKAIHVSPGMLDAEAYGVKTNIQDMASWLKANMNPDALSDS
ACT-9UOY32628.1_343EBOC        TWINVPKEEEAHYAWGYRDGKAIHVSPGMLDAEAYGVKTNIQDMASWLKANMNPDALSDS
ACT-9AMZ75637.1_173EOC         TWINVPKEEEAHYAWGYRDGKAIHVSPGMLDAEAYGVKTNIQDMASWLKANMNPDALSDS
ACT-6BBT43181.1_3396]EBCP      TWINVPKAEEAHYAWGYRDGKAVHVSPGMLDAEAYGVKTNVKDMANWVMVNMKPDSLQDS
ACT-6BBT43181.1_3396]EBOC      TWINVPKAEEAHYAWGYRDGKAVHVSPGMLDAEAYGVKTNVKDMANWVMVNMKPDSLQDS
ACT-46QUP91302.1_387EBCP       TWINVPKAEEAHYAWGYRDGKAVRVSPGMLDAQAYGVKTNVQDMANWVMANMAPEKVADA
ACT-46QUP91302.1_387EBOC       TWINVPKAEEAHYAWGYRDGKAVRVSPGMLDAQAYGVKTNVQDMANWVMANMAPEKVADA
ACT-67QLP16520.1_400EBCP       TWINVPKAEEAHYAWGYRDGKAVRVSPGMLDAQAYGVKTNVQDMANWVMANMAPEKVADA
ACT-67QLP16520.1_400EBOC       TWINVPKAEEAHYAWGYRDGKAVRVSPGMLDAQAYGVKTNVQDMANWVMANMAPEKVADA
ACT-84QOR49295.1_413EBCP       TWINVPKAEEAHYAWGYRDGKAVRVSPGMLDAQAYGVKTNVQDMANWVMANMAPEKVADA
ACT-84QOR49295.1_413EBOC       TWINVPKAEEAHYAWGYRDGKAVRVSPGMLDAQAYGVKTNVQDMANWVMANMAPEKVADA
ACT-74QOX68946.1_496EBCP       TWINVPKAEEAHYAWGYRDGKAVRVSPGMLDAQAYGVKTNVQDMANWVMANMAPEKVADA
ACT-74UIZ04332.1_460EBCP       TWINVPKAEEAHYAWGYRDGKAVRVSPGMLDAQAYGVKTNVQDMANWVMANMAPEKVADA
ACT-74QOX68946.1_496EBOC       TWINVPKAEEAHYAWGYRDGKAVRVSPGMLDAQAYGVKTNVQDMANWVMANMAPEKVADA
ACT-74UIZ04332.1_460EBOC       TWINVPKAEEAHYAWGYRDGKAVRVSPGMLDAQAYGVKTNVQDMANWVMANMAPEKVADA
ACT-74QQT40108.1_4574EOC       TWINVPKAEEAHYAWGYRDGKAVRVSPGMLDAQAYGVKTNVQDMANWVMANMAPEKVADA
ACT-16ATW90451.1_311EBCP       TWINVPKAEEAHYAWGYRDGKAVRVSPGMLDAQAYGVKTNVQDMANWVMANMAPEKVADA
ACT-16AVO82105.1_1257EBCP      TWINVPKAEEAHYAWGYRDGKAVRVSPGMLDAQAYGVKTNVQDMANWVMANMAPEKVADA
ACT-16AWQ41825.1_393EBCP       TWINVPKAEEAHYAWGYRDGKAVRVSPGMLDAQAYGVKTNVQDMANWVMANMAPEKVADA
ACT-16AWQ56038.1_393EBCP       TWINVPKAEEAHYAWGYRDGKAVRVSPGMLDAQAYGVKTNVQDMANWVMANMAPEKVADA
ACT-16QFH53420.1_474EBCP       TWINVPKAEEAHYAWGYRDGKAVRVSPGMLDAQAYGVKTNVQDMANWVMANMAPEKVADA
ACT-16QPO90952.1_396EBCP       TWINVPKAEEAHYAWGYRDGKAVRVSPGMLDAQAYGVKTNVQDMANWVMANMAPEKVADA
ACT-16QQA67679.1_472EBCP       TWINVPKAEEAHYAWGYRDGKAVRVSPGMLDAQAYGVKTNVQDMANWVMANMAPEKVADA
ACT-16UDV14809.1_2173EBCP      TWINVPKAEEAHYAWGYRDGKAVRVSPGMLDAQAYGVKTNVQDMANWVMANMAPEKVADA
ACT-16UDV41468.1_3105EBCP      TWINVPKAEEAHYAWGYRDGKAVRVSPGMLDAQAYGVKTNVQDMANWVMANMAPEKVADA
ACT-16UIZ08994.1_395EBCP       TWINVPKAEEAHYAWGYRDGKAVRVSPGMLDAQAYGVKTNVQDMANWVMANMAPEKVADA
ACT-16WJJ28417.1_386EBCP       TWINVPKAEEAHYAWGYRDGKAVRVSPGMLDAQAYGVKTNVQDMANWVMANMAPEKVADA
ACT-16ATW90451.1_311EBOC       TWINVPKAEEAHYAWGYRDGKAVRVSPGMLDAQAYGVKTNVQDMANWVMANMAPEKVADA
ACT-16AVO82105.1_1257EBOC      TWINVPKAEEAHYAWGYRDGKAVRVSPGMLDAQAYGVKTNVQDMANWVMANMAPEKVADA
ACT-16AWQ41825.1_393EBOC       TWINVPKAEEAHYAWGYRDGKAVRVSPGMLDAQAYGVKTNVQDMANWVMANMAPEKVADA
ACT-16AWQ56038.1_393EBOC       TWINVPKAEEAHYAWGYRDGKAVRVSPGMLDAQAYGVKTNVQDMANWVMANMAPEKVADA
ACT-16QFH53420.1_474EBOC       TWINVPKAEEAHYAWGYRDGKAVRVSPGMLDAQAYGVKTNVQDMANWVMANMAPEKVADA
ACT-16QPO90952.1_396EBOC       TWINVPKAEEAHYAWGYRDGKAVRVSPGMLDAQAYGVKTNVQDMANWVMANMAPEKVADA
ACT-16QQA67679.1_472EBOC       TWINVPKAEEAHYAWGYRDGKAVRVSPGMLDAQAYGVKTNVQDMANWVMANMAPEKVADA
ACT-16UDV14809.1_2173EBOC      TWINVPKAEEAHYAWGYRDGKAVRVSPGMLDAQAYGVKTNVQDMANWVMANMAPEKVADA
ACT-16UDV41468.1_3105EBOC      TWINVPKAEEAHYAWGYRDGKAVRVSPGMLDAQAYGVKTNVQDMANWVMANMAPEKVADA
ACT-16UIZ08994.1_395EBOC       TWINVPKAEEAHYAWGYRDGKAVRVSPGMLDAQAYGVKTNVQDMANWVMANMAPEKVADA
ACT-16WJJ28417.1_386EBOC       TWINVPKAEEAHYAWGYRDGKAVRVSPGMLDAQAYGVKTNVQDMANWVMANMAPEKVADA
```

```
ACT-23QLP51667.1_388EBCP      TWINVPKAEEEAHYAWGYRDGKAVRVSPGMLDAQAYGVKTNVQDMANWVMANMVPEKVADA
ACT-23QLP61663.1_388EBCP      TWINVPKAEEEAHYAWGYRDGKAVRVSPGMLDAQAYGVKTNVQDMANWVMANMVPEKVADA
ACT-23QLP66427.1_430EBCP      TWINVPKAEEEAHYAWGYRDGKAVRVSPGMLDAQAYGVKTNVQDMANWVMANMVPEKVADA
ACT-23QLU80799.1_388EBCP      TWINVPKAEEEAHYAWGYRDGKAVRVSPGMLDAQAYGVKTNVQDMANWVMANMVPEKVADA
ACT-23QLT97825.1_367EBCP      TWINVPKAEEEAHYAWGYRDGKAVRVSPGMLDAQAYGVKTNVQDMANWVMANMVPEKVADA
ACT-23QLU89187.1_4307EBCP     TWINVPKAEEEAHYAWGYRDGKAVRVSPGMLDAQAYGVKTNVQDMANWVMANMVPEKVADA
ACT-23QLV76440.1_389EBCP      TWINVPKAEEEAHYAWGYRDGKAVRVSPGMLDAQAYGVKTNVQDMANWVMANMVPEKVADA
ACT-23QLT57991.1_396EBCP      TWINVPKAEEEAHYAWGYRDGKAVRVSPGMLDAQAYGVKTNVQDMANWVMANMVPEKVADA
ACT-23QLV44058.1_369EBCP      TWINVPKAEEEAHYAWGYRDGKAVRVSPGMLDAQAYGVKTNVQDMANWVMANMVPEKVADA
ACT-23QLP51667.1_388EBOC      TWINVPKAEEEAHYAWGYRDGKAVRVSPGMLDAQAYGVKTNVQDMANWVMANMVPEKVADA
ACT-23QLP61663.1_388EBOC      TWINVPKAEEEAHYAWGYRDGKAVRVSPGMLDAQAYGVKTNVQDMANWVMANMVPEKVADA
ACT-23QLP66427.1_430EBOC      TWINVPKAEEEAHYAWGYRDGKAVRVSPGMLDAQAYGVKTNVQDMANWVMANMVPEKVADA
ACT-23QLU80799.1_388EBOC      TWINVPKAEEEAHYAWGYRDGKAVRVSPGMLDAQAYGVKTNVQDMANWVMANMVPEKVADA
ACT-23QLT97825.1_367EBOC      TWINVPKAEEEAHYAWGYRDGKAVRVSPGMLDAQAYGVKTNVQDMANWVMANMVPEKVADA
ACT-23QLU89187.1_4307EBOC     TWINVPKAEEEAHYAWGYRDGKAVRVSPGMLDAQAYGVKTNVQDMANWVMANMVPEKVADA
ACT-23QLV76440.1_389EBOC      TWINVPKAEEEAHYAWGYRDGKAVRVSPGMLDAQAYGVKTNVQDMANWVMANMVPEKVADA
ACT-23QLT57991.1_396EBOC      TWINVPKAEEEAHYAWGYRDGKAVRVSPGMLDAQAYGVKTNVQDMANWVMANMVPEKVADA
ACT-23QLV44058.1_369EBOC      TWINVPKAEEEAHYAWGYRDGKAVRVSPGMLDAQAYGVKTNVQDMANWVMANMVPEKVADA
ACT-115AXQ35263.1_2972EBCP    TWINVPKAEEEAHYAWGYRDGKAVRVSPGMLDAQAYGVKTNVQDMANWVMANMAPEKVADA
ACT-115QGR11187.1_384EBCP     TWINVPKAEEEAHYAWGYRDGKAVRVSPGMLDAQAYGVKTNVQDMANWVMANMAPEKVADA
ACT-115QIF26741.1_2106EBCP    TWINVPKAEEEAHYAWGYRDGKAVRVSPGMLDAQAYGVKTNVQDMANWVMANMAPEKVADA
ACT-115QIF32246.1_3030EBCP    TWINVPKAEEEAHYAWGYRDGKAVRVSPGMLDAQAYGVKTNVQDMANWVMANMAPEKVADA
ACT-115QOD35429.1_385EBCP     TWINVPKAEEEAHYAWGYRDGKAVRVSPGMLDAQAYGVKTNVQDMANWVMANMAPEKVADA
ACT-115QPD66621.1_385EBCP     TWINVPKAEEEAHYAWGYRDGKAVRVSPGMLDAQAYGVKTNVQDMANWVMANMAPEKVADA
ACT-115QSS35407.1_385EBCP     TWINVPKAEEEAHYAWGYRDGKAVRVSPGMLDAQAYGVKTNVQDMANWVMANMAPEKVADA
ACT-115URE88756.1_385EBCP     TWINVPKAEEEAHYAWGYRDGKAVRVSPGMLDAQAYGVKTNVQDMANWVMANMAPEKVADA
ACT-115USM81982.1_384EBCP     TWINVPKAEEEAHYAWGYRDGKAVRVSPGMLDAQAYGVKTNVQDMANWVMANMAPEKVADA
ACT-115AXQ35263.1_2972EBOC    TWINVPKAEEEAHYAWGYRDGKAVRVSPGMLDAQAYGVKTNVQDMANWVMANMAPEKVADA
ACT-115QGR11187.1_384EBOC     TWINVPKAEEEAHYAWGYRDGKAVRVSPGMLDAQAYGVKTNVQDMANWVMANMAPEKVADA
ACT-115QIF26741.1_2106EBOC    TWINVPKAEEEAHYAWGYRDGKAVRVSPGMLDAQAYGVKTNVQDMANWVMANMAPEKVADA
ACT-115QIF32246.1_3030EBOC    TWINVPKAEEEAHYAWGYRDGKAVRVSPGMLDAQAYGVKTNVQDMANWVMANMAPEKVADA
ACT-115QOD35429.1_385EBOC     TWINVPKAEEEAHYAWGYRDGKAVRVSPGMLDAQAYGVKTNVQDMANWVMANMAPEKVADA
ACT-115QPD66621.1_385EBOC     TWINVPKAEEEAHYAWGYRDGKAVRVSPGMLDAQAYGVKTNVQDMANWVMANMAPEKVADA
ACT-115QSS35407.1_385EBOC     TWINVPKAEEEAHYAWGYRDGKAVRVSPGMLDAQAYGVKTNVQDMANWVMANMAPEKVADA
ACT-115URE88756.1_385EBOC     TWINVPKAEEEAHYAWGYRDGKAVRVSPGMLDAQAYGVKTNVQDMANWVMANMAPEKVADA
ACT-115USM81982.1_384EBOC     TWINVPKAEEEAHYAWGYRDGKAVRVSPGMLDAQAYGVKTNVQDMANWVMANMAPEKVADA
ACT-25AVU48988.1_427EBCP      TWINVPKAEEEAHYAWGYRDGKAVRVSPGMLDAQAYGVKTNVQDMANWVMANMAPEKVADA
ACT-25UQQ46893.1_379EBCP      TWINVPKAEEEAHYAWGYRDGKAVRVSPGMLDAQAYGVKTNVQDMANWVMANMAPEKVADA
ACT-25WDO17280.1_310EBCP      TWINVPKAEEEAHYAWGYRDGKAVRVSPGMLDAQAYGVKTNVQDMANWVMANMAPEKVADA
ACT-25AVU48988.1_427EBOC      TWINVPKAEEEAHYAWGYRDGKAVRVSPGMLDAQAYGVKTNVQDMANWVMANMAPEKVADA
ACT-25UQQ46893.1_379EBOC      TWINVPKAEEEAHYAWGYRDGKAVRVSPGMLDAQAYGVKTNVQDMANWVMANMAPEKVADA
ACT-25WDO17280.1_310EBOC      TWINVPKAEEEAHYAWGYRDGKAVRVSPGMLDAQAYGVKTNVQDMANWVMANMAPEKVADA
ACT-25AOP89724.1_377EOC       TWINVPKAEEEAHYAWGYRDGKAVRVSPGMLDAQAYGVKTNVQDMANWVMANMAPEKVADA
ACT-45APR43249.1_2902EBCP     TWINVPKAEEEAHYAWGYRDGKAVRVSPGTLDAQAYGVKTNVQDMANWVMANMAPEKVADA
ACT-45ASA05885.1_3853EBCP     TWINVPKAEEEAHYAWGYRDGKAVRVSPGTLDAQAYGVKTNVQDMANWVMANMAPEKVADA
ACT-45ASB85359.1_3592EBCP     TWINVPKAEEEAHYAWGYRDGKAVRVSPGTLDAQAYGVKTNVQDMANWVMANMAPEKVADA
ACT-45AWR70714.1_4154EBCP     TWINVPKAEEEAHYAWGYRDGKAVRVSPGTLDAQAYGVKTNVQDMANWVMANMAPEKVADA
ACT-45AXM01511.1_4138EBCP     TWINVPKAEEEAHYAWGYRDGKAVRVSPGTLDAQAYGVKTNVQDMANWVMANMAPEKVADA
ACT-45AYU96034.1_2649EBCP     TWINVPKAEEEAHYAWGYRDGKAVRVSPGTLDAQAYGVKTNVQDMANWVMANMAPEKVADA
ACT-45QBH65043.1_3960EBCP     TWINVPKAEEEAHYAWGYRDGKAVRVSPGTLDAQAYGVKTNVQDMANWVMANMAPEKVADA
ACT-45QEL31100.1_1855EBCP     TWINVPKAEEEAHYAWGYRDGKAVRVSPGTLDAQAYGVKTNVQDMANWVMANMAPEKVADA
ACT-45ULQ22907.1_427EBCP      TWINVPKAEEEAHYAWGYRDGKAVRVSPGTLDAQAYGVKTNVQDMANWVMANMAPEKVADA
ACT-45ULQ25748.1_3478EBCP     TWINVPKAEEEAHYAWGYRDGKAVRVSPGTLDAQAYGVKTNVQDMANWVMANMAPEKVADA
ACT-45WCO72956.1_42EBCP       TWINVPKAEEEAHYAWGYRDGKAVRVSPGTLDAQAYGVKTNVQDMANWVMANMAPEKVADA
ACT-45APR43249.1_2902EBOC     TWINVPKAEEEAHYAWGYRDGKAVRVSPGTLDAQAYGVKTNVQDMANWVMANMAPEKVADA
ACT-45ASA05885.1_3853EBOC     TWINVPKAEEEAHYAWGYRDGKAVRVSPGTLDAQAYGVKTNVQDMANWVMANMAPEKVADA
ACT-45ASB85359.1_3592EBOC     TWINVPKAEEEAHYAWGYRDGKAVRVSPGTLDAQAYGVKTNVQDMANWVMANMAPEKVADA
ACT-45AWR70714.1_4154EBOC     TWINVPKAEEEAHYAWGYRDGKAVRVSPGTLDAQAYGVKTNVQDMANWVMANMAPEKVADA
ACT-45AXM01511.1_4138EBOC     TWINVPKAEEEAHYAWGYRDGKAVRVSPGTLDAQAYGVKTNVQDMANWVMANMAPEKVADA
ACT-45AYU96034.1_2649EBOC     TWINVPKAEEEAHYAWGYRDGKAVRVSPGTLDAQAYGVKTNVQDMANWVMANMAPEKVADA
ACT-45QBH65043.1_3960EBOC     TWINVPKAEEEAHYAWGYRDGKAVRVSPGTLDAQAYGVKTNVQDMANWVMANMAPEKVADA
ACT-45QEL31100.1_1855EBOC     TWINVPKAEEEAHYAWGYRDGKAVRVSPGTLDAQAYGVKTNVQDMANWVMANMAPEKVADA
ACT-45ULQ22907.1_427EBOC      TWINVPKAEEEAHYAWGYRDGKAVRVSPGTLDAQAYGVKTNVQDMANWVMANMAPEKVADA
ACT-45ULQ25748.1_3478EBOC     TWINVPKAEEEAHYAWGYRDGKAVRVSPGTLDAQAYGVKTNVQDMANWVMANMAPEKVADA
ACT-45WCO72956.1_42EBOC       TWINVPKAEEEAHYAWGYRDGKAVRVSPGTLDAQAYGVKTNVQDMANWVMANMAPEKVADA
ACT-C197QLA02472.1_2022EOC    TWINVPKAEEEAHYAWGYRDGKAVRVSPGMLDAQAYGVKTNVQDMANWVMANMAPEKVADA
ACT-64QMT06026.1_4255EBCP     TWIDVPKEDEAHYAWGYRDGKTVHVSPGMLDAEAYGVKTNVQDMASWVKANMNPAALPDS
ACT-64QMT06026.1_4255EBOC     TWIDVPKEDEAHYAWGYRDGKTVHVSPGMLDAEAYGVKTNVQDMASWVKANMNPAALPDS
ACT-C198QXA51435.1_2071EOC    TWIQVPKSEEQHYAWGYRDGKAVHVSPGMLDGEAYGVKSNAEDMASWVMVNMAPERVQAT
                      acc
```

```
                                  α9          β9   TT β10    β11          α10       α11   β12   β13
ACT-17BBW34093.1_49417]EBCP                                   310       320       330       340       350       360
ACT-17BBW34093.1_49417]EBCP   SLKQGIALAQSRYWRIGSMYQGLGWEMLNWPVEANTVVEGSDSKVALAPLPVAEVNPPAP
ACT-17BBW34093.1_49417]EBOC   SLKQGIALAQSRYWRIGSMYQGLGWEMLNWPVEANTVVEGSDSKVALAPLPVAEVNPPAP
7TI1_1|Chain                  SLKQGIALAQSRYWRIGSMYQGLGWEMLNWPVEANTVVEGSDSKVALAPLPVAEVNPPAP
ACT-37QPO56206.1_411EBCP      SLKQGIALAQSRYWRIGSMYQGLGWEMLNWPVEAKTVIEGSDNKVALAPLPVAEVNPPVP
ACT-37QPO65286.1_410EBCP      SLKQGIALAQSRYWRIGSMYQGLGWEMLNWPVEAKTVIEGSDNKVALAPLPVAEVNPPVP
ACT-37UKK25917.1_410EBCP      SLKQGIALAQSRYWRIGSMYQGLGWEMLNWPVEAKTVIEGSDNKVALAPLPVAEVNPPVP
ACT-37UKK30553.1_410EBCP      SLKQGIALAQSRYWRIGSMYQGLGWEMLNWPVEAKTVIEGSDNKVALAPLPVAEVNPPVP
ACT-37QPO56206.1_411EBOC      SLKQGIALAQSRYWRIGSMYQGLGWEMLNWPVEAKTVIEGSDNKVALAPLPVAEVNPPVP
ACT-37QPO65286.1_410EBOC      SLKQGIALAQSRYWRIGSMYQGLGWEMLNWPVEAKTVIEGSDNKVALAPLPVAEVNPPVP
ACT-37UKK25917.1_410EBOC      SLKQGIALAQSRYWRIGSMYQGLGWEMLNWPVEAKTVIEGSDNKVALAPLPVAEVNPPVP
ACT-37UKK30553.1_410EBOC      SLKQGIALAQSRYWRIGSMYQGLGWEMLNWPVEAKTVIEGSDNKVALAPLPVAEVNPPVP
ACT-65AXO42196.1_4001EBCP     SLKQGIALAQSRYWRIGSMYQGLGWEMLNWPVEANTVVEGSDSKVALAPLPVAEVNPPAP
ACT-65AXO42196.1_4001EBOC     SLKQGIALAQSRYWRIGSMYQGLGWEMLNWPVEANTVVEGSDSKVALAPLPVAEVNPPAP
ACT-65WBV31824.1_394EOC       SLKQGIALAQSRYWRIGSMYQGLGWEMLNWPVEANTVVEGSDSKVALAPLPVAEVNPPAP
ACT-17ARZ78489.1_1874EBCP     SLKQGIALAQSRYWRIGSMYQGLGWEMLNWPVEANTVVEGSDSKVALAPLPVAEVNPPAP
ACT-17ASQ75348.1_386EBCP      SLKQGIALAQSRYWRIGSMYQGLGWEMLNWPVEANTVVEGSDSKVALAPLPVAEVNPPAP
ACT-17AWV77932.1_4320EBCP     SLKQGIALAQSRYWRIGSMYQGLGWEMLNWPVEANTVVEGSDSKVALAPLPVAEVNPPAP
ACT-17QEQ46013.1_2619EBCP     SLKQGIALAQSRYWRIGSMYQGLGWEMLNWPVEANTVVEGSDSKVALAPLPVAEVNPPAP
ACT-17QFI20602.1_494EBCP      SLKQGIALAQSRYWRIGSMYQGLGWEMLNWPVEANTVVEGSDSKVALAPLPVAEVNPPAP
ACT-17QIE65424.1_3050EBCP     SLKQGIALAQSRYWRIGSMYQGLGWEMLNWPVEANTVVEGSDSKVALAPLPVAEVNPPAP
ACT-17QPD71520.1_416EBCP      SLKQGIALAQSRYWRIGSMYQGLGWEMLNWPVEANTVVEGSDSKVALAPLPVAEVNPPAP
ACT-17QPO45831.1_587EBCP      SLKQGIALAQSRYWRIGSMYQGLGWEMLNWPVEANTVVEGSDSKVALAPLPVAEVNPPAP
ACT-17QPO51098.1_586EBCP      SLKQGIALAQSRYWRIGSMYQGLGWEMLNWPVEANTVVEGSDSKVALAPLPVAEVNPPAP
ACT-17QPO69921.1_405EBCP      SLKQGIALAQSRYWRIGSMYQGLGWEMLNWPVEANTVVEGSDSKVALAPLPVAEVNPPAP
ACT-17QPO74759.1_405EBCP      SLKQGIALAQSRYWRIGSMYQGLGWEMLNWPVEANTVVEGSDSKVALAPLPVAEVNPPAP
ACT-17QPO85066.1_405EBCP      SLKQGIALAQSRYWRIGSMYQGLGWEMLNWPVEANTVVEGSDSKVALAPLPVAEVNPPAP
ACT-17QSB73424.1_1103EBCP     SLKQGIALAQSRYWRIGSMYQGLGWEMLNWPVEANTVVEGSDSKVALAPLPVAEVNPPAP
ACT-17QXY98988.1_1068EBCP     SLKQGIALAQSRYWRIGSMYQGLGWEMLNWPVEANTVVEGSDSKVALAPLPVAEVNPPAP
ACT-17UDV73425.1_430EBCP      SLKQGIALAQSRYWRIGSMYQGLGWEMLNWPVEANTVVEGSDSKVALAPLPVAEVNPPAP
ACT-17UDV69018.1_894EBCP      SLKQGIALAQSRYWRIGSMYQGLGWEMLNWPVEANTVVEGSDSKVALAPLPVAEVNPPAP
ACT-17UKK21116.1_588EBCP      SLKQGIALAQSRYWRIGSMYQGLGWEMLNWPVEANTVVEGSDSKVALAPLPVAEVNPPAP
ACT-17UOY44701.1_2566EBCP     SLKQGIALAQSRYWRIGSMYQGLGWEMLNWPVEANTVVEGSDSKVALAPLPVAEVNPPAP
ACT-17URL69109.1_2030EBCP     SLKQGIALAQSRYWRIGSMYQGLGWEMLNWPVEANTVVEGSDSKVALAPLPVAEVNPPAP
ACT-17UUQ70490.1_426EBCP      SLKQGIALAQSRYWRIGSMYQGLGWEMLNWPVEANTVVEGSDSKVALAPLPVAEVNPPAP
ACT-17UWA70738.1_427EBCP      SLKQGIALAQSRYWRIGSMYQGLGWEMLNWPVEANTVVEGSDSKVALAPLPVAEVNPPAP
ACT-17UWC98141.1_545EBCP      SLKQGIALAQSRYWRIGSMYQGLGWEMLNWPVEANTVVEGSDSKVALAPLPVAEVNPPAP
ACT-17UXC72711.1_448EBCP      SLKQGIALAQSRYWRIGSMYQGLGWEMLNWPVEANTVVEGSDSKVALAPLPVAEVNPPAP
ACT-17UXC77377.1_448EBCP      SLKQGIALAQSRYWRIGSMYQGLGWEMLNWPVEANTVVEGSDSKVALAPLPVAEVNPPAP
ACT-17UXC82023.1_448EBCP      SLKQGIALAQSRYWRIGSMYQGLGWEMLNWPVEANTVVEGSDSKVALAPLPVAEVNPPAP
ACT-17UZE41799.1_406EBCP      SLKQGIALAQSRYWRIGSMYQGLGWEMLNWPVEANTVVEGSDSKVALAPLPVAEVNPPAP
ACT-17UZH49391.1_396EBCP      SLKQGIALAQSRYWRIGSMYQGLGWEMLNWPVEANTVVEGSDSKVALAPLPVAEVNPPAP
ACT-17WAZ63377.1_405EBCP      SLKQGIALAQSRYWRIGSMYQGLGWEMLNWPVEANTVVEGSDSKVALAPLPVAEVNPPAP
ACT-17WDU86092.1_3769EBCP     SLKQGIALAQSRYWRIGSMYQGLGWEMLNWPVEANTVVEGSDSKVALAPLPVAEVNPPAP
ACT-17WDW12975.1_3764EBCP     SLKQGIALAQSRYWRIGSMYQGLGWEMLNWPVEANTVVEGSDSKVALAPLPVAEVNPPAP
ACT-17WGA71117.1_396EBCP      SLKQGIALAQSRYWRIGSMYQGLGWEMLNWPVEANTVVEGSDSKVALAPLPVAEVNPPAP
ACT-17WGA75603.1_396EBCP      SLKQGIALAQSRYWRIGSMYQGLGWEMLNWPVEANTVVEGSDSKVALAPLPVAEVNPPAP
ACT-17WGZ57121.1_2667EBCP     SLKQGIALAQSRYWRIGSMYQGLGWEMLNWPVEANTVVEGSDSKVALAPLPVAEVNPPAP
ACT-17ARZ78489.1_1874EBOC     SLKQGIALAQSRYWRIGSMYQGLGWEMLNWPVEANTVVEGSDSKVALAPLPVAEVNPPAP
ACT-17ASQ75348.1_386EBOC      SLKQGIALAQSRYWRIGSMYQGLGWEMLNWPVEANTVVEGSDSKVALAPLPVAEVNPPAP
ACT-17AWV77932.1_4320EBOC     SLKQGIALAQSRYWRIGSMYQGLGWEMLNWPVEANTVVEGSDSKVALAPLPVAEVNPPAP
ACT-17QEQ46013.1_2619EBOC     SLKQGIALAQSRYWRIGSMYQGLGWEMLNWPVEANTVVEGSDSKVALAPLPVAEVNPPAP
ACT-17QFI20602.1_494EBOC      SLKQGIALAQSRYWRIGSMYQGLGWEMLNWPVEANTVVEGSDSKVALAPLPVAEVNPPAP
ACT-17QIE65424.1_3050EBOC     SLKQGIALAQSRYWRIGSMYQGLGWEMLNWPVEANTVVEGSDSKVALAPLPVAEVNPPAP
ACT-17QPD71520.1_416EBOC      SLKQGIALAQSRYWRIGSMYQGLGWEMLNWPVEANTVVEGSDSKVALAPLPVAEVNPPAP
ACT-17QPO45831.1_587EBOC      SLKQGIALAQSRYWRIGSMYQGLGWEMLNWPVEANTVVEGSDSKVALAPLPVAEVNPPAP
ACT-17QPO51098.1_586EBOC      SLKQGIALAQSRYWRIGSMYQGLGWEMLNWPVEANTVVEGSDSKVALAPLPVAEVNPPAP
ACT-17QPO69921.1_405EBOC      SLKQGIALAQSRYWRIGSMYQGLGWEMLNWPVEANTVVEGSDSKVALAPLPVAEVNPPAP
ACT-17QPO74759.1_405EBOC      SLKQGIALAQSRYWRIGSMYQGLGWEMLNWPVEANTVVEGSDSKVALAPLPVAEVNPPAP
ACT-17QPO85066.1_405EBOC      SLKQGIALAQSRYWRIGSMYQGLGWEMLNWPVEANTVVEGSDSKVALAPLPVAEVNPPAP
ACT-17QSB73424.1_1103EBOC     SLKQGIALAQSRYWRIGSMYQGLGWEMLNWPVEANTVVEGSDSKVALAPLPVAEVNPPAP
ACT-17QXY98988.1_1068EBOC     SLKQGIALAQSRYWRIGSMYQGLGWEMLNWPVEANTVVEGSDSKVALAPLPVAEVNPPAP
ACT-17UDV73425.1_430EBOC      SLKQGIALAQSRYWRIGSMYQGLGWEMLNWPVEANTVVEGSDSKVALAPLPVAEVNPPAP
ACT-17UDV69018.1_894EBOC      SLKQGIALAQSRYWRIGSMYQGLGWEMLNWPVEANTVVEGSDSKVALAPLPVAEVNPPAP
ACT-17UKK21116.1_588EBOC      SLKQGIALAQSRYWRIGSMYQGLGWEMLNWPVEANTVVEGSDSKVALAPLPVAEVNPPAP
ACT-17UOY44701.1_2566EBOC     SLKQGIALAQSRYWRIGSMYQGLGWEMLNWPVEANTVVEGSDSKVALAPLPVAEVNPPAP
ACT-17URL69109.1_2030EBOC     SLKQGIALAQSRYWRIGSMYQGLGWEMLNWPVEANTVVEGSDSKVALAPLPVAEVNPPAP
ACT-17UUQ70490.1_426EBOC      SLKQGIALAQSRYWRIGSMYQGLGWEMLNWPVEANTVVEGSDSKVALAPLPVAEVNPPAP
ACT-17UWA70738.1_427EBOC      SLKQGIALAQSRYWRIGSMYQGLGWEMLNWPVEANTVVEGSDSKVALAPLPVAEVNPPAP
ACT-17UWC98141.1_545EBOC      SLKQGIALAQSRYWRIGSMYQGLGWEMLNWPVEANTVVEGSDSKVALAPLPVAEVNPPAP
ACT-17UXC72711.1_448EBOC      SLKQGIALAQSRYWRIGSMYQGLGWEMLNWPVEANTVVEGSDSKVALAPLPVAEVNPPAP
ACT-17UXC77377.1_448EBOC      SLKQGIALAQSRYWRIGSMYQGLGWEMLNWPVEANTVVEGSDSKVALAPLPVAEVNPPAP
ACT-17UXC82023.1_448EBOC      SLKQGIALAQSRYWRIGSMYQGLGWEMLNWPVEANTVVEGSDSKVALAPLPVAEVNPPAP
ACT-17UZE41799.1_406EBOC      SLKQGIALAQSRYWRIGSMYQGLGWEMLNWPVEANTVVEGSDSKVALAPLPVAEVNPPAP
ACT-17UZH49391.1_396EBOC      SLKQGIALAQSRYWRIGSMYQGLGWEMLNWPVEANTVVEGSDSKVALAPLPVAEVNPPAP
ACT-17WAZ63377.1_405EBOC      SLKQGIALAQSRYWRIGSMYQGLGWEMLNWPVEANTVVEGSDSKVALAPLPVAEVNPPAP
ACT-17WDU86092.1_3769EBOC     SLKQGIALAQSRYWRIGSMYQGLGWEMLNWPVEANTVVEGSDSKVALAPLPVAEVNPPAP
ACT-17WDW12975.1_3764EBOC     SLKQGIALAQSRYWRIGSMYQGLGWEMLNWPVEANTVVEGSDSKVALAPLPVAEVNPPAP
ACT-17WGA71117.1_396EBOC      SLKQGIALAQSRYWRIGSMYQGLGWEMLNWPVEANTVVEGSDSKVALAPLPVAEVNPPAP
ACT-17WGA75603.1_396EBOC      SLKQGIALAQSRYWRIGSMYQGLGWEMLNWPVEANTVVEGSDSKVALAPLPVAEVNPPAP
ACT-17WGZ57121.1_2667EBOC     SLKQGIALAQSRYWRIGSMYQGLGWEMLNWPVEANTVVEGSDSKVALAPLPVAEVNPPAP
ACT-17QEI60759.1_389EOC       SLKQGIALAQSRYWRIGSMYQGLGWEMLNWPVEANTVVEGSDSKVALAPLPVAEVNPPAP
ACT-17QEI70010.1_389EOC       SLKQGIALAQSRYWRIGSMYQGLGWEMLNWPVEANTVVEGSDSKVALAPLPVAEVNPPAP
ACT-17QEU13386.1_612EOC       SLKQGIALAQSRYWRIGSMYQGLGWEMLNWPVEANTVVEGSDSKVALAPLPVAEVNPPAP
ACT-17QHI59365.1_3674EOC      SLKQGIALAQSRYWRIGSMYQGLGWEMLNWPVEANTVVEGSDSKVALAPLPVAEVNPPAP
ACT-17UZQ58984.1_410EOC       SLKQGIALAQSRYWRIGSMYQGLGWEMLNWPVEANTVVEGSDSKVALAPLPVAEVNPPAP
ACT-40AWS79793.1_3058EBCP     SLKQGIALAQSRYWRIGSMYQGLGWEMLNWPVEANTVVEGSDSKVALAPLPVAEVNPPAP
ACT-40AXO44745.1_1607EBCP     SLKQGIALAQSRYWRIGSMYQGLGWEMLNWPVEANTVVEGSDSKVALAPLPVAEVNPPAP
ACT-40UXI63958.1_1746EBCP     SLKQGIALAQSRYWRIGSMYQGLGWEMLNWPVEANTVVEGSDSKVALAPLPVAEVNPPAP
ACT-40WDC52451.1_4284EBCP     SLKQGIALAQSRYWRIGSMYQGLGWEMLNWPVEANTVVEGSDSKVALAPLPVAEVNPPAP
ACT-40AWS79793.1_3058EBOC     SLKQGIALAQSRYWRIGSMYQGLGWEMLNWPVEANTVVEGSDSKVALAPLPVAEVNPPAP
ACT-40AXO44745.1_1607EBOC     SLKQGIALAQSRYWRIGSMYQGLGWEMLNWPVEANTVVEGSDSKVALAPLPVAEVNPPAP
ACT-40UXI63958.1_1746EBOC     SLKQGIALAQSRYWRIGSMYQGLGWEMLNWPVEANTVVEGSDSKVALAPLPVAEVNPPAP
ACT-40WDC52451.1_4284EBOC     SLKQGIALAQSRYWRIGSMYQGLGWEMLNWPVEANTVVEGSDSKVALAPLPVAEVNPPAP
ACT-44UWA09346.1_3724EBCP     SLKQGIALAQSRYWRIGSMYQGLGWEMLNWPVEANTVVEGSDSKVALAPLPVAEVNPPAP
ACT-44UWA09346.1_3724EBOC     SLKQGIALAQSRYWRIGSMYQGLGWEMLNWPVEANTVVEGSDSKVALAPLPVAEVNPPAP
ACT-90QFH83806.1_450EBCP      SLKQGIALAQSRYWRIGSMYQGLGWEMLNWPVEANTVIEGSDSKVALAPLPVAEVNPPAP
ACT-90QLO92194.1_500EBCP      SLKQGIALAQSRYWRIGSMYQGLGWEMLNWPVEANTVIEGSDSKVALAPLPVAEVNPPAP
ACT-90QLP01381.1_466EBCP      SLKQGIALAQSRYWRIGSMYQGLGWEMLNWPVEANTVIEGSDSKVALAPLPVAEVNPPAP
ACT-90QFH83806.1_450EBOC      SLKQGIALAQSRYWRIGSMYQGLGWEMLNWPVEANTVIEGSDSKVALAPLPVAEVNPPAP
ACT-90QLO92194.1_500EBOC      SLKQGIALAQSRYWRIGSMYQGLGWEMLNWPVEANTVIEGSDSKVALAPLPVAEVNPPAP
ACT-90QLP01381.1_466EBOC      SLKQGIALAQSRYWRIGSMYQGLGWEMLNWPVEANTVIEGSDSKVALAPLPVAEVNPPAP
ACT-24ASB73507.1_1185EBCP     SLKQGISLAQSRYWRIGSMYQGLGWEMLNWPVEANTVIDGSDSKVALAPLPVAEVNPPAP
ACT-24AVU18445.1_378EBCP      SLKQGISLAQSRYWRIGSMYQGLGWEMLNWPVEANTVIDGSDSKVALAPLPVAEVNPPAP
```

ACT-24QHC75881.1_374EBCP	SLKQGISLAQSRYWRIGSMYQGLGWEMLNWPVEANTVIDGSDSKVALAPLPVAEVNPPAP
ACT-24QLP84500.1_370EBCP	SLKQGISLAQSRYWRIGSMYQGLGWEMLNWPVEANTVIDGSDSKVALAPLPVAEVNPPAP
ACT-24QSH03071.1_370EBCP	SLKQGISLAQSRYWRIGSMYQGLGWEMLNWPVEANTVIDGSDSKVALAPLPVAEVNPPAP
ACT-24QSG93844.1_370EBCP	SLKQGISLAQSRYWRIGSMYQGLGWEMLNWPVEANTVIDGSDSKVALAPLPVAEVNPPAP
ACT-24QSG98466.1_370EBCP	SLKQGISLAQSRYWRIGSMYQGLGWEMLNWPVEANTVIDGSDSKVALAPLPVAEVNPPAP
ACT-24QSH12187.1_370EBCP	SLKQGISLAQSRYWRIGSMYQGLGWEMLNWPVEANTVIDGSDSKVALAPLPVAEVNPPAP
ACT-24QSH16801.1_370EBCP	SLKQGISLAQSRYWRIGSMYQGLGWEMLNWPVEANTVIDGSDSKVALAPLPVAEVNPPAP
ACT-24QSH07573.1_370EBCP	SLKQGISLAQSRYWRIGSMYQGLGWEMLNWPVEANTVIDGSDSKVALAPLPVAEVNPPAP
ACT-24QSH21409.1_370EBCP	SLKQGISLAQSRYWRIGSMYQGLGWEMLNWPVEANTVIDGSDSKVALAPLPVAEVNPPAP
ACT-24QSH26023.1_370EBCP	SLKQGISLAQSRYWRIGSMYQGLGWEMLNWPVEANTVIDGSDSKVALAPLPVAEVNPPAP
ACT-24QSH30639.1_370EBCP	SLKQGISLAQSRYWRIGSMYQGLGWEMLNWPVEANTVIDGSDSKVALAPLPVAEVNPPAP
ACT-24UIL48372.1_372EBCP	SLKQGISLAQSRYWRIGSMYQGLGWEMLNWPVEANTVIDGSDSKVALAPLPVAEVNPPAP
ACT-24UIM14687.1_372EBCP	SLKQGISLAQSRYWRIGSMYQGLGWEMLNWPVEANTVIDGSDSKVALAPLPVAEVNPPAP
ACT-24UXA74146.1_371EBCP	SLKQGISLAQSRYWRIGSMYQGLGWEMLNWPVEANTVIDGSDSKVALAPLPVAEVNPPAP
ACT-24WDY48721.1_372EBCP	SLKQGISLAQSRYWRIGSMYQGLGWEMLNWPVEANTVIDGSDSKVALAPLPVAEVNPPAP
ACT-24ASB73507.1_1185EBOC	SLKQGISLAQSRYWRIGSMYQGLGWEMLNWPVEANTVIDGSDSKVALAPLPVAEVNPPAP
ACT-24AVU18445.1_378EBOC	SLKQGISLAQSRYWRIGSMYQGLGWEMLNWPVEANTVIDGSDSKVALAPLPVAEVNPPAP
ACT-24QHC75881.1_374EBOC	SLKQGISLAQSRYWRIGSMYQGLGWEMLNWPVEANTVIDGSDSKVALAPLPVAEVNPPAP
ACT-24QLP84500.1_370EBOC	SLKQGISLAQSRYWRIGSMYQGLGWEMLNWPVEANTVIDGSDSKVALAPLPVAEVNPPAP
ACT-24QSH03071.1_370EBOC	SLKQGISLAQSRYWRIGSMYQGLGWEMLNWPVEANTVIDGSDSKVALAPLPVAEVNPPAP
ACT-24QSG93844.1_370EBOC	SLKQGISLAQSRYWRIGSMYQGLGWEMLNWPVEANTVIDGSDSKVALAPLPVAEVNPPAP
ACT-24QSG98466.1_370EBOC	SLKQGISLAQSRYWRIGSMYQGLGWEMLNWPVEANTVIDGSDSKVALAPLPVAEVNPPAP
ACT-24QSH12187.1_370EBOC	SLKQGISLAQSRYWRIGSMYQGLGWEMLNWPVEANTVIDGSDSKVALAPLPVAEVNPPAP
ACT-24QSH16801.1_370EBOC	SLKQGISLAQSRYWRIGSMYQGLGWEMLNWPVEANTVIDGSDSKVALAPLPVAEVNPPAP
ACT-24QSH07573.1_370EBOC	SLKQGISLAQSRYWRIGSMYQGLGWEMLNWPVEANTVIDGSDSKVALAPLPVAEVNPPAP
ACT-24QSH21409.1_370EBOC	SLKQGISLAQSRYWRIGSMYQGLGWEMLNWPVEANTVIDGSDSKVALAPLPVAEVNPPAP
ACT-24QSH26023.1_370EBOC	SLKQGISLAQSRYWRIGSMYQGLGWEMLNWPVEANTVIDGSDSKVALAPLPVAEVNPPAP
ACT-24QSH30639.1_370EBOC	SLKQGISLAQSRYWRIGSMYQGLGWEMLNWPVEANTVIDGSDSKVALAPLPVAEVNPPAP
ACT-24UIL48372.1_372EBOC	SLKQGISLAQSRYWRIGSMYQGLGWEMLNWPVEANTVIDGSDSKVALAPLPVAEVNPPAP
ACT-24UIM14687.1_372EBOC	SLKQGISLAQSRYWRIGSMYQGLGWEMLNWPVEANTVIDGSDSKVALAPLPVAEVNPPAP
ACT-24UXA74146.1_371EBOC	SLKQGISLAQSRYWRIGSMYQGLGWEMLNWPVEANTVIDGSDSKVALAPLPVAEVNPPAP
ACT-24WDY48721.1_372EBOC	SLKQGISLAQSRYWRIGSMYQGLGWEMLNWPVEANTVIDGSDSKVALAPLPVAEVNPPAP
ACT-89AVE73204.1_2453EBCP	SLKQGIALAQSRYWRIGSMYQGLGWEMLNWPVEANTVVEGSDSKVALAPLPVAEVNPPAP
ACT-89AVE73204.1_2453EBOC	SLKQGIALAQSRYWRIGSMYQGLGWEMLNWPVEANTVVEGSDSKVALAPLPVAEVNPPAP
ACT-56ASO99603.1_1270EBCP	SLKQGIALAQSRYWRIGSMYQGLGWEMLNWPVEANTVVEGSDSKVALAPLPVVEVNPPAP
ACT-56QDQ75241.1_392EBCP	SLKQGIALAQSRYWRIGSMYQGLGWEMLNWPVEANTVVEGSDSKVALAPLPVVEVNPPAP
ACT-56QFH98918.1_395EBCP	SLKQGIALAQSRYWRIGSMYQGLGWEMLNWPVEANTVVEGSDSKVALAPLPVVEVNPPAP
ACT-56QFH34334.1_395EBCP	SLKQGIALAQSRYWRIGSMYQGLGWEMLNWPVEANTVVEGSDSKVALAPLPVVEVNPPAP
ACT-56QFI32707.1_2233EBCP	SLKQGIALAQSRYWRIGSMYQGLGWEMLNWPVEANTVVEGSDSKVALAPLPVVEVNPPAP
ACT-56QMI65633.1_4184EBCP	SLKQGIALAQSRYWRIGSMYQGLGWEMLNWPVEANTVVEGSDSKVALAPLPVVEVNPPAP
ACT-56QMI71192.1_422EBCP	SLKQGIALAQSRYWRIGSMYQGLGWEMLNWPVEANTVVEGSDSKVALAPLPVVEVNPPAP
ACT-56UDV17856.1_8EBCP	SLKQGIALAQSRYWRIGSMYQGLGWEMLNWPVEANTVVEGSDSKVALAPLPVVEVNPPAP
ACT-56UDV39132.1_1376EBCP	SLKQGIALAQSRYWRIGSMYQGLGWEMLNWPVEANTVVEGSDSKVALAPLPVVEVNPPAP
ACT-56UWB10427.1_430EBCP	SLKQGIALAQSRYWRIGSMYQGLGWEMLNWPVEANTVVEGSDSKVALAPLPVVEVNPPAP
ACT-56UWC18315.1_415EBCP	SLKQGIALAQSRYWRIGSMYQGLGWEMLNWPVEANTVVEGSDSKVALAPLPVVEVNPPAP
ACT-56UWC23093.1_417EBCP	SLKQGIALAQSRYWRIGSMYQGLGWEMLNWPVEANTVVEGSDSKVALAPLPVVEVNPPAP
ACT-56WBT12607.1_393EBCP	SLKQGIALAQSRYWRIGSMYQGLGWEMLNWPVEANTVVEGSDSKVALAPLPVVEVNPPAP
ACT-56WBT28831.1_393EBCP	SLKQGIALAQSRYWRIGSMYQGLGWEMLNWPVEANTVVEGSDSKVALAPLPVVEVNPPAP
ACT-56WGB34617.1_392EBCP	SLKQGIALAQSRYWRIGSMYQGLGWEMLNWPVEANTVVEGSDSKVALAPLPVVEVNPPAP
ACT-56WGU03466.1_393EBCP	SLKQGIALAQSRYWRIGSMYQGLGWEMLNWPVEANTVVEGSDSKVALAPLPVVEVNPPAP
ACT-56ASO99603.1_1270EBOC	SLKQGIALAQSRYWRIGSMYQGLGWEMLNWPVEANTVVEGSDSKVALAPLPVVEVNPPAP
ACT-56QDQ75241.1_392EBOC	SLKQGIALAQSRYWRIGSMYQGLGWEMLNWPVEANTVVEGSDSKVALAPLPVVEVNPPAP
ACT-56QFH98918.1_395EBOC	SLKQGIALAQSRYWRIGSMYQGLGWEMLNWPVEANTVVEGSDSKVALAPLPVVEVNPPAP
ACT-56QFH34334.1_395EBOC	SLKQGIALAQSRYWRIGSMYQGLGWEMLNWPVEANTVVEGSDSKVALAPLPVVEVNPPAP
ACT-56QFI32707.1_2233EBOC	SLKQGIALAQSRYWRIGSMYQGLGWEMLNWPVEANTVVEGSDSKVALAPLPVVEVNPPAP
ACT-56QMI65633.1_4184EBOC	SLKQGIALAQSRYWRIGSMYQGLGWEMLNWPVEANTVVEGSDSKVALAPLPVVEVNPPAP
ACT-56QMI71192.1_422EBOC	SLKQGIALAQSRYWRIGSMYQGLGWEMLNWPVEANTVVEGSDSKVALAPLPVVEVNPPAP
ACT-56UDV17856.1_8EBOC	SLKQGIALAQSRYWRIGSMYQGLGWEMLNWPVEANTVVEGSDSKVALAPLPVVEVNPPAP
ACT-56UDV39132.1_1376EBOC	SLKQGIALAQSRYWRIGSMYQGLGWEMLNWPVEANTVVEGSDSKVALAPLPVVEVNPPAP
ACT-56UWB10427.1_430EBOC	SLKQGIALAQSRYWRIGSMYQGLGWEMLNWPVEANTVVEGSDSKVALAPLPVVEVNPPAP
ACT-56UWC18315.1_415EBOC	SLKQGIALAQSRYWRIGSMYQGLGWEMLNWPVEANTVVEGSDSKVALAPLPVVEVNPPAP
ACT-56UWC23093.1_417EBOC	SLKQGIALAQSRYWRIGSMYQGLGWEMLNWPVEANTVVEGSDSKVALAPLPVVEVNPPAP
ACT-56WBT12607.1_393EBOC	SLKQGIALAQSRYWRIGSMYQGLGWEMLNWPVEANTVVEGSDSKVALAPLPVVEVNPPAP
ACT-56WBT28831.1_393EBOC	SLKQGIALAQSRYWRIGSMYQGLGWEMLNWPVEANTVVEGSDSKVALAPLPVVEVNPPAP
ACT-56WGB34617.1_392EBOC	SLKQGIALAQSRYWRIGSMYQGLGWEMLNWPVEANTVVEGSDSKVALAPLPVVEVNPPAP
ACT-56WGU03466.1_393EBOC	SLKQGIALAQSRYWRIGSMYQGLGWEMLNWPVEANTVVEGSDSKVALAPLPVVEVNPPAP
ACT-56AOP76464.1_392EOC	SLKQGIALAQSRYWRIGSMYQGLGWEMLNWPVEANTVVEGSDSKVALAPLPVVEVNPPAP
ACT-56QXA77046.1_2672EOC	SLKQGIALAQSRYWRIGSMYQGLGWEMLNWPVEANTVVEGSDSKVALAPLPVVEVNPPAP
ACT-56WFP29817.1_397EOC	SLKQGIALAQSRYWRIGSMYQGLGWEMLNWPVEANTVVEGSDSKVALAPLPVVEVNPPAP
ACT-27AXO49795.1_1610EBCP	SLKQGIALAQSRYWRIGSMYQGLGWEMLNWPVEANTVVEGSDSKVALAPLPVVEVNPPAP
ACT-27QHO79915.1_457EBCP	SLKQGIALAQSRYWRIGSMYQGLGWEMLNWPVEANTVVEGSDSKVALAPLPVVEVNPPAP
ACT-27QHO97012.1_458EBCP	SLKQGIALAQSRYWRIGSMYQGLGWEMLNWPVEANTVVEGSDSKVALAPLPVVEVNPPAP
ACT-27QQH03712.1_3802EBCP	SLKQGIALAQSRYWRIGSMYQGLGWEMLNWPVEANTVVEGSDSKVALAPLPVVEVNPPAP
ACT-27QQH08784.1_3802EBCP	SLKQGIALAQSRYWRIGSMYQGLGWEMLNWPVEANTVVEGSDSKVALAPLPVVEVNPPAP
ACT-27UVZ91365.1_388EBCP	SLKQGIALAQSRYWRIGSMYQGLGWEMLNWPVEANTVVEGSDSKVALAPLPVVEVNPPAP
ACT-27AXO49795.1_1610EBOC	SLKQGIALAQSRYWRIGSMYQGLGWEMLNWPVEANTVVEGSDSKVALAPLPVVEVNPPAP
ACT-27QHO79915.1_457EBOC	SLKQGIALAQSRYWRIGSMYQGLGWEMLNWPVEANTVVEGSDSKVALAPLPVVEVNPPAP
ACT-27QHO97012.1_458EBOC	SLKQGIALAQSRYWRIGSMYQGLGWEMLNWPVEANTVVEGSDSKVALAPLPVVEVNPPAP
ACT-27QQH03712.1_3802EBOC	SLKQGIALAQSRYWRIGSMYQGLGWEMLNWPVEANTVVEGSDSKVALAPLPVVEVNPPAP
ACT-27QQH08784.1_3802EBOC	SLKQGIALAQSRYWRIGSMYQGLGWEMLNWPVEANTVVEGSDSKVALAPLPVVEVNPPAP
ACT-27UVZ91365.1_388EBOC	SLKQGIALAQSRYWRIGSMYQGLGWEMLNWPVEANTVVEGSDSKVALAPLPVVEVNPPAP
ACT-102QEO03155.1_4213EBCP	TLKQGIALAQSRYWRVGAMYQGLGWEMLNWPVEAQTVVEGSDNKVALAPLPVAEVNPPAP
ACT-102BBV79841.1_51928]EBCP	TLKQGIALAQSRYWRVGAMYQGLGWEMLNWPVEAQTVVEGSDNKVALAPLPVAEVNPPAP
ACT-102QZS47739.1_401EBCP	TLKQGIALAQSRYWRVGAMYQGLGWEMLNWPVEAQTVVEGSDNKVALAPLPVAEVNPPAP
ACT-102QEO03155.1_4213EBOC	TLKQGIALAQSRYWRVGAMYQGLGWEMLNWPVEAQTVVEGSDNKVALAPLPVAEVNPPAP
ACT-102BBV79841.1_51928]EBOC	TLKQGIALAQSRYWRVGAMYQGLGWEMLNWPVEAQTVVEGSDNKVALAPLPVAEVNPPAP
ACT-102QZS47739.1_401EBOC	TLKQGIALAQSRYWRVGAMYQGLGWEMLNWPVEAQTVVEGSDNKVALAPLPVAEVNPPAP
ACT-55QLP71015.1_413EBCP	TLKQGIALAQSRYWRIGSMYQGLGWEMLNWPVEANTVVGGSDSKVALAPLPVAEVNPPAP
ACT-55QLP80034.1_414EBCP	TLKQGIALAQSRYWRIGSMYQGLGWEMLNWPVEANTVVGGSDSKVALAPLPVAEVNPPAP
ACT-55QLS08818.1_415EBCP	TLKQGIALAQSRYWRIGSMYQGLGWEMLNWPVEANTVVGGSDSKVALAPLPVAEVNPPAP
ACT-55QLT25377.1_3621EBCP	TLKQGIALAQSRYWRIGSMYQGLGWEMLNWPVEANTVVGGSDSKVALAPLPVAEVNPPAP
ACT-55QLV12510.1_4006EBCP	TLKQGIALAQSRYWRIGSMYQGLGWEMLNWPVEANTVVGGSDSKVALAPLPVAEVNPPAP
ACT-55QLW04180.1_4182EBCP	TLKQGIALAQSRYWRIGSMYQGLGWEMLNWPVEANTVVGGSDSKVALAPLPVAEVNPPAP
ACT-55QLV08026.1_4006EBCP	TLKQGIALAQSRYWRIGSMYQGLGWEMLNWPVEANTVVGGSDSKVALAPLPVAEVNPPAP
ACT-55QLV96171.1_421EBCP	TLKQGIALAQSRYWRIGSMYQGLGWEMLNWPVEANTVVGGSDSKVALAPLPVAEVNPPAP
ACT-55QLW44293.1_405EBCP	TLKQGIALAQSRYWRIGSMYQGLGWEMLNWPVEANTVVGGSDSKVALAPLPVAEVNPPAP
ACT-55UBH32480.1_675EBCP	TLKQGIALAQSRYWRIGSMYQGLGWEMLNWPVEANTVVGGSDSKVALAPLPVAEVNPPAP
ACT-55UDV61223.1_2251EBCP	TLKQGIALAQSRYWRIGSMYQGLGWEMLNWPVEANTVVGGSDSKVALAPLPVAEVNPPAP
ACT-55QLP71015.1_413EBOC	TLKQGIALAQSRYWRIGSMYQGLGWEMLNWPVEANTVVGGSDSKVALAPLPVAEVNPPAP
ACT-55QLP80034.1_414EBOC	TLKQGIALAQSRYWRIGSMYQGLGWEMLNWPVEANTVVGGSDSKVALAPLPVAEVNPPAP
ACT-55QLS08818.1_415EBOC	TLKQGIALAQSRYWRIGSMYQGLGWEMLNWPVEANTVVGGSDSKVALAPLPVAEVNPPAP
ACT-55QLT25377.1_3621EBOC	TLKQGIALAQSRYWRIGSMYQGLGWEMLNWPVEANTVVGGSDSKVALAPLPVAEVNPPAP
ACT-55QLV12510.1_4006EBOC	TLKQGIALAQSRYWRIGSMYQGLGWEMLNWPVEANTVVGGSDSKVALAPLPVAEVNPPAP

```
ACT-55QLW04180.1_4182EBOC     TLKQGIALAQSRYWRIGSMYQGLGWEMLNWPVEANTVVGGSDSKVALAPLPVAEVNPPAP
ACT-55QLV08026.1_4006EBOC     TLKQGIALAQSRYWRIGSMYQGLGWEMLNWPVEANTVVGGSDSKVALAPLPVAEVNPPAP
ACT-55QLV96171.1_421EBOC      TLKQGIALAQSRYWRIGSMYQGLGWEMLNWPVEANTVVGGSDSKVALAPLPVAEVNPPAP
ACT-55QLW44293.1_405EBOC      TLKQGIALAQSRYWRIGSMYQGLGWEMLNWPVEANTVVGGSDSKVALAPLPVAEVNPPAP
ACT-55UBH32480.1_675EBOC      TLKQGIALAQSRYWRIGSMYQGLGWEMLNWPVEANTVVGGSDSKVALAPLPVAEVNPPAP
ACT-55UDV61223.1_2251EBOC     TLKQGIALAQSRYWRIGSMYQGLGWEMLNWPVEANTVVGGSDSKVALAPLPVAEVNPPAP
ACT-55AOP80857.1_409EOC       TLKQGIALAQSRYWRIGSMYQGLGWEMLNWPVEANTVVGGSDSKVALAPLPVAEVNPPAP
ACT-51BBW20048.1_38351]EBCP   TLKQGIALAQSRYWRVGAMYQGLGWEMLNWPVEAKTVVEGSDNKVALAPLPVAEVNPPAP
ACT-51BBW20048.1_38351]EBOC   TLKQGIALAQSRYWRVGAMYQGLGWEMLNWPVEAKTVVEGSDNKVALAPLPVAEVNPPAP
ACT-28URE95606.1_351EBCP      TLKQGIALAQSRYWRVGAMYQGLGWEMLNWPVEAKTVVEGSDNKVALAPLPVAEVNPPAP
ACT-28URE95606.1_351EBOC      TLKQGIALAQSRYWRVGAMYQGLGWEMLNWPVEAKTVVEGSDNKVALAPLPVAEVNPPAP
ACT-87AYL04658.1_1249EBCP     TLKQGIALAQSRYWRVGAMYQGLGWEMLNWPVEAKTVVEGSDNKVALAPLPVAEVNPPAP
ACT-87QIP18425.1_352EBCP      TLKQGIALAQSRYWRVGAMYQGLGWEMLNWPVEAKTVVEGSDNKVALAPLPVAEVNPPAP
ACT-87AYL04658.1_1249EBOC     TLKQGIALAQSRYWRVGAMYQGLGWEMLNWPVEAKTVVEGSDNKVALAPLPVAEVNPPAP
ACT-87QIP18425.1_352EBOC      TLKQGIALAQSRYWRVGAMYQGLGWEMLNWPVEAKTVVEGSDNKVALAPLPVAEVNPPAP
ACT-75AWX01586.1_1447EBCP     SLKQGIALAQSRYWRIGSMYQGLGWEMLNWPVEANTVVEGSDSKVALAPLPVAEVNPPAP
ACT-75AWX01586.1_1447EBOC     SLKQGIALAQSRYWRIGSMYQGLGWEMLNWPVEANTVVEGSDSKVALAPLPVAEVNPPAP
ACT-103URL27837.1_342EOC      TLKQGIALAQSRYWRVGAMYQGLGWEMLNWPVEAKTVVEGSDNKVALAPLPVAEVNPPVP
ACT-99UXJ64669.1_346EBCP      TLKQGIALAQSRYWRVGAMYQGLGWEMLNWPVESKTVVEGSDNKVALAPLPVAEVNPPAP
ACT-99UXJ64669.1_346EBOC      TLKQGIALAQSRYWRVGAMYQGLGWEMLNWPVESKTVVEGSDNKVALAPLPVAEVNPPAP
ACT-95QIB80518.1_348EBCP      TLKQGIALAQSRYWRVGAMYQGLGWEMLNWPVEAKTVVEGSDNKVALAPLPVAEVNPPVP
ACT-95BBV69349.1_35128]EBCP   TLKQGIALAQSRYWRVGAMYQGLGWEMLNWPVEAKTVVEGSDNKVALAPLPVAEVNPPVP
ACT-95UZQ68133.1_388EBCP      TLKQGIALAQSRYWRVGAMYQGLGWEMLNWPVEAKTVVEGSDNKVALAPLPVAEVNPPVP
ACT-95QIB80518.1_348EBOC      TLKQGIALAQSRYWRVGAMYQGLGWEMLNWPVEAKTVVEGSDNKVALAPLPVAEVNPPVP
ACT-95BBV69349.1_35128]EBOC   TLKQGIALAQSRYWRVGAMYQGLGWEMLNWPVEAKTVVEGSDNKVALAPLPVAEVNPPVP
ACT-95UZQ68133.1_388EBOC      TLKQGIALAQSRYWRVGAMYQGLGWEMLNWPVEAKTVVEGSDNKVALAPLPVAEVNPPVP
ACT-52QFH88560.1_350EBCP      TLKQGIALAQSRYWRVGAMYQGLGWEMLNWPVEAKTVVEGSDNKVALAPLPVAEVNPPAP
ACT-52BBS30224.1_43052]EBCP   TLKQGIALAQSRYWRVGAMYQGLGWEMLNWPVEAKTVVEGSDNKVALAPLPVAEVNPPAP
ACT-52WBN86316.1_4083EBCP     TLKQGIALAQSRYWRVGAMYQGLGWEMLNWPVEAKTVVEGSDNKVALAPLPVAEVNPPAP
ACT-52QFH88560.1_350EBOC      TLKQGIALAQSRYWRVGAMYQGLGWEMLNWPVEAKTVVEGSDNKVALAPLPVAEVNPPAP
ACT-52BBS30224.1_43052]EBOC   TLKQGIALAQSRYWRVGAMYQGLGWEMLNWPVEAKTVVEGSDNKVALAPLPVAEVNPPAP
ACT-52WBN86316.1_4083EBOC     TLKQGIALAQSRYWRVGAMYQGLGWEMLNWPVEAKTVVEGSDNKVALAPLPVAEVNPPAP
ACT-4QLR27288.1_395EBCP       SLRQGIALAQSRYWRVGAMYQGLGWEMLNWPVDAKTVVEGSDNKVALAPLPAREVNPPAP
ACT-4QPS68257.1_413EBCP       SLRQGIALAQSRYWRVGAMYQGLGWEMLNWPVDAKTVVEGSDNKVALAPLPAREVNPPAP
ACT-4QLR27288.1_395EBOC       SLRQGIALAQSRYWRVGAMYQGLGWEMLNWPVDAKTVVEGSDNKVALAPLPAREVNPPAP
ACT-4QPS68257.1_413EBOC       SLRQGIALAQSRYWRVGAMYQGLGWEMLNWPVDAKTVVEGSDNKVALAPLPAREVNPPAP
ACT-3UOY53510.1_358EBCP       SLRKGLTLAQSRYWRVGAMYQGLGWEMLNWPVDAKTVVEGSDNKVALAPLPAREVNPPAP
ACT-3UOY63785.1_4171EBCP      SLRKGLTLAQSRYWRVGAMYQGLGWEMLNWPVDAKTVVEGSDNKVALAPLPAREVNPPAP
ACT-3UOY53510.1_358EBOC       SLRKGLTLAQSRYWRVGAMYQGLGWEMLNWPVDAKTVVEGSDNKVALAPLPAREVNPPAP
ACT-3UOY63785.1_4171EBOC      SLRKGLTLAQSRYWRVGAMYQGLGWEMLNWPVDAKTVVEGSDNKVALAPLPAREVNPPAP
ACT-1QLV61731.1_472EBCP       SLRKGLTLAQSRYWRVGAMYQGLGWEMLNWPVDAKTVVEGSDNKVALAPLPAREVNPPAP
ACT-1UWA77567.1_382EBCP       SLRKGLTLAQSRYWRVGAMYQGLGWEMLNWPVDAKTVVEGSDNKVALAPLPAREVNPPAP
ACT-1QLV61731.1_472EBOC       SLRKGLTLAQSRYWRVGAMYQGLGWEMLNWPVDAKTVVEGSDNKVALAPLPAREVNPPAP
ACT-1UWA77567.1_382EBOC       SLRKGLTLAQSRYWRVGAMYQGLGWEMLNWPVDAKTVVEGSDNKVALAPLPAREVNPPAP
ACT-77QWZ50133.1_330EBCP      SLKQGITLAQSRYWRVGAMYQGLGWEMLNWPVDAKTVVGGSDNKVALAPLPAREVSPPVP
ACT-77UBH39796.1_3999EBCP     SLKQGITLAQSRYWRVGAMYQGLGWEMLNWPVDAKTVVGGSDNKVALAPLPAREVSPPVP
ACT-77UBH98103.1_3842EBCP     SLKQGITLAQSRYWRVGAMYQGLGWEMLNWPVDAKTVVGGSDNKVALAPLPAREVSPPVP
ACT-77UBH94108.1_193EBCP      SLKQGITLAQSRYWRVGAMYQGLGWEMLNWPVDAKTVVGGSDNKVALAPLPAREVSPPVP
ACT-77QWZ50133.1_330EBOC      SLKQGITLAQSRYWRVGAMYQGLGWEMLNWPVDAKTVVGGSDNKVALAPLPAREVSPPVP
ACT-77UBH39796.1_3999EBOC     SLKQGITLAQSRYWRVGAMYQGLGWEMLNWPVDAKTVVGGSDNKVALAPLPAREVSPPVP
ACT-77UBH98103.1_3842EBOC     SLKQGITLAQSRYWRVGAMYQGLGWEMLNWPVDAKTVVGGSDNKVALAPLPAREVSPPVP
ACT-77UBH94108.1_193EBOC      SLKQGITLAQSRYWRVGAMYQGLGWEMLNWPVDAKTVVGGSDNKVALAPLPAREVSPPVP
ACT-53QEL38380.1_485EBCP      SLGQGITLAQSRYWRVGAMYQGLGWEMLNWPVDAKTVVDGSDNKVALAPLPAREVNPPAP
ACT-53QEL38380.1_485EBOC      SLGQGITLAQSRYWRVGAMYQGLGWEMLNWPVDAKTVVDGSDNKVALAPLPAREVNPPAP
ACT-57QBB07838.1_4703EBCP     SLRKGLTLAQSRYWRVGAMYQGLGWEMLNWPVDAKTVVEGSDNKVALAPLPAREVNPPAP
ACT-57QYD27335.1_499EBCP      SLRKGLTLAQSRYWRVGAMYQGLGWEMLNWPVDAKTVVEGSDNKVALAPLPAREVNPPAP
ACT-57UBM17197.1_2672EBCP     SLRKGLTLAQSRYWRVGAMYQGLGWEMLNWPVDAKTVVEGSDNKVALAPLPAREVNPPAP
ACT-57UKB55021.1_380EBCP      SLRKGLTLAQSRYWRVGAMYQGLGWEMLNWPVDAKTVVEGSDNKVALAPLPAREVNPPAP
ACT-57QBB07838.1_4703EBOC     SLRKGLTLAQSRYWRVGAMYQGLGWEMLNWPVDAKTVVEGSDNKVALAPLPAREVNPPAP
ACT-57QYD27335.1_499EBOC      SLRKGLTLAQSRYWRVGAMYQGLGWEMLNWPVDAKTVVEGSDNKVALAPLPAREVNPPAP
ACT-57UBM17197.1_2672EBOC     SLRKGLTLAQSRYWRVGAMYQGLGWEMLNWPVDAKTVVEGSDNKVALAPLPAREVNPPAP
ACT-57UKB55021.1_380EBOC      SLRKGLTLAQSRYWRVGAMYQGLGWEMLNWPVDAKTVVEGSDNKVALAPLPAREVNPPAP
ACT-93ALL15984.1_396EOC       SLRKGLTLAQSRYWRVGAMYQGLGWEMLNWPVDAKTVVEGSDNKVALAPLPAREVNPPAP
ACT-9AOP85131.1_312EBCP       TLKQGIALAQSRYWRVGAMYQGLGWEMLNWPVEAKTVVEGSDNKVALAPLLVAEVNPPAP
ACT-9UOY32628.1_343EBCP       TLKQGIALAQSRYWRVGAMYQGLGWEMLNWPVEAKTVVEGSDNKVALAPLLVAEVNPPAP
ACT-9AOP85131.1_312EBOC       TLKQGIALAQSRYWRVGAMYQGLGWEMLNWPVEAKTVVEGSDNKVALAPLLVAEVNPPAP
ACT-9UOY32628.1_343EBOC       TLKQGIALAQSRYWRVGAMYQGLGWEMLNWPVEAKTVVEGSDNKVALAPLLVAEVNPPAP
ACT-9AMZ75637.1_173EOC        TLKQGIALAQSRYWRVGAMYQGLGWEMLNWPVEAKTVVEGSDNKVALAPLLVAEVNPPAP
ACT-6BBT43181.1_3396]EBCP     SLKEGITLAQSRYWRVGAMYQGLGWEMLNWPVDAKTVVEGSDNKVALAPLPAREVNPPAP
ACT-6BBT43181.1_3396]EBOC     SLKEGITLAQSRYWRVGAMYQGLGWEMLNWPVDAKTVVEGSDNKVALAPLPAREVNPPAP
ACT-46QUP91302.1_387EBCP      SLKQGIALAQSRYWRIGSMYQGLGWEMLNWPVEANTVVEGSDSKVALAPLPVAEVNPPAP
ACT-46QUP91302.1_387EBOC      SLKQGIALAQSRYWRIGSMYQGLGWEMLNWPVEANTVVEGSDSKVALAPLPVAEVNPPAP
ACT-67QLP16520.1_400EBCP      SLKQGIALAQSRYWRIGSMYQGLGWEMLNWPVEANTVIEGSDSKVALAPLPVAEVNPPAP
ACT-67QLP16520.1_400EBOC      SLKQGIALAQSRYWRIGSMYQGLGWEMLNWPVEANTVIEGSDSKVALAPLPVAEVNPPAP
ACT-84QOR49295.1_413EBCP      SLKQGIALAQSRYWRIGSMYQGLGWEMLNWPVEANTVVEGSDSKVALAPLPVAEVNPPAP
ACT-84QOR49295.1_413EBOC      SLKQGIALAQSRYWRIGSMYQGLGWEMLNWPVEANTVVEGSDSKVALAPLPVAEVNPPAP
ACT-74QOX68946.1_496EBCP      SLKQGIALAQSRYWRIGSMYQGLGWEMLNWPVEANTVVEGSDSKVALAPLPVAEVNPPAP
ACT-74UIZ04332.1_460EBCP      SLKQGIALAQSRYWRIGSMYQGLGWEMLNWPVEANTVVEGSDSKVALAPLPVAEVNPPAP
ACT-74QOX68946.1_496EBOC      SLKQGIALAQSRYWRIGSMYQGLGWEMLNWPVEANTVVEGSDSKVALAPLPVAEVNPPAP
ACT-74UIZ04332.1_460EBOC      SLKQGIALAQSRYWRIGSMYQGLGWEMLNWPVEANTVVEGSDSKVALAPLPVAEVNPPAP
ACT-74QQT40108.1_4574EOC      SLKQGIALAQSRYWRIGSMYQGLGWEMLNWPVEANTVVEGSDSKVALAPLPVAEVNPPAP
ACT-16ATW90451.1_311EBCP      SLKQGIALAQSRYWRIGSMYQGLGWEMLNWPVEANTVVEGSDSKVALAPLPVAEVNPPAP
ACT-16AVO82105.1_1257EBCP     SLKQGIALAQSRYWRIGSMYQGLGWEMLNWPVEANTVVEGSDSKVALAPLPVAEVNPPAP
ACT-16AWQ41825.1_393EBCP      SLKQGIALAQSRYWRIGSMYQGLGWEMLNWPVEANTVVEGSDSKVALAPLPVAEVNPPAP
ACT-16AWQ56038.1_393EBCP      SLKQGIALAQSRYWRIGSMYQGLGWEMLNWPVEANTVVEGSDSKVALAPLPVAEVNPPAP
ACT-16QFH53420.1_474EBCP      SLKQGIALAQSRYWRIGSMYQGLGWEMLNWPVEANTVVEGSDSKVALAPLPVAEVNPPAP
ACT-16QPO90952.1_396EBCP      SLKQGIALAQSRYWRIGSMYQGLGWEMLNWPVEANTVVEGSDSKVALAPLPVAEVNPPAP
ACT-16QQA67679.1_472EBCP      SLKQGIALAQSRYWRIGSMYQGLGWEMLNWPVEANTVVEGSDSKVALAPLPVAEVNPPAP
ACT-16UDV14809.1_2173EBCP     SLKQGIALAQSRYWRIGSMYQGLGWEMLNWPVEANTVVEGSDSKVALAPLPVAEVNPPAP
ACT-16UDV41468.1_3105EBCP     SLKQGIALAQSRYWRIGSMYQGLGWEMLNWPVEANTVVEGSDSKVALAPLPVAEVNPPAP
ACT-16UIZ08994.1_395EBCP      SLKQGIALAQSRYWRIGSMYQGLGWEMLNWPVEANTVVEGSDSKVALAPLPVAEVNPPAP
ACT-16WJJ28417.1_386EBCP      SLKQGIALAQSRYWRIGSMYQGLGWEMLNWPVEANTVVEGSDSKVALAPLPVAEVNPPAP
ACT-16ATW90451.1_311EBOC      SLKQGIALAQSRYWRIGSMYQGLGWEMLNWPVEANTVVEGSDSKVALAPLPVAEVNPPAP
ACT-16AVO82105.1_1257EBOC     SLKQGIALAQSRYWRIGSMYQGLGWEMLNWPVEANTVVEGSDSKVALAPLPVAEVNPPAP
ACT-16AWQ41825.1_393EBOC      SLKQGIALAQSRYWRIGSMYQGLGWEMLNWPVEANTVVEGSDSKVALAPLPVAEVNPPAP
ACT-16AWQ56038.1_393EBOC      SLKQGIALAQSRYWRIGSMYQGLGWEMLNWPVEANTVVEGSDSKVALAPLPVAEVNPPAP
ACT-16QFH53420.1_474EBOC      SLKQGIALAQSRYWRIGSMYQGLGWEMLNWPVEANTVVEGSDSKVALAPLPVAEVNPPAP
ACT-16QPO90952.1_396EBOC      SLKQGIALAQSRYWRIGSMYQGLGWEMLNWPVEANTVVEGSDSKVALAPLPVAEVNPPAP
ACT-16QQA67679.1_472EBOC      SLKQGIALAQSRYWRIGSMYQGLGWEMLNWPVEANTVVEGSDSKVALAPLPVAEVNPPAP
ACT-16UDV14809.1_2173EBOC     SLKQGIALAQSRYWRIGSMYQGLGWEMLNWPVEANTVVEGSDSKVALAPLPVAEVNPPAP
ACT-16UDV41468.1_3105EBOC     SLKQGIALAQSRYWRIGSMYQGLGWEMLNWPVEANTVVEGSDSKVALAPLPVAEVNPPAP
ACT-16UIZ08994.1_395EBOC      SLKQGIALAQSRYWRIGSMYQGLGWEMLNWPVEANTVVEGSDSKVALAPLPVAEVNPPAP
ACT-16WJJ28417.1_386EBOC      SLKQGIALAQSRYWRIGSMYQGLGWEMLNWPVEANTVVEGSDSKVALAPLPVAEVNPPAP
```

ACT-23QLP51667.1_388EBCP	SLKQGISLAQSRYWRIGSMYQGLGWEMLNWPVEANTVIEGSDSKVALAPLPVAEVNPPAP
ACT-23QLP61663.1_388EBCP	SLKQGISLAQSRYWRIGSMYQGLGWEMLNWPVEANTVIEGSDSKVALAPLPVAEVNPPAP
ACT-23QLP66427.1_430EBCP	SLKQGISLAQSRYWRIGSMYQGLGWEMLNWPVEANTVIEGSDSKVALAPLPVAEVNPPAP
ACT-23QLU80799.1_388EBCP	SLKQGISLAQSRYWRIGSMYQGLGWEMLNWPVEANTVIEGSDSKVALAPLPVAEVNPPAP
ACT-23QLT97825.1_367EBCP	SLKQGISLAQSRYWRIGSMYQGLGWEMLNWPVEANTVIEGSDSKVALAPLPVAEVNPPAP
ACT-23QLU89187.1_4307EBCP	SLKQGISLAQSRYWRIGSMYQGLGWEMLNWPVEANTVIEGSDSKVALAPLPVAEVNPPAP
ACT-23QLV76440.1_389EBCP	SLKQGISLAQSRYWRIGSMYQGLGWEMLNWPVEANTVIEGSDSKVALAPLPVAEVNPPAP
ACT-23QLT57991.1_396EBCP	SLKQGISLAQSRYWRIGSMYQGLGWEMLNWPVEANTVIEGSDSKVALAPLPVAEVNPPAP
ACT-23QLV44058.1_369EBCP	SLKQGISLAQSRYWRIGSMYQGLGWEMLNWPVEANTVIEGSDSKVALAPLPVAEVNPPAP
ACT-23QLP51667.1_388EBOC	SLKQGISLAQSRYWRIGSMYQGLGWEMLNWPVEANTVIEGSDSKVALAPLPVAEVNPPAP
ACT-23QLP61663.1_388EBOC	SLKQGISLAQSRYWRIGSMYQGLGWEMLNWPVEANTVIEGSDSKVALAPLPVAEVNPPAP
ACT-23QLP66427.1_430EBOC	SLKQGISLAQSRYWRIGSMYQGLGWEMLNWPVEANTVIEGSDSKVALAPLPVAEVNPPAP
ACT-23QLU80799.1_388EBOC	SLKQGISLAQSRYWRIGSMYQGLGWEMLNWPVEANTVIEGSDSKVALAPLPVAEVNPPAP
ACT-23QLT97825.1_367EBOC	SLKQGISLAQSRYWRIGSMYQGLGWEMLNWPVEANTVIEGSDSKVALAPLPVAEVNPPAP
ACT-23QLU89187.1_4307EBOC	SLKQGISLAQSRYWRIGSMYQGLGWEMLNWPVEANTVIEGSDSKVALAPLPVAEVNPPAP
ACT-23QLV76440.1_389EBOC	SLKQGISLAQSRYWRIGSMYQGLGWEMLNWPVEANTVIEGSDSKVALAPLPVAEVNPPAP
ACT-23QLT57991.1_396EBOC	SLKQGISLAQSRYWRIGSMYQGLGWEMLNWPVEANTVIEGSDSKVALAPLPVAEVNPPAP
ACT-23QLV44058.1_369EBOC	SLKQGISLAQSRYWRIGSMYQGLGWEMLNWPVEANTVIEGSDSKVALAPLPVAEVNPPAP
ACT-115AXQ35263.1_2972EBCP	SLKQGIALAQSRYWRIGSMYQGLGWEMLNWPVEANTVVEGSDSKVALAPLPVAEVNPPAP
ACT-115QGR11187.1_384EBCP	SLKQGIALAQSRYWRIGSMYQGLGWEMLNWPVEANTVVEGSDSKVALAPLPVAEVNPPAP
ACT-115QIF26741.1_2106EBCP	SLKQGIALAQSRYWRIGSMYQGLGWEMLNWPVEANTVVEGSDSKVALAPLPVAEVNPPAP
ACT-115QIF32246.1_3030EBCP	SLKQGIALAQSRYWRIGSMYQGLGWEMLNWPVEANTVVEGSDSKVALAPLPVAEVNPPAP
ACT-115QOD35429.1_385EBCP	SLKQGIALAQSRYWRIGSMYQGLGWEMLNWPVEANTVVEGSDSKVALAPLPVAEVNPPAP
ACT-115QPD66621.1_385EBCP	SLKQGIALAQSRYWRIGSMYQGLGWEMLNWPVEANTVVEGSDSKVALAPLPVAEVNPPAP
ACT-115QSS35407.1_385EBCP	SLKQGIALAQSRYWRIGSMYQGLGWEMLNWPVEANTVVEGSDSKVALAPLPVAEVNPPAP
ACT-115URE88756.1_385EBCP	SLKQGIALAQSRYWRIGSMYQGLGWEMLNWPVEANTVVEGSDSKVALAPLPVAEVNPPAP
ACT-115USM81982.1_384EBCP	SLKQGIALAQSRYWRIGSMYQGLGWEMLNWPVEANTVVEGSDSKVALAPLPVAEVNPPAP
ACT-115AXQ35263.1_2972EBOC	SLKQGIALAQSRYWRIGSMYQGLGWEMLNWPVEANTVVEGSDSKVALAPLPVAEVNPPAP
ACT-115QGR11187.1_384EBOC	SLKQGIALAQSRYWRIGSMYQGLGWEMLNWPVEANTVVEGSDSKVALAPLPVAEVNPPAP
ACT-115QIF26741.1_2106EBOC	SLKQGIALAQSRYWRIGSMYQGLGWEMLNWPVEANTVVEGSDSKVALAPLPVAEVNPPAP
ACT-115QIF32246.1_3030EBOC	SLKQGIALAQSRYWRIGSMYQGLGWEMLNWPVEANTVVEGSDSKVALAPLPVAEVNPPAP
ACT-115QOD35429.1_385EBOC	SLKQGIALAQSRYWRIGSMYQGLGWEMLNWPVEANTVVEGSDSKVALAPLPVAEVNPPAP
ACT-115QPD66621.1_385EBOC	SLKQGIALAQSRYWRIGSMYQGLGWEMLNWPVEANTVVEGSDSKVALAPLPVAEVNPPAP
ACT-115QSS35407.1_385EBOC	SLKQGIALAQSRYWRIGSMYQGLGWEMLNWPVEANTVVEGSDSKVALAPLPVAEVNPPAP
ACT-115URE88756.1_385EBOC	SLKQGIALAQSRYWRIGSMYQGLGWEMLNWPVEANTVVEGSDSKVALAPLPVAEVNPPAP
ACT-115USM81982.1_384EBOC	SLKQGIALAQSRYWRIGSMYQGLGWEMLNWPVEANTVVEGSDSKVALAPLPVAEVNPPAP
ACT-25AVU48988.1_427EBCP	SLKQGIALAQSRYWRIGSMYQGLGWEMLNWPVEANTVVEGSDSKVALAPLPVAEVNPPAP
ACT-25UQQ46893.1_379EBCP	SLKQGIALAQSRYWRIGSMYQGLGWEMLNWPVEANTVVEGSDSKVALAPLPVAEVNPPAP
ACT-25WDO17280.1_310EBCP	SLKQGIALAQSRYWRIGSMYQGLGWEMLNWPVEANTVVEGSDSKVALAPLPVAEVNPPAP
ACT-25AVU48988.1_427EBOC	SLKQGIALAQSRYWRIGSMYQGLGWEMLNWPVEANTVVEGSDSKVALAPLPVAEVNPPAP
ACT-25UQQ46893.1_379EBOC	SLKQGIALAQSRYWRIGSMYQGLGWEMLNWPVEANTVVEGSDSKVALAPLPVAEVNPPAP
ACT-25WDO17280.1_310EBOC	SLKQGIALAQSRYWRIGSMYQGLGWEMLNWPVEANTVVEGSDSKVALAPLPVAEVNPPAP
ACT-25AOP89724.1_377EOC	SLKQGIALAQSRYWRIGSMYQGLGWEMLNWPVEANTVVEGSDSKVALAPLPVAEVNPPAP
ACT-45APR43249.1_2902EBCP	SLKQGIALAQSRYWRIGSMYQGLGWEMLNWPVEANTVVEGSDSKVALAPLPVAEVNPPAP
ACT-45ASA05885.1_3853EBCP	SLKQGIALAQSRYWRIGSMYQGLGWEMLNWPVEANTVVEGSDSKVALAPLPVAEVNPPAP
ACT-45ASB85359.1_3592EBCP	SLKQGIALAQSRYWRIGSMYQGLGWEMLNWPVEANTVVEGSDSKVALAPLPVAEVNPPAP
ACT-45AWR70714.1_4154EBCP	SLKQGIALAQSRYWRIGSMYQGLGWEMLNWPVEANTVVEGSDSKVALAPLPVAEVNPPAP
ACT-45AXM01511.1_4138EBCP	SLKQGIALAQSRYWRIGSMYQGLGWEMLNWPVEANTVVEGSDSKVALAPLPVAEVNPPAP
ACT-45AYU96034.1_2649EBCP	SLKQGIALAQSRYWRIGSMYQGLGWEMLNWPVEANTVVEGSDSKVALAPLPVAEVNPPAP
ACT-45QBH65043.1_3960EBCP	SLKQGIALAQSRYWRIGSMYQGLGWEMLNWPVEANTVVEGSDSKVALAPLPVAEVNPPAP
ACT-45QEL31100.1_1855EBCP	SLKQGIALAQSRYWRIGSMYQGLGWEMLNWPVEANTVVEGSDSKVALAPLPVAEVNPPAP
ACT-45ULQ22907.1_427EBCP	SLKQGIALAQSRYWRIGSMYQGLGWEMLNWPVEANTVVEGSDSKVALAPLPVAEVNPPAP
ACT-45ULQ25748.1_3478EBCP	SLKQGIALAQSRYWRIGSMYQGLGWEMLNWPVEANTVVEGSDSKVALAPLPVAEVNPPAP
ACT-45WCO72956.1_42EBCP	SLKQGIALAQSRYWRIGSMYQGLGWEMLNWPVEANTVVEGSDSKVALAPLPVAEVNPPAP
ACT-45APR43249.1_2902EBOC	SLKQGIALAQSRYWRIGSMYQGLGWEMLNWPVEANTVVEGSDSKVALAPLPVAEVNPPAP
ACT-45ASA05885.1_3853EBOC	SLKQGIALAQSRYWRIGSMYQGLGWEMLNWPVEANTVVEGSDSKVALAPLPVAEVNPPAP
ACT-45ASB85359.1_3592EBOC	SLKQGIALAQSRYWRIGSMYQGLGWEMLNWPVEANTVVEGSDSKVALAPLPVAEVNPPAP
ACT-45AWR70714.1_4154EBOC	SLKQGIALAQSRYWRIGSMYQGLGWEMLNWPVEANTVVEGSDSKVALAPLPVAEVNPPAP
ACT-45AXM01511.1_4138EBOC	SLKQGIALAQSRYWRIGSMYQGLGWEMLNWPVEANTVVEGSDSKVALAPLPVAEVNPPAP
ACT-45AYU96034.1_2649EBOC	SLKQGIALAQSRYWRIGSMYQGLGWEMLNWPVEANTVVEGSDSKVALAPLPVAEVNPPAP
ACT-45QBH65043.1_3960EBOC	SLKQGIALAQSRYWRIGSMYQGLGWEMLNWPVEANTVVEGSDSKVALAPLPVAEVNPPAP
ACT-45QEL31100.1_1855EBOC	SLKQGIALAQSRYWRIGSMYQGLGWEMLNWPVEANTVVEGSDSKVALAPLPVAEVNPPAP
ACT-45ULQ22907.1_427EBOC	SLKQGIALAQSRYWRIGSMYQGLGWEMLNWPVEANTVVEGSDSKVALAPLPVAEVNPPAP
ACT-45ULQ25748.1_3478EBOC	SLKQGIALAQSRYWRIGSMYQGLGWEMLNWPVEANTVVEGSDSKVALAPLPVAEVNPPAP
ACT-45WCO72956.1_42EBOC	SLKQGIALAQSRYWRIGSMYQGLGWEMLNWPVEANTVVEGSDSKVALAPLPVAEVNPPAP
ACT-C197QLA02472.1_2022EOC	SLKQGISLAQSRYWRIGSMYQGLGWEMLNWPVEANTVIEGSDSKVALAPLPVAEVNPPAP
ACT-64QMT06026.1_4255EBCP	TLKQGIALAQSRYWRVGAMYQGLGWEMLNWPVEAKTVVEGSDNKVALAPLPVAEVNPPAP
ACT-64QMT06026.1_4255EBOC	TLKQGIALAQSRYWRVGAMYQGLGWEMLNWPVEAKTVVEGSDNKVALAPLPVAEVNPPAP
ACT-C198QXA51435.1_2071EOC	SLKQGIALAQSRYWRVGSMYQGLGWEMLNWPVDGKTIIDGSDNKIALAALPAREVNPPAP
acc	

```
                                β14         β15    η7     β16             α12
ACT-17BBW34093.1_49417]EBCP
                                  370        380         390        400        410
ACT-17BBW34093.1_49417]EBCP     PVKASWVHKTGSTGGFGSYVAFIPEKQIGIVMLANTSYPNPARVEAAYHILEAL..Q
ACT-17BBW34093.1_49417]EBOC     PVKASWVHKTGSTGGFGSYVAFIPEKQIGIVMLANTSYPNPARVEAAYHILEAL..Q
7TI1_1|Chain                    PVKASWVHKTGSTGGFGSYVAFIPEKQIGIVMLANTSYPNPARVEAAYHILEALQLE
ACT-37QPO56206.1_411EBCP        PVKASWVHKTGSTGGFGSYVAFIPEKQIGIVMLANKSYPNPARVEAAYHILDAL..Q
ACT-37QPO65286.1_410EBCP        PVKASWVHKTGSTGGFGSYVAFIPEKQIGIVMLANKSYPNPARVEAAYHILDAL..Q
ACT-37UKK25917.1_410EBCP        PVKASWVHKTGSTGGFGSYVAFIPEKQIGIVMLANKSYPNPARVEAAYHILDAL..Q
ACT-37UKK30553.1_410EBCP        PVKASWVHKTGSTGGFGSYVAFIPEKQIGIVMLANKSYPNPARVEAAYHILDAL..Q
ACT-37QPO56206.1_411EBOC        PVKASWVHKTGSTGGFGSYVAFIPEKQIGIVMLANKSYPNPARVEAAYHILDAL..Q
ACT-37QPO65286.1_410EBOC        PVKASWVHKTGSTGGFGSYVAFIPEKQIGIVMLANKSYPNPARVEAAYHILDAL..Q
ACT-37UKK25917.1_410EBOC        PVKASWVHKTGSTGGFGSYVAFIPEKQIGIVMLANKSYPNPARVEAAYHILDAL..Q
ACT-37UKK30553.1_410EBOC        PVKASWVHKTGSTGGFGSYVAFIPEKQIGIVMLANKSYPNPARVEAAYHILDAL..Q
ACT-65AXO42196.1_4001EBCP       PVKASWVHKTGSTGGFGSYVAFIPEKQIGIVMLANTSYPNPARVEAAYHILEAL..Q
ACT-65AXO42196.1_4001EBOC       PVKASWVHKTGSTGGFGSYVAFIPEKQIGIVMLANTSYPNPARVEAAYHILEAL..Q
ACT-65WBV31824.1_394EOC         PVKASWVHKTGSTGGFGSYVAFIPEKQIGIVMLANTSYPNPARVEAAYHILEAL..Q
ACT-17ARZ78489.1_1874EBCP       PVKASWVHKTGSTGGFGSYVAFIPEKQIGIVMLANTSYPNPARVEAAYHILEAL..Q
ACT-17ASQ75348.1_386EBCP        PVKASWVHKTGSTGGFGSYVAFIPEKQIGIVMLANTSYPNPARVEAAYHILEAL..Q
ACT-17AWV77932.1_4320EBCP       PVKASWVHKTGSTGGFGSYVAFIPEKQIGIVMLANTSYPNPARVEAAYHILEAL..Q
ACT-17QEQ46013.1_2619EBCP       PVKASWVHKTGSTGGFGSYVAFIPEKQIGIVMLANTSYPNPARVEAAYHILEAL..Q
ACT-17QFI20602.1_494EBCP        PVKASWVHKTGSTGGFGSYVAFIPEKQIGIVMLANTSYPNPARVEAAYHILEAL..Q
ACT-17QIE65424.1_3050EBCP       PVKASWVHKTGSTGGFGSYVAFIPEKQIGIVMLANTSYPNPARVEAAYHILEAL..Q
ACT-17QPD71520.1_416EBCP        PVKASWVHKTGSTGGFGSYVAFIPEKQIGIVMLANTSYPNPARVEAAYHILEAL..Q
ACT-17QPO45831.1_587EBCP        PVKASWVHKTGSTGGFGSYVAFIPEKQIGIVMLANTSYPNPARVEAAYHILEAL..Q
ACT-17QPO51098.1_586EBCP        PVKASWVHKTGSTGGFGSYVAFIPEKQIGIVMLANTSYPNPARVEAAYHILEAL..Q
ACT-17QPO69921.1_405EBCP        PVKASWVHKTGSTGGFGSYVAFIPEKQIGIVMLANTSYPNPARVEAAYHILEAL..Q
ACT-17QPO74759.1_405EBCP        PVKASWVHKTGSTGGFGSYVAFIPEKQIGIVMLANTSYPNPARVEAAYHILEAL..Q
ACT-17QPO85066.1_405EBCP        PVKASWVHKTGSTGGFGSYVAFIPEKQIGIVMLANTSYPNPARVEAAYHILEAL..Q
ACT-17QSB73424.1_1103EBCP       PVKASWVHKTGSTGGFGSYVAFIPEKQIGIVMLANTSYPNPARVEAAYHILEAL..Q
ACT-17QXY98988.1_1068EBCP       PVKASWVHKTGSTGGFGSYVAFIPEKQIGIVMLANTSYPNPARVEAAYHILEAL..Q
ACT-17UDV73425.1_430EBCP        PVKASWVHKTGSTGGFGSYVAFIPEKQIGIVMLANTSYPNPARVEAAYHILEAL..Q
ACT-17UDV69018.1_894EBCP        PVKASWVHKTGSTGGFGSYVAFIPEKQIGIVMLANTSYPNPARVEAAYHILEAL..Q
ACT-17UKK21116.1_588EBCP        PVKASWVHKTGSTGGFGSYVAFIPEKQIGIVMLANTSYPNPARVEAAYHILEAL..Q
ACT-17UOY44701.1_2566EBCP       PVKASWVHKTGSTGGFGSYVAFIPEKQIGIVMLANTSYPNPARVEAAYHILEAL..Q
ACT-17URL69109.1_2030EBCP       PVKASWVHKTGSTGGFGSYVAFIPEKQIGIVMLANTSYPNPARVEAAYHILEAL..Q
ACT-17UUQ70490.1_426EBCP        PVKASWVHKTGSTGGFGSYVAFIPEKQIGIVMLANTSYPNPARVEAAYHILEAL..Q
ACT-17UWA70738.1_427EBCP        PVKASWVHKTGSTGGFGSYVAFIPEKQIGIVMLANTSYPNPARVEAAYHILEAL..Q
ACT-17UWC98141.1_545EBCP        PVKASWVHKTGSTGGFGSYVAFIPEKQIGIVMLANTSYPNPARVEAAYHILEAL..Q
ACT-17UXC72711.1_448EBCP        PVKASWVHKTGSTGGFGSYVAFIPEKQIGIVMLANTSYPNPARVEAAYHILEAL..Q
ACT-17UXC77377.1_448EBCP        PVKASWVHKTGSTGGFGSYVAFIPEKQIGIVMLANTSYPNPARVEAAYHILEAL..Q
ACT-17UXC82023.1_448EBCP        PVKASWVHKTGSTGGFGSYVAFIPEKQIGIVMLANTSYPNPARVEAAYHILEAL..Q
ACT-17UZE41799.1_406EBCP        PVKASWVHKTGSTGGFGSYVAFIPEKQIGIVMLANTSYPNPARVEAAYHILEAL..Q
ACT-17UZH49391.1_396EBCP        PVKASWVHKTGSTGGFGSYVAFIPEKQIGIVMLANTSYPNPARVEAAYHILEAL..Q
ACT-17WAZ63377.1_405EBCP        PVKASWVHKTGSTGGFGSYVAFIPEKQIGIVMLANTSYPNPARVEAAYHILEAL..Q
ACT-17WDU86092.1_3769EBCP       PVKASWVHKTGSTGGFGSYVAFIPEKQIGIVMLANTSYPNPARVEAAYHILEAL..Q
ACT-17WDW12975.1_3764EBCP       PVKASWVHKTGSTGGFGSYVAFIPEKQIGIVMLANTSYPNPARVEAAYHILEAL..Q
ACT-17WGA71117.1_396EBCP        PVKASWVHKTGSTGGFGSYVAFIPEKQIGIVMLANTSYPNPARVEAAYHILEAL..Q
ACT-17WGA75603.1_396EBCP        PVKASWVHKTGSTGGFGSYVAFIPEKQIGIVMLANTSYPNPARVEAAYHILEAL..Q
ACT-17WGZ57121.1_2667EBCP       PVKASWVHKTGSTGGFGSYVAFIPEKQIGIVMLANTSYPNPARVEAAYHILEAL..Q
ACT-17ARZ78489.1_1874EBOC       PVKASWVHKTGSTGGFGSYVAFIPEKQIGIVMLANTSYPNPARVEAAYHILEAL..Q
ACT-17ASQ75348.1_386EBOC        PVKASWVHKTGSTGGFGSYVAFIPEKQIGIVMLANTSYPNPARVEAAYHILEAL..Q
ACT-17AWV77932.1_4320EBOC       PVKASWVHKTGSTGGFGSYVAFIPEKQIGIVMLANTSYPNPARVEAAYHILEAL..Q
ACT-17QEQ46013.1_2619EBOC       PVKASWVHKTGSTGGFGSYVAFIPEKQIGIVMLANTSYPNPARVEAAYHILEAL..Q
ACT-17QFI20602.1_494EBOC        PVKASWVHKTGSTGGFGSYVAFIPEKQIGIVMLANTSYPNPARVEAAYHILEAL..Q
ACT-17QIE65424.1_3050EBOC       PVKASWVHKTGSTGGFGSYVAFIPEKQIGIVMLANTSYPNPARVEAAYHILEAL..Q
ACT-17QPD71520.1_416EBOC        PVKASWVHKTGSTGGFGSYVAFIPEKQIGIVMLANTSYPNPARVEAAYHILEAL..Q
ACT-17QPO45831.1_587EBOC        PVKASWVHKTGSTGGFGSYVAFIPEKQIGIVMLANTSYPNPARVEAAYHILEAL..Q
ACT-17QPO51098.1_586EBOC        PVKASWVHKTGSTGGFGSYVAFIPEKQIGIVMLANTSYPNPARVEAAYHILEAL..Q
ACT-17QPO69921.1_405EBOC        PVKASWVHKTGSTGGFGSYVAFIPEKQIGIVMLANTSYPNPARVEAAYHILEAL..Q
ACT-17QPO74759.1_405EBOC        PVKASWVHKTGSTGGFGSYVAFIPEKQIGIVMLANTSYPNPARVEAAYHILEAL..Q
ACT-17QPO85066.1_405EBOC        PVKASWVHKTGSTGGFGSYVAFIPEKQIGIVMLANTSYPNPARVEAAYHILEAL..Q
ACT-17QSB73424.1_1103EBOC       PVKASWVHKTGSTGGFGSYVAFIPEKQIGIVMLANTSYPNPARVEAAYHILEAL..Q
ACT-17QXY98988.1_1068EBOC       PVKASWVHKTGSTGGFGSYVAFIPEKQIGIVMLANTSYPNPARVEAAYHILEAL..Q
ACT-17UDV73425.1_430EBOC        PVKASWVHKTGSTGGFGSYVAFIPEKQIGIVMLANTSYPNPARVEAAYHILEAL..Q
ACT-17UDV69018.1_894EBOC        PVKASWVHKTGSTGGFGSYVAFIPEKQIGIVMLANTSYPNPARVEAAYHILEAL..Q
ACT-17UKK21116.1_588EBOC        PVKASWVHKTGSTGGFGSYVAFIPEKQIGIVMLANTSYPNPARVEAAYHILEAL..Q
ACT-17UOY44701.1_2566EBOC       PVKASWVHKTGSTGGFGSYVAFIPEKQIGIVMLANTSYPNPARVEAAYHILEAL..Q
ACT-17URL69109.1_2030EBOC       PVKASWVHKTGSTGGFGSYVAFIPEKQIGIVMLANTSYPNPARVEAAYHILEAL..Q
ACT-17UUQ70490.1_426EBOC        PVKASWVHKTGSTGGFGSYVAFIPEKQIGIVMLANTSYPNPARVEAAYHILEAL..Q
ACT-17UWA70738.1_427EBOC        PVKASWVHKTGSTGGFGSYVAFIPEKQIGIVMLANTSYPNPARVEAAYHILEAL..Q
ACT-17UWC98141.1_545EBOC        PVKASWVHKTGSTGGFGSYVAFIPEKQIGIVMLANTSYPNPARVEAAYHILEAL..Q
ACT-17UXC72711.1_448EBOC        PVKASWVHKTGSTGGFGSYVAFIPEKQIGIVMLANTSYPNPARVEAAYHILEAL..Q
ACT-17UXC77377.1_448EBOC        PVKASWVHKTGSTGGFGSYVAFIPEKQIGIVMLANTSYPNPARVEAAYHILEAL..Q
ACT-17UXC82023.1_448EBOC        PVKASWVHKTGSTGGFGSYVAFIPEKQIGIVMLANTSYPNPARVEAAYHILEAL..Q
ACT-17UZE41799.1_406EBOC        PVKASWVHKTGSTGGFGSYVAFIPEKQIGIVMLANTSYPNPARVEAAYHILEAL..Q
ACT-17UZH49391.1_396EBOC        PVKASWVHKTGSTGGFGSYVAFIPEKQIGIVMLANTSYPNPARVEAAYHILEAL..Q
ACT-17WAZ63377.1_405EBOC        PVKASWVHKTGSTGGFGSYVAFIPEKQIGIVMLANTSYPNPARVEAAYHILEAL..Q
ACT-17WDU86092.1_3769EBOC       PVKASWVHKTGSTGGFGSYVAFIPEKQIGIVMLANTSYPNPARVEAAYHILEAL..Q
ACT-17WDW12975.1_3764EBOC       PVKASWVHKTGSTGGFGSYVAFIPEKQIGIVMLANTSYPNPARVEAAYHILEAL..Q
ACT-17WGA71117.1_396EBOC        PVKASWVHKTGSTGGFGSYVAFIPEKQIGIVMLANTSYPNPARVEAAYHILEAL..Q
ACT-17WGA75603.1_396EBOC        PVKASWVHKTGSTGGFGSYVAFIPEKQIGIVMLANTSYPNPARVEAAYHILEAL..Q
ACT-17WGZ57121.1_2667EBOC       PVKASWVHKTGSTGGFGSYVAFIPEKQIGIVMLANTSYPNPARVEAAYHILEAL..Q
ACT-17QEI60759.1_389EOC         PVKASWVHKTGSTGGFGSYVAFIPEKQIGIVMLANTSYPNPARVEAAYHILEAL..Q
ACT-17QEI70010.1_389EOC         PVKASWVHKTGSTGGFGSYVAFIPEKQIGIVMLANTSYPNPARVEAAYHILEAL..Q
ACT-17QEU13386.1_612EOC         PVKASWVHKTGSTGGFGSYVAFIPEKQIGIVMLANTSYPNPARVEAAYHILEAL..Q
ACT-17QHI59365.1_3674EOC        PVKASWVHKTGSTGGFGSYVAFIPEKQIGIVMLANTSYPNPARVEAAYHILEAL..Q
ACT-17UZQ58984.1_410EOC         PVKASWVHKTGSTGGFGSYVAFIPEKQIGIVMLANTSYPNPARVEAAYHILEAL..Q
ACT-40AWS79793.1_3058EBCP       PVKASWVHKTGSTGGFGSYVAFIPEKQIGIVMLANTSYPNPARVEAAYHILEAL..Q
ACT-40AXO44745.1_1607EBCP       PVKASWVHKTGSTGGFGSYVAFIPEKQIGIVMLANTSYPNPARVEAAYHILEAL..Q
ACT-40UXI63958.1_1746EBCP       PVKASWVHKTGSTGGFGSYVAFIPEKQIGIVMLANTSYPNPARVEAAYHILEAL..Q
ACT-40WDC52451.1_4284EBCP       PVKASWVHKTGSTGGFGSYVAFIPEKQIGIVMLANTSYPNPARVEAAYHILEAL..Q
ACT-40AWS79793.1_3058EBOC       PVKASWVHKTGSTGGFGSYVAFIPEKQIGIVMLANTSYPNPARVEAAYHILEAL..Q
ACT-40AXO44745.1_1607EBOC       PVKASWVHKTGSTGGFGSYVAFIPEKQIGIVMLANTSYPNPARVEAAYHILEAL..Q
ACT-40UXI63958.1_1746EBOC       PVKASWVHKTGSTGGFGSYVAFIPEKQIGIVMLANTSYPNPARVEAAYHILEAL..Q
ACT-40WDC52451.1_4284EBOC       PVKASWVHKTGSTGGFGSYVAFIPEKQIGIVMLANTSYPNPARVEAAYHILEAL..Q
ACT-44UWA09346.1_3724EBCP       PVKASWVHKTGSTGGFGSYVAFIPEKQIGIVMLANTNYPNPARVEAAYHILEAL..Q
ACT-44UWA09346.1_3724EBOC       PVKASWVHKTGSTGGFGSYVAFIPEKQIGIVMLANTNYPNPARVEAAYHILEAL..Q
ACT-90QFH83806.1_450EBCP        PVEASWVHKMGSTGGFGSYVAFIPEKQIGIVMLANKSYPNPARVEAAYHILDAL..Q
ACT-90QLO92194.1_500EBCP        PVEASWVHKMGSTGGFGSYVAFIPEKQIGIVMLANKSYPNPARVEAAYHILDAL..Q
ACT-90QLP01381.1_466EBCP        PVEASWVHKMGSTGGFGSYVAFIPEKQIGIVMLANKSYPNPARVEAAYHILDAL..Q
ACT-90QFH83806.1_450EBOC        PVEASWVHKMGSTGGFGSYVAFIPEKQIGIVMLANKSYPNPARVEAAYHILDAL..Q
ACT-90QLO92194.1_500EBOC        PVEASWVHKMGSTGGFGSYVAFIPEKQIGIVMLANKSYPNPARVEAAYHILDAL..Q
ACT-90QLP01381.1_466EBOC        PVEASWVHKMGSTGGFGSYVAFIPEKQIGIVMLANKSYPNPARVEAAYHILDAL..Q
ACT-24ASB73507.1_1185EBCP       PVKASWVHKTGSTGGFGSYVAFIPEKQIGIVMLANKSYPNPARVEAAYHILDAL..Q
ACT-24AVU18445.1_378EBCP        PVKASWVHKTGSTGGFGSYVAFIPEKQIGIVMLANKSYPNPARVEAAYHILDAL..Q
```

```
ACT-24QHC75881.1_374EBCP      PVKASWVHKTGSTGGFGSYVAFIPEKQIGIVMLANKSYPNPARVEAAYHILDAL..Q
ACT-24QLP84500.1_370EBCP      PVKASWVHKTGSTGGFGSYVAFIPEKQIGIVMLANKSYPNPARVEAAYHILDAL..Q
ACT-24QSH03071.1_370EBCP      PVKASWVHKTGSTGGFGSYVAFIPEKQIGIVMLANKSYPNPARVEAAYHILDAL..Q
ACT-24QSG93844.1_370EBCP      PVKASWVHKTGSTGGFGSYVAFIPEKQIGIVMLANKSYPNPARVEAAYHILDAL..Q
ACT-24QSG98466.1_370EBCP      PVKASWVHKTGSTGGFGSYVAFIPEKQIGIVMLANKSYPNPARVEAAYHILDAL..Q
ACT-24QSH12187.1_370EBCP      PVKASWVHKTGSTGGFGSYVAFIPEKQIGIVMLANKSYPNPARVEAAYHILDAL..Q
ACT-24QSH16801.1_370EBCP      PVKASWVHKTGSTGGFGSYVAFIPEKQIGIVMLANKSYPNPARVEAAYHILDAL..Q
ACT-24QSH07573.1_370EBCP      PVKASWVHKTGSTGGFGSYVAFIPEKQIGIVMLANKSYPNPARVEAAYHILDAL..Q
ACT-24QSH21409.1_370EBCP      PVKASWVHKTGSTGGFGSYVAFIPEKQIGIVMLANKSYPNPARVEAAYHILDAL..Q
ACT-24QSH26023.1_370EBCP      PVKASWVHKTGSTGGFGSYVAFIPEKQIGIVMLANKSYPNPARVEAAYHILDAL..Q
ACT-24QSH30639.1_370EBCP      PVKASWVHKTGSTGGFGSYVAFIPEKQIGIVMLANKSYPNPARVEAAYHILDAL..Q
ACT-24UIL48372.1_372EBCP      PVKASWVHKTGSTGGFGSYVAFIPEKQIGIVMLANKSYPNPARVEAAYHILDAL..Q
ACT-24UIM14687.1_372EBCP      PVKASWVHKTGSTGGFGSYVAFIPEKQIGIVMLANKSYPNPARVEAAYHILDAL..Q
ACT-24UXA74146.1_371EBCP      PVKASWVHKTGSTGGFGSYVAFIPEKQIGIVMLANKSYPNPARVEAAYHILDAL..Q
ACT-24WDY48721.1_372EBCP      PVKASWVHKTGSTGGFGSYVAFIPEKQIGIVMLANKSYPNPARVEAAYHILDAL..Q
ACT-24ASB73507.1_1185EBOC     PVKASWVHKTGSTGGFGSYVAFIPEKQIGIVMLANKSYPNPARVEAAYHILDAL..Q
ACT-24AVU18445.1_378EBOC      PVKASWVHKTGSTGGFGSYVAFIPEKQIGIVMLANKSYPNPARVEAAYHILDAL..Q
ACT-24QHC75881.1_374EBOC      PVKASWVHKTGSTGGFGSYVAFIPEKQIGIVMLANKSYPNPARVEAAYHILDAL..Q
ACT-24QLP84500.1_370EBOC      PVKASWVHKTGSTGGFGSYVAFIPEKQIGIVMLANKSYPNPARVEAAYHILDAL..Q
ACT-24QSH03071.1_370EBOC      PVKASWVHKTGSTGGFGSYVAFIPEKQIGIVMLANKSYPNPARVEAAYHILDAL..Q
ACT-24QSG93844.1_370EBOC      PVKASWVHKTGSTGGFGSYVAFIPEKQIGIVMLANKSYPNPARVEAAYHILDAL..Q
ACT-24QSG98466.1_370EBOC      PVKASWVHKTGSTGGFGSYVAFIPEKQIGIVMLANKSYPNPARVEAAYHILDAL..Q
ACT-24QSH12187.1_370EBOC      PVKASWVHKTGSTGGFGSYVAFIPEKQIGIVMLANKSYPNPARVEAAYHILDAL..Q
ACT-24QSH16801.1_370EBOC      PVKASWVHKTGSTGGFGSYVAFIPEKQIGIVMLANKSYPNPARVEAAYHILDAL..Q
ACT-24QSH07573.1_370EBOC      PVKASWVHKTGSTGGFGSYVAFIPEKQIGIVMLANKSYPNPARVEAAYHILDAL..Q
ACT-24QSH21409.1_370EBOC      PVKASWVHKTGSTGGFGSYVAFIPEKQIGIVMLANKSYPNPARVEAAYHILDAL..Q
ACT-24QSH26023.1_370EBOC      PVKASWVHKTGSTGGFGSYVAFIPEKQIGIVMLANKSYPNPARVEAAYHILDAL..Q
ACT-24QSH30639.1_370EBOC      PVKASWVHKTGSTGGFGSYVAFIPEKQIGIVMLANKSYPNPARVEAAYHILDAL..Q
ACT-24UIL48372.1_372EBOC      PVKASWVHKTGSTGGFGSYVAFIPEKQIGIVMLANKSYPNPARVEAAYHILDAL..Q
ACT-24UIM14687.1_372EBOC      PVKASWVHKTGSTGGFGSYVAFIPEKQIGIVMLANKSYPNPARVEAAYHILDAL..Q
ACT-24UXA74146.1_371EBOC      PVKASWVHKTGSTGGFGSYVAFIPEKQIGIVMLANKSYPNPARVEAAYHILDAL..Q
ACT-24WDY48721.1_372EBOC      PVKASWVHKTGSTGGFGSYVAFIPEKQIGIVMLANKSYPNPARVEAAYHILDAL..Q
ACT-89AVE73204.1_2453EBCP     PVKASWVHKTGSTGGFGSYVAFIPEKQIGIVMLANTSYPNPARVEAAYHILEAL..Q
ACT-89AVE73204.1_2453EBOC     PVKASWVHKTGSTGGFGSYVAFIPEKQIGIVMLANTSYPNPARVEAAYHILEAL..Q
ACT-56ASO99603.1_1270EBCP     PVKASWVHKTGSTGGFGSYVAFIPEKQIGIVMLANTSYPNPARVEAAYHILEAL..Q
ACT-56QDQ75241.1_392EBCP      PVKASWVHKTGSTGGFGSYVAFIPEKQIGIVMLANTSYPNPARVEAAYHILEAL..Q
ACT-56QFH98918.1_395EBCP      PVKASWVHKTGSTGGFGSYVAFIPEKQIGIVMLANTSYPNPARVEAAYHILEAL..Q
ACT-56QFH34334.1_395EBCP      PVKASWVHKTGSTGGFGSYVAFIPEKQIGIVMLANTSYPNPARVEAAYHILEAL..Q
ACT-56QFI32707.1_2233EBCP     PVKASWVHKTGSTGGFGSYVAFIPEKQIGIVMLANTSYPNPARVEAAYHILEAL..Q
ACT-56QMI65633.1_4184EBCP     PVKASWVHKTGSTGGFGSYVAFIPEKQIGIVMLANTSYPNPARVEAAYHILEAL..Q
ACT-56QMI71192.1_422EBCP      PVKASWVHKTGSTGGFGSYVAFIPEKQIGIVMLANTSYPNPARVEAAYHILEAL..Q
ACT-56UDV17856.1_8EBCP        PVKASWVHKTGSTGGFGSYVAFIPEKQIGIVMLANTSYPNPARVEAAYHILEAL..Q
ACT-56UDV39132.1_1376EBCP     PVKASWVHKTGSTGGFGSYVAFIPEKQIGIVMLANTSYPNPARVEAAYHILEAL..Q
ACT-56UWB10427.1_430EBCP      PVKASWVHKTGSTGGFGSYVAFIPEKQIGIVMLANTSYPNPARVEAAYHILEAL..Q
ACT-56UWC18315.1_415EBCP      PVKASWVHKTGSTGGFGSYVAFIPEKQIGIVMLANTSYPNPARVEAAYHILEAL..Q
ACT-56UWC23093.1_417EBCP      PVKASWVHKTGSTGGFGSYVAFIPEKQIGIVMLANTSYPNPARVEAAYHILEAL..Q
ACT-56WBT12607.1_393EBCP      PVKASWVHKTGSTGGFGSYVAFIPEKQIGIVMLANTSYPNPARVEAAYHILEAL..Q
ACT-56WBT28831.1_393EBCP      PVKASWVHKTGSTGGFGSYVAFIPEKQIGIVMLANTSYPNPARVEAAYHILEAL..Q
ACT-56WGB34617.1_392EBCP      PVKASWVHKTGSTGGFGSYVAFIPEKQIGIVMLANTSYPNPARVEAAYHILEAL..Q
ACT-56WGU03466.1_393EBCP      PVKASWVHKTGSTGGFGSYVAFIPEKQIGIVMLANTSYPNPARVEAAYHILEAL..Q
ACT-56ASO99603.1_1270EBOC     PVKASWVHKTGSTGGFGSYVAFIPEKQIGIVMLANTSYPNPARVEAAYHILEAL..Q
ACT-56QDQ75241.1_392EBOC      PVKASWVHKTGSTGGFGSYVAFIPEKQIGIVMLANTSYPNPARVEAAYHILEAL..Q
ACT-56QFH98918.1_395EBOC      PVKASWVHKTGSTGGFGSYVAFIPEKQIGIVMLANTSYPNPARVEAAYHILEAL..Q
ACT-56QFH34334.1_395EBOC      PVKASWVHKTGSTGGFGSYVAFIPEKQIGIVMLANTSYPNPARVEAAYHILEAL..Q
ACT-56QFI32707.1_2233EBOC     PVKASWVHKTGSTGGFGSYVAFIPEKQIGIVMLANTSYPNPARVEAAYHILEAL..Q
ACT-56QMI65633.1_4184EBOC     PVKASWVHKTGSTGGFGSYVAFIPEKQIGIVMLANTSYPNPARVEAAYHILEAL..Q
ACT-56QMI71192.1_422EBOC      PVKASWVHKTGSTGGFGSYVAFIPEKQIGIVMLANTSYPNPARVEAAYHILEAL..Q
ACT-56UDV17856.1_8EBOC        PVKASWVHKTGSTGGFGSYVAFIPEKQIGIVMLANTSYPNPARVEAAYHILEAL..Q
ACT-56UDV39132.1_1376EBOC     PVKASWVHKTGSTGGFGSYVAFIPEKQIGIVMLANTSYPNPARVEAAYHILEAL..Q
ACT-56UWB10427.1_430EBOC      PVKASWVHKTGSTGGFGSYVAFIPEKQIGIVMLANTSYPNPARVEAAYHILEAL..Q
ACT-56UWC18315.1_415EBOC      PVKASWVHKTGSTGGFGSYVAFIPEKQIGIVMLANTSYPNPARVEAAYHILEAL..Q
ACT-56UWC23093.1_417EBOC      PVKASWVHKTGSTGGFGSYVAFIPEKQIGIVMLANTSYPNPARVEAAYHILEAL..Q
ACT-56WBT12607.1_393EBOC      PVKASWVHKTGSTGGFGSYVAFIPEKQIGIVMLANTSYPNPARVEAAYHILEAL..Q
ACT-56WBT28831.1_393EBOC      PVKASWVHKTGSTGGFGSYVAFIPEKQIGIVMLANTSYPNPARVEAAYHILEAL..Q
ACT-56WGB34617.1_392EBOC      PVKASWVHKTGSTGGFGSYVAFIPEKQIGIVMLANTSYPNPARVEAAYHILEAL..Q
ACT-56WGU03466.1_393EBOC      PVKASWVHKTGSTGGFGSYVAFIPEKQIGIVMLANTSYPNPARVEAAYHILEAL..Q
ACT-56AOP76464.1_392EOC       PVKASWVHKTGSTGGFGSYVAFIPEKQIGIVMLANTSYPNPARVEAAYHILEAL..Q
ACT-56QXA77046.1_2672EOC      PVKASWVHKTGSTGGFGSYVAFIPEKQIGIVMLANTSYPNPARVEAAYHILEAL..Q
ACT-56WFP29817.1_397EOC       PVKASWVHKTGSTGGFGSYVAFIPEKQIGIVMLANTSYPNPARVEAAYHILEAL..Q
ACT-27AXO49795.1_1610EBCP     PVKASWVHKTGSTGGFGSYVAFIPEKQIGIVMLANTSYPNPARVEAAYHILEAL..Q
ACT-27QHO79915.1_457EBCP      PVKASWVHKTGSTGGFGSYVAFIPEKQIGIVMLANTSYPNPARVEAAYHILEAL..Q
ACT-27QHO97012.1_458EBCP      PVKASWVHKTGSTGGFGSYVAFIPEKQIGIVMLANTSYPNPARVEAAYHILEAL..Q
ACT-27QQH03712.1_3802EBCP     PVKASWVHKTGSTGGFGSYVAFIPEKQIGIVMLANTSYPNPARVEAAYHILEAL..Q
ACT-27QQH08784.1_3802EBCP     PVKASWVHKTGSTGGFGSYVAFIPEKQIGIVMLANTSYPNPARVEAAYHILEAL..Q
ACT-27UVZ91365.1_388EBCP      PVKASWVHKTGSTGGFGSYVAFIPEKQIGIVMLANTSYPNPARVEAAYHILEAL..Q
ACT-27AXO49795.1_1610EBOC     PVKASWVHKTGSTGGFGSYVAFIPEKQIGIVMLANTSYPNPARVEAAYHILEAL..Q
ACT-27QHO79915.1_457EBOC      PVKASWVHKTGSTGGFGSYVAFIPEKQIGIVMLANTSYPNPARVEAAYHILEAL..Q
ACT-27QHO97012.1_458EBOC      PVKASWVHKTGSTGGFGSYVAFIPEKQIGIVMLANTSYPNPARVEAAYHILEAL..Q
ACT-27QQH03712.1_3802EBOC     PVKASWVHKTGSTGGFGSYVAFIPEKQIGIVMLANTSYPNPARVEAAYHILEAL..Q
ACT-27QQH08784.1_3802EBOC     PVKASWVHKTGSTGGFGSYVAFIPEKQIGIVMLANTSYPNPARVEAAYHILEAL..Q
ACT-27UVZ91365.1_388EBOC      PVKASWVHKTGSTGGFGSYVAFIPEKQIGIVMLANTSYPNPARVEAAYHILEAL..Q
ACT-102QEO03155.1_4213EBCP   PVKASWVHKTGSTGGFGSYVAFIPEKELGIVMLANKSYPNPARVEAAYRILSAL..Q
ACT-102BBV79841.1_51928]EBCP PVKASWVHKTGSTGGFGSYVAFIPEKELGIVMLANKSYPNPARVEAAYRILSAL..Q
ACT-102QZS47739.1_401EBCP    PVKASWVHKTGSTGGFGSYVAFIPEKELGIVMLANKSYPNPARVEAAYRILSAL..Q
ACT-102QEO03155.1_4213EBOC   PVKASWVHKTGSTGGFGSYVAFIPEKELGIVMLANKSYPNPARVEAAYRILSAL..Q
ACT-102BBV79841.1_51928]EBOC PVKASWVHKTGSTGGFGSYVAFIPEKELGIVMLANKSYPNPARVEAAYRILSAL..Q
ACT-102QZS47739.1_401EBOC    PVKASWVHKTGSTGGFGSYVAFIPEKELGIVMLANKSYPNPARVEAAYRILSAL..Q
ACT-55QLP71015.1_413EBCP      PVKASWVHKTGSTGGFGSYVAFIPEKQIGIVMLANKSYPNPARVEAAYHILEAL..Q
ACT-55QLP80034.1_414EBCP      PVKASWVHKTGSTGGFGSYVAFIPEKQIGIVMLANKSYPNPARVEAAYHILEAL..Q
ACT-55QLS08818.1_415EBCP      PVKASWVHKTGSTGGFGSYVAFIPEKQIGIVMLANKSYPNPARVEAAYHILEAL..Q
ACT-55QLT25377.1_3621EBCP     PVKASWVHKTGSTGGFGSYVAFIPEKQIGIVMLANKSYPNPARVEAAYHILEAL..Q
ACT-55QLV12510.1_4006EBCP     PVKASWVHKTGSTGGFGSYVAFIPEKQIGIVMLANKSYPNPARVEAAYHILEAL..Q
ACT-55QLW04180.1_4182EBCP     PVKASWVHKTGSTGGFGSYVAFIPEKQIGIVMLANKSYPNPARVEAAYHILEAL..Q
ACT-55QLV08026.1_4006EBCP     PVKASWVHKTGSTGGFGSYVAFIPEKQIGIVMLANKSYPNPARVEAAYHILEAL..Q
ACT-55QLV96171.1_421EBCP      PVKASWVHKTGSTGGFGSYVAFIPEKQIGIVMLANKSYPNPARVEAAYHILEAL..Q
ACT-55QLW44293.1_405EBCP      PVKASWVHKTGSTGGFGSYVAFIPEKQIGIVMLANKSYPNPARVEAAYHILEAL..Q
ACT-55UBH32480.1_675EBCP      PVKASWVHKTGSTGGFGSYVAFIPEKQIGIVMLANKSYPNPARVEAAYHILEAL..Q
ACT-55UDV61223.1_2251EBCP     PVKASWVHKTGSTGGFGSYVAFIPEKQIGIVMLANKSYPNPARVEAAYHILEAL..Q
ACT-55QLP71015.1_413EBOC      PVKASWVHKTGSTGGFGSYVAFIPEKQIGIVMLANKSYPNPARVEAAYHILEAL..Q
ACT-55QLP80034.1_414EBOC      PVKASWVHKTGSTGGFGSYVAFIPEKQIGIVMLANKSYPNPARVEAAYHILEAL..Q
ACT-55QLS08818.1_415EBOC      PVKASWVHKTGSTGGFGSYVAFIPEKQIGIVMLANKSYPNPARVEAAYHILEAL..Q
ACT-55QLT25377.1_3621EBOC     PVKASWVHKTGSTGGFGSYVAFIPEKQIGIVMLANKSYPNPARVEAAYHILEAL..Q
ACT-55QLV12510.1_4006EBOC     PVKASWVHKTGSTGGFGSYVAFIPEKQIGIVMLANKSYPNPARVEAAYHILEAL..Q
```

ACT-55QLW04180.1_4182EBOC	PVKASWVHKTGSTGGFGSYVAFIPEKQIGIVMLANKSYPNPARVEAAYHILEAL..Q
ACT-55QLV08026.1_4006EBOC	PVKASWVHKTGSTGGFGSYVAFIPEKQIGIVMLANKSYPNPARVEAAYHILEAL..Q
ACT-55QLV96171.1_421EBOC	PVKASWVHKTGSTGGFGSYVAFIPEKQIGIVMLANKSYPNPARVEAAYHILEAL..Q
ACT-55QLW44293.1_405EBOC	PVKASWVHKTGSTGGFGSYVAFIPEKQIGIVMLANKSYPNPARVEAAYHILEAL..Q
ACT-55UBH32480.1_675EBOC	PVKASWVHKTGSTGGFGSYVAFIPEKQIGIVMLANKSYPNPARVEAAYHILEAL..Q
ACT-55UDV61223.1_2251EBOC	PVKASWVHKTGSTGGFGSYVAFIPEKQIGIVMLANKSYPNPARVEAAYHILEAL..Q
ACT-55AOP80857.1_409EOC	PVKASWVHKTGSTGGFGSYVAFIPEKQIGIVMLANKSYPNPARVEAAYHILEAL..Q
ACT-51BBW20048.1_38351]EBCP	PVKASWVHKTGSTGGFGSYVAFIPEKELGIVMLANKSYPNPARVEAAYRILSAL..Q
ACT-51BBW20048.1_38351]EBOC	PVKASWVHKTGSTGGFGSYVAFIPEKELGIVMLANKSYPNPARVEAAYRILSAL..Q
ACT-28URE95606.1_351EBCP	PVKASWVHKTGSTGGFGSYVAFIPEKELGIVMLANKSYPNPARVEAAYRILSAL..Q
ACT-28URE95606.1_351EBOC	PVKASWVHKTGSTGGFGSYVAFIPEKELGIVMLANKSYPNPARVEAAYRILSAL..Q
ACT-87AYL04658.1_1249EBCP	PVKASWVHKTGSTGGFGSYVAFIPEKELGIVMLANKSYPNPARVEAAYRILSAL..Q
ACT-87QIP18425.1_352EBCP	PVKASWVHKTGSTGGFGSYVAFIPEKELGIVMLANKSYPNPARVEAAYRILSAL..Q
ACT-87AYL04658.1_1249EBOC	PVKASWVHKTGSTGGFGSYVAFIPEKELGIVMLANKSYPNPARVEAAYRILSAL..Q
ACT-87QIP18425.1_352EBOC	PVKASWVHKTGSTGGFGSYVAFIPEKELGIVMLANKSYPNPARVEAAYRILSAL..Q
ACT-75AWX01586.1_1447EBCP	PVKASWVHKTGSTGGFGSYVAFIPEKQVGIVMLANKSYPNPARVEAAYHILEAL..Q
ACT-75AWX01586.1_1447EBOC	PVKASWVHKTGSTGGFGSYVAFIPEKQVGIVMLANKSYPNPARVEAAYHILEAL..Q
ACT-103URL27837.1_342EOC	PVKASWVHKTGSTGGFGSYVAFIPEKELGIVMLANKSYPNPARVEAAYRILSAL..Q
ACT-99UXJ64669.1_346EBCP	PVKASWVHKTGSTGGFGSYVAFIPEKELGIVMLANKSYPNPARVEAAYRILSAL..Q
ACT-99UXJ64669.1_346EBOC	PVKASWVHKTGSTGGFGSYVAFIPEKELGIVMLANKSYPNPARVEAAYRILSAL..Q
ACT-95QIB80518.1_348EBCP	PVKASWVHKTGSTGGFGSYVAFIPEKELGIVMLANKSYPNPARVEAAYRILSAL..Q
ACT-95BBV69349.1_35128]EBCP	PVKASWVHKTGSTGGFGSYVAFIPEKELGIVMLANKSYPNPARVEAAYRILSAL..Q
ACT-95UZQ68133.1_388EBCP	PVKASWVHKTGSTGGFGSYVAFIPEKELGIVMLANKSYPNPARVEAAYRILSAL..Q
ACT-95QIB80518.1_348EBOC	PVKASWVHKTGSTGGFGSYVAFIPEKELGIVMLANKSYPNPARVEAAYRILSAL..Q
ACT-95BBV69349.1_35128]EBOC	PVKASWVHKTGSTGGFGSYVAFIPEKELGIVMLANKSYPNPARVEAAYRILSAL..Q
ACT-95UZQ68133.1_388EBOC	PVKASWVHKTGSTGGFGSYVAFIPEKELGIVMLANKSYPNPARVEAAYRILSAL..Q
ACT-52QFH88560.1_350EBCP	PVKASWVHKTGSTGGFGSYVAFIPEKELGIVMLANKSYPNPARVEAAYRILSAL..Q
ACT-52BBS30224.1_43052]EBCP	PVKASWVHKTGSTGGFGSYVAFIPEKELGIVMLANKSYPNPARVEAAYRILSAL..Q
ACT-52WBN86316.1_4083EBCP	PVKASWVHKTGSTGGFGSYVAFIPEKELGIVMLANKSYPNPARVEAAYRILSAL..Q
ACT-52QFH88560.1_350EBOC	PVKASWVHKTGSTGGFGSYVAFIPEKELGIVMLANKSYPNPARVEAAYRILSAL..Q
ACT-52BBS30224.1_43052]EBOC	PVKASWVHKTGSTGGFGSYVAFIPEKELGIVMLANKSYPNPARVEAAYRILSAL..Q
ACT-52WBN86316.1_4083EBOC	PVKASWVHKTGSTGGFGSYVAFIPEKELGIVMLANKSYPNPARVEAAYRILSAL..Q
ACT-4QLR27288.1_395EBCP	PVNASWVHKTGSTGGFGSYVAFIPEKQLGIVMLANKSYPNPARVEAAYRILSAL...
ACT-4QPS68257.1_413EBCP	PVNASWVHKTGSTGGFGSYVAFIPEKQLGIVMLANKSYPNPARVEAAYRILSAL...
ACT-4QLR27288.1_395EBOC	PVNASWVHKTGSTGGFGSYVAFIPEKQLGIVMLANKSYPNPARVEAAYRILSAL...
ACT-4QPS68257.1_413EBOC	PVNASWVHKTGSTGGFGSYVAFIPEKQLGIVMLANKSYPNPARVEAAYRILSAL...
ACT-3UOY53510.1_358EBCP	PVNASWVHKTGSTGGFGSYVAFIPEKQLGIVMLANKSYPNPARVEAAYRILSAL...
ACT-3UOY63785.1_4171EBCP	PVNASWVHKTGSTGGFGSYVAFIPEKQLGIVMLANKSYPNPARVEAAYRILSAL...
ACT-3UOY53510.1_358EBOC	PVNASWVHKTGSTGGFGSYVAFIPEKQLGIVMLANKSYPNPARVEAAYRILSAL...
ACT-3UOY63785.1_4171EBOC	PVNASWVHKTGSTGGFGSYVAFIPEKQLGIVMLANKSYPNPARVEAAYRILSAL...
ACT-1QLV61731.1_472EBCP	PVNASWVHKTGSTGGFGSYVAFIPEKQLGIVMLANKSYPNPARVEAAYRILSAL...
ACT-1UWA77567.1_382EBCP	PVNASWVHKTGSTGGFGSYVAFIPEKQLGIVMLANKSYPNPARVEAAYRILSAL...
ACT-1QLV61731.1_472EBOC	PVNASWVHKTGSTGGFGSYVAFIPEKQLGIVMLANKSYPNPARVEAAYRILSAL...
ACT-1UWA77567.1_382EBOC	PVNASWVHKTGSTGGFGSYVAFIPEKQLGIVMLANKSYPNPARVEAAYRILSAL...
ACT-77QWZ50133.1_330EBCP	PVKASWVHKTGSTGGFGSYVAFIPEKQLGIVMLANKSYPNPARVEAAYRILDAL..Q
ACT-77UBH39796.1_3999EBCP	PVKASWVHKTGSTGGFGSYVAFIPEKQLGIVMLANKSYPNPARVEAAYRILDAL..Q
ACT-77UBH98103.1_3842EBCP	PVKASWVHKTGSTGGFGSYVAFIPEKQLGIVMLANKSYPNPARVEAAYRILDAL..Q
ACT-77UBH94108.1_193EBCP	PVKASWVHKTGSTGGFGSYVAFIPEKQLGIVMLANKSYPNPARVEAAYRILDAL..Q
ACT-77QWZ50133.1_330EBOC	PVKASWVHKTGSTGGFGSYVAFIPEKQLGIVMLANKSYPNPARVEAAYRILDAL..Q
ACT-77UBH39796.1_3999EBOC	PVKASWVHKTGSTGGFGSYVAFIPEKQLGIVMLANKSYPNPARVEAAYRILDAL..Q
ACT-77UBH98103.1_3842EBOC	PVKASWVHKTGSTGGFGSYVAFIPEKQLGIVMLANKSYPNPARVEAAYRILDAL..Q
ACT-77UBH94108.1_193EBOC	PVKASWVHKTGSTGGFGSYVAFIPEKQLGIVMLANKSYPNPARVEAAYRILDAL..Q
ACT-53QEL38380.1_485EBCP	PVKASWVHKTGSTGGFGSYVAFIPEKNLGIVMLANKSYPNPARVEAAYRILDAL..Q
ACT-53QEL38380.1_485EBOC	PVKASWVHKTGSTGGFGSYVAFIPEKNLGIVMLANKSYPNPARVEAAYRILDAL..Q
ACT-57QBB07838.1_4703EBCP	PVNASWVHKTGSTGGFGSYVAFIPEKQLGIVMLANKSYPNPARVEAAYRILDAL..Q
ACT-57QYD27335.1_499EBCP	PVNASWVHKTGSTGGFGSYVAFIPEKQLGIVMLANKSYPNPARVEAAYRILDAL..Q
ACT-57UBM17197.1_2672EBCP	PVNASWVHKTGSTGGFGSYVAFIPEKQLGIVMLANKSYPNPARVEAAYRILDAL..Q
ACT-57UKB55021.1_380EBCP	PVNASWVHKTGSTGGFGSYVAFIPEKQLGIVMLANKSYPNPARVEAAYRILDAL..Q
ACT-57QBB07838.1_4703EBOC	PVNASWVHKTGSTGGFGSYVAFIPEKQLGIVMLANKSYPNPARVEAAYRILDAL..Q
ACT-57QYD27335.1_499EBOC	PVNASWVHKTGSTGGFGSYVAFIPEKQLGIVMLANKSYPNPARVEAAYRILDAL..Q
ACT-57UBM17197.1_2672EBOC	PVNASWVHKTGSTGGFGSYVAFIPEKQLGIVMLANKSYPNPARVEAAYRILDAL..Q
ACT-57UKB55021.1_380EBOC	PVNASWVHKTGSTGGFGSYVAFIPEKQLGIVMLANKSYPNPARVEAAYRILDAL..Q
ACT-93ALL15984.1_396EOC	PVNASWVHKTGSTGGFGSYVAFIPEKQLGIVMLANKSYPNPARVEAAYRILDAL..Q
ACT-9AOP85131.1_312EBCP	PVKASWVHKTGSTGGFGSYVAFIPEKELGIVMLANKSYPNPARVEAAYRILSAL..Q
ACT-9UOY32628.1_343EBCP	PVKASWVHKTGSTGGFGSYVAFIPEKELGIVMLANKSYPNPARVEAAYRILSAL..Q
ACT-9AOP85131.1_312EBOC	PVKASWVHKTGSTGGFGSYVAFIPEKELGIVMLANKSYPNPARVEAAYRILSAL..Q
ACT-9UOY32628.1_343EBOC	PVKASWVHKTGSTGGFGSYVAFIPEKELGIVMLANKSYPNPARVEAAYRILSAL..Q
ACT-9AMZ75637.1_173EOC	PVKASWVHKTGSTGGFGSYVAFIPEKELGIVMLANKSYPNPARVEAAYRILSAL..Q
ACT-6BBT43181.1_3396]EBCP	PVNASWVHKTGSTGGFGSYVAFIPEKQLGIVMLANKSYPNPARVEAAYRILDAL..Q
ACT-6BBT43181.1_3396]EBOC	PVNASWVHKTGSTGGFGSYVAFIPEKQLGIVMLANKSYPNPARVEAAYRILDAL..Q
ACT-46QUP91302.1_387EBCP	PVKASWVHKTGSTGGFGSYVAFIPEKQIGIVMLANKSYPNPARVEAAYHILEAL..Q
ACT-46QUP91302.1_387EBOC	PVKASWVHKTGSTGGFGSYVAFIPEKQIGIVMLANKSYPNPARVEAAYHILEAL..Q
ACT-67QLP16520.1_400EBCP	PVKASWVHKTGSTGGFGSYVAFIPEKQIGIVMLANKSYPNPARVEAAYHILDAL..Q
ACT-67QLP16520.1_400EBOC	PVKASWVHKTGSTGGFGSYVAFIPEKQIGIVMLANKSYPNPARVEAAYHILDAL..Q
ACT-84QOR49295.1_413EBCP	PVKASWVHKTGSTGGFGSYVAFIPEKQIGIVMLANKSYPNPARVEAAYHILEAL..Q
ACT-84QOR49295.1_413EBOC	PVKASWVHKTGSTGGFGSYVAFIPEKQIGIVMLANKSYPNPARVEAAYHILEAL..Q
ACT-74QOX68946.1_496EBCP	PVKASWVHKTGSTGGFGSYVAFIPEKQIGIVMLANTSYPNPARVEAAYHILEAL..Q
ACT-74UIZ04332.1_460EBCP	PVKASWVHKTGSTGGFGSYVAFIPEKQIGIVMLANTSYPNPARVEAAYHILEAL..Q
ACT-74QOX68946.1_496EBOC	PVKASWVHKTGSTGGFGSYVAFIPEKQIGIVMLANTSYPNPARVEAAYHILEAL..Q
ACT-74UIZ04332.1_460EBOC	PVKASWVHKTGSTGGFGSYVAFIPEKQIGIVMLANTSYPNPARVEAAYHILEAL..Q
ACT-74QQT40108.1_4574EOC	PVKASWVHKTGSTGGFGSYVAFIPEKQIGIVMLANTSYPNPARVEAAYHILEAL..Q
ACT-16ATW90451.1_311EBCP	PVKASWVHKTGSTGGFGSYVAFIPEKQIGIVMLANKSYPNPARVEAAYHILEAL..Q
ACT-16AVO82105.1_1257EBCP	PVKASWVHKTGSTGGFGSYVAFIPEKQIGIVMLANKSYPNPARVEAAYHILEAL..Q
ACT-16AWQ41825.1_393EBCP	PVKASWVHKTGSTGGFGSYVAFIPEKQIGIVMLANKSYPNPARVEAAYHILEAL..Q
ACT-16AWQ56038.1_393EBCP	PVKASWVHKTGSTGGFGSYVAFIPEKQIGIVMLANKSYPNPARVEAAYHILEAL..Q
ACT-16QFH53420.1_474EBCP	PVKASWVHKTGSTGGFGSYVAFIPEKQIGIVMLANKSYPNPARVEAAYHILEAL..Q
ACT-16QPO90952.1_396EBCP	PVKASWVHKTGSTGGFGSYVAFIPEKQIGIVMLANKSYPNPARVEAAYHILEAL..Q
ACT-16QQA67679.1_472EBCP	PVKASWVHKTGSTGGFGSYVAFIPEKQIGIVMLANKSYPNPARVEAAYHILEAL..Q
ACT-16UDV14809.1_2173EBCP	PVKASWVHKTGSTGGFGSYVAFIPEKQIGIVMLANKSYPNPARVEAAYHILEAL..Q
ACT-16UDV41468.1_3105EBCP	PVKASWVHKTGSTGGFGSYVAFIPEKQIGIVMLANKSYPNPARVEAAYHILEAL..Q
ACT-16UIZ08994.1_395EBCP	PVKASWVHKTGSTGGFGSYVAFIPEKQIGIVMLANKSYPNPARVEAAYHILEAL..Q
ACT-16WJJ28417.1_386EBCP	PVKASWVHKTGSTGGFGSYVAFIPEKQIGIVMLANKSYPNPARVEAAYHILEAL..Q
ACT-16ATW90451.1_311EBOC	PVKASWVHKTGSTGGFGSYVAFIPEKQIGIVMLANKSYPNPARVEAAYHILEAL..Q
ACT-16AVO82105.1_1257EBOC	PVKASWVHKTGSTGGFGSYVAFIPEKQIGIVMLANKSYPNPARVEAAYHILEAL..Q
ACT-16AWQ41825.1_393EBOC	PVKASWVHKTGSTGGFGSYVAFIPEKQIGIVMLANKSYPNPARVEAAYHILEAL..Q
ACT-16AWQ56038.1_393EBOC	PVKASWVHKTGSTGGFGSYVAFIPEKQIGIVMLANKSYPNPARVEAAYHILEAL..Q
ACT-16QFH53420.1_474EBOC	PVKASWVHKTGSTGGFGSYVAFIPEKQIGIVMLANKSYPNPARVEAAYHILEAL..Q
ACT-16QPO90952.1_396EBOC	PVKASWVHKTGSTGGFGSYVAFIPEKQIGIVMLANKSYPNPARVEAAYHILEAL..Q
ACT-16QQA67679.1_472EBOC	PVKASWVHKTGSTGGFGSYVAFIPEKQIGIVMLANKSYPNPARVEAAYHILEAL..Q
ACT-16UDV14809.1_2173EBOC	PVKASWVHKTGSTGGFGSYVAFIPEKQIGIVMLANKSYPNPARVEAAYHILEAL..Q
ACT-16UDV41468.1_3105EBOC	PVKASWVHKTGSTGGFGSYVAFIPEKQIGIVMLANKSYPNPARVEAAYHILEAL..Q
ACT-16UIZ08994.1_395EBOC	PVKASWVHKTGSTGGFGSYVAFIPEKQIGIVMLANKSYPNPARVEAAYHILEAL..Q
ACT-16WJJ28417.1_386EBOC	PVKASWVHKTGSTGGFGSYVAFIPEKQIGIVMLANKSYPNPARVEAAYHILEAL..Q

```
ACT-23QLP51667.1_388EBCP      PVKASWVHKTGSTGGFGSYVAFIPEKQIGIVMLANKSYPNPARVEAAYHILDAL..Q
ACT-23QLP61663.1_388EBCP      PVKASWVHKTGSTGGFGSYVAFIPEKQIGIVMLANKSYPNPARVEAAYHILDAL..Q
ACT-23QLP66427.1_430EBCP      PVKASWVHKTGSTGGFGSYVAFIPEKQIGIVMLANKSYPNPARVEAAYHILDAL..Q
ACT-23QLU80799.1_388EBCP      PVKASWVHKTGSTGGFGSYVAFIPEKQIGIVMLANKSYPNPARVEAAYHILDAL..Q
ACT-23QLT97825.1_367EBCP      PVKASWVHKTGSTGGFGSYVAFIPEKQIGIVMLANKSYPNPARVEAAYHILDAL..Q
ACT-23QLU89187.1_4307EBCP     PVKASWVHKTGSTGGFGSYVAFIPEKQIGIVMLANKSYPNPARVEAAYHILDAL..Q
ACT-23QLV76440.1_389EBCP      PVKASWVHKTGSTGGFGSYVAFIPEKQIGIVMLANKSYPNPARVEAAYHILDAL..Q
ACT-23QLT57991.1_396EBCP      PVKASWVHKTGSTGGFGSYVAFIPEKQIGIVMLANKSYPNPARVEAAYHILDAL..Q
ACT-23QLV44058.1_369EBCP      PVKASWVHKTGSTGGFGSYVAFIPEKQIGIVMLANKSYPNPARVEAAYHILDAL..Q
ACT-23QLP51667.1_388EBOC      PVKASWVHKTGSTGGFGSYVAFIPEKQIGIVMLANKSYPNPARVEAAYHILDAL..Q
ACT-23QLP61663.1_388EBOC      PVKASWVHKTGSTGGFGSYVAFIPEKQIGIVMLANKSYPNPARVEAAYHILDAL..Q
ACT-23QLP66427.1_430EBOC      PVKASWVHKTGSTGGFGSYVAFIPEKQIGIVMLANKSYPNPARVEAAYHILDAL..Q
ACT-23QLU80799.1_388EBOC      PVKASWVHKTGSTGGFGSYVAFIPEKQIGIVMLANKSYPNPARVEAAYHILDAL..Q
ACT-23QLT97825.1_367EBOC      PVKASWVHKTGSTGGFGSYVAFIPEKQIGIVMLANKSYPNPARVEAAYHILDAL..Q
ACT-23QLU89187.1_4307EBOC     PVKASWVHKTGSTGGFGSYVAFIPEKQIGIVMLANKSYPNPARVEAAYHILDAL..Q
ACT-23QLV76440.1_389EBOC      PVKASWVHKTGSTGGFGSYVAFIPEKQIGIVMLANKSYPNPARVEAAYHILDAL..Q
ACT-23QLT57991.1_396EBOC      PVKASWVHKTGSTGGFGSYVAFIPEKQIGIVMLANKSYPNPARVEAAYHILDAL..Q
ACT-23QLV44058.1_369EBOC      PVKASWVHKTGSTGGFGSYVAFIPEKQIGIVMLANKSYPNPARVEAAYHILDAL..Q
ACT-115AXQ35263.1_2972EBCP    PVKASWVHKTGSTGGFGSYVAFIPEKQIGIVMLANKSYPNPARVEAAYHILKAL..Q
ACT-115QGR11187.1_384EBCP     PVKASWVHKTGSTGGFGSYVAFIPEKQIGIVMLANKSYPNPARVEAAYHILKAL..Q
ACT-115QIF26741.1_2106EBCP    PVKASWVHKTGSTGGFGSYVAFIPEKQIGIVMLANKSYPNPARVEAAYHILKAL..Q
ACT-115QIF32246.1_3030EBCP    PVKASWVHKTGSTGGFGSYVAFIPEKQIGIVMLANKSYPNPARVEAAYHILKAL..Q
ACT-115QOD35429.1_385EBCP     PVKASWVHKTGSTGGFGSYVAFIPEKQIGIVMLANKSYPNPARVEAAYHILKAL..Q
ACT-115QPD66621.1_385EBCP     PVKASWVHKTGSTGGFGSYVAFIPEKQIGIVMLANKSYPNPARVEAAYHILKAL..Q
ACT-115QSS35407.1_385EBCP     PVKASWVHKTGSTGGFGSYVAFIPEKQIGIVMLANKSYPNPARVEAAYHILKAL..Q
ACT-115URE88756.1_385EBCP     PVKASWVHKTGSTGGFGSYVAFIPEKQIGIVMLANKSYPNPARVEAAYHILKAL..Q
ACT-115USM81982.1_384EBCP     PVKASWVHKTGSTGGFGSYVAFIPEKQIGIVMLANKSYPNPARVEAAYHILKAL..Q
ACT-115AXQ35263.1_2972EBOC    PVKASWVHKTGSTGGFGSYVAFIPEKQIGIVMLANKSYPNPARVEAAYHILKAL..Q
ACT-115QGR11187.1_384EBOC     PVKASWVHKTGSTGGFGSYVAFIPEKQIGIVMLANKSYPNPARVEAAYHILKAL..Q
ACT-115QIF26741.1_2106EBOC    PVKASWVHKTGSTGGFGSYVAFIPEKQIGIVMLANKSYPNPARVEAAYHILKAL..Q
ACT-115QIF32246.1_3030EBOC    PVKASWVHKTGSTGGFGSYVAFIPEKQIGIVMLANKSYPNPARVEAAYHILKAL..Q
ACT-115QOD35429.1_385EBOC     PVKASWVHKTGSTGGFGSYVAFIPEKQIGIVMLANKSYPNPARVEAAYHILKAL..Q
ACT-115QPD66621.1_385EBOC     PVKASWVHKTGSTGGFGSYVAFIPEKQIGIVMLANKSYPNPARVEAAYHILKAL..Q
ACT-115QSS35407.1_385EBOC     PVKASWVHKTGSTGGFGSYVAFIPEKQIGIVMLANKSYPNPARVEAAYHILKAL..Q
ACT-115URE88756.1_385EBOC     PVKASWVHKTGSTGGFGSYVAFIPEKQIGIVMLANKSYPNPARVEAAYHILKAL..Q
ACT-115USM81982.1_384EBOC     PVKASWVHKTGSTGGFGSYVAFIPEKQIGIVMLANKSYPNPARVEAAYHILKAL..Q
ACT-25AVU48988.1_427EBCP      PVKASWVHKTGSTGGFGSYVAFIPEKQIGIVMLANKSYPNPARVEAAYHILKAL..Q
ACT-25UQQ46893.1_379EBCP      PVKASWVHKTGSTGGFGSYVAFIPEKQIGIVMLANKSYPNPARVEAAYHILKAL..Q
ACT-25WDO17280.1_310EBCP      PVKASWVHKTGSTGGFGSYVAFIPEKQIGIVMLANKSYPNPARVEAAYHILKAL..Q
ACT-25AVU48988.1_427EBOC      PVKASWVHKTGSTGGFGSYVAFIPEKQIGIVMLANKSYPNPARVEAAYHILKAL..Q
ACT-25UQQ46893.1_379EBOC      PVKASWVHKTGSTGGFGSYVAFIPEKQIGIVMLANKSYPNPARVEAAYHILKAL..Q
ACT-25WDO17280.1_310EBOC      PVKASWVHKTGSTGGFGSYVAFIPEKQIGIVMLANKSYPNPARVEAAYHILKAL..Q
ACT-25AOP89724.1_377EOC       PVKASWVHKTGSTGGFGSYVAFIPEKQIGIVMLANKSYPNPARVEAAYHILKAL..Q
ACT-45APR43249.1_2902EBCP     PVKASWVHKTGSTGGFGSYVAFIPEKQIGIVMLANKSYPNPARVEAAYHILKAL..Q
ACT-45ASA05885.1_3853EBCP     PVKASWVHKTGSTGGFGSYVAFIPEKQIGIVMLANKSYPNPARVEAAYHILKAL..Q
ACT-45ASB85359.1_3592EBCP     PVKASWVHKTGSTGGFGSYVAFIPEKQIGIVMLANKSYPNPARVEAAYHILKAL..Q
ACT-45AWR70714.1_4154EBCP     PVKASWVHKTGSTGGFGSYVAFIPEKQIGIVMLANKSYPNPARVEAAYHILKAL..Q
ACT-45AXM01511.1_4138EBCP     PVKASWVHKTGSTGGFGSYVAFIPEKQIGIVMLANKSYPNPARVEAAYHILKAL..Q
ACT-45AYU96034.1_2649EBCP     PVKASWVHKTGSTGGFGSYVAFIPEKQIGIVMLANKSYPNPARVEAAYHILKAL..Q
ACT-45QBH65043.1_3960EBCP     PVKASWVHKTGSTGGFGSYVAFIPEKQIGIVMLANKSYPNPARVEAAYHILKAL..Q
ACT-45QEL31100.1_1855EBCP     PVKASWVHKTGSTGGFGSYVAFIPEKQIGIVMLANKSYPNPARVEAAYHILKAL..Q
ACT-45ULQ22907.1_427EBCP      PVKASWVHKTGSTGGFGSYVAFIPEKQIGIVMLANKSYPNPARVEAAYHILKAL..Q
ACT-45ULQ25748.1_3478EBCP     PVKASWVHKTGSTGGFGSYVAFIPEKQIGIVMLANKSYPNPARVEAAYHILKAL..Q
ACT-45WCO72956.1_42EBCP       PVKASWVHKTGSTGGFGSYVAFIPEKQIGIVMLANKSYPNPARVEAAYHILKAL..Q
ACT-45APR43249.1_2902EBOC     PVKASWVHKTGSTGGFGSYVAFIPEKQIGIVMLANKSYPNPARVEAAYHILKAL..Q
ACT-45ASA05885.1_3853EBOC     PVKASWVHKTGSTGGFGSYVAFIPEKQIGIVMLANKSYPNPARVEAAYHILKAL..Q
ACT-45ASB85359.1_3592EBOC     PVKASWVHKTGSTGGFGSYVAFIPEKQIGIVMLANKSYPNPARVEAAYHILKAL..Q
ACT-45AWR70714.1_4154EBOC     PVKASWVHKTGSTGGFGSYVAFIPEKQIGIVMLANKSYPNPARVEAAYHILKAL..Q
ACT-45AXM01511.1_4138EBOC     PVKASWVHKTGSTGGFGSYVAFIPEKQIGIVMLANKSYPNPARVEAAYHILKAL..Q
ACT-45AYU96034.1_2649EBOC     PVKASWVHKTGSTGGFGSYVAFIPEKQIGIVMLANKSYPNPARVEAAYHILKAL..Q
ACT-45QBH65043.1_3960EBOC     PVKASWVHKTGSTGGFGSYVAFIPEKQIGIVMLANKSYPNPARVEAAYHILKAL..Q
ACT-45QEL31100.1_1855EBOC     PVKASWVHKTGSTGGFGSYVAFIPEKQIGIVMLANKSYPNPARVEAAYHILKAL..Q
ACT-45ULQ22907.1_427EBOC      PVKASWVHKTGSTGGFGSYVAFIPEKQIGIVMLANKSYPNPARVEAAYHILKAL..Q
ACT-45ULQ25748.1_3478EBOC     PVKASWVHKTGSTGGFGSYVAFIPEKQIGIVMLANKSYPNPARVEAAYHILKAL..Q
ACT-45WCO72956.1_42EBOC       PVKASWVHKTGSTGGFGSYVAFIPEKQIGIVMLANKSYPNPARVEAAYHILKAL..Q
ACT-C197QLA02472.1_2022EOC    PVEASWVHKTGSTGGFGSYVAFIPEKQIGIVMLANKSYPNPARVEAAYHILDAL..Q
ACT-64QMT06026.1_4255EBCP     PVKASWVHKTGSTGGFGSYVAFIPEKELGIVMLANKSYPNPARVEAAYRILSAL..Q
ACT-64QMT06026.1_4255EBOC     PVKASWVHKTGSTGGFGSYVAFIPEKELGIVMLANKSYPNPARVEAAYRILSAL..Q
ACT-C198QXA51435.1_2071EOC    PVKASWVHKTGSTGGFGSYVAFIPEKQLGIVMLANKSYPNPARVEAAYQILNAL..Q
                        acc
```

ADC enzyme group

```
                                                  α1              α2         β1
8FQV_1|Chains                                QQQQQQQQQQQQQ  QQQQQQQ    ────────→TT
                                            1        10        20        30
8FQV_1|Chains                      ......................MGNTPKDQEIKKLVDQNFKPLLEKYDVPGMAVGVIQNN
-154OCUAc18_RS13275_2608AbBOC      ...........................................................N
-154OCUAc18_RS13275_2608AbBCP      ...........................................................N
ADC-73BDE22771.1_1271AbBCP         ...................................................MAVGVIQNN
DC-87WP_068981619.1_1049AbBOC      MRFKKISCLLLSPLFIFSTSIYADNTPKDQEIKKLVDQNFKPLLEKYDVPGMAVGVIQNN
DC-87WP_068981619.1_1049AbBCP      MRFKKISCLLLSPLFIFSTSIYADNTPKDQEIKKLVDQNFKPLLEKYDVPGMAVGVIQNN
C-249WP_001211200.1_1039AbBOC      MRFKKISCLLLSPLFIFSTSIYADNTPKDQEIKKLVDQNFKPLLEKYDVPGMAVGVIQNN
C-249WP_001211200.1_1039AbBCP      MRFKKISCLLLSPLFIFSTSIYADNTPKDQEIKKLVDQNFKPLLEKYDVPGMAVGVIQNN
DC-32WP_004739487.1_2518AbBOC      MRFKKISCLLLSPLFIFSTSIYADNTPKDQEIKKLVDQNFKPLLEKYDVPGMAVGVIQNN
DC-32WP_004739487.1_3158AbBOC      MRFKKISCLLLSPLFIFSTSIYADNTPKDQEIKKLVDQNFKPLLEKYDVPGMAVGVIQNN
DC-32WP_004739487.1_2392AbBOC      MRFKKISCLLLSPLFIFSTSIYADNTPKDQEIKKLVDQNFKPLLEKYDVPGMAVGVIQNN
DC-32WP_004739487.1_2378AbBOC      MRFKKISCLLLSPLFIFSTSIYADNTPKDQEIKKLVDQNFKPLLEKYDVPGMAVGVIQNN
DC-32WP_004739487.1_2518AbBCP      MRFKKISCLLLSPLFIFSTSIYADNTPKDQEIKKLVDQNFKPLLEKYDVPGMAVGVIQNN
DC-32WP_004739487.1_3158AbBCP      MRFKKISCLLLSPLFIFSTSIYADNTPKDQEIKKLVDQNFKPLLEKYDVPGMAVGVIQNN
DC-32WP_004739487.1_2392AbBCP      MRFKKISCLLLSPLFIFSTSIYADNTPKDQEIKKLVDQNFKPLLEKYDVPGMAVGVIQNN
DC-32WP_004739487.1_2378AbBCP      MRFKKISCLLLSPLFIFSTSIYADNTPKDQEIKKLVDQNFKPLLEKYDVPGMAVGVIQNN
C-248WP_021511005.1_1080AbBOC      MRFKKISCLLLSPLFIFSTSIYADNTPKDQEIKKLVDQNFKPLLEKYDVPGMAVGVIQNN
C-248WP_021511005.1_1080AbBCP      MRFKKISCLLLSPLFIFSTSIYADNTPKDQEIKKLVDQNFKPLLEKYDVPGMAVGVIQNN
ADC-191UIY52419.1_2933AbOC         MRFKKISCLLLSPLFIFSTSIYAGNTPKDQEIKKLVDQNFKPLLEKYDVPGMAVGVIQNN
DC-191WP_003112187.1_3072AbOC      MRFKKISCLLLSPLFIFSTSIYAGNTPKDQEIKKLVDQNFKPLLEKYDVPGMAVGVIQNN
DC-191WP_003112187.1_2848AbOC      MRFKKISCLLLSPLFIFSTSIYAGNTPKDQEIKKLVDQNFKPLLEKYDVPGMAVGVIQNN
C-191WP_003112187.1_2694AbBOC      MRFKKISCLLLSPLFIFSTSIYAGNTPKDQEIKKLVDQNFKPLLEKYDVPGMAVGVIQNN
C-191WP_003112187.1_2694AbBCP      MRFKKISCLLLSPLFIFSTSIYAGNTPKDQEIKKLVDQNFKPLLEKYDVPGMAVGVIQNN
C-169WP_001211212.1_2709AbBOC      MRFKKISCLLLSPLFIFSTSIYAGNTPKDQEIKKLVDQNFKPLLEKYDVPGMAVGVIQNN
C-169WP_001211212.1_2457AbBOC      MRFKKISCLLLSPLFIFSTSIYAGNTPKDQEIKKLVDQNFKPLLEKYDVPGMAVGVIQNN
ADC-169UYQ18775.1_1073AbBOC        MRFKKISCLLLSPLFIFSTSIYAGNTPKDQEIKKLVDQNFKPLLEKYDVPGMAVGVIQNN
ADC-169UYQ29710.1_1071AbBOC        MRFKKISCLLLSPLFIFSTSIYAGNTPKDQEIKKLVDQNFKPLLEKYDVPGMAVGVIQNN
ADC-169UYQ59091.1_1072AbBOC        MRFKKISCLLLSPLFIFSTSIYAGNTPKDQEIKKLVDQNFKPLLEKYDVPGMAVGVIQNN
ADC-169WGT82884.1_1015AbBOC        MRFKKISCLLLSPLFIFSTSIYAGNTPKDQEIKKLVDQNFKPLLEKYDVPGMAVGVIQNN
C-169WP_001211212.1_2709AbBCP      MRFKKISCLLLSPLFIFSTSIYAGNTPKDQEIKKLVDQNFKPLLEKYDVPGMAVGVIQNN
C-169WP_001211212.1_2457AbBCP      MRFKKISCLLLSPLFIFSTSIYAGNTPKDQEIKKLVDQNFKPLLEKYDVPGMAVGVIQNN
ADC-169UYQ18775.1_1073AbBCP        MRFKKISCLLLSPLFIFSTSIYAGNTPKDQEIKKLVDQNFKPLLEKYDVPGMAVGVIQNN
ADC-169UYQ29710.1_1071AbBCP        MRFKKISCLLLSPLFIFSTSIYAGNTPKDQEIKKLVDQNFKPLLEKYDVPGMAVGVIQNN
ADC-169UYQ59091.1_1072AbBCP        MRFKKISCLLLSPLFIFSTSIYAGNTPKDQEIKKLVDQNFKPLLEKYDVPGMAVGVIQNN
ADC-169WGT82884.1_1015AbBCP        MRFKKISCLLLSPLFIFSTSIYAGNTPKDQEIKKLVDQNFKPLLEKYDVPGMAVGVIQNN
DC-99WP_068981630.1_2482AbBOC      MRFKKISCLLLSPLFIFSTSIYAGNTPKDQEIKKLVDQNFKPLLEKYDVPGMAVGIIQNN
DC-99WP_068981630.1_2482AbBCP      MRFKKISCLLLSPLFIFSTSIYAGNTPKDQEIKKLVDQNFKPLLEKYDVPGMAVGIIQNN
DC-222WP_031960999.1_1504AbOC      MRFKKISCLLLSPLFIFSTSIYAGNTPKDQEIKKLVDQNFKPLLEKYDVPGMAVGVIQNN
ADC-222UYP92921.1_1090AbBOC        MRFKKISCLLLSPLFIFSTSIYAGNTPKDQEIKKLVDQNFKPLLEKYDVPGMAVGVIQNN
ADC-222UYQ05421.1_2617AbBOC        MRFKKISCLLLSPLFIFSTSIYAGNTPKDQEIKKLVDQNFKPLLEKYDVPGMAVGVIQNN
ADC-222UYQ22523.1_1090AbBOC        MRFKKISCLLLSPLFIFSTSIYAGNTPKDQEIKKLVDQNFKPLLEKYDVPGMAVGVIQNN
ADC-222UYQ26181.1_1089AbBOC        MRFKKISCLLLSPLFIFSTSIYAGNTPKDQEIKKLVDQNFKPLLEKYDVPGMAVGVIQNN
ADC-222UYQ33449.1_1092AbBOC        MRFKKISCLLLSPLFIFSTSIYAGNTPKDQEIKKLVDQNFKPLLEKYDVPGMAVGVIQNN
ADC-222UYQ42503.1_2616AbBOC        MRFKKISCLLLSPLFIFSTSIYAGNTPKDQEIKKLVDQNFKPLLEKYDVPGMAVGVIQNN
ADC-222UYQ37112.1_1090AbBOC        MRFKKISCLLLSPLFIFSTSIYAGNTPKDQEIKKLVDQNFKPLLEKYDVPGMAVGVIQNN
ADC-222UYQ46167.1_2615AbBOC        MRFKKISCLLLSPLFIFSTSIYAGNTPKDQEIKKLVDQNFKPLLEKYDVPGMAVGVIQNN
ADC-222UYQ51934.1_1091AbBOC        MRFKKISCLLLSPLFIFSTSIYAGNTPKDQEIKKLVDQNFKPLLEKYDVPGMAVGVIQNN
ADC-222UYQ55558.1_1091AbBOC        MRFKKISCLLLSPLFIFSTSIYAGNTPKDQEIKKLVDQNFKPLLEKYDVPGMAVGVIQNN
ADC-222WBS68552.1_1096AbBOC        MRFKKISCLLLSPLFIFSTSIYAGNTPKDQEIKKLVDQNFKPLLEKYDVPGMAVGVIQNN
ADC-222WBS55174.1_2602AbBOC        MRFKKISCLLLSPLFIFSTSIYAGNTPKDQEIKKLVDQNFKPLLEKYDVPGMAVGVIQNN
ADC-222WBS51522.1_2600AbBOC        MRFKKISCLLLSPLFIFSTSIYAGNTPKDQEIKKLVDQNFKPLLEKYDVPGMAVGVIQNN
ADC-222UYP92921.1_1090AbBCP        MRFKKISCLLLSPLFIFSTSIYAGNTPKDQEIKKLVDQNFKPLLEKYDVPGMAVGVIQNN
ADC-222UYQ05421.1_2617AbBCP        MRFKKISCLLLSPLFIFSTSIYAGNTPKDQEIKKLVDQNFKPLLEKYDVPGMAVGVIQNN
ADC-222UYQ22523.1_1090AbBCP        MRFKKISCLLLSPLFIFSTSIYAGNTPKDQEIKKLVDQNFKPLLEKYDVPGMAVGVIQNN
ADC-222UYQ26181.1_1089AbBCP        MRFKKISCLLLSPLFIFSTSIYAGNTPKDQEIKKLVDQNFKPLLEKYDVPGMAVGVIQNN
ADC-222UYQ33449.1_1092AbBCP        MRFKKISCLLLSPLFIFSTSIYAGNTPKDQEIKKLVDQNFKPLLEKYDVPGMAVGVIQNN
ADC-222UYQ42503.1_2616AbBCP        MRFKKISCLLLSPLFIFSTSIYAGNTPKDQEIKKLVDQNFKPLLEKYDVPGMAVGVIQNN
ADC-222UYQ37112.1_1090AbBCP        MRFKKISCLLLSPLFIFSTSIYAGNTPKDQEIKKLVDQNFKPLLEKYDVPGMAVGVIQNN
ADC-222UYQ46167.1_2615AbBCP        MRFKKISCLLLSPLFIFSTSIYAGNTPKDQEIKKLVDQNFKPLLEKYDVPGMAVGVIQNN
ADC-222UYQ51934.1_1091AbBCP        MRFKKISCLLLSPLFIFSTSIYAGNTPKDQEIKKLVDQNFKPLLEKYDVPGMAVGVIQNN
ADC-222UYQ55558.1_1091AbBCP        MRFKKISCLLLSPLFIFSTSIYAGNTPKDQEIKKLVDQNFKPLLEKYDVPGMAVGVIQNN
ADC-222WBS68552.1_1096AbBCP        MRFKKISCLLLSPLFIFSTSIYAGNTPKDQEIKKLVDQNFKPLLEKYDVPGMAVGVIQNN
ADC-222WBS55174.1_2602AbBCP        MRFKKISCLLLSPLFIFSTSIYAGNTPKDQEIKKLVDQNFKPLLEKYDVPGMAVGVIQNN
ADC-222WBS51522.1_2600AbBCP        MRFKKISCLLLSPLFIFSTSIYAGNTPKDQEIKKLVDQNFKPLLEKYDVPGMAVGVIQNN
ADC-26WP_001211238.1_141AbOC       MRFKKISCLLLSPLFIFSTSIYAGNTPKDQEIKKLVDQNFKPLLEKYDVPGMAVGVIQNN
ADC-26WP_001211238.1_142AbOC       MRFKKISCLLLSPLFIFSTSIYAGNTPKDQEIKKLVDQNFKPLLEKYDVPGMAVGVIQNN
ADC-26WP_001211238.1_1AbOC         MRFKKISCLLLSPLFIFSTSIYAGNTPKDQEIKKLVDQNFKPLLEKYDVPGMAVGVIQNN
ADC-26WP_001211238.1_1113AbOC      MRFKKISCLLLSPLFIFSTSIYAGNTPKDQEIKKLVDQNFKPLLEKYDVPGMAVGVIQNN
ADC-26WP_001211238.1_1112AbOC      MRFKKISCLLLSPLFIFSTSIYAGNTPKDQEIKKLVDQNFKPLLEKYDVPGMAVGVIQNN
DC-26WP_001211238.1_1105AbBOC      MRFKKISCLLLSPLFIFSTSIYAGNTPKDQEIKKLVDQNFKPLLEKYDVPGMAVGVIQNN
DC-26WP_001211238.1_1111AbBOC      MRFKKISCLLLSPLFIFSTSIYAGNTPKDQEIKKLVDQNFKPLLEKYDVPGMAVGVIQNN
ADC-26WP_001211238.1_503AbBOC      MRFKKISCLLLSPLFIFSTSIYAGNTPKDQEIKKLVDQNFKPLLEKYDVPGMAVGVIQNN
DC-26WP_001211238.1_2774AbBOC      MRFKKISCLLLSPLFIFSTSIYAGNTPKDQEIKKLVDQNFKPLLEKYDVPGMAVGVIQNN
DC-26WP_001211238.1_1064AbBOC      MRFKKISCLLLSPLFIFSTSIYAGNTPKDQEIKKLVDQNFKPLLEKYDVPGMAVGVIQNN
DC-26WP_001211238.1_2484AbBOC      MRFKKISCLLLSPLFIFSTSIYAGNTPKDQEIKKLVDQNFKPLLEKYDVPGMAVGVIQNN
DC-26WP_001211238.1_3858AbBOC      MRFKKISCLLLSPLFIFSTSIYAGNTPKDQEIKKLVDQNFKPLLEKYDVPGMAVGVIQNN
ADC-26WP_001211238.1_142AbBOC      MRFKKISCLLLSPLFIFSTSIYAGNTPKDQEIKKLVDQNFKPLLEKYDVPGMAVGVIQNN
DC-26WP_001211238.1_1085AbBOC      MRFKKISCLLLSPLFIFSTSIYAGNTPKDQEIKKLVDQNFKPLLEKYDVPGMAVGVIQNN
DC-26WP_001211238.1_2525AbBOC      MRFKKISCLLLSPLFIFSTSIYAGNTPKDQEIKKLVDQNFKPLLEKYDVPGMAVGVIQNN
DC-26WP_001211238.1_2477AbBOC      MRFKKISCLLLSPLFIFSTSIYAGNTPKDQEIKKLVDQNFKPLLEKYDVPGMAVGVIQNN
DC-26WP_001211238.1_1069AbBOC      MRFKKISCLLLSPLFIFSTSIYAGNTPKDQEIKKLVDQNFKPLLEKYDVPGMAVGVIQNN
DC-26WP_001211238.1_1029AbBOC      MRFKKISCLLLSPLFIFSTSIYAGNTPKDQEIKKLVDQNFKPLLEKYDVPGMAVGVIQNN
DC-26WP_001211238.1_2443AbBOC      MRFKKISCLLLSPLFIFSTSIYAGNTPKDQEIKKLVDQNFKPLLEKYDVPGMAVGVIQNN
DC-26WP_001211238.1_1149AbBOC      MRFKKISCLLLSPLFIFSTSIYAGNTPKDQEIKKLVDQNFKPLLEKYDVPGMAVGVIQNN
DC-26WP_001211238.1_1148AbBOC      MRFKKISCLLLSPLFIFSTSIYAGNTPKDQEIKKLVDQNFKPLLEKYDVPGMAVGVIQNN
DC-26WP_001211238.1_1374AbBOC      MRFKKISCLLLSPLFIFSTSIYAGNTPKDQEIKKLVDQNFKPLLEKYDVPGMAVGVIQNN
DC-26WP_001211238.1_2862AbBOC      MRFKKISCLLLSPLFIFSTSIYAGNTPKDQEIKKLVDQNFKPLLEKYDVPGMAVGVIQNN
DC-26WP_001211238.1_2629AbBOC      MRFKKISCLLLSPLFIFSTSIYAGNTPKDQEIKKLVDQNFKPLLEKYDVPGMAVGVIQNN
DC-26WP_001211238.1_1355AbBOC      MRFKKISCLLLSPLFIFSTSIYAGNTPKDQEIKKLVDQNFKPLLEKYDVPGMAVGVIQNN
DC-26WP_001211238.1_3092AbBOC      MRFKKISCLLLSPLFIFSTSIYAGNTPKDQEIKKLVDQNFKPLLEKYDVPGMAVGVIQNN
DC-26WP_001211238.1_1086AbBOC      MRFKKISCLLLSPLFIFSTSIYAGNTPKDQEIKKLVDQNFKPLLEKYDVPGMAVGVIQNN
DC-26WP_001211238.1_1016AbBOC      MRFKKISCLLLSPLFIFSTSIYAGNTPKDQEIKKLVDQNFKPLLEKYDVPGMAVGVIQNN
ADC-26UUG48795.1_2867AbBOC        MRFKKISCLLLSPLFIFSTSIYAGNTPKDQEIKKLVDQNFKPLLEKYDVPGMAVGVIQNN
DC-26WP_001211238.1_2535AbBOC      MRFKKISCLLLSPLFIFSTSIYAGNTPKDQEIKKLVDQNFKPLLEKYDVPGMAVGVIQNN
ADC-26WEN46767.1_1086AbBOC        MRFKKISCLLLSPLFIFSTSIYAGNTPKDQEIKKLVDQNFKPLLEKYDVPGMAVGVIQNN
ADC-26WEX48630.1_1270AbBOC        MRFKKISCLLLSPLFIFSTSIYAGNTPKDQEIKKLVDQNFKPLLEKYDVPGMAVGVIQNN
ADC-26WGE92537.1_2034AbBOC        MRFKKISCLLLSPLFIFSTSIYAGNTPKDQEIKKLVDQNFKPLLEKYDVPGMAVGVIQNN
DC-26WP_001211238.1_1105AbBCP      MRFKKISCLLLSPLFIFSTSIYAGNTPKDQEIKKLVDQNFKPLLEKYDVPGMAVGVIQNN
DC-26WP_001211238.1_1111AbBCP      MRFKKISCLLLSPLFIFSTSIYAGNTPKDQEIKKLVDQNFKPLLEKYDVPGMAVGVIQNN
ADC-26WP_001211238.1_503AbBCP      MRFKKISCLLLSPLFIFSTSIYAGNTPKDQEIKKLVDQNFKPLLEKYDVPGMAVGVIQNN
DC-26WP_001211238.1_2774AbBCP      MRFKKISCLLLSPLFIFSTSIYAGNTPKDQEIKKLVDQNFKPLLEKYDVPGMAVGVIQNN
DC-26WP_001211238.1_1064AbBCP      MRFKKISCLLLSPLFIFSTSIYAGNTPKDQEIKKLVDQNFKPLLEKYDVPGMAVGVIQNN
DC-26WP_001211238.1_2484AbBCP      MRFKKISCLLLSPLFIFSTSIYAGNTPKDQEIKKLVDQNFKPLLEKYDVPGMAVGVIQNN
DC-26WP_001211238.1_3858AbBCP      MRFKKISCLLLSPLFIFSTSIYAGNTPKDQEIKKLVDQNFKPLLEKYDVPGMAVGVIQNN
ADC-26WP_001211238.1_142AbBCP      MRFKKISCLLLSPLFIFSTSIYAGNTPKDQEIKKLVDQNFKPLLEKYDVPGMAVGVIQNN
DC-26WP_001211238.1_1085AbBCP      MRFKKISCLLLSPLFIFSTSIYAGNTPKDQEIKKLVDQNFKPLLEKYDVPGMAVGVIQNN
DC-26WP_001211238.1_2525AbBCP      MRFKKISCLLLSPLFIFSTSIYAGNTPKDQEIKKLVDQNFKPLLEKYDVPGMAVGVIQNN
DC-26WP_001211238.1_2477AbBCP      MRFKKISCLLLSPLFIFSTSIYAGNTPKDQEIKKLVDQNFKPLLEKYDVPGMAVGVIQNN
DC-26WP_001211238.1_1069AbBCP      MRFKKISCLLLSPLFIFSTSIYAGNTPKDQEIKKLVDQNFKPLLEKYDVPGMAVGVIQNN
DC-26WP_001211238.1_1029AbBCP      MRFKKISCLLLSPLFIFSTSIYAGNTPKDQEIKKLVDQNFKPLLEKYDVPGMAVGVIQNN
DC-26WP_001211238.1_2443AbBCP      MRFKKISCLLLSPLFIFSTSIYAGNTPKDQEIKKLVDQNFKPLLEKYDVPGMAVGVIQNN
DC-26WP_001211238.1_1149AbBCP      MRFKKISCLLLSPLFIFSTSIYAGNTPKDQEIKKLVDQNFKPLLEKYDVPGMAVGVIQNN
DC-26WP_001211238.1_1148AbBCP      MRFKKISCLLLSPLFIFSTSIYAGNTPKDQEIKKLVDQNFKPLLEKYDVPGMAVGVIQNN
DC-26WP_001211238.1_1374AbBCP      MRFKKISCLLLSPLFIFSTSIYAGNTPKDQEIKKLVDQNFKPLLEKYDVPGMAVGVIQNN
DC-26WP_001211238.1_2862AbBCP      MRFKKISCLLLSPLFIFSTSIYAGNTPKDQEIKKLVDQNFKPLLEKYDVPGMAVGVIQNN
DC-26WP_001211238.1_2629AbBCP      MRFKKISCLLLSPLFIFSTSIYAGNTPKDQEIKKLVDQNFKPLLEKYDVPGMAVGVIQNN
DC-26WP_001211238.1_1355AbBCP      MRFKKISCLLLSPLFIFSTSIYAGNTPKDQEIKKLVDQNFKPLLEKYDVPGMAVGVIQNN
DC-26WP_001211238.1_3092AbBCP      MRFKKISCLLLSPLFIFSTSIYAGNTPKDQEIKKLVDQNFKPLLEKYDVPGMAVGVIQNN
DC-26WP_001211238.1_1086AbBCP      MRFKKISCLLLSPLFIFSTSIYAGNTPKDQEIKKLVDQNFKPLLEKYDVPGMAVGVIQNN
DC-26WP_001211238.1_1016AbBCP      MRFKKISCLLLSPLFIFSTSIYAGNTPKDQEIKKLVDQNFKPLLEKYDVPGMAVGVIQNN
ADC-26UUG48795.1_2867AbBCP        MRFKKISCLLLSPLFIFSTSIYAGNTPKDQEIKKLVDQNFKPLLEKYDVPGMAVGVIQNN
DC-26WP_001211238.1_2535AbBCP      MRFKKISCLLLSPLFIFSTSIYAGNTPKDQEIKKLVDQNFKPLLEKYDVPGMAVGVIQNN
ADC-26WEN46767.1_1086AbBCP        MRFKKISCLLLSPLFIFSTSIYAGNTPKDQEIKKLVDQNFKPLLEKYDVPGMAVGVIQNN
ADC-26WEX48630.1_1270AbBCP        MRFKKISCLLLSPLFIFSTSIYAGNTPKDQEIKKLVDQNFKPLLEKYDVPGMAVGVIQNN
ADC-26WGE92537.1_2034AbBCP        MRFKKISCLLLSPLFIFSTSIYAGNTPKDQEIKKLVDQNFKPLLEKYDVPGMAVGVIQNN
C-243WP_001211199.1_2530AbBOC      MRFKKISCLLLPPLFIFSTSIYAGNTPKDQEIKKLVDQNFKPLLEKYDVPGMAVGVIQNN
C-243WP_001211199.1_2530AbBCP      MRFKKISCLLLPPLFIFSTSIYAGNTPKDQEIKKLVDQNFKPLLEKYDVPGMAVGVIQNN
ADC-74WP_001211203.1_938AbBOC      MRFKKISCLLLSPLFIFSTSIYAGNTPKDQEIKKLVDQNFKPLLEKYDVPGMAVGVIQNN
ADC-74WP_001211203.1_2AbBOC        MRFKKISCLLLSPLFIFSTSIYAGNTPKDQEIKKLVDQNFKPLLEKYDVPGMAVGVIQNN
ADC-74WP_001211203.1_95AbBOC       MRFKKISCLLLSPLFIFSTSIYAGNTPKDQEIKKLVDQNFKPLLEKYDVPGMAVGVIQNN
ADC-74WP_001211203.1_142AbBOC      MRFKKISCLLLSPLFIFSTSIYAGNTPKDQEIKKLVDQNFKPLLEKYDVPGMAVGVIQNN
DC-74WP_001211203.1_2317AbBOC      MRFKKISCLLLSPLFIFSTSIYAGNTPKDQEIKKLVDQNFKPLLEKYDVPGMAVGVIQNN
ADC-74WP_001211203.1_570AbBOC      MRFKKISCLLLSPLFIFSTSIYAGNTPKDQEIKKLVDQNFKPLLEKYDVPGMAVGVIQNN
DC-74WP_001211203.1_2791AbBOC      MRFKKISCLLLSPLFIFSTSIYAGNTPKDQEIKKLVDQNFKPLLEKYDVPGMAVGVIQNN
DC-74WP_001211203.1_2790AbBOC      MRFKKISCLLLSPLFIFSTSIYAGNTPKDQEIKKLVDQNFKPLLEKYDVPGMAVGVIQNN
DC-74WP_001211203.1_1414AbBOC      MRFKKISCLLLSPLFIFSTSIYAGNTPKDQEIKKLVDQNFKPLLEKYDVPGMAVGVIQNN
ADC-74WP_001211203.1_938AbBCP      MRFKKISCLLLSPLFIFSTSIYAGNTPKDQEIKKLVDQNFKPLLEKYDVPGMAVGVIQNN
ADC-74WP_001211203.1_2AbBCP        MRFKKISCLLLSPLFIFSTSIYAGNTPKDQEIKKLVDQNFKPLLEKYDVPGMAVGVIQNN
ADC-74WP_001211203.1_95AbBCP       MRFKKISCLLLSPLFIFSTSIYAGNTPKDQEIKKLVDQNFKPLLEKYDVPGMAVGVIQNN
ADC-74WP_001211203.1_142AbBCP      MRFKKISCLLLSPLFIFSTSIYAGNTPKDQEIKKLVDQNFKPLLEKYDVPGMAVGVIQNN
DC-74WP_001211203.1_2317AbBCP      MRFKKISCLLLSPLFIFSTSIYAGNTPKDQEIKKLVDQNFKPLLEKYDVPGMAVGVIQNN
ADC-74WP_001211203.1_570AbBCP      MRFKKISCLLLSPLFIFSTSIYAGNTPKDQEIKKLVDQNFKPLLEKYDVPGMAVGVIQNN
DC-74WP_001211203.1_2791AbBCP      MRFKKISCLLLSPLFIFSTSIYAGNTPKDQEIKKLVDQNFKPLLEKYDVPGMAVGVIQNN
DC-74WP_001211203.1_2790AbBCP      MRFKKISCLLLSPLFIFSTSIYAGNTPKDQEIKKLVDQNFKPLLEKYDVPGMAVGVIQNN
DC-74WP_001211203.1_1414AbBCP      MRFKKISCLLLSPLFIFSTSIYAGNTPKDQEIKKLVDQNFKPLLEKYDVPGMAVGVIQNN
C-347WP_138149148.1_2656AbBOC      MRFKKISCLLLSPLFIFSTSIYAGNTPKDQEIKKLVDQNFKPLLEKYDVPGMAVGVIQNN
C-347WP_138149148.1_2656AbBCP      MRFKKISCLLLSPLFIFSTSIYAGNTPKDQEIKKLVDQNFKPLLEKYDVPGMAVGVIQNN
ADC-80WP_029424536.1_1068AbOC      MRFKKISCLLLSPLFIFSTSIYAGNTPKDQEIKKLVDQNFKPLLEKYDVPGMAVGVIQNN
DC-80WP_029424536.1_1061AbBOC      MRFKKISCLLLSPLFIFSTSIYAGNTPKDQEIKKLVDQNFKPLLEKYDVPGMAVGVIQNN
DC-80WP_029424536.1_2945AbBOC      MRFKKISCLLLSPLFIFSTSIYAGNTPKDQEIKKLVDQNFKPLLEKYDVPGMAVGVIQNN
DC-80WP_029424536.1_2729AbBOC      MRFKKISCLLLSPLFIFSTSIYAGNTPKDQEIKKLVDQNFKPLLEKYDVPGMAVGVIQNN
DC-80WP_029424536.1_1008AbBOC      MRFKKISCLLLSPLFIFSTSIYAGNTPKDQEIKKLVDQNFKPLLEKYDVPGMAVGVIQNN
```

DC-80WP_029424536.1_1061AbBCP	MRFKKISCLLLSPLFIFSTSIYAGNTPKDQEIKKLVDQNFKPLLEKYDVPGMAVGVIQNN
DC-80WP_029424536.1_2945AbBCP	MRFKKISCLLLSPLFIFSTSIYAGNTPKDQEIKKLVDQNFKPLLEKYDVPGMAVGVIQNN
DC-80WP_029424536.1_2729AbBCP	MRFKKISCLLLSPLFIFSTSIYAGNTPKDQEIKKLVDQNFKPLLEKYDVPGMAVGVIQNN
DC-80WP_029424536.1_1008AbBCP	MRFKKISCLLLSPLFIFSTSIYAGNTPKDQEIKKLVDQNFKPLLEKYDVPGMAVGVIQNN
DC-156WP_024436624.1_2681AbOC	MRFKKISCLLLPPLFIFSTSIYAGNTPKDQEIKKLVDQNFKPLLEKYDVPGMAVGVIQNN
C-156WP_024436624.1_2375AbBOC	MRFKKISCLLLPPLFIFSTSIYAGNTPKDQEIKKLVDQNFKPLLEKYDVPGMAVGVIQNN
C-156WP_024436624.1_1038AbBOC	MRFKKISCLLLPPLFIFSTSIYAGNTPKDQEIKKLVDQNFKPLLEKYDVPGMAVGVIQNN
C-156WP_024436624.1_1041AbBOC	MRFKKISCLLLPPLFIFSTSIYAGNTPKDQEIKKLVDQNFKPLLEKYDVPGMAVGVIQNN
C-156WP_024436624.1_1124AbBOC	MRFKKISCLLLPPLFIFSTSIYAGNTPKDQEIKKLVDQNFKPLLEKYDVPGMAVGVIQNN
C-156WP_024436624.1_1126AbBOC	MRFKKISCLLLPPLFIFSTSIYAGNTPKDQEIKKLVDQNFKPLLEKYDVPGMAVGVIQNN
C-156WP_024436624.1_2375AbBCP	MRFKKISCLLLPPLFIFSTSIYAGNTPKDQEIKKLVDQNFKPLLEKYDVPGMAVGVIQNN
C-156WP_024436624.1_1038AbBCP	MRFKKISCLLLPPLFIFSTSIYAGNTPKDQEIKKLVDQNFKPLLEKYDVPGMAVGVIQNN
C-156WP_024436624.1_1041AbBCP	MRFKKISCLLLPPLFIFSTSIYAGNTPKDQEIKKLVDQNFKPLLEKYDVPGMAVGVIQNN
C-156WP_024436624.1_1124AbBCP	MRFKKISCLLLPPLFIFSTSIYAGNTPKDQEIKKLVDQNFKPLLEKYDVPGMAVGVIQNN
C-156WP_024436624.1_1126AbBCP	MRFKKISCLLLPPLFIFSTSIYAGNTPKDQEIKKLVDQNFKPLLEKYDVPGMAVGVIQNN
C-165WP_001211239.1_1734AbBOC	MRFKKISCLLLSPLFIFSTSIYAGNTPKDQEIKKLVDQNFKPLLEKYDVPGMAVGVIQNN
C-165WP_001211239.1_1032AbBOC	MRFKKISCLLLSPLFIFSTSIYAGNTPKDQEIKKLVDQNFKPLLEKYDVPGMAVGVIQNN
C-165WP_001211239.1_1734AbBCP	MRFKKISCLLLSPLFIFSTSIYAGNTPKDQEIKKLVDQNFKPLLEKYDVPGMAVGVIQNN
C-165WP_001211239.1_1032AbBCP	MRFKKISCLLLSPLFIFSTSIYAGNTPKDQEIKKLVDQNFKPLLEKYDVPGMAVGVIQNN
C-263WP_109433738.1_2026AbBOC	MRFKKISCLLLSPLFIFSTSIYAGNTPKDQEIKKLVDQNFKPLLEKYDVPGMAVGVIQNN
DC-263WP_109433738.1_954AbBOC	MRFKKISCLLLSPLFIFSTSIYAGNTPKDQEIKKLVDQNFKPLLEKYDVPGMAVGVIQNN
C-263WP_109433738.1_1022AbBOC	MRFKKISCLLLSPLFIFSTSIYAGNTPKDQEIKKLVDQNFKPLLEKYDVPGMAVGVIQNN
C-263WP_109433738.1_3219AbBOC	MRFKKISCLLLSPLFIFSTSIYAGNTPKDQEIKKLVDQNFKPLLEKYDVPGMAVGVIQNN
C-263WP_109433738.1_2026AbBCP	MRFKKISCLLLSPLFIFSTSIYAGNTPKDQEIKKLVDQNFKPLLEKYDVPGMAVGVIQNN
DC-263WP_109433738.1_954AbBCP	MRFKKISCLLLSPLFIFSTSIYAGNTPKDQEIKKLVDQNFKPLLEKYDVPGMAVGVIQNN
C-263WP_109433738.1_1022AbBCP	MRFKKISCLLLSPLFIFSTSIYAGNTPKDQEIKKLVDQNFKPLLEKYDVPGMAVGVIQNN
C-263WP_109433738.1_3219AbBCP	MRFKKISCLLLSPLFIFSTSIYAGNTPKDQEIKKLVDQNFKPLLEKYDVPGMAVGVIQNN
DC-79WP_001159760.1_2471AbBOC	MQFKKISCLLLSPLFIFSTSIYADNTPKDQEIKKLVDQNFKPLLEKYDVPGMAVGVIQNN
ADC-79WP_001159760.1_670AbBOC	MQFKKISCLLLSPLFIFSTSIYADNTPKDQEIKKLVDQNFKPLLEKYDVPGMAVGVIQNN
DC-79WP_001159760.1_2535AbBOC	MQFKKISCLLLSPLFIFSTSIYADNTPKDQEIKKLVDQNFKPLLEKYDVPGMAVGVIQNN
DC-79WP_001159760.1_1080AbBOC	MQFKKISCLLLSPLFIFSTSIYADNTPKDQEIKKLVDQNFKPLLEKYDVPGMAVGVIQNN
DC-79WP_001159760.1_2471AbBCP	MQFKKISCLLLSPLFIFSTSIYADNTPKDQEIKKLVDQNFKPLLEKYDVPGMAVGVIQNN
ADC-79WP_001159760.1_670AbBCP	MQFKKISCLLLSPLFIFSTSIYADNTPKDQEIKKLVDQNFKPLLEKYDVPGMAVGVIQNN
DC-79WP_001159760.1_2535AbBCP	MQFKKISCLLLSPLFIFSTSIYADNTPKDQEIKKLVDQNFKPLLEKYDVPGMAVGVIQNN
DC-79WP_001159760.1_1080AbBCP	MQFKKISCLLLSPLFIFSTSIYADNTPKDQEIKKLVDQNFKPLLEKYDVPGMAVGVIQNN
ADC-6WP_017725267.1_1459AbBOC	MRFKKISCLLLSPLFIFSTSIYAGNTPKDQEIKKLVDQNFKPLLEKYDVPGMAVGVIQNN
ADC-6WP_017725267.1_1678AbBOC	MRFKKISCLLLSPLFIFSTSIYAGNTPKDQEIKKLVDQNFKPLLEKYDVPGMAVGVIQNN
ADC-6WP_017725267.1_2561AbBOC	MRFKKISCLLLSPLFIFSTSIYAGNTPKDQEIKKLVDQNFKPLLEKYDVPGMAVGVIQNN
ADC-6WP_017725267.1_1459AbBCP	MRFKKISCLLLSPLFIFSTSIYAGNTPKDQEIKKLVDQNFKPLLEKYDVPGMAVGVIQNN
ADC-6WP_017725267.1_1678AbBCP	MRFKKISCLLLSPLFIFSTSIYAGNTPKDQEIKKLVDQNFKPLLEKYDVPGMAVGVIQNN
ADC-6WP_017725267.1_2561AbBCP	MRFKKISCLLLSPLFIFSTSIYAGNTPKDQEIKKLVDQNFKPLLEKYDVPGMAVGVIQNN
ADC-1WP_004714775.1_3726AbBOC	MRFKKISCLLLSPLFIFSTSIYAGNTPKDQEIKKLVDQNFKPLLEKYDVPGMAVGVIQNN
ADC-1WP_004714775.1_3726AbBCP	MRFKKISCLLLSPLFIFSTSIYAGNTPKDQEIKKLVDQNFKPLLEKYDVPGMAVGVIQNN
C-181WP_046693238.1_1083AbBOC	MRFKKISCLLLSPLFIFSTSIYAGNTPKDQEIKKLVDQNFKPLLEKYDVPGMAVGVIQNN
C-181WP_046693238.1_1083AbBCP	MRFKKISCLLLSPLFIFSTSIYAGNTPKDQEIKKLVDQNFKPLLEKYDVPGMAVGVIQNN
C-238WP_002058891.1_2458AbBOC	MRFKKISCLLLSPLFIFSTSIYAGNTPKDQEIKKLVDQNFKPLLEKYDVPGMAVGVIQNN
C-238WP_002058891.1_2458AbBCP	MRFKKISCLLLSPLFIFSTSIYAGNTPKDQEIKKLVDQNFKPLLEKYDVPGMAVGVIQNN
C-186WP_072292271.1_1675AbBOC	MQFKKISCLLLSPLFIFSTSIYADNTPKDQEIKKLVDQNFKPLLEKYDVPGMAVGVIQNN
C-186WP_072292271.1_1675AbBCP	MQFKKISCLLLSPLFIFSTSIYADNTPKDQEIKKLVDQNFKPLLEKYDVPGMAVGVIQNN
DC-184WP_059247009.1_1188AbOC	MRFKKISCLLLSPLFIFSTSIYAGNTPKDQEIKKLVDQNFKPLLEKYDVPGMAVGIIQNN
ADC-328WP_069370341.1_666AbOC	MRFKKISCLLLSPLFIFSTSIYAGNTPKDQEIKKLVDQNFKPLLEKYDVPGMAVGVIQNN
DC-56WP_031973850.1_1911AbBOC	MRFKKISCLLLSPLFIFSTSIYAGNTPKDQEIKKLVDQNFKPLLEKYDVPGMAVGVIQNN
DC-56WP_031973850.1_2693AbBOC	MRFKKISCLLLSPLFIFSTSIYAGNTPKDQEIKKLVDQNFKPLLEKYDVPGMAVGVIQNN
DC-56WP_031973850.1_1911AbBCP	MRFKKISCLLLSPLFIFSTSIYAGNTPKDQEIKKLVDQNFKPLLEKYDVPGMAVGVIQNN
DC-56WP_031973850.1_2693AbBCP	MRFKKISCLLLSPLFIFSTSIYAGNTPKDQEIKKLVDQNFKPLLEKYDVPGMAVGVIQNN
DC-117WP_002157727.1_394AbBOC	MRFKKISCLLLSPLFIFSTSIYAGNTPKDQEIKKLVDQNFKPLLEKYDVPGMAVGVIQNN
DC-117WP_002157727.1_394AbBCP	MRFKKISCLLLSPLFIFSTSIYAGNTPKDQEIKKLVDQNFKPLLEKYDVPGMAVGVIQNN
ADC-25WP_001211217.1_2432AbOC	MRFKKISCLLLSPLFIFSTSIYAGNTPKDQEIKKLVDQNFKPLLEKYDVPGMAVGVIQNN
ADC-25WP_001211217.1_1025AbOC	MRFKKISCLLLSPLFIFSTSIYAGNTPKDQEIKKLVDQNFKPLLEKYDVPGMAVGVIQNN
ADC-25WP_001211217.1_3391AbOC	MRFKKISCLLLSPLFIFSTSIYAGNTPKDQEIKKLVDQNFKPLLEKYDVPGMAVGVIQNN
ADC-25WP_001211217.1_1042AbOC	MRFKKISCLLLSPLFIFSTSIYAGNTPKDQEIKKLVDQNFKPLLEKYDVPGMAVGVIQNN
ADC-25WP_001211217.1_1185AbOC	MRFKKISCLLLSPLFIFSTSIYAGNTPKDQEIKKLVDQNFKPLLEKYDVPGMAVGVIQNN
ADC-25WP_001211217.1_768AbOC	MRFKKISCLLLSPLFIFSTSIYAGNTPKDQEIKKLVDQNFKPLLEKYDVPGMAVGVIQNN
ADC-25WP_001211217.1_2605AbOC	MRFKKISCLLLSPLFIFSTSIYAGNTPKDQEIKKLVDQNFKPLLEKYDVPGMAVGVIQNN
DC-25WP_001211217.1_1117AbBOC	MRFKKISCLLLSPLFIFSTSIYAGNTPKDQEIKKLVDQNFKPLLEKYDVPGMAVGVIQNN
DC-25WP_001211217.1_1090AbBOC	MRFKKISCLLLSPLFIFSTSIYAGNTPKDQEIKKLVDQNFKPLLEKYDVPGMAVGVIQNN
DC-25WP_001211217.1_1089AbBOC	MRFKKISCLLLSPLFIFSTSIYAGNTPKDQEIKKLVDQNFKPLLEKYDVPGMAVGVIQNN
DC-25WP_001211217.1_1091AbBOC	MRFKKISCLLLSPLFIFSTSIYAGNTPKDQEIKKLVDQNFKPLLEKYDVPGMAVGVIQNN
DC-25WP_001211217.1_1604AbBOC	MRFKKISCLLLSPLFIFSTSIYAGNTPKDQEIKKLVDQNFKPLLEKYDVPGMAVGVIQNN
DC-25WP_001211217.1_1040AbBOC	MRFKKISCLLLSPLFIFSTSIYAGNTPKDQEIKKLVDQNFKPLLEKYDVPGMAVGVIQNN
ADC-25UYQ12932.1_2634AbBOC	MRFKKISCLLLSPLFIFSTSIYAGNTPKDQEIKKLVDQNFKPLLEKYDVPGMAVGVIQNN
ADC-25UYQ09083.1_2628AbBOC	MRFKKISCLLLSPLFIFSTSIYAGNTPKDQEIKKLVDQNFKPLLEKYDVPGMAVGVIQNN
ADC-25UYQ40822.1_1120AbBOC	MRFKKISCLLLSPLFIFSTSIYAGNTPKDQEIKKLVDQNFKPLLEKYDVPGMAVGVIQNN
DC-25WP_001211217.1_1117AbBCP	MRFKKISCLLLSPLFIFSTSIYAGNTPKDQEIKKLVDQNFKPLLEKYDVPGMAVGVIQNN
DC-25WP_001211217.1_1090AbBCP	MRFKKISCLLLSPLFIFSTSIYAGNTPKDQEIKKLVDQNFKPLLEKYDVPGMAVGVIQNN
DC-25WP_001211217.1_1089AbBCP	MRFKKISCLLLSPLFIFSTSIYAGNTPKDQEIKKLVDQNFKPLLEKYDVPGMAVGVIQNN
DC-25WP_001211217.1_1091AbBCP	MRFKKISCLLLSPLFIFSTSIYAGNTPKDQEIKKLVDQNFKPLLEKYDVPGMAVGVIQNN
DC-25WP_001211217.1_1604AbBCP	MRFKKISCLLLSPLFIFSTSIYAGNTPKDQEIKKLVDQNFKPLLEKYDVPGMAVGVIQNN
DC-25WP_001211217.1_1040AbBCP	MRFKKISCLLLSPLFIFSTSIYAGNTPKDQEIKKLVDQNFKPLLEKYDVPGMAVGVIQNN
ADC-25UYQ12932.1_2634AbBCP	MRFKKISCLLLSPLFIFSTSIYAGNTPKDQEIKKLVDQNFKPLLEKYDVPGMAVGVIQNN
ADC-25UYQ09083.1_2628AbBCP	MRFKKISCLLLSPLFIFSTSIYAGNTPKDQEIKKLVDQNFKPLLEKYDVPGMAVGVIQNN
ADC-25UYQ40822.1_1120AbBCP	MRFKKISCLLLSPLFIFSTSIYAGNTPKDQEIKKLVDQNFKPLLEKYDVPGMAVGVIQNN
C-268WP_105922788.1_2839AbBOC	MRFKKISCLLLSPLFIFSTSIYAGNTPKDQEIKKLVDQNFKPLLEKYDVPGMAVGVIQNN
ADC-268WDC47378.1_2492AbBOC	MRFKKISCLLLSPLFIFSTSIYAGNTPKDQEIKKLVDQNFKPLLEKYDVPGMAVGVIQNN
C-268WP_105922788.1_2839AbBCP	MRFKKISCLLLSPLFIFSTSIYAGNTPKDQEIKKLVDQNFKPLLEKYDVPGMAVGVIQNN
ADC-268WDC47378.1_2492AbBCP	MRFKKISCLLLSPLFIFSTSIYAGNTPKDQEIKKLVDQNFKPLLEKYDVPGMAVGVIQNN
DC-155WP_005128228.1_2591AbOC	MRFKKISCLLLSPLFIFSTSIYAGNTPKDQEIKKLVDQNFKPLLEKYDVPGMAVGVIQNN
C-155WP_005128228.1_2549AbBOC	MRFKKISCLLLSPLFIFSTSIYAGNTPKDQEIKKLVDQNFKPLLEKYDVPGMAVGVIQNN
C-155WP_005128228.1_2480AbBOC	MRFKKISCLLLSPLFIFSTSIYAGNTPKDQEIKKLVDQNFKPLLEKYDVPGMAVGVIQNN
C-155WP_005128228.1_2455AbBOC	MRFKKISCLLLSPLFIFSTSIYAGNTPKDQEIKKLVDQNFKPLLEKYDVPGMAVGVIQNN
C-155WP_005128228.1_2549AbBCP	MRFKKISCLLLSPLFIFSTSIYAGNTPKDQEIKKLVDQNFKPLLEKYDVPGMAVGVIQNN
C-155WP_005128228.1_2480AbBCP	MRFKKISCLLLSPLFIFSTSIYAGNTPKDQEIKKLVDQNFKPLLEKYDVPGMAVGVIQNN
C-155WP_005128228.1_2455AbBCP	MRFKKISCLLLSPLFIFSTSIYAGNTPKDQEIKKLVDQNFKPLLEKYDVPGMAVGVIQNN
ADC-76WP_001211237.1_2862AbOC	MRFKKISCLLLSPLFIFSTSIYAGNTPKDQEIKKLVDQNFKPLLEKYDVPGMAVGVIQNN
ADC-76WP_001211237.1_3431AbOC	MRFKKISCLLLSPLFIFSTSIYAGNTPKDQEIKKLVDQNFKPLLEKYDVPGMAVGVIQNN
ADC-76WP_001211237.1_2696AbOC	MRFKKISCLLLSPLFIFSTSIYAGNTPKDQEIKKLVDQNFKPLLEKYDVPGMAVGVIQNN
ADC-76WP_001211237.1_2890AbOC	MRFKKISCLLLSPLFIFSTSIYAGNTPKDQEIKKLVDQNFKPLLEKYDVPGMAVGVIQNN
DC-76WP_001211237.1_2723AbBOC	MRFKKISCLLLSPLFIFSTSIYAGNTPKDQEIKKLVDQNFKPLLEKYDVPGMAVGVIQNN
DC-76WP_001211237.1_1033AbBOC	MRFKKISCLLLSPLFIFSTSIYAGNTPKDQEIKKLVDQNFKPLLEKYDVPGMAVGVIQNN
ADC-76WP_001211237.1_526AbBOC	MRFKKISCLLLSPLFIFSTSIYAGNTPKDQEIKKLVDQNFKPLLEKYDVPGMAVGVIQNN
DC-76WP_001211237.1_1046AbBOC	MRFKKISCLLLSPLFIFSTSIYAGNTPKDQEIKKLVDQNFKPLLEKYDVPGMAVGVIQNN
DC-76WP_001211237.1_1040AbBOC	MRFKKISCLLLSPLFIFSTSIYAGNTPKDQEIKKLVDQNFKPLLEKYDVPGMAVGVIQNN
DC-76WP_001211237.1_3682AbBOC	MRFKKISCLLLSPLFIFSTSIYAGNTPKDQEIKKLVDQNFKPLLEKYDVPGMAVGVIQNN
DC-76WP_001211237.1_2579AbBOC	MRFKKISCLLLSPLFIFSTSIYAGNTPKDQEIKKLVDQNFKPLLEKYDVPGMAVGVIQNN
DC-76WP_001211237.1_2723AbBCP	MRFKKISCLLLSPLFIFSTSIYAGNTPKDQEIKKLVDQNFKPLLEKYDVPGMAVGVIQNN
DC-76WP_001211237.1_1033AbBCP	MRFKKISCLLLSPLFIFSTSIYAGNTPKDQEIKKLVDQNFKPLLEKYDVPGMAVGVIQNN
ADC-76WP_001211237.1_526AbBCP	MRFKKISCLLLSPLFIFSTSIYAGNTPKDQEIKKLVDQNFKPLLEKYDVPGMAVGVIQNN
DC-76WP_001211237.1_1046AbBCP	MRFKKISCLLLSPLFIFSTSIYAGNTPKDQEIKKLVDQNFKPLLEKYDVPGMAVGVIQNN
DC-76WP_001211237.1_1040AbBCP	MRFKKISCLLLSPLFIFSTSIYAGNTPKDQEIKKLVDQNFKPLLEKYDVPGMAVGVIQNN
DC-76WP_001211237.1_3682AbBCP	MRFKKISCLLLSPLFIFSTSIYAGNTPKDQEIKKLVDQNFKPLLEKYDVPGMAVGVIQNN
DC-76WP_001211237.1_2579AbBCP	MRFKKISCLLLSPLFIFSTSIYAGNTPKDQEIKKLVDQNFKPLLEKYDVPGMAVGVIQNN
DC-291WP_001211201.1_1060AbOC	MRFKKISCLLLSPLFIFSTSIYAGNTPKDQEIKKLVDQNFKPLLEKYDVPGIAVGVIQNN
C-291WP_001211201.1_2415AbBOC	MRFKKISCLLLSPLFIFSTSIYAGNTPKDQEIKKLVDQNFKPLLEKYDVPGIAVGVIQNN
C-291WP_001211201.1_2415AbBCP	MRFKKISCLLLSPLFIFSTSIYAGNTPKDQEIKKLVDQNFKPLLEKYDVPGIAVGVIQNN
DC-214WP_060454527.1_118AbBOC	MRFKKISCLLLSPLFIFSTSIYAGNTPKDQEIKKLVDQNFKPLLEKYDVPGMAVGVIQNN
DC-214WP_060454527.1_118AbBCP	MRFKKISCLLLSPLFIFSTSIYAGNTPKDQEIKKLVDQNFKPLLEKYDVPGMAVGVIQNN
C-185WP_001211210.1_2745AbBOC	MRFKKISCLLLSPLFIFSTSIYAGNTPKDQEIKKLVDQNFKPLLEKYDVPGMAVGVIQNN
ADC-185WEI07035.1_298AbBOC	MRFKKISCLLLSPLFIFSTSIYAGNTPKDQEIKKLVDQNFKPLLEKYDVPGMAVGVIQNN
C-185WP_001211210.1_2745AbBCP	MRFKKISCLLLSPLFIFSTSIYAGNTPKDQEIKKLVDQNFKPLLEKYDVPGMAVGVIQNN
ADC-185WEI07035.1_298AbBCP	MRFKKISCLLLSPLFIFSTSIYAGNTPKDQEIKKLVDQNFKPLLEKYDVPGMAVGVIQNN
ADC-11WP_001211205.1_1099AbOC	MRFKKISCLLLSPLFIFSTSIYAGNTPKDQEIKKLVDQNFKPLLEKYDVPGMAVGVIQNN
ADC-11WP_001211205.1_1035AbOC	MRFKKISCLLLSPLFIFSTSIYAGNTPKDQEIKKLVDQNFKPLLEKYDVPGMAVGVIQNN
DC-11WP_001211205.1_2603AbBOC	MRFKKISCLLLSPLFIFSTSIYAGNTPKDQEIKKLVDQNFKPLLEKYDVPGMAVGVIQNN
DC-11WP_001211205.1_1138AbBOC	MRFKKISCLLLSPLFIFSTSIYAGNTPKDQEIKKLVDQNFKPLLEKYDVPGMAVGVIQNN
DC-11WP_001211205.1_2874AbBOC	MRFKKISCLLLSPLFIFSTSIYAGNTPKDQEIKKLVDQNFKPLLEKYDVPGMAVGVIQNN
DC-11WP_001211205.1_2686AbBOC	MRFKKISCLLLSPLFIFSTSIYAGNTPKDQEIKKLVDQNFKPLLEKYDVPGMAVGVIQNN
ADC-11WP_001211205.1_130AbBOC	MRFKKISCLLLSPLFIFSTSIYAGNTPKDQEIKKLVDQNFKPLLEKYDVPGMAVGVIQNN
DC-11WP_001211205.1_1097AbBOC	MRFKKISCLLLSPLFIFSTSIYAGNTPKDQEIKKLVDQNFKPLLEKYDVPGMAVGVIQNN
ADC-11WP_001211205.1_684AbBOC	MRFKKISCLLLSPLFIFSTSIYAGNTPKDQEIKKLVDQNFKPLLEKYDVPGMAVGVIQNN
ADC-11WP_001211205.1_682AbBOC	MRFKKISCLLLSPLFIFSTSIYAGNTPKDQEIKKLVDQNFKPLLEKYDVPGMAVGVIQNN
DC-11WP_001211205.1_1111AbBOC	MRFKKISCLLLSPLFIFSTSIYAGNTPKDQEIKKLVDQNFKPLLEKYDVPGMAVGVIQNN
DC-11WP_001211205.1_1059AbBOC	MRFKKISCLLLSPLFIFSTSIYAGNTPKDQEIKKLVDQNFKPLLEKYDVPGMAVGVIQNN
DC-11WP_001211205.1_2754AbBOC	MRFKKISCLLLSPLFIFSTSIYAGNTPKDQEIKKLVDQNFKPLLEKYDVPGMAVGVIQNN
DC-11WP_001211205.1_2603AbBCP	MRFKKISCLLLSPLFIFSTSIYAGNTPKDQEIKKLVDQNFKPLLEKYDVPGMAVGVIQNN
DC-11WP_001211205.1_1138AbBCP	MRFKKISCLLLSPLFIFSTSIYAGNTPKDQEIKKLVDQNFKPLLEKYDVPGMAVGVIQNN
DC-11WP_001211205.1_2874AbBCP	MRFKKISCLLLSPLFIFSTSIYAGNTPKDQEIKKLVDQNFKPLLEKYDVPGMAVGVIQNN
DC-11WP_001211205.1_2686AbBCP	MRFKKISCLLLSPLFIFSTSIYAGNTPKDQEIKKLVDQNFKPLLEKYDVPGMAVGVIQNN
ADC-11WP_001211205.1_130AbBCP	MRFKKISCLLLSPLFIFSTSIYAGNTPKDQEIKKLVDQNFKPLLEKYDVPGMAVGVIQNN
DC-11WP_001211205.1_1097AbBCP	MRFKKISCLLLSPLFIFSTSIYAGNTPKDQEIKKLVDQNFKPLLEKYDVPGMAVGVIQNN
ADC-11WP_001211205.1_684AbBCP	MRFKKISCLLLSPLFIFSTSIYAGNTPKDQEIKKLVDQNFKPLLEKYDVPGMAVGVIQNN
ADC-11WP_001211205.1_682AbBCP	MRFKKISCLLLSPLFIFSTSIYAGNTPKDQEIKKLVDQNFKPLLEKYDVPGMAVGVIQNN
DC-11WP_001211205.1_1111AbBCP	MRFKKISCLLLSPLFIFSTSIYAGNTPKDQEIKKLVDQNFKPLLEKYDVPGMAVGVIQNN
DC-11WP_001211205.1_1059AbBCP	MRFKKISCLLLSPLFIFSTSIYAGNTPKDQEIKKLVDQNFKPLLEKYDVPGMAVGVIQNN
DC-11WP_001211205.1_2754AbBCP	MRFKKISCLLLSPLFIFSTSIYAGNTPKDQEIKKLVDQNFKPLLEKYDVPGMAVGVIQNN
C-269WP_032025626.1_2988AbBOC	MRFKKISCLLLSPLFIFSTSIYAGNTPKDQEIKKLVDQNFKPLLEKYDVPGMAVGVIQNN
C-269WP_032025626.1_2988AbBCP	MRFKKISCLLLSPLFIFSTSIYAGNTPKDQEIKKLVDQNFKPLLEKYDVPGMAVGVIQNN
C-166WP_101244937.1_1028AbBOC	MRFKKISCLLLSPLFIFSTSIYAGNTPKDQEIKKLVDQNFKPLLEKYDVPGMAVGVIQNN
C-166WP_101244937.1_1028AbBCP	MRFKKISCLLLSPLFIFSTSIYAGNTPKDQEIKKLVDQNFKPLLEKYDVPGMAVGVIQNN
C-175WP_001211208.1_2561AbBOC	MRFKKISCLLLSPLFIFSTSIYAGNTPKDQEIKKLVDQNFKPLLEKYDVPGMAVGVIQNN
C-175WP_001211208.1_2561AbBCP	MRFKKISCLLLSPLFIFSTSIYAGNTPKDQEIKKLVDQNFKPLLEKYDVPGMAVGVIQNN
C-154WP_005138362.1_2451AbBOC	MRFKKISCLLLSPLFIFNTSIYAGNTPKDQEIKKLVDQNFKPLLEKYDVPGMAVGVIQNN
DC-154WP_005138362.1_158AbBOC	MRFKKISCLLLSPLFIFNTSIYAGNTPKDQEIKKLVDQNFKPLLEKYDVPGMAVGVIQNN

C-154WP_005138362.1_1167AbBOC	MRFKKISCLLLSPLFIFNTSIYAGNTPKDQEIKKLVDQNFKPLLEKYDVPGMAVGVIQNN
C-154WP_005138362.1_2510AbBOC	MRFKKISCLLLSPLFIFNTSIYAGNTPKDQEIKKLVDQNFKPLLEKYDVPGMAVGVIQNN
C-154WP_005138362.1_2513AbBOC	MRFKKISCLLLSPLFIFNTSIYAGNTPKDQEIKKLVDQNFKPLLEKYDVPGMAVGVIQNN
C-154WP_005138362.1_2512AbBOC	MRFKKISCLLLSPLFIFNTSIYAGNTPKDQEIKKLVDQNFKPLLEKYDVPGMAVGVIQNN
C-154WP_005138362.1_2451AbBCP	MRFKKISCLLLSPLFIFNTSIYAGNTPKDQEIKKLVDQNFKPLLEKYDVPGMAVGVIQNN
DC-154WP_005138362.1_158AbBCP	MRFKKISCLLLSPLFIFNTSIYAGNTPKDQEIKKLVDQNFKPLLEKYDVPGMAVGVIQNN
C-154WP_005138362.1_1167AbBCP	MRFKKISCLLLSPLFIFNTSIYAGNTPKDQEIKKLVDQNFKPLLEKYDVPGMAVGVIQNN
C-154WP_005138362.1_2510AbBCP	MRFKKISCLLLSPLFIFNTSIYAGNTPKDQEIKKLVDQNFKPLLEKYDVPGMAVGVIQNN
C-154WP_005138362.1_2513AbBCP	MRFKKISCLLLSPLFIFNTSIYAGNTPKDQEIKKLVDQNFKPLLEKYDVPGMAVGVIQNN
C-154WP_005138362.1_2512AbBCP	MRFKKISCLLLSPLFIFNTSIYAGNTPKDQEIKKLVDQNFKPLLEKYDVPGMAVGVIQNN
C-240WP_001211207.1_1200AbBOC	MRFKKISCLLLSPLFIFSTSIYAGNTPKDQEIKKLVDQNFKPLLEKYDVPGMAVGVIQNN
C-240WP_001211207.1_1200AbBCP	MRFKKISCLLLSPLFIFSTSIYAGNTPKDQEIKKLVDQNFKPLLEKYDVPGMAVGVIQNN
ADC-75WP_063857817.1_1610AbOC	MRFKKISCLLLSPLFIFSTSIYAGNTPKDQEIKKLVDQNFKPLLEKYDVPGMAVGVIQNN
DC-18WP_002118772.1_1702AbBOC	MRFKKISCLLLPPLFIFSTSIYAGNTPKEQEVKKLVDQNFKPLLDKYDVPGMAVGVIQNN
DC-18WP_002118772.1_1702AbBCP	MRFKKISCLLLPPLFIFSTSIYAGNTPKEQEVKKLVDQNFKPLLDKYDVPGMAVGVIQNN
C-106WP_068981613.1_2650AbBOC	MRFKKISCLLLSPLFIFSTSIYAGNTPKDQEIKKLVDQNFKPLLEKYDVPGMAVGVIQNN
C-106WP_068981613.1_2650AbBCP	MRFKKISCLLLSPLFIFSTSIYAGNTPKDQEIKKLVDQNFKPLLEKYDVPGMAVGVIQNN
DC-50WP_031965243.1_2562AbBOC	MRFKKISCLLFSPLFIFSTSIYAGNTPKDQEIKKLVDQNFKPLLEKYDVPGMAVGVIQNN
ADC-50WP_031965243.1_206AbBOC	MRFKKISCLLFSPLFIFSTSIYAGNTPKDQEIKKLVDQNFKPLLEKYDVPGMAVGVIQNN
DC-50WP_031965243.1_2751AbBOC	MRFKKISCLLFSPLFIFSTSIYAGNTPKDQEIKKLVDQNFKPLLEKYDVPGMAVGVIQNN
DC-50WP_031965243.1_3766AbBOC	MRFKKISCLLFSPLFIFSTSIYAGNTPKDQEIKKLVDQNFKPLLEKYDVPGMAVGVIQNN
DC-50WP_031965243.1_2562AbBCP	MRFKKISCLLFSPLFIFSTSIYAGNTPKDQEIKKLVDQNFKPLLEKYDVPGMAVGVIQNN
ADC-50WP_031965243.1_206AbBCP	MRFKKISCLLFSPLFIFSTSIYAGNTPKDQEIKKLVDQNFKPLLEKYDVPGMAVGVIQNN
DC-50WP_031965243.1_2751AbBCP	MRFKKISCLLFSPLFIFSTSIYAGNTPKDQEIKKLVDQNFKPLLEKYDVPGMAVGVIQNN
DC-50WP_031965243.1_3766AbBCP	MRFKKISCLLFSPLFIFSTSIYAGNTPKDQEIKKLVDQNFKPLLEKYDVPGMAVGVIQNN
ADC-158WP_001211227.1_990AbOC	MRFKKISCLLLSPLFIFSTSIYAGNTPKDQEIKKLVDQNFKPLLEKYDVPGMAVGVIQNN
DC-158WP_001211227.1_1295AbOC	MRFKKISCLLLSPLFIFSTSIYAGNTPKDQEIKKLVDQNFKPLLEKYDVPGMAVGVIQNN
ADC-158WP_001211227.1_6AbOC	MRFKKISCLLLSPLFIFSTSIYAGNTPKDQEIKKLVDQNFKPLLEKYDVPGMAVGVIQNN
DC-158WP_001211227.1_991AbBOC	MRFKKISCLLLSPLFIFSTSIYAGNTPKDQEIKKLVDQNFKPLLEKYDVPGMAVGVIQNN
DC-158WP_001211227.1_207AbBOC	MRFKKISCLLLSPLFIFSTSIYAGNTPKDQEIKKLVDQNFKPLLEKYDVPGMAVGVIQNN
C-158WP_001211227.1_2735AbBOC	MRFKKISCLLLSPLFIFSTSIYAGNTPKDQEIKKLVDQNFKPLLEKYDVPGMAVGVIQNN
DC-158WP_001211227.1_996AbBOC	MRFKKISCLLLSPLFIFSTSIYAGNTPKDQEIKKLVDQNFKPLLEKYDVPGMAVGVIQNN
DC-158WP_001211227.1_796AbBOC	MRFKKISCLLLSPLFIFSTSIYAGNTPKDQEIKKLVDQNFKPLLEKYDVPGMAVGVIQNN
C-158WP_001211227.1_2822AbBOC	MRFKKISCLLLSPLFIFSTSIYAGNTPKDQEIKKLVDQNFKPLLEKYDVPGMAVGVIQNN
C-158WP_001211227.1_1339AbBOC	MRFKKISCLLLSPLFIFSTSIYAGNTPKDQEIKKLVDQNFKPLLEKYDVPGMAVGVIQNN
ADC-158WGQ04828.1_2712AbBOC	MRFKKISCLLLSPLFIFSTSIYAGNTPKDQEIKKLVDQNFKPLLEKYDVPGMAVGVIQNN
DC-158WP_001211227.1_991AbBCP	MRFKKISCLLLSPLFIFSTSIYAGNTPKDQEIKKLVDQNFKPLLEKYDVPGMAVGVIQNN
DC-158WP_001211227.1_207AbBCP	MRFKKISCLLLSPLFIFSTSIYAGNTPKDQEIKKLVDQNFKPLLEKYDVPGMAVGVIQNN
C-158WP_001211227.1_2735AbBCP	MRFKKISCLLLSPLFIFSTSIYAGNTPKDQEIKKLVDQNFKPLLEKYDVPGMAVGVIQNN
DC-158WP_001211227.1_996AbBCP	MRFKKISCLLLSPLFIFSTSIYAGNTPKDQEIKKLVDQNFKPLLEKYDVPGMAVGVIQNN
DC-158WP_001211227.1_796AbBCP	MRFKKISCLLLSPLFIFSTSIYAGNTPKDQEIKKLVDQNFKPLLEKYDVPGMAVGVIQNN
C-158WP_001211227.1_2822AbBCP	MRFKKISCLLLSPLFIFSTSIYAGNTPKDQEIKKLVDQNFKPLLEKYDVPGMAVGVIQNN
C-158WP_001211227.1_1339AbBCP	MRFKKISCLLLSPLFIFSTSIYAGNTPKDQEIKKLVDQNFKPLLEKYDVPGMAVGVIQNN
ADC-158WGQ04828.1_2712AbBCP	MRFKKISCLLLSPLFIFSTSIYAGNTPKDQEIKKLVDQNFKPLLEKYDVPGMAVGVIQNN
ADC-31WP_001211223.1_2986AbOC	MRFKKISCLLLSPLFIFSTSIYAGNTPKDQEIKKLVDQNFKPLLEKYDVPGMAVGVIQNN
DC-31WP_001211223.1_2698AbBOC	MRFKKISCLLLSPLFIFSTSIYAGNTPKDQEIKKLVDQNFKPLLEKYDVPGMAVGVIQNN
DC-31WP_001211223.1_2698AbBCP	MRFKKISCLLLSPLFIFSTSIYAGNTPKDQEIKKLVDQNFKPLLEKYDVPGMAVGVIQNN
ADC-30WP_001211218.1_1102AbOC	MRFKKISCLLLSPLFIFSTSIYAGNTPKDQEIKKLVDQNFKPLLEKYDVPGMAVGVIQNN
ADC-30WP_001211218.1_2565AbOC	MRFKKISCLLLSPLFIFSTSIYAGNTPKDQEIKKLVDQNFKPLLEKYDVPGMAVGVIQNN
ADC-30WP_001211218.1_1021AbOC	MRFKKISCLLLSPLFIFSTSIYAGNTPKDQEIKKLVDQNFKPLLEKYDVPGMAVGVIQNN
ADC-30WP_001211218.1_1088AbOC	MRFKKISCLLLSPLFIFSTSIYAGNTPKDQEIKKLVDQNFKPLLEKYDVPGMAVGVIQNN
ADC-30WP_001211218.1_1135AbOC	MRFKKISCLLLSPLFIFSTSIYAGNTPKDQEIKKLVDQNFKPLLEKYDVPGMAVGVIQNN
ADC-30WP_001211218.1_43AbOC	MRFKKISCLLLSPLFIFSTSIYAGNTPKDQEIKKLVDQNFKPLLEKYDVPGMAVGVIQNN
ADC-30WP_001211218.1_1041AbOC	MRFKKISCLLLSPLFIFSTSIYAGNTPKDQEIKKLVDQNFKPLLEKYDVPGMAVGVIQNN
ADC-30WP_001211218.1_1032AbOC	MRFKKISCLLLSPLFIFSTSIYAGNTPKDQEIKKLVDQNFKPLLEKYDVPGMAVGVIQNN
ADC-30WP_001211218.1_1047AbOC	MRFKKISCLLLSPLFIFSTSIYAGNTPKDQEIKKLVDQNFKPLLEKYDVPGMAVGVIQNN
ADC-30WP_001211218.1_1040AbOC	MRFKKISCLLLSPLFIFSTSIYAGNTPKDQEIKKLVDQNFKPLLEKYDVPGMAVGVIQNN
ADC-30WP_001211218.1_3388AbOC	MRFKKISCLLLSPLFIFSTSIYAGNTPKDQEIKKLVDQNFKPLLEKYDVPGMAVGVIQNN
ADC-30WP_001211218.1_650AbOC	MRFKKISCLLLSPLFIFSTSIYAGNTPKDQEIKKLVDQNFKPLLEKYDVPGMAVGVIQNN
ADC-30WP_001211218.1_2855AbOC	MRFKKISCLLLSPLFIFSTSIYAGNTPKDQEIKKLVDQNFKPLLEKYDVPGMAVGVIQNN
ADC-30WP_001211218.1_1034AbOC	MRFKKISCLLLSPLFIFSTSIYAGNTPKDQEIKKLVDQNFKPLLEKYDVPGMAVGVIQNN
ADC-30WP_001211218.1_79AbOC	MRFKKISCLLLSPLFIFSTSIYAGNTPKDQEIKKLVDQNFKPLLEKYDVPGMAVGVIQNN
ADC-30WP_001211218.1_3509AbOC	MRFKKISCLLLSPLFIFSTSIYAGNTPKDQEIKKLVDQNFKPLLEKYDVPGMAVGVIQNN
ADC-30WP_001211218.1_2399AbOC	MRFKKISCLLLSPLFIFSTSIYAGNTPKDQEIKKLVDQNFKPLLEKYDVPGMAVGVIQNN
ADC-30WP_001211218.1_2415AbOC	MRFKKISCLLLSPLFIFSTSIYAGNTPKDQEIKKLVDQNFKPLLEKYDVPGMAVGVIQNN
ADC-30WP_001211218.1_3032AbOC	MRFKKISCLLLSPLFIFSTSIYAGNTPKDQEIKKLVDQNFKPLLEKYDVPGMAVGVIQNN
ADC-30WP_001211218.1_832AbOC	MRFKKISCLLLSPLFIFSTSIYAGNTPKDQEIKKLVDQNFKPLLEKYDVPGMAVGVIQNN
ADC-30WP_001211218.1_1428AbOC	MRFKKISCLLLSPLFIFSTSIYAGNTPKDQEIKKLVDQNFKPLLEKYDVPGMAVGVIQNN
ADC-30WP_001211218.1_2340AbOC	MRFKKISCLLLSPLFIFSTSIYAGNTPKDQEIKKLVDQNFKPLLEKYDVPGMAVGVIQNN
ADC-30WP_001211218.1_2976AbOC	MRFKKISCLLLSPLFIFSTSIYAGNTPKDQEIKKLVDQNFKPLLEKYDVPGMAVGVIQNN
ADC-30WP_001211218.1_2305AbOC	MRFKKISCLLLSPLFIFSTSIYAGNTPKDQEIKKLVDQNFKPLLEKYDVPGMAVGVIQNN
ADC-30WP_001211218.1_1110AbOC	MRFKKISCLLLSPLFIFSTSIYAGNTPKDQEIKKLVDQNFKPLLEKYDVPGMAVGVIQNN
ADC-30WP_001211218.1_2797AbOC	MRFKKISCLLLSPLFIFSTSIYAGNTPKDQEIKKLVDQNFKPLLEKYDVPGMAVGVIQNN
ADC-30WP_001211218.1_1023AbOC	MRFKKISCLLLSPLFIFSTSIYAGNTPKDQEIKKLVDQNFKPLLEKYDVPGMAVGVIQNN
ADC-30WP_001211218.1_1022AbOC	MRFKKISCLLLSPLFIFSTSIYAGNTPKDQEIKKLVDQNFKPLLEKYDVPGMAVGVIQNN
ADC-30WP_001211218.1_2458AbOC	MRFKKISCLLLSPLFIFSTSIYAGNTPKDQEIKKLVDQNFKPLLEKYDVPGMAVGVIQNN
ADC-30WP_001211218.1_2513AbOC	MRFKKISCLLLSPLFIFSTSIYAGNTPKDQEIKKLVDQNFKPLLEKYDVPGMAVGVIQNN
ADC-30WP_001211218.1_2482AbOC	MRFKKISCLLLSPLFIFSTSIYAGNTPKDQEIKKLVDQNFKPLLEKYDVPGMAVGVIQNN
DC-30WP_001211218.1_2692AbBOC	MRFKKISCLLLSPLFIFSTSIYAGNTPKDQEIKKLVDQNFKPLLEKYDVPGMAVGVIQNN
DC-30WP_001211218.1_2549AbBOC	MRFKKISCLLLSPLFIFSTSIYAGNTPKDQEIKKLVDQNFKPLLEKYDVPGMAVGVIQNN
DC-30WP_001211218.1_1026AbBOC	MRFKKISCLLLSPLFIFSTSIYAGNTPKDQEIKKLVDQNFKPLLEKYDVPGMAVGVIQNN
ADC-30WP_001211218.1_373AbBOC	MRFKKISCLLLSPLFIFSTSIYAGNTPKDQEIKKLVDQNFKPLLEKYDVPGMAVGVIQNN
DC-30WP_001211218.1_2465AbBOC	MRFKKISCLLLSPLFIFSTSIYAGNTPKDQEIKKLVDQNFKPLLEKYDVPGMAVGVIQNN
DC-30WP_001211218.1_1027AbBOC	MRFKKISCLLLSPLFIFSTSIYAGNTPKDQEIKKLVDQNFKPLLEKYDVPGMAVGVIQNN
DC-30WP_001211218.1_2650AbBOC	MRFKKISCLLLSPLFIFSTSIYAGNTPKDQEIKKLVDQNFKPLLEKYDVPGMAVGVIQNN
DC-30WP_001211218.1_2674AbBOC	MRFKKISCLLLSPLFIFSTSIYAGNTPKDQEIKKLVDQNFKPLLEKYDVPGMAVGVIQNN
DC-30WP_001211218.1_2689AbBOC	MRFKKISCLLLSPLFIFSTSIYAGNTPKDQEIKKLVDQNFKPLLEKYDVPGMAVGVIQNN
DC-30WP_001211218.1_1105AbBOC	MRFKKISCLLLSPLFIFSTSIYAGNTPKDQEIKKLVDQNFKPLLEKYDVPGMAVGVIQNN
DC-30WP_001211218.1_2670AbBOC	MRFKKISCLLLSPLFIFSTSIYAGNTPKDQEIKKLVDQNFKPLLEKYDVPGMAVGVIQNN
DC-30WP_001211218.1_1019AbBOC	MRFKKISCLLLSPLFIFSTSIYAGNTPKDQEIKKLVDQNFKPLLEKYDVPGMAVGVIQNN
DC-30WP_001211218.1_1039AbBOC	MRFKKISCLLLSPLFIFSTSIYAGNTPKDQEIKKLVDQNFKPLLEKYDVPGMAVGVIQNN
DC-30WP_001211218.1_1023AbBOC	MRFKKISCLLLSPLFIFSTSIYAGNTPKDQEIKKLVDQNFKPLLEKYDVPGMAVGVIQNN
DC-30WP_001211218.1_3189AbBOC	MRFKKISCLLLSPLFIFSTSIYAGNTPKDQEIKKLVDQNFKPLLEKYDVPGMAVGVIQNN
ADC-30WP_001211218.1_594AbBOC	MRFKKISCLLLSPLFIFSTSIYAGNTPKDQEIKKLVDQNFKPLLEKYDVPGMAVGVIQNN
DC-30WP_001211218.1_1718AbBOC	MRFKKISCLLLSPLFIFSTSIYAGNTPKDQEIKKLVDQNFKPLLEKYDVPGMAVGVIQNN
DC-30WP_001211218.1_2484AbBOC	MRFKKISCLLLSPLFIFSTSIYAGNTPKDQEIKKLVDQNFKPLLEKYDVPGMAVGVIQNN
DC-30WP_001211218.1_3305AbBOC	MRFKKISCLLLSPLFIFSTSIYAGNTPKDQEIKKLVDQNFKPLLEKYDVPGMAVGVIQNN
DC-30WP_001211218.1_3346AbBOC	MRFKKISCLLLSPLFIFSTSIYAGNTPKDQEIKKLVDQNFKPLLEKYDVPGMAVGVIQNN
DC-30WP_001211218.1_1482AbBOC	MRFKKISCLLLSPLFIFSTSIYAGNTPKDQEIKKLVDQNFKPLLEKYDVPGMAVGVIQNN
DC-30WP_001211218.1_2574AbBOC	MRFKKISCLLLSPLFIFSTSIYAGNTPKDQEIKKLVDQNFKPLLEKYDVPGMAVGVIQNN
ADC-30WP_001211218.1_831AbBOC	MRFKKISCLLLSPLFIFSTSIYAGNTPKDQEIKKLVDQNFKPLLEKYDVPGMAVGVIQNN
DC-30WP_001211218.1_1826AbBOC	MRFKKISCLLLSPLFIFSTSIYAGNTPKDQEIKKLVDQNFKPLLEKYDVPGMAVGVIQNN
DC-30WP_001211218.1_1024AbBOC	MRFKKISCLLLSPLFIFSTSIYAGNTPKDQEIKKLVDQNFKPLLEKYDVPGMAVGVIQNN
DC-30WP_001211218.1_1618AbBOC	MRFKKISCLLLSPLFIFSTSIYAGNTPKDQEIKKLVDQNFKPLLEKYDVPGMAVGVIQNN
ADC-30WP_001211218.1_301AbBOC	MRFKKISCLLLSPLFIFSTSIYAGNTPKDQEIKKLVDQNFKPLLEKYDVPGMAVGVIQNN
ADC-30WP_001211218.1_940AbBOC	MRFKKISCLLLSPLFIFSTSIYAGNTPKDQEIKKLVDQNFKPLLEKYDVPGMAVGVIQNN
DC-30WP_001211218.1_2949AbBOC	MRFKKISCLLLSPLFIFSTSIYAGNTPKDQEIKKLVDQNFKPLLEKYDVPGMAVGVIQNN
DC-30WP_001211218.1_1028AbBOC	MRFKKISCLLLSPLFIFSTSIYAGNTPKDQEIKKLVDQNFKPLLEKYDVPGMAVGVIQNN
DC-30WP_001211218.1_2526AbBOC	MRFKKISCLLLSPLFIFSTSIYAGNTPKDQEIKKLVDQNFKPLLEKYDVPGMAVGVIQNN
DC-30WP_001211218.1_1040AbBOC	MRFKKISCLLLSPLFIFSTSIYAGNTPKDQEIKKLVDQNFKPLLEKYDVPGMAVGVIQNN
DC-30WP_001211218.1_1089AbBOC	MRFKKISCLLLSPLFIFSTSIYAGNTPKDQEIKKLVDQNFKPLLEKYDVPGMAVGVIQNN
DC-30WP_001211218.1_1099AbBOC	MRFKKISCLLLSPLFIFSTSIYAGNTPKDQEIKKLVDQNFKPLLEKYDVPGMAVGVIQNN
DC-30WP_001211218.1_1206AbBOC	MRFKKISCLLLSPLFIFSTSIYAGNTPKDQEIKKLVDQNFKPLLEKYDVPGMAVGVIQNN
DC-30WP_001211218.1_1167AbBOC	MRFKKISCLLLSPLFIFSTSIYAGNTPKDQEIKKLVDQNFKPLLEKYDVPGMAVGVIQNN
DC-30WP_001211218.1_1186AbBOC	MRFKKISCLLLSPLFIFSTSIYAGNTPKDQEIKKLVDQNFKPLLEKYDVPGMAVGVIQNN
DC-30WP_001211218.1_1165AbBOC	MRFKKISCLLLSPLFIFSTSIYAGNTPKDQEIKKLVDQNFKPLLEKYDVPGMAVGVIQNN
DC-30WP_001211218.1_1077AbBOC	MRFKKISCLLLSPLFIFSTSIYAGNTPKDQEIKKLVDQNFKPLLEKYDVPGMAVGVIQNN
DC-30WP_001211218.1_1078AbBOC	MRFKKISCLLLSPLFIFSTSIYAGNTPKDQEIKKLVDQNFKPLLEKYDVPGMAVGVIQNN
DC-30WP_001211218.1_1071AbBOC	MRFKKISCLLLSPLFIFSTSIYAGNTPKDQEIKKLVDQNFKPLLEKYDVPGMAVGVIQNN
DC-30WP_001211218.1_2694AbBOC	MRFKKISCLLLSPLFIFSTSIYAGNTPKDQEIKKLVDQNFKPLLEKYDVPGMAVGVIQNN
DC-30WP_001211218.1_3340AbBOC	MRFKKISCLLLSPLFIFSTSIYAGNTPKDQEIKKLVDQNFKPLLEKYDVPGMAVGVIQNN
DC-30WP_001211218.1_1022AbBOC	MRFKKISCLLLSPLFIFSTSIYAGNTPKDQEIKKLVDQNFKPLLEKYDVPGMAVGVIQNN
DC-30WP_001211218.1_1025AbBOC	MRFKKISCLLLSPLFIFSTSIYAGNTPKDQEIKKLVDQNFKPLLEKYDVPGMAVGVIQNN
DC-30WP_001211218.1_1158AbBOC	MRFKKISCLLLSPLFIFSTSIYAGNTPKDQEIKKLVDQNFKPLLEKYDVPGMAVGVIQNN
DC-30WP_001211218.1_1020AbBOC	MRFKKISCLLLSPLFIFSTSIYAGNTPKDQEIKKLVDQNFKPLLEKYDVPGMAVGVIQNN
DC-30WP_001211218.1_1021AbBOC	MRFKKISCLLLSPLFIFSTSIYAGNTPKDQEIKKLVDQNFKPLLEKYDVPGMAVGVIQNN
ADC-30WP_001211218.1_532AbBOC	MRFKKISCLLLSPLFIFSTSIYAGNTPKDQEIKKLVDQNFKPLLEKYDVPGMAVGVIQNN
ADC-30WP_001211218.1_539AbBOC	MRFKKISCLLLSPLFIFSTSIYAGNTPKDQEIKKLVDQNFKPLLEKYDVPGMAVGVIQNN
DC-30WP_001211218.1_1033AbBOC	MRFKKISCLLLSPLFIFSTSIYAGNTPKDQEIKKLVDQNFKPLLEKYDVPGMAVGVIQNN
DC-30WP_001211218.1_1069AbBOC	MRFKKISCLLLSPLFIFSTSIYAGNTPKDQEIKKLVDQNFKPLLEKYDVPGMAVGVIQNN
DC-30WP_001211218.1_1029AbBOC	MRFKKISCLLLSPLFIFSTSIYAGNTPKDQEIKKLVDQNFKPLLEKYDVPGMAVGVIQNN
DC-30WP_001211218.1_1030AbBOC	MRFKKISCLLLSPLFIFSTSIYAGNTPKDQEIKKLVDQNFKPLLEKYDVPGMAVGVIQNN
DC-30WP_001211218.1_1096AbBOC	MRFKKISCLLLSPLFIFSTSIYAGNTPKDQEIKKLVDQNFKPLLEKYDVPGMAVGVIQNN
DC-30WP_001211218.1_1037AbBOC	MRFKKISCLLLSPLFIFSTSIYAGNTPKDQEIKKLVDQNFKPLLEKYDVPGMAVGVIQNN
DC-30WP_001211218.1_1098AbBOC	MRFKKISCLLLSPLFIFSTSIYAGNTPKDQEIKKLVDQNFKPLLEKYDVPGMAVGVIQNN
DC-30WP_001211218.1_1046AbBOC	MRFKKISCLLLSPLFIFSTSIYAGNTPKDQEIKKLVDQNFKPLLEKYDVPGMAVGVIQNN
DC-30WP_001211218.1_1032AbBOC	MRFKKISCLLLSPLFIFSTSIYAGNTPKDQEIKKLVDQNFKPLLEKYDVPGMAVGVIQNN
DC-30WP_001211218.1_1070AbBOC	MRFKKISCLLLSPLFIFSTSIYAGNTPKDQEIKKLVDQNFKPLLEKYDVPGMAVGVIQNN
DC-30WP_001211218.1_1139AbBOC	MRFKKISCLLLSPLFIFSTSIYAGNTPKDQEIKKLVDQNFKPLLEKYDVPGMAVGVIQNN
DC-30WP_001211218.1_1031AbBOC	MRFKKISCLLLSPLFIFSTSIYAGNTPKDQEIKKLVDQNFKPLLEKYDVPGMAVGVIQNN
DC-30WP_001211218.1_1066AbBOC	MRFKKISCLLLSPLFIFSTSIYAGNTPKDQEIKKLVDQNFKPLLEKYDVPGMAVGVIQNN
DC-30WP_001211218.1_1102AbBOC	MRFKKISCLLLSPLFIFSTSIYAGNTPKDQEIKKLVDQNFKPLLEKYDVPGMAVGVIQNN
ADC-30USX57838.1_2957AbBOC	MRFKKISCLLLSPLFIFSTSIYAGNTPKDQEIKKLVDQNFKPLLEKYDVPGMAVGVIQNN
ADC-30USX59985.1_1918AbBOC	MRFKKISCLLLSPLFIFSTSIYAGNTPKDQEIKKLVDQNFKPLLEKYDVPGMAVGVIQNN
ADC-30USX63799.1_2772AbBOC	MRFKKISCLLLSPLFIFSTSIYAGNTPKDQEIKKLVDQNFKPLLEKYDVPGMAVGVIQNN
ADC-30USX74012.1_305AbBOC	MRFKKISCLLLSPLFIFSTSIYAGNTPKDQEIKKLVDQNFKPLLEKYDVPGMAVGVIQNN
ADC-30USZ52468.1_2397AbBOC	MRFKKISCLLLSPLFIFSTSIYAGNTPKDQEIKKLVDQNFKPLLEKYDVPGMAVGVIQNN
DC-30WP_001211218.1_2008AbBOC	MRFKKISCLLLSPLFIFSTSIYAGNTPKDQEIKKLVDQNFKPLLEKYDVPGMAVGVIQNN
ADC-30WCL78976.1_1026AbBOC	MRFKKISCLLLSPLFIFSTSIYAGNTPKDQEIKKLVDQNFKPLLEKYDVPGMAVGVIQNN
ADC-30WDO27826.1_1034AbBOC	MRFKKISCLLLSPLFIFSTSIYAGNTPKDQEIKKLVDQNFKPLLEKYDVPGMAVGVIQNN
DC-30WP_001211218.1_2125AbBOC	MRFKKISCLLLSPLFIFSTSIYAGNTPKDQEIKKLVDQNFKPLLEKYDVPGMAVGVIQNN
DC-30WP_001211218.1_2729AbBOC	MRFKKISCLLLSPLFIFSTSIYAGNTPKDQEIKKLVDQNFKPLLEKYDVPGMAVGVIQNN

```
DC-30WP_001211218.1_2692AbBCP    MRFKKISCLLLSPLFIFSTSIYAGNTPKDQEIKKLVDQNFKPLLEKYDVPGMAVGVIQNN
DC-30WP_001211218.1_2549AbBCP    MRFKKISCLLLSPLFIFSTSIYAGNTPKDQEIKKLVDQNFKPLLEKYDVPGMAVGVIQNN
DC-30WP_001211218.1_1026AbBCP    MRFKKISCLLLSPLFIFSTSIYAGNTPKDQEIKKLVDQNFKPLLEKYDVPGMAVGVIQNN
ADC-30WP_001211218.1_373AbBCP    MRFKKISCLLLSPLFIFSTSIYAGNTPKDQEIKKLVDQNFKPLLEKYDVPGMAVGVIQNN
DC-30WP_001211218.1_2465AbBCP    MRFKKISCLLLSPLFIFSTSIYAGNTPKDQEIKKLVDQNFKPLLEKYDVPGMAVGVIQNN
DC-30WP_001211218.1_1027AbBCP    MRFKKISCLLLSPLFIFSTSIYAGNTPKDQEIKKLVDQNFKPLLEKYDVPGMAVGVIQNN
DC-30WP_001211218.1_2650AbBCP    MRFKKISCLLLSPLFIFSTSIYAGNTPKDQEIKKLVDQNFKPLLEKYDVPGMAVGVIQNN
DC-30WP_001211218.1_2674AbBCP    MRFKKISCLLLSPLFIFSTSIYAGNTPKDQEIKKLVDQNFKPLLEKYDVPGMAVGVIQNN
DC-30WP_001211218.1_2689AbBCP    MRFKKISCLLLSPLFIFSTSIYAGNTPKDQEIKKLVDQNFKPLLEKYDVPGMAVGVIQNN
DC-30WP_001211218.1_1105AbBCP    MRFKKISCLLLSPLFIFSTSIYAGNTPKDQEIKKLVDQNFKPLLEKYDVPGMAVGVIQNN
DC-30WP_001211218.1_2670AbBCP    MRFKKISCLLLSPLFIFSTSIYAGNTPKDQEIKKLVDQNFKPLLEKYDVPGMAVGVIQNN
DC-30WP_001211218.1_1019AbBCP    MRFKKISCLLLSPLFIFSTSIYAGNTPKDQEIKKLVDQNFKPLLEKYDVPGMAVGVIQNN
DC-30WP_001211218.1_1039AbBCP    MRFKKISCLLLSPLFIFSTSIYAGNTPKDQEIKKLVDQNFKPLLEKYDVPGMAVGVIQNN
DC-30WP_001211218.1_1023AbBCP    MRFKKISCLLLSPLFIFSTSIYAGNTPKDQEIKKLVDQNFKPLLEKYDVPGMAVGVIQNN
DC-30WP_001211218.1_3189AbBCP    MRFKKISCLLLSPLFIFSTSIYAGNTPKDQEIKKLVDQNFKPLLEKYDVPGMAVGVIQNN
ADC-30WP_001211218.1_594AbBCP    MRFKKISCLLLSPLFIFSTSIYAGNTPKDQEIKKLVDQNFKPLLEKYDVPGMAVGVIQNN
DC-30WP_001211218.1_1718AbBCP    MRFKKISCLLLSPLFIFSTSIYAGNTPKDQEIKKLVDQNFKPLLEKYDVPGMAVGVIQNN
DC-30WP_001211218.1_2484AbBCP    MRFKKISCLLLSPLFIFSTSIYAGNTPKDQEIKKLVDQNFKPLLEKYDVPGMAVGVIQNN
DC-30WP_001211218.1_3305AbBCP    MRFKKISCLLLSPLFIFSTSIYAGNTPKDQEIKKLVDQNFKPLLEKYDVPGMAVGVIQNN
DC-30WP_001211218.1_3346AbBCP    MRFKKISCLLLSPLFIFSTSIYAGNTPKDQEIKKLVDQNFKPLLEKYDVPGMAVGVIQNN
DC-30WP_001211218.1_1482AbBCP    MRFKKISCLLLSPLFIFSTSIYAGNTPKDQEIKKLVDQNFKPLLEKYDVPGMAVGVIQNN
DC-30WP_001211218.1_2574AbBCP    MRFKKISCLLLSPLFIFSTSIYAGNTPKDQEIKKLVDQNFKPLLEKYDVPGMAVGVIQNN
ADC-30WP_001211218.1_831AbBCP    MRFKKISCLLLSPLFIFSTSIYAGNTPKDQEIKKLVDQNFKPLLEKYDVPGMAVGVIQNN
DC-30WP_001211218.1_1826AbBCP    MRFKKISCLLLSPLFIFSTSIYAGNTPKDQEIKKLVDQNFKPLLEKYDVPGMAVGVIQNN
DC-30WP_001211218.1_1024AbBCP    MRFKKISCLLLSPLFIFSTSIYAGNTPKDQEIKKLVDQNFKPLLEKYDVPGMAVGVIQNN
DC-30WP_001211218.1_1618AbBCP    MRFKKISCLLLSPLFIFSTSIYAGNTPKDQEIKKLVDQNFKPLLEKYDVPGMAVGVIQNN
ADC-30WP_001211218.1_301AbBCP    MRFKKISCLLLSPLFIFSTSIYAGNTPKDQEIKKLVDQNFKPLLEKYDVPGMAVGVIQNN
ADC-30WP_001211218.1_940AbBCP    MRFKKISCLLLSPLFIFSTSIYAGNTPKDQEIKKLVDQNFKPLLEKYDVPGMAVGVIQNN
DC-30WP_001211218.1_2949AbBCP    MRFKKISCLLLSPLFIFSTSIYAGNTPKDQEIKKLVDQNFKPLLEKYDVPGMAVGVIQNN
DC-30WP_001211218.1_1028AbBCP    MRFKKISCLLLSPLFIFSTSIYAGNTPKDQEIKKLVDQNFKPLLEKYDVPGMAVGVIQNN
DC-30WP_001211218.1_2526AbBCP    MRFKKISCLLLSPLFIFSTSIYAGNTPKDQEIKKLVDQNFKPLLEKYDVPGMAVGVIQNN
DC-30WP_001211218.1_1040AbBCP    MRFKKISCLLLSPLFIFSTSIYAGNTPKDQEIKKLVDQNFKPLLEKYDVPGMAVGVIQNN
DC-30WP_001211218.1_1089AbBCP    MRFKKISCLLLSPLFIFSTSIYAGNTPKDQEIKKLVDQNFKPLLEKYDVPGMAVGVIQNN
DC-30WP_001211218.1_1099AbBCP    MRFKKISCLLLSPLFIFSTSIYAGNTPKDQEIKKLVDQNFKPLLEKYDVPGMAVGVIQNN
DC-30WP_001211218.1_1206AbBCP    MRFKKISCLLLSPLFIFSTSIYAGNTPKDQEIKKLVDQNFKPLLEKYDVPGMAVGVIQNN
DC-30WP_001211218.1_1167AbBCP    MRFKKISCLLLSPLFIFSTSIYAGNTPKDQEIKKLVDQNFKPLLEKYDVPGMAVGVIQNN
DC-30WP_001211218.1_1186AbBCP    MRFKKISCLLLSPLFIFSTSIYAGNTPKDQEIKKLVDQNFKPLLEKYDVPGMAVGVIQNN
DC-30WP_001211218.1_1165AbBCP    MRFKKISCLLLSPLFIFSTSIYAGNTPKDQEIKKLVDQNFKPLLEKYDVPGMAVGVIQNN
DC-30WP_001211218.1_1077AbBCP    MRFKKISCLLLSPLFIFSTSIYAGNTPKDQEIKKLVDQNFKPLLEKYDVPGMAVGVIQNN
DC-30WP_001211218.1_1078AbBCP    MRFKKISCLLLSPLFIFSTSIYAGNTPKDQEIKKLVDQNFKPLLEKYDVPGMAVGVIQNN
DC-30WP_001211218.1_1071AbBCP    MRFKKISCLLLSPLFIFSTSIYAGNTPKDQEIKKLVDQNFKPLLEKYDVPGMAVGVIQNN
DC-30WP_001211218.1_2694AbBCP    MRFKKISCLLLSPLFIFSTSIYAGNTPKDQEIKKLVDQNFKPLLEKYDVPGMAVGVIQNN
DC-30WP_001211218.1_3340AbBCP    MRFKKISCLLLSPLFIFSTSIYAGNTPKDQEIKKLVDQNFKPLLEKYDVPGMAVGVIQNN
DC-30WP_001211218.1_1022AbBCP    MRFKKISCLLLSPLFIFSTSIYAGNTPKDQEIKKLVDQNFKPLLEKYDVPGMAVGVIQNN
DC-30WP_001211218.1_1025AbBCP    MRFKKISCLLLSPLFIFSTSIYAGNTPKDQEIKKLVDQNFKPLLEKYDVPGMAVGVIQNN
DC-30WP_001211218.1_1158AbBCP    MRFKKISCLLLSPLFIFSTSIYAGNTPKDQEIKKLVDQNFKPLLEKYDVPGMAVGVIQNN
DC-30WP_001211218.1_1020AbBCP    MRFKKISCLLLSPLFIFSTSIYAGNTPKDQEIKKLVDQNFKPLLEKYDVPGMAVGVIQNN
DC-30WP_001211218.1_1021AbBCP    MRFKKISCLLLSPLFIFSTSIYAGNTPKDQEIKKLVDQNFKPLLEKYDVPGMAVGVIQNN
ADC-30WP_001211218.1_532AbBCP    MRFKKISCLLLSPLFIFSTSIYAGNTPKDQEIKKLVDQNFKPLLEKYDVPGMAVGVIQNN
ADC-30WP_001211218.1_539AbBCP    MRFKKISCLLLSPLFIFSTSIYAGNTPKDQEIKKLVDQNFKPLLEKYDVPGMAVGVIQNN
DC-30WP_001211218.1_1033AbBCP    MRFKKISCLLLSPLFIFSTSIYAGNTPKDQEIKKLVDQNFKPLLEKYDVPGMAVGVIQNN
DC-30WP_001211218.1_1069AbBCP    MRFKKISCLLLSPLFIFSTSIYAGNTPKDQEIKKLVDQNFKPLLEKYDVPGMAVGVIQNN
DC-30WP_001211218.1_1029AbBCP    MRFKKISCLLLSPLFIFSTSIYAGNTPKDQEIKKLVDQNFKPLLEKYDVPGMAVGVIQNN
DC-30WP_001211218.1_1030AbBCP    MRFKKISCLLLSPLFIFSTSIYAGNTPKDQEIKKLVDQNFKPLLEKYDVPGMAVGVIQNN
DC-30WP_001211218.1_1096AbBCP    MRFKKISCLLLSPLFIFSTSIYAGNTPKDQEIKKLVDQNFKPLLEKYDVPGMAVGVIQNN
DC-30WP_001211218.1_1037AbBCP    MRFKKISCLLLSPLFIFSTSIYAGNTPKDQEIKKLVDQNFKPLLEKYDVPGMAVGVIQNN
DC-30WP_001211218.1_1098AbBCP    MRFKKISCLLLSPLFIFSTSIYAGNTPKDQEIKKLVDQNFKPLLEKYDVPGMAVGVIQNN
DC-30WP_001211218.1_1046AbBCP    MRFKKISCLLLSPLFIFSTSIYAGNTPKDQEIKKLVDQNFKPLLEKYDVPGMAVGVIQNN
DC-30WP_001211218.1_1032AbBCP    MRFKKISCLLLSPLFIFSTSIYAGNTPKDQEIKKLVDQNFKPLLEKYDVPGMAVGVIQNN
DC-30WP_001211218.1_1070AbBCP    MRFKKISCLLLSPLFIFSTSIYAGNTPKDQEIKKLVDQNFKPLLEKYDVPGMAVGVIQNN
DC-30WP_001211218.1_1139AbBCP    MRFKKISCLLLSPLFIFSTSIYAGNTPKDQEIKKLVDQNFKPLLEKYDVPGMAVGVIQNN
DC-30WP_001211218.1_1031AbBCP    MRFKKISCLLLSPLFIFSTSIYAGNTPKDQEIKKLVDQNFKPLLEKYDVPGMAVGVIQNN
DC-30WP_001211218.1_1066AbBCP    MRFKKISCLLLSPLFIFSTSIYAGNTPKDQEIKKLVDQNFKPLLEKYDVPGMAVGVIQNN
DC-30WP_001211218.1_1102AbBCP    MRFKKISCLLLSPLFIFSTSIYAGNTPKDQEIKKLVDQNFKPLLEKYDVPGMAVGVIQNN
ADC-30USX57838.1_2957AbBCP       MRFKKISCLLLSPLFIFSTSIYAGNTPKDQEIKKLVDQNFKPLLEKYDVPGMAVGVIQNN
ADC-30USX59985.1_1918AbBCP       MRFKKISCLLLSPLFIFSTSIYAGNTPKDQEIKKLVDQNFKPLLEKYDVPGMAVGVIQNN
ADC-30USX63799.1_2772AbBCP       MRFKKISCLLLSPLFIFSTSIYAGNTPKDQEIKKLVDQNFKPLLEKYDVPGMAVGVIQNN
ADC-30USX74012.1_305AbBCP        MRFKKISCLLLSPLFIFSTSIYAGNTPKDQEIKKLVDQNFKPLLEKYDVPGMAVGVIQNN
ADC-30USZ52468.1_2397AbBCP       MRFKKISCLLLSPLFIFSTSIYAGNTPKDQEIKKLVDQNFKPLLEKYDVPGMAVGVIQNN
DC-30WP_001211218.1_2008AbBCP    MRFKKISCLLLSPLFIFSTSIYAGNTPKDQEIKKLVDQNFKPLLEKYDVPGMAVGVIQNN
ADC-30WCL78976.1_1026AbBCP       MRFKKISCLLLSPLFIFSTSIYAGNTPKDQEIKKLVDQNFKPLLEKYDVPGMAVGVIQNN
ADC-30WDO27826.1_1034AbBCP       MRFKKISCLLLSPLFIFSTSIYAGNTPKDQEIKKLVDQNFKPLLEKYDVPGMAVGVIQNN
DC-30WP_001211218.1_2125AbBCP    MRFKKISCLLLSPLFIFSTSIYAGNTPKDQEIKKLVDQNFKPLLEKYDVPGMAVGVIQNN
DC-30WP_001211218.1_2729AbBCP    MRFKKISCLLLSPLFIFSTSIYAGNTPKDQEIKKLVDQNFKPLLEKYDVPGMAVGVIQNN
ADC-30ADX93237.1_2806AbBCP       MRFKKISCLLLSPLFIFSTSIYAGNTPKDQEIKKLVDQNFKPLLEKYDVPGMAVGVIQNN
ADC-82WP_001211216.1_2747AbOC    MRFKKISCLLLSPLFIFSTSIYAGNTPKDQEIKKLVDQNFKPLLEKYDVPGMAVGVIQNN
DC-82WP_001211216.1_1093AbBOC    MRFKKISCLLLSPLFIFSTSIYAGNTPKDQEIKKLVDQNFKPLLEKYDVPGMAVGVIQNN
DC-82WP_001211216.1_1071AbBOC    MRFKKISCLLLSPLFIFSTSIYAGNTPKDQEIKKLVDQNFKPLLEKYDVPGMAVGVIQNN
DC-82WP_001211216.1_1093AbBCP    MRFKKISCLLLSPLFIFSTSIYAGNTPKDQEIKKLVDQNFKPLLEKYDVPGMAVGVIQNN
DC-82WP_001211216.1_1071AbBCP    MRFKKISCLLLSPLFIFSTSIYAGNTPKDQEIKKLVDQNFKPLLEKYDVPGMAVGVIQNN
DC-162WP_031980335.1_1153AbOC    MRFKKISCLLLSPLFIFSTSIYAGNTPKDQEIKKLVDQNFKPLLEKYDVPGMAVGVIQNN
C-162WP_031980335.1_1028AbBOC    MRFKKISCLLLSPLFIFSTSIYAGNTPKDQEIKKLVDQNFKPLLEKYDVPGMAVGVIQNN
C-162WP_031980335.1_1023AbBOC    MRFKKISCLLLSPLFIFSTSIYAGNTPKDQEIKKLVDQNFKPLLEKYDVPGMAVGVIQNN
C-162WP_031980335.1_1028AbBCP    MRFKKISCLLLSPLFIFSTSIYAGNTPKDQEIKKLVDQNFKPLLEKYDVPGMAVGVIQNN
C-162WP_031980335.1_1023AbBCP    MRFKKISCLLLSPLFIFSTSIYAGNTPKDQEIKKLVDQNFKPLLEKYDVPGMAVGVIQNN
ADC-73WP_001211219.1_1012AbOC    MRFKKISCLLLSPLFIFSTSIYAGNTPKDQEIKKLVDQNFKPLLEKYDVPGMAVGVIQNN
ADC-73WP_001211219.1_1953AbOC    MRFKKISCLLLSPLFIFSTSIYAGNTPKDQEIKKLVDQNFKPLLEKYDVPGMAVGVIQNN
ADC-73WP_001211219.1_1014AbOC    MRFKKISCLLLSPLFIFSTSIYAGNTPKDQEIKKLVDQNFKPLLEKYDVPGMAVGVIQNN
ADC-73WP_001211219.1_1041AbOC    MRFKKISCLLLSPLFIFSTSIYAGNTPKDQEIKKLVDQNFKPLLEKYDVPGMAVGVIQNN
ADC-73WP_001211219.1_1028AbOC    MRFKKISCLLLSPLFIFSTSIYAGNTPKDQEIKKLVDQNFKPLLEKYDVPGMAVGVIQNN
ADC-73WP_001211219.1_2674AbOC    MRFKKISCLLLSPLFIFSTSIYAGNTPKDQEIKKLVDQNFKPLLEKYDVPGMAVGVIQNN
ADC-73WP_001211219.1_462AbOC     MRFKKISCLLLSPLFIFSTSIYAGNTPKDQEIKKLVDQNFKPLLEKYDVPGMAVGVIQNN
ADC-73WP_001211219.1_3530AbOC    MRFKKISCLLLSPLFIFSTSIYAGNTPKDQEIKKLVDQNFKPLLEKYDVPGMAVGVIQNN
ADC-73WP_001211219.1_3621AbOC    MRFKKISCLLLSPLFIFSTSIYAGNTPKDQEIKKLVDQNFKPLLEKYDVPGMAVGVIQNN
ADC-73WP_001211219.1_3381AbOC    MRFKKISCLLLSPLFIFSTSIYAGNTPKDQEIKKLVDQNFKPLLEKYDVPGMAVGVIQNN
ADC-73WP_001211219.1_2515AbOC    MRFKKISCLLLSPLFIFSTSIYAGNTPKDQEIKKLVDQNFKPLLEKYDVPGMAVGVIQNN
ADC-73WP_001211219.1_3574AbOC    MRFKKISCLLLSPLFIFSTSIYAGNTPKDQEIKKLVDQNFKPLLEKYDVPGMAVGVIQNN
ADC-73WP_001211219.1_1656AbOC    MRFKKISCLLLSPLFIFSTSIYAGNTPKDQEIKKLVDQNFKPLLEKYDVPGMAVGVIQNN
ADC-73WP_001211219.1_2591AbOC    MRFKKISCLLLSPLFIFSTSIYAGNTPKDQEIKKLVDQNFKPLLEKYDVPGMAVGVIQNN
ADC-73WP_001211219.1_2602AbOC    MRFKKISCLLLSPLFIFSTSIYAGNTPKDQEIKKLVDQNFKPLLEKYDVPGMAVGVIQNN
ADC-73WP_001211219.1_910AbOC     MRFKKISCLLLSPLFIFSTSIYAGNTPKDQEIKKLVDQNFKPLLEKYDVPGMAVGVIQNN
ADC-73WP_001211219.1_394AbOC     MRFKKISCLLLSPLFIFSTSIYAGNTPKDQEIKKLVDQNFKPLLEKYDVPGMAVGVIQNN
ADC-73WP_001211219.1_1030AbOC    MRFKKISCLLLSPLFIFSTSIYAGNTPKDQEIKKLVDQNFKPLLEKYDVPGMAVGVIQNN
ADC-73WP_001211219.1_1023AbOC    MRFKKISCLLLSPLFIFSTSIYAGNTPKDQEIKKLVDQNFKPLLEKYDVPGMAVGVIQNN
ADC-73UYQ66995.1_1967AbOC        MRFKKISCLLLSPLFIFSTSIYAGNTPKDQEIKKLVDQNFKPLLEKYDVPGMAVGVIQNN
ADC-73WP_001211219.1_906AbBOC    MRFKKISCLLLSPLFIFSTSIYAGNTPKDQEIKKLVDQNFKPLLEKYDVPGMAVGVIQNN
DC-73WP_001211219.1_2649AbBOC    MRFKKISCLLLSPLFIFSTSIYAGNTPKDQEIKKLVDQNFKPLLEKYDVPGMAVGVIQNN
DC-73WP_001211219.1_2693AbBOC    MRFKKISCLLLSPLFIFSTSIYAGNTPKDQEIKKLVDQNFKPLLEKYDVPGMAVGVIQNN
ADC-73AQU57925.1_2703AbBOC       MRFKKISCLLLSPLFIFSTSIYAGNTPKDQEIKKLVDQNFKPLLEKYDVPGMAVGVIQNN
DC-73WP_001211219.1_1026AbBOC    MRFKKISCLLLSPLFIFSTSIYAGNTPKDQEIKKLVDQNFKPLLEKYDVPGMAVGVIQNN
DC-73WP_001211219.1_1019AbBOC    MRFKKISCLLLSPLFIFSTSIYAGNTPKDQEIKKLVDQNFKPLLEKYDVPGMAVGVIQNN
DC-73WP_001211219.1_1024AbBOC    MRFKKISCLLLSPLFIFSTSIYAGNTPKDQEIKKLVDQNFKPLLEKYDVPGMAVGVIQNN
ADC-73WP_001211219.1_930AbBOC    MRFKKISCLLLSPLFIFSTSIYAGNTPKDQEIKKLVDQNFKPLLEKYDVPGMAVGVIQNN
DC-73WP_001211219.1_2609AbBOC    MRFKKISCLLLSPLFIFSTSIYAGNTPKDQEIKKLVDQNFKPLLEKYDVPGMAVGVIQNN
DC-73WP_001211219.1_3456AbBOC    MRFKKISCLLLSPLFIFSTSIYAGNTPKDQEIKKLVDQNFKPLLEKYDVPGMAVGVIQNN
DC-73WP_001211219.1_2614AbBOC    MRFKKISCLLLSPLFIFSTSIYAGNTPKDQEIKKLVDQNFKPLLEKYDVPGMAVGVIQNN
DC-73WP_001211219.1_1025AbBOC    MRFKKISCLLLSPLFIFSTSIYAGNTPKDQEIKKLVDQNFKPLLEKYDVPGMAVGVIQNN
DC-73WP_001211219.1_1030AbBOC    MRFKKISCLLLSPLFIFSTSIYAGNTPKDQEIKKLVDQNFKPLLEKYDVPGMAVGVIQNN
DC-73WP_001211219.1_1173AbBOC    MRFKKISCLLLSPLFIFSTSIYAGNTPKDQEIKKLVDQNFKPLLEKYDVPGMAVGVIQNN
DC-73WP_001211219.1_1021AbBOC    MRFKKISCLLLSPLFIFSTSIYAGNTPKDQEIKKLVDQNFKPLLEKYDVPGMAVGVIQNN
DC-73WP_001211219.1_2449AbBOC    MRFKKISCLLLSPLFIFSTSIYAGNTPKDQEIKKLVDQNFKPLLEKYDVPGMAVGVIQNN
DC-73WP_001211219.1_1176AbBOC    MRFKKISCLLLSPLFIFSTSIYAGNTPKDQEIKKLVDQNFKPLLEKYDVPGMAVGVIQNN
DC-73WP_001211219.1_1161AbBOC    MRFKKISCLLLSPLFIFSTSIYAGNTPKDQEIKKLVDQNFKPLLEKYDVPGMAVGVIQNN
DC-73WP_001211219.1_1070AbBOC    MRFKKISCLLLSPLFIFSTSIYAGNTPKDQEIKKLVDQNFKPLLEKYDVPGMAVGVIQNN
DC-73WP_001211219.1_1079AbBOC    MRFKKISCLLLSPLFIFSTSIYAGNTPKDQEIKKLVDQNFKPLLEKYDVPGMAVGVIQNN
DC-73WP_001211219.1_1078AbBOC    MRFKKISCLLLSPLFIFSTSIYAGNTPKDQEIKKLVDQNFKPLLEKYDVPGMAVGVIQNN
DC-73WP_001211219.1_1037AbBOC    MRFKKISCLLLSPLFIFSTSIYAGNTPKDQEIKKLVDQNFKPLLEKYDVPGMAVGVIQNN
DC-73WP_001211219.1_1035AbBOC    MRFKKISCLLLSPLFIFSTSIYAGNTPKDQEIKKLVDQNFKPLLEKYDVPGMAVGVIQNN
DC-73WP_001211219.1_1033AbBOC    MRFKKISCLLLSPLFIFSTSIYAGNTPKDQEIKKLVDQNFKPLLEKYDVPGMAVGVIQNN
DC-73WP_001211219.1_1041AbBOC    MRFKKISCLLLSPLFIFSTSIYAGNTPKDQEIKKLVDQNFKPLLEKYDVPGMAVGVIQNN
DC-73WP_001211219.1_1023AbBOC    MRFKKISCLLLSPLFIFSTSIYAGNTPKDQEIKKLVDQNFKPLLEKYDVPGMAVGVIQNN
ADC-73USX69547.1_2386AbBOC       MRFKKISCLLLSPLFIFSTSIYAGNTPKDQEIKKLVDQNFKPLLEKYDVPGMAVGVIQNN
DC-73WP_001211219.1_1034AbBOC    MRFKKISCLLLSPLFIFSTSIYAGNTPKDQEIKKLVDQNFKPLLEKYDVPGMAVGVIQNN
ADC-73WP_001211219.1_852AbBOC    MRFKKISCLLLSPLFIFSTSIYAGNTPKDQEIKKLVDQNFKPLLEKYDVPGMAVGVIQNN
DC-73WP_001211219.1_3070AbBOC    MRFKKISCLLLSPLFIFSTSIYAGNTPKDQEIKKLVDQNFKPLLEKYDVPGMAVGVIQNN
ADC-73WBS60953.1_1026AbBOC       MRFKKISCLLLSPLFIFSTSIYAGNTPKDQEIKKLVDQNFKPLLEKYDVPGMAVGVIQNN
ADC-73WDC39660.1_1023AbBOC       MRFKKISCLLLSPLFIFSTSIYAGNTPKDQEIKKLVDQNFKPLLEKYDVPGMAVGVIQNN
ADC-73WDC73972.1_1415AbBOC       MRFKKISCLLLSPLFIFSTSIYAGNTPKDQEIKKLVDQNFKPLLEKYDVPGMAVGVIQNN
ADC-73WDC70172.1_1037AbBOC       MRFKKISCLLLSPLFIFSTSIYAGNTPKDQEIKKLVDQNFKPLLEKYDVPGMAVGVIQNN
DC-73WP_001211219.1_1101AbBOC    MRFKKISCLLLSPLFIFSTSIYAGNTPKDQEIKKLVDQNFKPLLEKYDVPGMAVGVIQNN
DC-73WP_001211219.1_1667AbBOC    MRFKKISCLLLSPLFIFSTSIYAGNTPKDQEIKKLVDQNFKPLLEKYDVPGMAVGVIQNN
ADC-73WP_001211219.1_287AbBOC    MRFKKISCLLLSPLFIFSTSIYAGNTPKDQEIKKLVDQNFKPLLEKYDVPGMAVGVIQNN
DC-73WP_001211219.1_3177AbBOC    MRFKKISCLLLSPLFIFSTSIYAGNTPKDQEIKKLVDQNFKPLLEKYDVPGMAVGVIQNN
DC-73WP_001211219.1_2501AbBOC    MRFKKISCLLLSPLFIFSTSIYAGNTPKDQEIKKLVDQNFKPLLEKYDVPGMAVGVIQNN
DC-73WP_001211219.1_2535AbBOC    MRFKKISCLLLSPLFIFSTSIYAGNTPKDQEIKKLVDQNFKPLLEKYDVPGMAVGVIQNN
DC-73WP_001211219.1_3150AbBOC    MRFKKISCLLLSPLFIFSTSIYAGNTPKDQEIKKLVDQNFKPLLEKYDVPGMAVGVIQNN
DC-73WP_001211219.1_3714AbBOC    MRFKKISCLLLSPLFIFSTSIYAGNTPKDQEIKKLVDQNFKPLLEKYDVPGMAVGVIQNN
ADC-73WP_001211219.1_722AbBOC    MRFKKISCLLLSPLFIFSTSIYAGNTPKDQEIKKLVDQNFKPLLEKYDVPGMAVGVIQNN
DC-73WP_001211219.1_3650AbBOC    MRFKKISCLLLSPLFIFSTSIYAGNTPKDQEIKKLVDQNFKPLLEKYDVPGMAVGVIQNN
ADC-73WP_001211219.1_568AbBOC    MRFKKISCLLLSPLFIFSTSIYAGNTPKDQEIKKLVDQNFKPLLEKYDVPGMAVGVIQNN
DC-73WP_001211219.1_3226AbBOC    MRFKKISCLLLSPLFIFSTSIYAGNTPKDQEIKKLVDQNFKPLLEKYDVPGMAVGVIQNN
ADC-73WP_001211219.1_556AbBOC    MRFKKISCLLLSPLFIFSTSIYAGNTPKDQEIKKLVDQNFKPLLEKYDVPGMAVGVIQNN
```

```
DC-73WP_001211219.1_2436AbBOC   MRFKKISCLLLSPLFIFSTSIYAGNTPKDQEIKKLVDQNFKPLLEKYDVPGMAVGVIQNN
DC-73WP_001211219.1_1029AbBOC   MRFKKISCLLLSPLFIFSTSIYAGNTPKDQEIKKLVDQNFKPLLEKYDVPGMAVGVIQNN
DC-73WP_001211219.1_1013AbBOC   MRFKKISCLLLSPLFIFSTSIYAGNTPKDQEIKKLVDQNFKPLLEKYDVPGMAVGVIQNN
ADC-73WP_001211219.1_906AbBCP   MRFKKISCLLLSPLFIFSTSIYAGNTPKDQEIKKLVDQNFKPLLEKYDVPGMAVGVIQNN
DC-73WP_001211219.1_2649AbBCP   MRFKKISCLLLSPLFIFSTSIYAGNTPKDQEIKKLVDQNFKPLLEKYDVPGMAVGVIQNN
DC-73WP_001211219.1_2693AbBCP   MRFKKISCLLLSPLFIFSTSIYAGNTPKDQEIKKLVDQNFKPLLEKYDVPGMAVGVIQNN
ADC-73AQU57925.1_2703AbBCP      MRFKKISCLLLSPLFIFSTSIYAGNTPKDQEIKKLVDQNFKPLLEKYDVPGMAVGVIQNN
DC-73WP_001211219.1_1026AbBCP   MRFKKISCLLLSPLFIFSTSIYAGNTPKDQEIKKLVDQNFKPLLEKYDVPGMAVGVIQNN
DC-73WP_001211219.1_1019AbBCP   MRFKKISCLLLSPLFIFSTSIYAGNTPKDQEIKKLVDQNFKPLLEKYDVPGMAVGVIQNN
DC-73WP_001211219.1_1024AbBCP   MRFKKISCLLLSPLFIFSTSIYAGNTPKDQEIKKLVDQNFKPLLEKYDVPGMAVGVIQNN
ADC-73WP_001211219.1_930AbBCP   MRFKKISCLLLSPLFIFSTSIYAGNTPKDQEIKKLVDQNFKPLLEKYDVPGMAVGVIQNN
DC-73WP_001211219.1_2609AbBCP   MRFKKISCLLLSPLFIFSTSIYAGNTPKDQEIKKLVDQNFKPLLEKYDVPGMAVGVIQNN
DC-73WP_001211219.1_3456AbBCP   MRFKKISCLLLSPLFIFSTSIYAGNTPKDQEIKKLVDQNFKPLLEKYDVPGMAVGVIQNN
DC-73WP_001211219.1_2614AbBCP   MRFKKISCLLLSPLFIFSTSIYAGNTPKDQEIKKLVDQNFKPLLEKYDVPGMAVGVIQNN
DC-73WP_001211219.1_1025AbBCP   MRFKKISCLLLSPLFIFSTSIYAGNTPKDQEIKKLVDQNFKPLLEKYDVPGMAVGVIQNN
DC-73WP_001211219.1_1030AbBCP   MRFKKISCLLLSPLFIFSTSIYAGNTPKDQEIKKLVDQNFKPLLEKYDVPGMAVGVIQNN
DC-73WP_001211219.1_1173AbBCP   MRFKKISCLLLSPLFIFSTSIYAGNTPKDQEIKKLVDQNFKPLLEKYDVPGMAVGVIQNN
DC-73WP_001211219.1_1021AbBCP   MRFKKISCLLLSPLFIFSTSIYAGNTPKDQEIKKLVDQNFKPLLEKYDVPGMAVGVIQNN
DC-73WP_001211219.1_2449AbBCP   MRFKKISCLLLSPLFIFSTSIYAGNTPKDQEIKKLVDQNFKPLLEKYDVPGMAVGVIQNN
DC-73WP_001211219.1_1176AbBCP   MRFKKISCLLLSPLFIFSTSIYAGNTPKDQEIKKLVDQNFKPLLEKYDVPGMAVGVIQNN
DC-73WP_001211219.1_1161AbBCP   MRFKKISCLLLSPLFIFSTSIYAGNTPKDQEIKKLVDQNFKPLLEKYDVPGMAVGVIQNN
DC-73WP_001211219.1_1070AbBCP   MRFKKISCLLLSPLFIFSTSIYAGNTPKDQEIKKLVDQNFKPLLEKYDVPGMAVGVIQNN
DC-73WP_001211219.1_1079AbBCP   MRFKKISCLLLSPLFIFSTSIYAGNTPKDQEIKKLVDQNFKPLLEKYDVPGMAVGVIQNN
DC-73WP_001211219.1_1078AbBCP   MRFKKISCLLLSPLFIFSTSIYAGNTPKDQEIKKLVDQNFKPLLEKYDVPGMAVGVIQNN
DC-73WP_001211219.1_1037AbBCP   MRFKKISCLLLSPLFIFSTSIYAGNTPKDQEIKKLVDQNFKPLLEKYDVPGMAVGVIQNN
DC-73WP_001211219.1_1035AbBCP   MRFKKISCLLLSPLFIFSTSIYAGNTPKDQEIKKLVDQNFKPLLEKYDVPGMAVGVIQNN
DC-73WP_001211219.1_1033AbBCP   MRFKKISCLLLSPLFIFSTSIYAGNTPKDQEIKKLVDQNFKPLLEKYDVPGMAVGVIQNN
DC-73WP_001211219.1_1041AbBCP   MRFKKISCLLLSPLFIFSTSIYAGNTPKDQEIKKLVDQNFKPLLEKYDVPGMAVGVIQNN
DC-73WP_001211219.1_1023AbBCP   MRFKKISCLLLSPLFIFSTSIYAGNTPKDQEIKKLVDQNFKPLLEKYDVPGMAVGVIQNN
ADC-73USX69547.1_2386AbBCP      MRFKKISCLLLSPLFIFSTSIYAGNTPKDQEIKKLVDQNFKPLLEKYDVPGMAVGVIQNN
DC-73WP_001211219.1_1034AbBCP   MRFKKISCLLLSPLFIFSTSIYAGNTPKDQEIKKLVDQNFKPLLEKYDVPGMAVGVIQNN
ADC-73WP_001211219.1_852AbBCP   MRFKKISCLLLSPLFIFSTSIYAGNTPKDQEIKKLVDQNFKPLLEKYDVPGMAVGVIQNN
DC-73WP_001211219.1_3070AbBCP   MRFKKISCLLLSPLFIFSTSIYAGNTPKDQEIKKLVDQNFKPLLEKYDVPGMAVGVIQNN
ADC-73WBS60953.1_1026AbBCP      MRFKKISCLLLSPLFIFSTSIYAGNTPKDQEIKKLVDQNFKPLLEKYDVPGMAVGVIQNN
ADC-73WDC39660.1_1023AbBCP      MRFKKISCLLLSPLFIFSTSIYAGNTPKDQEIKKLVDQNFKPLLEKYDVPGMAVGVIQNN
ADC-73WDC73972.1_1415AbBCP      MRFKKISCLLLSPLFIFSTSIYAGNTPKDQEIKKLVDQNFKPLLEKYDVPGMAVGVIQNN
ADC-73WDC70172.1_1037AbBCP      MRFKKISCLLLSPLFIFSTSIYAGNTPKDQEIKKLVDQNFKPLLEKYDVPGMAVGVIQNN
DC-73WP_001211219.1_1101AbBCP   MRFKKISCLLLSPLFIFSTSIYAGNTPKDQEIKKLVDQNFKPLLEKYDVPGMAVGVIQNN
DC-73WP_001211219.1_1667AbBCP   MRFKKISCLLLSPLFIFSTSIYAGNTPKDQEIKKLVDQNFKPLLEKYDVPGMAVGVIQNN
ADC-73WP_001211219.1_287AbBCP   MRFKKISCLLLSPLFIFSTSIYAGNTPKDQEIKKLVDQNFKPLLEKYDVPGMAVGVIQNN
DC-73WP_001211219.1_3177AbBCP   MRFKKISCLLLSPLFIFSTSIYAGNTPKDQEIKKLVDQNFKPLLEKYDVPGMAVGVIQNN
DC-73WP_001211219.1_2501AbBCP   MRFKKISCLLLSPLFIFSTSIYAGNTPKDQEIKKLVDQNFKPLLEKYDVPGMAVGVIQNN
DC-73WP_001211219.1_2535AbBCP   MRFKKISCLLLSPLFIFSTSIYAGNTPKDQEIKKLVDQNFKPLLEKYDVPGMAVGVIQNN
DC-73WP_001211219.1_3150AbBCP   MRFKKISCLLLSPLFIFSTSIYAGNTPKDQEIKKLVDQNFKPLLEKYDVPGMAVGVIQNN
DC-73WP_001211219.1_3714AbBCP   MRFKKISCLLLSPLFIFSTSIYAGNTPKDQEIKKLVDQNFKPLLEKYDVPGMAVGVIQNN
ADC-73WP_001211219.1_722AbBCP   MRFKKISCLLLSPLFIFSTSIYAGNTPKDQEIKKLVDQNFKPLLEKYDVPGMAVGVIQNN
DC-73WP_001211219.1_3650AbBCP   MRFKKISCLLLSPLFIFSTSIYAGNTPKDQEIKKLVDQNFKPLLEKYDVPGMAVGVIQNN
ADC-73WP_001211219.1_568AbBCP   MRFKKISCLLLSPLFIFSTSIYAGNTPKDQEIKKLVDQNFKPLLEKYDVPGMAVGVIQNN
DC-73WP_001211219.1_3226AbBCP   MRFKKISCLLLSPLFIFSTSIYAGNTPKDQEIKKLVDQNFKPLLEKYDVPGMAVGVIQNN
ADC-73WP_001211219.1_556AbBCP   MRFKKISCLLLSPLFIFSTSIYAGNTPKDQEIKKLVDQNFKPLLEKYDVPGMAVGVIQNN
DC-73WP_001211219.1_2436AbBCP   MRFKKISCLLLSPLFIFSTSIYAGNTPKDQEIKKLVDQNFKPLLEKYDVPGMAVGVIQNN
DC-73WP_001211219.1_1029AbBCP   MRFKKISCLLLSPLFIFSTSIYAGNTPKDQEIKKLVDQNFKPLLEKYDVPGMAVGVIQNN
DC-73WP_001211219.1_1013AbBCP   MRFKKISCLLLSPLFIFSTSIYAGNTPKDQEIKKLVDQNFKPLLEKYDVPGMAVGVIQNN
ADC-73BDE18748.1_1038AbBCP      MRFKKISCLLLSPLFIFSTSIYAGNTPKDQEIKKLVDQNFKPLLEKYDVPGMAVGVIQNN
DC-115WP_001211215.1_2833AbOC   MRFKKISCLLLSPLFIFSTSIYAGNTPKDQEIKKLVDQNFKPLLEKYDVPGMAVGVIQNN
C-115WP_001211215.1_1156AbBOC   MRFKKISCLLLSPLFIFSTSIYAGNTPKDQEIKKLVDQNFKPLLEKYDVPGMAVGVIQNN
C-115WP_001211215.1_1156AbBCP   MRFKKISCLLLSPLFIFSTSIYAGNTPKDQEIKKLVDQNFKPLLEKYDVPGMAVGVIQNN
ADC-5WP_038405930.1_2542AbOC    MRFKKISCLLLSPLFIFSTSIYAGNTPKDQEIKKLVDQNFKPLLEKYDVPGMAVGVIQNN
ADC-5WP_038405930.1_1113AbBOC   MRFKKISCLLLSPLFIFSTSIYAGNTPKDQEIKKLVDQNFKPLLEKYDVPGMAVGVIQNN
ADC-5WP_038405930.1_1113AbBCP   MRFKKISCLLLSPLFIFSTSIYAGNTPKDQEIKKLVDQNFKPLLEKYDVPGMAVGVIQNN
C-236WP_176673601.1_1026AbBOC   MRFKKISCLLLSPLFIFSTSIYAGNTPKDQEIKKLVDQNFKPLLEKYDVPGMAVGVIQNN
C-236WP_176673601.1_1026AbBCP   MRFKKISCLLLSPLFIFSTSIYAGNTPKDQEIKKLVDQNFKPLLEKYDVPGMAVGVIQNN
DC-217WP_060470109.1_3584AbOC   MRFKKISCLLLSPLFIFSTSIYAGNTPKDQEIKKLVDQNFKPLLEKYDVPGMAVGVIQNN
ADC-217USX66614.1_2290AbBOC     MRFKKISCLLLSPLFIFSTSIYAGNTPKDQEIKKLVDQNFKPLLEKYDVPGMAVGVIQNN
ADC-217USX66614.1_2290AbBCP     MRFKKISCLLLSPLFIFSTSIYAGNTPKDQEIKKLVDQNFKPLLEKYDVPGMAVGVIQNN
ADC-247WP_032058755.1_382AbOC   MRFKKISCLLLSPLFIFSTSIYAGNTPKDQEIKKLVDQNFKPLLEKYDVPGMAVGVIQNN
C-247WP_032058755.1_1936AbBOC   MRFKKISCLLLSPLFIFSTSIYAGNTPKDQEIKKLVDQNFKPLLEKYDVPGMAVGVIQNN
C-247WP_032058755.1_1936AbBCP   MRFKKISCLLLSPLFIFSTSIYAGNTPKDQEIKKLVDQNFKPLLEKYDVPGMAVGVIQNN
DC-203WP_032000414.1_2940AbOC   MRFKKISCLLLSPLFIFSTSIYAGNTPKDQEIKKLVDQNFKPLLEKYDVPGMAVGVIQNN
C-203WP_032000414.1_1062AbBOC   MRFKKISCLLLSPLFIFSTSIYAGNTPKDQEIKKLVDQNFKPLLEKYDVPGMAVGVIQNN
DC-203WP_032000414.1_129AbBOC   MRFKKISCLLLSPLFIFSTSIYAGNTPKDQEIKKLVDQNFKPLLEKYDVPGMAVGVIQNN
C-203WP_032000414.1_2518AbBOC   MRFKKISCLLLSPLFIFSTSIYAGNTPKDQEIKKLVDQNFKPLLEKYDVPGMAVGVIQNN
C-203WP_032000414.1_1062AbBCP   MRFKKISCLLLSPLFIFSTSIYAGNTPKDQEIKKLVDQNFKPLLEKYDVPGMAVGVIQNN
DC-203WP_032000414.1_129AbBCP   MRFKKISCLLLSPLFIFSTSIYAGNTPKDQEIKKLVDQNFKPLLEKYDVPGMAVGVIQNN
C-203WP_032000414.1_2518AbBCP   MRFKKISCLLLSPLFIFSTSIYAGNTPKDQEIKKLVDQNFKPLLEKYDVPGMAVGVIQNN
DC-152WP_001211233.1_3734AbOC   MRFKKISCLLLSPLFIFSTSIYAGNTPKDQEIKKLVDQNFKPLLEKYDVPGMAVGVIQNN
C-152WP_001211233.1_1080AbBOC   MRFKKISCLLLSPLFIFSTSIYAGNTPKDQEIKKLVDQNFKPLLEKYDVPGMAVGVIQNN
DC-152WP_001211233.1_491AbBOC   MRFKKISCLLLSPLFIFSTSIYAGNTPKDQEIKKLVDQNFKPLLEKYDVPGMAVGVIQNN
ADC-152WP_001211233.1_46AbBOC   MRFKKISCLLLSPLFIFSTSIYAGNTPKDQEIKKLVDQNFKPLLEKYDVPGMAVGVIQNN
C-152WP_001211233.1_1545AbBOC   MRFKKISCLLLSPLFIFSTSIYAGNTPKDQEIKKLVDQNFKPLLEKYDVPGMAVGVIQNN
ADC-152WBS86980.1_1289AbBOC     MRFKKISCLLLSPLFIFSTSIYAGNTPKDQEIKKLVDQNFKPLLEKYDVPGMAVGVIQNN
ADC-152WBS81072.1_2581AbBOC     MRFKKISCLLLSPLFIFSTSIYAGNTPKDQEIKKLVDQNFKPLLEKYDVPGMAVGVIQNN
ADC-152WBS88504.1_2711AbBOC     MRFKKISCLLLSPLFIFSTSIYAGNTPKDQEIKKLVDQNFKPLLEKYDVPGMAVGVIQNN
ADC-152WBS92168.1_2401AbBOC     MRFKKISCLLLSPLFIFSTSIYAGNTPKDQEIKKLVDQNFKPLLEKYDVPGMAVGVIQNN
ADC-152WBS79617.1_1289AbBOC     MRFKKISCLLLSPLFIFSTSIYAGNTPKDQEIKKLVDQNFKPLLEKYDVPGMAVGVIQNN
C-152WP_001211233.1_1080AbBCP   MRFKKISCLLLSPLFIFSTSIYAGNTPKDQEIKKLVDQNFKPLLEKYDVPGMAVGVIQNN
DC-152WP_001211233.1_491AbBCP   MRFKKISCLLLSPLFIFSTSIYAGNTPKDQEIKKLVDQNFKPLLEKYDVPGMAVGVIQNN
ADC-152WP_001211233.1_46AbBCP   MRFKKISCLLLSPLFIFSTSIYAGNTPKDQEIKKLVDQNFKPLLEKYDVPGMAVGVIQNN
C-152WP_001211233.1_1545AbBCP   MRFKKISCLLLSPLFIFSTSIYAGNTPKDQEIKKLVDQNFKPLLEKYDVPGMAVGVIQNN
ADC-152WBS86980.1_1289AbBCP     MRFKKISCLLLSPLFIFSTSIYAGNTPKDQEIKKLVDQNFKPLLEKYDVPGMAVGVIQNN
ADC-152WBS81072.1_2581AbBCP     MRFKKISCLLLSPLFIFSTSIYAGNTPKDQEIKKLVDQNFKPLLEKYDVPGMAVGVIQNN
ADC-152WBS88504.1_2711AbBCP     MRFKKISCLLLSPLFIFSTSIYAGNTPKDQEIKKLVDQNFKPLLEKYDVPGMAVGVIQNN
ADC-152WBS92168.1_2401AbBCP     MRFKKISCLLLSPLFIFSTSIYAGNTPKDQEIKKLVDQNFKPLLEKYDVPGMAVGVIQNN
ADC-152WBS79617.1_1289AbBCP     MRFKKISCLLLSPLFIFSTSIYAGNTPKDQEIKKLVDQNFKPLLEKYDVPGMAVGVIQNN
ADC-52WP_001211232.1_990AbOC    MRFKKISCLLLSPLFIFSTSIYAGNTPKDQEIKKLVDQNFKPLLEKYDVPGMAVGVIQNN
DC-52WP_001211232.1_1087AbBOC   MRFKKISCLLLSPLFIFSTSIYAGNTPKDQEIKKLVDQNFKPLLEKYDVPGMAVGVIQNN
DC-52WP_001211232.1_1087AbBCP   MRFKKISCLLLSPLFIFSTSIYAGNTPKDQEIKKLVDQNFKPLLEKYDVPGMAVGVIQNN
C-163WP_057093064.1_2397AbBOC   MRFKKISCLLLSPLFIFSTSIYAGNTPKDQEIKKLVDQNFKPLLEKYDVPGMAVGVIQNN
C-163WP_057093064.1_2397AbBCP   MRFKKISCLLLSPLFIFSTSIYAGNTPKDQEIKKLVDQNFKPLLEKYDVPGMAVGVIQNN
ADC-33WP_001211220.1_2277AbOC   MRFKKISCLLLSPLFIFSTSIYAGNTPKDQEIKKLVDQNFKPLLEKYDVPGMAVGVIQNN
DC-33WP_001211220.1_1044AbBOC   MRFKKISCLLLSPLFIFSTSIYAGNTPKDQEIKKLVDQNFKPLLEKYDVPGMAVGVIQNN
DC-33WP_001211220.1_2452AbBOC   MRFKKISCLLLSPLFIFSTSIYAGNTPKDQEIKKLVDQNFKPLLEKYDVPGMAVGVIQNN
DC-33WP_001211220.1_1046AbBOC   MRFKKISCLLLSPLFIFSTSIYAGNTPKDQEIKKLVDQNFKPLLEKYDVPGMAVGVIQNN
DC-33WP_001211220.1_1025AbBOC   MRFKKISCLLLSPLFIFSTSIYAGNTPKDQEIKKLVDQNFKPLLEKYDVPGMAVGVIQNN
DC-33WP_001211220.1_1026AbBOC   MRFKKISCLLLSPLFIFSTSIYAGNTPKDQEIKKLVDQNFKPLLEKYDVPGMAVGVIQNN
DC-33WP_001211220.1_1033AbBOC   MRFKKISCLLLSPLFIFSTSIYAGNTPKDQEIKKLVDQNFKPLLEKYDVPGMAVGVIQNN
DC-33WP_001211220.1_1035AbBOC   MRFKKISCLLLSPLFIFSTSIYAGNTPKDQEIKKLVDQNFKPLLEKYDVPGMAVGVIQNN
DC-33WP_001211220.1_1041AbBOC   MRFKKISCLLLSPLFIFSTSIYAGNTPKDQEIKKLVDQNFKPLLEKYDVPGMAVGVIQNN
DC-33WP_001211220.1_1044AbBCP   MRFKKISCLLLSPLFIFSTSIYAGNTPKDQEIKKLVDQNFKPLLEKYDVPGMAVGVIQNN
DC-33WP_001211220.1_2452AbBCP   MRFKKISCLLLSPLFIFSTSIYAGNTPKDQEIKKLVDQNFKPLLEKYDVPGMAVGVIQNN
DC-33WP_001211220.1_1046AbBCP   MRFKKISCLLLSPLFIFSTSIYAGNTPKDQEIKKLVDQNFKPLLEKYDVPGMAVGVIQNN
DC-33WP_001211220.1_1025AbBCP   MRFKKISCLLLSPLFIFSTSIYAGNTPKDQEIKKLVDQNFKPLLEKYDVPGMAVGVIQNN
DC-33WP_001211220.1_1026AbBCP   MRFKKISCLLLSPLFIFSTSIYAGNTPKDQEIKKLVDQNFKPLLEKYDVPGMAVGVIQNN
DC-33WP_001211220.1_1033AbBCP   MRFKKISCLLLSPLFIFSTSIYAGNTPKDQEIKKLVDQNFKPLLEKYDVPGMAVGVIQNN
DC-33WP_001211220.1_1035AbBCP   MRFKKISCLLLSPLFIFSTSIYAGNTPKDQEIKKLVDQNFKPLLEKYDVPGMAVGVIQNN
DC-33WP_001211220.1_1041AbBCP   MRFKKISCLLLSPLFIFSTSIYAGNTPKDQEIKKLVDQNFKPLLEKYDVPGMAVGVIQNN
C-212WP_031975357.1_1297AbBOC   MRFKKISCLLLSPLFIFSTSIYAGNTPKDQEIKKLVDQNFKPLLEKYDVPGMAVGVIQNN
DC-212WP_031975357.1_501AbBOC   MRFKKISCLLLSPLFIFSTSIYAGNTPKDQEIKKLVDQNFKPLLEKYDVPGMAVGVIQNN
C-212WP_031975357.1_1297AbBCP   MRFKKISCLLLSPLFIFSTSIYAGNTPKDQEIKKLVDQNFKPLLEKYDVPGMAVGVIQNN
DC-212WP_031975357.1_501AbBCP   MRFKKISCLLLSPLFIFSTSIYAGNTPKDQEIKKLVDQNFKPLLEKYDVPGMAVGVIQNN
C-182WP_057691114.1_1055AbBOC   MRFKKISCLLLSPLFIFSTSIYAGNTPKDQEIKKLVDQNFKPLLEKYDVPGMAVGVIQNN
C-182WP_057691114.1_1055AbBCP   MRFKKISCLLLSPLFIFSTSIYAGNTPKDQEIKKLVDQNFKPLLEKYDVPGMAVGVIQNN
DC-81WP_059262723.1_1208AbBOC   MRFKKISCLLLSPLFIFSTSIYAGNTPKDQEIKKLVDQNFKPLLEKYDVPGMAVGVIQNN
DC-81WP_059262723.1_2841AbBOC   MRFKKISCLLLSPLFIFSTSIYAGNTPKDQEIKKLVDQNFKPLLEKYDVPGMAVGVIQNN
DC-81WP_059262723.1_1208AbBCP   MRFKKISCLLLSPLFIFSTSIYAGNTPKDQEIKKLVDQNFKPLLEKYDVPGMAVGVIQNN
DC-81WP_059262723.1_2841AbBCP   MRFKKISCLLLSPLFIFSTSIYAGNTPKDQEIKKLVDQNFKPLLEKYDVPGMAVGVIQNN
ADC-176WP_001159761.1_11AbOC    MQFKKISCLLLSPLFIFSTSIYADNTPKDQEIKKLVDQNFKPLLEKYDVPGMAVGVIQNN
DC-176WP_001159761.1_3466AbOC   MQFKKISCLLLSPLFIFSTSIYADNTPKDQEIKKLVDQNFKPLLEKYDVPGMAVGVIQNN
ADC-176WP_001159761.1_93AbBOP   MQFKKISCLLLSPLFIFSTSIYADNTPKDQEIKKLVDQNFKPLLEKYDVPGMAVGVIQNN
DC-176WP_001159761.1_247AbBOC   MQFKKISCLLLSPLFIFSTSIYADNTPKDQEIKKLVDQNFKPLLEKYDVPGMAVGVIQNN
ADC-176WP_001159761.1_9AbBOC    MQFKKISCLLLSPLFIFSTSIYADNTPKDQEIKKLVDQNFKPLLEKYDVPGMAVGVIQNN
C-176WP_001159761.1_2982AbBOC   MQFKKISCLLLSPLFIFSTSIYADNTPKDQEIKKLVDQNFKPLLEKYDVPGMAVGVIQNN
ADC-176QLG80706.1_1971AbBOC     MQFKKISCLLLSPLFIFSTSIYADNTPKDQEIKKLVDQNFKPLLEKYDVPGMAVGVIQNN
C-176WP_001159761.1_2851AbBOC   MQFKKISCLLLSPLFIFSTSIYADNTPKDQEIKKLVDQNFKPLLEKYDVPGMAVGVIQNN
C-176WP_001159761.1_1220AbBOC   MQFKKISCLLLSPLFIFSTSIYADNTPKDQEIKKLVDQNFKPLLEKYDVPGMAVGVIQNN
DC-176WP_001159761.1_386AbBOC   MQFKKISCLLLSPLFIFSTSIYADNTPKDQEIKKLVDQNFKPLLEKYDVPGMAVGVIQNN
ADC-176WP_001159761.1_11AbBOC   MQFKKISCLLLSPLFIFSTSIYADNTPKDQEIKKLVDQNFKPLLEKYDVPGMAVGVIQNN
DC-176WP_001159761.1_247AbBCP   MQFKKISCLLLSPLFIFSTSIYADNTPKDQEIKKLVDQNFKPLLEKYDVPGMAVGVIQNN
ADC-176WP_001159761.1_9AbBCP    MQFKKISCLLLSPLFIFSTSIYADNTPKDQEIKKLVDQNFKPLLEKYDVPGMAVGVIQNN
C-176WP_001159761.1_2982AbBCP   MQFKKISCLLLSPLFIFSTSIYADNTPKDQEIKKLVDQNFKPLLEKYDVPGMAVGVIQNN
ADC-176QLG80706.1_1971AbBCP     MQFKKISCLLLSPLFIFSTSIYADNTPKDQEIKKLVDQNFKPLLEKYDVPGMAVGVIQNN
C-176WP_001159761.1_2851AbBCP   MQFKKISCLLLSPLFIFSTSIYADNTPKDQEIKKLVDQNFKPLLEKYDVPGMAVGVIQNN
C-176WP_001159761.1_1220AbBCP   MQFKKISCLLLSPLFIFSTSIYADNTPKDQEIKKLVDQNFKPLLEKYDVPGMAVGVIQNN
DC-176WP_001159761.1_386AbBCP   MQFKKISCLLLSPLFIFSTSIYADNTPKDQEIKKLVDQNFKPLLEKYDVPGMAVGVIQNN
ADC-176WP_001159761.1_11AbBCP   MQFKKISCLLLSPLFIFSTSIYADNTPKDQEIKKLVDQNFKPLLEKYDVPGMAVGVIQNN
ADC-199WP_114166683.1_169AbOC   MQFKKISCLLLSPLFIFSTSIYADNTPKDQEIKKLVDQNFKPLLEKYDVPGMAVGVIQNN
DC-199WP_114166683.1_267AbBOC   MQFKKISCLLLSPLFIFSTSIYADNTPKDQEIKKLVDQNFKPLLEKYDVPGMAVGVIQNN
DC-199WP_114166683.1_267AbBCP   MQFKKISCLLLSPLFIFSTSIYADNTPKDQEIKKLVDQNFKPLLEKYDVPGMAVGVIQNN
                           acc
```

```
                                      β2              β3    β4  η1        α3                  η2   η3
8FQV_1|Chains                                            TT                              TT
                                      40        50        60        70        80        90
8FQV_1|Chains                         KKYEMYYGLQSVQDKKAVNSSTIFELGSVSKLFTATAGGYAKNKGKISFDDTPGKYWKEL
-154OCUAc18_RS13275_2608AbBOC         KKYEMYYGLQSVQDKKAVNSSTIFELGSVSKLFTATAGGYAKNKGKISFDDTPGKYWKVL
-154OCUAc18_RS13275_2608AbBCP         KKYEMYYGLQSVQDKKAVNSSTIFELGSVSKLFTATAGGYAKNKGKISFDDTPGKYWKVL
ADC-73BDE22771.1_1271AbBCP            KKYEMYYGLQSVQDKKAVNSSTIFELGSVSKLFTATAGGYAKNKGKISFDDTPGKYWKEL
DC-87WP_068981619.1_1049AbBOC         KKYEMYYGLQSVQDKKAVNSSTIFELGSVSKLFTATAGGYAKNKGKISFDDTPGKYWKEL
DC-87WP_068981619.1_1049AbBCP         KKYEMYYGLQSVQDKKAVNSSTIFELGSVSKLFTATAGGYAKNKGKISFDDTPGKYWKEL
C-249WP_001211200.1_1039AbBOC         KKYEMYYGLQSVQDKKAVNSSTIFELGSVSKLFTATAGGYAKNKGKISFDDTPGKYWKEL
C-249WP_001211200.1_1039AbBCP         KKYEMYYGLQSVQDKKAVNSSTIFELGSVSKLFTATAGGYAKNKGKISFDDTPGKYWKEL
DC-32WP_004739487.1_2518AbBOC         KKYEMYYGLQSVQDKKAVNSSTIFELGSVSKLFTATAGGYAKNKGKISFDDTPGKYWKEL
DC-32WP_004739487.1_3158AbBOC         KKYEMYYGLQSVQDKKAVNSSTIFELGSVSKLFTATAGGYAKNKGKISFDDTPGKYWKEL
DC-32WP_004739487.1_2392AbBOC         KKYEMYYGLQSVQDKKAVNSSTIFELGSVSKLFTATAGGYAKNKGKISFDDTPGKYWKEL
DC-32WP_004739487.1_2378AbBOC         KKYEMYYGLQSVQDKKAVNSSTIFELGSVSKLFTATAGGYAKNKGKISFDDTPGKYWKEL
DC-32WP_004739487.1_2518AbBCP         KKYEMYYGLQSVQDKKAVNSSTIFELGSVSKLFTATAGGYAKNKGKISFDDTPGKYWKEL
DC-32WP_004739487.1_3158AbBCP         KKYEMYYGLQSVQDKKAVNSSTIFELGSVSKLFTATAGGYAKNKGKISFDDTPGKYWKEL
DC-32WP_004739487.1_2392AbBCP         KKYEMYYGLQSVQDKKAVNSSTIFELGSVSKLFTATAGGYAKNKGKISFDDTPGKYWKEL
DC-32WP_004739487.1_2378AbBCP         KKYEMYYGLQSVQDKKAVNSSTIFELGSVSKLFTATAGGYAKNKGKISFDDTPGKYWKEL
C-248WP_021511005.1_1080AbBOC         KKYEMYYGLQSVQDKKAVNSSTIFELGSVSKLFTATAGGYAKNKGKISFKDTPGKYWKEL
C-248WP_021511005.1_1080AbBCP         KKYEMYYGLQSVQDKKAVNSSTIFELGSVSKLFTATAGGYAKNKGKISFKDTPGKYWKEL
ADC-191UIY52419.1_2933AbOC            KKYEMYYGLQSVQDKKAVNSSTIFELGSVSKLFTATAGGYAKNKGKISFDDTPGKYWKVL
DC-191WP_003112187.1_3072AbOC         KKYEMYYGLQSVQDKKAVNSSTIFELGSVSKLFTATAGGYAKNKGKISFDDTPGKYWKVL
DC-191WP_003112187.1_2848AbOC         KKYEMYYGLQSVQDKKAVNSSTIFELGSVSKLFTATAGGYAKNKGKISFDDTPGKYWKVL
C-191WP_003112187.1_2694AbBOC         KKYEMYYGLQSVQDKKAVNSSTIFELGSVSKLFTATAGGYAKNKGKISFDDTPGKYWKVL
C-191WP_003112187.1_2694AbBCP         KKYEMYYGLQSVQDKKAVNSSTIFELGSVSKLFTATAGGYAKNKGKISFDDTPGKYWKVL
C-169WP_001211212.1_2709AbBOC         KKYEMYYGLQSVQDKKAVNSSTIFELGSVSKLFTATAGGYAKNKGKISFDDRPGKYWKEL
C-169WP_001211212.1_2457AbBOC         KKYEMYYGLQSVQDKKAVNSSTIFELGSVSKLFTATAGGYAKNKGKISFDDRPGKYWKEL
ADC-169UYQ18775.1_1073AbBOC           KKYEMYYGLQSVQDKKAVNSSTIFELGSVSKLFTATAGGYAKNKGKISFDDRPGKYWKEL
ADC-169UYQ29710.1_1071AbBOC           KKYEMYYGLQSVQDKKAVNSSTIFELGSVSKLFTATAGGYAKNKGKISFDDRPGKYWKEL
ADC-169UYQ59091.1_1072AbBOC           KKYEMYYGLQSVQDKKAVNSSTIFELGSVSKLFTATAGGYAKNKGKISFDDRPGKYWKEL
ADC-169WGT82884.1_1015AbBOC           KKYEMYYGLQSVQDKKAVNSSTIFELGSVSKLFTATAGGYAKNKGKISFDDRPGKYWKEL
C-169WP_001211212.1_2709AbBCP         KKYEMYYGLQSVQDKKAVNSSTIFELGSVSKLFTATAGGYAKNKGKISFDDRPGKYWKEL
C-169WP_001211212.1_2457AbBCP         KKYEMYYGLQSVQDKKAVNSSTIFELGSVSKLFTATAGGYAKNKGKISFDDRPGKYWKEL
ADC-169UYQ18775.1_1073AbBCP           KKYEMYYGLQSVQDKKAVNSSTIFELGSVSKLFTATAGGYAKNKGKISFDDRPGKYWKEL
ADC-169UYQ29710.1_1071AbBCP           KKYEMYYGLQSVQDKKAVNSSTIFELGSVSKLFTATAGGYAKNKGKISFDDRPGKYWKEL
ADC-169UYQ59091.1_1072AbBCP           KKYEMYYGLQSVQDKKAVNSSTIFELGSVSKLFTATAGGYAKNKGKISFDDRPGKYWKEL
ADC-169WGT82884.1_1015AbBCP           KKYEMYYGLQSVQDKKAVNSSTIFELGSVSKLFTATAGGYAKNKGKISFDDRPGKYWKEL
DC-99WP_068981630.1_2482AbBOC         KKYEMYYGLQSVQDKKAVNSSTIFELGSVSKLFTATAGGYAKNKGKISFDDKPGKYWKEL
DC-99WP_068981630.1_2482AbBCP         KKYEMYYGLQSVQDKKAVNSSTIFELGSVSKLFTATAGGYAKNKGKISFDDKPGKYWKEL
DC-222WP_031960999.1_1504AbOC         KKYEMYYGLQSVQDKKAVNSSTIFELGSVSKLFTATAGGYAKNKGKISFDDTPGKYWKEL
ADC-222UYP92921.1_1090AbBOC           KKYEMYYGLQSVQDKKAVNSSTIFELGSVSKLFTATAGGYAKNKGKISFDDTPGKYWKEL
ADC-222UYQ05421.1_2617AbBOC           KKYEMYYGLQSVQDKKAVNSSTIFELGSVSKLFTATAGGYAKNKGKISFDDTPGKYWKEL
ADC-222UYQ22523.1_1090AbBOC           KKYEMYYGLQSVQDKKAVNSSTIFELGSVSKLFTATAGGYAKNKGKISFDDTPGKYWKEL
ADC-222UYQ26181.1_1089AbBOC           KKYEMYYGLQSVQDKKAVNSSTIFELGSVSKLFTATAGGYAKNKGKISFDDTPGKYWKEL
ADC-222UYQ33449.1_1092AbBOC           KKYEMYYGLQSVQDKKAVNSSTIFELGSVSKLFTATAGGYAKNKGKISFDDTPGKYWKEL
ADC-222UYQ42503.1_2616AbBOC           KKYEMYYGLQSVQDKKAVNSSTIFELGSVSKLFTATAGGYAKNKGKISFDDTPGKYWKEL
ADC-222UYQ37112.1_1090AbBOC           KKYEMYYGLQSVQDKKAVNSSTIFELGSVSKLFTATAGGYAKNKGKISFDDTPGKYWKEL
ADC-222UYQ46167.1_2615AbBOC           KKYEMYYGLQSVQDKKAVNSSTIFELGSVSKLFTATAGGYAKNKGKISFDDTPGKYWKEL
ADC-222UYQ51934.1_1091AbBOC           KKYEMYYGLQSVQDKKAVNSSTIFELGSVSKLFTATAGGYAKNKGKISFDDTPGKYWKEL
ADC-222UYQ55558.1_1091AbBOC           KKYEMYYGLQSVQDKKAVNSSTIFELGSVSKLFTATAGGYAKNKGKISFDDTPGKYWKEL
ADC-222WBS68552.1_1096AbBOC           KKYEMYYGLQSVQDKKAVNSSTIFELGSVSKLFTATAGGYAKNKGKISFDDTPGKYWKEL
ADC-222WBS55174.1_2602AbBOC           KKYEMYYGLQSVQDKKAVNSSTIFELGSVSKLFTATAGGYAKNKGKISFDDTPGKYWKEL
ADC-222WBS51522.1_2600AbBOC           KKYEMYYGLQSVQDKKAVNSSTIFELGSVSKLFTATAGGYAKNKGKISFDDTPGKYWKEL
ADC-222UYP92921.1_1090AbBCP           KKYEMYYGLQSVQDKKAVNSSTIFELGSVSKLFTATAGGYAKNKGKISFDDTPGKYWKEL
ADC-222UYQ05421.1_2617AbBCP           KKYEMYYGLQSVQDKKAVNSSTIFELGSVSKLFTATAGGYAKNKGKISFDDTPGKYWKEL
ADC-222UYQ22523.1_1090AbBCP           KKYEMYYGLQSVQDKKAVNSSTIFELGSVSKLFTATAGGYAKNKGKISFDDTPGKYWKEL
ADC-222UYQ26181.1_1089AbBCP           KKYEMYYGLQSVQDKKAVNSSTIFELGSVSKLFTATAGGYAKNKGKISFDDTPGKYWKEL
ADC-222UYQ33449.1_1092AbBCP           KKYEMYYGLQSVQDKKAVNSSTIFELGSVSKLFTATAGGYAKNKGKISFDDTPGKYWKEL
ADC-222UYQ42503.1_2616AbBCP           KKYEMYYGLQSVQDKKAVNSSTIFELGSVSKLFTATAGGYAKNKGKISFDDTPGKYWKEL
ADC-222UYQ37112.1_1090AbBCP           KKYEMYYGLQSVQDKKAVNSSTIFELGSVSKLFTATAGGYAKNKGKISFDDTPGKYWKEL
ADC-222UYQ46167.1_2615AbBCP           KKYEMYYGLQSVQDKKAVNSSTIFELGSVSKLFTATAGGYAKNKGKISFDDTPGKYWKEL
ADC-222UYQ51934.1_1091AbBCP           KKYEMYYGLQSVQDKKAVNSSTIFELGSVSKLFTATAGGYAKNKGKISFDDTPGKYWKEL
ADC-222UYQ55558.1_1091AbBCP           KKYEMYYGLQSVQDKKAVNSSTIFELGSVSKLFTATAGGYAKNKGKISFDDTPGKYWKEL
ADC-222WBS68552.1_1096AbBCP           KKYEMYYGLQSVQDKKAVNSSTIFELGSVSKLFTATAGGYAKNKGKISFDDTPGKYWKEL
ADC-222WBS55174.1_2602AbBCP           KKYEMYYGLQSVQDKKAVNSSTIFELGSVSKLFTATAGGYAKNKGKISFDDTPGKYWKEL
ADC-222WBS51522.1_2600AbBCP           KKYEMYYGLQSVQDKKAVNSSTIFELGSVSKLFTATAGGYAKNKGKISFDDTPGKYWKEL
ADC-26WP_001211238.1_141AbOC          KKYEMYYGLQSVQDKKAVNSSTIFELGSVSKLFTATAGGYAKNKGKISFDDTPGKYWKVL
ADC-26WP_001211238.1_142AbOC          KKYEMYYGLQSVQDKKAVNSSTIFELGSVSKLFTATAGGYAKNKGKISFDDTPGKYWKVL
ADC-26WP_001211238.1_1AbOC            KKYEMYYGLQSVQDKKAVNSSTIFELGSVSKLFTATAGGYAKNKGKISFDDTPGKYWKVL
ADC-26WP_001211238.1_1113AbOC         KKYEMYYGLQSVQDKKAVNSSTIFELGSVSKLFTATAGGYAKNKGKISFDDTPGKYWKVL
ADC-26WP_001211238.1_1112AbOC         KKYEMYYGLQSVQDKKAVNSSTIFELGSVSKLFTATAGGYAKNKGKISFDDTPGKYWKVL
DC-26WP_001211238.1_1105AbBOC         KKYEMYYGLQSVQDKKAVNSSTIFELGSVSKLFTATAGGYAKNKGKISFDDTPGKYWKVL
DC-26WP_001211238.1_1111AbBOC         KKYEMYYGLQSVQDKKAVNSSTIFELGSVSKLFTATAGGYAKNKGKISFDDTPGKYWKVL
ADC-26WP_001211238.1_503AbBOC         KKYEMYYGLQSVQDKKAVNSSTIFELGSVSKLFTATAGGYAKNKGKISFDDTPGKYWKVL
DC-26WP_001211238.1_2774AbBOC         KKYEMYYGLQSVQDKKAVNSSTIFELGSVSKLFTATAGGYAKNKGKISFDDTPGKYWKVL
DC-26WP_001211238.1_1064AbBOC         KKYEMYYGLQSVQDKKAVNSSTIFELGSVSKLFTATAGGYAKNKGKISFDDTPGKYWKVL
DC-26WP_001211238.1_2484AbBOC         KKYEMYYGLQSVQDKKAVNSSTIFELGSVSKLFTATAGGYAKNKGKISFDDTPGKYWKVL
DC-26WP_001211238.1_3858AbBOC         KKYEMYYGLQSVQDKKAVNSSTIFELGSVSKLFTATAGGYAKNKGKISFDDTPGKYWKVL
ADC-26WP_001211238.1_142AbBOC         KKYEMYYGLQSVQDKKAVNSSTIFELGSVSKLFTATAGGYAKNKGKISFDDTPGKYWKVL
DC-26WP_001211238.1_1085AbBOC         KKYEMYYGLQSVQDKKAVNSSTIFELGSVSKLFTATAGGYAKNKGKISFDDTPGKYWKVL
DC-26WP_001211238.1_2525AbBOC         KKYEMYYGLQSVQDKKAVNSSTIFELGSVSKLFTATAGGYAKNKGKISFDDTPGKYWKVL
DC-26WP_001211238.1_2477AbBOC         KKYEMYYGLQSVQDKKAVNSSTIFELGSVSKLFTATAGGYAKNKGKISFDDTPGKYWKVL
DC-26WP_001211238.1_1069AbBOC         KKYEMYYGLQSVQDKKAVNSSTIFELGSVSKLFTATAGGYAKNKGKISFDDTPGKYWKVL
DC-26WP_001211238.1_1029AbBOC         KKYEMYYGLQSVQDKKAVNSSTIFELGSVSKLFTATAGGYAKNKGKISFDDTPGKYWKVL
DC-26WP_001211238.1_2443AbBOC         KKYEMYYGLQSVQDKKAVNSSTIFELGSVSKLFTATAGGYAKNKGKISFDDTPGKYWKVL
DC-26WP_001211238.1_1149AbBOC         KKYEMYYGLQSVQDKKAVNSSTIFELGSVSKLFTATAGGYAKNKGKISFDDTPGKYWKVL
DC-26WP_001211238.1_1148AbBOC         KKYEMYYGLQSVQDKKAVNSSTIFELGSVSKLFTATAGGYAKNKGKISFDDTPGKYWKVL
DC-26WP_001211238.1_1374AbBOC         KKYEMYYGLQSVQDKKAVNSSTIFELGSVSKLFTATAGGYAKNKGKISFDDTPGKYWKVL
DC-26WP_001211238.1_2862AbBOC         KKYEMYYGLQSVQDKKAVNSSTIFELGSVSKLFTATAGGYAKNKGKISFDDTPGKYWKVL
DC-26WP_001211238.1_2629AbBOC         KKYEMYYGLQSVQDKKAVNSSTIFELGSVSKLFTATAGGYAKNKGKISFDDTPGKYWKVL
DC-26WP_001211238.1_1355AbBOC         KKYEMYYGLQSVQDKKAVNSSTIFELGSVSKLFTATAGGYAKNKGKISFDDTPGKYWKVL
DC-26WP_001211238.1_3092AbBOC         KKYEMYYGLQSVQDKKAVNSSTIFELGSVSKLFTATAGGYAKNKGKISFDDTPGKYWKVL
DC-26WP_001211238.1_1086AbBOC         KKYEMYYGLQSVQDKKAVNSSTIFELGSVSKLFTATAGGYAKNKGKISFDDTPGKYWKVL
DC-26WP_001211238.1_1016AbBOC         KKYEMYYGLQSVQDKKAVNSSTIFELGSVSKLFTATAGGYAKNKGKISFDDTPGKYWKVL
ADC-26UUG48795.1_2867AbBOC            KKYEMYYGLQSVQDKKAVNSSTIFELGSVSKLFTATAGGYAKNKGKISFDDTPGKYWKVL
DC-26WP_001211238.1_2535AbBOC         KKYEMYYGLQSVQDKKAVNSSTIFELGSVSKLFTATAGGYAKNKGKISFDDTPGKYWKVL
ADC-26WEN46767.1_1086AbBOC            KKYEMYYGLQSVQDKKAVNSSTIFELGSVSKLFTATAGGYAKNKGKISFDDTPGKYWKVL
ADC-26WEX48630.1_1270AbBOC            KKYEMYYGLQSVQDKKAVNSSTIFELGSVSKLFTATAGGYAKNKGKISFDDTPGKYWKVL
ADC-26WGE92537.1_2034AbBOC            KKYEMYYGLQSVQDKKAVNSSTIFELGSVSKLFTATAGGYAKNKGKISFDDTPGKYWKVL
DC-26WP_001211238.1_1105AbBCP         KKYEMYYGLQSVQDKKAVNSSTIFELGSVSKLFTATAGGYAKNKGKISFDDTPGKYWKVL
DC-26WP_001211238.1_1111AbBCP         KKYEMYYGLQSVQDKKAVNSSTIFELGSVSKLFTATAGGYAKNKGKISFDDTPGKYWKVL
ADC-26WP_001211238.1_503AbBCP         KKYEMYYGLQSVQDKKAVNSSTIFELGSVSKLFTATAGGYAKNKGKISFDDTPGKYWKVL
DC-26WP_001211238.1_2774AbBCP         KKYEMYYGLQSVQDKKAVNSSTIFELGSVSKLFTATAGGYAKNKGKISFDDTPGKYWKVL
DC-26WP_001211238.1_1064AbBCP         KKYEMYYGLQSVQDKKAVNSSTIFELGSVSKLFTATAGGYAKNKGKISFDDTPGKYWKVL
DC-26WP_001211238.1_2484AbBCP         KKYEMYYGLQSVQDKKAVNSSTIFELGSVSKLFTATAGGYAKNKGKISFDDTPGKYWKVL
DC-26WP_001211238.1_3858AbBCP         KKYEMYYGLQSVQDKKAVNSSTIFELGSVSKLFTATAGGYAKNKGKISFDDTPGKYWKVL
ADC-26WP_001211238.1_142AbBCP         KKYEMYYGLQSVQDKKAVNSSTIFELGSVSKLFTATAGGYAKNKGKISFDDTPGKYWKVL
DC-26WP_001211238.1_1085AbBCP         KKYEMYYGLQSVQDKKAVNSSTIFELGSVSKLFTATAGGYAKNKGKISFDDTPGKYWKVL
DC-26WP_001211238.1_2525AbBCP         KKYEMYYGLQSVQDKKAVNSSTIFELGSVSKLFTATAGGYAKNKGKISFDDTPGKYWKVL
DC-26WP_001211238.1_2477AbBCP         KKYEMYYGLQSVQDKKAVNSSTIFELGSVSKLFTATAGGYAKNKGKISFDDTPGKYWKVL
DC-26WP_001211238.1_1069AbBCP         KKYEMYYGLQSVQDKKAVNSSTIFELGSVSKLFTATAGGYAKNKGKISFDDTPGKYWKVL
DC-26WP_001211238.1_1029AbBCP         KKYEMYYGLQSVQDKKAVNSSTIFELGSVSKLFTATAGGYAKNKGKISFDDTPGKYWKVL
DC-26WP_001211238.1_2443AbBCP         KKYEMYYGLQSVQDKKAVNSSTIFELGSVSKLFTATAGGYAKNKGKISFDDTPGKYWKVL
DC-26WP_001211238.1_1149AbBCP         KKYEMYYGLQSVQDKKAVNSSTIFELGSVSKLFTATAGGYAKNKGKISFDDTPGKYWKVL
DC-26WP_001211238.1_1148AbBCP         KKYEMYYGLQSVQDKKAVNSSTIFELGSVSKLFTATAGGYAKNKGKISFDDTPGKYWKVL
DC-26WP_001211238.1_1374AbBCP         KKYEMYYGLQSVQDKKAVNSSTIFELGSVSKLFTATAGGYAKNKGKISFDDTPGKYWKVL
DC-26WP_001211238.1_2862AbBCP         KKYEMYYGLQSVQDKKAVNSSTIFELGSVSKLFTATAGGYAKNKGKISFDDTPGKYWKVL
DC-26WP_001211238.1_2629AbBCP         KKYEMYYGLQSVQDKKAVNSSTIFELGSVSKLFTATAGGYAKNKGKISFDDTPGKYWKVL
DC-26WP_001211238.1_1355AbBCP         KKYEMYYGLQSVQDKKAVNSSTIFELGSVSKLFTATAGGYAKNKGKISFDDTPGKYWKVL
DC-26WP_001211238.1_3092AbBCP         KKYEMYYGLQSVQDKKAVNSSTIFELGSVSKLFTATAGGYAKNKGKISFDDTPGKYWKVL
DC-26WP_001211238.1_1086AbBCP         KKYEMYYGLQSVQDKKAVNSSTIFELGSVSKLFTATAGGYAKNKGKISFDDTPGKYWKVL
DC-26WP_001211238.1_1016AbBCP         KKYEMYYGLQSVQDKKAVNSSTIFELGSVSKLFTATAGGYAKNKGKISFDDTPGKYWKVL
ADC-26UUG48795.1_2867AbBCP            KKYEMYYGLQSVQDKKAVNSSTIFELGSVSKLFTATAGGYAKNKGKISFDDTPGKYWKVL
DC-26WP_001211238.1_2535AbBCP         KKYEMYYGLQSVQDKKAVNSSTIFELGSVSKLFTATAGGYAKNKGKISFDDTPGKYWKVL
ADC-26WEN46767.1_1086AbBCP            KKYEMYYGLQSVQDKKAVNSSTIFELGSVSKLFTATAGGYAKNKGKISFDDTPGKYWKVL
ADC-26WEX48630.1_1270AbBCP            KKYEMYYGLQSVQDKKAVNSSTIFELGSVSKLFTATAGGYAKNKGKISFDDTPGKYWKVL
ADC-26WGE92537.1_2034AbBCP            KKYEMYYGLQSVQDKKAVNSSTIFELGSVSKLFTATAGGYAKNKGKISFDDTPGKYWKVL
C-243WP_001211199.1_2530AbBOC         KKYEMYYGLQSVQDKKAVNSSTIFELGSVSKLFTATAGGYAKNKGKISFDDTPGKYWKEL
C-243WP_001211199.1_2530AbBCP         KKYEMYYGLQSVQDKKAVNSSTIFELGSVSKLFTATAGGYAKNKGKISFDDTPGKYWKEL
ADC-74WP_001211203.1_938AbBOC         KKYEMYYGLQSVQDKKAVNRSTIFELGSVSKLFTATAGGYAKNKGKISFDDTPGKYWKEL
ADC-74WP_001211203.1_2AbBOC           KKYEMYYGLQSVQDKKAVNRSTIFELGSVSKLFTATAGGYAKNKGKISFDDTPGKYWKEL
ADC-74WP_001211203.1_95AbBOC          KKYEMYYGLQSVQDKKAVNRSTIFELGSVSKLFTATAGGYAKNKGKISFDDTPGKYWKEL
ADC-74WP_001211203.1_142AbBOC         KKYEMYYGLQSVQDKKAVNRSTIFELGSVSKLFTATAGGYAKNKGKISFDDTPGKYWKEL
DC-74WP_001211203.1_2317AbBOC         KKYEMYYGLQSVQDKKAVNRSTIFELGSVSKLFTATAGGYAKNKGKISFDDTPGKYWKEL
ADC-74WP_001211203.1_570AbBOC         KKYEMYYGLQSVQDKKAVNRSTIFELGSVSKLFTATAGGYAKNKGKISFDDTPGKYWKEL
DC-74WP_001211203.1_2791AbBOC         KKYEMYYGLQSVQDKKAVNRSTIFELGSVSKLFTATAGGYAKNKGKISFDDTPGKYWKEL
DC-74WP_001211203.1_2790AbBOC         KKYEMYYGLQSVQDKKAVNRSTIFELGSVSKLFTATAGGYAKNKGKISFDDTPGKYWKEL
DC-74WP_001211203.1_1414AbBOC         KKYEMYYGLQSVQDKKAVNRSTIFELGSVSKLFTATAGGYAKNKGKISFDDTPGKYWKEL
ADC-74WP_001211203.1_938AbBCP         KKYEMYYGLQSVQDKKAVNRSTIFELGSVSKLFTATAGGYAKNKGKISFDDTPGKYWKEL
ADC-74WP_001211203.1_2AbBCP           KKYEMYYGLQSVQDKKAVNRSTIFELGSVSKLFTATAGGYAKNKGKISFDDTPGKYWKEL
ADC-74WP_001211203.1_95AbBCP          KKYEMYYGLQSVQDKKAVNRSTIFELGSVSKLFTATAGGYAKNKGKISFDDTPGKYWKEL
ADC-74WP_001211203.1_142AbBCP         KKYEMYYGLQSVQDKKAVNRSTIFELGSVSKLFTATAGGYAKNKGKISFDDTPGKYWKEL
DC-74WP_001211203.1_2317AbBCP         KKYEMYYGLQSVQDKKAVNRSTIFELGSVSKLFTATAGGYAKNKGKISFDDTPGKYWKEL
ADC-74WP_001211203.1_570AbBCP         KKYEMYYGLQSVQDKKAVNRSTIFELGSVSKLFTATAGGYAKNKGKISFDDTPGKYWKEL
DC-74WP_001211203.1_2791AbBCP         KKYEMYYGLQSVQDKKAVNRSTIFELGSVSKLFTATAGGYAKNKGKISFDDTPGKYWKEL
DC-74WP_001211203.1_2790AbBCP         KKYEMYYGLQSVQDKKAVNRSTIFELGSVSKLFTATAGGYAKNKGKISFDDTPGKYWKEL
DC-74WP_001211203.1_1414AbBCP         KKYEMYYGLQSVQDKKAVNRSTIFELGSVSKLFTATAGGYAKNKGKISFDDTPGKYWKEL
C-347WP_138149148.1_2656AbBOC         KKYEMYYGLQSVQDKKAVNSSTIFELGSVSKLFTATAGAYAKNKGKISFDDTPGKYWKEL
C-347WP_138149148.1_2656AbBCP         KKYEMYYGLQSVQDKKAVNSSTIFELGSVSKLFTATAGAYAKNKGKISFDDTPGKYWKEL
ADC-80WP_029424536.1_1068AbOC         KKYEMYYGLQSVQDKKAVNSSTIFELGSVSKLFTATAGGYAKNKGKISFDDTPGKYWKVL
DC-80WP_029424536.1_1061AbBOC         KKYEMYYGLQSVQDKKAVNSSTIFELGSVSKLFTATAGGYAKNKGKISFDDTPGKYWKVL
DC-80WP_029424536.1_2945AbBOC         KKYEMYYGLQSVQDKKAVNSSTIFELGSVSKLFTATAGGYAKNKGKISFDDTPGKYWKVL
DC-80WP_029424536.1_2729AbBOC         KKYEMYYGLQSVQDKKAVNSSTIFELGSVSKLFTATAGGYAKNKGKISFDDTPGKYWKVL
DC-80WP_029424536.1_1008AbBOC         KKYEMYYGLQSVQDKKAVNSSTIFELGSVSKLFTATAGGYAKNKGKISFDDTPGKYWKVL
```

```
DC-80WP_029424536.1_1061AbBCP   KKYEMYYGLQSVQDKKAVNSSTIFELGSVSKLFTATAGGYAKNKGKISFDDTPGKYWKVL
DC-80WP_029424536.1_2945AbBCP   KKYEMYYGLQSVQDKKAVNSSTIFELGSVSKLFTATAGGYAKNKGKISFDDTPGKYWKVL
DC-80WP_029424536.1_2729AbBCP   KKYEMYYGLQSVQDKKAVNSSTIFELGSVSKLFTATAGGYAKNKGKISFDDTPGKYWKVL
DC-80WP_029424536.1_1008AbBCP   KKYEMYYGLQSVQDKKAVNSSTIFELGSVSKLFTATAGGYAKNKGKISFDDTPGKYWKVL
DC-156WP_024436624.1_2681AbOC   KKYEMYYGLQSVQDKKAVNSSTIFELGSVSKLFTATAGGYAKNKGKISFDDTPGKYWKEL
C-156WP_024436624.1_2375AbBOC   KKYEMYYGLQSVQDKKAVNSSTIFELGSVSKLFTATAGGYAKNKGKISFDDTPGKYWKEL
C-156WP_024436624.1_1038AbBOC   KKYEMYYGLQSVQDKKAVNSSTIFELGSVSKLFTATAGGYAKNKGKISFDDTPGKYWKEL
C-156WP_024436624.1_1041AbBOC   KKYEMYYGLQSVQDKKAVNSSTIFELGSVSKLFTATAGGYAKNKGKISFDDTPGKYWKEL
C-156WP_024436624.1_1124AbBOC   KKYEMYYGLQSVQDKKAVNSSTIFELGSVSKLFTATAGGYAKNKGKISFDDTPGKYWKEL
C-156WP_024436624.1_1126AbBOC   KKYEMYYGLQSVQDKKAVNSSTIFELGSVSKLFTATAGGYAKNKGKISFDDTPGKYWKEL
C-156WP_024436624.1_2375AbBCP   KKYEMYYGLQSVQDKKAVNSSTIFELGSVSKLFTATAGGYAKNKGKISFDDTPGKYWKEL
C-156WP_024436624.1_1038AbBCP   KKYEMYYGLQSVQDKKAVNSSTIFELGSVSKLFTATAGGYAKNKGKISFDDTPGKYWKEL
C-156WP_024436624.1_1041AbBCP   KKYEMYYGLQSVQDKKAVNSSTIFELGSVSKLFTATAGGYAKNKGKISFDDTPGKYWKEL
C-156WP_024436624.1_1124AbBCP   KKYEMYYGLQSVQDKKAVNSSTIFELGSVSKLFTATAGGYAKNKGKISFDDTPGKYWKEL
C-156WP_024436624.1_1126AbBCP   KKYEMYYGLQSVQDKKAVNSSTIFELGSVSKLFTATAGGYAKNKGKISFDDTPGKYWKEL
C-165WP_001211239.1_1734AbBOC   KKYEMYYGLQSVQDKKAVNSSTIFELGSVSKLFTATAGGYAKNKGKISFDDTPGKYWKVL
C-165WP_001211239.1_1032AbBOC   KKYEMYYGLQSVQDKKAVNSSTIFELGSVSKLFTATAGGYAKNKGKISFDDTPGKYWKVL
C-165WP_001211239.1_1734AbBCP   KKYEMYYGLQSVQDKKAVNSSTIFELGSVSKLFTATAGGYAKNKGKISFDDTPGKYWKVL
C-165WP_001211239.1_1032AbBCP   KKYEMYYGLQSVQDKKAVNSSTIFELGSVSKLFTATAGGYAKNKGKISFDDTPGKYWKVL
C-263WP_109433738.1_2026AbBOC   KKYEMYYGLQSVQDKKAVNSSTIFELGSVSKLFTATAGGYAKNKGKISFDDTPGKYWKEL
DC-263WP_109433738.1_954AbBOC   KKYEMYYGLQSVQDKKAVNSSTIFELGSVSKLFTATAGGYAKNKGKISFDDTPGKYWKEL
C-263WP_109433738.1_1022AbBOC   KKYEMYYGLQSVQDKKAVNSSTIFELGSVSKLFTATAGGYAKNKGKISFDDTPGKYWKEL
C-263WP_109433738.1_3219AbBOC   KKYEMYYGLQSVQDKKAVNSSTIFELGSVSKLFTATAGGYAKNKGKISFDDTPGKYWKEL
C-263WP_109433738.1_2026AbBCP   KKYEMYYGLQSVQDKKAVNSSTIFELGSVSKLFTATAGGYAKNKGKISFDDTPGKYWKEL
DC-263WP_109433738.1_954AbBCP   KKYEMYYGLQSVQDKKAVNSSTIFELGSVSKLFTATAGGYAKNKGKISFDDTPGKYWKEL
C-263WP_109433738.1_1022AbBCP   KKYEMYYGLQSVQDKKAVNSSTIFELGSVSKLFTATAGGYAKNKGKISFDDTPGKYWKEL
C-263WP_109433738.1_3219AbBCP   KKYEMYYGLQSVQDKKAVNSSTIFELGSVSKLFTATAGGYAKNKGKISFDDTPGKYWKEL
DC-79WP_001159760.1_2471AbBOC   KKYEMYYGLQSVQDKKAVNSSTIFELGSVSKLFTATAGGYAKNKGKISFDDTPGKYWKEL
ADC-79WP_001159760.1_670AbBOC   KKYEMYYGLQSVQDKKAVNSSTIFELGSVSKLFTATAGGYAKNKGKISFDDTPGKYWKEL
DC-79WP_001159760.1_2535AbBOC   KKYEMYYGLQSVQDKKAVNSSTIFELGSVSKLFTATAGGYAKNKGKISFDDTPGKYWKEL
DC-79WP_001159760.1_1080AbBOC   KKYEMYYGLQSVQDKKAVNSSTIFELGSVSKLFTATAGGYAKNKGKISFDDTPGKYWKEL
DC-79WP_001159760.1_2471AbBCP   KKYEMYYGLQSVQDKKAVNSSTIFELGSVSKLFTATAGGYAKNKGKISFDDTPGKYWKEL
ADC-79WP_001159760.1_670AbBCP   KKYEMYYGLQSVQDKKAVNSSTIFELGSVSKLFTATAGGYAKNKGKISFDDTPGKYWKEL
DC-79WP_001159760.1_2535AbBCP   KKYEMYYGLQSVQDKKAVNSSTIFELGSVSKLFTATAGGYAKNKGKISFDDTPGKYWKEL
DC-79WP_001159760.1_1080AbBCP   KKYEMYYGLQSVQDKKAVNSSTIFELGSVSKLFTATAGGYAKNKGKISFDDTPGKYWKEL
ADC-6WP_017725267.1_1459AbBOC   KKYEMYYGLQSVQDKKAVNSSTIFELGSVSKLFTATAGGYAKNKGKISFDDTPGKYWKEL
ADC-6WP_017725267.1_1678AbBOC   KKYEMYYGLQSVQDKKAVNSSTIFELGSVSKLFTATAGGYAKNKGKISFDDTPGKYWKEL
ADC-6WP_017725267.1_2561AbBOC   KKYEMYYGLQSVQDKKAVNSSTIFELGSVSKLFTATAGGYAKNKGKISFDDTPGKYWKEL
ADC-6WP_017725267.1_1459AbBCP   KKYEMYYGLQSVQDKKAVNSSTIFELGSVSKLFTATAGGYAKNKGKISFDDTPGKYWKEL
ADC-6WP_017725267.1_1678AbBCP   KKYEMYYGLQSVQDKKAVNSSTIFELGSVSKLFTATAGGYAKNKGKISFDDTPGKYWKEL
ADC-6WP_017725267.1_2561AbBCP   KKYEMYYGLQSVQDKKAVNSSTIFELGSVSKLFTATAGGYAKNKGKISFDDTPGKYWKEL
ADC-1WP_004714775.1_3726AbBOC   KKYEMYYGLQSVQDKKAVNSSTIFELGSVSKLFTATAGGYAKNKGKISFDDTPGKYWKEL
ADC-1WP_004714775.1_3726AbBCP   KKYEMYYGLQSVQDKKAVNSSTIFELGSVSKLFTATAGGYAKNKGKISFDDTPGKYWKEL
C-181WP_046693238.1_1083AbBOC   KKYEMYYGLQSVQDKKAVNSSTIFELGSVSKLFTATAGGYAKNKGKISFDDTPGKYWKEL
C-181WP_046693238.1_1083AbBCP   KKYEMYYGLQSVQDKKAVNSSTIFELGSVSKLFTATAGGYAKNKGKISFDDTPGKYWKEL
C-238WP_002058891.1_2458AbBOC   KKYEMYYGLQSVQDKKAVNSITIFELGSVSKLFTATAGGYAKNKGKISFDDTPGKYWKEL
C-238WP_002058891.1_2458AbBCP   KKYEMYYGLQSVQDKKAVNSITIFELGSVSKLFTATAGGYAKNKGKISFDDTPGKYWKEL
C-186WP_072292271.1_1675AbBOC   KKYEMYYGLQSVQDKKAVNSSTIFELGSVSKLFTATAGGYAKNKGKISFDDTPGKYWKEL
C-186WP_072292271.1_1675AbBCP   KKYEMYYGLQSVQDKKAVNSSTIFELGSVSKLFTATAGGYAKNKGKISFDDTPGKYWKEL
DC-184WP_059247009.1_1188AbOC   KKYEMYYGLQSVQDKKAVNSSTIFELGSVSKLFTATAGGYAKNKGKISFDDKPGKYWKEL
ADC-328WP_069370341.1_666AbOC   KKYEMYYGLQSVQDKKAVNSSTIFELGSVSKLFTATAGGYAKNKGKISFDDTPGKYWKEL
DC-56WP_031973850.1_1911AbBOC   KKYEMYYGLQSVQDKKAVNSSTIFELGSVSKLFTATAGGYAKNKGKISFDDTPGKYWKEL
DC-56WP_031973850.1_2693AbBOC   KKYEMYYGLQSVQDKKAVNSSTIFELGSVSKLFTATAGGYAKNKGKISFDDTPGKYWKEL
DC-56WP_031973850.1_1911AbBCP   KKYEMYYGLQSVQDKKAVNSSTIFELGSVSKLFTATAGGYAKNKGKISFDDTPGKYWKEL
DC-56WP_031973850.1_2693AbBCP   KKYEMYYGLQSVQDKKAVNSSTIFELGSVSKLFTATAGGYAKNKGKISFDDTPGKYWKEL
DC-117WP_002157727.1_394AbBOC   KKYEMYYGLQSVQDKKAVNSSTIFELGSVSKLFTATAGGYAKNKGKISFDDTPGKYWKEL
DC-117WP_002157727.1_394AbBCP   KKYEMYYGLQSVQDKKAVNSSTIFELGSVSKLFTATAGGYAKNKGKISFDDTPGKYWKEL
ADC-25WP_001211217.1_2432AbOC   KKYEMYYGLQSVQDKKAVNSSTIFELGSVSKLFTATAGGYAKNKGKISFDDTPGKYWKEL
ADC-25WP_001211217.1_1025AbOC   KKYEMYYGLQSVQDKKAVNSSTIFELGSVSKLFTATAGGYAKNKGKISFDDTPGKYWKEL
ADC-25WP_001211217.1_3391AbOC   KKYEMYYGLQSVQDKKAVNSSTIFELGSVSKLFTATAGGYAKNKGKISFDDTPGKYWKEL
ADC-25WP_001211217.1_1042AbOC   KKYEMYYGLQSVQDKKAVNSSTIFELGSVSKLFTATAGGYAKNKGKISFDDTPGKYWKEL
ADC-25WP_001211217.1_1185AbOC   KKYEMYYGLQSVQDKKAVNSSTIFELGSVSKLFTATAGGYAKNKGKISFDDTPGKYWKEL
ADC-25WP_001211217.1_768AbOC    KKYEMYYGLQSVQDKKAVNSSTIFELGSVSKLFTATAGGYAKNKGKISFDDTPGKYWKEL
ADC-25WP_001211217.1_2605AbOC   KKYEMYYGLQSVQDKKAVNSSTIFELGSVSKLFTATAGGYAKNKGKISFDDTPGKYWKEL
DC-25WP_001211217.1_1117AbBOC   KKYEMYYGLQSVQDKKAVNSSTIFELGSVSKLFTATAGGYAKNKGKISFDDTPGKYWKEL
DC-25WP_001211217.1_1090AbBOC   KKYEMYYGLQSVQDKKAVNSSTIFELGSVSKLFTATAGGYAKNKGKISFDDTPGKYWKEL
DC-25WP_001211217.1_1089AbBOC   KKYEMYYGLQSVQDKKAVNSSTIFELGSVSKLFTATAGGYAKNKGKISFDDTPGKYWKEL
DC-25WP_001211217.1_1091AbBOC   KKYEMYYGLQSVQDKKAVNSSTIFELGSVSKLFTATAGGYAKNKGKISFDDTPGKYWKEL
DC-25WP_001211217.1_1604AbBOC   KKYEMYYGLQSVQDKKAVNSSTIFELGSVSKLFTATAGGYAKNKGKISFDDTPGKYWKEL
DC-25WP_001211217.1_1040AbBOC   KKYEMYYGLQSVQDKKAVNSSTIFELGSVSKLFTATAGGYAKNKGKISFDDTPGKYWKEL
ADC-25UYQ12932.1_2634AbBOC      KKYEMYYGLQSVQDKKAVNSSTIFELGSVSKLFTATAGGYAKNKGKISFDDTPGKYWKEL
ADC-25UYQ09083.1_2628AbBOC      KKYEMYYGLQSVQDKKAVNSSTIFELGSVSKLFTATAGGYAKNKGKISFDDTPGKYWKEL
ADC-25UYQ40822.1_1120AbBOC      KKYEMYYGLQSVQDKKAVNSSTIFELGSVSKLFTATAGGYAKNKGKISFDDTPGKYWKEL
DC-25WP_001211217.1_1117AbBCP   KKYEMYYGLQSVQDKKAVNSSTIFELGSVSKLFTATAGGYAKNKGKISFDDTPGKYWKEL
DC-25WP_001211217.1_1090AbBCP   KKYEMYYGLQSVQDKKAVNSSTIFELGSVSKLFTATAGGYAKNKGKISFDDTPGKYWKEL
DC-25WP_001211217.1_1089AbBCP   KKYEMYYGLQSVQDKKAVNSSTIFELGSVSKLFTATAGGYAKNKGKISFDDTPGKYWKEL
DC-25WP_001211217.1_1091AbBCP   KKYEMYYGLQSVQDKKAVNSSTIFELGSVSKLFTATAGGYAKNKGKISFDDTPGKYWKEL
DC-25WP_001211217.1_1604AbBCP   KKYEMYYGLQSVQDKKAVNSSTIFELGSVSKLFTATAGGYAKNKGKISFDDTPGKYWKEL
DC-25WP_001211217.1_1040AbBCP   KKYEMYYGLQSVQDKKAVNSSTIFELGSVSKLFTATAGGYAKNKGKISFDDTPGKYWKEL
ADC-25UYQ12932.1_2634AbBCP      KKYEMYYGLQSVQDKKAVNSSTIFELGSVSKLFTATAGGYAKNKGKISFDDTPGKYWKEL
ADC-25UYQ09083.1_2628AbBCP      KKYEMYYGLQSVQDKKAVNSSTIFELGSVSKLFTATAGGYAKNKGKISFDDTPGKYWKEL
ADC-25UYQ40822.1_1120AbBCP      KKYEMYYGLQSVQDKKAVNSSTIFELGSVSKLFTATAGGYAKNKGKISFDDTPGKYWKEL
C-268WP_105922788.1_2839AbBOC   KKYEMYYGLQSVQDKKAVNSSTIFELGSVSKLFTATAGGYAKNKGKISFDDTPGKYWKEL
ADC-268WDC47378.1_2492AbBOC     KKYEMYYGLQSVQDKKAVNSSTIFELGSVSKLFTATAGGYAKNKGKISFDDTPGKYWKEL
C-268WP_105922788.1_2839AbBCP   KKYEMYYGLQSVQDKKAVNSSTIFELGSVSKLFTATAGGYAKNKGKISFDDTPGKYWKEL
ADC-268WDC47378.1_2492AbBCP     KKYEMYYGLQSVQDKKAVNSSTIFELGSVSKLFTATAGGYAKNKGKISFDDTPGKYWKEL
DC-155WP_005128228.1_2591AbOC   KKYEMYYGLQSVQDKKAVNSSTIFELGSVSKLFTGTAGGYAKNKGKISFDDKPGKYWKEL
C-155WP_005128228.1_2549AbBOC   KKYEMYYGLQSVQDKKAVNSSTIFELGSVSKLFTGTAGGYAKNKGKISFDDKPGKYWKEL
C-155WP_005128228.1_2480AbBOC   KKYEMYYGLQSVQDKKAVNSSTIFELGSVSKLFTGTAGGYAKNKGKISFDDKPGKYWKEL
C-155WP_005128228.1_2455AbBOC   KKYEMYYGLQSVQDKKAVNSSTIFELGSVSKLFTGTAGGYAKNKGKISFDDKPGKYWKEL
C-155WP_005128228.1_2549AbBCP   KKYEMYYGLQSVQDKKAVNSSTIFELGSVSKLFTGTAGGYAKNKGKISFDDKPGKYWKEL
C-155WP_005128228.1_2480AbBCP   KKYEMYYGLQSVQDKKAVNSSTIFELGSVSKLFTGTAGGYAKNKGKISFDDKPGKYWKEL
C-155WP_005128228.1_2455AbBCP   KKYEMYYGLQSVQDKKAVNSSTIFELGSVSKLFTGTAGGYAKNKGKISFDDKPGKYWKEL
ADC-76WP_001211237.1_2862AbOC   KKYEMYYGLQSVQDKKAVNSSTIFELGSVSKLFTATAGGYAKNKGKISFDDTPGKYWKVL
ADC-76WP_001211237.1_3431AbOC   KKYEMYYGLQSVQDKKAVNSSTIFELGSVSKLFTATAGGYAKNKGKISFDDTPGKYWKVL
ADC-76WP_001211237.1_2696AbOC   KKYEMYYGLQSVQDKKAVNSSTIFELGSVSKLFTATAGGYAKNKGKISFDDTPGKYWKVL
ADC-76WP_001211237.1_2890AbOC   KKYEMYYGLQSVQDKKAVNSSTIFELGSVSKLFTATAGGYAKNKGKISFDDTPGKYWKVL
DC-76WP_001211237.1_2723AbBOC   KKYEMYYGLQSVQDKKAVNSSTIFELGSVSKLFTATAGGYAKNKGKISFDDTPGKYWKVL
DC-76WP_001211237.1_1033AbBOC   KKYEMYYGLQSVQDKKAVNSSTIFELGSVSKLFTATAGGYAKNKGKISFDDTPGKYWKVL
ADC-76WP_001211237.1_526AbBOC   KKYEMYYGLQSVQDKKAVNSSTIFELGSVSKLFTATAGGYAKNKGKISFDDTPGKYWKVL
DC-76WP_001211237.1_1046AbBOC   KKYEMYYGLQSVQDKKAVNSSTIFELGSVSKLFTATAGGYAKNKGKISFDDTPGKYWKVL
DC-76WP_001211237.1_1040AbBOC   KKYEMYYGLQSVQDKKAVNSSTIFELGSVSKLFTATAGGYAKNKGKISFDDTPGKYWKVL
DC-76WP_001211237.1_3682AbBOC   KKYEMYYGLQSVQDKKAVNSSTIFELGSVSKLFTATAGGYAKNKGKISFDDTPGKYWKVL
DC-76WP_001211237.1_2579AbBOC   KKYEMYYGLQSVQDKKAVNSSTIFELGSVSKLFTATAGGYAKNKGKISFDDTPGKYWKVL
DC-76WP_001211237.1_2723AbBCP   KKYEMYYGLQSVQDKKAVNSSTIFELGSVSKLFTATAGGYAKNKGKISFDDTPGKYWKVL
DC-76WP_001211237.1_1033AbBCP   KKYEMYYGLQSVQDKKAVNSSTIFELGSVSKLFTATAGGYAKNKGKISFDDTPGKYWKVL
ADC-76WP_001211237.1_526AbBCP   KKYEMYYGLQSVQDKKAVNSSTIFELGSVSKLFTATAGGYAKNKGKISFDDTPGKYWKVL
DC-76WP_001211237.1_1046AbBCP   KKYEMYYGLQSVQDKKAVNSSTIFELGSVSKLFTATAGGYAKNKGKISFDDTPGKYWKVL
DC-76WP_001211237.1_1040AbBCP   KKYEMYYGLQSVQDKKAVNSSTIFELGSVSKLFTATAGGYAKNKGKISFDDTPGKYWKVL
DC-76WP_001211237.1_3682AbBCP   KKYEMYYGLQSVQDKKAVNSSTIFELGSVSKLFTATAGGYAKNKGKISFDDTPGKYWKVL
DC-76WP_001211237.1_2579AbBCP   KKYEMYYGLQSVQDKKAVNSSTIFELGSVSKLFTATAGGYAKNKGKISFDDTPGKYWKVL
DC-291WP_001211201.1_1060AbOC   KKYEMYYGLQSVQDKKAVNSSSIFELGSVSKLFTATAGAYAKNKGKISFDDTPGKYWKEL
C-291WP_001211201.1_2415AbBOC   KKYEMYYGLQSVQDKKAVNSSSIFELGSVSKLFTATAGAYAKNKGKISFDDTPGKYWKEL
C-291WP_001211201.1_2415AbBCP   KKYEMYYGLQSVQDKKAVNSSSIFELGSVSKLFTATAGAYAKNKGKISFDDTPGKYWKEL
DC-214WP_060454527.1_118AbBOC   KKYEMYYGLQSVQDKKAVNSSTIFELGSVSKLFTATAGGYAKNKGKISFDDTPGKYWKEL
DC-214WP_060454527.1_118AbBCP   KKYEMYYGLQSVQDKKAVNSSTIFELGSVSKLFTATAGGYAKNKGKISFDDTPGKYWKEL
C-185WP_001211210.1_2745AbBOC   KKYEMYYGLQSVQDKKAVNSSTIFELGSVSKLFNATAGGYAKNKGKISFDDTPGKYWKEL
ADC-185WEI07035.1_298AbBOC     KKYEMYYGLQSVQDKKAVNSSTIFELGSVSKLFNATAGGYAKNKGKISFDDTPGKYWKEL
C-185WP_001211210.1_2745AbBCP   KKYEMYYGLQSVQDKKAVNSSTIFELGSVSKLFNATAGGYAKNKGKISFDDTPGKYWKEL
ADC-185WEI07035.1_298AbBCP     KKYEMYYGLQSVQDKKAVNSSTIFELGSVSKLFNATAGGYAKNKGKISFDDTPGKYWKEL
ADC-11WP_001211205.1_1099AbOC   KKYEMYYGLQSVQDKKAVNRSTIFELGSVSKLFTATAGGYAKNKGKISFDDTPGKYWKEL
ADC-11WP_001211205.1_1035AbOC   KKYEMYYGLQSVQDKKAVNRSTIFELGSVSKLFTATAGGYAKNKGKISFDDTPGKYWKEL
DC-11WP_001211205.1_2603AbBOC   KKYEMYYGLQSVQDKKAVNRSTIFELGSVSKLFTATAGGYAKNKGKISFDDTPGKYWKEL
DC-11WP_001211205.1_1138AbBOC   KKYEMYYGLQSVQDKKAVNRSTIFELGSVSKLFTATAGGYAKNKGKISFDDTPGKYWKEL
DC-11WP_001211205.1_2874AbBOC   KKYEMYYGLQSVQDKKAVNRSTIFELGSVSKLFTATAGGYAKNKGKISFDDTPGKYWKEL
DC-11WP_001211205.1_2686AbBOC   KKYEMYYGLQSVQDKKAVNRSTIFELGSVSKLFTATAGGYAKNKGKISFDDTPGKYWKEL
ADC-11WP_001211205.1_130AbBOC   KKYEMYYGLQSVQDKKAVNRSTIFELGSVSKLFTATAGGYAKNKGKISFDDTPGKYWKEL
DC-11WP_001211205.1_1097AbBOC   KKYEMYYGLQSVQDKKAVNRSTIFELGSVSKLFTATAGGYAKNKGKISFDDTPGKYWKEL
ADC-11WP_001211205.1_684AbBOC   KKYEMYYGLQSVQDKKAVNRSTIFELGSVSKLFTATAGGYAKNKGKISFDDTPGKYWKEL
ADC-11WP_001211205.1_682AbBOC   KKYEMYYGLQSVQDKKAVNRSTIFELGSVSKLFTATAGGYAKNKGKISFDDTPGKYWKEL
DC-11WP_001211205.1_1111AbBOC   KKYEMYYGLQSVQDKKAVNRSTIFELGSVSKLFTATAGGYAKNKGKISFDDTPGKYWKEL
DC-11WP_001211205.1_1059AbBOC   KKYEMYYGLQSVQDKKAVNRSTIFELGSVSKLFTATAGGYAKNKGKISFDDTPGKYWKEL
DC-11WP_001211205.1_2754AbBOC   KKYEMYYGLQSVQDKKAVNRSTIFELGSVSKLFTATAGGYAKNKGKISFDDTPGKYWKEL
DC-11WP_001211205.1_2603AbBCP   KKYEMYYGLQSVQDKKAVNRSTIFELGSVSKLFTATAGGYAKNKGKISFDDTPGKYWKEL
DC-11WP_001211205.1_1138AbBCP   KKYEMYYGLQSVQDKKAVNRSTIFELGSVSKLFTATAGGYAKNKGKISFDDTPGKYWKEL
DC-11WP_001211205.1_2874AbBCP   KKYEMYYGLQSVQDKKAVNRSTIFELGSVSKLFTATAGGYAKNKGKISFDDTPGKYWKEL
DC-11WP_001211205.1_2686AbBCP   KKYEMYYGLQSVQDKKAVNRSTIFELGSVSKLFTATAGGYAKNKGKISFDDTPGKYWKEL
ADC-11WP_001211205.1_130AbBCP   KKYEMYYGLQSVQDKKAVNRSTIFELGSVSKLFTATAGGYAKNKGKISFDDTPGKYWKEL
DC-11WP_001211205.1_1097AbBCP   KKYEMYYGLQSVQDKKAVNRSTIFELGSVSKLFTATAGGYAKNKGKISFDDTPGKYWKEL
ADC-11WP_001211205.1_684AbBCP   KKYEMYYGLQSVQDKKAVNRSTIFELGSVSKLFTATAGGYAKNKGKISFDDTPGKYWKEL
ADC-11WP_001211205.1_682AbBCP   KKYEMYYGLQSVQDKKAVNRSTIFELGSVSKLFTATAGGYAKNKGKISFDDTPGKYWKEL
DC-11WP_001211205.1_1111AbBCP   KKYEMYYGLQSVQDKKAVNRSTIFELGSVSKLFTATAGGYAKNKGKISFDDTPGKYWKEL
DC-11WP_001211205.1_1059AbBCP   KKYEMYYGLQSVQDKKAVNRSTIFELGSVSKLFTATAGGYAKNKGKISFDDTPGKYWKEL
DC-11WP_001211205.1_2754AbBCP   KKYEMYYGLQSVQDKKAVNRSTIFELGSVSKLFTATAGGYAKNKGKISFDDTPGKYWKEL
C-269WP_032025626.1_2988AbBOC   KKYEMYYGLQSVQDKKAVNSSTIFELGSVSKLFTATAGGYAKNKGKISFDDTPGKYWKEL
C-269WP_032025626.1_2988AbBCP   KKYEMYYGLQSVQDKKAVNSSTIFELGSVSKLFTATAGGYAKNKGKISFDDTPGKYWKEL
C-166WP_101244937.1_1028AbBOC   KKYEMYYGLQSVQDKKAVNSSTIFELGSVSKLFTATAGGYAKNKGKISFDDTPGKYWKEL
C-166WP_101244937.1_1028AbBCP   KKYEMYYGLQSVQDKKAVNSSTIFELGSVSKLFTATAGGYAKNKGKISFDDTPGKYWKEL
C-175WP_001211208.1_2561AbBOC   KKYEMYYGLQSVQDKKAVNRSTIFELGSVSKLFTATAGGYAKNKGKISFDDTPGKYWKEL
C-175WP_001211208.1_2561AbBCP   KKYEMYYGLQSVQDKKAVNRSTIFELGSVSKLFTATAGGYAKNKGKISFDDTPGKYWKEL
C-154WP_005138362.1_2451AbBOC   KKYEMYYGLQSVQDKKAVNSSTIFELGSVSKLFTATAGGYAKNKGKISFDDTPGKYWKVL
DC-154WP_005138362.1_158AbBOC   KKYEMYYGLQSVQDKKAVNSSTIFELGSVSKLFTATAGGYAKNKGKISFDDTPGKYWKVL
```

C-154WP_005138362.1_1167AbBOC	KKYEMYYGLQSVQDKKAVNSSTIFELGSVSKLFTATAGGYAKNKGKISFDDTPGKYWKVL
C-154WP_005138362.1_2510AbBOC	KKYEMYYGLQSVQDKKAVNSSTIFELGSVSKLFTATAGGYAKNKGKISFDDTPGKYWKVL
C-154WP_005138362.1_2513AbBOC	KKYEMYYGLQSVQDKKAVNSSTIFELGSVSKLFTATAGGYAKNKGKISFDDTPGKYWKVL
C-154WP_005138362.1_2512AbBOC	KKYEMYYGLQSVQDKKAVNSSTIFELGSVSKLFTATAGGYAKNKGKISFDDTPGKYWKVL
C-154WP_005138362.1_2451AbBCP	KKYEMYYGLQSVQDKKAVNSSTIFELGSVSKLFTATAGGYAKNKGKISFDDTPGKYWKVL
DC-154WP_005138362.1_158AbBCP	KKYEMYYGLQSVQDKKAVNSSTIFELGSVSKLFTATAGGYAKNKGKISFDDTPGKYWKVL
C-154WP_005138362.1_1167AbBCP	KKYEMYYGLQSVQDKKAVNSSTIFELGSVSKLFTATAGGYAKNKGKISFDDTPGKYWKVL
C-154WP_005138362.1_2510AbBCP	KKYEMYYGLQSVQDKKAVNSSTIFELGSVSKLFTATAGGYAKNKGKISFDDTPGKYWKVL
C-154WP_005138362.1_2513AbBCP	KKYEMYYGLQSVQDKKAVNSSTIFELGSVSKLFTATAGGYAKNKGKISFDDTPGKYWKVL
C-154WP_005138362.1_2512AbBCP	KKYEMYYGLQSVQDKKAVNSSTIFELGSVSKLFTATAGGYAKNKGKISFDDTPGKYWKVL
C-240WP_001211207.1_1200AbBOC	KKYEMYYGLQSVQDKKAVNRSTIFELGSVSKLFTATAGGYAKNKGKISFDDTPGKYWKEL
C-240WP_001211207.1_1200AbBCP	KKYEMYYGLQSVQDKKAVNRSTIFELGSVSKLFTATAGGYAKNKGKISFDDTPGKYWKEL
ADC-75WP_063857817.1_1610AbOC	KKYEMYYGLQSVQDKKAVNSSTIFELGSVSKLFTATAGGYAKNKGKISFDDTPGKYWKEL
DC-18WP_002118772.1_1702AbBOC	KKYEIYYGLQSVQDKKAVNSSTIFELGSVSKLFTATAGGYAKAKGKISFDDTPGKYWKEL
DC-18WP_002118772.1_1702AbBCP	KKYEIYYGLQSVQDKKAVNSSTIFELGSVSKLFTATAGGYAKAKGKISFDDTPGKYWKEL
C-106WP_068981613.1_2650AbBOC	KKYEMYYGLQSVQDKKAVNSSTIFELGSVSKLFTATAGGYAKNKGKISFDDTPGKYWKEL
C-106WP_068981613.1_2650AbBCP	KKYEMYYGLQSVQDKKAVNSSTIFELGSVSKLFTATAGGYAKNKGKISFDDTPGKYWKEL
DC-50WP_031965243.1_2562AbBOC	KKYEMYYGLQSVQDKKAVNSSTIFELGSVSKLFTATAGGYAKNKGKISFDDTPGKYWKEL
ADC-50WP_031965243.1_206AbBOC	KKYEMYYGLQSVQDKKAVNSSTIFELGSVSKLFTATAGGYAKNKGKISFDDTPGKYWKEL
DC-50WP_031965243.1_2751AbBOC	KKYEMYYGLQSVQDKKAVNSSTIFELGSVSKLFTATAGGYAKNKGKISFDDTPGKYWKEL
DC-50WP_031965243.1_3766AbBOC	KKYEMYYGLQSVQDKKAVNSSTIFELGSVSKLFTATAGGYAKNKGKISFDDTPGKYWKEL
DC-50WP_031965243.1_2562AbBCP	KKYEMYYGLQSVQDKKAVNSSTIFELGSVSKLFTATAGGYAKNKGKISFDDTPGKYWKEL
ADC-50WP_031965243.1_206AbBCP	KKYEMYYGLQSVQDKKAVNSSTIFELGSVSKLFTATAGGYAKNKGKISFDDTPGKYWKEL
DC-50WP_031965243.1_2751AbBCP	KKYEMYYGLQSVQDKKAVNSSTIFELGSVSKLFTATAGGYAKNKGKISFDDTPGKYWKEL
DC-50WP_031965243.1_3766AbBCP	KKYEMYYGLQSVQDKKAVNSSTIFELGSVSKLFTATAGGYAKNKGKISFDDTPGKYWKEL
ADC-158WP_001211227.1_990AbOC	KKYEMYYGLQSVQDKKAVNSSTIFELGSVSKLFTATAGGYAKNKGKISFDDTPGKYWKEL
DC-158WP_001211227.1_1295AbOC	KKYEMYYGLQSVQDKKAVNSSTIFELGSVSKLFTATAGGYAKNKGKISFDDTPGKYWKEL
ADC-158WP_001211227.1_6AbOC	KKYEMYYGLQSVQDKKAVNSSTIFELGSVSKLFTATAGGYAKNKGKISFDDTPGKYWKEL
DC-158WP_001211227.1_991AbBOC	KKYEMYYGLQSVQDKKAVNSSTIFELGSVSKLFTATAGGYAKNKGKISFDDTPGKYWKEL
DC-158WP_001211227.1_207AbBOC	KKYEMYYGLQSVQDKKAVNSSTIFELGSVSKLFTATAGGYAKNKGKISFDDTPGKYWKEL
C-158WP_001211227.1_2735AbBOC	KKYEMYYGLQSVQDKKAVNSSTIFELGSVSKLFTATAGGYAKNKGKISFDDTPGKYWKEL
DC-158WP_001211227.1_996AbBOC	KKYEMYYGLQSVQDKKAVNSSTIFELGSVSKLFTATAGGYAKNKGKISFDDTPGKYWKEL
DC-158WP_001211227.1_796AbBOC	KKYEMYYGLQSVQDKKAVNSSTIFELGSVSKLFTATAGGYAKNKGKISFDDTPGKYWKEL
C-158WP_001211227.1_2822AbBOC	KKYEMYYGLQSVQDKKAVNSSTIFELGSVSKLFTATAGGYAKNKGKISFDDTPGKYWKEL
C-158WP_001211227.1_1339AbBOC	KKYEMYYGLQSVQDKKAVNSSTIFELGSVSKLFTATAGGYAKNKGKISFDDTPGKYWKEL
ADC-158WGQ04828.1_2712AbBOC	KKYEMYYGLQSVQDKKAVNSSTIFELGSVSKLFTATAGGYAKNKGKISFDDTPGKYWKEL
DC-158WP_001211227.1_991AbBCP	KKYEMYYGLQSVQDKKAVNSSTIFELGSVSKLFTATAGGYAKNKGKISFDDTPGKYWKEL
DC-158WP_001211227.1_207AbBCP	KKYEMYYGLQSVQDKKAVNSSTIFELGSVSKLFTATAGGYAKNKGKISFDDTPGKYWKEL
C-158WP_001211227.1_2735AbBCP	KKYEMYYGLQSVQDKKAVNSSTIFELGSVSKLFTATAGGYAKNKGKISFDDTPGKYWKEL
DC-158WP_001211227.1_996AbBCP	KKYEMYYGLQSVQDKKAVNSSTIFELGSVSKLFTATAGGYAKNKGKISFDDTPGKYWKEL
DC-158WP_001211227.1_796AbBCP	KKYEMYYGLQSVQDKKAVNSSTIFELGSVSKLFTATAGGYAKNKGKISFDDTPGKYWKEL
C-158WP_001211227.1_2822AbBCP	KKYEMYYGLQSVQDKKAVNSSTIFELGSVSKLFTATAGGYAKNKGKISFDDTPGKYWKEL
C-158WP_001211227.1_1339AbBCP	KKYEMYYGLQSVQDKKAVNSSTIFELGSVSKLFTATAGGYAKNKGKISFDDTPGKYWKEL
ADC-158WGQ04828.1_2712AbBCP	KKYEMYYGLQSVQDKKAVNSSTIFELGSVSKLFTATAGGYAKNKGKISFDDTPGKYWKEL
ADC-31WP_001211223.1_2986AbOC	KKYEMYYGLQSVQDKKAVNSSTIFELGSVSKLFTATAGGYAKNKGKISFDDTPGKYWKEL
DC-31WP_001211223.1_2698AbBOC	KKYEMYYGLQSVQDKKAVNSSTIFELGSVSKLFTATAGGYAKNKGKISFDDTPGKYWKEL
DC-31WP_001211223.1_2698AbBCP	KKYEMYYGLQSVQDKKAVNSSTIFELGSVSKLFTATAGGYAKNKGKISFDDTPGKYWKEL
ADC-30WP_001211218.1_1102AbOC	KKYEMYYGLQSVQDKKAVNSSTIFELGSVSKLFTATAGGYAKNKGKISFDDTPGKYWKEL
ADC-30WP_001211218.1_2565AbOC	KKYEMYYGLQSVQDKKAVNSSTIFELGSVSKLFTATAGGYAKNKGKISFDDTPGKYWKEL
ADC-30WP_001211218.1_1021AbOC	KKYEMYYGLQSVQDKKAVNSSTIFELGSVSKLFTATAGGYAKNKGKISFDDTPGKYWKEL
ADC-30WP_001211218.1_1088AbOC	KKYEMYYGLQSVQDKKAVNSSTIFELGSVSKLFTATAGGYAKNKGKISFDDTPGKYWKEL
ADC-30WP_001211218.1_1135AbOC	KKYEMYYGLQSVQDKKAVNSSTIFELGSVSKLFTATAGGYAKNKGKISFDDTPGKYWKEL
ADC-30WP_001211218.1_43AbOC	KKYEMYYGLQSVQDKKAVNSSTIFELGSVSKLFTATAGGYAKNKGKISFDDTPGKYWKEL
ADC-30WP_001211218.1_1041AbOC	KKYEMYYGLQSVQDKKAVNSSTIFELGSVSKLFTATAGGYAKNKGKISFDDTPGKYWKEL
ADC-30WP_001211218.1_1032AbOC	KKYEMYYGLQSVQDKKAVNSSTIFELGSVSKLFTATAGGYAKNKGKISFDDTPGKYWKEL
ADC-30WP_001211218.1_1047AbOC	KKYEMYYGLQSVQDKKAVNSSTIFELGSVSKLFTATAGGYAKNKGKISFDDTPGKYWKEL
ADC-30WP_001211218.1_1040AbOC	KKYEMYYGLQSVQDKKAVNSSTIFELGSVSKLFTATAGGYAKNKGKISFDDTPGKYWKEL
ADC-30WP_001211218.1_3388AbOC	KKYEMYYGLQSVQDKKAVNSSTIFELGSVSKLFTATAGGYAKNKGKISFDDTPGKYWKEL
ADC-30WP_001211218.1_650AbOC	KKYEMYYGLQSVQDKKAVNSSTIFELGSVSKLFTATAGGYAKNKGKISFDDTPGKYWKEL
ADC-30WP_001211218.1_2855AbOC	KKYEMYYGLQSVQDKKAVNSSTIFELGSVSKLFTATAGGYAKNKGKISFDDTPGKYWKEL
ADC-30WP_001211218.1_1034AbOC	KKYEMYYGLQSVQDKKAVNSSTIFELGSVSKLFTATAGGYAKNKGKISFDDTPGKYWKEL
ADC-30WP_001211218.1_79AbOC	KKYEMYYGLQSVQDKKAVNSSTIFELGSVSKLFTATAGGYAKNKGKISFDDTPGKYWKEL
ADC-30WP_001211218.1_3509AbOC	KKYEMYYGLQSVQDKKAVNSSTIFELGSVSKLFTATAGGYAKNKGKISFDDTPGKYWKEL
ADC-30WP_001211218.1_2399AbOC	KKYEMYYGLQSVQDKKAVNSSTIFELGSVSKLFTATAGGYAKNKGKISFDDTPGKYWKEL
ADC-30WP_001211218.1_2415AbOC	KKYEMYYGLQSVQDKKAVNSSTIFELGSVSKLFTATAGGYAKNKGKISFDDTPGKYWKEL
ADC-30WP_001211218.1_3032AbOC	KKYEMYYGLQSVQDKKAVNSSTIFELGSVSKLFTATAGGYAKNKGKISFDDTPGKYWKEL
ADC-30WP_001211218.1_832AbOC	KKYEMYYGLQSVQDKKAVNSSTIFELGSVSKLFTATAGGYAKNKGKISFDDTPGKYWKEL
ADC-30WP_001211218.1_1428AbOC	KKYEMYYGLQSVQDKKAVNSSTIFELGSVSKLFTATAGGYAKNKGKISFDDTPGKYWKEL
ADC-30WP_001211218.1_2340AbOC	KKYEMYYGLQSVQDKKAVNSSTIFELGSVSKLFTATAGGYAKNKGKISFDDTPGKYWKEL
ADC-30WP_001211218.1_2976AbOC	KKYEMYYGLQSVQDKKAVNSSTIFELGSVSKLFTATAGGYAKNKGKISFDDTPGKYWKEL
ADC-30WP_001211218.1_2305AbOC	KKYEMYYGLQSVQDKKAVNSSTIFELGSVSKLFTATAGGYAKNKGKISFDDTPGKYWKEL
ADC-30WP_001211218.1_1110AbOC	KKYEMYYGLQSVQDKKAVNSSTIFELGSVSKLFTATAGGYAKNKGKISFDDTPGKYWKEL
ADC-30WP_001211218.1_2797AbOC	KKYEMYYGLQSVQDKKAVNSSTIFELGSVSKLFTATAGGYAKNKGKISFDDTPGKYWKEL
ADC-30WP_001211218.1_1023AbOC	KKYEMYYGLQSVQDKKAVNSSTIFELGSVSKLFTATAGGYAKNKGKISFDDTPGKYWKEL
ADC-30WP_001211218.1_1022AbOC	KKYEMYYGLQSVQDKKAVNSSTIFELGSVSKLFTATAGGYAKNKGKISFDDTPGKYWKEL
ADC-30WP_001211218.1_2458AbOC	KKYEMYYGLQSVQDKKAVNSSTIFELGSVSKLFTATAGGYAKNKGKISFDDTPGKYWKEL
ADC-30WP_001211218.1_2513AbOC	KKYEMYYGLQSVQDKKAVNSSTIFELGSVSKLFTATAGGYAKNKGKISFDDTPGKYWKEL
ADC-30WP_001211218.1_2482AbOC	KKYEMYYGLQSVQDKKAVNSSTIFELGSVSKLFTATAGGYAKNKGKISFDDTPGKYWKEL
DC-30WP_001211218.1_2692AbBOC	KKYEMYYGLQSVQDKKAVNSSTIFELGSVSKLFTATAGGYAKNKGKISFDDTPGKYWKEL
DC-30WP_001211218.1_2549AbBOC	KKYEMYYGLQSVQDKKAVNSSTIFELGSVSKLFTATAGGYAKNKGKISFDDTPGKYWKEL
DC-30WP_001211218.1_1026AbBOC	KKYEMYYGLQSVQDKKAVNSSTIFELGSVSKLFTATAGGYAKNKGKISFDDTPGKYWKEL
ADC-30WP_001211218.1_373AbBOC	KKYEMYYGLQSVQDKKAVNSSTIFELGSVSKLFTATAGGYAKNKGKISFDDTPGKYWKEL
DC-30WP_001211218.1_2465AbBOC	KKYEMYYGLQSVQDKKAVNSSTIFELGSVSKLFTATAGGYAKNKGKISFDDTPGKYWKEL
DC-30WP_001211218.1_1027AbBOC	KKYEMYYGLQSVQDKKAVNSSTIFELGSVSKLFTATAGGYAKNKGKISFDDTPGKYWKEL
DC-30WP_001211218.1_2650AbBOC	KKYEMYYGLQSVQDKKAVNSSTIFELGSVSKLFTATAGGYAKNKGKISFDDTPGKYWKEL
DC-30WP_001211218.1_2674AbBOC	KKYEMYYGLQSVQDKKAVNSSTIFELGSVSKLFTATAGGYAKNKGKISFDDTPGKYWKEL
DC-30WP_001211218.1_2689AbBOC	KKYEMYYGLQSVQDKKAVNSSTIFELGSVSKLFTATAGGYAKNKGKISFDDTPGKYWKEL
DC-30WP_001211218.1_1105AbBOC	KKYEMYYGLQSVQDKKAVNSSTIFELGSVSKLFTATAGGYAKNKGKISFDDTPGKYWKEL
DC-30WP_001211218.1_2670AbBOC	KKYEMYYGLQSVQDKKAVNSSTIFELGSVSKLFTATAGGYAKNKGKISFDDTPGKYWKEL
DC-30WP_001211218.1_1019AbBOC	KKYEMYYGLQSVQDKKAVNSSTIFELGSVSKLFTATAGGYAKNKGKISFDDTPGKYWKEL
DC-30WP_001211218.1_1039AbBOC	KKYEMYYGLQSVQDKKAVNSSTIFELGSVSKLFTATAGGYAKNKGKISFDDTPGKYWKEL
DC-30WP_001211218.1_1023AbBOC	KKYEMYYGLQSVQDKKAVNSSTIFELGSVSKLFTATAGGYAKNKGKISFDDTPGKYWKEL
DC-30WP_001211218.1_3189AbBOC	KKYEMYYGLQSVQDKKAVNSSTIFELGSVSKLFTATAGGYAKNKGKISFDDTPGKYWKEL
ADC-30WP_001211218.1_594AbBOC	KKYEMYYGLQSVQDKKAVNSSTIFELGSVSKLFTATAGGYAKNKGKISFDDTPGKYWKEL
DC-30WP_001211218.1_1718AbBOC	KKYEMYYGLQSVQDKKAVNSSTIFELGSVSKLFTATAGGYAKNKGKISFDDTPGKYWKEL
DC-30WP_001211218.1_2484AbBOC	KKYEMYYGLQSVQDKKAVNSSTIFELGSVSKLFTATAGGYAKNKGKISFDDTPGKYWKEL
DC-30WP_001211218.1_3305AbBOC	KKYEMYYGLQSVQDKKAVNSSTIFELGSVSKLFTATAGGYAKNKGKISFDDTPGKYWKEL
DC-30WP_001211218.1_3346AbBOC	KKYEMYYGLQSVQDKKAVNSSTIFELGSVSKLFTATAGGYAKNKGKISFDDTPGKYWKEL
DC-30WP_001211218.1_1482AbBOC	KKYEMYYGLQSVQDKKAVNSSTIFELGSVSKLFTATAGGYAKNKGKISFDDTPGKYWKEL
DC-30WP_001211218.1_2574AbBOC	KKYEMYYGLQSVQDKKAVNSSTIFELGSVSKLFTATAGGYAKNKGKISFDDTPGKYWKEL
ADC-30WP_001211218.1_831AbBOC	KKYEMYYGLQSVQDKKAVNSSTIFELGSVSKLFTATAGGYAKNKGKISFDDTPGKYWKEL
DC-30WP_001211218.1_1826AbBOC	KKYEMYYGLQSVQDKKAVNSSTIFELGSVSKLFTATAGGYAKNKGKISFDDTPGKYWKEL
DC-30WP_001211218.1_1024AbBOC	KKYEMYYGLQSVQDKKAVNSSTIFELGSVSKLFTATAGGYAKNKGKISFDDTPGKYWKEL
DC-30WP_001211218.1_1618AbBOC	KKYEMYYGLQSVQDKKAVNSSTIFELGSVSKLFTATAGGYAKNKGKISFDDTPGKYWKEL
ADC-30WP_001211218.1_301AbBOC	KKYEMYYGLQSVQDKKAVNSSTIFELGSVSKLFTATAGGYAKNKGKISFDDTPGKYWKEL
ADC-30WP_001211218.1_940AbBOC	KKYEMYYGLQSVQDKKAVNSSTIFELGSVSKLFTATAGGYAKNKGKISFDDTPGKYWKEL
DC-30WP_001211218.1_2949AbBOC	KKYEMYYGLQSVQDKKAVNSSTIFELGSVSKLFTATAGGYAKNKGKISFDDTPGKYWKEL
DC-30WP_001211218.1_1028AbBOC	KKYEMYYGLQSVQDKKAVNSSTIFELGSVSKLFTATAGGYAKNKGKISFDDTPGKYWKEL
DC-30WP_001211218.1_2526AbBOC	KKYEMYYGLQSVQDKKAVNSSTIFELGSVSKLFTATAGGYAKNKGKISFDDTPGKYWKEL
DC-30WP_001211218.1_1040AbBOC	KKYEMYYGLQSVQDKKAVNSSTIFELGSVSKLFTATAGGYAKNKGKISFDDTPGKYWKEL
DC-30WP_001211218.1_1089AbBOC	KKYEMYYGLQSVQDKKAVNSSTIFELGSVSKLFTATAGGYAKNKGKISFDDTPGKYWKEL
DC-30WP_001211218.1_1099AbBOC	KKYEMYYGLQSVQDKKAVNSSTIFELGSVSKLFTATAGGYAKNKGKISFDDTPGKYWKEL
DC-30WP_001211218.1_1206AbBOC	KKYEMYYGLQSVQDKKAVNSSTIFELGSVSKLFTATAGGYAKNKGKISFDDTPGKYWKEL
DC-30WP_001211218.1_1167AbBOC	KKYEMYYGLQSVQDKKAVNSSTIFELGSVSKLFTATAGGYAKNKGKISFDDTPGKYWKEL
DC-30WP_001211218.1_1186AbBOC	KKYEMYYGLQSVQDKKAVNSSTIFELGSVSKLFTATAGGYAKNKGKISFDDTPGKYWKEL
DC-30WP_001211218.1_1165AbBOC	KKYEMYYGLQSVQDKKAVNSSTIFELGSVSKLFTATAGGYAKNKGKISFDDTPGKYWKEL
DC-30WP_001211218.1_1077AbBOC	KKYEMYYGLQSVQDKKAVNSSTIFELGSVSKLFTATAGGYAKNKGKISFDDTPGKYWKEL
DC-30WP_001211218.1_1078AbBOC	KKYEMYYGLQSVQDKKAVNSSTIFELGSVSKLFTATAGGYAKNKGKISFDDTPGKYWKEL
DC-30WP_001211218.1_1071AbBOC	KKYEMYYGLQSVQDKKAVNSSTIFELGSVSKLFTATAGGYAKNKGKISFDDTPGKYWKEL
DC-30WP_001211218.1_2694AbBOC	KKYEMYYGLQSVQDKKAVNSSTIFELGSVSKLFTATAGGYAKNKGKISFDDTPGKYWKEL
DC-30WP_001211218.1_3340AbBOC	KKYEMYYGLQSVQDKKAVNSSTIFELGSVSKLFTATAGGYAKNKGKISFDDTPGKYWKEL
DC-30WP_001211218.1_1022AbBOC	KKYEMYYGLQSVQDKKAVNSSTIFELGSVSKLFTATAGGYAKNKGKISFDDTPGKYWKEL
DC-30WP_001211218.1_1025AbBOC	KKYEMYYGLQSVQDKKAVNSSTIFELGSVSKLFTATAGGYAKNKGKISFDDTPGKYWKEL
DC-30WP_001211218.1_1158AbBOC	KKYEMYYGLQSVQDKKAVNSSTIFELGSVSKLFTATAGGYAKNKGKISFDDTPGKYWKEL
DC-30WP_001211218.1_1020AbBOC	KKYEMYYGLQSVQDKKAVNSSTIFELGSVSKLFTATAGGYAKNKGKISFDDTPGKYWKEL
DC-30WP_001211218.1_1021AbBOC	KKYEMYYGLQSVQDKKAVNSSTIFELGSVSKLFTATAGGYAKNKGKISFDDTPGKYWKEL
ADC-30WP_001211218.1_532AbBOC	KKYEMYYGLQSVQDKKAVNSSTIFELGSVSKLFTATAGGYAKNKGKISFDDTPGKYWKEL
ADC-30WP_001211218.1_539AbBOC	KKYEMYYGLQSVQDKKAVNSSTIFELGSVSKLFTATAGGYAKNKGKISFDDTPGKYWKEL
DC-30WP_001211218.1_1033AbBOC	KKYEMYYGLQSVQDKKAVNSSTIFELGSVSKLFTATAGGYAKNKGKISFDDTPGKYWKEL
DC-30WP_001211218.1_1069AbBOC	KKYEMYYGLQSVQDKKAVNSSTIFELGSVSKLFTATAGGYAKNKGKISFDDTPGKYWKEL
DC-30WP_001211218.1_1029AbBOC	KKYEMYYGLQSVQDKKAVNSSTIFELGSVSKLFTATAGGYAKNKGKISFDDTPGKYWKEL
DC-30WP_001211218.1_1030AbBOC	KKYEMYYGLQSVQDKKAVNSSTIFELGSVSKLFTATAGGYAKNKGKISFDDTPGKYWKEL
DC-30WP_001211218.1_1096AbBOC	KKYEMYYGLQSVQDKKAVNSSTIFELGSVSKLFTATAGGYAKNKGKISFDDTPGKYWKEL
DC-30WP_001211218.1_1037AbBOC	KKYEMYYGLQSVQDKKAVNSSTIFELGSVSKLFTATAGGYAKNKGKISFDDTPGKYWKEL
DC-30WP_001211218.1_1098AbBOC	KKYEMYYGLQSVQDKKAVNSSTIFELGSVSKLFTATAGGYAKNKGKISFDDTPGKYWKEL
DC-30WP_001211218.1_1046AbBOC	KKYEMYYGLQSVQDKKAVNSSTIFELGSVSKLFTATAGGYAKNKGKISFDDTPGKYWKEL
DC-30WP_001211218.1_1032AbBOC	KKYEMYYGLQSVQDKKAVNSSTIFELGSVSKLFTATAGGYAKNKGKISFDDTPGKYWKEL
DC-30WP_001211218.1_1070AbBOC	KKYEMYYGLQSVQDKKAVNSSTIFELGSVSKLFTATAGGYAKNKGKISFDDTPGKYWKEL
DC-30WP_001211218.1_1139AbBOC	KKYEMYYGLQSVQDKKAVNSSTIFELGSVSKLFTATAGGYAKNKGKISFDDTPGKYWKEL
DC-30WP_001211218.1_1031AbBOC	KKYEMYYGLQSVQDKKAVNSSTIFELGSVSKLFTATAGGYAKNKGKISFDDTPGKYWKEL
DC-30WP_001211218.1_1066AbBOC	KKYEMYYGLQSVQDKKAVNSSTIFELGSVSKLFTATAGGYAKNKGKISFDDTPGKYWKEL
DC-30WP_001211218.1_1102AbBOC	KKYEMYYGLQSVQDKKAVNSSTIFELGSVSKLFTATAGGYAKNKGKISFDDTPGKYWKEL
ADC-30USX57838.1_2957AbBOC	KKYEMYYGLQSVQDKKAVNSSTIFELGSVSKLFTATAGGYAKNKGKISFDDTPGKYWKEL
ADC-30USX59985.1_1918AbBOC	KKYEMYYGLQSVQDKKAVNSSTIFELGSVSKLFTATAGGYAKNKGKISFDDTPGKYWKEL
ADC-30USX63799.1_2772AbBOC	KKYEMYYGLQSVQDKKAVNSSTIFELGSVSKLFTATAGGYAKNKGKISFDDTPGKYWKEL
ADC-30USX74012.1_305AbBOC	KKYEMYYGLQSVQDKKAVNSSTIFELGSVSKLFTATAGGYAKNKGKISFDDTPGKYWKEL
ADC-30USZ52468.1_2397AbBOC	KKYEMYYGLQSVQDKKAVNSSTIFELGSVSKLFTATAGGYAKNKGKISFDDTPGKYWKEL
DC-30WP_001211218.1_2008AbBOC	KKYEMYYGLQSVQDKKAVNSSTIFELGSVSKLFTATAGGYAKNKGKISFDDTPGKYWKEL
ADC-30WCL78976.1_1026AbBOC	KKYEMYYGLQSVQDKKAVNSSTIFELGSVSKLFTATAGGYAKNKGKISFDDTPGKYWKEL
ADC-30WDO27826.1_1034AbBOC	KKYEMYYGLQSVQDKKAVNSSTIFELGSVSKLFTATAGGYAKNKGKISFDDTPGKYWKEL
DC-30WP_001211218.1_2125AbBOC	KKYEMYYGLQSVQDKKAVNSSTIFELGSVSKLFTATAGGYAKNKGKISFDDTPGKYWKEL
DC-30WP_001211218.1_2729AbBOC	KKYEMYYGLQSVQDKKAVNSSTIFELGSVSKLFTATAGGYAKNKGKISFDDTPGKYWKEL

DC-30WP_001211218.1_2692AbBCP	KKYEMYYGLQSVQDKKAVNSSTIFELGSVSKLFTATAGGYAKNKGKISFDDTPGKYWKEL
DC-30WP_001211218.1_2549AbBCP	KKYEMYYGLQSVQDKKAVNSSTIFELGSVSKLFTATAGGYAKNKGKISFDDTPGKYWKEL
DC-30WP_001211218.1_1026AbBCP	KKYEMYYGLQSVQDKKAVNSSTIFELGSVSKLFTATAGGYAKNKGKISFDDTPGKYWKEL
ADC-30WP_001211218.1_373AbBCP	KKYEMYYGLQSVQDKKAVNSSTIFELGSVSKLFTATAGGYAKNKGKISFDDTPGKYWKEL
DC-30WP_001211218.1_2465AbBCP	KKYEMYYGLQSVQDKKAVNSSTIFELGSVSKLFTATAGGYAKNKGKISFDDTPGKYWKEL
DC-30WP_001211218.1_1027AbBCP	KKYEMYYGLQSVQDKKAVNSSTIFELGSVSKLFTATAGGYAKNKGKISFDDTPGKYWKEL
DC-30WP_001211218.1_2650AbBCP	KKYEMYYGLQSVQDKKAVNSSTIFELGSVSKLFTATAGGYAKNKGKISFDDTPGKYWKEL
DC-30WP_001211218.1_2674AbBCP	KKYEMYYGLQSVQDKKAVNSSTIFELGSVSKLFTATAGGYAKNKGKISFDDTPGKYWKEL
DC-30WP_001211218.1_2689AbBCP	KKYEMYYGLQSVQDKKAVNSSTIFELGSVSKLFTATAGGYAKNKGKISFDDTPGKYWKEL
DC-30WP_001211218.1_1105AbBCP	KKYEMYYGLQSVQDKKAVNSSTIFELGSVSKLFTATAGGYAKNKGKISFDDTPGKYWKEL
DC-30WP_001211218.1_2670AbBCP	KKYEMYYGLQSVQDKKAVNSSTIFELGSVSKLFTATAGGYAKNKGKISFDDTPGKYWKEL
DC-30WP_001211218.1_1019AbBCP	KKYEMYYGLQSVQDKKAVNSSTIFELGSVSKLFTATAGGYAKNKGKISFDDTPGKYWKEL
DC-30WP_001211218.1_1039AbBCP	KKYEMYYGLQSVQDKKAVNSSTIFELGSVSKLFTATAGGYAKNKGKISFDDTPGKYWKEL
DC-30WP_001211218.1_1023AbBCP	KKYEMYYGLQSVQDKKAVNSSTIFELGSVSKLFTATAGGYAKNKGKISFDDTPGKYWKEL
DC-30WP_001211218.1_3189AbBCP	KKYEMYYGLQSVQDKKAVNSSTIFELGSVSKLFTATAGGYAKNKGKISFDDTPGKYWKEL
ADC-30WP_001211218.1_594AbBCP	KKYEMYYGLQSVQDKKAVNSSTIFELGSVSKLFTATAGGYAKNKGKISFDDTPGKYWKEL
DC-30WP_001211218.1_1718AbBCP	KKYEMYYGLQSVQDKKAVNSSTIFELGSVSKLFTATAGGYAKNKGKISFDDTPGKYWKEL
DC-30WP_001211218.1_2484AbBCP	KKYEMYYGLQSVQDKKAVNSSTIFELGSVSKLFTATAGGYAKNKGKISFDDTPGKYWKEL
DC-30WP_001211218.1_3305AbBCP	KKYEMYYGLQSVQDKKAVNSSTIFELGSVSKLFTATAGGYAKNKGKISFDDTPGKYWKEL
DC-30WP_001211218.1_3346AbBCP	KKYEMYYGLQSVQDKKAVNSSTIFELGSVSKLFTATAGGYAKNKGKISFDDTPGKYWKEL
DC-30WP_001211218.1_1482AbBCP	KKYEMYYGLQSVQDKKAVNSSTIFELGSVSKLFTATAGGYAKNKGKISFDDTPGKYWKEL
DC-30WP_001211218.1_2574AbBCP	KKYEMYYGLQSVQDKKAVNSSTIFELGSVSKLFTATAGGYAKNKGKISFDDTPGKYWKEL
ADC-30WP_001211218.1_831AbBCP	KKYEMYYGLQSVQDKKAVNSSTIFELGSVSKLFTATAGGYAKNKGKISFDDTPGKYWKEL
DC-30WP_001211218.1_1826AbBCP	KKYEMYYGLQSVQDKKAVNSSTIFELGSVSKLFTATAGGYAKNKGKISFDDTPGKYWKEL
DC-30WP_001211218.1_1024AbBCP	KKYEMYYGLQSVQDKKAVNSSTIFELGSVSKLFTATAGGYAKNKGKISFDDTPGKYWKEL
DC-30WP_001211218.1_1618AbBCP	KKYEMYYGLQSVQDKKAVNSSTIFELGSVSKLFTATAGGYAKNKGKISFDDTPGKYWKEL
ADC-30WP_001211218.1_301AbBCP	KKYEMYYGLQSVQDKKAVNSSTIFELGSVSKLFTATAGGYAKNKGKISFDDTPGKYWKEL
ADC-30WP_001211218.1_940AbBCP	KKYEMYYGLQSVQDKKAVNSSTIFELGSVSKLFTATAGGYAKNKGKISFDDTPGKYWKEL
DC-30WP_001211218.1_2949AbBCP	KKYEMYYGLQSVQDKKAVNSSTIFELGSVSKLFTATAGGYAKNKGKISFDDTPGKYWKEL
DC-30WP_001211218.1_1028AbBCP	KKYEMYYGLQSVQDKKAVNSSTIFELGSVSKLFTATAGGYAKNKGKISFDDTPGKYWKEL
DC-30WP_001211218.1_2526AbBCP	KKYEMYYGLQSVQDKKAVNSSTIFELGSVSKLFTATAGGYAKNKGKISFDDTPGKYWKEL
DC-30WP_001211218.1_1040AbBCP	KKYEMYYGLQSVQDKKAVNSSTIFELGSVSKLFTATAGGYAKNKGKISFDDTPGKYWKEL
DC-30WP_001211218.1_1089AbBCP	KKYEMYYGLQSVQDKKAVNSSTIFELGSVSKLFTATAGGYAKNKGKISFDDTPGKYWKEL
DC-30WP_001211218.1_1099AbBCP	KKYEMYYGLQSVQDKKAVNSSTIFELGSVSKLFTATAGGYAKNKGKISFDDTPGKYWKEL
DC-30WP_001211218.1_1206AbBCP	KKYEMYYGLQSVQDKKAVNSSTIFELGSVSKLFTATAGGYAKNKGKISFDDTPGKYWKEL
DC-30WP_001211218.1_1167AbBCP	KKYEMYYGLQSVQDKKAVNSSTIFELGSVSKLFTATAGGYAKNKGKISFDDTPGKYWKEL
DC-30WP_001211218.1_1186AbBCP	KKYEMYYGLQSVQDKKAVNSSTIFELGSVSKLFTATAGGYAKNKGKISFDDTPGKYWKEL
DC-30WP_001211218.1_1165AbBCP	KKYEMYYGLQSVQDKKAVNSSTIFELGSVSKLFTATAGGYAKNKGKISFDDTPGKYWKEL
DC-30WP_001211218.1_1077AbBCP	KKYEMYYGLQSVQDKKAVNSSTIFELGSVSKLFTATAGGYAKNKGKISFDDTPGKYWKEL
DC-30WP_001211218.1_1078AbBCP	KKYEMYYGLQSVQDKKAVNSSTIFELGSVSKLFTATAGGYAKNKGKISFDDTPGKYWKEL
DC-30WP_001211218.1_1071AbBCP	KKYEMYYGLQSVQDKKAVNSSTIFELGSVSKLFTATAGGYAKNKGKISFDDTPGKYWKEL
DC-30WP_001211218.1_2694AbBCP	KKYEMYYGLQSVQDKKAVNSSTIFELGSVSKLFTATAGGYAKNKGKISFDDTPGKYWKEL
DC-30WP_001211218.1_3340AbBCP	KKYEMYYGLQSVQDKKAVNSSTIFELGSVSKLFTATAGGYAKNKGKISFDDTPGKYWKEL
DC-30WP_001211218.1_1022AbBCP	KKYEMYYGLQSVQDKKAVNSSTIFELGSVSKLFTATAGGYAKNKGKISFDDTPGKYWKEL
DC-30WP_001211218.1_1025AbBCP	KKYEMYYGLQSVQDKKAVNSSTIFELGSVSKLFTATAGGYAKNKGKISFDDTPGKYWKEL
DC-30WP_001211218.1_1158AbBCP	KKYEMYYGLQSVQDKKAVNSSTIFELGSVSKLFTATAGGYAKNKGKISFDDTPGKYWKEL
DC-30WP_001211218.1_1020AbBCP	KKYEMYYGLQSVQDKKAVNSSTIFELGSVSKLFTATAGGYAKNKGKISFDDTPGKYWKEL
DC-30WP_001211218.1_1021AbBCP	KKYEMYYGLQSVQDKKAVNSSTIFELGSVSKLFTATAGGYAKNKGKISFDDTPGKYWKEL
ADC-30WP_001211218.1_532AbBCP	KKYEMYYGLQSVQDKKAVNSSTIFELGSVSKLFTATAGGYAKNKGKISFDDTPGKYWKEL
ADC-30WP_001211218.1_539AbBCP	KKYEMYYGLQSVQDKKAVNSSTIFELGSVSKLFTATAGGYAKNKGKISFDDTPGKYWKEL
DC-30WP_001211218.1_1033AbBCP	KKYEMYYGLQSVQDKKAVNSSTIFELGSVSKLFTATAGGYAKNKGKISFDDTPGKYWKEL
DC-30WP_001211218.1_1069AbBCP	KKYEMYYGLQSVQDKKAVNSSTIFELGSVSKLFTATAGGYAKNKGKISFDDTPGKYWKEL
DC-30WP_001211218.1_1029AbBCP	KKYEMYYGLQSVQDKKAVNSSTIFELGSVSKLFTATAGGYAKNKGKISFDDTPGKYWKEL
DC-30WP_001211218.1_1030AbBCP	KKYEMYYGLQSVQDKKAVNSSTIFELGSVSKLFTATAGGYAKNKGKISFDDTPGKYWKEL
DC-30WP_001211218.1_1096AbBCP	KKYEMYYGLQSVQDKKAVNSSTIFELGSVSKLFTATAGGYAKNKGKISFDDTPGKYWKEL
DC-30WP_001211218.1_1037AbBCP	KKYEMYYGLQSVQDKKAVNSSTIFELGSVSKLFTATAGGYAKNKGKISFDDTPGKYWKEL
DC-30WP_001211218.1_1098AbBCP	KKYEMYYGLQSVQDKKAVNSSTIFELGSVSKLFTATAGGYAKNKGKISFDDTPGKYWKEL
DC-30WP_001211218.1_1046AbBCP	KKYEMYYGLQSVQDKKAVNSSTIFELGSVSKLFTATAGGYAKNKGKISFDDTPGKYWKEL
DC-30WP_001211218.1_1032AbBCP	KKYEMYYGLQSVQDKKAVNSSTIFELGSVSKLFTATAGGYAKNKGKISFDDTPGKYWKEL
DC-30WP_001211218.1_1070AbBCP	KKYEMYYGLQSVQDKKAVNSSTIFELGSVSKLFTATAGGYAKNKGKISFDDTPGKYWKEL
DC-30WP_001211218.1_1139AbBCP	KKYEMYYGLQSVQDKKAVNSSTIFELGSVSKLFTATAGGYAKNKGKISFDDTPGKYWKEL
DC-30WP_001211218.1_1031AbBCP	KKYEMYYGLQSVQDKKAVNSSTIFELGSVSKLFTATAGGYAKNKGKISFDDTPGKYWKEL
DC-30WP_001211218.1_1066AbBCP	KKYEMYYGLQSVQDKKAVNSSTIFELGSVSKLFTATAGGYAKNKGKISFDDTPGKYWKEL
DC-30WP_001211218.1_1102AbBCP	KKYEMYYGLQSVQDKKAVNSSTIFELGSVSKLFTATAGGYAKNKGKISFDDTPGKYWKEL
ADC-30USX57838.1_2957AbBCP	KKYEMYYGLQSVQDKKAVNSSTIFELGSVSKLFTATAGGYAKNKGKISFDDTPGKYWKEL
ADC-30USX59985.1_1918AbBCP	KKYEMYYGLQSVQDKKAVNSSTIFELGSVSKLFTATAGGYAKNKGKISFDDTPGKYWKEL
ADC-30USX63799.1_2772AbBCP	KKYEMYYGLQSVQDKKAVNSSTIFELGSVSKLFTATAGGYAKNKGKISFDDTPGKYWKEL
ADC-30USX74012.1_305AbBCP	KKYEMYYGLQSVQDKKAVNSSTIFELGSVSKLFTATAGGYAKNKGKISFDDTPGKYWKEL
ADC-30USZ52468.1_2397AbBCP	KKYEMYYGLQSVQDKKAVNSSTIFELGSVSKLFTATAGGYAKNKGKISFDDTPGKYWKEL
DC-30WP_001211218.1_2008AbBCP	KKYEMYYGLQSVQDKKAVNSSTIFELGSVSKLFTATAGGYAKNKGKISFDDTPGKYWKEL
ADC-30WCL78976.1_1026AbBCP	KKYEMYYGLQSVQDKKAVNSSTIFELGSVSKLFTATAGGYAKNKGKISFDDTPGKYWKEL
ADC-30WDO27826.1_1034AbBCP	KKYEMYYGLQSVQDKKAVNSSTIFELGSVSKLFTATAGGYAKNKGKISFDDTPGKYWKEL
DC-30WP_001211218.1_2125AbBCP	KKYEMYYGLQSVQDKKAVNSSTIFELGSVSKLFTATAGGYAKNKGKISFDDTPGKYWKEL
DC-30WP_001211218.1_2729AbBCP	KKYEMYYGLQSVQDKKAVNSSTIFELGSVSKLFTATAGGYAKNKGKISFDDTPGKYWKEL
ADC-30ADX93237.1_2806AbBCP	KKYEMYYGLQSVQDKKAVNSSTIFELGSVSKLFTATAGGYAKNKGKISFDDTPGKYWKEL
ADC-82WP_001211216.1_2747AbOC	KKYEMYYGLQSVQDKKAVNSSTIFELGSVSKLFTATAGGYAKNKGKISFDDTPGKYWKEL
DC-82WP_001211216.1_1093AbBOC	KKYEMYYGLQSVQDKKAVNSSTIFELGSVSKLFTATAGGYAKNKGKISFDDTPGKYWKEL
DC-82WP_001211216.1_1071AbBOC	KKYEMYYGLQSVQDKKAVNSSTIFELGSVSKLFTATAGGYAKNKGKISFDDTPGKYWKEL
DC-82WP_001211216.1_1093AbBCP	KKYEMYYGLQSVQDKKAVNSSTIFELGSVSKLFTATAGGYAKNKGKISFDDTPGKYWKEL
DC-82WP_001211216.1_1071AbBCP	KKYEMYYGLQSVQDKKAVNSSTIFELGSVSKLFTATAGGYAKNKGKISFDDTPGKYWKEL
DC-162WP_031980335.1_1153AbOC	KKYEMYYGLQSVQDKKAVNSSTIFELGSVSKLFTATAGGYAKNKGKISFDDTPGKYWKEL
C-162WP_031980335.1_1028AbBOC	KKYEMYYGLQSVQDKKAVNSSTIFELGSVSKLFTATAGGYAKNKGKISFDDTPGKYWKEL
C-162WP_031980335.1_1023AbBOC	KKYEMYYGLQSVQDKKAVNSSTIFELGSVSKLFTATAGGYAKNKGKISFDDTPGKYWKEL
C-162WP_031980335.1_1028AbBCP	KKYEMYYGLQSVQDKKAVNSSTIFELGSVSKLFTATAGGYAKNKGKISFDDTPGKYWKEL
C-162WP_031980335.1_1023AbBCP	KKYEMYYGLQSVQDKKAVNSSTIFELGSVSKLFTATAGGYAKNKGKISFDDTPGKYWKEL
ADC-73WP_001211219.1_1012AbOC	KKYEMYYGLQSVQDKKAVNSSTIFELGSVSKLFTATAGGYAKNKGKISFDDTPGKYWKEL
ADC-73WP_001211219.1_1953AbOC	KKYEMYYGLQSVQDKKAVNSSTIFELGSVSKLFTATAGGYAKNKGKISFDDTPGKYWKEL
ADC-73WP_001211219.1_1014AbOC	KKYEMYYGLQSVQDKKAVNSSTIFELGSVSKLFTATAGGYAKNKGKISFDDTPGKYWKEL
ADC-73WP_001211219.1_1041AbOC	KKYEMYYGLQSVQDKKAVNSSTIFELGSVSKLFTATAGGYAKNKGKISFDDTPGKYWKEL
ADC-73WP_001211219.1_1028AbOC	KKYEMYYGLQSVQDKKAVNSSTIFELGSVSKLFTATAGGYAKNKGKISFDDTPGKYWKEL
ADC-73WP_001211219.1_2674AbOC	KKYEMYYGLQSVQDKKAVNSSTIFELGSVSKLFTATAGGYAKNKGKISFDDTPGKYWKEL
ADC-73WP_001211219.1_462AbOC	KKYEMYYGLQSVQDKKAVNSSTIFELGSVSKLFTATAGGYAKNKGKISFDDTPGKYWKEL
ADC-73WP_001211219.1_3530AbOC	KKYEMYYGLQSVQDKKAVNSSTIFELGSVSKLFTATAGGYAKNKGKISFDDTPGKYWKEL
ADC-73WP_001211219.1_3621AbOC	KKYEMYYGLQSVQDKKAVNSSTIFELGSVSKLFTATAGGYAKNKGKISFDDTPGKYWKEL
ADC-73WP_001211219.1_3381AbOC	KKYEMYYGLQSVQDKKAVNSSTIFELGSVSKLFTATAGGYAKNKGKISFDDTPGKYWKEL
ADC-73WP_001211219.1_2515AbOC	KKYEMYYGLQSVQDKKAVNSSTIFELGSVSKLFTATAGGYAKNKGKISFDDTPGKYWKEL
ADC-73WP_001211219.1_3574AbOC	KKYEMYYGLQSVQDKKAVNSSTIFELGSVSKLFTATAGGYAKNKGKISFDDTPGKYWKEL
ADC-73WP_001211219.1_1656AbOC	KKYEMYYGLQSVQDKKAVNSSTIFELGSVSKLFTATAGGYAKNKGKISFDDTPGKYWKEL
ADC-73WP_001211219.1_2591AbOC	KKYEMYYGLQSVQDKKAVNSSTIFELGSVSKLFTATAGGYAKNKGKISFDDTPGKYWKEL
ADC-73WP_001211219.1_2602AbOC	KKYEMYYGLQSVQDKKAVNSSTIFELGSVSKLFTATAGGYAKNKGKISFDDTPGKYWKEL
ADC-73WP_001211219.1_910AbOC	KKYEMYYGLQSVQDKKAVNSSTIFELGSVSKLFTATAGGYAKNKGKISFDDTPGKYWKEL
ADC-73WP_001211219.1_394AbOC	KKYEMYYGLQSVQDKKAVNSSTIFELGSVSKLFTATAGGYAKNKGKISFDDTPGKYWKEL
ADC-73WP_001211219.1_1030AbOC	KKYEMYYGLQSVQDKKAVNSSTIFELGSVSKLFTATAGGYAKNKGKISFDDTPGKYWKEL
ADC-73WP_001211219.1_1023AbOC	KKYEMYYGLQSVQDKKAVNSSTIFELGSVSKLFTATAGGYAKNKGKISFDDTPGKYWKEL
ADC-73UYQ66995.1_1967AbOC	KKYEMYYGLQSVQDKKAVNSSTIFELGSVSKLFTATAGGYAKNKGKISFDDTPGKYWKEL
ADC-73WP_001211219.1_906AbBOC	KKYEMYYGLQSVQDKKAVNSSTIFELGSVSKLFTATAGGYAKNKGKISFDDTPGKYWKEL
DC-73WP_001211219.1_2649AbBOC	KKYEMYYGLQSVQDKKAVNSSTIFELGSVSKLFTATAGGYAKNKGKISFDDTPGKYWKEL
DC-73WP_001211219.1_2693AbBOC	KKYEMYYGLQSVQDKKAVNSSTIFELGSVSKLFTATAGGYAKNKGKISFDDTPGKYWKEL
ADC-73AQU57925.1_2703AbBOC	KKYEMYYGLQSVQDKKAVNSSTIFELGSVSKLFTATAGGYAKNKGKISFDDTPGKYWKEL
DC-73WP_001211219.1_1026AbBOC	KKYEMYYGLQSVQDKKAVNSSTIFELGSVSKLFTATAGGYAKNKGKISFDDTPGKYWKEL
DC-73WP_001211219.1_1019AbBOC	KKYEMYYGLQSVQDKKAVNSSTIFELGSVSKLFTATAGGYAKNKGKISFDDTPGKYWKEL
DC-73WP_001211219.1_1024AbBOC	KKYEMYYGLQSVQDKKAVNSSTIFELGSVSKLFTATAGGYAKNKGKISFDDTPGKYWKEL
ADC-73WP_001211219.1_930AbBOC	KKYEMYYGLQSVQDKKAVNSSTIFELGSVSKLFTATAGGYAKNKGKISFDDTPGKYWKEL
DC-73WP_001211219.1_2609AbBOC	KKYEMYYGLQSVQDKKAVNSSTIFELGSVSKLFTATAGGYAKNKGKISFDDTPGKYWKEL
DC-73WP_001211219.1_3456AbBOC	KKYEMYYGLQSVQDKKAVNSSTIFELGSVSKLFTATAGGYAKNKGKISFDDTPGKYWKEL
DC-73WP_001211219.1_2614AbBOC	KKYEMYYGLQSVQDKKAVNSSTIFELGSVSKLFTATAGGYAKNKGKISFDDTPGKYWKEL
DC-73WP_001211219.1_1025AbBOC	KKYEMYYGLQSVQDKKAVNSSTIFELGSVSKLFTATAGGYAKNKGKISFDDTPGKYWKEL
DC-73WP_001211219.1_1030AbBOC	KKYEMYYGLQSVQDKKAVNSSTIFELGSVSKLFTATAGGYAKNKGKISFDDTPGKYWKEL
DC-73WP_001211219.1_1173AbBOC	KKYEMYYGLQSVQDKKAVNSSTIFELGSVSKLFTATAGGYAKNKGKISFDDTPGKYWKEL
DC-73WP_001211219.1_1021AbBOC	KKYEMYYGLQSVQDKKAVNSSTIFELGSVSKLFTATAGGYAKNKGKISFDDTPGKYWKEL
DC-73WP_001211219.1_2449AbBOC	KKYEMYYGLQSVQDKKAVNSSTIFELGSVSKLFTATAGGYAKNKGKISFDDTPGKYWKEL
DC-73WP_001211219.1_1176AbBOC	KKYEMYYGLQSVQDKKAVNSSTIFELGSVSKLFTATAGGYAKNKGKISFDDTPGKYWKEL
DC-73WP_001211219.1_1161AbBOC	KKYEMYYGLQSVQDKKAVNSSTIFELGSVSKLFTATAGGYAKNKGKISFDDTPGKYWKEL
DC-73WP_001211219.1_1070AbBOC	KKYEMYYGLQSVQDKKAVNSSTIFELGSVSKLFTATAGGYAKNKGKISFDDTPGKYWKEL
DC-73WP_001211219.1_1079AbBOC	KKYEMYYGLQSVQDKKAVNSSTIFELGSVSKLFTATAGGYAKNKGKISFDDTPGKYWKEL
DC-73WP_001211219.1_1078AbBOC	KKYEMYYGLQSVQDKKAVNSSTIFELGSVSKLFTATAGGYAKNKGKISFDDTPGKYWKEL
DC-73WP_001211219.1_1037AbBOC	KKYEMYYGLQSVQDKKAVNSSTIFELGSVSKLFTATAGGYAKNKGKISFDDTPGKYWKEL
DC-73WP_001211219.1_1035AbBOC	KKYEMYYGLQSVQDKKAVNSSTIFELGSVSKLFTATAGGYAKNKGKISFDDTPGKYWKEL
DC-73WP_001211219.1_1033AbBOC	KKYEMYYGLQSVQDKKAVNSSTIFELGSVSKLFTATAGGYAKNKGKISFDDTPGKYWKEL
DC-73WP_001211219.1_1041AbBOC	KKYEMYYGLQSVQDKKAVNSSTIFELGSVSKLFTATAGGYAKNKGKISFDDTPGKYWKEL
DC-73WP_001211219.1_1023AbBOC	KKYEMYYGLQSVQDKKAVNSSTIFELGSVSKLFTATAGGYAKNKGKISFDDTPGKYWKEL
ADC-73USX69547.1_2386AbBOC	KKYEMYYGLQSVQDKKAVNSSTIFELGSVSKLFTATAGGYAKNKGKISFDDTPGKYWKEL
DC-73WP_001211219.1_1034AbBOC	KKYEMYYGLQSVQDKKAVNSSTIFELGSVSKLFTATAGGYAKNKGKISFDDTPGKYWKEL
ADC-73WP_001211219.1_852AbBOC	KKYEMYYGLQSVQDKKAVNSSTIFELGSVSKLFTATAGGYAKNKGKISFDDTPGKYWKEL
DC-73WP_001211219.1_3070AbBOC	KKYEMYYGLQSVQDKKAVNSSTIFELGSVSKLFTATAGGYAKNKGKISFDDTPGKYWKEL
ADC-73WBS60953.1_1026AbBOC	KKYEMYYGLQSVQDKKAVNSSTIFELGSVSKLFTATAGGYAKNKGKISFDDTPGKYWKEL
ADC-73WDC39660.1_1023AbBOC	KKYEMYYGLQSVQDKKAVNSSTIFELGSVSKLFTATAGGYAKNKGKISFDDTPGKYWKEL
ADC-73WDC73972.1_1415AbBOC	KKYEMYYGLQSVQDKKAVNSSTIFELGSVSKLFTATAGGYAKNKGKISFDDTPGKYWKEL
ADC-73WDC70172.1_1037AbBOC	KKYEMYYGLQSVQDKKAVNSSTIFELGSVSKLFTATAGGYAKNKGKISFDDTPGKYWKEL
DC-73WP_001211219.1_1101AbBOC	KKYEMYYGLQSVQDKKAVNSSTIFELGSVSKLFTATAGGYAKNKGKISFDDTPGKYWKEL
DC-73WP_001211219.1_1667AbBOC	KKYEMYYGLQSVQDKKAVNSSTIFELGSVSKLFTATAGGYAKNKGKISFDDTPGKYWKEL
ADC-73WP_001211219.1_287AbBOC	KKYEMYYGLQSVQDKKAVNSSTIFELGSVSKLFTATAGGYAKNKGKISFDDTPGKYWKEL
DC-73WP_001211219.1_3177AbBOC	KKYEMYYGLQSVQDKKAVNSSTIFELGSVSKLFTATAGGYAKNKGKISFDDTPGKYWKEL
DC-73WP_001211219.1_2501AbBOC	KKYEMYYGLQSVQDKKAVNSSTIFELGSVSKLFTATAGGYAKNKGKISFDDTPGKYWKEL
DC-73WP_001211219.1_2535AbBOC	KKYEMYYGLQSVQDKKAVNSSTIFELGSVSKLFTATAGGYAKNKGKISFDDTPGKYWKEL
DC-73WP_001211219.1_3150AbBOC	KKYEMYYGLQSVQDKKAVNSSTIFELGSVSKLFTATAGGYAKNKGKISFDDTPGKYWKEL
DC-73WP_001211219.1_3714AbBOC	KKYEMYYGLQSVQDKKAVNSSTIFELGSVSKLFTATAGGYAKNKGKISFDDTPGKYWKEL
ADC-73WP_001211219.1_722AbBOC	KKYEMYYGLQSVQDKKAVNSSTIFELGSVSKLFTATAGGYAKNKGKISFDDTPGKYWKEL
DC-73WP_001211219.1_3650AbBOC	KKYEMYYGLQSVQDKKAVNSSTIFELGSVSKLFTATAGGYAKNKGKISFDDTPGKYWKEL
ADC-73WP_001211219.1_568AbBOC	KKYEMYYGLQSVQDKKAVNSSTIFELGSVSKLFTATAGGYAKNKGKISFDDTPGKYWKEL
DC-73WP_001211219.1_3226AbBOC	KKYEMYYGLQSVQDKKAVNSSTIFELGSVSKLFTATAGGYAKNKGKISFDDTPGKYWKEL
ADC-73WP_001211219.1_556AbBOC	KKYEMYYGLQSVQDKKAVNSSTIFELGSVSKLFTATAGGYAKNKGKISFDDTPGKYWKEL

```
DC-73WP_001211219.1_2436AbBOC   KKYEMYYGLQSVQDKKAVNSSTIFELGSVSKLFTATAGGYAKNKGKISFDDTPGKYWKEL
DC-73WP_001211219.1_1029AbBOC   KKYEMYYGLQSVQDKKAVNSSTIFELGSVSKLFTATAGGYAKNKGKISFDDTPGKYWKEL
DC-73WP_001211219.1_1013AbBOC   KKYEMYYGLQSVQDKKAVNSSTIFELGSVSKLFTATAGGYAKNKGKISFDDTPGKYWKEL
ADC-73WP_001211219.1_906AbBCP   KKYEMYYGLQSVQDKKAVNSSTIFELGSVSKLFTATAGGYAKNKGKISFDDTPGKYWKEL
DC-73WP_001211219.1_2649AbBCP   KKYEMYYGLQSVQDKKAVNSSTIFELGSVSKLFTATAGGYAKNKGKISFDDTPGKYWKEL
DC-73WP_001211219.1_2693AbBCP   KKYEMYYGLQSVQDKKAVNSSTIFELGSVSKLFTATAGGYAKNKGKISFDDTPGKYWKEL
ADC-73AQU57925.1_2703AbBCP      KKYEMYYGLQSVQDKKAVNSSTIFELGSVSKLFTATAGGYAKNKGKISFDDTPGKYWKEL
DC-73WP_001211219.1_1026AbBCP   KKYEMYYGLQSVQDKKAVNSSTIFELGSVSKLFTATAGGYAKNKGKISFDDTPGKYWKEL
DC-73WP_001211219.1_1019AbBCP   KKYEMYYGLQSVQDKKAVNSSTIFELGSVSKLFTATAGGYAKNKGKISFDDTPGKYWKEL
DC-73WP_001211219.1_1024AbBCP   KKYEMYYGLQSVQDKKAVNSSTIFELGSVSKLFTATAGGYAKNKGKISFDDTPGKYWKEL
ADC-73WP_001211219.1_930AbBCP   KKYEMYYGLQSVQDKKAVNSSTIFELGSVSKLFTATAGGYAKNKGKISFDDTPGKYWKEL
DC-73WP_001211219.1_2609AbBCP   KKYEMYYGLQSVQDKKAVNSSTIFELGSVSKLFTATAGGYAKNKGKISFDDTPGKYWKEL
DC-73WP_001211219.1_3456AbBCP   KKYEMYYGLQSVQDKKAVNSSTIFELGSVSKLFTATAGGYAKNKGKISFDDTPGKYWKEL
DC-73WP_001211219.1_2614AbBCP   KKYEMYYGLQSVQDKKAVNSSTIFELGSVSKLFTATAGGYAKNKGKISFDDTPGKYWKEL
DC-73WP_001211219.1_1025AbBCP   KKYEMYYGLQSVQDKKAVNSSTIFELGSVSKLFTATAGGYAKNKGKISFDDTPGKYWKEL
DC-73WP_001211219.1_1030AbBCP   KKYEMYYGLQSVQDKKAVNSSTIFELGSVSKLFTATAGGYAKNKGKISFDDTPGKYWKEL
DC-73WP_001211219.1_1173AbBCP   KKYEMYYGLQSVQDKKAVNSSTIFELGSVSKLFTATAGGYAKNKGKISFDDTPGKYWKEL
DC-73WP_001211219.1_1021AbBCP   KKYEMYYGLQSVQDKKAVNSSTIFELGSVSKLFTATAGGYAKNKGKISFDDTPGKYWKEL
DC-73WP_001211219.1_2449AbBCP   KKYEMYYGLQSVQDKKAVNSSTIFELGSVSKLFTATAGGYAKNKGKISFDDTPGKYWKEL
DC-73WP_001211219.1_1176AbBCP   KKYEMYYGLQSVQDKKAVNSSTIFELGSVSKLFTATAGGYAKNKGKISFDDTPGKYWKEL
DC-73WP_001211219.1_1161AbBCP   KKYEMYYGLQSVQDKKAVNSSTIFELGSVSKLFTATAGGYAKNKGKISFDDTPGKYWKEL
DC-73WP_001211219.1_1070AbBCP   KKYEMYYGLQSVQDKKAVNSSTIFELGSVSKLFTATAGGYAKNKGKISFDDTPGKYWKEL
DC-73WP_001211219.1_1079AbBCP   KKYEMYYGLQSVQDKKAVNSSTIFELGSVSKLFTATAGGYAKNKGKISFDDTPGKYWKEL
DC-73WP_001211219.1_1078AbBCP   KKYEMYYGLQSVQDKKAVNSSTIFELGSVSKLFTATAGGYAKNKGKISFDDTPGKYWKEL
DC-73WP_001211219.1_1037AbBCP   KKYEMYYGLQSVQDKKAVNSSTIFELGSVSKLFTATAGGYAKNKGKISFDDTPGKYWKEL
DC-73WP_001211219.1_1035AbBCP   KKYEMYYGLQSVQDKKAVNSSTIFELGSVSKLFTATAGGYAKNKGKISFDDTPGKYWKEL
DC-73WP_001211219.1_1033AbBCP   KKYEMYYGLQSVQDKKAVNSSTIFELGSVSKLFTATAGGYAKNKGKISFDDTPGKYWKEL
DC-73WP_001211219.1_1041AbBCP   KKYEMYYGLQSVQDKKAVNSSTIFELGSVSKLFTATAGGYAKNKGKISFDDTPGKYWKEL
DC-73WP_001211219.1_1023AbBCP   KKYEMYYGLQSVQDKKAVNSSTIFELGSVSKLFTATAGGYAKNKGKISFDDTPGKYWKEL
ADC-73USX69547.1_2386AbBCP      KKYEMYYGLQSVQDKKAVNSSTIFELGSVSKLFTATAGGYAKNKGKISFDDTPGKYWKEL
DC-73WP_001211219.1_1034AbBCP   KKYEMYYGLQSVQDKKAVNSSTIFELGSVSKLFTATAGGYAKNKGKISFDDTPGKYWKEL
ADC-73WP_001211219.1_852AbBCP   KKYEMYYGLQSVQDKKAVNSSTIFELGSVSKLFTATAGGYAKNKGKISFDDTPGKYWKEL
DC-73WP_001211219.1_3070AbBCP   KKYEMYYGLQSVQDKKAVNSSTIFELGSVSKLFTATAGGYAKNKGKISFDDTPGKYWKEL
ADC-73WBS60953.1_1026AbBCP      KKYEMYYGLQSVQDKKAVNSSTIFELGSVSKLFTATAGGYAKNKGKISFDDTPGKYWKEL
ADC-73WDC39660.1_1023AbBCP      KKYEMYYGLQSVQDKKAVNSSTIFELGSVSKLFTATAGGYAKNKGKISFDDTPGKYWKEL
ADC-73WDC73972.1_1415AbBCP      KKYEMYYGLQSVQDKKAVNSSTIFELGSVSKLFTATAGGYAKNKGKISFDDTPGKYWKEL
ADC-73WDC70172.1_1037AbBCP      KKYEMYYGLQSVQDKKAVNSSTIFELGSVSKLFTATAGGYAKNKGKISFDDTPGKYWKEL
DC-73WP_001211219.1_1101AbBCP   KKYEMYYGLQSVQDKKAVNSSTIFELGSVSKLFTATAGGYAKNKGKISFDDTPGKYWKEL
DC-73WP_001211219.1_1667AbBCP   KKYEMYYGLQSVQDKKAVNSSTIFELGSVSKLFTATAGGYAKNKGKISFDDTPGKYWKEL
ADC-73WP_001211219.1_287AbBCP   KKYEMYYGLQSVQDKKAVNSSTIFELGSVSKLFTATAGGYAKNKGKISFDDTPGKYWKEL
DC-73WP_001211219.1_3177AbBCP   KKYEMYYGLQSVQDKKAVNSSTIFELGSVSKLFTATAGGYAKNKGKISFDDTPGKYWKEL
DC-73WP_001211219.1_2501AbBCP   KKYEMYYGLQSVQDKKAVNSSTIFELGSVSKLFTATAGGYAKNKGKISFDDTPGKYWKEL
DC-73WP_001211219.1_2535AbBCP   KKYEMYYGLQSVQDKKAVNSSTIFELGSVSKLFTATAGGYAKNKGKISFDDTPGKYWKEL
DC-73WP_001211219.1_3150AbBCP   KKYEMYYGLQSVQDKKAVNSSTIFELGSVSKLFTATAGGYAKNKGKISFDDTPGKYWKEL
DC-73WP_001211219.1_3714AbBCP   KKYEMYYGLQSVQDKKAVNSSTIFELGSVSKLFTATAGGYAKNKGKISFDDTPGKYWKEL
ADC-73WP_001211219.1_722AbBCP   KKYEMYYGLQSVQDKKAVNSSTIFELGSVSKLFTATAGGYAKNKGKISFDDTPGKYWKEL
DC-73WP_001211219.1_3650AbBCP   KKYEMYYGLQSVQDKKAVNSSTIFELGSVSKLFTATAGGYAKNKGKISFDDTPGKYWKEL
ADC-73WP_001211219.1_568AbBCP   KKYEMYYGLQSVQDKKAVNSSTIFELGSVSKLFTATAGGYAKNKGKISFDDTPGKYWKEL
DC-73WP_001211219.1_3226AbBCP   KKYEMYYGLQSVQDKKAVNSSTIFELGSVSKLFTATAGGYAKNKGKISFDDTPGKYWKEL
ADC-73WP_001211219.1_556AbBCP   KKYEMYYGLQSVQDKKAVNSSTIFELGSVSKLFTATAGGYAKNKGKISFDDTPGKYWKEL
DC-73WP_001211219.1_2436AbBCP   KKYEMYYGLQSVQDKKAVNSSTIFELGSVSKLFTATAGGYAKNKGKISFDDTPGKYWKEL
DC-73WP_001211219.1_1029AbBCP   KKYEMYYGLQSVQDKKAVNSSTIFELGSVSKLFTATAGGYAKNKGKISFDDTPGKYWKEL
DC-73WP_001211219.1_1013AbBCP   KKYEMYYGLQSVQDKKAVNSSTIFELGSVSKLFTATAGGYAKNKGKISFDDTPGKYWKEL
ADC-73BDE18748.1_1038AbBCP      KKYEMYYGLQSVQDKKAVNSSTIFELGSVSKLFTATAGGYAKNKGKISFDDTPGKYWKEL
DC-115WP_001211215.1_2833AbOC   KKYEMYYGLQSVQDKKAVNSSTIFELGSVSKLFTATAGGYAKNKGKISFDDTPGKYWKEL
C-115WP_001211215.1_1156AbBOC   KKYEMYYGLQSVQDKKAVNSSTIFELGSVSKLFTATAGGYAKNKGKISFDDTPGKYWKEL
C-115WP_001211215.1_1156AbBCP   KKYEMYYGLQSVQDKKAVNSSTIFELGSVSKLFTATAGGYAKNKGKISFDDTPGKYWKEL
ADC-5WP_038405930.1_2542AbOC    KKYEMYYGLQSVQDKKAVNSSTIFELGSVSKLFTATAGGYAKNKGKISFDDTPGKYWKEL
ADC-5WP_038405930.1_1113AbBOC   KKYEMYYGLQSVQDKKAVNSSTIFELGSVSKLFTATAGGYAKNKGKISFDDTPGKYWKEL
ADC-5WP_038405930.1_1113AbBCP   KKYEMYYGLQSVQDKKAVNSSTIFELGSVSKLFTATAGGYAKNKGKISFDDTPGKYWKEL
C-236WP_176673601.1_1026AbBOC   KKYEMYYGLQSVQDKKAVNSSTIFELGSVSKLFTATAGGYAKNKGKISFDDTPGKYWKEL
C-236WP_176673601.1_1026AbBCP   KKYEMYYGLQSVQDKKAVNSSTIFELGSVSKLFTATAGGYAKNKGKISFDDTPGKYWKEL
DC-217WP_060470109.1_3584AbOC   KKYEMYYGLQSVQDKKAVNSSTIFELGSVSKLFTATAGGYAKNKGKISFDDTPGKYWKEL
ADC-217USX66614.1_2290AbBOC    KKYEMYYGLQSVQDKKAVNSSTIFELGSVSKLFTATAGGYAKNKGKISFDDTPGKYWKEL
ADC-217USX66614.1_2290AbBCP    KKYEMYYGLQSVQDKKAVNSSTIFELGSVSKLFTATAGGYAKNKGKISFDDTPGKYWKEL
ADC-247WP_032058755.1_382AbOC   KKYEMYYGLQSVQDKKAVNSSTIFELGSVSKLFTATAGGYAKNKGKISFDDTPGKYWKEL
C-247WP_032058755.1_1936AbBOC   KKYEMYYGLQSVQDKKAVNSSTIFELGSVSKLFTATAGGYAKNKGKISFDDTPGKYWKEL
C-247WP_032058755.1_1936AbBCP   KKYEMYYGLQSVQDKKAVNSSTIFELGSVSKLFTATAGGYAKNKGKISFDDTPGKYWKEL
DC-203WP_032000414.1_2940AbOC   KKYEMYYGLQSVQDKKAVNSSTIFELGSVSKLFTATAGGYAKNKGKISFDDTPGKYWKEL
C-203WP_032000414.1_1062AbBOC   KKYEMYYGLQSVQDKKAVNSSTIFELGSVSKLFTATAGGYAKNKGKISFDDTPGKYWKEL
DC-203WP_032000414.1_129AbBOC   KKYEMYYGLQSVQDKKAVNSSTIFELGSVSKLFTATAGGYAKNKGKISFDDTPGKYWKEL
C-203WP_032000414.1_2518AbBOC   KKYEMYYGLQSVQDKKAVNSSTIFELGSVSKLFTATAGGYAKNKGKISFDDTPGKYWKEL
C-203WP_032000414.1_1062AbBCP   KKYEMYYGLQSVQDKKAVNSSTIFELGSVSKLFTATAGGYAKNKGKISFDDTPGKYWKEL
DC-203WP_032000414.1_129AbBCP   KKYEMYYGLQSVQDKKAVNSSTIFELGSVSKLFTATAGGYAKNKGKISFDDTPGKYWKEL
C-203WP_032000414.1_2518AbBCP   KKYEMYYGLQSVQDKKAVNSSTIFELGSVSKLFTATAGGYAKNKGKISFDDTPGKYWKEL
DC-152WP_001211233.1_3734AbOC   KKYEMYYGLQSVQDKKAVNSSTIFELGSVSKLFTATAGGYAKNKGKISFDDTPGKYWKEL
C-152WP_001211233.1_1080AbBOC   KKYEMYYGLQSVQDKKAVNSSTIFELGSVSKLFTATAGGYAKNKGKISFDDTPGKYWKEL
DC-152WP_001211233.1_491AbBOC   KKYEMYYGLQSVQDKKAVNSSTIFELGSVSKLFTATAGGYAKNKGKISFDDTPGKYWKEL
ADC-152WP_001211233.1_46AbBOC   KKYEMYYGLQSVQDKKAVNSSTIFELGSVSKLFTATAGGYAKNKGKISFDDTPGKYWKEL
C-152WP_001211233.1_1545AbBOC   KKYEMYYGLQSVQDKKAVNSSTIFELGSVSKLFTATAGGYAKNKGKISFDDTPGKYWKEL
ADC-152WBS86980.1_1289AbBOC    KKYEMYYGLQSVQDKKAVNSSTIFELGSVSKLFTATAGGYAKNKGKISFDDTPGKYWKEL
ADC-152WBS81072.1_2581AbBOC    KKYEMYYGLQSVQDKKAVNSSTIFELGSVSKLFTATAGGYAKNKGKISFDDTPGKYWKEL
ADC-152WBS88504.1_2711AbBOC    KKYEMYYGLQSVQDKKAVNSSTIFELGSVSKLFTATAGGYAKNKGKISFDDTPGKYWKEL
ADC-152WBS92168.1_2401AbBOC    KKYEMYYGLQSVQDKKAVNSSTIFELGSVSKLFTATAGGYAKNKGKISFDDTPGKYWKEL
ADC-152WBS79617.1_1289AbBOC    KKYEMYYGLQSVQDKKAVNSSTIFELGSVSKLFTATAGGYAKNKGKISFDDTPGKYWKEL
C-152WP_001211233.1_1080AbBCP   KKYEMYYGLQSVQDKKAVNSSTIFELGSVSKLFTATAGGYAKNKGKISFDDTPGKYWKEL
DC-152WP_001211233.1_491AbBCP   KKYEMYYGLQSVQDKKAVNSSTIFELGSVSKLFTATAGGYAKNKGKISFDDTPGKYWKEL
ADC-152WP_001211233.1_46AbBCP   KKYEMYYGLQSVQDKKAVNSSTIFELGSVSKLFTATAGGYAKNKGKISFDDTPGKYWKEL
C-152WP_001211233.1_1545AbBCP   KKYEMYYGLQSVQDKKAVNSSTIFELGSVSKLFTATAGGYAKNKGKISFDDTPGKYWKEL
ADC-152WBS86980.1_1289AbBCP    KKYEMYYGLQSVQDKKAVNSSTIFELGSVSKLFTATAGGYAKNKGKISFDDTPGKYWKEL
ADC-152WBS81072.1_2581AbBCP    KKYEMYYGLQSVQDKKAVNSSTIFELGSVSKLFTATAGGYAKNKGKISFDDTPGKYWKEL
ADC-152WBS88504.1_2711AbBCP    KKYEMYYGLQSVQDKKAVNSSTIFELGSVSKLFTATAGGYAKNKGKISFDDTPGKYWKEL
ADC-152WBS92168.1_2401AbBCP    KKYEMYYGLQSVQDKKAVNSSTIFELGSVSKLFTATAGGYAKNKGKISFDDTPGKYWKEL
ADC-152WBS79617.1_1289AbBCP    KKYEMYYGLQSVQDKKAVNSSTIFELGSVSKLFTATAGGYAKNKGKISFDDTPGKYWKEL
ADC-52WP_001211232.1_990AbOC    KKYEMYYGLQSVQDKKAVNSSTIFELGSVSKLFTATAGGYAKNKGKISFDDTPGKYWKEL
DC-52WP_001211232.1_1087AbBOC   KKYEMYYGLQSVQDKKAVNSSTIFELGSVSKLFTATAGGYAKNKGKISFDDTPGKYWKEL
DC-52WP_001211232.1_1087AbBCP   KKYEMYYGLQSVQDKKAVNSSTIFELGSVSKLFTATAGGYAKNKGKISFDDTPGKYWKEL
C-163WP_057093064.1_2397AbBOC   KKYEMYYGLQSVQDKKAVNSSTIFELGSVSKLFTATAGGYAKNKGKISFDDTPGKYWKEL
C-163WP_057093064.1_2397AbBCP   KKYEMYYGLQSVQDKKAVNSSTIFELGSVSKLFTATAGGYAKNKGKISFDDTPGKYWKEL
ADC-33WP_001211220.1_2277AbOC   KKYEMYYGLQSVQDKKAVNSSTIFELGSVSKLFTATAGGYAKNKGKISFDDTPGKYWKEL
DC-33WP_001211220.1_1044AbBOC   KKYEMYYGLQSVQDKKAVNSSTIFELGSVSKLFTATAGGYAKNKGKISFDDTPGKYWKEL
DC-33WP_001211220.1_2452AbBOC   KKYEMYYGLQSVQDKKAVNSSTIFELGSVSKLFTATAGGYAKNKGKISFDDTPGKYWKEL
DC-33WP_001211220.1_1046AbBOC   KKYEMYYGLQSVQDKKAVNSSTIFELGSVSKLFTATAGGYAKNKGKISFDDTPGKYWKEL
DC-33WP_001211220.1_1025AbBOC   KKYEMYYGLQSVQDKKAVNSSTIFELGSVSKLFTATAGGYAKNKGKISFDDTPGKYWKEL
DC-33WP_001211220.1_1026AbBOC   KKYEMYYGLQSVQDKKAVNSSTIFELGSVSKLFTATAGGYAKNKGKISFDDTPGKYWKEL
DC-33WP_001211220.1_1033AbBOC   KKYEMYYGLQSVQDKKAVNSSTIFELGSVSKLFTATAGGYAKNKGKISFDDTPGKYWKEL
DC-33WP_001211220.1_1035AbBOC   KKYEMYYGLQSVQDKKAVNSSTIFELGSVSKLFTATAGGYAKNKGKISFDDTPGKYWKEL
DC-33WP_001211220.1_1041AbBOC   KKYEMYYGLQSVQDKKAVNSSTIFELGSVSKLFTATAGGYAKNKGKISFDDTPGKYWKEL
DC-33WP_001211220.1_1044AbBCP   KKYEMYYGLQSVQDKKAVNSSTIFELGSVSKLFTATAGGYAKNKGKISFDDTPGKYWKEL
DC-33WP_001211220.1_2452AbBCP   KKYEMYYGLQSVQDKKAVNSSTIFELGSVSKLFTATAGGYAKNKGKISFDDTPGKYWKEL
DC-33WP_001211220.1_1046AbBCP   KKYEMYYGLQSVQDKKAVNSSTIFELGSVSKLFTATAGGYAKNKGKISFDDTPGKYWKEL
DC-33WP_001211220.1_1025AbBCP   KKYEMYYGLQSVQDKKAVNSSTIFELGSVSKLFTATAGGYAKNKGKISFDDTPGKYWKEL
DC-33WP_001211220.1_1026AbBCP   KKYEMYYGLQSVQDKKAVNSSTIFELGSVSKLFTATAGGYAKNKGKISFDDTPGKYWKEL
DC-33WP_001211220.1_1033AbBCP   KKYEMYYGLQSVQDKKAVNSSTIFELGSVSKLFTATAGGYAKNKGKISFDDTPGKYWKEL
DC-33WP_001211220.1_1035AbBCP   KKYEMYYGLQSVQDKKAVNSSTIFELGSVSKLFTATAGGYAKNKGKISFDDTPGKYWKEL
DC-33WP_001211220.1_1041AbBCP   KKYEMYYGLQSVQDKKAVNSSTIFELGSVSKLFTATAGGYAKNKGKISFDDTPGKYWKEL
C-212WP_031975357.1_1297AbBOC   KKYEMYYGLQSVQDKKAVNSSTIFELGSVSKLFTATAGGYAKNKGKISFDDTPGKYWKEL
DC-212WP_031975357.1_501AbBOC   KKYEMYYGLQSVQDKKAVNSSTIFELGSVSKLFTATAGGYAKNKGKISFDDTPGKYWKEL
C-212WP_031975357.1_1297AbBCP   KKYEMYYGLQSVQDKKAVNSSTIFELGSVSKLFTATAGGYAKNKGKISFDDTPGKYWKEL
DC-212WP_031975357.1_501AbBCP   KKYEMYYGLQSVQDKKAVNSSTIFELGSVSKLFTATAGGYAKNKGKISFDDTPGKYWKEL
C-182WP_057691114.1_1055AbBOC   KKYEMYYGLQSVQDKKAVNSSTIFELGSVSKLFTATAGGYAKNKGKISFDDTPGKYWKEL
C-182WP_057691114.1_1055AbBCP   KKYEMYYGLQSVQDKKAVNSSTIFELGSVSKLFTATAGGYAKNKGKISFDDTPGKYWKEL
DC-81WP_059262723.1_1208AbBOC   KKYEMYYGLQSVQDKKAVNRSTIFELGSVSKLFTATAGGYAKNKGKISFDDTPGKYWKEL
DC-81WP_059262723.1_2841AbBOC   KKYEMYYGLQSVQDKKAVNRSTIFELGSVSKLFTATAGGYAKNKGKISFDDTPGKYWKEL
DC-81WP_059262723.1_1208AbBCP   KKYEMYYGLQSVQDKKAVNRSTIFELGSVSKLFTATAGGYAKNKGKISFDDTPGKYWKEL
DC-81WP_059262723.1_2841AbBCP   KKYEMYYGLQSVQDKKAVNRSTIFELGSVSKLFTATAGGYAKNKGKISFDDTPGKYWKEL
ADC-176WP_001159761.1_11AbOC    KKYEMYYGLQSVQDKKAVNSSTIFELGSVSKLFTATAGGYAKNKGKISFDDTPGKYWKEL
DC-176WP_001159761.1_3466AbOC   KKYEMYYGLQSVQDKKAVNSSTIFELGSVSKLFTATAGGYAKNKGKISFDDTPGKYWKEL
ADC-176WP_001159761.1_93AbBOP   KKYEMYYGLQSVQDKKAVNSSTIFELGSVSKLFTATAGGYAKNKGKISFDDTPGKYWKEL
DC-176WP_001159761.1_247AbBOC   KKYEMYYGLQSVQDKKAVNSSTIFELGSVSKLFTATAGGYAKNKGKISFDDTPGKYWKEL
ADC-176WP_001159761.1_9AbBOC    KKYEMYYGLQSVQDKKAVNSSTIFELGSVSKLFTATAGGYAKNKGKISFDDTPGKYWKEL
C-176WP_001159761.1_2982AbBOC   KKYEMYYGLQSVQDKKAVNSSTIFELGSVSKLFTATAGGYAKNKGKISFDDTPGKYWKEL
ADC-176QLG80706.1_1971AbBOC    KKYEMYYGLQSVQDKKAVNSSTIFELGSVSKLFTATAGGYAKNKGKISFDDTPGKYWKEL
C-176WP_001159761.1_2851AbBOC   KKYEMYYGLQSVQDKKAVNSSTIFELGSVSKLFTATAGGYAKNKGKISFDDTPGKYWKEL
C-176WP_001159761.1_1220AbBOC   KKYEMYYGLQSVQDKKAVNSSTIFELGSVSKLFTATAGGYAKNKGKISFDDTPGKYWKEL
DC-176WP_001159761.1_386AbBOC   KKYEMYYGLQSVQDKKAVNSSTIFELGSVSKLFTATAGGYAKNKGKISFDDTPGKYWKEL
ADC-176WP_001159761.1_11AbBOC   KKYEMYYGLQSVQDKKAVNSSTIFELGSVSKLFTATAGGYAKNKGKISFDDTPGKYWKEL
DC-176WP_001159761.1_247AbBCP   KKYEMYYGLQSVQDKKAVNSSTIFELGSVSKLFTATAGGYAKNKGKISFDDTPGKYWKEL
ADC-176WP_001159761.1_9AbBCP    KKYEMYYGLQSVQDKKAVNSSTIFELGSVSKLFTATAGGYAKNKGKISFDDTPGKYWKEL
C-176WP_001159761.1_2982AbBCP   KKYEMYYGLQSVQDKKAVNSSTIFELGSVSKLFTATAGGYAKNKGKISFDDTPGKYWKEL
ADC-176QLG80706.1_1971AbBCP    KKYEMYYGLQSVQDKKAVNSSTIFELGSVSKLFTATAGGYAKNKGKISFDDTPGKYWKEL
C-176WP_001159761.1_2851AbBCP   KKYEMYYGLQSVQDKKAVNSSTIFELGSVSKLFTATAGGYAKNKGKISFDDTPGKYWKEL
C-176WP_001159761.1_1220AbBCP   KKYEMYYGLQSVQDKKAVNSSTIFELGSVSKLFTATAGGYAKNKGKISFDDTPGKYWKEL
DC-176WP_001159761.1_386AbBCP   KKYEMYYGLQSVQDKKAVNSSTIFELGSVSKLFTATAGGYAKNKGKISFDDTPGKYWKEL
ADC-176WP_001159761.1_11AbBCP   KKYEMYYGLQSVQDKKAVNSSTIFELGSVSKLFTATAGGYAKNKGKISFDDTPGKYWKEL
ADC-199WP_114166683.1_169AbOC   KKYEMYYGLQSVQDKKAVNSSTIFELGSVSKLFTATAGGYAKNKGKISFDDTPGKYWKEL
DC-199WP_114166683.1_267AbBOC   KKYEMYYGLQSVQDKKAVNSSTIFELGSVSKLFTATAGGYAKNKGKISFDDTPGKYWKEL
DC-199WP_114166683.1_267AbBCP   KKYEMYYGLQSVQDKKAVNSSTIFELGSVSKLFTATAGGYAKNKGKISFDDTPGKYWKEL
                           acc
```

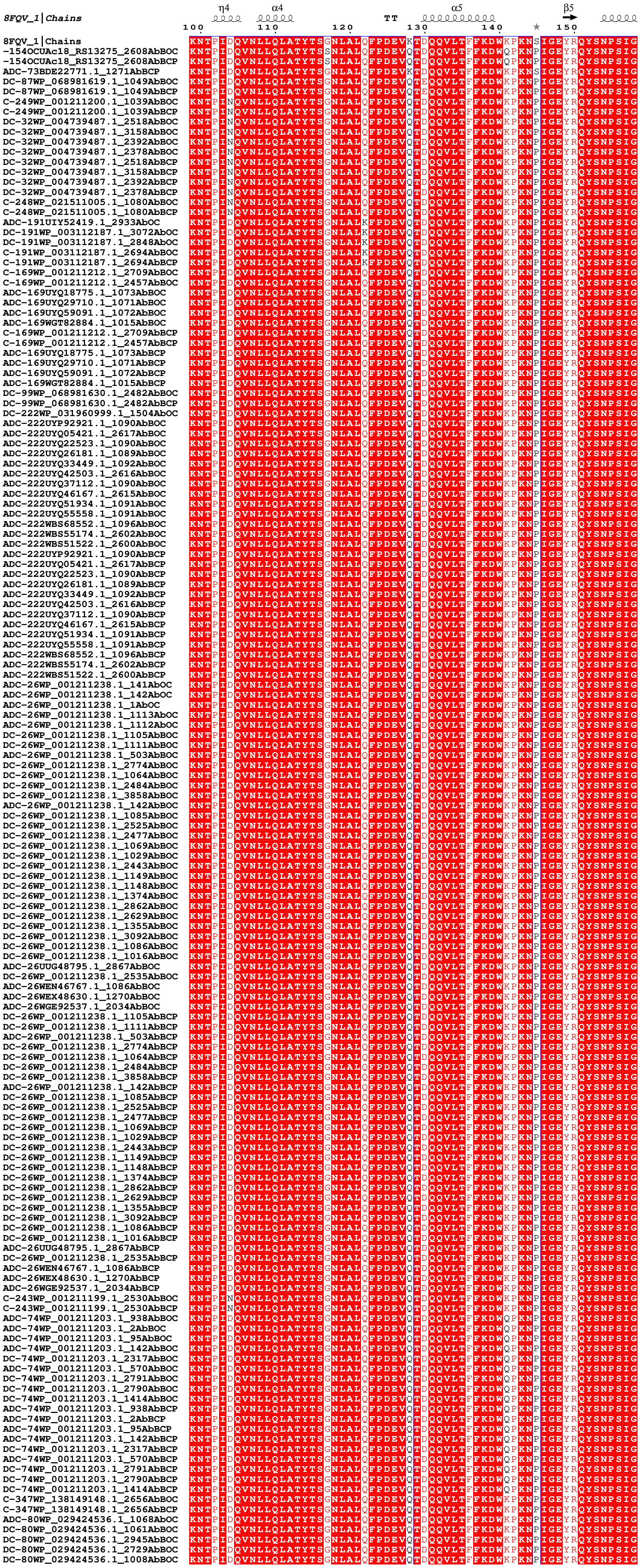
8FQV_1|Chains
η4
α4
α5
β5
TT
100
110
120
130
140
150

```
DC-80WP_029424536.1_1061AbBCP    KNTPIDQVNLLQLATYTSGNLALQFPDEVQTDQQVLTFFKDWKPKNPIGEYRQYSNPSIG
DC-80WP_029424536.1_2945AbBCP    KNTPIDQVNLLQLATYTSGNLALQFPDEVQTDQQVLTFFKDWKPKNPIGEYRQYSNPSIG
DC-80WP_029424536.1_2729AbBCP    KNTPIDQVNLLQLATYTSGNLALQFPDEVQTDQQVLTFFKDWKPKNPIGEYRQYSNPSIG
DC-80WP_029424536.1_1008AbBCP    KNTPIDQVNLLQLATYTSGNLALQFPDEVQTDQQVLTFFKDWKPKNPIGEYRQYSNPSIG
DC-156WP_024436624.1_2681AbOC    KNTPIDQVNLLQLATYTSGNLALQFPDEVQTDQQVLTFFKDWQPKNPIGEYRQYSNPSIG
C-156WP_024436624.1_2375AbBOC    KNTPIDQVNLLQLATYTSGNLALQFPDEVQTDQQVLTFFKDWQPKNPIGEYRQYSNPSIG
C-156WP_024436624.1_1038AbBOC    KNTPIDQVNLLQLATYTSGNLALQFPDEVQTDQQVLTFFKDWQPKNPIGEYRQYSNPSIG
C-156WP_024436624.1_1041AbBOC    KNTPIDQVNLLQLATYTSGNLALQFPDEVQTDQQVLTFFKDWQPKNPIGEYRQYSNPSIG
C-156WP_024436624.1_1124AbBOC    KNTPIDQVNLLQLATYTSGNLALQFPDEVQTDQQVLTFFKDWQPKNPIGEYRQYSNPSIG
C-156WP_024436624.1_1126AbBOC    KNTPIDQVNLLQLATYTSGNLALQFPDEVQTDQQVLTFFKDWQPKNPIGEYRQYSNPSIG
C-156WP_024436624.1_2375AbBCP    KNTPIDQVNLLQLATYTSGNLALQFPDEVQTDQQVLTFFKDWQPKNPIGEYRQYSNPSIG
C-156WP_024436624.1_1038AbBCP    KNTPIDQVNLLQLATYTSGNLALQFPDEVQTDQQVLTFFKDWQPKNPIGEYRQYSNPSIG
C-156WP_024436624.1_1041AbBCP    KNTPIDQVNLLQLATYTSGNLALQFPDEVQTDQQVLTFFKDWQPKNPIGEYRQYSNPSIG
C-156WP_024436624.1_1124AbBCP    KNTPIDQVNLLQLATYTSGNLALQFPDEVQTDQQVLTFFKDWQPKNPIGEYRQYSNPSIG
C-156WP_024436624.1_1126AbBCP    KNTPIDQVNLLQLATYTSGNLALQFPDEVQTDQQVLTFFKDWQPKNPIGEYRQYSNPSIG
C-165WP_001211239.1_1734AbBOC    KNTPIDQVNLLQLATYTSGNLALQFPDEVQTDQQVLTFFKDWKPKNPIGEYRQYSNPSIG
C-165WP_001211239.1_1032AbBOC    KNTPIDQVNLLQLATYTSGNLALQFPDEVQTDQQVLTFFKDWKPKNPIGEYRQYSNPSIG
C-165WP_001211239.1_1734AbBCP    KNTPIDQVNLLQLATYTSGNLALQFPDEVQTDQQVLTFFKDWKPKNPIGEYRQYSNPSIG
C-165WP_001211239.1_1032AbBCP    KNTPIDQVNLLQLATYTSGNLALQFPDEVQTDQQVLTFFKDWKPKNPIGEYRQYSNPSIG
C-263WP_109433738.1_2026AbBOC    KNTPIDQVNLLQLATYTSGNLALQFPDEVQTDQQVLTLFKDWKPKNPIGEYRQYSNPSIG
DC-263WP_109433738.1_954AbBOC    KNTPIDQVNLLQLATYTSGNLALQFPDEVQTDQQVLTLFKDWKPKNPIGEYRQYSNPSIG
C-263WP_109433738.1_1022AbBOC    KNTPIDQVNLLQLATYTSGNLALQFPDEVQTDQQVLTLFKDWKPKNPIGEYRQYSNPSIG
C-263WP_109433738.1_3219AbBOC    KNTPIDQVNLLQLATYTSGNLALQFPDEVQTDQQVLTLFKDWKPKNPIGEYRQYSNPSIG
C-263WP_109433738.1_2026AbBCP    KNTPIDQVNLLQLATYTSGNLALQFPDEVQTDQQVLTLFKDWKPKNPIGEYRQYSNPSIG
DC-263WP_109433738.1_954AbBCP    KNTPIDQVNLLQLATYTSGNLALQFPDEVQTDQQVLTLFKDWKPKNPIGEYRQYSNPSIG
C-263WP_109433738.1_1022AbBCP    KNTPIDQVNLLQLATYTSGNLALQFPDEVQTDQQVLTLFKDWKPKNPIGEYRQYSNPSIG
C-263WP_109433738.1_3219AbBCP    KNTPIDQVNLLQLATYTSGNLALQFPDEVQTDQQVLTLFKDWKPKNPIGEYRQYSNPSIG
DC-79WP_001159760.1_2471AbBOC    KNTPIDQVNLLQLATYTSGNLALQFPDEVQTDQQVLTFFKDWKPKNPIGEYRQYSNPSIG
ADC-79WP_001159760.1_670AbBOC    KNTPIDQVNLLQLATYTSGNLALQFPDEVQTDQQVLTFFKDWKPKNPIGEYRQYSNPSIG
DC-79WP_001159760.1_2535AbBOC    KNTPIDQVNLLQLATYTSGNLALQFPDEVQTDQQVLTFFKDWKPKNPIGEYRQYSNPSIG
DC-79WP_001159760.1_1080AbBOC    KNTPIDQVNLLQLATYTSGNLALQFPDEVQTDQQVLTFFKDWKPKNPIGEYRQYSNPSIG
DC-79WP_001159760.1_2471AbBCP    KNTPIDQVNLLQLATYTSGNLALQFPDEVQTDQQVLTFFKDWKPKNPIGEYRQYSNPSIG
ADC-79WP_001159760.1_670AbBCP    KNTPIDQVNLLQLATYTSGNLALQFPDEVQTDQQVLTFFKDWKPKNPIGEYRQYSNPSIG
DC-79WP_001159760.1_2535AbBCP    KNTPIDQVNLLQLATYTSGNLALQFPDEVQTDQQVLTFFKDWKPKNPIGEYRQYSNPSIG
DC-79WP_001159760.1_1080AbBCP    KNTPIDQVNLLQLATYTSGNLALQFPDEVQTDQQVLTFFKDWKPKNPIGEYRQYSNPSIG
ADC-6WP_017725267.1_1459AbBOC    KNTPIDQVNLLQLATYTSGNLALQFPDEVQTDQQVLTLFKDWKPKNPIGEYRQYSNPSIG
ADC-6WP_017725267.1_1678AbBOC    KNTPIDQVNLLQLATYTSGNLALQFPDEVQTDQQVLTLFKDWKPKNPIGEYRQYSNPSIG
ADC-6WP_017725267.1_2561AbBOC    KNTPIDQVNLLQLATYTSGNLALQFPDEVQTDQQVLTLFKDWKPKNPIGEYRQYSNPSIG
ADC-6WP_017725267.1_1459AbBCP    KNTPIDQVNLLQLATYTSGNLALQFPDEVQTDQQVLTLFKDWKPKNPIGEYRQYSNPSIG
ADC-6WP_017725267.1_1678AbBCP    KNTPIDQVNLLQLATYTSGNLALQFPDEVQTDQQVLTLFKDWKPKNPIGEYRQYSNPSIG
ADC-6WP_017725267.1_2561AbBCP    KNTPIDQVNLLQLATYTSGNLALQFPDEVQTDQQVLTLFKDWKPKNPIGEYRQYSNPSIG
ADC-1WP_004714775.1_3726AbBOC    KNTPIDQVNLLQLATYTSGNLALQFPDEVKTDQQVLTFFKDWKPKNSIGEYRQYSNPSIG
ADC-1WP_004714775.1_3726AbBCP    KNTPIDQVNLLQLATYTSGNLALQFPDEVKTDQQVLTFFKDWKPKNSIGEYRQYSNPSIG
C-181WP_046693238.1_1083AbBOC    KNTPIDQVNLLQLATYTSGNLALQFPDEVQTDQQVLTFFKDWQPKNPIGEYRQYSNPSIG
C-181WP_046693238.1_1083AbBCP    KNTPIDQVNLLQLATYTSGNLALQFPDEVQTDQQVLTFFKDWQPKNPIGEYRQYSNPSIG
C-238WP_002058891.1_2458AbBOC    KNTPIDQVNLLQLATYTSGNLALQFPDEVQTDQQVLTFFKDWKPKNPIGEYRQYSNPSIG
C-238WP_002058891.1_2458AbBCP    KNTPIDQVNLLQLATYTSGNLALQFPDEVQTDQQVLTFFKDWKPKNPIGEYRQYSNPSIG
C-186WP_072292271.1_1675AbBOC    KNTPIDQVNLLQLATYTSGNLALQFPDEVQTDQQVLTFFKDWKPKNPIGEYRQYSNPSIG
C-186WP_072292271.1_1675AbBCP    KNTPIDQVNLLQLATYTSGNLALQFPDEVQTDQQVLTFFKDWKPKNPIGEYRQYSNPSIG
DC-184WP_059247009.1_1188AbOC    KNTPIDQVNLLQLATYTSGNLALQFPDEVQTDQQVLTFFKDWQPKNPIGEYRQYSNPSIG
ADC-328WP_069370341.1_666AbOC    KNTPIDQVNLLQLATYTSGNLALQFPDEVQTDQQVLTFFKDWQPKNPIGEYRQYSNPSIG
DC-56WP_031973850.1_1911AbBOC    KNTPIDQVNLLQLATYTSGNLALQFPDEVKTDQQVLTFFKDWKPKNSIGEYQQYSNPSIG
DC-56WP_031973850.1_2693AbBOC    KNTPIDQVNLLQLATYTSGNLALQFPDEVKTDQQVLTFFKDWKPKNSIGEYQQYSNPSIG
DC-56WP_031973850.1_1911AbBCP    KNTPIDQVNLLQLATYTSGNLALQFPDEVKTDQQVLTFFKDWKPKNSIGEYQQYSNPSIG
DC-56WP_031973850.1_2693AbBCP    KNTPIDQVNLLQLATYTSGNLALQFPDEVKTDQQVLTFFKDWKPKNSIGEYQQYSNPSIG
DC-117WP_002157727.1_394AbBOC    KNTPIDQVNLLQLATYTSGNLALQFPDEVQTDQQVLTFFKDWKPKNPIGEYRQYSNPSIG
DC-117WP_002157727.1_394AbBCP    KNTPIDQVNLLQLATYTSGNLALQFPDEVQTDQQVLTFFKDWKPKNPIGEYRQYSNPSIG
ADC-25WP_001211217.1_2432AbOC    KNTPIDQVNLLQLATYTSGNLALQFPDEVKTDQQVLTFFKDWKPKNSIGEYRQYSNPSIG
ADC-25WP_001211217.1_1025AbOC    KNTPIDQVNLLQLATYTSGNLALQFPDEVKTDQQVLTFFKDWKPKNSIGEYRQYSNPSIG
ADC-25WP_001211217.1_3391AbOC    KNTPIDQVNLLQLATYTSGNLALQFPDEVKTDQQVLTFFKDWKPKNSIGEYRQYSNPSIG
ADC-25WP_001211217.1_1042AbOC    KNTPIDQVNLLQLATYTSGNLALQFPDEVKTDQQVLTFFKDWKPKNSIGEYRQYSNPSIG
ADC-25WP_001211217.1_1185AbOC    KNTPIDQVNLLQLATYTSGNLALQFPDEVKTDQQVLTFFKDWKPKNSIGEYRQYSNPSIG
ADC-25WP_001211217.1_768AbOC     KNTPIDQVNLLQLATYTSGNLALQFPDEVKTDQQVLTFFKDWKPKNSIGEYRQYSNPSIG
ADC-25WP_001211217.1_2605AbOC    KNTPIDQVNLLQLATYTSGNLALQFPDEVKTDQQVLTFFKDWKPKNSIGEYRQYSNPSIG
DC-25WP_001211217.1_1117AbBOC    KNTPIDQVNLLQLATYTSGNLALQFPDEVKTDQQVLTFFKDWKPKNSIGEYRQYSNPSIG
DC-25WP_001211217.1_1090AbBOC    KNTPIDQVNLLQLATYTSGNLALQFPDEVKTDQQVLTFFKDWKPKNSIGEYRQYSNPSIG
DC-25WP_001211217.1_1089AbBOC    KNTPIDQVNLLQLATYTSGNLALQFPDEVKTDQQVLTFFKDWKPKNSIGEYRQYSNPSIG
DC-25WP_001211217.1_1091AbBOC    KNTPIDQVNLLQLATYTSGNLALQFPDEVKTDQQVLTFFKDWKPKNSIGEYRQYSNPSIG
DC-25WP_001211217.1_1604AbBOC    KNTPIDQVNLLQLATYTSGNLALQFPDEVKTDQQVLTFFKDWKPKNSIGEYRQYSNPSIG
DC-25WP_001211217.1_1040AbBOC    KNTPIDQVNLLQLATYTSGNLALQFPDEVKTDQQVLTFFKDWKPKNSIGEYRQYSNPSIG
ADC-25UYQ12932.1_2634AbBOC      KNTPIDQVNLLQLATYTSGNLALQFPDEVKTDQQVLTFFKDWKPKNSIGEYRQYSNPSIG
ADC-25UYQ09083.1_2628AbBOC      KNTPIDQVNLLQLATYTSGNLALQFPDEVKTDQQVLTFFKDWKPKNSIGEYRQYSNPSIG
ADC-25UYQ40822.1_1120AbBOC      KNTPIDQVNLLQLATYTSGNLALQFPDEVKTDQQVLTFFKDWKPKNSIGEYRQYSNPSIG
DC-25WP_001211217.1_1117AbBCP    KNTPIDQVNLLQLATYTSGNLALQFPDEVKTDQQVLTFFKDWKPKNSIGEYRQYSNPSIG
DC-25WP_001211217.1_1090AbBCP    KNTPIDQVNLLQLATYTSGNLALQFPDEVKTDQQVLTFFKDWKPKNSIGEYRQYSNPSIG
DC-25WP_001211217.1_1089AbBCP    KNTPIDQVNLLQLATYTSGNLALQFPDEVKTDQQVLTFFKDWKPKNSIGEYRQYSNPSIG
DC-25WP_001211217.1_1091AbBCP    KNTPIDQVNLLQLATYTSGNLALQFPDEVKTDQQVLTFFKDWKPKNSIGEYRQYSNPSIG
DC-25WP_001211217.1_1604AbBCP    KNTPIDQVNLLQLATYTSGNLALQFPDEVKTDQQVLTFFKDWKPKNSIGEYRQYSNPSIG
DC-25WP_001211217.1_1040AbBCP    KNTPIDQVNLLQLATYTSGNLALQFPDEVKTDQQVLTFFKDWKPKNSIGEYRQYSNPSIG
ADC-25UYQ12932.1_2634AbBCP      KNTPIDQVNLLQLATYTSGNLALQFPDEVKTDQQVLTFFKDWKPKNSIGEYRQYSNPSIG
ADC-25UYQ09083.1_2628AbBCP      KNTPIDQVNLLQLATYTSGNLALQFPDEVKTDQQVLTFFKDWKPKNSIGEYRQYSNPSIG
ADC-25UYQ40822.1_1120AbBCP      KNTPIDQVNLLQLATYTSGNLALQFPDEVKTDQQVLTFFKDWKPKNSIGEYRQYSNPSIG
C-268WP_105922788.1_2839AbBOC    KNTPIDQVNLLQLATYTSGNLALQFPDEVKTDQQVLTFFKDWKPKNSIGEYQQYSNPSIG
ADC-268WDC47378.1_2492AbBOC     KNTPIDQVNLLQLATYTSGNLALQFPDEVKTDQQVLTFFKDWKPKNSIGEYQQYSNPSIG
C-268WP_105922788.1_2839AbBCP    KNTPIDQVNLLQLATYTSGNLALQFPDEVKTDQQVLTFFKDWKPKNSIGEYQQYSNPSIG
ADC-268WDC47378.1_2492AbBCP     KNTPIDQVNLLQLATYTSGNLALQFPDEVKTDQQVLTFFKDWKPKNSIGEYQQYSNPSIG
DC-155WP_005128228.1_2591AbOC    KNTPIDQVNLLQLATYTSGNLALQFPDEVQTDQQVLTFFKDWKPKNPIGEYRQYSNPSIG
C-155WP_005128228.1_2549AbBOC    KNTPIDQVNLLQLATYTSGNLALQFPDEVQTDQQVLTFFKDWKPKNPIGEYRQYSNPSIG
C-155WP_005128228.1_2480AbBOC    KNTPIDQVNLLQLATYTSGNLALQFPDEVQTDQQVLTFFKDWKPKNPIGEYRQYSNPSIG
C-155WP_005128228.1_2455AbBOC    KNTPIDQVNLLQLATYTSGNLALQFPDEVQTDQQVLTFFKDWKPKNPIGEYRQYSNPSIG
C-155WP_005128228.1_2549AbBCP    KNTPIDQVNLLQLATYTSGNLALQFPDEVQTDQQVLTFFKDWKPKNPIGEYRQYSNPSIG
C-155WP_005128228.1_2480AbBCP    KNTPIDQVNLLQLATYTSGNLALQFPDEVQTDQQVLTFFKDWKPKNPIGEYRQYSNPSIG
C-155WP_005128228.1_2455AbBCP    KNTPIDQVNLLQLATYTSGNLALQFPDEVQTDQQVLTFFKDWKPKNPIGEYRQYSNPSIG
ADC-76WP_001211237.1_2862AbOC    KNTPIDQVNLLQLATYTSGNLALQFPDEVQTDQQVLTFFKDWKPKNPIGESRQYSNPSIG
ADC-76WP_001211237.1_3431AbOC    KNTPIDQVNLLQLATYTSGNLALQFPDEVQTDQQVLTFFKDWKPKNPIGESRQYSNPSIG
ADC-76WP_001211237.1_2696AbOC    KNTPIDQVNLLQLATYTSGNLALQFPDEVQTDQQVLTFFKDWKPKNPIGESRQYSNPSIG
ADC-76WP_001211237.1_2890AbOC    KNTPIDQVNLLQLATYTSGNLALQFPDEVQTDQQVLTFFKDWKPKNPIGESRQYSNPSIG
DC-76WP_001211237.1_2723AbBOC    KNTPIDQVNLLQLATYTSGNLALQFPDEVQTDQQVLTFFKDWKPKNPIGESRQYSNPSIG
DC-76WP_001211237.1_1033AbBOC    KNTPIDQVNLLQLATYTSGNLALQFPDEVQTDQQVLTFFKDWKPKNPIGESRQYSNPSIG
ADC-76WP_001211237.1_526AbBOC    KNTPIDQVNLLQLATYTSGNLALQFPDEVQTDQQVLTFFKDWKPKNPIGESRQYSNPSIG
DC-76WP_001211237.1_1046AbBOC    KNTPIDQVNLLQLATYTSGNLALQFPDEVQTDQQVLTFFKDWKPKNPIGESRQYSNPSIG
DC-76WP_001211237.1_1040AbBOC    KNTPIDQVNLLQLATYTSGNLALQFPDEVQTDQQVLTFFKDWKPKNPIGESRQYSNPSIG
DC-76WP_001211237.1_3682AbBOC    KNTPIDQVNLLQLATYTSGNLALQFPDEVQTDQQVLTFFKDWKPKNPIGESRQYSNPSIG
DC-76WP_001211237.1_2579AbBOC    KNTPIDQVNLLQLATYTSGNLALQFPDEVQTDQQVLTFFKDWKPKNPIGESRQYSNPSIG
DC-76WP_001211237.1_2723AbBCP    KNTPIDQVNLLQLATYTSGNLALQFPDEVQTDQQVLTFFKDWKPKNPIGESRQYSNPSIG
DC-76WP_001211237.1_1033AbBCP    KNTPIDQVNLLQLATYTSGNLALQFPDEVQTDQQVLTFFKDWKPKNPIGESRQYSNPSIG
ADC-76WP_001211237.1_526AbBCP    KNTPIDQVNLLQLATYTSGNLALQFPDEVQTDQQVLTFFKDWKPKNPIGESRQYSNPSIG
DC-76WP_001211237.1_1046AbBCP    KNTPIDQVNLLQLATYTSGNLALQFPDEVQTDQQVLTFFKDWKPKNPIGESRQYSNPSIG
DC-76WP_001211237.1_1040AbBCP    KNTPIDQVNLLQLATYTSGNLALQFPDEVQTDQQVLTFFKDWKPKNPIGESRQYSNPSIG
DC-76WP_001211237.1_3682AbBCP    KNTPIDQVNLLQLATYTSGNLALQFPDEVQTDQQVLTFFKDWKPKNPIGESRQYSNPSIG
DC-76WP_001211237.1_2579AbBCP    KNTPIDQVNLLQLATYTSGNLALQFPDEVQTDQQVLTFFKDWKPKNPIGESRQYSNPSIG
DC-291WP_001211201.1_1060AbOC    KNTPIDQVNLLQLATYTSGNLALQFPDEVQTDQQVLTFFKDWKPKNPIGEYRQYSNPSIG
C-291WP_001211201.1_2415AbBOC    KNTPIDQVNLLQLATYTSGNLALQFPDEVQTDQQVLTFFKDWKPKNPIGEYRQYSNPSIG
C-291WP_001211201.1_2415AbBCP    KNTPIDQVNLLQLATYTSGNLALQFPDEVQTDQQVLTFFKDWKPKNPIGEYRQYSNPSIG
DC-214WP_060454527.1_118AbBOC    KNTSIDQVNLLQLATYTSGNLALQFPDEVQTDQQVLTFFKDWQPKNPIGEYRQYSNPSIG
DC-214WP_060454527.1_118AbBCP    KNTSIDQVNLLQLATYTSGNLALQFPDEVQTDQQVLTFFKDWQPKNPIGEYRQYSNPSIG
C-185WP_001211210.1_2745AbBOC    KNTPIDQVNLLQLATYTSGNLALQFPDEVQTDQQVLTFFKDWKPKNPIGEYRQYSNPSIG
ADC-185WEI07035.1_298AbBOC      KNTPIDQVNLLQLATYTSGNLALQFPDEVQTDQQVLTFFKDWKPKNPIGEYRQYSNPSIG
C-185WP_001211210.1_2745AbBCP    KNTPIDQVNLLQLATYTSGNLALQFPDEVQTDQQVLTFFKDWKPKNPIGEYRQYSNPSIG
ADC-185WEI07035.1_298AbBCP      KNTPIDQVNLLQLATYTSGNLALQFPDEVQTDQQVLTFFKDWKPKNPIGEYRQYSNPSIG
ADC-11WP_001211205.1_1099AbOC    KNTPIDQVNLLQLATYTSGNLALQFPDEVQTDQQVLTFFKDWQPKNPIGEYRQYSNPSIG
ADC-11WP_001211205.1_1035AbOC    KNTPIDQVNLLQLATYTSGNLALQFPDEVQTDQQVLTFFKDWQPKNPIGEYRQYSNPSIG
DC-11WP_001211205.1_2603AbBOC    KNTPIDQVNLLQLATYTSGNLALQFPDEVQTDQQVLTFFKDWQPKNPIGEYRQYSNPSIG
DC-11WP_001211205.1_1138AbBOC    KNTPIDQVNLLQLATYTSGNLALQFPDEVQTDQQVLTFFKDWQPKNPIGEYRQYSNPSIG
DC-11WP_001211205.1_2874AbBOC    KNTPIDQVNLLQLATYTSGNLALQFPDEVQTDQQVLTFFKDWQPKNPIGEYRQYSNPSIG
DC-11WP_001211205.1_2686AbBOC    KNTPIDQVNLLQLATYTSGNLALQFPDEVQTDQQVLTFFKDWQPKNPIGEYRQYSNPSIG
ADC-11WP_001211205.1_130AbBOC    KNTPIDQVNLLQLATYTSGNLALQFPDEVQTDQQVLTFFKDWQPKNPIGEYRQYSNPSIG
DC-11WP_001211205.1_1097AbBOC    KNTPIDQVNLLQLATYTSGNLALQFPDEVQTDQQVLTFFKDWQPKNPIGEYRQYSNPSIG
ADC-11WP_001211205.1_684AbBOC    KNTPIDQVNLLQLATYTSGNLALQFPDEVQTDQQVLTFFKDWQPKNPIGEYRQYSNPSIG
ADC-11WP_001211205.1_682AbBOC    KNTPIDQVNLLQLATYTSGNLALQFPDEVQTDQQVLTFFKDWQPKNPIGEYRQYSNPSIG
DC-11WP_001211205.1_1111AbBOC    KNTPIDQVNLLQLATYTSGNLALQFPDEVQTDQQVLTFFKDWQPKNPIGEYRQYSNPSIG
DC-11WP_001211205.1_1059AbBOC    KNTPIDQVNLLQLATYTSGNLALQFPDEVQTDQQVLTFFKDWQPKNPIGEYRQYSNPSIG
DC-11WP_001211205.1_2754AbBOC    KNTPIDQVNLLQLATYTSGNLALQFPDEVQTDQQVLTFFKDWQPKNPIGEYRQYSNPSIG
DC-11WP_001211205.1_2603AbBCP    KNTPIDQVNLLQLATYTSGNLALQFPDEVQTDQQVLTFFKDWQPKNPIGEYRQYSNPSIG
DC-11WP_001211205.1_1138AbBCP    KNTPIDQVNLLQLATYTSGNLALQFPDEVQTDQQVLTFFKDWQPKNPIGEYRQYSNPSIG
DC-11WP_001211205.1_2874AbBCP    KNTPIDQVNLLQLATYTSGNLALQFPDEVQTDQQVLTFFKDWQPKNPIGEYRQYSNPSIG
DC-11WP_001211205.1_2686AbBCP    KNTPIDQVNLLQLATYTSGNLALQFPDEVQTDQQVLTFFKDWQPKNPIGEYRQYSNPSIG
ADC-11WP_001211205.1_130AbBCP    KNTPIDQVNLLQLATYTSGNLALQFPDEVQTDQQVLTFFKDWQPKNPIGEYRQYSNPSIG
DC-11WP_001211205.1_1097AbBCP    KNTPIDQVNLLQLATYTSGNLALQFPDEVQTDQQVLTFFKDWQPKNPIGEYRQYSNPSIG
ADC-11WP_001211205.1_684AbBCP    KNTPIDQVNLLQLATYTSGNLALQFPDEVQTDQQVLTFFKDWQPKNPIGEYRQYSNPSIG
ADC-11WP_001211205.1_682AbBCP    KNTPIDQVNLLQLATYTSGNLALQFPDEVQTDQQVLTFFKDWQPKNPIGEYRQYSNPSIG
DC-11WP_001211205.1_1111AbBCP    KNTPIDQVNLLQLATYTSGNLALQFPDEVQTDQQVLTFFKDWQPKNPIGEYRQYSNPSIG
DC-11WP_001211205.1_1059AbBCP    KNTPIDQVNLLQLATYTSGNLALQFPDEVQTDQQVLTFFKDWQPKNPIGEYRQYSNPSIG
DC-11WP_001211205.1_2754AbBCP    KNTPIDQVNLLQLATYTSGNLALQFPDEVQTDQQVLTFFKDWQPKNPIGEYRQYSNPSIG
C-269WP_032025626.1_2988AbBOC    KNTPIDQVNLLQLATYTSGNLALQFPDEVQTDQQVLTFFKDWQPKNPIGEYRQYSNPSIG
C-269WP_032025626.1_2988AbBCP    KNTPIDQVNLLQLATYTSGNLALQFPDEVQTDQQVLTFFKDWQPKNPIGEYRQYSNPSIG
C-166WP_101244937.1_1028AbBOC    KNTPINQVNLLQLATYTSGNLALQFPDEVQTDQQVLTFFKDWKPKNPIGEYRQYSNPSIG
C-166WP_101244937.1_1028AbBCP    KNTPINQVNLLQLATYTSGNLALQFPDEVQTDQQVLTFFKDWKPKNPIGEYRQYSNPSIG
C-175WP_001211208.1_2561AbBOC    KNTPIDQVNLLQLATYTSGNLALQFPDEVQTDQQVLTFFKDWQPKNPIGEYRQYSNPSIG
C-175WP_001211208.1_2561AbBCP    KNTPIDQVNLLQLATYTSGNLALQFPDEVQTDQQVLTFFKDWQPKNPIGEYRQYSNPSIG
C-154WP_005138362.1_2451AbBOC    KNTPIDQVNLLQLATYTSSNLALQFPDEVQTDQQVLTFFKDWQPKNPIGEYRQYSNPSIG
DC-154WP_005138362.1_158AbBOC    KNTPIDQVNLLQLATYTSSNLALQFPDEVQTDQQVLTFFKDWQPKNPIGEYRQYSNPSIG
```

```
C-154WP_005138362.1_1167AbBOC      KNTPIDQVNLLQLATYTSSNLALQFPDEVQTDQQVLTFFKDWQPKNPIGEYRQYSNPSIG
C-154WP_005138362.1_2510AbBOC      KNTPIDQVNLLQLATYTSSNLALQFPDEVQTDQQVLTFFKDWQPKNPIGEYRQYSNPSIG
C-154WP_005138362.1_2513AbBOC      KNTPIDQVNLLQLATYTSSNLALQFPDEVQTDQQVLTFFKDWQPKNPIGEYRQYSNPSIG
C-154WP_005138362.1_2512AbBOC      KNTPIDQVNLLQLATYTSSNLALQFPDEVQTDQQVLTFFKDWQPKNPIGEYRQYSNPSIG
C-154WP_005138362.1_2451AbBCP      KNTPIDQVNLLQLATYTSSNLALQFPDEVQTDQQVLTFFKDWQPKNPIGEYRQYSNPSIG
DC-154WP_005138362.1_158AbBCP      KNTPIDQVNLLQLATYTSSNLALQFPDEVQTDQQVLTFFKDWQPKNPIGEYRQYSNPSIG
C-154WP_005138362.1_1167AbBCP      KNTPIDQVNLLQLATYTSSNLALQFPDEVQTDQQVLTFFKDWQPKNPIGEYRQYSNPSIG
C-154WP_005138362.1_2510AbBCP      KNTPIDQVNLLQLATYTSSNLALQFPDEVQTDQQVLTFFKDWQPKNPIGEYRQYSNPSIG
C-154WP_005138362.1_2513AbBCP      KNTPIDQVNLLQLATYTSSNLALQFPDEVQTDQQVLTFFKDWQPKNPIGEYRQYSNPSIG
C-154WP_005138362.1_2512AbBCP      KNTPIDQVNLLQLATYTSSNLALQFPDEVQTDQQVLTFFKDWQPKNPIGEYRQYSNPSIG
C-240WP_001211207.1_1200AbBOC      KNTPIDQVNLLQLATYTSGNLALQFPDEVQTDQQVLTFFKDWQPKNPIGEYRQYSNPSIG
C-240WP_001211207.1_1200AbBCP      KNTPIDQVNLLQLATYTSGNLALQFPDEVQTDQQVLTFFKDWQPKNPIGEYRQYSNPSIG
ADC-75WP_063857817.1_1610AbOC      KNTPIDQVNLLQLATYTSGNLALQFPDEVKTDQQVLTFFKDWKPKNSIGEYRQYSNPSIG
DC-18WP_002118772.1_1702AbBOC      KNTPIDQVNLLQLATYTSGNLALQFPDEVQTDQQVLTFFKDWKTKNAIGEYRQYSNPSIG
DC-18WP_002118772.1_1702AbBCP      KNTPIDQVNLLQLATYTSGNLALQFPDEVQTDQQVLTFFKDWKTKNAIGEYRQYSNPSIG
C-106WP_068981613.1_2650AbBOC      KNTPINQVNLLQLATYTSGNLALQFPDEVQTDQQVLTFFKDWKPKNPIGEYRQYSNPSIG
C-106WP_068981613.1_2650AbBCP      KNTPINQVNLLQLATYTSGNLALQFPDEVQTDQQVLTFFKDWKPKNPIGEYRQYSNPSIG
DC-50WP_031965243.1_2562AbBOC      KNTPIDQVNLLQLATYTSGNLALQFPDEVQTDQQVLTFFKDWKPKNPIGEYRQYSNPSIG
ADC-50WP_031965243.1_206AbBOC      KNTPIDQVNLLQLATYTSGNLALQFPDEVQTDQQVLTFFKDWKPKNPIGEYRQYSNPSIG
DC-50WP_031965243.1_2751AbBOC      KNTPIDQVNLLQLATYTSGNLALQFPDEVQTDQQVLTFFKDWKPKNPIGEYRQYSNPSIG
DC-50WP_031965243.1_3766AbBOC      KNTPIDQVNLLQLATYTSGNLALQFPDEVQTDQQVLTFFKDWKPKNPIGEYRQYSNPSIG
DC-50WP_031965243.1_2562AbBCP      KNTPIDQVNLLQLATYTSGNLALQFPDEVQTDQQVLTFFKDWKPKNPIGEYRQYSNPSIG
ADC-50WP_031965243.1_206AbBCP      KNTPIDQVNLLQLATYTSGNLALQFPDEVQTDQQVLTFFKDWKPKNPIGEYRQYSNPSIG
DC-50WP_031965243.1_2751AbBCP      KNTPIDQVNLLQLATYTSGNLALQFPDEVQTDQQVLTFFKDWKPKNPIGEYRQYSNPSIG
DC-50WP_031965243.1_3766AbBCP      KNTPIDQVNLLQLATYTSGNLALQFPDEVQTDQQVLTFFKDWKPKNPIGEYRQYSNPSIG
ADC-158WP_001211227.1_990AbOC      KNTPIDQVNLLQLATYTSGNLALQFPDEVQTDQQVLTFFKDWKPKNPIGEYRQYSNPSIG
DC-158WP_001211227.1_1295AbOC      KNTPIDQVNLLQLATYTSGNLALQFPDEVQTDQQVLTFFKDWKPKNPIGEYRQYSNPSIG
ADC-158WP_001211227.1_6AbOC        KNTPIDQVNLLQLATYTSGNLALQFPDEVQTDQQVLTFFKDWKPKNPIGEYRQYSNPSIG
DC-158WP_001211227.1_991AbBOC     KNTPIDQVNLLQLATYTSGNLALQFPDEVQTDQQVLTFFKDWKPKNPIGEYRQYSNPSIG
DC-158WP_001211227.1_207AbBOC     KNTPIDQVNLLQLATYTSGNLALQFPDEVQTDQQVLTFFKDWKPKNPIGEYRQYSNPSIG
C-158WP_001211227.1_2735AbBOC     KNTPIDQVNLLQLATYTSGNLALQFPDEVQTDQQVLTFFKDWKPKNPIGEYRQYSNPSIG
DC-158WP_001211227.1_996AbBOC     KNTPIDQVNLLQLATYTSGNLALQFPDEVQTDQQVLTFFKDWKPKNPIGEYRQYSNPSIG
DC-158WP_001211227.1_796AbBOC     KNTPIDQVNLLQLATYTSGNLALQFPDEVQTDQQVLTFFKDWKPKNPIGEYRQYSNPSIG
C-158WP_001211227.1_2822AbBOC     KNTPIDQVNLLQLATYTSGNLALQFPDEVQTDQQVLTFFKDWKPKNPIGEYRQYSNPSIG
C-158WP_001211227.1_1339AbBOC     KNTPIDQVNLLQLATYTSGNLALQFPDEVQTDQQVLTFFKDWKPKNPIGEYRQYSNPSIG
ADC-158WGQ04828.1_2712AbBOC       KNTPIDQVNLLQLATYTSGNLALQFPDEVQTDQQVLTFFKDWKPKNPIGEYRQYSNPSIG
DC-158WP_001211227.1_991AbBCP     KNTPIDQVNLLQLATYTSGNLALQFPDEVQTDQQVLTFFKDWKPKNPIGEYRQYSNPSIG
DC-158WP_001211227.1_207AbBCP     KNTPIDQVNLLQLATYTSGNLALQFPDEVQTDQQVLTFFKDWKPKNPIGEYRQYSNPSIG
C-158WP_001211227.1_2735AbBCP     KNTPIDQVNLLQLATYTSGNLALQFPDEVQTDQQVLTFFKDWKPKNPIGEYRQYSNPSIG
DC-158WP_001211227.1_996AbBCP     KNTPIDQVNLLQLATYTSGNLALQFPDEVQTDQQVLTFFKDWKPKNPIGEYRQYSNPSIG
DC-158WP_001211227.1_796AbBCP     KNTPIDQVNLLQLATYTSGNLALQFPDEVQTDQQVLTFFKDWKPKNPIGEYRQYSNPSIG
C-158WP_001211227.1_2822AbBCP     KNTPIDQVNLLQLATYTSGNLALQFPDEVQTDQQVLTFFKDWKPKNPIGEYRQYSNPSIG
C-158WP_001211227.1_1339AbBCP     KNTPIDQVNLLQLATYTSGNLALQFPDEVQTDQQVLTFFKDWKPKNPIGEYRQYSNPSIG
ADC-158WGQ04828.1_2712AbBCP       KNTPIDQVNLLQLATYTSGNLALQFPDEVQTDQQVLTFFKDWKPKNPIGEYRQYSNPSIG
ADC-31WP_001211223.1_2986AbOC      KNTPIDQVNLLQLATYTSGNLALQFPDEVKTDQQVLTFFKDWKPKNSIGEYRQYSNPSIG
DC-31WP_001211223.1_2698AbBOC      KNTPIDQVNLLQLATYTSGNLALQFPDEVKTDQQVLTFFKDWKPKNSIGEYRQYSNPSIG
DC-31WP_001211223.1_2698AbBCP      KNTPIDQVNLLQLATYTSGNLALQFPDEVKTDQQVLTFFKDWKPKNSIGEYRQYSNPSIG
ADC-30WP_001211218.1_1102AbOC      KNTPIDQVNLLQLATYTSGNLALQFPDEVKTDQQVLTFFKDWKPKNSIGEYRQYSNPSIG
ADC-30WP_001211218.1_2565AbOC      KNTPIDQVNLLQLATYTSGNLALQFPDEVKTDQQVLTFFKDWKPKNSIGEYRQYSNPSIG
ADC-30WP_001211218.1_1021AbOC      KNTPIDQVNLLQLATYTSGNLALQFPDEVKTDQQVLTFFKDWKPKNSIGEYRQYSNPSIG
ADC-30WP_001211218.1_1088AbOC      KNTPIDQVNLLQLATYTSGNLALQFPDEVKTDQQVLTFFKDWKPKNSIGEYRQYSNPSIG
ADC-30WP_001211218.1_1135AbOC      KNTPIDQVNLLQLATYTSGNLALQFPDEVKTDQQVLTFFKDWKPKNSIGEYRQYSNPSIG
ADC-30WP_001211218.1_43AbOC        KNTPIDQVNLLQLATYTSGNLALQFPDEVKTDQQVLTFFKDWKPKNSIGEYRQYSNPSIG
ADC-30WP_001211218.1_1041AbOC      KNTPIDQVNLLQLATYTSGNLALQFPDEVKTDQQVLTFFKDWKPKNSIGEYRQYSNPSIG
ADC-30WP_001211218.1_1032AbOC      KNTPIDQVNLLQLATYTSGNLALQFPDEVKTDQQVLTFFKDWKPKNSIGEYRQYSNPSIG
ADC-30WP_001211218.1_1047AbOC      KNTPIDQVNLLQLATYTSGNLALQFPDEVKTDQQVLTFFKDWKPKNSIGEYRQYSNPSIG
ADC-30WP_001211218.1_1040AbOC      KNTPIDQVNLLQLATYTSGNLALQFPDEVKTDQQVLTFFKDWKPKNSIGEYRQYSNPSIG
ADC-30WP_001211218.1_3388AbOC      KNTPIDQVNLLQLATYTSGNLALQFPDEVKTDQQVLTFFKDWKPKNSIGEYRQYSNPSIG
ADC-30WP_001211218.1_650AbOC       KNTPIDQVNLLQLATYTSGNLALQFPDEVKTDQQVLTFFKDWKPKNSIGEYRQYSNPSIG
ADC-30WP_001211218.1_2855AbOC      KNTPIDQVNLLQLATYTSGNLALQFPDEVKTDQQVLTFFKDWKPKNSIGEYRQYSNPSIG
ADC-30WP_001211218.1_1034AbOC      KNTPIDQVNLLQLATYTSGNLALQFPDEVKTDQQVLTFFKDWKPKNSIGEYRQYSNPSIG
ADC-30WP_001211218.1_79AbOC        KNTPIDQVNLLQLATYTSGNLALQFPDEVKTDQQVLTFFKDWKPKNSIGEYRQYSNPSIG
ADC-30WP_001211218.1_3509AbOC      KNTPIDQVNLLQLATYTSGNLALQFPDEVKTDQQVLTFFKDWKPKNSIGEYRQYSNPSIG
ADC-30WP_001211218.1_2399AbOC      KNTPIDQVNLLQLATYTSGNLALQFPDEVKTDQQVLTFFKDWKPKNSIGEYRQYSNPSIG
ADC-30WP_001211218.1_2415AbOC      KNTPIDQVNLLQLATYTSGNLALQFPDEVKTDQQVLTFFKDWKPKNSIGEYRQYSNPSIG
ADC-30WP_001211218.1_3032AbOC      KNTPIDQVNLLQLATYTSGNLALQFPDEVKTDQQVLTFFKDWKPKNSIGEYRQYSNPSIG
ADC-30WP_001211218.1_832AbOC       KNTPIDQVNLLQLATYTSGNLALQFPDEVKTDQQVLTFFKDWKPKNSIGEYRQYSNPSIG
ADC-30WP_001211218.1_1428AbOC      KNTPIDQVNLLQLATYTSGNLALQFPDEVKTDQQVLTFFKDWKPKNSIGEYRQYSNPSIG
ADC-30WP_001211218.1_2340AbOC      KNTPIDQVNLLQLATYTSGNLALQFPDEVKTDQQVLTFFKDWKPKNSIGEYRQYSNPSIG
ADC-30WP_001211218.1_2976AbOC      KNTPIDQVNLLQLATYTSGNLALQFPDEVKTDQQVLTFFKDWKPKNSIGEYRQYSNPSIG
ADC-30WP_001211218.1_2305AbOC      KNTPIDQVNLLQLATYTSGNLALQFPDEVKTDQQVLTFFKDWKPKNSIGEYRQYSNPSIG
ADC-30WP_001211218.1_1110AbOC      KNTPIDQVNLLQLATYTSGNLALQFPDEVKTDQQVLTFFKDWKPKNSIGEYRQYSNPSIG
ADC-30WP_001211218.1_2797AbOC      KNTPIDQVNLLQLATYTSGNLALQFPDEVKTDQQVLTFFKDWKPKNSIGEYRQYSNPSIG
ADC-30WP_001211218.1_1023AbOC      KNTPIDQVNLLQLATYTSGNLALQFPDEVKTDQQVLTFFKDWKPKNSIGEYRQYSNPSIG
ADC-30WP_001211218.1_1022AbOC      KNTPIDQVNLLQLATYTSGNLALQFPDEVKTDQQVLTFFKDWKPKNSIGEYRQYSNPSIG
ADC-30WP_001211218.1_2458AbOC      KNTPIDQVNLLQLATYTSGNLALQFPDEVKTDQQVLTFFKDWKPKNSIGEYRQYSNPSIG
ADC-30WP_001211218.1_2513AbOC      KNTPIDQVNLLQLATYTSGNLALQFPDEVKTDQQVLTFFKDWKPKNSIGEYRQYSNPSIG
ADC-30WP_001211218.1_2482AbOC      KNTPIDQVNLLQLATYTSGNLALQFPDEVKTDQQVLTFFKDWKPKNSIGEYRQYSNPSIG
DC-30WP_001211218.1_2692AbBOC      KNTPIDQVNLLQLATYTSGNLALQFPDEVKTDQQVLTFFKDWKPKNSIGEYRQYSNPSIG
DC-30WP_001211218.1_2549AbBOC      KNTPIDQVNLLQLATYTSGNLALQFPDEVKTDQQVLTFFKDWKPKNSIGEYRQYSNPSIG
DC-30WP_001211218.1_1026AbBOC      KNTPIDQVNLLQLATYTSGNLALQFPDEVKTDQQVLTFFKDWKPKNSIGEYRQYSNPSIG
ADC-30WP_001211218.1_373AbBOC      KNTPIDQVNLLQLATYTSGNLALQFPDEVKTDQQVLTFFKDWKPKNSIGEYRQYSNPSIG
DC-30WP_001211218.1_2465AbBOC      KNTPIDQVNLLQLATYTSGNLALQFPDEVKTDQQVLTFFKDWKPKNSIGEYRQYSNPSIG
DC-30WP_001211218.1_1027AbBOC      KNTPIDQVNLLQLATYTSGNLALQFPDEVKTDQQVLTFFKDWKPKNSIGEYRQYSNPSIG
DC-30WP_001211218.1_2650AbBOC      KNTPIDQVNLLQLATYTSGNLALQFPDEVKTDQQVLTFFKDWKPKNSIGEYRQYSNPSIG
DC-30WP_001211218.1_2674AbBOC      KNTPIDQVNLLQLATYTSGNLALQFPDEVKTDQQVLTFFKDWKPKNSIGEYRQYSNPSIG
DC-30WP_001211218.1_2689AbBOC      KNTPIDQVNLLQLATYTSGNLALQFPDEVKTDQQVLTFFKDWKPKNSIGEYRQYSNPSIG
DC-30WP_001211218.1_1105AbBOC      KNTPIDQVNLLQLATYTSGNLALQFPDEVKTDQQVLTFFKDWKPKNSIGEYRQYSNPSIG
DC-30WP_001211218.1_2670AbBOC      KNTPIDQVNLLQLATYTSGNLALQFPDEVKTDQQVLTFFKDWKPKNSIGEYRQYSNPSIG
DC-30WP_001211218.1_1019AbBOC      KNTPIDQVNLLQLATYTSGNLALQFPDEVKTDQQVLTFFKDWKPKNSIGEYRQYSNPSIG
DC-30WP_001211218.1_1039AbBOC      KNTPIDQVNLLQLATYTSGNLALQFPDEVKTDQQVLTFFKDWKPKNSIGEYRQYSNPSIG
DC-30WP_001211218.1_1023AbBOC      KNTPIDQVNLLQLATYTSGNLALQFPDEVKTDQQVLTFFKDWKPKNSIGEYRQYSNPSIG
DC-30WP_001211218.1_3189AbBOC      KNTPIDQVNLLQLATYTSGNLALQFPDEVKTDQQVLTFFKDWKPKNSIGEYRQYSNPSIG
ADC-30WP_001211218.1_594AbBOC      KNTPIDQVNLLQLATYTSGNLALQFPDEVKTDQQVLTFFKDWKPKNSIGEYRQYSNPSIG
DC-30WP_001211218.1_1718AbBOC      KNTPIDQVNLLQLATYTSGNLALQFPDEVKTDQQVLTFFKDWKPKNSIGEYRQYSNPSIG
DC-30WP_001211218.1_2484AbBOC      KNTPIDQVNLLQLATYTSGNLALQFPDEVKTDQQVLTFFKDWKPKNSIGEYRQYSNPSIG
DC-30WP_001211218.1_3305AbBOC      KNTPIDQVNLLQLATYTSGNLALQFPDEVKTDQQVLTFFKDWKPKNSIGEYRQYSNPSIG
DC-30WP_001211218.1_3346AbBOC      KNTPIDQVNLLQLATYTSGNLALQFPDEVKTDQQVLTFFKDWKPKNSIGEYRQYSNPSIG
DC-30WP_001211218.1_1482AbBOC      KNTPIDQVNLLQLATYTSGNLALQFPDEVKTDQQVLTFFKDWKPKNSIGEYRQYSNPSIG
DC-30WP_001211218.1_2574AbBOC      KNTPIDQVNLLQLATYTSGNLALQFPDEVKTDQQVLTFFKDWKPKNSIGEYRQYSNPSIG
ADC-30WP_001211218.1_831AbBOC      KNTPIDQVNLLQLATYTSGNLALQFPDEVKTDQQVLTFFKDWKPKNSIGEYRQYSNPSIG
DC-30WP_001211218.1_1826AbBOC      KNTPIDQVNLLQLATYTSGNLALQFPDEVKTDQQVLTFFKDWKPKNSIGEYRQYSNPSIG
DC-30WP_001211218.1_1024AbBOC      KNTPIDQVNLLQLATYTSGNLALQFPDEVKTDQQVLTFFKDWKPKNSIGEYRQYSNPSIG
DC-30WP_001211218.1_1618AbBOC      KNTPIDQVNLLQLATYTSGNLALQFPDEVKTDQQVLTFFKDWKPKNSIGEYRQYSNPSIG
ADC-30WP_001211218.1_301AbBOC      KNTPIDQVNLLQLATYTSGNLALQFPDEVKTDQQVLTFFKDWKPKNSIGEYRQYSNPSIG
ADC-30WP_001211218.1_940AbBOC      KNTPIDQVNLLQLATYTSGNLALQFPDEVKTDQQVLTFFKDWKPKNSIGEYRQYSNPSIG
DC-30WP_001211218.1_2949AbBOC      KNTPIDQVNLLQLATYTSGNLALQFPDEVKTDQQVLTFFKDWKPKNSIGEYRQYSNPSIG
DC-30WP_001211218.1_1028AbBOC      KNTPIDQVNLLQLATYTSGNLALQFPDEVKTDQQVLTFFKDWKPKNSIGEYRQYSNPSIG
DC-30WP_001211218.1_2526AbBOC      KNTPIDQVNLLQLATYTSGNLALQFPDEVKTDQQVLTFFKDWKPKNSIGEYRQYSNPSIG
DC-30WP_001211218.1_1040AbBOC      KNTPIDQVNLLQLATYTSGNLALQFPDEVKTDQQVLTFFKDWKPKNSIGEYRQYSNPSIG
DC-30WP_001211218.1_1089AbBOC      KNTPIDQVNLLQLATYTSGNLALQFPDEVKTDQQVLTFFKDWKPKNSIGEYRQYSNPSIG
DC-30WP_001211218.1_1099AbBOC      KNTPIDQVNLLQLATYTSGNLALQFPDEVKTDQQVLTFFKDWKPKNSIGEYRQYSNPSIG
DC-30WP_001211218.1_1206AbBOC      KNTPIDQVNLLQLATYTSGNLALQFPDEVKTDQQVLTFFKDWKPKNSIGEYRQYSNPSIG
DC-30WP_001211218.1_1167AbBOC      KNTPIDQVNLLQLATYTSGNLALQFPDEVKTDQQVLTFFKDWKPKNSIGEYRQYSNPSIG
DC-30WP_001211218.1_1186AbBOC      KNTPIDQVNLLQLATYTSGNLALQFPDEVKTDQQVLTFFKDWKPKNSIGEYRQYSNPSIG
DC-30WP_001211218.1_1165AbBOC      KNTPIDQVNLLQLATYTSGNLALQFPDEVKTDQQVLTFFKDWKPKNSIGEYRQYSNPSIG
DC-30WP_001211218.1_1077AbBOC      KNTPIDQVNLLQLATYTSGNLALQFPDEVKTDQQVLTFFKDWKPKNSIGEYRQYSNPSIG
DC-30WP_001211218.1_1078AbBOC      KNTPIDQVNLLQLATYTSGNLALQFPDEVKTDQQVLTFFKDWKPKNSIGEYRQYSNPSIG
DC-30WP_001211218.1_1071AbBOC      KNTPIDQVNLLQLATYTSGNLALQFPDEVKTDQQVLTFFKDWKPKNSIGEYRQYSNPSIG
DC-30WP_001211218.1_2694AbBOC      KNTPIDQVNLLQLATYTSGNLALQFPDEVKTDQQVLTFFKDWKPKNSIGEYRQYSNPSIG
DC-30WP_001211218.1_3340AbBOC      KNTPIDQVNLLQLATYTSGNLALQFPDEVKTDQQVLTFFKDWKPKNSIGEYRQYSNPSIG
DC-30WP_001211218.1_1022AbBOC      KNTPIDQVNLLQLATYTSGNLALQFPDEVKTDQQVLTFFKDWKPKNSIGEYRQYSNPSIG
DC-30WP_001211218.1_1025AbBOC      KNTPIDQVNLLQLATYTSGNLALQFPDEVKTDQQVLTFFKDWKPKNSIGEYRQYSNPSIG
DC-30WP_001211218.1_1158AbBOC      KNTPIDQVNLLQLATYTSGNLALQFPDEVKTDQQVLTFFKDWKPKNSIGEYRQYSNPSIG
DC-30WP_001211218.1_1020AbBOC      KNTPIDQVNLLQLATYTSGNLALQFPDEVKTDQQVLTFFKDWKPKNSIGEYRQYSNPSIG
DC-30WP_001211218.1_1021AbBOC      KNTPIDQVNLLQLATYTSGNLALQFPDEVKTDQQVLTFFKDWKPKNSIGEYRQYSNPSIG
ADC-30WP_001211218.1_532AbBOC      KNTPIDQVNLLQLATYTSGNLALQFPDEVKTDQQVLTFFKDWKPKNSIGEYRQYSNPSIG
ADC-30WP_001211218.1_539AbBOC      KNTPIDQVNLLQLATYTSGNLALQFPDEVKTDQQVLTFFKDWKPKNSIGEYRQYSNPSIG
DC-30WP_001211218.1_1033AbBOC      KNTPIDQVNLLQLATYTSGNLALQFPDEVKTDQQVLTFFKDWKPKNSIGEYRQYSNPSIG
DC-30WP_001211218.1_1069AbBOC      KNTPIDQVNLLQLATYTSGNLALQFPDEVKTDQQVLTFFKDWKPKNSIGEYRQYSNPSIG
DC-30WP_001211218.1_1029AbBOC      KNTPIDQVNLLQLATYTSGNLALQFPDEVKTDQQVLTFFKDWKPKNSIGEYRQYSNPSIG
DC-30WP_001211218.1_1030AbBOC      KNTPIDQVNLLQLATYTSGNLALQFPDEVKTDQQVLTFFKDWKPKNSIGEYRQYSNPSIG
DC-30WP_001211218.1_1096AbBOC      KNTPIDQVNLLQLATYTSGNLALQFPDEVKTDQQVLTFFKDWKPKNSIGEYRQYSNPSIG
DC-30WP_001211218.1_1037AbBOC      KNTPIDQVNLLQLATYTSGNLALQFPDEVKTDQQVLTFFKDWKPKNSIGEYRQYSNPSIG
DC-30WP_001211218.1_1098AbBOC      KNTPIDQVNLLQLATYTSGNLALQFPDEVKTDQQVLTFFKDWKPKNSIGEYRQYSNPSIG
DC-30WP_001211218.1_1046AbBOC      KNTPIDQVNLLQLATYTSGNLALQFPDEVKTDQQVLTFFKDWKPKNSIGEYRQYSNPSIG
DC-30WP_001211218.1_1032AbBOC      KNTPIDQVNLLQLATYTSGNLALQFPDEVKTDQQVLTFFKDWKPKNSIGEYRQYSNPSIG
DC-30WP_001211218.1_1070AbBOC      KNTPIDQVNLLQLATYTSGNLALQFPDEVKTDQQVLTFFKDWKPKNSIGEYRQYSNPSIG
DC-30WP_001211218.1_1139AbBOC      KNTPIDQVNLLQLATYTSGNLALQFPDEVKTDQQVLTFFKDWKPKNSIGEYRQYSNPSIG
DC-30WP_001211218.1_1031AbBOC      KNTPIDQVNLLQLATYTSGNLALQFPDEVKTDQQVLTFFKDWKPKNSIGEYRQYSNPSIG
DC-30WP_001211218.1_1066AbBOC      KNTPIDQVNLLQLATYTSGNLALQFPDEVKTDQQVLTFFKDWKPKNSIGEYRQYSNPSIG
DC-30WP_001211218.1_1102AbBOC      KNTPIDQVNLLQLATYTSGNLALQFPDEVKTDQQVLTFFKDWKPKNSIGEYRQYSNPSIG
ADC-30USX57838.1_2957AbBOC        KNTPIDQVNLLQLATYTSGNLALQFPDEVKTDQQVLTFFKDWKPKNSIGEYRQYSNPSIG
ADC-30USX59985.1_1918AbBOC        KNTPIDQVNLLQLATYTSGNLALQFPDEVKTDQQVLTFFKDWKPKNSIGEYRQYSNPSIG
ADC-30USX63799.1_2772AbBOC        KNTPIDQVNLLQLATYTSGNLALQFPDEVKTDQQVLTFFKDWKPKNSIGEYRQYSNPSIG
ADC-30USX74012.1_305AbBOC         KNTPIDQVNLLQLATYTSGNLALQFPDEVKTDQQVLTFFKDWKPKNSIGEYRQYSNPSIG
ADC-30USZ52468.1_2397AbBOC        KNTPIDQVNLLQLATYTSGNLALQFPDEVKTDQQVLTFFKDWKPKNSIGEYRQYSNPSIG
DC-30WP_001211218.1_2008AbBOC      KNTPIDQVNLLQLATYTSGNLALQFPDEVKTDQQVLTFFKDWKPKNSIGEYRQYSNPSIG
ADC-30WCL78976.1_1026AbBOC        KNTPIDQVNLLQLATYTSGNLALQFPDEVKTDQQVLTFFKDWKPKNSIGEYRQYSNPSIG
ADC-30WDO27826.1_1034AbBOC        KNTPIDQVNLLQLATYTSGNLALQFPDEVKTDQQVLTFFKDWKPKNSIGEYRQYSNPSIG
DC-30WP_001211218.1_2125AbBOC      KNTPIDQVNLLQLATYTSGNLALQFPDEVKTDQQVLTFFKDWKPKNSIGEYRQYSNPSIG
DC-30WP_001211218.1_2729AbBOC      KNTPIDQVNLLQLATYTSGNLALQFPDEVKTDQQVLTFFKDWKPKNSIGEYRQYSNPSIG
```

```
DC-30WP_001211218.1_2692AbBCP   KNTPIDQVNLLQLATYTSGNLALQFPDEVKTDQQVLTFFKDWKPKNSIGEYRQYSNPSIG
DC-30WP_001211218.1_2549AbBCP   KNTPIDQVNLLQLATYTSGNLALQFPDEVKTDQQVLTFFKDWKPKNSIGEYRQYSNPSIG
DC-30WP_001211218.1_1026AbBCP   KNTPIDQVNLLQLATYTSGNLALQFPDEVKTDQQVLTFFKDWKPKNSIGEYRQYSNPSIG
ADC-30WP_001211218.1_373AbBCP   KNTPIDQVNLLQLATYTSGNLALQFPDEVKTDQQVLTFFKDWKPKNSIGEYRQYSNPSIG
DC-30WP_001211218.1_2465AbBCP   KNTPIDQVNLLQLATYTSGNLALQFPDEVKTDQQVLTFFKDWKPKNSIGEYRQYSNPSIG
DC-30WP_001211218.1_1027AbBCP   KNTPIDQVNLLQLATYTSGNLALQFPDEVKTDQQVLTFFKDWKPKNSIGEYRQYSNPSIG
DC-30WP_001211218.1_2650AbBCP   KNTPIDQVNLLQLATYTSGNLALQFPDEVKTDQQVLTFFKDWKPKNSIGEYRQYSNPSIG
DC-30WP_001211218.1_2674AbBCP   KNTPIDQVNLLQLATYTSGNLALQFPDEVKTDQQVLTFFKDWKPKNSIGEYRQYSNPSIG
DC-30WP_001211218.1_2689AbBCP   KNTPIDQVNLLQLATYTSGNLALQFPDEVKTDQQVLTFFKDWKPKNSIGEYRQYSNPSIG
DC-30WP_001211218.1_1105AbBCP   KNTPIDQVNLLQLATYTSGNLALQFPDEVKTDQQVLTFFKDWKPKNSIGEYRQYSNPSIG
DC-30WP_001211218.1_2670AbBCP   KNTPIDQVNLLQLATYTSGNLALQFPDEVKTDQQVLTFFKDWKPKNSIGEYRQYSNPSIG
DC-30WP_001211218.1_1019AbBCP   KNTPIDQVNLLQLATYTSGNLALQFPDEVKTDQQVLTFFKDWKPKNSIGEYRQYSNPSIG
DC-30WP_001211218.1_1039AbBCP   KNTPIDQVNLLQLATYTSGNLALQFPDEVKTDQQVLTFFKDWKPKNSIGEYRQYSNPSIG
DC-30WP_001211218.1_1023AbBCP   KNTPIDQVNLLQLATYTSGNLALQFPDEVKTDQQVLTFFKDWKPKNSIGEYRQYSNPSIG
DC-30WP_001211218.1_3189AbBCP   KNTPIDQVNLLQLATYTSGNLALQFPDEVKTDQQVLTFFKDWKPKNSIGEYRQYSNPSIG
ADC-30WP_001211218.1_594AbBCP   KNTPIDQVNLLQLATYTSGNLALQFPDEVKTDQQVLTFFKDWKPKNSIGEYRQYSNPSIG
DC-30WP_001211218.1_1718AbBCP   KNTPIDQVNLLQLATYTSGNLALQFPDEVKTDQQVLTFFKDWKPKNSIGEYRQYSNPSIG
DC-30WP_001211218.1_2484AbBCP   KNTPIDQVNLLQLATYTSGNLALQFPDEVKTDQQVLTFFKDWKPKNSIGEYRQYSNPSIG
DC-30WP_001211218.1_3305AbBCP   KNTPIDQVNLLQLATYTSGNLALQFPDEVKTDQQVLTFFKDWKPKNSIGEYRQYSNPSIG
DC-30WP_001211218.1_3346AbBCP   KNTPIDQVNLLQLATYTSGNLALQFPDEVKTDQQVLTFFKDWKPKNSIGEYRQYSNPSIG
DC-30WP_001211218.1_1482AbBCP   KNTPIDQVNLLQLATYTSGNLALQFPDEVKTDQQVLTFFKDWKPKNSIGEYRQYSNPSIG
DC-30WP_001211218.1_2574AbBCP   KNTPIDQVNLLQLATYTSGNLALQFPDEVKTDQQVLTFFKDWKPKNSIGEYRQYSNPSIG
ADC-30WP_001211218.1_831AbBCP   KNTPIDQVNLLQLATYTSGNLALQFPDEVKTDQQVLTFFKDWKPKNSIGEYRQYSNPSIG
DC-30WP_001211218.1_1826AbBCP   KNTPIDQVNLLQLATYTSGNLALQFPDEVKTDQQVLTFFKDWKPKNSIGEYRQYSNPSIG
DC-30WP_001211218.1_1024AbBCP   KNTPIDQVNLLQLATYTSGNLALQFPDEVKTDQQVLTFFKDWKPKNSIGEYRQYSNPSIG
DC-30WP_001211218.1_1618AbBCP   KNTPIDQVNLLQLATYTSGNLALQFPDEVKTDQQVLTFFKDWKPKNSIGEYRQYSNPSIG
ADC-30WP_001211218.1_301AbBCP   KNTPIDQVNLLQLATYTSGNLALQFPDEVKTDQQVLTFFKDWKPKNSIGEYRQYSNPSIG
ADC-30WP_001211218.1_940AbBCP   KNTPIDQVNLLQLATYTSGNLALQFPDEVKTDQQVLTFFKDWKPKNSIGEYRQYSNPSIG
DC-30WP_001211218.1_2949AbBCP   KNTPIDQVNLLQLATYTSGNLALQFPDEVKTDQQVLTFFKDWKPKNSIGEYRQYSNPSIG
DC-30WP_001211218.1_1028AbBCP   KNTPIDQVNLLQLATYTSGNLALQFPDEVKTDQQVLTFFKDWKPKNSIGEYRQYSNPSIG
DC-30WP_001211218.1_2526AbBCP   KNTPIDQVNLLQLATYTSGNLALQFPDEVKTDQQVLTFFKDWKPKNSIGEYRQYSNPSIG
DC-30WP_001211218.1_1040AbBCP   KNTPIDQVNLLQLATYTSGNLALQFPDEVKTDQQVLTFFKDWKPKNSIGEYRQYSNPSIG
DC-30WP_001211218.1_1089AbBCP   KNTPIDQVNLLQLATYTSGNLALQFPDEVKTDQQVLTFFKDWKPKNSIGEYRQYSNPSIG
DC-30WP_001211218.1_1099AbBCP   KNTPIDQVNLLQLATYTSGNLALQFPDEVKTDQQVLTFFKDWKPKNSIGEYRQYSNPSIG
DC-30WP_001211218.1_1206AbBCP   KNTPIDQVNLLQLATYTSGNLALQFPDEVKTDQQVLTFFKDWKPKNSIGEYRQYSNPSIG
DC-30WP_001211218.1_1167AbBCP   KNTPIDQVNLLQLATYTSGNLALQFPDEVKTDQQVLTFFKDWKPKNSIGEYRQYSNPSIG
DC-30WP_001211218.1_1186AbBCP   KNTPIDQVNLLQLATYTSGNLALQFPDEVKTDQQVLTFFKDWKPKNSIGEYRQYSNPSIG
DC-30WP_001211218.1_1165AbBCP   KNTPIDQVNLLQLATYTSGNLALQFPDEVKTDQQVLTFFKDWKPKNSIGEYRQYSNPSIG
DC-30WP_001211218.1_1077AbBCP   KNTPIDQVNLLQLATYTSGNLALQFPDEVKTDQQVLTFFKDWKPKNSIGEYRQYSNPSIG
DC-30WP_001211218.1_1078AbBCP   KNTPIDQVNLLQLATYTSGNLALQFPDEVKTDQQVLTFFKDWKPKNSIGEYRQYSNPSIG
DC-30WP_001211218.1_1071AbBCP   KNTPIDQVNLLQLATYTSGNLALQFPDEVKTDQQVLTFFKDWKPKNSIGEYRQYSNPSIG
DC-30WP_001211218.1_2694AbBCP   KNTPIDQVNLLQLATYTSGNLALQFPDEVKTDQQVLTFFKDWKPKNSIGEYRQYSNPSIG
DC-30WP_001211218.1_3340AbBCP   KNTPIDQVNLLQLATYTSGNLALQFPDEVKTDQQVLTFFKDWKPKNSIGEYRQYSNPSIG
DC-30WP_001211218.1_1022AbBCP   KNTPIDQVNLLQLATYTSGNLALQFPDEVKTDQQVLTFFKDWKPKNSIGEYRQYSNPSIG
DC-30WP_001211218.1_1025AbBCP   KNTPIDQVNLLQLATYTSGNLALQFPDEVKTDQQVLTFFKDWKPKNSIGEYRQYSNPSIG
DC-30WP_001211218.1_1158AbBCP   KNTPIDQVNLLQLATYTSGNLALQFPDEVKTDQQVLTFFKDWKPKNSIGEYRQYSNPSIG
DC-30WP_001211218.1_1020AbBCP   KNTPIDQVNLLQLATYTSGNLALQFPDEVKTDQQVLTFFKDWKPKNSIGEYRQYSNPSIG
DC-30WP_001211218.1_1021AbBCP   KNTPIDQVNLLQLATYTSGNLALQFPDEVKTDQQVLTFFKDWKPKNSIGEYRQYSNPSIG
ADC-30WP_001211218.1_532AbBCP   KNTPIDQVNLLQLATYTSGNLALQFPDEVKTDQQVLTFFKDWKPKNSIGEYRQYSNPSIG
ADC-30WP_001211218.1_539AbBCP   KNTPIDQVNLLQLATYTSGNLALQFPDEVKTDQQVLTFFKDWKPKNSIGEYRQYSNPSIG
DC-30WP_001211218.1_1033AbBCP   KNTPIDQVNLLQLATYTSGNLALQFPDEVKTDQQVLTFFKDWKPKNSIGEYRQYSNPSIG
DC-30WP_001211218.1_1069AbBCP   KNTPIDQVNLLQLATYTSGNLALQFPDEVKTDQQVLTFFKDWKPKNSIGEYRQYSNPSIG
DC-30WP_001211218.1_1029AbBCP   KNTPIDQVNLLQLATYTSGNLALQFPDEVKTDQQVLTFFKDWKPKNSIGEYRQYSNPSIG
DC-30WP_001211218.1_1030AbBCP   KNTPIDQVNLLQLATYTSGNLALQFPDEVKTDQQVLTFFKDWKPKNSIGEYRQYSNPSIG
DC-30WP_001211218.1_1096AbBCP   KNTPIDQVNLLQLATYTSGNLALQFPDEVKTDQQVLTFFKDWKPKNSIGEYRQYSNPSIG
DC-30WP_001211218.1_1037AbBCP   KNTPIDQVNLLQLATYTSGNLALQFPDEVKTDQQVLTFFKDWKPKNSIGEYRQYSNPSIG
DC-30WP_001211218.1_1098AbBCP   KNTPIDQVNLLQLATYTSGNLALQFPDEVKTDQQVLTFFKDWKPKNSIGEYRQYSNPSIG
DC-30WP_001211218.1_1046AbBCP   KNTPIDQVNLLQLATYTSGNLALQFPDEVKTDQQVLTFFKDWKPKNSIGEYRQYSNPSIG
DC-30WP_001211218.1_1032AbBCP   KNTPIDQVNLLQLATYTSGNLALQFPDEVKTDQQVLTFFKDWKPKNSIGEYRQYSNPSIG
DC-30WP_001211218.1_1070AbBCP   KNTPIDQVNLLQLATYTSGNLALQFPDEVKTDQQVLTFFKDWKPKNSIGEYRQYSNPSIG
DC-30WP_001211218.1_1139AbBCP   KNTPIDQVNLLQLATYTSGNLALQFPDEVKTDQQVLTFFKDWKPKNSIGEYRQYSNPSIG
DC-30WP_001211218.1_1031AbBCP   KNTPIDQVNLLQLATYTSGNLALQFPDEVKTDQQVLTFFKDWKPKNSIGEYRQYSNPSIG
DC-30WP_001211218.1_1066AbBCP   KNTPIDQVNLLQLATYTSGNLALQFPDEVKTDQQVLTFFKDWKPKNSIGEYRQYSNPSIG
DC-30WP_001211218.1_1102AbBCP   KNTPIDQVNLLQLATYTSGNLALQFPDEVKTDQQVLTFFKDWKPKNSIGEYRQYSNPSIG
ADC-30USX57838.1_2957AbBCP      KNTPIDQVNLLQLATYTSGNLALQFPDEVKTDQQVLTFFKDWKPKNSIGEYRQYSNPSIG
ADC-30USX59985.1_1918AbBCP      KNTPIDQVNLLQLATYTSGNLALQFPDEVKTDQQVLTFFKDWKPKNSIGEYRQYSNPSIG
ADC-30USX63799.1_2772AbBCP      KNTPIDQVNLLQLATYTSGNLALQFPDEVKTDQQVLTFFKDWKPKNSIGEYRQYSNPSIG
ADC-30USX74012.1_305AbBCP       KNTPIDQVNLLQLATYTSGNLALQFPDEVKTDQQVLTFFKDWKPKNSIGEYRQYSNPSIG
ADC-30USZ52468.1_2397AbBCP      KNTPIDQVNLLQLATYTSGNLALQFPDEVKTDQQVLTFFKDWKPKNSIGEYRQYSNPSIG
DC-30WP_001211218.1_2008AbBCP   KNTPIDQVNLLQLATYTSGNLALQFPDEVKTDQQVLTFFKDWKPKNSIGEYRQYSNPSIG
ADC-30WCL78976.1_1026AbBCP      KNTPIDQVNLLQLATYTSGNLALQFPDEVKTDQQVLTFFKDWKPKNSIGEYRQYSNPSIG
ADC-30WDO27826.1_1034AbBCP      KNTPIDQVNLLQLATYTSGNLALQFPDEVKTDQQVLTFFKDWKPKNSIGEYRQYSNPSIG
DC-30WP_001211218.1_2125AbBCP   KNTPIDQVNLLQLATYTSGNLALQFPDEVKTDQQVLTFFKDWKPKNSIGEYRQYSNPSIG
DC-30WP_001211218.1_2729AbBCP   KNTPIDQVNLLQLATYTSGNLALQFPDEVKTDQQVLTFFKDWKPKNSIGEYRQYSNPSIG
ADC-30ADX93237.1_2806AbBCP      KNTPIDQVNLLQLATYTSGNLALQFPDEVKTDQQVLTFFKDWKPKNSIGEYRQYSNPSIG
ADC-82WP_001211216.1_2747AbOC   KNTPIDQVNLLQLATYTSGNLALQFPDEVKTDQQVLTFFKDWKPKNSIGEYRQYSNPSIG
DC-82WP_001211216.1_1093AbBOC   KNTPIDQVNLLQLATYTSGNLALQFPDEVKTDQQVLTFFKDWKPKNSIGEYRQYSNPSIG
DC-82WP_001211216.1_1071AbBOC   KNTPIDQVNLLQLATYTSGNLALQFPDEVKTDQQVLTFFKDWKPKNSIGEYRQYSNPSIG
DC-82WP_001211216.1_1093AbBCP   KNTPIDQVNLLQLATYTSGNLALQFPDEVKTDQQVLTFFKDWKPKNSIGEYRQYSNPSIG
DC-82WP_001211216.1_1071AbBCP   KNTPIDQVNLLQLATYTSGNLALQFPDEVKTDQQVLTFFKDWKPKNSIGEYRQYSNPSIG
DC-162WP_031980335.1_1153AbOC   KNTPIDQVNLLQLATYTSGNLALQFPDEVKTDQQVLTFFKDWKPKNSIGEYRQYSNPSIG
C-162WP_031980335.1_1028AbBOC   KNTPIDQVNLLQLATYTSGNLALQFPDEVKTDQQVLTFFKDWKPKNSIGEYRQYSNPSIG
C-162WP_031980335.1_1023AbBOC   KNTPIDQVNLLQLATYTSGNLALQFPDEVKTDQQVLTFFKDWKPKNSIGEYRQYSNPSIG
C-162WP_031980335.1_1028AbBCP   KNTPIDQVNLLQLATYTSGNLALQFPDEVKTDQQVLTFFKDWKPKNSIGEYRQYSNPSIG
C-162WP_031980335.1_1023AbBCP   KNTPIDQVNLLQLATYTSGNLALQFPDEVKTDQQVLTFFKDWKPKNSIGEYRQYSNPSIG
ADC-73WP_001211219.1_1012AbOC   KNTPIDQVNLLQLATYTSGNLALQFPDEVKTDQQVLTFFKDWKPKNSIGEYRQYSNPSIG
ADC-73WP_001211219.1_1953AbOC   KNTPIDQVNLLQLATYTSGNLALQFPDEVKTDQQVLTFFKDWKPKNSIGEYRQYSNPSIG
ADC-73WP_001211219.1_1014AbOC   KNTPIDQVNLLQLATYTSGNLALQFPDEVKTDQQVLTFFKDWKPKNSIGEYRQYSNPSIG
ADC-73WP_001211219.1_1041AbOC   KNTPIDQVNLLQLATYTSGNLALQFPDEVKTDQQVLTFFKDWKPKNSIGEYRQYSNPSIG
ADC-73WP_001211219.1_1028AbOC   KNTPIDQVNLLQLATYTSGNLALQFPDEVKTDQQVLTFFKDWKPKNSIGEYRQYSNPSIG
ADC-73WP_001211219.1_2674AbOC   KNTPIDQVNLLQLATYTSGNLALQFPDEVKTDQQVLTFFKDWKPKNSIGEYRQYSNPSIG
ADC-73WP_001211219.1_462AbOC    KNTPIDQVNLLQLATYTSGNLALQFPDEVKTDQQVLTFFKDWKPKNSIGEYRQYSNPSIG
ADC-73WP_001211219.1_3530AbOC   KNTPIDQVNLLQLATYTSGNLALQFPDEVKTDQQVLTFFKDWKPKNSIGEYRQYSNPSIG
ADC-73WP_001211219.1_3621AbOC   KNTPIDQVNLLQLATYTSGNLALQFPDEVKTDQQVLTFFKDWKPKNSIGEYRQYSNPSIG
ADC-73WP_001211219.1_3381AbOC   KNTPIDQVNLLQLATYTSGNLALQFPDEVKTDQQVLTFFKDWKPKNSIGEYRQYSNPSIG
ADC-73WP_001211219.1_2515AbOC   KNTPIDQVNLLQLATYTSGNLALQFPDEVKTDQQVLTFFKDWKPKNSIGEYRQYSNPSIG
ADC-73WP_001211219.1_3574AbOC   KNTPIDQVNLLQLATYTSGNLALQFPDEVKTDQQVLTFFKDWKPKNSIGEYRQYSNPSIG
ADC-73WP_001211219.1_1656AbOC   KNTPIDQVNLLQLATYTSGNLALQFPDEVKTDQQVLTFFKDWKPKNSIGEYRQYSNPSIG
ADC-73WP_001211219.1_2591AbOC   KNTPIDQVNLLQLATYTSGNLALQFPDEVKTDQQVLTFFKDWKPKNSIGEYRQYSNPSIG
ADC-73WP_001211219.1_2602AbOC   KNTPIDQVNLLQLATYTSGNLALQFPDEVKTDQQVLTFFKDWKPKNSIGEYRQYSNPSIG
ADC-73WP_001211219.1_910AbOC    KNTPIDQVNLLQLATYTSGNLALQFPDEVKTDQQVLTFFKDWKPKNSIGEYRQYSNPSIG
ADC-73WP_001211219.1_394AbOC    KNTPIDQVNLLQLATYTSGNLALQFPDEVKTDQQVLTFFKDWKPKNSIGEYRQYSNPSIG
ADC-73WP_001211219.1_1030AbOC   KNTPIDQVNLLQLATYTSGNLALQFPDEVKTDQQVLTFFKDWKPKNSIGEYRQYSNPSIG
ADC-73WP_001211219.1_1023AbOC   KNTPIDQVNLLQLATYTSGNLALQFPDEVKTDQQVLTFFKDWKPKNSIGEYRQYSNPSIG
ADC-73UYQ66995.1_1967AbOC       KNTPIDQVNLLQLATYTSGNLALQFPDEVKTDQQVLTFFKDWKPKNSIGEYRQYSNPSIG
ADC-73WP_001211219.1_906AbBOC   KNTPIDQVNLLQLATYTSGNLALQFPDEVKTDQQVLTFFKDWKPKNSIGEYRQYSNPSIG
DC-73WP_001211219.1_2649AbBOC   KNTPIDQVNLLQLATYTSGNLALQFPDEVKTDQQVLTFFKDWKPKNSIGEYRQYSNPSIG
DC-73WP_001211219.1_2693AbBOC   KNTPIDQVNLLQLATYTSGNLALQFPDEVKTDQQVLTFFKDWKPKNSIGEYRQYSNPSIG
ADC-73AQU57925.1_2703AbBOC      KNTPIDQVNLLQLATYTSGNLALQFPDEVKTDQQVLTFFKDWKPKNSIGEYRQYSNPSIG
DC-73WP_001211219.1_1026AbBOC   KNTPIDQVNLLQLATYTSGNLALQFPDEVKTDQQVLTFFKDWKPKNSIGEYRQYSNPSIG
DC-73WP_001211219.1_1019AbBOC   KNTPIDQVNLLQLATYTSGNLALQFPDEVKTDQQVLTFFKDWKPKNSIGEYRQYSNPSIG
DC-73WP_001211219.1_1024AbBOC   KNTPIDQVNLLQLATYTSGNLALQFPDEVKTDQQVLTFFKDWKPKNSIGEYRQYSNPSIG
ADC-73WP_001211219.1_930AbBOC   KNTPIDQVNLLQLATYTSGNLALQFPDEVKTDQQVLTFFKDWKPKNSIGEYRQYSNPSIG
DC-73WP_001211219.1_2609AbBOC   KNTPIDQVNLLQLATYTSGNLALQFPDEVKTDQQVLTFFKDWKPKNSIGEYRQYSNPSIG
DC-73WP_001211219.1_3456AbBOC   KNTPIDQVNLLQLATYTSGNLALQFPDEVKTDQQVLTFFKDWKPKNSIGEYRQYSNPSIG
DC-73WP_001211219.1_2614AbBOC   KNTPIDQVNLLQLATYTSGNLALQFPDEVKTDQQVLTFFKDWKPKNSIGEYRQYSNPSIG
DC-73WP_001211219.1_1025AbBOC   KNTPIDQVNLLQLATYTSGNLALQFPDEVKTDQQVLTFFKDWKPKNSIGEYRQYSNPSIG
DC-73WP_001211219.1_1030AbBOC   KNTPIDQVNLLQLATYTSGNLALQFPDEVKTDQQVLTFFKDWKPKNSIGEYRQYSNPSIG
DC-73WP_001211219.1_1173AbBOC   KNTPIDQVNLLQLATYTSGNLALQFPDEVKTDQQVLTFFKDWKPKNSIGEYRQYSNPSIG
DC-73WP_001211219.1_1021AbBOC   KNTPIDQVNLLQLATYTSGNLALQFPDEVKTDQQVLTFFKDWKPKNSIGEYRQYSNPSIG
DC-73WP_001211219.1_2449AbBOC   KNTPIDQVNLLQLATYTSGNLALQFPDEVKTDQQVLTFFKDWKPKNSIGEYRQYSNPSIG
DC-73WP_001211219.1_1176AbBOC   KNTPIDQVNLLQLATYTSGNLALQFPDEVKTDQQVLTFFKDWKPKNSIGEYRQYSNPSIG
DC-73WP_001211219.1_1161AbBOC   KNTPIDQVNLLQLATYTSGNLALQFPDEVKTDQQVLTFFKDWKPKNSIGEYRQYSNPSIG
DC-73WP_001211219.1_1070AbBOC   KNTPIDQVNLLQLATYTSGNLALQFPDEVKTDQQVLTFFKDWKPKNSIGEYRQYSNPSIG
DC-73WP_001211219.1_1079AbBOC   KNTPIDQVNLLQLATYTSGNLALQFPDEVKTDQQVLTFFKDWKPKNSIGEYRQYSNPSIG
DC-73WP_001211219.1_1078AbBOC   KNTPIDQVNLLQLATYTSGNLALQFPDEVKTDQQVLTFFKDWKPKNSIGEYRQYSNPSIG
DC-73WP_001211219.1_1037AbBOC   KNTPIDQVNLLQLATYTSGNLALQFPDEVKTDQQVLTFFKDWKPKNSIGEYRQYSNPSIG
DC-73WP_001211219.1_1035AbBOC   KNTPIDQVNLLQLATYTSGNLALQFPDEVKTDQQVLTFFKDWKPKNSIGEYRQYSNPSIG
DC-73WP_001211219.1_1033AbBOC   KNTPIDQVNLLQLATYTSGNLALQFPDEVKTDQQVLTFFKDWKPKNSIGEYRQYSNPSIG
DC-73WP_001211219.1_1041AbBOC   KNTPIDQVNLLQLATYTSGNLALQFPDEVKTDQQVLTFFKDWKPKNSIGEYRQYSNPSIG
DC-73WP_001211219.1_1023AbBOC   KNTPIDQVNLLQLATYTSGNLALQFPDEVKTDQQVLTFFKDWKPKNSIGEYRQYSNPSIG
ADC-73USX69547.1_2386AbBOC      KNTPIDQVNLLQLATYTSGNLALQFPDEVKTDQQVLTFFKDWKPKNSIGEYRQYSNPSIG
DC-73WP_001211219.1_1034AbBOC   KNTPIDQVNLLQLATYTSGNLALQFPDEVKTDQQVLTFFKDWKPKNSIGEYRQYSNPSIG
ADC-73WP_001211219.1_852AbBOC   KNTPIDQVNLLQLATYTSGNLALQFPDEVKTDQQVLTFFKDWKPKNSIGEYRQYSNPSIG
DC-73WP_001211219.1_3070AbBOC   KNTPIDQVNLLQLATYTSGNLALQFPDEVKTDQQVLTFFKDWKPKNSIGEYRQYSNPSIG
ADC-73WBS60953.1_1026AbBOC      KNTPIDQVNLLQLATYTSGNLALQFPDEVKTDQQVLTFFKDWKPKNSIGEYRQYSNPSIG
ADC-73WDC39660.1_1023AbBOC      KNTPIDQVNLLQLATYTSGNLALQFPDEVKTDQQVLTFFKDWKPKNSIGEYRQYSNPSIG
ADC-73WDC73972.1_1415AbBOC      KNTPIDQVNLLQLATYTSGNLALQFPDEVKTDQQVLTFFKDWKPKNSIGEYRQYSNPSIG
ADC-73WDC70172.1_1037AbBOC      KNTPIDQVNLLQLATYTSGNLALQFPDEVKTDQQVLTFFKDWKPKNSIGEYRQYSNPSIG
DC-73WP_001211219.1_1101AbBOC   KNTPIDQVNLLQLATYTSGNLALQFPDEVKTDQQVLTFFKDWKPKNSIGEYRQYSNPSIG
DC-73WP_001211219.1_1667AbBOC   KNTPIDQVNLLQLATYTSGNLALQFPDEVKTDQQVLTFFKDWKPKNSIGEYRQYSNPSIG
ADC-73WP_001211219.1_287AbBOC   KNTPIDQVNLLQLATYTSGNLALQFPDEVKTDQQVLTFFKDWKPKNSIGEYRQYSNPSIG
DC-73WP_001211219.1_3177AbBOC   KNTPIDQVNLLQLATYTSGNLALQFPDEVKTDQQVLTFFKDWKPKNSIGEYRQYSNPSIG
DC-73WP_001211219.1_2501AbBOC   KNTPIDQVNLLQLATYTSGNLALQFPDEVKTDQQVLTFFKDWKPKNSIGEYRQYSNPSIG
DC-73WP_001211219.1_2535AbBOC   KNTPIDQVNLLQLATYTSGNLALQFPDEVKTDQQVLTFFKDWKPKNSIGEYRQYSNPSIG
DC-73WP_001211219.1_3150AbBOC   KNTPIDQVNLLQLATYTSGNLALQFPDEVKTDQQVLTFFKDWKPKNSIGEYRQYSNPSIG
DC-73WP_001211219.1_3714AbBOC   KNTPIDQVNLLQLATYTSGNLALQFPDEVKTDQQVLTFFKDWKPKNSIGEYRQYSNPSIG
ADC-73WP_001211219.1_722AbBOC   KNTPIDQVNLLQLATYTSGNLALQFPDEVKTDQQVLTFFKDWKPKNSIGEYRQYSNPSIG
DC-73WP_001211219.1_3650AbBOC   KNTPIDQVNLLQLATYTSGNLALQFPDEVKTDQQVLTFFKDWKPKNSIGEYRQYSNPSIG
ADC-73WP_001211219.1_568AbBOC   KNTPIDQVNLLQLATYTSGNLALQFPDEVKTDQQVLTFFKDWKPKNSIGEYRQYSNPSIG
DC-73WP_001211219.1_3226AbBOC   KNTPIDQVNLLQLATYTSGNLALQFPDEVKTDQQVLTFFKDWKPKNSIGEYRQYSNPSIG
ADC-73WP_001211219.1_556AbBOC   KNTPIDQVNLLQLATYTSGNLALQFPDEVKTDQQVLTFFKDWKPKNSIGEYRQYSNPSIG
```

```
DC-73WP_001211219.1_2436AbBOC     KNTPIDQVNLLQLATYTSGNLALQFPDEVKTDQQVLTFFKDWKPKNSIGEYRQYSNPSIG
DC-73WP_001211219.1_1029AbBOC     KNTPIDQVNLLQLATYTSGNLALQFPDEVKTDQQVLTFFKDWKPKNSIGEYRQYSNPSIG
DC-73WP_001211219.1_1013AbBOC     KNTPIDQVNLLQLATYTSGNLALQFPDEVKTDQQVLTFFKDWKPKNSIGEYRQYSNPSIG
ADC-73WP_001211219.1_906AbBCP     KNTPIDQVNLLQLATYTSGNLALQFPDEVKTDQQVLTFFKDWKPKNSIGEYRQYSNPSIG
DC-73WP_001211219.1_2649AbBCP     KNTPIDQVNLLQLATYTSGNLALQFPDEVKTDQQVLTFFKDWKPKNSIGEYRQYSNPSIG
DC-73WP_001211219.1_2693AbBCP     KNTPIDQVNLLQLATYTSGNLALQFPDEVKTDQQVLTFFKDWKPKNSIGEYRQYSNPSIG
ADC-73AQU57925.1_2703AbBCP        KNTPIDQVNLLQLATYTSGNLALQFPDEVKTDQQVLTFFKDWKPKNSIGEYRQYSNPSIG
DC-73WP_001211219.1_1026AbBCP     KNTPIDQVNLLQLATYTSGNLALQFPDEVKTDQQVLTFFKDWKPKNSIGEYRQYSNPSIG
DC-73WP_001211219.1_1019AbBCP     KNTPIDQVNLLQLATYTSGNLALQFPDEVKTDQQVLTFFKDWKPKNSIGEYRQYSNPSIG
DC-73WP_001211219.1_1024AbBCP     KNTPIDQVNLLQLATYTSGNLALQFPDEVKTDQQVLTFFKDWKPKNSIGEYRQYSNPSIG
ADC-73WP_001211219.1_930AbBCP     KNTPIDQVNLLQLATYTSGNLALQFPDEVKTDQQVLTFFKDWKPKNSIGEYRQYSNPSIG
DC-73WP_001211219.1_2609AbBCP     KNTPIDQVNLLQLATYTSGNLALQFPDEVKTDQQVLTFFKDWKPKNSIGEYRQYSNPSIG
DC-73WP_001211219.1_3456AbBCP     KNTPIDQVNLLQLATYTSGNLALQFPDEVKTDQQVLTFFKDWKPKNSIGEYRQYSNPSIG
DC-73WP_001211219.1_2614AbBCP     KNTPIDQVNLLQLATYTSGNLALQFPDEVKTDQQVLTFFKDWKPKNSIGEYRQYSNPSIG
DC-73WP_001211219.1_1025AbBCP     KNTPIDQVNLLQLATYTSGNLALQFPDEVKTDQQVLTFFKDWKPKNSIGEYRQYSNPSIG
DC-73WP_001211219.1_1030AbBCP     KNTPIDQVNLLQLATYTSGNLALQFPDEVKTDQQVLTFFKDWKPKNSIGEYRQYSNPSIG
DC-73WP_001211219.1_1173AbBCP     KNTPIDQVNLLQLATYTSGNLALQFPDEVKTDQQVLTFFKDWKPKNSIGEYRQYSNPSIG
DC-73WP_001211219.1_1021AbBCP     KNTPIDQVNLLQLATYTSGNLALQFPDEVKTDQQVLTFFKDWKPKNSIGEYRQYSNPSIG
DC-73WP_001211219.1_2449AbBCP     KNTPIDQVNLLQLATYTSGNLALQFPDEVKTDQQVLTFFKDWKPKNSIGEYRQYSNPSIG
DC-73WP_001211219.1_1176AbBCP     KNTPIDQVNLLQLATYTSGNLALQFPDEVKTDQQVLTFFKDWKPKNSIGEYRQYSNPSIG
DC-73WP_001211219.1_1161AbBCP     KNTPIDQVNLLQLATYTSGNLALQFPDEVKTDQQVLTFFKDWKPKNSIGEYRQYSNPSIG
DC-73WP_001211219.1_1070AbBCP     KNTPIDQVNLLQLATYTSGNLALQFPDEVKTDQQVLTFFKDWKPKNSIGEYRQYSNPSIG
DC-73WP_001211219.1_1079AbBCP     KNTPIDQVNLLQLATYTSGNLALQFPDEVKTDQQVLTFFKDWKPKNSIGEYRQYSNPSIG
DC-73WP_001211219.1_1078AbBCP     KNTPIDQVNLLQLATYTSGNLALQFPDEVKTDQQVLTFFKDWKPKNSIGEYRQYSNPSIG
DC-73WP_001211219.1_1037AbBCP     KNTPIDQVNLLQLATYTSGNLALQFPDEVKTDQQVLTFFKDWKPKNSIGEYRQYSNPSIG
DC-73WP_001211219.1_1035AbBCP     KNTPIDQVNLLQLATYTSGNLALQFPDEVKTDQQVLTFFKDWKPKNSIGEYRQYSNPSIG
DC-73WP_001211219.1_1033AbBCP     KNTPIDQVNLLQLATYTSGNLALQFPDEVKTDQQVLTFFKDWKPKNSIGEYRQYSNPSIG
DC-73WP_001211219.1_1041AbBCP     KNTPIDQVNLLQLATYTSGNLALQFPDEVKTDQQVLTFFKDWKPKNSIGEYRQYSNPSIG
DC-73WP_001211219.1_1023AbBCP     KNTPIDQVNLLQLATYTSGNLALQFPDEVKTDQQVLTFFKDWKPKNSIGEYRQYSNPSIG
ADC-73USX69547.1_2386AbBCP        KNTPIDQVNLLQLATYTSGNLALQFPDEVKTDQQVLTFFKDWKPKNSIGEYRQYSNPSIG
DC-73WP_001211219.1_1034AbBCP     KNTPIDQVNLLQLATYTSGNLALQFPDEVKTDQQVLTFFKDWKPKNSIGEYRQYSNPSIG
ADC-73WP_001211219.1_852AbBCP     KNTPIDQVNLLQLATYTSGNLALQFPDEVKTDQQVLTFFKDWKPKNSIGEYRQYSNPSIG
DC-73WP_001211219.1_3070AbBCP     KNTPIDQVNLLQLATYTSGNLALQFPDEVKTDQQVLTFFKDWKPKNSIGEYRQYSNPSIG
ADC-73WBS60953.1_1026AbBCP        KNTPIDQVNLLQLATYTSGNLALQFPDEVKTDQQVLTFFKDWKPKNSIGEYRQYSNPSIG
ADC-73WDC39660.1_1023AbBCP        KNTPIDQVNLLQLATYTSGNLALQFPDEVKTDQQVLTFFKDWKPKNSIGEYRQYSNPSIG
ADC-73WDC73972.1_1415AbBCP        KNTPIDQVNLLQLATYTSGNLALQFPDEVKTDQQVLTFFKDWKPKNSIGEYRQYSNPSIG
ADC-73WDC70172.1_1037AbBCP        KNTPIDQVNLLQLATYTSGNLALQFPDEVKTDQQVLTFFKDWKPKNSIGEYRQYSNPSIG
DC-73WP_001211219.1_1101AbBCP     KNTPIDQVNLLQLATYTSGNLALQFPDEVKTDQQVLTFFKDWKPKNSIGEYRQYSNPSIG
DC-73WP_001211219.1_1667AbBCP     KNTPIDQVNLLQLATYTSGNLALQFPDEVKTDQQVLTFFKDWKPKNSIGEYRQYSNPSIG
ADC-73WP_001211219.1_287AbBCP     KNTPIDQVNLLQLATYTSGNLALQFPDEVKTDQQVLTFFKDWKPKNSIGEYRQYSNPSIG
DC-73WP_001211219.1_3177AbBCP     KNTPIDQVNLLQLATYTSGNLALQFPDEVKTDQQVLTFFKDWKPKNSIGEYRQYSNPSIG
DC-73WP_001211219.1_2501AbBCP     KNTPIDQVNLLQLATYTSGNLALQFPDEVKTDQQVLTFFKDWKPKNSIGEYRQYSNPSIG
DC-73WP_001211219.1_2535AbBCP     KNTPIDQVNLLQLATYTSGNLALQFPDEVKTDQQVLTFFKDWKPKNSIGEYRQYSNPSIG
DC-73WP_001211219.1_3150AbBCP     KNTPIDQVNLLQLATYTSGNLALQFPDEVKTDQQVLTFFKDWKPKNSIGEYRQYSNPSIG
DC-73WP_001211219.1_3714AbBCP     KNTPIDQVNLLQLATYTSGNLALQFPDEVKTDQQVLTFFKDWKPKNSIGEYRQYSNPSIG
ADC-73WP_001211219.1_722AbBCP     KNTPIDQVNLLQLATYTSGNLALQFPDEVKTDQQVLTFFKDWKPKNSIGEYRQYSNPSIG
DC-73WP_001211219.1_3650AbBCP     KNTPIDQVNLLQLATYTSGNLALQFPDEVKTDQQVLTFFKDWKPKNSIGEYRQYSNPSIG
ADC-73WP_001211219.1_568AbBCP     KNTPIDQVNLLQLATYTSGNLALQFPDEVKTDQQVLTFFKDWKPKNSIGEYRQYSNPSIG
DC-73WP_001211219.1_3226AbBCP     KNTPIDQVNLLQLATYTSGNLALQFPDEVKTDQQVLTFFKDWKPKNSIGEYRQYSNPSIG
ADC-73WP_001211219.1_556AbBCP     KNTPIDQVNLLQLATYTSGNLALQFPDEVKTDQQVLTFFKDWKPKNSIGEYRQYSNPSIG
DC-73WP_001211219.1_2436AbBCP     KNTPIDQVNLLQLATYTSGNLALQFPDEVKTDQQVLTFFKDWKPKNSIGEYRQYSNPSIG
DC-73WP_001211219.1_1029AbBCP     KNTPIDQVNLLQLATYTSGNLALQFPDEVKTDQQVLTFFKDWKPKNSIGEYRQYSNPSIG
DC-73WP_001211219.1_1013AbBCP     KNTPIDQVNLLQLATYTSGNLALQFPDEVKTDQQVLTFFKDWKPKNSIGEYRQYSNPSIG
ADC-73BDE18748.1_1038AbBCP        KNTPIDQVNLLQLATYTSGNLALQFPDEVKTDQQVLTFFKDWKPKNSIGEYRQYSNPSIG
DC-115WP_001211215.1_2833AbOC     KNTPIDQVNLLQLATYTSGNLALQFPDEVKTDQQVLTFFKDWKPKNSIGEYRQYSNPSIG
C-115WP_001211215.1_1156AbBOC     KNTPIDQVNLLQLATYTSGNLALQFPDEVKTDQQVLTFFKDWKPKNSIGEYRQYSNPSIG
C-115WP_001211215.1_1156AbBCP     KNTPIDQVNLLQLATYTSGNLALQFPDEVKTDQQVLTFFKDWKPKNSIGEYRQYSNPSIG
ADC-5WP_038405930.1_2542AbOC      KNTPIDQVNLLQLATYTSGNLALQFPDEVQTDQQVLTFFKDWQPKNPIGEYRQYSNPSIG
ADC-5WP_038405930.1_1113AbBOC     KNTPIDQVNLLQLATYTSGNLALQFPDEVQTDQQVLTFFKDWQPKNPIGEYRQYSNPSIG
ADC-5WP_038405930.1_1113AbBCP     KNTPIDQVNLLQLATYTSGNLALQFPDEVQTDQQVLTFFKDWQPKNPIGEYRQYSNPSIG
C-236WP_176673601.1_1026AbBOC     KNTSIDQVNLLQLATYTSGNLALRFPDEVQTDQQVLTFFKDWQPKNPIGEYRQYSNPSIG
C-236WP_176673601.1_1026AbBCP     KNTSIDQVNLLQLATYTSGNLALRFPDEVQTDQQVLTFFKDWQPKNPIGEYRQYSNPSIG
DC-217WP_060470109.1_3584AbOC     KNTPIDQVNLLQLATYTSGNLALQFPDEVKTDQQVLTFFKDWKPKNSIGEYRQYSNPSIG
ADC-217USX66614.1_2290AbBOC       KNTPIDQVNLLQLATYTSGNLALQFPDEVKTDQQVLTFFKDWKPKNSIGEYRQYSNPSIG
ADC-217USX66614.1_2290AbBCP       KNTPIDQVNLLQLATYTSGNLALQFPDEVKTDQQVLTFFKDWKPKNSIGEYRQYSNPSIG
ADC-247WP_032058755.1_382AbOC     KNTPIDQVNLLQLATYTSGNLALQFPDEVQTDQQVLTFFKDWQPKNPIGEYRQYSNPSIG
C-247WP_032058755.1_1936AbBOC     KNTPIDQVNLLQLATYTSGNLALQFPDEVQTDQQVLTFFKDWQPKNPIGEYRQYSNPSIG
C-247WP_032058755.1_1936AbBCP     KNTPIDQVNLLQLATYTSGNLALQFPDEVQTDQQVLTFFKDWQPKNPIGEYRQYSNPSIG
DC-203WP_032000414.1_2940AbOC     KNTPIDQVNLLQLATYTSGNLALQFPDEVQTDQQVLTFFKDWKPKNPIGEYRQYSNPSIG
C-203WP_032000414.1_1062AbBOC     KNTPIDQVNLLQLATYTSGNLALQFPDEVQTDQQVLTFFKDWKPKNPIGEYRQYSNPSIG
DC-203WP_032000414.1_129AbBOC     KNTPIDQVNLLQLATYTSGNLALQFPDEVQTDQQVLTFFKDWKPKNPIGEYRQYSNPSIG
C-203WP_032000414.1_2518AbBOC     KNTPIDQVNLLQLATYTSGNLALQFPDEVQTDQQVLTFFKDWKPKNPIGEYRQYSNPSIG
C-203WP_032000414.1_1062AbBCP     KNTPIDQVNLLQLATYTSGNLALQFPDEVQTDQQVLTFFKDWKPKNPIGEYRQYSNPSIG
DC-203WP_032000414.1_129AbBCP     KNTPIDQVNLLQLATYTSGNLALQFPDEVQTDQQVLTFFKDWKPKNPIGEYRQYSNPSIG
C-203WP_032000414.1_2518AbBCP     KNTPIDQVNLLQLATYTSGNLALQFPDEVQTDQQVLTFFKDWKPKNPIGEYRQYSNPSIG
DC-152WP_001211233.1_3734AbOC     KNTSIDQVNLLQLATYTSGNLALQFPDEVQTDQQVLTFFKDWQPKNPIGEYRQYSNPSIG
C-152WP_001211233.1_1080AbBOC     KNTSIDQVNLLQLATYTSGNLALQFPDEVQTDQQVLTFFKDWQPKNPIGEYRQYSNPSIG
DC-152WP_001211233.1_491AbBOC     KNTSIDQVNLLQLATYTSGNLALQFPDEVQTDQQVLTFFKDWQPKNPIGEYRQYSNPSIG
ADC-152WP_001211233.1_46AbBOC     KNTSIDQVNLLQLATYTSGNLALQFPDEVQTDQQVLTFFKDWQPKNPIGEYRQYSNPSIG
C-152WP_001211233.1_1545AbBOC     KNTSIDQVNLLQLATYTSGNLALQFPDEVQTDQQVLTFFKDWQPKNPIGEYRQYSNPSIG
ADC-152WBS86980.1_1289AbBOC       KNTSIDQVNLLQLATYTSGNLALQFPDEVQTDQQVLTFFKDWQPKNPIGEYRQYSNPSIG
ADC-152WBS81072.1_2581AbBOC       KNTSIDQVNLLQLATYTSGNLALQFPDEVQTDQQVLTFFKDWQPKNPIGEYRQYSNPSIG
ADC-152WBS88504.1_2711AbBOC       KNTSIDQVNLLQLATYTSGNLALQFPDEVQTDQQVLTFFKDWQPKNPIGEYRQYSNPSIG
ADC-152WBS92168.1_2401AbBOC       KNTSIDQVNLLQLATYTSGNLALQFPDEVQTDQQVLTFFKDWQPKNPIGEYRQYSNPSIG
ADC-152WBS79617.1_1289AbBOC       KNTSIDQVNLLQLATYTSGNLALQFPDEVQTDQQVLTFFKDWQPKNPIGEYRQYSNPSIG
C-152WP_001211233.1_1080AbBCP     KNTSIDQVNLLQLATYTSGNLALQFPDEVQTDQQVLTFFKDWQPKNPIGEYRQYSNPSIG
DC-152WP_001211233.1_491AbBCP     KNTSIDQVNLLQLATYTSGNLALQFPDEVQTDQQVLTFFKDWQPKNPIGEYRQYSNPSIG
ADC-152WP_001211233.1_46AbBCP     KNTSIDQVNLLQLATYTSGNLALQFPDEVQTDQQVLTFFKDWQPKNPIGEYRQYSNPSIG
C-152WP_001211233.1_1545AbBCP     KNTSIDQVNLLQLATYTSGNLALQFPDEVQTDQQVLTFFKDWQPKNPIGEYRQYSNPSIG
ADC-152WBS86980.1_1289AbBCP       KNTSIDQVNLLQLATYTSGNLALQFPDEVQTDQQVLTFFKDWQPKNPIGEYRQYSNPSIG
ADC-152WBS81072.1_2581AbBCP       KNTSIDQVNLLQLATYTSGNLALQFPDEVQTDQQVLTFFKDWQPKNPIGEYRQYSNPSIG
ADC-152WBS88504.1_2711AbBCP       KNTSIDQVNLLQLATYTSGNLALQFPDEVQTDQQVLTFFKDWQPKNPIGEYRQYSNPSIG
ADC-152WBS92168.1_2401AbBCP       KNTSIDQVNLLQLATYTSGNLALQFPDEVQTDQQVLTFFKDWQPKNPIGEYRQYSNPSIG
ADC-152WBS79617.1_1289AbBCP       KNTSIDQVNLLQLATYTSGNLALQFPDEVQTDQQVLTFFKDWQPKNPIGEYRQYSNPSIG
ADC-52WP_001211232.1_990AbOC      KNTSIDQVNLLQLATYTSGNLALQFPDEVQTDQQVLTFFKDWQPKNPIGEYRQYSNPSIG
DC-52WP_001211232.1_1087AbBOC     KNTSIDQVNLLQLATYTSGNLALQFPDEVQTDQQVLTFFKDWQPKNPIGEYRQYSNPSIG
DC-52WP_001211232.1_1087AbBCP     KNTSIDQVNLLQLATYTSGNLALQFPDEVQTDQQVLTFFKDWQPKNPIGEYRQYSNPSIG
C-163WP_057093064.1_2397AbBOC     KNTPIDQVNLLQLATYTSGNLALQFPDEVQTDQQVLTFFKDWKPKNPIGEYRQYSNPSIG
C-163WP_057093064.1_2397AbBCP     KNTPIDQVNLLQLATYTSGNLALQFPDEVQTDQQVLTFFKDWKPKNPIGEYRQYSNPSIG
ADC-33WP_001211220.1_2277AbOC     KNTPIDQVNLLQLATYTSGNLALQFPDEVKTDQQVLTFFKDWKPKNSIGEYRQYSNPSIG
DC-33WP_001211220.1_1044AbBOC     KNTPIDQVNLLQLATYTSGNLALQFPDEVKTDQQVLTFFKDWKPKNSIGEYRQYSNPSIG
DC-33WP_001211220.1_2452AbBOC     KNTPIDQVNLLQLATYTSGNLALQFPDEVKTDQQVLTFFKDWKPKNSIGEYRQYSNPSIG
DC-33WP_001211220.1_1046AbBOC     KNTPIDQVNLLQLATYTSGNLALQFPDEVKTDQQVLTFFKDWKPKNSIGEYRQYSNPSIG
DC-33WP_001211220.1_1025AbBOC     KNTPIDQVNLLQLATYTSGNLALQFPDEVKTDQQVLTFFKDWKPKNSIGEYRQYSNPSIG
DC-33WP_001211220.1_1026AbBOC     KNTPIDQVNLLQLATYTSGNLALQFPDEVKTDQQVLTFFKDWKPKNSIGEYRQYSNPSIG
DC-33WP_001211220.1_1033AbBOC     KNTPIDQVNLLQLATYTSGNLALQFPDEVKTDQQVLTFFKDWKPKNSIGEYRQYSNPSIG
DC-33WP_001211220.1_1035AbBOC     KNTPIDQVNLLQLATYTSGNLALQFPDEVKTDQQVLTFFKDWKPKNSIGEYRQYSNPSIG
DC-33WP_001211220.1_1041AbBOC     KNTPIDQVNLLQLATYTSGNLALQFPDEVKTDQQVLTFFKDWKPKNSIGEYRQYSNPSIG
DC-33WP_001211220.1_1044AbBCP     KNTPIDQVNLLQLATYTSGNLALQFPDEVKTDQQVLTFFKDWKPKNSIGEYRQYSNPSIG
DC-33WP_001211220.1_2452AbBCP     KNTPIDQVNLLQLATYTSGNLALQFPDEVKTDQQVLTFFKDWKPKNSIGEYRQYSNPSIG
DC-33WP_001211220.1_1046AbBCP     KNTPIDQVNLLQLATYTSGNLALQFPDEVKTDQQVLTFFKDWKPKNSIGEYRQYSNPSIG
DC-33WP_001211220.1_1025AbBCP     KNTPIDQVNLLQLATYTSGNLALQFPDEVKTDQQVLTFFKDWKPKNSIGEYRQYSNPSIG
DC-33WP_001211220.1_1026AbBCP     KNTPIDQVNLLQLATYTSGNLALQFPDEVKTDQQVLTFFKDWKPKNSIGEYRQYSNPSIG
DC-33WP_001211220.1_1033AbBCP     KNTPIDQVNLLQLATYTSGNLALQFPDEVKTDQQVLTFFKDWKPKNSIGEYRQYSNPSIG
DC-33WP_001211220.1_1035AbBCP     KNTPIDQVNLLQLATYTSGNLALQFPDEVKTDQQVLTFFKDWKPKNSIGEYRQYSNPSIG
DC-33WP_001211220.1_1041AbBCP     KNTPIDQVNLLQLATYTSGNLALQFPDEVKTDQQVLTFFKDWKPKNSIGEYRQYSNPSIG
C-212WP_031975357.1_1297AbBOC     KNTPIDQVNLLQLATYTSGNLALQFPDEVKTDQQVLTFFKDWKPKNSIGEYRQYSNPSIG
DC-212WP_031975357.1_501AbBOC     KNTPIDQVNLLQLATYTSGNLALQFPDEVKTDQQVLTFFKDWKPKNSIGEYRQYSNPSIG
C-212WP_031975357.1_1297AbBCP     KNTPIDQVNLLQLATYTSGNLALQFPDEVKTDQQVLTFFKDWKPKNSIGEYRQYSNPSIG
DC-212WP_031975357.1_501AbBCP     KNTPIDQVNLLQLATYTSGNLALQFPDEVKTDQQVLTFFKDWKPKNSIGEYRQYSNPSIG
C-182WP_057691114.1_1055AbBOC     KNTPIDQVNLLQLATYTSGNLALQFPDEVQTDQQVLTFFKDWQPKNPIGEYRQYSNPSIG
C-182WP_057691114.1_1055AbBCP     KNTPIDQVNLLQLATYTSGNLALQFPDEVQTDQQVLTFFKDWQPKNPIGEYRQYSNPSIG
DC-81WP_059262723.1_1208AbBOC     KNTPIDQVNLLQLATYTSGNLALQFPDEVQTDQQVLTFFKDWQPKNPIGEYRQYSNPSIG
DC-81WP_059262723.1_2841AbBOC     KNTPIDQVNLLQLATYTSGNLALQFPDEVQTDQQVLTFFKDWQPKNPIGEYRQYSNPSIG
DC-81WP_059262723.1_1208AbBCP     KNTPIDQVNLLQLATYTSGNLALQFPDEVQTDQQVLTFFKDWQPKNPIGEYRQYSNPSIG
DC-81WP_059262723.1_2841AbBCP     KNTPIDQVNLLQLATYTSGNLALQFPDEVQTDQQVLTFFKDWQPKNPIGEYRQYSNPSIG
ADC-176WP_001159761.1_11AbOC      KNTPIDQVNLLQLATYTSGNLALQFPDEVQTDQQVLTFFKDWKPKNPIGEYRQYSNPSIG
DC-176WP_001159761.1_3466AbOC     KNTPIDQVNLLQLATYTSGNLALQFPDEVQTDQQVLTFFKDWKPKNPIGEYRQYSNPSIG
ADC-176WP_001159761.1_93AbBOP     KNTPIDQVNLLQLATYTSGNLALQFPDEVQTDQQVLTFFKDWKPKNPIGEYRQYSNPSIG
DC-176WP_001159761.1_247AbBOC     KNTPIDQVNLLQLATYTSGNLALQFPDEVQTDQQVLTFFKDWKPKNPIGEYRQYSNPSIG
ADC-176WP_001159761.1_9AbBOC      KNTPIDQVNLLQLATYTSGNLALQFPDEVQTDQQVLTFFKDWKPKNPIGEYRQYSNPSIG
C-176WP_001159761.1_2982AbBOC     KNTPIDQVNLLQLATYTSGNLALQFPDEVQTDQQVLTFFKDWKPKNPIGEYRQYSNPSIG
ADC-176QLG80706.1_1971AbBOC       KNTPIDQVNLLQLATYTSGNLALQFPDEVQTDQQVLTFFKDWKPKNPIGEYRQYSNPSIG
C-176WP_001159761.1_2851AbBOC     KNTPIDQVNLLQLATYTSGNLALQFPDEVQTDQQVLTFFKDWKPKNPIGEYRQYSNPSIG
C-176WP_001159761.1_1220AbBOC     KNTPIDQVNLLQLATYTSGNLALQFPDEVQTDQQVLTFFKDWKPKNPIGEYRQYSNPSIG
DC-176WP_001159761.1_386AbBOC     KNTPIDQVNLLQLATYTSGNLALQFPDEVQTDQQVLTFFKDWKPKNPIGEYRQYSNPSIG
ADC-176WP_001159761.1_11AbBOC     KNTPIDQVNLLQLATYTSGNLALQFPDEVQTDQQVLTFFKDWKPKNPIGEYRQYSNPSIG
DC-176WP_001159761.1_247AbBCP     KNTPIDQVNLLQLATYTSGNLALQFPDEVQTDQQVLTFFKDWKPKNPIGEYRQYSNPSIG
ADC-176WP_001159761.1_9AbBCP      KNTPIDQVNLLQLATYTSGNLALQFPDEVQTDQQVLTFFKDWKPKNPIGEYRQYSNPSIG
C-176WP_001159761.1_2982AbBCP     KNTPIDQVNLLQLATYTSGNLALQFPDEVQTDQQVLTFFKDWKPKNPIGEYRQYSNPSIG
ADC-176QLG80706.1_1971AbBCP       KNTPIDQVNLLQLATYTSGNLALQFPDEVQTDQQVLTFFKDWKPKNPIGEYRQYSNPSIG
C-176WP_001159761.1_2851AbBCP     KNTPIDQVNLLQLATYTSGNLALQFPDEVQTDQQVLTFFKDWKPKNPIGEYRQYSNPSIG
C-176WP_001159761.1_1220AbBCP     KNTPIDQVNLLQLATYTSGNLALQFPDEVQTDQQVLTFFKDWKPKNPIGEYRQYSNPSIG
DC-176WP_001159761.1_386AbBCP     KNTPIDQVNLLQLATYTSGNLALQFPDEVQTDQQVLTFFKDWKPKNPIGEYRQYSNPSIG
ADC-176WP_001159761.1_11AbBCP     KNTPIDQVNLLQLATYTSGNLALQFPDEVQTDQQVLTFFKDWKPKNPIGEYRQYSNPSIG
ADC-199WP_114166683.1_169AbOC     KNTPIDQVNLLQLATYTSGNLALQFPDEVQTDQQVLTFFKDWKPKNPIGEYRQYSNPSIG
DC-199WP_114166683.1_267AbBOC     KNTPIDQVNLLQLATYTSGNLALQFPDEVQTDQQVLTFFKDWKPKNPIGEYRQYSNPSIG
DC-199WP_114166683.1_267AbBCP     KNTPIDQVNLLQLATYTSGNLALQFPDEVQTDQQVLTFFKDWKPKNPIGEYRQYSNPSIG
                             acc
```

```
                                   α6           α7     α8            η5      β6 TT β7
8FQV_1|Chains                      160       170       180       190       200       210
8FQV_1|Chains                      LFGKVVALSMNKPFDQVLEKTIFPALGLKHSYVNVPKTQMQNYAFGYNQENQPIRVNPGP
-154OCUAc18_RS13275_2608AbBOC      LFGKVVALSMNKPFDQVLEKTIFPALGLKHSYVNVPKTQMQNYAFGYNQENQPIRVNPGP
-154OCUAc18_RS13275_2608AbBCP      LFGKVVALSMNKPFDQVLEKTIFPALGLKHSYVNVPKTQMQNYAFGYNQENQPIRVNPGP
ADC-73BDE22771.1_1271AbBCP         LFGKVVALSMNKPFDQVLEKTIFPALGLKHSYVNVPKTQMQNYAFGYNQENQPIRVNPGP
DC-87WP_068981619.1_1049AbBOC      LFGEIVGLSMKQPFSQVLEKTIFPDLGLKHSYVNVPKTQMQNYAFGYNQENQPIRVNPGP
DC-87WP_068981619.1_1049AbBCP      LFGEIVGLSMKQPFSQVLEKTIFPDLGLKHSYVNVPKTQMQNYAFGYNQENQPIRVNPGP
C-249WP_001211200.1_1039AbBOC      LFGKVVALSMNKPFDQVLEKTIFPALGLKHSYVNVPKTQMQNYAFGYNQENQPIRVNPGP
C-249WP_001211200.1_1039AbBCP      LFGKVVALSMNKPFDQVLEKTIFPALGLKHSYVNVPKTQMQNYAFGYNQENQPIRVNPGP
DC-32WP_004739487.1_2518AbBOC      LFGKFVALSMNKPFDQVLEKTIFPALGLKHSYVNVPKTQMQNYAFGYNQENQPIRVNPGP
DC-32WP_004739487.1_3158AbBOC      LFGKFVALSMNKPFDQVLEKTIFPALGLKHSYVNVPKTQMQNYAFGYNQENQPIRVNPGP
DC-32WP_004739487.1_2392AbBOC      LFGKFVALSMNKPFDQVLEKTIFPALGLKHSYVNVPKTQMQNYAFGYNQENQPIRVNPGP
DC-32WP_004739487.1_2378AbBOC      LFGKFVALSMNKPFDQVLEKTIFPALGLKHSYVNVPKTQMQNYAFGYNQENQPIRVNPGP
DC-32WP_004739487.1_2518AbBCP      LFGKFVALSMNKPFDQVLEKTIFPALGLKHSYVNVPKTQMQNYAFGYNQENQPIRVNPGP
DC-32WP_004739487.1_3158AbBCP      LFGKFVALSMNKPFDQVLEKTIFPALGLKHSYVNVPKTQMQNYAFGYNQENQPIRVNPGP
DC-32WP_004739487.1_2392AbBCP      LFGKFVALSMNKPFDQVLEKTIFPALGLKHSYVNVPKTQMQNYAFGYNQENQPIRVNPGP
DC-32WP_004739487.1_2378AbBCP      LFGKFVALSMNKPFDQVLEKTIFPALGLKHSYVNVPKTQMQNYAFGYNQENQPIRVNPGP
C-248WP_021511005.1_1080AbBOC      LFGKVVALSMNKPFDQVLEKTIFPALGLKHSYVNVPKTQMQNYAFGYNQENQPIRVNPGP
C-248WP_021511005.1_1080AbBCP      LFGKVVALSMNKPFDQVLEKTIFPALGLKHSYVNVPKTQMQNYAFGYNQENQPIRVNPGP
ADC-191UIY52419.1_2933AbOC         LFGKVVALSMNKPFDQVLEKTIFPALGLKHSYVNVPKTQMQNYAFGYNQENQPIRVNPGP
DC-191WP_003112187.1_3072AbOC      LFGKVVALSMNKPFDQVLEKTIFPALGLKHSYVNVPKTQMQNYAFGYNQENQPIRVNPGP
DC-191WP_003112187.1_2848AbOC      LFGKVVALSMNKPFDQVLEKTIFPALGLKHSYVNVPKTQMQNYAFGYNQENQPIRVNPGP
C-191WP_003112187.1_2694AbBOC      LFGKVVALSMNKPFDQVLEKTIFPALGLKHSYVNVPKTQMQNYAFGYNQENQPIRVNPGP
C-191WP_003112187.1_2694AbBCP      LFGKVVALSMNKPFDQVLEKTIFPALGLKHSYVNVPKTQMQNYAFGYNQENQPIRVNPGP
C-169WP_001211212.1_2709AbBOC      LFGKVVALSMNKPFDQVLEKTIFPALGLKHSYVNVPKTQMQNYAFGYNQENQPIRVNPGP
C-169WP_001211212.1_2457AbBOC      LFGKVVALSMNKPFDQVLEKTIFPALGLKHSYVNVPKTQMQNYAFGYNQENQPIRVNPGP
ADC-169UYQ18775.1_1073AbBOC        LFGKVVALSMNKPFDQVLEKTIFPALGLKHSYVNVPKTQMQNYAFGYNQENQPIRVNPGP
ADC-169UYQ29710.1_1071AbBOC        LFGKVVALSMNKPFDQVLEKTIFPALGLKHSYVNVPKTQMQNYAFGYNQENQPIRVNPGP
ADC-169UYQ59091.1_1072AbBOC        LFGKVVALSMNKPFDQVLEKTIFPALGLKHSYVNVPKTQMQNYAFGYNQENQPIRVNPGP
ADC-169WGT82884.1_1015AbBOC        LFGKVVALSMNKPFDQVLEKTIFPALGLKHSYVNVPKTQMQNYAFGYNQENQPIRVNPGP
C-169WP_001211212.1_2709AbBCP      LFGKVVALSMNKPFDQVLEKTIFPALGLKHSYVNVPKTQMQNYAFGYNQENQPIRVNPGP
C-169WP_001211212.1_2457AbBCP      LFGKVVALSMNKPFDQVLEKTIFPALGLKHSYVNVPKTQMQNYAFGYNQENQPIRVNPGP
ADC-169UYQ18775.1_1073AbBCP        LFGKVVALSMNKPFDQVLEKTIFPALGLKHSYVNVPKTQMQNYAFGYNQENQPIRVNPGP
ADC-169UYQ29710.1_1071AbBCP        LFGKVVALSMNKPFDQVLEKTIFPALGLKHSYVNVPKTQMQNYAFGYNQENQPIRVNPGP
ADC-169UYQ59091.1_1072AbBCP        LFGKVVALSMNKPFDQVLEKTIFPALGLKHSYVNVPKTQMQNYAFGYNQENQPIRVNPGP
ADC-169WGT82884.1_1015AbBCP        LFGKVVALSMNKPFDQVLEKTIFPALGLKHSYVNVPKTQMQNYAFGYNQENQPIRVNPGP
DC-99WP_068981630.1_2482AbBOC      LFGKVVALSMNKPFDQVLEKTIFPALGLKHSYVNVPKTQMQNYAFGYNQENQPIRVNPGP
DC-99WP_068981630.1_2482AbBCP      LFGKVVALSMNKPFDQVLEKTIFPALGLKHSYVNVPKTQMQNYAFGYNQENQPIRVNPGP
DC-222WP_031960999.1_1504AbOC      LFGKVVALSMNKPFDQVLEKTIFPALGLKYSYVNVPKTQIQNYAFGYNQENQPIRVNPGP
ADC-222UYP92921.1_1090AbBOC        LFGKVVALSMNKPFDQVLEKTIFPALGLKYSYVNVPKTQIQNYAFGYNQENQPIRVNPGP
ADC-222UYQ05421.1_2617AbBOC        LFGKVVALSMNKPFDQVLEKTIFPALGLKYSYVNVPKTQIQNYAFGYNQENQPIRVNPGP
ADC-222UYQ22523.1_1090AbBOC        LFGKVVALSMNKPFDQVLEKTIFPALGLKYSYVNVPKTQIQNYAFGYNQENQPIRVNPGP
ADC-222UYQ26181.1_1089AbBOC        LFGKVVALSMNKPFDQVLEKTIFPALGLKYSYVNVPKTQIQNYAFGYNQENQPIRVNPGP
ADC-222UYQ33449.1_1092AbBOC        LFGKVVALSMNKPFDQVLEKTIFPALGLKYSYVNVPKTQIQNYAFGYNQENQPIRVNPGP
ADC-222UYQ42503.1_2616AbBOC        LFGKVVALSMNKPFDQVLEKTIFPALGLKYSYVNVPKTQIQNYAFGYNQENQPIRVNPGP
ADC-222UYQ37112.1_1090AbBOC        LFGKVVALSMNKPFDQVLEKTIFPALGLKYSYVNVPKTQIQNYAFGYNQENQPIRVNPGP
ADC-222UYQ46167.1_2615AbBOC        LFGKVVALSMNKPFDQVLEKTIFPALGLKYSYVNVPKTQIQNYAFGYNQENQPIRVNPGP
ADC-222UYQ51934.1_1091AbBOC        LFGKVVALSMNKPFDQVLEKTIFPALGLKYSYVNVPKTQIQNYAFGYNQENQPIRVNPGP
ADC-222UYQ55558.1_1091AbBOC        LFGKVVALSMNKPFDQVLEKTIFPALGLKYSYVNVPKTQIQNYAFGYNQENQPIRVNPGP
ADC-222WBS68552.1_1096AbBOC        LFGKVVALSMNKPFDQVLEKTIFPALGLKYSYVNVPKTQIQNYAFGYNQENQPIRVNPGP
ADC-222WBS55174.1_2602AbBOC        LFGKVVALSMNKPFDQVLEKTIFPALGLKYSYVNVPKTQIQNYAFGYNQENQPIRVNPGP
ADC-222WBS51522.1_2600AbBOC        LFGKVVALSMNKPFDQVLEKTIFPALGLKYSYVNVPKTQIQNYAFGYNQENQPIRVNPGP
ADC-222UYP92921.1_1090AbBCP        LFGKVVALSMNKPFDQVLEKTIFPALGLKYSYVNVPKTQIQNYAFGYNQENQPIRVNPGP
ADC-222UYQ05421.1_2617AbBCP        LFGKVVALSMNKPFDQVLEKTIFPALGLKYSYVNVPKTQIQNYAFGYNQENQPIRVNPGP
ADC-222UYQ22523.1_1090AbBCP        LFGKVVALSMNKPFDQVLEKTIFPALGLKYSYVNVPKTQIQNYAFGYNQENQPIRVNPGP
ADC-222UYQ26181.1_1089AbBCP        LFGKVVALSMNKPFDQVLEKTIFPALGLKYSYVNVPKTQIQNYAFGYNQENQPIRVNPGP
ADC-222UYQ33449.1_1092AbBCP        LFGKVVALSMNKPFDQVLEKTIFPALGLKYSYVNVPKTQIQNYAFGYNQENQPIRVNPGP
ADC-222UYQ42503.1_2616AbBCP        LFGKVVALSMNKPFDQVLEKTIFPALGLKYSYVNVPKTQIQNYAFGYNQENQPIRVNPGP
ADC-222UYQ37112.1_1090AbBCP        LFGKVVALSMNKPFDQVLEKTIFPALGLKYSYVNVPKTQIQNYAFGYNQENQPIRVNPGP
ADC-222UYQ46167.1_2615AbBCP        LFGKVVALSMNKPFDQVLEKTIFPALGLKYSYVNVPKTQIQNYAFGYNQENQPIRVNPGP
ADC-222UYQ51934.1_1091AbBCP        LFGKVVALSMNKPFDQVLEKTIFPALGLKYSYVNVPKTQIQNYAFGYNQENQPIRVNPGP
ADC-222UYQ55558.1_1091AbBCP        LFGKVVALSMNKPFDQVLEKTIFPALGLKYSYVNVPKTQIQNYAFGYNQENQPIRVNPGP
ADC-222WBS68552.1_1096AbBCP        LFGKVVALSMNKPFDQVLEKTIFPALGLKYSYVNVPKTQIQNYAFGYNQENQPIRVNPGP
ADC-222WBS55174.1_2602AbBCP        LFGKVVALSMNKPFDQVLEKTIFPALGLKYSYVNVPKTQIQNYAFGYNQENQPIRVNPGP
ADC-222WBS51522.1_2600AbBCP        LFGKVVALSMNKPFDQVLEKTIFPALGLKYSYVNVPKTQIQNYAFGYNQENQPIRVNPGP
ADC-26WP_001211238.1_141AbOC       LFGKVVALSMNKPFDQVLEKTIFPALGLKHSYVNVPKTQMQNYAFGYNQENQPIRVNPGP
ADC-26WP_001211238.1_142AbOC       LFGKVVALSMNKPFDQVLEKTIFPALGLKHSYVNVPKTQMQNYAFGYNQENQPIRVNPGP
ADC-26WP_001211238.1_1AbOC         LFGKVVALSMNKPFDQVLEKTIFPALGLKHSYVNVPKTQMQNYAFGYNQENQPIRVNPGP
ADC-26WP_001211238.1_1113AbOC      LFGKVVALSMNKPFDQVLEKTIFPALGLKHSYVNVPKTQMQNYAFGYNQENQPIRVNPGP
ADC-26WP_001211238.1_1112AbOC      LFGKVVALSMNKPFDQVLEKTIFPALGLKHSYVNVPKTQMQNYAFGYNQENQPIRVNPGP
DC-26WP_001211238.1_1105AbBOC      LFGKVVALSMNKPFDQVLEKTIFPALGLKHSYVNVPKTQMQNYAFGYNQENQPIRVNPGP
DC-26WP_001211238.1_1111AbBOC      LFGKVVALSMNKPFDQVLEKTIFPALGLKHSYVNVPKTQMQNYAFGYNQENQPIRVNPGP
ADC-26WP_001211238.1_503AbBOC      LFGKVVALSMNKPFDQVLEKTIFPALGLKHSYVNVPKTQMQNYAFGYNQENQPIRVNPGP
DC-26WP_001211238.1_2774AbBOC      LFGKVVALSMNKPFDQVLEKTIFPALGLKHSYVNVPKTQMQNYAFGYNQENQPIRVNPGP
DC-26WP_001211238.1_1064AbBOC      LFGKVVALSMNKPFDQVLEKTIFPALGLKHSYVNVPKTQMQNYAFGYNQENQPIRVNPGP
DC-26WP_001211238.1_2484AbBOC      LFGKVVALSMNKPFDQVLEKTIFPALGLKHSYVNVPKTQMQNYAFGYNQENQPIRVNPGP
DC-26WP_001211238.1_3858AbBOC      LFGKVVALSMNKPFDQVLEKTIFPALGLKHSYVNVPKTQMQNYAFGYNQENQPIRVNPGP
ADC-26WP_001211238.1_142AbBOC      LFGKVVALSMNKPFDQVLEKTIFPALGLKHSYVNVPKTQMQNYAFGYNQENQPIRVNPGP
DC-26WP_001211238.1_1085AbBOC      LFGKVVALSMNKPFDQVLEKTIFPALGLKHSYVNVPKTQMQNYAFGYNQENQPIRVNPGP
DC-26WP_001211238.1_2525AbBOC      LFGKVVALSMNKPFDQVLEKTIFPALGLKHSYVNVPKTQMQNYAFGYNQENQPIRVNPGP
DC-26WP_001211238.1_2477AbBOC      LFGKVVALSMNKPFDQVLEKTIFPALGLKHSYVNVPKTQMQNYAFGYNQENQPIRVNPGP
DC-26WP_001211238.1_1069AbBOC      LFGKVVALSMNKPFDQVLEKTIFPALGLKHSYVNVPKTQMQNYAFGYNQENQPIRVNPGP
DC-26WP_001211238.1_1029AbBOC      LFGKVVALSMNKPFDQVLEKTIFPALGLKHSYVNVPKTQMQNYAFGYNQENQPIRVNPGP
DC-26WP_001211238.1_2443AbBOC      LFGKVVALSMNKPFDQVLEKTIFPALGLKHSYVNVPKTQMQNYAFGYNQENQPIRVNPGP
DC-26WP_001211238.1_1149AbBOC      LFGKVVALSMNKPFDQVLEKTIFPALGLKHSYVNVPKTQMQNYAFGYNQENQPIRVNPGP
DC-26WP_001211238.1_1148AbBOC      LFGKVVALSMNKPFDQVLEKTIFPALGLKHSYVNVPKTQMQNYAFGYNQENQPIRVNPGP
DC-26WP_001211238.1_1374AbBOC      LFGKVVALSMNKPFDQVLEKTIFPALGLKHSYVNVPKTQMQNYAFGYNQENQPIRVNPGP
DC-26WP_001211238.1_2862AbBOC      LFGKVVALSMNKPFDQVLEKTIFPALGLKHSYVNVPKTQMQNYAFGYNQENQPIRVNPGP
DC-26WP_001211238.1_2629AbBOC      LFGKVVALSMNKPFDQVLEKTIFPALGLKHSYVNVPKTQMQNYAFGYNQENQPIRVNPGP
DC-26WP_001211238.1_1355AbBOC      LFGKVVALSMNKPFDQVLEKTIFPALGLKHSYVNVPKTQMQNYAFGYNQENQPIRVNPGP
DC-26WP_001211238.1_3092AbBOC      LFGKVVALSMNKPFDQVLEKTIFPALGLKHSYVNVPKTQMQNYAFGYNQENQPIRVNPGP
DC-26WP_001211238.1_1086AbBOC      LFGKVVALSMNKPFDQVLEKTIFPALGLKHSYVNVPKTQMQNYAFGYNQENQPIRVNPGP
DC-26WP_001211238.1_1016AbBOC      LFGKVVALSMNKPFDQVLEKTIFPALGLKHSYVNVPKTQMQNYAFGYNQENQPIRVNPGP
ADC-26UUG48795.1_2867AbBOC        LFGKVVALSMNKPFDQVLEKTIFPALGLKHSYVNVPKTQMQNYAFGYNQENQPIRVNPGP
DC-26WP_001211238.1_2535AbBOC      LFGKVVALSMNKPFDQVLEKTIFPALGLKHSYVNVPKTQMQNYAFGYNQENQPIRVNPGP
ADC-26WEN46767.1_1086AbBOC        LFGKVVALSMNKPFDQVLEKTIFPALGLKHSYVNVPKTQMQNYAFGYNQENQPIRVNPGP
ADC-26WEX48630.1_1270AbBOC        LFGKVVALSMNKPFDQVLEKTIFPALGLKHSYVNVPKTQMQNYAFGYNQENQPIRVNPGP
ADC-26WGE92537.1_2034AbBOC        LFGKVVALSMNKPFDQVLEKTIFPALGLKHSYVNVPKTQMQNYAFGYNQENQPIRVNPGP
DC-26WP_001211238.1_1105AbBCP      LFGKVVALSMNKPFDQVLEKTIFPALGLKHSYVNVPKTQMQNYAFGYNQENQPIRVNPGP
DC-26WP_001211238.1_1111AbBCP      LFGKVVALSMNKPFDQVLEKTIFPALGLKHSYVNVPKTQMQNYAFGYNQENQPIRVNPGP
ADC-26WP_001211238.1_503AbBCP      LFGKVVALSMNKPFDQVLEKTIFPALGLKHSYVNVPKTQMQNYAFGYNQENQPIRVNPGP
DC-26WP_001211238.1_2774AbBCP      LFGKVVALSMNKPFDQVLEKTIFPALGLKHSYVNVPKTQMQNYAFGYNQENQPIRVNPGP
DC-26WP_001211238.1_1064AbBCP      LFGKVVALSMNKPFDQVLEKTIFPALGLKHSYVNVPKTQMQNYAFGYNQENQPIRVNPGP
DC-26WP_001211238.1_2484AbBCP      LFGKVVALSMNKPFDQVLEKTIFPALGLKHSYVNVPKTQMQNYAFGYNQENQPIRVNPGP
DC-26WP_001211238.1_3858AbBCP      LFGKVVALSMNKPFDQVLEKTIFPALGLKHSYVNVPKTQMQNYAFGYNQENQPIRVNPGP
ADC-26WP_001211238.1_142AbBCP      LFGKVVALSMNKPFDQVLEKTIFPALGLKHSYVNVPKTQMQNYAFGYNQENQPIRVNPGP
DC-26WP_001211238.1_1085AbBCP      LFGKVVALSMNKPFDQVLEKTIFPALGLKHSYVNVPKTQMQNYAFGYNQENQPIRVNPGP
DC-26WP_001211238.1_2525AbBCP      LFGKVVALSMNKPFDQVLEKTIFPALGLKHSYVNVPKTQMQNYAFGYNQENQPIRVNPGP
DC-26WP_001211238.1_2477AbBCP      LFGKVVALSMNKPFDQVLEKTIFPALGLKHSYVNVPKTQMQNYAFGYNQENQPIRVNPGP
DC-26WP_001211238.1_1069AbBCP      LFGKVVALSMNKPFDQVLEKTIFPALGLKHSYVNVPKTQMQNYAFGYNQENQPIRVNPGP
DC-26WP_001211238.1_1029AbBCP      LFGKVVALSMNKPFDQVLEKTIFPALGLKHSYVNVPKTQMQNYAFGYNQENQPIRVNPGP
DC-26WP_001211238.1_2443AbBCP      LFGKVVALSMNKPFDQVLEKTIFPALGLKHSYVNVPKTQMQNYAFGYNQENQPIRVNPGP
DC-26WP_001211238.1_1149AbBCP      LFGKVVALSMNKPFDQVLEKTIFPALGLKHSYVNVPKTQMQNYAFGYNQENQPIRVNPGP
DC-26WP_001211238.1_1148AbBCP      LFGKVVALSMNKPFDQVLEKTIFPALGLKHSYVNVPKTQMQNYAFGYNQENQPIRVNPGP
DC-26WP_001211238.1_1374AbBCP      LFGKVVALSMNKPFDQVLEKTIFPALGLKHSYVNVPKTQMQNYAFGYNQENQPIRVNPGP
DC-26WP_001211238.1_2862AbBCP      LFGKVVALSMNKPFDQVLEKTIFPALGLKHSYVNVPKTQMQNYAFGYNQENQPIRVNPGP
DC-26WP_001211238.1_2629AbBCP      LFGKVVALSMNKPFDQVLEKTIFPALGLKHSYVNVPKTQMQNYAFGYNQENQPIRVNPGP
DC-26WP_001211238.1_1355AbBCP      LFGKVVALSMNKPFDQVLEKTIFPALGLKHSYVNVPKTQMQNYAFGYNQENQPIRVNPGP
DC-26WP_001211238.1_3092AbBCP      LFGKVVALSMNKPFDQVLEKTIFPALGLKHSYVNVPKTQMQNYAFGYNQENQPIRVNPGP
DC-26WP_001211238.1_1086AbBCP      LFGKVVALSMNKPFDQVLEKTIFPALGLKHSYVNVPKTQMQNYAFGYNQENQPIRVNPGP
DC-26WP_001211238.1_1016AbBCP      LFGKVVALSMNKPFDQVLEKTIFPALGLKHSYVNVPKTQMQNYAFGYNQENQPIRVNPGP
ADC-26UUG48795.1_2867AbBCP        LFGKVVALSMNKPFDQVLEKTIFPALGLKHSYVNVPKTQMQNYAFGYNQENQPIRVNPGP
DC-26WP_001211238.1_2535AbBCP      LFGKVVALSMNKPFDQVLEKTIFPALGLKHSYVNVPKTQMQNYAFGYNQENQPIRVNPGP
ADC-26WEN46767.1_1086AbBCP        LFGKVVALSMNKPFDQVLEKTIFPALGLKHSYVNVPKTQMQNYAFGYNQENQPIRVNPGP
ADC-26WEX48630.1_1270AbBCP        LFGKVVALSMNKPFDQVLEKTIFPALGLKHSYVNVPKTQMQNYAFGYNQENQPIRVNPGP
ADC-26WGE92537.1_2034AbBCP        LFGKVVALSMNKPFDQVLEKTIFPALGLKHSYVNVPKTQMQNYAFGYNQENQPIRVNPGP
C-243WP_001211199.1_2530AbBOC      LFGKFVALSMNKPFDQVLEKTIFPALGLKHSYVNVPKTQMQNYAFGYNQENQPIRVNPGP
C-243WP_001211199.1_2530AbBCP      LFGKFVALSMNKPFDQVLEKTIFPALGLKHSYVNVPKTQMQNYAFGYNQENQPIRVNPGP
ADC-74WP_001211203.1_938AbBOC      LFGKVVALSMNKPFDQVLEKTIFPALGLKHSYVNVPKTQMQNYAFGYNQENQPIRANPGP
ADC-74WP_001211203.1_2AbBOC        LFGKVVALSMNKPFDQVLEKTIFPALGLKHSYVNVPKTQMQNYAFGYNQENQPIRANPGP
ADC-74WP_001211203.1_95AbBOC       LFGKVVALSMNKPFDQVLEKTIFPALGLKHSYVNVPKTQMQNYAFGYNQENQPIRANPGP
ADC-74WP_001211203.1_142AbBOC      LFGKVVALSMNKPFDQVLEKTIFPALGLKHSYVNVPKTQMQNYAFGYNQENQPIRANPGP
DC-74WP_001211203.1_2317AbBOC      LFGKVVALSMNKPFDQVLEKTIFPALGLKHSYVNVPKTQMQNYAFGYNQENQPIRANPGP
ADC-74WP_001211203.1_570AbBOC      LFGKVVALSMNKPFDQVLEKTIFPALGLKHSYVNVPKTQMQNYAFGYNQENQPIRANPGP
DC-74WP_001211203.1_2791AbBOC      LFGKVVALSMNKPFDQVLEKTIFPALGLKHSYVNVPKTQMQNYAFGYNQENQPIRANPGP
DC-74WP_001211203.1_2790AbBOC      LFGKVVALSMNKPFDQVLEKTIFPALGLKHSYVNVPKTQMQNYAFGYNQENQPIRANPGP
DC-74WP_001211203.1_1414AbBOC      LFGKVVALSMNKPFDQVLEKTIFPALGLKHSYVNVPKTQMQNYAFGYNQENQPIRANPGP
ADC-74WP_001211203.1_938AbBCP      LFGKVVALSMNKPFDQVLEKTIFPALGLKHSYVNVPKTQMQNYAFGYNQENQPIRANPGP
ADC-74WP_001211203.1_2AbBCP        LFGKVVALSMNKPFDQVLEKTIFPALGLKHSYVNVPKTQMQNYAFGYNQENQPIRANPGP
ADC-74WP_001211203.1_95AbBCP       LFGKVVALSMNKPFDQVLEKTIFPALGLKHSYVNVPKTQMQNYAFGYNQENQPIRANPGP
ADC-74WP_001211203.1_142AbBCP      LFGKVVALSMNKPFDQVLEKTIFPALGLKHSYVNVPKTQMQNYAFGYNQENQPIRANPGP
DC-74WP_001211203.1_2317AbBCP      LFGKVVALSMNKPFDQVLEKTIFPALGLKHSYVNVPKTQMQNYAFGYNQENQPIRANPGP
ADC-74WP_001211203.1_570AbBCP      LFGKVVALSMNKPFDQVLEKTIFPALGLKHSYVNVPKTQMQNYAFGYNQENQPIRANPGP
DC-74WP_001211203.1_2791AbBCP      LFGKVVALSMNKPFDQVLEKTIFPALGLKHSYVNVPKTQMQNYAFGYNQENQPIRANPGP
DC-74WP_001211203.1_2790AbBCP      LFGKVVALSMNKPFDQVLEKTIFPALGLKHSYVNVPKTQMQNYAFGYNQENQPIRANPGP
DC-74WP_001211203.1_1414AbBCP      LFGKVVALSMNKPFDQVLEKTIFPALGLKHSYVNVPKTQMQNYAFGYNQENQPIRANPGP
C-347WP_138149148.1_2656AbBOC      LFGKVVALSMNKPFDQVLEKTIFPALGLKHSYVNVPKTQMQNYAFGYNQENQPIRVNPGP
C-347WP_138149148.1_2656AbBCP      LFGKVVALSMNKPFDQVLEKTIFPALGLKHSYVNVPKTQMQNYAFGYNQENQPIRVNPGP
ADC-80WP_029424536.1_1068AbOC      LFGKVVALSMNKPFDQVLEKTIFPALGLKHSYVNVPKTQMQNYAFGYNQENQPIRVNPGP
DC-80WP_029424536.1_1061AbBOC      LFGKVVALSMNKPFDQVLEKTIFPALGLKHSYVNVPKTQMQNYAFGYNQENQPIRVNPGP
DC-80WP_029424536.1_2945AbBOC      LFGKVVALSMNKPFDQVLEKTIFPALGLKHSYVNVPKTQMQNYAFGYNQENQPIRVNPGP
DC-80WP_029424536.1_2729AbBOC      LFGKVVALSMNKPFDQVLEKTIFPALGLKHSYVNVPKTQMQNYAFGYNQENQPIRVNPGP
DC-80WP_029424536.1_1008AbBOC      LFGKVVALSMNKPFDQVLEKTIFPALGLKHSYVNVPKTQMQNYAFGYNQENQPIRVNPGP
```

```
DC-80WP_029424536.1_1061AbBCP    LFGKVVALSMNKPFDQVLEKTIFPALGLKHSYVNVPKTQMQNYAFGYNQENQPIRVNPGP
DC-80WP_029424536.1_2945AbBCP    LFGKVVALSMNKPFDQVLEKTIFPALGLKHSYVNVPKTQMQNYAFGYNQENQPIRVNPGP
DC-80WP_029424536.1_2729AbBCP    LFGKVVALSMNKPFDQVLEKTIFPALGLKHSYVNVPKTQMQNYAFGYNQENQPIRVNPGP
DC-80WP_029424536.1_1008AbBCP    LFGKVVALSMNKPFDQVLEKTIFPALGLKHSYVNVPKTQMQNYAFGYNQENQPIRVNPGP
DC-156WP_024436624.1_2681AbOC    LFGKVVALSMNKPFDQALEKTIFPALGLKHSYVNVPKTQMQNYAFGYNQENQPIRVNPGP
C-156WP_024436624.1_2375AbBOC    LFGKVVALSMNKPFDQALEKTIFPALGLKHSYVNVPKTQMQNYAFGYNQENQPIRVNPGP
C-156WP_024436624.1_1038AbBOC    LFGKVVALSMNKPFDQALEKTIFPALGLKHSYVNVPKTQMQNYAFGYNQENQPIRVNPGP
C-156WP_024436624.1_1041AbBOC    LFGKVVALSMNKPFDQALEKTIFPALGLKHSYVNVPKTQMQNYAFGYNQENQPIRVNPGP
C-156WP_024436624.1_1124AbBOC    LFGKVVALSMNKPFDQALEKTIFPALGLKHSYVNVPKTQMQNYAFGYNQENQPIRVNPGP
C-156WP_024436624.1_1126AbBOC    LFGKVVALSMNKPFDQALEKTIFPALGLKHSYVNVPKTQMQNYAFGYNQENQPIRVNPGP
C-156WP_024436624.1_2375AbBCP    LFGKVVALSMNKPFDQALEKTIFPALGLKHSYVNVPKTQMQNYAFGYNQENQPIRVNPGP
C-156WP_024436624.1_1038AbBCP    LFGKVVALSMNKPFDQALEKTIFPALGLKHSYVNVPKTQMQNYAFGYNQENQPIRVNPGP
C-156WP_024436624.1_1041AbBCP    LFGKVVALSMNKPFDQALEKTIFPALGLKHSYVNVPKTQMQNYAFGYNQENQPIRVNPGP
C-156WP_024436624.1_1124AbBCP    LFGKVVALSMNKPFDQALEKTIFPALGLKHSYVNVPKTQMQNYAFGYNQENQPIRVNPGP
C-156WP_024436624.1_1126AbBCP    LFGKVVALSMNKPFDQALEKTIFPALGLKHSYVNVPKTQMQNYAFGYNQENQPIRVNPGP
C-165WP_001211239.1_1734AbBOC    LFGKVVALSMNKPFDQVLEKTIFPALGLKHSYVNVPKTQMQNYAFGYNQENQPIRVNPGP
C-165WP_001211239.1_1032AbBOC    LFGKVVALSMNKPFDQVLEKTIFPALGLKHSYVNVPKTQMQNYAFGYNQENQPIRVNPGP
C-165WP_001211239.1_1734AbBCP    LFGKVVALSMNKPFDQVLEKTIFPALGLKHSYVNVPKTQMQNYAFGYNQENQPIRVNPGP
C-165WP_001211239.1_1032AbBCP    LFGKVVALSMNKPFDQVLEKTIFPALGLKHSYVNVPKTQMQNYAFGYNQENQPIRVNPGP
C-263WP_109433738.1_2026AbBOC    LFGKVVALSMNKPFDQVLEKTIFPALGLKHSYVNVPKTQMQNYAFGYNQENQPIRVNPGP
DC-263WP_109433738.1_954AbBOC    LFGKVVALSMNKPFDQVLEKTIFPALGLKHSYVNVPKTQMQNYAFGYNQENQPIRVNPGP
C-263WP_109433738.1_1022AbBOC    LFGKVVALSMNKPFDQVLEKTIFPALGLKHSYVNVPKTQMQNYAFGYNQENQPIRVNPGP
C-263WP_109433738.1_3219AbBOC    LFGKVVALSMNKPFDQVLEKTIFPALGLKHSYVNVPKTQMQNYAFGYNQENQPIRVNPGP
C-263WP_109433738.1_2026AbBCP    LFGKVVALSMNKPFDQVLEKTIFPALGLKHSYVNVPKTQMQNYAFGYNQENQPIRVNPGP
DC-263WP_109433738.1_954AbBCP    LFGKVVALSMNKPFDQVLEKTIFPALGLKHSYVNVPKTQMQNYAFGYNQENQPIRVNPGP
C-263WP_109433738.1_1022AbBCP    LFGKVVALSMNKPFDQVLEKTIFPALGLKHSYVNVPKTQMQNYAFGYNQENQPIRVNPGP
C-263WP_109433738.1_3219AbBCP    LFGKVVALSMNKPFDQVLEKTIFPALGLKHSYVNVPKTQMQNYAFGYNQENQPIRVNPGP
DC-79WP_001159760.1_2471AbBOC    LFGKVVALSMNKPFDQVLEKTIFPALGLKHSYVNVPKTQMQNYAFGYNQENQPIRVNPGP
ADC-79WP_001159760.1_670AbBOC    LFGKVVALSMNKPFDQVLEKTIFPALGLKHSYVNVPKTQMQNYAFGYNQENQPIRVNPGP
DC-79WP_001159760.1_2535AbBOC    LFGKVVALSMNKPFDQVLEKTIFPALGLKHSYVNVPKTQMQNYAFGYNQENQPIRVNPGP
DC-79WP_001159760.1_1080AbBOC    LFGKVVALSMNKPFDQVLEKTIFPALGLKHSYVNVPKTQMQNYAFGYNQENQPIRVNPGP
DC-79WP_001159760.1_2471AbBCP    LFGKVVALSMNKPFDQVLEKTIFPALGLKHSYVNVPKTQMQNYAFGYNQENQPIRVNPGP
ADC-79WP_001159760.1_670AbBCP    LFGKVVALSMNKPFDQVLEKTIFPALGLKHSYVNVPKTQMQNYAFGYNQENQPIRVNPGP
DC-79WP_001159760.1_2535AbBCP    LFGKVVALSMNKPFDQVLEKTIFPALGLKHSYVNVPKTQMQNYAFGYNQENQPIRVNPGP
DC-79WP_001159760.1_1080AbBCP    LFGKVVALSMNKPFDQVLEKTIFPALGLKHSYVNVPKTQMQNYAFGYNQENQPIRVNPGP
ADC-6WP_017725267.1_1459AbBOC    LFGKVVALSMNKPFDQVLEKTIFPALGLKHSYVNVPKTQMQNYAFGYNQENQPIRVNPGP
ADC-6WP_017725267.1_1678AbBOC    LFGKVVALSMNKPFDQVLEKTIFPALGLKHSYVNVPKTQMQNYAFGYNQENQPIRVNPGP
ADC-6WP_017725267.1_2561AbBOC    LFGKVVALSMNKPFDQVLEKTIFPALGLKHSYVNVPKTQMQNYAFGYNQENQPIRVNPGP
ADC-6WP_017725267.1_1459AbBCP    LFGKVVALSMNKPFDQVLEKTIFPALGLKHSYVNVPKTQMQNYAFGYNQENQPIRVNPGP
ADC-6WP_017725267.1_1678AbBCP    LFGKVVALSMNKPFDQVLEKTIFPALGLKHSYVNVPKTQMQNYAFGYNQENQPIRVNPGP
ADC-6WP_017725267.1_2561AbBCP    LFGKVVALSMNKPFDQVLEKTIFPALGLKHSYVNVPKTQMQNYAFGYNQENQPIRVNPGP
ADC-1WP_004714775.1_3726AbBOC    LFGKVVALSMNKPFDQVLEKTIFPALGLKHSYVNVPKTQMQNYAFGYNQENQPIRVNPGP
ADC-1WP_004714775.1_3726AbBCP    LFGKVVALSMNKPFDQVLEKTIFPALGLKHSYVNVPKTQMQNYAFGYNQENQPIRVNPGP
C-181WP_046693238.1_1083AbBOC    LFGKVVALSMNKPFDQVLEKTIFPALGLKHSYVNVPKTQMQNYAFGYNQENQPIRVNPGP
C-181WP_046693238.1_1083AbBCP    LFGKVVALSMNKPFDQVLEKTIFPALGLKHSYVNVPKTQMQNYAFGYNQENQPIRVNPGP
C-238WP_002058891.1_2458AbBOC    LFGKVVALSMNKPFDQVLEKTIFPALGLKHSYVNVPKTQMQNYAFGYNQENQPIRVNPGP
C-238WP_002058891.1_2458AbBCP    LFGKVVALSMNKPFDQVLEKTIFPALGLKHSYVNVPKTQMQNYAFGYNQENQPIRVNPGP
C-186WP_072292271.1_1675AbBOC    LFGKVVALSMNKPFDQVLEKTIFPALGLKHSYVNVPKTQMQNYAFGYNQENQPIRVNPGP
C-186WP_072292271.1_1675AbBCP    LFGKVVALSMNKPFDQVLEKTIFPALGLKHSYVNVPKTQMQNYAFGYNQENQPIRVNPGP
DC-184WP_059247009.1_1188AbOC    LFGKVVALSMNKPFDQVLEETIFPALGLKHSYVNVPKTQMQNYAFGYNQENQPIRVNPGP
ADC-328WP_069370341.1_666AbOC    LFGKVVALSMNKPFDQVLEKTIFPALGLKHSYVNVAKTQMQNYAFGYNQENQPIRVNPGP
DC-56WP_031973850.1_1911AbBOC    LFGKVVALSMNKPFDQVLEKTIFPALGLKHSYVNVPKTQMQNYAFGYNQENQPIRVNPGP
DC-56WP_031973850.1_2693AbBOC    LFGKVVALSMNKPFDQVLEKTIFPALGLKHSYVNVPKTQMQNYAFGYNQENQPIRVNPGP
DC-56WP_031973850.1_1911AbBCP    LFGKVVALSMNKPFDQVLEKTIFPALGLKHSYVNVPKTQMQNYAFGYNQENQPIRVNPGP
DC-56WP_031973850.1_2693AbBCP    LFGKVVALSMNKPFDQVLEKTIFPALGLKHSYVNVPKTQMQNYAFGYNQENQPIRVNPGP
DC-117WP_002157727.1_394AbBOC    LFGKVVALSMNKPFDQVLEKTIFPALGLKHSYVNVPKTQMQNYAFGYNQENQPIRVNPGP
DC-117WP_002157727.1_394AbBCP    LFGKVVALSMNKPFDQVLEKTIFPALGLKHSYVNVPKTQMQNYAFGYNQENQPIRVNPGP
ADC-25WP_001211217.1_2432AbOC    LFGKVVALSMNKPFDQVLEKTIFPALGLKHSYVNVPKTQMQNYAFGYNQENQPIRVNPGP
ADC-25WP_001211217.1_1025AbOC    LFGKVVALSMNKPFDQVLEKTIFPALGLKHSYVNVPKTQMQNYAFGYNQENQPIRVNPGP
ADC-25WP_001211217.1_3391AbOC    LFGKVVALSMNKPFDQVLEKTIFPALGLKHSYVNVPKTQMQNYAFGYNQENQPIRVNPGP
ADC-25WP_001211217.1_1042AbOC    LFGKVVALSMNKPFDQVLEKTIFPALGLKHSYVNVPKTQMQNYAFGYNQENQPIRVNPGP
ADC-25WP_001211217.1_1185AbOC    LFGKVVALSMNKPFDQVLEKTIFPALGLKHSYVNVPKTQMQNYAFGYNQENQPIRVNPGP
ADC-25WP_001211217.1_768AbOC     LFGKVVALSMNKPFDQVLEKTIFPALGLKHSYVNVPKTQMQNYAFGYNQENQPIRVNPGP
ADC-25WP_001211217.1_2605AbOC    LFGKVVALSMNKPFDQVLEKTIFPALGLKHSYVNVPKTQMQNYAFGYNQENQPIRVNPGP
DC-25WP_001211217.1_1117AbBOC    LFGKVVALSMNKPFDQVLEKTIFPALGLKHSYVNVPKTQMQNYAFGYNQENQPIRVNPGP
DC-25WP_001211217.1_1090AbBOC    LFGKVVALSMNKPFDQVLEKTIFPALGLKHSYVNVPKTQMQNYAFGYNQENQPIRVNPGP
DC-25WP_001211217.1_1089AbBOC    LFGKVVALSMNKPFDQVLEKTIFPALGLKHSYVNVPKTQMQNYAFGYNQENQPIRVNPGP
DC-25WP_001211217.1_1091AbBOC    LFGKVVALSMNKPFDQVLEKTIFPALGLKHSYVNVPKTQMQNYAFGYNQENQPIRVNPGP
DC-25WP_001211217.1_1604AbBOC    LFGKVVALSMNKPFDQVLEKTIFPALGLKHSYVNVPKTQMQNYAFGYNQENQPIRVNPGP
DC-25WP_001211217.1_1040AbBOC    LFGKVVALSMNKPFDQVLEKTIFPALGLKHSYVNVPKTQMQNYAFGYNQENQPIRVNPGP
ADC-25UYQ12932.1_2634AbBOC       LFGKVVALSMNKPFDQVLEKTIFPALGLKHSYVNVPKTQMQNYAFGYNQENQPIRVNPGP
ADC-25UYQ09083.1_2628AbBOC       LFGKVVALSMNKPFDQVLEKTIFPALGLKHSYVNVPKTQMQNYAFGYNQENQPIRVNPGP
ADC-25UYQ40822.1_1120AbBOC       LFGKVVALSMNKPFDQVLEKTIFPALGLKHSYVNVPKTQMQNYAFGYNQENQPIRVNPGP
DC-25WP_001211217.1_1117AbBCP    LFGKVVALSMNKPFDQVLEKTIFPALGLKHSYVNVPKTQMQNYAFGYNQENQPIRVNPGP
DC-25WP_001211217.1_1090AbBCP    LFGKVVALSMNKPFDQVLEKTIFPALGLKHSYVNVPKTQMQNYAFGYNQENQPIRVNPGP
DC-25WP_001211217.1_1089AbBCP    LFGKVVALSMNKPFDQVLEKTIFPALGLKHSYVNVPKTQMQNYAFGYNQENQPIRVNPGP
DC-25WP_001211217.1_1091AbBCP    LFGKVVALSMNKPFDQVLEKTIFPALGLKHSYVNVPKTQMQNYAFGYNQENQPIRVNPGP
DC-25WP_001211217.1_1604AbBCP    LFGKVVALSMNKPFDQVLEKTIFPALGLKHSYVNVPKTQMQNYAFGYNQENQPIRVNPGP
DC-25WP_001211217.1_1040AbBCP    LFGKVVALSMNKPFDQVLEKTIFPALGLKHSYVNVPKTQMQNYAFGYNQENQPIRVNPGP
ADC-25UYQ12932.1_2634AbBCP       LFGKVVALSMNKPFDQVLEKTIFPALGLKHSYVNVPKTQMQNYAFGYNQENQPIRVNPGP
ADC-25UYQ09083.1_2628AbBCP       LFGKVVALSMNKPFDQVLEKTIFPALGLKHSYVNVPKTQMQNYAFGYNQENQPIRVNPGP
ADC-25UYQ40822.1_1120AbBCP       LFGKVVALSMNKPFDQVLEKTIFPALGLKHSYVNVPKTQMQNYAFGYNQENQPIRVNPGP
C-268WP_105922788.1_2839AbBOC    LFGKVVALSMNKPFDQVLEKTIFPALGLKHSYVNVPKTQMQNYAFGYNQENQPIRVNPGP
ADC-268WDC47378.1_2492AbBOC      LFGKVVALSMNKPFDQVLEKTIFPALGLKHSYVNVPKTQMQNYAFGYNQENQPIRVNPGP
C-268WP_105922788.1_2839AbBCP    LFGKVVALSMNKPFDQVLEKTIFPALGLKHSYVNVPKTQMQNYAFGYNQENQPIRVNPGP
ADC-268WDC47378.1_2492AbBCP      LFGKVVALSMNKPFDQVLEKTIFPALGLKHSYVNVPKTQMQNYAFGYNQENQPIRVNPGP
DC-155WP_005128228.1_2591AbOC    LFGKVVALSMNKPFDQVLEKTIFPALGLKHSYVNVPKTQMQNYAFGYNQENQPIRVNPGP
C-155WP_005128228.1_2549AbBOC    LFGKVVALSMNKPFDQVLEKTIFPALGLKHSYVNVPKTQMQNYAFGYNQENQPIRVNPGP
C-155WP_005128228.1_2480AbBOC    LFGKVVALSMNKPFDQVLEKTIFPALGLKHSYVNVPKTQMQNYAFGYNQENQPIRVNPGP
C-155WP_005128228.1_2455AbBOC    LFGKVVALSMNKPFDQVLEKTIFPALGLKHSYVNVPKTQMQNYAFGYNQENQPIRVNPGP
C-155WP_005128228.1_2549AbBCP    LFGKVVALSMNKPFDQVLEKTIFPALGLKHSYVNVPKTQMQNYAFGYNQENQPIRVNPGP
C-155WP_005128228.1_2480AbBCP    LFGKVVALSMNKPFDQVLEKTIFPALGLKHSYVNVPKTQMQNYAFGYNQENQPIRVNPGP
C-155WP_005128228.1_2455AbBCP    LFGKVVALSMNKPFDQVLEKTIFPALGLKHSYVNVPKTQMQNYAFGYNQENQPIRVNPGP
ADC-76WP_001211237.1_2862AbOC    LFGKVVALSMNKPFDQVLEKTIFPALGLKHSYVNVPKTQMQNYAFGYNQENQPIRVNPGP
ADC-76WP_001211237.1_3431AbOC    LFGKVVALSMNKPFDQVLEKTIFPALGLKHSYVNVPKTQMQNYAFGYNQENQPIRVNPGP
ADC-76WP_001211237.1_2696AbOC    LFGKVVALSMNKPFDQVLEKTIFPALGLKHSYVNVPKTQMQNYAFGYNQENQPIRVNPGP
ADC-76WP_001211237.1_2890AbOC    LFGKVVALSMNKPFDQVLEKTIFPALGLKHSYVNVPKTQMQNYAFGYNQENQPIRVNPGP
DC-76WP_001211237.1_2723AbBOC    LFGKVVALSMNKPFDQVLEKTIFPALGLKHSYVNVPKTQMQNYAFGYNQENQPIRVNPGP
DC-76WP_001211237.1_1033AbBOC    LFGKVVALSMNKPFDQVLEKTIFPALGLKHSYVNVPKTQMQNYAFGYNQENQPIRVNPGP
ADC-76WP_001211237.1_526AbBOC    LFGKVVALSMNKPFDQVLEKTIFPALGLKHSYVNVPKTQMQNYAFGYNQENQPIRVNPGP
DC-76WP_001211237.1_1046AbBOC    LFGKVVALSMNKPFDQVLEKTIFPALGLKHSYVNVPKTQMQNYAFGYNQENQPIRVNPGP
DC-76WP_001211237.1_1040AbBOC    LFGKVVALSMNKPFDQVLEKTIFPALGLKHSYVNVPKTQMQNYAFGYNQENQPIRVNPGP
DC-76WP_001211237.1_3682AbBOC    LFGKVVALSMNKPFDQVLEKTIFPALGLKHSYVNVPKTQMQNYAFGYNQENQPIRVNPGP
DC-76WP_001211237.1_2579AbBOC    LFGKVVALSMNKPFDQVLEKTIFPALGLKHSYVNVPKTQMQNYAFGYNQENQPIRVNPGP
DC-76WP_001211237.1_2723AbBCP    LFGKVVALSMNKPFDQVLEKTIFPALGLKHSYVNVPKTQMQNYAFGYNQENQPIRVNPGP
DC-76WP_001211237.1_1033AbBCP    LFGKVVALSMNKPFDQVLEKTIFPALGLKHSYVNVPKTQMQNYAFGYNQENQPIRVNPGP
ADC-76WP_001211237.1_526AbBCP    LFGKVVALSMNKPFDQVLEKTIFPALGLKHSYVNVPKTQMQNYAFGYNQENQPIRVNPGP
DC-76WP_001211237.1_1046AbBCP    LFGKVVALSMNKPFDQVLEKTIFPALGLKHSYVNVPKTQMQNYAFGYNQENQPIRVNPGP
DC-76WP_001211237.1_1040AbBCP    LFGKVVALSMNKPFDQVLEKTIFPALGLKHSYVNVPKTQMQNYAFGYNQENQPIRVNPGP
DC-76WP_001211237.1_3682AbBCP    LFGKVVALSMNKPFDQVLEKTIFPALGLKHSYVNVPKTQMQNYAFGYNQENQPIRVNPGP
DC-76WP_001211237.1_2579AbBCP    LFGKVVALSMNKPFDQVLEKTIFPALGLKHSYVNVPKTQMQNYAFGYNQENQPIRVNPGP
DC-291WP_001211201.1_1060AbOC    LFGKVVALSMNKPFDQVLEKTIFPALGLKHSYVNVPKTQMQNYAFGYNQENQPIRVTPGP
C-291WP_001211201.1_2415AbBOC    LFGKVVALSMNKPFDQVLEKTIFPALGLKHSYVNVPKTQMQNYAFGYNQENQPIRVTPGP
C-291WP_001211201.1_2415AbBCP    LFGKVVALSMNKPFDQVLEKTIFPALGLKHSYVNVPKTQMQNYAFGYNQENQPIRVTPGP
DC-214WP_060454527.1_118AbBOC    LFGKVVALSMNKPFDQVLEKTIFPALGLKHSYVNVAKTQMQNYAFGYNQENQPIRVNPGP
DC-214WP_060454527.1_118AbBCP    LFGKVVALSMNKPFDQVLEKTIFPALGLKHSYVNVAKTQMQNYAFGYNQENQPIRVNPGP
C-185WP_001211210.1_2745AbBOC    LFGKVVALSMNKPFDQVLEKTIFPALGLKHSYVNVPKTQMQNYAVGYNQENQPIRVNPGP
ADC-185WEI07035.1_298AbBOC      LFGKVVALSMNKPFDQVLEKTIFPALGLKHSYVNVPKTQMQNYAVGYNQENQPIRVNPGP
C-185WP_001211210.1_2745AbBCP    LFGKVVALSMNKPFDQVLEKTIFPALGLKHSYVNVPKTQMQNYAVGYNQENQPIRVNPGP
ADC-185WEI07035.1_298AbBCP      LFGKVVALSMNKPFDQVLEKTIFPALGLKHSYVNVPKTQMQNYAVGYNQENQPIRVNPGP
ADC-11WP_001211205.1_1099AbOC    LFGKVVALSMNKPFDQVLEKTIFPALGLKHSYVNVPKTQMQNYAFGYNQENQPIRVNPGP
ADC-11WP_001211205.1_1035AbOC    LFGKVVALSMNKPFDQVLEKTIFPALGLKHSYVNVPKTQMQNYAFGYNQENQPIRVNPGP
DC-11WP_001211205.1_2603AbBOC    LFGKVVALSMNKPFDQVLEKTIFPALGLKHSYVNVPKTQMQNYAFGYNQENQPIRVNPGP
DC-11WP_001211205.1_1138AbBOC    LFGKVVALSMNKPFDQVLEKTIFPALGLKHSYVNVPKTQMQNYAFGYNQENQPIRVNPGP
DC-11WP_001211205.1_2874AbBOC    LFGKVVALSMNKPFDQVLEKTIFPALGLKHSYVNVPKTQMQNYAFGYNQENQPIRVNPGP
DC-11WP_001211205.1_2686AbBOC    LFGKVVALSMNKPFDQVLEKTIFPALGLKHSYVNVPKTQMQNYAFGYNQENQPIRVNPGP
ADC-11WP_001211205.1_130AbBOC    LFGKVVALSMNKPFDQVLEKTIFPALGLKHSYVNVPKTQMQNYAFGYNQENQPIRVNPGP
DC-11WP_001211205.1_1097AbBOC    LFGKVVALSMNKPFDQVLEKTIFPALGLKHSYVNVPKTQMQNYAFGYNQENQPIRVNPGP
ADC-11WP_001211205.1_684AbBOC    LFGKVVALSMNKPFDQVLEKTIFPALGLKHSYVNVPKTQMQNYAFGYNQENQPIRVNPGP
ADC-11WP_001211205.1_682AbBOC    LFGKVVALSMNKPFDQVLEKTIFPALGLKHSYVNVPKTQMQNYAFGYNQENQPIRVNPGP
DC-11WP_001211205.1_1111AbBOC    LFGKVVALSMNKPFDQVLEKTIFPALGLKHSYVNVPKTQMQNYAFGYNQENQPIRVNPGP
DC-11WP_001211205.1_1059AbBOC    LFGKVVALSMNKPFDQVLEKTIFPALGLKHSYVNVPKTQMQNYAFGYNQENQPIRVNPGP
DC-11WP_001211205.1_2754AbBOC    LFGKVVALSMNKPFDQVLEKTIFPALGLKHSYVNVPKTQMQNYAFGYNQENQPIRVNPGP
DC-11WP_001211205.1_2603AbBCP    LFGKVVALSMNKPFDQVLEKTIFPALGLKHSYVNVPKTQMQNYAFGYNQENQPIRVNPGP
DC-11WP_001211205.1_1138AbBCP    LFGKVVALSMNKPFDQVLEKTIFPALGLKHSYVNVPKTQMQNYAFGYNQENQPIRVNPGP
DC-11WP_001211205.1_2874AbBCP    LFGKVVALSMNKPFDQVLEKTIFPALGLKHSYVNVPKTQMQNYAFGYNQENQPIRVNPGP
DC-11WP_001211205.1_2686AbBCP    LFGKVVALSMNKPFDQVLEKTIFPALGLKHSYVNVPKTQMQNYAFGYNQENQPIRVNPGP
ADC-11WP_001211205.1_130AbBCP    LFGKVVALSMNKPFDQVLEKTIFPALGLKHSYVNVPKTQMQNYAFGYNQENQPIRVNPGP
DC-11WP_001211205.1_1097AbBCP    LFGKVVALSMNKPFDQVLEKTIFPALGLKHSYVNVPKTQMQNYAFGYNQENQPIRVNPGP
ADC-11WP_001211205.1_684AbBCP    LFGKVVALSMNKPFDQVLEKTIFPALGLKHSYVNVPKTQMQNYAFGYNQENQPIRVNPGP
ADC-11WP_001211205.1_682AbBCP    LFGKVVALSMNKPFDQVLEKTIFPALGLKHSYVNVPKTQMQNYAFGYNQENQPIRVNPGP
DC-11WP_001211205.1_1111AbBCP    LFGKVVALSMNKPFDQVLEKTIFPALGLKHSYVNVPKTQMQNYAFGYNQENQPIRVNPGP
DC-11WP_001211205.1_1059AbBCP    LFGKVVALSMNKPFDQVLEKTIFPALGLKHSYVNVPKTQMQNYAFGYNQENQPIRVNPGP
DC-11WP_001211205.1_2754AbBCP    LFGKVVALSMNKPFDQVLEKTIFPALGLKHSYVNVPKTQMQNYAFGYNQENQPIRVNPGP
C-269WP_032025626.1_2988AbBOC    LFGKVVALSMNKPFDQALEKTIFPALGLKHSYVNVPKTQMQNYAFGYNQENQPIRVNPGP
C-269WP_032025626.1_2988AbBCP    LFGKVVALSMNKPFDQALEKTIFPALGLKHSYVNVPKTQMQNYAFGYNQENQPIRVNPGP
C-166WP_101244937.1_1028AbBOC    LFGKVVALSMNKPFDQVLEKTIFPALGLKHSYVNVPKTQMQNYAFGYNQENQPIRVNPGP
C-166WP_101244937.1_1028AbBCP    LFGKVVALSMNKPFDQVLEKTIFPALGLKHSYVNVPKTQMQNYAFGYNQENQPIRVNPGP
C-175WP_001211208.1_2561AbBOC    LFRKVVALSMNKPFDQVLEKTIFPALGLKHSYVNVPKTQMQNYAFGYNQENQPIRVNPGP
C-175WP_001211208.1_2561AbBCP    LFRKVVALSMNKPFDQVLEKTIFPALGLKHSYVNVPKTQMQNYAFGYNQENQPIRVNPGP
C-154WP_005138362.1_2451AbBOC    LFGKVVALSMNKPFDQVLEKTIFPALGLKHSYVNVPKTQMQNYAFGYNQENQPIRVNPGP
DC-154WP_005138362.1_158AbBOC    LFGKVVALSMNKPFDQVLEKTIFPALGLKHSYVNVPKTQMQNYAFGYNQENQPIRVNPGP
```

```
C-154WP_005138362.1_1167AbBOC    LFGKVVALSMNKPFDQVLEKTIFPALGLKHSYVNVPKTQMQNYAFGYNQENQPIRVNPGP
C-154WP_005138362.1_2510AbBOC    LFGKVVALSMNKPFDQVLEKTIFPALGLKHSYVNVPKTQMQNYAFGYNQENQPIRVNPGP
C-154WP_005138362.1_2513AbBOC    LFGKVVALSMNKPFDQVLEKTIFPALGLKHSYVNVPKTQMQNYAFGYNQENQPIRVNPGP
C-154WP_005138362.1_2512AbBOC    LFGKVVALSMNKPFDQVLEKTIFPALGLKHSYVNVPKTQMQNYAFGYNQENQPIRVNPGP
C-154WP_005138362.1_2451AbBCP    LFGKVVALSMNKPFDQVLEKTIFPALGLKHSYVNVPKTQMQNYAFGYNQENQPIRVNPGP
DC-154WP_005138362.1_158AbBCP    LFGKVVALSMNKPFDQVLEKTIFPALGLKHSYVNVPKTQMQNYAFGYNQENQPIRVNPGP
C-154WP_005138362.1_1167AbBCP    LFGKVVALSMNKPFDQVLEKTIFPALGLKHSYVNVPKTQMQNYAFGYNQENQPIRVNPGP
C-154WP_005138362.1_2510AbBCP    LFGKVVALSMNKPFDQVLEKTIFPALGLKHSYVNVPKTQMQNYAFGYNQENQPIRVNPGP
C-154WP_005138362.1_2513AbBCP    LFGKVVALSMNKPFDQVLEKTIFPALGLKHSYVNVPKTQMQNYAFGYNQENQPIRVNPGP
C-154WP_005138362.1_2512AbBCP    LFGKVVALSMNKPFDQVLEKTIFPALGLKHSYVNVPKTQMQNYAFGYNQENQPIRVNPGP
C-240WP_001211207.1_1200AbBOC    LFGKVVALSMNKPFDQVLEKTIFPALGLKHSYVNVPKTQMQNYAFGYNQENQPIRVNPGP
C-240WP_001211207.1_1200AbBCP    LFGKVVALSMNKPFDQVLEKTIFPALGLKHSYVNVPKTQMQNYAFGYNQENQPIRVNPGP
ADC-75WP_063857817.1_1610AbOC    LFGKVVALSMNKPFDQVLEKTIFPALGLKHSYVNVPKTQMQNYAFGYNQENQPIRVNPGP
DC-18WP_002118772.1_1702AbBOC    LFGKVVALSMNKPFDQVLEKTIFPPLHLKNSYVNVPKTQMQNYAYGYNQENQPIRVNPGP
DC-18WP_002118772.1_1702AbBCP    LFGKVVALSMNKPFDQVLEKTIFPPLHLKNSYVNVPKTQMQNYAYGYNQENQPIRVNPGP
C-106WP_068981613.1_2650AbBOC    LFGKVVALSMNKPFDQVLEKTIFPALGLKHSYVNVPKTQMQNYAFGYNQENQPIRVNPGP
C-106WP_068981613.1_2650AbBCP    LFGKVVALSMNKPFDQVLEKTIFPALGLKHSYVNVPKTQMQNYAFGYNQENQPIRVNPGP
DC-50WP_031965243.1_2562AbBOC    LFGKVVALSMNKPFDQVLEKTIFPALGLKHSYVNVAKTQMQNYAFGYNQENQPIRVNPGP
ADC-50WP_031965243.1_206AbBOC    LFGKVVALSMNKPFDQVLEKTIFPALGLKHSYVNVAKTQMQNYAFGYNQENQPIRVNPGP
DC-50WP_031965243.1_2751AbBOC    LFGKVVALSMNKPFDQVLEKTIFPALGLKHSYVNVAKTQMQNYAFGYNQENQPIRVNPGP
DC-50WP_031965243.1_3766AbBOC    LFGKVVALSMNKPFDQVLEKTIFPALGLKHSYVNVAKTQMQNYAFGYNQENQPIRVNPGP
DC-50WP_031965243.1_2562AbBCP    LFGKVVALSMNKPFDQVLEKTIFPALGLKHSYVNVAKTQMQNYAFGYNQENQPIRVNPGP
ADC-50WP_031965243.1_206AbBCP    LFGKVVALSMNKPFDQVLEKTIFPALGLKHSYVNVAKTQMQNYAFGYNQENQPIRVNPGP
DC-50WP_031965243.1_2751AbBCP    LFGKVVALSMNKPFDQVLEKTIFPALGLKHSYVNVAKTQMQNYAFGYNQENQPIRVNPGP
DC-50WP_031965243.1_3766AbBCP    LFGKVVALSMNKPFDQVLEKTIFPALGLKHSYVNVAKTQMQNYAFGYNQENQPIRVNPGP
ADC-158WP_001211227.1_990AbOC    LFGKVVALSMNKPFDQVLEKTIFPALGLKHSYVNVPKTQMQNYAFGYNQENQPIRVNPGP
DC-158WP_001211227.1_1295AbOC    LFGKVVALSMNKPFDQVLEKTIFPALGLKHSYVNVPKTQMQNYAFGYNQENQPIRVNPGP
ADC-158WP_001211227.1_6AbOC      LFGKVVALSMNKPFDQVLEKTIFPALGLKHSYVNVPKTQMQNYAFGYNQENQPIRVNPGP
DC-158WP_001211227.1_991AbBOC   LFGKVVALSMNKPFDQVLEKTIFPALGLKHSYVNVPKTQMQNYAFGYNQENQPIRVNPGP
DC-158WP_001211227.1_207AbBOC   LFGKVVALSMNKPFDQVLEKTIFPALGLKHSYVNVPKTQMQNYAFGYNQENQPIRVNPGP
C-158WP_001211227.1_2735AbBOC   LFGKVVALSMNKPFDQVLEKTIFPALGLKHSYVNVPKTQMQNYAFGYNQENQPIRVNPGP
DC-158WP_001211227.1_996AbBOC   LFGKVVALSMNKPFDQVLEKTIFPALGLKHSYVNVPKTQMQNYAFGYNQENQPIRVNPGP
DC-158WP_001211227.1_796AbBOC   LFGKVVALSMNKPFDQVLEKTIFPALGLKHSYVNVPKTQMQNYAFGYNQENQPIRVNPGP
C-158WP_001211227.1_2822AbBOC   LFGKVVALSMNKPFDQVLEKTIFPALGLKHSYVNVPKTQMQNYAFGYNQENQPIRVNPGP
C-158WP_001211227.1_1339AbBOC   LFGKVVALSMNKPFDQVLEKTIFPALGLKHSYVNVPKTQMQNYAFGYNQENQPIRVNPGP
ADC-158WGQ04828.1_2712AbBOC     LFGKVVALSMNKPFDQVLEKTIFPALGLKHSYVNVPKTQMQNYAFGYNQENQPIRVNPGP
DC-158WP_001211227.1_991AbBCP   LFGKVVALSMNKPFDQVLEKTIFPALGLKHSYVNVPKTQMQNYAFGYNQENQPIRVNPGP
DC-158WP_001211227.1_207AbBCP   LFGKVVALSMNKPFDQVLEKTIFPALGLKHSYVNVPKTQMQNYAFGYNQENQPIRVNPGP
C-158WP_001211227.1_2735AbBCP   LFGKVVALSMNKPFDQVLEKTIFPALGLKHSYVNVPKTQMQNYAFGYNQENQPIRVNPGP
DC-158WP_001211227.1_996AbBCP   LFGKVVALSMNKPFDQVLEKTIFPALGLKHSYVNVPKTQMQNYAFGYNQENQPIRVNPGP
DC-158WP_001211227.1_796AbBCP   LFGKVVALSMNKPFDQVLEKTIFPALGLKHSYVNVPKTQMQNYAFGYNQENQPIRVNPGP
C-158WP_001211227.1_2822AbBCP   LFGKVVALSMNKPFDQVLEKTIFPALGLKHSYVNVPKTQMQNYAFGYNQENQPIRVNPGP
C-158WP_001211227.1_1339AbBCP   LFGKVVALSMNKPFDQVLEKTIFPALGLKHSYVNVPKTQMQNYAFGYNQENQPIRVNPGP
ADC-158WGQ04828.1_2712AbBCP     LFGKVVALSMNKPFDQVLEKTIFPALGLKHSYVNVPKTQMQNYAFGYNQENQPIRVNPGP
ADC-31WP_001211223.1_2986AbOC   LFGKVVALSMNKPLDQVLEKTIFPALGLKHSYVNVPKTQMQNYAFGYNQENQPIRVNPGP
DC-31WP_001211223.1_2698AbBOC   LFGKVVALSMNKPLDQVLEKTIFPALGLKHSYVNVPKTQMQNYAFGYNQENQPIRVNPGP
DC-31WP_001211223.1_2698AbBCP   LFGKVVALSMNKPLDQVLEKTIFPALGLKHSYVNVPKTQMQNYAFGYNQENQPIRVNPGP
ADC-30WP_001211218.1_1102AbOC   LFGKVVALSMNKPFDQVLEKTIFPALGLKHSYVNVPKTQMQNYAFGYNQENQPIRVNPGP
ADC-30WP_001211218.1_2565AbOC   LFGKVVALSMNKPFDQVLEKTIFPALGLKHSYVNVPKTQMQNYAFGYNQENQPIRVNPGP
ADC-30WP_001211218.1_1021AbOC   LFGKVVALSMNKPFDQVLEKTIFPALGLKHSYVNVPKTQMQNYAFGYNQENQPIRVNPGP
ADC-30WP_001211218.1_1088AbOC   LFGKVVALSMNKPFDQVLEKTIFPALGLKHSYVNVPKTQMQNYAFGYNQENQPIRVNPGP
ADC-30WP_001211218.1_1135AbOC   LFGKVVALSMNKPFDQVLEKTIFPALGLKHSYVNVPKTQMQNYAFGYNQENQPIRVNPGP
ADC-30WP_001211218.1_43AbOC     LFGKVVALSMNKPFDQVLEKTIFPALGLKHSYVNVPKTQMQNYAFGYNQENQPIRVNPGP
ADC-30WP_001211218.1_1041AbOC   LFGKVVALSMNKPFDQVLEKTIFPALGLKHSYVNVPKTQMQNYAFGYNQENQPIRVNPGP
ADC-30WP_001211218.1_1032AbOC   LFGKVVALSMNKPFDQVLEKTIFPALGLKHSYVNVPKTQMQNYAFGYNQENQPIRVNPGP
ADC-30WP_001211218.1_1047AbOC   LFGKVVALSMNKPFDQVLEKTIFPALGLKHSYVNVPKTQMQNYAFGYNQENQPIRVNPGP
ADC-30WP_001211218.1_1040AbOC   LFGKVVALSMNKPFDQVLEKTIFPALGLKHSYVNVPKTQMQNYAFGYNQENQPIRVNPGP
ADC-30WP_001211218.1_3388AbOC   LFGKVVALSMNKPFDQVLEKTIFPALGLKHSYVNVPKTQMQNYAFGYNQENQPIRVNPGP
ADC-30WP_001211218.1_650AbOC    LFGKVVALSMNKPFDQVLEKTIFPALGLKHSYVNVPKTQMQNYAFGYNQENQPIRVNPGP
ADC-30WP_001211218.1_2855AbOC   LFGKVVALSMNKPFDQVLEKTIFPALGLKHSYVNVPKTQMQNYAFGYNQENQPIRVNPGP
ADC-30WP_001211218.1_1034AbOC   LFGKVVALSMNKPFDQVLEKTIFPALGLKHSYVNVPKTQMQNYAFGYNQENQPIRVNPGP
ADC-30WP_001211218.1_79AbOC     LFGKVVALSMNKPFDQVLEKTIFPALGLKHSYVNVPKTQMQNYAFGYNQENQPIRVNPGP
ADC-30WP_001211218.1_3509AbOC   LFGKVVALSMNKPFDQVLEKTIFPALGLKHSYVNVPKTQMQNYAFGYNQENQPIRVNPGP
ADC-30WP_001211218.1_2399AbOC   LFGKVVALSMNKPFDQVLEKTIFPALGLKHSYVNVPKTQMQNYAFGYNQENQPIRVNPGP
ADC-30WP_001211218.1_2415AbOC   LFGKVVALSMNKPFDQVLEKTIFPALGLKHSYVNVPKTQMQNYAFGYNQENQPIRVNPGP
ADC-30WP_001211218.1_3032AbOC   LFGKVVALSMNKPFDQVLEKTIFPALGLKHSYVNVPKTQMQNYAFGYNQENQPIRVNPGP
ADC-30WP_001211218.1_832AbOC    LFGKVVALSMNKPFDQVLEKTIFPALGLKHSYVNVPKTQMQNYAFGYNQENQPIRVNPGP
ADC-30WP_001211218.1_1428AbOC   LFGKVVALSMNKPFDQVLEKTIFPALGLKHSYVNVPKTQMQNYAFGYNQENQPIRVNPGP
ADC-30WP_001211218.1_2340AbOC   LFGKVVALSMNKPFDQVLEKTIFPALGLKHSYVNVPKTQMQNYAFGYNQENQPIRVNPGP
ADC-30WP_001211218.1_2976AbOC   LFGKVVALSMNKPFDQVLEKTIFPALGLKHSYVNVPKTQMQNYAFGYNQENQPIRVNPGP
ADC-30WP_001211218.1_2305AbOC   LFGKVVALSMNKPFDQVLEKTIFPALGLKHSYVNVPKTQMQNYAFGYNQENQPIRVNPGP
ADC-30WP_001211218.1_1110AbOC   LFGKVVALSMNKPFDQVLEKTIFPALGLKHSYVNVPKTQMQNYAFGYNQENQPIRVNPGP
ADC-30WP_001211218.1_2797AbOC   LFGKVVALSMNKPFDQVLEKTIFPALGLKHSYVNVPKTQMQNYAFGYNQENQPIRVNPGP
ADC-30WP_001211218.1_1023AbOC   LFGKVVALSMNKPFDQVLEKTIFPALGLKHSYVNVPKTQMQNYAFGYNQENQPIRVNPGP
ADC-30WP_001211218.1_1022AbOC   LFGKVVALSMNKPFDQVLEKTIFPALGLKHSYVNVPKTQMQNYAFGYNQENQPIRVNPGP
ADC-30WP_001211218.1_2458AbOC   LFGKVVALSMNKPFDQVLEKTIFPALGLKHSYVNVPKTQMQNYAFGYNQENQPIRVNPGP
ADC-30WP_001211218.1_2513AbOC   LFGKVVALSMNKPFDQVLEKTIFPALGLKHSYVNVPKTQMQNYAFGYNQENQPIRVNPGP
ADC-30WP_001211218.1_2482AbOC   LFGKVVALSMNKPFDQVLEKTIFPALGLKHSYVNVPKTQMQNYAFGYNQENQPIRVNPGP
DC-30WP_001211218.1_2692AbBOC   LFGKVVALSMNKPFDQVLEKTIFPALGLKHSYVNVPKTQMQNYAFGYNQENQPIRVNPGP
DC-30WP_001211218.1_2549AbBOC   LFGKVVALSMNKPFDQVLEKTIFPALGLKHSYVNVPKTQMQNYAFGYNQENQPIRVNPGP
DC-30WP_001211218.1_1026AbBOC   LFGKVVALSMNKPFDQVLEKTIFPALGLKHSYVNVPKTQMQNYAFGYNQENQPIRVNPGP
ADC-30WP_001211218.1_373AbBOC   LFGKVVALSMNKPFDQVLEKTIFPALGLKHSYVNVPKTQMQNYAFGYNQENQPIRVNPGP
DC-30WP_001211218.1_2465AbBOC   LFGKVVALSMNKPFDQVLEKTIFPALGLKHSYVNVPKTQMQNYAFGYNQENQPIRVNPGP
DC-30WP_001211218.1_1027AbBOC   LFGKVVALSMNKPFDQVLEKTIFPALGLKHSYVNVPKTQMQNYAFGYNQENQPIRVNPGP
DC-30WP_001211218.1_2650AbBOC   LFGKVVALSMNKPFDQVLEKTIFPALGLKHSYVNVPKTQMQNYAFGYNQENQPIRVNPGP
DC-30WP_001211218.1_2674AbBOC   LFGKVVALSMNKPFDQVLEKTIFPALGLKHSYVNVPKTQMQNYAFGYNQENQPIRVNPGP
DC-30WP_001211218.1_2689AbBOC   LFGKVVALSMNKPFDQVLEKTIFPALGLKHSYVNVPKTQMQNYAFGYNQENQPIRVNPGP
DC-30WP_001211218.1_1105AbBOC   LFGKVVALSMNKPFDQVLEKTIFPALGLKHSYVNVPKTQMQNYAFGYNQENQPIRVNPGP
DC-30WP_001211218.1_2670AbBOC   LFGKVVALSMNKPFDQVLEKTIFPALGLKHSYVNVPKTQMQNYAFGYNQENQPIRVNPGP
DC-30WP_001211218.1_1019AbBOC   LFGKVVALSMNKPFDQVLEKTIFPALGLKHSYVNVPKTQMQNYAFGYNQENQPIRVNPGP
DC-30WP_001211218.1_1039AbBOC   LFGKVVALSMNKPFDQVLEKTIFPALGLKHSYVNVPKTQMQNYAFGYNQENQPIRVNPGP
DC-30WP_001211218.1_1023AbBOC   LFGKVVALSMNKPFDQVLEKTIFPALGLKHSYVNVPKTQMQNYAFGYNQENQPIRVNPGP
DC-30WP_001211218.1_3189AbBOC   LFGKVVALSMNKPFDQVLEKTIFPALGLKHSYVNVPKTQMQNYAFGYNQENQPIRVNPGP
ADC-30WP_001211218.1_594AbBOC   LFGKVVALSMNKPFDQVLEKTIFPALGLKHSYVNVPKTQMQNYAFGYNQENQPIRVNPGP
DC-30WP_001211218.1_1718AbBOC   LFGKVVALSMNKPFDQVLEKTIFPALGLKHSYVNVPKTQMQNYAFGYNQENQPIRVNPGP
DC-30WP_001211218.1_2484AbBOC   LFGKVVALSMNKPFDQVLEKTIFPALGLKHSYVNVPKTQMQNYAFGYNQENQPIRVNPGP
DC-30WP_001211218.1_3305AbBOC   LFGKVVALSMNKPFDQVLEKTIFPALGLKHSYVNVPKTQMQNYAFGYNQENQPIRVNPGP
DC-30WP_001211218.1_3346AbBOC   LFGKVVALSMNKPFDQVLEKTIFPALGLKHSYVNVPKTQMQNYAFGYNQENQPIRVNPGP
DC-30WP_001211218.1_1482AbBOC   LFGKVVALSMNKPFDQVLEKTIFPALGLKHSYVNVPKTQMQNYAFGYNQENQPIRVNPGP
DC-30WP_001211218.1_2574AbBOC   LFGKVVALSMNKPFDQVLEKTIFPALGLKHSYVNVPKTQMQNYAFGYNQENQPIRVNPGP
ADC-30WP_001211218.1_831AbBOC   LFGKVVALSMNKPFDQVLEKTIFPALGLKHSYVNVPKTQMQNYAFGYNQENQPIRVNPGP
DC-30WP_001211218.1_1826AbBOC   LFGKVVALSMNKPFDQVLEKTIFPALGLKHSYVNVPKTQMQNYAFGYNQENQPIRVNPGP
DC-30WP_001211218.1_1024AbBOC   LFGKVVALSMNKPFDQVLEKTIFPALGLKHSYVNVPKTQMQNYAFGYNQENQPIRVNPGP
DC-30WP_001211218.1_1618AbBOC   LFGKVVALSMNKPFDQVLEKTIFPALGLKHSYVNVPKTQMQNYAFGYNQENQPIRVNPGP
ADC-30WP_001211218.1_301AbBOC   LFGKVVALSMNKPFDQVLEKTIFPALGLKHSYVNVPKTQMQNYAFGYNQENQPIRVNPGP
ADC-30WP_001211218.1_940AbBOC   LFGKVVALSMNKPFDQVLEKTIFPALGLKHSYVNVPKTQMQNYAFGYNQENQPIRVNPGP
DC-30WP_001211218.1_2949AbBOC   LFGKVVALSMNKPFDQVLEKTIFPALGLKHSYVNVPKTQMQNYAFGYNQENQPIRVNPGP
DC-30WP_001211218.1_1028AbBOC   LFGKVVALSMNKPFDQVLEKTIFPALGLKHSYVNVPKTQMQNYAFGYNQENQPIRVNPGP
DC-30WP_001211218.1_2526AbBOC   LFGKVVALSMNKPFDQVLEKTIFPALGLKHSYVNVPKTQMQNYAFGYNQENQPIRVNPGP
DC-30WP_001211218.1_1040AbBOC   LFGKVVALSMNKPFDQVLEKTIFPALGLKHSYVNVPKTQMQNYAFGYNQENQPIRVNPGP
DC-30WP_001211218.1_1089AbBOC   LFGKVVALSMNKPFDQVLEKTIFPALGLKHSYVNVPKTQMQNYAFGYNQENQPIRVNPGP
DC-30WP_001211218.1_1099AbBOC   LFGKVVALSMNKPFDQVLEKTIFPALGLKHSYVNVPKTQMQNYAFGYNQENQPIRVNPGP
DC-30WP_001211218.1_1206AbBOC   LFGKVVALSMNKPFDQVLEKTIFPALGLKHSYVNVPKTQMQNYAFGYNQENQPIRVNPGP
DC-30WP_001211218.1_1167AbBOC   LFGKVVALSMNKPFDQVLEKTIFPALGLKHSYVNVPKTQMQNYAFGYNQENQPIRVNPGP
DC-30WP_001211218.1_1186AbBOC   LFGKVVALSMNKPFDQVLEKTIFPALGLKHSYVNVPKTQMQNYAFGYNQENQPIRVNPGP
DC-30WP_001211218.1_1165AbBOC   LFGKVVALSMNKPFDQVLEKTIFPALGLKHSYVNVPKTQMQNYAFGYNQENQPIRVNPGP
DC-30WP_001211218.1_1077AbBOC   LFGKVVALSMNKPFDQVLEKTIFPALGLKHSYVNVPKTQMQNYAFGYNQENQPIRVNPGP
DC-30WP_001211218.1_1078AbBOC   LFGKVVALSMNKPFDQVLEKTIFPALGLKHSYVNVPKTQMQNYAFGYNQENQPIRVNPGP
DC-30WP_001211218.1_1071AbBOC   LFGKVVALSMNKPFDQVLEKTIFPALGLKHSYVNVPKTQMQNYAFGYNQENQPIRVNPGP
DC-30WP_001211218.1_2694AbBOC   LFGKVVALSMNKPFDQVLEKTIFPALGLKHSYVNVPKTQMQNYAFGYNQENQPIRVNPGP
DC-30WP_001211218.1_3340AbBOC   LFGKVVALSMNKPFDQVLEKTIFPALGLKHSYVNVPKTQMQNYAFGYNQENQPIRVNPGP
DC-30WP_001211218.1_1022AbBOC   LFGKVVALSMNKPFDQVLEKTIFPALGLKHSYVNVPKTQMQNYAFGYNQENQPIRVNPGP
DC-30WP_001211218.1_1025AbBOC   LFGKVVALSMNKPFDQVLEKTIFPALGLKHSYVNVPKTQMQNYAFGYNQENQPIRVNPGP
DC-30WP_001211218.1_1158AbBOC   LFGKVVALSMNKPFDQVLEKTIFPALGLKHSYVNVPKTQMQNYAFGYNQENQPIRVNPGP
DC-30WP_001211218.1_1020AbBOC   LFGKVVALSMNKPFDQVLEKTIFPALGLKHSYVNVPKTQMQNYAFGYNQENQPIRVNPGP
DC-30WP_001211218.1_1021AbBOC   LFGKVVALSMNKPFDQVLEKTIFPALGLKHSYVNVPKTQMQNYAFGYNQENQPIRVNPGP
ADC-30WP_001211218.1_532AbBOC   LFGKVVALSMNKPFDQVLEKTIFPALGLKHSYVNVPKTQMQNYAFGYNQENQPIRVNPGP
ADC-30WP_001211218.1_539AbBOC   LFGKVVALSMNKPFDQVLEKTIFPALGLKHSYVNVPKTQMQNYAFGYNQENQPIRVNPGP
DC-30WP_001211218.1_1033AbBOC   LFGKVVALSMNKPFDQVLEKTIFPALGLKHSYVNVPKTQMQNYAFGYNQENQPIRVNPGP
DC-30WP_001211218.1_1069AbBOC   LFGKVVALSMNKPFDQVLEKTIFPALGLKHSYVNVPKTQMQNYAFGYNQENQPIRVNPGP
DC-30WP_001211218.1_1029AbBOC   LFGKVVALSMNKPFDQVLEKTIFPALGLKHSYVNVPKTQMQNYAFGYNQENQPIRVNPGP
DC-30WP_001211218.1_1030AbBOC   LFGKVVALSMNKPFDQVLEKTIFPALGLKHSYVNVPKTQMQNYAFGYNQENQPIRVNPGP
DC-30WP_001211218.1_1096AbBOC   LFGKVVALSMNKPFDQVLEKTIFPALGLKHSYVNVPKTQMQNYAFGYNQENQPIRVNPGP
DC-30WP_001211218.1_1037AbBOC   LFGKVVALSMNKPFDQVLEKTIFPALGLKHSYVNVPKTQMQNYAFGYNQENQPIRVNPGP
DC-30WP_001211218.1_1098AbBOC   LFGKVVALSMNKPFDQVLEKTIFPALGLKHSYVNVPKTQMQNYAFGYNQENQPIRVNPGP
DC-30WP_001211218.1_1046AbBOC   LFGKVVALSMNKPFDQVLEKTIFPALGLKHSYVNVPKTQMQNYAFGYNQENQPIRVNPGP
DC-30WP_001211218.1_1032AbBOC   LFGKVVALSMNKPFDQVLEKTIFPALGLKHSYVNVPKTQMQNYAFGYNQENQPIRVNPGP
DC-30WP_001211218.1_1070AbBOC   LFGKVVALSMNKPFDQVLEKTIFPALGLKHSYVNVPKTQMQNYAFGYNQENQPIRVNPGP
DC-30WP_001211218.1_1139AbBOC   LFGKVVALSMNKPFDQVLEKTIFPALGLKHSYVNVPKTQMQNYAFGYNQENQPIRVNPGP
DC-30WP_001211218.1_1031AbBOC   LFGKVVALSMNKPFDQVLEKTIFPALGLKHSYVNVPKTQMQNYAFGYNQENQPIRVNPGP
DC-30WP_001211218.1_1066AbBOC   LFGKVVALSMNKPFDQVLEKTIFPALGLKHSYVNVPKTQMQNYAFGYNQENQPIRVNPGP
DC-30WP_001211218.1_1102AbBOC   LFGKVVALSMNKPFDQVLEKTIFPALGLKHSYVNVPKTQMQNYAFGYNQENQPIRVNPGP
ADC-30USX57838.1_2957AbBOC      LFGKVVALSMNKPFDQVLEKTIFPALGLKHSYVNVPKTQMQNYAFGYNQENQPIRVNPGP
ADC-30USX59985.1_1918AbBOC      LFGKVVALSMNKPFDQVLEKTIFPALGLKHSYVNVPKTQMQNYAFGYNQENQPIRVNPGP
ADC-30USX63799.1_2772AbBOC      LFGKVVALSMNKPFDQVLEKTIFPALGLKHSYVNVPKTQMQNYAFGYNQENQPIRVNPGP
ADC-30USX74012.1_305AbBOC       LFGKVVALSMNKPFDQVLEKTIFPALGLKHSYVNVPKTQMQNYAFGYNQENQPIRVNPGP
ADC-30USZ52468.1_2397AbBOC      LFGKVVALSMNKPFDQVLEKTIFPALGLKHSYVNVPKTQMQNYAFGYNQENQPIRVNPGP
DC-30WP_001211218.1_2008AbBOC   LFGKVVALSMNKPFDQVLEKTIFPALGLKHSYVNVPKTQMQNYAFGYNQENQPIRVNPGP
ADC-30WCL78976.1_1026AbBOC      LFGKVVALSMNKPFDQVLEKTIFPALGLKHSYVNVPKTQMQNYAFGYNQENQPIRVNPGP
ADC-30WDO27826.1_1034AbBOC      LFGKVVALSMNKPFDQVLEKTIFPALGLKHSYVNVPKTQMQNYAFGYNQENQPIRVNPGP
DC-30WP_001211218.1_2125AbBOC   LFGKVVALSMNKPFDQVLEKTIFPALGLKHSYVNVPKTQMQNYAFGYNQENQPIRVNPGP
DC-30WP_001211218.1_2729AbBOC   LFGKVVALSMNKPFDQVLEKTIFPALGLKHSYVNVPKTQMQNYAFGYNQENQPIRVNPGP
```

```
DC-30WP_001211218.1_2692AbBCP   LFGKVVALSMNKPFDQVLEKTIFFALGLKHSYVNVPKTQMQNYAFGYNQENQPIRVNPGP
DC-30WP_001211218.1_2549AbBCP   LFGKVVALSMNKPFDQVLEKTIFFALGLKHSYVNVPKTQMQNYAFGYNQENQPIRVNPGP
DC-30WP_001211218.1_1026AbBCP   LFGKVVALSMNKPFDQVLEKTIFFALGLKHSYVNVPKTQMQNYAFGYNQENQPIRVNPGP
ADC-30WP_001211218.1_373AbBCP   LFGKVVALSMNKPFDQVLEKTIFFALGLKHSYVNVPKTQMQNYAFGYNQENQPIRVNPGP
DC-30WP_001211218.1_2465AbBCP   LFGKVVALSMNKPFDQVLEKTIFFALGLKHSYVNVPKTQMQNYAFGYNQENQPIRVNPGP
DC-30WP_001211218.1_1027AbBCP   LFGKVVALSMNKPFDQVLEKTIFFALGLKHSYVNVPKTQMQNYAFGYNQENQPIRVNPGP
DC-30WP_001211218.1_2650AbBCP   LFGKVVALSMNKPFDQVLEKTIFFALGLKHSYVNVPKTQMQNYAFGYNQENQPIRVNPGP
DC-30WP_001211218.1_2674AbBCP   LFGKVVALSMNKPFDQVLEKTIFFALGLKHSYVNVPKTQMQNYAFGYNQENQPIRVNPGP
DC-30WP_001211218.1_2689AbBCP   LFGKVVALSMNKPFDQVLEKTIFFALGLKHSYVNVPKTQMQNYAFGYNQENQPIRVNPGP
DC-30WP_001211218.1_1105AbBCP   LFGKVVALSMNKPFDQVLEKTIFFALGLKHSYVNVPKTQMQNYAFGYNQENQPIRVNPGP
DC-30WP_001211218.1_2670AbBCP   LFGKVVALSMNKPFDQVLEKTIFFALGLKHSYVNVPKTQMQNYAFGYNQENQPIRVNPGP
DC-30WP_001211218.1_1019AbBCP   LFGKVVALSMNKPFDQVLEKTIFFALGLKHSYVNVPKTQMQNYAFGYNQENQPIRVNPGP
DC-30WP_001211218.1_1039AbBCP   LFGKVVALSMNKPFDQVLEKTIFFALGLKHSYVNVPKTQMQNYAFGYNQENQPIRVNPGP
DC-30WP_001211218.1_1023AbBCP   LFGKVVALSMNKPFDQVLEKTIFFALGLKHSYVNVPKTQMQNYAFGYNQENQPIRVNPGP
DC-30WP_001211218.1_3189AbBCP   LFGKVVALSMNKPFDQVLEKTIFFALGLKHSYVNVPKTQMQNYAFGYNQENQPIRVNPGP
ADC-30WP_001211218.1_594AbBCP   LFGKVVALSMNKPFDQVLEKTIFFALGLKHSYVNVPKTQMQNYAFGYNQENQPIRVNPGP
DC-30WP_001211218.1_1718AbBCP   LFGKVVALSMNKPFDQVLEKTIFFALGLKHSYVNVPKTQMQNYAFGYNQENQPIRVNPGP
DC-30WP_001211218.1_2484AbBCP   LFGKVVALSMNKPFDQVLEKTIFFALGLKHSYVNVPKTQMQNYAFGYNQENQPIRVNPGP
DC-30WP_001211218.1_3305AbBCP   LFGKVVALSMNKPFDQVLEKTIFFALGLKHSYVNVPKTQMQNYAFGYNQENQPIRVNPGP
DC-30WP_001211218.1_3346AbBCP   LFGKVVALSMNKPFDQVLEKTIFFALGLKHSYVNVPKTQMQNYAFGYNQENQPIRVNPGP
DC-30WP_001211218.1_1482AbBCP   LFGKVVALSMNKPFDQVLEKTIFFALGLKHSYVNVPKTQMQNYAFGYNQENQPIRVNPGP
DC-30WP_001211218.1_2574AbBCP   LFGKVVALSMNKPFDQVLEKTIFFALGLKHSYVNVPKTQMQNYAFGYNQENQPIRVNPGP
ADC-30WP_001211218.1_831AbBCP   LFGKVVALSMNKPFDQVLEKTIFFALGLKHSYVNVPKTQMQNYAFGYNQENQPIRVNPGP
DC-30WP_001211218.1_1826AbBCP   LFGKVVALSMNKPFDQVLEKTIFFALGLKHSYVNVPKTQMQNYAFGYNQENQPIRVNPGP
DC-30WP_001211218.1_1024AbBCP   LFGKVVALSMNKPFDQVLEKTIFFALGLKHSYVNVPKTQMQNYAFGYNQENQPIRVNPGP
DC-30WP_001211218.1_1618AbBCP   LFGKVVALSMNKPFDQVLEKTIFFALGLKHSYVNVPKTQMQNYAFGYNQENQPIRVNPGP
ADC-30WP_001211218.1_301AbBCP   LFGKVVALSMNKPFDQVLEKTIFFALGLKHSYVNVPKTQMQNYAFGYNQENQPIRVNPGP
ADC-30WP_001211218.1_940AbBCP   LFGKVVALSMNKPFDQVLEKTIFFALGLKHSYVNVPKTQMQNYAFGYNQENQPIRVNPGP
DC-30WP_001211218.1_2949AbBCP   LFGKVVALSMNKPFDQVLEKTIFFALGLKHSYVNVPKTQMQNYAFGYNQENQPIRVNPGP
DC-30WP_001211218.1_1028AbBCP   LFGKVVALSMNKPFDQVLEKTIFFALGLKHSYVNVPKTQMQNYAFGYNQENQPIRVNPGP
DC-30WP_001211218.1_2526AbBCP   LFGKVVALSMNKPFDQVLEKTIFFALGLKHSYVNVPKTQMQNYAFGYNQENQPIRVNPGP
DC-30WP_001211218.1_1040AbBCP   LFGKVVALSMNKPFDQVLEKTIFFALGLKHSYVNVPKTQMQNYAFGYNQENQPIRVNPGP
DC-30WP_001211218.1_1089AbBCP   LFGKVVALSMNKPFDQVLEKTIFFALGLKHSYVNVPKTQMQNYAFGYNQENQPIRVNPGP
DC-30WP_001211218.1_1099AbBCP   LFGKVVALSMNKPFDQVLEKTIFFALGLKHSYVNVPKTQMQNYAFGYNQENQPIRVNPGP
DC-30WP_001211218.1_1206AbBCP   LFGKVVALSMNKPFDQVLEKTIFFALGLKHSYVNVPKTQMQNYAFGYNQENQPIRVNPGP
DC-30WP_001211218.1_1167AbBCP   LFGKVVALSMNKPFDQVLEKTIFFALGLKHSYVNVPKTQMQNYAFGYNQENQPIRVNPGP
DC-30WP_001211218.1_1186AbBCP   LFGKVVALSMNKPFDQVLEKTIFFALGLKHSYVNVPKTQMQNYAFGYNQENQPIRVNPGP
DC-30WP_001211218.1_1165AbBCP   LFGKVVALSMNKPFDQVLEKTIFFALGLKHSYVNVPKTQMQNYAFGYNQENQPIRVNPGP
DC-30WP_001211218.1_1077AbBCP   LFGKVVALSMNKPFDQVLEKTIFFALGLKHSYVNVPKTQMQNYAFGYNQENQPIRVNPGP
DC-30WP_001211218.1_1078AbBCP   LFGKVVALSMNKPFDQVLEKTIFFALGLKHSYVNVPKTQMQNYAFGYNQENQPIRVNPGP
DC-30WP_001211218.1_1071AbBCP   LFGKVVALSMNKPFDQVLEKTIFFALGLKHSYVNVPKTQMQNYAFGYNQENQPIRVNPGP
DC-30WP_001211218.1_2694AbBCP   LFGKVVALSMNKPFDQVLEKTIFFALGLKHSYVNVPKTQMQNYAFGYNQENQPIRVNPGP
DC-30WP_001211218.1_3340AbBCP   LFGKVVALSMNKPFDQVLEKTIFFALGLKHSYVNVPKTQMQNYAFGYNQENQPIRVNPGP
DC-30WP_001211218.1_1022AbBCP   LFGKVVALSMNKPFDQVLEKTIFFALGLKHSYVNVPKTQMQNYAFGYNQENQPIRVNPGP
DC-30WP_001211218.1_1025AbBCP   LFGKVVALSMNKPFDQVLEKTIFFALGLKHSYVNVPKTQMQNYAFGYNQENQPIRVNPGP
DC-30WP_001211218.1_1158AbBCP   LFGKVVALSMNKPFDQVLEKTIFFALGLKHSYVNVPKTQMQNYAFGYNQENQPIRVNPGP
DC-30WP_001211218.1_1020AbBCP   LFGKVVALSMNKPFDQVLEKTIFFALGLKHSYVNVPKTQMQNYAFGYNQENQPIRVNPGP
DC-30WP_001211218.1_1021AbBCP   LFGKVVALSMNKPFDQVLEKTIFFALGLKHSYVNVPKTQMQNYAFGYNQENQPIRVNPGP
ADC-30WP_001211218.1_532AbBCP   LFGKVVALSMNKPFDQVLEKTIFFALGLKHSYVNVPKTQMQNYAFGYNQENQPIRVNPGP
ADC-30WP_001211218.1_539AbBCP   LFGKVVALSMNKPFDQVLEKTIFFALGLKHSYVNVPKTQMQNYAFGYNQENQPIRVNPGP
DC-30WP_001211218.1_1033AbBCP   LFGKVVALSMNKPFDQVLEKTIFFALGLKHSYVNVPKTQMQNYAFGYNQENQPIRVNPGP
DC-30WP_001211218.1_1069AbBCP   LFGKVVALSMNKPFDQVLEKTIFFALGLKHSYVNVPKTQMQNYAFGYNQENQPIRVNPGP
DC-30WP_001211218.1_1029AbBCP   LFGKVVALSMNKPFDQVLEKTIFFALGLKHSYVNVPKTQMQNYAFGYNQENQPIRVNPGP
DC-30WP_001211218.1_1030AbBCP   LFGKVVALSMNKPFDQVLEKTIFFALGLKHSYVNVPKTQMQNYAFGYNQENQPIRVNPGP
DC-30WP_001211218.1_1096AbBCP   LFGKVVALSMNKPFDQVLEKTIFFALGLKHSYVNVPKTQMQNYAFGYNQENQPIRVNPGP
DC-30WP_001211218.1_1037AbBCP   LFGKVVALSMNKPFDQVLEKTIFFALGLKHSYVNVPKTQMQNYAFGYNQENQPIRVNPGP
DC-30WP_001211218.1_1098AbBCP   LFGKVVALSMNKPFDQVLEKTIFFALGLKHSYVNVPKTQMQNYAFGYNQENQPIRVNPGP
DC-30WP_001211218.1_1046AbBCP   LFGKVVALSMNKPFDQVLEKTIFFALGLKHSYVNVPKTQMQNYAFGYNQENQPIRVNPGP
DC-30WP_001211218.1_1032AbBCP   LFGKVVALSMNKPFDQVLEKTIFFALGLKHSYVNVPKTQMQNYAFGYNQENQPIRVNPGP
DC-30WP_001211218.1_1070AbBCP   LFGKVVALSMNKPFDQVLEKTIFFALGLKHSYVNVPKTQMQNYAFGYNQENQPIRVNPGP
DC-30WP_001211218.1_1139AbBCP   LFGKVVALSMNKPFDQVLEKTIFFALGLKHSYVNVPKTQMQNYAFGYNQENQPIRVNPGP
DC-30WP_001211218.1_1031AbBCP   LFGKVVALSMNKPFDQVLEKTIFFALGLKHSYVNVPKTQMQNYAFGYNQENQPIRVNPGP
DC-30WP_001211218.1_1066AbBCP   LFGKVVALSMNKPFDQVLEKTIFFALGLKHSYVNVPKTQMQNYAFGYNQENQPIRVNPGP
DC-30WP_001211218.1_1102AbBCP   LFGKVVALSMNKPFDQVLEKTIFFALGLKHSYVNVPKTQMQNYAFGYNQENQPIRVNPGP
ADC-30USX57838.1_2957AbBCP      LFGKVVALSMNKPFDQVLEKTIFFALGLKHSYVNVPKTQMQNYAFGYNQENQPIRVNPGP
ADC-30USX59985.1_1918AbBCP      LFGKVVALSMNKPFDQVLEKTIFFALGLKHSYVNVPKTQMQNYAFGYNQENQPIRVNPGP
ADC-30USX63799.1_2772AbBCP      LFGKVVALSMNKPFDQVLEKTIFFALGLKHSYVNVPKTQMQNYAFGYNQENQPIRVNPGP
ADC-30USX74012.1_305AbBCP       LFGKVVALSMNKPFDQVLEKTIFFALGLKHSYVNVPKTQMQNYAFGYNQENQPIRVNPGP
ADC-30USZ52468.1_2397AbBCP      LFGKVVALSMNKPFDQVLEKTIFFALGLKHSYVNVPKTQMQNYAFGYNQENQPIRVNPGP
DC-30WP_001211218.1_2008AbBCP   LFGKVVALSMNKPFDQVLEKTIFFALGLKHSYVNVPKTQMQNYAFGYNQENQPIRVNPGP
ADC-30WCL78976.1_1026AbBCP      LFGKVVALSMNKPFDQVLEKTIFFALGLKHSYVNVPKTQMQNYAFGYNQENQPIRVNPGP
ADC-30WDO27826.1_1034AbBCP      LFGKVVALSMNKPFDQVLEKTIFFALGLKHSYVNVPKTQMQNYAFGYNQENQPIRVNPGP
DC-30WP_001211218.1_2125AbBCP   LFGKVVALSMNKPFDQVLEKTIFFALGLKHSYVNVPKTQMQNYAFGYNQENQPIRVNPGP
DC-30WP_001211218.1_2729AbBCP   LFGKVVALSMNKPFDQVLEKTIFFALGLKHSYVNVPKTQMQNYAFGYNQENQPIRVNPGP
ADC-30ADX93237.1_2806AbBCP      LFGKVVALSMNKPFDQVLEKTIFFALGLKHSYVNVPKTQMQNYAFGYNQENQPIRVNPGP
ADC-82WP_001211216.1_2747AbOC   LFGKVVALSMNKPFDQVLEKTIFFALGLKHSYVNVPKTQMQNYAFGYNQENQPIRVNPGP
DC-82WP_001211216.1_1093AbBOC   LFGKVVALSMNKPFDQVLEKTIFFALGLKHSYVNVPKTQMQNYAFGYNQENQPIRVNPGP
DC-82WP_001211216.1_1071AbBOC   LFGKVVALSMNKPFDQVLEKTIFFALGLKHSYVNVPKTQMQNYAFGYNQENQPIRVNPGP
DC-82WP_001211216.1_1093AbBCP   LFGKVVALSMNKPFDQVLEKTIFFALGLKHSYVNVPKTQMQNYAFGYNQENQPIRVNPGP
DC-82WP_001211216.1_1071AbBCP   LFGKVVALSMNKPFDQVLEKTIFFALGLKHSYVNVPKTQMQNYAFGYNQENQPIRVNPGP
DC-162WP_031980335.1_1153AbOC   LFGKVVALSMNKPFDQVLEKTIFFALGLKHSYVNVPKTQMQNYAFGYNQENQPIRVNPGP
C-162WP_031980335.1_1028AbBOC   LFGKVVALSMNKPFDQVLEKTIFFALGLKHSYVNVPKTQMQNYAFGYNQENQPIRVNPGP
C-162WP_031980335.1_1023AbBOC   LFGKVVALSMNKPFDQVLEKTIFFALGLKHSYVNVPKTQMQNYAFGYNQENQPIRVNPGP
C-162WP_031980335.1_1028AbBCP   LFGKVVALSMNKPFDQVLEKTIFFALGLKHSYVNVPKTQMQNYAFGYNQENQPIRVNPGP
C-162WP_031980335.1_1023AbBCP   LFGKVVALSMNKPFDQVLEKTIFFALGLKHSYVNVPKTQMQNYAFGYNQENQPIRVNPGP
ADC-73WP_001211219.1_1012AbOC   LFGKVVALSMNKPFDQVLEKTIFFALGLKHSYVNVPKTQMQNYAFGYNQENQPIRVNPGP
ADC-73WP_001211219.1_1953AbOC   LFGKVVALSMNKPFDQVLEKTIFFALGLKHSYVNVPKTQMQNYAFGYNQENQPIRVNPGP
ADC-73WP_001211219.1_1014AbOC   LFGKVVALSMNKPFDQVLEKTIFFALGLKHSYVNVPKTQMQNYAFGYNQENQPIRVNPGP
ADC-73WP_001211219.1_1041AbOC   LFGKVVALSMNKPFDQVLEKTIFFALGLKHSYVNVPKTQMQNYAFGYNQENQPIRVNPGP
ADC-73WP_001211219.1_1028AbOC   LFGKVVALSMNKPFDQVLEKTIFFALGLKHSYVNVPKTQMQNYAFGYNQENQPIRVNPGP
ADC-73WP_001211219.1_2674AbOC   LFGKVVALSMNKPFDQVLEKTIFFALGLKHSYVNVPKTQMQNYAFGYNQENQPIRVNPGP
ADC-73WP_001211219.1_462AbOC    LFGKVVALSMNKPFDQVLEKTIFFALGLKHSYVNVPKTQMQNYAFGYNQENQPIRVNPGP
ADC-73WP_001211219.1_3530AbOC   LFGKVVALSMNKPFDQVLEKTIFFALGLKHSYVNVPKTQMQNYAFGYNQENQPIRVNPGP
ADC-73WP_001211219.1_3621AbOC   LFGKVVALSMNKPFDQVLEKTIFFALGLKHSYVNVPKTQMQNYAFGYNQENQPIRVNPGP
ADC-73WP_001211219.1_3381AbOC   LFGKVVALSMNKPFDQVLEKTIFFALGLKHSYVNVPKTQMQNYAFGYNQENQPIRVNPGP
ADC-73WP_001211219.1_2515AbOC   LFGKVVALSMNKPFDQVLEKTIFFALGLKHSYVNVPKTQMQNYAFGYNQENQPIRVNPGP
ADC-73WP_001211219.1_3574AbOC   LFGKVVALSMNKPFDQVLEKTIFFALGLKHSYVNVPKTQMQNYAFGYNQENQPIRVNPGP
ADC-73WP_001211219.1_1656AbOC   LFGKVVALSMNKPFDQVLEKTIFFALGLKHSYVNVPKTQMQNYAFGYNQENQPIRVNPGP
ADC-73WP_001211219.1_2591AbOC   LFGKVVALSMNKPFDQVLEKTIFFALGLKHSYVNVPKTQMQNYAFGYNQENQPIRVNPGP
ADC-73WP_001211219.1_2602AbOC   LFGKVVALSMNKPFDQVLEKTIFFALGLKHSYVNVPKTQMQNYAFGYNQENQPIRVNPGP
ADC-73WP_001211219.1_910AbOC    LFGKVVALSMNKPFDQVLEKTIFFALGLKHSYVNVPKTQMQNYAFGYNQENQPIRVNPGP
ADC-73WP_001211219.1_394AbOC    LFGKVVALSMNKPFDQVLEKTIFFALGLKHSYVNVPKTQMQNYAFGYNQENQPIRVNPGP
ADC-73WP_001211219.1_1030AbOC   LFGKVVALSMNKPFDQVLEKTIFFALGLKHSYVNVPKTQMQNYAFGYNQENQPIRVNPGP
ADC-73WP_001211219.1_1023AbOC   LFGKVVALSMNKPFDQVLEKTIFFALGLKHSYVNVPKTQMQNYAFGYNQENQPIRVNPGP
ADC-73UYQ66995.1_1967AbOC      LFGKVVALSMNKPFDQVLEKTIFFALGLKHSYVNVPKTQMQNYAFGYNQENQPIRVNPGP
ADC-73WP_001211219.1_906AbBOC   LFGKVVALSMNKPFDQVLEKTIFFALGLKHSYVNVPKTQMQNYAFGYNQENQPIRVNPGP
DC-73WP_001211219.1_2649AbBOC   LFGKVVALSMNKPFDQVLEKTIFFALGLKHSYVNVPKTQMQNYAFGYNQENQPIRVNPGP
DC-73WP_001211219.1_2693AbBOC   LFGKVVALSMNKPFDQVLEKTIFFALGLKHSYVNVPKTQMQNYAFGYNQENQPIRVNPGP
ADC-73AQU57925.1_2703AbBOC      LFGKVVALSMNKPFDQVLEKTIFFALGLKHSYVNVPKTQMQNYAFGYNQENQPIRVNPGP
DC-73WP_001211219.1_1026AbBOC   LFGKVVALSMNKPFDQVLEKTIFFALGLKHSYVNVPKTQMQNYAFGYNQENQPIRVNPGP
DC-73WP_001211219.1_1019AbBOC   LFGKVVALSMNKPFDQVLEKTIFFALGLKHSYVNVPKTQMQNYAFGYNQENQPIRVNPGP
DC-73WP_001211219.1_1024AbBOC   LFGKVVALSMNKPFDQVLEKTIFFALGLKHSYVNVPKTQMQNYAFGYNQENQPIRVNPGP
ADC-73WP_001211219.1_930AbBOC   LFGKVVALSMNKPFDQVLEKTIFFALGLKHSYVNVPKTQMQNYAFGYNQENQPIRVNPGP
DC-73WP_001211219.1_2609AbBOC   LFGKVVALSMNKPFDQVLEKTIFFALGLKHSYVNVPKTQMQNYAFGYNQENQPIRVNPGP
DC-73WP_001211219.1_3456AbBOC   LFGKVVALSMNKPFDQVLEKTIFFALGLKHSYVNVPKTQMQNYAFGYNQENQPIRVNPGP
DC-73WP_001211219.1_2614AbBOC   LFGKVVALSMNKPFDQVLEKTIFFALGLKHSYVNVPKTQMQNYAFGYNQENQPIRVNPGP
DC-73WP_001211219.1_1025AbBOC   LFGKVVALSMNKPFDQVLEKTIFFALGLKHSYVNVPKTQMQNYAFGYNQENQPIRVNPGP
DC-73WP_001211219.1_1030AbBOC   LFGKVVALSMNKPFDQVLEKTIFFALGLKHSYVNVPKTQMQNYAFGYNQENQPIRVNPGP
DC-73WP_001211219.1_1173AbBOC   LFGKVVALSMNKPFDQVLEKTIFFALGLKHSYVNVPKTQMQNYAFGYNQENQPIRVNPGP
DC-73WP_001211219.1_1021AbBOC   LFGKVVALSMNKPFDQVLEKTIFFALGLKHSYVNVPKTQMQNYAFGYNQENQPIRVNPGP
DC-73WP_001211219.1_2449AbBOC   LFGKVVALSMNKPFDQVLEKTIFFALGLKHSYVNVPKTQMQNYAFGYNQENQPIRVNPGP
DC-73WP_001211219.1_1176AbBOC   LFGKVVALSMNKPFDQVLEKTIFFALGLKHSYVNVPKTQMQNYAFGYNQENQPIRVNPGP
DC-73WP_001211219.1_1161AbBOC   LFGKVVALSMNKPFDQVLEKTIFFALGLKHSYVNVPKTQMQNYAFGYNQENQPIRVNPGP
DC-73WP_001211219.1_1070AbBOC   LFGKVVALSMNKPFDQVLEKTIFFALGLKHSYVNVPKTQMQNYAFGYNQENQPIRVNPGP
DC-73WP_001211219.1_1079AbBOC   LFGKVVALSMNKPFDQVLEKTIFFALGLKHSYVNVPKTQMQNYAFGYNQENQPIRVNPGP
DC-73WP_001211219.1_1078AbBOC   LFGKVVALSMNKPFDQVLEKTIFFALGLKHSYVNVPKTQMQNYAFGYNQENQPIRVNPGP
DC-73WP_001211219.1_1037AbBOC   LFGKVVALSMNKPFDQVLEKTIFFALGLKHSYVNVPKTQMQNYAFGYNQENQPIRVNPGP
DC-73WP_001211219.1_1035AbBOC   LFGKVVALSMNKPFDQVLEKTIFFALGLKHSYVNVPKTQMQNYAFGYNQENQPIRVNPGP
DC-73WP_001211219.1_1033AbBOC   LFGKVVALSMNKPFDQVLEKTIFFALGLKHSYVNVPKTQMQNYAFGYNQENQPIRVNPGP
DC-73WP_001211219.1_1041AbBOC   LFGKVVALSMNKPFDQVLEKTIFFALGLKHSYVNVPKTQMQNYAFGYNQENQPIRVNPGP
DC-73WP_001211219.1_1023AbBOC   LFGKVVALSMNKPFDQVLEKTIFFALGLKHSYVNVPKTQMQNYAFGYNQENQPIRVNPGP
ADC-73USX69547.1_2386AbBOC      LFGKVVALSMNKPFDQVLEKTIFFALGLKHSYVNVPKTQMQNYAFGYNQENQPIRVNPGP
DC-73WP_001211219.1_1034AbBOC   LFGKVVALSMNKPFDQVLEKTIFFALGLKHSYVNVPKTQMQNYAFGYNQENQPIRVNPGP
ADC-73WP_001211219.1_852AbBOC   LFGKVVALSMNKPFDQVLEKTIFFALGLKHSYVNVPKTQMQNYAFGYNQENQPIRVNPGP
DC-73WP_001211219.1_3070AbBOC   LFGKVVALSMNKPFDQVLEKTIFFALGLKHSYVNVPKTQMQNYAFGYNQENQPIRVNPGP
ADC-73WBS60953.1_1026AbBOC      LFGKVVALSMNKPFDQVLEKTIFFALGLKHSYVNVPKTQMQNYAFGYNQENQPIRVNPGP
ADC-73WDC39660.1_1023AbBOC      LFGKVVALSMNKPFDQVLEKTIFFALGLKHSYVNVPKTQMQNYAFGYNQENQPIRVNPGP
ADC-73WDC73972.1_1415AbBOC      LFGKVVALSMNKPFDQVLEKTIFFALGLKHSYVNVPKTQMQNYAFGYNQENQPIRVNPGP
ADC-73WDC70172.1_1037AbBOC      LFGKVVALSMNKPFDQVLEKTIFFALGLKHSYVNVPKTQMQNYAFGYNQENQPIRVNPGP
DC-73WP_001211219.1_1101AbBOC   LFGKVVALSMNKPFDQVLEKTIFFALGLKHSYVNVPKTQMQNYAFGYNQENQPIRVNPGP
DC-73WP_001211219.1_1667AbBOC   LFGKVVALSMNKPFDQVLEKTIFFALGLKHSYVNVPKTQMQNYAFGYNQENQPIRVNPGP
ADC-73WP_001211219.1_287AbBOC   LFGKVVALSMNKPFDQVLEKTIFFALGLKHSYVNVPKTQMQNYAFGYNQENQPIRVNPGP
DC-73WP_001211219.1_3177AbBOC   LFGKVVALSMNKPFDQVLEKTIFFALGLKHSYVNVPKTQMQNYAFGYNQENQPIRVNPGP
DC-73WP_001211219.1_2501AbBOC   LFGKVVALSMNKPFDQVLEKTIFFALGLKHSYVNVPKTQMQNYAFGYNQENQPIRVNPGP
DC-73WP_001211219.1_2535AbBOC   LFGKVVALSMNKPFDQVLEKTIFFALGLKHSYVNVPKTQMQNYAFGYNQENQPIRVNPGP
DC-73WP_001211219.1_3150AbBOC   LFGKVVALSMNKPFDQVLEKTIFFALGLKHSYVNVPKTQMQNYAFGYNQENQPIRVNPGP
DC-73WP_001211219.1_3714AbBOC   LFGKVVALSMNKPFDQVLEKTIFFALGLKHSYVNVPKTQMQNYAFGYNQENQPIRVNPGP
ADC-73WP_001211219.1_722AbBOC   LFGKVVALSMNKPFDQVLEKTIFFALGLKHSYVNVPKTQMQNYAFGYNQENQPIRVNPGP
DC-73WP_001211219.1_3650AbBOC   LFGKVVALSMNKPFDQVLEKTIFFALGLKHSYVNVPKTQMQNYAFGYNQENQPIRVNPGP
ADC-73WP_001211219.1_568AbBOC   LFGKVVALSMNKPFDQVLEKTIFFALGLKHSYVNVPKTQMQNYAFGYNQENQPIRVNPGP
DC-73WP_001211219.1_3226AbBOC   LFGKVVALSMNKPFDQVLEKTIFFALGLKHSYVNVPKTQMQNYAFGYNQENQPIRVNPGP
ADC-73WP_001211219.1_556AbBOC   LFGKVVALSMNKPFDQVLEKTIFFALGLKHSYVNVPKTQMQNYAFGYNQENQPIRVNPGP
```

DC-73WP_001211219.1_2436AbBOC	LFGKVVALSMNKPFDQVLEKTIFPALGLKHSYVNVPKTQMQNYAFGYNQENQPIRVNPGP
DC-73WP_001211219.1_1029AbBOC	LFGKVVALSMNKPFDQVLEKTIFPALGLKHSYVNVPKTQMQNYAFGYNQENQPIRVNPGP
DC-73WP_001211219.1_1013AbBOC	LFGKVVALSMNKPFDQVLEKTIFPALGLKHSYVNVPKTQMQNYAFGYNQENQPIRVNPGP
ADC-73WP_001211219.1_906AbBCP	LFGKVVALSMNKPFDQVLEKTIFPALGLKHSYVNVPKTQMQNYAFGYNQENQPIRVNPGP
DC-73WP_001211219.1_2649AbBCP	LFGKVVALSMNKPFDQVLEKTIFPALGLKHSYVNVPKTQMQNYAFGYNQENQPIRVNPGP
DC-73WP_001211219.1_2693AbBCP	LFGKVVALSMNKPFDQVLEKTIFPALGLKHSYVNVPKTQMQNYAFGYNQENQPIRVNPGP
ADC-73AQU57925.1_2703AbBCP	LFGKVVALSMNKPFDQVLEKTIFPALGLKHSYVNVPKTQMQNYAFGYNQENQPIRVNPGP
DC-73WP_001211219.1_1026AbBCP	LFGKVVALSMNKPFDQVLEKTIFPALGLKHSYVNVPKTQMQNYAFGYNQENQPIRVNPGP
DC-73WP_001211219.1_1019AbBCP	LFGKVVALSMNKPFDQVLEKTIFPALGLKHSYVNVPKTQMQNYAFGYNQENQPIRVNPGP
DC-73WP_001211219.1_1024AbBCP	LFGKVVALSMNKPFDQVLEKTIFPALGLKHSYVNVPKTQMQNYAFGYNQENQPIRVNPGP
ADC-73WP_001211219.1_930AbBCP	LFGKVVALSMNKPFDQVLEKTIFPALGLKHSYVNVPKTQMQNYAFGYNQENQPIRVNPGP
DC-73WP_001211219.1_2609AbBCP	LFGKVVALSMNKPFDQVLEKTIFPALGLKHSYVNVPKTQMQNYAFGYNQENQPIRVNPGP
DC-73WP_001211219.1_3456AbBCP	LFGKVVALSMNKPFDQVLEKTIFPALGLKHSYVNVPKTQMQNYAFGYNQENQPIRVNPGP
DC-73WP_001211219.1_2614AbBCP	LFGKVVALSMNKPFDQVLEKTIFPALGLKHSYVNVPKTQMQNYAFGYNQENQPIRVNPGP
DC-73WP_001211219.1_1025AbBCP	LFGKVVALSMNKPFDQVLEKTIFPALGLKHSYVNVPKTQMQNYAFGYNQENQPIRVNPGP
DC-73WP_001211219.1_1030AbBCP	LFGKVVALSMNKPFDQVLEKTIFPALGLKHSYVNVPKTQMQNYAFGYNQENQPIRVNPGP
DC-73WP_001211219.1_1173AbBCP	LFGKVVALSMNKPFDQVLEKTIFPALGLKHSYVNVPKTQMQNYAFGYNQENQPIRVNPGP
DC-73WP_001211219.1_1021AbBCP	LFGKVVALSMNKPFDQVLEKTIFPALGLKHSYVNVPKTQMQNYAFGYNQENQPIRVNPGP
DC-73WP_001211219.1_2449AbBCP	LFGKVVALSMNKPFDQVLEKTIFPALGLKHSYVNVPKTQMQNYAFGYNQENQPIRVNPGP
DC-73WP_001211219.1_1176AbBCP	LFGKVVALSMNKPFDQVLEKTIFPALGLKHSYVNVPKTQMQNYAFGYNQENQPIRVNPGP
DC-73WP_001211219.1_1161AbBCP	LFGKVVALSMNKPFDQVLEKTIFPALGLKHSYVNVPKTQMQNYAFGYNQENQPIRVNPGP
DC-73WP_001211219.1_1070AbBCP	LFGKVVALSMNKPFDQVLEKTIFPALGLKHSYVNVPKTQMQNYAFGYNQENQPIRVNPGP
DC-73WP_001211219.1_1079AbBCP	LFGKVVALSMNKPFDQVLEKTIFPALGLKHSYVNVPKTQMQNYAFGYNQENQPIRVNPGP
DC-73WP_001211219.1_1078AbBCP	LFGKVVALSMNKPFDQVLEKTIFPALGLKHSYVNVPKTQMQNYAFGYNQENQPIRVNPGP
DC-73WP_001211219.1_1037AbBCP	LFGKVVALSMNKPFDQVLEKTIFPALGLKHSYVNVPKTQMQNYAFGYNQENQPIRVNPGP
DC-73WP_001211219.1_1035AbBCP	LFGKVVALSMNKPFDQVLEKTIFPALGLKHSYVNVPKTQMQNYAFGYNQENQPIRVNPGP
DC-73WP_001211219.1_1033AbBCP	LFGKVVALSMNKPFDQVLEKTIFPALGLKHSYVNVPKTQMQNYAFGYNQENQPIRVNPGP
DC-73WP_001211219.1_1041AbBCP	LFGKVVALSMNKPFDQVLEKTIFPALGLKHSYVNVPKTQMQNYAFGYNQENQPIRVNPGP
DC-73WP_001211219.1_1023AbBCP	LFGKVVALSMNKPFDQVLEKTIFPALGLKHSYVNVPKTQMQNYAFGYNQENQPIRVNPGP
ADC-73USX69547.1_2386AbBCP	LFGKVVALSMNKPFDQVLEKTIFPALGLKHSYVNVPKTQMQNYAFGYNQENQPIRVNPGP
DC-73WP_001211219.1_1034AbBCP	LFGKVVALSMNKPFDQVLEKTIFPALGLKHSYVNVPKTQMQNYAFGYNQENQPIRVNPGP
ADC-73WP_001211219.1_852AbBCP	LFGKVVALSMNKPFDQVLEKTIFPALGLKHSYVNVPKTQMQNYAFGYNQENQPIRVNPGP
DC-73WP_001211219.1_3070AbBCP	LFGKVVALSMNKPFDQVLEKTIFPALGLKHSYVNVPKTQMQNYAFGYNQENQPIRVNPGP
ADC-73WBS60953.1_1026AbBCP	LFGKVVALSMNKPFDQVLEKTIFPALGLKHSYVNVPKTQMQNYAFGYNQENQPIRVNPGP
ADC-73WDC39660.1_1023AbBCP	LFGKVVALSMNKPFDQVLEKTIFPALGLKHSYVNVPKTQMQNYAFGYNQENQPIRVNPGP
ADC-73WDC73972.1_1415AbBCP	LFGKVVALSMNKPFDQVLEKTIFPALGLKHSYVNVPKTQMQNYAFGYNQENQPIRVNPGP
ADC-73WDC70172.1_1037AbBCP	LFGKVVALSMNKPFDQVLEKTIFPALGLKHSYVNVPKTQMQNYAFGYNQENQPIRVNPGP
DC-73WP_001211219.1_1101AbBCP	LFGKVVALSMNKPFDQVLEKTIFPALGLKHSYVNVPKTQMQNYAFGYNQENQPIRVNPGP
DC-73WP_001211219.1_1667AbBCP	LFGKVVALSMNKPFDQVLEKTIFPALGLKHSYVNVPKTQMQNYAFGYNQENQPIRVNPGP
ADC-73WP_001211219.1_287AbBCP	LFGKVVALSMNKPFDQVLEKTIFPALGLKHSYVNVPKTQMQNYAFGYNQENQPIRVNPGP
DC-73WP_001211219.1_3177AbBCP	LFGKVVALSMNKPFDQVLEKTIFPALGLKHSYVNVPKTQMQNYAFGYNQENQPIRVNPGP
DC-73WP_001211219.1_2501AbBCP	LFGKVVALSMNKPFDQVLEKTIFPALGLKHSYVNVPKTQMQNYAFGYNQENQPIRVNPGP
DC-73WP_001211219.1_2535AbBCP	LFGKVVALSMNKPFDQVLEKTIFPALGLKHSYVNVPKTQMQNYAFGYNQENQPIRVNPGP
DC-73WP_001211219.1_3150AbBCP	LFGKVVALSMNKPFDQVLEKTIFPALGLKHSYVNVPKTQMQNYAFGYNQENQPIRVNPGP
DC-73WP_001211219.1_3714AbBCP	LFGKVVALSMNKPFDQVLEKTIFPALGLKHSYVNVPKTQMQNYAFGYNQENQPIRVNPGP
ADC-73WP_001211219.1_722AbBCP	LFGKVVALSMNKPFDQVLEKTIFPALGLKHSYVNVPKTQMQNYAFGYNQENQPIRVNPGP
DC-73WP_001211219.1_3650AbBCP	LFGKVVALSMNKPFDQVLEKTIFPALGLKHSYVNVPKTQMQNYAFGYNQENQPIRVNPGP
ADC-73WP_001211219.1_568AbBCP	LFGKVVALSMNKPFDQVLEKTIFPALGLKHSYVNVPKTQMQNYAFGYNQENQPIRVNPGP
DC-73WP_001211219.1_3226AbBCP	LFGKVVALSMNKPFDQVLEKTIFPALGLKHSYVNVPKTQMQNYAFGYNQENQPIRVNPGP
ADC-73WP_001211219.1_556AbBCP	LFGKVVALSMNKPFDQVLEKTIFPALGLKHSYVNVPKTQMQNYAFGYNQENQPIRVNPGP
DC-73WP_001211219.1_2436AbBCP	LFGKVVALSMNKPFDQVLEKTIFPALGLKHSYVNVPKTQMQNYAFGYNQENQPIRVNPGP
DC-73WP_001211219.1_1029AbBCP	LFGKVVALSMNKPFDQVLEKTIFPALGLKHSYVNVPKTQMQNYAFGYNQENQPIRVNPGP
DC-73WP_001211219.1_1013AbBCP	LFGKVVALSMNKPFDQVLEKTIFPALGLKHSYVNVPKTQMQNYAFGYNQENQPIRVNPGP
ADC-73BDE18748.1_1038AbBCP	LFGKVVALSMNKPFDQVLEKTIFPALGLKHSYVNVPKTQMQNYAFGYNQENQPIRVNPGP
DC-115WP_001211215.1_2833AbOC	LFGKVVALSMNKPFDQVLEKTIFPALGLKHSYVNVPKTQMQNYAFGYNQENQPIRANPGP
C-115WP_001211215.1_1156AbBOC	LFGKVVALSMNKPFDQVLEKTIFPALGLKHSYVNVPKTQMQNYAFGYNQENQPIRANPGP
C-115WP_001211215.1_1156AbBCP	LFGKVVALSMNKPFDQVLEKTIFPALGLKHSYVNVPKTQMQNYAFGYNQENQPIRANPGP
ADC-5WP_038405930.1_2542AbOC	LFGKVVALSMNKPFDQVLEKTIFPALGLKHSYVNVPKTQMQNYAFGYNQENQPIRVNPGP
ADC-5WP_038405930.1_1113AbBOC	LFGKVVALSMNKPFDQVLEKTIFPALGLKHSYVNVPKTQMQNYAFGYNQENQPIRVNPGP
ADC-5WP_038405930.1_1113AbBCP	LFGKVVALSMNKPFDQVLEKTIFPALGLKHSYVNVPKTQMQNYAFGYNQENQPIRVNPGP
C-236WP_176673601.1_1026AbBOC	LFGKVVALSMNKPFDQVLEKTIFPALGLKHSYVNVAKTQMQNYAFGYNQENQPIRVNPGP
C-236WP_176673601.1_1026AbBCP	LFGKVVALSMNKPFDQVLEKTIFPALGLKHSYVNVAKTQMQNYAFGYNQENQPIRVNPGP
DC-217WP_060470109.1_3584AbOC	LFGKVVALSMNKPFDQVLEKTIFPALGLKHSYVNVPKTQMQNYAFGYNQENQPIRVNPGP
ADC-217USX66614.1_2290AbBOC	LFGKVVALSMNKPFDQVLEKTIFPALGLKHSYVNVPKTQMQNYAFGYNQENQPIRVNPGP
ADC-217USX66614.1_2290AbBCP	LFGKVVALSMNKPFDQVLEKTIFPALGLKHSYVNVPKTQMQNYAFGYNQENQPIRVNPGP
ADC-247WP_032058755.1_382AbOC	LFGKVVALSMNKPFDQVLEKTIFPALGLKHSYVNVPKTQMQNYAFGYNQENQPIRVNPGP
C-247WP_032058755.1_1936AbBOC	LFGKVVALSMNKPFDQVLEKTIFPALGLKHSYVNVPKTQMQNYAFGYNQENQPIRVNPGP
C-247WP_032058755.1_1936AbBCP	LFGKVVALSMNKPFDQVLEKTIFPALGLKHSYVNVPKTQMQNYAFGYNQENQPIRVNPGP
DC-203WP_032000414.1_2940AbOC	LFGKVVSLSMNKPFDQVLEKTIFPALGLKHSYVNVPKTQMQNYAFGYNQENQPIRVNPGP
C-203WP_032000414.1_1062AbBOC	LFGKVVSLSMNKPFDQVLEKTIFPALGLKHSYVNVPKTQMQNYAFGYNQENQPIRVNPGP
DC-203WP_032000414.1_129AbBOC	LFGKVVSLSMNKPFDQVLEKTIFPALGLKHSYVNVPKTQMQNYAFGYNQENQPIRVNPGP
C-203WP_032000414.1_2518AbBOC	LFGKVVSLSMNKPFDQVLEKTIFPALGLKHSYVNVPKTQMQNYAFGYNQENQPIRVNPGP
C-203WP_032000414.1_1062AbBCP	LFGKVVSLSMNKPFDQVLEKTIFPALGLKHSYVNVPKTQMQNYAFGYNQENQPIRVNPGP
DC-203WP_032000414.1_129AbBCP	LFGKVVSLSMNKPFDQVLEKTIFPALGLKHSYVNVPKTQMQNYAFGYNQENQPIRVNPGP
C-203WP_032000414.1_2518AbBCP	LFGKVVSLSMNKPFDQVLEKTIFPALGLKHSYVNVPKTQMQNYAFGYNQENQPIRVNPGP
DC-152WP_001211233.1_3734AbOC	LFGKVVALSMNKPFDQVLEKTIFPALGLKHSYVNVAKTQMQNYAFGYNQENQPIRVNPGP
C-152WP_001211233.1_1080AbBOC	LFGKVVALSMNKPFDQVLEKTIFPALGLKHSYVNVAKTQMQNYAFGYNQENQPIRVNPGP
DC-152WP_001211233.1_491AbBOC	LFGKVVALSMNKPFDQVLEKTIFPALGLKHSYVNVAKTQMQNYAFGYNQENQPIRVNPGP
ADC-152WP_001211233.1_46AbBOC	LFGKVVALSMNKPFDQVLEKTIFPALGLKHSYVNVAKTQMQNYAFGYNQENQPIRVNPGP
C-152WP_001211233.1_1545AbBOC	LFGKVVALSMNKPFDQVLEKTIFPALGLKHSYVNVAKTQMQNYAFGYNQENQPIRVNPGP
ADC-152WBS86980.1_1289AbBOC	LFGKVVALSMNKPFDQVLEKTIFPALGLKHSYVNVAKTQMQNYAFGYNQENQPIRVNPGP
ADC-152WBS81072.1_2581AbBOC	LFGKVVALSMNKPFDQVLEKTIFPALGLKHSYVNVAKTQMQNYAFGYNQENQPIRVNPGP
ADC-152WBS88504.1_2711AbBOC	LFGKVVALSMNKPFDQVLEKTIFPALGLKHSYVNVAKTQMQNYAFGYNQENQPIRVNPGP
ADC-152WBS92168.1_2401AbBOC	LFGKVVALSMNKPFDQVLEKTIFPALGLKHSYVNVAKTQMQNYAFGYNQENQPIRVNPGP
ADC-152WBS79617.1_1289AbBOC	LFGKVVALSMNKPFDQVLEKTIFPALGLKHSYVNVAKTQMQNYAFGYNQENQPIRVNPGP
C-152WP_001211233.1_1080AbBCP	LFGKVVALSMNKPFDQVLEKTIFPALGLKHSYVNVAKTQMQNYAFGYNQENQPIRVNPGP
DC-152WP_001211233.1_491AbBCP	LFGKVVALSMNKPFDQVLEKTIFPALGLKHSYVNVAKTQMQNYAFGYNQENQPIRVNPGP
ADC-152WP_001211233.1_46AbBCP	LFGKVVALSMNKPFDQVLEKTIFPALGLKHSYVNVAKTQMQNYAFGYNQENQPIRVNPGP
C-152WP_001211233.1_1545AbBCP	LFGKVVALSMNKPFDQVLEKTIFPALGLKHSYVNVAKTQMQNYAFGYNQENQPIRVNPGP
ADC-152WBS86980.1_1289AbBCP	LFGKVVALSMNKPFDQVLEKTIFPALGLKHSYVNVAKTQMQNYAFGYNQENQPIRVNPGP
ADC-152WBS81072.1_2581AbBCP	LFGKVVALSMNKPFDQVLEKTIFPALGLKHSYVNVAKTQMQNYAFGYNQENQPIRVNPGP
ADC-152WBS88504.1_2711AbBCP	LFGKVVALSMNKPFDQVLEKTIFPALGLKHSYVNVAKTQMQNYAFGYNQENQPIRVNPGP
ADC-152WBS92168.1_2401AbBCP	LFGKVVALSMNKPFDQVLEKTIFPALGLKHSYVNVAKTQMQNYAFGYNQENQPIRVNPGP
ADC-152WBS79617.1_1289AbBCP	LFGKVVALSMNKPFDQVLEKTIFPALGLKHSYVNVAKTQMQNYAFGYNQENQPIRVNPGP
ADC-52WP_001211232.1_990AbOC	LFGKVVALSMNKPFDQVLEKTIFPALGLKHSYVNVAKTQMQNYAFGYNQENQPIRVNPGP
DC-52WP_001211232.1_1087AbBOC	LFGKVVALSMNKPFDQVLEKTIFPALGLKHSYVNVAKTQMQNYAFGYNQENQPIRVNPGP
DC-52WP_001211232.1_1087AbBCP	LFGKVVALSMNKPFDQVLEKTIFPALGLKHSYVNVAKTQMQNYAFGYNQENQPIRVNPGP
C-163WP_057093064.1_2397AbBOC	LFGKVVALSMNKPFDQVLEKTIFPALGLKHSYVNVPKTQMQNYAFGYNQENQPIRVNPGP
C-163WP_057093064.1_2397AbBCP	LFGKVVALSMNKPFDQVLEKTIFPALGLKHSYVNVPKTQMQNYAFGYNQENQPIRVNPGP
ADC-33WP_001211220.1_2277AbOC	LFGKVVALSMNKPFDQVLEKTIFPALGLKHSYVNVPKTQMQNYAFGYNQENQPIRVNRGP
DC-33WP_001211220.1_1044AbBOC	LFGKVVALSMNKPFDQVLEKTIFPALGLKHSYVNVPKTQMQNYAFGYNQENQPIRVNRGP
DC-33WP_001211220.1_2452AbBOC	LFGKVVALSMNKPFDQVLEKTIFPALGLKHSYVNVPKTQMQNYAFGYNQENQPIRVNRGP
DC-33WP_001211220.1_1046AbBOC	LFGKVVALSMNKPFDQVLEKTIFPALGLKHSYVNVPKTQMQNYAFGYNQENQPIRVNRGP
DC-33WP_001211220.1_1025AbBOC	LFGKVVALSMNKPFDQVLEKTIFPALGLKHSYVNVPKTQMQNYAFGYNQENQPIRVNRGP
DC-33WP_001211220.1_1026AbBOC	LFGKVVALSMNKPFDQVLEKTIFPALGLKHSYVNVPKTQMQNYAFGYNQENQPIRVNRGP
DC-33WP_001211220.1_1033AbBOC	LFGKVVALSMNKPFDQVLEKTIFPALGLKHSYVNVPKTQMQNYAFGYNQENQPIRVNRGP
DC-33WP_001211220.1_1035AbBOC	LFGKVVALSMNKPFDQVLEKTIFPALGLKHSYVNVPKTQMQNYAFGYNQENQPIRVNRGP
DC-33WP_001211220.1_1041AbBOC	LFGKVVALSMNKPFDQVLEKTIFPALGLKHSYVNVPKTQMQNYAFGYNQENQPIRVNRGP
DC-33WP_001211220.1_1044AbBCP	LFGKVVALSMNKPFDQVLEKTIFPALGLKHSYVNVPKTQMQNYAFGYNQENQPIRVNRGP
DC-33WP_001211220.1_2452AbBCP	LFGKVVALSMNKPFDQVLEKTIFPALGLKHSYVNVPKTQMQNYAFGYNQENQPIRVNRGP
DC-33WP_001211220.1_1046AbBCP	LFGKVVALSMNKPFDQVLEKTIFPALGLKHSYVNVPKTQMQNYAFGYNQENQPIRVNRGP
DC-33WP_001211220.1_1025AbBCP	LFGKVVALSMNKPFDQVLEKTIFPALGLKHSYVNVPKTQMQNYAFGYNQENQPIRVNRGP
DC-33WP_001211220.1_1026AbBCP	LFGKVVALSMNKPFDQVLEKTIFPALGLKHSYVNVPKTQMQNYAFGYNQENQPIRVNRGP
DC-33WP_001211220.1_1033AbBCP	LFGKVVALSMNKPFDQVLEKTIFPALGLKHSYVNVPKTQMQNYAFGYNQENQPIRVNRGP
DC-33WP_001211220.1_1035AbBCP	LFGKVVALSMNKPFDQVLEKTIFPALGLKHSYVNVPKTQMQNYAFGYNQENQPIRVNRGP
DC-33WP_001211220.1_1041AbBCP	LFGKVVALSMNKPFDQVLEKTIFPALGLKHSYVNVPKTQMQNYAFGYNQENQPIRVNRGP
C-212WP_031975357.1_1297AbBOC	LFGKVVALSMNKPFDQVLEKTIFPALGLKHSYVNVPKTQMQNYDFGYNQENQPIRVNPGP
DC-212WP_031975357.1_501AbBOC	LFGKVVALSMNKPFDQVLEKTIFPALGLKHSYVNVPKTQMQNYDFGYNQENQPIRVNPGP
C-212WP_031975357.1_1297AbBCP	LFGKVVALSMNKPFDQVLEKTIFPALGLKHSYVNVPKTQMQNYDFGYNQENQPIRVNPGP
DC-212WP_031975357.1_501AbBCP	LFGKVVALSMNKPFDQVLEKTIFPALGLKHSYVNVPKTQMQNYDFGYNQENQPIRVNPGP
C-182WP_057691114.1_1055AbBOC	LFGKVVALSMNKPFDQVLEKTIFPALGLKHSYVNVPKTQMQNYAFGYNQENQPIRVNPGP
C-182WP_057691114.1_1055AbBCP	LFGKVVALSMNKPFDQVLEKTIFPALGLKHSYVNVPKTQMQNYAFGYNQENQPIRVNPGP
DC-81WP_059262723.1_1208AbBOC	LFGKVVALSMNKPFDQVLEKTIFPALGLKHSYVNVPKTQMQNYAFGYNQENQPIRVNPGP
DC-81WP_059262723.1_2841AbBOC	LFGKVVALSMNKPFDQVLEKTIFPALGLKHSYVNVPKTQMQNYAFGYNQENQPIRVNPGP
DC-81WP_059262723.1_1208AbBCP	LFGKVVALSMNKPFDQVLEKTIFPALGLKHSYVNVPKTQMQNYAFGYNQENQPIRVNPGP
DC-81WP_059262723.1_2841AbBCP	LFGKVVALSMNKPFDQVLEKTIFPALGLKHSYVNVPKTQMQNYAFGYNQENQPIRVNPGP
ADC-176WP_001159761.1_11AbOC	LFGKVVALSMNKPFDQVLEKTIFPALGLKHSYVNVPKTQMQNYAFGYNQENQPIRVNPGP
DC-176WP_001159761.1_3466AbOC	LFGKVVALSMNKPFDQVLEKTIFPALGLKHSYVNVPKTQMQNYAFGYNQENQPIRVNPGP
ADC-176WP_001159761.1_93AbBOP	LFGKVVALSMNKPFDQVLEKTIFPALGLKHSYVNVPKTQMQNYAFGYNQENQPIRVNPGP
DC-176WP_001159761.1_247AbBOC	LFGKVVALSMNKPFDQVLEKTIFPALGLKHSYVNVPKTQMQNYAFGYNQENQPIRVNPGP
ADC-176WP_001159761.1_9AbBOC	LFGKVVALSMNKPFDQVLEKTIFPALGLKHSYVNVPKTQMQNYAFGYNQENQPIRVNPGP
C-176WP_001159761.1_2982AbBOC	LFGKVVALSMNKPFDQVLEKTIFPALGLKHSYVNVPKTQMQNYAFGYNQENQPIRVNPGP
ADC-176QLG80706.1_1971AbBOC	LFGKVVALSMNKPFDQVLEKTIFPALGLKHSYVNVPKTQMQNYAFGYNQENQPIRVNPGP
C-176WP_001159761.1_2851AbBOC	LFGKVVALSMNKPFDQVLEKTIFPALGLKHSYVNVPKTQMQNYAFGYNQENQPIRVNPGP
C-176WP_001159761.1_1220AbBOC	LFGKVVALSMNKPFDQVLEKTIFPALGLKHSYVNVPKTQMQNYAFGYNQENQPIRVNPGP
DC-176WP_001159761.1_386AbBOC	LFGKVVALSMNKPFDQVLEKTIFPALGLKHSYVNVPKTQMQNYAFGYNQENQPIRVNPGP
ADC-176WP_001159761.1_11AbBOC	LFGKVVALSMNKPFDQVLEKTIFPALGLKHSYVNVPKTQMQNYAFGYNQENQPIRVNPGP
DC-176WP_001159761.1_247AbBCP	LFGKVVALSMNKPFDQVLEKTIFPALGLKHSYVNVPKTQMQNYAFGYNQENQPIRVNPGP
ADC-176WP_001159761.1_9AbBCP	LFGKVVALSMNKPFDQVLEKTIFPALGLKHSYVNVPKTQMQNYAFGYNQENQPIRVNPGP
C-176WP_001159761.1_2982AbBCP	LFGKVVALSMNKPFDQVLEKTIFPALGLKHSYVNVPKTQMQNYAFGYNQENQPIRVNPGP
ADC-176QLG80706.1_1971AbBCP	LFGKVVALSMNKPFDQVLEKTIFPALGLKHSYVNVPKTQMQNYAFGYNQENQPIRVNPGP
C-176WP_001159761.1_2851AbBCP	LFGKVVALSMNKPFDQVLEKTIFPALGLKHSYVNVPKTQMQNYAFGYNQENQPIRVNPGP
C-176WP_001159761.1_1220AbBCP	LFGKVVALSMNKPFDQVLEKTIFPALGLKHSYVNVPKTQMQNYAFGYNQENQPIRVNPGP
DC-176WP_001159761.1_386AbBCP	LFGKVVALSMNKPFDQVLEKTIFPALGLKHSYVNVPKTQMQNYAFGYNQENQPIRVNPGP
ADC-176WP_001159761.1_11AbBCP	LFGKVVALSMNKPFDQVLEKTIFPALGLKHSYVNVPKTQMQNYAFGYNQENQPIRVNPGP
ADC-199WP_114166683.1_169AbOC	LFGKVVALSMNKPFDQVLEKTIFPALGLKHSYVNVPKTQMQNYAFGYNQENQPIRVNPGP
DC-199WP_114166683.1_267AbBOC	LFGKVVALSMNKPFDQVLEKTIFPALGLKHSYVNVPKTQMQNYAFGYNQENQPIRVNPGP
DC-199WP_114166683.1_267AbBCP	LFGKVVALSMNKPFDQVLEKTIFPALGLKHSYVNVPKTQMQNYAFGYNQENQPIRVNPGP
acc	

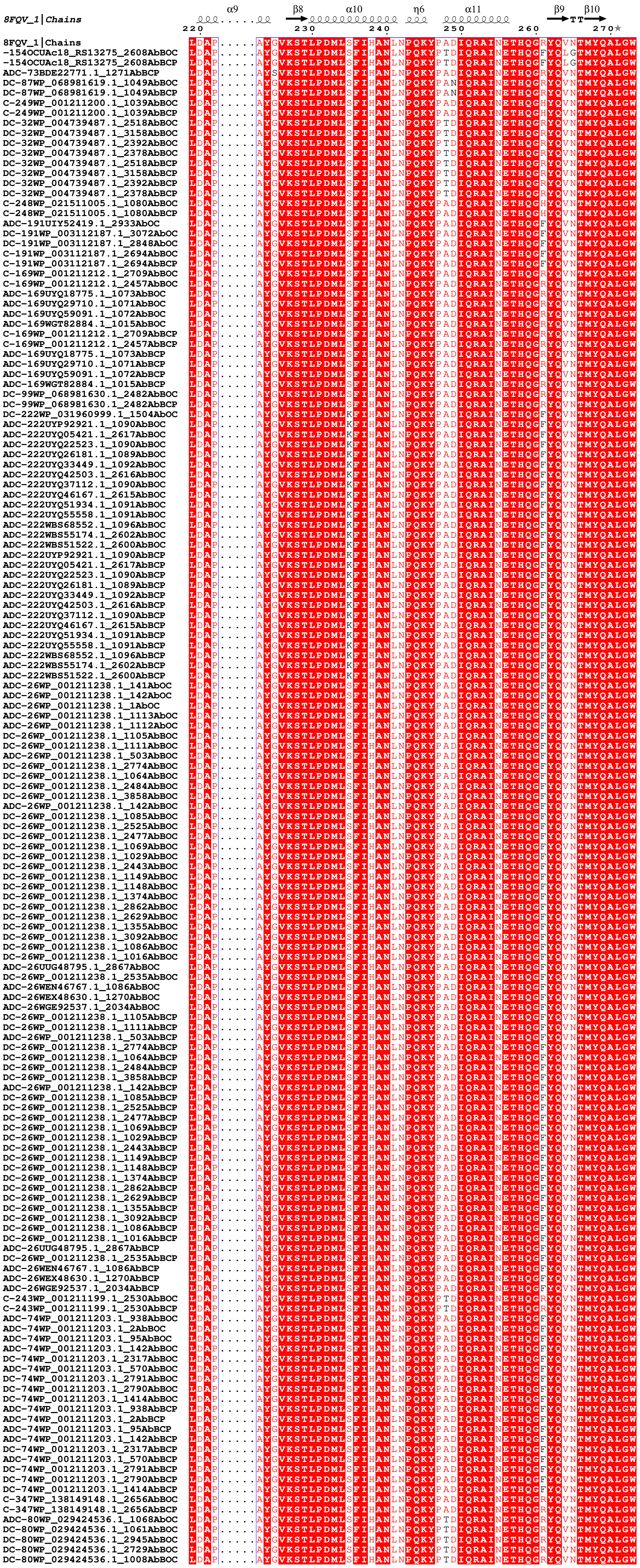
8FQV_1|Chains
α9
β8
α10
η6
α11
β9
β10
220
230
240
250
260
270

```
DC-80WP_029424536.1_1061AbBCP   LDAP.....AYGVKSTLPDMLSFIHANLNPQKYPTDIQRAINETHQGRYQVNTMYQALGW
DC-80WP_029424536.1_2945AbBCP   LDAP.....AYGVKSTLPDMLSFIHANLNPQKYPTDIQRAINETHQGRYQVNTMYQALGW
DC-80WP_029424536.1_2729AbBCP   LDAP.....AYGVKSTLPDMLSFIHANLNPQKYPTDIQRAINETHQGRYQVNTMYQALGW
DC-80WP_029424536.1_1008AbBCP   LDAP.....AYGVKSTLPDMLSFIHANLNPQKYPTDIQRAINETHQGRYQVNTMYQALGW
DC-156WP_024436624.1_2681AbOC   LDAP.....AYGVKSTLPDMLSFIYANLNPQKYPADIQRAINETHQGRYQVNTMYQALGW
C-156WP_024436624.1_2375AbBOC   LDAP.....AYGVKSTLPDMLSFIYANLNPQKYPADIQRAINETHQGRYQVNTMYQALGW
C-156WP_024436624.1_1038AbBOC   LDAP.....AYGVKSTLPDMLSFIYANLNPQKYPADIQRAINETHQGRYQVNTMYQALGW
C-156WP_024436624.1_1041AbBOC   LDAP.....AYGVKSTLPDMLSFIYANLNPQKYPADIQRAINETHQGRYQVNTMYQALGW
C-156WP_024436624.1_1124AbBOC   LDAP.....AYGVKSTLPDMLSFIYANLNPQKYPADIQRAINETHQGRYQVNTMYQALGW
C-156WP_024436624.1_1126AbBOC   LDAP.....AYGVKSTLPDMLSFIYANLNPQKYPADIQRAINETHQGRYQVNTMYQALGW
C-156WP_024436624.1_2375AbBCP   LDAP.....AYGVKSTLPDMLSFIYANLNPQKYPADIQRAINETHQGRYQVNTMYQALGW
C-156WP_024436624.1_1038AbBCP   LDAP.....AYGVKSTLPDMLSFIYANLNPQKYPADIQRAINETHQGRYQVNTMYQALGW
C-156WP_024436624.1_1041AbBCP   LDAP.....AYGVKSTLPDMLSFIYANLNPQKYPADIQRAINETHQGRYQVNTMYQALGW
C-156WP_024436624.1_1124AbBCP   LDAP.....AYGVKSTLPDMLSFIYANLNPQKYPADIQRAINETHQGRYQVNTMYQALGW
C-156WP_024436624.1_1126AbBCP   LDAP.....AYGVKSTLPDMLSFIYANLNPQKYPADIQRAINETHQGRYQVNTMYQALGW
C-165WP_001211239.1_1734AbBOC   LDAP.....AYGVKSTLPDMLSFIHANLNPQKYPTDIQRAINETHQGRYQVNTMYQALGW
C-165WP_001211239.1_1032AbBOC   LDAP.....AYGVKSTLPDMLSFIHANLNPQKYPTDIQRAINETHQGRYQVNTMYQALGW
C-165WP_001211239.1_1734AbBCP   LDAP.....AYGVKSTLPDMLSFIHANLNPQKYPTDIQRAINETHQGRYQVNTMYQALGW
C-165WP_001211239.1_1032AbBCP   LDAP.....AYGVKSTLPDMLSFIHANLNPQKYPTDIQRAINETHQGRYQVNTMYQALGW
C-263WP_109433738.1_2026AbBOC   LDAP.....AYSVKSTLPDMLSFIHANLNPQKYPADIQRAINETHQGRYQVNTMYQALGW
DC-263WP_109433738.1_954AbBOC   LDAP.....AYSVKSTLPDMLSFIHANLNPQKYPADIQRAINETHQGRYQVNTMYQALGW
C-263WP_109433738.1_1022AbBOC   LDAP.....AYSVKSTLPDMLSFIHANLNPQKYPADIQRAINETHQGRYQVNTMYQALGW
C-263WP_109433738.1_3219AbBOC   LDAP.....AYSVKSTLPDMLSFIHANLNPQKYPADIQRAINETHQGRYQVNTMYQALGW
C-263WP_109433738.1_2026AbBCP   LDAP.....AYSVKSTLPDMLSFIHANLNPQKYPADIQRAINETHQGRYQVNTMYQALGW
DC-263WP_109433738.1_954AbBCP   LDAP.....AYSVKSTLPDMLSFIHANLNPQKYPADIQRAINETHQGRYQVNTMYQALGW
C-263WP_109433738.1_1022AbBCP   LDAP.....AYSVKSTLPDMLSFIHANLNPQKYPADIQRAINETHQGRYQVNTMYQALGW
C-263WP_109433738.1_3219AbBCP   LDAP.....AYSVKSTLPDMLSFIHANLNPQKYPADIQRAINETHQGRYQVNTMYQALGW
DC-79WP_001159760.1_2471AbBOC   LDAP.....AYGVKSTLPDMLSFIHANLNPQKYPADIQRAINETHQGFYQVNTMYQALGW
ADC-79WP_001159760.1_670AbBOC   LDAP.....AYGVKSTLPDMLSFIHANLNPQKYPADIQRAINETHQGFYQVNTMYQALGW
DC-79WP_001159760.1_2535AbBOC   LDAP.....AYGVKSTLPDMLSFIHANLNPQKYPADIQRAINETHQGFYQVNTMYQALGW
DC-79WP_001159760.1_1080AbBOC   LDAP.....AYGVKSTLPDMLSFIHANLNPQKYPADIQRAINETHQGFYQVNTMYQALGW
DC-79WP_001159760.1_2471AbBCP   LDAP.....AYGVKSTLPDMLSFIHANLNPQKYPADIQRAINETHQGFYQVNTMYQALGW
ADC-79WP_001159760.1_670AbBCP   LDAP.....AYGVKSTLPDMLSFIHANLNPQKYPADIQRAINETHQGFYQVNTMYQALGW
DC-79WP_001159760.1_2535AbBCP   LDAP.....AYGVKSTLPDMLSFIHANLNPQKYPADIQRAINETHQGFYQVNTMYQALGW
DC-79WP_001159760.1_1080AbBCP   LDAP.....AYGVKSTLPDMLSFIHANLNPQKYPADIQRAINETHQGFYQVNTMYQALGW
ADC-6WP_017725267.1_1459AbBOC   LDAP.....AYGVKSTLPDMLSFIHANLNPQKYPADIQRAINETHQGRYQVNTMYQALGW
ADC-6WP_017725267.1_1678AbBOC   LDAP.....AYGVKSTLPDMLSFIHANLNPQKYPADIQRAINETHQGRYQVNTMYQALGW
ADC-6WP_017725267.1_2561AbBOC   LDAP.....AYGVKSTLPDMLSFIHANLNPQKYPADIQRAINETHQGRYQVNTMYQALGW
ADC-6WP_017725267.1_1459AbBCP   LDAP.....AYGVKSTLPDMLSFIHANLNPQKYPADIQRAINETHQGRYQVNTMYQALGW
ADC-6WP_017725267.1_1678AbBCP   LDAP.....AYGVKSTLPDMLSFIHANLNPQKYPADIQRAINETHQGRYQVNTMYQALGW
ADC-6WP_017725267.1_2561AbBCP   LDAP.....AYGVKSTLPDMLSFIHANLNPQKYPADIQRAINETHQGRYQVNTMYQALGW
ADC-1WP_004714775.1_3726AbBOC   LGAP.....AYGVKSTLPDMLSFIHANLNPQKYPADIQRAINETHQGRYQVNTMYQALGW
ADC-1WP_004714775.1_3726AbBCP   LGAP.....AYGVKSTLPDMLSFIHANLNPQKYPADIQRAINETHQGRYQVNTMYQALGW
C-181WP_046693238.1_1083AbBOC   LGAP.....AYGVKSTLPDMLSFIHANLTPQKYPTDIQRAINETHQGFYQVGTMYQALGW
C-181WP_046693238.1_1083AbBCP   LGAP.....AYGVKSTLPDMLSFIHANLTPQKYPTDIQRAINETHQGFYQVGTMYQALGW
C-238WP_002058891.1_2458AbBOC   LDAP.....AYGVKSTLPDMLSFIHANLNPQKYPADIQRAINETHQGRYQVNTMYQALGW
C-238WP_002058891.1_2458AbBCP   LDAP.....AYGVKSTLPDMLSFIHANLNPQKYPADIQRAINETHQGRYQVNTMYQALGW
C-186WP_072292271.1_1675AbBOC   LDAP.....AYGVKSTLPDMLSFIHANLNPQKYPADIQRAINETHQGFYQVNTMYQALGW
C-186WP_072292271.1_1675AbBCP   LDAP.....AYGVKSTLPDMLSFIHANLNPQKYPADIQRAINETHQGFYQVNTMYQALGW
DC-184WP_059247009.1_1188AbOC   LDAP.....AYGVKSTLPDMLSFIHANLNPQKYPADIQRAINETHQGFYQVNTMYQALGW
ADC-328WP_069370341.1_666AbOC   LDAP.....AYGVKSTLPDMLSFIHANLNPQKYPTDIQRAINETHQGRYQVNTMYQALGW
DC-56WP_031973850.1_1911AbBOC   LDAP.....AYGVKSTLPDMLSFIHANLNPQKYPADIQRAINETHQGRYQVNTMYQALGW
DC-56WP_031973850.1_2693AbBOC   LDAP.....AYGVKSTLPDMLSFIHANLNPQKYPADIQRAINETHQGRYQVNTMYQALGW
DC-56WP_031973850.1_1911AbBCP   LDAP.....AYGVKSTLPDMLSFIHANLNPQKYPADIQRAINETHQGRYQVNTMYQALGW
DC-56WP_031973850.1_2693AbBCP   LDAP.....AYGVKSTLPDMLSFIHANLNPQKYPADIQRAINETHQGRYQVNTMYQALGW
DC-117WP_002157727.1_394AbBOC   LDAP.....AYGVKSTLPDMLSFIHANLNPQKYPADIQRAINETHQGRYQVNTMYQALGW
DC-117WP_002157727.1_394AbBCP   LDAP.....AYGVKSTLPDMLSFIHANLNPQKYPADIQRAINETHQGRYQVNTMYQALGW
ADC-25WP_001211217.1_2432AbOC   LDAP.....AYGVKSTLPDMLSFIHANLNPQKYPADIQRAINETHQGRYQVNTMYQALGW
ADC-25WP_001211217.1_1025AbOC   LDAP.....AYGVKSTLPDMLSFIHANLNPQKYPADIQRAINETHQGRYQVNTMYQALGW
ADC-25WP_001211217.1_3391AbOC   LDAP.....AYGVKSTLPDMLSFIHANLNPQKYPADIQRAINETHQGRYQVNTMYQALGW
ADC-25WP_001211217.1_1042AbOC   LDAP.....AYGVKSTLPDMLSFIHANLNPQKYPADIQRAINETHQGRYQVNTMYQALGW
ADC-25WP_001211217.1_1185AbOC   LDAP.....AYGVKSTLPDMLSFIHANLNPQKYPADIQRAINETHQGRYQVNTMYQALGW
ADC-25WP_001211217.1_768AbOC    LDAP.....AYGVKSTLPDMLSFIHANLNPQKYPADIQRAINETHQGRYQVNTMYQALGW
ADC-25WP_001211217.1_2605AbOC   LDAP.....AYGVKSTLPDMLSFIHANLNPQKYPADIQRAINETHQGRYQVNTMYQALGW
DC-25WP_001211217.1_1117AbBOC   LDAP.....AYGVKSTLPDMLSFIHANLNPQKYPADIQRAINETHQGRYQVNTMYQALGW
DC-25WP_001211217.1_1090AbBOC   LDAP.....AYGVKSTLPDMLSFIHANLNPQKYPADIQRAINETHQGRYQVNTMYQALGW
DC-25WP_001211217.1_1089AbBOC   LDAP.....AYGVKSTLPDMLSFIHANLNPQKYPADIQRAINETHQGRYQVNTMYQALGW
DC-25WP_001211217.1_1091AbBOC   LDAP.....AYGVKSTLPDMLSFIHANLNPQKYPADIQRAINETHQGRYQVNTMYQALGW
DC-25WP_001211217.1_1604AbBOC   LDAP.....AYGVKSTLPDMLSFIHANLNPQKYPADIQRAINETHQGRYQVNTMYQALGW
DC-25WP_001211217.1_1040AbBOC   LDAP.....AYGVKSTLPDMLSFIHANLNPQKYPADIQRAINETHQGRYQVNTMYQALGW
ADC-25UYQ12932.1_2634AbBOC      LDAP.....AYGVKSTLPDMLSFIHANLNPQKYPADIQRAINETHQGRYQVNTMYQALGW
ADC-25UYQ09083.1_2628AbBOC      LDAP.....AYGVKSTLPDMLSFIHANLNPQKYPADIQRAINETHQGRYQVNTMYQALGW
ADC-25UYQ40822.1_1120AbBOC      LDAP.....AYGVKSTLPDMLSFIHANLNPQKYPADIQRAINETHQGRYQVNTMYQALGW
DC-25WP_001211217.1_1117AbBCP   LDAP.....AYGVKSTLPDMLSFIHANLNPQKYPADIQRAINETHQGRYQVNTMYQALGW
DC-25WP_001211217.1_1090AbBCP   LDAP.....AYGVKSTLPDMLSFIHANLNPQKYPADIQRAINETHQGRYQVNTMYQALGW
DC-25WP_001211217.1_1089AbBCP   LDAP.....AYGVKSTLPDMLSFIHANLNPQKYPADIQRAINETHQGRYQVNTMYQALGW
DC-25WP_001211217.1_1091AbBCP   LDAP.....AYGVKSTLPDMLSFIHANLNPQKYPADIQRAINETHQGRYQVNTMYQALGW
DC-25WP_001211217.1_1604AbBCP   LDAP.....AYGVKSTLPDMLSFIHANLNPQKYPADIQRAINETHQGRYQVNTMYQALGW
DC-25WP_001211217.1_1040AbBCP   LDAP.....AYGVKSTLPDMLSFIHANLNPQKYPADIQRAINETHQGRYQVNTMYQALGW
ADC-25UYQ12932.1_2634AbBCP      LDAP.....AYGVKSTLPDMLSFIHANLNPQKYPADIQRAINETHQGRYQVNTMYQALGW
ADC-25UYQ09083.1_2628AbBCP      LDAP.....AYGVKSTLPDMLSFIHANLNPQKYPADIQRAINETHQGRYQVNTMYQALGW
ADC-25UYQ40822.1_1120AbBCP      LDAP.....AYGVKSTLPDMLSFIHANLNPQKYPADIQRAINETHQGRYQVNTMYQALGW
C-268WP_105922788.1_2839AbBOC   LDAP.....AYSVKSTLPDMLSFIHANLNPQKYPADIQRAINETHQGRYQVNTMYQALGW
ADC-268WDC47378.1_2492AbBOC     LDAP.....AYSVKSTLPDMLSFIHANLNPQKYPADIQRAINETHQGRYQVNTMYQALGW
C-268WP_105922788.1_2839AbBCP   LDAP.....AYSVKSTLPDMLSFIHANLNPQKYPADIQRAINETHQGRYQVNTMYQALGW
ADC-268WDC47378.1_2492AbBCP     LDAP.....AYSVKSTLPDMLSFIHANLNPQKYPADIQRAINETHQGRYQVNTMYQALGW
DC-155WP_005128228.1_2591AbOC   LDAP.....AYGVKSTLPDMLSFIHANLNPQKYSADIQRAINETHQGRYQINTMYQALGW
C-155WP_005128228.1_2549AbBOC   LDAP.....AYGVKSTLPDMLSFIHANLNPQKYSADIQRAINETHQGRYQINTMYQALGW
C-155WP_005128228.1_2480AbBOC   LDAP.....AYGVKSTLPDMLSFIHANLNPQKYSADIQRAINETHQGRYQINTMYQALGW
C-155WP_005128228.1_2455AbBOC   LDAP.....AYGVKSTLPDMLSFIHANLNPQKYSADIQRAINETHQGRYQINTMYQALGW
C-155WP_005128228.1_2549AbBCP   LDAP.....AYGVKSTLPDMLSFIHANLNPQKYSADIQRAINETHQGRYQINTMYQALGW
C-155WP_005128228.1_2480AbBCP   LDAP.....AYGVKSTLPDMLSFIHANLNPQKYSADIQRAINETHQGRYQINTMYQALGW
C-155WP_005128228.1_2455AbBCP   LDAP.....AYGVKSTLPDMLSFIHANLNPQKYSADIQRAINETHQGRYQINTMYQALGW
ADC-76WP_001211237.1_2862AbOC   LDAP.....AYGVKSTLPDMLSFIHANLNPQKYPTDIQRAINETHQGRYQVNTMYQALGW
ADC-76WP_001211237.1_3431AbOC   LDAP.....AYGVKSTLPDMLSFIHANLNPQKYPTDIQRAINETHQGRYQVNTMYQALGW
ADC-76WP_001211237.1_2696AbOC   LDAP.....AYGVKSTLPDMLSFIHANLNPQKYPTDIQRAINETHQGRYQVNTMYQALGW
ADC-76WP_001211237.1_2890AbOC   LDAP.....AYGVKSTLPDMLSFIHANLNPQKYPTDIQRAINETHQGRYQVNTMYQALGW
DC-76WP_001211237.1_2723AbBOC   LDAP.....AYGVKSTLPDMLSFIHANLNPQKYPTDIQRAINETHQGRYQVNTMYQALGW
DC-76WP_001211237.1_1033AbBOC   LDAP.....AYGVKSTLPDMLSFIHANLNPQKYPTDIQRAINETHQGRYQVNTMYQALGW
ADC-76WP_001211237.1_526AbBOC   LDAP.....AYGVKSTLPDMLSFIHANLNPQKYPTDIQRAINETHQGRYQVNTMYQALGW
DC-76WP_001211237.1_1046AbBOC   LDAP.....AYGVKSTLPDMLSFIHANLNPQKYPTDIQRAINETHQGRYQVNTMYQALGW
DC-76WP_001211237.1_1040AbBOC   LDAP.....AYGVKSTLPDMLSFIHANLNPQKYPTDIQRAINETHQGRYQVNTMYQALGW
DC-76WP_001211237.1_3682AbBOC   LDAP.....AYGVKSTLPDMLSFIHANLNPQKYPTDIQRAINETHQGRYQVNTMYQALGW
DC-76WP_001211237.1_2579AbBOC   LDAP.....AYGVKSTLPDMLSFIHANLNPQKYPTDIQRAINETHQGRYQVNTMYQALGW
DC-76WP_001211237.1_2723AbBCP   LDAP.....AYGVKSTLPDMLSFIHANLNPQKYPTDIQRAINETHQGRYQVNTMYQALGW
DC-76WP_001211237.1_1033AbBCP   LDAP.....AYGVKSTLPDMLSFIHANLNPQKYPTDIQRAINETHQGRYQVNTMYQALGW
ADC-76WP_001211237.1_526AbBCP   LDAP.....AYGVKSTLPDMLSFIHANLNPQKYPTDIQRAINETHQGRYQVNTMYQALGW
DC-76WP_001211237.1_1046AbBCP   LDAP.....AYGVKSTLPDMLSFIHANLNPQKYPTDIQRAINETHQGRYQVNTMYQALGW
DC-76WP_001211237.1_1040AbBCP   LDAP.....AYGVKSTLPDMLSFIHANLNPQKYPTDIQRAINETHQGRYQVNTMYQALGW
DC-76WP_001211237.1_3682AbBCP   LDAP.....AYGVKSTLPDMLSFIHANLNPQKYPTDIQRAINETHQGRYQVNTMYQALGW
DC-76WP_001211237.1_2579AbBCP   LDAP.....AYGVKSTLPDMLSFIHANLNPQKYPTDIQRAINETHQGRYQVNTMYQALGW
DC-291WP_001211201.1_1060AbOC   LDAP.....AYGVKSTLPDMLSFIHANLNPQKYPTDIQRAINETHQGRYQVNTMYQALGW
C-291WP_001211201.1_2415AbBOC   LDAP.....AYGVKSTLPDMLSFIHANLNPQKYPTDIQRAINETHQGRYQVNTMYQALGW
C-291WP_001211201.1_2415AbBCP   LDAP.....AYGVKSTLPDMLSFIHANLNPQKYPTDIQRAINETHQGRYQVNTMYQALGW
DC-214WP_060454527.1_118AbBOC   LDAP.....AYSVKSTLPDMLKFIHANLNPQKYPTDIQRAINETHQGRYQVNTMYQALGW
DC-214WP_060454527.1_118AbBCP   LDAP.....AYSVKSTLPDMLKFIHANLNPQKYPTDIQRAINETHQGRYQVNTMYQALGW
C-185WP_001211210.1_2745AbBOC   LDAP.....AYGVKSTLPDMLSFINANLNPQKYPTDIQRAINETHQGRYQVNTMYQALGW
ADC-185WEI07035.1_298AbBOC     LDAP.....AYGVKSTLPDMLSFINANLNPQKYPTDIQRAINETHQGRYQVNTMYQALGW
C-185WP_001211210.1_2745AbBCP   LDAP.....AYGVKSTLPDMLSFINANLNPQKYPTDIQRAINETHQGRYQVNTMYQALGW
ADC-185WEI07035.1_298AbBCP     LDAP.....AYGVKSTLPDMLSFINANLNPQKYPTDIQRAINETHQGRYQVNTMYQALGW
ADC-11WP_001211205.1_1099AbOC   LDAP.....AYGVKSTLPDMLSFIHANLNPQKYPADIQRAINETHQGFYQVNTMYQALGW
ADC-11WP_001211205.1_1035AbOC   LDAP.....AYGVKSTLPDMLSFIHANLNPQKYPADIQRAINETHQGFYQVNTMYQALGW
DC-11WP_001211205.1_2603AbBOC   LDAP.....AYGVKSTLPDMLSFIHANLNPQKYPADIQRAINETHQGFYQVNTMYQALGW
DC-11WP_001211205.1_1138AbBOC   LDAP.....AYGVKSTLPDMLSFIHANLNPQKYPADIQRAINETHQGFYQVNTMYQALGW
DC-11WP_001211205.1_2874AbBOC   LDAP.....AYGVKSTLPDMLSFIHANLNPQKYPADIQRAINETHQGFYQVNTMYQALGW
DC-11WP_001211205.1_2686AbBOC   LDAP.....AYGVKSTLPDMLSFIHANLNPQKYPADIQRAINETHQGFYQVNTMYQALGW
ADC-11WP_001211205.1_130AbBOC   LDAP.....AYGVKSTLPDMLSFIHANLNPQKYPADIQRAINETHQGFYQVNTMYQALGW
DC-11WP_001211205.1_1097AbBOC   LDAP.....AYGVKSTLPDMLSFIHANLNPQKYPADIQRAINETHQGFYQVNTMYQALGW
ADC-11WP_001211205.1_684AbBOC   LDAP.....AYGVKSTLPDMLSFIHANLNPQKYPADIQRAINETHQGFYQVNTMYQALGW
ADC-11WP_001211205.1_682AbBOC   LDAP.....AYGVKSTLPDMLSFIHANLNPQKYPADIQRAINETHQGFYQVNTMYQALGW
DC-11WP_001211205.1_1111AbBOC   LDAP.....AYGVKSTLPDMLSFIHANLNPQKYPADIQRAINETHQGFYQVNTMYQALGW
DC-11WP_001211205.1_1059AbBOC   LDAP.....AYGVKSTLPDMLSFIHANLNPQKYPADIQRAINETHQGFYQVNTMYQALGW
DC-11WP_001211205.1_2754AbBOC   LDAP.....AYGVKSTLPDMLSFIHANLNPQKYPADIQRAINETHQGFYQVNTMYQALGW
DC-11WP_001211205.1_2603AbBCP   LDAP.....AYGVKSTLPDMLSFIHANLNPQKYPADIQRAINETHQGFYQVNTMYQALGW
DC-11WP_001211205.1_1138AbBCP   LDAP.....AYGVKSTLPDMLSFIHANLNPQKYPADIQRAINETHQGFYQVNTMYQALGW
DC-11WP_001211205.1_2874AbBCP   LDAP.....AYGVKSTLPDMLSFIHANLNPQKYPADIQRAINETHQGFYQVNTMYQALGW
DC-11WP_001211205.1_2686AbBCP   LDAP.....AYGVKSTLPDMLSFIHANLNPQKYPADIQRAINETHQGFYQVNTMYQALGW
ADC-11WP_001211205.1_130AbBCP   LDAP.....AYGVKSTLPDMLSFIHANLNPQKYPADIQRAINETHQGFYQVNTMYQALGW
DC-11WP_001211205.1_1097AbBCP   LDAP.....AYGVKSTLPDMLSFIHANLNPQKYPADIQRAINETHQGFYQVNTMYQALGW
ADC-11WP_001211205.1_684AbBCP   LDAP.....AYGVKSTLPDMLSFIHANLNPQKYPADIQRAINETHQGFYQVNTMYQALGW
ADC-11WP_001211205.1_682AbBCP   LDAP.....AYGVKSTLPDMLSFIHANLNPQKYPADIQRAINETHQGFYQVNTMYQALGW
DC-11WP_001211205.1_1111AbBCP   LDAP.....AYGVKSTLPDMLSFIHANLNPQKYPADIQRAINETHQGFYQVNTMYQALGW
DC-11WP_001211205.1_1059AbBCP   LDAP.....AYGVKSTLPDMLSFIHANLNPQKYPADIQRAINETHQGFYQVNTMYQALGW
DC-11WP_001211205.1_2754AbBCP   LDAP.....AYGVKSTLPDMLSFIHANLNPQKYPADIQRAINETHQGFYQVNTMYQALGW
C-269WP_032025626.1_2988AbBOC   LDAP.....AYGVKSTLPDMLSFIYANLNPQKYPADIQRAINETHQGRYQVNTMYQALGW
C-269WP_032025626.1_2988AbBCP   LDAP.....AYGVKSTLPDMLSFIYANLNPQKYPADIQRAINETHQGRYQVNTMYQALGW
C-166WP_101244937.1_1028AbBOC   LDAP.....AYGVKSTLPDMLSFIHANLNPQKYPADIQRAINETHQGHYQVNTMYQALGW
C-166WP_101244937.1_1028AbBCP   LDAP.....AYGVKSTLPDMLSFIHANLNPQKYPADIQRAINETHQGHYQVNTMYQALGW
C-175WP_001211208.1_2561AbBOC   LDAP.....AYGVKSTLPDMLSFIHANLNPQKYPADIQRAINETHQGFYQVNTMYQALGW
C-175WP_001211208.1_2561AbBCP   LDAP.....AYGVKSTLPDMLSFIHANLNPQKYPADIQRAINETHQGFYQVNTMYQALGW
C-154WP_005138362.1_2451AbBOC   LDAP.....AYGVKSTLPDMLSFIHANLNPQKYPTDIQRAINETHQGFYQLGTMYQALGW
DC-154WP_005138362.1_158AbBOC   LDAP.....AYGVKSTLPDMLSFIHANLNPQKYPTDIQRAINETHQGFYQLGTMYQALGW
```

C-154WP_005138362.1_1167AbBOC	LDAP.....AYGVKSTLPDMLSFIHANLNPQKYPTDIQRAINETHQGFYQLGTMYQALGW
C-154WP_005138362.1_2510AbBOC	LDAP.....AYGVKSTLPDMLSFIHANLNPQKYPTDIQRAINETHQGFYQLGTMYQALGW
C-154WP_005138362.1_2513AbBOC	LDAP.....AYGVKSTLPDMLSFIHANLNPQKYPTDIQRAINETHQGFYQLGTMYQALGW
C-154WP_005138362.1_2512AbBOC	LDAP.....AYGVKSTLPDMLSFIHANLNPQKYPTDIQRAINETHQGFYQLGTMYQALGW
C-154WP_005138362.1_2451AbBCP	LDAP.....AYGVKSTLPDMLSFIHANLNPQKYPTDIQRAINETHQGFYQLGTMYQALGW
DC-154WP_005138362.1_158AbBCP	LDAP.....AYGVKSTLPDMLSFIHANLNPQKYPTDIQRAINETHQGFYQLGTMYQALGW
C-154WP_005138362.1_1167AbBCP	LDAP.....AYGVKSTLPDMLSFIHANLNPQKYPTDIQRAINETHQGFYQLGTMYQALGW
C-154WP_005138362.1_2510AbBCP	LDAP.....AYGVKSTLPDMLSFIHANLNPQKYPTDIQRAINETHQGFYQLGTMYQALGW
C-154WP_005138362.1_2513AbBCP	LDAP.....AYGVKSTLPDMLSFIHANLNPQKYPTDIQRAINETHQGFYQLGTMYQALGW
C-154WP_005138362.1_2512AbBCP	LDAP.....AYGVKSTLPDMLSFIHANLNPQKYPTDIQRAINETHQGFYQLGTMYQALGW
C-240WP_001211207.1_1200AbBOC	LDAP.....AYGVKSTLPDMLSFIHANLNPQKYPADIQRAINETHQGFYQVNTMYQALGW
C-240WP_001211207.1_1200AbBCP	LDAP.....AYGVKSTLPDMLSFIHANLNPQKYPADIQRAINETHQGFYQVNTMYQALGW
ADC-75WP_063857817.1_1610AbOC	LDAP.....AYGVKSTLPDMLSFIHANLNPQKYPADIQRAINETHQGFYQVNTMYQALGW
DC-18WP_002118772.1_1702AbBOC	LDAP.....AYGVKSTLPDMLTFINANLNPQKYPKDIQRAISETHQGFYQVGTMYQALGW
DC-18WP_002118772.1_1702AbBCP	LDAP.....AYGVKSTLPDMLTFINANLNPQKYPKDIQRAISETHQGFYQVGTMYQALGW
C-106WP_068981613.1_2650AbBOC	LDAP.....AYGVKSTLPDMLSFIHANLNPQKYPTDIQRAINETHQGRYQVNTMYQALGW
C-106WP_068981613.1_2650AbBCP	LDAP.....AYGVKSTLPDMLSFIHANLNPQKYPTDIQRAINETHQGRYQVNTMYQALGW
DC-50WP_031965243.1_2562AbBOC	LDAP.....AYGVKSTLPDMLSFIHANLNPQKYPADIQRAINETHQGFYQVNTMYQALGW
ADC-50WP_031965243.1_206AbBOC	LDAP.....AYGVKSTLPDMLSFIHANLNPQKYPADIQRAINETHQGFYQVNTMYQALGW
DC-50WP_031965243.1_2751AbBOC	LDAP.....AYGVKSTLPDMLSFIHANLNPQKYPADIQRAINETHQGFYQVNTMYQALGW
DC-50WP_031965243.1_3766AbBOC	LDAP.....AYGVKSTLPDMLSFIHANLNPQKYPADIQRAINETHQGFYQVNTMYQALGW
DC-50WP_031965243.1_2562AbBCP	LDAP.....AYGVKSTLPDMLSFIHANLNPQKYPADIQRAINETHQGFYQVNTMYQALGW
ADC-50WP_031965243.1_206AbBCP	LDAP.....AYGVKSTLPDMLSFIHANLNPQKYPADIQRAINETHQGFYQVNTMYQALGW
DC-50WP_031965243.1_2751AbBCP	LDAP.....AYGVKSTLPDMLSFIHANLNPQKYPADIQRAINETHQGFYQVNTMYQALGW
DC-50WP_031965243.1_3766AbBCP	LDAP.....AYGVKSTLPDMLSFIHANLNPQKYPADIQRAINETHQGFYQVNTMYQALGW
ADC-158WP_001211227.1_990AbOC	LDAP.....AYGVKSTLPDMLSFIHANLNPQKYPTDIQRAINETHQGRYQVNTMYQALGW
DC-158WP_001211227.1_1295AbOC	LDAP.....AYGVKSTLPDMLSFIHANLNPQKYPTDIQRAINETHQGRYQVNTMYQALGW
ADC-158WP_001211227.1_6AbOC	LDAP.....AYGVKSTLPDMLSFIHANLNPQKYPTDIQRAINETHQGRYQVNTMYQALGW
DC-158WP_001211227.1_991AbBOC	LDAP.....AYGVKSTLPDMLSFIHANLNPQKYPTDIQRAINETHQGRYQVNTMYQALGW
DC-158WP_001211227.1_207AbBOC	LDAP.....AYGVKSTLPDMLSFIHANLNPQKYPTDIQRAINETHQGRYQVNTMYQALGW
C-158WP_001211227.1_2735AbBOC	LDAP.....AYGVKSTLPDMLSFIHANLNPQKYPTDIQRAINETHQGRYQVNTMYQALGW
DC-158WP_001211227.1_996AbBOC	LDAP.....AYGVKSTLPDMLSFIHANLNPQKYPTDIQRAINETHQGRYQVNTMYQALGW
DC-158WP_001211227.1_796AbBOC	LDAP.....AYGVKSTLPDMLSFIHANLNPQKYPTDIQRAINETHQGRYQVNTMYQALGW
C-158WP_001211227.1_2822AbBOC	LDAP.....AYGVKSTLPDMLSFIHANLNPQKYPTDIQRAINETHQGRYQVNTMYQALGW
C-158WP_001211227.1_1339AbBOC	LDAP.....AYGVKSTLPDMLSFIHANLNPQKYPTDIQRAINETHQGRYQVNTMYQALGW
ADC-158WGQ04828.1_2712AbBOC	LDAP.....AYGVKSTLPDMLSFIHANLNPQKYPTDIQRAINETHQGRYQVNTMYQALGW
DC-158WP_001211227.1_991AbBCP	LDAP.....AYGVKSTLPDMLSFIHANLNPQKYPTDIQRAINETHQGRYQVNTMYQALGW
DC-158WP_001211227.1_207AbBCP	LDAP.....AYGVKSTLPDMLSFIHANLNPQKYPTDIQRAINETHQGRYQVNTMYQALGW
C-158WP_001211227.1_2735AbBCP	LDAP.....AYGVKSTLPDMLSFIHANLNPQKYPTDIQRAINETHQGRYQVNTMYQALGW
DC-158WP_001211227.1_996AbBCP	LDAP.....AYGVKSTLPDMLSFIHANLNPQKYPTDIQRAINETHQGRYQVNTMYQALGW
DC-158WP_001211227.1_796AbBCP	LDAP.....AYGVKSTLPDMLSFIHANLNPQKYPTDIQRAINETHQGRYQVNTMYQALGW
C-158WP_001211227.1_2822AbBCP	LDAP.....AYGVKSTLPDMLSFIHANLNPQKYPTDIQRAINETHQGRYQVNTMYQALGW
C-158WP_001211227.1_1339AbBCP	LDAP.....AYGVKSTLPDMLSFIHANLNPQKYPTDIQRAINETHQGRYQVNTMYQALGW
ADC-158WGQ04828.1_2712AbBCP	LDAP.....AYGVKSTLPDMLSFIHANLNPQKYPTDIQRAINETHQGRYQVNTMYQALGW
ADC-31WP_001211223.1_2986AbOC	LDAP.....AYGVKSTLPDMLSFIHANLNPQKYPADIQRAINETHQGRYQVNTMYQALGW
DC-31WP_001211223.1_2698AbBOC	LDAP.....AYGVKSTLPDMLSFIHANLNPQKYPADIQRAINETHQGRYQVNTMYQALGW
DC-31WP_001211223.1_2698AbBCP	LDAP.....AYGVKSTLPDMLSFIHANLNPQKYPADIQRAINETHQGRYQVNTMYQALGW
ADC-30WP_001211218.1_1102AbOC	LDAP.....AYGVKSTLPDMLSFIHANLNPQKYPADIQRAINETHQGRYQVNTMYQALGW
ADC-30WP_001211218.1_2565AbOC	LDAP.....AYGVKSTLPDMLSFIHANLNPQKYPADIQRAINETHQGRYQVNTMYQALGW
ADC-30WP_001211218.1_1021AbOC	LDAP.....AYGVKSTLPDMLSFIHANLNPQKYPADIQRAINETHQGRYQVNTMYQALGW
ADC-30WP_001211218.1_1088AbOC	LDAP.....AYGVKSTLPDMLSFIHANLNPQKYPADIQRAINETHQGRYQVNTMYQALGW
ADC-30WP_001211218.1_1135AbOC	LDAP.....AYGVKSTLPDMLSFIHANLNPQKYPADIQRAINETHQGRYQVNTMYQALGW
ADC-30WP_001211218.1_43AbOC	LDAP.....AYGVKSTLPDMLSFIHANLNPQKYPADIQRAINETHQGRYQVNTMYQALGW
ADC-30WP_001211218.1_1041AbOC	LDAP.....AYGVKSTLPDMLSFIHANLNPQKYPADIQRAINETHQGRYQVNTMYQALGW
ADC-30WP_001211218.1_1032AbOC	LDAP.....AYGVKSTLPDMLSFIHANLNPQKYPADIQRAINETHQGRYQVNTMYQALGW
ADC-30WP_001211218.1_1047AbOC	LDAP.....AYGVKSTLPDMLSFIHANLNPQKYPADIQRAINETHQGRYQVNTMYQALGW
ADC-30WP_001211218.1_1040AbOC	LDAP.....AYGVKSTLPDMLSFIHANLNPQKYPADIQRAINETHQGRYQVNTMYQALGW
ADC-30WP_001211218.1_3388AbOC	LDAP.....AYGVKSTLPDMLSFIHANLNPQKYPADIQRAINETHQGRYQVNTMYQALGW
ADC-30WP_001211218.1_650AbOC	LDAP.....AYGVKSTLPDMLSFIHANLNPQKYPADIQRAINETHQGRYQVNTMYQALGW
ADC-30WP_001211218.1_2855AbOC	LDAP.....AYGVKSTLPDMLSFIHANLNPQKYPADIQRAINETHQGRYQVNTMYQALGW
ADC-30WP_001211218.1_1034AbOC	LDAP.....AYGVKSTLPDMLSFIHANLNPQKYPADIQRAINETHQGRYQVNTMYQALGW
ADC-30WP_001211218.1_79AbOC	LDAP.....AYGVKSTLPDMLSFIHANLNPQKYPADIQRAINETHQGRYQVNTMYQALGW
ADC-30WP_001211218.1_3509AbOC	LDAP.....AYGVKSTLPDMLSFIHANLNPQKYPADIQRAINETHQGRYQVNTMYQALGW
ADC-30WP_001211218.1_2399AbOC	LDAP.....AYGVKSTLPDMLSFIHANLNPQKYPADIQRAINETHQGRYQVNTMYQALGW
ADC-30WP_001211218.1_2415AbOC	LDAP.....AYGVKSTLPDMLSFIHANLNPQKYPADIQRAINETHQGRYQVNTMYQALGW
ADC-30WP_001211218.1_3032AbOC	LDAP.....AYGVKSTLPDMLSFIHANLNPQKYPADIQRAINETHQGRYQVNTMYQALGW
ADC-30WP_001211218.1_832AbOC	LDAP.....AYGVKSTLPDMLSFIHANLNPQKYPADIQRAINETHQGRYQVNTMYQALGW
ADC-30WP_001211218.1_1428AbOC	LDAP.....AYGVKSTLPDMLSFIHANLNPQKYPADIQRAINETHQGRYQVNTMYQALGW
ADC-30WP_001211218.1_2340AbOC	LDAP.....AYGVKSTLPDMLSFIHANLNPQKYPADIQRAINETHQGRYQVNTMYQALGW
ADC-30WP_001211218.1_2976AbOC	LDAP.....AYGVKSTLPDMLSFIHANLNPQKYPADIQRAINETHQGRYQVNTMYQALGW
ADC-30WP_001211218.1_2305AbOC	LDAP.....AYGVKSTLPDMLSFIHANLNPQKYPADIQRAINETHQGRYQVNTMYQALGW
ADC-30WP_001211218.1_1110AbOC	LDAP.....AYGVKSTLPDMLSFIHANLNPQKYPADIQRAINETHQGRYQVNTMYQALGW
ADC-30WP_001211218.1_2797AbOC	LDAP.....AYGVKSTLPDMLSFIHANLNPQKYPADIQRAINETHQGRYQVNTMYQALGW
ADC-30WP_001211218.1_1023AbOC	LDAP.....AYGVKSTLPDMLSFIHANLNPQKYPADIQRAINETHQGRYQVNTMYQALGW
ADC-30WP_001211218.1_1022AbOC	LDAP.....AYGVKSTLPDMLSFIHANLNPQKYPADIQRAINETHQGRYQVNTMYQALGW
ADC-30WP_001211218.1_2458AbOC	LDAP.....AYGVKSTLPDMLSFIHANLNPQKYPADIQRAINETHQGRYQVNTMYQALGW
ADC-30WP_001211218.1_2513AbOC	LDAP.....AYGVKSTLPDMLSFIHANLNPQKYPADIQRAINETHQGRYQVNTMYQALGW
ADC-30WP_001211218.1_2482AbOC	LDAP.....AYGVKSTLPDMLSFIHANLNPQKYPADIQRAINETHQGRYQVNTMYQALGW
DC-30WP_001211218.1_2692AbBOC	LDAP.....AYGVKSTLPDMLSFIHANLNPQKYPADIQRAINETHQGRYQVNTMYQALGW
DC-30WP_001211218.1_2549AbBOC	LDAP.....AYGVKSTLPDMLSFIHANLNPQKYPADIQRAINETHQGRYQVNTMYQALGW
DC-30WP_001211218.1_1026AbBOC	LDAP.....AYGVKSTLPDMLSFIHANLNPQKYPADIQRAINETHQGRYQVNTMYQALGW
ADC-30WP_001211218.1_373AbBOC	LDAP.....AYGVKSTLPDMLSFIHANLNPQKYPADIQRAINETHQGRYQVNTMYQALGW
DC-30WP_001211218.1_2465AbBOC	LDAP.....AYGVKSTLPDMLSFIHANLNPQKYPADIQRAINETHQGRYQVNTMYQALGW
DC-30WP_001211218.1_1027AbBOC	LDAP.....AYGVKSTLPDMLSFIHANLNPQKYPADIQRAINETHQGRYQVNTMYQALGW
DC-30WP_001211218.1_2650AbBOC	LDAP.....AYGVKSTLPDMLSFIHANLNPQKYPADIQRAINETHQGRYQVNTMYQALGW
DC-30WP_001211218.1_2674AbBOC	LDAP.....AYGVKSTLPDMLSFIHANLNPQKYPADIQRAINETHQGRYQVNTMYQALGW
DC-30WP_001211218.1_2689AbBOC	LDAP.....AYGVKSTLPDMLSFIHANLNPQKYPADIQRAINETHQGRYQVNTMYQALGW
DC-30WP_001211218.1_1105AbBOC	LDAP.....AYGVKSTLPDMLSFIHANLNPQKYPADIQRAINETHQGRYQVNTMYQALGW
DC-30WP_001211218.1_2670AbBOC	LDAP.....AYGVKSTLPDMLSFIHANLNPQKYPADIQRAINETHQGRYQVNTMYQALGW
DC-30WP_001211218.1_1019AbBOC	LDAP.....AYGVKSTLPDMLSFIHANLNPQKYPADIQRAINETHQGRYQVNTMYQALGW
DC-30WP_001211218.1_1039AbBOC	LDAP.....AYGVKSTLPDMLSFIHANLNPQKYPADIQRAINETHQGRYQVNTMYQALGW
DC-30WP_001211218.1_1023AbBOC	LDAP.....AYGVKSTLPDMLSFIHANLNPQKYPADIQRAINETHQGRYQVNTMYQALGW
DC-30WP_001211218.1_3189AbBOC	LDAP.....AYGVKSTLPDMLSFIHANLNPQKYPADIQRAINETHQGRYQVNTMYQALGW
ADC-30WP_001211218.1_594AbBOC	LDAP.....AYGVKSTLPDMLSFIHANLNPQKYPADIQRAINETHQGRYQVNTMYQALGW
DC-30WP_001211218.1_1718AbBOC	LDAP.....AYGVKSTLPDMLSFIHANLNPQKYPADIQRAINETHQGRYQVNTMYQALGW
DC-30WP_001211218.1_2484AbBOC	LDAP.....AYGVKSTLPDMLSFIHANLNPQKYPADIQRAINETHQGRYQVNTMYQALGW
DC-30WP_001211218.1_3305AbBOC	LDAP.....AYGVKSTLPDMLSFIHANLNPQKYPADIQRAINETHQGRYQVNTMYQALGW
DC-30WP_001211218.1_3346AbBOC	LDAP.....AYGVKSTLPDMLSFIHANLNPQKYPADIQRAINETHQGRYQVNTMYQALGW
DC-30WP_001211218.1_1482AbBOC	LDAP.....AYGVKSTLPDMLSFIHANLNPQKYPADIQRAINETHQGRYQVNTMYQALGW
DC-30WP_001211218.1_2574AbBOC	LDAP.....AYGVKSTLPDMLSFIHANLNPQKYPADIQRAINETHQGRYQVNTMYQALGW
ADC-30WP_001211218.1_831AbBOC	LDAP.....AYGVKSTLPDMLSFIHANLNPQKYPADIQRAINETHQGRYQVNTMYQALGW
DC-30WP_001211218.1_1826AbBOC	LDAP.....AYGVKSTLPDMLSFIHANLNPQKYPADIQRAINETHQGRYQVNTMYQALGW
DC-30WP_001211218.1_1024AbBOC	LDAP.....AYGVKSTLPDMLSFIHANLNPQKYPADIQRAINETHQGRYQVNTMYQALGW
DC-30WP_001211218.1_1618AbBOC	LDAP.....AYGVKSTLPDMLSFIHANLNPQKYPADIQRAINETHQGRYQVNTMYQALGW
ADC-30WP_001211218.1_301AbBOC	LDAP.....AYGVKSTLPDMLSFIHANLNPQKYPADIQRAINETHQGRYQVNTMYQALGW
ADC-30WP_001211218.1_940AbBOC	LDAP.....AYGVKSTLPDMLSFIHANLNPQKYPADIQRAINETHQGRYQVNTMYQALGW
DC-30WP_001211218.1_2949AbBOC	LDAP.....AYGVKSTLPDMLSFIHANLNPQKYPADIQRAINETHQGRYQVNTMYQALGW
DC-30WP_001211218.1_1028AbBOC	LDAP.....AYGVKSTLPDMLSFIHANLNPQKYPADIQRAINETHQGRYQVNTMYQALGW
DC-30WP_001211218.1_2526AbBOC	LDAP.....AYGVKSTLPDMLSFIHANLNPQKYPADIQRAINETHQGRYQVNTMYQALGW
DC-30WP_001211218.1_1040AbBOC	LDAP.....AYGVKSTLPDMLSFIHANLNPQKYPADIQRAINETHQGRYQVNTMYQALGW
DC-30WP_001211218.1_1089AbBOC	LDAP.....AYGVKSTLPDMLSFIHANLNPQKYPADIQRAINETHQGRYQVNTMYQALGW
DC-30WP_001211218.1_1099AbBOC	LDAP.....AYGVKSTLPDMLSFIHANLNPQKYPADIQRAINETHQGRYQVNTMYQALGW
DC-30WP_001211218.1_1206AbBOC	LDAP.....AYGVKSTLPDMLSFIHANLNPQKYPADIQRAINETHQGRYQVNTMYQALGW
DC-30WP_001211218.1_1167AbBOC	LDAP.....AYGVKSTLPDMLSFIHANLNPQKYPADIQRAINETHQGRYQVNTMYQALGW
DC-30WP_001211218.1_1186AbBOC	LDAP.....AYGVKSTLPDMLSFIHANLNPQKYPADIQRAINETHQGRYQVNTMYQALGW
DC-30WP_001211218.1_1165AbBOC	LDAP.....AYGVKSTLPDMLSFIHANLNPQKYPADIQRAINETHQGRYQVNTMYQALGW
DC-30WP_001211218.1_1077AbBOC	LDAP.....AYGVKSTLPDMLSFIHANLNPQKYPADIQRAINETHQGRYQVNTMYQALGW
DC-30WP_001211218.1_1078AbBOC	LDAP.....AYGVKSTLPDMLSFIHANLNPQKYPADIQRAINETHQGRYQVNTMYQALGW
DC-30WP_001211218.1_1071AbBOC	LDAP.....AYGVKSTLPDMLSFIHANLNPQKYPADIQRAINETHQGRYQVNTMYQALGW
DC-30WP_001211218.1_2694AbBOC	LDAP.....AYGVKSTLPDMLSFIHANLNPQKYPADIQRAINETHQGRYQVNTMYQALGW
DC-30WP_001211218.1_3340AbBOC	LDAP.....AYGVKSTLPDMLSFIHANLNPQKYPADIQRAINETHQGRYQVNTMYQALGW
DC-30WP_001211218.1_1022AbBOC	LDAP.....AYGVKSTLPDMLSFIHANLNPQKYPADIQRAINETHQGRYQVNTMYQALGW
DC-30WP_001211218.1_1025AbBOC	LDAP.....AYGVKSTLPDMLSFIHANLNPQKYPADIQRAINETHQGRYQVNTMYQALGW
DC-30WP_001211218.1_1158AbBOC	LDAP.....AYGVKSTLPDMLSFIHANLNPQKYPADIQRAINETHQGRYQVNTMYQALGW
DC-30WP_001211218.1_1020AbBOC	LDAP.....AYGVKSTLPDMLSFIHANLNPQKYPADIQRAINETHQGRYQVNTMYQALGW
DC-30WP_001211218.1_1021AbBOC	LDAP.....AYGVKSTLPDMLSFIHANLNPQKYPADIQRAINETHQGRYQVNTMYQALGW
ADC-30WP_001211218.1_532AbBOC	LDAP.....AYGVKSTLPDMLSFIHANLNPQKYPADIQRAINETHQGRYQVNTMYQALGW
ADC-30WP_001211218.1_539AbBOC	LDAP.....AYGVKSTLPDMLSFIHANLNPQKYPADIQRAINETHQGRYQVNTMYQALGW
DC-30WP_001211218.1_1033AbBOC	LDAP.....AYGVKSTLPDMLSFIHANLNPQKYPADIQRAINETHQGRYQVNTMYQALGW
DC-30WP_001211218.1_1069AbBOC	LDAP.....AYGVKSTLPDMLSFIHANLNPQKYPADIQRAINETHQGRYQVNTMYQALGW
DC-30WP_001211218.1_1029AbBOC	LDAP.....AYGVKSTLPDMLSFIHANLNPQKYPADIQRAINETHQGRYQVNTMYQALGW
DC-30WP_001211218.1_1030AbBOC	LDAP.....AYGVKSTLPDMLSFIHANLNPQKYPADIQRAINETHQGRYQVNTMYQALGW
DC-30WP_001211218.1_1096AbBOC	LDAP.....AYGVKSTLPDMLSFIHANLNPQKYPADIQRAINETHQGRYQVNTMYQALGW
DC-30WP_001211218.1_1037AbBOC	LDAP.....AYGVKSTLPDMLSFIHANLNPQKYPADIQRAINETHQGRYQVNTMYQALGW
DC-30WP_001211218.1_1098AbBOC	LDAP.....AYGVKSTLPDMLSFIHANLNPQKYPADIQRAINETHQGRYQVNTMYQALGW
DC-30WP_001211218.1_1046AbBOC	LDAP.....AYGVKSTLPDMLSFIHANLNPQKYPADIQRAINETHQGRYQVNTMYQALGW
DC-30WP_001211218.1_1032AbBOC	LDAP.....AYGVKSTLPDMLSFIHANLNPQKYPADIQRAINETHQGRYQVNTMYQALGW
DC-30WP_001211218.1_1070AbBOC	LDAP.....AYGVKSTLPDMLSFIHANLNPQKYPADIQRAINETHQGRYQVNTMYQALGW
DC-30WP_001211218.1_1139AbBOC	LDAP.....AYGVKSTLPDMLSFIHANLNPQKYPADIQRAINETHQGRYQVNTMYQALGW
DC-30WP_001211218.1_1031AbBOC	LDAP.....AYGVKSTLPDMLSFIHANLNPQKYPADIQRAINETHQGRYQVNTMYQALGW
DC-30WP_001211218.1_1066AbBOC	LDAP.....AYGVKSTLPDMLSFIHANLNPQKYPADIQRAINETHQGRYQVNTMYQALGW
DC-30WP_001211218.1_1102AbBOC	LDAP.....AYGVKSTLPDMLSFIHANLNPQKYPADIQRAINETHQGRYQVNTMYQALGW
ADC-30USX57838.1_2957AbBOC	LDAP.....AYGVKSTLPDMLSFIHANLNPQKYPADIQRAINETHQGRYQVNTMYQALGW
ADC-30USX59985.1_1918AbBOC	LDAP.....AYGVKSTLPDMLSFIHANLNPQKYPADIQRAINETHQGRYQVNTMYQALGW
ADC-30USX63799.1_2772AbBOC	LDAP.....AYGVKSTLPDMLSFIHANLNPQKYPADIQRAINETHQGRYQVNTMYQALGW
ADC-30USX74012.1_305AbBOC	LDAP.....AYGVKSTLPDMLSFIHANLNPQKYPADIQRAINETHQGRYQVNTMYQALGW
ADC-30USZ52468.1_2397AbBOC	LDAP.....AYGVKSTLPDMLSFIHANLNPQKYPADIQRAINETHQGRYQVNTMYQALGW
DC-30WP_001211218.1_2008AbBOC	LDAP.....AYGVKSTLPDMLSFIHANLNPQKYPADIQRAINETHQGRYQVNTMYQALGW
ADC-30WCL78976.1_1026AbBOC	LDAP.....AYGVKSTLPDMLSFIHANLNPQKYPADIQRAINETHQGRYQVNTMYQALGW
ADC-30WDO27826.1_1034AbBOC	LDAP.....AYGVKSTLPDMLSFIHANLNPQKYPADIQRAINETHQGRYQVNTMYQALGW
DC-30WP_001211218.1_2125AbBOC	LDAP.....AYGVKSTLPDMLSFIHANLNPQKYPADIQRAINETHQGRYQVNTMYQALGW
DC-30WP_001211218.1_2729AbBOC	LDAP.....AYGVKSTLPDMLSFIHANLNPQKYPADIQRAINETHQGRYQVNTMYQALGW

```
DC-30WP_001211218.1_2692AbBCP  LDAP.....AYGVKSTLPDMLSFIHANLNPQKYPADIQRAINETHQGRYQVNTMYQALGW
DC-30WP_001211218.1_2549AbBCP  LDAP.....AYGVKSTLPDMLSFIHANLNPQKYPADIQRAINETHQGRYQVNTMYQALGW
DC-30WP_001211218.1_1026AbBCP  LDAP.....AYGVKSTLPDMLSFIHANLNPQKYPADIQRAINETHQGRYQVNTMYQALGW
ADC-30WP_001211218.1_373AbBCP  LDAP.....AYGVKSTLPDMLSFIHANLNPQKYPADIQRAINETHQGRYQVNTMYQALGW
DC-30WP_001211218.1_2465AbBCP  LDAP.....AYGVKSTLPDMLSFIHANLNPQKYPADIQRAINETHQGRYQVNTMYQALGW
DC-30WP_001211218.1_1027AbBCP  LDAP.....AYGVKSTLPDMLSFIHANLNPQKYPADIQRAINETHQGRYQVNTMYQALGW
DC-30WP_001211218.1_2650AbBCP  LDAP.....AYGVKSTLPDMLSFIHANLNPQKYPADIQRAINETHQGRYQVNTMYQALGW
DC-30WP_001211218.1_2674AbBCP  LDAP.....AYGVKSTLPDMLSFIHANLNPQKYPADIQRAINETHQGRYQVNTMYQALGW
DC-30WP_001211218.1_2689AbBCP  LDAP.....AYGVKSTLPDMLSFIHANLNPQKYPADIQRAINETHQGRYQVNTMYQALGW
DC-30WP_001211218.1_1105AbBCP  LDAP.....AYGVKSTLPDMLSFIHANLNPQKYPADIQRAINETHQGRYQVNTMYQALGW
DC-30WP_001211218.1_2670AbBCP  LDAP.....AYGVKSTLPDMLSFIHANLNPQKYPADIQRAINETHQGRYQVNTMYQALGW
DC-30WP_001211218.1_1019AbBCP  LDAP.....AYGVKSTLPDMLSFIHANLNPQKYPADIQRAINETHQGRYQVNTMYQALGW
DC-30WP_001211218.1_1039AbBCP  LDAP.....AYGVKSTLPDMLSFIHANLNPQKYPADIQRAINETHQGRYQVNTMYQALGW
DC-30WP_001211218.1_1023AbBCP  LDAP.....AYGVKSTLPDMLSFIHANLNPQKYPADIQRAINETHQGRYQVNTMYQALGW
DC-30WP_001211218.1_3189AbBCP  LDAP.....AYGVKSTLPDMLSFIHANLNPQKYPADIQRAINETHQGRYQVNTMYQALGW
ADC-30WP_001211218.1_594AbBCP  LDAP.....AYGVKSTLPDMLSFIHANLNPQKYPADIQRAINETHQGRYQVNTMYQALGW
DC-30WP_001211218.1_1718AbBCP  LDAP.....AYGVKSTLPDMLSFIHANLNPQKYPADIQRAINETHQGRYQVNTMYQALGW
DC-30WP_001211218.1_2484AbBCP  LDAP.....AYGVKSTLPDMLSFIHANLNPQKYPADIQRAINETHQGRYQVNTMYQALGW
DC-30WP_001211218.1_3305AbBCP  LDAP.....AYGVKSTLPDMLSFIHANLNPQKYPADIQRAINETHQGRYQVNTMYQALGW
DC-30WP_001211218.1_3346AbBCP  LDAP.....AYGVKSTLPDMLSFIHANLNPQKYPADIQRAINETHQGRYQVNTMYQALGW
DC-30WP_001211218.1_1482AbBCP  LDAP.....AYGVKSTLPDMLSFIHANLNPQKYPADIQRAINETHQGRYQVNTMYQALGW
DC-30WP_001211218.1_2574AbBCP  LDAP.....AYGVKSTLPDMLSFIHANLNPQKYPADIQRAINETHQGRYQVNTMYQALGW
ADC-30WP_001211218.1_831AbBCP  LDAP.....AYGVKSTLPDMLSFIHANLNPQKYPADIQRAINETHQGRYQVNTMYQALGW
DC-30WP_001211218.1_1826AbBCP  LDAP.....AYGVKSTLPDMLSFIHANLNPQKYPADIQRAINETHQGRYQVNTMYQALGW
DC-30WP_001211218.1_1024AbBCP  LDAP.....AYGVKSTLPDMLSFIHANLNPQKYPADIQRAINETHQGRYQVNTMYQALGW
DC-30WP_001211218.1_1618AbBCP  LDAP.....AYGVKSTLPDMLSFIHANLNPQKYPADIQRAINETHQGRYQVNTMYQALGW
ADC-30WP_001211218.1_301AbBCP  LDAP.....AYGVKSTLPDMLSFIHANLNPQKYPADIQRAINETHQGRYQVNTMYQALGW
ADC-30WP_001211218.1_940AbBCP  LDAP.....AYGVKSTLPDMLSFIHANLNPQKYPADIQRAINETHQGRYQVNTMYQALGW
DC-30WP_001211218.1_2949AbBCP  LDAP.....AYGVKSTLPDMLSFIHANLNPQKYPADIQRAINETHQGRYQVNTMYQALGW
DC-30WP_001211218.1_1028AbBCP  LDAP.....AYGVKSTLPDMLSFIHANLNPQKYPADIQRAINETHQGRYQVNTMYQALGW
DC-30WP_001211218.1_2526AbBCP  LDAP.....AYGVKSTLPDMLSFIHANLNPQKYPADIQRAINETHQGRYQVNTMYQALGW
DC-30WP_001211218.1_1040AbBCP  LDAP.....AYGVKSTLPDMLSFIHANLNPQKYPADIQRAINETHQGRYQVNTMYQALGW
DC-30WP_001211218.1_1089AbBCP  LDAP.....AYGVKSTLPDMLSFIHANLNPQKYPADIQRAINETHQGRYQVNTMYQALGW
DC-30WP_001211218.1_1099AbBCP  LDAP.....AYGVKSTLPDMLSFIHANLNPQKYPADIQRAINETHQGRYQVNTMYQALGW
DC-30WP_001211218.1_1206AbBCP  LDAP.....AYGVKSTLPDMLSFIHANLNPQKYPADIQRAINETHQGRYQVNTMYQALGW
DC-30WP_001211218.1_1167AbBCP  LDAP.....AYGVKSTLPDMLSFIHANLNPQKYPADIQRAINETHQGRYQVNTMYQALGW
DC-30WP_001211218.1_1186AbBCP  LDAP.....AYGVKSTLPDMLSFIHANLNPQKYPADIQRAINETHQGRYQVNTMYQALGW
DC-30WP_001211218.1_1165AbBCP  LDAP.....AYGVKSTLPDMLSFIHANLNPQKYPADIQRAINETHQGRYQVNTMYQALGW
DC-30WP_001211218.1_1077AbBCP  LDAP.....AYGVKSTLPDMLSFIHANLNPQKYPADIQRAINETHQGRYQVNTMYQALGW
DC-30WP_001211218.1_1078AbBCP  LDAP.....AYGVKSTLPDMLSFIHANLNPQKYPADIQRAINETHQGRYQVNTMYQALGW
DC-30WP_001211218.1_1071AbBCP  LDAP.....AYGVKSTLPDMLSFIHANLNPQKYPADIQRAINETHQGRYQVNTMYQALGW
DC-30WP_001211218.1_2694AbBCP  LDAP.....AYGVKSTLPDMLSFIHANLNPQKYPADIQRAINETHQGRYQVNTMYQALGW
DC-30WP_001211218.1_3340AbBCP  LDAP.....AYGVKSTLPDMLSFIHANLNPQKYPADIQRAINETHQGRYQVNTMYQALGW
DC-30WP_001211218.1_1022AbBCP  LDAP.....AYGVKSTLPDMLSFIHANLNPQKYPADIQRAINETHQGRYQVNTMYQALGW
DC-30WP_001211218.1_1025AbBCP  LDAP.....AYGVKSTLPDMLSFIHANLNPQKYPADIQRAINETHQGRYQVNTMYQALGW
DC-30WP_001211218.1_1158AbBCP  LDAP.....AYGVKSTLPDMLSFIHANLNPQKYPADIQRAINETHQGRYQVNTMYQALGW
DC-30WP_001211218.1_1020AbBCP  LDAP.....AYGVKSTLPDMLSFIHANLNPQKYPADIQRAINETHQGRYQVNTMYQALGW
DC-30WP_001211218.1_1021AbBCP  LDAP.....AYGVKSTLPDMLSFIHANLNPQKYPADIQRAINETHQGRYQVNTMYQALGW
ADC-30WP_001211218.1_532AbBCP  LDAP.....AYGVKSTLPDMLSFIHANLNPQKYPADIQRAINETHQGRYQVNTMYQALGW
ADC-30WP_001211218.1_539AbBCP  LDAP.....AYGVKSTLPDMLSFIHANLNPQKYPADIQRAINETHQGRYQVNTMYQALGW
DC-30WP_001211218.1_1033AbBCP  LDAP.....AYGVKSTLPDMLSFIHANLNPQKYPADIQRAINETHQGRYQVNTMYQALGW
DC-30WP_001211218.1_1069AbBCP  LDAP.....AYGVKSTLPDMLSFIHANLNPQKYPADIQRAINETHQGRYQVNTMYQALGW
DC-30WP_001211218.1_1029AbBCP  LDAP.....AYGVKSTLPDMLSFIHANLNPQKYPADIQRAINETHQGRYQVNTMYQALGW
DC-30WP_001211218.1_1030AbBCP  LDAP.....AYGVKSTLPDMLSFIHANLNPQKYPADIQRAINETHQGRYQVNTMYQALGW
DC-30WP_001211218.1_1096AbBCP  LDAP.....AYGVKSTLPDMLSFIHANLNPQKYPADIQRAINETHQGRYQVNTMYQALGW
DC-30WP_001211218.1_1037AbBCP  LDAP.....AYGVKSTLPDMLSFIHANLNPQKYPADIQRAINETHQGRYQVNTMYQALGW
DC-30WP_001211218.1_1098AbBCP  LDAP.....AYGVKSTLPDMLSFIHANLNPQKYPADIQRAINETHQGRYQVNTMYQALGW
DC-30WP_001211218.1_1046AbBCP  LDAP.....AYGVKSTLPDMLSFIHANLNPQKYPADIQRAINETHQGRYQVNTMYQALGW
DC-30WP_001211218.1_1032AbBCP  LDAP.....AYGVKSTLPDMLSFIHANLNPQKYPADIQRAINETHQGRYQVNTMYQALGW
DC-30WP_001211218.1_1070AbBCP  LDAP.....AYGVKSTLPDMLSFIHANLNPQKYPADIQRAINETHQGRYQVNTMYQALGW
DC-30WP_001211218.1_1139AbBCP  LDAP.....AYGVKSTLPDMLSFIHANLNPQKYPADIQRAINETHQGRYQVNTMYQALGW
DC-30WP_001211218.1_1031AbBCP  LDAP.....AYGVKSTLPDMLSFIHANLNPQKYPADIQRAINETHQGRYQVNTMYQALGW
DC-30WP_001211218.1_1066AbBCP  LDAP.....AYGVKSTLPDMLSFIHANLNPQKYPADIQRAINETHQGRYQVNTMYQALGW
DC-30WP_001211218.1_1102AbBCP  LDAP.....AYGVKSTLPDMLSFIHANLNPQKYPADIQRAINETHQGRYQVNTMYQALGW
ADC-30USX57838.1_2957AbBCP     LDAP.....AYGVKSTLPDMLSFIHANLNPQKYPADIQRAINETHQGRYQVNTMYQALGW
ADC-30USX59985.1_1918AbBCP     LDAP.....AYGVKSTLPDMLSFIHANLNPQKYPADIQRAINETHQGRYQVNTMYQALGW
ADC-30USX63799.1_2772AbBCP     LDAP.....AYGVKSTLPDMLSFIHANLNPQKYPADIQRAINETHQGRYQVNTMYQALGW
ADC-30USX74012.1_305AbBCP      LDAP.....AYGVKSTLPDMLSFIHANLNPQKYPADIQRAINETHQGRYQVNTMYQALGW
ADC-30USZ52468.1_2397AbBCP     LDAP.....AYGVKSTLPDMLSFIHANLNPQKYPADIQRAINETHQGRYQVNTMYQALGW
DC-30WP_001211218.1_2008AbBCP  LDAP.....AYGVKSTLPDMLSFIHANLNPQKYPADIQRAINETHQGRYQVNTMYQALGW
ADC-30WCL78976.1_1026AbBCP     LDAP.....AYGVKSTLPDMLSFIHANLNPQKYPADIQRAINETHQGRYQVNTMYQALGW
ADC-30WDO27826.1_1034AbBCP     LDAP.....AYGVKSTLPDMLSFIHANLNPQKYPADIQRAINETHQGRYQVNTMYQALGW
DC-30WP_001211218.1_2125AbBCP  LDAP.....AYGVKSTLPDMLSFIHANLNPQKYPADIQRAINETHQGRYQVNTMYQALGW
DC-30WP_001211218.1_2729AbBCP  LDAP.....AYGVKSTLPDMLSFIHANLNPQKYPADIQRAINETHQGRYQVNTMYQALGW
ADC-30ADX93237.1_2806AbBCP     LDAP.....AYGVKSTLPDMLSFIHANLNPQKYPADIQRAINETHQGRYQVNTMYQALGW
ADC-82WP_001211216.1_2747AbOC  LDAL.....AYGVKSTLPDMLSFIHANLNPQKYPADIQRAINETHQGRYQVNTMYQALGW
DC-82WP_001211216.1_1093AbBOC  LDAL.....AYGVKSTLPDMLSFIHANLNPQKYPADIQRAINETHQGRYQVNTMYQALGW
DC-82WP_001211216.1_1071AbBOC  LDAL.....AYGVKSTLPDMLSFIHANLNPQKYPADIQRAINETHQGRYQVNTMYQALGW
DC-82WP_001211216.1_1093AbBCP  LDAL.....AYGVKSTLPDMLSFIHANLNPQKYPADIQRAINETHQGRYQVNTMYQALGW
DC-82WP_001211216.1_1071AbBCP  LDAL.....AYGVKSTLPDMLSFIHANLNPQKYPADIQRAINETHQGRYQVNTMYQALGW
DC-162WP_031980335.1_1153AbOC  LDAP.....EYGVKSTLPDMLSFIHANLNPQKYPADIQRAINETHQGRYQVNTMYQALGW
C-162WP_031980335.1_1028AbBOC  LDAP.....EYGVKSTLPDMLSFIHANLNPQKYPADIQRAINETHQGRYQVNTMYQALGW
C-162WP_031980335.1_1023AbBOC  LDAP.....EYGVKSTLPDMLSFIHANLNPQKYPADIQRAINETHQGRYQVNTMYQALGW
C-162WP_031980335.1_1028AbBCP  LDAP.....EYGVKSTLPDMLSFIHANLNPQKYPADIQRAINETHQGRYQVNTMYQALGW
C-162WP_031980335.1_1023AbBCP  LDAP.....EYGVKSTLPDMLSFIHANLNPQKYPADIQRAINETHQGRYQVNTMYQALGW
ADC-73WP_001211219.1_1012AbOC  LDAP.....AYSVKSTLPDMLSFIHANLNPQKYPADIQRAINETHQGRYQVNTMYQALGW
ADC-73WP_001211219.1_1953AbOC  LDAP.....AYSVKSTLPDMLSFIHANLNPQKYPADIQRAINETHQGRYQVNTMYQALGW
ADC-73WP_001211219.1_1014AbOC  LDAP.....AYSVKSTLPDMLSFIHANLNPQKYPADIQRAINETHQGRYQVNTMYQALGW
ADC-73WP_001211219.1_1041AbOC  LDAP.....AYSVKSTLPDMLSFIHANLNPQKYPADIQRAINETHQGRYQVNTMYQALGW
ADC-73WP_001211219.1_1028AbOC  LDAP.....AYSVKSTLPDMLSFIHANLNPQKYPADIQRAINETHQGRYQVNTMYQALGW
ADC-73WP_001211219.1_2674AbOC  LDAP.....AYSVKSTLPDMLSFIHANLNPQKYPADIQRAINETHQGRYQVNTMYQALGW
ADC-73WP_001211219.1_462AbOC   LDAP.....AYSVKSTLPDMLSFIHANLNPQKYPADIQRAINETHQGRYQVNTMYQALGW
ADC-73WP_001211219.1_3530AbOC  LDAP.....AYSVKSTLPDMLSFIHANLNPQKYPADIQRAINETHQGRYQVNTMYQALGW
ADC-73WP_001211219.1_3621AbOC  LDAP.....AYSVKSTLPDMLSFIHANLNPQKYPADIQRAINETHQGRYQVNTMYQALGW
ADC-73WP_001211219.1_3381AbOC  LDAP.....AYSVKSTLPDMLSFIHANLNPQKYPADIQRAINETHQGRYQVNTMYQALGW
ADC-73WP_001211219.1_2515AbOC  LDAP.....AYSVKSTLPDMLSFIHANLNPQKYPADIQRAINETHQGRYQVNTMYQALGW
ADC-73WP_001211219.1_3574AbOC  LDAP.....AYSVKSTLPDMLSFIHANLNPQKYPADIQRAINETHQGRYQVNTMYQALGW
ADC-73WP_001211219.1_1656AbOC  LDAP.....AYSVKSTLPDMLSFIHANLNPQKYPADIQRAINETHQGRYQVNTMYQALGW
ADC-73WP_001211219.1_2591AbOC  LDAP.....AYSVKSTLPDMLSFIHANLNPQKYPADIQRAINETHQGRYQVNTMYQALGW
ADC-73WP_001211219.1_2602AbOC  LDAP.....AYSVKSTLPDMLSFIHANLNPQKYPADIQRAINETHQGRYQVNTMYQALGW
ADC-73WP_001211219.1_910AbOC   LDAP.....AYSVKSTLPDMLSFIHANLNPQKYPADIQRAINETHQGRYQVNTMYQALGW
ADC-73WP_001211219.1_394AbOC   LDAP.....AYSVKSTLPDMLSFIHANLNPQKYPADIQRAINETHQGRYQVNTMYQALGW
ADC-73WP_001211219.1_1030AbOC  LDAP.....AYSVKSTLPDMLSFIHANLNPQKYPADIQRAINETHQGRYQVNTMYQALGW
ADC-73WP_001211219.1_1023AbOC  LDAP.....AYSVKSTLPDMLSFIHANLNPQKYPADIQRAINETHQGRYQVNTMYQALGW
ADC-73UYQ66995.1_1967AbOC      LDAP.....AYSVKSTLPDMLSFIHANLNPQKYPADIQRAINETHQGRYQVNTMYQALGW
ADC-73WP_001211219.1_906AbBOC  LDAP.....AYSVKSTLPDMLSFIHANLNPQKYPADIQRAINETHQGRYQVNTMYQALGW
DC-73WP_001211219.1_2649AbBOC  LDAP.....AYSVKSTLPDMLSFIHANLNPQKYPADIQRAINETHQGRYQVNTMYQALGW
DC-73WP_001211219.1_2693AbBOC  LDAP.....AYSVKSTLPDMLSFIHANLNPQKYPADIQRAINETHQGRYQVNTMYQALGW
ADC-73AQU57925.1_2703AbBOC     LDAP.....AYSVKSTLPDMLSFIHANLNPQKYPADIQRAINETHQGRYQVNTMYQALGW
DC-73WP_001211219.1_1026AbBOC  LDAP.....AYSVKSTLPDMLSFIHANLNPQKYPADIQRAINETHQGRYQVNTMYQALGW
DC-73WP_001211219.1_1019AbBOC  LDAP.....AYSVKSTLPDMLSFIHANLNPQKYPADIQRAINETHQGRYQVNTMYQALGW
DC-73WP_001211219.1_1024AbBOC  LDAP.....AYSVKSTLPDMLSFIHANLNPQKYPADIQRAINETHQGRYQVNTMYQALGW
ADC-73WP_001211219.1_930AbBOC  LDAP.....AYSVKSTLPDMLSFIHANLNPQKYPADIQRAINETHQGRYQVNTMYQALGW
DC-73WP_001211219.1_2609AbBOC  LDAP.....AYSVKSTLPDMLSFIHANLNPQKYPADIQRAINETHQGRYQVNTMYQALGW
DC-73WP_001211219.1_3456AbBOC  LDAP.....AYSVKSTLPDMLSFIHANLNPQKYPADIQRAINETHQGRYQVNTMYQALGW
DC-73WP_001211219.1_2614AbBOC  LDAP.....AYSVKSTLPDMLSFIHANLNPQKYPADIQRAINETHQGRYQVNTMYQALGW
DC-73WP_001211219.1_1025AbBOC  LDAP.....AYSVKSTLPDMLSFIHANLNPQKYPADIQRAINETHQGRYQVNTMYQALGW
DC-73WP_001211219.1_1030AbBOC  LDAP.....AYSVKSTLPDMLSFIHANLNPQKYPADIQRAINETHQGRYQVNTMYQALGW
DC-73WP_001211219.1_1173AbBOC  LDAP.....AYSVKSTLPDMLSFIHANLNPQKYPADIQRAINETHQGRYQVNTMYQALGW
DC-73WP_001211219.1_1021AbBOC  LDAP.....AYSVKSTLPDMLSFIHANLNPQKYPADIQRAINETHQGRYQVNTMYQALGW
DC-73WP_001211219.1_2449AbBOC  LDAP.....AYSVKSTLPDMLSFIHANLNPQKYPADIQRAINETHQGRYQVNTMYQALGW
DC-73WP_001211219.1_1176AbBOC  LDAP.....AYSVKSTLPDMLSFIHANLNPQKYPADIQRAINETHQGRYQVNTMYQALGW
DC-73WP_001211219.1_1161AbBOC  LDAP.....AYSVKSTLPDMLSFIHANLNPQKYPADIQRAINETHQGRYQVNTMYQALGW
DC-73WP_001211219.1_1070AbBOC  LDAP.....AYSVKSTLPDMLSFIHANLNPQKYPADIQRAINETHQGRYQVNTMYQALGW
DC-73WP_001211219.1_1079AbBOC  LDAP.....AYSVKSTLPDMLSFIHANLNPQKYPADIQRAINETHQGRYQVNTMYQALGW
DC-73WP_001211219.1_1078AbBOC  LDAP.....AYSVKSTLPDMLSFIHANLNPQKYPADIQRAINETHQGRYQVNTMYQALGW
DC-73WP_001211219.1_1037AbBOC  LDAP.....AYSVKSTLPDMLSFIHANLNPQKYPADIQRAINETHQGRYQVNTMYQALGW
DC-73WP_001211219.1_1035AbBOC  LDAP.....AYSVKSTLPDMLSFIHANLNPQKYPADIQRAINETHQGRYQVNTMYQALGW
DC-73WP_001211219.1_1033AbBOC  LDAP.....AYSVKSTLPDMLSFIHANLNPQKYPADIQRAINETHQGRYQVNTMYQALGW
DC-73WP_001211219.1_1041AbBOC  LDAP.....AYSVKSTLPDMLSFIHANLNPQKYPADIQRAINETHQGRYQVNTMYQALGW
DC-73WP_001211219.1_1023AbBOC  LDAP.....AYSVKSTLPDMLSFIHANLNPQKYPADIQRAINETHQGRYQVNTMYQALGW
ADC-73USX69547.1_2386AbBOC     LDAP.....AYSVKSTLPDMLSFIHANLNPQKYPADIQRAINETHQGRYQVNTMYQALGW
DC-73WP_001211219.1_1034AbBOC  LDAP.....AYSVKSTLPDMLSFIHANLNPQKYPADIQRAINETHQGRYQVNTMYQALGW
ADC-73WP_001211219.1_852AbBOC  LDAP.....AYSVKSTLPDMLSFIHANLNPQKYPADIQRAINETHQGRYQVNTMYQALGW
DC-73WP_001211219.1_3070AbBOC  LDAP.....AYSVKSTLPDMLSFIHANLNPQKYPADIQRAINETHQGRYQVNTMYQALGW
ADC-73WBS60953.1_1026AbBOC     LDAP.....AYSVKSTLPDMLSFIHANLNPQKYPADIQRAINETHQGRYQVNTMYQALGW
ADC-73WDC39660.1_1023AbBOC     LDAP.....AYSVKSTLPDMLSFIHANLNPQKYPADIQRAINETHQGRYQVNTMYQALGW
ADC-73WDC73972.1_1415AbBOC     LDAP.....AYSVKSTLPDMLSFIHANLNPQKYPADIQRAINETHQGRYQVNTMYQALGW
ADC-73WDC70172.1_1037AbBOC     LDAP.....AYSVKSTLPDMLSFIHANLNPQKYPADIQRAINETHQGRYQVNTMYQALGW
DC-73WP_001211219.1_1101AbBOC  LDAP.....AYSVKSTLPDMLSFIHANLNPQKYPADIQRAINETHQGRYQVNTMYQALGW
DC-73WP_001211219.1_1667AbBOC  LDAP.....AYSVKSTLPDMLSFIHANLNPQKYPADIQRAINETHQGRYQVNTMYQALGW
ADC-73WP_001211219.1_287AbBOC  LDAP.....AYSVKSTLPDMLSFIHANLNPQKYPADIQRAINETHQGRYQVNTMYQALGW
DC-73WP_001211219.1_3177AbBOC  LDAP.....AYSVKSTLPDMLSFIHANLNPQKYPADIQRAINETHQGRYQVNTMYQALGW
DC-73WP_001211219.1_2501AbBOC  LDAP.....AYSVKSTLPDMLSFIHANLNPQKYPADIQRAINETHQGRYQVNTMYQALGW
DC-73WP_001211219.1_2535AbBOC  LDAP.....AYSVKSTLPDMLSFIHANLNPQKYPADIQRAINETHQGRYQVNTMYQALGW
DC-73WP_001211219.1_3150AbBOC  LDAP.....AYSVKSTLPDMLSFIHANLNPQKYPADIQRAINETHQGRYQVNTMYQALGW
DC-73WP_001211219.1_3714AbBOC  LDAP.....AYSVKSTLPDMLSFIHANLNPQKYPADIQRAINETHQGRYQVNTMYQALGW
ADC-73WP_001211219.1_722AbBOC  LDAP.....AYSVKSTLPDMLSFIHANLNPQKYPADIQRAINETHQGRYQVNTMYQALGW
DC-73WP_001211219.1_3650AbBOC  LDAP.....AYSVKSTLPDMLSFIHANLNPQKYPADIQRAINETHQGRYQVNTMYQALGW
ADC-73WP_001211219.1_568AbBOC  LDAP.....AYSVKSTLPDMLSFIHANLNPQKYPADIQRAINETHQGRYQVNTMYQALGW
DC-73WP_001211219.1_3226AbBOC  LDAP.....AYSVKSTLPDMLSFIHANLNPQKYPADIQRAINETHQGRYQVNTMYQALGW
ADC-73WP_001211219.1_556AbBOC  LDAP.....AYSVKSTLPDMLSFIHANLNPQKYPADIQRAINETHQGRYQVNTMYQALGW
```

```
DC-73WP_001211219.1_2436AbBOC     LDAP.....AYSVKSTLPDMLSFIHANLNPQKYPADIQRAINETHQGRYQVNTMYQALGW
DC-73WP_001211219.1_1029AbBOC     LDAP.....AYSVKSTLPDMLSFIHANLNPQKYPADIQRAINETHQGRYQVNTMYQALGW
DC-73WP_001211219.1_1013AbBOC     LDAP.....AYSVKSTLPDMLSFIHANLNPQKYPADIQRAINETHQGRYQVNTMYQALGW
ADC-73WP_001211219.1_906AbBCP     LDAP.....AYSVKSTLPDMLSFIHANLNPQKYPADIQRAINETHQGRYQVNTMYQALGW
DC-73WP_001211219.1_2649AbBCP     LDAP.....AYSVKSTLPDMLSFIHANLNPQKYPADIQRAINETHQGRYQVNTMYQALGW
DC-73WP_001211219.1_2693AbBCP     LDAP.....AYSVKSTLPDMLSFIHANLNPQKYPADIQRAINETHQGRYQVNTMYQALGW
ADC-73AQU57925.1_2703AbBCP       LDAP.....AYSVKSTLPDMLSFIHANLNPQKYPADIQRAINETHQGRYQVNTMYQALGW
DC-73WP_001211219.1_1026AbBCP     LDAP.....AYSVKSTLPDMLSFIHANLNPQKYPADIQRAINETHQGRYQVNTMYQALGW
DC-73WP_001211219.1_1019AbBCP     LDAP.....AYSVKSTLPDMLSFIHANLNPQKYPADIQRAINETHQGRYQVNTMYQALGW
DC-73WP_001211219.1_1024AbBCP     LDAP.....AYSVKSTLPDMLSFIHANLNPQKYPADIQRAINETHQGRYQVNTMYQALGW
ADC-73WP_001211219.1_930AbBCP     LDAP.....AYSVKSTLPDMLSFIHANLNPQKYPADIQRAINETHQGRYQVNTMYQALGW
DC-73WP_001211219.1_2609AbBCP     LDAP.....AYSVKSTLPDMLSFIHANLNPQKYPADIQRAINETHQGRYQVNTMYQALGW
DC-73WP_001211219.1_3456AbBCP     LDAP.....AYSVKSTLPDMLSFIHANLNPQKYPADIQRAINETHQGRYQVNTMYQALGW
DC-73WP_001211219.1_2614AbBCP     LDAP.....AYSVKSTLPDMLSFIHANLNPQKYPADIQRAINETHQGRYQVNTMYQALGW
DC-73WP_001211219.1_1025AbBCP     LDAP.....AYSVKSTLPDMLSFIHANLNPQKYPADIQRAINETHQGRYQVNTMYQALGW
DC-73WP_001211219.1_1030AbBCP     LDAP.....AYSVKSTLPDMLSFIHANLNPQKYPADIQRAINETHQGRYQVNTMYQALGW
DC-73WP_001211219.1_1173AbBCP     LDAP.....AYSVKSTLPDMLSFIHANLNPQKYPADIQRAINETHQGRYQVNTMYQALGW
DC-73WP_001211219.1_1021AbBCP     LDAP.....AYSVKSTLPDMLSFIHANLNPQKYPADIQRAINETHQGRYQVNTMYQALGW
DC-73WP_001211219.1_2449AbBCP     LDAP.....AYSVKSTLPDMLSFIHANLNPQKYPADIQRAINETHQGRYQVNTMYQALGW
DC-73WP_001211219.1_1176AbBCP     LDAP.....AYSVKSTLPDMLSFIHANLNPQKYPADIQRAINETHQGRYQVNTMYQALGW
DC-73WP_001211219.1_1161AbBCP     LDAP.....AYSVKSTLPDMLSFIHANLNPQKYPADIQRAINETHQGRYQVNTMYQALGW
DC-73WP_001211219.1_1070AbBCP     LDAP.....AYSVKSTLPDMLSFIHANLNPQKYPADIQRAINETHQGRYQVNTMYQALGW
DC-73WP_001211219.1_1079AbBCP     LDAP.....AYSVKSTLPDMLSFIHANLNPQKYPADIQRAINETHQGRYQVNTMYQALGW
DC-73WP_001211219.1_1078AbBCP     LDAP.....AYSVKSTLPDMLSFIHANLNPQKYPADIQRAINETHQGRYQVNTMYQALGW
DC-73WP_001211219.1_1037AbBCP     LDAP.....AYSVKSTLPDMLSFIHANLNPQKYPADIQRAINETHQGRYQVNTMYQALGW
DC-73WP_001211219.1_1035AbBCP     LDAP.....AYSVKSTLPDMLSFIHANLNPQKYPADIQRAINETHQGRYQVNTMYQALGW
DC-73WP_001211219.1_1033AbBCP     LDAP.....AYSVKSTLPDMLSFIHANLNPQKYPADIQRAINETHQGRYQVNTMYQALGW
DC-73WP_001211219.1_1041AbBCP     LDAP.....AYSVKSTLPDMLSFIHANLNPQKYPADIQRAINETHQGRYQVNTMYQALGW
DC-73WP_001211219.1_1023AbBCP     LDAP.....AYSVKSTLPDMLSFIHANLNPQKYPADIQRAINETHQGRYQVNTMYQALGW
ADC-73USX69547.1_2386AbBCP       LDAP.....AYSVKSTLPDMLSFIHANLNPQKYPADIQRAINETHQGRYQVNTMYQALGW
DC-73WP_001211219.1_1034AbBCP     LDAP.....AYSVKSTLPDMLSFIHANLNPQKYPADIQRAINETHQGRYQVNTMYQALGW
ADC-73WP_001211219.1_852AbBCP     LDAP.....AYSVKSTLPDMLSFIHANLNPQKYPADIQRAINETHQGRYQVNTMYQALGW
DC-73WP_001211219.1_3070AbBCP     LDAP.....AYSVKSTLPDMLSFIHANLNPQKYPADIQRAINETHQGRYQVNTMYQALGW
ADC-73WBS60953.1_1026AbBCP       LDAP.....AYSVKSTLPDMLSFIHANLNPQKYPADIQRAINETHQGRYQVNTMYQALGW
ADC-73WDC39660.1_1023AbBCP       LDAP.....AYSVKSTLPDMLSFIHANLNPQKYPADIQRAINETHQGRYQVNTMYQALGW
ADC-73WDC73972.1_1415AbBCP       LDAP.....AYSVKSTLPDMLSFIHANLNPQKYPADIQRAINETHQGRYQVNTMYQALGW
ADC-73WDC70172.1_1037AbBCP       LDAP.....AYSVKSTLPDMLSFIHANLNPQKYPADIQRAINETHQGRYQVNTMYQALGW
DC-73WP_001211219.1_1101AbBCP     LDAP.....AYSVKSTLPDMLSFIHANLNPQKYPADIQRAINETHQGRYQVNTMYQALGW
DC-73WP_001211219.1_1667AbBCP     LDAP.....AYSVKSTLPDMLSFIHANLNPQKYPADIQRAINETHQGRYQVNTMYQALGW
ADC-73WP_001211219.1_287AbBCP     LDAP.....AYSVKSTLPDMLSFIHANLNPQKYPADIQRAINETHQGRYQVNTMYQALGW
DC-73WP_001211219.1_3177AbBCP     LDAP.....AYSVKSTLPDMLSFIHANLNPQKYPADIQRAINETHQGRYQVNTMYQALGW
DC-73WP_001211219.1_2501AbBCP     LDAP.....AYSVKSTLPDMLSFIHANLNPQKYPADIQRAINETHQGRYQVNTMYQALGW
DC-73WP_001211219.1_2535AbBCP     LDAP.....AYSVKSTLPDMLSFIHANLNPQKYPADIQRAINETHQGRYQVNTMYQALGW
DC-73WP_001211219.1_3150AbBCP     LDAP.....AYSVKSTLPDMLSFIHANLNPQKYPADIQRAINETHQGRYQVNTMYQALGW
DC-73WP_001211219.1_3714AbBCP     LDAP.....AYSVKSTLPDMLSFIHANLNPQKYPADIQRAINETHQGRYQVNTMYQALGW
ADC-73WP_001211219.1_722AbBCP     LDAP.....AYSVKSTLPDMLSFIHANLNPQKYPADIQRAINETHQGRYQVNTMYQALGW
DC-73WP_001211219.1_3650AbBCP     LDAP.....AYSVKSTLPDMLSFIHANLNPQKYPADIQRAINETHQGRYQVNTMYQALGW
ADC-73WP_001211219.1_568AbBCP     LDAP.....AYSVKSTLPDMLSFIHANLNPQKYPADIQRAINETHQGRYQVNTMYQALGW
DC-73WP_001211219.1_3226AbBCP     LDAP.....AYSVKSTLPDMLSFIHANLNPQKYPADIQRAINETHQGRYQVNTMYQALGW
ADC-73WP_001211219.1_556AbBCP     LDAP.....AYSVKSTLPDMLSFIHANLNPQKYPADIQRAINETHQGRYQVNTMYQALGW
DC-73WP_001211219.1_2436AbBCP     LDAP.....AYSVKSTLPDMLSFIHANLNPQKYPADIQRAINETHQGRYQVNTMYQALGW
DC-73WP_001211219.1_1029AbBCP     LDAP.....AYSVKSTLPDMLSFIHANLNPQKYPADIQRAINETHQGRYQVNTMYQALGW
DC-73WP_001211219.1_1013AbBCP     LDAP.....AYSVKSTLPDMLSFIHANLNPQKYPADIQRAINETHQGRYQVNTMYQALGW
ADC-73BDE18748.1_1038AbBCP       LDAP.....AYSVKSTLPDMLSFIHANLNPQKYPADIQRAINETHQGRYQVNTMYQALGW
DC-115WP_001211215.1_2833AbOC     LDAP.....AYGVKSTLPDMLSFIHANLNPQKYPADIQRAINETHQGRYQVNTMYQALGW
C-115WP_001211215.1_1156AbBOC     LDAP.....AYGVKSTLPDMLSFIHANLNPQKYPADIQRAINETHQGRYQVNTMYQALGW
C-115WP_001211215.1_1156AbBCP     LDAP.....AYGVKSTLPDMLSFIHANLNPQKYPADIQRAINETHQGRYQVNTMYQALGW
ADC-5WP_038405930.1_2542AbOC      LDAP.....AYGVKSTLPDMLSFIHANLTPQKYPTDIQRAINETHQGFYQVGTMYQALGW
ADC-5WP_038405930.1_1113AbBOC     LDAP.....AYGVKSTLPDMLSFIHANLTPQKYPTDIQRAINETHQGFYQVGTMYQALGW
ADC-5WP_038405930.1_1113AbBCP     LDAP.....AYGVKSTLPDMLSFIHANLTPQKYPTDIQRAINETHQGFYQVGTMYQALGW
C-236WP_176673601.1_1026AbBOC     LDAP.....AYGVKSTLPDMLKFIHANLNPQKYPTDIQRAINETHQGRYQVNTMYQALGW
C-236WP_176673601.1_1026AbBCP     LDAP.....AYGVKSTLPDMLKFIHANLNPQKYPTDIQRAINETHQGRYQVNTMYQALGW
DC-217WP_060470109.1_3584AbOC     LDAP.....AYGVKSTLPDMLSFIHANLNPQKYPADIQRAINETHQGRYQENTMYQALGW
ADC-217USX66614.1_2290AbBOC      LDAP.....AYGVKSTLPDMLSFIHANLNPQKYPADIQRAINETHQGRYQENTMYQALGW
ADC-217USX66614.1_2290AbBCP      LDAP.....AYGVKSTLPDMLSFIHANLNPQKYPADIQRAINETHQGRYQENTMYQALGW
ADC-247WP_032058755.1_382AbOC     LDAP.....AYGVKSTLPDMLSFIYANLNPQKYPADIQRAINETHQGRYQVNTMYQALGW
C-247WP_032058755.1_1936AbBOC     LDAP.....AYGVKSTLPDMLSFIYANLNPQKYPADIQRAINETHQGRYQVNTMYQALGW
C-247WP_032058755.1_1936AbBCP     LDAP.....AYGVKSTLPDMLSFIYANLNPQKYPADIQRAINETHQGRYQVNTMYQALGW
DC-203WP_032000414.1_2940AbOC     LDAP.....AYGVKSTLPDMLSFIHANLNPQKYPADIQRAINETHQGRYQVNTMYQALGW
C-203WP_032000414.1_1062AbBOC     LDAP.....AYGVKSTLPDMLSFIHANLNPQKYPADIQRAINETHQGRYQVNTMYQALGW
DC-203WP_032000414.1_129AbBOC     LDAP.....AYGVKSTLPDMLSFIHANLNPQKYPADIQRAINETHQGRYQVNTMYQALGW
C-203WP_032000414.1_2518AbBOC     LDAP.....AYGVKSTLPDMLSFIHANLNPQKYPADIQRAINETHQGRYQVNTMYQALGW
C-203WP_032000414.1_1062AbBCP     LDAP.....AYGVKSTLPDMLSFIHANLNPQKYPADIQRAINETHQGRYQVNTMYQALGW
DC-203WP_032000414.1_129AbBCP     LDAP.....AYGVKSTLPDMLSFIHANLNPQKYPADIQRAINETHQGRYQVNTMYQALGW
C-203WP_032000414.1_2518AbBCP     LDAP.....AYGVKSTLPDMLSFIHANLNPQKYPADIQRAINETHQGRYQVNTMYQALGW
DC-152WP_001211233.1_3734AbOC     LDAP.....AYGVKSTLPDMLKFIHANLNPQKYPTDIQRAINETHQGRYQVNTMYQALGW
C-152WP_001211233.1_1080AbBOC     LDAP.....AYGVKSTLPDMLKFIHANLNPQKYPTDIQRAINETHQGRYQVNTMYQALGW
DC-152WP_001211233.1_491AbBOC     LDAP.....AYGVKSTLPDMLKFIHANLNPQKYPTDIQRAINETHQGRYQVNTMYQALGW
ADC-152WP_001211233.1_46AbBOC     LDAP.....AYGVKSTLPDMLKFIHANLNPQKYPTDIQRAINETHQGRYQVNTMYQALGW
C-152WP_001211233.1_1545AbBOC     LDAP.....AYGVKSTLPDMLKFIHANLNPQKYPTDIQRAINETHQGRYQVNTMYQALGW
ADC-152WBS86980.1_1289AbBOC      LDAP.....AYGVKSTLPDMLKFIHANLNPQKYPTDIQRAINETHQGRYQVNTMYQALGW
ADC-152WBS81072.1_2581AbBOC      LDAP.....AYGVKSTLPDMLKFIHANLNPQKYPTDIQRAINETHQGRYQVNTMYQALGW
ADC-152WBS88504.1_2711AbBOC      LDAP.....AYGVKSTLPDMLKFIHANLNPQKYPTDIQRAINETHQGRYQVNTMYQALGW
ADC-152WBS92168.1_2401AbBOC      LDAP.....AYGVKSTLPDMLKFIHANLNPQKYPTDIQRAINETHQGRYQVNTMYQALGW
ADC-152WBS79617.1_1289AbBOC      LDAP.....AYGVKSTLPDMLKFIHANLNPQKYPTDIQRAINETHQGRYQVNTMYQALGW
C-152WP_001211233.1_1080AbBCP     LDAP.....AYGVKSTLPDMLKFIHANLNPQKYPTDIQRAINETHQGRYQVNTMYQALGW
DC-152WP_001211233.1_491AbBCP     LDAP.....AYGVKSTLPDMLKFIHANLNPQKYPTDIQRAINETHQGRYQVNTMYQALGW
ADC-152WP_001211233.1_46AbBCP     LDAP.....AYGVKSTLPDMLKFIHANLNPQKYPTDIQRAINETHQGRYQVNTMYQALGW
C-152WP_001211233.1_1545AbBCP     LDAP.....AYGVKSTLPDMLKFIHANLNPQKYPTDIQRAINETHQGRYQVNTMYQALGW
ADC-152WBS86980.1_1289AbBCP      LDAP.....AYGVKSTLPDMLKFIHANLNPQKYPTDIQRAINETHQGRYQVNTMYQALGW
ADC-152WBS81072.1_2581AbBCP      LDAP.....AYGVKSTLPDMLKFIHANLNPQKYPTDIQRAINETHQGRYQVNTMYQALGW
ADC-152WBS88504.1_2711AbBCP      LDAP.....AYGVKSTLPDMLKFIHANLNPQKYPTDIQRAINETHQGRYQVNTMYQALGW
ADC-152WBS92168.1_2401AbBCP      LDAP.....AYGVKSTLPDMLKFIHANLNPQKYPTDIQRAINETHQGRYQVNTMYQALGW
ADC-152WBS79617.1_1289AbBCP      LDAP.....AYGVKSTLPDMLKFIHANLNPQKYPTDIQRAINETHQGRYQVNTMYQALGW
ADC-52WP_001211232.1_990AbOC      LDAP.....AYGVKSTLPDMLKFIHANLNPQKYPTDIQRAINETHQGRYQVNTMYQALGW
DC-52WP_001211232.1_1087AbBOC     LDAP.....AYGVKSTLPDMLKFIHANLNPQKYPTDIQRAINETHQGRYQVNTMYQALGW
DC-52WP_001211232.1_1087AbBCP     LDAP.....AYGVKSTLPDMLKFIHANLNPQKYPTDIQRAINETHQGRYQVNTMYQALGW
C-163WP_057093064.1_2397AbBOC     LDAP.....AYGVKSTLPDMLSFIHANFNPQKYPADIQRAINETHQGRYQVNTMYQALGW
C-163WP_057093064.1_2397AbBCP     LDAP.....AYGVKSTLPDMLSFIHANFNPQKYPADIQRAINETHQGRYQVNTMYQALGW
ADC-33WP_001211220.1_2277AbOC     LDAA....PAYGVKSTLPDMLSFIHANLNPQKYPADIQRAINETHQGRYQVNTMYQALGW
DC-33WP_001211220.1_1044AbBOC     LDAA....PAYGVKSTLPDMLSFIHANLNPQKYPADIQRAINETHQGRYQVNTMYQALGW
DC-33WP_001211220.1_2452AbBOC     LDAA....PAYGVKSTLPDMLSFIHANLNPQKYPADIQRAINETHQGRYQVNTMYQALGW
DC-33WP_001211220.1_1046AbBOC     LDAA....PAYGVKSTLPDMLSFIHANLNPQKYPADIQRAINETHQGRYQVNTMYQALGW
DC-33WP_001211220.1_1025AbBOC     LDAA....PAYGVKSTLPDMLSFIHANLNPQKYPADIQRAINETHQGRYQVNTMYQALGW
DC-33WP_001211220.1_1026AbBOC     LDAA....PAYGVKSTLPDMLSFIHANLNPQKYPADIQRAINETHQGRYQVNTMYQALGW
DC-33WP_001211220.1_1033AbBOC     LDAA....PAYGVKSTLPDMLSFIHANLNPQKYPADIQRAINETHQGRYQVNTMYQALGW
DC-33WP_001211220.1_1035AbBOC     LDAA....PAYGVKSTLPDMLSFIHANLNPQKYPADIQRAINETHQGRYQVNTMYQALGW
DC-33WP_001211220.1_1041AbBOC     LDAA....PAYGVKSTLPDMLSFIHANLNPQKYPADIQRAINETHQGRYQVNTMYQALGW
DC-33WP_001211220.1_1044AbBCP     LDAA....PAYGVKSTLPDMLSFIHANLNPQKYPADIQRAINETHQGRYQVNTMYQALGW
DC-33WP_001211220.1_2452AbBCP     LDAA....PAYGVKSTLPDMLSFIHANLNPQKYPADIQRAINETHQGRYQVNTMYQALGW
DC-33WP_001211220.1_1046AbBCP     LDAA....PAYGVKSTLPDMLSFIHANLNPQKYPADIQRAINETHQGRYQVNTMYQALGW
DC-33WP_001211220.1_1025AbBCP     LDAA....PAYGVKSTLPDMLSFIHANLNPQKYPADIQRAINETHQGRYQVNTMYQALGW
DC-33WP_001211220.1_1026AbBCP     LDAA....PAYGVKSTLPDMLSFIHANLNPQKYPADIQRAINETHQGRYQVNTMYQALGW
DC-33WP_001211220.1_1033AbBCP     LDAA....PAYGVKSTLPDMLSFIHANLNPQKYPADIQRAINETHQGRYQVNTMYQALGW
DC-33WP_001211220.1_1035AbBCP     LDAA....PAYGVKSTLPDMLSFIHANLNPQKYPADIQRAINETHQGRYQVNTMYQALGW
DC-33WP_001211220.1_1041AbBCP     LDAA....PAYGVKSTLPDMLSFIHANLNPQKYPADIQRAINETHQGRYQVNTMYQALGW
C-212WP_031975357.1_1297AbBOC     LDAL....AAYGVKSTLPDMLSFIHANLNPQKYPADIQRAINETHQGRYQVNTMYQALGW
DC-212WP_031975357.1_501AbBOC     LDAL....AAYGVKSTLPDMLSFIHANLNPQKYPADIQRAINETHQGRYQVNTMYQALGW
C-212WP_031975357.1_1297AbBCP     LDAL....AAYGVKSTLPDMLSFIHANLNPQKYPADIQRAINETHQGRYQVNTMYQALGW
DC-212WP_031975357.1_501AbBCP     LDAL....AAYGVKSTLPDMLSFIHANLNPQKYPADIQRAINETHQGRYQVNTMYQALGW
C-182WP_057691114.1_1055AbBOC     LDAPA..SPAYGVKSTLPDMLSFIHANLTPQKYPTDIQRAINETHQGFYQVGTMYQALGW
C-182WP_057691114.1_1055AbBCP     LDAPA..SPAYGVKSTLPDMLSFIHANLTPQKYPTDIQRAINETHQGFYQVGTMYQALGW
DC-81WP_059262723.1_1208AbBOC     LDAPAYGAPAYGVKSTLPDMLSFIHANLNPQKYPADIQRAINETHQGFYQVNTMYQALGW
DC-81WP_059262723.1_2841AbBOC     LDAPAYGAPAYGVKSTLPDMLSFIHANLNPQKYPADIQRAINETHQGFYQVNTMYQALGW
DC-81WP_059262723.1_1208AbBCP     LDAPAYGAPAYGVKSTLPDMLSFIHANLNPQKYPADIQRAINETHQGFYQVNTMYQALGW
DC-81WP_059262723.1_2841AbBCP     LDAPAYGAPAYGVKSTLPDMLSFIHANLNPQKYPADIQRAINETHQGFYQVNTMYQALGW
ADC-176WP_001159761.1_11AbOC      LDAPAYGVPAYGVKSTLPDMLSFIHANLNPQKYPADIQRAINETHQGFYQVNTMYQALGW
DC-176WP_001159761.1_3466AbOC     LDAPAYGVPAYGVKSTLPDMLSFIHANLNPQKYPADIQRAINETHQGFYQVNTMYQALGW
ADC-176WP_001159761.1_93AbBOP     LDAPAYGVPAYGVKSTLPDMLSFIHANLNPQKYPADIQRAINETHQGFYQVNTMYQALGW
DC-176WP_001159761.1_247AbBOC     LDAPAYGVPAYGVKSTLPDMLSFIHANLNPQKYPADIQRAINETHQGFYQVNTMYQALGW
ADC-176WP_001159761.1_9AbBOC      LDAPAYGVPAYGVKSTLPDMLSFIHANLNPQKYPADIQRAINETHQGFYQVNTMYQALGW
C-176WP_001159761.1_2982AbBOC     LDAPAYGVPAYGVKSTLPDMLSFIHANLNPQKYPADIQRAINETHQGFYQVNTMYQALGW
ADC-176QLG80706.1_1971AbBOC      LDAPAYGVPAYGVKSTLPDMLSFIHANLNPQKYPADIQRAINETHQGFYQVNTMYQALGW
C-176WP_001159761.1_2851AbBOC     LDAPAYGVPAYGVKSTLPDMLSFIHANLNPQKYPADIQRAINETHQGFYQVNTMYQALGW
C-176WP_001159761.1_1220AbBOC     LDAPAYGVPAYGVKSTLPDMLSFIHANLNPQKYPADIQRAINETHQGFYQVNTMYQALGW
DC-176WP_001159761.1_386AbBOC     LDAPAYGVPAYGVKSTLPDMLSFIHANLNPQKYPADIQRAINETHQGFYQVNTMYQALGW
ADC-176WP_001159761.1_11AbBOC     LDAPAYGVPAYGVKSTLPDMLSFIHANLNPQKYPADIQRAINETHQGFYQVNTMYQALGW
DC-176WP_001159761.1_247AbBCP     LDAPAYGVPAYGVKSTLPDMLSFIHANLNPQKYPADIQRAINETHQGFYQVNTMYQALGW
ADC-176WP_001159761.1_9AbBCP      LDAPAYGVPAYGVKSTLPDMLSFIHANLNPQKYPADIQRAINETHQGFYQVNTMYQALGW
C-176WP_001159761.1_2982AbBCP     LDAPAYGVPAYGVKSTLPDMLSFIHANLNPQKYPADIQRAINETHQGFYQVNTMYQALGW
ADC-176QLG80706.1_1971AbBCP      LDAPAYGVPAYGVKSTLPDMLSFIHANLNPQKYPADIQRAINETHQGFYQVNTMYQALGW
C-176WP_001159761.1_2851AbBCP     LDAPAYGVPAYGVKSTLPDMLSFIHANLNPQKYPADIQRAINETHQGFYQVNTMYQALGW
C-176WP_001159761.1_1220AbBCP     LDAPAYGVPAYGVKSTLPDMLSFIHANLNPQKYPADIQRAINETHQGFYQVNTMYQALGW
DC-176WP_001159761.1_386AbBCP     LDAPAYGVPAYGVKSTLPDMLSFIHANLNPQKYPADIQRAINETHQGFYQVNTMYQALGW
ADC-176WP_001159761.1_11AbBCP     LDAPAYGVPAYGVKSTLPDMLSFIHANLNPQKYPADIQRAINETHQGFYQVNTMYQALGW
ADC-199WP_114166683.1_169AbOC     LDAPAYGVPAYGVKSTLPDMLSFIHANLNPQKYPADIQRAINETHQGFYQVNTMYQALGW
DC-199WP_114166683.1_267AbBOC     LDAPAYGVPAYGVKSTLPDMLSFIHANLNPQKYPADIQRAINETHQGFYQVNTMYQALGW
DC-199WP_114166683.1_267AbBCP     LDAPAYGVPAYGVKSTLPDMLSFIHANLNPQKYPADIQRAINETHQGFYQVNTMYQALGW
                             acc
```

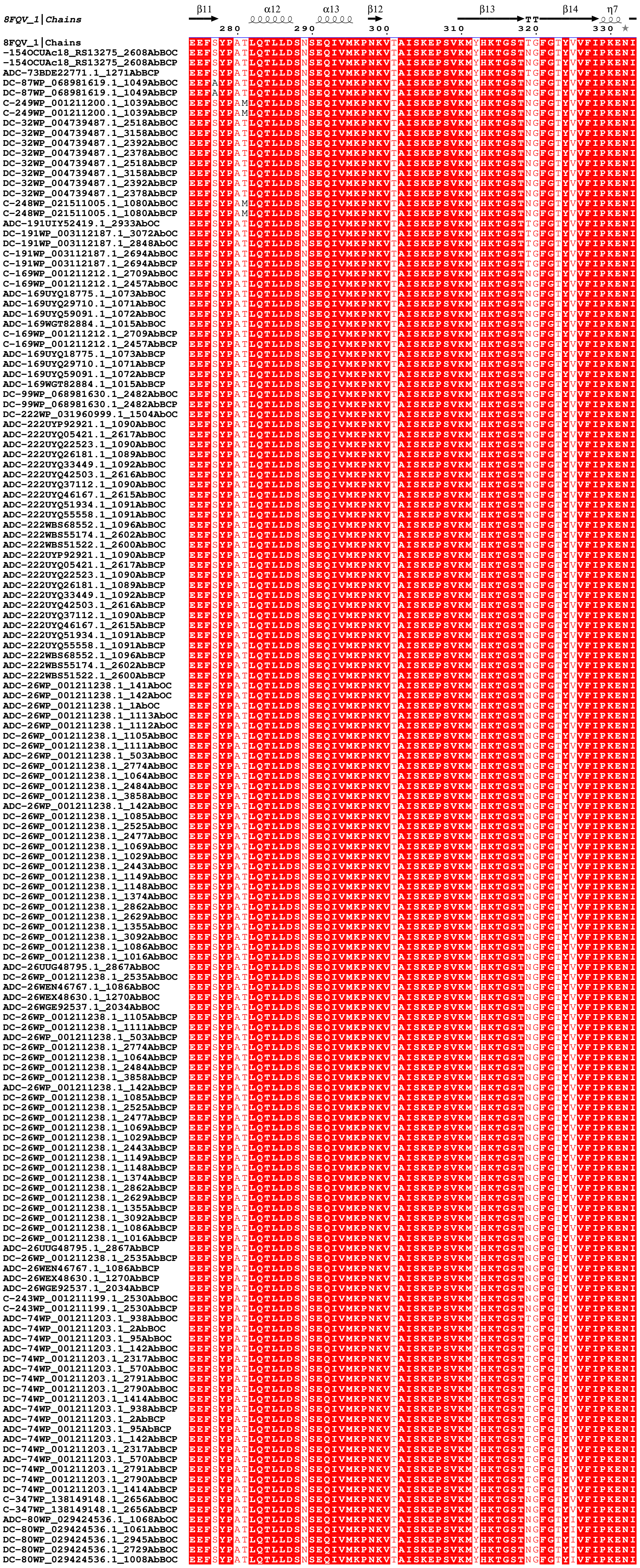
8FQV_1|Chains
β11
α12
α13
β12
β13
β14
η7
TT
280
290
300
310
320
330

```
DC-80WP_029424536.1_1061AbBCP   EEFSYPATLQTLLDSNSEQIVMKPNKVTAISKEPSVKMYHKTGSTNGFGTYIVFIPKENI
DC-80WP_029424536.1_2945AbBCP   EEFSYPATLQTLLDSNSEQIVMKPNKVTAISKEPSVKMYHKTGSTNGFGTYIVFIPKENI
DC-80WP_029424536.1_2729AbBCP   EEFSYPATLQTLLDSNSEQIVMKPNKVTAISKEPSVKMYHKTGSTNGFGTYIVFIPKENI
DC-80WP_029424536.1_1008AbBCP   EEFSYPATLQTLLDSNSEQIVMKPNKVTAISKEPSVKMYHKTGSTNGFGTYIVFIPKENI
DC-156WP_024436624.1_2681AbOC   EEFSYPATLQTLLDSNSEQIVMKPNKVTAISKEPSVKMYHKTGSTNGFGTYVVFIPKENI
C-156WP_024436624.1_2375AbBOC   EEFSYPATLQTLLDSNSEQIVMKPNKVTAISKEPSVKMYHKTGSTNGFGTYVVFIPKENI
C-156WP_024436624.1_1038AbBOC   EEFSYPATLQTLLDSNSEQIVMKPNKVTAISKEPSVKMYHKTGSTNGFGTYVVFIPKENI
C-156WP_024436624.1_1041AbBOC   EEFSYPATLQTLLDSNSEQIVMKPNKVTAISKEPSVKMYHKTGSTNGFGTYVVFIPKENI
C-156WP_024436624.1_1124AbBOC   EEFSYPATLQTLLDSNSEQIVMKPNKVTAISKEPSVKMYHKTGSTNGFGTYVVFIPKENI
C-156WP_024436624.1_1126AbBOC   EEFSYPATLQTLLDSNSEQIVMKPNKVTAISKEPSVKMYHKTGSTNGFGTYVVFIPKENI
C-156WP_024436624.1_2375AbBCP   EEFSYPATLQTLLDSNSEQIVMKPNKVTAISKEPSVKMYHKTGSTNGFGTYVVFIPKENI
C-156WP_024436624.1_1038AbBCP   EEFSYPATLQTLLDSNSEQIVMKPNKVTAISKEPSVKMYHKTGSTNGFGTYVVFIPKENI
C-156WP_024436624.1_1041AbBCP   EEFSYPATLQTLLDSNSEQIVMKPNKVTAISKEPSVKMYHKTGSTNGFGTYVVFIPKENI
C-156WP_024436624.1_1124AbBCP   EEFSYPATLQTLLDSNSEQIVMKPNKVTAISKEPSVKMYHKTGSTNGFGTYVVFIPKENI
C-156WP_024436624.1_1126AbBCP   EEFSYPATLQTLLDSNSEQIVMKPNKVTAISKEPSVKMYHKTGSTNGFGTYVVFIPKENI
C-165WP_001211239.1_1734AbBOC   EEFSYPATLQTLLDSNSEQIVMKPNKVTAISKEPSVKMYHKTGSTNGFGTYVVFIPKENI
C-165WP_001211239.1_1032AbBOC   EEFSYPATLQTLLDSNSEQIVMKPNKVTAISKEPSVKMYHKTGSTNGFGTYVVFIPKENI
C-165WP_001211239.1_1734AbBCP   EEFSYPATLQTLLDSNSEQIVMKPNKVTAISKEPSVKMYHKTGSTNGFGTYVVFIPKENI
C-165WP_001211239.1_1032AbBCP   EEFSYPATLQTLLDSNSEQIVMKPNKVTAISKEPSVKMYHKTGSTNGFGTYVVFIPKENI
C-263WP_109433738.1_2026AbBOC   EEFSYPAMLQTLLDSNSEQIVMKPNKVTAISKEPSVKMYHKTGSTTGFGTYVVFIPKENI
DC-263WP_109433738.1_954AbBOC   EEFSYPAMLQTLLDSNSEQIVMKPNKVTAISKEPSVKMYHKTGSTTGFGTYVVFIPKENI
C-263WP_109433738.1_1022AbBOC   EEFSYPAMLQTLLDSNSEQIVMKPNKVTAISKEPSVKMYHKTGSTTGFGTYVVFIPKENI
C-263WP_109433738.1_3219AbBOC   EEFSYPAMLQTLLDSNSEQIVMKPNKVTAISKEPSVKMYHKTGSTTGFGTYVVFIPKENI
C-263WP_109433738.1_2026AbBCP   EEFSYPAMLQTLLDSNSEQIVMKPNKVTAISKEPSVKMYHKTGSTTGFGTYVVFIPKENI
DC-263WP_109433738.1_954AbBCP   EEFSYPAMLQTLLDSNSEQIVMKPNKVTAISKEPSVKMYHKTGSTTGFGTYVVFIPKENI
C-263WP_109433738.1_1022AbBCP   EEFSYPAMLQTLLDSNSEQIVMKPNKVTAISKEPSVKMYHKTGSTTGFGTYVVFIPKENI
C-263WP_109433738.1_3219AbBCP   EEFSYPAMLQTLLDSNSEQIVMKPNKVTAISKEPSVKMYHKTGSTTGFGTYVVFIPKENI
DC-79WP_001159760.1_2471AbBOC   EEFSYPATLQTLLDSNSEQIVMKPNKVTAISKEPSVKMYHKTGSTNGFGTYVVFIPKENI
ADC-79WP_001159760.1_670AbBOC   EEFSYPATLQTLLDSNSEQIVMKPNKVTAISKEPSVKMYHKTGSTNGFGTYVVFIPKENI
DC-79WP_001159760.1_2535AbBOC   EEFSYPATLQTLLDSNSEQIVMKPNKVTAISKEPSVKMYHKTGSTNGFGTYVVFIPKENI
DC-79WP_001159760.1_1080AbBOC   EEFSYPATLQTLLDSNSEQIVMKPNKVTAISKEPSVKMYHKTGSTNGFGTYVVFIPKENI
DC-79WP_001159760.1_2471AbBCP   EEFSYPATLQTLLDSNSEQIVMKPNKVTAISKEPSVKMYHKTGSTNGFGTYVVFIPKENI
ADC-79WP_001159760.1_670AbBCP   EEFSYPATLQTLLDSNSEQIVMKPNKVTAISKEPSVKMYHKTGSTNGFGTYVVFIPKENI
DC-79WP_001159760.1_2535AbBCP   EEFSYPATLQTLLDSNSEQIVMKPNKVTAISKEPSVKMYHKTGSTNGFGTYVVFIPKENI
DC-79WP_001159760.1_1080AbBCP   EEFSYPATLQTLLDSNSEQIVMKPNKVTAISKEPSVKMYHKTGSTNGFGTYVVFIPKENI
ADC-6WP_017725267.1_1459AbBOC   EEFSYPAMLQTLLDSNSEQIVMKPNKVTAISKEPSVKMYHKTGSTNGFGTYVVFIPKENI
ADC-6WP_017725267.1_1678AbBOC   EEFSYPAMLQTLLDSNSEQIVMKPNKVTAISKEPSVKMYHKTGSTNGFGTYVVFIPKENI
ADC-6WP_017725267.1_2561AbBOC   EEFSYPAMLQTLLDSNSEQIVMKPNKVTAISKEPSVKMYHKTGSTNGFGTYVVFIPKENI
ADC-6WP_017725267.1_1459AbBCP   EEFSYPAMLQTLLDSNSEQIVMKPNKVTAISKEPSVKMYHKTGSTNGFGTYVVFIPKENI
ADC-6WP_017725267.1_1678AbBCP   EEFSYPAMLQTLLDSNSEQIVMKPNKVTAISKEPSVKMYHKTGSTNGFGTYVVFIPKENI
ADC-6WP_017725267.1_2561AbBCP   EEFSYPAMLQTLLDSNSEQIVMKPNKVTAISKEPSVKMYHKTGSTNGFGTYVVFIPKENI
ADC-1WP_004714775.1_3726AbBOC   EEFSYPATLQTLLDSNSEQIVMKPNKVTAISKEPSVKMYHKTGSTNRFGTYVVFIPKENI
ADC-1WP_004714775.1_3726AbBCP   EEFSYPATLQTLLDSNSEQIVMKPNKVTAISKEPSVKMYHKTGSTNRFGTYVVFIPKENI
C-181WP_046693238.1_1083AbBOC   EEFSYPATLQTLLDSNSEQIVMKPNKVTAISKEPSVKMYHKTGSTNRFGTYVVFIPKENI
C-181WP_046693238.1_1083AbBCP   EEFSYPATLQTLLDSNSEQIVMKPNKVTAISKEPSVKMYHKTGSTNRFGTYVVFIPKENI
C-238WP_002058891.1_2458AbBOC   EEFSYPATLQTLLDSNSEQIVMKPNKVTAISKEPSVKMYHKTGSTNGFGTYVVFIPKENI
C-238WP_002058891.1_2458AbBCP   EEFSYPATLQTLLDSNSEQIVMKPNKVTAISKEPSVKMYHKTGSTNGFGTYVVFIPKENI
C-186WP_072292271.1_1675AbBOC   EEFSYPATLQTLLDSNSEQIVMKPNKVTAISKEPSVKMYHKTGSTSGFGTYVVFIPKENI
C-186WP_072292271.1_1675AbBCP   EEFSYPATLQTLLDSNSEQIVMKPNKVTAISKEPSVKMYHKTGSTSGFGTYVVFIPKENI
DC-184WP_059247009.1_1188AbOC   EEFSYPATLQTLLDSNSEQIVMKPNKVTAISKEPSVKMYHKTGSTNGFGTYIVFIPKENI
ADC-328WP_069370341.1_666AbOC   EEFSYPATLQTLLDSNSEQIVMKPNKVTAISKEPSVKMYHKTGSTNGFGTYVVFIPKENI
DC-56WP_031973850.1_1911AbBOC   EEFSYPATLQTLLDSNSEQIVMKPNKVTAISKEPSVKMYHKTGSTTGFGTYVVFIPKENI
DC-56WP_031973850.1_2693AbBOC   EEFSYPATLQTLLDSNSEQIVMKPNKVTAISKEPSVKMYHKTGSTTGFGTYVVFIPKENI
DC-56WP_031973850.1_1911AbBCP   EEFSYPATLQTLLDSNSEQIVMKPNKVTAISKEPSVKMYHKTGSTTGFGTYVVFIPKENI
DC-56WP_031973850.1_2693AbBCP   EEFSYPATLQTLLDSNSEQIVMKPNKVTAISKEPSVKMYHKTGSTTGFGTYVVFIPKENI
DC-117WP_002157727.1_394AbBOC   EEFSYPATLQTLLDSNSEQIVMKPNKVTAISKEPSVKMYHKTGSTNGFGTYVVFIPKENI
DC-117WP_002157727.1_394AbBCP   EEFSYPATLQTLLDSNSEQIVMKPNKVTAISKEPSVKMYHKTGSTNGFGTYVVFIPKENI
ADC-25WP_001211217.1_2432AbOC   EEFSYPATLQTLLDSNSEQIVMKPNKVTAISKEPSVKMYHKTGSTNGFGTYVVFIPKENI
ADC-25WP_001211217.1_1025AbOC   EEFSYPATLQTLLDSNSEQIVMKPNKVTAISKEPSVKMYHKTGSTNGFGTYVVFIPKENI
ADC-25WP_001211217.1_3391AbOC   EEFSYPATLQTLLDSNSEQIVMKPNKVTAISKEPSVKMYHKTGSTNGFGTYVVFIPKENI
ADC-25WP_001211217.1_1042AbOC   EEFSYPATLQTLLDSNSEQIVMKPNKVTAISKEPSVKMYHKTGSTNGFGTYVVFIPKENI
ADC-25WP_001211217.1_1185AbOC   EEFSYPATLQTLLDSNSEQIVMKPNKVTAISKEPSVKMYHKTGSTNGFGTYVVFIPKENI
ADC-25WP_001211217.1_768AbOC    EEFSYPATLQTLLDSNSEQIVMKPNKVTAISKEPSVKMYHKTGSTNGFGTYVVFIPKENI
ADC-25WP_001211217.1_2605AbOC   EEFSYPATLQTLLDSNSEQIVMKPNKVTAISKEPSVKMYHKTGSTNGFGTYVVFIPKENI
DC-25WP_001211217.1_1117AbBOC   EEFSYPATLQTLLDSNSEQIVMKPNKVTAISKEPSVKMYHKTGSTNGFGTYVVFIPKENI
DC-25WP_001211217.1_1090AbBOC   EEFSYPATLQTLLDSNSEQIVMKPNKVTAISKEPSVKMYHKTGSTNGFGTYVVFIPKENI
DC-25WP_001211217.1_1089AbBOC   EEFSYPATLQTLLDSNSEQIVMKPNKVTAISKEPSVKMYHKTGSTNGFGTYVVFIPKENI
DC-25WP_001211217.1_1091AbBOC   EEFSYPATLQTLLDSNSEQIVMKPNKVTAISKEPSVKMYHKTGSTNGFGTYVVFIPKENI
DC-25WP_001211217.1_1604AbBOC   EEFSYPATLQTLLDSNSEQIVMKPNKVTAISKEPSVKMYHKTGSTNGFGTYVVFIPKENI
DC-25WP_001211217.1_1040AbBOC   EEFSYPATLQTLLDSNSEQIVMKPNKVTAISKEPSVKMYHKTGSTNGFGTYVVFIPKENI
ADC-25UYQ12932.1_2634AbBOC      EEFSYPATLQTLLDSNSEQIVMKPNKVTAISKEPSVKMYHKTGSTNGFGTYVVFIPKENI
ADC-25UYQ09083.1_2628AbBOC      EEFSYPATLQTLLDSNSEQIVMKPNKVTAISKEPSVKMYHKTGSTNGFGTYVVFIPKENI
ADC-25UYQ40822.1_1120AbBOC      EEFSYPATLQTLLDSNSEQIVMKPNKVTAISKEPSVKMYHKTGSTNGFGTYVVFIPKENI
DC-25WP_001211217.1_1117AbBCP   EEFSYPATLQTLLDSNSEQIVMKPNKVTAISKEPSVKMYHKTGSTNGFGTYVVFIPKENI
DC-25WP_001211217.1_1090AbBCP   EEFSYPATLQTLLDSNSEQIVMKPNKVTAISKEPSVKMYHKTGSTNGFGTYVVFIPKENI
DC-25WP_001211217.1_1089AbBCP   EEFSYPATLQTLLDSNSEQIVMKPNKVTAISKEPSVKMYHKTGSTNGFGTYVVFIPKENI
DC-25WP_001211217.1_1091AbBCP   EEFSYPATLQTLLDSNSEQIVMKPNKVTAISKEPSVKMYHKTGSTNGFGTYVVFIPKENI
DC-25WP_001211217.1_1604AbBCP   EEFSYPATLQTLLDSNSEQIVMKPNKVTAISKEPSVKMYHKTGSTNGFGTYVVFIPKENI
DC-25WP_001211217.1_1040AbBCP   EEFSYPATLQTLLDSNSEQIVMKPNKVTAISKEPSVKMYHKTGSTNGFGTYVVFIPKENI
ADC-25UYQ12932.1_2634AbBCP      EEFSYPATLQTLLDSNSEQIVMKPNKVTAISKEPSVKMYHKTGSTNGFGTYVVFIPKENI
ADC-25UYQ09083.1_2628AbBCP      EEFSYPATLQTLLDSNSEQIVMKPNKVTAISKEPSVKMYHKTGSTNGFGTYVVFIPKENI
ADC-25UYQ40822.1_1120AbBCP      EEFSYPATLQTLLDSNSEQIVMKPNKVTAISKEPSVKMYHKTGSTNGFGTYVVFIPKENI
C-268WP_105922788.1_2839AbBOC   EEFSYPATLQTLLDSNSEQIVMKPNKVTAISKEPSVKMYHKTGSTTGFGTYVVFIPKENI
ADC-268WDC47378.1_2492AbBOC     EEFSYPATLQTLLDSNSEQIVMKPNKVTAISKEPSVKMYHKTGSTTGFGTYVVFIPKENI
C-268WP_105922788.1_2839AbBCP   EEFSYPATLQTLLDSNSEQIVMKPNKVTAISKEPSVKMYHKTGSTTGFGTYVVFIPKENI
ADC-268WDC47378.1_2492AbBCP     EEFSYPATLQTLLDSNSEQIVMKPNKVTAISKEPSVKMYHKTGSTTGFGTYVVFIPKENI
DC-155WP_005128228.1_2591AbOC   EEFSYPATLQTLLDSNSEQIVMKPNKVTAISKEPSVKMYHKTGSTNGFGTYVVFIPKENI
C-155WP_005128228.1_2549AbBOC   EEFSYPATLQTLLDSNSEQIVMKPNKVTAISKEPSVKMYHKTGSTNGFGTYVVFIPKENI
C-155WP_005128228.1_2480AbBOC   EEFSYPATLQTLLDSNSEQIVMKPNKVTAISKEPSVKMYHKTGSTNGFGTYVVFIPKENI
C-155WP_005128228.1_2455AbBOC   EEFSYPATLQTLLDSNSEQIVMKPNKVTAISKEPSVKMYHKTGSTNGFGTYVVFIPKENI
C-155WP_005128228.1_2549AbBCP   EEFSYPATLQTLLDSNSEQIVMKPNKVTAISKEPSVKMYHKTGSTNGFGTYVVFIPKENI
C-155WP_005128228.1_2480AbBCP   EEFSYPATLQTLLDSNSEQIVMKPNKVTAISKEPSVKMYHKTGSTNGFGTYVVFIPKENI
C-155WP_005128228.1_2455AbBCP   EEFSYPATLQTLLDSNSEQIVMKPNKVTAISKEPSVKMYHKTGSTNGFGTYVVFIPKENI
ADC-76WP_001211237.1_2862AbOC   EEFSYPATLQTLLDSNSEQIVMKPNKVTAISKEPSVKMYHKTGSTNGFGTYVVFIPKENI
ADC-76WP_001211237.1_3431AbOC   EEFSYPATLQTLLDSNSEQIVMKPNKVTAISKEPSVKMYHKTGSTNGFGTYVVFIPKENI
ADC-76WP_001211237.1_2696AbOC   EEFSYPATLQTLLDSNSEQIVMKPNKVTAISKEPSVKMYHKTGSTNGFGTYVVFIPKENI
ADC-76WP_001211237.1_2890AbOC   EEFSYPATLQTLLDSNSEQIVMKPNKVTAISKEPSVKMYHKTGSTNGFGTYVVFIPKENI
DC-76WP_001211237.1_2723AbBOC   EEFSYPATLQTLLDSNSEQIVMKPNKVTAISKEPSVKMYHKTGSTNGFGTYVVFIPKENI
DC-76WP_001211237.1_1033AbBOC   EEFSYPATLQTLLDSNSEQIVMKPNKVTAISKEPSVKMYHKTGSTNGFGTYVVFIPKENI
ADC-76WP_001211237.1_526AbBOC   EEFSYPATLQTLLDSNSEQIVMKPNKVTAISKEPSVKMYHKTGSTNGFGTYVVFIPKENI
DC-76WP_001211237.1_1046AbBOC   EEFSYPATLQTLLDSNSEQIVMKPNKVTAISKEPSVKMYHKTGSTNGFGTYVVFIPKENI
DC-76WP_001211237.1_1040AbBOC   EEFSYPATLQTLLDSNSEQIVMKPNKVTAISKEPSVKMYHKTGSTNGFGTYVVFIPKENI
DC-76WP_001211237.1_3682AbBOC   EEFSYPATLQTLLDSNSEQIVMKPNKVTAISKEPSVKMYHKTGSTNGFGTYVVFIPKENI
DC-76WP_001211237.1_2579AbBOC   EEFSYPATLQTLLDSNSEQIVMKPNKVTAISKEPSVKMYHKTGSTNGFGTYVVFIPKENI
DC-76WP_001211237.1_2723AbBCP   EEFSYPATLQTLLDSNSEQIVMKPNKVTAISKEPSVKMYHKTGSTNGFGTYVVFIPKENI
DC-76WP_001211237.1_1033AbBCP   EEFSYPATLQTLLDSNSEQIVMKPNKVTAISKEPSVKMYHKTGSTNGFGTYVVFIPKENI
ADC-76WP_001211237.1_526AbBCP   EEFSYPATLQTLLDSNSEQIVMKPNKVTAISKEPSVKMYHKTGSTNGFGTYVVFIPKENI
DC-76WP_001211237.1_1046AbBCP   EEFSYPATLQTLLDSNSEQIVMKPNKVTAISKEPSVKMYHKTGSTNGFGTYVVFIPKENI
DC-76WP_001211237.1_1040AbBCP   EEFSYPATLQTLLDSNSEQIVMKPNKVTAISKEPSVKMYHKTGSTNGFGTYVVFIPKENI
DC-76WP_001211237.1_3682AbBCP   EEFSYPATLQTLLDSNSEQIVMKPNKVTAISKEPSVKMYHKTGSTNGFGTYVVFIPKENI
DC-76WP_001211237.1_2579AbBCP   EEFSYPATLQTLLDSNSEQIVMKPNKVTAISKEPSVKMYHKTGSTNGFGTYVVFIPKENI
DC-291WP_001211201.1_1060AbOC   EEFSYPATLQTLLDSNSEQIVMKPNKVTAISKEPSVKMYHKTGSTNGFGTYVVFIPKENI
C-291WP_001211201.1_2415AbBOC   EEFSYPATLQTLLDSNSEQIVMKPNKVTAISKEPSVKMYHKTGSTNGFGTYVVFIPKENI
C-291WP_001211201.1_2415AbBCP   EEFSYPATLQTLLDSNSEQIVMKPNKVTAISKEPSVKMYHKTGSTNGFGTYVVFIPKENI
DC-214WP_060454527.1_118AbBOC   EEFSYPATLQTLLDSNSEQIVMKPNKVTAISKEPSVKMYHKTGSTTGFGTYVVFIPKENI
DC-214WP_060454527.1_118AbBCP   EEFSYPATLQTLLDSNSEQIVMKPNKVTAISKEPSVKMYHKTGSTTGFGTYVVFIPKENI
C-185WP_001211210.1_2745AbBOC   EEFSYPATLQTLLDSNSEQIVMKPNKVTAISKEPSVKMYHKTGSTTGFGTYVVFIPKENI
ADC-185WEI07035.1_298AbBOC     EEFSYPATLQTLLDSNSEQIVMKPNKVTAISKEPSVKMYHKTGSTTGFGTYVVFIPKENI
C-185WP_001211210.1_2745AbBCP   EEFSYPATLQTLLDSNSEQIVMKPNKVTAISKEPSVKMYHKTGSTTGFGTYVVFIPKENI
ADC-185WEI07035.1_298AbBCP     EEFSYPATLQTLLDSNSEQIVMKPNKVTAISKEPSVKMYHKTGSTTGFGTYVVFIPKENI
ADC-11WP_001211205.1_1099AbOC   EEFSYPATLQTLLDSNSEQIVMKPNKVTAISKEPSVKMYHKTGSTNGFGTYVVFIPKENI
ADC-11WP_001211205.1_1035AbOC   EEFSYPATLQTLLDSNSEQIVMKPNKVTAISKEPSVKMYHKTGSTNGFGTYVVFIPKENI
DC-11WP_001211205.1_2603AbBOC   EEFSYPATLQTLLDSNSEQIVMKPNKVTAISKEPSVKMYHKTGSTNGFGTYVVFIPKENI
DC-11WP_001211205.1_1138AbBOC   EEFSYPATLQTLLDSNSEQIVMKPNKVTAISKEPSVKMYHKTGSTNGFGTYVVFIPKENI
DC-11WP_001211205.1_2874AbBOC   EEFSYPATLQTLLDSNSEQIVMKPNKVTAISKEPSVKMYHKTGSTNGFGTYVVFIPKENI
DC-11WP_001211205.1_2686AbBOC   EEFSYPATLQTLLDSNSEQIVMKPNKVTAISKEPSVKMYHKTGSTNGFGTYVVFIPKENI
ADC-11WP_001211205.1_130AbBOC   EEFSYPATLQTLLDSNSEQIVMKPNKVTAISKEPSVKMYHKTGSTNGFGTYVVFIPKENI
DC-11WP_001211205.1_1097AbBOC   EEFSYPATLQTLLDSNSEQIVMKPNKVTAISKEPSVKMYHKTGSTNGFGTYVVFIPKENI
ADC-11WP_001211205.1_684AbBOC   EEFSYPATLQTLLDSNSEQIVMKPNKVTAISKEPSVKMYHKTGSTNGFGTYVVFIPKENI
ADC-11WP_001211205.1_682AbBOC   EEFSYPATLQTLLDSNSEQIVMKPNKVTAISKEPSVKMYHKTGSTNGFGTYVVFIPKENI
DC-11WP_001211205.1_1111AbBOC   EEFSYPATLQTLLDSNSEQIVMKPNKVTAISKEPSVKMYHKTGSTNGFGTYVVFIPKENI
DC-11WP_001211205.1_1059AbBOC   EEFSYPATLQTLLDSNSEQIVMKPNKVTAISKEPSVKMYHKTGSTNGFGTYVVFIPKENI
DC-11WP_001211205.1_2754AbBOC   EEFSYPATLQTLLDSNSEQIVMKPNKVTAISKEPSVKMYHKTGSTNGFGTYVVFIPKENI
DC-11WP_001211205.1_2603AbBCP   EEFSYPATLQTLLDSNSEQIVMKPNKVTAISKEPSVKMYHKTGSTNGFGTYVVFIPKENI
DC-11WP_001211205.1_1138AbBCP   EEFSYPATLQTLLDSNSEQIVMKPNKVTAISKEPSVKMYHKTGSTNGFGTYVVFIPKENI
DC-11WP_001211205.1_2874AbBCP   EEFSYPATLQTLLDSNSEQIVMKPNKVTAISKEPSVKMYHKTGSTNGFGTYVVFIPKENI
DC-11WP_001211205.1_2686AbBCP   EEFSYPATLQTLLDSNSEQIVMKPNKVTAISKEPSVKMYHKTGSTNGFGTYVVFIPKENI
ADC-11WP_001211205.1_130AbBCP   EEFSYPATLQTLLDSNSEQIVMKPNKVTAISKEPSVKMYHKTGSTNGFGTYVVFIPKENI
DC-11WP_001211205.1_1097AbBCP   EEFSYPATLQTLLDSNSEQIVMKPNKVTAISKEPSVKMYHKTGSTNGFGTYVVFIPKENI
ADC-11WP_001211205.1_684AbBCP   EEFSYPATLQTLLDSNSEQIVMKPNKVTAISKEPSVKMYHKTGSTNGFGTYVVFIPKENI
ADC-11WP_001211205.1_682AbBCP   EEFSYPATLQTLLDSNSEQIVMKPNKVTAISKEPSVKMYHKTGSTNGFGTYVVFIPKENI
DC-11WP_001211205.1_1111AbBCP   EEFSYPATLQTLLDSNSEQIVMKPNKVTAISKEPSVKMYHKTGSTNGFGTYVVFIPKENI
DC-11WP_001211205.1_1059AbBCP   EEFSYPATLQTLLDSNSEQIVMKPNKVTAISKEPSVKMYHKTGSTNGFGTYVVFIPKENI
DC-11WP_001211205.1_2754AbBCP   EEFSYPATLQTLLDSNSEQIVMKPNKVTAISKEPSVKMYHKTGSTNGFGTYVVFIPKENI
C-269WP_032025626.1_2988AbBOC   EEFSYPATLQTLLDSNSEQIVMKPNKVTAISKEPSVKMYHKTGSTNGFGTYVVFIPKENI
C-269WP_032025626.1_2988AbBCP   EEFSYPATLQTLLDSNSEQIVMKPNKVTAISKEPSVKMYHKTGSTNGFGTYVVFIPKENI
C-166WP_101244937.1_1028AbBOC   EEFSYPAMLQTLLDSNSEQIVMKPNKVTAISKEPSVKMYHKTGSTNGFGTYVVFIPKENI
C-166WP_101244937.1_1028AbBCP   EEFSYPAMLQTLLDSNSEQIVMKPNKVTAISKEPSVKMYHKTGSTNGFGTYVVFIPKENI
C-175WP_001211208.1_2561AbBOC   EEFSYPATLQTLLDSSSEQIVMKPNKVTAISKEPSVKMYHKTGSTNGFGTYVVFIPKENI
C-175WP_001211208.1_2561AbBCP   EEFSYPATLQTLLDSSSEQIVMKPNKVTAISKEPSVKMYHKTGSTNGFGTYVVFIPKENI
C-154WP_005138362.1_2451AbBOC   EEFSYPATLQTLLDSNSEQIVMKPNKVTAISKEPSVKMYHKTGSTNGFGTYVVFIPKENI
DC-154WP_005138362.1_158AbBOC   EEFSYPATLQTLLDSNSEQIVMKPNKVTAISKEPSVKMYHKTGSTNGFGTYVVFIPKENI
```

C-154WP_005138362.1_1167AbBOC	EEFSYPATLQTLLDSNSEQIVMKPNKVTAISKEPSVKMYHKTGSTNGFGTYVVFIPKENI
C-154WP_005138362.1_2510AbBOC	EEFSYPATLQTLLDSNSEQIVMKPNKVTAISKEPSVKMYHKTGSTNGFGTYVVFIPKENI
C-154WP_005138362.1_2513AbBOC	EEFSYPATLQTLLDSNSEQIVMKPNKVTAISKEPSVKMYHKTGSTNGFGTYVVFIPKENI
C-154WP_005138362.1_2512AbBOC	EEFSYPATLQTLLDSNSEQIVMKPNKVTAISKEPSVKMYHKTGSTNGFGTYVVFIPKENI
C-154WP_005138362.1_2451AbBCP	EEFSYPATLQTLLDSNSEQIVMKPNKVTAISKEPSVKMYHKTGSTNGFGTYVVFIPKENI
DC-154WP_005138362.1_158AbBCP	EEFSYPATLQTLLDSNSEQIVMKPNKVTAISKEPSVKMYHKTGSTNGFGTYVVFIPKENI
C-154WP_005138362.1_1167AbBCP	EEFSYPATLQTLLDSNSEQIVMKPNKVTAISKEPSVKMYHKTGSTNGFGTYVVFIPKENI
C-154WP_005138362.1_2510AbBCP	EEFSYPATLQTLLDSNSEQIVMKPNKVTAISKEPSVKMYHKTGSTNGFGTYVVFIPKENI
C-154WP_005138362.1_2513AbBCP	EEFSYPATLQTLLDSNSEQIVMKPNKVTAISKEPSVKMYHKTGSTNGFGTYVVFIPKENI
C-154WP_005138362.1_2512AbBCP	EEFSYPATLQTLLDSNSEQIVMKPNKVTAISKEPSVKMYHKTGSTNGFGTYVVFIPKENI
C-240WP_001211207.1_1200AbBOC	EEFSYPATLQTLLDSNSEQIVMKPNKVTAISKEPSVKMYHKTGSTTGFGTYVVFIPKENI
C-240WP_001211207.1_1200AbBCP	EEFSYPATLQTLLDSNSEQIVMKPNKVTAISKEPSVKMYHKTGSTTGFGTYVVFIPKENI
ADC-75WP_063857817.1_1610AbOC	EEFSYPATLQTLLDSNSEQIVMKPNKVTAISKEPSVKMYHKTGSTTGFGTYVVFIPKENI
DC-18WP_002118772.1_1702AbBOC	EEFSYPAPLQTLLDSNSEQIVMKPNKVTAISKEPSVKMFHKTGSTNGFGSYVVFIPKENI
DC-18WP_002118772.1_1702AbBCP	EEFSYPAPLQTLLDSNSEQIVMKPNKVTAISKEPSVKMFHKTGSTNGFGSYVVFIPKENI
C-106WP_068981613.1_2650AbBOC	EEFSYPATLQTLLDSNSEQIVMKPNKVTAISKEPSVKMYHKTGSTNGFGTYVVFIPKENI
C-106WP_068981613.1_2650AbBCP	EEFSYPATLQTLLDSNSEQIVMKPNKVTAISKEPSVKMYHKTGSTNGFGTYVVFIPKENI
DC-50WP_031965243.1_2562AbBOC	EEFSYPTPLQTLLDSNSEQIVMKPNKVTAISKEPSVKMYHKTGSTNGFGTYVVFIPKENI
ADC-50WP_031965243.1_206AbBOC	EEFSYPTPLQTLLDSNSEQIVMKPNKVTAISKEPSVKMYHKTGSTNGFGTYVVFIPKENI
DC-50WP_031965243.1_2751AbBOC	EEFSYPTPLQTLLDSNSEQIVMKPNKVTAISKEPSVKMYHKTGSTNGFGTYVVFIPKENI
DC-50WP_031965243.1_3766AbBOC	EEFSYPTPLQTLLDSNSEQIVMKPNKVTAISKEPSVKMYHKTGSTNGFGTYVVFIPKENI
DC-50WP_031965243.1_2562AbBCP	EEFSYPTPLQTLLDSNSEQIVMKPNKVTAISKEPSVKMYHKTGSTNGFGTYVVFIPKENI
ADC-50WP_031965243.1_206AbBCP	EEFSYPTPLQTLLDSNSEQIVMKPNKVTAISKEPSVKMYHKTGSTNGFGTYVVFIPKENI
DC-50WP_031965243.1_2751AbBCP	EEFSYPTPLQTLLDSNSEQIVMKPNKVTAISKEPSVKMYHKTGSTNGFGTYVVFIPKENI
DC-50WP_031965243.1_3766AbBCP	EEFSYPTPLQTLLDSNSEQIVMKPNKVTAISKEPSVKMYHKTGSTNGFGTYVVFIPKENI
ADC-158WP_001211227.1_990AbOC	EEFSYPATLQTLLDSNSEQIVMKPNKVTAISKEPSVKMYHKTGSTNGFGTYVVFIPKENI
DC-158WP_001211227.1_1295AbOC	EEFSYPATLQTLLDSNSEQIVMKPNKVTAISKEPSVKMYHKTGSTNGFGTYVVFIPKENI
ADC-158WP_001211227.1_6AbOC	EEFSYPATLQTLLDSNSEQIVMKPNKVTAISKEPSVKMYHKTGSTNGFGTYVVFIPKENI
DC-158WP_001211227.1_991AbBOC	EEFSYPATLQTLLDSNSEQIVMKPNKVTAISKEPSVKMYHKTGSTNGFGTYVVFIPKENI
DC-158WP_001211227.1_207AbBOC	EEFSYPATLQTLLDSNSEQIVMKPNKVTAISKEPSVKMYHKTGSTNGFGTYVVFIPKENI
C-158WP_001211227.1_2735AbBOC	EEFSYPATLQTLLDSNSEQIVMKPNKVTAISKEPSVKMYHKTGSTNGFGTYVVFIPKENI
DC-158WP_001211227.1_996AbBOC	EEFSYPATLQTLLDSNSEQIVMKPNKVTAISKEPSVKMYHKTGSTNGFGTYVVFIPKENI
DC-158WP_001211227.1_796AbBOC	EEFSYPATLQTLLDSNSEQIVMKPNKVTAISKEPSVKMYHKTGSTNGFGTYVVFIPKENI
C-158WP_001211227.1_2822AbBOC	EEFSYPATLQTLLDSNSEQIVMKPNKVTAISKEPSVKMYHKTGSTNGFGTYVVFIPKENI
C-158WP_001211227.1_1339AbBOC	EEFSYPATLQTLLDSNSEQIVMKPNKVTAISKEPSVKMYHKTGSTNGFGTYVVFIPKENI
ADC-158WGQ04828.1_2712AbBOC	EEFSYPATLQTLLDSNSEQIVMKPNKVTAISKEPSVKMYHKTGSTNGFGTYVVFIPKENI
DC-158WP_001211227.1_991AbBCP	EEFSYPATLQTLLDSNSEQIVMKPNKVTAISKEPSVKMYHKTGSTNGFGTYVVFIPKENI
DC-158WP_001211227.1_207AbBCP	EEFSYPATLQTLLDSNSEQIVMKPNKVTAISKEPSVKMYHKTGSTNGFGTYVVFIPKENI
C-158WP_001211227.1_2735AbBCP	EEFSYPATLQTLLDSNSEQIVMKPNKVTAISKEPSVKMYHKTGSTNGFGTYVVFIPKENI
DC-158WP_001211227.1_996AbBCP	EEFSYPATLQTLLDSNSEQIVMKPNKVTAISKEPSVKMYHKTGSTNGFGTYVVFIPKENI
DC-158WP_001211227.1_796AbBCP	EEFSYPATLQTLLDSNSEQIVMKPNKVTAISKEPSVKMYHKTGSTNGFGTYVVFIPKENI
C-158WP_001211227.1_2822AbBCP	EEFSYPATLQTLLDSNSEQIVMKPNKVTAISKEPSVKMYHKTGSTNGFGTYVVFIPKENI
C-158WP_001211227.1_1339AbBCP	EEFSYPATLQTLLDSNSEQIVMKPNKVTAISKEPSVKMYHKTGSTNGFGTYVVFIPKENI
ADC-158WGQ04828.1_2712AbBCP	EEFSYPATLQTLLDSNSEQIVMKPNKVTAISKEPSVKMYHKTGSTNGFGTYVVFIPKENI
ADC-31WP_001211223.1_2986AbOC	EEFSYPATLQTLLDSNSEQIVMKPNKVTAISKEPSVKMYHKTGSTSGFGTYVVFIPKENI
DC-31WP_001211223.1_2698AbBOC	EEFSYPATLQTLLDSNSEQIVMKPNKVTAISKEPSVKMYHKTGSTSGFGTYVVFIPKENI
DC-31WP_001211223.1_2698AbBCP	EEFSYPATLQTLLDSNSEQIVMKPNKVTAISKEPSVKMYHKTGSTSGFGTYVVFIPKENI
ADC-30WP_001211218.1_1102AbOC	EEFSYPATLQTLLDSNSEQIVMKPNKVTAISKEPSVKMYHKTGSTTGFGTYVVFIPKENI
ADC-30WP_001211218.1_2565AbOC	EEFSYPATLQTLLDSNSEQIVMKPNKVTAISKEPSVKMYHKTGSTTGFGTYVVFIPKENI
ADC-30WP_001211218.1_1021AbOC	EEFSYPATLQTLLDSNSEQIVMKPNKVTAISKEPSVKMYHKTGSTTGFGTYVVFIPKENI
ADC-30WP_001211218.1_1088AbOC	EEFSYPATLQTLLDSNSEQIVMKPNKVTAISKEPSVKMYHKTGSTTGFGTYVVFIPKENI
ADC-30WP_001211218.1_1135AbOC	EEFSYPATLQTLLDSNSEQIVMKPNKVTAISKEPSVKMYHKTGSTTGFGTYVVFIPKENI
ADC-30WP_001211218.1_43AbOC	EEFSYPATLQTLLDSNSEQIVMKPNKVTAISKEPSVKMYHKTGSTTGFGTYVVFIPKENI
ADC-30WP_001211218.1_1041AbOC	EEFSYPATLQTLLDSNSEQIVMKPNKVTAISKEPSVKMYHKTGSTTGFGTYVVFIPKENI
ADC-30WP_001211218.1_1032AbOC	EEFSYPATLQTLLDSNSEQIVMKPNKVTAISKEPSVKMYHKTGSTTGFGTYVVFIPKENI
ADC-30WP_001211218.1_1047AbOC	EEFSYPATLQTLLDSNSEQIVMKPNKVTAISKEPSVKMYHKTGSTTGFGTYVVFIPKENI
ADC-30WP_001211218.1_1040AbOC	EEFSYPATLQTLLDSNSEQIVMKPNKVTAISKEPSVKMYHKTGSTTGFGTYVVFIPKENI
ADC-30WP_001211218.1_3388AbOC	EEFSYPATLQTLLDSNSEQIVMKPNKVTAISKEPSVKMYHKTGSTTGFGTYVVFIPKENI
ADC-30WP_001211218.1_650AbOC	EEFSYPATLQTLLDSNSEQIVMKPNKVTAISKEPSVKMYHKTGSTTGFGTYVVFIPKENI
ADC-30WP_001211218.1_2855AbOC	EEFSYPATLQTLLDSNSEQIVMKPNKVTAISKEPSVKMYHKTGSTTGFGTYVVFIPKENI
ADC-30WP_001211218.1_1034AbOC	EEFSYPATLQTLLDSNSEQIVMKPNKVTAISKEPSVKMYHKTGSTTGFGTYVVFIPKENI
ADC-30WP_001211218.1_79AbOC	EEFSYPATLQTLLDSNSEQIVMKPNKVTAISKEPSVKMYHKTGSTTGFGTYVVFIPKENI
ADC-30WP_001211218.1_3509AbOC	EEFSYPATLQTLLDSNSEQIVMKPNKVTAISKEPSVKMYHKTGSTTGFGTYVVFIPKENI
ADC-30WP_001211218.1_2399AbOC	EEFSYPATLQTLLDSNSEQIVMKPNKVTAISKEPSVKMYHKTGSTTGFGTYVVFIPKENI
ADC-30WP_001211218.1_2415AbOC	EEFSYPATLQTLLDSNSEQIVMKPNKVTAISKEPSVKMYHKTGSTTGFGTYVVFIPKENI
ADC-30WP_001211218.1_3032AbOC	EEFSYPATLQTLLDSNSEQIVMKPNKVTAISKEPSVKMYHKTGSTTGFGTYVVFIPKENI
ADC-30WP_001211218.1_832AbOC	EEFSYPATLQTLLDSNSEQIVMKPNKVTAISKEPSVKMYHKTGSTTGFGTYVVFIPKENI
ADC-30WP_001211218.1_1428AbOC	EEFSYPATLQTLLDSNSEQIVMKPNKVTAISKEPSVKMYHKTGSTTGFGTYVVFIPKENI
ADC-30WP_001211218.1_2340AbOC	EEFSYPATLQTLLDSNSEQIVMKPNKVTAISKEPSVKMYHKTGSTTGFGTYVVFIPKENI
ADC-30WP_001211218.1_2976AbOC	EEFSYPATLQTLLDSNSEQIVMKPNKVTAISKEPSVKMYHKTGSTTGFGTYVVFIPKENI
ADC-30WP_001211218.1_2305AbOC	EEFSYPATLQTLLDSNSEQIVMKPNKVTAISKEPSVKMYHKTGSTTGFGTYVVFIPKENI
ADC-30WP_001211218.1_1110AbOC	EEFSYPATLQTLLDSNSEQIVMKPNKVTAISKEPSVKMYHKTGSTTGFGTYVVFIPKENI
ADC-30WP_001211218.1_2797AbOC	EEFSYPATLQTLLDSNSEQIVMKPNKVTAISKEPSVKMYHKTGSTTGFGTYVVFIPKENI
ADC-30WP_001211218.1_1023AbOC	EEFSYPATLQTLLDSNSEQIVMKPNKVTAISKEPSVKMYHKTGSTTGFGTYVVFIPKENI
ADC-30WP_001211218.1_1022AbOC	EEFSYPATLQTLLDSNSEQIVMKPNKVTAISKEPSVKMYHKTGSTTGFGTYVVFIPKENI
ADC-30WP_001211218.1_2458AbOC	EEFSYPATLQTLLDSNSEQIVMKPNKVTAISKEPSVKMYHKTGSTTGFGTYVVFIPKENI
ADC-30WP_001211218.1_2513AbOC	EEFSYPATLQTLLDSNSEQIVMKPNKVTAISKEPSVKMYHKTGSTTGFGTYVVFIPKENI
ADC-30WP_001211218.1_2482AbOC	EEFSYPATLQTLLDSNSEQIVMKPNKVTAISKEPSVKMYHKTGSTTGFGTYVVFIPKENI
DC-30WP_001211218.1_2692AbBOC	EEFSYPATLQTLLDSNSEQIVMKPNKVTAISKEPSVKMYHKTGSTTGFGTYVVFIPKENI
DC-30WP_001211218.1_2549AbBOC	EEFSYPATLQTLLDSNSEQIVMKPNKVTAISKEPSVKMYHKTGSTTGFGTYVVFIPKENI
DC-30WP_001211218.1_1026AbBOC	EEFSYPATLQTLLDSNSEQIVMKPNKVTAISKEPSVKMYHKTGSTTGFGTYVVFIPKENI
ADC-30WP_001211218.1_373AbBOC	EEFSYPATLQTLLDSNSEQIVMKPNKVTAISKEPSVKMYHKTGSTTGFGTYVVFIPKENI
DC-30WP_001211218.1_2465AbBOC	EEFSYPATLQTLLDSNSEQIVMKPNKVTAISKEPSVKMYHKTGSTTGFGTYVVFIPKENI
DC-30WP_001211218.1_1027AbBOC	EEFSYPATLQTLLDSNSEQIVMKPNKVTAISKEPSVKMYHKTGSTTGFGTYVVFIPKENI
DC-30WP_001211218.1_2650AbBOC	EEFSYPATLQTLLDSNSEQIVMKPNKVTAISKEPSVKMYHKTGSTTGFGTYVVFIPKENI
DC-30WP_001211218.1_2674AbBOC	EEFSYPATLQTLLDSNSEQIVMKPNKVTAISKEPSVKMYHKTGSTTGFGTYVVFIPKENI
DC-30WP_001211218.1_2689AbBOC	EEFSYPATLQTLLDSNSEQIVMKPNKVTAISKEPSVKMYHKTGSTTGFGTYVVFIPKENI
DC-30WP_001211218.1_1105AbBOC	EEFSYPATLQTLLDSNSEQIVMKPNKVTAISKEPSVKMYHKTGSTTGFGTYVVFIPKENI
DC-30WP_001211218.1_2670AbBOC	EEFSYPATLQTLLDSNSEQIVMKPNKVTAISKEPSVKMYHKTGSTTGFGTYVVFIPKENI
DC-30WP_001211218.1_1019AbBOC	EEFSYPATLQTLLDSNSEQIVMKPNKVTAISKEPSVKMYHKTGSTTGFGTYVVFIPKENI
DC-30WP_001211218.1_1039AbBOC	EEFSYPATLQTLLDSNSEQIVMKPNKVTAISKEPSVKMYHKTGSTTGFGTYVVFIPKENI
DC-30WP_001211218.1_1023AbBOC	EEFSYPATLQTLLDSNSEQIVMKPNKVTAISKEPSVKMYHKTGSTTGFGTYVVFIPKENI
DC-30WP_001211218.1_3189AbBOC	EEFSYPATLQTLLDSNSEQIVMKPNKVTAISKEPSVKMYHKTGSTTGFGTYVVFIPKENI
ADC-30WP_001211218.1_594AbBOC	EEFSYPATLQTLLDSNSEQIVMKPNKVTAISKEPSVKMYHKTGSTTGFGTYVVFIPKENI
DC-30WP_001211218.1_1718AbBOC	EEFSYPATLQTLLDSNSEQIVMKPNKVTAISKEPSVKMYHKTGSTTGFGTYVVFIPKENI
DC-30WP_001211218.1_2484AbBOC	EEFSYPATLQTLLDSNSEQIVMKPNKVTAISKEPSVKMYHKTGSTTGFGTYVVFIPKENI
DC-30WP_001211218.1_3305AbBOC	EEFSYPATLQTLLDSNSEQIVMKPNKVTAISKEPSVKMYHKTGSTTGFGTYVVFIPKENI
DC-30WP_001211218.1_3346AbBOC	EEFSYPATLQTLLDSNSEQIVMKPNKVTAISKEPSVKMYHKTGSTTGFGTYVVFIPKENI
DC-30WP_001211218.1_1482AbBOC	EEFSYPATLQTLLDSNSEQIVMKPNKVTAISKEPSVKMYHKTGSTTGFGTYVVFIPKENI
DC-30WP_001211218.1_2574AbBOC	EEFSYPATLQTLLDSNSEQIVMKPNKVTAISKEPSVKMYHKTGSTTGFGTYVVFIPKENI
ADC-30WP_001211218.1_831AbBOC	EEFSYPATLQTLLDSNSEQIVMKPNKVTAISKEPSVKMYHKTGSTTGFGTYVVFIPKENI
DC-30WP_001211218.1_1826AbBOC	EEFSYPATLQTLLDSNSEQIVMKPNKVTAISKEPSVKMYHKTGSTTGFGTYVVFIPKENI
DC-30WP_001211218.1_1024AbBOC	EEFSYPATLQTLLDSNSEQIVMKPNKVTAISKEPSVKMYHKTGSTTGFGTYVVFIPKENI
DC-30WP_001211218.1_1618AbBOC	EEFSYPATLQTLLDSNSEQIVMKPNKVTAISKEPSVKMYHKTGSTTGFGTYVVFIPKENI
ADC-30WP_001211218.1_301AbBOC	EEFSYPATLQTLLDSNSEQIVMKPNKVTAISKEPSVKMYHKTGSTTGFGTYVVFIPKENI
ADC-30WP_001211218.1_940AbBOC	EEFSYPATLQTLLDSNSEQIVMKPNKVTAISKEPSVKMYHKTGSTTGFGTYVVFIPKENI
DC-30WP_001211218.1_2949AbBOC	EEFSYPATLQTLLDSNSEQIVMKPNKVTAISKEPSVKMYHKTGSTTGFGTYVVFIPKENI
DC-30WP_001211218.1_1028AbBOC	EEFSYPATLQTLLDSNSEQIVMKPNKVTAISKEPSVKMYHKTGSTTGFGTYVVFIPKENI
DC-30WP_001211218.1_2526AbBOC	EEFSYPATLQTLLDSNSEQIVMKPNKVTAISKEPSVKMYHKTGSTTGFGTYVVFIPKENI
DC-30WP_001211218.1_1040AbBOC	EEFSYPATLQTLLDSNSEQIVMKPNKVTAISKEPSVKMYHKTGSTTGFGTYVVFIPKENI
DC-30WP_001211218.1_1089AbBOC	EEFSYPATLQTLLDSNSEQIVMKPNKVTAISKEPSVKMYHKTGSTTGFGTYVVFIPKENI
DC-30WP_001211218.1_1099AbBOC	EEFSYPATLQTLLDSNSEQIVMKPNKVTAISKEPSVKMYHKTGSTTGFGTYVVFIPKENI
DC-30WP_001211218.1_1206AbBOC	EEFSYPATLQTLLDSNSEQIVMKPNKVTAISKEPSVKMYHKTGSTTGFGTYVVFIPKENI
DC-30WP_001211218.1_1167AbBOC	EEFSYPATLQTLLDSNSEQIVMKPNKVTAISKEPSVKMYHKTGSTTGFGTYVVFIPKENI
DC-30WP_001211218.1_1186AbBOC	EEFSYPATLQTLLDSNSEQIVMKPNKVTAISKEPSVKMYHKTGSTTGFGTYVVFIPKENI
DC-30WP_001211218.1_1165AbBOC	EEFSYPATLQTLLDSNSEQIVMKPNKVTAISKEPSVKMYHKTGSTTGFGTYVVFIPKENI
DC-30WP_001211218.1_1077AbBOC	EEFSYPATLQTLLDSNSEQIVMKPNKVTAISKEPSVKMYHKTGSTTGFGTYVVFIPKENI
DC-30WP_001211218.1_1078AbBOC	EEFSYPATLQTLLDSNSEQIVMKPNKVTAISKEPSVKMYHKTGSTTGFGTYVVFIPKENI
DC-30WP_001211218.1_1071AbBOC	EEFSYPATLQTLLDSNSEQIVMKPNKVTAISKEPSVKMYHKTGSTTGFGTYVVFIPKENI
DC-30WP_001211218.1_2694AbBOC	EEFSYPATLQTLLDSNSEQIVMKPNKVTAISKEPSVKMYHKTGSTTGFGTYVVFIPKENI
DC-30WP_001211218.1_3340AbBOC	EEFSYPATLQTLLDSNSEQIVMKPNKVTAISKEPSVKMYHKTGSTTGFGTYVVFIPKENI
DC-30WP_001211218.1_1022AbBOC	EEFSYPATLQTLLDSNSEQIVMKPNKVTAISKEPSVKMYHKTGSTTGFGTYVVFIPKENI
DC-30WP_001211218.1_1025AbBOC	EEFSYPATLQTLLDSNSEQIVMKPNKVTAISKEPSVKMYHKTGSTTGFGTYVVFIPKENI
DC-30WP_001211218.1_1158AbBOC	EEFSYPATLQTLLDSNSEQIVMKPNKVTAISKEPSVKMYHKTGSTTGFGTYVVFIPKENI
DC-30WP_001211218.1_1020AbBOC	EEFSYPATLQTLLDSNSEQIVMKPNKVTAISKEPSVKMYHKTGSTTGFGTYVVFIPKENI
DC-30WP_001211218.1_1021AbBOC	EEFSYPATLQTLLDSNSEQIVMKPNKVTAISKEPSVKMYHKTGSTTGFGTYVVFIPKENI
ADC-30WP_001211218.1_532AbBOC	EEFSYPATLQTLLDSNSEQIVMKPNKVTAISKEPSVKMYHKTGSTTGFGTYVVFIPKENI
ADC-30WP_001211218.1_539AbBOC	EEFSYPATLQTLLDSNSEQIVMKPNKVTAISKEPSVKMYHKTGSTTGFGTYVVFIPKENI
DC-30WP_001211218.1_1033AbBOC	EEFSYPATLQTLLDSNSEQIVMKPNKVTAISKEPSVKMYHKTGSTTGFGTYVVFIPKENI
DC-30WP_001211218.1_1069AbBOC	EEFSYPATLQTLLDSNSEQIVMKPNKVTAISKEPSVKMYHKTGSTTGFGTYVVFIPKENI
DC-30WP_001211218.1_1029AbBOC	EEFSYPATLQTLLDSNSEQIVMKPNKVTAISKEPSVKMYHKTGSTTGFGTYVVFIPKENI
DC-30WP_001211218.1_1030AbBOC	EEFSYPATLQTLLDSNSEQIVMKPNKVTAISKEPSVKMYHKTGSTTGFGTYVVFIPKENI
DC-30WP_001211218.1_1096AbBOC	EEFSYPATLQTLLDSNSEQIVMKPNKVTAISKEPSVKMYHKTGSTTGFGTYVVFIPKENI
DC-30WP_001211218.1_1037AbBOC	EEFSYPATLQTLLDSNSEQIVMKPNKVTAISKEPSVKMYHKTGSTTGFGTYVVFIPKENI
DC-30WP_001211218.1_1098AbBOC	EEFSYPATLQTLLDSNSEQIVMKPNKVTAISKEPSVKMYHKTGSTTGFGTYVVFIPKENI
DC-30WP_001211218.1_1046AbBOC	EEFSYPATLQTLLDSNSEQIVMKPNKVTAISKEPSVKMYHKTGSTTGFGTYVVFIPKENI
DC-30WP_001211218.1_1032AbBOC	EEFSYPATLQTLLDSNSEQIVMKPNKVTAISKEPSVKMYHKTGSTTGFGTYVVFIPKENI
DC-30WP_001211218.1_1070AbBOC	EEFSYPATLQTLLDSNSEQIVMKPNKVTAISKEPSVKMYHKTGSTTGFGTYVVFIPKENI
DC-30WP_001211218.1_1139AbBOC	EEFSYPATLQTLLDSNSEQIVMKPNKVTAISKEPSVKMYHKTGSTTGFGTYVVFIPKENI
DC-30WP_001211218.1_1031AbBOC	EEFSYPATLQTLLDSNSEQIVMKPNKVTAISKEPSVKMYHKTGSTTGFGTYVVFIPKENI
DC-30WP_001211218.1_1066AbBOC	EEFSYPATLQTLLDSNSEQIVMKPNKVTAISKEPSVKMYHKTGSTTGFGTYVVFIPKENI
DC-30WP_001211218.1_1102AbBOC	EEFSYPATLQTLLDSNSEQIVMKPNKVTAISKEPSVKMYHKTGSTTGFGTYVVFIPKENI
ADC-30USX57838.1_2957AbBOC	EEFSYPATLQTLLDSNSEQIVMKPNKVTAISKEPSVKMYHKTGSTTGFGTYVVFIPKENI
ADC-30USX59985.1_1918AbBOC	EEFSYPATLQTLLDSNSEQIVMKPNKVTAISKEPSVKMYHKTGSTTGFGTYVVFIPKENI
ADC-30USX63799.1_2772AbBOC	EEFSYPATLQTLLDSNSEQIVMKPNKVTAISKEPSVKMYHKTGSTTGFGTYVVFIPKENI
ADC-30USX74012.1_305AbBOC	EEFSYPATLQTLLDSNSEQIVMKPNKVTAISKEPSVKMYHKTGSTTGFGTYVVFIPKENI
ADC-30USZ52468.1_2397AbBOC	EEFSYPATLQTLLDSNSEQIVMKPNKVTAISKEPSVKMYHKTGSTTGFGTYVVFIPKENI
DC-30WP_001211218.1_2008AbBOC	EEFSYPATLQTLLDSNSEQIVMKPNKVTAISKEPSVKMYHKTGSTTGFGTYVVFIPKENI
ADC-30WCL78976.1_1026AbBOC	EEFSYPATLQTLLDSNSEQIVMKPNKVTAISKEPSVKMYHKTGSTTGFGTYVVFIPKENI
ADC-30WDO27826.1_1034AbBOC	EEFSYPATLQTLLDSNSEQIVMKPNKVTAISKEPSVKMYHKTGSTTGFGTYVVFIPKENI
DC-30WP_001211218.1_2125AbBOC	EEFSYPATLQTLLDSNSEQIVMKPNKVTAISKEPSVKMYHKTGSTTGFGTYVVFIPKENI
DC-30WP_001211218.1_2729AbBOC	EEFSYPATLQTLLDSNSEQIVMKPNKVTAISKEPSVKMYHKTGSTTGFGTYVVFIPKENI

```
DC-30WP_001211218.1_2692AbBCP    EEFSYPATLQTLLDSNSEQIVMKPNKVTAISKEPSVKMYHKTGSTTGFGTYVVFIPKENI
DC-30WP_001211218.1_2549AbBCP    EEFSYPATLQTLLDSNSEQIVMKPNKVTAISKEPSVKMYHKTGSTTGFGTYVVFIPKENI
DC-30WP_001211218.1_1026AbBCP    EEFSYPATLQTLLDSNSEQIVMKPNKVTAISKEPSVKMYHKTGSTTGFGTYVVFIPKENI
ADC-30WP_001211218.1_373AbBCP    EEFSYPATLQTLLDSNSEQIVMKPNKVTAISKEPSVKMYHKTGSTTGFGTYVVFIPKENI
DC-30WP_001211218.1_2465AbBCP    EEFSYPATLQTLLDSNSEQIVMKPNKVTAISKEPSVKMYHKTGSTTGFGTYVVFIPKENI
DC-30WP_001211218.1_1027AbBCP    EEFSYPATLQTLLDSNSEQIVMKPNKVTAISKEPSVKMYHKTGSTTGFGTYVVFIPKENI
DC-30WP_001211218.1_2650AbBCP    EEFSYPATLQTLLDSNSEQIVMKPNKVTAISKEPSVKMYHKTGSTTGFGTYVVFIPKENI
DC-30WP_001211218.1_2674AbBCP    EEFSYPATLQTLLDSNSEQIVMKPNKVTAISKEPSVKMYHKTGSTTGFGTYVVFIPKENI
DC-30WP_001211218.1_2689AbBCP    EEFSYPATLQTLLDSNSEQIVMKPNKVTAISKEPSVKMYHKTGSTTGFGTYVVFIPKENI
DC-30WP_001211218.1_1105AbBCP    EEFSYPATLQTLLDSNSEQIVMKPNKVTAISKEPSVKMYHKTGSTTGFGTYVVFIPKENI
DC-30WP_001211218.1_2670AbBCP    EEFSYPATLQTLLDSNSEQIVMKPNKVTAISKEPSVKMYHKTGSTTGFGTYVVFIPKENI
DC-30WP_001211218.1_1019AbBCP    EEFSYPATLQTLLDSNSEQIVMKPNKVTAISKEPSVKMYHKTGSTTGFGTYVVFIPKENI
DC-30WP_001211218.1_1039AbBCP    EEFSYPATLQTLLDSNSEQIVMKPNKVTAISKEPSVKMYHKTGSTTGFGTYVVFIPKENI
DC-30WP_001211218.1_1023AbBCP    EEFSYPATLQTLLDSNSEQIVMKPNKVTAISKEPSVKMYHKTGSTTGFGTYVVFIPKENI
DC-30WP_001211218.1_3189AbBCP    EEFSYPATLQTLLDSNSEQIVMKPNKVTAISKEPSVKMYHKTGSTTGFGTYVVFIPKENI
ADC-30WP_001211218.1_594AbBCP    EEFSYPATLQTLLDSNSEQIVMKPNKVTAISKEPSVKMYHKTGSTTGFGTYVVFIPKENI
DC-30WP_001211218.1_1718AbBCP    EEFSYPATLQTLLDSNSEQIVMKPNKVTAISKEPSVKMYHKTGSTTGFGTYVVFIPKENI
DC-30WP_001211218.1_2484AbBCP    EEFSYPATLQTLLDSNSEQIVMKPNKVTAISKEPSVKMYHKTGSTTGFGTYVVFIPKENI
DC-30WP_001211218.1_3305AbBCP    EEFSYPATLQTLLDSNSEQIVMKPNKVTAISKEPSVKMYHKTGSTTGFGTYVVFIPKENI
DC-30WP_001211218.1_3346AbBCP    EEFSYPATLQTLLDSNSEQIVMKPNKVTAISKEPSVKMYHKTGSTTGFGTYVVFIPKENI
DC-30WP_001211218.1_1482AbBCP    EEFSYPATLQTLLDSNSEQIVMKPNKVTAISKEPSVKMYHKTGSTTGFGTYVVFIPKENI
DC-30WP_001211218.1_2574AbBCP    EEFSYPATLQTLLDSNSEQIVMKPNKVTAISKEPSVKMYHKTGSTTGFGTYVVFIPKENI
ADC-30WP_001211218.1_831AbBCP    EEFSYPATLQTLLDSNSEQIVMKPNKVTAISKEPSVKMYHKTGSTTGFGTYVVFIPKENI
DC-30WP_001211218.1_1826AbBCP    EEFSYPATLQTLLDSNSEQIVMKPNKVTAISKEPSVKMYHKTGSTTGFGTYVVFIPKENI
DC-30WP_001211218.1_1024AbBCP    EEFSYPATLQTLLDSNSEQIVMKPNKVTAISKEPSVKMYHKTGSTTGFGTYVVFIPKENI
DC-30WP_001211218.1_1618AbBCP    EEFSYPATLQTLLDSNSEQIVMKPNKVTAISKEPSVKMYHKTGSTTGFGTYVVFIPKENI
ADC-30WP_001211218.1_301AbBCP    EEFSYPATLQTLLDSNSEQIVMKPNKVTAISKEPSVKMYHKTGSTTGFGTYVVFIPKENI
ADC-30WP_001211218.1_940AbBCP    EEFSYPATLQTLLDSNSEQIVMKPNKVTAISKEPSVKMYHKTGSTTGFGTYVVFIPKENI
DC-30WP_001211218.1_2949AbBCP    EEFSYPATLQTLLDSNSEQIVMKPNKVTAISKEPSVKMYHKTGSTTGFGTYVVFIPKENI
DC-30WP_001211218.1_1028AbBCP    EEFSYPATLQTLLDSNSEQIVMKPNKVTAISKEPSVKMYHKTGSTTGFGTYVVFIPKENI
DC-30WP_001211218.1_2526AbBCP    EEFSYPATLQTLLDSNSEQIVMKPNKVTAISKEPSVKMYHKTGSTTGFGTYVVFIPKENI
DC-30WP_001211218.1_1040AbBCP    EEFSYPATLQTLLDSNSEQIVMKPNKVTAISKEPSVKMYHKTGSTTGFGTYVVFIPKENI
DC-30WP_001211218.1_1089AbBCP    EEFSYPATLQTLLDSNSEQIVMKPNKVTAISKEPSVKMYHKTGSTTGFGTYVVFIPKENI
DC-30WP_001211218.1_1099AbBCP    EEFSYPATLQTLLDSNSEQIVMKPNKVTAISKEPSVKMYHKTGSTTGFGTYVVFIPKENI
DC-30WP_001211218.1_1206AbBCP    EEFSYPATLQTLLDSNSEQIVMKPNKVTAISKEPSVKMYHKTGSTTGFGTYVVFIPKENI
DC-30WP_001211218.1_1167AbBCP    EEFSYPATLQTLLDSNSEQIVMKPNKVTAISKEPSVKMYHKTGSTTGFGTYVVFIPKENI
DC-30WP_001211218.1_1186AbBCP    EEFSYPATLQTLLDSNSEQIVMKPNKVTAISKEPSVKMYHKTGSTTGFGTYVVFIPKENI
DC-30WP_001211218.1_1165AbBCP    EEFSYPATLQTLLDSNSEQIVMKPNKVTAISKEPSVKMYHKTGSTTGFGTYVVFIPKENI
DC-30WP_001211218.1_1077AbBCP    EEFSYPATLQTLLDSNSEQIVMKPNKVTAISKEPSVKMYHKTGSTTGFGTYVVFIPKENI
DC-30WP_001211218.1_1078AbBCP    EEFSYPATLQTLLDSNSEQIVMKPNKVTAISKEPSVKMYHKTGSTTGFGTYVVFIPKENI
DC-30WP_001211218.1_1071AbBCP    EEFSYPATLQTLLDSNSEQIVMKPNKVTAISKEPSVKMYHKTGSTTGFGTYVVFIPKENI
DC-30WP_001211218.1_2694AbBCP    EEFSYPATLQTLLDSNSEQIVMKPNKVTAISKEPSVKMYHKTGSTTGFGTYVVFIPKENI
DC-30WP_001211218.1_3340AbBCP    EEFSYPATLQTLLDSNSEQIVMKPNKVTAISKEPSVKMYHKTGSTTGFGTYVVFIPKENI
DC-30WP_001211218.1_1022AbBCP    EEFSYPATLQTLLDSNSEQIVMKPNKVTAISKEPSVKMYHKTGSTTGFGTYVVFIPKENI
DC-30WP_001211218.1_1025AbBCP    EEFSYPATLQTLLDSNSEQIVMKPNKVTAISKEPSVKMYHKTGSTTGFGTYVVFIPKENI
DC-30WP_001211218.1_1158AbBCP    EEFSYPATLQTLLDSNSEQIVMKPNKVTAISKEPSVKMYHKTGSTTGFGTYVVFIPKENI
DC-30WP_001211218.1_1020AbBCP    EEFSYPATLQTLLDSNSEQIVMKPNKVTAISKEPSVKMYHKTGSTTGFGTYVVFIPKENI
DC-30WP_001211218.1_1021AbBCP    EEFSYPATLQTLLDSNSEQIVMKPNKVTAISKEPSVKMYHKTGSTTGFGTYVVFIPKENI
ADC-30WP_001211218.1_532AbBCP    EEFSYPATLQTLLDSNSEQIVMKPNKVTAISKEPSVKMYHKTGSTTGFGTYVVFIPKENI
ADC-30WP_001211218.1_539AbBCP    EEFSYPATLQTLLDSNSEQIVMKPNKVTAISKEPSVKMYHKTGSTTGFGTYVVFIPKENI
DC-30WP_001211218.1_1033AbBCP    EEFSYPATLQTLLDSNSEQIVMKPNKVTAISKEPSVKMYHKTGSTTGFGTYVVFIPKENI
DC-30WP_001211218.1_1069AbBCP    EEFSYPATLQTLLDSNSEQIVMKPNKVTAISKEPSVKMYHKTGSTTGFGTYVVFIPKENI
DC-30WP_001211218.1_1029AbBCP    EEFSYPATLQTLLDSNSEQIVMKPNKVTAISKEPSVKMYHKTGSTTGFGTYVVFIPKENI
DC-30WP_001211218.1_1030AbBCP    EEFSYPATLQTLLDSNSEQIVMKPNKVTAISKEPSVKMYHKTGSTTGFGTYVVFIPKENI
DC-30WP_001211218.1_1096AbBCP    EEFSYPATLQTLLDSNSEQIVMKPNKVTAISKEPSVKMYHKTGSTTGFGTYVVFIPKENI
DC-30WP_001211218.1_1037AbBCP    EEFSYPATLQTLLDSNSEQIVMKPNKVTAISKEPSVKMYHKTGSTTGFGTYVVFIPKENI
DC-30WP_001211218.1_1098AbBCP    EEFSYPATLQTLLDSNSEQIVMKPNKVTAISKEPSVKMYHKTGSTTGFGTYVVFIPKENI
DC-30WP_001211218.1_1046AbBCP    EEFSYPATLQTLLDSNSEQIVMKPNKVTAISKEPSVKMYHKTGSTTGFGTYVVFIPKENI
DC-30WP_001211218.1_1032AbBCP    EEFSYPATLQTLLDSNSEQIVMKPNKVTAISKEPSVKMYHKTGSTTGFGTYVVFIPKENI
DC-30WP_001211218.1_1070AbBCP    EEFSYPATLQTLLDSNSEQIVMKPNKVTAISKEPSVKMYHKTGSTTGFGTYVVFIPKENI
DC-30WP_001211218.1_1139AbBCP    EEFSYPATLQTLLDSNSEQIVMKPNKVTAISKEPSVKMYHKTGSTTGFGTYVVFIPKENI
DC-30WP_001211218.1_1031AbBCP    EEFSYPATLQTLLDSNSEQIVMKPNKVTAISKEPSVKMYHKTGSTTGFGTYVVFIPKENI
DC-30WP_001211218.1_1066AbBCP    EEFSYPATLQTLLDSNSEQIVMKPNKVTAISKEPSVKMYHKTGSTTGFGTYVVFIPKENI
DC-30WP_001211218.1_1102AbBCP    EEFSYPATLQTLLDSNSEQIVMKPNKVTAISKEPSVKMYHKTGSTTGFGTYVVFIPKENI
ADC-30USX57838.1_2957AbBCP      EEFSYPATLQTLLDSNSEQIVMKPNKVTAISKEPSVKMYHKTGSTTGFGTYVVFIPKENI
ADC-30USX59985.1_1918AbBCP      EEFSYPATLQTLLDSNSEQIVMKPNKVTAISKEPSVKMYHKTGSTTGFGTYVVFIPKENI
ADC-30USX63799.1_2772AbBCP      EEFSYPATLQTLLDSNSEQIVMKPNKVTAISKEPSVKMYHKTGSTTGFGTYVVFIPKENI
ADC-30USX74012.1_305AbBCP       EEFSYPATLQTLLDSNSEQIVMKPNKVTAISKEPSVKMYHKTGSTTGFGTYVVFIPKENI
ADC-30USZ52468.1_2397AbBCP      EEFSYPATLQTLLDSNSEQIVMKPNKVTAISKEPSVKMYHKTGSTTGFGTYVVFIPKENI
DC-30WP_001211218.1_2008AbBCP    EEFSYPATLQTLLDSNSEQIVMKPNKVTAISKEPSVKMYHKTGSTTGFGTYVVFIPKENI
ADC-30WCL78976.1_1026AbBCP      EEFSYPATLQTLLDSNSEQIVMKPNKVTAISKEPSVKMYHKTGSTTGFGTYVVFIPKENI
ADC-30WDO27826.1_1034AbBCP      EEFSYPATLQTLLDSNSEQIVMKPNKVTAISKEPSVKMYHKTGSTTGFGTYVVFIPKENI
DC-30WP_001211218.1_2125AbBCP    EEFSYPATLQTLLDSNSEQIVMKPNKVTAISKEPSVKMYHKTGSTTGFGTYVVFIPKENI
DC-30WP_001211218.1_2729AbBCP    EEFSYPATLQTLLDSNSEQIVMKPNKVTAISKEPSVKMYHKTGSTTGFGTYVVFIPKENI
ADC-30ADX93237.1_2806AbBCP      EEFSYPATLQTLLDSNSEQIVMKPNKVTAISKEPSVKMYHKTGSTTGFGTYVVFIPKENI
ADC-82WP_001211216.1_2747AbOC    EEFSYPATLQTLLDSNSEQIVMKPNKVTAISKEPSVKMYHKTGSTTGFGTYVVFIPKENI
DC-82WP_001211216.1_1093AbBOC    EEFSYPATLQTLLDSNSEQIVMKPNKVTAISKEPSVKMYHKTGSTTGFGTYVVFIPKENI
DC-82WP_001211216.1_1071AbBOC    EEFSYPATLQTLLDSNSEQIVMKPNKVTAISKEPSVKMYHKTGSTTGFGTYVVFIPKENI
DC-82WP_001211216.1_1093AbBCP    EEFSYPATLQTLLDSNSEQIVMKPNKVTAISKEPSVKMYHKTGSTTGFGTYVVFIPKENI
DC-82WP_001211216.1_1071AbBCP    EEFSYPATLQTLLDSNSEQIVMKPNKVTAISKEPSVKMYHKTGSTTGFGTYVVFIPKENI
DC-162WP_031980335.1_1153AbOC    EEFSYPATLQTLLDSNSEQIVMKPNKVTAISKEPSVKMYHKTGSTTGFGTYVVFIPKENI
C-162WP_031980335.1_1028AbBOC    EEFSYPATLQTLLDSNSEQIVMKPNKVTAISKEPSVKMYHKTGSTTGFGTYVVFIPKENI
C-162WP_031980335.1_1023AbBOC    EEFSYPATLQTLLDSNSEQIVMKPNKVTAISKEPSVKMYHKTGSTTGFGTYVVFIPKENI
C-162WP_031980335.1_1028AbBCP    EEFSYPATLQTLLDSNSEQIVMKPNKVTAISKEPSVKMYHKTGSTTGFGTYVVFIPKENI
C-162WP_031980335.1_1023AbBCP    EEFSYPATLQTLLDSNSEQIVMKPNKVTAISKEPSVKMYHKTGSTTGFGTYVVFIPKENI
ADC-73WP_001211219.1_1012AbOC    EEFSYPATLQTLLDSNSEQIVMKPNKVTAISKEPSVKMYHKTGSTTGFGTYVVFIPKENI
ADC-73WP_001211219.1_1953AbOC    EEFSYPATLQTLLDSNSEQIVMKPNKVTAISKEPSVKMYHKTGSTTGFGTYVVFIPKENI
ADC-73WP_001211219.1_1014AbOC    EEFSYPATLQTLLDSNSEQIVMKPNKVTAISKEPSVKMYHKTGSTTGFGTYVVFIPKENI
ADC-73WP_001211219.1_1041AbOC    EEFSYPATLQTLLDSNSEQIVMKPNKVTAISKEPSVKMYHKTGSTTGFGTYVVFIPKENI
ADC-73WP_001211219.1_1028AbOC    EEFSYPATLQTLLDSNSEQIVMKPNKVTAISKEPSVKMYHKTGSTTGFGTYVVFIPKENI
ADC-73WP_001211219.1_2674AbOC    EEFSYPATLQTLLDSNSEQIVMKPNKVTAISKEPSVKMYHKTGSTTGFGTYVVFIPKENI
ADC-73WP_001211219.1_462AbOC     EEFSYPATLQTLLDSNSEQIVMKPNKVTAISKEPSVKMYHKTGSTTGFGTYVVFIPKENI
ADC-73WP_001211219.1_3530AbOC    EEFSYPATLQTLLDSNSEQIVMKPNKVTAISKEPSVKMYHKTGSTTGFGTYVVFIPKENI
ADC-73WP_001211219.1_3621AbOC    EEFSYPATLQTLLDSNSEQIVMKPNKVTAISKEPSVKMYHKTGSTTGFGTYVVFIPKENI
ADC-73WP_001211219.1_3381AbOC    EEFSYPATLQTLLDSNSEQIVMKPNKVTAISKEPSVKMYHKTGSTTGFGTYVVFIPKENI
ADC-73WP_001211219.1_2515AbOC    EEFSYPATLQTLLDSNSEQIVMKPNKVTAISKEPSVKMYHKTGSTTGFGTYVVFIPKENI
ADC-73WP_001211219.1_3574AbOC    EEFSYPATLQTLLDSNSEQIVMKPNKVTAISKEPSVKMYHKTGSTTGFGTYVVFIPKENI
ADC-73WP_001211219.1_1656AbOC    EEFSYPATLQTLLDSNSEQIVMKPNKVTAISKEPSVKMYHKTGSTTGFGTYVVFIPKENI
ADC-73WP_001211219.1_2591AbOC    EEFSYPATLQTLLDSNSEQIVMKPNKVTAISKEPSVKMYHKTGSTTGFGTYVVFIPKENI
ADC-73WP_001211219.1_2602AbOC    EEFSYPATLQTLLDSNSEQIVMKPNKVTAISKEPSVKMYHKTGSTTGFGTYVVFIPKENI
ADC-73WP_001211219.1_910AbOC     EEFSYPATLQTLLDSNSEQIVMKPNKVTAISKEPSVKMYHKTGSTTGFGTYVVFIPKENI
ADC-73WP_001211219.1_394AbOC     EEFSYPATLQTLLDSNSEQIVMKPNKVTAISKEPSVKMYHKTGSTTGFGTYVVFIPKENI
ADC-73WP_001211219.1_1030AbOC    EEFSYPATLQTLLDSNSEQIVMKPNKVTAISKEPSVKMYHKTGSTTGFGTYVVFIPKENI
ADC-73WP_001211219.1_1023AbOC    EEFSYPATLQTLLDSNSEQIVMKPNKVTAISKEPSVKMYHKTGSTTGFGTYVVFIPKENI
ADC-73UYQ66995.1_1967AbOC       EEFSYPATLQTLLDSNSEQIVMKPNKVTAISKEPSVKMYHKTGSTTGFGTYVVFIPKENI
ADC-73WP_001211219.1_906AbBOC    EEFSYPATLQTLLDSNSEQIVMKPNKVTAISKEPSVKMYHKTGSTTGFGTYVVFIPKENI
DC-73WP_001211219.1_2649AbBOC    EEFSYPATLQTLLDSNSEQIVMKPNKVTAISKEPSVKMYHKTGSTTGFGTYVVFIPKENI
DC-73WP_001211219.1_2693AbBOC    EEFSYPATLQTLLDSNSEQIVMKPNKVTAISKEPSVKMYHKTGSTTGFGTYVVFIPKENI
ADC-73AQU57925.1_2703AbBOC      EEFSYPATLQTLLDSNSEQIVMKPNKVTAISKEPSVKMYHKTGSTTGFGTYVVFIPKENI
DC-73WP_001211219.1_1026AbBOC    EEFSYPATLQTLLDSNSEQIVMKPNKVTAISKEPSVKMYHKTGSTTGFGTYVVFIPKENI
DC-73WP_001211219.1_1019AbBOC    EEFSYPATLQTLLDSNSEQIVMKPNKVTAISKEPSVKMYHKTGSTTGFGTYVVFIPKENI
DC-73WP_001211219.1_1024AbBOC    EEFSYPATLQTLLDSNSEQIVMKPNKVTAISKEPSVKMYHKTGSTTGFGTYVVFIPKENI
ADC-73WP_001211219.1_930AbBOC    EEFSYPATLQTLLDSNSEQIVMKPNKVTAISKEPSVKMYHKTGSTTGFGTYVVFIPKENI
DC-73WP_001211219.1_2609AbBOC    EEFSYPATLQTLLDSNSEQIVMKPNKVTAISKEPSVKMYHKTGSTTGFGTYVVFIPKENI
DC-73WP_001211219.1_3456AbBOC    EEFSYPATLQTLLDSNSEQIVMKPNKVTAISKEPSVKMYHKTGSTTGFGTYVVFIPKENI
DC-73WP_001211219.1_2614AbBOC    EEFSYPATLQTLLDSNSEQIVMKPNKVTAISKEPSVKMYHKTGSTTGFGTYVVFIPKENI
DC-73WP_001211219.1_1025AbBOC    EEFSYPATLQTLLDSNSEQIVMKPNKVTAISKEPSVKMYHKTGSTTGFGTYVVFIPKENI
DC-73WP_001211219.1_1030AbBOC    EEFSYPATLQTLLDSNSEQIVMKPNKVTAISKEPSVKMYHKTGSTTGFGTYVVFIPKENI
DC-73WP_001211219.1_1173AbBOC    EEFSYPATLQTLLDSNSEQIVMKPNKVTAISKEPSVKMYHKTGSTTGFGTYVVFIPKENI
DC-73WP_001211219.1_1021AbBOC    EEFSYPATLQTLLDSNSEQIVMKPNKVTAISKEPSVKMYHKTGSTTGFGTYVVFIPKENI
DC-73WP_001211219.1_2449AbBOC    EEFSYPATLQTLLDSNSEQIVMKPNKVTAISKEPSVKMYHKTGSTTGFGTYVVFIPKENI
DC-73WP_001211219.1_1176AbBOC    EEFSYPATLQTLLDSNSEQIVMKPNKVTAISKEPSVKMYHKTGSTTGFGTYVVFIPKENI
DC-73WP_001211219.1_1161AbBOC    EEFSYPATLQTLLDSNSEQIVMKPNKVTAISKEPSVKMYHKTGSTTGFGTYVVFIPKENI
DC-73WP_001211219.1_1070AbBOC    EEFSYPATLQTLLDSNSEQIVMKPNKVTAISKEPSVKMYHKTGSTTGFGTYVVFIPKENI
DC-73WP_001211219.1_1079AbBOC    EEFSYPATLQTLLDSNSEQIVMKPNKVTAISKEPSVKMYHKTGSTTGFGTYVVFIPKENI
DC-73WP_001211219.1_1078AbBOC    EEFSYPATLQTLLDSNSEQIVMKPNKVTAISKEPSVKMYHKTGSTTGFGTYVVFIPKENI
DC-73WP_001211219.1_1037AbBOC    EEFSYPATLQTLLDSNSEQIVMKPNKVTAISKEPSVKMYHKTGSTTGFGTYVVFIPKENI
DC-73WP_001211219.1_1035AbBOC    EEFSYPATLQTLLDSNSEQIVMKPNKVTAISKEPSVKMYHKTGSTTGFGTYVVFIPKENI
DC-73WP_001211219.1_1033AbBOC    EEFSYPATLQTLLDSNSEQIVMKPNKVTAISKEPSVKMYHKTGSTTGFGTYVVFIPKENI
DC-73WP_001211219.1_1041AbBOC    EEFSYPATLQTLLDSNSEQIVMKPNKVTAISKEPSVKMYHKTGSTTGFGTYVVFIPKENI
DC-73WP_001211219.1_1023AbBOC    EEFSYPATLQTLLDSNSEQIVMKPNKVTAISKEPSVKMYHKTGSTTGFGTYVVFIPKENI
ADC-73USX69547.1_2386AbBOC      EEFSYPATLQTLLDSNSEQIVMKPNKVTAISKEPSVKMYHKTGSTTGFGTYVVFIPKENI
DC-73WP_001211219.1_1034AbBOC    EEFSYPATLQTLLDSNSEQIVMKPNKVTAISKEPSVKMYHKTGSTTGFGTYVVFIPKENI
ADC-73WP_001211219.1_852AbBOC    EEFSYPATLQTLLDSNSEQIVMKPNKVTAISKEPSVKMYHKTGSTTGFGTYVVFIPKENI
DC-73WP_001211219.1_3070AbBOC    EEFSYPATLQTLLDSNSEQIVMKPNKVTAISKEPSVKMYHKTGSTTGFGTYVVFIPKENI
ADC-73WBS60953.1_1026AbBOC      EEFSYPATLQTLLDSNSEQIVMKPNKVTAISKEPSVKMYHKTGSTTGFGTYVVFIPKENI
ADC-73WDC39660.1_1023AbBOC      EEFSYPATLQTLLDSNSEQIVMKPNKVTAISKEPSVKMYHKTGSTTGFGTYVVFIPKENI
ADC-73WDC73972.1_1415AbBOC      EEFSYPATLQTLLDSNSEQIVMKPNKVTAISKEPSVKMYHKTGSTTGFGTYVVFIPKENI
ADC-73WDC70172.1_1037AbBOC      EEFSYPATLQTLLDSNSEQIVMKPNKVTAISKEPSVKMYHKTGSTTGFGTYVVFIPKENI
DC-73WP_001211219.1_1101AbBOC    EEFSYPATLQTLLDSNSEQIVMKPNKVTAISKEPSVKMYHKTGSTTGFGTYVVFIPKENI
DC-73WP_001211219.1_1667AbBOC    EEFSYPATLQTLLDSNSEQIVMKPNKVTAISKEPSVKMYHKTGSTTGFGTYVVFIPKENI
ADC-73WP_001211219.1_287AbBOC    EEFSYPATLQTLLDSNSEQIVMKPNKVTAISKEPSVKMYHKTGSTTGFGTYVVFIPKENI
DC-73WP_001211219.1_3177AbBOC    EEFSYPATLQTLLDSNSEQIVMKPNKVTAISKEPSVKMYHKTGSTTGFGTYVVFIPKENI
DC-73WP_001211219.1_2501AbBOC    EEFSYPATLQTLLDSNSEQIVMKPNKVTAISKEPSVKMYHKTGSTTGFGTYVVFIPKENI
DC-73WP_001211219.1_2535AbBOC    EEFSYPATLQTLLDSNSEQIVMKPNKVTAISKEPSVKMYHKTGSTTGFGTYVVFIPKENI
DC-73WP_001211219.1_3150AbBOC    EEFSYPATLQTLLDSNSEQIVMKPNKVTAISKEPSVKMYHKTGSTTGFGTYVVFIPKENI
DC-73WP_001211219.1_3714AbBOC    EEFSYPATLQTLLDSNSEQIVMKPNKVTAISKEPSVKMYHKTGSTTGFGTYVVFIPKENI
ADC-73WP_001211219.1_722AbBOC    EEFSYPATLQTLLDSNSEQIVMKPNKVTAISKEPSVKMYHKTGSTTGFGTYVVFIPKENI
DC-73WP_001211219.1_3650AbBOC    EEFSYPATLQTLLDSNSEQIVMKPNKVTAISKEPSVKMYHKTGSTTGFGTYVVFIPKENI
ADC-73WP_001211219.1_568AbBOC    EEFSYPATLQTLLDSNSEQIVMKPNKVTAISKEPSVKMYHKTGSTTGFGTYVVFIPKENI
DC-73WP_001211219.1_3226AbBOC    EEFSYPATLQTLLDSNSEQIVMKPNKVTAISKEPSVKMYHKTGSTTGFGTYVVFIPKENI
ADC-73WP_001211219.1_556AbBOC    EEFSYPATLQTLLDSNSEQIVMKPNKVTAISKEPSVKMYHKTGSTTGFGTYVVFIPKENI
```

DC-73WP_001211219.1_2436AbBOC	EEFSYPATLQTLLDSNSEQIVMKPNKVTAISKEPSVKMYHKTGSTTGFGTYVVFIPKENI
DC-73WP_001211219.1_1029AbBOC	EEFSYPATLQTLLDSNSEQIVMKPNKVTAISKEPSVKMYHKTGSTTGFGTYVVFIPKENI
DC-73WP_001211219.1_1013AbBOC	EEFSYPATLQTLLDSNSEQIVMKPNKVTAISKEPSVKMYHKTGSTTGFGTYVVFIPKENI
ADC-73WP_001211219.1_906AbBCP	EEFSYPATLQTLLDSNSEQIVMKPNKVTAISKEPSVKMYHKTGSTTGFGTYVVFIPKENI
DC-73WP_001211219.1_2649AbBCP	EEFSYPATLQTLLDSNSEQIVMKPNKVTAISKEPSVKMYHKTGSTTGFGTYVVFIPKENI
DC-73WP_001211219.1_2693AbBCP	EEFSYPATLQTLLDSNSEQIVMKPNKVTAISKEPSVKMYHKTGSTTGFGTYVVFIPKENI
ADC-73AQU57925.1_2703AbBCP	EEFSYPATLQTLLDSNSEQIVMKPNKVTAISKEPSVKMYHKTGSTTGFGTYVVFIPKENI
DC-73WP_001211219.1_1026AbBCP	EEFSYPATLQTLLDSNSEQIVMKPNKVTAISKEPSVKMYHKTGSTTGFGTYVVFIPKENI
DC-73WP_001211219.1_1019AbBCP	EEFSYPATLQTLLDSNSEQIVMKPNKVTAISKEPSVKMYHKTGSTTGFGTYVVFIPKENI
DC-73WP_001211219.1_1024AbBCP	EEFSYPATLQTLLDSNSEQIVMKPNKVTAISKEPSVKMYHKTGSTTGFGTYVVFIPKENI
ADC-73WP_001211219.1_930AbBCP	EEFSYPATLQTLLDSNSEQIVMKPNKVTAISKEPSVKMYHKTGSTTGFGTYVVFIPKENI
DC-73WP_001211219.1_2609AbBCP	EEFSYPATLQTLLDSNSEQIVMKPNKVTAISKEPSVKMYHKTGSTTGFGTYVVFIPKENI
DC-73WP_001211219.1_3456AbBCP	EEFSYPATLQTLLDSNSEQIVMKPNKVTAISKEPSVKMYHKTGSTTGFGTYVVFIPKENI
DC-73WP_001211219.1_2614AbBCP	EEFSYPATLQTLLDSNSEQIVMKPNKVTAISKEPSVKMYHKTGSTTGFGTYVVFIPKENI
DC-73WP_001211219.1_1025AbBCP	EEFSYPATLQTLLDSNSEQIVMKPNKVTAISKEPSVKMYHKTGSTTGFGTYVVFIPKENI
DC-73WP_001211219.1_1030AbBCP	EEFSYPATLQTLLDSNSEQIVMKPNKVTAISKEPSVKMYHKTGSTTGFGTYVVFIPKENI
DC-73WP_001211219.1_1173AbBCP	EEFSYPATLQTLLDSNSEQIVMKPNKVTAISKEPSVKMYHKTGSTTGFGTYVVFIPKENI
DC-73WP_001211219.1_1021AbBCP	EEFSYPATLQTLLDSNSEQIVMKPNKVTAISKEPSVKMYHKTGSTTGFGTYVVFIPKENI
DC-73WP_001211219.1_2449AbBCP	EEFSYPATLQTLLDSNSEQIVMKPNKVTAISKEPSVKMYHKTGSTTGFGTYVVFIPKENI
DC-73WP_001211219.1_1176AbBCP	EEFSYPATLQTLLDSNSEQIVMKPNKVTAISKEPSVKMYHKTGSTTGFGTYVVFIPKENI
DC-73WP_001211219.1_1161AbBCP	EEFSYPATLQTLLDSNSEQIVMKPNKVTAISKEPSVKMYHKTGSTTGFGTYVVFIPKENI
DC-73WP_001211219.1_1070AbBCP	EEFSYPATLQTLLDSNSEQIVMKPNKVTAISKEPSVKMYHKTGSTTGFGTYVVFIPKENI
DC-73WP_001211219.1_1079AbBCP	EEFSYPATLQTLLDSNSEQIVMKPNKVTAISKEPSVKMYHKTGSTTGFGTYVVFIPKENI
DC-73WP_001211219.1_1078AbBCP	EEFSYPATLQTLLDSNSEQIVMKPNKVTAISKEPSVKMYHKTGSTTGFGTYVVFIPKENI
DC-73WP_001211219.1_1037AbBCP	EEFSYPATLQTLLDSNSEQIVMKPNKVTAISKEPSVKMYHKTGSTTGFGTYVVFIPKENI
DC-73WP_001211219.1_1035AbBCP	EEFSYPATLQTLLDSNSEQIVMKPNKVTAISKEPSVKMYHKTGSTTGFGTYVVFIPKENI
DC-73WP_001211219.1_1033AbBCP	EEFSYPATLQTLLDSNSEQIVMKPNKVTAISKEPSVKMYHKTGSTTGFGTYVVFIPKENI
DC-73WP_001211219.1_1041AbBCP	EEFSYPATLQTLLDSNSEQIVMKPNKVTAISKEPSVKMYHKTGSTTGFGTYVVFIPKENI
DC-73WP_001211219.1_1023AbBCP	EEFSYPATLQTLLDSNSEQIVMKPNKVTAISKEPSVKMYHKTGSTTGFGTYVVFIPKENI
ADC-73USX69547.1_2386AbBCP	EEFSYPATLQTLLDSNSEQIVMKPNKVTAISKEPSVKMYHKTGSTTGFGTYVVFIPKENI
DC-73WP_001211219.1_1034AbBCP	EEFSYPATLQTLLDSNSEQIVMKPNKVTAISKEPSVKMYHKTGSTTGFGTYVVFIPKENI
ADC-73WP_001211219.1_852AbBCP	EEFSYPATLQTLLDSNSEQIVMKPNKVTAISKEPSVKMYHKTGSTTGFGTYVVFIPKENI
DC-73WP_001211219.1_3070AbBCP	EEFSYPATLQTLLDSNSEQIVMKPNKVTAISKEPSVKMYHKTGSTTGFGTYVVFIPKENI
ADC-73WBS60953.1_1026AbBCP	EEFSYPATLQTLLDSNSEQIVMKPNKVTAISKEPSVKMYHKTGSTTGFGTYVVFIPKENI
ADC-73WDC39660.1_1023AbBCP	EEFSYPATLQTLLDSNSEQIVMKPNKVTAISKEPSVKMYHKTGSTTGFGTYVVFIPKENI
ADC-73WDC73972.1_1415AbBCP	EEFSYPATLQTLLDSNSEQIVMKPNKVTAISKEPSVKMYHKTGSTTGFGTYVVFIPKENI
ADC-73WDC70172.1_1037AbBCP	EEFSYPATLQTLLDSNSEQIVMKPNKVTAISKEPSVKMYHKTGSTTGFGTYVVFIPKENI
DC-73WP_001211219.1_1101AbBCP	EEFSYPATLQTLLDSNSEQIVMKPNKVTAISKEPSVKMYHKTGSTTGFGTYVVFIPKENI
DC-73WP_001211219.1_1667AbBCP	EEFSYPATLQTLLDSNSEQIVMKPNKVTAISKEPSVKMYHKTGSTTGFGTYVVFIPKENI
ADC-73WP_001211219.1_287AbBCP	EEFSYPATLQTLLDSNSEQIVMKPNKVTAISKEPSVKMYHKTGSTTGFGTYVVFIPKENI
DC-73WP_001211219.1_3177AbBCP	EEFSYPATLQTLLDSNSEQIVMKPNKVTAISKEPSVKMYHKTGSTTGFGTYVVFIPKENI
DC-73WP_001211219.1_2501AbBCP	EEFSYPATLQTLLDSNSEQIVMKPNKVTAISKEPSVKMYHKTGSTTGFGTYVVFIPKENI
DC-73WP_001211219.1_2535AbBCP	EEFSYPATLQTLLDSNSEQIVMKPNKVTAISKEPSVKMYHKTGSTTGFGTYVVFIPKENI
DC-73WP_001211219.1_3150AbBCP	EEFSYPATLQTLLDSNSEQIVMKPNKVTAISKEPSVKMYHKTGSTTGFGTYVVFIPKENI
DC-73WP_001211219.1_3714AbBCP	EEFSYPATLQTLLDSNSEQIVMKPNKVTAISKEPSVKMYHKTGSTTGFGTYVVFIPKENI
ADC-73WP_001211219.1_722AbBCP	EEFSYPATLQTLLDSNSEQIVMKPNKVTAISKEPSVKMYHKTGSTTGFGTYVVFIPKENI
DC-73WP_001211219.1_3650AbBCP	EEFSYPATLQTLLDSNSEQIVMKPNKVTAISKEPSVKMYHKTGSTTGFGTYVVFIPKENI
ADC-73WP_001211219.1_568AbBCP	EEFSYPATLQTLLDSNSEQIVMKPNKVTAISKEPSVKMYHKTGSTTGFGTYVVFIPKENI
DC-73WP_001211219.1_3226AbBCP	EEFSYPATLQTLLDSNSEQIVMKPNKVTAISKEPSVKMYHKTGSTTGFGTYVVFIPKENI
ADC-73WP_001211219.1_556AbBCP	EEFSYPATLQTLLDSNSEQIVMKPNKVTAISKEPSVKMYHKTGSTTGFGTYVVFIPKENI
DC-73WP_001211219.1_2436AbBCP	EEFSYPATLQTLLDSNSEQIVMKPNKVTAISKEPSVKMYHKTGSTTGFGTYVVFIPKENI
DC-73WP_001211219.1_1029AbBCP	EEFSYPATLQTLLDSNSEQIVMKPNKVTAISKEPSVKMYHKTGSTTGFGTYVVFIPKENI
DC-73WP_001211219.1_1013AbBCP	EEFSYPATLQTLLDSNSEQIVMKPNKVTAISKEPSVKMYHKTGSTTGFGTYVVFIPKENI
ADC-73BDE18748.1_1038AbBCP	EEFSYPATLQTLLDSNSEQIVMKPNKVTAISKEPSVKMYHKTGSTTGFGTYVVFIPKENI
DC-115WP_001211215.1_2833AbOC	EEFSYPATLQTLLDSNSEQIVMKPNKVTAISKEPSVKMYHKTGSTTGFGTYVVFIPKENI
C-115WP_001211215.1_1156AbBOC	EEFSYPATLQTLLDSNSEQIVMKPNKVTAISKEPSVKMYHKTGSTTGFGTYVVFIPKENI
C-115WP_001211215.1_1156AbBCP	EEFSYPATLQTLLDSNSEQIVMKPNKVTAISKEPSVKMYHKTGSTTGFGTYVVFIPKENI
ADC-5WP_038405930.1_2542AbOC	EEFSYPATLQTLLDSNSEQIVMKPNKVTAISKEPSVKMYHKTGSTNGFGTYVVFIPKENI
ADC-5WP_038405930.1_1113AbBOC	EEFSYPATLQTLLDSNSEQIVMKPNKVTAISKEPSVKMYHKTGSTNGFGTYVVFIPKENI
ADC-5WP_038405930.1_1113AbBCP	EEFSYPATLQTLLDSNSEQIVMKPNKVTAISKEPSVKMYHKTGSTNGFGTYVVFIPKENI
C-236WP_176673601.1_1026AbBOC	EEFSYPATLQTLLDSNSEQIVMKPNKVTAISKEPSVKMYHKTGSTTGFGTYVVFIPKENI
C-236WP_176673601.1_1026AbBCP	EEFSYPATLQTLLDSNSEQIVMKPNKVTAISKEPSVKMYHKTGSTTGFGTYVVFIPKENI
DC-217WP_060470109.1_3584AbOC	EEFSYPATLQTLLDSNSEQIVMKPNKVTAISKEPSVKMYHKTGSTTGFGTYVVFIPKENI
ADC-217USX66614.1_2290AbBOC	EEFSYPATLQTLLDSNSEQIVMKPNKVTAISKEPSVKMYHKTGSTTGFGTYVVFIPKENI
ADC-217USX66614.1_2290AbBCP	EEFSYPATLQTLLDSNSEQIVMKPNKVTAISKEPSVKMYHKTGSTTGFGTYVVFIPKENI
ADC-247WP_032058755.1_382AbOC	EEFSYPATLQTLLDSNSEQIVMKPNKVTAISKEPSVKMYHKTGSTNGFGTYVVFIPKENI
C-247WP_032058755.1_1936AbBOC	EEFSYPATLQTLLDSNSEQIVMKPNKVTAISKEPSVKMYHKTGSTNGFGTYVVFIPKENI
C-247WP_032058755.1_1936AbBCP	EEFSYPATLQTLLDSNSEQIVMKPNKVTAISKEPSVKMYHKTGSTNGFGTYVVFIPKENI
DC-203WP_032000414.1_2940AbOC	EEFSYPATLQTLLDSNSEQIVMKPNKVSAISKEPSVKMYHKTGSTNGFGTYVVFIPKENI
C-203WP_032000414.1_1062AbBOC	EEFSYPATLQTLLDSNSEQIVMKPNKVSAISKEPSVKMYHKTGSTNGFGTYVVFIPKENI
DC-203WP_032000414.1_129AbBOC	EEFSYPATLQTLLDSNSEQIVMKPNKVSAISKEPSVKMYHKTGSTNGFGTYVVFIPKENI
C-203WP_032000414.1_2518AbBOC	EEFSYPATLQTLLDSNSEQIVMKPNKVSAISKEPSVKMYHKTGSTNGFGTYVVFIPKENI
C-203WP_032000414.1_1062AbBCP	EEFSYPATLQTLLDSNSEQIVMKPNKVSAISKEPSVKMYHKTGSTNGFGTYVVFIPKENI
DC-203WP_032000414.1_129AbBCP	EEFSYPATLQTLLDSNSEQIVMKPNKVSAISKEPSVKMYHKTGSTNGFGTYVVFIPKENI
C-203WP_032000414.1_2518AbBCP	EEFSYPATLQTLLDSNSEQIVMKPNKVSAISKEPSVKMYHKTGSTNGFGTYVVFIPKENI
DC-152WP_001211233.1_3734AbOC	EEFSYPATLQTLLDSNSEQIVMKPNKVTAISKEPSVKMYHKTGSTSGFGTYVVFIPKENI
C-152WP_001211233.1_1080AbBOC	EEFSYPATLQTLLDSNSEQIVMKPNKVTAISKEPSVKMYHKTGSTSGFGTYVVFIPKENI
DC-152WP_001211233.1_491AbBOC	EEFSYPATLQTLLDSNSEQIVMKPNKVTAISKEPSVKMYHKTGSTSGFGTYVVFIPKENI
ADC-152WP_001211233.1_46AbBOC	EEFSYPATLQTLLDSNSEQIVMKPNKVTAISKEPSVKMYHKTGSTSGFGTYVVFIPKENI
C-152WP_001211233.1_1545AbBOC	EEFSYPATLQTLLDSNSEQIVMKPNKVTAISKEPSVKMYHKTGSTSGFGTYVVFIPKENI
ADC-152WBS86980.1_1289AbBOC	EEFSYPATLQTLLDSNSEQIVMKPNKVTAISKEPSVKMYHKTGSTSGFGTYVVFIPKENI
ADC-152WBS81072.1_2581AbBOC	EEFSYPATLQTLLDSNSEQIVMKPNKVTAISKEPSVKMYHKTGSTSGFGTYVVFIPKENI
ADC-152WBS88504.1_2711AbBOC	EEFSYPATLQTLLDSNSEQIVMKPNKVTAISKEPSVKMYHKTGSTSGFGTYVVFIPKENI
ADC-152WBS92168.1_2401AbBOC	EEFSYPATLQTLLDSNSEQIVMKPNKVTAISKEPSVKMYHKTGSTSGFGTYVVFIPKENI
ADC-152WBS79617.1_1289AbBOC	EEFSYPATLQTLLDSNSEQIVMKPNKVTAISKEPSVKMYHKTGSTSGFGTYVVFIPKENI
C-152WP_001211233.1_1080AbBCP	EEFSYPATLQTLLDSNSEQIVMKPNKVTAISKEPSVKMYHKTGSTSGFGTYVVFIPKENI
DC-152WP_001211233.1_491AbBCP	EEFSYPATLQTLLDSNSEQIVMKPNKVTAISKEPSVKMYHKTGSTSGFGTYVVFIPKENI
ADC-152WP_001211233.1_46AbBCP	EEFSYPATLQTLLDSNSEQIVMKPNKVTAISKEPSVKMYHKTGSTSGFGTYVVFIPKENI
C-152WP_001211233.1_1545AbBCP	EEFSYPATLQTLLDSNSEQIVMKPNKVTAISKEPSVKMYHKTGSTSGFGTYVVFIPKENI
ADC-152WBS86980.1_1289AbBCP	EEFSYPATLQTLLDSNSEQIVMKPNKVTAISKEPSVKMYHKTGSTSGFGTYVVFIPKENI
ADC-152WBS81072.1_2581AbBCP	EEFSYPATLQTLLDSNSEQIVMKPNKVTAISKEPSVKMYHKTGSTSGFGTYVVFIPKENI
ADC-152WBS88504.1_2711AbBCP	EEFSYPATLQTLLDSNSEQIVMKPNKVTAISKEPSVKMYHKTGSTSGFGTYVVFIPKENI
ADC-152WBS92168.1_2401AbBCP	EEFSYPATLQTLLDSNSEQIVMKPNKVTAISKEPSVKMYHKTGSTSGFGTYVVFIPKENI
ADC-152WBS79617.1_1289AbBCP	EEFSYPATLQTLLDSNSEQIVMKPNKVTAISKEPSVKMYHKTGSTSGFGTYVVFIPKENI
ADC-52WP_001211232.1_990AbOC	EEFSYPATLQTLLDSNSEQIVMKPNKVTAISKEPSVKMYHKTGSTNGFGTYVVFIPKENI
DC-52WP_001211232.1_1087AbBOC	EEFSYPATLQTLLDSNSEQIVMKPNKVTAISKEPSVKMYHKTGSTNGFGTYVVFIPKENI
DC-52WP_001211232.1_1087AbBCP	EEFSYPATLQTLLDSNSEQIVMKPNKVTAISKEPSVKMYHKTGSTNGFGTYVVFIPKENI
C-163WP_057093064.1_2397AbBOC	EEFSYPAPLQTLLDSNSEQIVMKPNKVTAISKEPSVKMYHKTGSTNGFGTYVVFIPKENI
C-163WP_057093064.1_2397AbBCP	EEFSYPAPLQTLLDSNSEQIVMKPNKVTAISKEPSVKMYHKTGSTNGFGTYVVFIPKENI
ADC-33WP_001211220.1_2277AbOC	EEFSYPATLQTLLDSNSEQIVMKPNKVTAISKEPSVKMYHKTGSTNGFGTYVVFIPKENI
DC-33WP_001211220.1_1044AbBOC	EEFSYPATLQTLLDSNSEQIVMKPNKVTAISKEPSVKMYHKTGSTNGFGTYVVFIPKENI
DC-33WP_001211220.1_2452AbBOC	EEFSYPATLQTLLDSNSEQIVMKPNKVTAISKEPSVKMYHKTGSTNGFGTYVVFIPKENI
DC-33WP_001211220.1_1046AbBOC	EEFSYPATLQTLLDSNSEQIVMKPNKVTAISKEPSVKMYHKTGSTNGFGTYVVFIPKENI
DC-33WP_001211220.1_1025AbBOC	EEFSYPATLQTLLDSNSEQIVMKPNKVTAISKEPSVKMYHKTGSTNGFGTYVVFIPKENI
DC-33WP_001211220.1_1026AbBOC	EEFSYPATLQTLLDSNSEQIVMKPNKVTAISKEPSVKMYHKTGSTNGFGTYVVFIPKENI
DC-33WP_001211220.1_1033AbBOC	EEFSYPATLQTLLDSNSEQIVMKPNKVTAISKEPSVKMYHKTGSTNGFGTYVVFIPKENI
DC-33WP_001211220.1_1035AbBOC	EEFSYPATLQTLLDSNSEQIVMKPNKVTAISKEPSVKMYHKTGSTNGFGTYVVFIPKENI
DC-33WP_001211220.1_1041AbBOC	EEFSYPATLQTLLDSNSEQIVMKPNKVTAISKEPSVKMYHKTGSTNGFGTYVVFIPKENI
DC-33WP_001211220.1_1044AbBCP	EEFSYPATLQTLLDSNSEQIVMKPNKVTAISKEPSVKMYHKTGSTNGFGTYVVFIPKENI
DC-33WP_001211220.1_2452AbBCP	EEFSYPATLQTLLDSNSEQIVMKPNKVTAISKEPSVKMYHKTGSTNGFGTYVVFIPKENI
DC-33WP_001211220.1_1046AbBCP	EEFSYPATLQTLLDSNSEQIVMKPNKVTAISKEPSVKMYHKTGSTNGFGTYVVFIPKENI
DC-33WP_001211220.1_1025AbBCP	EEFSYPATLQTLLDSNSEQIVMKPNKVTAISKEPSVKMYHKTGSTNGFGTYVVFIPKENI
DC-33WP_001211220.1_1026AbBCP	EEFSYPATLQTLLDSNSEQIVMKPNKVTAISKEPSVKMYHKTGSTNGFGTYVVFIPKENI
DC-33WP_001211220.1_1033AbBCP	EEFSYPATLQTLLDSNSEQIVMKPNKVTAISKEPSVKMYHKTGSTNGFGTYVVFIPKENI
DC-33WP_001211220.1_1035AbBCP	EEFSYPATLQTLLDSNSEQIVMKPNKVTAISKEPSVKMYHKTGSTNGFGTYVVFIPKENI
DC-33WP_001211220.1_1041AbBCP	EEFSYPATLQTLLDSNSEQIVMKPNKVTAISKEPSVKMYHKTGSTNGFGTYVVFIPKENI
C-212WP_031975357.1_1297AbBOC	EEFSYPATLQTLLDSNSEQIVMKPNKVTAISKEPSVKMYHKTGSTNGFGTYVVFIPKENI
DC-212WP_031975357.1_501AbBOC	EEFSYPATLQTLLDSNSEQIVMKPNKVTAISKEPSVKMYHKTGSTNGFGTYVVFIPKENI
C-212WP_031975357.1_1297AbBCP	EEFSYPATLQTLLDSNSEQIVMKPNKVTAISKEPSVKMYHKTGSTNGFGTYVVFIPKENI
DC-212WP_031975357.1_501AbBCP	EEFSYPATLQTLLDSNSEQIVMKPNKVTAISKEPSVKMYHKTGSTNGFGTYVVFIPKENI
C-182WP_057691114.1_1055AbBOC	EEFSYPATLQTLLDSNSEQIVMKPNKVTAISKEPSVKMYHKTGSTTGFGTYVVFIPKENI
C-182WP_057691114.1_1055AbBCP	EEFSYPATLQTLLDSNSEQIVMKPNKVTAISKEPSVKMYHKTGSTTGFGTYVVFIPKENI
DC-81WP_059262723.1_1208AbBOC	EEFSYPATLQTLLDSNSEQIVMKPNKVTAISKEPSVKMYHKTGSTNGFGTYVVFIPKENI
DC-81WP_059262723.1_2841AbBOC	EEFSYPATLQTLLDSNSEQIVMKPNKVTAISKEPSVKMYHKTGSTNGFGTYVVFIPKENI
DC-81WP_059262723.1_1208AbBCP	EEFSYPATLQTLLDSNSEQIVMKPNKVTAISKEPSVKMYHKTGSTNGFGTYVVFIPKENI
DC-81WP_059262723.1_2841AbBCP	EEFSYPATLQTLLDSNSEQIVMKPNKVTAISKEPSVKMYHKTGSTNGFGTYVVFIPKENI
ADC-176WP_001159761.1_11AbOC	EEFSYPATLQTLLDSNSEQIVMKPNKVTAISKEPSVKMYHKTGSTNGFGTYVVFIPKENI
DC-176WP_001159761.1_3466AbOC	EEFSYPATLQTLLDSNSEQIVMKPNKVTAISKEPSVKMYHKTGSTNGFGTYVVFIPKENI
ADC-176WP_001159761.1_93AbBOP	EEFSYPATLQTLLDSNSEQIVMKPNKVTAISKEPSVKMYHKTGSTNGFGTYVVFIPKENI
DC-176WP_001159761.1_247AbBOC	EEFSYPATLQTLLDSNSEQIVMKPNKVTAISKEPSVKMYHKTGSTNGFGTYVVFIPKENI
ADC-176WP_001159761.1_9AbBOC	EEFSYPATLQTLLDSNSEQIVMKPNKVTAISKEPSVKMYHKTGSTNGFGTYVVFIPKENI
C-176WP_001159761.1_2982AbBOC	EEFSYPATLQTLLDSNSEQIVMKPNKVTAISKEPSVKMYHKTGSTNGFGTYVVFIPKENI
ADC-176QLG80706.1_1971AbBOC	EEFSYPATLQTLLDSNSEQIVMKPNKVTAISKEPSVKMYHKTGSTNGFGTYVVFIPKENI
C-176WP_001159761.1_2851AbBOC	EEFSYPATLQTLLDSNSEQIVMKPNKVTAISKEPSVKMYHKTGSTNGFGTYVVFIPKENI
C-176WP_001159761.1_1220AbBOC	EEFSYPATLQTLLDSNSEQIVMKPNKVTAISKEPSVKMYHKTGSTNGFGTYVVFIPKENI
DC-176WP_001159761.1_386AbBOC	EEFSYPATLQTLLDSNSEQIVMKPNKVTAISKEPSVKMYHKTGSTNGFGTYVVFIPKENI
ADC-176WP_001159761.1_11AbBOC	EEFSYPATLQTLLDSNSEQIVMKPNKVTAISKEPSVKMYHKTGSTNGFGTYVVFIPKENI
DC-176WP_001159761.1_247AbBCP	EEFSYPATLQTLLDSNSEQIVMKPNKVTAISKEPSVKMYHKTGSTNGFGTYVVFIPKENI
ADC-176WP_001159761.1_9AbBCP	EEFSYPATLQTLLDSNSEQIVMKPNKVTAISKEPSVKMYHKTGSTNGFGTYVVFIPKENI
C-176WP_001159761.1_2982AbBCP	EEFSYPATLQTLLDSNSEQIVMKPNKVTAISKEPSVKMYHKTGSTNGFGTYVVFIPKENI
ADC-176QLG80706.1_1971AbBCP	EEFSYPATLQTLLDSNSEQIVMKPNKVTAISKEPSVKMYHKTGSTNGFGTYVVFIPKENI
C-176WP_001159761.1_2851AbBCP	EEFSYPATLQTLLDSNSEQIVMKPNKVTAISKEPSVKMYHKTGSTNGFGTYVVFIPKENI
C-176WP_001159761.1_1220AbBCP	EEFSYPATLQTLLDSNSEQIVMKPNKVTAISKEPSVKMYHKTGSTNGFGTYVVFIPKENI
DC-176WP_001159761.1_386AbBCP	EEFSYPATLQTLLDSNSEQIVMKPNKVTAISKEPSVKMYHKTGSTNGFGTYVVFIPKENI
ADC-176WP_001159761.1_11AbBCP	EEFSYPATLQTLLDSNSEQIVMKPNKVTAISKEPSVKMYHKTGSTNGFGTYVVFIPKENI
ADC-199WP_114166683.1_169AbOC	EEFSYPATLQTLLDSNSEQIVMKPNKVTAISKEPSVKMYHKTGSTTGFGTYVVFIPKENI
DC-199WP_114166683.1_267AbBOC	EEFSYPATLQTLLDSNSEQIVMKPNKVTAISKEPSVKMYHKTGSTTGFGTYVVFIPKENI
DC-199WP_114166683.1_267AbBCP	EEFSYPATLQTLLDSNSEQIVMKPNKVTAISKEPSVKMYHKTGSTTGFGTYVVFIPKENI
acc	

8FQV_1|Chains — β15 (340) α14 (350, 360)

Sequence	340 … 360
8FQV_1|Chains	GLVMLTNKRIPNEERIKAAYAVLNAIKK
-154OCUAc18_RS13275_2608AbBOC	GLVMLTNKRIPNEERIKAAYAVLNAIKE
-154OCUAc18_RS13275_2608AbBCP	GLVMLTNKRIPNEERIKAAYAVLNAIKE
ADC-73BDE22771.1_1271AbBCP	GLVMLTNKRIPNEERIKAAYAVLNAIKK
DC-87WP_068981619.1_1049AbBOC	GLVMLTNKRIPNEERIKAAYAVLNAIKK
DC-87WP_068981619.1_1049AbBCP	GLVMLTNKRIPNEERIKAAYAVLNAIKK
C-249WP_001211200.1_1039AbBOC	GLVMLTNKRIPNEERIKAAYTVLNAIKK
C-249WP_001211200.1_1039AbBCP	GLVMLTNKRIPNEERIKAAYTVLNAIKK
DC-32WP_004739487.1_2518AbBOC	GLVMLTNKRIPNEERIKAAYAVLSAIKK
DC-32WP_004739487.1_3158AbBOC	GLVMLTNKRIPNEERIKAAYAVLSAIKK
DC-32WP_004739487.1_2392AbBOC	GLVMLTNKRIPNEERIKAAYAVLSAIKK
DC-32WP_004739487.1_2378AbBOC	GLVMLTNKRIPNEERIKAAYAVLSAIKK
DC-32WP_004739487.1_2518AbBCP	GLVMLTNKRIPNEERIKAAYAVLSAIKK
DC-32WP_004739487.1_3158AbBCP	GLVMLTNKRIPNEERIKAAYAVLSAIKK
DC-32WP_004739487.1_2392AbBCP	GLVMLTNKRIPNEERIKAAYAVLSAIKK
DC-32WP_004739487.1_2378AbBCP	GLVMLTNKRIPNEERIKAAYAVLSAIKK
C-248WP_021511005.1_1080AbBOC	GLVMLTNKRIPNEERIKAAYTVLNAIKK
C-248WP_021511005.1_1080AbBCP	GLVMLTNKRIPNEERIKAAYTVLNAIKK
ADC-191UIY52419.1_2933AbOC	GLVMLTNKRIPNEERIKAAYAVLDAIKK
DC-191WP_003112187.1_3072AbOC	GLVMLTNKRIPNEERIKAAYAVLDAIKK
DC-191WP_003112187.1_2848AbOC	GLVMLTNKRIPNEERIKAAYAVLDAIKK
C-191WP_003112187.1_2694AbBOC	GLVMLTNKRIPNEERIKAAYAVLDAIKK
C-191WP_003112187.1_2694AbBCP	GLVMLTNKRIPNEERIKAAYAVLDAIKK
C-169WP_001211212.1_2709AbBOC	GLVMLTNKRIPNEERIKAAYAVLSAIKK
C-169WP_001211212.1_2457AbBOC	GLVMLTNKRIPNEERIKAAYAVLSAIKK
ADC-169UYQ18775.1_1073AbBOC	GLVMLTNKRIPNEERIKAAYAVLSAIKK
ADC-169UYQ29710.1_1071AbBOC	GLVMLTNKRIPNEERIKAAYAVLSAIKK
ADC-169UYQ59091.1_1072AbBOC	GLVMLTNKRIPNEERIKAAYAVLSAIKK
ADC-169WGT82884.1_1015AbBOC	GLVMLTNKRIPNEERIKAAYAVLSAIKK
C-169WP_001211212.1_2709AbBCP	GLVMLTNKRIPNEERIKAAYAVLSAIKK
C-169WP_001211212.1_2457AbBCP	GLVMLTNKRIPNEERIKAAYAVLSAIKK
ADC-169UYQ18775.1_1073AbBCP	GLVMLTNKRIPNEERIKAAYAVLSAIKK
ADC-169UYQ29710.1_1071AbBCP	GLVMLTNKRIPNEERIKAAYAVLSAIKK
ADC-169UYQ59091.1_1072AbBCP	GLVMLTNKRIPNEERIKAAYAVLSAIKK
ADC-169WGT82884.1_1015AbBCP	GLVMLTNKRIPNEERIKAAYAVLSAIKK
DC-99WP_068981630.1_2482AbBOC	GLVMLTNKRIPNEERIKAAYAVLNAIKK
DC-99WP_068981630.1_2482AbBCP	GLVMLTNKRIPNEERIKAAYAVLNAIKK
DC-222WP_031960999.1_1504AbOC	GLVMLTNKRIPNEERIKAAYAVLNAIKK
ADC-222UYP92921.1_1090AbBOC	GLVMLTNKRIPNEERIKAAYAVLNAIKK
ADC-222UYQ05421.1_2617AbBOC	GLVMLTNKRIPNEERIKAAYAVLNAIKK
ADC-222UYQ22523.1_1090AbBOC	GLVMLTNKRIPNEERIKAAYAVLNAIKK
ADC-222UYQ26181.1_1089AbBOC	GLVMLTNKRIPNEERIKAAYAVLNAIKK
ADC-222UYQ33449.1_1092AbBOC	GLVMLTNKRIPNEERIKAAYAVLNAIKK
ADC-222UYQ42503.1_2616AbBOC	GLVMLTNKRIPNEERIKAAYAVLNAIKK
ADC-222UYQ37112.1_1090AbBOC	GLVMLTNKRIPNEERIKAAYAVLNAIKK
ADC-222UYQ46167.1_2615AbBOC	GLVMLTNKRIPNEERIKAAYAVLNAIKK
ADC-222UYQ51934.1_1091AbBOC	GLVMLTNKRIPNEERIKAAYAVLNAIKK
ADC-222UYQ55558.1_1091AbBOC	GLVMLTNKRIPNEERIKAAYAVLNAIKK
ADC-222WBS68552.1_1096AbBOC	GLVMLTNKRIPNEERIKAAYAVLNAIKK
ADC-222WBS55174.1_2602AbBOC	GLVMLTNKRIPNEERIKAAYAVLNAIKK
ADC-222WBS51522.1_2600AbBOC	GLVMLTNKRIPNEERIKAAYAVLNAIKK
ADC-222UYP92921.1_1090AbBCP	GLVMLTNKRIPNEERIKAAYAVLNAIKK
ADC-222UYQ05421.1_2617AbBCP	GLVMLTNKRIPNEERIKAAYAVLNAIKK
ADC-222UYQ22523.1_1090AbBCP	GLVMLTNKRIPNEERIKAAYAVLNAIKK
ADC-222UYQ26181.1_1089AbBCP	GLVMLTNKRIPNEERIKAAYAVLNAIKK
ADC-222UYQ33449.1_1092AbBCP	GLVMLTNKRIPNEERIKAAYAVLNAIKK
ADC-222UYQ42503.1_2616AbBCP	GLVMLTNKRIPNEERIKAAYAVLNAIKK
ADC-222UYQ37112.1_1090AbBCP	GLVMLTNKRIPNEERIKAAYAVLNAIKK
ADC-222UYQ46167.1_2615AbBCP	GLVMLTNKRIPNEERIKAAYAVLNAIKK
ADC-222UYQ51934.1_1091AbBCP	GLVMLTNKRIPNEERIKAAYAVLNAIKK
ADC-222UYQ55558.1_1091AbBCP	GLVMLTNKRIPNEERIKAAYAVLNAIKK
ADC-222WBS68552.1_1096AbBCP	GLVMLTNKRIPNEERIKAAYAVLNAIKK
ADC-222WBS55174.1_2602AbBCP	GLVMLTNKRIPNEERIKAAYAVLNAIKK
ADC-222WBS51522.1_2600AbBCP	GLVMLTNKRIPNEERIKAAYAVLNAIKK
ADC-26WP_001211238.1_141AbOC	GLVMLTNKRIPNEERIKAAYAVLNAIKK
ADC-26WP_001211238.1_142AbOC	GLVMLTNKRIPNEERIKAAYAVLNAIKK
ADC-26WP_001211238.1_1AbOC	GLVMLTNKRIPNEERIKAAYAVLNAIKK
ADC-26WP_001211238.1_1113AbOC	GLVMLTNKRIPNEERIKAAYAVLNAIKK
ADC-26WP_001211238.1_1112AbOC	GLVMLTNKRIPNEERIKAAYAVLNAIKK
DC-26WP_001211238.1_1105AbBOC	GLVMLTNKRIPNEERIKAAYAVLNAIKK
DC-26WP_001211238.1_1111AbBOC	GLVMLTNKRIPNEERIKAAYAVLNAIKK
ADC-26WP_001211238.1_503AbBOC	GLVMLTNKRIPNEERIKAAYAVLNAIKK
DC-26WP_001211238.1_2774AbBOC	GLVMLTNKRIPNEERIKAAYAVLNAIKK
DC-26WP_001211238.1_1064AbBOC	GLVMLTNKRIPNEERIKAAYAVLNAIKK
DC-26WP_001211238.1_2484AbBOC	GLVMLTNKRIPNEERIKAAYAVLNAIKK
DC-26WP_001211238.1_3858AbBOC	GLVMLTNKRIPNEERIKAAYAVLNAIKK
ADC-26WP_001211238.1_142AbBOC	GLVMLTNKRIPNEERIKAAYAVLNAIKK
DC-26WP_001211238.1_1085AbBOC	GLVMLTNKRIPNEERIKAAYAVLNAIKK
DC-26WP_001211238.1_2525AbBOC	GLVMLTNKRIPNEERIKAAYAVLNAIKK
DC-26WP_001211238.1_2477AbBOC	GLVMLTNKRIPNEERIKAAYAVLNAIKK
DC-26WP_001211238.1_1069AbBOC	GLVMLTNKRIPNEERIKAAYAVLNAIKK
DC-26WP_001211238.1_1029AbBOC	GLVMLTNKRIPNEERIKAAYAVLNAIKK
DC-26WP_001211238.1_2443AbBOC	GLVMLTNKRIPNEERIKAAYAVLNAIKK
DC-26WP_001211238.1_1149AbBOC	GLVMLTNKRIPNEERIKAAYAVLNAIKK
DC-26WP_001211238.1_1148AbBOC	GLVMLTNKRIPNEERIKAAYAVLNAIKK
DC-26WP_001211238.1_1374AbBOC	GLVMLTNKRIPNEERIKAAYAVLNAIKK
DC-26WP_001211238.1_2862AbBOC	GLVMLTNKRIPNEERIKAAYAVLNAIKK
DC-26WP_001211238.1_2629AbBOC	GLVMLTNKRIPNEERIKAAYAVLNAIKK
DC-26WP_001211238.1_1355AbBOC	GLVMLTNKRIPNEERIKAAYAVLNAIKK
DC-26WP_001211238.1_3092AbBOC	GLVMLTNKRIPNEERIKAAYAVLNAIKK
DC-26WP_001211238.1_1086AbBOC	GLVMLTNKRIPNEERIKAAYAVLNAIKK
DC-26WP_001211238.1_1016AbBOC	GLVMLTNKRIPNEERIKAAYAVLNAIKK
ADC-26UUG48795.1_2867AbBOC	GLVMLTNKRIPNEERIKAAYAVLNAIKK
DC-26WP_001211238.1_2535AbBOC	GLVMLTNKRIPNEERIKAAYAVLNAIKK
ADC-26WEN46767.1_1086AbBOC	GLVMLTNKRIPNEERIKAAYAVLNAIKK
ADC-26WEX48630.1_1270AbBOC	GLVMLTNKRIPNEERIKAAYAVLNAIKK
ADC-26WGE92537.1_2034AbBOC	GLVMLTNKRIPNEERIKAAYAVLNAIKK
DC-26WP_001211238.1_1105AbBCP	GLVMLTNKRIPNEERIKAAYAVLNAIKK
DC-26WP_001211238.1_1111AbBCP	GLVMLTNKRIPNEERIKAAYAVLNAIKK
ADC-26WP_001211238.1_503AbBCP	GLVMLTNKRIPNEERIKAAYAVLNAIKK
DC-26WP_001211238.1_2774AbBCP	GLVMLTNKRIPNEERIKAAYAVLNAIKK
DC-26WP_001211238.1_1064AbBCP	GLVMLTNKRIPNEERIKAAYAVLNAIKK
DC-26WP_001211238.1_2484AbBCP	GLVMLTNKRIPNEERIKAAYAVLNAIKK
DC-26WP_001211238.1_3858AbBCP	GLVMLTNKRIPNEERIKAAYAVLNAIKK
ADC-26WP_001211238.1_142AbBCP	GLVMLTNKRIPNEERIKAAYAVLNAIKK
DC-26WP_001211238.1_1085AbBCP	GLVMLTNKRIPNEERIKAAYAVLNAIKK
DC-26WP_001211238.1_2525AbBCP	GLVMLTNKRIPNEERIKAAYAVLNAIKK
DC-26WP_001211238.1_2477AbBCP	GLVMLTNKRIPNEERIKAAYAVLNAIKK
DC-26WP_001211238.1_1069AbBCP	GLVMLTNKRIPNEERIKAAYAVLNAIKK
DC-26WP_001211238.1_1029AbBCP	GLVMLTNKRIPNEERIKAAYAVLNAIKK
DC-26WP_001211238.1_2443AbBCP	GLVMLTNKRIPNEERIKAAYAVLNAIKK
DC-26WP_001211238.1_1149AbBCP	GLVMLTNKRIPNEERIKAAYAVLNAIKK
DC-26WP_001211238.1_1148AbBCP	GLVMLTNKRIPNEERIKAAYAVLNAIKK
DC-26WP_001211238.1_1374AbBCP	GLVMLTNKRIPNEERIKAAYAVLNAIKK
DC-26WP_001211238.1_2862AbBCP	GLVMLTNKRIPNEERIKAAYAVLNAIKK
DC-26WP_001211238.1_2629AbBCP	GLVMLTNKRIPNEERIKAAYAVLNAIKK
DC-26WP_001211238.1_1355AbBCP	GLVMLTNKRIPNEERIKAAYAVLNAIKK
DC-26WP_001211238.1_3092AbBCP	GLVMLTNKRIPNEERIKAAYAVLNAIKK
DC-26WP_001211238.1_1086AbBCP	GLVMLTNKRIPNEERIKAAYAVLNAIKK
DC-26WP_001211238.1_1016AbBCP	GLVMLTNKRIPNEERIKAAYAVLNAIKK
ADC-26UUG48795.1_2867AbBCP	GLVMLTNKRIPNEERIKAAYAVLNAIKK
DC-26WP_001211238.1_2535AbBCP	GLVMLTNKRIPNEERIKAAYAVLNAIKK
ADC-26WEN46767.1_1086AbBCP	GLVMLTNKRIPNEERIKAAYAVLNAIKK
ADC-26WEX48630.1_1270AbBCP	GLVMLTNKRIPNEERIKAAYAVLNAIKK
ADC-26WGE92537.1_2034AbBCP	GLVMLTNKRIPNEERIKAAYAVLNAIKK
C-243WP_001211199.1_2530AbBOC	GLVMLTNKRIPNEERIKAAYAVLSAIKK
C-243WP_001211199.1_2530AbBCP	GLVMLTNKRIPNEERIKAAYAVLSAIKK
ADC-74WP_001211203.1_938AbBOC	GLVMLTNKRIPNEERIKAAYAVLDAIKK
ADC-74WP_001211203.1_2AbBOC	GLVMLTNKRIPNEERIKAAYAVLDAIKK
ADC-74WP_001211203.1_95AbBOC	GLVMLTNKRIPNEERIKAAYAVLDAIKK
ADC-74WP_001211203.1_142AbBOC	GLVMLTNKRIPNEERIKAAYAVLDAIKK
DC-74WP_001211203.1_2317AbBOC	GLVMLTNKRIPNEERIKAAYAVLDAIKK
ADC-74WP_001211203.1_570AbBOC	GLVMLTNKRIPNEERIKAAYAVLDAIKK
DC-74WP_001211203.1_2791AbBOC	GLVMLTNKRIPNEERIKAAYAVLDAIKK
DC-74WP_001211203.1_2790AbBOC	GLVMLTNKRIPNEERIKAAYAVLDAIKK
DC-74WP_001211203.1_1414AbBOC	GLVMLTNKRIPNEERIKAAYAVLDAIKK
ADC-74WP_001211203.1_938AbBCP	GLVMLTNKRIPNEERIKAAYAVLDAIKK
ADC-74WP_001211203.1_2AbBCP	GLVMLTNKRIPNEERIKAAYAVLDAIKK
ADC-74WP_001211203.1_95AbBCP	GLVMLTNKRIPNEERIKAAYAVLDAIKK
ADC-74WP_001211203.1_142AbBCP	GLVMLTNKRIPNEERIKAAYAVLDAIKK
DC-74WP_001211203.1_2317AbBCP	GLVMLTNKRIPNEERIKAAYAVLDAIKK
ADC-74WP_001211203.1_570AbBCP	GLVMLTNKRIPNEERIKAAYAVLDAIKK
DC-74WP_001211203.1_2791AbBCP	GLVMLTNKRIPNEERIKAAYAVLDAIKK
DC-74WP_001211203.1_2790AbBCP	GLVMLTNKRIPNEERIKAAYAVLDAIKK
DC-74WP_001211203.1_1414AbBCP	GLVMLTNKRIPNEERIKAAYAVLDAIKK
C-347WP_138149148.1_2656AbBOC	GLVMLTNKRIPNEERIKAAYAVLSAIKK
C-347WP_138149148.1_2656AbBCP	GLVMLTNKRIPNEERIKAAYAVLSAIKK
ADC-80WP_029424536.1_1068AbOC	GLVMLTNKRIPNEERIKAAYAVLSAIKK
DC-80WP_029424536.1_1061AbBOC	GLVMLTNKRIPNEERIKAAYAVLSAIKK
DC-80WP_029424536.1_2945AbBOC	GLVMLTNKRIPNEERIKAAYAVLSAIKK
DC-80WP_029424536.1_2729AbBOC	GLVMLTNKRIPNEERIKAAYAVLSAIKK
DC-80WP_029424536.1_1008AbBOC	GLVMLTNKRIPNEERIKAAYAVLSAIKK

DC-80WP_029424536.1_1061AbBCP	GLVMLTNKRIPNEERIKAAYAVLSAIKK
DC-80WP_029424536.1_2945AbBCP	GLVMLTNKRIPNEERIKAAYAVLSAIKK
DC-80WP_029424536.1_2729AbBCP	GLVMLTNKRIPNEERIKAAYAVLSAIKK
DC-80WP_029424536.1_1008AbBCP	GLVMLTNKRIPNEERIKAAYAVLSAIKK
DC-156WP_024436624.1_2681AbOC	GLVMLTNKRIPNEERIKAAYAVLSAIKK
C-156WP_024436624.1_2375AbBOC	GLVMLTNKRIPNEERIKAAYAVLSAIKK
C-156WP_024436624.1_1038AbBOC	GLVMLTNKRIPNEERIKAAYAVLSAIKK
C-156WP_024436624.1_1041AbBOC	GLVMLTNKRIPNEERIKAAYAVLSAIKK
C-156WP_024436624.1_1124AbBOC	GLVMLTNKRIPNEERIKAAYAVLSAIKK
C-156WP_024436624.1_1126AbBOC	GLVMLTNKRIPNEERIKAAYAVLSAIKK
C-156WP_024436624.1_2375AbBCP	GLVMLTNKRIPNEERIKAAYAVLSAIKK
C-156WP_024436624.1_1038AbBCP	GLVMLTNKRIPNEERIKAAYAVLSAIKK
C-156WP_024436624.1_1041AbBCP	GLVMLTNKRIPNEERIKAAYAVLSAIKK
C-156WP_024436624.1_1124AbBCP	GLVMLTNKRIPNEERIKAAYAVLSAIKK
C-156WP_024436624.1_1126AbBCP	GLVMLTNKRIPNEERIKAAYAVLSAIKK
C-165WP_001211239.1_1734AbBOC	GLVMLTNKRIPNEERIKAAYAVLNAIKK
C-165WP_001211239.1_1032AbBOC	GLVMLTNKRIPNEERIKAAYAVLNAIKK
C-165WP_001211239.1_1734AbBCP	GLVMLTNKRIPNEERIKAAYAVLNAIKK
C-165WP_001211239.1_1032AbBCP	GLVMLTNKRIPNEERIKAAYAVLNAIKK
C-263WP_109433738.1_2026AbBOC	GLVMLTNKRIPNEERIKAAYAVLNAIKK
DC-263WP_109433738.1_954AbBOC	GLVMLTNKRIPNEERIKAAYAVLNAIKK
C-263WP_109433738.1_1022AbBOC	GLVMLTNKRIPNEERIKAAYAVLNAIKK
C-263WP_109433738.1_3219AbBOC	GLVMLTNKRIPNEERIKAAYAVLNAIKK
C-263WP_109433738.1_2026AbBCP	GLVMLTNKRIPNEERIKAAYAVLNAIKK
DC-263WP_109433738.1_954AbBCP	GLVMLTNKRIPNEERIKAAYAVLNAIKK
C-263WP_109433738.1_1022AbBCP	GLVMLTNKRIPNEERIKAAYAVLNAIKK
C-263WP_109433738.1_3219AbBCP	GLVMLTNKRIPNEERIKAAYAVLNAIKK
DC-79WP_001159760.1_2471AbBOC	GLVMLTNKRIPNEERIKAAYAVLNAIKK
ADC-79WP_001159760.1_670AbBOC	GLVMLTNKRIPNEERIKAAYAVLNAIKK
DC-79WP_001159760.1_2535AbBOC	GLVMLTNKRIPNEERIKAAYAVLNAIKK
DC-79WP_001159760.1_1080AbBOC	GLVMLTNKRIPNEERIKAAYAVLNAIKK
DC-79WP_001159760.1_2471AbBCP	GLVMLTNKRIPNEERIKAAYAVLNAIKK
ADC-79WP_001159760.1_670AbBCP	GLVMLTNKRIPNEERIKAAYAVLNAIKK
DC-79WP_001159760.1_2535AbBCP	GLVMLTNKRIPNEERIKAAYAVLNAIKK
DC-79WP_001159760.1_1080AbBCP	GLVMLTNKRIPNEERIKAAYAVLNAIKK
ADC-6WP_017725267.1_1459AbBOC	GLVMLTNKRIPNEERIKAAYAVLNAIKK
ADC-6WP_017725267.1_1678AbBOC	GLVMLTNKRIPNEERIKAAYAVLNAIKK
ADC-6WP_017725267.1_2561AbBOC	GLVMLTNKRIPNEERIKAAYAVLNAIKK
ADC-6WP_017725267.1_1459AbBCP	GLVMLTNKRIPNEERIKAAYAVLNAIKK
ADC-6WP_017725267.1_1678AbBCP	GLVMLTNKRIPNEERIKAAYAVLNAIKK
ADC-6WP_017725267.1_2561AbBCP	GLVMLTNKRIPNEERIKAAYAVLNAIKK
ADC-1WP_004714775.1_3726AbBOC	GLVMLTNKRIPNEERIKAAYAVLNAIKK
ADC-1WP_004714775.1_3726AbBCP	GLVMLTNKRIPNEERIKAAYAVLNAIKK
C-181WP_046693238.1_1083AbBOC	GLVMLTNKRIPNEERIKAAYAVLNAIKK
C-181WP_046693238.1_1083AbBCP	GLVMLTNKRIPNEERIKAAYAVLNAIKK
C-238WP_002058891.1_2458AbBOC	GLVMLTNKRIPNEERIKAAYAVLSAIKK
C-238WP_002058891.1_2458AbBCP	GLVMLTNKRIPNEERIKAAYAVLSAIKK
C-186WP_072292271.1_1675AbBOC	GLVMLTNKRIPNEERIKAAYAVLNAIKK
C-186WP_072292271.1_1675AbBCP	GLVMLTNKRIPNEERIKAAYAVLNAIKK
DC-184WP_059247009.1_1188AbOC	GLVMLTNKRIPNEERIKAAYAVLSAIKK
ADC-328WP_069370341.1_666AbOC	GLVMLTNKRIPNEERIKAAYAVLSAIKK
DC-56WP_031973850.1_1911AbBOC	GLVMLTNKRIPNEERIKAAYAVLNAIKK
DC-56WP_031973850.1_2693AbBOC	GLVMLTNKRIPNEERIKAAYAVLNAIKK
DC-56WP_031973850.1_1911AbBCP	GLVMLTNKRIPNEERIKAAYAVLNAIKK
DC-56WP_031973850.1_2693AbBCP	GLVMLTNKRIPNEERIKAAYAVLNAIKK
DC-117WP_002157727.1_394AbBOC	GLVMLTNKRIPNEERIKAAYAVLNAIKK
DC-117WP_002157727.1_394AbBCP	GLVMLTNKRIPNEERIKAAYAVLNAIKK
ADC-25WP_001211217.1_2432AbOC	GLVMLTNKRIPNEERIKAAYAVLNAIKK
ADC-25WP_001211217.1_1025AbOC	GLVMLTNKRIPNEERIKAAYAVLNAIKK
ADC-25WP_001211217.1_3391AbOC	GLVMLTNKRIPNEERIKAAYAVLNAIKK
ADC-25WP_001211217.1_1042AbOC	GLVMLTNKRIPNEERIKAAYAVLNAIKK
ADC-25WP_001211217.1_1185AbOC	GLVMLTNKRIPNEERIKAAYAVLNAIKK
ADC-25WP_001211217.1_768AbOC	GLVMLTNKRIPNEERIKAAYAVLNAIKK
ADC-25WP_001211217.1_2605AbOC	GLVMLTNKRIPNEERIKAAYAVLNAIKK
DC-25WP_001211217.1_1117AbBOC	GLVMLTNKRIPNEERIKAAYAVLNAIKK
DC-25WP_001211217.1_1090AbBOC	GLVMLTNKRIPNEERIKAAYAVLNAIKK
DC-25WP_001211217.1_1089AbBOC	GLVMLTNKRIPNEERIKAAYAVLNAIKK
DC-25WP_001211217.1_1091AbBOC	GLVMLTNKRIPNEERIKAAYAVLNAIKK
DC-25WP_001211217.1_1604AbBOC	GLVMLTNKRIPNEERIKAAYAVLNAIKK
DC-25WP_001211217.1_1040AbBOC	GLVMLTNKRIPNEERIKAAYAVLNAIKK
ADC-25UYQ12932.1_2634AbBOC	GLVMLTNKRIPNEERIKAAYAVLNAIKK
ADC-25UYQ09083.1_2628AbBOC	GLVMLTNKRIPNEERIKAAYAVLNAIKK
ADC-25UYQ40822.1_1120AbBOC	GLVMLTNKRIPNEERIKAAYAVLNAIKK
DC-25WP_001211217.1_1117AbBCP	GLVMLTNKRIPNEERIKAAYAVLNAIKK
DC-25WP_001211217.1_1090AbBCP	GLVMLTNKRIPNEERIKAAYAVLNAIKK
DC-25WP_001211217.1_1089AbBCP	GLVMLTNKRIPNEERIKAAYAVLNAIKK
DC-25WP_001211217.1_1091AbBCP	GLVMLTNKRIPNEERIKAAYAVLNAIKK
DC-25WP_001211217.1_1604AbBCP	GLVMLTNKRIPNEERIKAAYAVLNAIKK
DC-25WP_001211217.1_1040AbBCP	GLVMLTNKRIPNEERIKAAYAVLNAIKK
ADC-25UYQ12932.1_2634AbBCP	GLVMLTNKRIPNEERIKAAYAVLNAIKK
ADC-25UYQ09083.1_2628AbBCP	GLVMLTNKRIPNEERIKAAYAVLNAIKK
ADC-25UYQ40822.1_1120AbBCP	GLVMLTNKRIPNEERIKAAYAVLNAIKK
C-268WP_105922788.1_2839AbBOC	GLVMLTNKRIPNEERIKAAYAVLNAIKK
ADC-268WDC47378.1_2492AbBOC	GLVMLTNKRIPNEERIKAAYAVLNAIKK
C-268WP_105922788.1_2839AbBCP	GLVMLTNKRIPNEERIKAAYAVLNAIKK
ADC-268WDC47378.1_2492AbBCP	GLVMLTNKRIPNEERIKAAYAVLNAIKK
DC-155WP_005128228.1_2591AbOC	GLVMLTNKRIPNEERIKAAYAVLSAIKK
C-155WP_005128228.1_2549AbBOC	GLVMLTNKRIPNEERIKAAYAVLSAIKK
C-155WP_005128228.1_2480AbBOC	GLVMLTNKRIPNEERIKAAYAVLSAIKK
C-155WP_005128228.1_2455AbBOC	GLVMLTNKRIPNEERIKAAYAVLSAIKK
C-155WP_005128228.1_2549AbBCP	GLVMLTNKRIPNEERIKAAYAVLSAIKK
C-155WP_005128228.1_2480AbBCP	GLVMLTNKRIPNEERIKAAYAVLSAIKK
C-155WP_005128228.1_2455AbBCP	GLVMLTNKRIPNEERIKAAYAVLSAIKK
ADC-76WP_001211237.1_2862AbOC	GLVMLTNKRIPNEERIKAAYAVLNAIKK
ADC-76WP_001211237.1_3431AbOC	GLVMLTNKRIPNEERIKAAYAVLNAIKK
ADC-76WP_001211237.1_2696AbOC	GLVMLTNKRIPNEERIKAAYAVLNAIKK
ADC-76WP_001211237.1_2890AbOC	GLVMLTNKRIPNEERIKAAYAVLNAIKK
DC-76WP_001211237.1_2723AbBOC	GLVMLTNKRIPNEERIKAAYAVLNAIKK
DC-76WP_001211237.1_1033AbBOC	GLVMLTNKRIPNEERIKAAYAVLNAIKK
ADC-76WP_001211237.1_526AbBOC	GLVMLTNKRIPNEERIKAAYAVLNAIKK
DC-76WP_001211237.1_1046AbBOC	GLVMLTNKRIPNEERIKAAYAVLNAIKK
DC-76WP_001211237.1_1040AbBOC	GLVMLTNKRIPNEERIKAAYAVLNAIKK
DC-76WP_001211237.1_3682AbBOC	GLVMLTNKRIPNEERIKAAYAVLNAIKK
DC-76WP_001211237.1_2579AbBOC	GLVMLTNKRIPNEERIKAAYAVLNAIKK
DC-76WP_001211237.1_2723AbBCP	GLVMLTNKRIPNEERIKAAYAVLNAIKK
DC-76WP_001211237.1_1033AbBCP	GLVMLTNKRIPNEERIKAAYAVLNAIKK
ADC-76WP_001211237.1_526AbBCP	GLVMLTNKRIPNEERIKAAYAVLNAIKK
DC-76WP_001211237.1_1046AbBCP	GLVMLTNKRIPNEERIKAAYAVLNAIKK
DC-76WP_001211237.1_1040AbBCP	GLVMLTNKRIPNEERIKAAYAVLNAIKK
DC-76WP_001211237.1_3682AbBCP	GLVMLTNKRIPNEERIKAAYAVLNAIKK
DC-76WP_001211237.1_2579AbBCP	GLVMLTNKRIPNEERIKAAYAVLNAIKK
DC-291WP_001211201.1_1060AbOC	GLVMLTNKRIPNEERIKAAYAVLNAIKK
C-291WP_001211201.1_2415AbBOC	GLVMLTNKRIPNEERIKAAYAVLNAIKK
C-291WP_001211201.1_2415AbBCP	GLVMLTNKRIPNEERIKAAYAVLNAIKK
DC-214WP_060454527.1_118AbBOC	GLVMLTNKRIPNEERIKAAYAVLNAIKE
DC-214WP_060454527.1_118AbBCP	GLVMLTNKRIPNEERIKAAYAVLNAIKE
C-185WP_001211210.1_2745AbBOC	GLVMLTNKRIPNEERIKAAYAVLNAIKK
ADC-185WEI07035.1_298AbBOC	GLVMLTNKRIPNEERIKAAYAVLNAIKK
C-185WP_001211210.1_2745AbBCP	GLVMLTNKRIPNEERIKAAYAVLNAIKK
ADC-185WEI07035.1_298AbBCP	GLVMLTNKRIPNEERIKAAYAVLNAIKK
ADC-11WP_001211205.1_1099AbOC	GLVMLTNKRIPNEERIKAAYAVLDAIKK
ADC-11WP_001211205.1_1035AbOC	GLVMLTNKRIPNEERIKAAYAVLDAIKK
DC-11WP_001211205.1_2603AbBOC	GLVMLTNKRIPNEERIKAAYAVLDAIKK
DC-11WP_001211205.1_1138AbBOC	GLVMLTNKRIPNEERIKAAYAVLDAIKK
DC-11WP_001211205.1_2874AbBOC	GLVMLTNKRIPNEERIKAAYAVLDAIKK
DC-11WP_001211205.1_2686AbBOC	GLVMLTNKRIPNEERIKAAYAVLDAIKK
ADC-11WP_001211205.1_130AbBOC	GLVMLTNKRIPNEERIKAAYAVLDAIKK
DC-11WP_001211205.1_1097AbBOC	GLVMLTNKRIPNEERIKAAYAVLDAIKK
ADC-11WP_001211205.1_684AbBOC	GLVMLTNKRIPNEERIKAAYAVLDAIKK
ADC-11WP_001211205.1_682AbBOC	GLVMLTNKRIPNEERIKAAYAVLDAIKK
DC-11WP_001211205.1_1111AbBOC	GLVMLTNKRIPNEERIKAAYAVLDAIKK
DC-11WP_001211205.1_1059AbBOC	GLVMLTNKRIPNEERIKAAYAVLDAIKK
DC-11WP_001211205.1_2754AbBOC	GLVMLTNKRIPNEERIKAAYAVLDAIKK
DC-11WP_001211205.1_2603AbBCP	GLVMLTNKRIPNEERIKAAYAVLDAIKK
DC-11WP_001211205.1_1138AbBCP	GLVMLTNKRIPNEERIKAAYAVLDAIKK
DC-11WP_001211205.1_2874AbBCP	GLVMLTNKRIPNEERIKAAYAVLDAIKK
DC-11WP_001211205.1_2686AbBCP	GLVMLTNKRIPNEERIKAAYAVLDAIKK
ADC-11WP_001211205.1_130AbBCP	GLVMLTNKRIPNEERIKAAYAVLDAIKK
DC-11WP_001211205.1_1097AbBCP	GLVMLTNKRIPNEERIKAAYAVLDAIKK
ADC-11WP_001211205.1_684AbBCP	GLVMLTNKRIPNEERIKAAYAVLDAIKK
ADC-11WP_001211205.1_682AbBCP	GLVMLTNKRIPNEERIKAAYAVLDAIKK
DC-11WP_001211205.1_1111AbBCP	GLVMLTNKRIPNEERIKAAYAVLDAIKK
DC-11WP_001211205.1_1059AbBCP	GLVMLTNKRIPNEERIKAAYAVLDAIKK
DC-11WP_001211205.1_2754AbBCP	GLVMLTNKRIPNEERIKAAYAVLDAIKK
C-269WP_032025626.1_2988AbBOC	GLVMLTNKRIPNEERIKAAYAVLSAIKK
C-269WP_032025626.1_2988AbBCP	GLVMLTNKRIPNEERIKAAYAVLSAIKK
C-166WP_101244937.1_1028AbBOC	GLVMLTNKRIPNEERIKAAYTVLNAIKK
C-166WP_101244937.1_1028AbBCP	GLVMLTNKRIPNEERIKAAYTVLNAIKK
C-175WP_001211208.1_2561AbBOC	GLVMLTNKRIPNEERIKAAYAVLDAIKK
C-175WP_001211208.1_2561AbBCP	GLVMLTNKRIPNEERIKAAYAVLDAIKK
C-154WP_005138362.1_2451AbBOC	GLVMLTNKRIPNEERIKAAYAVLNAIKE
DC-154WP_005138362.1_158AbBOC	GLVMLTNKRIPNEERIKAAYAVLNAIKE

```
C-154WP_005138362.1_1167AbBOC     GLVMLTNKRIPNEERIKAAYAVLNAIKE
C-154WP_005138362.1_2510AbBOC     GLVMLTNKRIPNEERIKAAYAVLNAIKE
C-154WP_005138362.1_2513AbBOC     GLVMLTNKRIPNEERIKAAYAVLNAIKE
C-154WP_005138362.1_2512AbBOC     GLVMLTNKRIPNEERIKAAYAVLNAIKE
C-154WP_005138362.1_2451AbBCP     GLVMLTNKRIPNEERIKAAYAVLNAIKE
DC-154WP_005138362.1_158AbBCP     GLVMLTNKRIPNEERIKAAYAVLNAIKE
C-154WP_005138362.1_1167AbBCP     GLVMLTNKRIPNEERIKAAYAVLNAIKE
C-154WP_005138362.1_2510AbBCP     GLVMLTNKRIPNEERIKAAYAVLNAIKE
C-154WP_005138362.1_2513AbBCP     GLVMLTNKRIPNEERIKAAYAVLNAIKE
C-154WP_005138362.1_2512AbBCP     GLVMLTNKRIPNEERIKAAYAVLNAIKE
C-240WP_001211207.1_1200AbBOC     GLVMLTNKRIPNEERIKAAYAVLDAIKK
C-240WP_001211207.1_1200AbBCP     GLVMLTNKRIPNEERIKAAYAVLDAIKK
ADC-75WP_063857817.1_1610AbOC     GLVMLTNKRIPNEERIKAAYAVLNAIKK
DC-18WP_002118772.1_1702AbBOC     GLVMLTNKRIPNEERIKAAYAVLNAIKK
DC-18WP_002118772.1_1702AbBCP     GLVMLTNKRIPNEERIKAAYAVLNAIKK
C-106WP_068981613.1_2650AbBOC     GLVMLTNKRIPNEERIKAAYAVLNAIKK
C-106WP_068981613.1_2650AbBCP     GLVMLTNKRIPNEERIKAAYAVLNAIKK
DC-50WP_031965243.1_2562AbBOC     GLVMLTNKRIPNEERIKAAYAVLNAIKK
ADC-50WP_031965243.1_206AbBOC     GLVMLTNKRIPNEERIKAAYAVLNAIKK
DC-50WP_031965243.1_2751AbBOC     GLVMLTNKRIPNEERIKAAYAVLNAIKK
DC-50WP_031965243.1_3766AbBOC     GLVMLTNKRIPNEERIKAAYAVLNAIKK
DC-50WP_031965243.1_2562AbBCP     GLVMLTNKRIPNEERIKAAYAVLNAIKK
ADC-50WP_031965243.1_206AbBCP     GLVMLTNKRIPNEERIKAAYAVLNAIKK
DC-50WP_031965243.1_2751AbBCP     GLVMLTNKRIPNEERIKAAYAVLNAIKK
DC-50WP_031965243.1_3766AbBCP     GLVMLTNKRIPNEERIKAAYAVLNAIKK
ADC-158WP_001211227.1_990AbOC     GLVMLTNKRIPNEERIKAAYAVLNAIKK
DC-158WP_001211227.1_1295AbOC     GLVMLTNKRIPNEERIKAAYAVLNAIKK
ADC-158WP_001211227.1_6AbOC       GLVMLTNKRIPNEERIKAAYAVLNAIKK
DC-158WP_001211227.1_991AbBOC     GLVMLTNKRIPNEERIKAAYAVLNAIKK
DC-158WP_001211227.1_207AbBOC     GLVMLTNKRIPNEERIKAAYAVLNAIKK
C-158WP_001211227.1_2735AbBOC     GLVMLTNKRIPNEERIKAAYAVLNAIKK
DC-158WP_001211227.1_996AbBOC     GLVMLTNKRIPNEERIKAAYAVLNAIKK
DC-158WP_001211227.1_796AbBOC     GLVMLTNKRIPNEERIKAAYAVLNAIKK
C-158WP_001211227.1_2822AbBOC     GLVMLTNKRIPNEERIKAAYAVLNAIKK
C-158WP_001211227.1_1339AbBOC     GLVMLTNKRIPNEERIKAAYAVLNAIKK
ADC-158WGQ04828.1_2712AbBOC       GLVMLTNKRIPNEERIKAAYAVLNAIKK
DC-158WP_001211227.1_991AbBCP     GLVMLTNKRIPNEERIKAAYAVLNAIKK
DC-158WP_001211227.1_207AbBCP     GLVMLTNKRIPNEERIKAAYAVLNAIKK
C-158WP_001211227.1_2735AbBCP     GLVMLTNKRIPNEERIKAAYAVLNAIKK
DC-158WP_001211227.1_996AbBCP     GLVMLTNKRIPNEERIKAAYAVLNAIKK
DC-158WP_001211227.1_796AbBCP     GLVMLTNKRIPNEERIKAAYAVLNAIKK
C-158WP_001211227.1_2822AbBCP     GLVMLTNKRIPNEERIKAAYAVLNAIKK
C-158WP_001211227.1_1339AbBCP     GLVMLTNKRIPNEERIKAAYAVLNAIKK
ADC-158WGQ04828.1_2712AbBCP       GLVMLTNKRIPNEERIKAAYAVLNAIKK
ADC-31WP_001211223.1_2986AbOC     GLVMLTNKRIPNEERIKAAYAVLNAIKK
DC-31WP_001211223.1_2698AbBOC     GLVMLTNKRIPNEERIKAAYAVLNAIKK
DC-31WP_001211223.1_2698AbBCP     GLVMLTNKRIPNEERIKAAYAVLNAIKK
ADC-30WP_001211218.1_1102AbOC     GLVMLTNKRIPNEERIKAAYAVLNAIKK
ADC-30WP_001211218.1_2565AbOC     GLVMLTNKRIPNEERIKAAYAVLNAIKK
ADC-30WP_001211218.1_1021AbOC     GLVMLTNKRIPNEERIKAAYAVLNAIKK
ADC-30WP_001211218.1_1088AbOC     GLVMLTNKRIPNEERIKAAYAVLNAIKK
ADC-30WP_001211218.1_1135AbOC     GLVMLTNKRIPNEERIKAAYAVLNAIKK
ADC-30WP_001211218.1_43AbOC       GLVMLTNKRIPNEERIKAAYAVLNAIKK
ADC-30WP_001211218.1_1041AbOC     GLVMLTNKRIPNEERIKAAYAVLNAIKK
ADC-30WP_001211218.1_1032AbOC     GLVMLTNKRIPNEERIKAAYAVLNAIKK
ADC-30WP_001211218.1_1047AbOC     GLVMLTNKRIPNEERIKAAYAVLNAIKK
ADC-30WP_001211218.1_1040AbOC     GLVMLTNKRIPNEERIKAAYAVLNAIKK
ADC-30WP_001211218.1_3388AbOC     GLVMLTNKRIPNEERIKAAYAVLNAIKK
ADC-30WP_001211218.1_650AbOC      GLVMLTNKRIPNEERIKAAYAVLNAIKK
ADC-30WP_001211218.1_2855AbOC     GLVMLTNKRIPNEERIKAAYAVLNAIKK
ADC-30WP_001211218.1_1034AbOC     GLVMLTNKRIPNEERIKAAYAVLNAIKK
ADC-30WP_001211218.1_79AbOC       GLVMLTNKRIPNEERIKAAYAVLNAIKK
ADC-30WP_001211218.1_3509AbOC     GLVMLTNKRIPNEERIKAAYAVLNAIKK
ADC-30WP_001211218.1_2399AbOC     GLVMLTNKRIPNEERIKAAYAVLNAIKK
ADC-30WP_001211218.1_2415AbOC     GLVMLTNKRIPNEERIKAAYAVLNAIKK
ADC-30WP_001211218.1_3032AbOC     GLVMLTNKRIPNEERIKAAYAVLNAIKK
ADC-30WP_001211218.1_832AbOC      GLVMLTNKRIPNEERIKAAYAVLNAIKK
ADC-30WP_001211218.1_1428AbOC     GLVMLTNKRIPNEERIKAAYAVLNAIKK
ADC-30WP_001211218.1_2340AbOC     GLVMLTNKRIPNEERIKAAYAVLNAIKK
ADC-30WP_001211218.1_2976AbOC     GLVMLTNKRIPNEERIKAAYAVLNAIKK
ADC-30WP_001211218.1_2305AbOC     GLVMLTNKRIPNEERIKAAYAVLNAIKK
ADC-30WP_001211218.1_1110AbOC     GLVMLTNKRIPNEERIKAAYAVLNAIKK
ADC-30WP_001211218.1_2797AbOC     GLVMLTNKRIPNEERIKAAYAVLNAIKK
ADC-30WP_001211218.1_1023AbOC     GLVMLTNKRIPNEERIKAAYAVLNAIKK
ADC-30WP_001211218.1_1022AbOC     GLVMLTNKRIPNEERIKAAYAVLNAIKK
ADC-30WP_001211218.1_2458AbOC     GLVMLTNKRIPNEERIKAAYAVLNAIKK
ADC-30WP_001211218.1_2513AbOC     GLVMLTNKRIPNEERIKAAYAVLNAIKK
ADC-30WP_001211218.1_2482AbOC     GLVMLTNKRIPNEERIKAAYAVLNAIKK
DC-30WP_001211218.1_2692AbBOC     GLVMLTNKRIPNEERIKAAYAVLNAIKK
DC-30WP_001211218.1_2549AbBOC     GLVMLTNKRIPNEERIKAAYAVLNAIKK
DC-30WP_001211218.1_1026AbBOC     GLVMLTNKRIPNEERIKAAYAVLNAIKK
ADC-30WP_001211218.1_373AbBOC     GLVMLTNKRIPNEERIKAAYAVLNAIKK
DC-30WP_001211218.1_2465AbBOC     GLVMLTNKRIPNEERIKAAYAVLNAIKK
DC-30WP_001211218.1_1027AbBOC     GLVMLTNKRIPNEERIKAAYAVLNAIKK
DC-30WP_001211218.1_2650AbBOC     GLVMLTNKRIPNEERIKAAYAVLNAIKK
DC-30WP_001211218.1_2674AbBOC     GLVMLTNKRIPNEERIKAAYAVLNAIKK
DC-30WP_001211218.1_2689AbBOC     GLVMLTNKRIPNEERIKAAYAVLNAIKK
DC-30WP_001211218.1_1105AbBOC     GLVMLTNKRIPNEERIKAAYAVLNAIKK
DC-30WP_001211218.1_2670AbBOC     GLVMLTNKRIPNEERIKAAYAVLNAIKK
DC-30WP_001211218.1_1019AbBOC     GLVMLTNKRIPNEERIKAAYAVLNAIKK
DC-30WP_001211218.1_1039AbBOC     GLVMLTNKRIPNEERIKAAYAVLNAIKK
DC-30WP_001211218.1_1023AbBOC     GLVMLTNKRIPNEERIKAAYAVLNAIKK
DC-30WP_001211218.1_3189AbBOC     GLVMLTNKRIPNEERIKAAYAVLNAIKK
ADC-30WP_001211218.1_594AbBOC     GLVMLTNKRIPNEERIKAAYAVLNAIKK
DC-30WP_001211218.1_1718AbBOC     GLVMLTNKRIPNEERIKAAYAVLNAIKK
DC-30WP_001211218.1_2484AbBOC     GLVMLTNKRIPNEERIKAAYAVLNAIKK
DC-30WP_001211218.1_3305AbBOC     GLVMLTNKRIPNEERIKAAYAVLNAIKK
DC-30WP_001211218.1_3346AbBOC     GLVMLTNKRIPNEERIKAAYAVLNAIKK
DC-30WP_001211218.1_1482AbBOC     GLVMLTNKRIPNEERIKAAYAVLNAIKK
DC-30WP_001211218.1_2574AbBOC     GLVMLTNKRIPNEERIKAAYAVLNAIKK
ADC-30WP_001211218.1_831AbBOC     GLVMLTNKRIPNEERIKAAYAVLNAIKK
DC-30WP_001211218.1_1826AbBOC     GLVMLTNKRIPNEERIKAAYAVLNAIKK
DC-30WP_001211218.1_1024AbBOC     GLVMLTNKRIPNEERIKAAYAVLNAIKK
DC-30WP_001211218.1_1618AbBOC     GLVMLTNKRIPNEERIKAAYAVLNAIKK
ADC-30WP_001211218.1_301AbBOC     GLVMLTNKRIPNEERIKAAYAVLNAIKK
ADC-30WP_001211218.1_940AbBOC     GLVMLTNKRIPNEERIKAAYAVLNAIKK
DC-30WP_001211218.1_2949AbBOC     GLVMLTNKRIPNEERIKAAYAVLNAIKK
DC-30WP_001211218.1_1028AbBOC     GLVMLTNKRIPNEERIKAAYAVLNAIKK
DC-30WP_001211218.1_2526AbBOC     GLVMLTNKRIPNEERIKAAYAVLNAIKK
DC-30WP_001211218.1_1040AbBOC     GLVMLTNKRIPNEERIKAAYAVLNAIKK
DC-30WP_001211218.1_1089AbBOC     GLVMLTNKRIPNEERIKAAYAVLNAIKK
DC-30WP_001211218.1_1099AbBOC     GLVMLTNKRIPNEERIKAAYAVLNAIKK
DC-30WP_001211218.1_1206AbBOC     GLVMLTNKRIPNEERIKAAYAVLNAIKK
DC-30WP_001211218.1_1167AbBOC     GLVMLTNKRIPNEERIKAAYAVLNAIKK
DC-30WP_001211218.1_1186AbBOC     GLVMLTNKRIPNEERIKAAYAVLNAIKK
DC-30WP_001211218.1_1165AbBOC     GLVMLTNKRIPNEERIKAAYAVLNAIKK
DC-30WP_001211218.1_1077AbBOC     GLVMLTNKRIPNEERIKAAYAVLNAIKK
DC-30WP_001211218.1_1078AbBOC     GLVMLTNKRIPNEERIKAAYAVLNAIKK
DC-30WP_001211218.1_1071AbBOC     GLVMLTNKRIPNEERIKAAYAVLNAIKK
DC-30WP_001211218.1_2694AbBOC     GLVMLTNKRIPNEERIKAAYAVLNAIKK
DC-30WP_001211218.1_3340AbBOC     GLVMLTNKRIPNEERIKAAYAVLNAIKK
DC-30WP_001211218.1_1022AbBOC     GLVMLTNKRIPNEERIKAAYAVLNAIKK
DC-30WP_001211218.1_1025AbBOC     GLVMLTNKRIPNEERIKAAYAVLNAIKK
DC-30WP_001211218.1_1158AbBOC     GLVMLTNKRIPNEERIKAAYAVLNAIKK
DC-30WP_001211218.1_1020AbBOC     GLVMLTNKRIPNEERIKAAYAVLNAIKK
DC-30WP_001211218.1_1021AbBOC     GLVMLTNKRIPNEERIKAAYAVLNAIKK
ADC-30WP_001211218.1_532AbBOC     GLVMLTNKRIPNEERIKAAYAVLNAIKK
ADC-30WP_001211218.1_539AbBOC     GLVMLTNKRIPNEERIKAAYAVLNAIKK
DC-30WP_001211218.1_1033AbBOC     GLVMLTNKRIPNEERIKAAYAVLNAIKK
DC-30WP_001211218.1_1069AbBOC     GLVMLTNKRIPNEERIKAAYAVLNAIKK
DC-30WP_001211218.1_1029AbBOC     GLVMLTNKRIPNEERIKAAYAVLNAIKK
DC-30WP_001211218.1_1030AbBOC     GLVMLTNKRIPNEERIKAAYAVLNAIKK
DC-30WP_001211218.1_1096AbBOC     GLVMLTNKRIPNEERIKAAYAVLNAIKK
DC-30WP_001211218.1_1037AbBOC     GLVMLTNKRIPNEERIKAAYAVLNAIKK
DC-30WP_001211218.1_1098AbBOC     GLVMLTNKRIPNEERIKAAYAVLNAIKK
DC-30WP_001211218.1_1046AbBOC     GLVMLTNKRIPNEERIKAAYAVLNAIKK
DC-30WP_001211218.1_1032AbBOC     GLVMLTNKRIPNEERIKAAYAVLNAIKK
DC-30WP_001211218.1_1070AbBOC     GLVMLTNKRIPNEERIKAAYAVLNAIKK
DC-30WP_001211218.1_1139AbBOC     GLVMLTNKRIPNEERIKAAYAVLNAIKK
DC-30WP_001211218.1_1031AbBOC     GLVMLTNKRIPNEERIKAAYAVLNAIKK
DC-30WP_001211218.1_1066AbBOC     GLVMLTNKRIPNEERIKAAYAVLNAIKK
DC-30WP_001211218.1_1102AbBOC     GLVMLTNKRIPNEERIKAAYAVLNAIKK
ADC-30USX57838.1_2957AbBOC        GLVMLTNKRIPNEERIKAAYAVLNAIKK
ADC-30USX59985.1_1918AbBOC        GLVMLTNKRIPNEERIKAAYAVLNAIKK
ADC-30USX63799.1_2772AbBOC        GLVMLTNKRIPNEERIKAAYAVLNAIKK
ADC-30USX74012.1_305AbBOC         GLVMLTNKRIPNEERIKAAYAVLNAIKK
ADC-30USZ52468.1_2397AbBOC        GLVMLTNKRIPNEERIKAAYAVLNAIKK
DC-30WP_001211218.1_2008AbBOC     GLVMLTNKRIPNEERIKAAYAVLNAIKK
ADC-30WCL78976.1_1026AbBOC        GLVMLTNKRIPNEERIKAAYAVLNAIKK
ADC-30WDO27826.1_1034AbBOC        GLVMLTNKRIPNEERIKAAYAVLNAIKK
DC-30WP_001211218.1_2125AbBOC     GLVMLTNKRIPNEERIKAAYAVLNAIKK
DC-30WP_001211218.1_2729AbBOC     GLVMLTNKRIPNEERIKAAYAVLNAIKK
```

DC-30WP_001211218.1_2692AbBCP GLVMLTNKRIPNEERIKAAYAVLNAIKK
DC-30WP_001211218.1_2549AbBCP GLVMLTNKRIPNEERIKAAYAVLNAIKK
DC-30WP_001211218.1_1026AbBCP GLVMLTNKRIPNEERIKAAYAVLNAIKK
ADC-30WP_001211218.1_373AbBCP GLVMLTNKRIPNEERIKAAYAVLNAIKK
DC-30WP_001211218.1_2465AbBCP GLVMLTNKRIPNEERIKAAYAVLNAIKK
DC-30WP_001211218.1_1027AbBCP GLVMLTNKRIPNEERIKAAYAVLNAIKK
DC-30WP_001211218.1_2650AbBCP GLVMLTNKRIPNEERIKAAYAVLNAIKK
DC-30WP_001211218.1_2674AbBCP GLVMLTNKRIPNEERIKAAYAVLNAIKK
DC-30WP_001211218.1_2689AbBCP GLVMLTNKRIPNEERIKAAYAVLNAIKK
DC-30WP_001211218.1_1105AbBCP GLVMLTNKRIPNEERIKAAYAVLNAIKK
DC-30WP_001211218.1_2670AbBCP GLVMLTNKRIPNEERIKAAYAVLNAIKK
DC-30WP_001211218.1_1019AbBCP GLVMLTNKRIPNEERIKAAYAVLNAIKK
DC-30WP_001211218.1_1039AbBCP GLVMLTNKRIPNEERIKAAYAVLNAIKK
DC-30WP_001211218.1_1023AbBCP GLVMLTNKRIPNEERIKAAYAVLNAIKK
DC-30WP_001211218.1_3189AbBCP GLVMLTNKRIPNEERIKAAYAVLNAIKK
ADC-30WP_001211218.1_594AbBCP GLVMLTNKRIPNEERIKAAYAVLNAIKK
DC-30WP_001211218.1_1718AbBCP GLVMLTNKRIPNEERIKAAYAVLNAIKK
DC-30WP_001211218.1_2484AbBCP GLVMLTNKRIPNEERIKAAYAVLNAIKK
DC-30WP_001211218.1_3305AbBCP GLVMLTNKRIPNEERIKAAYAVLNAIKK
DC-30WP_001211218.1_3346AbBCP GLVMLTNKRIPNEERIKAAYAVLNAIKK
DC-30WP_001211218.1_1482AbBCP GLVMLTNKRIPNEERIKAAYAVLNAIKK
DC-30WP_001211218.1_2574AbBCP GLVMLTNKRIPNEERIKAAYAVLNAIKK
ADC-30WP_001211218.1_831AbBCP GLVMLTNKRIPNEERIKAAYAVLNAIKK
DC-30WP_001211218.1_1826AbBCP GLVMLTNKRIPNEERIKAAYAVLNAIKK
DC-30WP_001211218.1_1024AbBCP GLVMLTNKRIPNEERIKAAYAVLNAIKK
DC-30WP_001211218.1_1618AbBCP GLVMLTNKRIPNEERIKAAYAVLNAIKK
ADC-30WP_001211218.1_301AbBCP GLVMLTNKRIPNEERIKAAYAVLNAIKK
ADC-30WP_001211218.1_940AbBCP GLVMLTNKRIPNEERIKAAYAVLNAIKK
DC-30WP_001211218.1_2949AbBCP GLVMLTNKRIPNEERIKAAYAVLNAIKK
DC-30WP_001211218.1_1028AbBCP GLVMLTNKRIPNEERIKAAYAVLNAIKK
DC-30WP_001211218.1_2526AbBCP GLVMLTNKRIPNEERIKAAYAVLNAIKK
DC-30WP_001211218.1_1040AbBCP GLVMLTNKRIPNEERIKAAYAVLNAIKK
DC-30WP_001211218.1_1089AbBCP GLVMLTNKRIPNEERIKAAYAVLNAIKK
DC-30WP_001211218.1_1099AbBCP GLVMLTNKRIPNEERIKAAYAVLNAIKK
DC-30WP_001211218.1_1206AbBCP GLVMLTNKRIPNEERIKAAYAVLNAIKK
DC-30WP_001211218.1_1167AbBCP GLVMLTNKRIPNEERIKAAYAVLNAIKK
DC-30WP_001211218.1_1186AbBCP GLVMLTNKRIPNEERIKAAYAVLNAIKK
DC-30WP_001211218.1_1165AbBCP GLVMLTNKRIPNEERIKAAYAVLNAIKK
DC-30WP_001211218.1_1077AbBCP GLVMLTNKRIPNEERIKAAYAVLNAIKK
DC-30WP_001211218.1_1078AbBCP GLVMLTNKRIPNEERIKAAYAVLNAIKK
DC-30WP_001211218.1_1071AbBCP GLVMLTNKRIPNEERIKAAYAVLNAIKK
DC-30WP_001211218.1_2694AbBCP GLVMLTNKRIPNEERIKAAYAVLNAIKK
DC-30WP_001211218.1_3340AbBCP GLVMLTNKRIPNEERIKAAYAVLNAIKK
DC-30WP_001211218.1_1022AbBCP GLVMLTNKRIPNEERIKAAYAVLNAIKK
DC-30WP_001211218.1_1025AbBCP GLVMLTNKRIPNEERIKAAYAVLNAIKK
DC-30WP_001211218.1_1158AbBCP GLVMLTNKRIPNEERIKAAYAVLNAIKK
DC-30WP_001211218.1_1020AbBCP GLVMLTNKRIPNEERIKAAYAVLNAIKK
DC-30WP_001211218.1_1021AbBCP GLVMLTNKRIPNEERIKAAYAVLNAIKK
ADC-30WP_001211218.1_532AbBCP GLVMLTNKRIPNEERIKAAYAVLNAIKK
ADC-30WP_001211218.1_539AbBCP GLVMLTNKRIPNEERIKAAYAVLNAIKK
DC-30WP_001211218.1_1033AbBCP GLVMLTNKRIPNEERIKAAYAVLNAIKK
DC-30WP_001211218.1_1069AbBCP GLVMLTNKRIPNEERIKAAYAVLNAIKK
DC-30WP_001211218.1_1029AbBCP GLVMLTNKRIPNEERIKAAYAVLNAIKK
DC-30WP_001211218.1_1030AbBCP GLVMLTNKRIPNEERIKAAYAVLNAIKK
DC-30WP_001211218.1_1096AbBCP GLVMLTNKRIPNEERIKAAYAVLNAIKK
DC-30WP_001211218.1_1037AbBCP GLVMLTNKRIPNEERIKAAYAVLNAIKK
DC-30WP_001211218.1_1098AbBCP GLVMLTNKRIPNEERIKAAYAVLNAIKK
DC-30WP_001211218.1_1046AbBCP GLVMLTNKRIPNEERIKAAYAVLNAIKK
DC-30WP_001211218.1_1032AbBCP GLVMLTNKRIPNEERIKAAYAVLNAIKK
DC-30WP_001211218.1_1070AbBCP GLVMLTNKRIPNEERIKAAYAVLNAIKK
DC-30WP_001211218.1_1139AbBCP GLVMLTNKRIPNEERIKAAYAVLNAIKK
DC-30WP_001211218.1_1031AbBCP GLVMLTNKRIPNEERIKAAYAVLNAIKK
DC-30WP_001211218.1_1066AbBCP GLVMLTNKRIPNEERIKAAYAVLNAIKK
DC-30WP_001211218.1_1102AbBCP GLVMLTNKRIPNEERIKAAYAVLNAIKK
ADC-30USX57838.1_2957AbBCP GLVMLTNKRIPNEERIKAAYAVLNAIKK
ADC-30USX59985.1_1918AbBCP GLVMLTNKRIPNEERIKAAYAVLNAIKK
ADC-30USX63799.1_2772AbBCP GLVMLTNKRIPNEERIKAAYAVLNAIKK
ADC-30USX74012.1_305AbBCP GLVMLTNKRIPNEERIKAAYAVLNAIKK
ADC-30USZ52468.1_2397AbBCP GLVMLTNKRIPNEERIKAAYAVLNAIKK
DC-30WP_001211218.1_2008AbBCP GLVMLTNKRIPNEERIKAAYAVLNAIKK
ADC-30WCL78976.1_1026AbBCP GLVMLTNKRIPNEERIKAAYAVLNAIKK
ADC-30WDO27826.1_1034AbBCP GLVMLTNKRIPNEERIKAAYAVLNAIKK
DC-30WP_001211218.1_2125AbBCP GLVMLTNKRIPNEERIKAAYAVLNAIKK
DC-30WP_001211218.1_2729AbBCP GLVMLTNKRIPNEERIKAAYAVLNAIKK
ADC-30ADX93237.1_2806AbBCP GLVMLTNKRIPNEERIKAAYAVLNAIKK
ADC-82WP_001211216.1_2747AbOC GLVMLTNKRIPNEERIKAAYAVLNAIKK
DC-82WP_001211216.1_1093AbBOC GLVMLTNKRIPNEERIKAAYAVLNAIKK
DC-82WP_001211216.1_1071AbBOC GLVMLTNKRIPNEERIKAAYAVLNAIKK
DC-82WP_001211216.1_1093AbBCP GLVMLTNKRIPNEERIKAAYAVLNAIKK
DC-82WP_001211216.1_1071AbBCP GLVMLTNKRIPNEERIKAAYAVLNAIKK
DC-162WP_031980335.1_1153AbOC GLVMLTNKRIPNEERIKAAYAVLNAIKK
C-162WP_031980335.1_1028AbBOC GLVMLTNKRIPNEERIKAAYAVLNAIKK
C-162WP_031980335.1_1023AbBOC GLVMLTNKRIPNEERIKAAYAVLNAIKK
C-162WP_031980335.1_1028AbBCP GLVMLTNKRIPNEERIKAAYAVLNAIKK
C-162WP_031980335.1_1023AbBCP GLVMLTNKRIPNEERIKAAYAVLNAIKK
ADC-73WP_001211219.1_1012AbOC GLVMLTNKRIPNEERIKAAYAVLNAIKK
ADC-73WP_001211219.1_1953AbOC GLVMLTNKRIPNEERIKAAYAVLNAIKK
ADC-73WP_001211219.1_1014AbOC GLVMLTNKRIPNEERIKAAYAVLNAIKK
ADC-73WP_001211219.1_1041AbOC GLVMLTNKRIPNEERIKAAYAVLNAIKK
ADC-73WP_001211219.1_1028AbOC GLVMLTNKRIPNEERIKAAYAVLNAIKK
ADC-73WP_001211219.1_2674AbOC GLVMLTNKRIPNEERIKAAYAVLNAIKK
ADC-73WP_001211219.1_462AbOC GLVMLTNKRIPNEERIKAAYAVLNAIKK
ADC-73WP_001211219.1_3530AbOC GLVMLTNKRIPNEERIKAAYAVLNAIKK
ADC-73WP_001211219.1_3621AbOC GLVMLTNKRIPNEERIKAAYAVLNAIKK
ADC-73WP_001211219.1_3381AbOC GLVMLTNKRIPNEERIKAAYAVLNAIKK
ADC-73WP_001211219.1_2515AbOC GLVMLTNKRIPNEERIKAAYAVLNAIKK
ADC-73WP_001211219.1_3574AbOC GLVMLTNKRIPNEERIKAAYAVLNAIKK
ADC-73WP_001211219.1_1656AbOC GLVMLTNKRIPNEERIKAAYAVLNAIKK
ADC-73WP_001211219.1_2591AbOC GLVMLTNKRIPNEERIKAAYAVLNAIKK
ADC-73WP_001211219.1_2602AbOC GLVMLTNKRIPNEERIKAAYAVLNAIKK
ADC-73WP_001211219.1_910AbOC GLVMLTNKRIPNEERIKAAYAVLNAIKK
ADC-73WP_001211219.1_394AbOC GLVMLTNKRIPNEERIKAAYAVLNAIKK
ADC-73WP_001211219.1_1030AbOC GLVMLTNKRIPNEERIKAAYAVLNAIKK
ADC-73WP_001211219.1_1023AbOC GLVMLTNKRIPNEERIKAAYAVLNAIKK
ADC-73UYQ66995.1_1967AbOC GLVMLTNKRIPNEERIKAAYAVLNAIKK
ADC-73WP_001211219.1_906AbBOC GLVMLTNKRIPNEERIKAAYAVLNAIKK
DC-73WP_001211219.1_2649AbBOC GLVMLTNKRIPNEERIKAAYAVLNAIKK
DC-73WP_001211219.1_2693AbBOC GLVMLTNKRIPNEERIKAAYAVLNAIKK
ADC-73AQU57925.1_2703AbBOC GLVMLTNKRIPNEERIKAAYAVLNAIKK
DC-73WP_001211219.1_1026AbBOC GLVMLTNKRIPNEERIKAAYAVLNAIKK
DC-73WP_001211219.1_1019AbBOC GLVMLTNKRIPNEERIKAAYAVLNAIKK
DC-73WP_001211219.1_1024AbBOC GLVMLTNKRIPNEERIKAAYAVLNAIKK
ADC-73WP_001211219.1_930AbBOC GLVMLTNKRIPNEERIKAAYAVLNAIKK
DC-73WP_001211219.1_2609AbBOC GLVMLTNKRIPNEERIKAAYAVLNAIKK
DC-73WP_001211219.1_3456AbBOC GLVMLTNKRIPNEERIKAAYAVLNAIKK
DC-73WP_001211219.1_2614AbBOC GLVMLTNKRIPNEERIKAAYAVLNAIKK
DC-73WP_001211219.1_1025AbBOC GLVMLTNKRIPNEERIKAAYAVLNAIKK
DC-73WP_001211219.1_1030AbBOC GLVMLTNKRIPNEERIKAAYAVLNAIKK
DC-73WP_001211219.1_1173AbBOC GLVMLTNKRIPNEERIKAAYAVLNAIKK
DC-73WP_001211219.1_1021AbBOC GLVMLTNKRIPNEERIKAAYAVLNAIKK
DC-73WP_001211219.1_2449AbBOC GLVMLTNKRIPNEERIKAAYAVLNAIKK
DC-73WP_001211219.1_1176AbBOC GLVMLTNKRIPNEERIKAAYAVLNAIKK
DC-73WP_001211219.1_1161AbBOC GLVMLTNKRIPNEERIKAAYAVLNAIKK
DC-73WP_001211219.1_1070AbBOC GLVMLTNKRIPNEERIKAAYAVLNAIKK
DC-73WP_001211219.1_1079AbBOC GLVMLTNKRIPNEERIKAAYAVLNAIKK
DC-73WP_001211219.1_1078AbBOC GLVMLTNKRIPNEERIKAAYAVLNAIKK
DC-73WP_001211219.1_1037AbBOC GLVMLTNKRIPNEERIKAAYAVLNAIKK
DC-73WP_001211219.1_1035AbBOC GLVMLTNKRIPNEERIKAAYAVLNAIKK
DC-73WP_001211219.1_1033AbBOC GLVMLTNKRIPNEERIKAAYAVLNAIKK
DC-73WP_001211219.1_1041AbBOC GLVMLTNKRIPNEERIKAAYAVLNAIKK
DC-73WP_001211219.1_1023AbBOC GLVMLTNKRIPNEERIKAAYAVLNAIKK
ADC-73USX69547.1_2386AbBOC GLVMLTNKRIPNEERIKAAYAVLNAIKK
DC-73WP_001211219.1_1034AbBOC GLVMLTNKRIPNEERIKAAYAVLNAIKK
ADC-73WP_001211219.1_852AbBOC GLVMLTNKRIPNEERIKAAYAVLNAIKK
DC-73WP_001211219.1_3070AbBOC GLVMLTNKRIPNEERIKAAYAVLNAIKK
ADC-73WBS60953.1_1026AbBOC GLVMLTNKRIPNEERIKAAYAVLNAIKK
ADC-73WDC39660.1_1023AbBOC GLVMLTNKRIPNEERIKAAYAVLNAIKK
ADC-73WDC73972.1_1415AbBOC GLVMLTNKRIPNEERIKAAYAVLNAIKK
ADC-73WDC70172.1_1037AbBOC GLVMLTNKRIPNEERIKAAYAVLNAIKK
DC-73WP_001211219.1_1101AbBOC GLVMLTNKRIPNEERIKAAYAVLNAIKK
DC-73WP_001211219.1_1667AbBOC GLVMLTNKRIPNEERIKAAYAVLNAIKK
ADC-73WP_001211219.1_287AbBOC GLVMLTNKRIPNEERIKAAYAVLNAIKK
DC-73WP_001211219.1_3177AbBOC GLVMLTNKRIPNEERIKAAYAVLNAIKK
DC-73WP_001211219.1_2501AbBOC GLVMLTNKRIPNEERIKAAYAVLNAIKK
DC-73WP_001211219.1_2535AbBOC GLVMLTNKRIPNEERIKAAYAVLNAIKK
DC-73WP_001211219.1_3150AbBOC GLVMLTNKRIPNEERIKAAYAVLNAIKK
DC-73WP_001211219.1_3714AbBOC GLVMLTNKRIPNEERIKAAYAVLNAIKK
ADC-73WP_001211219.1_722AbBOC GLVMLTNKRIPNEERIKAAYAVLNAIKK
DC-73WP_001211219.1_3650AbBOC GLVMLTNKRIPNEERIKAAYAVLNAIKK
ADC-73WP_001211219.1_568AbBOC GLVMLTNKRIPNEERIKAAYAVLNAIKK
DC-73WP_001211219.1_3226AbBOC GLVMLTNKRIPNEERIKAAYAVLNAIKK
ADC-73WP_001211219.1_556AbBOC GLVMLTNKRIPNEERIKAAYAVLNAIKK

DC-73WP_001211219.1_2436AbBOC	GLVMLTNKRIPNEERIKAAYAVLNAIKK
DC-73WP_001211219.1_1029AbBOC	GLVMLTNKRIPNEERIKAAYAVLNAIKK
DC-73WP_001211219.1_1013AbBOC	GLVMLTNKRIPNEERIKAAYAVLNAIKK
ADC-73WP_001211219.1_906AbBCP	GLVMLTNKRIPNEERIKAAYAVLNAIKK
DC-73WP_001211219.1_2649AbBCP	GLVMLTNKRIPNEERIKAAYAVLNAIKK
DC-73WP_001211219.1_2693AbBCP	GLVMLTNKRIPNEERIKAAYAVLNAIKK
ADC-73AQU57925.1_2703AbBCP	GLVMLTNKRIPNEERIKAAYAVLNAIKK
DC-73WP_001211219.1_1026AbBCP	GLVMLTNKRIPNEERIKAAYAVLNAIKK
DC-73WP_001211219.1_1019AbBCP	GLVMLTNKRIPNEERIKAAYAVLNAIKK
DC-73WP_001211219.1_1024AbBCP	GLVMLTNKRIPNEERIKAAYAVLNAIKK
ADC-73WP_001211219.1_930AbBCP	GLVMLTNKRIPNEERIKAAYAVLNAIKK
DC-73WP_001211219.1_2609AbBCP	GLVMLTNKRIPNEERIKAAYAVLNAIKK
DC-73WP_001211219.1_3456AbBCP	GLVMLTNKRIPNEERIKAAYAVLNAIKK
DC-73WP_001211219.1_2614AbBCP	GLVMLTNKRIPNEERIKAAYAVLNAIKK
DC-73WP_001211219.1_1025AbBCP	GLVMLTNKRIPNEERIKAAYAVLNAIKK
DC-73WP_001211219.1_1030AbBCP	GLVMLTNKRIPNEERIKAAYAVLNAIKK
DC-73WP_001211219.1_1173AbBCP	GLVMLTNKRIPNEERIKAAYAVLNAIKK
DC-73WP_001211219.1_1021AbBCP	GLVMLTNKRIPNEERIKAAYAVLNAIKK
DC-73WP_001211219.1_2449AbBCP	GLVMLTNKRIPNEERIKAAYAVLNAIKK
DC-73WP_001211219.1_1176AbBCP	GLVMLTNKRIPNEERIKAAYAVLNAIKK
DC-73WP_001211219.1_1161AbBCP	GLVMLTNKRIPNEERIKAAYAVLNAIKK
DC-73WP_001211219.1_1070AbBCP	GLVMLTNKRIPNEERIKAAYAVLNAIKK
DC-73WP_001211219.1_1079AbBCP	GLVMLTNKRIPNEERIKAAYAVLNAIKK
DC-73WP_001211219.1_1078AbBCP	GLVMLTNKRIPNEERIKAAYAVLNAIKK
DC-73WP_001211219.1_1037AbBCP	GLVMLTNKRIPNEERIKAAYAVLNAIKK
DC-73WP_001211219.1_1035AbBCP	GLVMLTNKRIPNEERIKAAYAVLNAIKK
DC-73WP_001211219.1_1033AbBCP	GLVMLTNKRIPNEERIKAAYAVLNAIKK
DC-73WP_001211219.1_1041AbBCP	GLVMLTNKRIPNEERIKAAYAVLNAIKK
DC-73WP_001211219.1_1023AbBCP	GLVMLTNKRIPNEERIKAAYAVLNAIKK
ADC-73USX69547.1_2386AbBCP	GLVMLTNKRIPNEERIKAAYAVLNAIKK
DC-73WP_001211219.1_1034AbBCP	GLVMLTNKRIPNEERIKAAYAVLNAIKK
ADC-73WP_001211219.1_852AbBCP	GLVMLTNKRIPNEERIKAAYAVLNAIKK
DC-73WP_001211219.1_3070AbBCP	GLVMLTNKRIPNEERIKAAYAVLNAIKK
ADC-73WBS60953.1_1026AbBCP	GLVMLTNKRIPNEERIKAAYAVLNAIKK
ADC-73WDC39660.1_1023AbBCP	GLVMLTNKRIPNEERIKAAYAVLNAIKK
ADC-73WDC73972.1_1415AbBCP	GLVMLTNKRIPNEERIKAAYAVLNAIKK
ADC-73WDC70172.1_1037AbBCP	GLVMLTNKRIPNEERIKAAYAVLNAIKK
DC-73WP_001211219.1_1101AbBCP	GLVMLTNKRIPNEERIKAAYAVLNAIKK
DC-73WP_001211219.1_1667AbBCP	GLVMLTNKRIPNEERIKAAYAVLNAIKK
ADC-73WP_001211219.1_287AbBCP	GLVMLTNKRIPNEERIKAAYAVLNAIKK
DC-73WP_001211219.1_3177AbBCP	GLVMLTNKRIPNEERIKAAYAVLNAIKK
DC-73WP_001211219.1_2501AbBCP	GLVMLTNKRIPNEERIKAAYAVLNAIKK
DC-73WP_001211219.1_2535AbBCP	GLVMLTNKRIPNEERIKAAYAVLNAIKK
DC-73WP_001211219.1_3150AbBCP	GLVMLTNKRIPNEERIKAAYAVLNAIKK
DC-73WP_001211219.1_3714AbBCP	GLVMLTNKRIPNEERIKAAYAVLNAIKK
ADC-73WP_001211219.1_722AbBCP	GLVMLTNKRIPNEERIKAAYAVLNAIKK
DC-73WP_001211219.1_3650AbBCP	GLVMLTNKRIPNEERIKAAYAVLNAIKK
ADC-73WP_001211219.1_568AbBCP	GLVMLTNKRIPNEERIKAAYAVLNAIKK
DC-73WP_001211219.1_3226AbBCP	GLVMLTNKRIPNEERIKAAYAVLNAIKK
ADC-73WP_001211219.1_556AbBCP	GLVMLTNKRIPNEERIKAAYAVLNAIKK
DC-73WP_001211219.1_2436AbBCP	GLVMLTNKRIPNEERIKAAYAVLNAIKK
DC-73WP_001211219.1_1029AbBCP	GLVMLTNKRIPNEERIKAAYAVLNAIKK
DC-73WP_001211219.1_1013AbBCP	GLVMLTNKRIPNEERIKAAYAVLNAIKK
ADC-73BDE18748.1_1038AbBCP	GLVMLTNKRIPNEERIKAAYAVLNAIKK
DC-115WP_001211215.1_2833AbOC	GLVMLTNKRIPNEERIKAAYAVLNAIKK
C-115WP_001211215.1_1156AbBOC	GLVMLTNKRIPNEERIKAAYAVLNAIKK
C-115WP_001211215.1_1156AbBCP	GLVMLTNKRIPNEERIKAAYAVLNAIKK
ADC-5WP_038405930.1_2542AbOC	GLVMLTNKRIPNEERIKAAYAVLNAIKK
ADC-5WP_038405930.1_1113AbBOC	GLVMLTNKRIPNEERIKAAYAVLNAIKK
ADC-5WP_038405930.1_1113AbBCP	GLVMLTNKRIPNEERIKAAYAVLNAIKK
C-236WP_176673601.1_1026AbBOC	GLVMLTNKRIPNEERIKAAYAVLNAIKE
C-236WP_176673601.1_1026AbBCP	GLVMLTNKRIPNEERIKAAYAVLNAIKE
DC-217WP_060470109.1_3584AbOC	GLVMLTNKRIPNEERIKAAYAVLNAIKK
ADC-217USX66614.1_2290AbBOC	GLVMLTNKRIPNEERIKAAYAVLNAIKK
ADC-217USX66614.1_2290AbBCP	GLVMLTNKRIPNEERIKAAYAVLNAIKK
ADC-247WP_032058755.1_382AbOC	GLVMLTNKRIPNEERIKAAYAVLNAIKE
C-247WP_032058755.1_1936AbBOC	GLVMLTNKRIPNEERIKAAYAVLNAIKE
C-247WP_032058755.1_1936AbBCP	GLVMLTNKRIPNEERIKAAYAVLNAIKE
DC-203WP_032000414.1_2940AbOC	GLVMLTNKRIPNEERIKAAYAVLNAIKK
C-203WP_032000414.1_1062AbBOC	GLVMLTNKRIPNEERIKAAYAVLNAIKK
DC-203WP_032000414.1_129AbBOC	GLVMLTNKRIPNEERIKAAYAVLNAIKK
C-203WP_032000414.1_2518AbBOC	GLVMLTNKRIPNEERIKAAYAVLNAIKK
C-203WP_032000414.1_1062AbBCP	GLVMLTNKRIPNEERIKAAYAVLNAIKK
DC-203WP_032000414.1_129AbBCP	GLVMLTNKRIPNEERIKAAYAVLNAIKK
C-203WP_032000414.1_2518AbBCP	GLVMLTNKRIPNEERIKAAYAVLNAIKK
DC-152WP_001211233.1_3734AbOC	GLVMLTNKRIPNEERIKAAYAVLNAIKE
C-152WP_001211233.1_1080AbBOC	GLVMLTNKRIPNEERIKAAYAVLNAIKE
DC-152WP_001211233.1_491AbBOC	GLVMLTNKRIPNEERIKAAYAVLNAIKE
ADC-152WP_001211233.1_46AbBOC	GLVMLTNKRIPNEERIKAAYAVLNAIKE
C-152WP_001211233.1_1545AbBOC	GLVMLTNKRIPNEERIKAAYAVLNAIKE
ADC-152WBS86980.1_1289AbBOC	GLVMLTNKRIPNEERIKAAYAVLNAIKE
ADC-152WBS81072.1_2581AbBOC	GLVMLTNKRIPNEERIKAAYAVLNAIKE
ADC-152WBS88504.1_2711AbBOC	GLVMLTNKRIPNEERIKAAYAVLNAIKE
ADC-152WBS92168.1_2401AbBOC	GLVMLTNKRIPNEERIKAAYAVLNAIKE
ADC-152WBS79617.1_1289AbBOC	GLVMLTNKRIPNEERIKAAYAVLNAIKE
C-152WP_001211233.1_1080AbBCP	GLVMLTNKRIPNEERIKAAYAVLNAIKE
DC-152WP_001211233.1_491AbBCP	GLVMLTNKRIPNEERIKAAYAVLNAIKE
ADC-152WP_001211233.1_46AbBCP	GLVMLTNKRIPNEERIKAAYAVLNAIKE
C-152WP_001211233.1_1545AbBCP	GLVMLTNKRIPNEERIKAAYAVLNAIKE
ADC-152WBS86980.1_1289AbBCP	GLVMLTNKRIPNEERIKAAYAVLNAIKE
ADC-152WBS81072.1_2581AbBCP	GLVMLTNKRIPNEERIKAAYAVLNAIKE
ADC-152WBS88504.1_2711AbBCP	GLVMLTNKRIPNEERIKAAYAVLNAIKE
ADC-152WBS92168.1_2401AbBCP	GLVMLTNKRIPNEERIKAAYAVLNAIKE
ADC-152WBS79617.1_1289AbBCP	GLVMLTNKRIPNEERIKAAYAVLNAIKE
ADC-52WP_001211232.1_990AbOC	GLVMLTNKRIPNEERIKAAYAVLNAIKE
DC-52WP_001211232.1_1087AbBOC	GLVMLTNKRIPNEERIKAAYAVLNAIKE
DC-52WP_001211232.1_1087AbBCP	GLVMLTNKRIPNEERIKAAYAVLNAIKE
C-163WP_057093064.1_2397AbBOC	GLVMLTNKRIPNEERIKAAYAVLNAIKK
C-163WP_057093064.1_2397AbBCP	GLVMLTNKRIPNEERIKAAYAVLNAIKK
ADC-33WP_001211220.1_2277AbOC	GLVMLTNKRIPNEERIKAAYAVLNAIKK
DC-33WP_001211220.1_1044AbBOC	GLVMLTNKRIPNEERIKAAYAVLNAIKK
DC-33WP_001211220.1_2452AbBOC	GLVMLTNKRIPNEERIKAAYAVLNAIKK
DC-33WP_001211220.1_1046AbBOC	GLVMLTNKRIPNEERIKAAYAVLNAIKK
DC-33WP_001211220.1_1025AbBOC	GLVMLTNKRIPNEERIKAAYAVLNAIKK
DC-33WP_001211220.1_1026AbBOC	GLVMLTNKRIPNEERIKAAYAVLNAIKK
DC-33WP_001211220.1_1033AbBOC	GLVMLTNKRIPNEERIKAAYAVLNAIKK
DC-33WP_001211220.1_1035AbBOC	GLVMLTNKRIPNEERIKAAYAVLNAIKK
DC-33WP_001211220.1_1041AbBOC	GLVMLTNKRIPNEERIKAAYAVLNAIKK
DC-33WP_001211220.1_1044AbBCP	GLVMLTNKRIPNEERIKAAYAVLNAIKK
DC-33WP_001211220.1_2452AbBCP	GLVMLTNKRIPNEERIKAAYAVLNAIKK
DC-33WP_001211220.1_1046AbBCP	GLVMLTNKRIPNEERIKAAYAVLNAIKK
DC-33WP_001211220.1_1025AbBCP	GLVMLTNKRIPNEERIKAAYAVLNAIKK
DC-33WP_001211220.1_1026AbBCP	GLVMLTNKRIPNEERIKAAYAVLNAIKK
DC-33WP_001211220.1_1033AbBCP	GLVMLTNKRIPNEERIKAAYAVLNAIKK
DC-33WP_001211220.1_1035AbBCP	GLVMLTNKRIPNEERIKAAYAVLNAIKK
DC-33WP_001211220.1_1041AbBCP	GLVMLTNKRIPNEERIKAAYAVLNAIKK
C-212WP_031975357.1_1297AbBOC	GLVMLTNKRIPNEERIKAAYAVLNAIKK
DC-212WP_031975357.1_501AbBOC	GLVMLTNKRIPNEERIKAAYAVLNAIKK
C-212WP_031975357.1_1297AbBCP	GLVMLTNKRIPNEERIKAAYAVLNAIKK
DC-212WP_031975357.1_501AbBCP	GLVMLTNKRIPNEERIKAAYAVLNAIKK
C-182WP_057691114.1_1055AbBOC	GLVMLTNKRIPNEERIKAAYAVLNAIKK
C-182WP_057691114.1_1055AbBCP	GLVMLTNKRIPNEERIKAAYAVLNAIKK
DC-81WP_059262723.1_1208AbBOC	GLVMLTNKRIPNEERIKAAYAVLDAIKK
DC-81WP_059262723.1_2841AbBOC	GLVMLTNKRIPNEERIKAAYAVLDAIKK
DC-81WP_059262723.1_1208AbBCP	GLVMLTNKRIPNEERIKAAYAVLDAIKK
DC-81WP_059262723.1_2841AbBCP	GLVMLTNKRIPNEERIKAAYAVLDAIKK
ADC-176WP_001159761.1_11AbOC	GLVMLTNKRIPNEERIKAAYAVLNAIKK
DC-176WP_001159761.1_3466AbOC	GLVMLTNKRIPNEERIKAAYAVLNAIKK
ADC-176WP_001159761.1_93AbBOP	GLVMLTNKRIPNEERIKAAYAVLNAIKK
DC-176WP_001159761.1_247AbBOC	GLVMLTNKRIPNEERIKAAYAVLNAIKK
ADC-176WP_001159761.1_9AbBOC	GLVMLTNKRIPNEERIKAAYAVLNAIKK
C-176WP_001159761.1_2982AbBOC	GLVMLTNKRIPNEERIKAAYAVLNAIKK
ADC-176QLG80706.1_1971AbBOC	GLVMLTNKRIPNEERIKAAYAVLNAIKK
C-176WP_001159761.1_2851AbBOC	GLVMLTNKRIPNEERIKAAYAVLNAIKK
C-176WP_001159761.1_1220AbBOC	GLVMLTNKRIPNEERIKAAYAVLNAIKK
DC-176WP_001159761.1_386AbBOC	GLVMLTNKRIPNEERIKAAYAVLNAIKK
ADC-176WP_001159761.1_11AbBOC	GLVMLTNKRIPNEERIKAAYAVLNAIKK
DC-176WP_001159761.1_247AbBCP	GLVMLTNKRIPNEERIKAAYAVLNAIKK
ADC-176WP_001159761.1_9AbBCP	GLVMLTNKRIPNEERIKAAYAVLNAIKK
C-176WP_001159761.1_2982AbBCP	GLVMLTNKRIPNEERIKAAYAVLNAIKK
ADC-176QLG80706.1_1971AbBCP	GLVMLTNKRIPNEERIKAAYAVLNAIKK
C-176WP_001159761.1_2851AbBCP	GLVMLTNKRIPNEERIKAAYAVLNAIKK
C-176WP_001159761.1_1220AbBCP	GLVMLTNKRIPNEERIKAAYAVLNAIKK
DC-176WP_001159761.1_386AbBCP	GLVMLTNKRIPNEERIKAAYAVLNAIKK
ADC-176WP_001159761.1_11AbBCP	GLVMLTNKRIPNEERIKAAYAVLNAIKK
ADC-199WP_114166683.1_169AbOC	GLVMLTNKRIPNEERIKAAYAVLNAIKK
DC-199WP_114166683.1_267AbBOC	GLVMLTNKRIPNEERIKAAYAVLNAIKK
DC-199WP_114166683.1_267AbBCP	GLVMLTNKRIPNEERIKAAYAVLNAIKK
acc	

CMH enzyme group

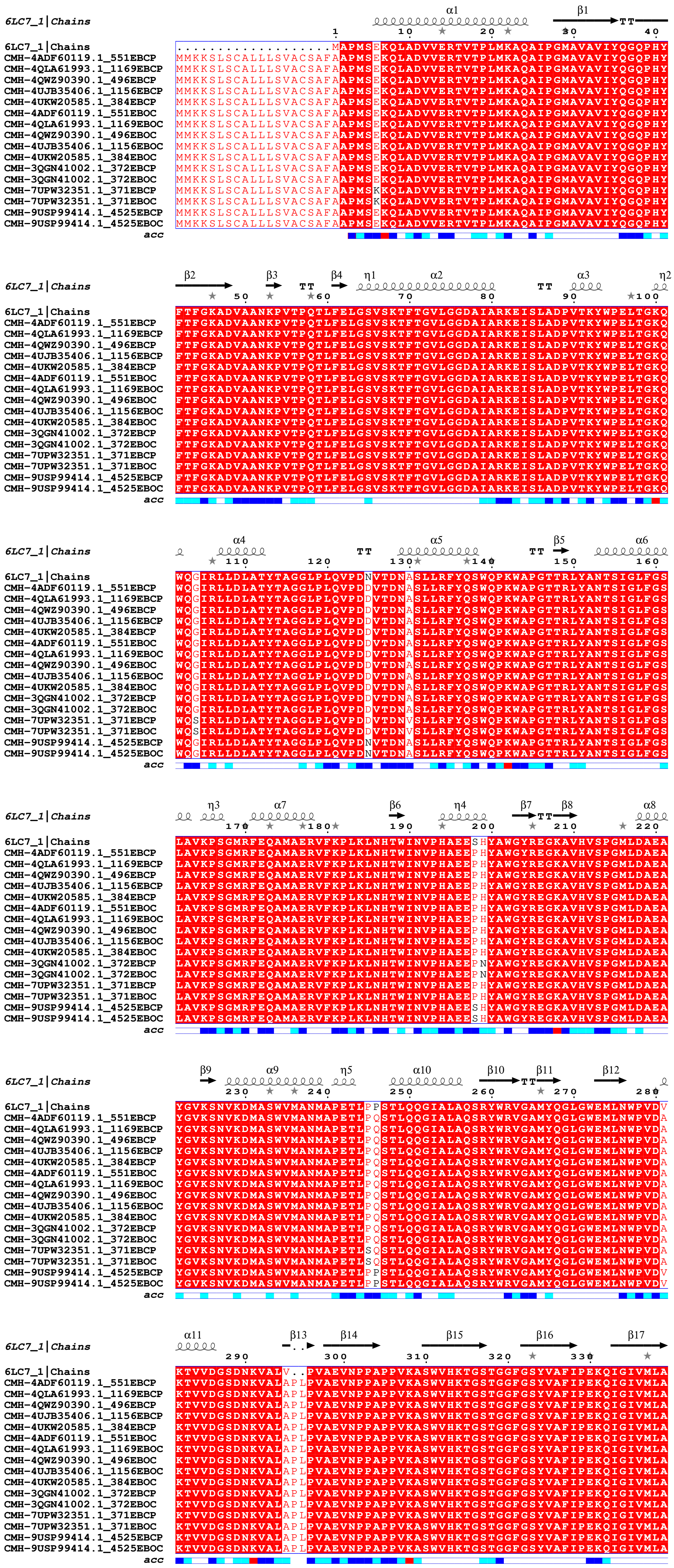

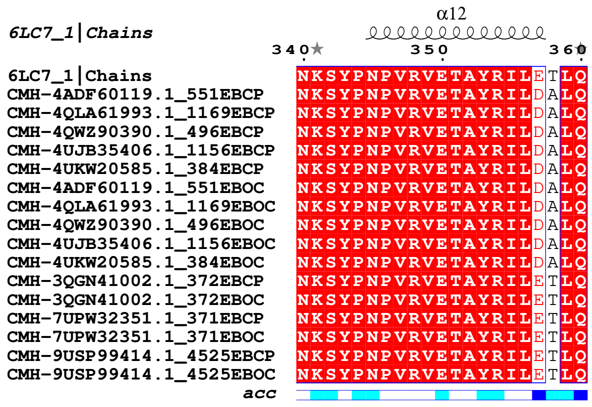

α12
6LC7_1|Chains
340
350
360
6LC7_1|Chains NKSYPNPVRVETAYRILETLQ
CMH-4ADF60119.1_551EBCP NKSYPNPVRVETAYRILDALQ
CMH-4QLA61993.1_1169EBCP NKSYPNPVRVETAYRILDALQ
CMH-4QWZ90390.1_496EBCP NKSYPNPVRVETAYRILDALQ
CMH-4UJB35406.1_1156EBCP NKSYPNPVRVETAYRILDALQ
CMH-4UKW20585.1_384EBCP NKSYPNPVRVETAYRILDALQ
CMH-4ADF60119.1_551EBOC NKSYPNPVRVETAYRILDALQ
CMH-4QLA61993.1_1169EBOC NKSYPNPVRVETAYRILDALQ
CMH-4QWZ90390.1_496EBOC NKSYPNPVRVETAYRILDALQ
CMH-4UJB35406.1_1156EBOC NKSYPNPVRVETAYRILDALQ
CMH-4UKW20585.1_384EBOC NKSYPNPVRVETAYRILDALQ
CMH-3QGN41002.1_372EBCP NKSYPNPVRVETAYRILETLQ
CMH-3QGN41002.1_372EBOC NKSYPNPVRVETAYRILETLQ
CMH-7UPW32351.1_371EBCP NKSYPNPVRVETAYRILETLQ
CMH-7UPW32351.1_371EBOC NKSYPNPVRVETAYRILETLQ
CMH-9USP99414.1_4525EBCP NKSYPNPVRVETAYRILETLQ
CMH-9USP99414.1_4525EBOC NKSYPNPVRVETAYRILETLQ
acc

CMY enzyme group

```
                                                                  α1                   β1
1ZC2_1|Chains                                           QQQQQQQQQQQQQQQQQQQQ    ──────►TT────
                                    1        10        20        30        40
1ZC2_1|Chains                ....................AAKTEQQIADIVNRTITPLMQEQAIPGMAVAVIYQGKPYY
CMY-6APR50799.1_118KpBOP     .MKKSLCCALLLTASFSTFAAAKTEQQIADIVNRTITPLMQEQAIPGMAVAVIYQGKPYY
CMY-172QWN55233.1_12KpBOP    MMKKSLCCALLLTASFSTFAAAKTEQQIADIVNRTITPLMQEQAIPGMAVAVIYQGKPYY
CMY-16UUO75464.1_89KpBOP     MMKKSLCCALLLTASFSTFAAAKTEQQIADIVNRTITPLMQEQAIPGMAVAVIYQGKPYY
CMY-16UUO81049.1_89KpBOP     MMKKSLCCALLLTASFSTFAAAKTEQQIADIVNRTITPLMQEQAIPGMAVAVIYQGKPYY
CMY-16UUO86788.1_89KpBOP     MMKKSLCCALLLTASFSTFAAAKTEQQIADIVNRTITPLMQEQAIPGMAVAVIYQGKPYY
CMY-16UUO92461.1_89KpBOP     MMKKSLCCALLLTASFSTFAAAKTEQQIADIVNRTITPLMQEQAIPGMAVAVIYQGKPYY
CMY-16QDF35631.1_89KpBOP     MMKKSLCCALLLTASFSTFAAAKTEQQIADIVNRTITPLMQEQAIPGMAVAVIYQGKPYY
CMY-16UNX16403.1_84KpBOP     MMKKSLCCALLLTASFSTFAAAKTEQQIADIVNRTITPLMQEQAIPGMAVAVIYQGKPYY
CMY-16UNX21943.1_84KpBOP     MMKKSLCCALLLTASFSTFAAAKTEQQIADIVNRTITPLMQEQAIPGMAVAVIYQGKPYY
CMY-16UNX27570.1_84KpBOP     MMKKSLCCALLLTASFSTFAAAKTEQQIADIVNRTITPLMQEQAIPGMAVAVIYQGKPYY
CMY-16UNX33189.1_84KpBOP     MMKKSLCCALLLTASFSTFAAAKTEQQIADIVNRTITPLMQEQAIPGMAVAVIYQGKPYY
CMY-16UNX38815.1_84KpBOP     MMKKSLCCALLLTASFSTFAAAKTEQQIADIVNRTITPLMQEQAIPGMAVAVIYQGKPYY
CMY-16UPJ08365.1_92KpBOP     MMKKSLCCALLLTASFSTFAAAKTEQQIADIVNRTITPLMQEQAIPGMAVAVIYQGKPYY
CMY-16UZR69464.1_84KpBOP     MMKKSLCCALLLTASFSTFAAAKTEQQIADIVNRTITPLMQEQAIPGMAVAVIYQGKPYY
CMY-2APV17249.1_39KpBOP      MMKKSLCCALLLTASFSTFAAAKTEQQIADIVNRTITPLMQEQAIPGMAVAVIYQGKPYY
CMY-2AWA65772.1_91KpBOP      MMKKSLCCALLLTASFSTFAAAKTEQQIADIVNRTITPLMQEQAIPGMAVAVIYQGKPYY
CMY-2AXR54296.1_245KpBOP     MMKKSLCCALLLTASFSTFAAAKTEQQIADIVNRTITPLMQEQAIPGMAVAVIYQGKPYY
CMY-2QHP23472.1_90KpBOP      MMKKSLCCALLLTASFSTFAAAKTEQQIADIVNRTITPLMQEQAIPGMAVAVIYQGKPYY
CMY-2QDJ85822.1_82KpBOP      MMKKSLCCALLLTASFSTFAAAKTEQQIADIVNRTITPLMQEQAIPGMAVAVIYQGKPYY
CMY-2QPO35250.1_81KpBOP      MMKKSLCCALLLTASFSTFAAAKTEQQIADIVNRTITPLMQEQAIPGMAVAVIYQGKPYY
CMY-2QSS32777.1_201KpBOP     MMKKSLCCALLLTASFSTFAAAKTEQQIADIVNRTITPLMQEQAIPGMAVAVIYQGKPYY
CMY-2QRS83438.1_15KpBOP      MMKKSLCCALLLTASFSTFAAAKTEQQIADIVNRTITPLMQEQAIPGMAVAVIYQGKPYY
CMY-2QRS83685.1_9KpBOP       MMKKSLCCALLLTASFSTFAAAKTEQQIADIVNRTITPLMQEQAIPGMAVAVIYQGKPYY
CMY-2QWC35995.1_96KpBOP      MMKKSLCCALLLTASFSTFAAAKTEQQIADIVNRTITPLMQEQAIPGMAVAVIYQGKPYY
CMY-2QYG29695.1_60KpBOP      MMKKSLCCALLLTASFSTFAAAKTEQQIADIVNRTITPLMQEQAIPGMAVAVIYQGKPYY
CMY-2WHQ94139.1_86KpBOP      MMKKSLCCALLLTASFSTFAAAKTEQQIADIVNRTITPLMQEQAIPGMAVAVIYQGKPYY
CMY-2URI50714.1_76KpBOP      MMKKSLCCALLLTASFSTFAAAKTEQQIADIVNRTITPLMQEQAIPGMAVAVIYQGKPYY
CMY-2UVN34488.1_87KpBOP      MMKKSLCCALLLTASFSTFAAAKTEQQIADIVNRTITPLMQEQAIPGMAVAVIYQGKPYY
CMY-2WGT20880.1_12]PaBOP     MMKKSLCCALLLTASFSTFAAAKTEQQIADIVNRTITPLMQEQAIPGMAVAVIYQGKPYY
CMY-6AHI38880.1_KP_B_O_P     MMKKSLCCALLLTASFSTFAAAKTEQQIADIVNRTITPLMQEQAIPGMAVAVIYQGKPYY
CMY-6ASC37399.1_197KpBOP     MMKKSLCCALLLTASFSTFAAAKTEQQIADIVNRTITPLMQEQAIPGMAVAVIYQGKPYY
CMY-6ASG57098.1_58KpBOP      MMKKSLCCALLLTASFSTFAAAKTEQQIADIVNRTITPLMQEQAIPGMAVAVIYQGKPYY
CMY-6QCU88724.1_132KpBOP     MMKKSLCCALLLTASFSTFAAAKTEQQIADIVNRTITPLMQEQAIPGMAVAVIYQGKPYY
CMY-6QKE17902.1_198KpBOP     MMKKSLCCALLLTASFSTFAAAKTEQQIADIVNRTITPLMQEQAIPGMAVAVIYQGKPYY
CMY-6UOO52757.1_38KpBOP      MMKKSLCCALLLTASFSTFAAAKTEQQIADIVNRTITPLMQEQAIPGMAVAVIYQGKPYY
CMY-6UIM30323.1_9KpBOP       MMKKSLCCALLLTASFSTFAAAKTEQQIADIVNRTITPLMQEQAIPGMAVAVIYQGKPYY
CMY-6WCT39764.1_83KpBOP      MMKKSLCCALLLTASFSTFAAAKTEQQIADIVNRTITPLMQEQAIPGMAVAVIYQGKPYY
CMY-4ART02669.1_5424KpBOC    MMKKSLCCALLLTASFSTFAAAKTEQQIADIVNRTITPLMQEQAIPGMAVAVIYQGKPYY
CMY-4ASC24424.1_4160KpBOC    MMKKSLCCALLLTASFSTFAAAKTEQQIADIVNRTITPLMQEQAIPGMAVAVIYQGKPYY
CMY-4ARX47474.1_15KpBOP      MMKKSLCCALLLTASFSTFAAAKTEQQIADIVNRTITPLMQEQAIPGMAVAVIYQGKPYY
CMY-4QBE75668.1_87KpBOP      MMKKSLCCALLLTASFSTFAAAKTEQQIADIVNRTITPLMQEQAIPGMAVAVIYQGKPYY
CMY-4QBG04957.1_118KpBOP     MMKKSLCCALLLTASFSTFAAAKTEQQIADIVNRTITPLMQEQAIPGMAVAVIYQGKPYY
CMY-4QND06991.1_75KpBOP      MMKKSLCCALLLTASFSTFAAAKTEQQIADIVNRTITPLMQEQAIPGMAVAVIYQGKPYY
CMY-4QUC48797.1_83KpBOP      MMKKSLCCALLLTASFSTFAAAKTEQQIADIVNRTITPLMQEQAIPGMAVAVIYQGKPYY
CMY-4QXQ40376.1_84KpBOP      MMKKSLCCALLLTASFSTFAAAKTEQQIADIVNRTITPLMQEQAIPGMAVAVIYQGKPYY
CMY-4QXQ84010.1_84KpBOP      MMKKSLCCALLLTASFSTFAAAKTEQQIADIVNRTITPLMQEQAIPGMAVAVIYQGKPYY
CMY-4QXU12732.1_84KpBOP      MMKKSLCCALLLTASFSTFAAAKTEQQIADIVNRTITPLMQEQAIPGMAVAVIYQGKPYY
CMY-4USP93622.1_3KpBOP       MMKKSLCCALLLTASFSTFAAAKTEQQIADIVNRTITPLMQEQAIPGMAVAVIYQGKPYY
CMY-4UWX16977.1_88KpBOP      MMKKSLCCALLLTASFSTFAAAKTEQQIADIVNRTITPLMQEQAIPGMAVAVIYQGKPYY
CMY-4UWX22345.1_88KpBOP      MMKKSLCCALLLTASFSTFAAAKTEQQIADIVNRTITPLMQEQAIPGMAVAVIYQGKPYY
CMY-4WP_015056382.1_5342KpBCP MMKKSLCCALLLTASFSTFAAAKTEQQIADIVNRTITPLMQEQAIPGMAVAVIYQGKPYY
CMY-4WP_015056382.1_3939KpBCP MMKKSLCCALLLTASFSTFAAAKTEQQIADIVNRTITPLMQEQAIPGMAVAVIYQGKPYY
CMY-6CRN69_08345_1633KpBOC   MMKKSLCCALLLTASFSTFAAAKTEQQIADIVNRTITPLMQEQAIPGMAVAVIYQGKPYY
CMY-174QQL36767.1_136KpBOP   MMKKSLCCALLLTASFSTFAAAKTEQQIADIVNRTITPLMQEQAIPGMAVAVIYQGKPYY
                        acc

                             β2        β3     β4 η1          α2                     η2
1ZC2_1|Chains                ───────►  ─►  TT ─► QQQQQQQQQQQQQQQQQQQ     TT          QQ
                                      50        60        70        80        90       100
1ZC2_1|Chains                FTWGKADIANNHPVTQQTLFELGSVSKTFNGVLGGDAIARGEIKLSDPVTKYWPELTGKQ
CMY-6APR50799.1_118KpBOP     FTWGKADIANNHPVTQQTLFELGSVSKTFNGVLGGDAIARGEIKLSDPVTKYWPELTGKQ
CMY-172QWN55233.1_12KpBOP    FTWGKADIANNHPVTQQTLFELGSVSKTFNGVLGGDAIARGEIKLSDPVTKYWPELTGKQ
CMY-16UUO75464.1_89KpBOP     FTWGKADIANNHPVTQQTLFELGSVSKTFNGVLGGDAIARGEIKLSDPVTKYWPELTGKQ
CMY-16UUO81049.1_89KpBOP     FTWGKADIANNHPVTQQTLFELGSVSKTFNGVLGGDAIARGEIKLSDPVTKYWPELTGKQ
CMY-16UUO86788.1_89KpBOP     FTWGKADIANNHPVTQQTLFELGSVSKTFNGVLGGDAIARGEIKLSDPVTKYWPELTGKQ
CMY-16UUO92461.1_89KpBOP     FTWGKADIANNHPVTQQTLFELGSVSKTFNGVLGGDAIARGEIKLSDPVTKYWPELTGKQ
CMY-16QDF35631.1_89KpBOP     FTWGKADIANNHPVTQQTLFELGSVSKTFNGVLGGDAIARGEIKLSDPVTKYWPELTGKQ
CMY-16UNX16403.1_84KpBOP     FTWGKADIANNHPVTQQTLFELGSVSKTFNGVLGGDAIARGEIKLSDPVTKYWPELTGKQ
CMY-16UNX21943.1_84KpBOP     FTWGKADIANNHPVTQQTLFELGSVSKTFNGVLGGDAIARGEIKLSDPVTKYWPELTGKQ
CMY-16UNX27570.1_84KpBOP     FTWGKADIANNHPVTQQTLFELGSVSKTFNGVLGGDAIARGEIKLSDPVTKYWPELTGKQ
CMY-16UNX33189.1_84KpBOP     FTWGKADIANNHPVTQQTLFELGSVSKTFNGVLGGDAIARGEIKLSDPVTKYWPELTGKQ
CMY-16UNX38815.1_84KpBOP     FTWGKADIANNHPVTQQTLFELGSVSKTFNGVLGGDAIARGEIKLSDPVTKYWPELTGKQ
CMY-16UPJ08365.1_92KpBOP     FTWGKADIANNHPVTQQTLFELGSVSKTFNGVLGGDAIARGEIKLSDPVTKYWPELTGKQ
CMY-16UZR69464.1_84KpBOP     FTWGKADIANNHPVTQQTLFELGSVSKTFNGVLGGDAIARGEIKLSDPVTKYWPELTGKQ
CMY-2APV17249.1_39KpBOP      FTWGKADIANNHPVTQQTLFELGSVSKTFNGVLGGDAIARGEIKLSDPVTKYWPELTGKQ
CMY-2AWA65772.1_91KpBOP      FTWGKADIANNHPVTQQTLFELGSVSKTFNGVLGGDAIARGEIKLSDPVTKYWPELTGKQ
CMY-2AXR54296.1_245KpBOP     FTWGKADIANNHPVTQQTLFELGSVSKTFNGVLGGDAIARGEIKLSDPVTKYWPELTGKQ
CMY-2QHP23472.1_90KpBOP      FTWGKADIANNHPVTQQTLFELGSVSKTFNGVLGGDAIARGEIKLSDPVTKYWPELTGKQ
CMY-2QDJ85822.1_82KpBOP      FTWGKADIANNHPVTQQTLFELGSVSKTFNGVLGGDAIARGEIKLSDPVTKYWPELTGKQ
CMY-2QPO35250.1_81KpBOP      FTWGKADIANNHPVTQQTLFELGSVSKTFNGVLGGDAIARGEIKLSDPVTKYWPELTGKQ
CMY-2QSS32777.1_201KpBOP     FTWGKADIANNHPVTQQTLFELGSVSKTFNGVLGGDAIARGEIKLSDPVTKYWPELTGKQ
CMY-2QRS83438.1_15KpBOP      FTWGKADIANNHPVTQQTLFELGSVSKTFNGVLGGDAIARGEIKLSDPVTKYWPELTGKQ
CMY-2QRS83685.1_9KpBOP       FTWGKADIANNHPVTQQTLFELGSVSKTFNGVLGGDAIARGEIKLSDPVTKYWPELTGKQ
CMY-2QWC35995.1_96KpBOP      FTWGKADIANNHPVTQQTLFELGSVSKTFNGVLGGDAIARGEIKLSDPVTKYWPELTGKQ
CMY-2QYG29695.1_60KpBOP      FTWGKADIANNHPVTQQTLFELGSVSKTFNGVLGGDAIARGEIKLSDPVTKYWPELTGKQ
CMY-2WHQ94139.1_86KpBOP      FTWGKADIANNHPVTQQTLFELGSVSKTFNGVLGGDAIARGEIKLSDPVTKYWPELTGKQ
CMY-2URI50714.1_76KpBOP      FTWGKADIANNHPVTQQTLFELGSVSKTFNGVLGGDAIARGEIKLSDPVTKYWPELTGKQ
CMY-2UVN34488.1_87KpBOP      FTWGKADIANNHPVTQQTLFELGSVSKTFNGVLGGDAIARGEIKLSDPVTKYWPELTGKQ
CMY-2WGT20880.1_12]PaBOP     FTWGKADIANNHPVTQQTLFELGSVSKTFNGVLGGDAIARGEIKLSDPVTKYWPELTGKQ
CMY-6AHI38880.1_KP_B_O_P     FTWGKADIANNHPVTQQTLFELGSVSKTFNGVLGGDAIARGEIKLSDPVTKYWPELTGKQ
CMY-6ASC37399.1_197KpBOP     FTWGKADIANNHPVTQQTLFELGSVSKTFNGVLGGDAIARGEIKLSDPVTKYWPELTGKQ
CMY-6ASG57098.1_58KpBOP      FTWGKADIANNHPVTQQTLFELGSVSKTFNGVLGGDAIARGEIKLSDPVTKYWPELTGKQ
CMY-6QCU88724.1_132KpBOP     FTWGKADIANNHPVTQQTLFELGSVSKTFNGVLGGDAIARGEIKLSDPVTKYWPELTGKQ
CMY-6QKE17902.1_198KpBOP     FTWGKADIANNHPVTQQTLFELGSVSKTFNGVLGGDAIARGEIKLSDPVTKYWPELTGKQ
CMY-6UOO52757.1_38KpBOP      FTWGKADIANNHPVTQQTLFELGSVSKTFNGVLGGDAIARGEIKLSDPVTKYWPELTGKQ
CMY-6UIM30323.1_9KpBOP       FTWGKADIANNHPVTQQTLFELGSVSKTFNGVLGGDAIARGEIKLSDPVTKYWPELTGKQ
CMY-6WCT39764.1_83KpBOP      FTWGKADIANNHPVTQQTLFELGSVSKTFNGVLGGDAIARGEIKLSDPVTKYWPELTGKQ
CMY-4ART02669.1_5424KpBOC    FTWGKADIANNHPVTQQTLFELGSVSKTFNGVLGGDAIARGEIKLSDPVTKYWPELTGKQ
CMY-4ASC24424.1_4160KpBOC    FTWGKADIANNHPVTQQTLFELGSVSKTFNGVLGGDAIARGEIKLSDPVTKYWPELTGKQ
CMY-4ARX47474.1_15KpBOP      FTWGKADIANNHPVTQQTLFELGSVSKTFNGVLGGDAIARGEIKLSDPVTKYWPELTGKQ
CMY-4QBE75668.1_87KpBOP      FTWGKADIANNHPVTQQTLFELGSVSKTFNGVLGGDAIARGEIKLSDPVTKYWPELTGKQ
CMY-4QBG04957.1_118KpBOP     FTWGKADIANNHPVTQQTLFELGSVSKTFNGVLGGDAIARGEIKLSDPVTKYWPELTGKQ
CMY-4QND06991.1_75KpBOP      FTWGKADIANNHPVTQQTLFELGSVSKTFNGVLGGDAIARGEIKLSDPVTKYWPELTGKQ
CMY-4QUC48797.1_83KpBOP      FTWGKADIANNHPVTQQTLFELGSVSKTFNGVLGGDAIARGEIKLSDPVTKYWPELTGKQ
CMY-4QXQ40376.1_84KpBOP      FTWGKADIANNHPVTQQTLFELGSVSKTFNGVLGGDAIARGEIKLSDPVTKYWPELTGKQ
CMY-4QXQ84010.1_84KpBOP      FTWGKADIANNHPVTQQTLFELGSVSKTFNGVLGGDAIARGEIKLSDPVTKYWPELTGKQ
CMY-4QXU12732.1_84KpBOP      FTWGKADIANNHPVTQQTLFELGSVSKTFNGVLGGDAIARGEIKLSDPVTKYWPELTGKQ
CMY-4USP93622.1_3KpBOP       FTWGKADIANNHPVTQQTLFELGSVSKTFNGVLGGDAIARGEIKLSDPVTKYWPELTGKQ
CMY-4UWX16977.1_88KpBOP      FTWGKADIANNHPVTQQTLFELGSVSKTFNGVLGGDAIARGEIKLSDPVTKYWPELTGKQ
CMY-4UWX22345.1_88KpBOP      FTWGKADIANNHPVTQQTLFELGSVSKTFNGVLGGDAIARGEIKLSDPVTKYWPELTGKQ
CMY-4WP_015056382.1_5342KpBCP FTWGKADIANNHPVTQQTLFELGSVSKTFNGVLGGDAIARGEIKLSDPVTKYWPELTGKQ
CMY-4WP_015056382.1_3939KpBCP FTWGKADIANNHPVTQQTLFELGSVSKTFNGVLGGDAIARGEIKLSDPVTKYWPELTGKQ
CMY-6CRN69_08345_1633KpBOC   FTWGKADIANNHPVTQQTLFELGSVSKTFNGVLGGDAIARGEIKLSDPVTKYWPELTGKQ
CMY-174QQL36767.1_136KpBOP   FTWGKADIANNHPVTQQTLFELGSVSKTFNGVLGGDAIARGEIKLSDPVTKYWPELTGKQ
                        acc
```

```
                                      α3                     α4                 β5      α5
1ZC2_1|Chains                                           TT
                                        110       120       130       140       150       160
1ZC2_1|Chains                      WQGIRLLHLATYTAGGLPLQIPDDVRDKAALLHFYQNWQPQWTPGAKRLYANSSIGLFGA
CMY-6APR50799.1_118KpBOP           WQGIRLLHLATYTAGGLPLQIPDDVRDKAALLHFYQNWQPQWTPGAKRLYANSSIGLFGA
CMY-172QWN55233.1_12KpBOP          WQGIRLLHLATYTAGGLPLQIPDDVRDKAALLHFYQNWQPQWTPGAKRLYANSSIGLFGA
CMY-16UUO75464.1_89KpBOP           WQGIRLLHLATYTAGGLPLQIPDDVRDKAALLHFYQNWQPQWTPGAKRLYSNSSIGLFGA
CMY-16UUO81049.1_89KpBOP           WQGIRLLHLATYTAGGLPLQIPDDVRDKAALLHFYQNWQPQWTPGAKRLYSNSSIGLFGA
CMY-16UUO86788.1_89KpBOP           WQGIRLLHLATYTAGGLPLQIPDDVRDKAALLHFYQNWQPQWTPGAKRLYSNSSIGLFGA
CMY-16UUO92461.1_89KpBOP           WQGIRLLHLATYTAGGLPLQIPDDVRDKAALLHFYQNWQPQWTPGAKRLYSNSSIGLFGA
CMY-16QDF35631.1_89KpBOP           WQGIRLLHLATYTAGGLPLQIPDDVRDKAALLHFYQNWQPQWTPGAKRLYSNSSIGLFGA
CMY-16UNX16403.1_84KpBOP           WQGIRLLHLATYTAGGLPLQIPDDVRDKAALLHFYQNWQPQWTPGAKRLYSNSSIGLFGA
CMY-16UNX21943.1_84KpBOP           WQGIRLLHLATYTAGGLPLQIPDDVRDKAALLHFYQNWQPQWTPGAKRLYSNSSIGLFGA
CMY-16UNX27570.1_84KpBOP           WQGIRLLHLATYTAGGLPLQIPDDVRDKAALLHFYQNWQPQWTPGAKRLYSNSSIGLFGA
CMY-16UNX33189.1_84KpBOP           WQGIRLLHLATYTAGGLPLQIPDDVRDKAALLHFYQNWQPQWTPGAKRLYSNSSIGLFGA
CMY-16UNX38815.1_84KpBOP           WQGIRLLHLATYTAGGLPLQIPDDVRDKAALLHFYQNWQPQWTPGAKRLYSNSSIGLFGA
CMY-16UPJ08365.1_92KpBOP           WQGIRLLHLATYTAGGLPLQIPDDVRDKAALLHFYQNWQPQWTPGAKRLYSNSSIGLFGA
CMY-16UZR69464.1_84KpBOP           WQGIRLLHLATYTAGGLPLQIPDDVRDKAALLHFYQNWQPQWTPGAKRLYSNSSIGLFGA
CMY-2APV17249.1_39KpBOP            WQGIRLLHLATYTAGGLPLQIPDDVRDKAALLHFYQNWQPQWTPGAKRLYANSSIGLFGA
CMY-2AWA65772.1_91KpBOP            WQGIRLLHLATYTAGGLPLQIPDDVRDKAALLHFYQNWQPQWTPGAKRLYANSSIGLFGA
CMY-2AXR54296.1_245KpBOP           WQGIRLLHLATYTAGGLPLQIPDDVRDKAALLHFYQNWQPQWTPGAKRLYANSSIGLFGA
CMY-2QHP23472.1_90KpBOP            WQGIRLLHLATYTAGGLPLQIPDDVRDKAALLHFYQNWQPQWTPGAKRLYANSSIGLFGA
CMY-2QDJ85822.1_82KpBOP            WQGIRLLHLATYTAGGLPLQIPDDVRDKAALLHFYQNWQPQWTPGAKRLYANSSIGLFGA
CMY-2QPO35250.1_81KpBOP            WQGIRLLHLATYTAGGLPLQIPDDVRDKAALLHFYQNWQPQWTPGAKRLYANSSIGLFGA
CMY-2QSS32777.1_201KpBOP           WQGIRLLHLATYTAGGLPLQIPDDVRDKAALLHFYQNWQPQWTPGAKRLYANSSIGLFGA
CMY-2QRS83438.1_15KpBOP            WQGIRLLHLATYTAGGLPLQIPDDVRDKAALLHFYQNWQPQWTPGAKRLYANSSIGLFGA
CMY-2QRS83685.1_9KpBOP             WQGIRLLHLATYTAGGLPLQIPDDVRDKAALLHFYQNWQPQWTPGAKRLYANSSIGLFGA
CMY-2QWC35995.1_96KpBOP            WQGIRLLHLATYTAGGLPLQIPDDVRDKAALLHFYQNWQPQWTPGAKRLYANSSIGLFGA
CMY-2QYG29695.1_60KpBOP            WQGIRLLHLATYTAGGLPLQIPDDVRDKAALLHFYQNWQPQWTPGAKRLYANSSIGLFGA
CMY-2WHQ94139.1_86KpBOP            WQGIRLLHLATYTAGGLPLQIPDDVRDKAALLHFYQNWQPQWTPGAKRLYANSSIGLFGA
CMY-2URI50714.1_76KpBOP            WQGIRLLHLATYTAGGLPLQIPDDVRDKAALLHFYQNWQPQWTPGAKRLYANSSIGLFGA
CMY-2UVN34488.1_87KpBOP            WQGIRLLHLATYTAGGLPLQIPDDVRDKAALLHFYQNWQPQWTPGAKRLYANSSIGLFGA
CMY-2WGT20880.1_12]PaBOP           WQGIRLLHLATYTAGGLPLQIPDDVRDKAALLHFYQNWQPQWTPGAKRLYANSSIGLFGA
CMY-6AHI38880.1_KP_B_O_P           WQGIRLLHLATYTAGGLPLQIPDDVRDKAALLHFYQNWQPQWTPGAKRLYANSSIGLFGA
CMY-6ASC37399.1_197KpBOP           WQGIRLLHLATYTAGGLPLQIPDDVRDKAALLHFYQNWQPQWTPGAKRLYANSSIGLFGA
CMY-6ASG57098.1_58KpBOP            WQGIRLLHLATYTAGGLPLQIPDDVRDKAALLHFYQNWQPQWTPGAKRLYANSSIGLFGA
CMY-6QCU88724.1_132KpBOP           WQGIRLLHLATYTAGGLPLQIPDDVRDKAALLHFYQNWQPQWTPGAKRLYANSSIGLFGA
CMY-6QKE17902.1_198KpBOP           WQGIRLLHLATYTAGGLPLQIPDDVRDKAALLHFYQNWQPQWTPGAKRLYANSSIGLFGA
CMY-6UOO52757.1_38KpBOP            WQGIRLLHLATYTAGGLPLQIPDDVRDKAALLHFYQNWQPQWTPGAKRLYANSSIGLFGA
CMY-6UIM30323.1_9KpBOP             WQGIRLLHLATYTAGGLPLQIPDDVRDKAALLHFYQNWQPQWTPGAKRLYANSSIGLFGA
CMY-6WCT39764.1_83KpBOP            WQGIRLLHLATYTAGGLPLQIPDDVRDKAALLHFYQNWQPQWTPGAKRLYANSSIGLFGA
CMY-4ART02669.1_5424KpBOC          WQGIRLLHLATYTAGGLPLQIPDDVRDKAALLHFYQNWQPQWTPGAKRLYANSSIGLFGA
CMY-4ASC24424.1_4160KpBOC          WQGIRLLHLATYTAGGLPLQIPDDVRDKAALLHFYQNWQPQWTPGAKRLYANSSIGLFGA
CMY-4ARX47474.1_15KpBOP            WQGIRLLHLATYTAGGLPLQIPDDVRDKAALLHFYQNWQPQWTPGAKRLYANSSIGLFGA
CMY-4QBE75668.1_87KpBOP            WQGIRLLHLATYTAGGLPLQIPDDVRDKAALLHFYQNWQPQWTPGAKRLYANSSIGLFGA
CMY-4QBG04957.1_118KpBOP           WQGIRLLHLATYTAGGLPLQIPDDVRDKAALLHFYQNWQPQWTPGAKRLYANSSIGLFGA
CMY-4QND06991.1_75KpBOP            WQGIRLLHLATYTAGGLPLQIPDDVRDKAALLHFYQNWQPQWTPGAKRLYANSSIGLFGA
CMY-4QUC48797.1_83KpBOP            WQGIRLLHLATYTAGGLPLQIPDDVRDKAALLHFYQNWQPQWTPGAKRLYANSSIGLFGA
CMY-4QXQ40376.1_84KpBOP            WQGIRLLHLATYTAGGLPLQIPDDVRDKAALLHFYQNWQPQWTPGAKRLYANSSIGLFGA
CMY-4QXQ84010.1_84KpBOP            WQGIRLLHLATYTAGGLPLQIPDDVRDKAALLHFYQNWQPQWTPGAKRLYANSSIGLFGA
CMY-4QXU12732.1_84KpBOP            WQGIRLLHLATYTAGGLPLQIPDDVRDKAALLHFYQNWQPQWTPGAKRLYANSSIGLFGA
CMY-4USP93622.1_3KpBOP             WQGIRLLHLATYTAGGLPLQIPDDVRDKAALLHFYQNWQPQWTPGAKRLYANSSIGLFGA
CMY-4UWX16977.1_88KpBOP            WQGIRLLHLATYTAGGLPLQIPDDVRDKAALLHFYQNWQPQWTPGAKRLYANSSIGLFGA
CMY-4UWX22345.1_88KpBOP            WQGIRLLHLATYTAGGLPLQIPDDVRDKAALLHFYQNWQPQWTPGAKRLYANSSIGLFGA
CMY-4WP_015056382.1_5342KpBCP      WQGIRLLHLATYTAGGLPLQIPDDVRDKAALLHFYQNWQPQWTPGAKRLYANSSIGLFGA
CMY-4WP_015056382.1_3939KpBCP      WQGIRLLHLATYTAGGLPLQIPDDVRDKAALLHFYQNWQPQWTPGAKRLYANSSIGLFGA
CMY-6CRN69_08345_1633KpBOC        WQGIRLLHLATYTAGGLPLQIPDDVRDKAALLHFYQNWQPQWTPGAKRLYANSSIGLFGA
CMY-174QQL36767.1_136KpBOP         WQGIRLLHLATYTAGGLPLQIPDDVRDKAALLHFYQNWQPQWTPGAKRLYANSSIGLFGA
                              acc
```

```
                                      η3     α6                β6      η4      β7   β8          α7
1ZC2_1|Chains                                                                       TT
                                        170       180       190       200       210       220
1ZC2_1|Chains                      LAVKPSGMSYEEAMTRRVLQPLKLAHTWITVPQNEQKDYAWGYREGKPVHVSPGQLDAEA
CMY-6APR50799.1_118KpBOP           LAVKPSGMSYEEAMTRRVLQPLKLAHTWITVPQNEQKDYALGYREGKPVHVSPGQLDAEA
CMY-172QWN55233.1_12KpBOP          LAVKPSGMSYEEAMTRRVLQPLKLAHTWITVPQNEQKDYAWGYREGKPVHVSPGQLDAEA
CMY-16UUO75464.1_89KpBOP           LAVKPSGMSYEEAMTRRVLQPLKLAHTWITVPQNEQKDYARGYREGKPVHVSPGQLDAEA
CMY-16UUO81049.1_89KpBOP           LAVKPSGMSYEEAMTRRVLQPLKLAHTWITVPQNEQKDYARGYREGKPVHVSPGQLDAEA
CMY-16UUO86788.1_89KpBOP           LAVKPSGMSYEEAMTRRVLQPLKLAHTWITVPQNEQKDYARGYREGKPVHVSPGQLDAEA
CMY-16UUO92461.1_89KpBOP           LAVKPSGMSYEEAMTRRVLQPLKLAHTWITVPQNEQKDYARGYREGKPVHVSPGQLDAEA
CMY-16QDF35631.1_89KpBOP           LAVKPSGMSYEEAMTRRVLQPLKLAHTWITVPQNEQKDYARGYREGKPVHVSPGQLDAEA
CMY-16UNX16403.1_84KpBOP           LAVKPSGMSYEEAMTRRVLQPLKLAHTWITVPQNEQKDYARGYREGKPVHVSPGQLDAEA
CMY-16UNX21943.1_84KpBOP           LAVKPSGMSYEEAMTRRVLQPLKLAHTWITVPQNEQKDYARGYREGKPVHVSPGQLDAEA
CMY-16UNX27570.1_84KpBOP           LAVKPSGMSYEEAMTRRVLQPLKLAHTWITVPQNEQKDYARGYREGKPVHVSPGQLDAEA
CMY-16UNX33189.1_84KpBOP           LAVKPSGMSYEEAMTRRVLQPLKLAHTWITVPQNEQKDYARGYREGKPVHVSPGQLDAEA
CMY-16UNX38815.1_84KpBOP           LAVKPSGMSYEEAMTRRVLQPLKLAHTWITVPQNEQKDYARGYREGKPVHVSPGQLDAEA
CMY-16UPJ08365.1_92KpBOP           LAVKPSGMSYEEAMTRRVLQPLKLAHTWITVPQNEQKDYARGYREGKPVHVSPGQLDAEA
CMY-16UZR69464.1_84KpBOP           LAVKPSGMSYEEAMTRRVLQPLKLAHTWITVPQNEQKDYARGYREGKPVHVSPGQLDAEA
CMY-2APV17249.1_39KpBOP            LAVKPSGMSYEEAMTRRVLQPLKLAHTWITVPQNEQKDYAWGYREGKPVHVSPGQLDAEA
CMY-2AWA65772.1_91KpBOP            LAVKPSGMSYEEAMTRRVLQPLKLAHTWITVPQNEQKDYAWGYREGKPVHVSPGQLDAEA
CMY-2AXR54296.1_245KpBOP           LAVKPSGMSYEEAMTRRVLQPLKLAHTWITVPQNEQKDYAWGYREGKPVHVSPGQLDAEA
CMY-2QHP23472.1_90KpBOP            LAVKPSGMSYEEAMTRRVLQPLKLAHTWITVPQNEQKDYAWGYREGKPVHVSPGQLDAEA
CMY-2QDJ85822.1_82KpBOP            LAVKPSGMSYEEAMTRRVLQPLKLAHTWITVPQNEQKDYAWGYREGKPVHVSPGQLDAEA
CMY-2QPO35250.1_81KpBOP            LAVKPSGMSYEEAMTRRVLQPLKLAHTWITVPQNEQKDYAWGYREGKPVHVSPGQLDAEA
CMY-2QSS32777.1_201KpBOP           LAVKPSGMSYEEAMTRRVLQPLKLAHTWITVPQNEQKDYAWGYREGKPVHVSPGQLDAEA
CMY-2QRS83438.1_15KpBOP            LAVKPSGMSYEEAMTRRVLQPLKLAHTWITVPQNEQKDYAWGYREGKPVHVSPGQLDAEA
CMY-2QRS83685.1_9KpBOP             LAVKPSGMSYEEAMTRRVLQPLKLAHTWITVPQNEQKDYAWGYREGKPVHVSPGQLDAEA
CMY-2QWC35995.1_96KpBOP            LAVKPSGMSYEEAMTRRVLQPLKLAHTWITVPQNEQKDYAWGYREGKPVHVSPGQLDAEA
CMY-2QYG29695.1_60KpBOP            LAVKPSGMSYEEAMTRRVLQPLKLAHTWITVPQNEQKDYAWGYREGKPVHVSPGQLDAEA
CMY-2WHQ94139.1_86KpBOP            LAVKPSGMSYEEAMTRRVLQPLKLAHTWITVPQNEQKDYAWGYREGKPVHVSPGQLDAEA
CMY-2URI50714.1_76KpBOP            LAVKPSGMSYEEAMTRRVLQPLKLAHTWITVPQNEQKDYAWGYREGKPVHVSPGQLDAEA
CMY-2UVN34488.1_87KpBOP            LAVKPSGMSYEEAMTRRVLQPLKLAHTWITVPQNEQKDYAWGYREGKPVHVSPGQLDAEA
CMY-2WGT20880.1_12]PaBOP           LAVKPSGMSYEEAMTRRVLQPLKLAHTWITVPQNEQKDYAWGYREGKPVHVSPGQLDAEA
CMY-6AHI38880.1_KP_B_O_P           LAVKPSGMSYEEAMTRRVLQPLKLAHTWITVPQNEQKDYALGYREGKPVHVSPGQLDAEA
CMY-6ASC37399.1_197KpBOP           LAVKPSGMSYEEAMTRRVLQPLKLAHTWITVPQNEQKDYALGYREGKPVHVSPGQLDAEA
CMY-6ASG57098.1_58KpBOP            LAVKPSGMSYEEAMTRRVLQPLKLAHTWITVPQNEQKDYALGYREGKPVHVSPGQLDAEA
CMY-6QCU88724.1_132KpBOP           LAVKPSGMSYEEAMTRRVLQPLKLAHTWITVPQNEQKDYALGYREGKPVHVSPGQLDAEA
CMY-6QKE17902.1_198KpBOP           LAVKPSGMSYEEAMTRRVLQPLKLAHTWITVPQNEQKDYALGYREGKPVHVSPGQLDAEA
CMY-6UOO52757.1_38KpBOP            LAVKPSGMSYEEAMTRRVLQPLKLAHTWITVPQNEQKDYALGYREGKPVHVSPGQLDAEA
CMY-6UIM30323.1_9KpBOP             LAVKPSGMSYEEAMTRRVLQPLKLAHTWITVPQNEQKDYALGYREGKPVHVSPGQLDAEA
CMY-6WCT39764.1_83KpBOP            LAVKPSGMSYEEAMTRRVLQPLKLAHTWITVPQNEQKDYALGYREGKPVHVSPGQLDAEA
CMY-4ART02669.1_5424KpBOC          LAVKPSGMSYEEAMTRRVLQPLKLAHTWITVPQNEQKDYARGYREGKPVHVSPGQLDAEA
CMY-4ASC24424.1_4160KpBOC          LAVKPSGMSYEEAMTRRVLQPLKLAHTWITVPQNEQKDYARGYREGKPVHVSPGQLDAEA
CMY-4ARX47474.1_15KpBOP            LAVKPSGMSYEEAMTRRVLQPLKLAHTWITVPQNEQKDYARGYREGKPVHVSPGQLDAEA
CMY-4QBE75668.1_87KpBOP            LAVKPSGMSYEEAMTRRVLQPLKLAHTWITVPQNEQKDYARGYREGKPVHVSPGQLDAEA
CMY-4QBG04957.1_118KpBOP           LAVKPSGMSYEEAMTRRVLQPLKLAHTWITVPQNEQKDYARGYREGKPVHVSPGQLDAEA
CMY-4QND06991.1_75KpBOP            LAVKPSGMSYEEAMTRRVLQPLKLAHTWITVPQNEQKDYARGYREGKPVHVSPGQLDAEA
CMY-4QUC48797.1_83KpBOP            LAVKPSGMSYEEAMTRRVLQPLKLAHTWITVPQNEQKDYARGYREGKPVHVSPGQLDAEA
CMY-4QXQ40376.1_84KpBOP            LAVKPSGMSYEEAMTRRVLQPLKLAHTWITVPQNEQKDYARGYREGKPVHVSPGQLDAEA
CMY-4QXQ84010.1_84KpBOP            LAVKPSGMSYEEAMTRRVLQPLKLAHTWITVPQNEQKDYARGYREGKPVHVSPGQLDAEA
CMY-4QXU12732.1_84KpBOP            LAVKPSGMSYEEAMTRRVLQPLKLAHTWITVPQNEQKDYARGYREGKPVHVSPGQLDAEA
CMY-4USP93622.1_3KpBOP             LAVKPSGMSYEEAMTRRVLQPLKLAHTWITVPQNEQKDYARGYREGKPVHVSPGQLDAEA
CMY-4UWX16977.1_88KpBOP            LAVKPSGMSYEEAMTRRVLQPLKLAHTWITVPQNEQKDYARGYREGKPVHVSPGQLDAEA
CMY-4UWX22345.1_88KpBOP            LAVKPSGMSYEEAMTRRVLQPLKLAHTWITVPQNEQKDYARGYREGKPVHVSPGQLDAEA
CMY-4WP_015056382.1_5342KpBCP      LAVKPSGMSYEEAMTRRVLQPLKLAHTWITVPQNEQKDYARGYREGKPVHVSPGQLDAEA
CMY-4WP_015056382.1_3939KpBCP      LAVKPSGMSYEEAMTRRVLQPLKLAHTWITVPQNEQKDYARGYREGKPVHVSPGQLDAEA
CMY-6CRN69_08345_1633KpBOC        LAVKPSGMSYEEAMTRRVLQPLKLAHTWITVPQNEQKDYALGYREGKPVHVSPGQLDAEA
CMY-174QQL36767.1_136KpBOP         LAVKPSGMSYEEAMTRRVLQPLKLAHTWITVPQNEQKDYAWGYREGKPVHVSPGQLDAEA
                              acc
```

```
1ZC2_1|Chains                     β9      α8             TT        α9          β10  TT β11   β12       
                                          230       240       250       260       270       280
1ZC2_1|Chains                     YGVKSSVIDMARWVQANMDASHVQEKTLQQGIALAQSRYWRIGDMYQGLGWEMLNWPLKA
CMY-6APR50799.1_118KpBOP          YGVKSSVIDMARWVQANMDASHVQEKTLQQGIALAQSRYWRIGDMYQGLGWEMLNWPLKA
CMY-172QWN55233.1_12KpBOP         YGVKSSVIDMARWVQANMDASHVQEKTLQQGIALAQSRYWRIGDMYQGLGWEMLNWPLKA
CMY-16UUO75464.1_89KpBOP          YGVKSSVIDMARWVQANMDASHVQEKTLQQGIALAQSRYWRIGDMYQGLGWEMLNWPLKA
CMY-16UUO81049.1_89KpBOP          YGVKSSVIDMARWVQANMDASHVQEKTLQQGIALAQSRYWRIGDMYQGLGWEMLNWPLKA
CMY-16UUO86788.1_89KpBOP          YGVKSSVIDMARWVQANMDASHVQEKTLQQGIALAQSRYWRIGDMYQGLGWEMLNWPLKA
CMY-16UUO92461.1_89KpBOP          YGVKSSVIDMARWVQANMDASHVQEKTLQQGIALAQSRYWRIGDMYQGLGWEMLNWPLKA
CMY-16QDF35631.1_89KpBOP          YGVKSSVIDMARWVQANMDASHVQEKTLQQGIALAQSRYWRIGDMYQGLGWEMLNWPLKA
CMY-16UNX16403.1_84KpBOP          YGVKSSVIDMARWVQANMDASHVQEKTLQQGIALAQSRYWRIGDMYQGLGWEMLNWPLKA
CMY-16UNX21943.1_84KpBOP          YGVKSSVIDMARWVQANMDASHVQEKTLQQGIALAQSRYWRIGDMYQGLGWEMLNWPLKA
CMY-16UNX27570.1_84KpBOP          YGVKSSVIDMARWVQANMDASHVQEKTLQQGIALAQSRYWRIGDMYQGLGWEMLNWPLKA
CMY-16UNX33189.1_84KpBOP          YGVKSSVIDMARWVQANMDASHVQEKTLQQGIALAQSRYWRIGDMYQGLGWEMLNWPLKA
CMY-16UNX38815.1_84KpBOP          YGVKSSVIDMARWVQANMDASHVQEKTLQQGIALAQSRYWRIGDMYQGLGWEMLNWPLKA
CMY-16UPJ08365.1_92KpBOP          YGVKSSVIDMARWVQANMDASHVQEKTLQQGIALAQSRYWRIGDMYQGLGWEMLNWPLKA
CMY-16UZR69464.1_84KpBOP          YGVKSSVIDMARWVQANMDASHVQEKTLQQGIALAQSRYWRIGDMYQGLGWEMLNWPLKA
CMY-2APV17249.1_39KpBOP           YGVKSSVIDMARWVQANMDASHVQEKTLQQGIALAQSRYWRIGDMYQGLGWEMLNWPLKA
CMY-2AWA65772.1_91KpBOP           YGVKSSVIDMARWVQANMDASHVQEKTLQQGIALAQSRYWRIGDMYQGLGWEMLNWPLKA
CMY-2AXR54296.1_245KpBOP          YGVKSSVIDMARWVQANMDASHVQEKTLQQGIALAQSRYWRIGDMYQGLGWEMLNWPLKA
CMY-2QHP23472.1_90KpBOP           YGVKSSVIDMARWVQANMDASHVQEKTLQQGIALAQSRYWRIGDMYQGLGWEMLNWPLKA
CMY-2QDJ85822.1_82KpBOP           YGVKSSVIDMARWVQANMDASHVQEKTLQQGIALAQSRYWRIGDMYQGLGWEMLNWPLKA
CMY-2QPO35250.1_81KpBOP           YGVKSSVIDMARWVQANMDASHVQEKTLQQGIALAQSRYWRIGDMYQGLGWEMLNWPLKA
CMY-2QSS32777.1_201KpBOP          YGVKSSVIDMARWVQANMDASHVQEKTLQQGIALAQSRYWRIGDMYQGLGWEMLNWPLKA
CMY-2QRS83438.1_15KpBOP           YGVKSSVIDMARWVQANMDASHVQEKTLQQGIALAQSRYWRIGDMYQGLGWEMLNWPLKA
CMY-2QRS83685.1_9KpBOP            YGVKSSVIDMARWVQANMDASHVQEKTLQQGIALAQSRYWRIGDMYQGLGWEMLNWPLKA
CMY-2QWC35995.1_96KpBOP           YGVKSSVIDMARWVQANMDASHVQEKTLQQGIALAQSRYWRIGDMYQGLGWEMLNWPLKA
CMY-2QYG29695.1_60KpBOP           YGVKSSVIDMARWVQANMDASHVQEKTLQQGIALAQSRYWRIGDMYQGLGWEMLNWPLKA
CMY-2WHQ94139.1_86KpBOP           YGVKSSVIDMARWVQANMDASHVQEKTLQQGIALAQSRYWRIGDMYQGLGWEMLNWPLKA
CMY-2URI50714.1_76KpBOP           YGVKSSVIDMARWVQANMDASHVQEKTLQQGIALAQSRYWRIGDMYQGLGWEMLNWPLKA
CMY-2UVN34488.1_87KpBOP           YGVKSSVIDMARWVQANMDASHVQEKTLQQGIALAQSRYWRIGDMYQGLGWEMLNWPLKA
CMY-2WGT20880.1_12]PaBOP          YGVKSSVIDMARWVQANMDASHVQEKTLQQGIALAQSRYWRIGDMYQGLGWEMLNWPLKA
CMY-6AHI38880.1_KP_B_O_P          YGVKSSVIDMARWVQANMDASHVQEKTLQQGIALAQSRYWRIGDMYQGLGWEMLNWPLKA
CMY-6ASC37399.1_197KpBOP          YGVKSSVIDMARWVQANMDASHVQEKTLQQGIALAQSRYWRIGDMYQGLGWEMLNWPLKA
CMY-6ASG57098.1_58KpBOP           YGVKSSVIDMARWVQANMDASHVQEKTLQQGIALAQSRYWRIGDMYQGLGWEMLNWPLKA
CMY-6QCU88724.1_132KpBOP          YGVKSSVIDMARWVQANMDASHVQEKTLQQGIALAQSRYWRIGDMYQGLGWEMLNWPLKA
CMY-6QKE17902.1_198KpBOP          YGVKSSVIDMARWVQANMDASHVQEKTLQQGIALAQSRYWRIGDMYQGLGWEMLNWPLKA
CMY-6UOO52757.1_38KpBOP           YGVKSSVIDMARWVQANMDASHVQEKTLQQGIALAQSRYWRIGDMYQGLGWEMLNWPLKA
CMY-6UIM30323.1_9KpBOP            YGVKSSVIDMARWVQANMDASHVQEKTLQQGIALAQSRYWRIGDMYQGLGWEMLNWPLKA
CMY-6WCT39764.1_83KpBOP           YGVKSSVIDMARWVQANMDASHVQEKTLQQGIALAQSRYWRIGDMYQGLGWEMLNWPLKA
CMY-4ART02669.1_5424KpBOC         YGVKSSVIDMARWVQANMDASHVQEKTLQQGIALAQSRYWRIGDMYQGLGWEMLNWPLKA
CMY-4ASC24424.1_4160KpBOC         YGVKSSVIDMARWVQANMDASHVQEKTLQQGIALAQSRYWRIGDMYQGLGWEMLNWPLKA
CMY-4ARX47474.1_15KpBOP           YGVKSSVIDMARWVQANMDASHVQEKTLQQGIALAQSRYWRIGDMYQGLGWEMLNWPLKA
CMY-4QBE75668.1_87KpBOP           YGVKSSVIDMARWVQANMDASHVQEKTLQQGIALAQSRYWRIGDMYQGLGWEMLNWPLKA
CMY-4QBG04957.1_118KpBOP          YGVKSSVIDMARWVQANMDASHVQEKTLQQGIALAQSRYWRIGDMYQGLGWEMLNWPLKA
CMY-4QND06991.1_75KpBOP           YGVKSSVIDMARWVQANMDASHVQEKTLQQGIALAQSRYWRIGDMYQGLGWEMLNWPLKA
CMY-4QUC48797.1_83KpBOP           YGVKSSVIDMARWVQANMDASHVQEKTLQQGIALAQSRYWRIGDMYQGLGWEMLNWPLKA
CMY-4QXQ40376.1_84KpBOP           YGVKSSVIDMARWVQANMDASHVQEKTLQQGIALAQSRYWRIGDMYQGLGWEMLNWPLKA
CMY-4QXQ84010.1_84KpBOP           YGVKSSVIDMARWVQANMDASHVQEKTLQQGIALAQSRYWRIGDMYQGLGWEMLNWPLKA
CMY-4QXU12732.1_84KpBOP           YGVKSSVIDMARWVQANMDASHVQEKTLQQGIALAQSRYWRIGDMYQGLGWEMLNWPLKA
CMY-4USP93622.1_3KpBOP            YGVKSSVIDMARWVQANMDASHVQEKTLQQGIALAQSRYWRIGDMYQGLGWEMLNWPLKA
CMY-4UWX16977.1_88KpBOP           YGVKSSVIDMARWVQANMDASHVQEKTLQQGIALAQSRYWRIGDMYQGLGWEMLNWPLKA
CMY-4UWX22345.1_88KpBOP           YGVKSSVIDMARWVQANMDASHVQEKTLQQGIALAQSRYWRIGDMYQGLGWEMLNWPLKA
CMY-4WP_015056382.1_5342KpBCP     YGVKSSVIDMARWVQANMDASHVQEKTLQQGIALAQSRYWRIGDMYQGLGWEMLNWPLKA
CMY-4WP_015056382.1_3939KpBCP     YGVKSSVIDMARWVQANMDASHVQEKTLQQGIALAQSRYWRIGDMYQGLGWEMLNWPLKA
CMY-6CRN69_08345_1633KpBOC       YGVKSSVIDMARWVQANMDASHVQEKTLQQGIALAQSRYWRIGDMYQGLG..........
CMY-174QQL36767.1_136KpBOP        YGVKSSVIDMARWVQANMDASHVQEKTLQQGIALAQSRYWRIGDMYQGLGWEMLNWPLKA
                             acc
```

```
1ZC2_1|Chains                     α10      α11     β13  β14           β15          β16   η5  β17
                                          290       300       310       320       330       340
1ZC2_1|Chains                     DSIINGSDSKVALAALPAVEVNPPAPAVKASWVHKTGSTGGFGSYVAFVPEKNLGIVMLA
CMY-6APR50799.1_118KpBOP          DSIINGSDSKVALAALPAVEVNPPAPAVKASWVHKTGSTGGFGSYVAFVPEKNLGIVMLA
CMY-172QWN55233.1_12KpBOP         DSIINGSDS...LAALPAVEVNPPAPAVKASWVHKTGSTGGFGSYVAFVPEKNLGIVMLA
CMY-16UUO75464.1_89KpBOP          DSIINGSDSKVALAALPAVEVNPPAPAVKASWVHKTGSTGGFGSYVAFVPEKNLGIVMLA
CMY-16UUO81049.1_89KpBOP          DSIINGSDSKVALAALPAVEVNPPAPAVKASWVHKTGSTGGFGSYVAFVPEKNLGIVMLA
CMY-16UUO86788.1_89KpBOP          DSIINGSDSKVALAALPAVEVNPPAPAVKASWVHKTGSTGGFGSYVAFVPEKNLGIVMLA
CMY-16UUO92461.1_89KpBOP          DSIINGSDSKVALAALPAVEVNPPAPAVKASWVHKTGSTGGFGSYVAFVPEKNLGIVMLA
CMY-16QDF35631.1_89KpBOP          DSIINGSDSKVALAALPAVEVNPPAPAVKASWVHKTGSTGGFGSYVAFVPEKNLGIVMLA
CMY-16UNX16403.1_84KpBOP          DSIINGSDSKVALAALPAVEVNPPAPAVKASWVHKTGSTGGFGSYVAFVPEKNLGIVMLA
CMY-16UNX21943.1_84KpBOP          DSIINGSDSKVALAALPAVEVNPPAPAVKASWVHKTGSTGGFGSYVAFVPEKNLGIVMLA
CMY-16UNX27570.1_84KpBOP          DSIINGSDSKVALAALPAVEVNPPAPAVKASWVHKTGSTGGFGSYVAFVPEKNLGIVMLA
CMY-16UNX33189.1_84KpBOP          DSIINGSDSKVALAALPAVEVNPPAPAVKASWVHKTGSTGGFGSYVAFVPEKNLGIVMLA
CMY-16UNX38815.1_84KpBOP          DSIINGSDSKVALAALPAVEVNPPAPAVKASWVHKTGSTGGFGSYVAFVPEKNLGIVMLA
CMY-16UPJ08365.1_92KpBOP          DSIINGSDSKVALAALPAVEVNPPAPAVKASWVHKTGSTGGFGSYVAFVPEKNLGIVMLA
CMY-16UZR69464.1_84KpBOP          DSIINGSDSKVALAALPAVEVNPPAPAVKASWVHKTGSTGGFGSYVAFVPEKNLGIVMLA
CMY-2APV17249.1_39KpBOP           DSIINGSDSKVALAALPAVEVNPPAPAVKASWVHKTGSTGGFGSYVAFVPEKNLGIVMLA
CMY-2AWA65772.1_91KpBOP           DSIINGSDSKVALAALPAVEVNPPAPAVKASWVHKTGSTGGFGSYVAFVPEKNLGIVMLA
CMY-2AXR54296.1_245KpBOP          DSIINGSDSKVALAALPAVEVNPPAPAVKASWVHKTGSTGGFGSYVAFVPEKNLGIVMLA
CMY-2QHP23472.1_90KpBOP           DSIINGSDSKVALAALPAVEVNPPAPAVKASWVHKTGSTGGFGSYVAFVPEKNLGIVMLA
CMY-2QDJ85822.1_82KpBOP           DSIINGSDSKVALAALPAVEVNPPAPAVKASWVHKTGSTGGFGSYVAFVPEKNLGIVMLA
CMY-2QPO35250.1_81KpBOP           DSIINGSDSKVALAALPAVEVNPPAPAVKASWVHKTGSTGGFGSYVAFVPEKNLGIVMLA
CMY-2QSS32777.1_201KpBOP          DSIINGSDSKVALAALPAVEVNPPAPAVKASWVHKTGSTGGFGSYVAFVPEKNLGIVMLA
CMY-2QRS83438.1_15KpBOP           DSIINGSDSKVALAALPAVEVNPPAPAVKASWVHKTGSTGGFGSYVAFVPEKNLGIVMLA
CMY-2QRS83685.1_9KpBOP            DSIINGSDSKVALAALPAVEVNPPAPAVKASWVHKTGSTGGFGSYVAFVPEKNLGIVMLA
CMY-2QWC35995.1_96KpBOP           DSIINGSDSKVALAALPAVEVNPPAPAVKASWVHKTGSTGGFGSYVAFVPEKNLGIVMLA
CMY-2QYG29695.1_60KpBOP           DSIINGSDSKVALAALPAVEVNPPAPAVKASWVHKTGSTGGFGSYVAFVPEKNLGIVMLA
CMY-2WHQ94139.1_86KpBOP           DSIINGSDSKVALAALPAVEVNPPAPAVKASWVHKTGSTGGFGSYVAFVPEKNLGIVMLA
CMY-2URI50714.1_76KpBOP           DSIINGSDSKVALAALPAVEVNPPAPAVKASWVHKTGSTGGFGSYVAFVPEKNLGIVMLA
CMY-2UVN34488.1_87KpBOP           DSIINGSDSKVALAALPAVEVNPPAPAVKASWVHKTGSTGGFGSYVAFVPEKNLGIVMLA
CMY-2WGT20880.1_12]PaBOP          DSIINGSDSKVALAALPAVEVNPPAPAVKASWVHKTGSTGGFGSYVAFVPEKNLGIVMLA
CMY-6AHI38880.1_KP_B_O_P          DSIINGSDSKVALAALPAVEVNPPAPAVKASWVHKTGSTGGFGSYVAFVPEKNLGIVMLA
CMY-6ASC37399.1_197KpBOP          DSIINGSDSKVALAALPAVEVNPPAPAVKASWVHKTGSTGGFGSYVAFVPEKNLGIVMLA
CMY-6ASG57098.1_58KpBOP           DSIINGSDSKVALAALPAVEVNPPAPAVKASWVHKTGSTGGFGSYVAFVPEKNLGIVMLA
CMY-6QCU88724.1_132KpBOP          DSIINGSDSKVALAALPAVEVNPPAPAVKASWVHKTGSTGGFGSYVAFVPEKNLGIVMLA
CMY-6QKE17902.1_198KpBOP          DSIINGSDSKVALAALPAVEVNPPAPAVKASWVHKTGSTGGFGSYVAFVPEKNLGIVMLA
CMY-6UOO52757.1_38KpBOP           DSIINGSDSKVALAALPAVEVNPPAPAVKASWVHKTGSTGGFGSYVAFVPEKNLGIVMLA
CMY-6UIM30323.1_9KpBOP            DSIINGSDSKVALAALPAVEVNPPAPAVKASWVHKTGSTGGFGSYVAFVPEKNLGIVMLA
CMY-6WCT39764.1_83KpBOP           DSIINGSDSKVALAALPAVEVNPPAPAVKASWVHKTGSTGGFGSYVAFVPEKNLGIVMLA
CMY-4ART02669.1_5424KpBOC         DSIINGSDSKVALAALPAVEVNPPAPAVKASWVHKTGSTGGFGSYVAFVPEKNLGIVMLA
CMY-4ASC24424.1_4160KpBOC         DSIINGSDSKVALAALPAVEVNPPAPAVKASWVHKTGSTGGFGSYVAFVPEKNLGIVMLA
CMY-4ARX47474.1_15KpBOP           DSIINGSDSKVALAALPAVEVNPPAPAVKASWVHKTGSTGGFGSYVAFVPEKNLGIVMLA
CMY-4QBE75668.1_87KpBOP           DSIINGSDSKVALAALPAVEVNPPAPAVKASWVHKTGSTGGFGSYVAFVPEKNLGIVMLA
CMY-4QBG04957.1_118KpBOP          DSIINGSDSKVALAALPAVEVNPPAPAVKASWVHKTGSTGGFGSYVAFVPEKNLGIVMLA
CMY-4QND06991.1_75KpBOP           DSIINGSDSKVALAALPAVEVNPPAPAVKASWVHKTGSTGGFGSYVAFVPEKNLGIVMLA
CMY-4QUC48797.1_83KpBOP           DSIINGSDSKVALAALPAVEVNPPAPAVKASWVHKTGSTGGFGSYVAFVPEKNLGIVMLA
CMY-4QXQ40376.1_84KpBOP           DSIINGSDSKVALAALPAVEVNPPAPAVKASWVHKTGSTGGFGSYVAFVPEKNLGIVMLA
CMY-4QXQ84010.1_84KpBOP           DSIINGSDSKVALAALPAVEVNPPAPAVKASWVHKTGSTGGFGSYVAFVPEKNLGIVMLA
CMY-4QXU12732.1_84KpBOP           DSIINGSDSKVALAALPAVEVNPPAPAVKASWVHKTGSTGGFGSYVAFVPEKNLGIVMLA
CMY-4USP93622.1_3KpBOP            DSIINGSDSKVALAALPAVEVNPPAPAVKASWVHKTGSTGGFGSYVAFVPEKNLGIVMLA
CMY-4UWX16977.1_88KpBOP           DSIINGSDSKVALAALPAVEVNPPAPAVKASWVHKTGSTGGFGSYVAFVPEKNLGIVMLA
CMY-4UWX22345.1_88KpBOP           DSIINGSDSKVALAALPAVEVNPPAPAVKASWVHKTGSTGGFGSYVAFVPEKNLGIVMLA
CMY-4WP_015056382.1_5342KpBCP     DSIINGSDSKVALAALPAVEVNPPAPAVKASWVHKTGSTGGFGSYVAFVPEKNLGIVMLA
CMY-4WP_015056382.1_3939KpBCP     DSIINGSDSKVALAALPAVEVNPPAPAVKASWVHKTGSTGGFGSYVAFVPEKNLGIVMLA
CMY-6CRN69_08345_1633KpBOC       ............................................................
CMY-174QQL36767.1_136KpBOP        DSIINGSDSKVALAALPAVEVNPPAPAVKASWVHKTGSTGGFGSYVAFVPEKNLGIVMLA
                             acc
```

1ZC2_1|Chains	α12 (350 … 360)
1ZC2_1|Chains	NKSYPNPVRVEAAWRILEKLQ
CMY-6APR50799.1_118KpBOP	NKSYPNPVRVEAAWRILEKLQ
CMY-172QWN55233.1_12KpBOP	NKSYPIPVRVEAAWRILEKLQ
CMY-16UUO75464.1_89KpBOP	NKSYPNPVRVEAAWRILEKLQ
CMY-16UUO81049.1_89KpBOP	NKSYPNPVRVEAAWRILEKLQ
CMY-16UUO86788.1_89KpBOP	NKSYPNPVRVEAAWRILEKLQ
CMY-16UUO92461.1_89KpBOP	NKSYPNPVRVEAAWRILEKLQ
CMY-16QDF35631.1_89KpBOP	NKSYPNPVRVEAAWRILEKLQ
CMY-16UNX16403.1_84KpBOP	NKSYPNPVRVEAAWRILEKLQ
CMY-16UNX21943.1_84KpBOP	NKSYPNPVRVEAAWRILEKLQ
CMY-16UNX27570.1_84KpBOP	NKSYPNPVRVEAAWRILEKLQ
CMY-16UNX33189.1_84KpBOP	NKSYPNPVRVEAAWRILEKLQ
CMY-16UNX38815.1_84KpBOP	NKSYPNPVRVEAAWRILEKLQ
CMY-16UPJ08365.1_92KpBOP	NKSYPNPVRVEAAWRILEKLQ
CMY-16UZR69464.1_84KpBOP	NKSYPNPVRVEAAWRILEKLQ
CMY-2APV17249.1_39KpBOP	NKSYPNPVRVEAAWRILEKLQ
CMY-2AWA65772.1_91KpBOP	NKSYPNPVRVEAAWRILEKLQ
CMY-2AXR54296.1_245KpBOP	NKSYPNPVRVEAAWRILEKLQ
CMY-2QHP23472.1_90KpBOP	NKSYPNPVRVEAAWRILEKLQ
CMY-2QDJ85822.1_82KpBOP	NKSYPNPVRVEAAWRILEKLQ
CMY-2QPO35250.1_81KpBOP	NKSYPNPVRVEAAWRILEKLQ
CMY-2QSS32777.1_201KpBOP	NKSYPNPVRVEAAWRILEKLQ
CMY-2QRS83438.1_15KpBOP	NKSYPNPVRVEAAWRILEKLQ
CMY-2QRS83685.1_9KpBOP	NKSYPNPVRVEAAWRILEKLQ
CMY-2QWC35995.1_96KpBOP	NKSYPNPVRVEAAWRILEKLQ
CMY-2QYG29695.1_60KpBOP	NKSYPNPVRVEAAWRILEKLQ
CMY-2WHQ94139.1_86KpBOP	NKSYPNPVRVEAAWRILEKLQ
CMY-2URI50714.1_76KpBOP	NKSYPNPVRVEAAWRILEKLQ
CMY-2UVN34488.1_87KpBOP	NKSYPNPVRVEAAWRILEKLQ
CMY-2WGT20880.1_12]PaBOP	NKSYPNPVRVEAAWRILEKLQ
CMY-6AHI38880.1_KP_B_O_P	NKSYPNPVRVEAAWRILEKLQ
CMY-6ASC37399.1_197KpBOP	NKSYPNPVRVEAAWRILEKLQ
CMY-6ASG57098.1_58KpBOP	NKSYPNPVRVEAAWRILEKLQ
CMY-6QCU88724.1_132KpBOP	NKSYPNPVRVEAAWRILEKLQ
CMY-6QKE17902.1_198KpBOP	NKSYPNPVRVEAAWRILEKLQ
CMY-6UOO52757.1_38KpBOP	NKSYPNPVRVEAAWRILEKLQ
CMY-6UIM30323.1_9KpBOP	NKSYPNPVRVEAAWRILEKLQ
CMY-6WCT39764.1_83KpBOP	NKSYPNPVRVEAAWRILEKLQ
CMY-4ART02669.1_5424KpBOC	NKSYPNPVRVEAAWRILEKLQ
CMY-4ASC24424.1_4160KpBOC	NKSYPNPVRVEAAWRILEKLQ
CMY-4ARX47474.1_15KpBOP	NKSYPNPVRVEAAWRILEKLQ
CMY-4QBE75668.1_87KpBOP	NKSYPNPVRVEAAWRILEKLQ
CMY-4QBG04957.1_118KpBOP	NKSYPNPVRVEAAWRILEKLQ
CMY-4QND06991.1_75KpBOP	NKSYPNPVRVEAAWRILEKLQ
CMY-4QUC48797.1_83KpBOP	NKSYPNPVRVEAAWRILEKLQ
CMY-4QXQ40376.1_84KpBOP	NKSYPNPVRVEAAWRILEKLQ
CMY-4QXQ84010.1_84KpBOP	NKSYPNPVRVEAAWRILEKLQ
CMY-4QXU12732.1_84KpBOP	NKSYPNPVRVEAAWRILEKLQ
CMY-4USP93622.1_3KpBOP	NKSYPNPVRVEAAWRILEKLQ
CMY-4UWX16977.1_88KpBOP	NKSYPNPVRVEAAWRILEKLQ
CMY-4UWX22345.1_88KpBOP	NKSYPNPVRVEAAWRILEKLQ
CMY-4WP_015056382.1_5342KpBCP	NKSYPNPVRVEAAWRILEKLQ
CMY-4WP_015056382.1_3939KpBCP	NKSYPNPVRVEAAWRILEKLQ
CMY-6CRN69_08345_1633KpBOC	....
CMY-174QQL36767.1_136KpBOP	NKSYPNPVRVEAARRILEKLQ
acc	

DHA enzyme group

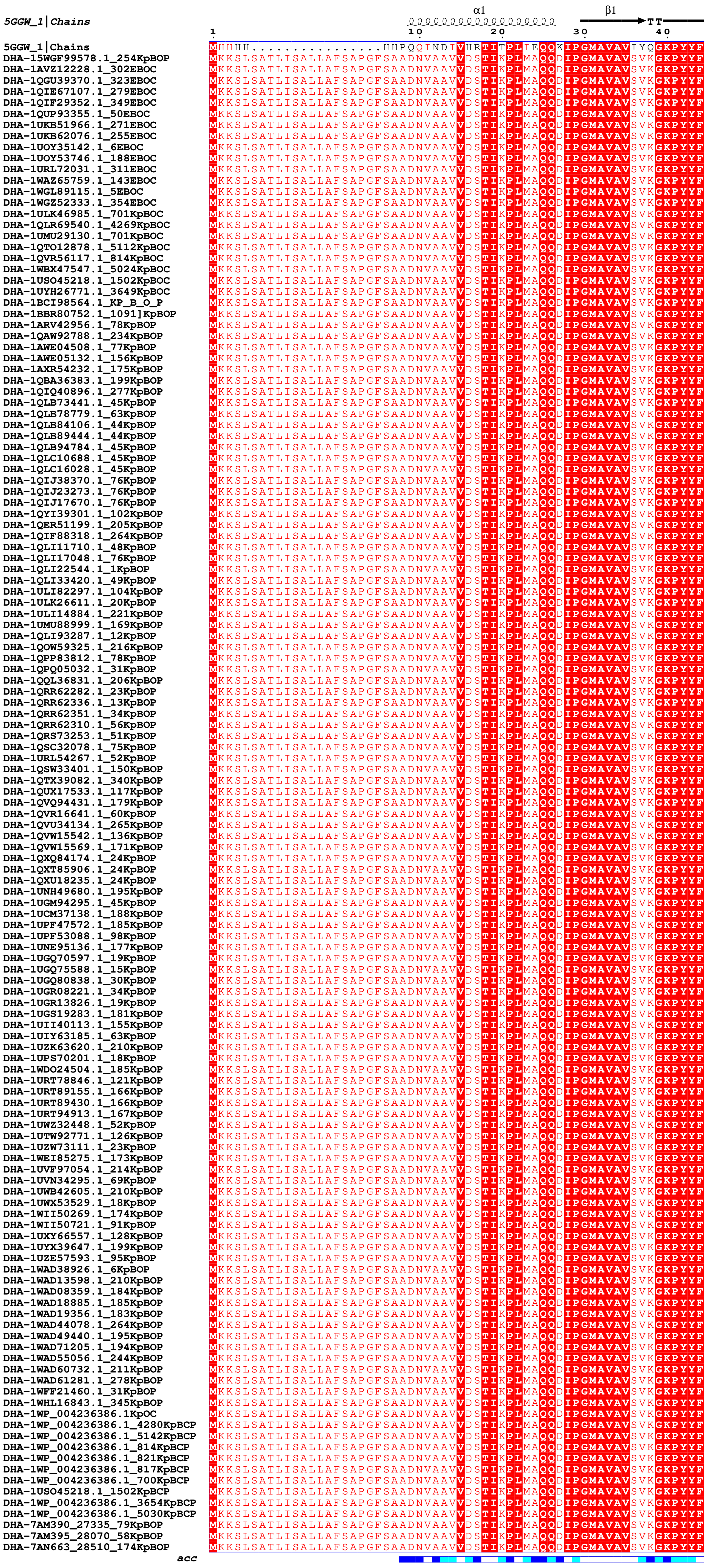

```
                                  β2            β3          β4  η1             α2                   η2       η3
5GGW_1|Chains                                          TT                                   TT        TT
                                       50           60          70          80          90         100
5GGW_1|Chains                     TWGYADIAKKQPVTQQTLFELGSVSKTFTGVLGGDAIARGEIKLSDPATKYWPELTAKQW
DHA-15WGF99578.1_254KpBOP         NYGFADIQAKQPVTENTLFELGSVSKTFTGVLGAVSVAKKEMALNDPAAKYQPELALPQW
DHA-1AVZ12228.1_302EBOC           NYGFADIQAKQPVTENTLFELGSVSKTFTGVLGAVSVAKKEMALNDPAAKYQPELALPQW
DHA-1QGU39370.1_323EBOC           NYGFADIQAKQPVTENTLFELGSVSKTFTGVLGAVSVAKKEMALNDPAAKYQPELALPQW
DHA-1QIE67107.1_279EBOC           NYGFADIQAKQPVTENTLFELGSVSKTFTGVLGAVSVAKKEMALNDPAAKYQPELALPQW
DHA-1QIF29352.1_349EBOC           NYGFADIQAKQPVTENTLFELGSVSKTFTGVLGAVSVAKKEMALNDPAAKYQPELALPQW
DHA-1QUP93355.1_50EBOC            NYGFADIQAKQPVTENTLFELGSVSKTFTGVLGAVSVAKKEMALNDPAAKYQPELALPQW
DHA-1UKB51966.1_271EBOC           NYGFADIQAKQPVTENTLFELGSVSKTFTGVLGAVSVAKKEMALNDPAAKYQPELALPQW
DHA-1UKB62076.1_255EBOC           NYGFADIQAKQPVTENTLFELGSVSKTFTGVLGAVSVAKKEMALNDPAAKYQPELALPQW
DHA-1UOY35142.1_6EBOC             NYGFADIQAKQPVTENTLFELGSVSKTFTGVLGAVSVAKKEMALNDPAAKYQPELALPQW
DHA-1UOY53746.1_188EBOC           NYGFADIQAKQPVTENTLFELGSVSKTFTGVLGAVSVAKKEMALNDPAAKYQPELALPQW
DHA-1URL72031.1_311EBOC           NYGFADIQAKQPVTENTLFELGSVSKTFTGVLGAVSVAKKEMALNDPAAKYQPELALPQW
DHA-1WAZ65759.1_143EBOC           NYGFADIQAKQPVTENTLFELGSVSKTFTGVLGAVSVAKKEMALNDPAAKYQPELALPQW
DHA-1WGL89115.1_5EBOC             NYGFADIQAKQPVTENTLFELGSVSKTFTGVLGAVSVAKKEMALNDPAAKYQPELALPQW
DHA-1WGZ52333.1_354EBOC           NYGFADIQAKQPVTENTLFELGSVSKTFTGVLGAVSVAKKEMALNDPAAKYQPELALPQW
DHA-1ULK46985.1_701KpBOC          NYGFADIQAKQPVTENTLFELGSVSKTFTGVLGAVSVAKKEMALNDPAAKYQPELALPQW
DHA-1QLR69540.1_4269KpBOC         NYGFADIQAKQPVTENTLFELGSVSKTFTGVLGAVSVAKKEMALNDPAAKYQPELALPQW
DHA-1UMU29130.1_701KpBOC          NYGFADIQAKQPVTENTLFELGSVSKTFTGVLGAVSVAKKEMALNDPAAKYQPELALPQW
DHA-1QTO12878.1_5112KpBOC         NYGFADIQAKQPVTENTLFELGSVSKTFTGVLGAVSVAKKEMALNDPAAKYQPELALPQW
DHA-1QVR56117.1_814KpBOC          NYGFADIQAKQPVTENTLFELGSVSKTFTGVLGAVSVAKKEMALNDPAAKYQPELALPQW
DHA-1WBX47547.1_5024KpBOC         NYGFADIQAKQPVTENTLFELGSVSKTFTGVLGAVSVAKKEMALNDPAAKYQPELALPQW
DHA-1USO45218.1_1502KpBOC         NYGFADIQAKQPVTENTLFELGSVSKTFTGVLGAVSVAKKEMALNDPAAKYQPELALPQW
DHA-1UYH26771.1_3649KpBOC         NYGFADIQAKQPVTENTLFELGSVSKTFTGVLGAVSVAKKEMALNDPAAKYQPELALPQW
DHA-1BCI98564.1_KP_B_O_P          NYGFADIQAKQPVTENTLFELGSVSKTFTGVLGAVSVAKKEMALNDPAAKYQPELALPQW
DHA-1BBR80752.1_1091]KpBOP        NYGFADIQAKQPVTENTLFELGSVSKTFTGVLGAVSVAKKEMALNDPAAKYQPELALPQW
DHA-1ARV42956.1_78KpBOP           NYGFADIQAKQPVTENTLFELGSVSKTFTGVLGAVSVAKKEMALNDPAAKYQPELALPQW
DHA-1QAW92788.1_234KpBOP          NYGFADIQAKQPVTENTLFELGSVSKTFTGVLGAVSVAKKEMALNDPAAKYQPELALPQW
DHA-1AWE04508.1_77KpBOP           NYGFADIQAKQPVTENTLFELGSVSKTFTGVLGAVSVAKKEMALNDPAAKYQPELALPQW
DHA-1AWE05132.1_156KpBOP          NYGFADIQAKQPVTENTLFELGSVSKTFTGVLGAVSVAKKEMALNDPAAKYQPELALPQW
DHA-1AXR54232.1_175KpBOP          NYGFADIQAKQPVTENTLFELGSVSKTFTGVLGAVSVAKKEMALNDPAAKYQPELALPQW
DHA-1QBA36383.1_199KpBOP          NYGFADIQAKQPVTENTLFELGSVSKTFTGVLGAVSVAKKEMALNDPAAKYQPELALPQW
DHA-1QIQ40896.1_277KpBOP          NYGFADIQAKQPVTENTLFELGSVSKTFTGVLGAVSVAKKEMALNDPAAKYQPELALPQW
DHA-1QLB73441.1_45KpBOP           NYGFADIQAKQPVTENTLFELGSVSKTFTGVLGAVSVAKKEMALNDPAAKYQPELALPQW
DHA-1QLB78779.1_63KpBOP           NYGFADIQAKQPVTENTLFELGSVSKTFTGVLGAVSVAKKEMALNDPAAKYQPELALPQW
DHA-1QLB84106.1_44KpBOP           NYGFADIQAKQPVTENTLFELGSVSKTFTGVLGAVSVAKKEMALNDPAAKYQPELALPQW
DHA-1QLB89444.1_44KpBOP           NYGFADIQAKQPVTENTLFELGSVSKTFTGVLGAVSVAKKEMALNDPAAKYQPELALPQW
DHA-1QLB94784.1_45KpBOP           NYGFADIQAKQPVTENTLFELGSVSKTFTGVLGAVSVAKKEMALNDPAAKYQPELALPQW
DHA-1QLC10688.1_45KpBOP           NYGFADIQAKQPVTENTLFELGSVSKTFTGVLGAVSVAKKEMALNDPAAKYQPELALPQW
DHA-1QLC16028.1_45KpBOP           NYGFADIQAKQPVTENTLFELGSVSKTFTGVLGAVSVAKKEMALNDPAAKYQPELALPQW
DHA-1QIJ38370.1_76KpBOP           NYGFADIQAKQPVTENTLFELGSVSKTFTGVLGAVSVAKKEMALNDPAAKYQPELALPQW
DHA-1QIJ23273.1_76KpBOP           NYGFADIQAKQPVTENTLFELGSVSKTFTGVLGAVSVAKKEMALNDPAAKYQPELALPQW
DHA-1QIJ17670.1_76KpBOP           NYGFADIQAKQPVTENTLFELGSVSKTFTGVLGAVSVAKKEMALNDPAAKYQPELALPQW
DHA-1QYI39301.1_102KpBOP          NYGFADIQAKQPVTENTLFELGSVSKTFTGVLGAVSVAKKEMALNDPAAKYQPELALPQW
DHA-1QER51199.1_205KpBOP          NYGFADIQAKQPVTENTLFELGSVSKTFTGVLGAVSVAKKEMALNDPAAKYQPELALPQW
DHA-1QIF88318.1_264KpBOP          NYGFADIQAKQPVTENTLFELGSVSKTFTGVLGAVSVAKKEMALNDPAAKYQPELALPQW
DHA-1QLI11710.1_48KpBOP           NYGFADIQAKQPVTENTLFELGSVSKTFTGVLGAVSVAKKEMALNDPAAKYQPELALPQW
DHA-1QLI17048.1_76KpBOP           NYGFADIQAKQPVTENTLFELGSVSKTFTGVLGAVSVAKKEMALNDPAAKYQPELALPQW
DHA-1QLI22544.1_1KpBOP            NYGFADIQAKQPVTENTLFELGSVSKTFTGVLGAVSVAKKEMALNDPAAKYQPELALPQW
DHA-1QLI33420.1_49KpBOP           NYGFADIQAKQPVTENTLFELGSVSKTFTGVLGAVSVAKKEMALNDPAAKYQPELALPQW
DHA-1ULI82297.1_104KpBOP          NYGFADIQAKQPVTENTLFELGSVSKTFTGVLGAVSVAKKEMALNDPAAKYQPELALPQW
DHA-1ULK26611.1_20KpBOP           NYGFADIQAKQPVTENTLFELGSVSKTFTGVLGAVSVAKKEMALNDPAAKYQPELALPQW
DHA-1ULI14884.1_221KpBOP          NYGFADIQAKQPVTENTLFELGSVSKTFTGVLGAVSVAKKEMALNDPAAKYQPELALPQW
DHA-1UMU88999.1_169KpBOP          NYGFADIQAKQPVTENTLFELGSVSKTFTGVLGAVSVAKKEMALNDPAAKYQPELALPQW
DHA-1QLI93287.1_12KpBOP           NYGFADIQAKQPVTENTLFELGSVSKTFTGVLGAVSVAKKEMALNDPAAKYQPELALPQW
DHA-1QOW59325.1_216KpBOP          NYGFADIQAKQPVTENTLFELGSVSKTFTGVLGAVSVAKKEMALNDPAAKYQPELALPQW
DHA-1QPP83812.1_78KpBOP           NYGFADIQAKQPVTENTLFELGSVSKTFTGVLGAVSVAKKEMALNDPAAKYQPELALPQW
DHA-1QPQ05032.1_31KpBOP           NYGFADIQAKQPVTENTLFELGSVSKTFTGVLGAVSVAKKEMALNDPAAKYQPELALPQW
DHA-1QQL36831.1_206KpBOP          NYGFADIQAKQPVTENTLFELGSVSKTFTGVLGAVSVAKKEMALNDPAAKYQPELALPQW
DHA-1QRR62282.1_23KpBOP           NYGFADIQAKQPVTENTLFELGSVSKTFTGVLGAVSVAKKEMALNDPAAKYQPELALPQW
DHA-1QRR62336.1_13KpBOP           NYGFADIQAKQPVTENTLFELGSVSKTFTGVLGAVSVAKKEMALNDPAAKYQPELALPQW
DHA-1QRR62351.1_34KpBOP           NYGFADIQAKQPVTENTLFELGSVSKTFTGVLGAVSVAKKEMALNDPAAKYQPELALPQW
DHA-1QRR62310.1_56KpBOP           NYGFADIQAKQPVTENTLFELGSVSKTFTGVLGAVSVAKKEMALNDPAAKYQPELALPQW
DHA-1QRS73253.1_51KpBOP           NYGFADIQAKQPVTENTLFELGSVSKTFTGVLGAVSVAKKEMALNDPAAKYQPELALPQW
DHA-1QSC32078.1_75KpBOP           NYGFADIQAKQPVTENTLFELGSVSKTFTGVLGAVSVAKKEMALNDPAAKYQPELALPQW
DHA-1URL54267.1_52KpBOP           NYGFADIQAKQPVTENTLFELGSVSKTFTGVLGAVSVAKKEMALNDPAAKYQPELALPQW
DHA-1QSW33401.1_150KpBOP          NYGFADIQAKQPVTENTLFELGSVSKTFTGVLGAVSVAKKEMALNDPAAKYQPELALPQW
DHA-1QTX39082.1_340KpBOP          NYGFADIQAKQPVTENTLFELGSVSKTFTGVLGAVSVAKKEMALNDPAAKYQPELALPQW
DHA-1QUX17533.1_117KpBOP          NYGFADIQAKQPVTENTLFELGSVSKTFTGVLGAVSVAKKEMALNDPAAKYQPELALPQW
DHA-1QVQ94431.1_179KpBOP          NYGFADIQAKQPVTENTLFELGSVSKTFTGVLGAVSVAKKEMALNDPAAKYQPELALPQW
DHA-1QVR16641.1_60KpBOP           NYGFADIQAKQPVTENTLFELGSVSKTFTGVLGAVSVAKKEMALNDPAAKYQPELALPQW
DHA-1QVU34134.1_265KpBOP          NYGFADIQAKQPVTENTLFELGSVSKTFTGVLGAVSVAKKEMALNDPAAKYQPELALPQW
DHA-1QVW15542.1_136KpBOP          NYGFADIQAKQPVTENTLFELGSVSKTFTGVLGAVSVAKKEMALNDPAAKYQPELALPQW
DHA-1QVW15569.1_171KpBOP          NYGFADIQAKQPVTENTLFELGSVSKTFTGVLGAVSVAKKEMALNDPAAKYQPELALPQW
DHA-1QXQ84174.1_24KpBOP           NYGFADIQAKQPVTENTLFELGSVSKTFTGVLGAVSVAKKEMALNDPAAKYQPELALPQW
DHA-1QXT85906.1_24KpBOP           NYGFADIQAKQPVTENTLFELGSVSKTFTGVLGAVSVAKKEMALNDPAAKYQPELALPQW
DHA-1QXU18235.1_24KpBOP           NYGFADIQAKQPVTENTLFELGSVSKTFTGVLGAVSVAKKEMALNDPAAKYQPELALPQW
DHA-1UNH49680.1_195KpBOP          NYGFADIQAKQPVTENTLFELGSVSKTFTGVLGAVSVAKKEMALNDPAAKYQPELALPQW
DHA-1UGM94295.1_45KpBOP           NYGFADIQAKQPVTENTLFELGSVSKTFTGVLGAVSVAKKEMALNDPAAKYQPELALPQW
DHA-1UCM37138.1_188KpBOP          NYGFADIQAKQPVTENTLFELGSVSKTFTGVLGAVSVAKKEMALNDPAAKYQPELALPQW
DHA-1UPF47572.1_185KpBOP          NYGFADIQAKQPVTENTLFELGSVSKTFTGVLGAVSVAKKEMALNDPAAKYQPELALPQW
DHA-1UPF53088.1_98KpBOP           NYGFADIQAKQPVTENTLFELGSVSKTFTGVLGAVSVAKKEMALNDPAAKYQPELALPQW
DHA-1UNE95136.1_177KpBOP          NYGFADIQAKQPVTENTLFELGSVSKTFTGVLGAVSVAKKEMALNDPAAKYQPELALPQW
DHA-1UGQ70597.1_19KpBOP           NYGFADIQAKQPVTENTLFELGSVSKTFTGVLGAVSVAKKEMALNDPAAKYQPELALPQW
DHA-1UGQ75588.1_15KpBOP           NYGFADIQAKQPVTENTLFELGSVSKTFTGVLGAVSVAKKEMALNDPAAKYQPELALPQW
DHA-1UGQ80838.1_30KpBOP           NYGFADIQAKQPVTENTLFELGSVSKTFTGVLGAVSVAKKEMALNDPAAKYQPELALPQW
DHA-1UGR08221.1_34KpBOP           NYGFADIQAKQPVTENTLFELGSVSKTFTGVLGAVSVAKKEMALNDPAAKYQPELALPQW
DHA-1UGR13826.1_19KpBOP           NYGFADIQAKQPVTENTLFELGSVSKTFTGVLGAVSVAKKEMALNDPAAKYQPELALPQW
DHA-1UGS19283.1_181KpBOP          NYGFADIQAKQPVTENTLFELGSVSKTFTGVLGAVSVAKKEMALNDPAAKYQPELALPQW
DHA-1UII40113.1_155KpBOP          NYGFADIQAKQPVTENTLFELGSVSKTFTGVLGAVSVAKKEMALNDPAAKYQPELALPQW
DHA-1UIY63185.1_63KpBOP           NYGFADIQAKQPVTENTLFELGSVSKTFTGVLGAVSVAKKEMALNDPAAKYQPELALPQW
DHA-1UZK63620.1_210KpBOP          NYGFADIQAKQPVTENTLFELGSVSKTFTGVLGAVSVAKKEMALNDPAAKYQPELALPQW
DHA-1UPS70201.1_18KpBOP           NYGFADIQAKQPVTENTLFELGSVSKTFTGVLGAVSVAKKEMALNDPAAKYQPELALPQW
DHA-1WDO24504.1_185KpBOP          NYGFADIQAKQPVTENTLFELGSVSKTFTGVLGAVSVAKKEMALNDPAAKYQPELALPQW
DHA-1URT78846.1_121KpBOP          NYGFADIQAKQPVTENTLFELGSVSKTFTGVLGAVSVAKKEMALNDPAAKYQPELALPQW
DHA-1URT89155.1_166KpBOP          NYGFADIQAKQPVTENTLFELGSVSKTFTGVLGAVSVAKKEMALNDPAAKYQPELALPQW
DHA-1URT89430.1_166KpBOP          NYGFADIQAKQPVTENTLFELGSVSKTFTGVLGAVSVAKKEMALNDPAAKYQPELALPQW
DHA-1URT94913.1_167KpBOP          NYGFADIQAKQPVTENTLFELGSVSKTFTGVLGAVSVAKKEMALNDPAAKYQPELALPQW
DHA-1UWZ32448.1_52KpBOP           NYGFADIQAKQPVTENTLFELGSVSKTFTGVLGAVSVAKKEMALNDPAAKYQPELALPQW
DHA-1UTW92771.1_126KpBOP          NYGFADIQAKQPVTENTLFELGSVSKTFTGVLGAVSVAKKEMALNDPAAKYQPELALPQW
DHA-1UZW73111.1_23KpBOP           NYGFADIQAKQPVTENTLFELGSVSKTFTGVLGAVSVAKKEMALNDPAAKYQPELALPQW
DHA-1WEI85275.1_173KpBOP          NYGFADIQAKQPVTENTLFELGSVSKTFTGVLGAVSVAKKEMALNDPAAKYQPELALPQW
DHA-1UVF97054.1_214KpBOP          NYGFADIQAKQPVTENTLFELGSVSKTFTGVLGAVSVAKKEMALNDPAAKYQPELALPQW
DHA-1UVN34295.1_69KpBOP           NYGFADIQAKQPVTENTLFELGSVSKTFTGVLGAVSVAKKEMALNDPAAKYQPELALPQW
DHA-1UWB42605.1_210KpBOP          NYGFADIQAKQPVTENTLFELGSVSKTFTGVLGAVSVAKKEMALNDPAAKYQPELALPQW
DHA-1UWX53529.1_18KpBOP           NYGFADIQAKQPVTENTLFELGSVSKTFTGVLGAVSVAKKEMALNDPAAKYQPELALPQW
DHA-1WII50269.1_174KpBOP          NYGFADIQAKQPVTENTLFELGSVSKTFTGVLGAVSVAKKEMALNDPAAKYQPELALPQW
DHA-1WII50721.1_91KpBOP           NYGFADIQAKQPVTENTLFELGSVSKTFTGVLGAVSVAKKEMALNDPAAKYQPELALPQW
DHA-1UXY66557.1_128KpBOP          NYGFADIQAKQPVTENTLFELGSVSKTFTGVLGAVSVAKKEMALNDPAAKYQPELALPQW
DHA-1UYX39647.1_199KpBOP          NYGFADIQAKQPVTENTLFELGSVSKTFTGVLGAVSVAKKEMALNDPAAKYQPELALPQW
DHA-1UZE57593.1_95KpBOP           NYGFADIQAKQPVTENTLFELGSVSKTFTGVLGAVSVAKKEMALNDPAAKYQPELALPQW
DHA-1WAD38926.1_6KpBOP            NYGFADIQAKQPVTENTLFELGSVSKTFTGVLGAVSVAKKEMALNDPAAKYQPELALPQW
DHA-1WAD13598.1_210KpBOP          NYGFADIQAKQPVTENTLFELGSVSKTFTGVLGAVSVAKKEMALNDPAAKYQPELALPQW
DHA-1WAD08359.1_184KpBOP          NYGFADIQAKQPVTENTLFELGSVSKTFTGVLGAVSVAKKEMALNDPAAKYQPELALPQW
DHA-1WAD18885.1_185KpBOP          NYGFADIQAKQPVTENTLFELGSVSKTFTGVLGAVSVAKKEMALNDPAAKYQPELALPQW
DHA-1WAD19356.1_183KpBOP          NYGFADIQAKQPVTENTLFELGSVSKTFTGVLGAVSVAKKEMALNDPAAKYQPELALPQW
DHA-1WAD44078.1_264KpBOP          NYGFADIQAKQPVTENTLFELGSVSKTFTGVLGAVSVAKKEMALNDPAAKYQPELALPQW
DHA-1WAD49440.1_195KpBOP          NYGFADIQAKQPVTENTLFELGSVSKTFTGVLGAVSVAKKEMALNDPAAKYQPELALPQW
DHA-1WAD71205.1_194KpBOP          NYGFADIQAKQPVTENTLFELGSVSKTFTGVLGAVSVAKKEMALNDPAAKYQPELALPQW
DHA-1WAD55056.1_244KpBOP          NYGFADIQAKQPVTENTLFELGSVSKTFTGVLGAVSVAKKEMALNDPAAKYQPELALPQW
DHA-1WAD60732.1_211KpBOP          NYGFADIQAKQPVTENTLFELGSVSKTFTGVLGAVSVAKKEMALNDPAAKYQPELALPQW
DHA-1WAD61281.1_278KpBOP          NYGFADIQAKQPVTENTLFELGSVSKTFTGVLGAVSVAKKEMALNDPAAKYQPELALPQW
DHA-1WFF21460.1_31KpBOP           NYGFADIQAKQPVTENTLFELGSVSKTFTGVLGAVSVAKKEMALNDPAAKYQPELALPQW
DHA-1WHL16843.1_345KpBOP          NYGFADIQAKQPVTENTLFELGSVSKTFTGVLGAVSVAKKEMALNDPAAKYQPELALPQW
DHA-1WP_004236386.1KpOC           NYGFADIQAKQPVTENTLFELGSVSKTFTGVLGAVSVAKKEMALNDPAAKYQPELALPQW
DHA-1WP_004236386.1_4280KpBCP     NYGFADIQAKQPVTENTLFELGSVSKTFTGVLGAVSVAKKEMALNDPAAKYQPELALPQW
DHA-1WP_004236386.1_5142KpBCP     NYGFADIQAKQPVTENTLFELGSVSKTFTGVLGAVSVAKKEMALNDPAAKYQPELALPQW
DHA-1WP_004236386.1_814KpBCP      NYGFADIQAKQPVTENTLFELGSVSKTFTGVLGAVSVAKKEMALNDPAAKYQPELALPQW
DHA-1WP_004236386.1_821KpBCP      NYGFADIQAKQPVTENTLFELGSVSKTFTGVLGAVSVAKKEMALNDPAAKYQPELALPQW
DHA-1WP_004236386.1_817KpBCP      NYGFADIQAKQPVTENTLFELGSVSKTFTGVLGAVSVAKKEMALNDPAAKYQPELALPQW
DHA-1WP_004236386.1_700KpBCP      NYGFADIQAKQPVTENTLFELGSVSKTFTGVLGAVSVAKKEMALNDPAAKYQPELALPQW
DHA-1USO45218.1_1502KpBCP         NYGFADIQAKQPVTENTLFELGSVSKTFTGVLGAVSVAKKEMALNDPAAKYQPELALPQW
DHA-1WP_004236386.1_3654KpBCP     NYGFADIQAKQPVTENTLFELGSVSKTFTGVLGAVSVAKKEMALNDPAAKYQPELALPQW
DHA-1WP_004236386.1_5030KpBCP     NYGFADIQAKQPVTENTLFELGSVSKTFTGVLGAVSVAKKEMALNDPAAKYQPELALPQW
DHA-7AM390_27335_79KpBOP          NYGFADIQAKQPVTENTLFELGSVSKTFTGVLGAVSVAKKEMALNDPAAKYQPELALPQW
DHA-7AM395_28070_58KpBOP          NYGFADIQAKQPVTENTLFELGSVSKTFTGVLGAVSVAKKEMALNDPAAKYQPELALPQW
DHA-7AN663_28510_174KpBOP         NYGFADIQAKQPVTENTLFELGSVSKTFTGVLGAVSVAKKEMALNDPAAKYQPELALPQW
                             acc
```

```
                                       α3                       α4               β5        α5
5GGW_1|Chains                         ℓℓℓℓℓ             TT    ℓℓℓℓℓℓℓℓℓℓ       TT →    ℓℓℓℓℓℓℓℓℓℓℓ
                                        110        120        130        140        150        160

5GGW_1|Chains                         NGITLLHLATYTAGGLPLQVPDEVKSSSDLLRFYQNWQPAWAPGTQRLYANSSIGLFGAL
DHA-15WGF99578.1_254KpBOP             KGITLLDLATYTAGGLPLQVPDAVKSRADLLNFYQQWQPSRKPGYMRLYANSSIGLFGAL
DHA-1AVZ12228.1_302EBOC               KGITLLDLATYTAGGLPLQVPDAVKSRADLLNFYQQWQPSRKPGDMRLYANSSIGLFGAL
DHA-1QGU39370.1_323EBOC               KGITLLDLATYTAGGLPLQVPDAVKSRADLLNFYQQWQPSRKPGDMRLYANSSIGLFGAL
DHA-1QIE67107.1_279EBOC               KGITLLDLATYTAGGLPLQVPDAVKSRADLLNFYQQWQPSRKPGDMRLYANSSIGLFGAL
DHA-1QIF29352.1_349EBOC               KGITLLDLATYTAGGLPLQVPDAVKSRADLLNFYQQWQPSRKPGDMRLYANSSIGLFGAL
DHA-1QUP93355.1_50EBOC                KGITLLDLATYTAGGLPLQVPDAVKSRADLLNFYQQWQPSRKPGDMRLYANSSIGLFGAL
DHA-1UKB51966.1_271EBOC               KGITLLDLATYTAGGLPLQVPDAVKSRADLLNFYQQWQPSRKPGDMRLYANSSIGLFGAL
DHA-1UKB62076.1_255EBOC               KGITLLDLATYTAGGLPLQVPDAVKSRADLLNFYQQWQPSRKPGDMRLYANSSIGLFGAL
DHA-1UOY35142.1_6EBOC                 KGITLLDLATYTAGGLPLQVPDAVKSRADLLNFYQQWQPSRKPGDMRLYANSSIGLFGAL
DHA-1UOY53746.1_188EBOC               KGITLLDLATYTAGGLPLQVPDAVKSRADLLNFYQQWQPSRKPGDMRLYANSSIGLFGAL
DHA-1URL72031.1_311EBOC               KGITLLDLATYTAGGLPLQVPDAVKSRADLLNFYQQWQPSRKPGDMRLYANSSIGLFGAL
DHA-1WAZ65759.1_143EBOC               KGITLLDLATYTAGGLPLQVPDAVKSRADLLNFYQQWQPSRKPGDMRLYANSSIGLFGAL
DHA-1WGL89115.1_5EBOC                 KGITLLDLATYTAGGLPLQVPDAVKSRADLLNFYQQWQPSRKPGDMRLYANSSIGLFGAL
DHA-1WGZ52333.1_354EBOC               KGITLLDLATYTAGGLPLQVPDAVKSRADLLNFYQQWQPSRKPGDMRLYANSSIGLFGAL
DHA-1ULK46985.1_701KpBOC              KGITLLDLATYTAGGLPLQVPDAVKSRADLLNFYQQWQPSRKPGDMRLYANSSIGLFGAL
DHA-1QLR69540.1_4269KpBOC             KGITLLDLATYTAGGLPLQVPDAVKSRADLLNFYQQWQPSRKPGDMRLYANSSIGLFGAL
DHA-1UMU29130.1_701KpBOC              KGITLLDLATYTAGGLPLQVPDAVKSRADLLNFYQQWQPSRKPGDMRLYANSSIGLFGAL
DHA-1QTO12878.1_5112KpBOC             KGITLLDLATYTAGGLPLQVPDAVKSRADLLNFYQQWQPSRKPGDMRLYANSSIGLFGAL
DHA-1QVR56117.1_814KpBOC              KGITLLDLATYTAGGLPLQVPDAVKSRADLLNFYQQWQPSRKPGDMRLYANSSIGLFGAL
DHA-1WBX47547.1_5024KpBOC             KGITLLDLATYTAGGLPLQVPDAVKSRADLLNFYQQWQPSRKPGDMRLYANSSIGLFGAL
DHA-1USO45218.1_1502KpBOC             KGITLLDLATYTAGGLPLQVPDAVKSRADLLNFYQQWQPSRKPGDMRLYANSSIGLFGAL
DHA-1UYH26771.1_3649KpBOC             KGITLLDLATYTAGGLPLQVPDAVKSRADLLNFYQQWQPSRKPGDMRLYANSSIGLFGAL
DHA-1BCI98564.1_KP_B_O_P              KGITLLDLATYTAGGLPLQVPDAVKSRADLLNFYQQWQPSRKPGDMRLYANSSIGLFGAL
DHA-1BBR80752.1_1091]KpBOP            KGITLLDLATYTAGGLPLQVPDAVKSRADLLNFYQQWQPSRKPGDMRLYANSSIGLFGAL
DHA-1ARV42956.1_78KpBOP               KGITLLDLATYTAGGLPLQVPDAVKSRADLLNFYQQWQPSRKPGDMRLYANSSIGLFGAL
DHA-1QAW92788.1_234KpBOP              KGITLLDLATYTAGGLPLQVPDAVKSRADLLNFYQQWQPSRKPGDMRLYANSSIGLFGAL
DHA-1AWE04508.1_77KpBOP               KGITLLDLATYTAGGLPLQVPDAVKSRADLLNFYQQWQPSRKPGDMRLYANSSIGLFGAL
DHA-1AWE05132.1_156KpBOP              KGITLLDLATYTAGGLPLQVPDAVKSRADLLNFYQQWQPSRKPGDMRLYANSSIGLFGAL
DHA-1AXR54232.1_175KpBOP              KGITLLDLATYTAGGLPLQVPDAVKSRADLLNFYQQWQPSRKPGDMRLYANSSIGLFGAL
DHA-1QBA36383.1_199KpBOP              KGITLLDLATYTAGGLPLQVPDAVKSRADLLNFYQQWQPSRKPGDMRLYANSSIGLFGAL
DHA-1QIQ40896.1_277KpBOP              KGITLLDLATYTAGGLPLQVPDAVKSRADLLNFYQQWQPSRKPGDMRLYANSSIGLFGAL
DHA-1QLB73441.1_45KpBOP               KGITLLDLATYTAGGLPLQVPDAVKSRADLLNFYQQWQPSRKPGDMRLYANSSIGLFGAL
DHA-1QLB78779.1_63KpBOP               KGITLLDLATYTAGGLPLQVPDAVKSRADLLNFYQQWQPSRKPGDMRLYANSSIGLFGAL
DHA-1QLB84106.1_44KpBOP               KGITLLDLATYTAGGLPLQVPDAVKSRADLLNFYQQWQPSRKPGDMRLYANSSIGLFGAL
DHA-1QLB89444.1_44KpBOP               KGITLLDLATYTAGGLPLQVPDAVKSRADLLNFYQQWQPSRKPGDMRLYANSSIGLFGAL
DHA-1QLB94784.1_45KpBOP               KGITLLDLATYTAGGLPLQVPDAVKSRADLLNFYQQWQPSRKPGDMRLYANSSIGLFGAL
DHA-1QLC10688.1_45KpBOP               KGITLLDLATYTAGGLPLQVPDAVKSRADLLNFYQQWQPSRKPGDMRLYANSSIGLFGAL
DHA-1QLC16028.1_45KpBOP               KGITLLDLATYTAGGLPLQVPDAVKSRADLLNFYQQWQPSRKPGDMRLYANSSIGLFGAL
DHA-1QIJ38370.1_76KpBOP               KGITLLDLATYTAGGLPLQVPDAVKSRADLLNFYQQWQPSRKPGDMRLYANSSIGLFGAL
DHA-1QIJ23273.1_76KpBOP               KGITLLDLATYTAGGLPLQVPDAVKSRADLLNFYQQWQPSRKPGDMRLYANSSIGLFGAL
DHA-1QIJ17670.1_76KpBOP               KGITLLDLATYTAGGLPLQVPDAVKSRADLLNFYQQWQPSRKPGDMRLYANSSIGLFGAL
DHA-1QYI39301.1_102KpBOP              KGITLLDLATYTAGGLPLQVPDAVKSRADLLNFYQQWQPSRKPGDMRLYANSSIGLFGAL
DHA-1QER51199.1_205KpBOP              KGITLLDLATYTAGGLPLQVPDAVKSRADLLNFYQQWQPSRKPGDMRLYANSSIGLFGAL
DHA-1QIF88318.1_264KpBOP              KGITLLDLATYTAGGLPLQVPDAVKSRADLLNFYQQWQPSRKPGDMRLYANSSIGLFGAL
DHA-1QLI11710.1_48KpBOP               KGITLLDLATYTAGGLPLQVPDAVKSRADLLNFYQQWQPSRKPGDMRLYANSSIGLFGAL
DHA-1QLI17048.1_76KpBOP               KGITLLDLATYTAGGLPLQVPDAVKSRADLLNFYQQWQPSRKPGDMRLYANSSIGLFGAL
DHA-1QLI22544.1_1KpBOP                KGITLLDLATYTAGGLPLQVPDAVKSRADLLNFYQQWQPSRKPGDMRLYANSSIGLFGAL
DHA-1QLI33420.1_49KpBOP               KGITLLDLATYTAGGLPLQVPDAVKSRADLLNFYQQWQPSRKPGDMRLYANSSIGLFGAL
DHA-1ULI82297.1_104KpBOP              KGITLLDLATYTAGGLPLQVPDAVKSRADLLNFYQQWQPSRKPGDMRLYANSSIGLFGAL
DHA-1ULK26611.1_20KpBOP               KGITLLDLATYTAGGLPLQVPDAVKSRADLLNFYQQWQPSRKPGDMRLYANSSIGLFGAL
DHA-1ULI14884.1_221KpBOP              KGITLLDLATYTAGGLPLQVPDAVKSRADLLNFYQQWQPSRKPGDMRLYANSSIGLFGAL
DHA-1UMU88999.1_169KpBOP              KGITLLDLATYTAGGLPLQVPDAVKSRADLLNFYQQWQPSRKPGDMRLYANSSIGLFGAL
DHA-1QLI93287.1_12KpBOP               KGITLLDLATYTAGGLPLQVPDAVKSRADLLNFYQQWQPSRKPGDMRLYANSSIGLFGAL
DHA-1QOW59325.1_216KpBOP              KGITLLDLATYTAGGLPLQVPDAVKSRADLLNFYQQWQPSRKPGDMRLYANSSIGLFGAL
DHA-1QPP83812.1_78KpBOP               KGITLLDLATYTAGGLPLQVPDAVKSRADLLNFYQQWQPSRKPGDMRLYANSSIGLFGAL
DHA-1QPQ05032.1_31KpBOP               KGITLLDLATYTAGGLPLQVPDAVKSRADLLNFYQQWQPSRKPGDMRLYANSSIGLFGAL
DHA-1QQL36831.1_206KpBOP              KGITLLDLATYTAGGLPLQVPDAVKSRADLLNFYQQWQPSRKPGDMRLYANSSIGLFGAL
DHA-1QRR62282.1_23KpBOP               KGITLLDLATYTAGGLPLQVPDAVKSRADLLNFYQQWQPSRKPGDMRLYANSSIGLFGAL
DHA-1QRR62336.1_13KpBOP               KGITLLDLATYTAGGLPLQVPDAVKSRADLLNFYQQWQPSRKPGDMRLYANSSIGLFGAL
DHA-1QRR62351.1_34KpBOP               KGITLLDLATYTAGGLPLQVPDAVKSRADLLNFYQQWQPSRKPGDMRLYANSSIGLFGAL
DHA-1QRR62310.1_56KpBOP               KGITLLDLATYTAGGLPLQVPDAVKSRADLLNFYQQWQPSRKPGDMRLYANSSIGLFGAL
DHA-1QRS73253.1_51KpBOP               KGITLLDLATYTAGGLPLQVPDAVKSRADLLNFYQQWQPSRKPGDMRLYANSSIGLFGAL
DHA-1QSC32078.1_75KpBOP               KGITLLDLATYTAGGLPLQVPDAVKSRADLLNFYQQWQPSRKPGDMRLYANSSIGLFGAL
DHA-1URL54267.1_52KpBOP               KGITLLDLATYTAGGLPLQVPDAVKSRADLLNFYQQWQPSRKPGDMRLYANSSIGLFGAL
DHA-1QSW33401.1_150KpBOP              KGITLLDLATYTAGGLPLQVPDAVKSRADLLNFYQQWQPSRKPGDMRLYANSSIGLFGAL
DHA-1QTX39082.1_340KpBOP              KGITLLDLATYTAGGLPLQVPDAVKSRADLLNFYQQWQPSRKPGDMRLYANSSIGLFGAL
DHA-1QUX17533.1_117KpBOP              KGITLLDLATYTAGGLPLQVPDAVKSRADLLNFYQQWQPSRKPGDMRLYANSSIGLFGAL
DHA-1QVQ94431.1_179KpBOP              KGITLLDLATYTAGGLPLQVPDAVKSRADLLNFYQQWQPSRKPGDMRLYANSSIGLFGAL
DHA-1QVR16641.1_60KpBOP               KGITLLDLATYTAGGLPLQVPDAVKSRADLLNFYQQWQPSRKPGDMRLYANSSIGLFGAL
DHA-1QVU34134.1_265KpBOP              KGITLLDLATYTAGGLPLQVPDAVKSRADLLNFYQQWQPSRKPGDMRLYANSSIGLFGAL
DHA-1QVW15542.1_136KpBOP              KGITLLDLATYTAGGLPLQVPDAVKSRADLLNFYQQWQPSRKPGDMRLYANSSIGLFGAL
DHA-1QVW15569.1_171KpBOP              KGITLLDLATYTAGGLPLQVPDAVKSRADLLNFYQQWQPSRKPGDMRLYANSSIGLFGAL
DHA-1QXQ84174.1_24KpBOP               KGITLLDLATYTAGGLPLQVPDAVKSRADLLNFYQQWQPSRKPGDMRLYANSSIGLFGAL
DHA-1QXT85906.1_24KpBOP               KGITLLDLATYTAGGLPLQVPDAVKSRADLLNFYQQWQPSRKPGDMRLYANSSIGLFGAL
DHA-1QXU18235.1_24KpBOP               KGITLLDLATYTAGGLPLQVPDAVKSRADLLNFYQQWQPSRKPGDMRLYANSSIGLFGAL
DHA-1UNH49680.1_195KpBOP              KGITLLDLATYTAGGLPLQVPDAVKSRADLLNFYQQWQPSRKPGDMRLYANSSIGLFGAL
DHA-1UGM94295.1_45KpBOP               KGITLLDLATYTAGGLPLQVPDAVKSRADLLNFYQQWQPSRKPGDMRLYANSSIGLFGAL
DHA-1UCM37138.1_188KpBOP              KGITLLDLATYTAGGLPLQVPDAVKSRADLLNFYQQWQPSRKPGDMRLYANSSIGLFGAL
DHA-1UPF47572.1_185KpBOP              KGITLLDLATYTAGGLPLQVPDAVKSRADLLNFYQQWQPSRKPGDMRLYANSSIGLFGAL
DHA-1UPF53088.1_98KpBOP               KGITLLDLATYTAGGLPLQVPDAVKSRADLLNFYQQWQPSRKPGDMRLYANSSIGLFGAL
DHA-1UNE95136.1_177KpBOP              KGITLLDLATYTAGGLPLQVPDAVKSRADLLNFYQQWQPSRKPGDMRLYANSSIGLFGAL
DHA-1UGQ70597.1_19KpBOP               KGITLLDLATYTAGGLPLQVPDAVKSRADLLNFYQQWQPSRKPGDMRLYANSSIGLFGAL
DHA-1UGQ75588.1_15KpBOP               KGITLLDLATYTAGGLPLQVPDAVKSRADLLNFYQQWQPSRKPGDMRLYANSSIGLFGAL
DHA-1UGQ80838.1_30KpBOP               KGITLLDLATYTAGGLPLQVPDAVKSRADLLNFYQQWQPSRKPGDMRLYANSSIGLFGAL
DHA-1UGR08221.1_34KpBOP               KGITLLDLATYTAGGLPLQVPDAVKSRADLLNFYQQWQPSRKPGDMRLYANSSIGLFGAL
DHA-1UGR13826.1_19KpBOP               KGITLLDLATYTAGGLPLQVPDAVKSRADLLNFYQQWQPSRKPGDMRLYANSSIGLFGAL
DHA-1UGS19283.1_181KpBOP              KGITLLDLATYTAGGLPLQVPDAVKSRADLLNFYQQWQPSRKPGDMRLYANSSIGLFGAL
DHA-1UII40113.1_155KpBOP              KGITLLDLATYTAGGLPLQVPDAVKSRADLLNFYQQWQPSRKPGDMRLYANSSIGLFGAL
DHA-1UIY63185.1_63KpBOP               KGITLLDLATYTAGGLPLQVPDAVKSRADLLNFYQQWQPSRKPGDMRLYANSSIGLFGAL
DHA-1UZK63620.1_210KpBOP              KGITLLDLATYTAGGLPLQVPDAVKSRADLLNFYQQWQPSRKPGDMRLYANSSIGLFGAL
DHA-1UPS70201.1_18KpBOP               KGITLLDLATYTAGGLPLQVPDAVKSRADLLNFYQQWQPSRKPGDMRLYANSSIGLFGAL
DHA-1WDO24504.1_185KpBOP              KGITLLDLATYTAGGLPLQVPDAVKSRADLLNFYQQWQPSRKPGDMRLYANSSIGLFGAL
DHA-1URT78846.1_121KpBOP              KGITLLDLATYTAGGLPLQVPDAVKSRADLLNFYQQWQPSRKPGDMRLYANSSIGLFGAL
DHA-1URT89155.1_166KpBOP              KGITLLDLATYTAGGLPLQVPDAVKSRADLLNFYQQWQPSRKPGDMRLYANSSIGLFGAL
DHA-1URT89430.1_166KpBOP              KGITLLDLATYTAGGLPLQVPDAVKSRADLLNFYQQWQPSRKPGDMRLYANSSIGLFGAL
DHA-1URT94913.1_167KpBOP              KGITLLDLATYTAGGLPLQVPDAVKSRADLLNFYQQWQPSRKPGDMRLYANSSIGLFGAL
DHA-1UWZ32448.1_52KpBOP               KGITLLDLATYTAGGLPLQVPDAVKSRADLLNFYQQWQPSRKPGDMRLYANSSIGLFGAL
DHA-1UTW92771.1_126KpBOP              KGITLLDLATYTAGGLPLQVPDAVKSRADLLNFYQQWQPSRKPGDMRLYANSSIGLFGAL
DHA-1UZW73111.1_23KpBOP               KGITLLDLATYTAGGLPLQVPDAVKSRADLLNFYQQWQPSRKPGDMRLYANSSIGLFGAL
DHA-1WEI85275.1_173KpBOP              KGITLLDLATYTAGGLPLQVPDAVKSRADLLNFYQQWQPSRKPGDMRLYANSSIGLFGAL
DHA-1UVF97054.1_214KpBOP              KGITLLDLATYTAGGLPLQVPDAVKSRADLLNFYQQWQPSRKPGDMRLYANSSIGLFGAL
DHA-1UVN34295.1_69KpBOP               KGITLLDLATYTAGGLPLQVPDAVKSRADLLNFYQQWQPSRKPGDMRLYANSSIGLFGAL
DHA-1UWB42605.1_210KpBOP              KGITLLDLATYTAGGLPLQVPDAVKSRADLLNFYQQWQPSRKPGDMRLYANSSIGLFGAL
DHA-1UWX53529.1_18KpBOP               KGITLLDLATYTAGGLPLQVPDAVKSRADLLNFYQQWQPSRKPGDMRLYANSSIGLFGAL
DHA-1WII50269.1_174KpBOP              KGITLLDLATYTAGGLPLQVPDAVKSRADLLNFYQQWQPSRKPGDMRLYANSSIGLFGAL
DHA-1WII50721.1_91KpBOP               KGITLLDLATYTAGGLPLQVPDAVKSRADLLNFYQQWQPSRKPGDMRLYANSSIGLFGAL
DHA-1UXY66557.1_128KpBOP              KGITLLDLATYTAGGLPLQVPDAVKSRADLLNFYQQWQPSRKPGDMRLYANSSIGLFGAL
DHA-1UYX39647.1_199KpBOP              KGITLLDLATYTAGGLPLQVPDAVKSRADLLNFYQQWQPSRKPGDMRLYANSSIGLFGAL
DHA-1UZE57593.1_95KpBOP               KGITLLDLATYTAGGLPLQVPDAVKSRADLLNFYQQWQPSRKPGDMRLYANSSIGLFGAL
DHA-1WAD38926.1_6KpBOP                KGITLLDLATYTAGGLPLQVPDAVKSRADLLNFYQQWQPSRKPGDMRLYANSSIGLFGAL
DHA-1WAD13598.1_210KpBOP              KGITLLDLATYTAGGLPLQVPDAVKSRADLLNFYQQWQPSRKPGDMRLYANSSIGLFGAL
DHA-1WAD08359.1_184KpBOP              KGITLLDLATYTAGGLPLQVPDAVKSRADLLNFYQQWQPSRKPGDMRLYANSSIGLFGAL
DHA-1WAD18885.1_185KpBOP              KGITLLDLATYTAGGLPLQVPDAVKSRADLLNFYQQWQPSRKPGDMRLYANSSIGLFGAL
DHA-1WAD19356.1_183KpBOP              KGITLLDLATYTAGGLPLQVPDAVKSRADLLNFYQQWQPSRKPGDMRLYANSSIGLFGAL
DHA-1WAD44078.1_264KpBOP              KGITLLDLATYTAGGLPLQVPDAVKSRADLLNFYQQWQPSRKPGDMRLYANSSIGLFGAL
DHA-1WAD49440.1_195KpBOP              KGITLLDLATYTAGGLPLQVPDAVKSRADLLNFYQQWQPSRKPGDMRLYANSSIGLFGAL
DHA-1WAD71205.1_194KpBOP              KGITLLDLATYTAGGLPLQVPDAVKSRADLLNFYQQWQPSRKPGDMRLYANSSIGLFGAL
DHA-1WAD55056.1_244KpBOP              KGITLLDLATYTAGGLPLQVPDAVKSRADLLNFYQQWQPSRKPGDMRLYANSSIGLFGAL
DHA-1WAD60732.1_211KpBOP              KGITLLDLATYTAGGLPLQVPDAVKSRADLLNFYQQWQPSRKPGDMRLYANSSIGLFGAL
DHA-1WAD61281.1_278KpBOP              KGITLLDLATYTAGGLPLQVPDAVKSRADLLNFYQQWQPSRKPGDMRLYANSSIGLFGAL
DHA-1WFF21460.1_31KpBOP               KGITLLDLATYTAGGLPLQVPDAVKSRADLLNFYQQWQPSRKPGDMRLYANSSIGLFGAL
DHA-1WHL16843.1_345KpBOP              KGITLLDLATYTAGGLPLQVPDAVKSRADLLNFYQQWQPSRKPGDMRLYANSSIGLFGAL
DHA-1WP_004236386.1KpOC               KGITLLDLATYTAGGLPLQVPDAVKSRADLLNFYQQWQPSRKPGDMRLYANSSIGLFGAL
DHA-1WP_004236386.1_4280KpBCP         KGITLLDLATYTAGGLPLQVPDAVKSRADLLNFYQQWQPSRKPGDMRLYANSSIGLFGAL
DHA-1WP_004236386.1_5142KpBCP         KGITLLDLATYTAGGLPLQVPDAVKSRADLLNFYQQWQPSRKPGDMRLYANSSIGLFGAL
DHA-1WP_004236386.1_814KpBCP          KGITLLDLATYTAGGLPLQVPDAVKSRADLLNFYQQWQPSRKPGDMRLYANSSIGLFGAL
DHA-1WP_004236386.1_821KpBCP          KGITLLDLATYTAGGLPLQVPDAVKSRADLLNFYQQWQPSRKPGDMRLYANSSIGLFGAL
DHA-1WP_004236386.1_817KpBCP          KGITLLDLATYTAGGLPLQVPDAVKSRADLLNFYQQWQPSRKPGDMRLYANSSIGLFGAL
DHA-1WP_004236386.1_700KpBCP          KGITLLDLATYTAGGLPLQVPDAVKSRADLLNFYQQWQPSRKPGDMRLYANSSIGLFGAL
DHA-1USO45218.1_1502KpBCP             KGITLLDLATYTAGGLPLQVPDAVKSRADLLNFYQQWQPSRKPGDMRLYANSSIGLFGAL
DHA-1WP_004236386.1_3654KpBCP         KGITLLDLATYTAGGLPLQVPDAVKSRADLLNFYQQWQPSRKPGDMRLYANSSIGLFGAL
DHA-1WP_004236386.1_5030KpBCP         KGITLLDLATYTAGGLPLQVPDAVKSRADLLNFYQQWQPSRKPGDMRLYANSSIGLFGAL
DHA-7AM390_27335_79KpBOP              KGITLLDLATYTAGGLPLQVPDAVKSRADLLNFYQQWQPSRKPGDMRLYANSSIGLFGAL
DHA-7AM395_28070_58KpBOP              KGITLLDLATYTAGGLPLQVPDAVKSRADLLNFYQQWQPSRKPGDMRLYANSSIGLFGAL
DHA-7AN663_28510_174KpBOP             KGITLLDLATYTAGGLPLQVPDAVKSRADLLNFYQQWQPSRKPGDMRLYANSSIGLFGAL
                                 acc
```

```
                          η4      α6                      η5      β6  β7       α7
5GGW_1|Chains                 170       180       190       200       210       220

5GGW_1|Chains                         AVKPSGLSFEQAMQTRVFQPLKLNHTWINVPPPEEKNYAWGYREGKAVHVSPGALDAEAY
DHA-15WGF99578.1_254KpBOP             TANAAGMPYEQLLTARILAPLGLSHTFITVPESAQSQYAYGYKNKKPVRVSPGQLDAESY
DHA-1AVZ12228.1_302EBOC               TANAAGMPYEQLLTARILAPLGLSHTFITVPESAQSQYAYGYKNKKPVRVSPGQLDAESY
DHA-1QGU39370.1_323EBOC               TANAAGMPYEQLLTARILAPLGLSHTFITVPESAQSQYAYGYKNKKPVRVSPGQLDAESY
DHA-1QIE67107.1_279EBOC               TANAAGMPYEQLLTARILAPLGLSHTFITVPESAQSQYAYGYKNKKPVRVSPGQLDAESY
DHA-1QIF29352.1_349EBOC               TANAAGMPYEQLLTARILAPLGLSHTFITVPESAQSQYAYGYKNKKPVRVSPGQLDAESY
DHA-1QUP93355.1_50EBOC                TANAAGMPYEQLLTARILAPLGLSHTFITVPESAQSQYAYGYKNKKPVRVSPGQLDAESY
DHA-1UKB51966.1_271EBOC               TANAAGMPYEQLLTARILAPLGLSHTFITVPESAQSQYAYGYKNKKPVRVSPGQLDAESY
DHA-1UKB62076.1_255EBOC               TANAAGMPYEQLLTARILAPLGLSHTFITVPESAQSQYAYGYKNKKPVRVSPGQLDAESY
DHA-1UOY35142.1_6EBOC                 TANAAGMPYEQLLTARILAPLGLSHTFITVPESAQSQYAYGYKNKKPVRVSPGQLDAESY
DHA-1UOY53746.1_188EBOC               TANAAGMPYEQLLTARILAPLGLSHTFITVPESAQSQYAYGYKNKKPVRVSPGQLDAESY
DHA-1URL72031.1_311EBOC               TANAAGMPYEQLLTARILAPLGLSHTFITVPESAQSQYAYGYKNKKPVRVSPGQLDAESY
DHA-1WAZ65759.1_143EBOC               TANAAGMPYEQLLTARILAPLGLSHTFITVPESAQSQYAYGYKNKKPVRVSPGQLDAESY
DHA-1WGL89115.1_5EBOC                 TANAAGMPYEQLLTARILAPLGLSHTFITVPESAQSQYAYGYKNKKPVRVSPGQLDAESY
DHA-1WGZ52333.1_354EBOC               TANAAGMPYEQLLTARILAPLGLSHTFITVPESAQSQYAYGYKNKKPVRVSPGQLDAESY
DHA-1ULK46985.1_701KpBOC              TANAAGMPYEQLLTARILAPLGLSHTFITVPESAQSQYAYGYKNKKPVRVSPGQLDAESY
DHA-1QLR69540.1_4269KpBOC             TANAAGMPYEQLLTARILAPLGLSHTFITVPESAQSQYAYGYKNKKPVRVSPGQLDAESY
DHA-1UMU29130.1_701KpBOC              TANAAGMPYEQLLTARILAPLGLSHTFITVPESAQSQYAYGYKNKKPVRVSPGQLDAESY
DHA-1QTO12878.1_5112KpBOC             TANAAGMPYEQLLTARILAPLGLSHTFITVPESAQSQYAYGYKNKKPVRVSPGQLDAESY
DHA-1QVR56117.1_814KpBOC              TANAAGMPYEQLLTARILAPLGLSHTFITVPESAQSQYAYGYKNKKPVRVSPGQLDAESY
DHA-1WBX47547.1_5024KpBOC             TANAAGMPYEQLLTARILAPLGLSHTFITVPESAQSQYAYGYKNKKPVRVSPGQLDAESY
DHA-1USO45218.1_1502KpBOC             TANAAGMPYEQLLTARILAPLGLSHTFITVPESAQSQYAYGYKNKKPVRVSPGQLDAESY
DHA-1UYH26771.1_3649KpBOC             TANAAGMPYEQLLTARILAPLGLSHTFITVPESAQSQYAYGYKNKKPVRVSPGQLDAESY
DHA-1BCI98564.1_KP_B_O_P              TANAAGMPYEQLLTARILAPLGLSHTFITVPESAQSQYAYGYKNKKPVRVSPGQLDAESY
DHA-1BBR80752.1_1091]KpBOP            TANAAGMPYEQLLTARILAPLGLSHTFITVPESAQSQYAYGYKNKKPVRVSPGQLDAESY
DHA-1ARV42956.1_78KpBOP               TANAAGMPYEQLLTARILAPLGLSHTFITVPESAQSQYAYGYKNKKPVRVSPGQLDAESY
DHA-1QAW92788.1_234KpBOP              TANAAGMPYEQLLTARILAPLGLSHTFITVPESAQSQYAYGYKNKKPVRVSPGQLDAESY
DHA-1AWE04508.1_77KpBOP               TANAAGMPYEQLLTARILAPLGLSHTFITVPESAQSQYAYGYKNKKPVRVSPGQLDAESY
DHA-1AWE05132.1_156KpBOP              TANAAGMPYEQLLTARILAPLGLSHTFITVPESAQSQYAYGYKNKKPVRVSPGQLDAESY
DHA-1AXR54232.1_175KpBOP              TANAAGMPYEQLLTARILAPLGLSHTFITVPESAQSQYAYGYKNKKPVRVSPGQLDAESY
DHA-1QBA36383.1_199KpBOP              TANAAGMPYEQLLTARILAPLGLSHTFITVPESAQSQYAYGYKNKKPVRVSPGQLDAESY
DHA-1QIQ40896.1_277KpBOP              TANAAGMPYEQLLTARILAPLGLSHTFITVPESAQSQYAYGYKNKKPVRVSPGQLDAESY
DHA-1QLB73441.1_45KpBOP               TANAAGMPYEQLLTARILAPLGLSHTFITVPESAQSQYAYGYKNKKPVRVSPGQLDAESY
DHA-1QLB78779.1_63KpBOP               TANAAGMPYEQLLTARILAPLGLSHTFITVPESAQSQYAYGYKNKKPVRVSPGQLDAESY
DHA-1QLB84106.1_44KpBOP               TANAAGMPYEQLLTARILAPLGLSHTFITVPESAQSQYAYGYKNKKPVRVSPGQLDAESY
DHA-1QLB89444.1_44KpBOP               TANAAGMPYEQLLTARILAPLGLSHTFITVPESAQSQYAYGYKNKKPVRVSPGQLDAESY
DHA-1QLB94784.1_45KpBOP               TANAAGMPYEQLLTARILAPLGLSHTFITVPESAQSQYAYGYKNKKPVRVSPGQLDAESY
DHA-1QLC10688.1_45KpBOP               TANAAGMPYEQLLTARILAPLGLSHTFITVPESAQSQYAYGYKNKKPVRVSPGQLDAESY
DHA-1QLC16028.1_45KpBOP               TANAAGMPYEQLLTARILAPLGLSHTFITVPESAQSQYAYGYKNKKPVRVSPGQLDAESY
DHA-1QIJ38370.1_76KpBOP               TANAAGMPYEQLLTARILAPLGLSHTFITVPESAQSQYAYGYKNKKPVRVSPGQLDAESY
DHA-1QIJ23273.1_76KpBOP               TANAAGMPYEQLLTARILAPLGLSHTFITVPESAQSQYAYGYKNKKPVRVSPGQLDAESY
DHA-1QIJ17670.1_76KpBOP               TANAAGMPYEQLLTARILAPLGLSHTFITVPESAQSQYAYGYKNKKPVRVSPGQLDAESY
DHA-1QYI39301.1_102KpBOP              TANAAGMPYEQLLTARILAPLGLSHTFITVPESAQSQYAYGYKNKKPVRVSPGQLDAESY
DHA-1QER51199.1_205KpBOP              TANAAGMPYEQLLTARILAPLGLSHTFITVPESAQSQYAYGYKNKKPVRVSPGQLDAESY
DHA-1QIF88318.1_264KpBOP              TANAAGMPYEQLLTARILAPLGLSHTFITVPESAQSQYAYGYKNKKPVRVSPGQLDAESY
DHA-1QLI11710.1_48KpBOP               TANAAGMPYEQLLTARILAPLGLSHTFITVPESAQSQYAYGYKNKKPVRVSPGQLDAESY
DHA-1QLI17048.1_76KpBOP               TANAAGMPYEQLLTARILAPLGLSHTFITVPESAQSQYAYGYKNKKPVRVSPGQLDAESY
DHA-1QLI22544.1_1KpBOP                TANAAGMPYEQLLTARILAPLGLSHTFITVPESAQSQYAYGYKNKKPVRVSPGQLDAESY
DHA-1QLI33420.1_49KpBOP               TANAAGMPYEQLLTARILAPLGLSHTFITVPESAQSQYAYGYKNKKPVRVSPGQLDAESY
DHA-1ULI82297.1_104KpBOP              TANAAGMPYEQLLTARILAPLGLSHTFITVPESAQSQYAYGYKNKKPVRVSPGQLDAESY
DHA-1ULK26611.1_20KpBOP               TANAAGMPYEQLLTARILAPLGLSHTFITVPESAQSQYAYGYKNKKPVRVSPGQLDAESY
DHA-1ULI14884.1_221KpBOP              TANAAGMPYEQLLTARILAPLGLSHTFITVPESAQSQYAYGYKNKKPVRVSPGQLDAESY
DHA-1UMU88999.1_169KpBOP              TANAAGMPYEQLLTARILAPLGLSHTFITVPESAQSQYAYGYKNKKPVRVSPGQLDAESY
DHA-1QLI93287.1_12KpBOP               TANAAGMPYEQLLTARILAPLGLSHTFITVPESAQSQYAYGYKNKKPVRVSPGQLDAESY
DHA-1QOW59325.1_216KpBOP              TANAAGMPYEQLLTARILAPLGLSHTFITVPESAQSQYAYGYKNKKPVRVSPGQLDAESY
DHA-1QPP83812.1_78KpBOP               TANAAGMPYEQLLTARILAPLGLSHTFITVPESAQSQYAYGYKNKKPVRVSPGQLDAESY
DHA-1QPQ05032.1_31KpBOP               TANAAGMPYEQLLTARILAPLGLSHTFITVPESAQSQYAYGYKNKKPVRVSPGQLDAESY
DHA-1QQL36831.1_206KpBOP              TANAAGMPYEQLLTARILAPLGLSHTFITVPESAQSQYAYGYKNKKPVRVSPGQLDAESY
DHA-1QRR62282.1_23KpBOP               TANAAGMPYEQLLTARILAPLGLSHTFITVPESAQSQYAYGYKNKKPVRVSPGQLDAESY
DHA-1QRR62336.1_13KpBOP               TANAAGMPYEQLLTARILAPLGLSHTFITVPESAQSQYAYGYKNKKPVRVSPGQLDAESY
DHA-1QRR62351.1_34KpBOP               TANAAGMPYEQLLTARILAPLGLSHTFITVPESAQSQYAYGYKNKKPVRVSPGQLDAESY
DHA-1QRR62310.1_56KpBOP               TANAAGMPYEQLLTARILAPLGLSHTFITVPESAQSQYAYGYKNKKPVRVSPGQLDAESY
DHA-1QRS73253.1_51KpBOP               TANAAGMPYEQLLTARILAPLGLSHTFITVPESAQSQYAYGYKNKKPVRVSPGQLDAESY
DHA-1QSC32078.1_75KpBOP               TANAAGMPYEQLLTARILAPLGLSHTFITVPESAQSQYAYGYKNKKPVRVSPGQLDAESY
DHA-1URL54267.1_52KpBOP               TANAAGMPYEQLLTARILAPLGLSHTFITVPESAQSQYAYGYKNKKPVRVSPGQLDAESY
DHA-1QSW33401.1_150KpBOP              TANAAGMPYEQLLTARILAPLGLSHTFITVPESAQSQYAYGYKNKKPVRVSPGQLDAESY
DHA-1QTX39082.1_340KpBOP              TANAAGMPYEQLLTARILAPLGLSHTFITVPESAQSQYAYGYKNKKPVRVSPGQLDAESY
DHA-1QUX17533.1_117KpBOP              TANAAGMPYEQLLTARILAPLGLSHTFITVPESAQSQYAYGYKNKKPVRVSPGQLDAESY
DHA-1QVQ94431.1_179KpBOP              TANAAGMPYEQLLTARILAPLGLSHTFITVPESAQSQYAYGYKNKKPVRVSPGQLDAESY
DHA-1QVR16641.1_60KpBOP               TANAAGMPYEQLLTARILAPLGLSHTFITVPESAQSQYAYGYKNKKPVRVSPGQLDAESY
DHA-1QVU34134.1_265KpBOP              TANAAGMPYEQLLTARILAPLGLSHTFITVPESAQSQYAYGYKNKKPVRVSPGQLDAESY
DHA-1QVW15542.1_136KpBOP              TANAAGMPYEQLLTARILAPLGLSHTFITVPESAQSQYAYGYKNKKPVRVSPGQLDAESY
DHA-1QVW15569.1_171KpBOP              TANAAGMPYEQLLTARILAPLGLSHTFITVPESAQSQYAYGYKNKKPVRVSPGQLDAESY
DHA-1QXQ84174.1_24KpBOP               TANAAGMPYEQLLTARILAPLGLSHTFITVPESAQSQYAYGYKNKKPVRVSPGQLDAESY
DHA-1QXT85906.1_24KpBOP               TANAAGMPYEQLLTARILAPLGLSHTFITVPESAQSQYAYGYKNKKPVRVSPGQLDAESY
DHA-1QXU18235.1_24KpBOP               TANAAGMPYEQLLTARILAPLGLSHTFITVPESAQSQYAYGYKNKKPVRVSPGQLDAESY
DHA-1UNH49680.1_195KpBOP              TANAAGMPYEQLLTARILAPLGLSHTFITVPESAQSQYAYGYKNKKPVRVSPGQLDAESY
DHA-1UGM94295.1_45KpBOP               TANAAGMPYEQLLTARILAPLGLSHTFITVPESAQSQYAYGYKNKKPVRVSPGQLDAESY
DHA-1UCM37138.1_188KpBOP              TANAAGMPYEQLLTARILAPLGLSHTFITVPESAQSQYAYGYKNKKPVRVSPGQLDAESY
DHA-1UPF47572.1_185KpBOP              TANAAGMPYEQLLTARILAPLGLSHTFITVPESAQSQYAYGYKNKKPVRVSPGQLDAESY
DHA-1UPF53088.1_98KpBOP               TANAAGMPYEQLLTARILAPLGLSHTFITVPESAQSQYAYGYKNKKPVRVSPGQLDAESY
DHA-1UNE95136.1_177KpBOP              TANAAGMPYEQLLTARILAPLGLSHTFITVPESAQSQYAYGYKNKKPVRVSPGQLDAESY
DHA-1UGQ70597.1_19KpBOP               TANAAGMPYEQLLTARILAPLGLSHTFITVPESAQSQYAYGYKNKKPVRVSPGQLDAESY
DHA-1UGQ75588.1_15KpBOP               TANAAGMPYEQLLTARILAPLGLSHTFITVPESAQSQYAYGYKNKKPVRVSPGQLDAESY
DHA-1UGQ80838.1_30KpBOP               TANAAGMPYEQLLTARILAPLGLSHTFITVPESAQSQYAYGYKNKKPVRVSPGQLDAESY
DHA-1UGR08221.1_34KpBOP               TANAAGMPYEQLLTARILAPLGLSHTFITVPESAQSQYAYGYKNKKPVRVSPGQLDAESY
DHA-1UGR13826.1_19KpBOP               TANAAGMPYEQLLTARILAPLGLSHTFITVPESAQSQYAYGYKNKKPVRVSPGQLDAESY
DHA-1UGS19283.1_181KpBOP              TANAAGMPYEQLLTARILAPLGLSHTFITVPESAQSQYAYGYKNKKPVRVSPGQLDAESY
DHA-1UII40113.1_155KpBOP              TANAAGMPYEQLLTARILAPLGLSHTFITVPESAQSQYAYGYKNKKPVRVSPGQLDAESY
DHA-1UIY63185.1_63KpBOP               TANAAGMPYEQLLTARILAPLGLSHTFITVPESAQSQYAYGYKNKKPVRVSPGQLDAESY
DHA-1UZK63620.1_210KpBOP              TANAAGMPYEQLLTARILAPLGLSHTFITVPESAQSQYAYGYKNKKPVRVSPGQLDAESY
DHA-1UPS70201.1_18KpBOP               TANAAGMPYEQLLTARILAPLGLSHTFITVPESAQSQYAYGYKNKKPVRVSPGQLDAESY
DHA-1WDO24504.1_185KpBOP              TANAAGMPYEQLLTARILAPLGLSHTFITVPESAQSQYAYGYKNKKPVRVSPGQLDAESY
DHA-1URT78846.1_121KpBOP              TANAAGMPYEQLLTARILAPLGLSHTFITVPESAQSQYAYGYKNKKPVRVSPGQLDAESY
DHA-1URT89155.1_166KpBOP              TANAAGMPYEQLLTARILAPLGLSHTFITVPESAQSQYAYGYKNKKPVRVSPGQLDAESY
DHA-1URT89430.1_166KpBOP              TANAAGMPYEQLLTARILAPLGLSHTFITVPESAQSQYAYGYKNKKPVRVSPGQLDAESY
DHA-1URT94913.1_167KpBOP              TANAAGMPYEQLLTARILAPLGLSHTFITVPESAQSQYAYGYKNKKPVRVSPGQLDAESY
DHA-1UWZ32448.1_52KpBOP               TANAAGMPYEQLLTARILAPLGLSHTFITVPESAQSQYAYGYKNKKPVRVSPGQLDAESY
DHA-1UTW92771.1_126KpBOP              TANAAGMPYEQLLTARILAPLGLSHTFITVPESAQSQYAYGYKNKKPVRVSPGQLDAESY
DHA-1UZW73111.1_23KpBOP               TANAAGMPYEQLLTARILAPLGLSHTFITVPESAQSQYAYGYKNKKPVRVSPGQLDAESY
DHA-1WEI85275.1_173KpBOP              TANAAGMPYEQLLTARILAPLGLSHTFITVPESAQSQYAYGYKNKKPVRVSPGQLDAESY
DHA-1UVF97054.1_214KpBOP              TANAAGMPYEQLLTARILAPLGLSHTFITVPESAQSQYAYGYKNKKPVRVSPGQLDAESY
DHA-1UVN34295.1_69KpBOP               TANAAGMPYEQLLTARILAPLGLSHTFITVPESAQSQYAYGYKNKKPVRVSPGQLDAESY
DHA-1UWB42605.1_210KpBOP              TANAAGMPYEQLLTARILAPLGLSHTFITVPESAQSQYAYGYKNKKPVRVSPGQLDAESY
DHA-1UWX53529.1_18KpBOP               TANAAGMPYEQLLTARILAPLGLSHTFITVPESAQSQYAYGYKNKKPVRVSPGQLDAESY
DHA-1WII50269.1_174KpBOP              TANAAGMPYEQLLTARILAPLGLSHTFITVPESAQSQYAYGYKNKKPVRVSPGQLDAESY
DHA-1WII50721.1_91KpBOP               TANAAGMPYEQLLTARILAPLGLSHTFITVPESAQSQYAYGYKNKKPVRVSPGQLDAESY
DHA-1UXY66557.1_128KpBOP              TANAAGMPYEQLLTARILAPLGLSHTFITVPESAQSQYAYGYKNKKPVRVSPGQLDAESY
DHA-1UYX39647.1_199KpBOP              TANAAGMPYEQLLTARILAPLGLSHTFITVPESAQSQYAYGYKNKKPVRVSPGQLDAESY
DHA-1UZE57593.1_95KpBOP               TANAAGMPYEQLLTARILAPLGLSHTFITVPESAQSQYAYGYKNKKPVRVSPGQLDAESY
DHA-1WAD38926.1_6KpBOP                TANAAGMPYEQLLTARILAPLGLSHTFITVPESAQSQYAYGYKNKKPVRVSPGQLDAESY
DHA-1WAD13598.1_210KpBOP              TANAAGMPYEQLLTARILAPLGLSHTFITVPESAQSQYAYGYKNKKPVRVSPGQLDAESY
DHA-1WAD08359.1_184KpBOP              TANAAGMPYEQLLTARILAPLGLSHTFITVPESAQSQYAYGYKNKKPVRVSPGQLDAESY
DHA-1WAD18885.1_185KpBOP              TANAAGMPYEQLLTARILAPLGLSHTFITVPESAQSQYAYGYKNKKPVRVSPGQLDAESY
DHA-1WAD19356.1_183KpBOP              TANAAGMPYEQLLTARILAPLGLSHTFITVPESAQSQYAYGYKNKKPVRVSPGQLDAESY
DHA-1WAD44078.1_264KpBOP              TANAAGMPYEQLLTARILAPLGLSHTFITVPESAQSQYAYGYKNKKPVRVSPGQLDAESY
DHA-1WAD49440.1_195KpBOP              TANAAGMPYEQLLTARILAPLGLSHTFITVPESAQSQYAYGYKNKKPVRVSPGQLDAESY
DHA-1WAD71205.1_194KpBOP              TANAAGMPYEQLLTARILAPLGLSHTFITVPESAQSQYAYGYKNKKPVRVSPGQLDAESY
DHA-1WAD55056.1_244KpBOP              TANAAGMPYEQLLTARILAPLGLSHTFITVPESAQSQYAYGYKNKKPVRVSPGQLDAESY
DHA-1WAD60732.1_211KpBOP              TANAAGMPYEQLLTARILAPLGLSHTFITVPESAQSQYAYGYKNKKPVRVSPGQLDAESY
DHA-1WAD61281.1_278KpBOP              TANAAGMPYEQLLTARILAPLGLSHTFITVPESAQSQYAYGYKNKKPVRVSPGQLDAESY
DHA-1WFF21460.1_31KpBOP               TANAAGMPYEQLLTARILAPLGLSHTFITVPESAQSQYAYGYKNKKPVRVSPGQLDAESY
DHA-1WHL16843.1_345KpBOP              TANAAGMPYEQLLTARILAPLGLSHTFITVPESAQSQYAYGYKNKKPVRVSPGQLDAESY
DHA-1WP_004236386.1KpOC               TANAAGMPYEQLLTARILAPLGLSHTFITVPESAQSQYAYGYKNKKPVRVSPGQLDAESY
DHA-1WP_004236386.1_4280KpBCP         TANAAGMPYEQLLTARILAPLGLSHTFITVPESAQSQYAYGYKNKKPVRVSPGQLDAESY
DHA-1WP_004236386.1_5142KpBCP         TANAAGMPYEQLLTARILAPLGLSHTFITVPESAQSQYAYGYKNKKPVRVSPGQLDAESY
DHA-1WP_004236386.1_814KpBCP          TANAAGMPYEQLLTARILAPLGLSHTFITVPESAQSQYAYGYKNKKPVRVSPGQLDAESY
DHA-1WP_004236386.1_821KpBCP          TANAAGMPYEQLLTARILAPLGLSHTFITVPESAQSQYAYGYKNKKPVRVSPGQLDAESY
DHA-1WP_004236386.1_817KpBCP          TANAAGMPYEQLLTARILAPLGLSHTFITVPESAQSQYAYGYKNKKPVRVSPGQLDAESY
DHA-1WP_004236386.1_700KpBCP          TANAAGMPYEQLLTARILAPLGLSHTFITVPESAQSQYAYGYKNKKPVRVSPGQLDAESY
DHA-1USO45218.1_1502KpBCP             TANAAGMPYEQLLTARILAPLGLSHTFITVPESAQSQYAYGYKNKKPVRVSPGQLDAESY
DHA-1WP_004236386.1_3654KpBCP         TANAAGMPYEQLLTARILAPLGLSHTFITVPESAQSQYAYGYKNKKPVRVSPGQLDAESY
DHA-1WP_004236386.1_5030KpBCP         TANAAGMPYEQLLTARILAPLGLSHTFITVPESAQSQYAYGYKNKKPVRVSPGQLDAESY
DHA-7AM390_27335_79KpBOP              TANAAGMPYEQLLTARILAPLGLSHTFITVPESAQSQYAYGYKNKKPVRVSPGQLDAESY
DHA-7AM395_28070_58KpBOP              TANAAGMPYEQLLTARILAPLGLSHTFITVPESAQSQYAYGYKNKKPVRVSPGQLDAESY
DHA-7AN663_28510_174KpBOP             TANAAGMPYEQLLTARILAPLGLSHTFITVPESAQSQYAYGYKNKKPVRVSPGQLDAESY
                                acc
```

```
                                   β8      α8          η6       α9           β9   β10      β11
5GGW_1|Chains                          230       240       250       260       270       280
5GGW_1|Chains                      GVKSTIEDMARWVRSNMNPRDINDKTLQQGIQLAQSRYWQTGDMYQGLGWEMLDWPVNPD
DHA-15WGF99578.1_254KpBOP          GVKSASKDMLRWAEMNMEPSRAGNADLEMAMYLAQTRYYKTAAINQGLGWEMYDWPQQKD
DHA-1AVZ12228.1_302EBOC            GVKSASKDMLRWAEMNMEPSRAGNADLEMAMYLAQTRYYKTAAINQGLGWEMYDWPQQKD
DHA-1QGU39370.1_323EBOC            GVKSASKDMLRWAEMNMEPSRAGNADLEMAMYLAQTRYYKTAAINQGLGWEMYDWPQQKD
DHA-1QIE67107.1_279EBOC            GVKSASKDMLRWAEMNMEPSRAGNADLEMAMYLAQTRYYKTAAINQGLGWEMYDWPQQKD
DHA-1QIF29352.1_349EBOC            GVKSASKDMLRWAEMNMEPSRAGNADLEMAMYLAQTRYYKTAAINQGLGWEMYDWPQQKD
DHA-1QUP93355.1_50EBOC             GVKSASKDMLRWAEMNMEPSRAGNADLEMAMYLAQTRYYKTAAINQGLGWEMYDWPQQKD
DHA-1UKB51966.1_271EBOC            GVKSASKDMLRWAEMNMEPSRAGNADLEMAMYLAQTRYYKTAAINQGLGWEMYDWPQQKD
DHA-1UKB62076.1_255EBOC            GVKSASKDMLRWAEMNMEPSRAGNADLEMAMYLAQTRYYKTAAINQGLGWEMYDWPQQKD
DHA-1UOY35142.1_6EBOC              GVKSASKDMLRWAEMNMEPSRAGNADLEMAMYLAQTRYYKTAAINQGLGWEMYDWPQQKD
DHA-1UOY53746.1_188EBOC            GVKSASKDMLRWAEMNMEPSRAGNADLEMAMYLAQTRYYKTAAINQGLGWEMYDWPQQKD
DHA-1URL72031.1_311EBOC            GVKSASKDMLRWAEMNMEPSRAGNADLEMAMYLAQTRYYKTAAINQGLGWEMYDWPQQKD
DHA-1WAZ65759.1_143EBOC            GVKSASKDMLRWAEMNMEPSRAGNADLEMAMYLAQTRYYKTAAINQGLGWEMYDWPQQKD
DHA-1WGL89115.1_5EBOC              GVKSASKDMLRWAEMNMEPSRAGNADLEMAMYLAQTRYYKTAAINQGLGWEMYDWPQQKD
DHA-1WGZ52333.1_354EBOC            GVKSASKDMLRWAEMNMEPSRAGNADLEMAMYLAQTRYYKTAAINQGLGWEMYDWPQQKD
DHA-1ULK46985.1_701KpBOC           GVKSASKDMLRWAEMNMEPSRAGNADLEMAMYLAQTRYYKTAAINQGLGWEMYDWPQQKD
DHA-1QLR69540.1_4269KpBOC          GVKSASKDMLRWAEMNMEPSRAGNADLEMAMYLAQTRYYKTAAINQGLGWEMYDWPQQKD
DHA-1UMU29130.1_701KpBOC           GVKSASKDMLRWAEMNMEPSRAGNADLEMAMYLAQTRYYKTAAINQGLGWEMYDWPQQKD
DHA-1QTO12878.1_5112KpBOC          GVKSASKDMLRWAEMNMEPSRAGNADLEMAMYLAQTRYYKTAAINQGLGWEMYDWPQQKD
DHA-1QVR56117.1_814KpBOC           GVKSASKDMLRWAEMNMEPSRAGNADLEMAMYLAQTRYYKTAAINQGLGWEMYDWPQQKD
DHA-1WBX47547.1_5024KpBOC          GVKSASKDMLRWAEMNMEPSRAGNADLEMAMYLAQTRYYKTAAINQGLGWEMYDWPQQKD
DHA-1USO45218.1_1502KpBOC          GVKSASKDMLRWAEMNMEPSRAGNADLEMAMYLAQTRYYKTAAINQGLGWEMYDWPQQKD
DHA-1UYH26771.1_3649KpBOC          GVKSASKDMLRWAEMNMEPSRAGNADLEMAMYLAQTRYYKTAAINQGLGWEMYDWPQQKD
DHA-1BCI98564.1_KP_B_O_P           GVKSASKDMLRWAEMNMEPSRAGNADLEMAMYLAQTRYYKTAAINQGLGWEMYDWPQQKD
DHA-1BBR80752.1_1091]KpBOP         GVKSASKDMLRWAEMNMEPSRAGNADLEMAMYLAQTRYYKTAAINQGLGWEMYDWPQQKD
DHA-1ARV42956.1_78KpBOP            GVKSASKDMLRWAEMNMEPSRAGNADLEMAMYLAQTRYYKTAAINQGLGWEMYDWPQQKD
DHA-1QAW92788.1_234KpBOP           GVKSASKDMLRWAEMNMEPSRAGNADLEMAMYLAQTRYYKTAAINQGLGWEMYDWPQQKD
DHA-1AWE04508.1_77KpBOP            GVKSASKDMLRWAEMNMEPSRAGNADLEMAMYLAQTRYYKTAAINQGLGWEMYDWPQQKD
DHA-1AWE05132.1_156KpBOP           GVKSASKDMLRWAEMNMEPSRAGNADLEMAMYLAQTRYYKTAAINQGLGWEMYDWPQQKD
DHA-1AXR54232.1_175KpBOP           GVKSASKDMLRWAEMNMEPSRAGNADLEMAMYLAQTRYYKTAAINQGLGWEMYDWPQQKD
DHA-1QBA36383.1_199KpBOP           GVKSASKDMLRWAEMNMEPSRAGNADLEMAMYLAQTRYYKTAAINQGLGWEMYDWPQQKD
DHA-1QIQ40896.1_277KpBOP           GVKSASKDMLRWAEMNMEPSRAGNADLEMAMYLAQTRYYKTAAINQGLGWEMYDWPQQKD
DHA-1QLB73441.1_45KpBOP            GVKSASKDMLRWAEMNMEPSRAGNADLEMAMYLAQTRYYKTAAINQGLGWEMYDWPQQKD
DHA-1QLB78779.1_63KpBOP            GVKSASKDMLRWAEMNMEPSRAGNADLEMAMYLAQTRYYKTAAINQGLGWEMYDWPQQKD
DHA-1QLB84106.1_44KpBOP            GVKSASKDMLRWAEMNMEPSRAGNADLEMAMYLAQTRYYKTAAINQGLGWEMYDWPQQKD
DHA-1QLB89444.1_44KpBOP            GVKSASKDMLRWAEMNMEPSRAGNADLEMAMYLAQTRYYKTAAINQGLGWEMYDWPQQKD
DHA-1QLB94784.1_45KpBOP            GVKSASKDMLRWAEMNMEPSRAGNADLEMAMYLAQTRYYKTAAINQGLGWEMYDWPQQKD
DHA-1QLC10688.1_45KpBOP            GVKSASKDMLRWAEMNMEPSRAGNADLEMAMYLAQTRYYKTAAINQGLGWEMYDWPQQKD
DHA-1QLC16028.1_45KpBOP            GVKSASKDMLRWAEMNMEPSRAGNADLEMAMYLAQTRYYKTAAINQGLGWEMYDWPQQKD
DHA-1QIJ38370.1_76KpBOP            GVKSASKDMLRWAEMNMEPSRAGNADLEMAMYLAQTRYYKTAAINQGLGWEMYDWPQQKD
DHA-1QIJ23273.1_76KpBOP            GVKSASKDMLRWAEMNMEPSRAGNADLEMAMYLAQTRYYKTAAINQGLGWEMYDWPQQKD
DHA-1QIJ17670.1_76KpBOP            GVKSASKDMLRWAEMNMEPSRAGNADLEMAMYLAQTRYYKTAAINQGLGWEMYDWPQQKD
DHA-1QYI39301.1_102KpBOP           GVKSASKDMLRWAEMNMEPSRAGNADLEMAMYLAQTRYYKTAAINQGLGWEMYDWPQQKD
DHA-1QER51199.1_205KpBOP           GVKSASKDMLRWAEMNMEPSRAGNADLEMAMYLAQTRYYKTAAINQGLGWEMYDWPQQKD
DHA-1QIF88318.1_264KpBOP           GVKSASKDMLRWAEMNMEPSRAGNADLEMAMYLAQTRYYKTAAINQGLGWEMYDWPQQKD
DHA-1QLI11710.1_48KpBOP            GVKSASKDMLRWAEMNMEPSRAGNADLEMAMYLAQTRYYKTAAINQGLGWEMYDWPQQKD
DHA-1QLI17048.1_76KpBOP            GVKSASKDMLRWAEMNMEPSRAGNADLEMAMYLAQTRYYKTAAINQGLGWEMYDWPQQKD
DHA-1QLI22544.1_1KpBOP             GVKSASKDMLRWAEMNMEPSRAGNADLEMAMYLAQTRYYKTAAINQGLGWEMYDWPQQKD
DHA-1QLI33420.1_49KpBOP            GVKSASKDMLRWAEMNMEPSRAGNADLEMAMYLAQTRYYKTAAINQGLGWEMYDWPQQKD
DHA-1ULI82297.1_104KpBOP           GVKSASKDMLRWAEMNMEPSRAGNADLEMAMYLAQTRYYKTAAINQGLGWEMYDWPQQKD
DHA-1ULK26611.1_20KpBOP            GVKSASKDMLRWAEMNMEPSRAGNADLEMAMYLAQTRYYKTAAINQGLGWEMYDWPQQKD
DHA-1ULI14884.1_221KpBOP           GVKSASKDMLRWAEMNMEPSRAGNADLEMAMYLAQTRYYKTAAINQGLGWEMYDWPQQKD
DHA-1UMU88999.1_169KpBOP           GVKSASKDMLRWAEMNMEPSRAGNADLEMAMYLAQTRYYKTAAINQGLGWEMYDWPQQKD
DHA-1QLI93287.1_12KpBOP            GVKSASKDMLRWAEMNMEPSRAGNADLEMAMYLAQTRYYKTAAINQGLGWEMYDWPQQKD
DHA-1QOW59325.1_216KpBOP           GVKSASKDMLRWAEMNMEPSRAGNADLEMAMYLAQTRYYKTAAINQGLGWEMYDWPQQKD
DHA-1QPP83812.1_78KpBOP            GVKSASKDMLRWAEMNMEPSRAGNADLEMAMYLAQTRYYKTAAINQGLGWEMYDWPQQKD
DHA-1QPQ05032.1_31KpBOP            GVKSASKDMLRWAEMNMEPSRAGNADLEMAMYLAQTRYYKTAAINQGLGWEMYDWPQQKD
DHA-1QQL36831.1_206KpBOP           GVKSASKDMLRWAEMNMEPSRAGNADLEMAMYLAQTRYYKTAAINQGLGWEMYDWPQQKD
DHA-1QRR62282.1_23KpBOP            GVKSASKDMLRWAEMNMEPSRAGNADLEMAMYLAQTRYYKTAAINQGLGWEMYDWPQQKD
DHA-1QRR62336.1_13KpBOP            GVKSASKDMLRWAEMNMEPSRAGNADLEMAMYLAQTRYYKTAAINQGLGWEMYDWPQQKD
DHA-1QRR62351.1_34KpBOP            GVKSASKDMLRWAEMNMEPSRAGNADLEMAMYLAQTRYYKTAAINQGLGWEMYDWPQQKD
DHA-1QRR62310.1_56KpBOP            GVKSASKDMLRWAEMNMEPSRAGNADLEMAMYLAQTRYYKTAAINQGLGWEMYDWPQQKD
DHA-1QRS73253.1_51KpBOP            GVKSASKDMLRWAEMNMEPSRAGNADLEMAMYLAQTRYYKTAAINQGLGWEMYDWPQQKD
DHA-1QSC32078.1_75KpBOP            GVKSASKDMLRWAEMNMEPSRAGNADLEMAMYLAQTRYYKTAAINQGLGWEMYDWPQQKD
DHA-1URL54267.1_52KpBOP            GVKSASKDMLRWAEMNMEPSRAGNADLEMAMYLAQTRYYKTAAINQGLGWEMYDWPQQKD
DHA-1QSW33401.1_150KpBOP           GVKSASKDMLRWAEMNMEPSRAGNADLEMAMYLAQTRYYKTAAINQGLGWEMYDWPQQKD
DHA-1QTX39082.1_340KpBOP           GVKSASKDMLRWAEMNMEPSRAGNADLEMAMYLAQTRYYKTAAINQGLGWEMYDWPQQKD
DHA-1QUX17533.1_117KpBOP           GVKSASKDMLRWAEMNMEPSRAGNADLEMAMYLAQTRYYKTAAINQGLGWEMYDWPQQKD
DHA-1QVQ94431.1_179KpBOP           GVKSASKDMLRWAEMNMEPSRAGNADLEMAMYLAQTRYYKTAAINQGLGWEMYDWPQQKD
DHA-1QVR16641.1_60KpBOP            GVKSASKDMLRWAEMNMEPSRAGNADLEMAMYLAQTRYYKTAAINQGLGWEMYDWPQQKD
DHA-1QVU34134.1_265KpBOP           GVKSASKDMLRWAEMNMEPSRAGNADLEMAMYLAQTRYYKTAAINQGLGWEMYDWPQQKD
DHA-1QVW15542.1_136KpBOP           GVKSASKDMLRWAEMNMEPSRAGNADLEMAMYLAQTRYYKTAAINQGLGWEMYDWPQQKD
DHA-1QVW15569.1_171KpBOP           GVKSASKDMLRWAEMNMEPSRAGNADLEMAMYLAQTRYYKTAAINQGLGWEMYDWPQQKD
DHA-1QXQ84174.1_24KpBOP            GVKSASKDMLRWAEMNMEPSRAGNADLEMAMYLAQTRYYKTAAINQGLGWEMYDWPQQKD
DHA-1QXT85906.1_24KpBOP            GVKSASKDMLRWAEMNMEPSRAGNADLEMAMYLAQTRYYKTAAINQGLGWEMYDWPQQKD
DHA-1QXU18235.1_24KpBOP            GVKSASKDMLRWAEMNMEPSRAGNADLEMAMYLAQTRYYKTAAINQGLGWEMYDWPQQKD
DHA-1UNH49680.1_195KpBOP           GVKSASKDMLRWAEMNMEPSRAGNADLEMAMYLAQTRYYKTAAINQGLGWEMYDWPQQKD
DHA-1UGM94295.1_45KpBOP            GVKSASKDMLRWAEMNMEPSRAGNADLEMAMYLAQTRYYKTAAINQGLGWEMYDWPQQKD
DHA-1UCM37138.1_188KpBOP           GVKSASKDMLRWAEMNMEPSRAGNADLEMAMYLAQTRYYKTAAINQGLGWEMYDWPQQKD
DHA-1UPF47572.1_185KpBOP           GVKSASKDMLRWAEMNMEPSRAGNADLEMAMYLAQTRYYKTAAINQGLGWEMYDWPQQKD
DHA-1UPF53088.1_98KpBOP            GVKSASKDMLRWAEMNMEPSRAGNADLEMAMYLAQTRYYKTAAINQGLGWEMYDWPQQKD
DHA-1UNE95136.1_177KpBOP           GVKSASKDMLRWAEMNMEPSRAGNADLEMAMYLAQTRYYKTAAINQGLGWEMYDWPQQKD
DHA-1UGQ70597.1_19KpBOP            GVKSASKDMLRWAEMNMEPSRAGNADLEMAMYLAQTRYYKTAAINQGLGWEMYDWPQQKD
DHA-1UGQ75588.1_15KpBOP            GVKSASKDMLRWAEMNMEPSRAGNADLEMAMYLAQTRYYKTAAINQGLGWEMYDWPQQKD
DHA-1UGQ80838.1_30KpBOP            GVKSASKDMLRWAEMNMEPSRAGNADLEMAMYLAQTRYYKTAAINQGLGWEMYDWPQQKD
DHA-1UGR08221.1_34KpBOP            GVKSASKDMLRWAEMNMEPSRAGNADLEMAMYLAQTRYYKTAAINQGLGWEMYDWPQQKD
DHA-1UGR13826.1_19KpBOP            GVKSASKDMLRWAEMNMEPSRAGNADLEMAMYLAQTRYYKTAAINQGLGWEMYDWPQQKD
DHA-1UGS19283.1_181KpBOP           GVKSASKDMLRWAEMNMEPSRAGNADLEMAMYLAQTRYYKTAAINQGLGWEMYDWPQQKD
DHA-1UII40113.1_155KpBOP           GVKSASKDMLRWAEMNMEPSRAGNADLEMAMYLAQTRYYKTAAINQGLGWEMYDWPQQKD
DHA-1UIY63185.1_63KpBOP            GVKSASKDMLRWAEMNMEPSRAGNADLEMAMYLAQTRYYKTAAINQGLGWEMYDWPQQKD
DHA-1UZK63620.1_210KpBOP           GVKSASKDMLRWAEMNMEPSRAGNADLEMAMYLAQTRYYKTAAINQGLGWEMYDWPQQKD
DHA-1UPS70201.1_18KpBOP            GVKSASKDMLRWAEMNMEPSRAGNADLEMAMYLAQTRYYKTAAINQGLGWEMYDWPQQKD
DHA-1WDO24504.1_185KpBOP           GVKSASKDMLRWAEMNMEPSRAGNADLEMAMYLAQTRYYKTAAINQGLGWEMYDWPQQKD
DHA-1URT78846.1_121KpBOP           GVKSASKDMLRWAEMNMEPSRAGNADLEMAMYLAQTRYYKTAAINQGLGWEMYDWPQQKD
DHA-1URT89155.1_166KpBOP           GVKSASKDMLRWAEMNMEPSRAGNADLEMAMYLAQTRYYKTAAINQGLGWEMYDWPQQKD
DHA-1URT89430.1_166KpBOP           GVKSASKDMLRWAEMNMEPSRAGNADLEMAMYLAQTRYYKTAAINQGLGWEMYDWPQQKD
DHA-1URT94913.1_167KpBOP           GVKSASKDMLRWAEMNMEPSRAGNADLEMAMYLAQTRYYKTAAINQGLGWEMYDWPQQKD
DHA-1UWZ32448.1_52KpBOP            GVKSASKDMLRWAEMNMEPSRAGNADLEMAMYLAQTRYYKTAAINQGLGWEMYDWPQQKD
DHA-1UTW92771.1_126KpBOP           GVKSASKDMLRWAEMNMEPSRAGNADLEMAMYLAQTRYYKTAAINQGLGWEMYDWPQQKD
DHA-1UZW73111.1_23KpBOP            GVKSASKDMLRWAEMNMEPSRAGNADLEMAMYLAQTRYYKTAAINQGLGWEMYDWPQQKD
DHA-1WEI85275.1_173KpBOP           GVKSASKDMLRWAEMNMEPSRAGNADLEMAMYLAQTRYYKTAAINQGLGWEMYDWPQQKD
DHA-1UVF97054.1_214KpBOP           GVKSASKDMLRWAEMNMEPSRAGNADLEMAMYLAQTRYYKTAAINQGLGWEMYDWPQQKD
DHA-1UVN34295.1_69KpBOP            GVKSASKDMLRWAEMNMEPSRAGNADLEMAMYLAQTRYYKTAAINQGLGWEMYDWPQQKD
DHA-1UWB42605.1_210KpBOP           GVKSASKDMLRWAEMNMEPSRAGNADLEMAMYLAQTRYYKTAAINQGLGWEMYDWPQQKD
DHA-1UWX53529.1_18KpBOP            GVKSASKDMLRWAEMNMEPSRAGNADLEMAMYLAQTRYYKTAAINQGLGWEMYDWPQQKD
DHA-1WII50269.1_174KpBOP           GVKSASKDMLRWAEMNMEPSRAGNADLEMAMYLAQTRYYKTAAINQGLGWEMYDWPQQKD
DHA-1WII50721.1_91KpBOP            GVKSASKDMLRWAEMNMEPSRAGNADLEMAMYLAQTRYYKTAAINQGLGWEMYDWPQQKD
DHA-1UXY66557.1_128KpBOP           GVKSASKDMLRWAEMNMEPSRAGNADLEMAMYLAQTRYYKTAAINQGLGWEMYDWPQQKD
DHA-1UYX39647.1_199KpBOP           GVKSASKDMLRWAEMNMEPSRAGNADLEMAMYLAQTRYYKTAAINQGLGWEMYDWPQQKD
DHA-1UZE57593.1_95KpBOP            GVKSASKDMLRWAEMNMEPSRAGNADLEMAMYLAQTRYYKTAAINQGLGWEMYDWPQQKD
DHA-1WAD38926.1_6KpBOP             GVKSASKDMLRWAEMNMEPSRAGNADLEMAMYLAQTRYYKTAAINQGLGWEMYDWPQQKD
DHA-1WAD13598.1_210KpBOP           GVKSASKDMLRWAEMNMEPSRAGNADLEMAMYLAQTRYYKTAAINQGLGWEMYDWPQQKD
DHA-1WAD08359.1_184KpBOP           GVKSASKDMLRWAEMNMEPSRAGNADLEMAMYLAQTRYYKTAAINQGLGWEMYDWPQQKD
DHA-1WAD18885.1_185KpBOP           GVKSASKDMLRWAEMNMEPSRAGNADLEMAMYLAQTRYYKTAAINQGLGWEMYDWPQQKD
DHA-1WAD19356.1_183KpBOP           GVKSASKDMLRWAEMNMEPSRAGNADLEMAMYLAQTRYYKTAAINQGLGWEMYDWPQQKD
DHA-1WAD44078.1_264KpBOP           GVKSASKDMLRWAEMNMEPSRAGNADLEMAMYLAQTRYYKTAAINQGLGWEMYDWPQQKD
DHA-1WAD49440.1_195KpBOP           GVKSASKDMLRWAEMNMEPSRAGNADLEMAMYLAQTRYYKTAAINQGLGWEMYDWPQQKD
DHA-1WAD71205.1_194KpBOP           GVKSASKDMLRWAEMNMEPSRAGNADLEMAMYLAQTRYYKTAAINQGLGWEMYDWPQQKD
DHA-1WAD55056.1_244KpBOP           GVKSASKDMLRWAEMNMEPSRAGNADLEMAMYLAQTRYYKTAAINQGLGWEMYDWPQQKD
DHA-1WAD60732.1_211KpBOP           GVKSASKDMLRWAEMNMEPSRAGNADLEMAMYLAQTRYYKTAAINQGLGWEMYDWPQQKD
DHA-1WAD61281.1_278KpBOP           GVKSASKDMLRWAEMNMEPSRAGNADLEMAMYLAQTRYYKTAAINQGLGWEMYDWPQQKD
DHA-1WFF21460.1_31KpBOP            GVKSASKDMLRWAEMNMEPSRAGNADLEMAMYLAQTRYYKTAAINQGLGWEMYDWPQQKD
DHA-1WHL16843.1_345KpBOP           GVKSASKDMLRWAEMNMEPSRAGNADLEMAMYLAQTRYYKTAAINQGLGWEMYDWPQQKD
DHA-1WP_004236386.1KpOC            GVKSASKDMLRWAEMNMEPSRAGNADLEMAMYLAQTRYYKTAAINQGLGWEMYDWPQQKD
DHA-1WP_004236386.1_4280KpBCP      GVKSASKDMLRWAEMNMEPSRAGNADLEMAMYLAQTRYYKTAAINQGLGWEMYDWPQQKD
DHA-1WP_004236386.1_5142KpBCP      GVKSASKDMLRWAEMNMEPSRAGNADLEMAMYLAQTRYYKTAAINQGLGWEMYDWPQQKD
DHA-1WP_004236386.1_814KpBCP       GVKSASKDMLRWAEMNMEPSRAGNADLEMAMYLAQTRYYKTAAINQGLGWEMYDWPQQKD
DHA-1WP_004236386.1_821KpBCP       GVKSASKDMLRWAEMNMEPSRAGNADLEMAMYLAQTRYYKTAAINQGLGWEMYDWPQQKD
DHA-1WP_004236386.1_817KpBCP       GVKSASKDMLRWAEMNMEPSRAGNADLEMAMYLAQTRYYKTAAINQGLGWEMYDWPQQKD
DHA-1WP_004236386.1_700KpBCP       GVKSASKDMLRWAEMNMEPSRAGNADLEMAMYLAQTRYYKTAAINQGLGWEMYDWPQQKD
DHA-1USO45218.1_1502KpBCP         GVKSASKDMLRWAEMNMEPSRAGNADLEMAMYLAQTRYYKTAAINQGLGWEMYDWPQQKD
DHA-1WP_004236386.1_3654KpBCP      GVKSASKDMLRWAEMNMEPSRAGNADLEMAMYLAQTRYYKTAAINQGLGWEMYDWPQQKD
DHA-1WP_004236386.1_5030KpBCP      GVKSASKDMLRWAEMNMEPSRAGNADLEMAMYLAQTRYYKTAAINQGLGWEMYDWPQQKD
DHA-7AM390_27335_79KpBOP           GVKSASKDMLRWAEMNMEPSRAGNA...................................
DHA-7AM395_28070_58KpBOP           GVKSASKDMLRWAEMNMEPSRAGNA...................................
DHA-7AN663_28510_174KpBOP          GVKSASKDMLRWAEMNMEPSRAGNA...................................
                              acc
```

```
                                 α10        α11      β12   β13              β14                β15      η7     β16
5GGW_1|Chains
                                        290               300            310             320             330             340
5GGW_1|Chains                     SIINGSGNKIALAAHPVKAITPPTPAVRASWVHKTGATGGFGSYVAFIPEKELGIVMLAN
DHA-15WGF99578.1_254KpBOP         MIINGVTNEVALQPHPVTD.NQVQPYNRASWVHKTGATTGFGAYVAFIPEKQVAIVILAN
DHA-1AVZ12228.1_302EBOC           MIINGVTNEVALQPHPVTD.NQVQPYNRASWVHKTGATTGFGAYVAFIPEKQVAIVILAN
DHA-1QGU39370.1_323EBOC           MIINGVTNEVALQPHPVTD.NQVQPYNRASWVHKTGATTGFGAYVAFIPEKQVAIVILAN
DHA-1QIE67107.1_279EBOC           MIINGVTNEVALQPHPVTD.NQVQPYNRASWVHKTGATTGFGAYVAFIPEKQVAIVILAN
DHA-1QIF29352.1_349EBOC           MIINGVTNEVALQPHPVTD.NQVQPYNRASWVHKTGATTGFGAYVAFIPEKQVAIVILAN
DHA-1QUP93355.1_50EBOC            MIINGVTNEVALQPHPVTD.NQVQPYNRASWVHKTGATTGFGAYVAFIPEKQVAIVILAN
DHA-1UKB51966.1_271EBOC           MIINGVTNEVALQPHPVTD.NQVQPYNRASWVHKTGATTGFGAYVAFIPEKQVAIVILAN
DHA-1UKB62076.1_255EBOC           MIINGVTNEVALQPHPVTD.NQVQPYNRASWVHKTGATTGFGAYVAFIPEKQVAIVILAN
DHA-1UOY35142.1_6EBOC             MIINGVTNEVALQPHPVTD.NQVQPYNRASWVHKTGATTGFGAYVAFIPEKQVAIVILAN
DHA-1UOY53746.1_188EBOC           MIINGVTNEVALQPHPVTD.NQVQPYNRASWVHKTGATTGFGAYVAFIPEKQVAIVILAN
DHA-1URL72031.1_311EBOC           MIINGVTNEVALQPHPVTD.NQVQPYNRASWVHKTGATTGFGAYVAFIPEKQVAIVILAN
DHA-1WAZ65759.1_143EBOC           MIINGVTNEVALQPHPVTD.NQVQPYNRASWVHKTGATTGFGAYVAFIPEKQVAIVILAN
DHA-1WGL89115.1_5EBOC             MIINGVTNEVALQPHPVTD.NQVQPYNRASWVHKTGATTGFGAYVAFIPEKQVAIVILAN
DHA-1WGZ52333.1_354EBOC           MIINGVTNEVALQPHPVTD.NQVQPYNRASWVHKTGATTGFGAYVAFIPEKQVAIVILAN
DHA-1ULK46985.1_701KpBOC          MIINGVTNEVALQPHPVTD.NQVQPYNRASWVHKTGATTGFGAYVAFIPEKQVAIVILAN
DHA-1QLR69540.1_4269KpBOC         MIINGVTNEVALQPHPVTD.NQVQPYNRASWVHKTGATTGFGAYVAFIPEKQVAIVILAN
DHA-1UMU29130.1_701KpBOC          MIINGVTNEVALQPHPVTD.NQVQPYNRASWVHKTGATTGFGAYVAFIPEKQVAIVILAN
DHA-1QTO12878.1_5112KpBOC         MIINGVTNEVALQPHPVTD.NQVQPYNRASWVHKTGATTGFGAYVAFIPEKQVAIVILAN
DHA-1QVR56117.1_814KpBOC          MIINGVTNEVALQPHPVTD.NQVQPYNRASWVHKTGATTGFGAYVAFIPEKQVAIVILAN
DHA-1WBX47547.1_5024KpBOC         MIINGVTNEVALQPHPVTD.NQVQPYNRASWVHKTGATTGFGAYVAFIPEKQVAIVILAN
DHA-1USO45218.1_1502KpBOC         MIINGVTNEVALQPHPVTD.NQVQPYNRASWVHKTGATTGFGAYVAFIPEKQVAIVILAN
DHA-1UYH26771.1_3649KpBOC         MIINGVTNEVALQPHPVTD.NQVQPYNRASWVHKTGATTGFGAYVAFIPEKQVAIVILAN
DHA-1BCI98564.1_KP_B_O_P          MIINGVTNEVALQPHPVTD.NQVQPYNRASWVHKTGATTGFGAYVAFIPEKQVAIVILAN
DHA-1BBR80752.1_1091]KpBOP        MIINGVTNEVALQPHPVTD.NQVQPYNRASWVHKTGATTGFGAYVAFIPEKQVAIVILAN
DHA-1ARV42956.1_78KpBOP           MIINGVTNEVALQPHPVTD.NQVQPYNRASWVHKTGATTGFGAYVAFIPEKQVAIVILAN
DHA-1QAW92788.1_234KpBOP          MIINGVTNEVALQPHPVTD.NQVQPYNRASWVHKTGATTGFGAYVAFIPEKQVAIVILAN
DHA-1AWE04508.1_77KpBOP           MIINGVTNEVALQPHPVTD.NQVQPYNRASWVHKTGATTGFGAYVAFIPEKQVAIVILAN
DHA-1AWE05132.1_156KpBOP          MIINGVTNEVALQPHPVTD.NQVQPYNRASWVHKTGATTGFGAYVAFIPEKQVAIVILAN
DHA-1AXR54232.1_175KpBOP          MIINGVTNEVALQPHPVTD.NQVQPYNRASWVHKTGATTGFGAYVAFIPEKQVAIVILAN
DHA-1QBA36383.1_199KpBOP          MIINGVTNEVALQPHPVTD.NQVQPYNRASWVHKTGATTGFGAYVAFIPEKQVAIVILAN
DHA-1QIQ40896.1_277KpBOP          MIINGVTNEVALQPHPVTD.NQVQPYNRASWVHKTGATTGFGAYVAFIPEKQVAIVILAN
DHA-1QLB73441.1_45KpBOP           MIINGVTNEVALQPHPVTD.NQVQPYNRASWVHKTGATTGFGAYVAFIPEKQVAIVILAN
DHA-1QLB78779.1_63KpBOP           MIINGVTNEVALQPHPVTD.NQVQPYNRASWVHKTGATTGFGAYVAFIPEKQVAIVILAN
DHA-1QLB84106.1_44KpBOP           MIINGVTNEVALQPHPVTD.NQVQPYNRASWVHKTGATTGFGAYVAFIPEKQVAIVILAN
DHA-1QLB89444.1_44KpBOP           MIINGVTNEVALQPHPVTD.NQVQPYNRASWVHKTGATTGFGAYVAFIPEKQVAIVILAN
DHA-1QLB94784.1_45KpBOP           MIINGVTNEVALQPHPVTD.NQVQPYNRASWVHKTGATTGFGAYVAFIPEKQVAIVILAN
DHA-1QLC10688.1_45KpBOP           MIINGVTNEVALQPHPVTD.NQVQPYNRASWVHKTGATTGFGAYVAFIPEKQVAIVILAN
DHA-1QLC16028.1_45KpBOP           MIINGVTNEVALQPHPVTD.NQVQPYNRASWVHKTGATTGFGAYVAFIPEKQVAIVILAN
DHA-1QIJ38370.1_76KpBOP           MIINGVTNEVALQPHPVTD.NQVQPYNRASWVHKTGATTGFGAYVAFIPEKQVAIVILAN
DHA-1QIJ23273.1_76KpBOP           MIINGVTNEVALQPHPVTD.NQVQPYNRASWVHKTGATTGFGAYVAFIPEKQVAIVILAN
DHA-1QIJ17670.1_76KpBOP           MIINGVTNEVALQPHPVTD.NQVQPYNRASWVHKTGATTGFGAYVAFIPEKQVAIVILAN
DHA-1QYI39301.1_102KpBOP          MIINGVTNEVALQPHPVTD.NQVQPYNRASWVHKTGATTGFGAYVAFIPEKQVAIVILAN
DHA-1QER51199.1_205KpBOP          MIINGVTNEVALQPHPVTD.NQVQPYNRASWVHKTGATTGFGAYVAFIPEKQVAIVILAN
DHA-1QIF88318.1_264KpBOP          MIINGVTNEVALQPHPVTD.NQVQPYNRASWVHKTGATTGFGAYVAFIPEKQVAIVILAN
DHA-1QLI11710.1_48KpBOP           MIINGVTNEVALQPHPVTD.NQVQPYNRASWVHKTGATTGFGAYVAFIPEKQVAIVILAN
DHA-1QLI17048.1_76KpBOP           MIINGVTNEVALQPHPVTD.NQVQPYNRASWVHKTGATTGFGAYVAFIPEKQVAIVILAN
DHA-1QLI22544.1_1KpBOP            MIINGVTNEVALQPHPVTD.NQVQPYNRASWVHKTGATTGFGAYVAFIPEKQVAIVILAN
DHA-1QLI33420.1_49KpBOP           MIINGVTNEVALQPHPVTD.NQVQPYNRASWVHKTGATTGFGAYVAFIPEKQVAIVILAN
DHA-1ULI82297.1_104KpBOP          MIINGVTNEVALQPHPVTD.NQVQPYNRASWVHKTGATTGFGAYVAFIPEKQVAIVILAN
DHA-1ULK26611.1_20KpBOP           MIINGVTNEVALQPHPVTD.NQVQPYNRASWVHKTGATTGFGAYVAFIPEKQVAIVILAN
DHA-1ULI14884.1_221KpBOP          MIINGVTNEVALQPHPVTD.NQVQPYNRASWVHKTGATTGFGAYVAFIPEKQVAIVILAN
DHA-1UMU88999.1_169KpBOP          MIINGVTNEVALQPHPVTD.NQVQPYNRASWVHKTGATTGFGAYVAFIPEKQVAIVILAN
DHA-1QLI93287.1_12KpBOP           MIINGVTNEVALQPHPVTD.NQVQPYNRASWVHKTGATTGFGAYVAFIPEKQVAIVILAN
DHA-1QOW59325.1_216KpBOP          MIINGVTNEVALQPHPVTD.NQVQPYNRASWVHKTGATTGFGAYVAFIPEKQVAIVILAN
DHA-1QPP83812.1_78KpBOP           MIINGVTNEVALQPHPVTD.NQVQPYNRASWVHKTGATTGFGAYVAFIPEKQVAIVILAN
DHA-1QPQ05032.1_31KpBOP           MIINGVTNEVALQPHPVTD.NQVQPYNRASWVHKTGATTGFGAYVAFIPEKQVAIVILAN
DHA-1QQL36831.1_206KpBOP          MIINGVTNEVALQPHPVTD.NQVQPYNRASWVHKTGATTGFGAYVAFIPEKQVAIVILAN
DHA-1QRR62282.1_23KpBOP           MIINGVTNEVALQPHPVTD.NQVQPYNRASWVHKTGATTGFGAYVAFIPEKQVAIVILAN
DHA-1QRR62336.1_13KpBOP           MIINGVTNEVALQPHPVTD.NQVQPYNRASWVHKTGATTGFGAYVAFIPEKQVAIVILAN
DHA-1QRR62351.1_34KpBOP           MIINGVTNEVALQPHPVTD.NQVQPYNRASWVHKTGATTGFGAYVAFIPEKQVAIVILAN
DHA-1QRR62310.1_56KpBOP           MIINGVTNEVALQPHPVTD.NQVQPYNRASWVHKTGATTGFGAYVAFIPEKQVAIVILAN
DHA-1QRS73253.1_51KpBOP           MIINGVTNEVALQPHPVTD.NQVQPYNRASWVHKTGATTGFGAYVAFIPEKQVAIVILAN
DHA-1QSC32078.1_75KpBOP           MIINGVTNEVALQPHPVTD.NQVQPYNRASWVHKTGATTGFGAYVAFIPEKQVAIVILAN
DHA-1URL54267.1_52KpBOP           MIINGVTNEVALQPHPVTD.NQVQPYNRASWVHKTGATTGFGAYVAFIPEKQVAIVILAN
DHA-1QSW33401.1_150KpBOP          MIINGVTNEVALQPHPVTD.NQVQPYNRASWVHKTGATTGFGAYVAFIPEKQVAIVILAN
DHA-1QTX39082.1_340KpBOP          MIINGVTNEVALQPHPVTD.NQVQPYNRASWVHKTGATTGFGAYVAFIPEKQVAIVILAN
DHA-1QUX17533.1_117KpBOP          MIINGVTNEVALQPHPVTD.NQVQPYNRASWVHKTGATTGFGAYVAFIPEKQVAIVILAN
DHA-1QVQ94431.1_179KpBOP          MIINGVTNEVALQPHPVTD.NQVQPYNRASWVHKTGATTGFGAYVAFIPEKQVAIVILAN
DHA-1QVR16641.1_60KpBOP           MIINGVTNEVALQPHPVTD.NQVQPYNRASWVHKTGATTGFGAYVAFIPEKQVAIVILAN
DHA-1QVU34134.1_265KpBOP          MIINGVTNEVALQPHPVTD.NQVQPYNRASWVHKTGATTGFGAYVAFIPEKQVAIVILAN
DHA-1QVW15542.1_136KpBOP          MIINGVTNEVALQPHPVTD.NQVQPYNRASWVHKTGATTGFGAYVAFIPEKQVAIVILAN
DHA-1QVW15569.1_171KpBOP          MIINGVTNEVALQPHPVTD.NQVQPYNRASWVHKTGATTGFGAYVAFIPEKQVAIVILAN
DHA-1QXQ84174.1_24KpBOP           MIINGVTNEVALQPHPVTD.NQVQPYNRASWVHKTGATTGFGAYVAFIPEKQVAIVILAN
DHA-1QXT85906.1_24KpBOP           MIINGVTNEVALQPHPVTD.NQVQPYNRASWVHKTGATTGFGAYVAFIPEKQVAIVILAN
DHA-1QXU18235.1_24KpBOP           MIINGVTNEVALQPHPVTD.NQVQPYNRASWVHKTGATTGFGAYVAFIPEKQVAIVILAN
DHA-1UNH49680.1_195KpBOP          MIINGVTNEVALQPHPVTD.NQVQPYNRASWVHKTGATTGFGAYVAFIPEKQVAIVILAN
DHA-1UGM94295.1_45KpBOP           MIINGVTNEVALQPHPVTD.NQVQPYNRASWVHKTGATTGFGAYVAFIPEKQVAIVILAN
DHA-1UCM37138.1_188KpBOP          MIINGVTNEVALQPHPVTD.NQVQPYNRASWVHKTGATTGFGAYVAFIPEKQVAIVILAN
DHA-1UPF47572.1_185KpBOP          MIINGVTNEVALQPHPVTD.NQVQPYNRASWVHKTGATTGFGAYVAFIPEKQVAIVILAN
DHA-1UPF53088.1_98KpBOP           MIINGVTNEVALQPHPVTD.NQVQPYNRASWVHKTGATTGFGAYVAFIPEKQVAIVILAN
DHA-1UNE95136.1_177KpBOP          MIINGVTNEVALQPHPVTD.NQVQPYNRASWVHKTGATTGFGAYVAFIPEKQVAIVILAN
DHA-1UGQ70597.1_19KpBOP           MIINGVTNEVALQPHPVTD.NQVQPYNRASWVHKTGATTGFGAYVAFIPEKQVAIVILAN
DHA-1UGQ75588.1_15KpBOP           MIINGVTNEVALQPHPVTD.NQVQPYNRASWVHKTGATTGFGAYVAFIPEKQVAIVILAN
DHA-1UGQ80838.1_30KpBOP           MIINGVTNEVALQPHPVTD.NQVQPYNRASWVHKTGATTGFGAYVAFIPEKQVAIVILAN
DHA-1UGR08221.1_34KpBOP           MIINGVTNEVALQPHPVTD.NQVQPYNRASWVHKTGATTGFGAYVAFIPEKQVAIVILAN
DHA-1UGR13826.1_19KpBOP           MIINGVTNEVALQPHPVTD.NQVQPYNRASWVHKTGATTGFGAYVAFIPEKQVAIVILAN
DHA-1UGS19283.1_181KpBOP          MIINGVTNEVALQPHPVTD.NQVQPYNRASWVHKTGATTGFGAYVAFIPEKQVAIVILAN
DHA-1UII40113.1_155KpBOP          MIINGVTNEVALQPHPVTD.NQVQPYNRASWVHKTGATTGFGAYVAFIPEKQVAIVILAN
DHA-1UIY63185.1_63KpBOP           MIINGVTNEVALQPHPVTD.NQVQPYNRASWVHKTGATTGFGAYVAFIPEKQVAIVILAN
DHA-1UZK63620.1_210KpBOP          MIINGVTNEVALQPHPVTD.NQVQPYNRASWVHKTGATTGFGAYVAFIPEKQVAIVILAN
DHA-1UPS70201.1_18KpBOP           MIINGVTNEVALQPHPVTD.NQVQPYNRASWVHKTGATTGFGAYVAFIPEKQVAIVILAN
DHA-1WDO24504.1_185KpBOP          MIINGVTNEVALQPHPVTD.NQVQPYNRASWVHKTGATTGFGAYVAFIPEKQVAIVILAN
DHA-1URT78846.1_121KpBOP          MIINGVTNEVALQPHPVTD.NQVQPYNRASWVHKTGATTGFGAYVAFIPEKQVAIVILAN
DHA-1URT89155.1_166KpBOP          MIINGVTNEVALQPHPVTD.NQVQPYNRASWVHKTGATTGFGAYVAFIPEKQVAIVILAN
DHA-1URT89430.1_166KpBOP          MIINGVTNEVALQPHPVTD.NQVQPYNRASWVHKTGATTGFGAYVAFIPEKQVAIVILAN
DHA-1URT94913.1_167KpBOP          MIINGVTNEVALQPHPVTD.NQVQPYNRASWVHKTGATTGFGAYVAFIPEKQVAIVILAN
DHA-1UWZ32448.1_52KpBOP           MIINGVTNEVALQPHPVTD.NQVQPYNRASWVHKTGATTGFGAYVAFIPEKQVAIVILAN
DHA-1UTW92771.1_126KpBOP          MIINGVTNEVALQPHPVTD.NQVQPYNRASWVHKTGATTGFGAYVAFIPEKQVAIVILAN
DHA-1UZW73111.1_23KpBOP           MIINGVTNEVALQPHPVTD.NQVQPYNRASWVHKTGATTGFGAYVAFIPEKQVAIVILAN
DHA-1WEI85275.1_173KpBOP          MIINGVTNEVALQPHPVTD.NQVQPYNRASWVHKTGATTGFGAYVAFIPEKQVAIVILAN
DHA-1UVF97054.1_214KpBOP          MIINGVTNEVALQPHPVTD.NQVQPYNRASWVHKTGATTGFGAYVAFIPEKQVAIVILAN
DHA-1UVN34295.1_69KpBOP           MIINGVTNEVALQPHPVTD.NQVQPYNRASWVHKTGATTGFGAYVAFIPEKQVAIVILAN
DHA-1UWB42605.1_210KpBOP          MIINGVTNEVALQPHPVTD.NQVQPYNRASWVHKTGATTGFGAYVAFIPEKQVAIVILAN
DHA-1UWX53529.1_18KpBOP           MIINGVTNEVALQPHPVTD.NQVQPYNRASWVHKTGATTGFGAYVAFIPEKQVAIVILAN
DHA-1WII50269.1_174KpBOP          MIINGVTNEVALQPHPVTD.NQVQPYNRASWVHKTGATTGFGAYVAFIPEKQVAIVILAN
DHA-1WII50721.1_91KpBOP           MIINGVTNEVALQPHPVTD.NQVQPYNRASWVHKTGATTGFGAYVAFIPEKQVAIVILAN
DHA-1UXY66557.1_128KpBOP          MIINGVTNEVALQPHPVTD.NQVQPYNRASWVHKTGATTGFGAYVAFIPEKQVAIVILAN
DHA-1UYX39647.1_199KpBOP          MIINGVTNEVALQPHPVTD.NQVQPYNRASWVHKTGATTGFGAYVAFIPEKQVAIVILAN
DHA-1UZE57593.1_95KpBOP           MIINGVTNEVALQPHPVTD.NQVQPYNRASWVHKTGATTGFGAYVAFIPEKQVAIVILAN
DHA-1WAD38926.1_6KpBOP            MIINGVTNEVALQPHPVTD.NQVQPYNRASWVHKTGATTGFGAYVAFIPEKQVAIVILAN
DHA-1WAD13598.1_210KpBOP          MIINGVTNEVALQPHPVTD.NQVQPYNRASWVHKTGATTGFGAYVAFIPEKQVAIVILAN
DHA-1WAD08359.1_184KpBOP          MIINGVTNEVALQPHPVTD.NQVQPYNRASWVHKTGATTGFGAYVAFIPEKQVAIVILAN
DHA-1WAD18885.1_185KpBOP          MIINGVTNEVALQPHPVTD.NQVQPYNRASWVHKTGATTGFGAYVAFIPEKQVAIVILAN
DHA-1WAD19356.1_183KpBOP          MIINGVTNEVALQPHPVTD.NQVQPYNRASWVHKTGATTGFGAYVAFIPEKQVAIVILAN
DHA-1WAD44078.1_264KpBOP          MIINGVTNEVALQPHPVTD.NQVQPYNRASWVHKTGATTGFGAYVAFIPEKQVAIVILAN
DHA-1WAD49440.1_195KpBOP          MIINGVTNEVALQPHPVTD.NQVQPYNRASWVHKTGATTGFGAYVAFIPEKQVAIVILAN
DHA-1WAD71205.1_194KpBOP          MIINGVTNEVALQPHPVTD.NQVQPYNRASWVHKTGATTGFGAYVAFIPEKQVAIVILAN
DHA-1WAD55056.1_244KpBOP          MIINGVTNEVALQPHPVTD.NQVQPYNRASWVHKTGATTGFGAYVAFIPEKQVAIVILAN
DHA-1WAD60732.1_211KpBOP          MIINGVTNEVALQPHPVTD.NQVQPYNRASWVHKTGATTGFGAYVAFIPEKQVAIVILAN
DHA-1WAD61281.1_278KpBOP          MIINGVTNEVALQPHPVTD.NQVQPYNRASWVHKTGATTGFGAYVAFIPEKQVAIVILAN
DHA-1WFF21460.1_31KpBOP           MIINGVTNEVALQPHPVTD.NQVQPYNRASWVHKTGATTGFGAYVAFIPEKQVAIVILAN
DHA-1WHL16843.1_345KpBOP          MIINGVTNEVALQPHPVTD.NQVQPYNRASWVHKTGATTGFGAYVAFIPEKQVAIVILAN
DHA-1WP_004236386.1KpOC           MIINGVTNEVALQPHPVTD.NQVQPYNRASWVHKTGATTGFGAYVAFIPEKQVAIVILAN
DHA-1WP_004236386.1_4280KpBCP     MIINGVTNEVALQPHPVTD.NQVQPYNRASWVHKTGATTGFGAYVAFIPEKQVAIVILAN
DHA-1WP_004236386.1_5142KpBCP     MIINGVTNEVALQPHPVTD.NQVQPYNRASWVHKTGATTGFGAYVAFIPEKQVAIVILAN
DHA-1WP_004236386.1_814KpBCP      MIINGVTNEVALQPHPVTD.NQVQPYNRASWVHKTGATTGFGAYVAFIPEKQVAIVILAN
DHA-1WP_004236386.1_821KpBCP      MIINGVTNEVALQPHPVTD.NQVQPYNRASWVHKTGATTGFGAYVAFIPEKQVAIVILAN
DHA-1WP_004236386.1_817KpBCP      MIINGVTNEVALQPHPVTD.NQVQPYNRASWVHKTGATTGFGAYVAFIPEKQVAIVILAN
DHA-1WP_004236386.1_700KpBCP      MIINGVTNEVALQPHPVTD.NQVQPYNRASWVHKTGATTGFGAYVAFIPEKQVAIVILAN
DHA-1USO45218.1_1502KpBCP         MIINGVTNEVALQPHPVTD.NQVQPYNRASWVHKTGATTGFGAYVAFIPEKQVAIVILAN
DHA-1WP_004236386.1_3654KpBCP     MIINGVTNEVALQPHPVTD.NQVQPYNRASWVHKTGATTGFGAYVAFIPEKQVAIVILAN
DHA-1WP_004236386.1_5030KpBCP     MIINGVTNEVALQPHPVTD.NQVQPYNRASWVHKTGATTGFGAYVAFIPEKQVAIVILAN
DHA-7AM390_27335_79KpBOP          ............................................................
DHA-7AM395_28070_58KpBOP          ............................................................
DHA-7AN663_28510_174KpBOP         ............................................................
                              acc
```

```
                                              α12
5GGW_1|Chains                           350          360

5GGW_1|Chains                           KNYPNPARVAAAWQILNALQ
DHA-15WGF99578.1_254KpBOP               KNYPNTERVKAAQAILSALE
DHA-1AVZ12228.1_302EBOC                 KNYPNTERVKAAQAILSALE
DHA-1QGU39370.1_323EBOC                 KNYPNTERVKAAQAILSALE
DHA-1QIE67107.1_279EBOC                 KNYPNTERVKAAQAILSALE
DHA-1QIF29352.1_349EBOC                 KNYPNTERVKAAQAILSALE
DHA-1QUP93355.1_50EBOC                  KNYPNTERVKAAQAILSALE
DHA-1UKB51966.1_271EBOC                 KNYPNTERVKAAQAILSALE
DHA-1UKB62076.1_255EBOC                 KNYPNTERVKAAQAILSALE
DHA-1UOY35142.1_6EBOC                   KNYPNTERVKAAQAILSALE
DHA-1UOY53746.1_188EBOC                 KNYPNTERVKAAQAILSALE
DHA-1URL72031.1_311EBOC                 KNYPNTERVKAAQAILSALE
DHA-1WAZ65759.1_143EBOC                 KNYPNTERVKAAQAILSALE
DHA-1WGL89115.1_5EBOC                   KNYPNTERVKAAQAILSALE
DHA-1WGZ52333.1_354EBOC                 KNYPNTERVKAAQAILSALE
DHA-1ULK46985.1_701KpBOC                KNYPNTERVKAAQAILSALE
DHA-1QLR69540.1_4269KpBOC               KNYPNTERVKAAQAILSALE
DHA-1UMU29130.1_701KpBOC                KNYPNTERVKAAQAILSALE
DHA-1QTO12878.1_5112KpBOC               KNYPNTERVKAAQAILSALE
DHA-1QVR56117.1_814KpBOC                KNYPNTERVKAAQAILSALE
DHA-1WBX47547.1_5024KpBOC               KNYPNTERVKAAQAILSALE
DHA-1USO45218.1_1502KpBOC               KNYPNTERVKAAQAILSALE
DHA-1UYH26771.1_3649KpBOC               KNYPNTERVKAAQAILSALE
DHA-1BCI98564.1_KP_B_O_P                KNYPNTERVKAAQAILSALE
DHA-1BBR80752.1_1091]KpBOP              KNYPNTERVKAAQAILSALE
DHA-1ARV42956.1_78KpBOP                 KNYPNTERVKAAQAILSALE
DHA-1QAW92788.1_234KpBOP                KNYPNTERVKAAQAILSALE
DHA-1AWE04508.1_77KpBOP                 KNYPNTERVKAAQAILSALE
DHA-1AWE05132.1_156KpBOP                KNYPNTERVKAAQAILSALE
DHA-1AXR54232.1_175KpBOP                KNYPNTERVKAAQAILSALE
DHA-1QBA36383.1_199KpBOP                KNYPNTERVKAAQAILSALE
DHA-1QIQ40896.1_277KpBOP                KNYPNTERVKAAQAILSALE
DHA-1QLB73441.1_45KpBOP                 KNYPNTERVKAAQAILSALE
DHA-1QLB78779.1_63KpBOP                 KNYPNTERVKAAQAILSALE
DHA-1QLB84106.1_44KpBOP                 KNYPNTERVKAAQAILSALE
DHA-1QLB89444.1_44KpBOP                 KNYPNTERVKAAQAILSALE
DHA-1QLB94784.1_45KpBOP                 KNYPNTERVKAAQAILSALE
DHA-1QLC10688.1_45KpBOP                 KNYPNTERVKAAQAILSALE
DHA-1QLC16028.1_45KpBOP                 KNYPNTERVKAAQAILSALE
DHA-1QIJ38370.1_76KpBOP                 KNYPNTERVKAAQAILSALE
DHA-1QIJ23273.1_76KpBOP                 KNYPNTERVKAAQAILSALE
DHA-1QIJ17670.1_76KpBOP                 KNYPNTERVKAAQAILSALE
DHA-1QYI39301.1_102KpBOP                KNYPNTERVKAAQAILSALE
DHA-1QER51199.1_205KpBOP                KNYPNTERVKAAQAILSALE
DHA-1QIF88318.1_264KpBOP                KNYPNTERVKAAQAILSALE
DHA-1QLI11710.1_48KpBOP                 KNYPNTERVKAAQAILSALE
DHA-1QLI17048.1_76KpBOP                 KNYPNTERVKAAQAILSALE
DHA-1QLI22544.1_1KpBOP                  KNYPNTERVKAAQAILSALE
DHA-1QLI33420.1_49KpBOP                 KNYPNTERVKAAQAILSALE
DHA-1ULI82297.1_104KpBOP                KNYPNTERVKAAQAILSALE
DHA-1ULK26611.1_20KpBOP                 KNYPNTERVKAAQAILSALE
DHA-1ULI14884.1_221KpBOP                KNYPNTERVKAAQAILSALE
DHA-1UMU88999.1_169KpBOP                KNYPNTERVKAAQAILSALE
DHA-1QLI93287.1_12KpBOP                 KNYPNTERVKAAQAILSALE
DHA-1QOW59325.1_216KpBOP                KNYPNTERVKAAQAILSALE
DHA-1QPP83812.1_78KpBOP                 KNYPNTERVKAAQAILSALE
DHA-1QPQ05032.1_31KpBOP                 KNYPNTERVKAAQAILSALE
DHA-1QQL36831.1_206KpBOP                KNYPNTERVKAAQAILSALE
DHA-1QRR62282.1_23KpBOP                 KNYPNTERVKAAQAILSALE
DHA-1QRR62336.1_13KpBOP                 KNYPNTERVKAAQAILSALE
DHA-1QRR62351.1_34KpBOP                 KNYPNTERVKAAQAILSALE
DHA-1QRR62310.1_56KpBOP                 KNYPNTERVKAAQAILSALE
DHA-1QRS73253.1_51KpBOP                 KNYPNTERVKAAQAILSALE
DHA-1QSC32078.1_75KpBOP                 KNYPNTERVKAAQAILSALE
DHA-1URL54267.1_52KpBOP                 KNYPNTERVKAAQAILSALE
DHA-1QSW33401.1_150KpBOP                KNYPNTERVKAAQAILSALE
DHA-1QTX39082.1_340KpBOP                KNYPNTERVKAAQAILSALE
DHA-1QUX17533.1_117KpBOP                KNYPNTERVKAAQAILSALE
DHA-1QVQ94431.1_179KpBOP                KNYPNTERVKAAQAILSALE
DHA-1QVR16641.1_60KpBOP                 KNYPNTERVKAAQAILSALE
DHA-1QVU34134.1_265KpBOP                KNYPNTERVKAAQAILSALE
DHA-1QVW15542.1_136KpBOP                KNYPNTERVKAAQAILSALE
DHA-1QVW15569.1_171KpBOP                KNYPNTERVKAAQAILSALE
DHA-1QXQ84174.1_24KpBOP                 KNYPNTERVKAAQAILSALE
DHA-1QXT85906.1_24KpBOP                 KNYPNTERVKAAQAILSALE
DHA-1QXU18235.1_24KpBOP                 KNYPNTERVKAAQAILSALE
DHA-1UNH49680.1_195KpBOP                KNYPNTERVKAAQAILSALE
DHA-1UGM94295.1_45KpBOP                 KNYPNTERVKAAQAILSALE
DHA-1UCM37138.1_188KpBOP                KNYPNTERVKAAQAILSALE
DHA-1UPF47572.1_185KpBOP                KNYPNTERVKAAQAILSALE
DHA-1UPF53088.1_98KpBOP                 KNYPNTERVKAAQAILSALE
DHA-1UNE95136.1_177KpBOP                KNYPNTERVKAAQAILSALE
DHA-1UGQ70597.1_19KpBOP                 KNYPNTERVKAAQAILSALE
DHA-1UGQ75588.1_15KpBOP                 KNYPNTERVKAAQAILSALE
DHA-1UGQ80838.1_30KpBOP                 KNYPNTERVKAAQAILSALE
DHA-1UGR08221.1_34KpBOP                 KNYPNTERVKAAQAILSALE
DHA-1UGR13826.1_19KpBOP                 KNYPNTERVKAAQAILSALE
DHA-1UGS19283.1_181KpBOP                KNYPNTERVKAAQAILSALE
DHA-1UII40113.1_155KpBOP                KNYPNTERVKAAQAILSALE
DHA-1UIY63185.1_63KpBOP                 KNYPNTERVKAAQAILSALE
DHA-1UZK63620.1_210KpBOP                KNYPNTERVKAAQAILSALE
DHA-1UPS70201.1_18KpBOP                 KNYPNTERVKAAQAILSALE
DHA-1WDO24504.1_185KpBOP                KNYPNTERVKAAQAILSALE
DHA-1URT78846.1_121KpBOP                KNYPNTERVKAAQAILSALE
DHA-1URT89155.1_166KpBOP                KNYPNTERVKAAQAILSALE
DHA-1URT89430.1_166KpBOP                KNYPNTERVKAAQAILSALE
DHA-1URT94913.1_167KpBOP                KNYPNTERVKAAQAILSALE
DHA-1UWZ32448.1_52KpBOP                 KNYPNTERVKAAQAILSALE
DHA-1UTW92771.1_126KpBOP                KNYPNTERVKAAQAILSALE
DHA-1UZW73111.1_23KpBOP                 KNYPNTERVKAAQAILSALE
DHA-1WEI85275.1_173KpBOP                KNYPNTERVKAAQAILSALE
DHA-1UVF97054.1_214KpBOP                KNYPNTERVKAAQAILSALE
DHA-1UVN34295.1_69KpBOP                 KNYPNTERVKAAQAILSALE
DHA-1UWB42605.1_210KpBOP                KNYPNTERVKAAQAILSALE
DHA-1UWX53529.1_18KpBOP                 KNYPNTERVKAAQAILSALE
DHA-1WII50269.1_174KpBOP                KNYPNTERVKAAQAILSALE
DHA-1WII50721.1_91KpBOP                 KNYPNTERVKAAQAILSALE
DHA-1UXY66557.1_128KpBOP                KNYPNTERVKAAQAILSALE
DHA-1UYX39647.1_199KpBOP                KNYPNTERVKAAQAILSALE
DHA-1UZE57593.1_95KpBOP                 KNYPNTERVKAAQAILSALE
DHA-1WAD38926.1_6KpBOP                  KNYPNTERVKAAQAILSALE
DHA-1WAD13598.1_210KpBOP                KNYPNTERVKAAQAILSALE
DHA-1WAD08359.1_184KpBOP                KNYPNTERVKAAQAILSALE
DHA-1WAD18885.1_185KpBOP                KNYPNTERVKAAQAILSALE
DHA-1WAD19356.1_183KpBOP                KNYPNTERVKAAQAILSALE
DHA-1WAD44078.1_264KpBOP                KNYPNTERVKAAQAILSALE
DHA-1WAD49440.1_195KpBOP                KNYPNTERVKAAQAILSALE
DHA-1WAD71205.1_194KpBOP                KNYPNTERVKAAQAILSALE
DHA-1WAD55056.1_244KpBOP                KNYPNTERVKAAQAILSALE
DHA-1WAD60732.1_211KpBOP                KNYPNTERVKAAQAILSALE
DHA-1WAD61281.1_278KpBOP                KNYPNTERVKAAQAILSALE
DHA-1WFF21460.1_31KpBOP                 KNYPNTERVKAAQAILSALE
DHA-1WHL16843.1_345KpBOP                KNYPNTERVKAAQAILSALE
DHA-1WP_004236386.1KpOC                 KNYPNTERVKAAQAILSALE
DHA-1WP_004236386.1_4280KpBCP           KNYPNTERVKAAQAILSALE
DHA-1WP_004236386.1_5142KpBCP           KNYPNTERVKAAQAILSALE
DHA-1WP_004236386.1_814KpBCP            KNYPNTERVKAAQAILSALE
DHA-1WP_004236386.1_821KpBCP            KNYPNTERVKAAQAILSALE
DHA-1WP_004236386.1_817KpBCP            KNYPNTERVKAAQAILSALE
DHA-1WP_004236386.1_700KpBCP            KNYPNTERVKAAQAILSALE
DHA-1USO45218.1_1502KpBCP               KNYPNTERVKAAQAILSALE
DHA-1WP_004236386.1_3654KpBCP           KNYPNTERVKAAQAILSALE
DHA-1WP_004236386.1_5030KpBCP           KNYPNTERVKAAQAILSALE
DHA-7AM390_27335_79KpBOP                ....................
DHA-7AM395_28070_58KpBOP                ....................
DHA-7AN663_28510_174KpBOP               ....................
                                  acc
```

MIR enzyme group

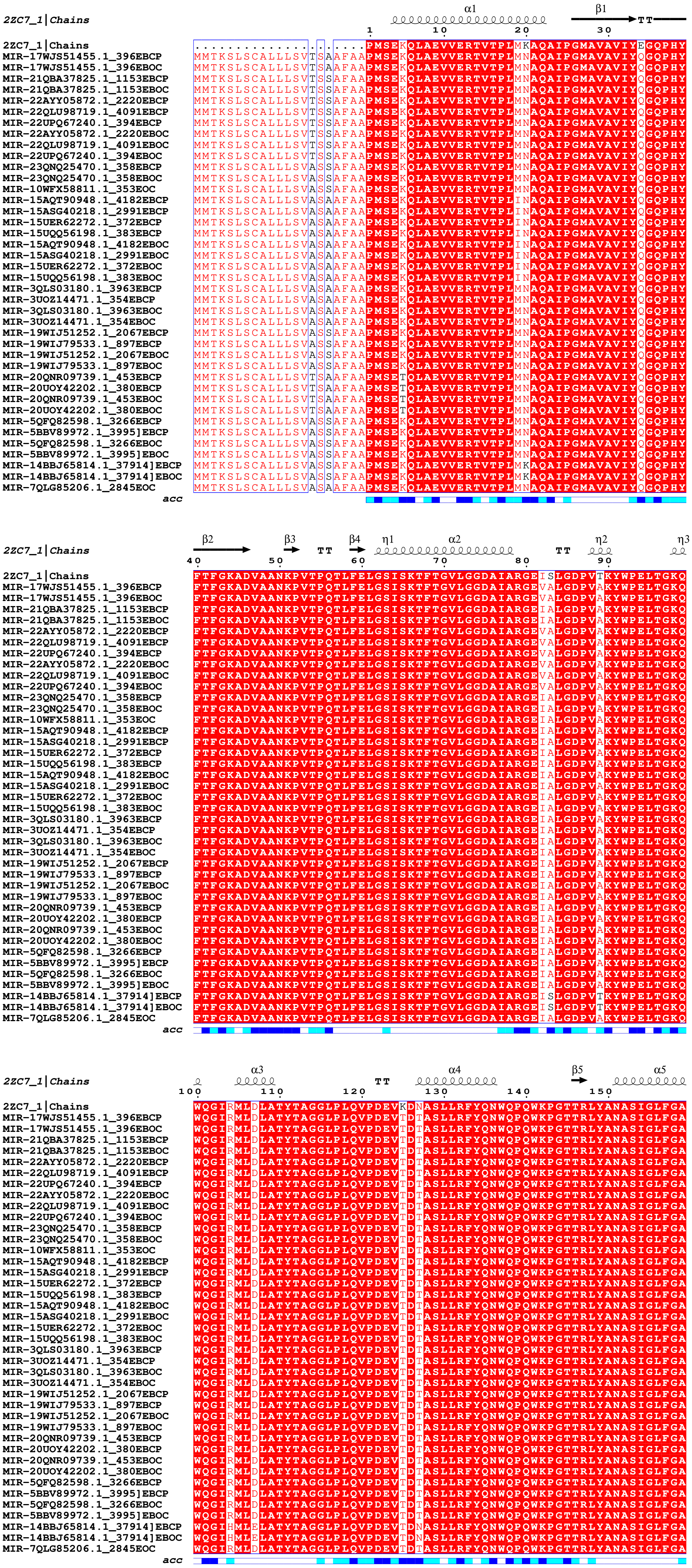

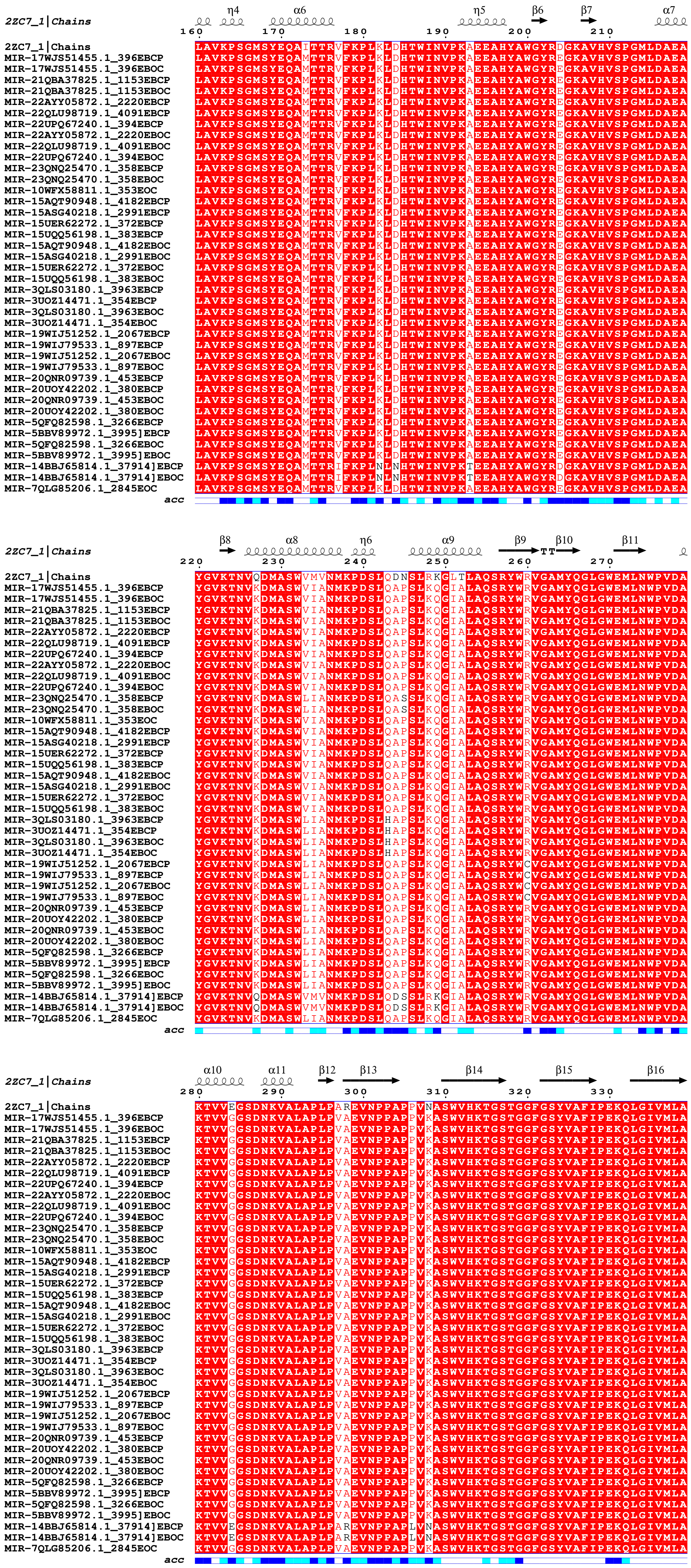
2ZC7_1|Chains
η4 α6 η5 β6 β7 α7
160 170 180 190 200 210
2ZC7_1|Chains LAVKPSGMSYEQAITTRVFKPLKLDHTWINVPKAEEAHYAWGYRDGKAVHVSPGMLDAEA
MIR-17WJS51455.1_396EBCP LAVKPSGMSYEQAMTTRVFKPLKLDHTWINVPKAEEAHYAWGYREGKAVHVSPGMLDAEA
MIR-17WJS51455.1_396EBOC LAVKPSGMSYEQAMTTRVFKPLKLDHTWINVPKAEEAHYAWGYREGKAVHVSPGMLDAEA
MIR-21QBA37825.1_1153EBCP LAVKPSGMSYEQAMTTRVFKPLKLDHTWINVPKAEEAHYAWGYREGKAVHVSPGMLDAEA
MIR-21QBA37825.1_1153EBOC LAVKPSGMSYEQAMTTRVFKPLKLDHTWINVPKAEEAHYAWGYREGKAVHVSPGMLDAEA
MIR-22AYY05872.1_2220EBCP LAVKPSGMSYEQAMTTRVFKPLKLDHTWINVPKAEEAHYAWGYREGKAVHVSPGMLDAEA
MIR-22QLU98719.1_4091EBCP LAVKPSGMSYEQAMTTRVFKPLKLDHTWINVPKAEEAHYAWGYREGKAVHVSPGMLDAEA
MIR-22UPQ67240.1_394EBCP LAVKPSGMSYEQAMTTRVFKPLKLDHTWINVPKAEEAHYAWGYREGKAVHVSPGMLDAEA
MIR-22AYY05872.1_2220EBOC LAVKPSGMSYEQAMTTRVFKPLKLDHTWINVPKAEEAHYAWGYREGKAVHVSPGMLDAEA
MIR-22QLU98719.1_4091EBOC LAVKPSGMSYEQAMTTRVFKPLKLDHTWINVPKAEEAHYAWGYREGKAVHVSPGMLDAEA
MIR-22UPQ67240.1_394EBOC LAVKPSGMSYEQAMTTRVFKPLKLDHTWINVPKAEEAHYAWGYREGKAVHVSPGMLDAEA
MIR-23QNQ25470.1_358EBCP LAVKPSGMSYEQAMTTRVFKPLKLDHTWINVPKAEEAHYAWGYREGKAVHVSPGMLDAEA
MIR-23QNQ25470.1_358EBOC LAVKPSGMSYEQAMTTRVFKPLKLDHTWINVPKAEEAHYAWGYREGKAVHVSPGMLDAEA
MIR-10WFX58811.1_353EOC LAVKPSGMSYEQAMTTRVFKPLKLDHTWINVPKAEEAHYAWGYREGKAVHVSPGMLDAEA
MIR-15AQT90948.1_4182EBCP LAVKPSGMSYEQAMTTRVFKPLKLDHTWINVPKAEEAHYAWGYREGKAVHVSPGMLDAEA
MIR-15ASG40218.1_2991EBCP LAVKPSGMSYEQAMTTRVFKPLKLDHTWINVPKAEEAHYAWGYREGKAVHVSPGMLDAEA
MIR-15UER62272.1_372EBCP LAVKPSGMSYEQAMTTRVFKPLKLDHTWINVPKAEEAHYAWGYREGKAVHVSPGMLDAEA
MIR-15UQQ56198.1_383EBCP LAVKPSGMSYEQAMTTRVFKPLKLDHTWINVPKAEEAHYAWGYREGKAVHVSPGMLDAEA
MIR-15AQT90948.1_4182EBOC LAVKPSGMSYEQAMTTRVFKPLKLDHTWINVPKAEEAHYAWGYREGKAVHVSPGMLDAEA
MIR-15ASG40218.1_2991EBOC LAVKPSGMSYEQAMTTRVFKPLKLDHTWINVPKAEEAHYAWGYREGKAVHVSPGMLDAEA
MIR-15UER62272.1_372EBOC LAVKPSGMSYEQAMTTRVFKPLKLDHTWINVPKAEEAHYAWGYREGKAVHVSPGMLDAEA
MIR-15UQQ56198.1_383EBOC LAVKPSGMSYEQAMTTRVFKPLKLDHTWINVPKAEEAHYAWGYREGKAVHVSPGMLDAEA
MIR-3QLS03180.1_3963EBCP LAVKPSGMSYEQAMTTRVFKPLKLDHTWINVPKAEEAHYAWGYREGKAVHVSPGMLDAEA
MIR-3UOZ14471.1_354EBCP LAVKPSGMSYEQAMTTRVFKPLKLDHTWINVPKAEEAHYAWGYREGKAVHVSPGMLDAEA
MIR-3QLS03180.1_3963EBOC LAVKPSGMSYEQAMTTRVFKPLKLDHTWINVPKAEEAHYAWGYREGKAVHVSPGMLDAEA
MIR-3UOZ14471.1_354EBOC LAVKPSGMSYEQAMTTRVFKPLKLDHTWINVPKAEEAHYAWGYREGKAVHVSPGMLDAEA
MIR-19WIJ51252.1_2067EBCP LAVKPSGMSYEQAMTTRVFKPLKLDHTWINVPKAEEAHYAWGYREGKAVHVSPGMLDAEA
MIR-19WIJ79533.1_897EBCP LAVKPSGMSYEQAMTTRVFKPLKLDHTWINVPKAEEAHYAWGYREGKAVHVSPGMLDAEA
MIR-19WIJ51252.1_2067EBOC LAVKPSGMSYEQAMTTRVFKPLKLDHTWINVPKAEEAHYAWGYREGKAVHVSPGMLDAEA
MIR-19WIJ79533.1_897EBOC LAVKPSGMSYEQAMTTRVFKPLKLDHTWINVPKAEEAHYAWGYREGKAVHVSPGMLDAEA
MIR-20QNR09739.1_453EBCP LAVKPSGMSYEQAMTTRVFKPLKLDHTWINVPKAEEAHYAWGYREGKAVHVSPGMLDAEA
MIR-20UOY42202.1_380EBCP LAVKPSGMSYEQAMTTRVFKPLKLDHTWINVPKAEEAHYAWGYREGKAVHVSPGMLDAEA
MIR-20QNR09739.1_453EBOC LAVKPSGMSYEQAMTTRVFKPLKLDHTWINVPKAEEAHYAWGYREGKAVHVSPGMLDAEA
MIR-20UOY42202.1_380EBOC LAVKPSGMSYEQAMTTRVFKPLKLDHTWINVPKAEEAHYAWGYREGKAVHVSPGMLDAEA
MIR-5QFQ82598.1_3266EBCP LAVKPSGMSYEQAMTTRVFKPLKLDHTWINVPKAEEAHYAWGYREGKAVHVSPGMLDAEA
MIR-5BBV89972.1_3995]EBCP LAVKPSGMSYEQAMTTRVFKPLKLDHTWINVPKAEEAHYAWGYREGKAVHVSPGMLDAEA
MIR-5QFQ82598.1_3266EBOC LAVKPSGMSYEQAMTTRVFKPLKLDHTWINVPKAEEAHYAWGYREGKAVHVSPGMLDAEA
MIR-5BBV89972.1_3995]EBOC LAVKPSGMSYEQAMTTRVFKPLKLDHTWINVPKAEEAHYAWGYREGKAVHVSPGMLDAEA
MIR-14BBJ65814.1_37914]EBCP LAVKPSGMSYEQAMTTRIFKPLNLNHTWINVPKTEEAHYAWGYRDGKAVHVSPGMLDAEA
MIR-14BBJ65814.1_37914]EBOC LAVKPSGMSYEQAMTTRIFKPLNLNHTWINVPKTEEAHYAWGYRDGKAVHVSPGMLDAEA
MIR-7QLG85206.1_2845EOC LAVKPSGMSYEQAMTTRVFKPLKLDHTWINVPKAEEAHYAWGYREGKAVHVSPGMLDAEA
acc
2ZC7_1|Chains
β8 α8 η6 α9 β9 TT β10 β11
220 230 240 250 260 270
2ZC7_1|Chains YGVKTNVQDMASWVMVNMKPDSLQDNSLRKGLTLAQSRYWRVGAMYQGLGWEMLNWPVDA
MIR-17WJS51455.1_396EBCP YGVKTNVKDMASWVIANMKPDSLQAPSLKQGIALAQSRYWRVGAMYQGLGWEMLNWPVDA
MIR-17WJS51455.1_396EBOC YGVKTNVKDMASWVIANMKPDSLQAPSLKQGIALAQSRYWRVGAMYQGLGWEMLNWPVDA
MIR-21QBA37825.1_1153EBCP YGVKTNVKDMASWVIANMKPDSLQAPSLKQGIALAQSRYWRVGAMYQGLGWEMLNWPVDA
MIR-21QBA37825.1_1153EBOC YGVKTNVKDMASWVIANMKPDSLQAPSLKQGIALAQSRYWRVGAMYQGLGWEMLNWPVDA
MIR-22AYY05872.1_2220EBCP YGVKTNVKDMASWVIANMKPDSLQAPSLKQGIALAQSRYWRVGAMYQGLGWEMLNWPVDA
MIR-22QLU98719.1_4091EBCP YGVKTNVKDMASWVIANMKPDSLQAPSLKQGIALAQSRYWRVGAMYQGLGWEMLNWPVDA
MIR-22UPQ67240.1_394EBCP YGVKTNVKDMASWVIANMKPDSLQAPSLKQGIALAQSRYWRVGAMYQGLGWEMLNWPVDA
MIR-22AYY05872.1_2220EBOC YGVKTNVKDMASWVIANMKPDSLQAPSLKQGIALAQSRYWRVGAMYQGLGWEMLNWPVDA
MIR-22QLU98719.1_4091EBOC YGVKTNVKDMASWVIANMKPDSLQAPSLKQGIALAQSRYWRVGAMYQGLGWEMLNWPVDA
MIR-22UPQ67240.1_394EBOC YGVKTNVKDMASWVIANMKPDSLQAPSLKQGIALAQSRYWRVGAMYQGLGWEMLNWPVDA
MIR-23QNQ25470.1_358EBCP YGVKTNVKDMASWLIANMKPDSLQASSLKQGIALAQSRYWRVGAMYQGLGWEMLNWPVDA
MIR-23QNQ25470.1_358EBOC YGVKTNVKDMASWLIANMKPDSLQASSLKQGIALAQSRYWRVGAMYQGLGWEMLNWPVDA
MIR-10WFX58811.1_353EOC YGVKTNVKDMASWVIANMKPDSLQAPSLKQGIALAQSRYWRVGAMYQGLGWEMLNWPVDA
MIR-15AQT90948.1_4182EBCP YGVKTNVKDMASWVIANMKPDSLQAPSLKQGIALAQSRYWRVGAMYQGLGWEMLNWPVDA
MIR-15ASG40218.1_2991EBCP YGVKTNVKDMASWVIANMKPDSLQAPSLKQGIALAQSRYWRVGAMYQGLGWEMLNWPVDA
MIR-15UER62272.1_372EBCP YGVKTNVKDMASWVIANMKPDSLQAPSLKQGIALAQSRYWRVGAMYQGLGWEMLNWPVDA
MIR-15UQQ56198.1_383EBCP YGVKTNVKDMASWVIANMKPDSLQAPSLKQGIALAQSRYWRVGAMYQGLGWEMLNWPVDA
MIR-15AQT90948.1_4182EBOC YGVKTNVKDMASWVIANMKPDSLQAPSLKQGIALAQSRYWRVGAMYQGLGWEMLNWPVDA
MIR-15ASG40218.1_2991EBOC YGVKTNVKDMASWVIANMKPDSLQAPSLKQGIALAQSRYWRVGAMYQGLGWEMLNWPVDA
MIR-15UER62272.1_372EBOC YGVKTNVKDMASWVIANMKPDSLQAPSLKQGIALAQSRYWRVGAMYQGLGWEMLNWPVDA
MIR-15UQQ56198.1_383EBOC YGVKTNVKDMASWVIANMKPDSLQAPSLKQGIALAQSRYWRVGAMYQGLGWEMLNWPVDA
MIR-3QLS03180.1_3963EBCP YGVKTNVKDMASWLIANMKPDSLHAPSLKQGIALAQSRYWRVGAMYQGLGWEMLNWPVDA
MIR-3UOZ14471.1_354EBCP YGVKTNVKDMASWLIANMKPDSLHAPSLKQGIALAQSRYWRVGAMYQGLGWEMLNWPVDA
MIR-3QLS03180.1_3963EBOC YGVKTNVKDMASWLIANMKPDSLHAPSLKQGIALAQSRYWRVGAMYQGLGWEMLNWPVDA
MIR-3UOZ14471.1_354EBOC YGVKTNVKDMASWLIANMKPDSLHAPSLKQGIALAQSRYWRVGAMYQGLGWEMLNWPVDA
MIR-19WIJ51252.1_2067EBCP YGVKTNVKDMASWLIANMKPDSLQAPSLKQGIALAQSRYWCVGAMYQGLGWEMLNWPVDA
MIR-19WIJ79533.1_897EBCP YGVKTNVKDMASWLIANMKPDSLQAPSLKQGIALAQSRYWCVGAMYQGLGWEMLNWPVDA
MIR-19WIJ51252.1_2067EBOC YGVKTNVKDMASWLIANMKPDSLQAPSLKQGIALAQSRYWCVGAMYQGLGWEMLNWPVDA
MIR-19WIJ79533.1_897EBOC YGVKTNVKDMASWLIANMKPDSLQAPSLKQGIALAQSRYWCVGAMYQGLGWEMLNWPVDA
MIR-20QNR09739.1_453EBCP YGVKTNVKDMASWLIANMKPDSLQAPSLKQGIALAQSRYWRVGAMYQGLGWEMLNWPVDA
MIR-20UOY42202.1_380EBCP YGVKTNVKDMASWLIANMKPDSLQAPSLKQGIALAQSRYWRVGAMYQGLGWEMLNWPVDA
MIR-20QNR09739.1_453EBOC YGVKTNVKDMASWLIANMKPDSLQAPSLKQGIALAQSRYWRVGAMYQGLGWEMLNWPVDA
MIR-20UOY42202.1_380EBOC YGVKTNVKDMASWLIANMKPDSLQAPSLKQGIALAQSRYWRVGAMYQGLGWEMLNWPVDA
MIR-5QFQ82598.1_3266EBCP YGVKTNVKDMASWLIANMKPDSLQAPSLKQGIALAQSRYWRVGAMYQGLGWEMLNWPVDA
MIR-5BBV89972.1_3995]EBCP YGVKTNVKDMASWLIANMKPDSLQAPSLKQGIALAQSRYWRVGAMYQGLGWEMLNWPVDA
MIR-5QFQ82598.1_3266EBOC YGVKTNVKDMASWLIANMKPDSLQAPSLKQGIALAQSRYWRVGAMYQGLGWEMLNWPVDA
MIR-5BBV89972.1_3995]EBOC YGVKTNVKDMASWLIANMKPDSLQAPSLKQGIALAQSRYWRVGAMYQGLGWEMLNWPVDA
MIR-14BBJ65814.1_37914]EBCP YGVKTNVQDMASWVMVNMKPDSLQDSSLRKGIALAQSRYWRVGAMYQGLGWEMLNWPVDA
MIR-14BBJ65814.1_37914]EBOC YGVKTNVQDMASWVMVNMKPDSLQDSSLRKGIALAQSRYWRVGAMYQGLGWEMLNWPVDA
MIR-7QLG85206.1_2845EOC YGVKTNVKDMASWLIANMKPDSLQAPSLKQGIALAQSRYWRVGAMYQGLGWEMLNWPVDA
acc
2ZC7_1|Chains
α10 α11 β12 β13 β14 β15 β16
280 290 300 310 320 330
2ZC7_1|Chains KTVVEGSDNKVALAPLPAREVNPPAPPVNASWVHKTGSTGGFGSYVAFIPEKQLGIVMLA
MIR-17WJS51455.1_396EBCP KTVVGGSDNKVALAPLPVAEVNPPAPPVKASWVHKTGSTGGFGSYVAFIPEKQLGIVMLA
MIR-17WJS51455.1_396EBOC KTVVGGSDNKVALAPLPVAEVNPPAPPVKASWVHKTGSTGGFGSYVAFIPEKQLGIVMLA
MIR-21QBA37825.1_1153EBCP KTVVGGSDNKVALAPLPVAEVNPPAPPVKASWVHKTGSTGGFGSYVAFIPEKQLGIVMLA
MIR-21QBA37825.1_1153EBOC KTVVGGSDNKVALAPLPVAEVNPPAPPVKASWVHKTGSTGGFGSYVAFIPEKQLGIVMLA
MIR-22AYY05872.1_2220EBCP KTVVGGSDNKVALAPLPVAEVNPPAPPVKASWVHKTGSTGGFGSYVAFIPEKQLGIVMLA
MIR-22QLU98719.1_4091EBCP KTVVGGSDNKVALAPLPVAEVNPPAPPVKASWVHKTGSTGGFGSYVAFIPEKQLGIVMLA
MIR-22UPQ67240.1_394EBCP KTVVGGSDNKVALAPLPVAEVNPPAPPVKASWVHKTGSTGGFGSYVAFIPEKQLGIVMLA
MIR-22AYY05872.1_2220EBOC KTVVGGSDNKVALAPLPVAEVNPPAPPVKASWVHKTGSTGGFGSYVAFIPEKQLGIVMLA
MIR-22QLU98719.1_4091EBOC KTVVGGSDNKVALAPLPVAEVNPPAPPVKASWVHKTGSTGGFGSYVAFIPEKQLGIVMLA
MIR-22UPQ67240.1_394EBOC KTVVGGSDNKVALAPLPVAEVNPPAPPVKASWVHKTGSTGGFGSYVAFIPEKQLGIVMLA
MIR-23QNQ25470.1_358EBCP KTVVGGSDNKVALAPLPVAEVNPPAPPVKASWVHKTGSTGGFGSYVAFIPEKQLGIVMLA
MIR-23QNQ25470.1_358EBOC KTVVGGSDNKVALAPLPVAEVNPPAPPVKASWVHKTGSTGGFGSYVAFIPEKQLGIVMLA
MIR-10WFX58811.1_353EOC KTVVGGSDNKVALAPLPVAEVNPPAPPVKASWVHKTGSTGGFGSYVAFIPEKQLGIVMLA
MIR-15AQT90948.1_4182EBCP KTVVGGSDNKVALAPLPVAEVNPPAPPVKASWVHKTGSTGGFGSYVAFIPEKQLGIVMLA
MIR-15ASG40218.1_2991EBCP KTVVGGSDNKVALAPLPVAEVNPPAPPVKASWVHKTGSTGGFGSYVAFIPEKQLGIVMLA
MIR-15UER62272.1_372EBCP KTVVGGSDNKVALAPLPVAEVNPPAPPVKASWVHKTGSTGGFGSYVAFIPEKQLGIVMLA
MIR-15UQQ56198.1_383EBCP KTVVGGSDNKVALAPLPVAEVNPPAPPVKASWVHKTGSTGGFGSYVAFIPEKQLGIVMLA
MIR-15AQT90948.1_4182EBOC KTVVGGSDNKVALAPLPVAEVNPPAPPVKASWVHKTGSTGGFGSYVAFIPEKQLGIVMLA
MIR-15ASG40218.1_2991EBOC KTVVGGSDNKVALAPLPVAEVNPPAPPVKASWVHKTGSTGGFGSYVAFIPEKQLGIVMLA
MIR-15UER62272.1_372EBOC KTVVGGSDNKVALAPLPVAEVNPPAPPVKASWVHKTGSTGGFGSYVAFIPEKQLGIVMLA
MIR-15UQQ56198.1_383EBOC KTVVGGSDNKVALAPLPVAEVNPPAPPVKASWVHKTGSTGGFGSYVAFIPEKQLGIVMLA
MIR-3QLS03180.1_3963EBCP KTVVGGSDNKVALAPLPVAEVNPPAPPVKASWVHKTGSTGGFGSYVAFIPEKQLGIVMLA
MIR-3UOZ14471.1_354EBCP KTVVGGSDNKVALAPLPVAEVNPPAPPVKASWVHKTGSTGGFGSYVAFIPEKQLGIVMLA
MIR-3QLS03180.1_3963EBOC KTVVGGSDNKVALAPLPVAEVNPPAPPVKASWVHKTGSTGGFGSYVAFIPEKQLGIVMLA
MIR-3UOZ14471.1_354EBOC KTVVGGSDNKVALAPLPVAEVNPPAPPVKASWVHKTGSTGGFGSYVAFIPEKQLGIVMLA
MIR-19WIJ51252.1_2067EBCP KTVVGGSDNKVALAPLPVAEVNPPAPPVKASWVHKTGSTGGFGSYVAFIPEKQLGIVMLA
MIR-19WIJ79533.1_897EBCP KTVVGGSDNKVALAPLPVAEVNPPAPPVKASWVHKTGSTGGFGSYVAFIPEKQLGIVMLA
MIR-19WIJ51252.1_2067EBOC KTVVGGSDNKVALAPLPVAEVNPPAPPVKASWVHKTGSTGGFGSYVAFIPEKQLGIVMLA
MIR-19WIJ79533.1_897EBOC KTVVGGSDNKVALAPLPVAEVNPPAPPVKASWVHKTGSTGGFGSYVAFIPEKQLGIVMLA
MIR-20QNR09739.1_453EBCP KTVVGGSDNKVALAPLPVAEVNPPAPPVKASWVHKTGSTGGFGSYVAFIPEKQLGIVMLA
MIR-20UOY42202.1_380EBCP KTVVGGSDNKVALAPLPVAEVNPPAPPVKASWVHKTGSTGGFGSYVAFIPEKQLGIVMLA
MIR-20QNR09739.1_453EBOC KTVVGGSDNKVALAPLPVAEVNPPAPPVKASWVHKTGSTGGFGSYVAFIPEKQLGIVMLA
MIR-20UOY42202.1_380EBOC KTVVGGSDNKVALAPLPVAEVNPPAPPVKASWVHKTGSTGGFGSYVAFIPEKQLGIVMLA
MIR-5QFQ82598.1_3266EBCP KTVVGGSDNKVALAPLPVAEVNPPAPPVKASWVHKTGSTGGFGSYVAFIPEKQLGIVMLA
MIR-5BBV89972.1_3995]EBCP KTVVGGSDNKVALAPLPVAEVNPPAPPVKASWVHKTGSTGGFGSYVAFIPEKQLGIVMLA
MIR-5QFQ82598.1_3266EBOC KTVVGGSDNKVALAPLPVAEVNPPAPPVKASWVHKTGSTGGFGSYVAFIPEKQLGIVMLA
MIR-5BBV89972.1_3995]EBOC KTVVGGSDNKVALAPLPVAEVNPPAPPVKASWVHKTGSTGGFGSYVAFIPEKQLGIVMLA
MIR-14BBJ65814.1_37914]EBCP KTVVEGSDNKVALAPLPAREVNPPAPLVNASWVHKTGSTGGFGSYVAFIPEKQLGIVMLA
MIR-14BBJ65814.1_37914]EBOC KTVVEGSDNKVALAPLPAREVNPPAPLVNASWVHKTGSTGGFGSYVAFIPEKQLGIVMLA
MIR-7QLG85206.1_2845EOC KTVVGGSDNKVALAPLPVAEVNPPAPPVKASWVHKTGSTGGFGSYVAFIPEKQLGIVMLA
acc

```
                                               α12
2ZC7_1|Chains                           ℓℓℓℓℓℓℓℓℓℓℓℓℓ
                                340       350
                                  .         .
2ZC7_1|Chains                   NKSYPNPARVEAAYRILSAL.
MIR-17WJS51455.1_396EBCP        NKSYPNPARVEAAYRILDALQ
MIR-17WJS51455.1_396EBOC        NKSYPNPARVEAAYRILDALQ
MIR-21QBA37825.1_1153EBCP       NKSYPNPARVEAAYRILDALQ
MIR-21QBA37825.1_1153EBOC       NKSYPNPARVEAAYRILDALQ
MIR-22AYY05872.1_2220EBCP       NKSYPNPARVEAAYRILDALQ
MIR-22QLU98719.1_4091EBCP       NKSYPNPARVEAAYRILDALQ
MIR-22UPQ67240.1_394EBCP        NKSYPNPARVEAAYRILDALQ
MIR-22AYY05872.1_2220EBOC       NKSYPNPARVEAAYRILDALQ
MIR-22QLU98719.1_4091EBOC       NKSYPNPARVEAAYRILDALQ
MIR-22UPQ67240.1_394EBOC        NKSYPNPARVEAAYRILDALQ
MIR-23QNQ25470.1_358EBCP        NKSYPNPARVEAAYRILDALQ
MIR-23QNQ25470.1_358EBOC        NKSYPNPARVEAAYRILDALQ
MIR-10WFX58811.1_353EOC         NKSYPNPARVEAAYRILDALQ
MIR-15AQT90948.1_4182EBCP       NKSYPNPARVEAAYRILDALQ
MIR-15ASG40218.1_2991EBCP       NKSYPNPARVEAAYRILDALQ
MIR-15UER62272.1_372EBCP        NKSYPNPARVEAAYRILDALQ
MIR-15UQQ56198.1_383EBCP        NKSYPNPARVEAAYRILDALQ
MIR-15AQT90948.1_4182EBOC       NKSYPNPARVEAAYRILDALQ
MIR-15ASG40218.1_2991EBOC       NKSYPNPARVEAAYRILDALQ
MIR-15UER62272.1_372EBOC        NKSYPNPARVEAAYRILDALQ
MIR-15UQQ56198.1_383EBOC        NKSYPNPARVEAAYRILDALQ
MIR-3QLS03180.1_3963EBCP        NKSYPNPARVEAAYRILDALQ
MIR-3UOZ14471.1_354EBCP         NKSYPNPARVEAAYRILDALQ
MIR-3QLS03180.1_3963EBOC        NKSYPNPARVEAAYRILDALQ
MIR-3UOZ14471.1_354EBOC         NKSYPNPARVEAAYRILDALQ
MIR-19WIJ51252.1_2067EBCP       NKSYPNPARVEAAYRILDALQ
MIR-19WIJ79533.1_897EBCP        NKSYPNPARVEAAYRILDALQ
MIR-19WIJ51252.1_2067EBOC       NKSYPNPARVEAAYRILDALQ
MIR-19WIJ79533.1_897EBOC        NKSYPNPARVEAAYRILDALQ
MIR-20QNR09739.1_453EBCP        NKSYPNPARVEAAYRILDALQ
MIR-20UOY42202.1_380EBCP        NKSYPNPARVEAAYRILDALQ
MIR-20QNR09739.1_453EBOC        NKSYPNPARVEAAYRILDALQ
MIR-20UOY42202.1_380EBOC        NKSYPNPARVEAAYRILDALQ
MIR-5QFQ82598.1_3266EBCP        NKSYPNPARVEAAYRILDALQ
MIR-5BBV89972.1_3995]EBCP       NKSYPNPARVEAAYRILDALQ
MIR-5QFQ82598.1_3266EBOC        NKSYPNPARVEAAYRILDALQ
MIR-5BBV89972.1_3995]EBOC       NKSYPNPARVEAAYRILDALQ
MIR-14BBJ65814.1_37914]EBCP     NKSYPNPARVEAAYRILDALQ
MIR-14BBJ65814.1_37914]EBOC     NKSYPNPARVEAAYRILDALQ
MIR-7QLG85206.1_2845EOC         NKSYPNPARVEAAYRILDALQ
                            acc
```

PDC enzyme group

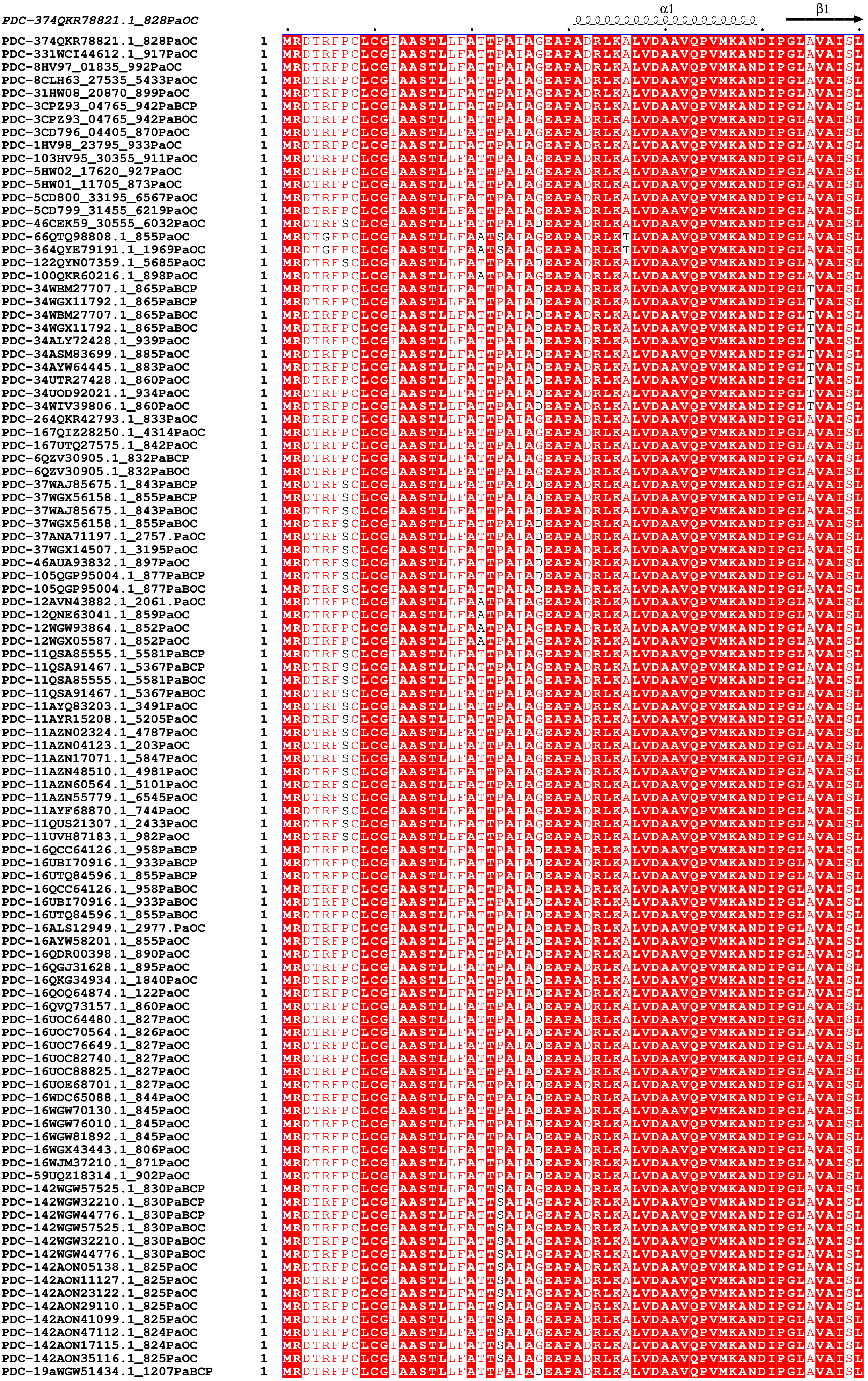

```
PDC-19aWGW37666.1_6817PaBCP     1   MRDTRFPCLCGIAASTLLFATTPAIADEAPADRLKALVDAAVQPVMKANDIPGLAVAISL
PDC-19aWGW59864.1_3660PaBCP     1   MRDTRFPCLCGIAASTLLFATTPAIADEAPADRLKALVDAAVQPVMKANDIPGLAVAISL
PDC-19aWGX49838.1_877PaBCP      1   MRDTRFPCLCGIAASTLLFATTPAIADEAPADRLKALVDAAVQPVMKANDIPGLAVAISL
PDC-19aWGX24308.1_877PaBCP      1   MRDTRFPCLCGIAASTLLFATTPAIADEAPADRLKALVDAAVQPVMKANDIPGLAVAISL
PDC-19aWGX35552.1_5487PaBCP     1   MRDTRFPCLCGIAASTLLFATTPAIADEAPADRLKALVDAAVQPVMKANDIPGLAVAISL
PDC-19aWKA37694.1_2446PaBCP     1   MRDTRFPCLCGIAASTLLFATTPAIADEAPADRLKALVDAAVQPVMKANDIPGLAVAISL
PDC-19aWGW51434.1_1207PaBOC     1   MRDTRFPCLCGIAASTLLFATTPAIADEAPADRLKALVDAAVQPVMKANDIPGLAVAISL
PDC-19aWGW37666.1_6817PaBOC     1   MRDTRFPCLCGIAASTLLFATTPAIADEAPADRLKALVDAAVQPVMKANDIPGLAVAISL
PDC-19aWGW59864.1_3660PaBOC     1   MRDTRFPCLCGIAASTLLFATTPAIADEAPADRLKALVDAAVQPVMKANDIPGLAVAISL
PDC-19aWGX49838.1_877PaBOC      1   MRDTRFPCLCGIAASTLLFATTPAIADEAPADRLKALVDAAVQPVMKANDIPGLAVAISL
PDC-19aWGX24308.1_877PaBOC      1   MRDTRFPCLCGIAASTLLFATTPAIADEAPADRLKALVDAAVQPVMKANDIPGLAVAISL
PDC-19aWGX35552.1_5487PaBOC     1   MRDTRFPCLCGIAASTLLFATTPAIADEAPADRLKALVDAAVQPVMKANDIPGLAVAISL
PDC-19aWKA37694.1_2446PaBOC     1   MRDTRFPCLCGIAASTLLFATTPAIADEAPADRLKALVDAAVQPVMKANDIPGLAVAISL
PDC-19aAPB63370.1_616PaOC       1   MRDTRFPCLCGIAASTLLFATTPAIADEAPADRLKALVDAAVQPVMKANDIPGLAVAISL
PDC-19aARI89529.1_870PaOC       1   MRDTRFPCLCGIAASTLLFATTPAIADEAPADRLKALVDAAVQPVMKANDIPGLAVAISL
PDC-19aARI95964.1_871PaOC       1   MRDTRFPCLCGIAASTLLFATTPAIADEAPADRLKALVDAAVQPVMKANDIPGLAVAISL
PDC-19aASA13594.1_977PaOC       1   MRDTRFPCLCGIAASTLLFATTPAIADEAPADRLKALVDAAVQPVMKANDIPGLAVAISL
PDC-19aASA27558.1_962PaOC       1   MRDTRFPCLCGIAASTLLFATTPAIADEAPADRLKALVDAAVQPVMKANDIPGLAVAISL
PDC-19aASD01901.1_971PaOC       1   MRDTRFPCLCGIAASTLLFATTPAIADEAPADRLKALVDAAVQPVMKANDIPGLAVAISL
PDC-19aUAC92160.1_908PaOC       1   MRDTRFPCLCGIAASTLLFATTPAIADEAPADRLKALVDAAVQPVMKANDIPGLAVAISL
PDC-19aUAC98839.1_942PaOC       1   MRDTRFPCLCGIAASTLLFATTPAIADEAPADRLKALVDAAVQPVMKANDIPGLAVAISL
PDC-19aUTQ33604.1_830PaOC       1   MRDTRFPCLCGIAASTLLFATTPAIADEAPADRLKALVDAAVQPVMKANDIPGLAVAISL
PDC-19aUOO58611.1_830PaOC       1   MRDTRFPCLCGIAASTLLFATTPAIADEAPADRLKALVDAAVQPVMKANDIPGLAVAISL
PDC-19aWDA50910.1_883PaOC       1   MRDTRFPCLCGIAASTLLFATTPAIADEAPADRLKALVDAAVQPVMKANDIPGLAVAISL
PDC-19aWGX30960.1_941PaOC       1   MRDTRFPCLCGIAASTLLFATTPAIADEAPADRLKALVDAAVQPVMKANDIPGLAVAISL
PDC-19aWGX61448.1_5603PaOC      1   MRDTRFPCLCGIAASTLLFATTPAIADEAPADRLKALVDAAVQPVMKANDIPGLAVAISL
PDC-19aWGX82118.1_941PaOC       1   MRDTRFPCLCGIAASTLLFATTPAIADEAPADRLKALVDAAVQPVMKANDIPGLAVAISL
PDC-19aWIO49783.1_187PaOC       1   MRDTRFPCLCGIAASTLLFATTPAIADEAPADRLKALVDAAVQPVMKANDIPGLAVAISL
PDC-24QZV43725.1_941PaBCP      1   MRDTRFPCLCGIAASTLLFAATPAIAGEAPADRLKALVDAAVQPVMKANDIPGLAVAISL
PDC-24QZV43725.1_941PaBOC      1   MRDTRFPCLCGIAASTLLFAATPAIAGEAPADRLKALVDAAVQPVMKANDIPGLAVAISL
PDC-24QDR06751.1_860PaOC       1   MRDTRFPCLCGIAASTLLFAATPAIAGEAPADRLKALVDAAVQPVMKANDIPGLAVAISL
PDC-24UGR36796.1_3412PaOC      1   MRDTRFPCLCGIAASTLLFAATPAIAGEAPADRLKALVDAAVQPVMKANDIPGLAVAISL
PDC-24UTQ39921.1_833PaOC       1   MRDTRFPCLCGIAASTLLFAATPAIAGEAPADRLKALVDAAVQPVMKANDIPGLAVAISL
PDC-137QKE94864.1_852PaOC      1   MRDTRFPCLCGIAASTLLLATTPAIAGEAPADRLKALVDAAVQPVMKANDIPGLAVAISL
PDC-147UGR30133.1_1950PaOC     1   MRDTRFPCLCGIAASTLLFATTPAIAGEAPADRLKALVDAAVQPVMKANDIPGLAVAISL
PDC-8AYY41152.1_5352PaBCP      1   MRDTRFPCLCGIAASTLLFATTPAIAGEAPADRLKALVDAAVQPVMKANDIPGLAVAISL
PDC-8QOE14337.1_211PaBCP       1   MRDTRFPCLCGIAASTLLFATTPAIAGEAPADRLKALVDAAVQPVMKANDIPGLAVAISL
PDC-8QPN15577.1_911PaBCP       1   MRDTRFPCLCGIAASTLLFATTPAIAGEAPADRLKALVDAAVQPVMKANDIPGLAVAISL
PDC-8QPN23993.1_908PaBCP       1   MRDTRFPCLCGIAASTLLFATTPAIAGEAPADRLKALVDAAVQPVMKANDIPGLAVAISL
PDC-8QUE64702.1_909PaBCP       1   MRDTRFPCLCGIAASTLLFATTPAIAGEAPADRLKALVDAAVQPVMKANDIPGLAVAISL
PDC-8QYA88773.1_2249PaBCP      1   MRDTRFPCLCGIAASTLLFATTPAIAGEAPADRLKALVDAAVQPVMKANDIPGLAVAISL
PDC-8QYF01720.1_5249PaBCP      1   MRDTRFPCLCGIAASTLLFATTPAIAGEAPADRLKALVDAAVQPVMKANDIPGLAVAISL
PDC-8QZU99504.1_908PaBCP       1   MRDTRFPCLCGIAASTLLFATTPAIAGEAPADRLKALVDAAVQPVMKANDIPGLAVAISL
PDC-8QZV05727.1_908PaBCP       1   MRDTRFPCLCGIAASTLLFATTPAIAGEAPADRLKALVDAAVQPVMKANDIPGLAVAISL
PDC-8QZV12028.1_910PaBCP       1   MRDTRFPCLCGIAASTLLFATTPAIAGEAPADRLKALVDAAVQPVMKANDIPGLAVAISL
PDC-8QZV18112.1_863PaBCP       1   MRDTRFPCLCGIAASTLLFATTPAIAGEAPADRLKALVDAAVQPVMKANDIPGLAVAISL
PDC-8QZV24387.1_912PaBCP       1   MRDTRFPCLCGIAASTLLFATTPAIAGEAPADRLKALVDAAVQPVMKANDIPGLAVAISL
PDC-8QZV68851.1_908PaBCP       1   MRDTRFPCLCGIAASTLLFATTPAIAGEAPADRLKALVDAAVQPVMKANDIPGLAVAISL
PDC-8QZV81338.1_870PaBCP       1   MRDTRFPCLCGIAASTLLFATTPAIAGEAPADRLKALVDAAVQPVMKANDIPGLAVAISL
PDC-8QZV87769.1_908PaBCP       1   MRDTRFPCLCGIAASTLLFATTPAIAGEAPADRLKALVDAAVQPVMKANDIPGLAVAISL
PDC-8QZV94048.1_908PaBCP       1   MRDTRFPCLCGIAASTLLFATTPAIAGEAPADRLKALVDAAVQPVMKANDIPGLAVAISL
PDC-8UXH53497.1_877PaBCP       1   MRDTRFPCLCGIAASTLLFATTPAIAGEAPADRLKALVDAAVQPVMKANDIPGLAVAISL
PDC-8UXH66521.1_877PaBCP       1   MRDTRFPCLCGIAASTLLFATTPAIAGEAPADRLKALVDAAVQPVMKANDIPGLAVAISL
PDC-8UXH70664.1_4987PaBCP      1   MRDTRFPCLCGIAASTLLFATTPAIAGEAPADRLKALVDAAVQPVMKANDIPGLAVAISL
PDC-8UUW81479.1_5273PaBCP      1   MRDTRFPCLCGIAASTLLFATTPAIAGEAPADRLKALVDAAVQPVMKANDIPGLAVAISL
PDC-8WDM07130.1_910PaBCP       1   MRDTRFPCLCGIAASTLLFATTPAIAGEAPADRLKALVDAAVQPVMKANDIPGLAVAISL
PDC-8WHM62936.1_909PaBCP       1   MRDTRFPCLCGIAASTLLFATTPAIAGEAPADRLKALVDAAVQPVMKANDIPGLAVAISL
PDC-8WHM69354.1_909PaBCP       1   MRDTRFPCLCGIAASTLLFATTPAIAGEAPADRLKALVDAAVQPVMKANDIPGLAVAISL
PDC-8WHM75768.1_909PaBCP       1   MRDTRFPCLCGIAASTLLFATTPAIAGEAPADRLKALVDAAVQPVMKANDIPGLAVAISL
PDC-8WHM84263.1_909PaBCP       1   MRDTRFPCLCGIAASTLLFATTPAIAGEAPADRLKALVDAAVQPVMKANDIPGLAVAISL
PDC-8AYY41152.1_5352PaBOC      1   MRDTRFPCLCGIAASTLLFATTPAIAGEAPADRLKALVDAAVQPVMKANDIPGLAVAISL
PDC-8QOE14337.1_211PaBOC       1   MRDTRFPCLCGIAASTLLFATTPAIAGEAPADRLKALVDAAVQPVMKANDIPGLAVAISL
PDC-8QPN15577.1_911PaBOC       1   MRDTRFPCLCGIAASTLLFATTPAIAGEAPADRLKALVDAAVQPVMKANDIPGLAVAISL
PDC-8QPN23993.1_908PaBOC       1   MRDTRFPCLCGIAASTLLFATTPAIAGEAPADRLKALVDAAVQPVMKANDIPGLAVAISL
PDC-8QUE64702.1_909PaBOC       1   MRDTRFPCLCGIAASTLLFATTPAIAGEAPADRLKALVDAAVQPVMKANDIPGLAVAISL
PDC-8QYA88773.1_2249PaBOC      1   MRDTRFPCLCGIAASTLLFATTPAIAGEAPADRLKALVDAAVQPVMKANDIPGLAVAISL
PDC-8QYF01720.1_5249PaBOC      1   MRDTRFPCLCGIAASTLLFATTPAIAGEAPADRLKALVDAAVQPVMKANDIPGLAVAISL
PDC-8QZU99504.1_908PaBOC       1   MRDTRFPCLCGIAASTLLFATTPAIAGEAPADRLKALVDAAVQPVMKANDIPGLAVAISL
PDC-8QZV05727.1_908PaBOC       1   MRDTRFPCLCGIAASTLLFATTPAIAGEAPADRLKALVDAAVQPVMKANDIPGLAVAISL
PDC-8QZV12028.1_910PaBOC       1   MRDTRFPCLCGIAASTLLFATTPAIAGEAPADRLKALVDAAVQPVMKANDIPGLAVAISL
PDC-8QZV18112.1_863PaBOC       1   MRDTRFPCLCGIAASTLLFATTPAIAGEAPADRLKALVDAAVQPVMKANDIPGLAVAISL
PDC-8QZV24387.1_912PaBOC       1   MRDTRFPCLCGIAASTLLFATTPAIAGEAPADRLKALVDAAVQPVMKANDIPGLAVAISL
PDC-8QZV68851.1_908PaBOC       1   MRDTRFPCLCGIAASTLLFATTPAIAGEAPADRLKALVDAAVQPVMKANDIPGLAVAISL
PDC-8QZV81338.1_870PaBOC       1   MRDTRFPCLCGIAASTLLFATTPAIAGEAPADRLKALVDAAVQPVMKANDIPGLAVAISL
PDC-8QZV87769.1_908PaBOC       1   MRDTRFPCLCGIAASTLLFATTPAIAGEAPADRLKALVDAAVQPVMKANDIPGLAVAISL
PDC-8QZV94048.1_908PaBOC       1   MRDTRFPCLCGIAASTLLFATTPAIAGEAPADRLKALVDAAVQPVMKANDIPGLAVAISL
PDC-8UXH53497.1_877PaBOC       1   MRDTRFPCLCGIAASTLLFATTPAIAGEAPADRLKALVDAAVQPVMKANDIPGLAVAISL
PDC-8UXH66521.1_877PaBOC       1   MRDTRFPCLCGIAASTLLFATTPAIAGEAPADRLKALVDAAVQPVMKANDIPGLAVAISL
PDC-8UXH70664.1_4987PaBOC      1   MRDTRFPCLCGIAASTLLFATTPAIAGEAPADRLKALVDAAVQPVMKANDIPGLAVAISL
PDC-8UUW81479.1_5273PaBOC      1   MRDTRFPCLCGIAASTLLFATTPAIAGEAPADRLKALVDAAVQPVMKANDIPGLAVAISL
PDC-8WDM07130.1_910PaBOC       1   MRDTRFPCLCGIAASTLLFATTPAIAGEAPADRLKALVDAAVQPVMKANDIPGLAVAISL
PDC-8WHM62936.1_909PaBOC       1   MRDTRFPCLCGIAASTLLFATTPAIAGEAPADRLKALVDAAVQPVMKANDIPGLAVAISL
PDC-8WHM69354.1_909PaBOC       1   MRDTRFPCLCGIAASTLLFATTPAIAGEAPADRLKALVDAAVQPVMKANDIPGLAVAISL
PDC-8WHM75768.1_909PaBOC       1   MRDTRFPCLCGIAASTLLFATTPAIAGEAPADRLKALVDAAVQPVMKANDIPGLAVAISL
PDC-8WHM84263.1_909PaBOC       1   MRDTRFPCLCGIAASTLLFATTPAIAGEAPADRLKALVDAAVQPVMKANDIPGLAVAISL
PDC-8AMA35328.1_919.PaOC       1   MRDTRFPCLCGIAASTLLFATTPAIAGEAPADRLKALVDAAVQPVMKANDIPGLAVAISL
PDC-8ANI06966.1_119PaOC        1   MRDTRFPCLCGIAASTLLFATTPAIAGEAPADRLKALVDAAVQPVMKANDIPGLAVAISL
PDC-8AYW70752.1_952PaOC        1   MRDTRFPCLCGIAASTLLFATTPAIAGEAPADRLKALVDAAVQPVMKANDIPGLAVAISL
PDC-8AYZ76972.1_2272PaOC       1   MRDTRFPCLCGIAASTLLFATTPAIAGEAPADRLKALVDAAVQPVMKANDIPGLAVAISL
PDC-8QKP85289.1_778PaOC        1   MRDTRFPCLCGIAASTLLFATTPAIAGEAPADRLKALVDAAVQPVMKANDIPGLAVAISL
PDC-8QKR30573.1_5806PaOC       1   MRDTRFPCLCGIAASTLLFATTPAIAGEAPADRLKALVDAAVQPVMKANDIPGLAVAISL
PDC-8QLF19879.1_939PaOC        1   MRDTRFPCLCGIAASTLLFATTPAIAGEAPADRLKALVDAAVQPVMKANDIPGLAVAISL
PDC-8QYE59452.1_5107PaOC       1   MRDTRFPCLCGIAASTLLFATTPAIAGEAPADRLKALVDAAVQPVMKANDIPGLAVAISL
PDC-8QZV98104.1_4956PaOC       1   MRDTRFPCLCGIAASTLLFATTPAIAGEAPADRLKALVDAAVQPVMKANDIPGLAVAISL
PDC-8UFK73541.1_1068PaOC       1   MRDTRFPCLCGIAASTLLFATTPAIAGEAPADRLKALVDAAVQPVMKANDIPGLAVAISL
PDC-8UGR33975.2_368PaOC        1   MRDTRFPCLCGIAASTLLFATTPAIAGEAPADRLKALVDAAVQPVMKANDIPGLAVAISL
PDC-8UTN26696.1_862PaOC        1   MRDTRFPCLCGIAASTLLFATTPAIAGEAPADRLKALVDAAVQPVMKANDIPGLAVAISL
PDC-8UTQ91314.1_910PaOC        1   MRDTRFPCLCGIAASTLLFATTPAIAGEAPADRLKALVDAAVQPVMKANDIPGLAVAISL
PDC-8UNK85299.1_868PaOC        1   MRDTRFPCLCGIAASTLLFATTPAIAGEAPADRLKALVDAAVQPVMKANDIPGLAVAISL
PDC-8UNK97662.1_866PaOC        1   MRDTRFPCLCGIAASTLLFATTPAIAGEAPADRLKALVDAAVQPVMKANDIPGLAVAISL
PDC-8USI83494.1_833PaOC        1   MRDTRFPCLCGIAASTLLFATTPAIAGEAPADRLKALVDAAVQPVMKANDIPGLAVAISL
PDC-8WAO16415.1_5521PaOC       1   MRDTRFPCLCGIAASTLLFATTPAIAGEAPADRLKALVDAAVQPVMKANDIPGLAVAISL
PDC-98QZD62144.1_4346PaBCP     1   MRDTRFPCLCGIAASTLLFATTPAIAGEAPADRLKALVDAAVQPVMKANDIPGLAVAISL
```

PDC-98QZH48390.1_4214PaBCP	1	MRDTRFPCLCGIAASTLLFATTPAIAGEAPADRLKALVDAAVQPVMKANDIPGLAVAISL
PDC-98QZD62144.1_4346PaBOC	1	MRDTRFPCLCGIAASTLLFATTPAIAGEAPADRLKALVDAAVQPVMKANDIPGLAVAISL
PDC-98QZH48390.1_4214PaBOC	1	MRDTRFPCLCGIAASTLLFATTPAIAGEAPADRLKALVDAAVQPVMKANDIPGLAVAISL
PDC-98ALP56563.1_1203.PaOC	1	MRDTRFPCLCGIAASTLLFATTPAIAGEAPADRLKALVDAAVQPVMKANDIPGLAVAISL
PDC-98UTL97623.1_830PaOC	1	MRDTRFPCLCGIAASTLLFATTPAIAGEAPADRLKALVDAAVQPVMKANDIPGLAVAISL
PDC-457UTN40061.1_863PaOC	1	MRDTRFPCLCGIAASTLLFAATPAIAGEAPADRLKALVDAAVQPVMKANDIPGLAVAISL
PDC-23UXI89283.1_830PaOC	1	MRDTRFPCLCGIAASTLLFATTPAIADEAPADRLKALVDAAVQPVMKANDIPGLAVAISL
PDC-245AUA75408.1_866PaOC	1	MRDTRFPCLCGIAASTLLFATTPAIADEAPADRLKALVDAAVQPVMKANDIPGLAVAISL
PDC-31QZW06655.1_840PaBCP	1	MRDTRFPCLCGIAASTLLFATTPAIAGEAPADRLKALVDAAVQPVMKANDIPGLAVAISL
PDC-31QZW06655.1_840PaBOC	1	MRDTRFPCLCGIAASTLLFATTPAIAGEAPADRLKALVDAAVQPVMKANDIPGLAVAISL
PDC-31AXN27248.1_931PaOC	1	MRDTRFPCLCGIAASTLLFATTPAIAGEAPADRLKALVDAAVQPVMKANDIPGLAVAISL
PDC-31QBC07904.1_3021PaOC	1	MRDTRFPCLCGIAASTLLFATTPAIAGEAPADRLKALVDAAVQPVMKANDIPGLAVAISL
PDC-31UFM90275.1_905PaOC	1	MRDTRFPCLCGIAASTLLFATTPAIAGEAPADRLKALVDAAVQPVMKANDIPGLAVAISL
PDC-31UFM98870.1_905PaOC	1	MRDTRFPCLCGIAASTLLFATTPAIAGEAPADRLKALVDAAVQPVMKANDIPGLAVAISL
PDC-31UNT25139.1_901PaOC	1	MRDTRFPCLCGIAASTLLFATTPAIAGEAPADRLKALVDAAVQPVMKANDIPGLAVAISL
PDC-31UWJ96607.1_4267PaOC	1	MRDTRFPCLCGIAASTLLFATTPAIAGEAPADRLKALVDAAVQPVMKANDIPGLAVAISL
PDC-31WGX88300.1_823PaOC	1	MRDTRFPCLCGIAASTLLFATTPAIAGEAPADRLKALVDAAVQPVMKANDIPGLAVAISL
PDC-31WHM08550.1_859PaOC	1	MRDTRFPCLCGIAASTLLFATTPAIAGEAPADRLKALVDAAVQPVMKANDIPGLAVAISL
PDC-39AYK21377.1_872PaOC	1	MRDTRFPCLCGIAASTLLFATTPAIAGEAPADRLKALVDAAVQPVMKANDIPGLAVAISL
PDC-39UJC21946.1_837PaOC	1	MRDTRFPCLCGIAASTLLFATTPAIAGEAPADRLKALVDAAVQPVMKANDIPGLAVAISL
PDC-30UPL39337.1_928PaBCP	1	MRDTRFPCLCGIAASTLLFAATPAIAGEAPADRLKALVDAAVQPVMKANDIPGLAVAISL
PDC-30UPL39337.1_928PaBOC	1	MRDTRFPCLCGIAASTLLFAATPAIAGEAPADRLKALVDAAVQPVMKANDIPGLAVAISL
PDC-30UHL95932.1_5029PaOC	1	MRDTRFPCLCGIAASTLLFAATPAIAGEAPADRLKALVDAAVQPVMKANDIPGLAVAISL
PDC-30UML99445.1_838PaOC	1	MRDTRFPCLCGIAASTLLFAATPAIAGEAPADRLKALVDAAVQPVMKANDIPGLAVAISL
PDC-80WAJ79202.1_935PaOC	1	MRDTRFPCLCGIAASTLLFATTPAIAGEAPADRLKALVDAAVQPVMKANDIPGLAVAISL
8SDL_1|Chain	1	MRDTRFPCLCGIAASTLLFATTPAIAGEAPADRLKALVDAAVQPVMKANDIPGLAVAISL
PDC-3UTN33167.1_589PaBCP	1	MRDTRFPCLCGIAASTLLFATTPAIAGEAPADRLKALVDAAVQPVMKANDIPGLAVAISL
PDC-3WCI38158.1_984PaBCP	1	MRDTRFPCLCGIAASTLLFATTPAIAGEAPADRLKALVDAAVQPVMKANDIPGLAVAISL
PDC-3WCI50909.1_867PaBCP	1	MRDTRFPCLCGIAASTLLFATTPAIAGEAPADRLKALVDAAVQPVMKANDIPGLAVAISL
PDC-3WCI90621.1_961PaBCP	1	MRDTRFPCLCGIAASTLLFATTPAIAGEAPADRLKALVDAAVQPVMKANDIPGLAVAISL
PDC-3WEO48793.1_832PaBCP	1	MRDTRFPCLCGIAASTLLFATTPAIAGEAPADRLKALVDAAVQPVMKANDIPGLAVAISL
PDC-3CAI9907424.1_19963]PaBCP	1	MRDTRFPCLCGIAASTLLFATTPAIAGEAPADRLKALVDAAVQPVMKANDIPGLAVAISL
PDC-3UTN33167.1_589PaBOC	1	MRDTRFPCLCGIAASTLLFATTPAIAGEAPADRLKALVDAAVQPVMKANDIPGLAVAISL
PDC-3WCI38158.1_984PaBOC	1	MRDTRFPCLCGIAASTLLFATTPAIAGEAPADRLKALVDAAVQPVMKANDIPGLAVAISL
PDC-3WCI50909.1_867PaBOC	1	MRDTRFPCLCGIAASTLLFATTPAIAGEAPADRLKALVDAAVQPVMKANDIPGLAVAISL
PDC-3WCI90621.1_961PaBOC	1	MRDTRFPCLCGIAASTLLFATTPAIAGEAPADRLKALVDAAVQPVMKANDIPGLAVAISL
PDC-3WEO48793.1_832PaBOC	1	MRDTRFPCLCGIAASTLLFATTPAIAGEAPADRLKALVDAAVQPVMKANDIPGLAVAISL
PDC-3CAI9907424.1_19963]PaBOC	1	MRDTRFPCLCGIAASTLLFATTPAIAGEAPADRLKALVDAAVQPVMKANDIPGLAVAISL
PDC-3AHA17954.1_839.PaOC	1	MRDTRFPCLCGIAASTLLFATTPAIAGEAPADRLKALVDAAVQPVMKANDIPGLAVAISL
PDC-3ALE46683.1_840.PaOC	1	MRDTRFPCLCGIAASTLLFATTPAIAGEAPADRLKALVDAAVQPVMKANDIPGLAVAISL
PDC-3ARC78082.1_863.PaOC	1	MRDTRFPCLCGIAASTLLFATTPAIAGEAPADRLKALVDAAVQPVMKANDIPGLAVAISL
PDC-3ARG53386.1_5885PaOC	1	MRDTRFPCLCGIAASTLLFATTPAIAGEAPADRLKALVDAAVQPVMKANDIPGLAVAISL
PDC-3WAC79950.1_4482PaOC	1	MRDTRFPCLCGIAASTLLFATTPAIAGEAPADRLKALVDAAVQPVMKANDIPGLAVAISL
PDC-3WAE22012.1_4863PaOC	1	MRDTRFPCLCGIAASTLLFATTPAIAGEAPADRLKALVDAAVQPVMKANDIPGLAVAISL
PDC-3WAE29433.1_777PaOC	1	MRDTRFPCLCGIAASTLLFATTPAIAGEAPADRLKALVDAAVQPVMKANDIPGLAVAISL
PDC-3WAW42145.1_847PaOC	1	MRDTRFPCLCGIAASTLLFATTPAIAGEAPADRLKALVDAAVQPVMKANDIPGLAVAISL
PDC-3WCI57477.1_911PaOC	1	MRDTRFPCLCGIAASTLLFATTPAIAGEAPADRLKALVDAAVQPVMKANDIPGLAVAISL
PDC-3WCI77402.1_900PaOC	1	MRDTRFPCLCGIAASTLLFATTPAIAGEAPADRLKALVDAAVQPVMKANDIPGLAVAISL
PDC-3WCI64002.1_913PaOC	1	MRDTRFPCLCGIAASTLLFATTPAIAGEAPADRLKALVDAAVQPVMKANDIPGLAVAISL
PDC-3WCI83994.1_905PaOC	1	MRDTRFPCLCGIAASTLLFATTPAIAGEAPADRLKALVDAAVQPVMKANDIPGLAVAISL
PDC-3WJM48814.1_842PaOC	1	MRDTRFPCLCGIAASTLLFATTPAIAGEAPADRLKALVDAAVQPVMKANDIPGLAVAISL
PDC-374ANP60834.1_3741PaBCP	1	MRDTRFPCLCGIAASTLLFATTPAIAGEAPADRLKALVDAAVQPVMKANDIPGLAVAISL
PDC-374AYZ86971.1_5950PaBCP	1	MRDTRFPCLCGIAASTLLFATTPAIAGEAPADRLKALVDAAVQPVMKANDIPGLAVAISL
PDC-374QZV75115.1_843PaBCP	1	MRDTRFPCLCGIAASTLLFATTPAIAGEAPADRLKALVDAAVQPVMKANDIPGLAVAISL
PDC-374QZW12711.1_843PaBCP	1	MRDTRFPCLCGIAASTLLFATTPAIAGEAPADRLKALVDAAVQPVMKANDIPGLAVAISL
PDC-374UEG06685.1_907PaBCP	1	MRDTRFPCLCGIAASTLLFATTPAIAGEAPADRLKALVDAAVQPVMKANDIPGLAVAISL
PDC-374UGW95802.1_984PaBCP	1	MRDTRFPCLCGIAASTLLFATTPAIAGEAPADRLKALVDAAVQPVMKANDIPGLAVAISL
PDC-374WGW26222.1_839PaBCP	1	MRDTRFPCLCGIAASTLLFATTPAIAGEAPADRLKALVDAAVQPVMKANDIPGLAVAISL
PDC-374ANP60834.1_3741PaBOC	1	MRDTRFPCLCGIAASTLLFATTPAIAGEAPADRLKALVDAAVQPVMKANDIPGLAVAISL
PDC-374AYZ86971.1_5950PaBOC	1	MRDTRFPCLCGIAASTLLFATTPAIAGEAPADRLKALVDAAVQPVMKANDIPGLAVAISL
PDC-374QZV75115.1_843PaBOC	1	MRDTRFPCLCGIAASTLLFATTPAIAGEAPADRLKALVDAAVQPVMKANDIPGLAVAISL
PDC-374QZW12711.1_843PaBOC	1	MRDTRFPCLCGIAASTLLFATTPAIAGEAPADRLKALVDAAVQPVMKANDIPGLAVAISL
PDC-374UEG06685.1_907PaBOC	1	MRDTRFPCLCGIAASTLLFATTPAIAGEAPADRLKALVDAAVQPVMKANDIPGLAVAISL
PDC-374UGW95802.1_984PaBOC	1	MRDTRFPCLCGIAASTLLFATTPAIAGEAPADRLKALVDAAVQPVMKANDIPGLAVAISL
PDC-374WGW26222.1_839PaBOC	1	MRDTRFPCLCGIAASTLLFATTPAIAGEAPADRLKALVDAAVQPVMKANDIPGLAVAISL
PDC-374AOP56366.1_851PaOC	1	MRDTRFPCLCGIAASTLLFATTPAIAGEAPADRLKALVDAAVQPVMKANDIPGLAVAISL
PDC-374AVE31445.1_873.PaOC	1	MRDTRFPCLCGIAASTLLFATTPAIAGEAPADRLKALVDAAVQPVMKANDIPGLAVAISL
PDC-374QAS71510.1_873PaOC	1	MRDTRFPCLCGIAASTLLFATTPAIAGEAPADRLKALVDAAVQPVMKANDIPGLAVAISL
PDC-374QDD38027.1_4463PaOC	1	MRDTRFPCLCGIAASTLLFATTPAIAGEAPADRLKALVDAAVQPVMKANDIPGLAVAISL
PDC-374QKF05339.1_5830PaOC	1	MRDTRFPCLCGIAASTLLFATTPAIAGEAPADRLKALVDAAVQPVMKANDIPGLAVAISL
PDC-374QKK83983.1_853PaOC	1	MRDTRFPCLCGIAASTLLFATTPAIAGEAPADRLKALVDAAVQPVMKANDIPGLAVAISL
PDC-374QKS41466.1_4199PaOC	1	MRDTRFPCLCGIAASTLLFATTPAIAGEAPADRLKALVDAAVQPVMKANDIPGLAVAISL
PDC-374QPZ80284.1_873PaOC	1	MRDTRFPCLCGIAASTLLFATTPAIAGEAPADRLKALVDAAVQPVMKANDIPGLAVAISL
PDC-374QQW07466.1_922PaOC	1	MRDTRFPCLCGIAASTLLFATTPAIAGEAPADRLKALVDAAVQPVMKANDIPGLAVAISL
PDC-374QZU93305.1_843PaOC	1	MRDTRFPCLCGIAASTLLFATTPAIAGEAPADRLKALVDAAVQPVMKANDIPGLAVAISL
PDC-374UAC85653.1_934PaOC	1	MRDTRFPCLCGIAASTLLFATTPAIAGEAPADRLKALVDAAVQPVMKANDIPGLAVAISL
PDC-374UGR20250.1_3485PaOC	1	MRDTRFPCLCGIAASTLLFATTPAIAGEAPADRLKALVDAAVQPVMKANDIPGLAVAISL
PDC-374UIU12727.1_851PaOC	1	MRDTRFPCLCGIAASTLLFATTPAIAGEAPADRLKALVDAAVQPVMKANDIPGLAVAISL
PDC-374UJC03215.1_956PaOC	1	MRDTRFPCLCGIAASTLLFATTPAIAGEAPADRLKALVDAAVQPVMKANDIPGLAVAISL
PDC-374UJC09425.1_959PaOC	1	MRDTRFPCLCGIAASTLLFATTPAIAGEAPADRLKALVDAAVQPVMKANDIPGLAVAISL
PDC-374UJF57976.1_829PaOC	1	MRDTRFPCLCGIAASTLLFATTPAIAGEAPADRLKALVDAAVQPVMKANDIPGLAVAISL
PDC-374UJF63613.1_829PaOC	1	MRDTRFPCLCGIAASTLLFATTPAIAGEAPADRLKALVDAAVQPVMKANDIPGLAVAISL
PDC-374UJF68041.1_5361PaOC	1	MRDTRFPCLCGIAASTLLFATTPAIAGEAPADRLKALVDAAVQPVMKANDIPGLAVAISL
PDC-374UJF74874.1_829PaOC	1	MRDTRFPCLCGIAASTLLFATTPAIAGEAPADRLKALVDAAVQPVMKANDIPGLAVAISL
PDC-374UJF80517.1_829PaOC	1	MRDTRFPCLCGIAASTLLFATTPAIAGEAPADRLKALVDAAVQPVMKANDIPGLAVAISL
PDC-374UJF86151.1_829PaOC	1	MRDTRFPCLCGIAASTLLFATTPAIAGEAPADRLKALVDAAVQPVMKANDIPGLAVAISL
PDC-374UJF90590.1_5359PaOC	1	MRDTRFPCLCGIAASTLLFATTPAIAGEAPADRLKALVDAAVQPVMKANDIPGLAVAISL
PDC-374UJF97432.1_830PaOC	1	MRDTRFPCLCGIAASTLLFATTPAIAGEAPADRLKALVDAAVQPVMKANDIPGLAVAISL
PDC-374UJG01877.1_5359PaOC	1	MRDTRFPCLCGIAASTLLFATTPAIAGEAPADRLKALVDAAVQPVMKANDIPGLAVAISL
PDC-374UJG08704.1_829PaOC	1	MRDTRFPCLCGIAASTLLFATTPAIAGEAPADRLKALVDAAVQPVMKANDIPGLAVAISL
PDC-374UJG14426.1_829PaOC	1	MRDTRFPCLCGIAASTLLFATTPAIAGEAPADRLKALVDAAVQPVMKANDIPGLAVAISL
PDC-374UJG20129.1_829PaOC	1	MRDTRFPCLCGIAASTLLFATTPAIAGEAPADRLKALVDAAVQPVMKANDIPGLAVAISL
PDC-374UJG25828.1_829PaOC	1	MRDTRFPCLCGIAASTLLFATTPAIAGEAPADRLKALVDAAVQPVMKANDIPGLAVAISL
PDC-374UJG31693.1_884PaOC	1	MRDTRFPCLCGIAASTLLFATTPAIAGEAPADRLKALVDAAVQPVMKANDIPGLAVAISL
PDC-374UJG36239.1_5358PaOC	1	MRDTRFPCLCGIAASTLLFATTPAIAGEAPADRLKALVDAAVQPVMKANDIPGLAVAISL
PDC-374UGR51117.1_68PaOC	1	MRDTRFPCLCGIAASTLLFATTPAIAGEAPADRLKALVDAAVQPVMKANDIPGLAVAISL
PDC-374UON75698.1_5570PaOC	1	MRDTRFPCLCGIAASTLLFATTPAIAGEAPADRLKALVDAAVQPVMKANDIPGLAVAISL
PDC-374UPG04116.1_3174PaOC	1	MRDTRFPCLCGIAASTLLFATTPAIAGEAPADRLKALVDAAVQPVMKANDIPGLAVAISL
PDC-374UPZ07602.1_2096PaOC	1	MRDTRFPCLCGIAASTLLFATTPAIAGEAPADRLKALVDAAVQPVMKANDIPGLAVAISL
PDC-374URM55875.1_5937PaOC	1	MRDTRFPCLCGIAASTLLFATTPAIAGEAPADRLKALVDAAVQPVMKANDIPGLAVAISL
PDC-374USV13808.1_3922PaOC	1	MRDTRFPCLCGIAASTLLFATTPAIAGEAPADRLKALVDAAVQPVMKANDIPGLAVAISL
PDC-374USV19196.1_4087PaOC	1	MRDTRFPCLCGIAASTLLFATTPAIAGEAPADRLKALVDAAVQPVMKANDIPGLAVAISL
PDC-374USX84542.1_851PaOC	1	MRDTRFPCLCGIAASTLLFATTPAIAGEAPADRLKALVDAAVQPVMKANDIPGLAVAISL

PDC-374USX78872.1_851PaOC	1	MRDTRFPCLCGIAASTLLFATTPAIAGEAPADRLKALVDAAVQPVMKANDIPGLAVAISL
PDC-374UUJ85605.1_1747PaOC	1	MRDTRFPCLCGIAASTLLFATTPAIAGEAPADRLKALVDAAVQPVMKANDIPGLAVAISL
PDC-374UVS71982.1_4220PaOC	1	MRDTRFPCLCGIAASTLLFATTPAIAGEAPADRLKALVDAAVQPVMKANDIPGLAVAISL
PDC-374UWI85046.1_4210PaOC	1	MRDTRFPCLCGIAASTLLFATTPAIAGEAPADRLKALVDAAVQPVMKANDIPGLAVAISL
PDC-374UWI92968.1_851PaOC	1	MRDTRFPCLCGIAASTLLFATTPAIAGEAPADRLKALVDAAVQPVMKANDIPGLAVAISL
PDC-374UXA22714.1_4203PaOC	1	MRDTRFPCLCGIAASTLLFATTPAIAGEAPADRLKALVDAAVQPVMKANDIPGLAVAISL
PDC-374UXA30656.1_926PaOC	1	MRDTRFPCLCGIAASTLLFATTPAIAGEAPADRLKALVDAAVQPVMKANDIPGLAVAISL
PDC-374UXA44988.1_926PaOC	1	MRDTRFPCLCGIAASTLLFATTPAIAGEAPADRLKALVDAAVQPVMKANDIPGLAVAISL
PDC-374UXN39470.1_4222PaOC	1	MRDTRFPCLCGIAASTLLFATTPAIAGEAPADRLKALVDAAVQPVMKANDIPGLAVAISL
PDC-374UZA87382.1_4980PaOC	1	MRDTRFPCLCGIAASTLLFATTPAIAGEAPADRLKALVDAAVQPVMKANDIPGLAVAISL
PDC-374WBM66401.1_846PaOC	1	MRDTRFPCLCGIAASTLLFATTPAIAGEAPADRLKALVDAAVQPVMKANDIPGLAVAISL
PDC-374WGJ75339.1_925PaOC	1	MRDTRFPCLCGIAASTLLFATTPAIAGEAPADRLKALVDAAVQPVMKANDIPGLAVAISL
PDC-374WGW87865.1_837PaOC	1	MRDTRFPCLCGIAASTLLFATTPAIAGEAPADRLKALVDAAVQPVMKANDIPGLAVAISL
PDC-103QES80695.1_910PaOC	1	MRDTRFPCLCGIAASTLLFATTPAIAGEAPADRLKALVDAAVQPVMKANDIPGLAVAISL
PDC-103QPV53905.1_6224PaOC	1	MRDTRFPCLCGIAASTLLFATTPAIAGEAPADRLKALVDAAVQPVMKANDIPGLAVAISL
PDC-35AVR81260.1_945.PaOC	1	MRDTRFPCLCGIAASTLLFATTPAIADEAPADRLKALVDAAVQPVMKANDIPGLAVAISL
PDC-35QJA35930.1_977PaOC	1	MRDTRFPCLCGIAASTLLFATTPAIADEAPADRLKALVDAAVQPVMKANDIPGLAVAISL
PDC-35AYZ46521.1_3210PaOC	1	MRDTRFPCLCGIAASTLLFATTPAIADEAPADRLKALVDAAVQPVMKANDIPGLAVAISL
PDC-35AYZ59004.1_3976PaOC	1	MRDTRFPCLCGIAASTLLFATTPAIADEAPADRLKALVDAAVQPVMKANDIPGLAVAISL
PDC-35QDR21260.1_3098PaOC	1	MRDTRFPCLCGIAASTLLFATTPAIADEAPADRLKALVDAAVQPVMKANDIPGLAVAISL
PDC-35QEK40749.1_862PaOC	1	MRDTRFPCLCGIAASTLLFATTPAIADEAPADRLKALVDAAVQPVMKANDIPGLAVAISL
PDC-35AUB00033.3_866PaOC	1	MRDTRFPCLCGIAASTLLFATTPAIADEAPADRLKALVDAAVQPVMKANDIPGLAVAISL
PDC-35QPP34775.1_1311PaOC	1	MRDTRFPCLCGIAASTLLFATTPAIADEAPADRLKALVDAAVQPVMKANDIPGLAVAISL
PDC-35QLF38648.1_891PaOC	1	MRDTRFPCLCGIAASTLLFATTPAIADEAPADRLKALVDAAVQPVMKANDIPGLAVAISL
PDC-35QLF14474.1_2055PaOC	1	MRDTRFPCLCGIAASTLLFATTPAIADEAPADRLKALVDAAVQPVMKANDIPGLAVAISL
PDC-35QQV60864.1_5728PaOC	1	MRDTRFPCLCGIAASTLLFATTPAIADEAPADRLKALVDAAVQPVMKANDIPGLAVAISL
PDC-35QQW01132.1_862PaOC	1	MRDTRFPCLCGIAASTLLFATTPAIADEAPADRLKALVDAAVQPVMKANDIPGLAVAISL
PDC-35QQW17992.1_5232PaOC	1	MRDTRFPCLCGIAASTLLFATTPAIADEAPADRLKALVDAAVQPVMKANDIPGLAVAISL
PDC-35QQW23330.1_862PaOC	1	MRDTRFPCLCGIAASTLLFATTPAIADEAPADRLKALVDAAVQPVMKANDIPGLAVAISL
PDC-35QZE42332.1_862PaOC	1	MRDTRFPCLCGIAASTLLFATTPAIADEAPADRLKALVDAAVQPVMKANDIPGLAVAISL
PDC-35UFM83790.1_826PaOC	1	MRDTRFPCLCGIAASTLLFATTPAIADEAPADRLKALVDAAVQPVMKANDIPGLAVAISL
PDC-35UJC15714.1_892PaOC	1	MRDTRFPCLCGIAASTLLFATTPAIADEAPADRLKALVDAAVQPVMKANDIPGLAVAISL
PDC-35UTR21121.1_931PaOC	1	MRDTRFPCLCGIAASTLLFATTPAIADEAPADRLKALVDAAVQPVMKANDIPGLAVAISL
PDC-35UUP09555.1_853PaOC	1	MRDTRFPCLCGIAASTLLFATTPAIADEAPADRLKALVDAAVQPVMKANDIPGLAVAISL
PDC-35UWG56199.1_885PaOC	1	MRDTRFPCLCGIAASTLLFATTPAIADEAPADRLKALVDAAVQPVMKANDIPGLAVAISL
PDC-43UNT30829.1_824PaOC	1	MRDTRFPCLCGIAASTLLFATTPAIAGEAPADRLKALVDAAVQPVMKANDIPGLAVAISL
PDC-45ALZ89573.1_855.PaOC	1	MRDTRFPCLCGIAASTLLFATTPAIAGEAPADRLKALVDAAVQPVMKANDIPGLAVAISL
PDC-45QKL11315.1_849PaOC	1	MRDTRFPCLCGIAASTLLFATTPAIAGEAPADRLKALVDAAVQPVMKANDIPGLAVAISL
PDC-45QQV94504.1_850PaOC	1	MRDTRFPCLCGIAASTLLFATTPAIAGEAPADRLKALVDAAVQPVMKANDIPGLAVAISL
PDC-162UJB98759.1_2776PaOC	1	MRDTRFPCLCGIAASTLLFATTPAIAGEAPADRLKALVDAAVQPVMKANDIPGLAVAISL
PDC-123QYE90948.1_3103PaOC	1	MRDTRFPCLCGIAASTLLFATTPAIADEAPADRLKALVDAAVQPVMKANDIPGLAVAISL
PDC-5UXH60990.1_2046PaBCP	1	MRDTRFPCLCGIAASTLLFATTPAIAGEAPADRLKALVDAAVQPVMKANDIPGLAVAISL
PDC-5WBA37364.1_905PaBCP	1	MRDTRFPCLCGIAASTLLFATTPAIAGEAPADRLKALVDAAVQPVMKANDIPGLAVAISL
PDC-5WGJ68312.1_842PaBCP	1	MRDTRFPCLCGIAASTLLFATTPAIAGEAPADRLKALVDAAVQPVMKANDIPGLAVAISL
PDC-5WGT14845.1_882PaBCP	1	MRDTRFPCLCGIAASTLLFATTPAIAGEAPADRLKALVDAAVQPVMKANDIPGLAVAISL
PDC-5WGX69730.1_872PaBCP	1	MRDTRFPCLCGIAASTLLFATTPAIAGEAPADRLKALVDAAVQPVMKANDIPGLAVAISL
PDC-5CAI9893238.1_8405]PaBCP	1	MRDTRFPCLCGIAASTLLFATTPAIAGEAPADRLKALVDAAVQPVMKANDIPGLAVAISL
PDC-5CAI9905252.1_60335]PaBCP	1	MRDTRFPCLCGIAASTLLFATTPAIAGEAPADRLKALVDAAVQPVMKANDIPGLAVAISL
PDC-5UXH60990.1_2046PaBOC	1	MRDTRFPCLCGIAASTLLFATTPAIAGEAPADRLKALVDAAVQPVMKANDIPGLAVAISL
PDC-5WBA37364.1_905PaBOC	1	MRDTRFPCLCGIAASTLLFATTPAIAGEAPADRLKALVDAAVQPVMKANDIPGLAVAISL
PDC-5WGJ68312.1_842PaBOC	1	MRDTRFPCLCGIAASTLLFATTPAIAGEAPADRLKALVDAAVQPVMKANDIPGLAVAISL
PDC-5WGT14845.1_882PaBOC	1	MRDTRFPCLCGIAASTLLFATTPAIAGEAPADRLKALVDAAVQPVMKANDIPGLAVAISL
PDC-5WGX69730.1_872PaBOC	1	MRDTRFPCLCGIAASTLLFATTPAIAGEAPADRLKALVDAAVQPVMKANDIPGLAVAISL
PDC-5CAI9893238.1_8405]PaBOC	1	MRDTRFPCLCGIAASTLLFATTPAIAGEAPADRLKALVDAAVQPVMKANDIPGLAVAISL
PDC-5CAI9905252.1_60335]PaBOC	1	MRDTRFPCLCGIAASTLLFATTPAIAGEAPADRLKALVDAAVQPVMKANDIPGLAVAISL
PDC-5ALU48309.1_2211.PaOC	1	MRDTRFPCLCGIAASTLLFATTPAIAGEAPADRLKALVDAAVQPVMKANDIPGLAVAISL
PDC-5AMX89317.1_4085.PaOC	1	MRDTRFPCLCGIAASTLLFATTPAIAGEAPADRLKALVDAAVQPVMKANDIPGLAVAISL
PDC-5ATH01452.1_827PaOC	1	MRDTRFPCLCGIAASTLLFATTPAIAGEAPADRLKALVDAAVQPVMKANDIPGLAVAISL
PDC-5UTN13111.1_830PaOC	1	MRDTRFPCLCGIAASTLLFATTPAIAGEAPADRLKALVDAAVQPVMKANDIPGLAVAISL
PDC-5UUH88148.1_851PaOC	1	MRDTRFPCLCGIAASTLLFATTPAIAGEAPADRLKALVDAAVQPVMKANDIPGLAVAISL
PDC-5UUH98459.1_806PaOC	1	MRDTRFPCLCGIAASTLLFATTPAIAGEAPADRLKALVDAAVQPVMKANDIPGLAVAISL
PDC-5UVH93148.1_837PaOC	1	MRDTRFPCLCGIAASTLLFATTPAIAGEAPADRLKALVDAAVQPVMKANDIPGLAVAISL
PDC-5UWU62523.1_171PaOC	1	MRDTRFPCLCGIAASTLLFATTPAIAGEAPADRLKALVDAAVQPVMKANDIPGLAVAISL
PDC-5WDZ01346.1_910PaOC	1	MRDTRFPCLCGIAASTLLFATTPAIAGEAPADRLKALVDAAVQPVMKANDIPGLAVAISL
PDC-5WEO42644.1_846PaOC	1	MRDTRFPCLCGIAASTLLFATTPAIAGEAPADRLKALVDAAVQPVMKANDIPGLAVAISL
PDC-5WGW99708.1_849PaOC	1	MRDTRFPCLCGIAASTLLFATTPAIAGEAPADRLKALVDAAVQPVMKANDIPGLAVAISL
PDC-5WJM42992.1_848PaOC	1	MRDTRFPCLCGIAASTLLFATTPAIAGEAPADRLKALVDAAVQPVMKANDIPGLAVAISL
PDC-60QWY08308.1_854PaBCP	1	MRDTRFPCLCGIAASTLLFATTPAIAGEAPADRLKALVDAAVQPVMKANDIPGLAVAISL
PDC-60QZV50082.1_838PaBCP	1	MRDTRFPCLCGIAASTLLFATTPAIAGEAPADRLKALVDAAVQPVMKANDIPGLAVAISL
PDC-60QWY08308.1_854PaBOC	1	MRDTRFPCLCGIAASTLLFATTPAIAGEAPADRLKALVDAAVQPVMKANDIPGLAVAISL
PDC-60QZV50082.1_838PaBOC	1	MRDTRFPCLCGIAASTLLFATTPAIAGEAPADRLKALVDAAVQPVMKANDIPGLAVAISL
PDC-60QPZ60674.1_902PaOC	1	MRDTRFPCLCGIAASTLLFATTPAIAGEAPADRLKALVDAAVQPVMKANDIPGLAVAISL
PDC-60WAJ92260.1_847PaOC	1	MRDTRFPCLCGIAASTLLFATTPAIAGEAPADRLKALVDAAVQPVMKANDIPGLAVAISL
PDC-60WAJ98065.1_847PaOC	1	MRDTRFPCLCGIAASTLLFATTPAIAGEAPADRLKALVDAAVQPVMKANDIPGLAVAISL
PDC-15UNL10022.1_837PaBCP	1	MRDTRFPCLCGIAASTLLFATTPAIAGEAPADRLKALVDAAVQPVMKANDIPGLAVAISL
PDC-15UNL10022.1_837PaBOC	1	MRDTRFPCLCGIAASTLLFATTPAIAGEAPADRLKALVDAAVQPVMKANDIPGLAVAISL
PDC-15AOT38867.1_3370PaOC	1	MRDTRFPCLCGIAASTLLFATTPAIAGEAPADRLKALVDAAVQPVMKANDIPGLAVAISL
PDC-15ALZ27705.1_859PaOC	1	MRDTRFPCLCGIAASTLLFATTPAIAGEAPADRLKALVDAAVQPVMKANDIPGLAVAISL
PDC-15AXC19915.1_1529PaOC	1	MRDTRFPCLCGIAASTLLFATTPAIAGEAPADRLKALVDAAVQPVMKANDIPGLAVAISL
PDC-15UTP69334.1_895PaOC	1	MRDTRFPCLCGIAASTLLFATTPAIAGEAPADRLKALVDAAVQPVMKANDIPGLAVAISL
PDC-15UNK91447.1_837PaOC	1	MRDTRFPCLCGIAASTLLFATTPAIAGEAPADRLKALVDAAVQPVMKANDIPGLAVAISL
PDC-15UNL03855.1_837PaOC	1	MRDTRFPCLCGIAASTLLFATTPAIAGEAPADRLKALVDAAVQPVMKANDIPGLAVAISL
PDC-28UYM61272.1_6539PaOC	1	MRDTRFPCLCGIAASTLLLATTPAIAGEAPADRLKALVDAAVQPVMKANDIPGLAVAISL
PDC-22QPZ72804.1_848PaOC	1	MRDTRFPCLCGIAASTLLLATTPAIAGEAPADRLKALVDAAVQPVMKANDIPGLAVAISL
PDC-22QQM08170.1_4816PaOC	1	MRDTRFPCLCGIAASTLLLATTPAIAGEAPADRLKALVDAAVQPVMKANDIPGLAVAISL
PDC-97QKR35301.1_4456PaOC	1	MRDTRFPCLCGIAASTLLFATTPAIAGEAPADRLKALVDAAVQPVMKANDIPGLAVAISL
PDC-51WGV68048.1_897PaOC	1	MRDTRFPCLCGIAASTLLFATTPAIAGEAPADRLKALVDAAVQPVMKANDIPGLAVAISL
PDC-1HW06_09835_867PaOC	1	MRDTRFPCLCGIAASTLLFATTPAIAGEAPADRLKALVDAAVQPVMKANDIPGLAVAISL
PDC-151QQV49515.1_805PaOC	1	MRHATILNLCGLAASTLFFATTSAFATEAPAERLKALVDAAVQPVMKANDIPGLAVAITL
consensus>70		MRdtrfpcLCGiAASTLlfAtTpAiAgEAPA#RLKaLVDAAVQPVMKANDIPGLaVAIsL
acc		

```
PDC-374QKR78821.1_828PaOC          TT  β2  β3  TT  β4  η1  α2  TT  η2

PDC-374QKR78821.1_828PaOC      61  KGEPHYFSYGLASKEDGRRVTPETLFEIGSVSKTFTATLAGYALTQDKMRLDDRASQHWP
PDC-331WCI44612.1_917PaOC      61  KGEPHYFSYGLASKEDGRRVTPETLFEIGSVSKTFTATLAGYALAQDKMRLDDRASQHWP
PDC-8HV97_01835_992PaOC        61  KGEPHYFSYGLASKEDGRRVTPETLFEIGSVSKTFTATLAGYALAQDKMRLDDRASQHWP
PDC-8CLH63_27535_5433PaOC      61  KGEPHYFSYGLASKEDGRRVTPETLFEIGSVSKTFTATLAGYALAQDKMRLDDRASQHWP
PDC-31HW08_20870_899PaOC       61  KGEPHYFSYGLASKEDGRRVTPETLFEIGSVSKTFTATLAGYALAQDKMRLDDRASQHWP
PDC-3CPZ93_04765_942PaBCP      61  KGEPHYFSYGLASKEDGRRVTPETLFEIGSVSKTFTATLAGYALAQDKMRLDDRASQHWP
PDC-3CPZ93_04765_942PaBOC      61  KGEPHYFSYGLASKEDGRRVTPETLFEIGSVSKTFTATLAGYALAQDKMRLDDRASQHWP
PDC-3CD796_04405_870PaOC       61  KGEPHYFSYGLASKEDGRRVTPETLFEIGSVSKTFTATLAGYALAQDKMRLDDRASQHWP
PDC-1HV98_23795_933PaOC        61  KGEPHYFSYGLASKEDGRRVTPETLFEIGSVSKTFTATLAGYALTQDKMRLDDRASQHWP
PDC-103HV95_30355_911PaOC      61  KGEPHYFSYGLASKEDGRRVTLETLFEIGSVSKTFTATLAGYALAQDKMRLDDRASQHWP
PDC-5HW02_17620_927PaOC        61  KGEPHYFSYGLASKEDGRQVTPETLFEIGSVSKTFTATLAGYALAQDKMRLDDRASQHWP
PDC-5HW01_11705_873PaOC        61  KGEPHYFSYGLASKEDGRQVTPETLFEIGSVSKTFTATLAGYALAQDKMRLDDRASQHWP
PDC-5CD800_33195_6567PaOC      61  KGEPHYFSYGLASKEDGRQVTPETLFEIGSVSKTFTATLAGYALAQDKMRLDDRASQHWP
PDC-5CD799_31455_6219PaOC      61  KGEPHYFSYGLASKEDGRQVTPETLFEIGSVSKTFTATLAGYALAQDKMRLDDRASQHWP
PDC-46CEK59_30555_6032PaOC     61  KGEPHYFSYGLASKEDGRRVTPETLFEIGSVSKTFTATLAGYALAQDKMRLDDRASQHWP
PDC-66QTQ98808.1_855PaOC       61  KGEPHYFSYGLASKEDGRRVTPETLFEIGSVSKTFTATLAGYALAQDKMRLDDRASLHWP
PDC-364QYE79191.1_1969PaOC     61  KGEPHYFSYGLASKEDGRRVTPETLFEIGSVSKSFTATLAGYALAQDKMRLDDRASLHWP
PDC-122QYN07359.1_5685PaOC     61  KGEPHYFSYGLASKEDGRRVTPETLFEIGSVSKTFTATLAGYALAQDKMRLDDRASQHWP
PDC-100QKR60216.1_898PaOC      61  KGEPHYFSYGLASKEDGRRVTPETLFEIGSVSKTFTATLAGYALAQDKMRLDDRASQHWP
PDC-34WBM27707.1_865PaBCP      61  KGEPHYFSYGLASKEDGRRVTPETLFEIGSVSKTFTATLAGYALAQDKMRLDDRASQHWP
PDC-34WGX11792.1_865PaBCP      61  KGEPHYFSYGLASKEDGRRVTPETLFEIGSVSKTFTATLAGYALAQDKMRLDDRASQHWP
PDC-34WBM27707.1_865PaBOC      61  KGEPHYFSYGLASKEDGRRVTPETLFEIGSVSKTFTATLAGYALAQDKMRLDDRASQHWP
PDC-34WGX11792.1_865PaBOC      61  KGEPHYFSYGLASKEDGRRVTPETLFEIGSVSKTFTATLAGYALAQDKMRLDDRASQHWP
PDC-34ALY72428.1_939PaOC       61  KGEPHYFSYGLASKEDGRRVTPETLFEIGSVSKTFTATLAGYALAQDKMRLDDRASQHWP
PDC-34ASM83699.1_885PaOC       61  KGEPHYFSYGLASKEDGRRVTPETLFEIGSVSKTFTATLAGYALAQDKMRLDDRASQHWP
PDC-34AYW64445.1_883PaOC       61  KGEPHYFSYGLASKEDGRRVTPETLFEIGSVSKTFTATLAGYALAQDKMRLDDRASQHWP
PDC-34UTR27428.1_860PaOC       61  KGEPHYFSYGLASKEDGRRVTPETLFEIGSVSKTFTATLAGYALAQDKMRLDDRASQHWP
PDC-34UOD92021.1_934PaOC       61  KGEPHYFSYGLASKEDGRRVTPETLFEIGSVSKTFTATLAGYALAQDKMRLDDRASQHWP
PDC-34WIV39806.1_860PaOC       61  KGEPHYFSYGLASKEDGRRVTPETLFEIGSVSKTFTATLAGYALAQDKMRLDDRASQHWP
PDC-264QKR42793.1_833PaOC      61  KGEPHYFSYGLASKEDGRQVTPETLFEIGSVSKTFTATLAGYALAQDKMRLDDRASQHWP
PDC-167QIZ28250.1_4314PaOC     61  KGEPHYFSYGLASKEDGRRVTPETLFEIGSVSKTFTATLAGYALAQDKMRLDDRASQHWP
PDC-167UTQ27575.1_842PaOC      61  KGEPHYFSYGLASKEDGRRVTPETLFEIGSVSKTFTATLAGYALAQDKMRLDDRASQHWP
PDC-6QZV30905.1_832PaBCP       61  KGEPHYFSYGLASKEDGRQVTPETLFEIGSVSKTFTATLAGYALTQDKMRLDDRASQHWP
PDC-6QZV30905.1_832PaBOC       61  KGEPHYFSYGLASKEDGRQVTPETLFEIGSVSKTFTATLAGYALTQDKMRLDDRASQHWP
PDC-37WAJ85675.1_843PaBCP      61  KGEPHYFSYGLASKEDGRRVTPETLFEIGSVSKTFTATLAGYALAQDKMRLDDRASQHWP
PDC-37WGX56158.1_855PaBCP      61  KGEPHYFSYGLASKEDGRRVTPETLFEIGSVSKTFTATLAGYALAQDKMRLDDRASQHWP
PDC-37WAJ85675.1_843PaBOC      61  KGEPHYFSYGLASKEDGRRVTPETLFEIGSVSKTFTATLAGYALAQDKMRLDDRASQHWP
PDC-37WGX56158.1_855PaBOC      61  KGEPHYFSYGLASKEDGRRVTPETLFEIGSVSKTFTATLAGYALAQDKMRLDDRASQHWP
PDC-37ANA71197.1_2757.PaOC     61  KGEPHYFSYGLASKEDGRRVTPETLFEIGSVSKTFTATLAGYALAQDKMRLDDRASQHWP
PDC-37WGX14507.1_3195PaOC      61  KGEPHYFSYGLASKEDGRRVTPETLFEIGSVSKTFTATLAGYALAQDKMRLDDRASQHWP
PDC-46AUA93832.1_897PaOC       61  KGEPHYFSYGLASKEDGRRVTPETLFEIGSVSKTFTATLAGYALAQDKMRLDDRASQHWP
PDC-105QGP95004.1_877PaBCP     61  KGEPHYFSYGLASKEDGRRVTPETLFEIGSVSKTFTATLAGYALAQDKMRLDDRASQHWP
PDC-105QGP95004.1_877PaBOC     61  KGEPHYFSYGLASKEDGRRVTPETLFEIGSVSKTFTATLAGYALAQDKMRLDDRASQHWP
PDC-12AVN43882.1_2061.PaOC     61  KGEPHYFSYGLASKEDGRRVTPETLFEIGSVSKTFTATLAGYALAQDKMRLDDRASQHWP
PDC-12QNE63041.1_859PaOC       61  KGEPHYFSYGLASKEDGRRVTPETLFEIGSVSKTFTATLAGYALAQDKMRLDDRASQHWP
PDC-12WGW93864.1_852PaOC       61  KGEPHYFSYGLASKEDGRRVTPETLFEIGSVSKTFTATLAGYALAQDKMRLDDRASQHWP
PDC-12WGX05587.1_852PaOC       61  KGEPHYFSYGLASKEDGRRVTPETLFEIGSVSKTFTATLAGYALAQDKMRLDDRASQHWP
PDC-11QSA85555.1_5581PaBCP     61  KGEPHYFSYGLASKEDGRRVTPETLFEIGSVSKTFTATLAGYALAQDKMRLDDRASQHWP
PDC-11QSA91467.1_5367PaBCP     61  KGEPHYFSYGLASKEDGRRVTPETLFEIGSVSKTFTATLAGYALAQDKMRLDDRASQHWP
PDC-11QSA85555.1_5581PaBOC     61  KGEPHYFSYGLASKEDGRRVTPETLFEIGSVSKTFTATLAGYALAQDKMRLDDRASQHWP
PDC-11QSA91467.1_5367PaBOC     61  KGEPHYFSYGLASKEDGRRVTPETLFEIGSVSKTFTATLAGYALAQDKMRLDDRASQHWP
PDC-11AYQ83203.1_3491PaOC      61  KGEPHYFSYGLASKEDGRRVTPETLFEIGSVSKTFTATLAGYALAQDKMRLDDRASQHWP
PDC-11AYR15208.1_5205PaOC      61  KGEPHYFSYGLASKEDGRRVTPETLFEIGSVSKTFTATLAGYALAQDKMRLDDRASQHWP
PDC-11AZN02324.1_4787PaOC      61  KGEPHYFSYGLASKEDGRRVTPETLFEIGSVSKTFTATLAGYALAQDKMRLDDRASQHWP
PDC-11AZN04123.1_203PaOC       61  KGEPHYFSYGLASKEDGRRVTPETLFEIGSVSKTFTATLAGYALAQDKMRLDDRASQHWP
PDC-11AZN17071.1_5847PaOC      61  KGEPHYFSYGLASKEDGRRVTPETLFEIGSVSKTFTATLAGYALAQDKMRLDDRASQHWP
PDC-11AZN48510.1_4981PaOC      61  KGEPHYFSYGLASKEDGRRVTPETLFEIGSVSKTFTATLAGYALAQDKMRLDDRASQHWP
PDC-11AZN60564.1_5101PaOC      61  KGEPHYFSYGLASKEDGRRVTPETLFEIGSVSKTFTATLAGYALAQDKMRLDDRASQHWP
PDC-11AZN55779.1_6545PaOC      61  KGEPHYFSYGLASKEDGRRVTPETLFEIGSVSKTFTATLAGYALAQDKMRLDDRASQHWP
PDC-11AYF68870.1_744PaOC       61  KGEPHYFSYGLASKEDGRRVTPETLFEIGSVSKTFTATLAGYALAQDKMRLDDRASQHWP
PDC-11QUS21307.1_2433PaOC      61  KGEPHYFSYGLASKEDGRRVTPETLFEIGSVSKTFTATLAGYALAQDKMRLDDRASQHWP
PDC-11UVH87183.1_982PaOC       61  KGEPHYFSYGLASKEDGRRVTPETLFEIGSVSKTFTATLAGYALAQDKMRLDDRASQHWP
PDC-16QCC64126.1_958PaBCP      61  KGEPHYFSYGLASKEDGRRVTPETLFEIGSVSKTFTATLAGYALAQDKMRLDDRASQHWP
PDC-16UBI70916.1_933PaBCP      61  KGEPHYFSYGLASKEDGRRVTPETLFEIGSVSKTFTATLAGYALAQDKMRLDDRASQHWP
PDC-16UTQ84596.1_855PaBCP      61  KGEPHYFSYGLASKEDGRRVTPETLFEIGSVSKTFTATLAGYALAQDKMRLDDRASQHWP
PDC-16QCC64126.1_958PaBOC      61  KGEPHYFSYGLASKEDGRRVTPETLFEIGSVSKTFTATLAGYALAQDKMRLDDRASQHWP
PDC-16UBI70916.1_933PaBOC      61  KGEPHYFSYGLASKEDGRRVTPETLFEIGSVSKTFTATLAGYALAQDKMRLDDRASQHWP
PDC-16UTQ84596.1_855PaBOC      61  KGEPHYFSYGLASKEDGRRVTPETLFEIGSVSKTFTATLAGYALAQDKMRLDDRASQHWP
PDC-16ALS12949.1_2977.PaOC     61  KGEPHYFSYGLASKEDGRRVTPETLFEIGSVSKTFTATLAGYALAQDKMRLDDRASQHWP
PDC-16AYW58201.1_855PaOC       61  KGEPHYFSYGLASKEDGRRVTPETLFEIGSVSKTFTATLAGYALAQDKMRLDDRASQHWP
PDC-16QDR00398.1_890PaOC       61  KGEPHYFSYGLASKEDGRRVTPETLFEIGSVSKTFTATLAGYALAQDKMRLDDRASQHWP
PDC-16QGJ31628.1_895PaOC       61  KGEPHYFSYGLASKEDGRRVTPETLFEIGSVSKTFTATLAGYALAQDKMRLDDRASQHWP
PDC-16QKG34934.1_1840PaOC      61  KGEPHYFSYGLASKEDGRRVTPETLFEIGSVSKTFTATLAGYALAQDKMRLDDRASQHWP
PDC-16QOQ64874.1_122PaOC       61  KGEPHYFSYGLASKEDGRRVTPETLFEIGSVSKTFTATLAGYALAQDKMRLDDRASQHWP
PDC-16QVQ73157.1_860PaOC       61  KGEPHYFSYGLASKEDGRRVTPETLFEIGSVSKTFTATLAGYALAQDKMRLDDRASQHWP
PDC-16UOC64480.1_827PaOC       61  KGEPHYFSYGLASKEDGRRVTPETLFEIGSVSKTFTATLAGYALAQDKMRLDDRASQHWP
PDC-16UOC70564.1_826PaOC       61  KGEPHYFSYGLASKEDGRRVTPETLFEIGSVSKTFTATLAGYALAQDKMRLDDRASQHWP
PDC-16UOC76649.1_827PaOC       61  KGEPHYFSYGLASKEDGRRVTPETLFEIGSVSKTFTATLAGYALAQDKMRLDDRASQHWP
PDC-16UOC82740.1_827PaOC       61  KGEPHYFSYGLASKEDGRRVTPETLFEIGSVSKTFTATLAGYALAQDKMRLDDRASQHWP
PDC-16UOC88825.1_827PaOC       61  KGEPHYFSYGLASKEDGRRVTPETLFEIGSVSKTFTATLAGYALAQDKMRLDDRASQHWP
PDC-16UOE68701.1_827PaOC       61  KGEPHYFSYGLASKEDGRRVTPETLFEIGSVSKTFTATLAGYALAQDKMRLDDRASQHWP
PDC-16WDC65088.1_844PaOC       61  KGEPHYFSYGLASKEDGRRVTPETLFEIGSVSKTFTATLAGYALAQDKMRLDDRASQHWP
PDC-16WGW70130.1_845PaOC       61  KGEPHYFSYGLASKEDGRRVTPETLFEIGSVSKTFTATLAGYALAQDKMRLDDRASQHWP
PDC-16WGW76010.1_845PaOC       61  KGEPHYFSYGLASKEDGRRVTPETLFEIGSVSKTFTATLAGYALAQDKMRLDDRASQHWP
PDC-16WGW81892.1_845PaOC       61  KGEPHYFSYGLASKEDGRRVTPETLFEIGSVSKTFTATLAGYALAQDKMRLDDRASQHWP
PDC-16WGX43443.1_806PaOC       61  KGEPHYFSYGLASKEDGRRVTPETLFEIGSVSKTFTATLAGYALAQDKMRLDDRASQHWP
PDC-16WJM37210.1_871PaOC       61  KGEPHYFSYGLASKEDGRRVTPETLFEIGSVSKTFTATLAGYALAQDKMRLDDRASQHWP
PDC-59UQZ18314.1_902PaOC       61  KGEPHYFSYGLASKEDGRRVTPETLFEIGSVSKTFTATLAGYALAQDKMRLDDRASQHWP
PDC-142WGW57525.1_830PaBCP     61  KGEPHYFSYGLASKEDGRRVTPETLFEIGSVSKTFTATLAGYALAQDKMRLDDRASQHWP
PDC-142WGW32210.1_830PaBCP     61  KGEPHYFSYGLASKEDGRRVTPETLFEIGSVSKTFTATLAGYALAQDKMRLDDRASQHWP
PDC-142WGW44776.1_830PaBCP     61  KGEPHYFSYGLASKEDGRRVTPETLFEIGSVSKTFTATLAGYALAQDKMRLDDRASQHWP
PDC-142WGW57525.1_830PaBOC     61  KGEPHYFSYGLASKEDGRRVTPETLFEIGSVSKTFTATLAGYALAQDKMRLDDRASQHWP
PDC-142WGW32210.1_830PaBOC     61  KGEPHYFSYGLASKEDGRRVTPETLFEIGSVSKTFTATLAGYALAQDKMRLDDRASQHWP
PDC-142WGW44776.1_830PaBOC     61  KGEPHYFSYGLASKEDGRRVTPETLFEIGSVSKTFTATLAGYALAQDKMRLDDRASQHWP
PDC-142AON05138.1_825PaOC      61  KGEPHYFSYGLASKEDGRRVTPETLFEIGSVSKTFTATLAGYALAQDKMRLDDRASQHWP
PDC-142AON11127.1_825PaOC      61  KGEPHYFSYGLASKEDGRRVTPETLFEIGSVSKTFTATLAGYALAQDKMRLDDRASQHWP
PDC-142AON23122.1_825PaOC      61  KGEPHYFSYGLASKEDGRRVTPETLFEIGSVSKTFTATLAGYALAQDKMRLDDRASQHWP
PDC-142AON29110.1_825PaOC      61  KGEPHYFSYGLASKEDGRRVTPETLFEIGSVSKTFTATLAGYALAQDKMRLDDRASQHWP
PDC-142AON41099.1_825PaOC      61  KGEPHYFSYGLASKEDGRRVTPETLFEIGSVSKTFTATLAGYALAQDKMRLDDRASQHWP
PDC-142AON47112.1_824PaOC      61  KGEPHYFSYGLASKEDGRRVTPETLFEIGSVSKTFTATLAGYALAQDKMRLDDRASQHWP
PDC-142AON17115.1_824PaOC      61  KGEPHYFSYGLASKEDGRRVTPETLFEIGSVSKTFTATLAGYALAQDKMRLDDRASQHWP
PDC-142AON35116.1_825PaOC      61  KGEPHYFSYGLASKEDGRRVTPETLFEIGSVSKTFTATLAGYALAQDKMRLDDRASQHWP
PDC-19aWGW51434.1_1207PaBCP    61  KGEPHYFSYGLASKEDGRRVTPETLFEIGSVSKTFTATLAGYALAQDKMRLDDRASQHWP
```

PDC-19aWGW37666.1_6817PaBCP	61	KGEPHYFSYGLASKEDGRRVTPETLFEIGSVSKTFTATLAGYALAQDKMRLDDRASQHWP
PDC-19aWGW59864.1_3660PaBCP	61	KGEPHYFSYGLASKEDGRRVTPETLFEIGSVSKTFTATLAGYALAQDKMRLDDRASQHWP
PDC-19aWGX49838.1_877PaBCP	61	KGEPHYFSYGLASKEDGRRVTPETLFEIGSVSKTFTATLAGYALAQDKMRLDDRASQHWP
PDC-19aWGX24308.1_877PaBCP	61	KGEPHYFSYGLASKEDGRRVTPETLFEIGSVSKTFTATLAGYALAQDKMRLDDRASQHWP
PDC-19aWGX35552.1_5487PaBCP	61	KGEPHYFSYGLASKEDGRRVTPETLFEIGSVSKTFTATLAGYALAQDKMRLDDRASQHWP
PDC-19aWKA37694.1_2446PaBCP	61	KGEPHYFSYGLASKEDGRRVTPETLFEIGSVSKTFTATLAGYALAQDKMRLDDRASQHWP
PDC-19aWGW51434.1_1207PaBOC	61	KGEPHYFSYGLASKEDGRRVTPETLFEIGSVSKTFTATLAGYALAQDKMRLDDRASQHWP
PDC-19aWGW37666.1_6817PaBOC	61	KGEPHYFSYGLASKEDGRRVTPETLFEIGSVSKTFTATLAGYALAQDKMRLDDRASQHWP
PDC-19aWGW59864.1_3660PaBOC	61	KGEPHYFSYGLASKEDGRRVTPETLFEIGSVSKTFTATLAGYALAQDKMRLDDRASQHWP
PDC-19aWGX49838.1_877PaBOC	61	KGEPHYFSYGLASKEDGRRVTPETLFEIGSVSKTFTATLAGYALAQDKMRLDDRASQHWP
PDC-19aWGX24308.1_877PaBOC	61	KGEPHYFSYGLASKEDGRRVTPETLFEIGSVSKTFTATLAGYALAQDKMRLDDRASQHWP
PDC-19aWGX35552.1_5487PaBOC	61	KGEPHYFSYGLASKEDGRRVTPETLFEIGSVSKTFTATLAGYALAQDKMRLDDRASQHWP
PDC-19aWKA37694.1_2446PaBOC	61	KGEPHYFSYGLASKEDGRRVTPETLFEIGSVSKTFTATLAGYALAQDKMRLDDRASQHWP
PDC-19aAPB63370.1_616PaOC	61	KGEPHYFSYGLASKEDGRRVTPETLFEIGSVSKTFTATLAGYALAQDKMRLDDRASQHWP
PDC-19aARI89529.1_870PaOC	61	KGEPHYFSYGLASKEDGRRVTPETLFEIGSVSKTFTATLAGYALAQDKMRLDDRASQHWP
PDC-19aARI95964.1_871PaOC	61	KGEPHYFSYGLASKEDGRRVTPETLFEIGSVSKTFTATLAGYALAQDKMRLDDRASQHWP
PDC-19aASA13594.1_977PaOC	61	KGEPHYFSYGLASKEDGRRVTPETLFEIGSVSKTFTATLAGYALAQDKMRLDDRASQHWP
PDC-19aASA27558.1_962PaOC	61	KGEPHYFSYGLASKEDGRRVTPETLFEIGSVSKTFTATLAGYALAQDKMRLDDRASQHWP
PDC-19aASD01901.1_971PaOC	61	KGEPHYFSYGLASKEDGRRVTPETLFEIGSVSKTFTATLAGYALAQDKMRLDDRASQHWP
PDC-19aUAC92160.1_908PaOC	61	KGEPHYFSYGLASKEDGRRVTPETLFEIGSVSKTFTATLAGYALAQDKMRLDDRASQHWP
PDC-19aUAC98839.1_942PaOC	61	KGEPHYFSYGLASKEDGRRVTPETLFEIGSVSKTFTATLAGYALAQDKMRLDDRASQHWP
PDC-19aUTQ33604.1_830PaOC	61	KGEPHYFSYGLASKEDGRRVTPETLFEIGSVSKTFTATLAGYALAQDKMRLDDRASQHWP
PDC-19aUOO58611.1_830PaOC	61	KGEPHYFSYGLASKEDGRRVTPETLFEIGSVSKTFTATLAGYALAQDKMRLDDRASQHWP
PDC-19aWDA50910.1_883PaOC	61	KGEPHYFSYGLASKEDGRRVTPETLFEIGSVSKTFTATLAGYALAQDKMRLDDRASQHWP
PDC-19aWGX30960.1_941PaOC	61	KGEPHYFSYGLASKEDGRRVTPETLFEIGSVSKTFTATLAGYALAQDKMRLDDRASQHWP
PDC-19aWGX61448.1_5603PaOC	61	KGEPHYFSYGLASKEDGRRVTPETLFEIGSVSKTFTATLAGYALAQDKMRLDDRASQHWP
PDC-19aWGX82118.1_941PaOC	61	KGEPHYFSYGLASKEDGRRVTPETLFEIGSVSKTFTATLAGYALAQDKMRLDDRASQHWP
PDC-19aWIO49783.1_187PaOC	61	KGEPHYFSYGLASKEDGRRVTPETLFEIGSVSKTFTATLAGYALAQDKMRLDDRASQHWP
PDC-24QZV43725.1_941PaBCP	61	KGEPHYFSYGLASKEDGRRVTPETLFEIGSVSKTFTATLAGYALAQDKMRLDDRASQHWP
PDC-24QZV43725.1_941PaBOC	61	KGEPHYFSYGLASKEDGRRVTPETLFEIGSVSKTFTATLAGYALAQDKMRLDDRASQHWP
PDC-24QDR06751.1_860PaOC	61	KGEPHYFSYGLASKEDGRRVTPETLFEIGSVSKTFTATLAGYALAQDKMRLDDRASQHWP
PDC-24UGR36796.1_3412PaOC	61	KGEPHYFSYGLASKEDGRRVTPETLFEIGSVSKTFTATLAGYALAQDKMRLDDRASQHWP
PDC-24UTQ39921.1_833PaOC	61	KGEPHYFSYGLASKEDGRRVTPETLFEIGSVSKTFTATLAGYALAQDKMRLDDRASQHWP
PDC-137QKE94864.1_852PaOC	61	KGEPHYFSYGLASKEDGRRVTPETLFEIGSVSKTFTATLAGYALAQDKMRLDDSASQHWP
PDC-147UGR30133.1_1950PaOC	61	KGEPHYFSYGLASKEDGRRVTPETLFEIGSVSKTFTATLAGYALAQDKMRLDDRASQHWP
PDC-8AYY41152.1_5352PaBCP	61	KGEPHYFSYGLASKEDGRRVTPETLFEIGSVSKTFTATLAGYALAQDKMRLDDRASQHWP
PDC-8QOE14337.1_211PaBCP	61	KGEPHYFSYGLASKEDGRRVTPETLFEIGSVSKTFTATLAGYALAQDKMRLDDRASQHWP
PDC-8QPN15577.1_911PaBCP	61	KGEPHYFSYGLASKEDGRRVTPETLFEIGSVSKTFTATLAGYALAQDKMRLDDRASQHWP
PDC-8QPN23993.1_908PaBCP	61	KGEPHYFSYGLASKEDGRRVTPETLFEIGSVSKTFTATLAGYALAQDKMRLDDRASQHWP
PDC-8QUE64702.1_909PaBCP	61	KGEPHYFSYGLASKEDGRRVTPETLFEIGSVSKTFTATLAGYALAQDKMRLDDRASQHWP
PDC-8QYA88773.1_2249PaBCP	61	KGEPHYFSYGLASKEDGRRVTPETLFEIGSVSKTFTATLAGYALAQDKMRLDDRASQHWP
PDC-8QYF01720.1_5249PaBCP	61	KGEPHYFSYGLASKEDGRRVTPETLFEIGSVSKTFTATLAGYALAQDKMRLDDRASQHWP
PDC-8QZU99504.1_908PaBCP	61	KGEPHYFSYGLASKEDGRRVTPETLFEIGSVSKTFTATLAGYALAQDKMRLDDRASQHWP
PDC-8QZV05727.1_908PaBCP	61	KGEPHYFSYGLASKEDGRRVTPETLFEIGSVSKTFTATLAGYALAQDKMRLDDRASQHWP
PDC-8QZV12028.1_910PaBCP	61	KGEPHYFSYGLASKEDGRRVTPETLFEIGSVSKTFTATLAGYALAQDKMRLDDRASQHWP
PDC-8QZV18112.1_863PaBCP	61	KGEPHYFSYGLASKEDGRRVTPETLFEIGSVSKTFTATLAGYALAQDKMRLDDRASQHWP
PDC-8QZV24387.1_912PaBCP	61	KGEPHYFSYGLASKEDGRRVTPETLFEIGSVSKTFTATLAGYALAQDKMRLDDRASQHWP
PDC-8QZV68851.1_908PaBCP	61	KGEPHYFSYGLASKEDGRRVTPETLFEIGSVSKTFTATLAGYALAQDKMRLDDRASQHWP
PDC-8QZV81338.1_870PaBCP	61	KGEPHYFSYGLASKEDGRRVTPETLFEIGSVSKTFTATLAGYALAQDKMRLDDRASQHWP
PDC-8QZV87769.1_908PaBCP	61	KGEPHYFSYGLASKEDGRRVTPETLFEIGSVSKTFTATLAGYALAQDKMRLDDRASQHWP
PDC-8QZV94048.1_908PaBCP	61	KGEPHYFSYGLASKEDGRRVTPETLFEIGSVSKTFTATLAGYALAQDKMRLDDRASQHWP
PDC-8UXH53497.1_877PaBCP	61	KGEPHYFSYGLASKEDGRRVTPETLFEIGSVSKTFTATLAGYALAQDKMRLDDRASQHWP
PDC-8UXH66521.1_877PaBCP	61	KGEPHYFSYGLASKEDGRRVTPETLFEIGSVSKTFTATLAGYALAQDKMRLDDRASQHWP
PDC-8UXH70664.1_4987PaBCP	61	KGEPHYFSYGLASKEDGRRVTPETLFEIGSVSKTFTATLAGYALAQDKMRLDDRASQHWP
PDC-8UUW81479.1_5273PaBCP	61	KGEPHYFSYGLASKEDGRRVTPETLFEIGSVSKTFTATLAGYALAQDKMRLDDRASQHWP
PDC-8WDM07130.1_910PaBCP	61	KGEPHYFSYGLASKEDGRRVTPETLFEIGSVSKTFTATLAGYALAQDKMRLDDRASQHWP
PDC-8WHM62936.1_909PaBCP	61	KGEPHYFSYGLASKEDGRRVTPETLFEIGSVSKTFTATLAGYALAQDKMRLDDRASQHWP
PDC-8WHM69354.1_909PaBCP	61	KGEPHYFSYGLASKEDGRRVTPETLFEIGSVSKTFTATLAGYALAQDKMRLDDRASQHWP
PDC-8WHM75768.1_909PaBCP	61	KGEPHYFSYGLASKEDGRRVTPETLFEIGSVSKTFTATLAGYALAQDKMRLDDRASQHWP
PDC-8WHM84263.1_909PaBCP	61	KGEPHYFSYGLASKEDGRRVTPETLFEIGSVSKTFTATLAGYALAQDKMRLDDRASQHWP
PDC-8AYY41152.1_5352PaBOC	61	KGEPHYFSYGLASKEDGRRVTPETLFEIGSVSKTFTATLAGYALAQDKMRLDDRASQHWP
PDC-8QOE14337.1_211PaBOC	61	KGEPHYFSYGLASKEDGRRVTPETLFEIGSVSKTFTATLAGYALAQDKMRLDDRASQHWP
PDC-8QPN15577.1_911PaBOC	61	KGEPHYFSYGLASKEDGRRVTPETLFEIGSVSKTFTATLAGYALAQDKMRLDDRASQHWP
PDC-8QPN23993.1_908PaBOC	61	KGEPHYFSYGLASKEDGRRVTPETLFEIGSVSKTFTATLAGYALAQDKMRLDDRASQHWP
PDC-8QUE64702.1_909PaBOC	61	KGEPHYFSYGLASKEDGRRVTPETLFEIGSVSKTFTATLAGYALAQDKMRLDDRASQHWP
PDC-8QYA88773.1_2249PaBOC	61	KGEPHYFSYGLASKEDGRRVTPETLFEIGSVSKTFTATLAGYALAQDKMRLDDRASQHWP
PDC-8QYF01720.1_5249PaBOC	61	KGEPHYFSYGLASKEDGRRVTPETLFEIGSVSKTFTATLAGYALAQDKMRLDDRASQHWP
PDC-8QZU99504.1_908PaBOC	61	KGEPHYFSYGLASKEDGRRVTPETLFEIGSVSKTFTATLAGYALAQDKMRLDDRASQHWP
PDC-8QZV05727.1_908PaBOC	61	KGEPHYFSYGLASKEDGRRVTPETLFEIGSVSKTFTATLAGYALAQDKMRLDDRASQHWP
PDC-8QZV12028.1_910PaBOC	61	KGEPHYFSYGLASKEDGRRVTPETLFEIGSVSKTFTATLAGYALAQDKMRLDDRASQHWP
PDC-8QZV18112.1_863PaBOC	61	KGEPHYFSYGLASKEDGRRVTPETLFEIGSVSKTFTATLAGYALAQDKMRLDDRASQHWP
PDC-8QZV24387.1_912PaBOC	61	KGEPHYFSYGLASKEDGRRVTPETLFEIGSVSKTFTATLAGYALAQDKMRLDDRASQHWP
PDC-8QZV68851.1_908PaBOC	61	KGEPHYFSYGLASKEDGRRVTPETLFEIGSVSKTFTATLAGYALAQDKMRLDDRASQHWP
PDC-8QZV81338.1_870PaBOC	61	KGEPHYFSYGLASKEDGRRVTPETLFEIGSVSKTFTATLAGYALAQDKMRLDDRASQHWP
PDC-8QZV87769.1_908PaBOC	61	KGEPHYFSYGLASKEDGRRVTPETLFEIGSVSKTFTATLAGYALAQDKMRLDDRASQHWP
PDC-8QZV94048.1_908PaBOC	61	KGEPHYFSYGLASKEDGRRVTPETLFEIGSVSKTFTATLAGYALAQDKMRLDDRASQHWP
PDC-8UXH53497.1_877PaBOC	61	KGEPHYFSYGLASKEDGRRVTPETLFEIGSVSKTFTATLAGYALAQDKMRLDDRASQHWP
PDC-8UXH66521.1_877PaBOC	61	KGEPHYFSYGLASKEDGRRVTPETLFEIGSVSKTFTATLAGYALAQDKMRLDDRASQHWP
PDC-8UXH70664.1_4987PaBOC	61	KGEPHYFSYGLASKEDGRRVTPETLFEIGSVSKTFTATLAGYALAQDKMRLDDRASQHWP
PDC-8UUW81479.1_5273PaBOC	61	KGEPHYFSYGLASKEDGRRVTPETLFEIGSVSKTFTATLAGYALAQDKMRLDDRASQHWP
PDC-8WDM07130.1_910PaBOC	61	KGEPHYFSYGLASKEDGRRVTPETLFEIGSVSKTFTATLAGYALAQDKMRLDDRASQHWP
PDC-8WHM62936.1_909PaBOC	61	KGEPHYFSYGLASKEDGRRVTPETLFEIGSVSKTFTATLAGYALAQDKMRLDDRASQHWP
PDC-8WHM69354.1_909PaBOC	61	KGEPHYFSYGLASKEDGRRVTPETLFEIGSVSKTFTATLAGYALAQDKMRLDDRASQHWP
PDC-8WHM75768.1_909PaBOC	61	KGEPHYFSYGLASKEDGRRVTPETLFEIGSVSKTFTATLAGYALAQDKMRLDDRASQHWP
PDC-8WHM84263.1_909PaBOC	61	KGEPHYFSYGLASKEDGRRVTPETLFEIGSVSKTFTATLAGYALAQDKMRLDDRASQHWP
PDC-8AMA35328.1_919.PaOC	61	KGEPHYFSYGLASKEDGRRVTPETLFEIGSVSKTFTATLAGYALAQDKMRLDDRASQHWP
PDC-8ANI06966.1_119PaOC	61	KGEPHYFSYGLASKEDGRRVTPETLFEIGSVSKTFTATLAGYALAQDKMRLDDRASQHWP
PDC-8AYW70752.1_952PaOC	61	KGEPHYFSYGLASKEDGRRVTPETLFEIGSVSKTFTATLAGYALAQDKMRLDDRASQHWP
PDC-8AYZ76972.1_2272PaOC	61	KGEPHYFSYGLASKEDGRRVTPETLFEIGSVSKTFTATLAGYALAQDKMRLDDRASQHWP
PDC-8QKP85289.1_778PaOC	61	KGEPHYFSYGLASKEDGRRVTPETLFEIGSVSKTFTATLAGYALAQDKMRLDDRASQHWP
PDC-8QKR30573.1_5806PaOC	61	KGEPHYFSYGLASKEDGRRVTPETLFEIGSVSKTFTATLAGYALAQDKMRLDDRASQHWP
PDC-8QLF19879.1_939PaOC	61	KGEPHYFSYGLASKEDGRRVTPETLFEIGSVSKTFTATLAGYALAQDKMRLDDRASQHWP
PDC-8QYE59452.1_5107PaOC	61	KGEPHYFSYGLASKEDGRRVTPETLFEIGSVSKTFTATLAGYALAQDKMRLDDRASQHWP
PDC-8QZV98104.1_4956PaOC	61	KGEPHYFSYGLASKEDGRRVTPETLFEIGSVSKTFTATLAGYALAQDKMRLDDRASQHWP
PDC-8UFK73541.1_1068PaOC	61	KGEPHYFSYGLASKEDGRRVTPETLFEIGSVSKTFTATLAGYALAQDKMRLDDRASQHWP
PDC-8UGR33975.2_368PaOC	61	KGEPHYFSYGLASKEDGRRVTPETLFEIGSVSKTFTATLAGYALAQDKMRLDDRASQHWP
PDC-8UTN26696.1_862PaOC	61	KGEPHYFSYGLASKEDGRRVTPETLFEIGSVSKTFTATLAGYALAQDKMRLDDRASQHWP
PDC-8UTQ91314.1_910PaOC	61	KGEPHYFSYGLASKEDGRRVTPETLFEIGSVSKTFTATLAGYALAQDKMRLDDRASQHWP
PDC-8UNK85299.1_868PaOC	61	KGEPHYFSYGLASKEDGRRVTPETLFEIGSVSKTFTATLAGYALAQDKMRLDDRASQHWP
PDC-8UNK97662.1_866PaOC	61	KGEPHYFSYGLASKEDGRRVTPETLFEIGSVSKTFTATLAGYALAQDKMRLDDRASQHWP
PDC-8USI83494.1_833PaOC	61	KGEPHYFSYGLASKEDGRRVTPETLFEIGSVSKTFTATLAGYALAQDKMRLDDRASQHWP
PDC-8WAO16415.1_5521PaOC	61	KGEPHYFSYGLASKEDGRRVTPETLFEIGSVSKTFTATLAGYALAQDKMRLDDRASQHWP
PDC-98QZD62144.1_4346PaBCP	61	KGEPHYFSYGLASKEDGRRVTPETLFEIGSVSKTFTATLAGYALTQDKMRLDDRASQHWP

```
PDC-98QZH48390.1_4214PaBCP       61  KGEPHYFSYGLASKEDGRRVTPETLFEIGSVSKTFTATLAGYALTQDKMRLDDRASQHWP
PDC-98QZD62144.1_4346PaBOC       61  KGEPHYFSYGLASKEDGRRVTPETLFEIGSVSKTFTATLAGYALTQDKMRLDDRASQHWP
PDC-98QZH48390.1_4214PaBOC       61  KGEPHYFSYGLASKEDGRRVTPETLFEIGSVSKTFTATLAGYALTQDKMRLDDRASQHWP
PDC-98ALP56563.1_1203.PaOC       61  KGEPHYFSYGLASKEDGRRVTPETLFEIGSVSKTFTATLAGYALTQDKMRLDDRASQHWP
PDC-98UTL97623.1_830PaOC         61  KGEPHYFSYGLASKEDGRRVTPETLFEIGSVSKTFTATLAGYALTQDKMRLDDRASQHWP
PDC-457UTN40061.1_863PaOC        61  KGEPHYFSYGLASKEDGRRVTPETLFEIGSVSKTFTATLAGYALAQDKMRLDDRASQHWP
PDC-23UXI89283.1_830PaOC         61  KGEPHYFSYGLASKEDGRRVTPETLFEIGSVSKTFTATLAGYALAQDKMRLDDRASQHWP
PDC-245AUA75408.1_866PaOC        61  KGEPHYFSYGLASKEDGRRVTPETLFEIGSVSKTFTVTLAGYALAQDKMRLDDRASQHWP
PDC-31QZW06655.1_840PaBCP        61  KGEPHYFSYGLASKEDGRRVTPETLFEIGSVSKTFTATLAGYALAQDKMRLDDRASQHWP
PDC-31QZW06655.1_840PaBOC        61  KGEPHYFSYGLASKEDGRRVTPETLFEIGSVSKTFTATLAGYALAQDKMRLDDRASQHWP
PDC-31AXN27248.1_931PaOC         61  KGEPHYFSYGLASKEDGRRVTPETLFEIGSVSKTFTATLAGYALAQDKMRLDDRASQHWP
PDC-31QBC07904.1_3021PaOC        61  KGEPHYFSYGLASKEDGRRVTPETLFEIGSVSKTFTATLAGYALAQDKMRLDDRASQHWP
PDC-31UFM90275.1_905PaOC         61  KGEPHYFSYGLASKEDGRRVTPETLFEIGSVSKTFTATLAGYALAQDKMRLDDRASQHWP
PDC-31UFM98870.1_905PaOC         61  KGEPHYFSYGLASKEDGRRVTPETLFEIGSVSKTFTATLAGYALAQDKMRLDDRASQHWP
PDC-31UNT25139.1_901PaOC         61  KGEPHYFSYGLASKEDGRRVTPETLFEIGSVSKTFTATLAGYALAQDKMRLDDRASQHWP
PDC-31UWJ96607.1_4267PaOC        61  KGEPHYFSYGLASKEDGRRVTPETLFEIGSVSKTFTATLAGYALAQDKMRLDDRASQHWP
PDC-31WGX88300.1_823PaOC         61  KGEPHYFSYGLASKEDGRRVTPETLFEIGSVSKTFTATLAGYALAQDKMRLDDRASQHWP
PDC-31WHM08550.1_859PaOC         61  KGEPHYFSYGLASKEDGRRVTPETLFEIGSVSKTFTATLAGYALAQDKMRLDDRASQHWP
PDC-39AYK21377.1_872PaOC         61  KGEPHYFSYGLASKEDGRRVTPETLFEIGSVSKTFTATLAGYALAQDKMRLDDRASQHWP
PDC-39UJC21946.1_837PaOC         61  KGEPHYFSYGLASKEDGRRVTPETLFEIGSVSKTFTATLAGYALAQDKMRLDDRASQHWP
PDC-30UPL39337.1_928PaBCP        61  KGEPHYFSYGLASKEDGRRVTPETLFEIGSVSKTFTATLAGYALAQDKMRLDDRASQHWP
PDC-30UPL39337.1_928PaBOC        61  KGEPHYFSYGLASKEDGRRVTPETLFEIGSVSKTFTATLAGYALAQDKMRLDDRASQHWP
PDC-30UHL95932.1_5029PaOC        61  KGEPHYFSYGLASKEDGRRVTPETLFEIGSVSKTFTATLAGYALAQDKMRLDDRASQHWP
PDC-30UML99445.1_838PaOC         61  KGEPHYFSYGLASKEDGRRVTPETLFEIGSVSKTFTATLAGYALAQDKMRLDDRASQHWP
PDC-80WAJ79202.1_935PaOC         61  KGEPHYFSYGLASKEDGRRVTPETLFEIGSVSKTFTATLAGYALTQDKMRLDDRASQHWP
8SDL_1|Chain                     61  KGEPHYFSYGLASKEDGRRVTPETLFEIGSVSKTFTATLAGYALAQDKMRLDDRASQHWP
PDC-3UTN33167.1_589PaBCP         61  KGEPHYFSYGLASKEDGRRVTPETLFEIGSVSKTFTATLAGYALAQDKMRLDDRASQHWP
PDC-3WCI38158.1_984PaBCP         61  KGEPHYFSYGLASKEDGRRVTPETLFEIGSVSKTFTATLAGYALAQDKMRLDDRASQHWP
PDC-3WCI50909.1_867PaBCP         61  KGEPHYFSYGLASKEDGRRVTPETLFEIGSVSKTFTATLAGYALAQDKMRLDDRASQHWP
PDC-3WCI90621.1_961PaBCP         61  KGEPHYFSYGLASKEDGRRVTPETLFEIGSVSKTFTATLAGYALAQDKMRLDDRASQHWP
PDC-3WEO48793.1_832PaBCP         61  KGEPHYFSYGLASKEDGRRVTPETLFEIGSVSKTFTATLAGYALAQDKMRLDDRASQHWP
PDC-3CAI9907424.1_19963]PaBCP    61  KGEPHYFSYGLASKEDGRRVTPETLFEIGSVSKTFTATLAGYALAQDKMRLDDRASQHWP
PDC-3UTN33167.1_589PaBOC         61  KGEPHYFSYGLASKEDGRRVTPETLFEIGSVSKTFTATLAGYALAQDKMRLDDRASQHWP
PDC-3WCI38158.1_984PaBOC         61  KGEPHYFSYGLASKEDGRRVTPETLFEIGSVSKTFTATLAGYALAQDKMRLDDRASQHWP
PDC-3WCI50909.1_867PaBOC         61  KGEPHYFSYGLASKEDGRRVTPETLFEIGSVSKTFTATLAGYALAQDKMRLDDRASQHWP
PDC-3WCI90621.1_961PaBOC         61  KGEPHYFSYGLASKEDGRRVTPETLFEIGSVSKTFTATLAGYALAQDKMRLDDRASQHWP
PDC-3WEO48793.1_832PaBOC         61  KGEPHYFSYGLASKEDGRRVTPETLFEIGSVSKTFTATLAGYALAQDKMRLDDRASQHWP
PDC-3CAI9907424.1_19963]PaBOC    61  KGEPHYFSYGLASKEDGRRVTPETLFEIGSVSKTFTATLAGYALAQDKMRLDDRASQHWP
PDC-3AHA17954.1_839.PaOC         61  KGEPHYFSYGLASKEDGRRVTPETLFEIGSVSKTFTATLAGYALAQDKMRLDDRASQHWP
PDC-3ALE46683.1_840.PaOC         61  KGEPHYFSYGLASKEDGRRVTPETLFEIGSVSKTFTATLAGYALAQDKMRLDDRASQHWP
PDC-3ARC78082.1_863.PaOC         61  KGEPHYFSYGLASKEDGRRVTPETLFEIGSVSKTFTATLAGYALAQDKMRLDDRASQHWP
PDC-3ARG53386.1_5885PaOC         61  KGEPHYFSYGLASKEDGRRVTPETLFEIGSVSKTFTATLAGYALAQDKMRLDDRASQHWP
PDC-3WAC79950.1_4482PaOC         61  KGEPHYFSYGLASKEDGRRVTPETLFEIGSVSKTFTATLAGYALAQDKMRLDDRASQHWP
PDC-3WAE22012.1_4863PaOC         61  KGEPHYFSYGLASKEDGRRVTPETLFEIGSVSKTFTATLAGYALAQDKMRLDDRASQHWP
PDC-3WAE29433.1_777PaOC          61  KGEPHYFSYGLASKEDGRRVTPETLFEIGSVSKTFTATLAGYALAQDKMRLDDRASQHWP
PDC-3WAW42145.1_847PaOC          61  KGEPHYFSYGLASKEDGRRVTPETLFEIGSVSKTFTATLAGYALAQDKMRLDDRASQHWP
PDC-3WCI57477.1_911PaOC          61  KGEPHYFSYGLASKEDGRRVTPETLFEIGSVSKTFTATLAGYALAQDKMRLDDRASQHWP
PDC-3WCI77402.1_900PaOC          61  KGEPHYFSYGLASKEDGRRVTPETLFEIGSVSKTFTATLAGYALAQDKMRLDDRASQHWP
PDC-3WCI64002.1_913PaOC          61  KGEPHYFSYGLASKEDGRRVTPETLFEIGSVSKTFTATLAGYALAQDKMRLDDRASQHWP
PDC-3WCI83994.1_905PaOC          61  KGEPHYFSYGLASKEDGRRVTPETLFEIGSVSKTFTATLAGYALAQDKMRLDDRASQHWP
PDC-3WJM48814.1_842PaOC          61  KGEPHYFSYGLASKEDGRRVTPETLFEIGSVSKTFTATLAGYALAQDKMRLDDRASQHWP
PDC-374ANP60834.1_3741PaBCP      61  KGEPHYFSYGLASKEDGRRVTPETLFEIGSVSKTFTATLAGYALTQDKMRLDDRASQHWP
PDC-374AYZ86971.1_5950PaBCP      61  KGEPHYFSYGLASKEDGRRVTPETLFEIGSVSKTFTATLAGYALTQDKMRLDDRASQHWP
PDC-374QZV75115.1_843PaBCP       61  KGEPHYFSYGLASKEDGRRVTPETLFEIGSVSKTFTATLAGYALTQDKMRLDDRASQHWP
PDC-374QZW12711.1_843PaBCP       61  KGEPHYFSYGLASKEDGRRVTPETLFEIGSVSKTFTATLAGYALTQDKMRLDDRASQHWP
PDC-374UEG06685.1_907PaBCP       61  KGEPHYFSYGLASKEDGRRVTPETLFEIGSVSKTFTATLAGYALTQDKMRLDDRASQHWP
PDC-374UGW95802.1_984PaBCP       61  KGEPHYFSYGLASKEDGRRVTPETLFEIGSVSKTFTATLAGYALTQDKMRLDDRASQHWP
PDC-374WGW26222.1_839PaBCP       61  KGEPHYFSYGLASKEDGRRVTPETLFEIGSVSKTFTATLAGYALTQDKMRLDDRASQHWP
PDC-374ANP60834.1_3741PaBOC      61  KGEPHYFSYGLASKEDGRRVTPETLFEIGSVSKTFTATLAGYALTQDKMRLDDRASQHWP
PDC-374AYZ86971.1_5950PaBOC      61  KGEPHYFSYGLASKEDGRRVTPETLFEIGSVSKTFTATLAGYALTQDKMRLDDRASQHWP
PDC-374QZV75115.1_843PaBOC       61  KGEPHYFSYGLASKEDGRRVTPETLFEIGSVSKTFTATLAGYALTQDKMRLDDRASQHWP
PDC-374QZW12711.1_843PaBOC       61  KGEPHYFSYGLASKEDGRRVTPETLFEIGSVSKTFTATLAGYALTQDKMRLDDRASQHWP
PDC-374UEG06685.1_907PaBOC       61  KGEPHYFSYGLASKEDGRRVTPETLFEIGSVSKTFTATLAGYALTQDKMRLDDRASQHWP
PDC-374UGW95802.1_984PaBOC       61  KGEPHYFSYGLASKEDGRRVTPETLFEIGSVSKTFTATLAGYALTQDKMRLDDRASQHWP
PDC-374WGW26222.1_839PaBOC       61  KGEPHYFSYGLASKEDGRRVTPETLFEIGSVSKTFTATLAGYALTQDKMRLDDRASQHWP
PDC-374AOP56366.1_851PaOC        61  KGEPHYFSYGLASKEDGRRVTPETLFEIGSVSKTFTATLAGYALTQDKMRLDDRASQHWP
PDC-374AVE31445.1_873.PaOC       61  KGEPHYFSYGLASKEDGRRVTPETLFEIGSVSKTFTATLAGYALTQDKMRLDDRASQHWP
PDC-374QAS71510.1_873PaOC        61  KGEPHYFSYGLASKEDGRRVTPETLFEIGSVSKTFTATLAGYALTQDKMRLDDRASQHWP
PDC-374QDD38027.1_4463PaOC       61  KGEPHYFSYGLASKEDGRRVTPETLFEIGSVSKTFTATLAGYALTQDKMRLDDRASQHWP
PDC-374QKF05339.1_5830PaOC       61  KGEPHYFSYGLASKEDGRRVTPETLFEIGSVSKTFTATLAGYALTQDKMRLDDRASQHWP
PDC-374QKK83983.1_853PaOC        61  KGEPHYFSYGLASKEDGRRVTPETLFEIGSVSKTFTATLAGYALTQDKMRLDDRASQHWP
PDC-374QKS41466.1_4199PaOC       61  KGEPHYFSYGLASKEDGRRVTPETLFEIGSVSKTFTATLAGYALTQDKMRLDDRASQHWP
PDC-374QPZ80284.1_873PaOC        61  KGEPHYFSYGLASKEDGRRVTPETLFEIGSVSKTFTATLAGYALTQDKMRLDDRASQHWP
PDC-374QQW07466.1_922PaOC        61  KGEPHYFSYGLASKEDGRRVTPETLFEIGSVSKTFTATLAGYALTQDKMRLDDRASQHWP
PDC-374QZU93305.1_843PaOC        61  KGEPHYFSYGLASKEDGRRVTPETLFEIGSVSKTFTATLAGYALTQDKMRLDDRASQHWP
PDC-374UAC85653.1_934PaOC        61  KGEPHYFSYGLASKEDGRRVTPETLFEIGSVSKTFTATLAGYALTQDKMRLDDRASQHWP
PDC-374UGR20250.1_3485PaOC       61  KGEPHYFSYGLASKEDGRRVTPETLFEIGSVSKTFTATLAGYALTQDKMRLDDRASQHWP
PDC-374UIU12727.1_851PaOC        61  KGEPHYFSYGLASKEDGRRVTPETLFEIGSVSKTFTATLAGYALTQDKMRLDDRASQHWP
PDC-374UJC03215.1_956PaOC        61  KGEPHYFSYGLASKEDGRRVTPETLFEIGSVSKTFTATLAGYALTQDKMRLDDRASQHWP
PDC-374UJC09425.1_959PaOC        61  KGEPHYFSYGLASKEDGRRVTPETLFEIGSVSKTFTATLAGYALTQDKMRLDDRASQHWP
PDC-374UJF57976.1_829PaOC        61  KGEPHYFSYGLASKEDGRRVTPETLFEIGSVSKTFTATLAGYALTQDKMRLDDRASQHWP
PDC-374UJF63613.1_829PaOC        61  KGEPHYFSYGLASKEDGRRVTPETLFEIGSVSKTFTATLAGYALTQDKMRLDDRASQHWP
PDC-374UJF68041.1_5361PaOC       61  KGEPHYFSYGLASKEDGRRVTPETLFEIGSVSKTFTATLAGYALTQDKMRLDDRASQHWP
PDC-374UJF74874.1_829PaOC        61  KGEPHYFSYGLASKEDGRRVTPETLFEIGSVSKTFTATLAGYALTQDKMRLDDRASQHWP
PDC-374UJF80517.1_829PaOC        61  KGEPHYFSYGLASKEDGRRVTPETLFEIGSVSKTFTATLAGYALTQDKMRLDDRASQHWP
PDC-374UJF86151.1_829PaOC        61  KGEPHYFSYGLASKEDGRRVTPETLFEIGSVSKTFTATLAGYALTQDKMRLDDRASQHWP
PDC-374UJF90590.1_5359PaOC       61  KGEPHYFSYGLASKEDGRRVTPETLFEIGSVSKTFTATLAGYALTQDKMRLDDRASQHWP
PDC-374UJF97432.1_830PaOC        61  KGEPHYFSYGLASKEDGRRVTPETLFEIGSVSKTFTATLAGYALTQDKMRLDDRASQHWP
PDC-374UJG01877.1_5359PaOC       61  KGEPHYFSYGLASKEDGRRVTPETLFEIGSVSKTFTATLAGYALTQDKMRLDDRASQHWP
PDC-374UJG08704.1_829PaOC        61  KGEPHYFSYGLASKEDGRRVTPETLFEIGSVSKTFTATLAGYALTQDKMRLDDRASQHWP
PDC-374UJG14426.1_829PaOC        61  KGEPHYFSYGLASKEDGRRVTPETLFEIGSVSKTFTATLAGYALTQDKMRLDDRASQHWP
PDC-374UJG20129.1_829PaOC        61  KGEPHYFSYGLASKEDGRRVTPETLFEIGSVSKTFTATLAGYALTQDKMRLDDRASQHWP
PDC-374UJG25828.1_829PaOC        61  KGEPHYFSYGLASKEDGRRVTPETLFEIGSVSKTFTATLAGYALTQDKMRLDDRASQHWP
PDC-374UJG31693.1_884PaOC        61  KGEPHYFSYGLASKEDGRRVTPETLFEIGSVSKTFTATLAGYALTQDKMRLDDRASQHWP
PDC-374UJG36239.1_5358PaOC       61  KGEPHYFSYGLASKEDGRRVTPETLFEIGSVSKTFTATLAGYALTQDKMRLDDRASQHWP
PDC-374UGR51117.1_68PaOC         61  KGEPHYFSYGLASKEDGRRVTPETLFEIGSVSKTFTATLAGYALTQDKMRLDDRASQHWP
PDC-374UON75698.1_5570PaOC       61  KGEPHYFSYGLASKEDGRRVTPETLFEIGSVSKTFTATLAGYALTQDKMRLDDRASQHWP
PDC-374UPG04116.1_3174PaOC       61  KGEPHYFSYGLASKEDGRRVTPETLFEIGSVSKTFTATLAGYALTQDKMRLDDRASQHWP
PDC-374UPZ07602.1_2096PaOC       61  KGEPHYFSYGLASKEDGRRVTPETLFEIGSVSKTFTATLAGYALTQDKMRLDDRASQHWP
PDC-374URM55875.1_5937PaOC       61  KGEPHYFSYGLASKEDGRRVTPETLFEIGSVSKTFTATLAGYALTQDKMRLDDRASQHWP
PDC-374USV13808.1_3922PaOC       61  KGEPHYFSYGLASKEDGRRVTPETLFEIGSVSKTFTATLAGYALTQDKMRLDDRASQHWP
PDC-374USV19196.1_4087PaOC       61  KGEPHYFSYGLASKEDGRRVTPETLFEIGSVSKTFTATLAGYALTQDKMRLDDRASQHWP
PDC-374USX84542.1_851PaOC        61  KGEPHYFSYGLASKEDGRRVTPETLFEIGSVSKTFTATLAGYALTQDKMRLDDRASQHWP
```

```
PDC-374USX78872.1_851PaOC         61  KGEPHYFSYGLASKEDGRRVTPETLFEIGSVSKTFTATLAGYALTQDKMRLDDRASQHWP
PDC-374UUJ85605.1_1747PaOC        61  KGEPHYFSYGLASKEDGRRVTPETLFEIGSVSKTFTATLAGYALTQDKMRLDDRASQHWP
PDC-374UVS71982.1_4220PaOC        61  KGEPHYFSYGLASKEDGRRVTPETLFEIGSVSKTFTATLAGYALTQDKMRLDDRASQHWP
PDC-374UWI85046.1_4210PaOC        61  KGEPHYFSYGLASKEDGRRVTPETLFEIGSVSKTFTATLAGYALTQDKMRLDDRASQHWP
PDC-374UWI92968.1_851PaOC         61  KGEPHYFSYGLASKEDGRRVTPETLFEIGSVSKTFTATLAGYALTQDKMRLDDRASQHWP
PDC-374UXA22714.1_4203PaOC        61  KGEPHYFSYGLASKEDGRRVTPETLFEIGSVSKTFTATLAGYALTQDKMRLDDRASQHWP
PDC-374UXA30656.1_926PaOC         61  KGEPHYFSYGLASKEDGRRVTPETLFEIGSVSKTFTATLAGYALTQDKMRLDDRASQHWP
PDC-374UXA44988.1_926PaOC         61  KGEPHYFSYGLASKEDGRRVTPETLFEIGSVSKTFTATLAGYALTQDKMRLDDRASQHWP
PDC-374UXN39470.1_4222PaOC        61  KGEPHYFSYGLASKEDGRRVTPETLFEIGSVSKTFTATLAGYALTQDKMRLDDRASQHWP
PDC-374UZA87382.1_4980PaOC        61  KGEPHYFSYGLASKEDGRRVTPETLFEIGSVSKTFTATLAGYALTQDKMRLDDRASQHWP
PDC-374WBM66401.1_846PaOC         61  KGEPHYFSYGLASKEDGRRVTPETLFEIGSVSKTFTATLAGYALTQDKMRLDDRASQHWP
PDC-374WGJ75339.1_925PaOC         61  KGEPHYFSYGLASKEDGRRVTPETLFEIGSVSKTFTATLAGYALTQDKMRLDDRASQHWP
PDC-374WGW87865.1_837PaOC         61  KGEPHYFSYGLASKEDGRRVTPETLFEIGSVSKTFTATLAGYALTQDKMRLDDRASQHWP
PDC-103QES80695.1_910PaOC         61  KGEPHYFSYGLASKEDGRRVTLETLFEIGSVSKTFTATLAGYALAQDKMRLDDRASQHWP
PDC-103QPV53905.1_6224PaOC        61  KGEPHYFSYGLASKEDGRRVTLETLFEIGSVSKTFTATLAGYALAQDKMRLDDRASQHWP
PDC-35AVR81260.1_945.PaOC         61  KGEPHYFSYGLASKEDGRRVTPETLFEIGSVSKTFTVTLAGYALAQDKMRLDDRASQHWP
PDC-35QJA35930.1_977PaOC          61  KGEPHYFSYGLASKEDGRRVTPETLFEIGSVSKTFTVTLAGYALAQDKMRLDDRASQHWP
PDC-35AYZ46521.1_3210PaOC         61  KGEPHYFSYGLASKEDGRRVTPETLFEIGSVSKTFTVTLAGYALAQDKMRLDDRASQHWP
PDC-35AYZ59004.1_3976PaOC         61  KGEPHYFSYGLASKEDGRRVTPETLFEIGSVSKTFTVTLAGYALAQDKMRLDDRASQHWP
PDC-35QDR21260.1_3098PaOC         61  KGEPHYFSYGLASKEDGRRVTPETLFEIGSVSKTFTVTLAGYALAQDKMRLDDRASQHWP
PDC-35QEK40749.1_862PaOC          61  KGEPHYFSYGLASKEDGRRVTPETLFEIGSVSKTFTVTLAGYALAQDKMRLDDRASQHWP
PDC-35AUB00033.3_866PaOC          61  KGEPHYFSYGLASKEDGRRVTPETLFEIGSVSKTFTVTLAGYALAQDKMRLDDRASQHWP
PDC-35QPP34775.1_1311PaOC         61  KGEPHYFSYGLASKEDGRRVTPETLFEIGSVSKTFTVTLAGYALAQDKMRLDDRASQHWP
PDC-35QLF38648.1_891PaOC          61  KGEPHYFSYGLASKEDGRRVTPETLFEIGSVSKTFTVTLAGYALAQDKMRLDDRASQHWP
PDC-35QLF14474.1_2055PaOC         61  KGEPHYFSYGLASKEDGRRVTPETLFEIGSVSKTFTVTLAGYALAQDKMRLDDRASQHWP
PDC-35QQV60864.1_5728PaOC         61  KGEPHYFSYGLASKEDGRRVTPETLFEIGSVSKTFTVTLAGYALAQDKMRLDDRASQHWP
PDC-35QQW01132.1_862PaOC          61  KGEPHYFSYGLASKEDGRRVTPETLFEIGSVSKTFTVTLAGYALAQDKMRLDDRASQHWP
PDC-35QQW17992.1_5232PaOC         61  KGEPHYFSYGLASKEDGRRVTPETLFEIGSVSKTFTVTLAGYALAQDKMRLDDRASQHWP
PDC-35QQW23330.1_862PaOC          61  KGEPHYFSYGLASKEDGRRVTPETLFEIGSVSKTFTVTLAGYALAQDKMRLDDRASQHWP
PDC-35QZE42332.1_862PaOC          61  KGEPHYFSYGLASKEDGRRVTPETLFEIGSVSKTFTVTLAGYALAQDKMRLDDRASQHWP
PDC-35UFM83790.1_826PaOC          61  KGEPHYFSYGLASKEDGRRVTPETLFEIGSVSKTFTVTLAGYALAQDKMRLDDRASQHWP
PDC-35UJC15714.1_892PaOC          61  KGEPHYFSYGLASKEDGRRVTPETLFEIGSVSKTFTVTLAGYALAQDKMRLDDRASQHWP
PDC-35UTR21121.1_931PaOC          61  KGEPHYFSYGLASKEDGRRVTPETLFEIGSVSKTFTVTLAGYALAQDKMRLDDRASQHWP
PDC-35UUP09555.1_853PaOC          61  KGEPHYFSYGLASKEDGRRVTPETLFEIGSVSKTFTVTLAGYALAQDKMRLDDRASQHWP
PDC-35UWG56199.1_885PaOC          61  KGEPHYFSYGLASKEDGRRVTPETLFEIGSVSKTFTVTLAGYALAQDKMRLDDRASQHWP
PDC-43UNT30829.1_824PaOC          61  KGEPHYFSYGLASKEDGRRVTPETLFEIGSVSKTFTATLAGYALAQDKMRLDDRASQHWP
PDC-45ALZ89573.1_855.PaOC         61  KGEPHYFSYGLASKEDGRQVTPETLFEIGSVSKTFTATLAGYALAQDKMRLDDRASQHWP
PDC-45QKL11315.1_849PaOC          61  KGEPHYFSYGLASKEDGRQVTPETLFEIGSVSKTFTATLAGYALAQDKMRLDDRASQHWP
PDC-45QQV94504.1_850PaOC          61  KGEPHYFSYGLASKEDGRQVTPETLFEIGSVSKTFTATLAGYALAQDKMRLDDRASQHWP
PDC-162UJB98759.1_2776PaOC        61  KGEPHYFSYGLASKEDGRQVTPETLFEIGSVSKTFTATLAGYALAQDKMRLDDRASQHWP
PDC-123QYE90948.1_3103PaOC        61  KGEPHYFSYGLASKEDGRQVTPETLFEIGSVSKTFTATLAGYALAQDKMRLDDRASQHWP
PDC-5UXH60990.1_2046PaBCP         61  KGEPHYFSYGLASKEDGRQVTPETLFEIGSVSKTFTATLAGYALAQDKMRLDDRASQHWP
PDC-5WBA37364.1_905PaBCP          61  KGEPHYFSYGLASKEDGRQVTPETLFEIGSVSKTFTATLAGYALAQDKMRLDDRASQHWP
PDC-5WGJ68312.1_842PaBCP          61  KGEPHYFSYGLASKEDGRQVTPETLFEIGSVSKTFTATLAGYALAQDKMRLDDRASQHWP
PDC-5WGT14845.1_882PaBCP          61  KGEPHYFSYGLASKEDGRQVTPETLFEIGSVSKTFTATLAGYALAQDKMRLDDRASQHWP
PDC-5WGX69730.1_872PaBCP          61  KGEPHYFSYGLASKEDGRQVTPETLFEIGSVSKTFTATLAGYALAQDKMRLDDRASQHWP
PDC-5CAI9893238.1_8405]PaBCP      61  KGEPHYFSYGLASKEDGRQVTPETLFEIGSVSKTFTATLAGYALAQDKMRLDDRASQHWP
PDC-5CAI9905252.1_60335]PaBCP     61  KGEPHYFSYGLASKEDGRQVTPETLFEIGSVSKTFTATLAGYALAQDKMRLDDRASQHWP
PDC-5UXH60990.1_2046PaBOC         61  KGEPHYFSYGLASKEDGRQVTPETLFEIGSVSKTFTATLAGYALAQDKMRLDDRASQHWP
PDC-5WBA37364.1_905PaBOC          61  KGEPHYFSYGLASKEDGRQVTPETLFEIGSVSKTFTATLAGYALAQDKMRLDDRASQHWP
PDC-5WGJ68312.1_842PaBOC          61  KGEPHYFSYGLASKEDGRQVTPETLFEIGSVSKTFTATLAGYALAQDKMRLDDRASQHWP
PDC-5WGT14845.1_882PaBOC          61  KGEPHYFSYGLASKEDGRQVTPETLFEIGSVSKTFTATLAGYALAQDKMRLDDRASQHWP
PDC-5WGX69730.1_872PaBOC          61  KGEPHYFSYGLASKEDGRQVTPETLFEIGSVSKTFTATLAGYALAQDKMRLDDRASQHWP
PDC-5CAI9893238.1_8405]PaBOC      61  KGEPHYFSYGLASKEDGRQVTPETLFEIGSVSKTFTATLAGYALAQDKMRLDDRASQHWP
PDC-5CAI9905252.1_60335]PaBOC     61  KGEPHYFSYGLASKEDGRQVTPETLFEIGSVSKTFTATLAGYALAQDKMRLDDRASQHWP
PDC-5ALU48309.1_2211.PaOC         61  KGEPHYFSYGLASKEDGRQVTPETLFEIGSVSKTFTATLAGYALAQDKMRLDDRASQHWP
PDC-5AMX89317.1_4085.PaOC         61  KGEPHYFSYGLASKEDGRQVTPETLFEIGSVSKTFTATLAGYALAQDKMRLDDRASQHWP
PDC-5ATH01452.1_827PaOC           61  KGEPHYFSYGLASKEDGRQVTPETLFEIGSVSKTFTATLAGYALAQDKMRLDDRASQHWP
PDC-5UTN13111.1_830PaOC           61  KGEPHYFSYGLASKEDGRQVTPETLFEIGSVSKTFTATLAGYALAQDKMRLDDRASQHWP
PDC-5UUH88148.1_851PaOC           61  KGEPHYFSYGLASKEDGRQVTPETLFEIGSVSKTFTATLAGYALAQDKMRLDDRASQHWP
PDC-5UUH98459.1_806PaOC           61  KGEPHYFSYGLASKEDGRQVTPETLFEIGSVSKTFTATLAGYALAQDKMRLDDRASQHWP
PDC-5UVH93148.1_837PaOC           61  KGEPHYFSYGLASKEDGRQVTPETLFEIGSVSKTFTATLAGYALAQDKMRLDDRASQHWP
PDC-5UWU62523.1_171PaOC           61  KGEPHYFSYGLASKEDGRQVTPETLFEIGSVSKTFTATLAGYALAQDKMRLDDRASQHWP
PDC-5WDZ01346.1_910PaOC           61  KGEPHYFSYGLASKEDGRQVTPETLFEIGSVSKTFTATLAGYALAQDKMRLDDRASQHWP
PDC-5WEO42644.1_846PaOC           61  KGEPHYFSYGLASKEDGRQVTPETLFEIGSVSKTFTATLAGYALAQDKMRLDDRASQHWP
PDC-5WGW99708.1_849PaOC           61  KGEPHYFSYGLASKEDGRQVTPETLFEIGSVSKTFTATLAGYALAQDKMRLDDRASQHWP
PDC-5WJM42992.1_848PaOC           61  KGEPHYFSYGLASKEDGRQVTPETLFEIGSVSKTFTATLAGYALAQDKMRLDDRASQHWP
PDC-60QWY08308.1_854PaBCP         61  KGEPHYFSYGLASKEDGRQVTPETLFEIGSVSKTFTATLAGYALAQDKMRLDDRASQHWP
PDC-60QZV50082.1_838PaBCP         61  KGEPHYFSYGLASKEDGRQVTPETLFEIGSVSKTFTATLAGYALAQDKMRLDDRASQHWP
PDC-60QWY08308.1_854PaBOC         61  KGEPHYFSYGLASKEDGRQVTPETLFEIGSVSKTFTATLAGYALAQDKMRLDDRASQHWP
PDC-60QZV50082.1_838PaBOC         61  KGEPHYFSYGLASKEDGRQVTPETLFEIGSVSKTFTATLAGYALAQDKMRLDDRASQHWP
PDC-60QPZ60674.1_902PaOC          61  KGEPHYFSYGLASKEDGRQVTPETLFEIGSVSKTFTATLAGYALAQDKMRLDDRASQHWP
PDC-60WAJ92260.1_847PaOC          61  KGEPHYFSYGLASKEDGRQVTPETLFEIGSVSKTFTATLAGYALAQDKMRLDDRASQHWP
PDC-60WAJ98065.1_847PaOC          61  KGEPHYFSYGLASKEDGRQVTPETLFEIGSVSKTFTATLAGYALAQDKMRLDDRASQHWP
PDC-15UNL10022.1_837PaBCP         61  KGEPHYFSYGLASKEDGRQVTPETLFEIGSVSKTFTATLAGYALAQDKMRLDDRASQHWP
PDC-15UNL10022.1_837PaBOC         61  KGEPHYFSYGLASKEDGRQVTPETLFEIGSVSKTFTATLAGYALAQDKMRLDDRASQHWP
PDC-15AOT38867.1_3370PaOC         61  KGEPHYFSYGLASKEDGRQVTPETLFEIGSVSKTFTATLAGYALAQDKMRLDDRASQHWP
PDC-15ALZ27705.1_859PaOC          61  KGEPHYFSYGLASKEDGRQVTPETLFEIGSVSKTFTATLAGYALAQDKMRLDDRASQHWP
PDC-15AXC19915.1_1529PaOC         61  KGEPHYFSYGLASKEDGRQVTPETLFEIGSVSKTFTATLAGYALAQDKMRLDDRASQHWP
PDC-15UTP69334.1_895PaOC          61  KGEPHYFSYGLASKEDGRQVTPETLFEIGSVSKTFTATLAGYALAQDKMRLDDRASQHWP
PDC-15UNK91447.1_837PaOC          61  KGEPHYFSYGLASKEDGRQVTPETLFEIGSVSKTFTATLAGYALAQDKMRLDDRASQHWP
PDC-15UNL03855.1_837PaOC          61  KGEPHYFSYGLASKEDGRQVTPETLFEIGSVSKTFTATLAGYALAQDKMRLDDRASQHWP
PDC-28UYM61272.1_6539PaOC         61  KGEPHYFSYGLASKEDGRQVTPETLFEIGSVSKTFTATLAGYALAQDKMRLDDRASQHWP
PDC-22QPZ72804.1_848PaOC          61  KGEPHYFSYGLASKEDGRQVTPETLFEIGSVSKTFTATLAGYALAQDKMRLDDRASQHWP
PDC-22QQM08170.1_4816PaOC         61  KGEPHYFSYGLASKEDGRQVTPETLFEIGSVSKTFTATLAGYALAQDKMRLDDRASQHWP
PDC-97QKR35301.1_4456PaOC         61  KGEPHYFSYGLASKEDGRRVTPETLFEIGSVSKTFTATLAGYALAQDKMRLDDRASQHWP
PDC-51WGV68048.1_897PaOC          61  KGEPHYFSYGLASKEDGRQVTPETLFEIGSVSKTFTATLAGYALAQDKMRLDDRASQHWP
PDC-1HW06_09835_867PaOC           61  KGEPHYFSYGLASKEDGRRVTPETLFEIGSVSKTFTATLAGYALTQDKMRLDDRASQHWP
PDC-151QQV49515.1_805PaOC         61  KGEPHYFSYGVASKEDARKVTPETLFEIGSVSKTFTATLAGYALAQDKMRLDDRASRHWP
consensus>70                          KGEPHYFSYGlASKEDgRrVTpETLFEIGSVSKtFTaTLAGYALaQDKMRLDDrASqHWP
                               acc
```

PDC-374QKR78821.1_828PaOC η3 η4 α3 TT α4 TT β5

PDC-374QKR78821.1_828PaOC	121	ALQGSRFDGISLLDLATYTAGGLPLQFPDSVQKDQAQIRDYYRQWQPTYAPGSQRLYSNP
PDC-331WCI44612.1_917PaOC	121	ALQGSRFDGISLLDLATYTAGGLPLQFPDSVQKDQAQIRDYYRQWQPTYAPGSQRLYSNP
PDC-8HV97_01835_992PaOC	121	ALQGSRFDGISLLDLATYTAGGLPLQFPDSVQKDQAQIRDYYRQWQPTYAPGSQRRYSNP
PDC-8CLH63_27535_5433PaOC	121	ALQGSRFDGISLLDLATYTAGGLPLQFPDSVQKDQAQIRDYYRQWQPTYAPGSQRRYSNP
PDC-31HW08_20870_899PaOC	121	ALQGSRFDGISLLDLATYTAGGLPLQFPDSVQKDQAQIRDYYRQWQPTYAPGSQRLYSNP
PDC-3CPZ93_04765_942PaBCP	121	ALQGSRFDGISLLDLATYTAGGLPLQFPDSVQKDQAQIRDYYRQWQPTYAPGSQRLYSNP
PDC-3CPZ93_04765_942PaBOC	121	ALQGSRFDGISLLDLATYTAGGLPLQFPDSVQKDQAQIRDYYRQWQPTYAPGSQRLYSNP
PDC-3CD796_04405_870PaOC	121	ALQGSRFDGISLLDLATYTAGGLPLQFPDSVQKDQAQIRDYYRQWQPTYAPGSQRLYSNP
PDC-1HV98_23795_933PaOC	121	ALQGSRFDGISLLDLATYTAGGLPLQFPDSVQKDQAQIRDYYRQWQPTYAPGSQRLYSNP
PDC-103HV95_30355_911PaOC	121	ALQGSRFDGISLLDLATYTAGGLPLQFPDSVQKDQAQIRDYYRQWQPTYAPGSQRLYSNP
PDC-5HW02_17620_927PaOC	121	ALQGSRFDGISLLDLATYTAGGLPLQFPDSVQKDQAQIRDYYRQWQPTYAPGSQRLYSNP
PDC-5HW01_11705_873PaOC	121	ALQGSRFDGISLLDLATYTAGGLPLQFPDSVQKDQAQIRDYYRQWQPTYAPGSQRLYSNP
PDC-5CD800_33195_6567PaOC	121	ALQGSRFDGISLLDLATYTAGGLPLQFPDSVQKDQAQIRDYYRQWQPTYAPGSQRLYSNP
PDC-5CD799_31455_6219PaOC	121	ALQGSRFDGISLLDLATYTAGGLPLQFPDSVQKDQAQIRDYYRQWQPTYAPGSQRLYSNP
PDC-46CEK59_30555_6032PaOC	121	ALQGSRFDGISLLDLATYTAGGLPLQFPDSVQKDQAQIRDYYRQWQPTYAPGSQRLYSNP
PDC-66QTQ98808.1_855PaOC	121	ALQGSRFDGISLLDLATYTAGGLPLQFPDSVQKDQAQIRDYYRQWQPTYTPGSQRLYSNP
PDC-364QYE79191.1_1969PaOC	121	ALQGSRFDGISLLDLATYTAGGLPLQFPDSVQKDQAQIRDYYRQWQPTYTPGSQRLYSNP
PDC-122QYN07359.1_5685PaOC	121	ALQGSRFDGISLLDLATYTAGGLPLQFPDSVQKDQAQIRDYYRQWQPTYAPGSQRLYSNP
PDC-100QKR60216.1_898PaOC	121	ALQGSRFDGISLLDLATYTAGGLPLQFPDSVQKDRVQIRDYYRQWQPTYAPGSQRLYSNP
PDC-34WBM27707.1_865PaBCP	121	ALQGSRFDGISLLDLATYTAGGLPLQFPDSVQKDRAQIRDYYRQWQPTYAPGSQRLYSNP
PDC-34WGX11792.1_865PaBCP	121	ALQGSRFDGISLLDLATYTAGGLPLQFPDSVQKDRAQIRDYYRQWQPTYAPGSQRLYSNP
PDC-34WBM27707.1_865PaBOC	121	ALQGSRFDGISLLDLATYTAGGLPLQFPDSVQKDRAQIRDYYRQWQPTYAPGSQRLYSNP
PDC-34WGX11792.1_865PaBOC	121	ALQGSRFDGISLLDLATYTAGGLPLQFPDSVQKDRAQIRDYYRQWQPTYAPGSQRLYSNP
PDC-34ALY72428.1_939PaOC	121	ALQGSRFDGISLLDLATYTAGGLPLQFPDSVQKDRAQIRDYYRQWQPTYAPGSQRLYSNP
PDC-34ASM83699.1_885PaOC	121	ALQGSRFDGISLLDLATYTAGGLPLQFPDSVQKDRAQIRDYYRQWQPTYAPGSQRLYSNP
PDC-34AYW64445.1_883PaOC	121	ALQGSRFDGISLLDLATYTAGGLPLQFPDSVQKDRAQIRDYYRQWQPTYAPGSQRLYSNP
PDC-34UTR27428.1_860PaOC	121	ALQGSRFDGISLLDLATYTAGGLPLQFPDSVQKDRAQIRDYYRQWQPTYAPGSQRLYSNP
PDC-34UOD92021.1_934PaOC	121	ALQGSRFDGISLLDLATYTAGGLPLQFPDSVQKDRAQIRDYYRQWQPTYAPGSQRLYSNP
PDC-34WIV39806.1_860PaOC	121	ALQGSRFDGISLLDLATYTAGGLPLQFPDSVQKDRAQIRDYYRQWQPTYAPGSQRLYSNP
PDC-264QKR42793.1_833PaOC	121	ALQGSRFDGISLLDLATYTAGGLPLQLPDSVQKDQAQIRDYYRQWQPTYAPGSQRLYSNP
PDC-167QIZ28250.1_4314PaOC	121	ALQGSRFDGISLLDLATYTAGGLPLQFPDSVQKDQAQIRDYYRQWQPTYAPGSQRLYSNP
PDC-167UTQ27575.1_842PaOC	121	ALQGSRFDGISLLDLATYTAGGLPLQFPDSVQKDQAQIRDYYRQWQPTYAPGSQRLYSNP
PDC-6QZV30905.1_832PaBCP	121	ALQGSRFDGISLLDLATYTAGGLPLQFPDSVQKDQAQIRDYYRQWQPTYAPGSQRLYSNP
PDC-6QZV30905.1_832PaBOC	121	ALQGSRFDGISLLDLATYTAGGLPLQFPDSVQKDQAQIRDYYRQWQPTYAPGSQRLYSNP
PDC-37WAJ85675.1_843PaBCP	121	ALQGSRFDGISLLDLATYTAGGLPLQFPDSVQKDQAQIRDYYRQWQPTYAPGSQRLYSNP
PDC-37WGX56158.1_855PaBCP	121	ALQGSRFDGISLLDLATYTAGGLPLQFPDSVQKDQAQIRDYYRQWQPTYAPGSQRLYSNP
PDC-37WAJ85675.1_843PaBOC	121	ALQGSRFDGISLLDLATYTAGGLPLQFPDSVQKDQAQIRDYYRQWQPTYAPGSQRLYSNP
PDC-37WGX56158.1_855PaBOC	121	ALQGSRFDGISLLDLATYTAGGLPLQFPDSVQKDQAQIRDYYRQWQPTYAPGSQRLYSNP
PDC-37ANA71197.1_2757.PaOC	121	ALQGSRFDGISLLDLATYTAGGLPLQFPDSVQKDQAQIRDYYRQWQPTYAPGSQRLYSNP
PDC-37WGX14507.1_3195PaOC	121	ALQGSRFDGISLLDLATYTAGGLPLQFPDSVQKDQAQIRDYYRQWQPTYAPGSQRLYSNP
PDC-46AUA93832.1_897PaOC	121	ALQGSRFDGISLLDLATYTAGGLPLQFPDSVQKDQAQIRDYYRQWQPTYAPGSQRLYSNP
PDC-105QGP95004.1_877PaBCP	121	ALQGSRFDGISLLDLATYTAGGLPLQFPDSVQKDQTQIRDYYRQWQPTYAPGSQRLYSNP
PDC-105QGP95004.1_877PaBOC	121	ALQGSRFDGISLLDLATYTAGGLPLQFPDSVQKDQTQIRDYYRQWQPTYAPGSQRLYSNP
PDC-12AVN43882.1_2061.PaOC	121	ALQGSRFDGISLLDLATYTAGGLPLQFPDSVQKDQAQIRDYYRQWQPTYAPGSQRLYSNP
PDC-12QNE63041.1_859PaOC	121	ALQGSRFDGISLLDLATYTAGGLPLQFPDSVQKDQAQIRDYYRQWQPTYAPGSQRLYSNP
PDC-12WGW93864.1_852PaOC	121	ALQGSRFDGISLLDLATYTAGGLPLQFPDSVQKDQAQIRDYYRQWQPTYAPGSQRLYSNP
PDC-12WGX05587.1_852PaOC	121	ALQGSRFDGISLLDLATYTAGGLPLQFPDSVQKDQAQIRDYYRQWQPTYAPGSQRLYSNP
PDC-11QSA85555.1_5581PaBCP	121	ALQGSRFDGISLLDLATYTAGGLPLQFPDSVQKDQAQIRDYYRQWQPTYAPGSQRLYSNP
PDC-11QSA91467.1_5367PaBCP	121	ALQGSRFDGISLLDLATYTAGGLPLQFPDSVQKDQAQIRDYYRQWQPTYAPGSQRLYSNP
PDC-11QSA85555.1_5581PaBOC	121	ALQGSRFDGISLLDLATYTAGGLPLQFPDSVQKDQAQIRDYYRQWQPTYAPGSQRLYSNP
PDC-11QSA91467.1_5367PaBOC	121	ALQGSRFDGISLLDLATYTAGGLPLQFPDSVQKDQAQIRDYYRQWQPTYAPGSQRLYSNP
PDC-11AYQ83203.1_3491PaOC	121	ALQGSRFDGISLLDLATYTAGGLPLQFPDSVQKDQAQIRDYYRQWQPTYAPGSQRLYSNP
PDC-11AYR15208.1_5205PaOC	121	ALQGSRFDGISLLDLATYTAGGLPLQFPDSVQKDQAQIRDYYRQWQPTYAPGSQRLYSNP
PDC-11AZN02324.1_4787PaOC	121	ALQGSRFDGISLLDLATYTAGGLPLQFPDSVQKDQAQIRDYYRQWQPTYAPGSQRLYSNP
PDC-11AZN04123.1_203PaOC	121	ALQGSRFDGISLLDLATYTAGGLPLQFPDSVQKDQAQIRDYYRQWQPTYAPGSQRLYSNP
PDC-11AZN17071.1_5847PaOC	121	ALQGSRFDGISLLDLATYTAGGLPLQFPDSVQKDQAQIRDYYRQWQPTYAPGSQRLYSNP
PDC-11AZN48510.1_4981PaOC	121	ALQGSRFDGISLLDLATYTAGGLPLQFPDSVQKDQAQIRDYYRQWQPTYAPGSQRLYSNP
PDC-11AZN60564.1_5101PaOC	121	ALQGSRFDGISLLDLATYTAGGLPLQFPDSVQKDQAQIRDYYRQWQPTYAPGSQRLYSNP
PDC-11AZN55779.1_6545PaOC	121	ALQGSRFDGISLLDLATYTAGGLPLQFPDSVQKDQAQIRDYYRQWQPTYAPGSQRLYSNP
PDC-11AYF68870.1_744PaOC	121	ALQGSRFDGISLLDLATYTAGGLPLQFPDSVQKDQAQIRDYYRQWQPTYAPGSQRLYSNP
PDC-11QUS21307.1_2433PaOC	121	ALQGSRFDGISLLDLATYTAGGLPLQFPDSVQKDQAQIRDYYRQWQPTYAPGSQRLYSNP
PDC-11UVH87183.1_982PaOC	121	ALQGSRFDGISLLDLATYTAGGLPLQFPDSVQKDQAQIRDYYRQWQPTYAPGSQRLYSNP
PDC-16QCC64126.1_958PaBCP	121	ALQGSRFDGISLLDLATYTAGGLPLQFPDSVQKDQAQIRDYYRQWQPTYAPGSQRLYSNP
PDC-16UBI70916.1_933PaBCP	121	ALQGSRFDGISLLDLATYTAGGLPLQFPDSVQKDQAQIRDYYRQWQPTYAPGSQRLYSNP
PDC-16UTQ84596.1_855PaBCP	121	ALQGSRFDGISLLDLATYTAGGLPLQFPDSVQKDQAQIRDYYRQWQPTYAPGSQRLYSNP
PDC-16QCC64126.1_958PaBOC	121	ALQGSRFDGISLLDLATYTAGGLPLQFPDSVQKDQAQIRDYYRQWQPTYAPGSQRLYSNP
PDC-16UBI70916.1_933PaBOC	121	ALQGSRFDGISLLDLATYTAGGLPLQFPDSVQKDQAQIRDYYRQWQPTYAPGSQRLYSNP
PDC-16UTQ84596.1_855PaBOC	121	ALQGSRFDGISLLDLATYTAGGLPLQFPDSVQKDQAQIRDYYRQWQPTYAPGSQRLYSNP
PDC-16ALS12949.1_2977.PaOC	121	ALQGSRFDGISLLDLATYTAGGLPLQFPDSVQKDQAQIRDYYRQWQPTYAPGSQRLYSNP
PDC-16AYW58201.1_855PaOC	121	ALQGSRFDGISLLDLATYTAGGLPLQFPDSVQKDQAQIRDYYRQWQPTYAPGSQRLYSNP
PDC-16QDR00398.1_890PaOC	121	ALQGSRFDGISLLDLATYTAGGLPLQFPDSVQKDQAQIRDYYRQWQPTYAPGSQRLYSNP
PDC-16QGJ31628.1_895PaOC	121	ALQGSRFDGISLLDLATYTAGGLPLQFPDSVQKDQAQIRDYYRQWQPTYAPGSQRLYSNP
PDC-16QKG34934.1_1840PaOC	121	ALQGSRFDGISLLDLATYTAGGLPLQFPDSVQKDQAQIRDYYRQWQPTYAPGSQRLYSNP
PDC-16QOQ64874.1_122PaOC	121	ALQGSRFDGISLLDLATYTAGGLPLQFPDSVQKDQAQIRDYYRQWQPTYAPGSQRLYSNP
PDC-16QVQ73157.1_860PaOC	121	ALQGSRFDGISLLDLATYTAGGLPLQFPDSVQKDQAQIRDYYRQWQPTYAPGSQRLYSNP
PDC-16UOC64480.1_827PaOC	121	ALQGSRFDGISLLDLATYTAGGLPLQFPDSVQKDQAQIRDYYRQWQPTYAPGSQRLYSNP
PDC-16UOC70564.1_826PaOC	121	ALQGSRFDGISLLDLATYTAGGLPLQFPDSVQKDQAQIRDYYRQWQPTYAPGSQRLYSNP
PDC-16UOC76649.1_827PaOC	121	ALQGSRFDGISLLDLATYTAGGLPLQFPDSVQKDQAQIRDYYRQWQPTYAPGSQRLYSNP
PDC-16UOC82740.1_827PaOC	121	ALQGSRFDGISLLDLATYTAGGLPLQFPDSVQKDQAQIRDYYRQWQPTYAPGSQRLYSNP
PDC-16UOC88825.1_827PaOC	121	ALQGSRFDGISLLDLATYTAGGLPLQFPDSVQKDQAQIRDYYRQWQPTYAPGSQRLYSNP
PDC-16UOE68701.1_827PaOC	121	ALQGSRFDGISLLDLATYTAGGLPLQFPDSVQKDQAQIRDYYRQWQPTYAPGSQRLYSNP
PDC-16WDC65088.1_844PaOC	121	ALQGSRFDGISLLDLATYTAGGLPLQFPDSVQKDQAQIRDYYRQWQPTYAPGSQRLYSNP
PDC-16WGW70130.1_845PaOC	121	ALQGSRFDGISLLDLATYTAGGLPLQFPDSVQKDQAQIRDYYRQWQPTYAPGSQRLYSNP
PDC-16WGW76010.1_845PaOC	121	ALQGSRFDGISLLDLATYTAGGLPLQFPDSVQKDQAQIRDYYRQWQPTYAPGSQRLYSNP
PDC-16WGW81892.1_845PaOC	121	ALQGSRFDGISLLDLATYTAGGLPLQFPDSVQKDQAQIRDYYRQWQPTYAPGSQRLYSNP
PDC-16WGX43443.1_806PaOC	121	ALQGSRFDGISLLDLATYTAGGLPLQFPDSVQKDQAQIRDYYRQWQPTYAPGSQRLYSNP
PDC-16WJM37210.1_871PaOC	121	ALQGSRFDGISLLDLATYTAGGLPLQFPDSVQKDQAQIRDYYRQWQPTYAPGSQRLYSNP
PDC-59UQZ18314.1_902PaOC	121	ALQGSRFDGISLLDLATYTAGGLPLQFPDSVQKDQAQIRDYYRQWQPTYAPGSQRLYSNP
PDC-142WGW57525.1_830PaBCP	121	ALQGSRFDGISLLDLATYTAGGLPLQFPDSVQKDQAQIRDYYRQWQPTYAPGSQRLYSNP
PDC-142WGW32210.1_830PaBCP	121	ALQGSRFDGISLLDLATYTAGGLPLQFPDSVQKDQAQIRDYYRQWQPTYAPGSQRLYSNP
PDC-142WGW44776.1_830PaBCP	121	ALQGSRFDGISLLDLATYTAGGLPLQFPDSVQKDQAQIRDYYRQWQPTYAPGSQRLYSNP
PDC-142WGW57525.1_830PaBOC	121	ALQGSRFDGISLLDLATYTAGGLPLQFPDSVQKDQAQIRDYYRQWQPTYAPGSQRLYSNP
PDC-142WGW32210.1_830PaBOC	121	ALQGSRFDGISLLDLATYTAGGLPLQFPDSVQKDQAQIRDYYRQWQPTYAPGSQRLYSNP
PDC-142WGW44776.1_830PaBOC	121	ALQGSRFDGISLLDLATYTAGGLPLQFPDSVQKDQAQIRDYYRQWQPTYAPGSQRLYSNP
PDC-142AON05138.1_825PaOC	121	ALQGSRFDGISLLDLATYTAGGLPLQFPDSVQKDQAQIRDYYRQWQPTYAPGSQRLYSNP
PDC-142AON11127.1_825PaOC	121	ALQGSRFDGISLLDLATYTAGGLPLQFPDSVQKDQAQIRDYYRQWQPTYAPGSQRLYSNP
PDC-142AON23122.1_825PaOC	121	ALQGSRFDGISLLDLATYTAGGLPLQFPDSVQKDQAQIRDYYRQWQPTYAPGSQRLYSNP
PDC-142AON29110.1_825PaOC	121	ALQGSRFDGISLLDLATYTAGGLPLQFPDSVQKDQAQIRDYYRQWQPTYAPGSQRLYSNP
PDC-142AON41099.1_825PaOC	121	ALQGSRFDGISLLDLATYTAGGLPLQFPDSVQKDQAQIRDYYRQWQPTYAPGSQRLYSNP
PDC-142AON47112.1_824PaOC	121	ALQGSRFDGISLLDLATYTAGGLPLQFPDSVQKDQAQIRDYYRQWQPTYAPGSQRLYSNP
PDC-142AON17115.1_824PaOC	121	ALQGSRFDGISLLDLATYTAGGLPLQFPDSVQKDQAQIRDYYRQWQPTYAPGSQRLYSNP
PDC-142AON35116.1_825PaOC	121	ALQGSRFDGISLLDLATYTAGGLPLQFPDSVQKDQAQIRDYYRQWQPTYAPGSQRLYSNP
PDC-19aWGW51434.1_1207PaBCP	121	ALQGSRFDGISLLDLATYTAGGLPLQFPDSVQKDQAQIRDYYRQWQPTYAPGSQRLYSNP

```
PDC-19aWGW37666.1_6817PaBCP     121  ALQGSRFDGISLLDLATYTAGGLPLQFPDSVQKDQAQIRDYYRQWQPTYAPGSQRLYSNP
PDC-19aWGW59864.1_3660PaBCP     121  ALQGSRFDGISLLDLATYTAGGLPLQFPDSVQKDQAQIRDYYRQWQPTYAPGSQRLYSNP
PDC-19aWGX49838.1_877PaBCP      121  ALQGSRFDGISLLDLATYTAGGLPLQFPDSVQKDQAQIRDYYRQWQPTYAPGSQRLYSNP
PDC-19aWGX24308.1_877PaBCP      121  ALQGSRFDGISLLDLATYTAGGLPLQFPDSVQKDQAQIRDYYRQWQPTYAPGSQRLYSNP
PDC-19aWGX35552.1_5487PaBCP     121  ALQGSRFDGISLLDLATYTAGGLPLQFPDSVQKDQAQIRDYYRQWQPTYAPGSQRLYSNP
PDC-19aWKA37694.1_2446PaBCP     121  ALQGSRFDGISLLDLATYTAGGLPLQFPDSVQKDQAQIRDYYRQWQPTYAPGSQRLYSNP
PDC-19aWGW51434.1_1207PaBOC     121  ALQGSRFDGISLLDLATYTAGGLPLQFPDSVQKDQAQIRDYYRQWQPTYAPGSQRLYSNP
PDC-19aWGW37666.1_6817PaBOC     121  ALQGSRFDGISLLDLATYTAGGLPLQFPDSVQKDQAQIRDYYRQWQPTYAPGSQRLYSNP
PDC-19aWGW59864.1_3660PaBOC     121  ALQGSRFDGISLLDLATYTAGGLPLQFPDSVQKDQAQIRDYYRQWQPTYAPGSQRLYSNP
PDC-19aWGX49838.1_877PaBOC      121  ALQGSRFDGISLLDLATYTAGGLPLQFPDSVQKDQAQIRDYYRQWQPTYAPGSQRLYSNP
PDC-19aWGX24308.1_877PaBOC      121  ALQGSRFDGISLLDLATYTAGGLPLQFPDSVQKDQAQIRDYYRQWQPTYAPGSQRLYSNP
PDC-19aWGX35552.1_5487PaBOC     121  ALQGSRFDGISLLDLATYTAGGLPLQFPDSVQKDQAQIRDYYRQWQPTYAPGSQRLYSNP
PDC-19aWKA37694.1_2446PaBOC     121  ALQGSRFDGISLLDLATYTAGGLPLQFPDSVQKDQAQIRDYYRQWQPTYAPGSQRLYSNP
PDC-19aAPB63370.1_616PaOC       121  ALQGSRFDGISLLDLATYTAGGLPLQFPDSVQKDQAQIRDYYRQWQPTYAPGSQRLYSNP
PDC-19aARI89529.1_870PaOC       121  ALQGSRFDGISLLDLATYTAGGLPLQFPDSVQKDQAQIRDYYRQWQPTYAPGSQRLYSNP
PDC-19aARI95964.1_871PaOC       121  ALQGSRFDGISLLDLATYTAGGLPLQFPDSVQKDQAQIRDYYRQWQPTYAPGSQRLYSNP
PDC-19aASA13594.1_977PaOC       121  ALQGSRFDGISLLDLATYTAGGLPLQFPDSVQKDQAQIRDYYRQWQPTYAPGSQRLYSNP
PDC-19aASA27558.1_962PaOC       121  ALQGSRFDGISLLDLATYTAGGLPLQFPDSVQKDQAQIRDYYRQWQPTYAPGSQRLYSNP
PDC-19aASD01901.1_971PaOC       121  ALQGSRFDGISLLDLATYTAGGLPLQFPDSVQKDQAQIRDYYRQWQPTYAPGSQRLYSNP
PDC-19aUAC92160.1_908PaOC       121  ALQGSRFDGISLLDLATYTAGGLPLQFPDSVQKDQAQIRDYYRQWQPTYAPGSQRLYSNP
PDC-19aUAC98839.1_942PaOC       121  ALQGSRFDGISLLDLATYTAGGLPLQFPDSVQKDQAQIRDYYRQWQPTYAPGSQRLYSNP
PDC-19aUTQ33604.1_830PaOC       121  ALQGSRFDGISLLDLATYTAGGLPLQFPDSVQKDQAQIRDYYRQWQPTYAPGSQRLYSNP
PDC-19aUOO58611.1_830PaOC       121  ALQGSRFDGISLLDLATYTAGGLPLQFPDSVQKDQAQIRDYYRQWQPTYAPGSQRLYSNP
PDC-19aWDA50910.1_883PaOC       121  ALQGSRFDGISLLDLATYTAGGLPLQFPDSVQKDQAQIRDYYRQWQPTYAPGSQRLYSNP
PDC-19aWGX30960.1_941PaOC       121  ALQGSRFDGISLLDLATYTAGGLPLQFPDSVQKDQAQIRDYYRQWQPTYAPGSQRLYSNP
PDC-19aWGX61448.1_5603PaOC      121  ALQGSRFDGISLLDLATYTAGGLPLQFPDSVQKDQAQIRDYYRQWQPTYAPGSQRLYSNP
PDC-19aWGX82118.1_941PaOC       121  ALQGSRFDGISLLDLATYTAGGLPLQFPDSVQKDQAQIRDYYRQWQPTYAPGSQRLYSNP
PDC-19aWIO49783.1_187PaOC       121  ALQGSRFDGISLLDLATYTAGGLPLQFPDSVQKDQAQIRDYYRQWQPTYAPGSQRLYSNP
PDC-24QZV43725.1_941PaBCP      121  ALQGSRFDGISLLDLATYTAGGLPLQFPDSVQKDQAQIRDYYRQWQPTYAPGSQRLYSNP
PDC-24QZV43725.1_941PaBOC      121  ALQGSRFDGISLLDLATYTAGGLPLQFPDSVQKDQAQIRDYYRQWQPTYAPGSQRLYSNP
PDC-24QDR06751.1_860PaOC       121  ALQGSRFDGISLLDLATYTAGGLPLQFPDSVQKDQAQIRDYYRQWQPTYAPGSQRLYSNP
PDC-24UGR36796.1_3412PaOC      121  ALQGSRFDGISLLDLATYTAGGLPLQFPDSVQKDQAQIRDYYRQWQPTYAPGSQRLYSNP
PDC-24UTQ39921.1_833PaOC       121  ALQGSRFDGISLLDLATYTAGGLPLQFPDSVQKDQAQIRDYYRQWQPTYAPGSQRLYSNP
PDC-137QKE94864.1_852PaOC      121  ALQGSRFDGISLLDLATYTAGGLPLQFPDSVQKDQAQIRDYYRQWQPTYAPGSQRLYSNP
PDC-147UGR30133.1_1950PaOC     121  ALQGSRFDGISLLDLATYTAGGLPLQFPDSVQKDQAQIRDYYRQWQPTYAPGSQRLYSNP
PDC-8AYY41152.1_5352PaBCP      121  ALQGSRFDGISLLDLATYTAGGLPLQFPDSVQKDQAQIRDYYRQWQPTYAPGSQRRYSNP
PDC-8QOE14337.1_211PaBCP       121  ALQGSRFDGISLLDLATYTAGGLPLQFPDSVQKDQAQIRDYYRQWQPTYAPGSQRRYSNP
PDC-8QPN15577.1_911PaBCP       121  ALQGSRFDGISLLDLATYTAGGLPLQFPDSVQKDQAQIRDYYRQWQPTYAPGSQRRYSNP
PDC-8QPN23993.1_908PaBCP       121  ALQGSRFDGISLLDLATYTAGGLPLQFPDSVQKDQAQIRDYYRQWQPTYAPGSQRRYSNP
PDC-8QUE64702.1_909PaBCP       121  ALQGSRFDGISLLDLATYTAGGLPLQFPDSVQKDQAQIRDYYRQWQPTYAPGSQRRYSNP
PDC-8QYA88773.1_2249PaBCP      121  ALQGSRFDGISLLDLATYTAGGLPLQFPDSVQKDQAQIRDYYRQWQPTYAPGSQRRYSNP
PDC-8QYF01720.1_5249PaBCP      121  ALQGSRFDGISLLDLATYTAGGLPLQFPDSVQKDQAQIRDYYRQWQPTYAPGSQRRYSNP
PDC-8QZU99504.1_908PaBCP       121  ALQGSRFDGISLLDLATYTAGGLPLQFPDSVQKDQAQIRDYYRQWQPTYAPGSQRRYSNP
PDC-8QZV05727.1_908PaBCP       121  ALQGSRFDGISLLDLATYTAGGLPLQFPDSVQKDQAQIRDYYRQWQPTYAPGSQRRYSNP
PDC-8QZV12028.1_910PaBCP       121  ALQGSRFDGISLLDLATYTAGGLPLQFPDSVQKDQAQIRDYYRQWQPTYAPGSQRRYSNP
PDC-8QZV18112.1_863PaBCP       121  ALQGSRFDGISLLDLATYTAGGLPLQFPDSVQKDQAQIRDYYRQWQPTYAPGSQRRYSNP
PDC-8QZV24387.1_912PaBCP       121  ALQGSRFDGISLLDLATYTAGGLPLQFPDSVQKDQAQIRDYYRQWQPTYAPGSQRRYSNP
PDC-8QZV68851.1_908PaBCP       121  ALQGSRFDGISLLDLATYTAGGLPLQFPDSVQKDQAQIRDYYRQWQPTYAPGSQRRYSNP
PDC-8QZV81338.1_870PaBCP       121  ALQGSRFDGISLLDLATYTAGGLPLQFPDSVQKDQAQIRDYYRQWQPTYAPGSQRRYSNP
PDC-8QZV87769.1_908PaBCP       121  ALQGSRFDGISLLDLATYTAGGLPLQFPDSVQKDQAQIRDYYRQWQPTYAPGSQRRYSNP
PDC-8QZV94048.1_908PaBCP       121  ALQGSRFDGISLLDLATYTAGGLPLQFPDSVQKDQAQIRDYYRQWQPTYAPGSQRRYSNP
PDC-8UXH53497.1_877PaBCP       121  ALQGSRFDGISLLDLATYTAGGLPLQFPDSVQKDQAQIRDYYRQWQPTYAPGSQRRYSNP
PDC-8UXH66521.1_877PaBCP       121  ALQGSRFDGISLLDLATYTAGGLPLQFPDSVQKDQAQIRDYYRQWQPTYAPGSQRRYSNP
PDC-8UXH70664.1_4987PaBCP      121  ALQGSRFDGISLLDLATYTAGGLPLQFPDSVQKDQAQIRDYYRQWQPTYAPGSQRRYSNP
PDC-8UUW81479.1_5273PaBCP      121  ALQGSRFDGISLLDLATYTAGGLPLQFPDSVQKDQAQIRDYYRQWQPTYAPGSQRRYSNP
PDC-8WDM07130.1_910PaBCP       121  ALQGSRFDGISLLDLATYTAGGLPLQFPDSVQKDQAQIRDYYRQWQPTYAPGSQRRYSNP
PDC-8WHM62936.1_909PaBCP       121  ALQGSRFDGISLLDLATYTAGGLPLQFPDSVQKDQAQIRDYYRQWQPTYAPGSQRRYSNP
PDC-8WHM69354.1_909PaBCP       121  ALQGSRFDGISLLDLATYTAGGLPLQFPDSVQKDQAQIRDYYRQWQPTYAPGSQRRYSNP
PDC-8WHM75768.1_909PaBCP       121  ALQGSRFDGISLLDLATYTAGGLPLQFPDSVQKDQAQIRDYYRQWQPTYAPGSQRRYSNP
PDC-8WHM84263.1_909PaBCP       121  ALQGSRFDGISLLDLATYTAGGLPLQFPDSVQKDQAQIRDYYRQWQPTYAPGSQRRYSNP
PDC-8AYY41152.1_5352PaBOC      121  ALQGSRFDGISLLDLATYTAGGLPLQFPDSVQKDQAQIRDYYRQWQPTYAPGSQRRYSNP
PDC-8QOE14337.1_211PaBOC       121  ALQGSRFDGISLLDLATYTAGGLPLQFPDSVQKDQAQIRDYYRQWQPTYAPGSQRRYSNP
PDC-8QPN15577.1_911PaBOC       121  ALQGSRFDGISLLDLATYTAGGLPLQFPDSVQKDQAQIRDYYRQWQPTYAPGSQRRYSNP
PDC-8QPN23993.1_908PaBOC       121  ALQGSRFDGISLLDLATYTAGGLPLQFPDSVQKDQAQIRDYYRQWQPTYAPGSQRRYSNP
PDC-8QUE64702.1_909PaBOC       121  ALQGSRFDGISLLDLATYTAGGLPLQFPDSVQKDQAQIRDYYRQWQPTYAPGSQRRYSNP
PDC-8QYA88773.1_2249PaBOC      121  ALQGSRFDGISLLDLATYTAGGLPLQFPDSVQKDQAQIRDYYRQWQPTYAPGSQRRYSNP
PDC-8QYF01720.1_5249PaBOC      121  ALQGSRFDGISLLDLATYTAGGLPLQFPDSVQKDQAQIRDYYRQWQPTYAPGSQRRYSNP
PDC-8QZU99504.1_908PaBOC       121  ALQGSRFDGISLLDLATYTAGGLPLQFPDSVQKDQAQIRDYYRQWQPTYAPGSQRRYSNP
PDC-8QZV05727.1_908PaBOC       121  ALQGSRFDGISLLDLATYTAGGLPLQFPDSVQKDQAQIRDYYRQWQPTYAPGSQRRYSNP
PDC-8QZV12028.1_910PaBOC       121  ALQGSRFDGISLLDLATYTAGGLPLQFPDSVQKDQAQIRDYYRQWQPTYAPGSQRRYSNP
PDC-8QZV18112.1_863PaBOC       121  ALQGSRFDGISLLDLATYTAGGLPLQFPDSVQKDQAQIRDYYRQWQPTYAPGSQRRYSNP
PDC-8QZV24387.1_912PaBOC       121  ALQGSRFDGISLLDLATYTAGGLPLQFPDSVQKDQAQIRDYYRQWQPTYAPGSQRRYSNP
PDC-8QZV68851.1_908PaBOC       121  ALQGSRFDGISLLDLATYTAGGLPLQFPDSVQKDQAQIRDYYRQWQPTYAPGSQRRYSNP
PDC-8QZV81338.1_870PaBOC       121  ALQGSRFDGISLLDLATYTAGGLPLQFPDSVQKDQAQIRDYYRQWQPTYAPGSQRRYSNP
PDC-8QZV87769.1_908PaBOC       121  ALQGSRFDGISLLDLATYTAGGLPLQFPDSVQKDQAQIRDYYRQWQPTYAPGSQRRYSNP
PDC-8QZV94048.1_908PaBOC       121  ALQGSRFDGISLLDLATYTAGGLPLQFPDSVQKDQAQIRDYYRQWQPTYAPGSQRRYSNP
PDC-8UXH53497.1_877PaBOC       121  ALQGSRFDGISLLDLATYTAGGLPLQFPDSVQKDQAQIRDYYRQWQPTYAPGSQRRYSNP
PDC-8UXH66521.1_877PaBOC       121  ALQGSRFDGISLLDLATYTAGGLPLQFPDSVQKDQAQIRDYYRQWQPTYAPGSQRRYSNP
PDC-8UXH70664.1_4987PaBOC      121  ALQGSRFDGISLLDLATYTAGGLPLQFPDSVQKDQAQIRDYYRQWQPTYAPGSQRRYSNP
PDC-8UUW81479.1_5273PaBOC      121  ALQGSRFDGISLLDLATYTAGGLPLQFPDSVQKDQAQIRDYYRQWQPTYAPGSQRRYSNP
PDC-8WDM07130.1_910PaBOC       121  ALQGSRFDGISLLDLATYTAGGLPLQFPDSVQKDQAQIRDYYRQWQPTYAPGSQRRYSNP
PDC-8WHM62936.1_909PaBOC       121  ALQGSRFDGISLLDLATYTAGGLPLQFPDSVQKDQAQIRDYYRQWQPTYAPGSQRRYSNP
PDC-8WHM69354.1_909PaBOC       121  ALQGSRFDGISLLDLATYTAGGLPLQFPDSVQKDQAQIRDYYRQWQPTYAPGSQRRYSNP
PDC-8WHM75768.1_909PaBOC       121  ALQGSRFDGISLLDLATYTAGGLPLQFPDSVQKDQAQIRDYYRQWQPTYAPGSQRRYSNP
PDC-8WHM84263.1_909PaBOC       121  ALQGSRFDGISLLDLATYTAGGLPLQFPDSVQKDQAQIRDYYRQWQPTYAPGSQRRYSNP
PDC-8AMA35328.1_919.PaOC       121  ALQGSRFDGISLLDLATYTAGGLPLQFPDSVQKDQAQIRDYYRQWQPTYAPGSQRRYSNP
PDC-8ANI06966.1_119PaOC        121  ALQGSRFDGISLLDLATYTAGGLPLQFPDSVQKDQAQIRDYYRQWQPTYAPGSQRRYSNP
PDC-8AYW70752.1_952PaOC        121  ALQGSRFDGISLLDLATYTAGGLPLQFPDSVQKDQAQIRDYYRQWQPTYAPGSQRRYSNP
PDC-8AYZ76972.1_2272PaOC       121  ALQGSRFDGISLLDLATYTAGGLPLQFPDSVQKDQAQIRDYYRQWQPTYAPGSQRRYSNP
PDC-8QKP85289.1_778PaOC        121  ALQGSRFDGISLLDLATYTAGGLPLQFPDSVQKDQAQIRDYYRQWQPTYAPGSQRRYSNP
PDC-8QKR30573.1_5806PaOC       121  ALQGSRFDGISLLDLATYTAGGLPLQFPDSVQKDQAQIRDYYRQWQPTYAPGSQRRYSNP
PDC-8QLF19879.1_939PaOC        121  ALQGSRFDGISLLDLATYTAGGLPLQFPDSVQKDQAQIRDYYRQWQPTYAPGSQRRYSNP
PDC-8QYE59452.1_5107PaOC       121  ALQGSRFDGISLLDLATYTAGGLPLQFPDSVQKDQAQIRDYYRQWQPTYAPGSQRRYSNP
PDC-8QZV98104.1_4956PaOC       121  ALQGSRFDGISLLDLATYTAGGLPLQFPDSVQKDQAQIRDYYRQWQPTYAPGSQRRYSNP
PDC-8UFK73541.1_1068PaOC       121  ALQGSRFDGISLLDLATYTAGGLPLQFPDSVQKDQAQIRDYYRQWQPTYAPGSQRRYSNP
PDC-8UGR33975.2_368PaOC        121  ALQGSRFDGISLLDLATYTAGGLPLQFPDSVQKDQAQIRDYYRQWQPTYAPGSQRRYSNP
PDC-8UTN26696.1_862PaOC        121  ALQGSRFDGISLLDLATYTAGGLPLQFPDSVQKDQAQIRDYYRQWQPTYAPGSQRRYSNP
PDC-8UTQ91314.1_910PaOC        121  ALQGSRFDGISLLDLATYTAGGLPLQFPDSVQKDQAQIRDYYRQWQPTYAPGSQRRYSNP
PDC-8UNK85299.1_868PaOC        121  ALQGSRFDGISLLDLATYTAGGLPLQFPDSVQKDQAQIRDYYRQWQPTYAPGSQRRYSNP
PDC-8UNK97662.1_866PaOC        121  ALQGSRFDGISLLDLATYTAGGLPLQFPDSVQKDQAQIRDYYRQWQPTYAPGSQRRYSNP
PDC-8USI83494.1_833PaOC        121  ALQGSRFDGISLLDLATYTAGGLPLQFPDSVQKDQAQIRDYYRQWQPTYAPGSQRRYSNP
PDC-8WAO16415.1_5521PaOC       121  ALQGSRFDGISLLDLATYTAGGLPLQFPDSVQKDQAQIRDYYRQWQPTYAPGSQRRYSNP
PDC-98QZD62144.1_4346PaBCP     121  ALQGSRFDGISLLDLATYTAGGLPLQFPDSVQKDQAQIRDYYRQWQPTYAPGSQRRYSNP
```

```
PDC-98QZH48390.1_4214PaBCP       121   ALQGSRFDGISLLDLATYTAGGLPLQFPDSVQKDQAQIRDYYRQWQPTYAPGSQRRYSNP
PDC-98QZD62144.1_4346PaBOC       121   ALQGSRFDGISLLDLATYTAGGLPLQFPDSVQKDQAQIRDYYRQWQPTYAPGSQRRYSNP
PDC-98QZH48390.1_4214PaBOC       121   ALQGSRFDGISLLDLATYTAGGLPLQFPDSVQKDQAQIRDYYRQWQPTYAPGSQRRYSNP
PDC-98ALP56563.1_1203.PaOC       121   ALQGSRFDGISLLDLATYTAGGLPLQFPDSVQKDQAQIRDYYRQWQPTYAPGSQRRYSNP
PDC-98UTL97623.1_830PaOC         121   ALQGSRFDGISLLDLATYTAGGLPLQFPDSVQKDQAQIRDYYRQWQPTYAPGSQRRYSNP
PDC-457UTN40061.1_863PaOC        121   ALQGSRFDGISLLDLATYTAGGLPLQFPDSVQKDQAQIRDYYRQWQPTYAPGSQRLYSNP
PDC-23UXI89283.1_830PaOC         121   ALQGSRFDGISLLDLATYTAGGLPLQFPDSVQKDRAQIRDYYRQWQPTYAPGSQRLYSNP
PDC-245AUA75408.1_866PaOC        121   ALQGSRFDGISLLDLATYTAGGLPLQFPDSVQKDQAQIRDYYRQWQPTYAPGSQRLYSNP
PDC-31QZW06655.1_840PaBCP        121   ALQGSRFDGISLLDLATYTAGGLPLQFPDSVQKDQAQIRDYYRQWQPTYAPGSQRLYSNP
PDC-31QZW06655.1_840PaBOC        121   ALQGSRFDGISLLDLATYTAGGLPLQFPDSVQKDQAQIRDYYRQWQPTYAPGSQRLYSNP
PDC-31AXN27248.1_931PaOC         121   ALQGSRFDGISLLDLATYTAGGLPLQFPDSVQKDQAQIRDYYRQWQPTYAPGSQRLYSNP
PDC-31QBC07904.1_3021PaOC        121   ALQGSRFDGISLLDLATYTAGGLPLQFPDSVQKDQAQIRDYYRQWQPTYAPGSQRLYSNP
PDC-31UFM90275.1_905PaOC         121   ALQGSRFDGISLLDLATYTAGGLPLQFPDSVQKDQAQIRDYYRQWQPTYAPGSQRLYSNP
PDC-31UFM98870.1_905PaOC         121   ALQGSRFDGISLLDLATYTAGGLPLQFPDSVQKDQAQIRDYYRQWQPTYAPGSQRLYSNP
PDC-31UNT25139.1_901PaOC         121   ALQGSRFDGISLLDLATYTAGGLPLQFPDSVQKDQAQIRDYYRQWQPTYAPGSQRLYSNP
PDC-31UWJ96607.1_4267PaOC        121   ALQGSRFDGISLLDLATYTAGGLPLQFPDSVQKDQAQIRDYYRQWQPTYAPGSQRLYSNP
PDC-31WGX88300.1_823PaOC         121   ALQGSRFDGISLLDLATYTAGGLPLQFPDSVQKDQAQIRDYYRQWQPTYAPGSQRLYSNP
PDC-31WHM08550.1_859PaOC         121   ALQGSRFDGISLLDLATYTAGGLPLQFPDSVQKDQAQIRDYYRQWQPTYAPGSQRLYSNP
PDC-39AYK21377.1_872PaOC         121   ALQGSRFDGISLLDLATYTAGGLPLQFPDSVQKDQAQIRDYYRQWQPTYAPGSQRLYSNP
PDC-39UJC21946.1_837PaOC         121   ALQGSRFDGISLLDLATYTAGGLPLQFPDSVQKDQAQIRDYYRQWQPTYAPGSQRLYSNP
PDC-30UPL39337.1_928PaBCP        121   ALQGSRFDGISLLDLATYTAGGLPLQFPDSVQKDQAQIRDYYRQWQPTYAPGSQRLYSNP
PDC-30UPL39337.1_928PaBOC        121   ALQGSRFDGISLLDLATYTAGGLPLQFPDSVQKDQAQIRDYYRQWQPTYAPGSQRLYSNP
PDC-30UHL95932.1_5029PaOC        121   ALQGSRFDGISLLDLATYTAGGLPLQFPDSVQKDQAQIRDYYRQWQPTYAPGSQRLYSNP
PDC-30UML99445.1_838PaOC         121   ALQGSRFDGISLLDLATYTAGGLPLQFPDSVQKDQAQIRDYYRQWQPTYAPGSQRLYSNP
PDC-80WAJ79202.1_935PaOC         121   ALQGSRFDGISLLDLATYTAGGLPLQFPDSVQKDQAQIRDYYRQWQPTYAPGSQRLYSNP
8SDL_1|Chain                     121   ALQGSRFDGISLLDLATYTAGGLPLQFPDSVQKDQAQIRDYYRQWQPTYAPGSQRLYSNP
PDC-3UTN33167.1_589PaBCP         121   ALQGSRFDGISLLDLATYTAGGLPLQFPDSVQKDQAQIRDYYRQWQPTYAPGSQRLYSNP
PDC-3WCI38158.1_984PaBCP         121   ALQGSRFDGISLLDLATYTAGGLPLQFPDSVQKDQAQIRDYYRQWQPTYAPGSQRLYSNP
PDC-3WCI50909.1_867PaBCP         121   ALQGSRFDGISLLDLATYTAGGLPLQFPDSVQKDQAQIRDYYRQWQPTYAPGSQRLYSNP
PDC-3WCI90621.1_961PaBCP         121   ALQGSRFDGISLLDLATYTAGGLPLQFPDSVQKDQAQIRDYYRQWQPTYAPGSQRLYSNP
PDC-3WEO48793.1_832PaBCP         121   ALQGSRFDGISLLDLATYTAGGLPLQFPDSVQKDQAQIRDYYRQWQPTYAPGSQRLYSNP
PDC-3CAI9907424.1_19963]PaBCP    121   ALQGSRFDGISLLDLATYTAGGLPLQFPDSVQKDQAQIRDYYRQWQPTYAPGSQRLYSNP
PDC-3UTN33167.1_589PaBOC         121   ALQGSRFDGISLLDLATYTAGGLPLQFPDSVQKDQAQIRDYYRQWQPTYAPGSQRLYSNP
PDC-3WCI38158.1_984PaBOC         121   ALQGSRFDGISLLDLATYTAGGLPLQFPDSVQKDQAQIRDYYRQWQPTYAPGSQRLYSNP
PDC-3WCI50909.1_867PaBOC         121   ALQGSRFDGISLLDLATYTAGGLPLQFPDSVQKDQAQIRDYYRQWQPTYAPGSQRLYSNP
PDC-3WCI90621.1_961PaBOC         121   ALQGSRFDGISLLDLATYTAGGLPLQFPDSVQKDQAQIRDYYRQWQPTYAPGSQRLYSNP
PDC-3WEO48793.1_832PaBOC         121   ALQGSRFDGISLLDLATYTAGGLPLQFPDSVQKDQAQIRDYYRQWQPTYAPGSQRLYSNP
PDC-3CAI9907424.1_19963]PaBOC    121   ALQGSRFDGISLLDLATYTAGGLPLQFPDSVQKDQAQIRDYYRQWQPTYAPGSQRLYSNP
PDC-3AHA17954.1_839.PaOC         121   ALQGSRFDGISLLDLATYTAGGLPLQFPDSVQKDQAQIRDYYRQWQPTYAPGSQRLYSNP
PDC-3ALE46683.1_840.PaOC         121   ALQGSRFDGISLLDLATYTAGGLPLQFPDSVQKDQAQIRDYYRQWQPTYAPGSQRLYSNP
PDC-3ARC78082.1_863.PaOC         121   ALQGSRFDGISLLDLATYTAGGLPLQFPDSVQKDQAQIRDYYRQWQPTYAPGSQRLYSNP
PDC-3ARG53386.1_5885PaOC         121   ALQGSRFDGISLLDLATYTAGGLPLQFPDSVQKDQAQIRDYYRQWQPTYAPGSQRLYSNP
PDC-3WAC79950.1_4482PaOC         121   ALQGSRFDGISLLDLATYTAGGLPLQFPDSVQKDQAQIRDYYRQWQPTYAPGSQRLYSNP
PDC-3WAE22012.1_4863PaOC         121   ALQGSRFDGISLLDLATYTAGGLPLQFPDSVQKDQAQIRDYYRQWQPTYAPGSQRLYSNP
PDC-3WAE29433.1_777PaOC          121   ALQGSRFDGISLLDLATYTAGGLPLQFPDSVQKDQAQIRDYYRQWQPTYAPGSQRLYSNP
PDC-3WAW42145.1_847PaOC          121   ALQGSRFDGISLLDLATYTAGGLPLQFPDSVQKDQAQIRDYYRQWQPTYAPGSQRLYSNP
PDC-3WCI57477.1_911PaOC          121   ALQGSRFDGISLLDLATYTAGGLPLQFPDSVQKDQAQIRDYYRQWQPTYAPGSQRLYSNP
PDC-3WCI77402.1_900PaOC          121   ALQGSRFDGISLLDLATYTAGGLPLQFPDSVQKDQAQIRDYYRQWQPTYAPGSQRLYSNP
PDC-3WCI64002.1_913PaOC          121   ALQGSRFDGISLLDLATYTAGGLPLQFPDSVQKDQAQIRDYYRQWQPTYAPGSQRLYSNP
PDC-3WCI83994.1_905PaOC          121   ALQGSRFDGISLLDLATYTAGGLPLQFPDSVQKDQAQIRDYYRQWQPTYAPGSQRLYSNP
PDC-3WJM48814.1_842PaOC          121   ALQGSRFDGISLLDLATYTAGGLPLQFPDSVQKDQAQIRDYYRQWQPTYAPGSQRLYSNP
PDC-374ANP60834.1_3741PaBCP      121   ALQGSRFDGISLLDLATYTAGGLPLQFPDSVQKDQAQIRDYYRQWQPTYAPGSQRLYSNP
PDC-374AYZ86971.1_5950PaBCP      121   ALQGSRFDGISLLDLATYTAGGLPLQFPDSVQKDQAQIRDYYRQWQPTYAPGSQRLYSNP
PDC-374QZV75115.1_843PaBCP       121   ALQGSRFDGISLLDLATYTAGGLPLQFPDSVQKDQAQIRDYYRQWQPTYAPGSQRLYSNP
PDC-374QZW12711.1_843PaBCP       121   ALQGSRFDGISLLDLATYTAGGLPLQFPDSVQKDQAQIRDYYRQWQPTYAPGSQRLYSNP
PDC-374UEG06685.1_907PaBCP       121   ALQGSRFDGISLLDLATYTAGGLPLQFPDSVQKDQAQIRDYYRQWQPTYAPGSQRLYSNP
PDC-374UGW95802.1_984PaBCP       121   ALQGSRFDGISLLDLATYTAGGLPLQFPDSVQKDQAQIRDYYRQWQPTYAPGSQRLYSNP
PDC-374WGW26222.1_839PaBCP       121   ALQGSRFDGISLLDLATYTAGGLPLQFPDSVQKDQAQIRDYYRQWQPTYAPGSQRLYSNP
PDC-374ANP60834.1_3741PaBOC      121   ALQGSRFDGISLLDLATYTAGGLPLQFPDSVQKDQAQIRDYYRQWQPTYAPGSQRLYSNP
PDC-374AYZ86971.1_5950PaBOC      121   ALQGSRFDGISLLDLATYTAGGLPLQFPDSVQKDQAQIRDYYRQWQPTYAPGSQRLYSNP
PDC-374QZV75115.1_843PaBOC       121   ALQGSRFDGISLLDLATYTAGGLPLQFPDSVQKDQAQIRDYYRQWQPTYAPGSQRLYSNP
PDC-374QZW12711.1_843PaBOC       121   ALQGSRFDGISLLDLATYTAGGLPLQFPDSVQKDQAQIRDYYRQWQPTYAPGSQRLYSNP
PDC-374UEG06685.1_907PaBOC       121   ALQGSRFDGISLLDLATYTAGGLPLQFPDSVQKDQAQIRDYYRQWQPTYAPGSQRLYSNP
PDC-374UGW95802.1_984PaBOC       121   ALQGSRFDGISLLDLATYTAGGLPLQFPDSVQKDQAQIRDYYRQWQPTYAPGSQRLYSNP
PDC-374WGW26222.1_839PaBOC       121   ALQGSRFDGISLLDLATYTAGGLPLQFPDSVQKDQAQIRDYYRQWQPTYAPGSQRLYSNP
PDC-374AOP56366.1_851PaOC        121   ALQGSRFDGISLLDLATYTAGGLPLQFPDSVQKDQAQIRDYYRQWQPTYAPGSQRLYSNP
PDC-374AVE31445.1_873.PaOC       121   ALQGSRFDGISLLDLATYTAGGLPLQFPDSVQKDQAQIRDYYRQWQPTYAPGSQRLYSNP
PDC-374QAS71510.1_873PaOC        121   ALQGSRFDGISLLDLATYTAGGLPLQFPDSVQKDQAQIRDYYRQWQPTYAPGSQRLYSNP
PDC-374QDD38027.1_4463PaOC       121   ALQGSRFDGISLLDLATYTAGGLPLQFPDSVQKDQAQIRDYYRQWQPTYAPGSQRLYSNP
PDC-374QKF05339.1_5830PaOC       121   ALQGSRFDGISLLDLATYTAGGLPLQFPDSVQKDQAQIRDYYRQWQPTYAPGSQRLYSNP
PDC-374QKK83983.1_853PaOC        121   ALQGSRFDGISLLDLATYTAGGLPLQFPDSVQKDQAQIRDYYRQWQPTYAPGSQRLYSNP
PDC-374QKS41466.1_4199PaOC       121   ALQGSRFDGISLLDLATYTAGGLPLQFPDSVQKDQAQIRDYYRQWQPTYAPGSQRLYSNP
PDC-374QPZ80284.1_873PaOC        121   ALQGSRFDGISLLDLATYTAGGLPLQFPDSVQKDQAQIRDYYRQWQPTYAPGSQRLYSNP
PDC-374QQW07466.1_922PaOC        121   ALQGSRFDGISLLDLATYTAGGLPLQFPDSVQKDQAQIRDYYRQWQPTYAPGSQRLYSNP
PDC-374QZU93305.1_843PaOC        121   ALQGSRFDGISLLDLATYTAGGLPLQFPDSVQKDQAQIRDYYRQWQPTYAPGSQRLYSNP
PDC-374UAC85653.1_934PaOC        121   ALQGSRFDGISLLDLATYTAGGLPLQFPDSVQKDQAQIRDYYRQWQPTYAPGSQRLYSNP
PDC-374UGR20250.1_3485PaOC       121   ALQGSRFDGISLLDLATYTAGGLPLQFPDSVQKDQAQIRDYYRQWQPTYAPGSQRLYSNP
PDC-374UIU12727.1_851PaOC        121   ALQGSRFDGISLLDLATYTAGGLPLQFPDSVQKDQAQIRDYYRQWQPTYAPGSQRLYSNP
PDC-374UJC03215.1_956PaOC        121   ALQGSRFDGISLLDLATYTAGGLPLQFPDSVQKDQAQIRDYYRQWQPTYAPGSQRLYSNP
PDC-374UJC09425.1_959PaOC        121   ALQGSRFDGISLLDLATYTAGGLPLQFPDSVQKDQAQIRDYYRQWQPTYAPGSQRLYSNP
PDC-374UJF57976.1_829PaOC        121   ALQGSRFDGISLLDLATYTAGGLPLQFPDSVQKDQAQIRDYYRQWQPTYAPGSQRLYSNP
PDC-374UJF63613.1_829PaOC        121   ALQGSRFDGISLLDLATYTAGGLPLQFPDSVQKDQAQIRDYYRQWQPTYAPGSQRLYSNP
PDC-374UJF68041.1_5361PaOC       121   ALQGSRFDGISLLDLATYTAGGLPLQFPDSVQKDQAQIRDYYRQWQPTYAPGSQRLYSNP
PDC-374UJF74874.1_829PaOC        121   ALQGSRFDGISLLDLATYTAGGLPLQFPDSVQKDQAQIRDYYRQWQPTYAPGSQRLYSNP
PDC-374UJF80517.1_829PaOC        121   ALQGSRFDGISLLDLATYTAGGLPLQFPDSVQKDQAQIRDYYRQWQPTYAPGSQRLYSNP
PDC-374UJF86151.1_829PaOC        121   ALQGSRFDGISLLDLATYTAGGLPLQFPDSVQKDQAQIRDYYRQWQPTYAPGSQRLYSNP
PDC-374UJF90590.1_5359PaOC       121   ALQGSRFDGISLLDLATYTAGGLPLQFPDSVQKDQAQIRDYYRQWQPTYAPGSQRLYSNP
PDC-374UJF97432.1_830PaOC        121   ALQGSRFDGISLLDLATYTAGGLPLQFPDSVQKDQAQIRDYYRQWQPTYAPGSQRLYSNP
PDC-374UJG01877.1_5359PaOC       121   ALQGSRFDGISLLDLATYTAGGLPLQFPDSVQKDQAQIRDYYRQWQPTYAPGSQRLYSNP
PDC-374UJG08704.1_829PaOC        121   ALQGSRFDGISLLDLATYTAGGLPLQFPDSVQKDQAQIRDYYRQWQPTYAPGSQRLYSNP
PDC-374UJG14426.1_829PaOC        121   ALQGSRFDGISLLDLATYTAGGLPLQFPDSVQKDQAQIRDYYRQWQPTYAPGSQRLYSNP
PDC-374UJG20129.1_829PaOC        121   ALQGSRFDGISLLDLATYTAGGLPLQFPDSVQKDQAQIRDYYRQWQPTYAPGSQRLYSNP
PDC-374UJG25828.1_829PaOC        121   ALQGSRFDGISLLDLATYTAGGLPLQFPDSVQKDQAQIRDYYRQWQPTYAPGSQRLYSNP
PDC-374UJG31693.1_884PaOC        121   ALQGSRFDGISLLDLATYTAGGLPLQFPDSVQKDQAQIRDYYRQWQPTYAPGSQRLYSNP
PDC-374UJG36239.1_5358PaOC       121   ALQGSRFDGISLLDLATYTAGGLPLQFPDSVQKDQAQIRDYYRQWQPTYAPGSQRLYSNP
PDC-374UGR51117.1_68PaOC         121   ALQGSRFDGISLLDLATYTAGGLPLQFPDSVQKDQAQIRDYYRQWQPTYAPGSQRLYSNP
PDC-374UON75698.1_5570PaOC       121   ALQGSRFDGISLLDLATYTAGGLPLQFPDSVQKDQAQIRDYYRQWQPTYAPGSQRLYSNP
PDC-374UPG04116.1_3174PaOC       121   ALQGSRFDGISLLDLATYTAGGLPLQFPDSVQKDQAQIRDYYRQWQPTYAPGSQRLYSNP
PDC-374UPZ07602.1_2096PaOC       121   ALQGSRFDGISLLDLATYTAGGLPLQFPDSVQKDQAQIRDYYRQWQPTYAPGSQRLYSNP
PDC-374URM55875.1_5937PaOC       121   ALQGSRFDGISLLDLATYTAGGLPLQFPDSVQKDQAQIRDYYRQWQPTYAPGSQRLYSNP
PDC-374USV13808.1_3922PaOC       121   ALQGSRFDGISLLDLATYTAGGLPLQFPDSVQKDQAQIRDYYRQWQPTYAPGSQRLYSNP
PDC-374USV19196.1_4087PaOC       121   ALQGSRFDGISLLDLATYTAGGLPLQFPDSVQKDQAQIRDYYRQWQPTYAPGSQRLYSNP
PDC-374USX84542.1_851PaOC        121   ALQGSRFDGISLLDLATYTAGGLPLQFPDSVQKDQAQIRDYYRQWQPTYAPGSQRLYSNP
```

```
PDC-374USX78872.1_851PaOC      121  ALQGSRFDGISLLDLATYTAGGLPLQFPDSVQKDQAQIRDYYRQWQPTYAPGSQRLYSNP
PDC-374UUJ85605.1_1747PaOC     121  ALQGSRFDGISLLDLATYTAGGLPLQFPDSVQKDQAQIRDYYRQWQPTYAPGSQRLYSNP
PDC-374UVS71982.1_4220PaOC     121  ALQGSRFDGISLLDLATYTAGGLPLQFPDSVQKDQAQIRDYYRQWQPTYAPGSQRLYSNP
PDC-374UWI85046.1_4210PaOC     121  ALQGSRFDGISLLDLATYTAGGLPLQFPDSVQKDQAQIRDYYRQWQPTYAPGSQRLYSNP
PDC-374UWI92968.1_851PaOC      121  ALQGSRFDGISLLDLATYTAGGLPLQFPDSVQKDQAQIRDYYRQWQPTYAPGSQRLYSNP
PDC-374UXA22714.1_4203PaOC     121  ALQGSRFDGISLLDLATYTAGGLPLQFPDSVQKDQAQIRDYYRQWQPTYAPGSQRLYSNP
PDC-374UXA30656.1_926PaOC      121  ALQGSRFDGISLLDLATYTAGGLPLQFPDSVQKDQAQIRDYYRQWQPTYAPGSQRLYSNP
PDC-374UXA44988.1_926PaOC      121  ALQGSRFDGISLLDLATYTAGGLPLQFPDSVQKDQAQIRDYYRQWQPTYAPGSQRLYSNP
PDC-374UXN39470.1_4222PaOC     121  ALQGSRFDGISLLDLATYTAGGLPLQFPDSVQKDQAQIRDYYRQWQPTYAPGSQRLYSNP
PDC-374UZA87382.1_4980PaOC     121  ALQGSRFDGISLLDLATYTAGGLPLQFPDSVQKDQAQIRDYYRQWQPTYAPGSQRLYSNP
PDC-374WBM66401.1_846PaOC      121  ALQGSRFDGISLLDLATYTAGGLPLQFPDSVQKDQAQIRDYYRQWQPTYAPGSQRLYSNP
PDC-374WGJ75339.1_925PaOC      121  ALQGSRFDGISLLDLATYTAGGLPLQFPDSVQKDQAQIRDYYRQWQPTYAPGSQRLYSNP
PDC-374WGW87865.1_837PaOC      121  ALQGSRFDGISLLDLATYTAGGLPLQFPDSVQKDQAQIRDYYRQWQPTYAPGSQRLYSNP
PDC-103QES80695.1_910PaOC      121  ALQGSRFDGISLLDLATYTAGGLPLQFPDSVQKDQAQIRDYYRQWQPTYAPGSQRLYSNP
PDC-103QPV53905.1_6224PaOC     121  ALQGSRFDGISLLDLATYTAGGLPLQFPDSVQKDQAQIRDYYRQWQPTYAPGSQRLYSNP
PDC-35AVR81260.1_945.PaOC      121  ALQGSRFDGISLLDLATYTAGGLPLQFPDSVQKDQAQIRDYYRQWQPTYAPGSQRLYSNP
PDC-35QJA35930.1_977PaOC       121  ALQGSRFDGISLLDLATYTAGGLPLQFPDSVQKDQAQIRDYYRQWQPTYAPGSQRLYSNP
PDC-35AYZ46521.1_3210PaOC      121  ALQGSRFDGISLLDLATYTAGGLPLQFPDSVQKDQAQIRDYYRQWQPTYAPGSQRLYSNP
PDC-35AYZ59004.1_3976PaOC      121  ALQGSRFDGISLLDLATYTAGGLPLQFPDSVQKDQAQIRDYYRQWQPTYAPGSQRLYSNP
PDC-35QDR21260.1_3098PaOC      121  ALQGSRFDGISLLDLATYTAGGLPLQFPDSVQKDQAQIRDYYRQWQPTYAPGSQRLYSNP
PDC-35QEK40749.1_862PaOC       121  ALQGSRFDGISLLDLATYTAGGLPLQFPDSVQKDQAQIRDYYRQWQPTYAPGSQRLYSNP
PDC-35AUB00033.3_866PaOC       121  ALQGSRFDGISLLDLATYTAGGLPLQFPDSVQKDQAQIRDYYRQWQPTYAPGSQRLYSNP
PDC-35QPP34775.1_1311PaOC      121  ALQGSRFDGISLLDLATYTAGGLPLQFPDSVQKDQAQIRDYYRQWQPTYAPGSQRLYSNP
PDC-35QLF38648.1_891PaOC       121  ALQGSRFDGISLLDLATYTAGGLPLQFPDSVQKDQAQIRDYYRQWQPTYAPGSQRLYSNP
PDC-35QLF14474.1_2055PaOC      121  ALQGSRFDGISLLDLATYTAGGLPLQFPDSVQKDQAQIRDYYRQWQPTYAPGSQRLYSNP
PDC-35QQV60864.1_5728PaOC      121  ALQGSRFDGISLLDLATYTAGGLPLQFPDSVQKDQAQIRDYYRQWQPTYAPGSQRLYSNP
PDC-35QQW01132.1_862PaOC       121  ALQGSRFDGISLLDLATYTAGGLPLQFPDSVQKDQAQIRDYYRQWQPTYAPGSQRLYSNP
PDC-35QQW17992.1_5232PaOC      121  ALQGSRFDGISLLDLATYTAGGLPLQFPDSVQKDQAQIRDYYRQWQPTYAPGSQRLYSNP
PDC-35QQW23330.1_862PaOC       121  ALQGSRFDGISLLDLATYTAGGLPLQFPDSVQKDQAQIRDYYRQWQPTYAPGSQRLYSNP
PDC-35QZE42332.1_862PaOC       121  ALQGSRFDGISLLDLATYTAGGLPLQFPDSVQKDQAQIRDYYRQWQPTYAPGSQRLYSNP
PDC-35UFM83790.1_826PaOC       121  ALQGSRFDGISLLDLATYTAGGLPLQFPDSVQKDQAQIRDYYRQWQPTYAPGSQRLYSNP
PDC-35UJC15714.1_892PaOC       121  ALQGSRFDGISLLDLATYTAGGLPLQFPDSVQKDQAQIRDYYRQWQPTYAPGSQRLYSNP
PDC-35UTR21121.1_931PaOC       121  ALQGSRFDGISLLDLATYTAGGLPLQFPDSVQKDQAQIRDYYRQWQPTYAPGSQRLYSNP
PDC-35UUP09555.1_853PaOC       121  ALQGSRFDGISLLDLATYTAGGLPLQFPDSVQKDQAQIRDYYRQWQPTYAPGSQRLYSNP
PDC-35UWG56199.1_885PaOC       121  ALQGSRFDGISLLDLATYTAGGLPLQFPDSVQKDQAQIRDYYRQWQPTYAPGSQRLYSNP
PDC-43UNT30829.1_824PaOC       121  ALQGSRFDGISLLDLATYTAGGLPLQFPDSVQKDQAQIRDYYRQWQPTYAPGSQRLYSNP
PDC-45ALZ89573.1_855.PaOC      121  ALQGSRFDGISLLDLATYTAGGLPLQFPDSVQKDQAQIRDYYRQWQPTYAPGSQRLYSNP
PDC-45QKL11315.1_849PaOC       121  ALQGSRFDGISLLDLATYTAGGLPLQFPDSVQKDQAQIRDYYRQWQPTYAPGSQRLYSNP
PDC-45QQV94504.1_850PaOC       121  ALQGSRFDGISLLDLATYTAGGLPLQFPDSVQKDQAQIRDYYRQWQPTYAPGSQRLYSNP
PDC-162UJB98759.1_2776PaOC     121  ALQGSRFDGISLLDLATYTAGGLPLQFPDSVQKDQAQIRDYYRQWQPTYAPGSQRLYSNP
PDC-123QYE90948.1_3103PaOC     121  ALQGSRFDGISLLDLATYTAGGLPLQFPDSVQKDQAQIRDYYRQWQPTYAPGSQRLYSNP
PDC-5UXH60990.1_2046PaBCP      121  ALQGSRFDGISLLDLATYTAGGLPLQFPDSVQKDQAQIRDYYRQWQPTYAPGSQRLYSNP
PDC-5WBA37364.1_905PaBCP       121  ALQGSRFDGISLLDLATYTAGGLPLQFPDSVQKDQAQIRDYYRQWQPTYAPGSQRLYSNP
PDC-5WGJ68312.1_842PaBCP       121  ALQGSRFDGISLLDLATYTAGGLPLQFPDSVQKDQAQIRDYYRQWQPTYAPGSQRLYSNP
PDC-5WGT14845.1_882PaBCP       121  ALQGSRFDGISLLDLATYTAGGLPLQFPDSVQKDQAQIRDYYRQWQPTYAPGSQRLYSNP
PDC-5WGX69730.1_872PaBCP       121  ALQGSRFDGISLLDLATYTAGGLPLQFPDSVQKDQAQIRDYYRQWQPTYAPGSQRLYSNP
PDC-5CAI9893238.1_8405]PaBCP   121  ALQGSRFDGISLLDLATYTAGGLPLQFPDSVQKDQAQIRDYYRQWQPTYAPGSQRLYSNP
PDC-5CAI9905252.1_60335]PaBCP  121  ALQGSRFDGISLLDLATYTAGGLPLQFPDSVQKDQAQIRDYYRQWQPTYAPGSQRLYSNP
PDC-5UXH60990.1_2046PaBOC      121  ALQGSRFDGISLLDLATYTAGGLPLQFPDSVQKDQAQIRDYYRQWQPTYAPGSQRLYSNP
PDC-5WBA37364.1_905PaBOC       121  ALQGSRFDGISLLDLATYTAGGLPLQFPDSVQKDQAQIRDYYRQWQPTYAPGSQRLYSNP
PDC-5WGJ68312.1_842PaBOC       121  ALQGSRFDGISLLDLATYTAGGLPLQFPDSVQKDQAQIRDYYRQWQPTYAPGSQRLYSNP
PDC-5WGT14845.1_882PaBOC       121  ALQGSRFDGISLLDLATYTAGGLPLQFPDSVQKDQAQIRDYYRQWQPTYAPGSQRLYSNP
PDC-5WGX69730.1_872PaBOC       121  ALQGSRFDGISLLDLATYTAGGLPLQFPDSVQKDQAQIRDYYRQWQPTYAPGSQRLYSNP
PDC-5CAI9893238.1_8405]PaBOC   121  ALQGSRFDGISLLDLATYTAGGLPLQFPDSVQKDQAQIRDYYRQWQPTYAPGSQRLYSNP
PDC-5CAI9905252.1_60335]PaBOC  121  ALQGSRFDGISLLDLATYTAGGLPLQFPDSVQKDQAQIRDYYRQWQPTYAPGSQRLYSNP
PDC-5ALU48309.1_2211.PaOC      121  ALQGSRFDGISLLDLATYTAGGLPLQFPDSVQKDQAQIRDYYRQWQPTYAPGSQRLYSNP
PDC-5AMX89317.1_4085.PaOC      121  ALQGSRFDGISLLDLATYTAGGLPLQFPDSVQKDQAQIRDYYRQWQPTYAPGSQRLYSNP
PDC-5ATH01452.1_827PaOC        121  ALQGSRFDGISLLDLATYTAGGLPLQFPDSVQKDQAQIRDYYRQWQPTYAPGSQRLYSNP
PDC-5UTN13111.1_830PaOC        121  ALQGSRFDGISLLDLATYTAGGLPLQFPDSVQKDQAQIRDYYRQWQPTYAPGSQRLYSNP
PDC-5UUH88148.1_851PaOC        121  ALQGSRFDGISLLDLATYTAGGLPLQFPDSVQKDQAQIRDYYRQWQPTYAPGSQRLYSNP
PDC-5UUH98459.1_806PaOC        121  ALQGSRFDGISLLDLATYTAGGLPLQFPDSVQKDQAQIRDYYRQWQPTYAPGSQRLYSNP
PDC-5UVH93148.1_837PaOC        121  ALQGSRFDGISLLDLATYTAGGLPLQFPDSVQKDQAQIRDYYRQWQPTYAPGSQRLYSNP
PDC-5UWU62523.1_171PaOC        121  ALQGSRFDGISLLDLATYTAGGLPLQFPDSVQKDQAQIRDYYRQWQPTYAPGSQRLYSNP
PDC-5WDZ01346.1_910PaOC        121  ALQGSRFDGISLLDLATYTAGGLPLQFPDSVQKDQAQIRDYYRQWQPTYAPGSQRLYSNP
PDC-5WEO42644.1_846PaOC        121  ALQGSRFDGISLLDLATYTAGGLPLQFPDSVQKDQAQIRDYYRQWQPTYAPGSQRLYSNP
PDC-5WGW99708.1_849PaOC        121  ALQGSRFDGISLLDLATYTAGGLPLQFPDSVQKDQAQIRDYYRQWQPTYAPGSQRLYSNP
PDC-5WJM42992.1_848PaOC        121  ALQGSRFDGISLLDLATYTAGGLPLQFPDSVQKDQAQIRDYYRQWQPTYAPGSQRLYSNP
PDC-60QWY08308.1_854PaBCP      121  ALQGSRFDGISLLDLATYTAGGLPLQFPDSVQKDQAQIRDYYRQWQPTYAPGSQRLYSNP
PDC-60QZV50082.1_838PaBCP      121  ALQGSRFDGISLLDLATYTAGGLPLQFPDSVQKDQAQIRDYYRQWQPTYAPGSQRLYSNP
PDC-60QWY08308.1_854PaBOC      121  ALQGSRFDGISLLDLATYTAGGLPLQFPDSVQKDQAQIRDYYRQWQPTYAPGSQRLYSNP
PDC-60QZV50082.1_838PaBOC      121  ALQGSRFDGISLLDLATYTAGGLPLQFPDSVQKDQAQIRDYYRQWQPTYAPGSQRLYSNP
PDC-60QPZ60674.1_902PaOC       121  ALQGSRFDGISLLDLATYTAGGLPLQFPDSVQKDQAQIRDYYRQWQPTYAPGSQRLYSNP
PDC-60WAJ92260.1_847PaOC       121  ALQGSRFDGISLLDLATYTAGGLPLQFPDSVQKDQAQIRDYYRQWQPTYAPGSQRLYSNP
PDC-60WAJ98065.1_847PaOC       121  ALQGSRFDGISLLDLATYTAGGLPLQFPDSVQKDQAQIRDYYRQWQPTYAPGSQRLYSNP
PDC-15UNL10022.1_837PaBCP      121  ALQGSRFDGISLLDLATYTAGGLPLQFPDSVQKDQAQIRDYYRQWQPTYAPGSQRLYSNP
PDC-15UNL10022.1_837PaBOC      121  ALQGSRFDGISLLDLATYTAGGLPLQFPDSVQKDQAQIRDYYRQWQPTYAPGSQRLYSNP
PDC-15AOT38867.1_3370PaOC      121  ALQGSRFDGISLLDLATYTAGGLPLQFPDSVQKDQAQIRDYYRQWQPTYAPGSQRLYSNP
PDC-15ALZ27705.1_859PaOC       121  ALQGSRFDGISLLDLATYTAGGLPLQFPDSVQKDQAQIRDYYRQWQPTYAPGSQRLYSNP
PDC-15AXC19915.1_1529PaOC      121  ALQGSRFDGISLLDLATYTAGGLPLQFPDSVQKDQAQIRDYYRQWQPTYAPGSQRLYSNP
PDC-15UTP69334.1_895PaOC       121  ALQGSRFDGISLLDLATYTAGGLPLQFPDSVQKDQAQIRDYYRQWQPTYAPGSQRLYSNP
PDC-15UNK91447.1_837PaOC       121  ALQGSRFDGISLLDLATYTAGGLPLQFPDSVQKDQAQIRDYYRQWQPTYAPGSQRLYSNP
PDC-15UNL03855.1_837PaOC       121  ALQGSRFDGISLLDLATYTAGGLPLQFPDSVQKDQAQIRDYYRQWQPTYAPGSQRLYSNP
PDC-28UYM61272.1_6539PaOC      121  ALQGSRFDGISLLDLATYTAGGLPLQFPDSVQKDQAQIRDYYRQWQPTYAPGSQRLYSNP
PDC-22QPZ72804.1_848PaOC       121  ALQGSRFDGISLLDLATYTAGGLPLQFPDSVQKDQAQIRDYYRQWQPTYAPGSQRLYSNP
PDC-22QQM08170.1_4816PaOC      121  ALQGSRFDGISLLDLATYTAGGLPLQFPDSVQKDQAQIRDYYRQWQPTYAPGSQRLYSNP
PDC-97QKR35301.1_4456PaOC      121  ALQGSRFDGISLLDLATYTAGGLPLQFPDSVQKDQAQIRDYYRQWQPTYAPGSQRLYSNP
PDC-51WGV68048.1_897PaOC       121  ALQGSRFDGISLLDLATYTAGGLPLQFPDSVQKDQAQIRDYYRQWQPTYAPGSQRLYSNP
PDC-1HW06_09835_867PaOC        121  ALQGSRFDGISLLDLATYTAGGLPLQFPDSVQKDQAQIRDYYRQWQPTYAPGSQRLYSNP
PDC-151QQV49515.1_805PaOC      121  ALQGSRFDGISLLDLGTYTAGGLPLQFPDVVQKDPAQIRDYYRQWQPTYAPGSHRQYSNP
consensus>70                        ALQGSRFDGISLLDLaTYTAGGLPLQfPDsVQKDqaQIRDYYRQWQPTYaPGSqRlYSNP
                          acc
```

```
                                                 α5                  α6        α7          β6        η5          η6
*PDC-374QKR78821.1_828PaOC*

PDC-374QKR78821.1_828PaOC        181  SIGLFGYLAARSLGQPFERLMEQQVFPALGLEQTHLDVPEAALAQYAQGYGKDDRPLRVG
PDC-331WCI44612.1_917PaOC        181  SIGLFGYLAARSLGQPFERLMEQQVFPALGLEQTHLDVPEAALAQYAQGYG.........
PDC-8HV97_01835_992PaOC          181  SIGLFGYLAARSLGQPFERLMEQQVFPALGLEQTHLDVPEAALAQYAQGYGKDDRPLRVG
PDC-8CLH63_27535_5433PaOC        181  SIGLFGYLAARSLGQPFERLMEQQVFPALGLEQTHLDVPEAALAQYAQGYGKDDRPLRVG
PDC-31HW08_20870_899PaOC         181  SIGLFGYLAARSLGQPFERLMEQQLFPALGLEQTHLDVPEAALAQYAQGYGKDDRPLRVG
PDC-3CPZ93_04765_942PaBCP        181  SIGLFGYLAARSLGQPFERLMEQQVFPALGLEQTHLDVPEAALAQYAQGYGKDDRPLRVG
PDC-3CPZ93_04765_942PaBOC        181  SIGLFGYLAARSLGQPFERLMEQQVFPALGLEQTHLDVPEAALAQYAQGYGKDDRPLRVG
PDC-3CD796_04405_870PaOC         181  SIGLFGYLAARSLGQPFERLMEQQVFPALGLEQTHLDVPEAALAQYAQGYGKDDRPLRVG
PDC-1HV98_23795_933PaOC          181  SIGLFGYLAARSLGQPFERLMEQQVFPALGLEQTHLDVPEAALAQYAQGYGKDDRPLRVG
PDC-103HV95_30355_911PaOC        181  SIGLFGYLAARSLGQPFERLMEQQVFPALGLEQTHLDVPEAALAQYAQGYGKDDRPLRVG
PDC-5HW02_17620_927PaOC          181  SIGLFGYLAARSLGQPFERLMEQQVFPALGLEQTHLDVPEAALAQYAQGYGKDDRPLRVG
PDC-5HW01_11705_873PaOC          181  SIGLFGYLAARSLGQPFERLMEQQVFPALGLEQTHLDVPEAALAQYAQGYGKDDRPLRVG
PDC-5CD800_33195_6567PaOC        181  SIGLFGYLAARSLGQPFERLMEQQVFPALGLEQTHLDVPEAALAQYAQGYGKDDRPLRVG
PDC-5CD799_31455_6219PaOC        181  SIGLFGYLAARSLGQPFERLMEQQVFPALGLEQTHLDVPEAALAQYAQGYGKDDRPLRVG
PDC-46CEK59_30555_6032PaOC       181  SIGLFGYLAARSLGQPFERLMEQQLFPALGLEQTHLDVPEAALAQYAQGYGKDDRPLRVG
PDC-66QTQ98808.1_855PaOC         181  SIGLFGYLAARSLGQPFERIMEQQLFPALGLEQTHLDVPEAALAQYAQGYGKDDRPLRVG
PDC-364QYE79191.1_1969PaOC       181  SIGLFGYLAARSLGQPFERIMEQQLFPALGLEQTHLDVPEAALAQYAQGYGKDDRPLRVG
PDC-122QYN07359.1_5685PaOC       181  SIGLFGYLAARSLGQPFERLMEQQLFPALGLEQTHLDVPEAALAQYAQGYGKDDRPLRVG
PDC-100QKR60216.1_898PaOC        181  SIGLFGYLAARSLGQPFERLMEQQLFPALGLEQTHLDVPEAALAQYAQGYGKDDRPLRVG
PDC-34WBM27707.1_865PaBCP        181  SIGLFGYLAARSLGQPFERLMEQQLFPALGLEQTHLDVPEAALAQYAQGYGKDDRPLRVG
PDC-34WGX11792.1_865PaBCP        181  SIGLFGYLAARSLGQPFERLMEQQLFPALGLEQTHLDVPEAALAQYAQGYGKDDRPLRVG
PDC-34WBM27707.1_865PaBOC        181  SIGLFGYLAARSLGQPFERLMEQQLFPALGLEQTHLDVPEAALAQYAQGYGKDDRPLRVG
PDC-34WGX11792.1_865PaBOC        181  SIGLFGYLAARSLGQPFERLMEQQLFPALGLEQTHLDVPEAALAQYAQGYGKDDRPLRVG
PDC-34ALY72428.1_939PaOC         181  SIGLFGYLAARSLGQPFERLMEQQLFPALGLEQTHLDVPEAALAQYAQGYGKDDRPLRVG
PDC-34ASM83699.1_885PaOC         181  SIGLFGYLAARSLGQPFERLMEQQLFPALGLEQTHLDVPEAALAQYAQGYGKDDRPLRVG
PDC-34AYW64445.1_883PaOC         181  SIGLFGYLAARSLGQPFERLMEQQLFPALGLEQTHLDVPEAALAQYAQGYGKDDRPLRVG
PDC-34UTR27428.1_860PaOC         181  SIGLFGYLAARSLGQPFERLMEQQLFPALGLEQTHLDVPEAALAQYAQGYGKDDRPLRVG
PDC-34UOD92021.1_934PaOC         181  SIGLFGYLAARSLGQPFERLMEQQLFPALGLEQTHLDVPEAALAQYAQGYGKDDRPLRVG
PDC-34WIV39806.1_860PaOC         181  SIGLFGYLAARSLGQPFERLMEQQLFPALGLEQTHLDVPEAALAQYAQGYGKDDRPLRVG
PDC-264QKR42793.1_833PaOC        181  SIGLFGYLAARSLGQPFERLMEQQVFPALGLEQTHLDVPEAALAQYAQGYGKDDRPLRVG
PDC-167QIZ28250.1_4314PaOC       181  SIGLFGYLAARSLGQPFERIMEQRLFPALGLEQTHLDVPEAALAQYAQGYGKDDRPLRVG
PDC-167UTQ27575.1_842PaOC        181  SIGLFGYLAARSLGQPFERIMEQRLFPALGLEQTHLDVPEAALAQYAQGYGKDDRPLRVG
PDC-6QZV30905.1_832PaBCP         181  SIGLFGYLAARSLGQPFERLMEQQVFPALGLEQTHLDVPEAALAQYAQGYGKDDRPLRVG
PDC-6QZV30905.1_832PaBOC         181  SIGLFGYLAARSLGQPFERLMEQQVFPALGLEQTHLDVPEAALAQYAQGYGKDDRPLRVG
PDC-37WAJ85675.1_843PaBCP        181  SIGLFGYLAARSLGQPFERLMEQQLFPALGLEQTHLDVPEAALAQYAQGYGKDDRPLRVG
PDC-37WGX56158.1_855PaBCP        181  SIGLFGYLAARSLGQPFERLMEQQLFPALGLEQTHLDVPEAALAQYAQGYGKDDRPLRVG
PDC-37WAJ85675.1_843PaBOC        181  SIGLFGYLAARSLGQPFERLMEQQLFPALGLEQTHLDVPEAALAQYAQGYGKDDRPLRVG
PDC-37WGX56158.1_855PaBOC        181  SIGLFGYLAARSLGQPFERLMEQQLFPALGLEQTHLDVPEAALAQYAQGYGKDDRPLRVG
PDC-37ANA71197.1_2757.PaOC       181  SIGLFGYLAARSLGQPFERLMEQQLFPALGLEQTHLDVPEAALAQYAQGYGKDDRPLRVG
PDC-37WGX14507.1_3195PaOC        181  SIGLFGYLAARSLGQPFERLMEQQLFPALGLEQTHLDVPEAALAQYAQGYGKDDRPLRVG
PDC-46AUA93832.1_897PaOC         181  SIGLFGYLAARSLGQPFERLMEQQLFPALGLEQTHLDVPEAALAQYAQGYGKDDRPLRVG
PDC-105QGP95004.1_877PaBCP       181  SIGLFGYLAARSLGQPFERLMEQQLFPALGLEQTHLDVPEAALAQYAQGYGKDDRPLRVG
PDC-105QGP95004.1_877PaBOC       181  SIGLFGYLAARSLGQPFERLMEQQLFPALGLEQTHLDVPEAALAQYAQGYGKDDRPLRVG
PDC-12AVN43882.1_2061.PaOC       181  SIGLFGYLAARSLGQPFERLMEQQLFPALGLEQTHLDVPEAALAQYAQGYGKDDRPLRVG
PDC-12QNE63041.1_859PaOC         181  SIGLFGYLAARSLGQPFERLMEQQLFPALGLEQTHLDVPEAALAQYAQGYGKDDRPLRVG
PDC-12WGW93864.1_852PaOC         181  SIGLFGYLAARSLGQPFERLMEQQLFPALGLEQTHLDVPEAALAQYAQGYGKDDRPLRVG
PDC-12WGX05587.1_852PaOC         181  SIGLFGYLAARSLGQPFERLMEQQLFPALGLEQTHLDVPEAALAQYAQGYGKDDRPLRVG
PDC-11QSA85555.1_5581PaBCP       181  SIGLFGYLAARSLGQPFERLMEQQLFPALGLEQTHLDVPEAALAQYAQGYGKDDRPLRVG
PDC-11QSA91467.1_5367PaBCP       181  SIGLFGYLAARSLGQPFERLMEQQLFPALGLEQTHLDVPEAALAQYAQGYGKDDRPLRVG
PDC-11QSA85555.1_5581PaBOC       181  SIGLFGYLAARSLGQPFERLMEQQLFPALGLEQTHLDVPEAALAQYAQGYGKDDRPLRVG
PDC-11QSA91467.1_5367PaBOC       181  SIGLFGYLAARSLGQPFERLMEQQLFPALGLEQTHLDVPEAALAQYAQGYGKDDRPLRVG
PDC-11AYQ83203.1_3491PaOC        181  SIGLFGYLAARSLGQPFERLMEQQLFPALGLEQTHLDVPEAALAQYAQGYGKDDRPLRVG
PDC-11AYR15208.1_5205PaOC        181  SIGLFGYLAARSLGQPFERLMEQQLFPALGLEQTHLDVPEAALAQYAQGYGKDDRPLRVG
PDC-11AZN02324.1_4787PaOC        181  SIGLFGYLAARSLGQPFERLMEQQLFPALGLEQTHLDVPEAALAQYAQGYGKDDRPLRVG
PDC-11AZN04123.1_203PaOC         181  SIGLFGYLAARSLGQPFERLMEQQLFPALGLEQTHLDVPEAALAQYAQGYGKDDRPLRVG
PDC-11AZN17071.1_5847PaOC        181  SIGLFGYLAARSLGQPFERLMEQQLFPALGLEQTHLDVPEAALAQYAQGYGKDDRPLRVG
PDC-11AZN48510.1_4981PaOC        181  SIGLFGYLAARSLGQPFERLMEQQLFPALGLEQTHLDVPEAALAQYAQGYGKDDRPLRVG
PDC-11AZN60564.1_5101PaOC        181  SIGLFGYLAARSLGQPFERLMEQQLFPALGLEQTHLDVPEAALAQYAQGYGKDDRPLRVG
PDC-11AZN55779.1_6545PaOC        181  SIGLFGYLAARSLGQPFERLMEQQLFPALGLEQTHLDVPEAALAQYAQGYGKDDRPLRVG
PDC-11AYF68870.1_744PaOC         181  SIGLFGYLAARSLGQPFERLMEQQLFPALGLEQTHLDVPEAALAQYAQGYGKDDRPLRVG
PDC-11QUS21307.1_2433PaOC        181  SIGLFGYLAARSLGQPFERLMEQQLFPALGLEQTHLDVPEAALAQYAQGYGKDDRPLRVG
PDC-11UVH87183.1_982PaOC         181  SIGLFGYLAARSLGQPFERLMEQQLFPALGLEQTHLDVPEAALAQYAQGYGKDDRPLRVG
PDC-16QCC64126.1_958PaBCP        181  SIGLFGYLAARSLGQPFERLMEQQLFPALGLEQTHLDVPEAALAQYAQGYGKDDRPLRVG
PDC-16UBI70916.1_933PaBCP        181  SIGLFGYLAARSLGQPFERLMEQQLFPALGLEQTHLDVPEAALAQYAQGYGKDDRPLRVG
PDC-16UTQ84596.1_855PaBCP        181  SIGLFGYLAARSLGQPFERLMEQQLFPALGLEQTHLDVPEAALAQYAQGYGKDDRPLRVG
PDC-16QCC64126.1_958PaBOC        181  SIGLFGYLAARSLGQPFERLMEQQLFPALGLEQTHLDVPEAALAQYAQGYGKDDRPLRVG
PDC-16UBI70916.1_933PaBOC        181  SIGLFGYLAARSLGQPFERLMEQQLFPALGLEQTHLDVPEAALAQYAQGYGKDDRPLRVG
PDC-16UTQ84596.1_855PaBOC        181  SIGLFGYLAARSLGQPFERLMEQQLFPALGLEQTHLDVPEAALAQYAQGYGKDDRPLRVG
PDC-16ALS12949.1_2977.PaOC       181  SIGLFGYLAARSLGQPFERLMEQQLFPALGLEQTHLDVPEAALAQYAQGYGKDDRPLRVG
PDC-16AYW58201.1_855PaOC         181  SIGLFGYLAARSLGQPFERLMEQQLFPALGLEQTHLDVPEAALAQYAQGYGKDDRPLRVG
PDC-16QDR00398.1_890PaOC         181  SIGLFGYLAARSLGQPFERLMEQQLFPALGLEQTHLDVPEAALAQYAQGYGKDDRPLRVG
PDC-16QGJ31628.1_895PaOC         181  SIGLFGYLAARSLGQPFERLMEQQLFPALGLEQTHLDVPEAALAQYAQGYGKDDRPLRVG
PDC-16QKG34934.1_1840PaOC        181  SIGLFGYLAARSLGQPFERLMEQQLFPALGLEQTHLDVPEAALAQYAQGYGKDDRPLRVG
PDC-16QOQ64874.1_122PaOC         181  SIGLFGYLAARSLGQPFERLMEQQLFPALGLEQTHLDVPEAALAQYAQGYGKDDRPLRVG
PDC-16QVQ73157.1_860PaOC         181  SIGLFGYLAARSLGQPFERLMEQQLFPALGLEQTHLDVPEAALAQYAQGYGKDDRPLRVG
PDC-16UOC64480.1_827PaOC         181  SIGLFGYLAARSLGQPFERLMEQQLFPALGLEQTHLDVPEAALAQYAQGYGKDDRPLRVG
PDC-16UOC70564.1_826PaOC         181  SIGLFGYLAARSLGQPFERLMEQQLFPALGLEQTHLDVPEAALAQYAQGYGKDDRPLRVG
PDC-16UOC76649.1_827PaOC         181  SIGLFGYLAARSLGQPFERLMEQQLFPALGLEQTHLDVPEAALAQYAQGYGKDDRPLRVG
PDC-16UOC82740.1_827PaOC         181  SIGLFGYLAARSLGQPFERLMEQQLFPALGLEQTHLDVPEAALAQYAQGYGKDDRPLRVG
PDC-16UOC88825.1_827PaOC         181  SIGLFGYLAARSLGQPFERLMEQQLFPALGLEQTHLDVPEAALAQYAQGYGKDDRPLRVG
PDC-16UOE68701.1_827PaOC         181  SIGLFGYLAARSLGQPFERLMEQQLFPALGLEQTHLDVPEAALAQYAQGYGKDDRPLRVG
PDC-16WDC65088.1_844PaOC         181  SIGLFGYLAARSLGQPFERLMEQQLFPALGLEQTHLDVPEAALAQYAQGYGKDDRPLRVG
PDC-16WGW70130.1_845PaOC         181  SIGLFGYLAARSLGQPFERLMEQQLFPALGLEQTHLDVPEAALAQYAQGYGKDDRPLRVG
PDC-16WGW76010.1_845PaOC         181  SIGLFGYLAARSLGQPFERLMEQQLFPALGLEQTHLDVPEAALAQYAQGYGKDDRPLRVG
PDC-16WGW81892.1_845PaOC         181  SIGLFGYLAARSLGQPFERLMEQQLFPALGLEQTHLDVPEAALAQYAQGYGKDDRPLRVG
PDC-16WGX43443.1_806PaOC         181  SIGLFGYLAARSLGQPFERLMEQQLFPALGLEQTHLDVPEAALAQYAQGYGKDDRPLRVG
PDC-16WJM37210.1_871PaOC         181  SIGLFGYLAARSLGQPFERLMEQQLFPALGLEQTHLDVPEAALAQYAQGYGKDDRPLRVG
PDC-59UQZ18314.1_902PaOC         181  SIGLFGYLAARSLGQPFERLMEQQLFPALGLEQTHLDVPEAALAQYAQGYGKDDRPLRVG
PDC-142WGW57525.1_830PaBCP       181  SIGLFGYLAARSLGQPFERLMEQQVFPALGLEQTHLDVPEAALAQYAQGYGKDDRPLRVG
PDC-142WGW32210.1_830PaBCP       181  SIGLFGYLAARSLGQPFERLMEQQVFPALGLEQTHLDVPEAALAQYAQGYGKDDRPLRVG
PDC-142WGW44776.1_830PaBCP       181  SIGLFGYLAARSLGQPFERLMEQQVFPALGLEQTHLDVPEAALAQYAQGYGKDDRPLRVG
PDC-142WGW57525.1_830PaBOC       181  SIGLFGYLAARSLGQPFERLMEQQVFPALGLEQTHLDVPEAALAQYAQGYGKDDRPLRVG
PDC-142WGW32210.1_830PaBOC       181  SIGLFGYLAARSLGQPFERLMEQQVFPALGLEQTHLDVPEAALAQYAQGYGKDDRPLRVG
PDC-142WGW44776.1_830PaBOC       181  SIGLFGYLAARSLGQPFERLMEQQVFPALGLEQTHLDVPEAALAQYAQGYGKDDRPLRVG
PDC-142AON05138.1_825PaOC        181  SIGLFGYLAARSLGQPFERLMEQQVFPALGLEQTHLDVPEAALAQYAQGYGKDDRPLRVG
PDC-142AON11127.1_825PaOC        181  SIGLFGYLAARSLGQPFERLMEQQVFPALGLEQTHLDVPEAALAQYAQGYGKDDRPLRVG
PDC-142AON23122.1_825PaOC        181  SIGLFGYLAARSLGQPFERLMEQQVFPALGLEQTHLDVPEAALAQYAQGYGKDDRPLRVG
PDC-142AON29110.1_825PaOC        181  SIGLFGYLAARSLGQPFERLMEQQVFPALGLEQTHLDVPEAALAQYAQGYGKDDRPLRVG
PDC-142AON41099.1_825PaOC        181  SIGLFGYLAARSLGQPFERLMEQQVFPALGLEQTHLDVPEAALAQYAQGYGKDDRPLRVG
PDC-142AON47112.1_824PaOC        181  SIGLFGYLAARSLGQPFERLMEQQVFPALGLEQTHLDVPEAALAQYAQGYGKDDRPLRVG
PDC-142AON17115.1_824PaOC        181  SIGLFGYLAARSLGQPFERLMEQQVFPALGLEQTHLDVPEAALAQYAQGYGKDDRPLRVG
PDC-142AON35116.1_825PaOC        181  SIGLFGYLAARSLGQPFERLMEQQVFPALGLEQTHLDVPEAALAQYAQGYGKDDRPLRVG
PDC-19aWGW51434.1_1207PaBCP      181  SIGLFGYLAARSLGQPFERLMEQQLFPALGLEQTHLDVPEAALAQYAQGYGKDDRPLRVG
```

```
PDC-19aWGW37666.1_6817PaBCP    181  SIGLFGYLAARSLGQPFERLMEQQLFPALGLEQTHLDVPEAALAQYAQGYGKDDRPLRVG
PDC-19aWGW59864.1_3660PaBCP    181  SIGLFGYLAARSLGQPFERLMEQQLFPALGLEQTHLDVPEAALAQYAQGYGKDDRPLRVG
PDC-19aWGX49838.1_877PaBCP     181  SIGLFGYLAARSLGQPFERLMEQQLFPALGLEQTHLDVPEAALAQYAQGYGKDDRPLRVG
PDC-19aWGX24308.1_877PaBCP     181  SIGLFGYLAARSLGQPFERLMEQQLFPALGLEQTHLDVPEAALAQYAQGYGKDDRPLRVG
PDC-19aWGX35552.1_5487PaBCP    181  SIGLFGYLAARSLGQPFERLMEQQLFPALGLEQTHLDVPEAALAQYAQGYGKDDRPLRVG
PDC-19aWKA37694.1_2446PaBCP    181  SIGLFGYLAARSLGQPFERLMEQQLFPALGLEQTHLDVPEAALAQYAQGYGKDDRPLRVG
PDC-19aWGW51434.1_1207PaBOC    181  SIGLFGYLAARSLGQPFERLMEQQLFPALGLEQTHLDVPEAALAQYAQGYGKDDRPLRVG
PDC-19aWGW37666.1_6817PaBOC    181  SIGLFGYLAARSLGQPFERLMEQQLFPALGLEQTHLDVPEAALAQYAQGYGKDDRPLRVG
PDC-19aWGW59864.1_3660PaBOC    181  SIGLFGYLAARSLGQPFERLMEQQLFPALGLEQTHLDVPEAALAQYAQGYGKDDRPLRVG
PDC-19aWGX49838.1_877PaBOC     181  SIGLFGYLAARSLGQPFERLMEQQLFPALGLEQTHLDVPEAALAQYAQGYGKDDRPLRVG
PDC-19aWGX24308.1_877PaBOC     181  SIGLFGYLAARSLGQPFERLMEQQLFPALGLEQTHLDVPEAALAQYAQGYGKDDRPLRVG
PDC-19aWGX35552.1_5487PaBOC    181  SIGLFGYLAARSLGQPFERLMEQQLFPALGLEQTHLDVPEAALAQYAQGYGKDDRPLRVG
PDC-19aWKA37694.1_2446PaBOC    181  SIGLFGYLAARSLGQPFERLMEQQLFPALGLEQTHLDVPEAALAQYAQGYGKDDRPLRVG
PDC-19aAPB63370.1_616PaOC      181  SIGLFGYLAARSLGQPFERLMEQQLFPALGLEQTHLDVPEAALAQYAQGYGKDDRPLRVG
PDC-19aARI89529.1_870PaOC      181  SIGLFGYLAARSLGQPFERLMEQQLFPALGLEQTHLDVPEAALAQYAQGYGKDDRPLRVG
PDC-19aARI95964.1_871PaOC      181  SIGLFGYLAARSLGQPFERLMEQQLFPALGLEQTHLDVPEAALAQYAQGYGKDDRPLRVG
PDC-19aASA13594.1_977PaOC      181  SIGLFGYLAARSLGQPFERLMEQQLFPALGLEQTHLDVPEAALAQYAQGYGKDDRPLRVG
PDC-19aASA27558.1_962PaOC      181  SIGLFGYLAARSLGQPFERLMEQQLFPALGLEQTHLDVPEAALAQYAQGYGKDDRPLRVG
PDC-19aASD01901.1_971PaOC      181  SIGLFGYLAARSLGQPFERLMEQQLFPALGLEQTHLDVPEAALAQYAQGYGKDDRPLRVG
PDC-19aUAC92160.1_908PaOC      181  SIGLFGYLAARSLGQPFERLMEQQLFPALGLEQTHLDVPEAALAQYAQGYGKDDRPLRVG
PDC-19aUAC98839.1_942PaOC      181  SIGLFGYLAARSLGQPFERLMEQQLFPALGLEQTHLDVPEAALAQYAQGYGKDDRPLRVG
PDC-19aUTQ33604.1_830PaOC      181  SIGLFGYLAARSLGQPFERLMEQQLFPALGLEQTHLDVPEAALAQYAQGYGKDDRPLRVG
PDC-19aUOO58611.1_830PaOC      181  SIGLFGYLAARSLGQPFERLMEQQLFPALGLEQTHLDVPEAALAQYAQGYGKDDRPLRVG
PDC-19aWDA50910.1_883PaOC      181  SIGLFGYLAARSLGQPFERLMEQQLFPALGLEQTHLDVPEAALAQYAQGYGKDDRPLRVG
PDC-19aWGX30960.1_941PaOC      181  SIGLFGYLAARSLGQPFERLMEQQLFPALGLEQTHLDVPEAALAQYAQGYGKDDRPLRVG
PDC-19aWGX61448.1_5603PaOC     181  SIGLFGYLAARSLGQPFERLMEQQLFPALGLEQTHLDVPEAALAQYAQGYGKDDRPLRVG
PDC-19aWGX82118.1_941PaOC      181  SIGLFGYLAARSLGQPFERLMEQQLFPALGLEQTHLDVPEAALAQYAQGYGKDDRPLRVG
PDC-19aWIO49783.1_187PaOC      181  SIGLFGYLAARSLGQPFERLMEQQLFPALGLEQTHLDVPEAALAQYAQGYGKDDRPLRVG
PDC-24QZV43725.1_941PaBCP      181  SIGLFGYLAARSLGQPFERLMEQQVFPALGLEQTHLDVPEAALAQYAQGYGKDDRPLRVG
PDC-24QZV43725.1_941PaBOC      181  SIGLFGYLAARSLGQPFERLMEQQVFPALGLEQTHLDVPEAALAQYAQGYGKDDRPLRVG
PDC-24QDR06751.1_860PaOC       181  SIGLFGYLAARSLGQPFERLMEQQVFPALGLEQTHLDVPEAALAQYAQGYGKDDRPLRVG
PDC-24UGR36796.1_3412PaOC      181  SIGLFGYLAARSLGQPFERLMEQQVFPALGLEQTHLDVPEAALAQYAQGYGKDDRPLRVG
PDC-24UTQ39921.1_833PaOC       181  SIGLFGYLAARSLGQPFERLMEQQVFPALGLEQTHLDVPEAALAQYAQGYGKDDRPLRVG
PDC-137QKE94864.1_852PaOC     181  SIGLFGYLAARSLGQPFERLMEQQLFPALGLEQTHLDVPEAALAQYAQGYGKDDRPLRVG
PDC-147UGR30133.1_1950PaOC    181  SIGLFGYLAARSLGQPFKRLMEQQLFPALGLEQTHLDVPEAALAQYAQGYGKDDRPLRVG
PDC-8AYY41152.1_5352PaBCP     181  SIGLFGYLAARSLGQPFERLMEQQVFPALGLEQTHLDVPEAALAQYAQGYGKDDRPLRVG
PDC-8QOE14337.1_211PaBCP      181  SIGLFGYLAARSLGQPFERLMEQQVFPALGLEQTHLDVPEAALAQYAQGYGKDDRPLRVG
PDC-8QPN15577.1_911PaBCP      181  SIGLFGYLAARSLGQPFERLMEQQVFPALGLEQTHLDVPEAALAQYAQGYGKDDRPLRVG
PDC-8QPN23993.1_908PaBCP      181  SIGLFGYLAARSLGQPFERLMEQQVFPALGLEQTHLDVPEAALAQYAQGYGKDDRPLRVG
PDC-8QUE64702.1_909PaBCP      181  SIGLFGYLAARSLGQPFERLMEQQVFPALGLEQTHLDVPEAALAQYAQGYGKDDRPLRVG
PDC-8QYA88773.1_2249PaBCP     181  SIGLFGYLAARSLGQPFERLMEQQVFPALGLEQTHLDVPEAALAQYAQGYGKDDRPLRVG
PDC-8QYF01720.1_5249PaBCP     181  SIGLFGYLAARSLGQPFERLMEQQVFPALGLEQTHLDVPEAALAQYAQGYGKDDRPLRVG
PDC-8QZU99504.1_908PaBCP      181  SIGLFGYLAARSLGQPFERLMEQQVFPALGLEQTHLDVPEAALAQYAQGYGKDDRPLRVG
PDC-8QZV05727.1_908PaBCP      181  SIGLFGYLAARSLGQPFERLMEQQVFPALGLEQTHLDVPEAALAQYAQGYGKDDRPLRVG
PDC-8QZV12028.1_910PaBCP      181  SIGLFGYLAARSLGQPFERLMEQQVFPALGLEQTHLDVPEAALAQYAQGYGKDDRPLRVG
PDC-8QZV18112.1_863PaBCP      181  SIGLFGYLAARSLGQPFERLMEQQVFPALGLEQTHLDVPEAALAQYAQGYGKDDRPLRVG
PDC-8QZV24387.1_912PaBCP      181  SIGLFGYLAARSLGQPFERLMEQQVFPALGLEQTHLDVPEAALAQYAQGYGKDDRPLRVG
PDC-8QZV68851.1_908PaBCP      181  SIGLFGYLAARSLGQPFERLMEQQVFPALGLEQTHLDVPEAALAQYAQGYGKDDRPLRVG
PDC-8QZV81338.1_870PaBCP      181  SIGLFGYLAARSLGQPFERLMEQQVFPALGLEQTHLDVPEAALAQYAQGYGKDDRPLRVG
PDC-8QZV87769.1_908PaBCP      181  SIGLFGYLAARSLGQPFERLMEQQVFPALGLEQTHLDVPEAALAQYAQGYGKDDRPLRVG
PDC-8QZV94048.1_908PaBCP      181  SIGLFGYLAARSLGQPFERLMEQQVFPALGLEQTHLDVPEAALAQYAQGYGKDDRPLRVG
PDC-8UXH53497.1_877PaBCP      181  SIGLFGYLAARSLGQPFERLMEQQVFPALGLEQTHLDVPEAALAQYAQGYGKDDRPLRVG
PDC-8UXH66521.1_877PaBCP      181  SIGLFGYLAARSLGQPFERLMEQQVFPALGLEQTHLDVPEAALAQYAQGYGKDDRPLRVG
PDC-8UXH70664.1_4987PaBCP     181  SIGLFGYLAARSLGQPFERLMEQQVFPALGLEQTHLDVPEAALAQYAQGYGKDDRPLRVG
PDC-8UUW81479.1_5273PaBCP     181  SIGLFGYLAARSLGQPFERLMEQQVFPALGLEQTHLDVPEAALAQYAQGYGKDDRPLRVG
PDC-8WDM07130.1_910PaBCP      181  SIGLFGYLAARSLGQPFERLMEQQVFPALGLEQTHLDVPEAALAQYAQGYGKDDRPLRVG
PDC-8WHM62936.1_909PaBCP      181  SIGLFGYLAARSLGQPFERLMEQQVFPALGLEQTHLDVPEAALAQYAQGYGKDDRPLRVG
PDC-8WHM69354.1_909PaBCP      181  SIGLFGYLAARSLGQPFERLMEQQVFPALGLEQTHLDVPEAALAQYAQGYGKDDRPLRVG
PDC-8WHM75768.1_909PaBCP      181  SIGLFGYLAARSLGQPFERLMEQQVFPALGLEQTHLDVPEAALAQYAQGYGKDDRPLRVG
PDC-8WHM84263.1_909PaBCP      181  SIGLFGYLAARSLGQPFERLMEQQVFPALGLEQTHLDVPEAALAQYAQGYGKDDRPLRVG
PDC-8AYY41152.1_5352PaBOC     181  SIGLFGYLAARSLGQPFERLMEQQVFPALGLEQTHLDVPEAALAQYAQGYGKDDRPLRVG
PDC-8QOE14337.1_211PaBOC      181  SIGLFGYLAARSLGQPFERLMEQQVFPALGLEQTHLDVPEAALAQYAQGYGKDDRPLRVG
PDC-8QPN15577.1_911PaBOC      181  SIGLFGYLAARSLGQPFERLMEQQVFPALGLEQTHLDVPEAALAQYAQGYGKDDRPLRVG
PDC-8QPN23993.1_908PaBOC      181  SIGLFGYLAARSLGQPFERLMEQQVFPALGLEQTHLDVPEAALAQYAQGYGKDDRPLRVG
PDC-8QUE64702.1_909PaBOC      181  SIGLFGYLAARSLGQPFERLMEQQVFPALGLEQTHLDVPEAALAQYAQGYGKDDRPLRVG
PDC-8QYA88773.1_2249PaBOC     181  SIGLFGYLAARSLGQPFERLMEQQVFPALGLEQTHLDVPEAALAQYAQGYGKDDRPLRVG
PDC-8QYF01720.1_5249PaBOC     181  SIGLFGYLAARSLGQPFERLMEQQVFPALGLEQTHLDVPEAALAQYAQGYGKDDRPLRVG
PDC-8QZU99504.1_908PaBOC      181  SIGLFGYLAARSLGQPFERLMEQQVFPALGLEQTHLDVPEAALAQYAQGYGKDDRPLRVG
PDC-8QZV05727.1_908PaBOC      181  SIGLFGYLAARSLGQPFERLMEQQVFPALGLEQTHLDVPEAALAQYAQGYGKDDRPLRVG
PDC-8QZV12028.1_910PaBOC      181  SIGLFGYLAARSLGQPFERLMEQQVFPALGLEQTHLDVPEAALAQYAQGYGKDDRPLRVG
PDC-8QZV18112.1_863PaBOC      181  SIGLFGYLAARSLGQPFERLMEQQVFPALGLEQTHLDVPEAALAQYAQGYGKDDRPLRVG
PDC-8QZV24387.1_912PaBOC      181  SIGLFGYLAARSLGQPFERLMEQQVFPALGLEQTHLDVPEAALAQYAQGYGKDDRPLRVG
PDC-8QZV68851.1_908PaBOC      181  SIGLFGYLAARSLGQPFERLMEQQVFPALGLEQTHLDVPEAALAQYAQGYGKDDRPLRVG
PDC-8QZV81338.1_870PaBOC      181  SIGLFGYLAARSLGQPFERLMEQQVFPALGLEQTHLDVPEAALAQYAQGYGKDDRPLRVG
PDC-8QZV87769.1_908PaBOC      181  SIGLFGYLAARSLGQPFERLMEQQVFPALGLEQTHLDVPEAALAQYAQGYGKDDRPLRVG
PDC-8QZV94048.1_908PaBOC      181  SIGLFGYLAARSLGQPFERLMEQQVFPALGLEQTHLDVPEAALAQYAQGYGKDDRPLRVG
PDC-8UXH53497.1_877PaBOC      181  SIGLFGYLAARSLGQPFERLMEQQVFPALGLEQTHLDVPEAALAQYAQGYGKDDRPLRVG
PDC-8UXH66521.1_877PaBOC      181  SIGLFGYLAARSLGQPFERLMEQQVFPALGLEQTHLDVPEAALAQYAQGYGKDDRPLRVG
PDC-8UXH70664.1_4987PaBOC     181  SIGLFGYLAARSLGQPFERLMEQQVFPALGLEQTHLDVPEAALAQYAQGYGKDDRPLRVG
PDC-8UUW81479.1_5273PaBOC     181  SIGLFGYLAARSLGQPFERLMEQQVFPALGLEQTHLDVPEAALAQYAQGYGKDDRPLRVG
PDC-8WDM07130.1_910PaBOC      181  SIGLFGYLAARSLGQPFERLMEQQVFPALGLEQTHLDVPEAALAQYAQGYGKDDRPLRVG
PDC-8WHM62936.1_909PaBOC      181  SIGLFGYLAARSLGQPFERLMEQQVFPALGLEQTHLDVPEAALAQYAQGYGKDDRPLRVG
PDC-8WHM69354.1_909PaBOC      181  SIGLFGYLAARSLGQPFERLMEQQVFPALGLEQTHLDVPEAALAQYAQGYGKDDRPLRVG
PDC-8WHM75768.1_909PaBOC      181  SIGLFGYLAARSLGQPFERLMEQQVFPALGLEQTHLDVPEAALAQYAQGYGKDDRPLRVG
PDC-8WHM84263.1_909PaBOC      181  SIGLFGYLAARSLGQPFERLMEQQVFPALGLEQTHLDVPEAALAQYAQGYGKDDRPLRVG
PDC-8AMA35328.1_919.PaOC      181  SIGLFGYLAARSLGQPFERLMEQQVFPALGLEQTHLDVPEAALAQYAQGYGKDDRPLRVG
PDC-8ANI06966.1_119PaOC       181  SIGLFGYLAARSLGQPFERLMEQQVFPALGLEQTHLDVPEAALAQYAQGYGKDDRPLRVG
PDC-8AYW70752.1_952PaOC       181  SIGLFGYLAARSLGQPFERLMEQQVFPALGLEQTHLDVPEAALAQYAQGYGKDDRPLRVG
PDC-8AYZ76972.1_2272PaOC      181  SIGLFGYLAARSLGQPFERLMEQQVFPALGLEQTHLDVPEAALAQYAQGYGKDDRPLRVG
PDC-8QKP85289.1_778PaOC       181  SIGLFGYLAARSLGQPFERLMEQQVFPALGLEQTHLDVPEAALAQYAQGYGKDDRPLRVG
PDC-8QKR30573.1_5806PaOC      181  SIGLFGYLAARSLGQPFERLMEQQVFPALGLEQTHLDVPEAALAQYAQGYGKDDRPLRVG
PDC-8QLF19879.1_939PaOC       181  SIGLFGYLAARSLGQPFERLMEQQVFPALGLEQTHLDVPEAALAQYAQGYGKDDRPLRVG
PDC-8QYE59452.1_5107PaOC      181  SIGLFGYLAARSLGQPFERLMEQQVFPALGLEQTHLDVPEAALAQYAQGYGKDDRPLRVG
PDC-8QZV98104.1_4956PaOC      181  SIGLFGYLAARSLGQPFERLMEQQVFPALGLEQTHLDVPEAALAQYAQGYGKDDRPLRVG
PDC-8UFK73541.1_1068PaOC      181  SIGLFGYLAARSLGQPFERLMEQQVFPALGLEQTHLDVPEAALAQYAQGYGKDDRPLRVG
PDC-8UGR33975.2_368PaOC       181  SIGLFGYLAARSLGQPFERLMEQQVFPALGLEQTHLDVPEAALAQYAQGYGKDDRPLRVG
PDC-8UTN26696.1_862PaOC       181  SIGLFGYLAARSLGQPFERLMEQQVFPALGLEQTHLDVPEAALAQYAQGYGKDDRPLRVG
PDC-8UTQ91314.1_910PaOC       181  SIGLFGYLAARSLGQPFERLMEQQVFPALGLEQTHLDVPEAALAQYAQGYGKDDRPLRVG
PDC-8UNK85299.1_868PaOC       181  SIGLFGYLAARSLGQPFERLMEQQVFPALGLEQTHLDVPEAALAQYAQGYGKDDRPLRVG
PDC-8UNK97662.1_866PaOC       181  SIGLFGYLAARSLGQPFERLMEQQVFPALGLEQTHLDVPEAALAQYAQGYGKDDRPLRVG
PDC-8USI83494.1_833PaOC       181  SIGLFGYLAARSLGQPFERLMEQQVFPALGLEQTHLDVPEAALAQYAQGYGKDDRPLRVG
PDC-8WAO16415.1_5521PaOC      181  SIGLFGYLAARSLGQPFERLMEQQVFPALGLEQTHLDVPEAALAQYAQGYGKDDRPLRVG
PDC-98QZD62144.1_4346PaBCP    181  SIGLFGYLAARSLGQPFERLMEQQVFPALGLEQTHLDVPEAALAQYAQGYGKDDRPLRVG
```

PDC-98QZH48390.1_4214PaBCP	181	SIGLFGYLAARSLGQPFERLMEQQVFPALGLEQTHLDVPEAALAQYAQGYGKDDRPLRVG
PDC-98QZD62144.1_4346PaBOC	181	SIGLFGYLAARSLGQPFERLMEQQVFPALGLEQTHLDVPEAALAQYAQGYGKDDRPLRVG
PDC-98QZH48390.1_4214PaBOC	181	SIGLFGYLAARSLGQPFERLMEQQVFPALGLEQTHLDVPEAALAQYAQGYGKDDRPLRVG
PDC-98ALP56563.1_1203.PaOC	181	SIGLFGYLAARSLGQPFERLMEQQVFPALGLEQTHLDVPEAALAQYAQGYGKDDRPLRVG
PDC-98UTL97623.1_830PaOC	181	SIGLFGYLAARSLGQPFERLMEQQVFPALGLEQTHLDVPEAALAQYAQGYGKDDRPLRVG
PDC-457UTN40061.1_863PaOC	181	SIGLFGYLAARSLGQPFERLMEQQLFPALGLEQTHLDVPEAALAQYAQGYGKDDRPLRVG
PDC-23UXI89283.1_830PaOC	181	SIGLFGYLAARSLGQPFERLMEQQLFPALGLEQTHLDVPEAALAQYAQGYGKDDRPLRVG
PDC-245AUA75408.1_866PaOC	181	SIDLFGYLAARSLGQPFERLMEQQLFPALGLEQTHLDVPEAALAQYAQGYGKDDRPLRVG
PDC-31QZW06655.1_840PaBCP	181	SIGLFGYLAARSLGQPFERLMEQQLFPALGLEQTHLDVPEAALAQYAQGYGKDDRPLRVG
PDC-31QZW06655.1_840PaBOC	181	SIGLFGYLAARSLGQPFERLMEQQLFPALGLEQTHLDVPEAALAQYAQGYGKDDRPLRVG
PDC-31AXN27248.1_931PaOC	181	SIGLFGYLAARSLGQPFERLMEQQLFPALGLEQTHLDVPEAALAQYAQGYGKDDRPLRVG
PDC-31QBC07904.1_3021PaOC	181	SIGLFGYLAARSLGQPFERLMEQQLFPALGLEQTHLDVPEAALAQYAQGYGKDDRPLRVG
PDC-31UFM90275.1_905PaOC	181	SIGLFGYLAARSLGQPFERLMEQQLFPALGLEQTHLDVPEAALAQYAQGYGKDDRPLRVG
PDC-31UFM98870.1_905PaOC	181	SIGLFGYLAARSLGQPFERLMEQQLFPALGLEQTHLDVPEAALAQYAQGYGKDDRPLRVG
PDC-31UNT25139.1_901PaOC	181	SIGLFGYLAARSLGQPFERLMEQQLFPALGLEQTHLDVPEAALAQYAQGYGKDDRPLRVG
PDC-31UWJ96607.1_4267PaOC	181	SIGLFGYLAARSLGQPFERLMEQQLFPALGLEQTHLDVPEAALAQYAQGYGKDDRPLRVG
PDC-31WGX88300.1_823PaOC	181	SIGLFGYLAARSLGQPFERLMEQQLFPALGLEQTHLDVPEAALAQYAQGYGKDDRPLRVG
PDC-31WHM08550.1_859PaOC	181	SIGLFGYLAARSLGQPFERLMEQQLFPALGLEQTHLDVPEAALAQYAQGYGKDDRPLRVG
PDC-39AYK21377.1_872PaOC	181	SIGLFGYLAARSLGQPFERLMEQQLFPALGLEQTHLDVPEAALAQYAQGYGKDDRPLRVG
PDC-39UJC21946.1_837PaOC	181	SIGLFGYLAARSLGQPFERLMEQQLFPALGLEQTHLDVPEAALAQYAQGYGKDDRPLRVG
PDC-30UPL39337.1_928PaBCP	181	SIGLFGYLAARSLGQPFERLMEQQLFPALGLEQTHLDVPEAALAQYAQGYGKDDRPLRVG
PDC-30UPL39337.1_928PaBOC	181	SIGLFGYLAARSLGQPFERLMEQQLFPALGLEQTHLDVPEAALAQYAQGYGKDDRPLRVG
PDC-30UHL95932.1_5029PaOC	181	SIGLFGYLAARSLGQPFERLMEQQLFPALGLEQTHLDVPEAALAQYAQGYGKDDRPLRVG
PDC-30UML99445.1_838PaOC	181	SIGLFGYLAARSLGQPFERLMEQQLFPALGLEQTHLDVPEAALAQYAQGYGKDDRPLRVG
PDC-80WAJ79202.1_935PaOC	181	SIGLFGYLAARSLGQPFERLMEQQVFPALGLEQTHLDVPEAALAQYAQGYGKDDRPLRVG
8SDL_1|Chain	181	SIGLFGYLAARSLGQPFERLMEQQVFPALGLEQTHLDVPEAALAQYAQGYGKDDRPLRVG
PDC-3UTN33167.1_589PaBCP	181	SIGLFGYLAARSLGQPFERLMEQQVFPALGLEQTHLDVPEAALAQYAQGYGKDDRPLRVG
PDC-3WCI38158.1_984PaBCP	181	SIGLFGYLAARSLGQPFERLMEQQVFPALGLEQTHLDVPEAALAQYAQGYGKDDRPLRVG
PDC-3WCI50909.1_867PaBCP	181	SIGLFGYLAARSLGQPFERLMEQQVFPALGLEQTHLDVPEAALAQYAQGYGKDDRPLRVG
PDC-3WCI90621.1_961PaBCP	181	SIGLFGYLAARSLGQPFERLMEQQVFPALGLEQTHLDVPEAALAQYAQGYGKDDRPLRVG
PDC-3WEO48793.1_832PaBCP	181	SIGLFGYLAARSLGQPFERLMEQQVFPALGLEQTHLDVPEAALAQYAQGYGKDDRPLRVG
PDC-3CAI9907424.1_19963]PaBCP	181	SIGLFGYLAARSLGQPFERLMEQQVFPALGLEQTHLDVPEAALAQYAQGYGKDDRPLRVG
PDC-3UTN33167.1_589PaBOC	181	SIGLFGYLAARSLGQPFERLMEQQVFPALGLEQTHLDVPEAALAQYAQGYGKDDRPLRVG
PDC-3WCI38158.1_984PaBOC	181	SIGLFGYLAARSLGQPFERLMEQQVFPALGLEQTHLDVPEAALAQYAQGYGKDDRPLRVG
PDC-3WCI50909.1_867PaBOC	181	SIGLFGYLAARSLGQPFERLMEQQVFPALGLEQTHLDVPEAALAQYAQGYGKDDRPLRVG
PDC-3WCI90621.1_961PaBOC	181	SIGLFGYLAARSLGQPFERLMEQQVFPALGLEQTHLDVPEAALAQYAQGYGKDDRPLRVG
PDC-3WEO48793.1_832PaBOC	181	SIGLFGYLAARSLGQPFERLMEQQVFPALGLEQTHLDVPEAALAQYAQGYGKDDRPLRVG
PDC-3CAI9907424.1_19963]PaBOC	181	SIGLFGYLAARSLGQPFERLMEQQVFPALGLEQTHLDVPEAALAQYAQGYGKDDRPLRVG
PDC-3AHA17954.1_839.PaOC	181	SIGLFGYLAARSLGQPFERLMEQQVFPALGLEQTHLDVPEAALAQYAQGYGKDDRPLRVG
PDC-3ALE46683.1_840.PaOC	181	SIGLFGYLAARSLGQPFERLMEQQVFPALGLEQTHLDVPEAALAQYAQGYGKDDRPLRVG
PDC-3ARC78082.1_863.PaOC	181	SIGLFGYLAARSLGQPFERLMEQQVFPALGLEQTHLDVPEAALAQYAQGYGKDDRPLRVG
PDC-3ARG53386.1_5885PaOC	181	SIGLFGYLAARSLGQPFERLMEQQVFPALGLEQTHLDVPEAALAQYAQGYGKDDRPLRVG
PDC-3WAC79950.1_4482PaOC	181	SIGLFGYLAARSLGQPFERLMEQQVFPALGLEQTHLDVPEAALAQYAQGYGKDDRPLRVG
PDC-3WAE22012.1_4863PaOC	181	SIGLFGYLAARSLGQPFERLMEQQVFPALGLEQTHLDVPEAALAQYAQGYGKDDRPLRVG
PDC-3WAE29433.1_777PaOC	181	SIGLFGYLAARSLGQPFERLMEQQVFPALGLEQTHLDVPEAALAQYAQGYGKDDRPLRVG
PDC-3WAW42145.1_847PaOC	181	SIGLFGYLAARSLGQPFERLMEQQVFPALGLEQTHLDVPEAALAQYAQGYGKDDRPLRVG
PDC-3WCI57477.1_911PaOC	181	SIGLFGYLAARSLGQPFERLMEQQVFPALGLEQTHLDVPEAALAQYAQGYGKDDRPLRVG
PDC-3WCI77402.1_900PaOC	181	SIGLFGYLAARSLGQPFERLMEQQVFPALGLEQTHLDVPEAALAQYAQGYGKDDRPLRVG
PDC-3WCI64002.1_913PaOC	181	SIGLFGYLAARSLGQPFERLMEQQVFPALGLEQTHLDVPEAALAQYAQGYGKDDRPLRVG
PDC-3WCI83994.1_905PaOC	181	SIGLFGYLAARSLGQPFERLMEQQVFPALGLEQTHLDVPEAALAQYAQGYGKDDRPLRVG
PDC-3WJM48814.1_842PaOC	181	SIGLFGYLAARSLGQPFERLMEQQVFPALGLEQTHLDVPEAALAQYAQGYGKDDRPLRVG
PDC-374ANP60834.1_3741PaBCP	181	SIGLFGYLAARSLGQPFERLMEQQVFPALGLEQTHLDVPEAALAQYAQGYGKDDRPLRVG
PDC-374AYZ86971.1_5950PaBCP	181	SIGLFGYLAARSLGQPFERLMEQQVFPALGLEQTHLDVPEAALAQYAQGYGKDDRPLRVG
PDC-374QZV75115.1_843PaBCP	181	SIGLFGYLAARSLGQPFERLMEQQVFPALGLEQTHLDVPEAALAQYAQGYGKDDRPLRVG
PDC-374QZW12711.1_843PaBCP	181	SIGLFGYLAARSLGQPFERLMEQQVFPALGLEQTHLDVPEAALAQYAQGYGKDDRPLRVG
PDC-374UEG06685.1_907PaBCP	181	SIGLFGYLAARSLGQPFERLMEQQVFPALGLEQTHLDVPEAALAQYAQGYGKDDRPLRVG
PDC-374UGW95802.1_984PaBCP	181	SIGLFGYLAARSLGQPFERLMEQQVFPALGLEQTHLDVPEAALAQYAQGYGKDDRPLRVG
PDC-374WGW26222.1_839PaBCP	181	SIGLFGYLAARSLGQPFERLMEQQVFPALGLEQTHLDVPEAALAQYAQGYGKDDRPLRVG
PDC-374ANP60834.1_3741PaBOC	181	SIGLFGYLAARSLGQPFERLMEQQVFPALGLEQTHLDVPEAALAQYAQGYGKDDRPLRVG
PDC-374AYZ86971.1_5950PaBOC	181	SIGLFGYLAARSLGQPFERLMEQQVFPALGLEQTHLDVPEAALAQYAQGYGKDDRPLRVG
PDC-374QZV75115.1_843PaBOC	181	SIGLFGYLAARSLGQPFERLMEQQVFPALGLEQTHLDVPEAALAQYAQGYGKDDRPLRVG
PDC-374QZW12711.1_843PaBOC	181	SIGLFGYLAARSLGQPFERLMEQQVFPALGLEQTHLDVPEAALAQYAQGYGKDDRPLRVG
PDC-374UEG06685.1_907PaBOC	181	SIGLFGYLAARSLGQPFERLMEQQVFPALGLEQTHLDVPEAALAQYAQGYGKDDRPLRVG
PDC-374UGW95802.1_984PaBOC	181	SIGLFGYLAARSLGQPFERLMEQQVFPALGLEQTHLDVPEAALAQYAQGYGKDDRPLRVG
PDC-374WGW26222.1_839PaBOC	181	SIGLFGYLAARSLGQPFERLMEQQVFPALGLEQTHLDVPEAALAQYAQGYGKDDRPLRVG
PDC-374AOP56366.1_851PaOC	181	SIGLFGYLAARSLGQPFERLMEQQVFPALGLEQTHLDVPEAALAQYAQGYGKDDRPLRVG
PDC-374AVE31445.1_873.PaOC	181	SIGLFGYLAARSLGQPFERLMEQQVFPALGLEQTHLDVPEAALAQYAQGYGKDDRPLRVG
PDC-374QAS71510.1_873PaOC	181	SIGLFGYLAARSLGQPFERLMEQQVFPALGLEQTHLDVPEAALAQYAQGYGKDDRPLRVG
PDC-374QDD38027.1_4463PaOC	181	SIGLFGYLAARSLGQPFERLMEQQVFPALGLEQTHLDVPEAALAQYAQGYGKDDRPLRVG
PDC-374QKF05339.1_5830PaOC	181	SIGLFGYLAARSLGQPFERLMEQQVFPALGLEQTHLDVPEAALAQYAQGYGKDDRPLRVG
PDC-374QKK83983.1_853PaOC	181	SIGLFGYLAARSLGQPFERLMEQQVFPALGLEQTHLDVPEAALAQYAQGYGKDDRPLRVG
PDC-374QKS41466.1_4199PaOC	181	SIGLFGYLAARSLGQPFERLMEQQVFPALGLEQTHLDVPEAALAQYAQGYGKDDRPLRVG
PDC-374QPZ80284.1_873PaOC	181	SIGLFGYLAARSLGQPFERLMEQQVFPALGLEQTHLDVPEAALAQYAQGYGKDDRPLRVG
PDC-374QQW07466.1_922PaOC	181	SIGLFGYLAARSLGQPFERLMEQQVFPALGLEQTHLDVPEAALAQYAQGYGKDDRPLRVG
PDC-374QZU93305.1_843PaOC	181	SIGLFGYLAARSLGQPFERLMEQQVFPALGLEQTHLDVPEAALAQYAQGYGKDDRPLRVG
PDC-374UAC85653.1_934PaOC	181	SIGLFGYLAARSLGQPFERLMEQQVFPALGLEQTHLDVPEAALAQYAQGYGKDDRPLRVG
PDC-374UGR20250.1_3485PaOC	181	SIGLFGYLAARSLGQPFERLMEQQVFPALGLEQTHLDVPEAALAQYAQGYGKDDRPLRVG
PDC-374UIU12727.1_851PaOC	181	SIGLFGYLAARSLGQPFERLMEQQVFPALGLEQTHLDVPEAALAQYAQGYGKDDRPLRVG
PDC-374UJC03215.1_956PaOC	181	SIGLFGYLAARSLGQPFERLMEQQVFPALGLEQTHLDVPEAALAQYAQGYGKDDRPLRVG
PDC-374UJC09425.1_959PaOC	181	SIGLFGYLAARSLGQPFERLMEQQVFPALGLEQTHLDVPEAALAQYAQGYGKDDRPLRVG
PDC-374UJF57976.1_829PaOC	181	SIGLFGYLAARSLGQPFERLMEQQVFPALGLEQTHLDVPEAALAQYAQGYGKDDRPLRVG
PDC-374UJF63613.1_829PaOC	181	SIGLFGYLAARSLGQPFERLMEQQVFPALGLEQTHLDVPEAALAQYAQGYGKDDRPLRVG
PDC-374UJF68041.1_5361PaOC	181	SIGLFGYLAARSLGQPFERLMEQQVFPALGLEQTHLDVPEAALAQYAQGYGKDDRPLRVG
PDC-374UJF74874.1_829PaOC	181	SIGLFGYLAARSLGQPFERLMEQQVFPALGLEQTHLDVPEAALAQYAQGYGKDDRPLRVG
PDC-374UJF80517.1_829PaOC	181	SIGLFGYLAARSLGQPFERLMEQQVFPALGLEQTHLDVPEAALAQYAQGYGKDDRPLRVG
PDC-374UJF86151.1_829PaOC	181	SIGLFGYLAARSLGQPFERLMEQQVFPALGLEQTHLDVPEAALAQYAQGYGKDDRPLRVG
PDC-374UJF90590.1_5359PaOC	181	SIGLFGYLAARSLGQPFERLMEQQVFPALGLEQTHLDVPEAALAQYAQGYGKDDRPLRVG
PDC-374UJF97432.1_830PaOC	181	SIGLFGYLAARSLGQPFERLMEQQVFPALGLEQTHLDVPEAALAQYAQGYGKDDRPLRVG
PDC-374UJG01877.1_5359PaOC	181	SIGLFGYLAARSLGQPFERLMEQQVFPALGLEQTHLDVPEAALAQYAQGYGKDDRPLRVG
PDC-374UJG08704.1_829PaOC	181	SIGLFGYLAARSLGQPFERLMEQQVFPALGLEQTHLDVPEAALAQYAQGYGKDDRPLRVG
PDC-374UJG14426.1_829PaOC	181	SIGLFGYLAARSLGQPFERLMEQQVFPALGLEQTHLDVPEAALAQYAQGYGKDDRPLRVG
PDC-374UJG20129.1_829PaOC	181	SIGLFGYLAARSLGQPFERLMEQQVFPALGLEQTHLDVPEAALAQYAQGYGKDDRPLRVG
PDC-374UJG25828.1_829PaOC	181	SIGLFGYLAARSLGQPFERLMEQQVFPALGLEQTHLDVPEAALAQYAQGYGKDDRPLRVG
PDC-374UJG31693.1_884PaOC	181	SIGLFGYLAARSLGQPFERLMEQQVFPALGLEQTHLDVPEAALAQYAQGYGKDDRPLRVG
PDC-374UJG36239.1_5358PaOC	181	SIGLFGYLAARSLGQPFERLMEQQVFPALGLEQTHLDVPEAALAQYAQGYGKDDRPLRVG
PDC-374UGR51117.1_68PaOC	181	SIGLFGYLAARSLGQPFERLMEQQVFPALGLEQTHLDVPEAALAQYAQGYGKDDRPLRVG
PDC-374UON75698.1_5570PaOC	181	SIGLFGYLAARSLGQPFERLMEQQVFPALGLEQTHLDVPEAALAQYAQGYGKDDRPLRVG
PDC-374UPG04116.1_3174PaOC	181	SIGLFGYLAARSLGQPFERLMEQQVFPALGLEQTHLDVPEAALAQYAQGYGKDDRPLRVG
PDC-374UPZ07602.1_2096PaOC	181	SIGLFGYLAARSLGQPFERLMEQQVFPALGLEQTHLDVPEAALAQYAQGYGKDDRPLRVG
PDC-374URM55875.1_5937PaOC	181	SIGLFGYLAARSLGQPFERLMEQQVFPALGLEQTHLDVPEAALAQYAQGYGKDDRPLRVG
PDC-374USV13808.1_3922PaOC	181	SIGLFGYLAARSLGQPFERLMEQQVFPALGLEQTHLDVPEAALAQYAQGYGKDDRPLRVG
PDC-374USV19196.1_4087PaOC	181	SIGLFGYLAARSLGQPFERLMEQQVFPALGLEQTHLDVPEAALAQYAQGYGKDDRPLRVG
PDC-374USX84542.1_851PaOC	181	SIGLFGYLAARSLGQPFERLMEQQVFPALGLEQTHLDVPEAALAQYAQGYGKDDRPLRVG

```
PDC-374USX78872.1_851PaOC        181  SIGLFGYLAARSLGQPFERLMEQQVFPALGLEQTHLDVPEAALAQYAQGYGKDDRPLRVG
PDC-374UUJ85605.1_1747PaOC       181  SIGLFGYLAARSLGQPFERLMEQQVFPALGLEQTHLDVPEAALAQYAQGYGKDDRPLRVG
PDC-374UVS71982.1_4220PaOC       181  SIGLFGYLAARSLGQPFERLMEQQVFPALGLEQTHLDVPEAALAQYAQGYGKDDRPLRVG
PDC-374UWI85046.1_4210PaOC       181  SIGLFGYLAARSLGQPFERLMEQQVFPALGLEQTHLDVPEAALAQYAQGYGKDDRPLRVG
PDC-374UWI92968.1_851PaOC        181  SIGLFGYLAARSLGQPFERLMEQQVFPALGLEQTHLDVPEAALAQYAQGYGKDDRPLRVG
PDC-374UXA22714.1_4203PaOC       181  SIGLFGYLAARSLGQPFERLMEQQVFPALGLEQTHLDVPEAALAQYAQGYGKDDRPLRVG
PDC-374UXA30656.1_926PaOC        181  SIGLFGYLAARSLGQPFERLMEQQVFPALGLEQTHLDVPEAALAQYAQGYGKDDRPLRVG
PDC-374UXA44988.1_926PaOC        181  SIGLFGYLAARSLGQPFERLMEQQVFPALGLEQTHLDVPEAALAQYAQGYGKDDRPLRVG
PDC-374UXN39470.1_4222PaOC       181  SIGLFGYLAARSLGQPFERLMEQQVFPALGLEQTHLDVPEAALAQYAQGYGKDDRPLRVG
PDC-374UZA87382.1_4980PaOC       181  SIGLFGYLAARSLGQPFERLMEQQVFPALGLEQTHLDVPEAALAQYAQGYGKDDRPLRVG
PDC-374WBM66401.1_846PaOC        181  SIGLFGYLAARSLGQPFERLMEQQVFPALGLEQTHLDVPEAALAQYAQGYGKDDRPLRVG
PDC-374WGJ75339.1_925PaOC        181  SIGLFGYLAARSLGQPFERLMEQQVFPALGLEQTHLDVPEAALAQYAQGYGKDDRPLRVG
PDC-374WGW87865.1_837PaOC        181  SIGLFGYLAARSLGQPFERLMEQQVFPALGLEQTHLDVPEAALAQYAQGYGKDDRPLRVG
PDC-103QES80695.1_910PaOC        181  SIGLFGYLAARSLGQPFERLMEQQVFPALGLEQTHLDVPEAALAQYAQGYGKDDRPLRVG
PDC-103QPV53905.1_6224PaOC       181  SIGLFGYLAARSLGQPFERLMEQQVFPALGLEQTHLDVPEAALAQYAQGYGKDDRPLRVG
PDC-35AVR81260.1_945.PaOC        181  SIGLFGYLAARSLGQPFERLMEQQLFPALGLEQTHLDVPEAALAQYAQGYGKDDRPLRVG
PDC-35QJA35930.1_977PaOC         181  SIGLFGYLAARSLGQPFERLMEQQLFPALGLEQTHLDVPEAALAQYAQGYGKDDRPLRVG
PDC-35AYZ46521.1_3210PaOC        181  SIGLFGYLAARSLGQPFERLMEQQLFPALGLEQTHLDVPEAALAQYAQGYGKDDRPLRVG
PDC-35AYZ59004.1_3976PaOC        181  SIGLFGYLAARSLGQPFERLMEQQLFPALGLEQTHLDVPEAALAQYAQGYGKDDRPLRVG
PDC-35QDR21260.1_3098PaOC        181  SIGLFGYLAARSLGQPFERLMEQQLFPALGLEQTHLDVPEAALAQYAQGYGKDDRPLRVG
PDC-35QEK40749.1_862PaOC         181  SIGLFGYLAARSLGQPFERLMEQQLFPALGLEQTHLDVPEAALAQYAQGYGKDDRPLRVG
PDC-35AUB00033.3_866PaOC         181  SIGLFGYLAARSLGQPFERLMEQQLFPALGLEQTHLDVPEAALAQYAQGYGKDDRPLRVG
PDC-35QPP34775.1_1311PaOC        181  SIGLFGYLAARSLGQPFERLMEQQLFPALGLEQTHLDVPEAALAQYAQGYGKDDRPLRVG
PDC-35QLF38648.1_891PaOC         181  SIGLFGYLAARSLGQPFERLMEQQLFPALGLEQTHLDVPEAALAQYAQGYGKDDRPLRVG
PDC-35QLF14474.1_2055PaOC        181  SIGLFGYLAARSLGQPFERLMEQQLFPALGLEQTHLDVPEAALAQYAQGYGKDDRPLRVG
PDC-35QQV60864.1_5728PaOC        181  SIGLFGYLAARSLGQPFERLMEQQLFPALGLEQTHLDVPEAALAQYAQGYGKDDRPLRVG
PDC-35QQW01132.1_862PaOC         181  SIGLFGYLAARSLGQPFERLMEQQLFPALGLEQTHLDVPEAALAQYAQGYGKDDRPLRVG
PDC-35QQW17992.1_5232PaOC        181  SIGLFGYLAARSLGQPFERLMEQQLFPALGLEQTHLDVPEAALAQYAQGYGKDDRPLRVG
PDC-35QQW23330.1_862PaOC         181  SIGLFGYLAARSLGQPFERLMEQQLFPALGLEQTHLDVPEAALAQYAQGYGKDDRPLRVG
PDC-35QZE42332.1_862PaOC         181  SIGLFGYLAARSLGQPFERLMEQQLFPALGLEQTHLDVPEAALAQYAQGYGKDDRPLRVG
PDC-35UFM83790.1_826PaOC         181  SIGLFGYLAARSLGQPFERLMEQQLFPALGLEQTHLDVPEAALAQYAQGYGKDDRPLRVG
PDC-35UJC15714.1_892PaOC         181  SIGLFGYLAARSLGQPFERLMEQQLFPALGLEQTHLDVPEAALAQYAQGYGKDDRPLRVG
PDC-35UTR21121.1_931PaOC         181  SIGLFGYLAARSLGQPFERLMEQQLFPALGLEQTHLDVPEAALAQYAQGYGKDDRPLRVG
PDC-35UUP09555.1_853PaOC         181  SIGLFGYLAARSLGQPFERLMEQQLFPALGLEQTHLDVPEAALAQYAQGYGKDDRPLRVG
PDC-35UWG56199.1_885PaOC         181  SIGLFGYLAARSLGQPFERLMEQQLFPALGLEQTHLDVPEAALAQYAQGYGKDDRPLRVG
PDC-43UNT30829.1_824PaOC         181  SIGLFGYLAARSLGQPFERLMEQQVFPALGLEQTHLDVPEAALAQYAQGYGKDDRPLRVG
PDC-45ALZ89573.1_855.PaOC        181  SIGLFGYLAARSLGQPFERLMEQQLFPALGLEQTHLDVPEAALAQYAQGYGKDDRPLRVG
PDC-45QKL11315.1_849PaOC         181  SIGLFGYLAARSLGQPFERLMEQQLFPALGLEQTHLDVPEAALAQYAQGYGKDDRPLRVG
PDC-45QQV94504.1_850PaOC         181  SIGLFGYLAARSLGQPFERLMEQQLFPALGLEQTHLDVPEAALAQYAQGYGKDDRPLRVG
PDC-162UJB98759.1_2776PaOC       181  SIGLFGYLAARSLGQPFERLMEQQVFPALGLEQTHLDVPEAALAQYAQGYGKDDRPLRVG
PDC-123QYE90948.1_3103PaOC       181  SIGLFGYLAARSLGQPFERLMEQQVFPALGLEQTHLDVPEAALAQYAQGYGKDDRPLRVG
PDC-5UXH60990.1_2046PaBCP        181  SIGLFGYLAARSLGQPFERLMEQQVFPALGLEQTHLDVPEAALAQYAQGYGKDDRPLRVG
PDC-5WBA37364.1_905PaBCP         181  SIGLFGYLAARSLGQPFERLMEQQVFPALGLEQTHLDVPEAALAQYAQGYGKDDRPLRVG
PDC-5WGJ68312.1_842PaBCP         181  SIGLFGYLAARSLGQPFERLMEQQVFPALGLEQTHLDVPEAALAQYAQGYGKDDRPLRVG
PDC-5WGT14845.1_882PaBCP         181  SIGLFGYLAARSLGQPFERLMEQQVFPALGLEQTHLDVPEAALAQYAQGYGKDDRPLRVG
PDC-5WGX69730.1_872PaBCP         181  SIGLFGYLAARSLGQPFERLMEQQVFPALGLEQTHLDVPEAALAQYAQGYGKDDRPLRVG
PDC-5CAI9893238.1_8405]PaBCP     181  SIGLFGYLAARSLGQPFERLMEQQVFPALGLEQTHLDVPEAALAQYAQGYGKDDRPLRVG
PDC-5CAI9905252.1_60335]PaBCP    181  SIGLFGYLAARSLGQPFERLMEQQVFPALGLEQTHLDVPEAALAQYAQGYGKDDRPLRVG
PDC-5UXH60990.1_2046PaBOC        181  SIGLFGYLAARSLGQPFERLMEQQVFPALGLEQTHLDVPEAALAQYAQGYGKDDRPLRVG
PDC-5WBA37364.1_905PaBOC         181  SIGLFGYLAARSLGQPFERLMEQQVFPALGLEQTHLDVPEAALAQYAQGYGKDDRPLRVG
PDC-5WGJ68312.1_842PaBOC         181  SIGLFGYLAARSLGQPFERLMEQQVFPALGLEQTHLDVPEAALAQYAQGYGKDDRPLRVG
PDC-5WGT14845.1_882PaBOC         181  SIGLFGYLAARSLGQPFERLMEQQVFPALGLEQTHLDVPEAALAQYAQGYGKDDRPLRVG
PDC-5WGX69730.1_872PaBOC         181  SIGLFGYLAARSLGQPFERLMEQQVFPALGLEQTHLDVPEAALAQYAQGYGKDDRPLRVG
PDC-5CAI9893238.1_8405]PaBOC     181  SIGLFGYLAARSLGQPFERLMEQQVFPALGLEQTHLDVPEAALAQYAQGYGKDDRPLRVG
PDC-5CAI9905252.1_60335]PaBOC    181  SIGLFGYLAARSLGQPFERLMEQQVFPALGLEQTHLDVPEAALAQYAQGYGKDDRPLRVG
PDC-5ALU48309.1_2211.PaOC        181  SIGLFGYLAARSLGQPFERLMEQQVFPALGLEQTHLDVPEAALAQYAQGYGKDDRPLRVG
PDC-5AMX89317.1_4085.PaOC        181  SIGLFGYLAARSLGQPFERLMEQQVFPALGLEQTHLDVPEAALAQYAQGYGKDDRPLRVG
PDC-5ATH01452.1_827PaOC          181  SIGLFGYLAARSLGQPFERLMEQQVFPALGLEQTHLDVPEAALAQYAQGYGKDDRPLRVG
PDC-5UTN13111.1_830PaOC          181  SIGLFGYLAARSLGQPFERLMEQQVFPALGLEQTHLDVPEAALAQYAQGYGKDDRPLRVG
PDC-5UUH88148.1_851PaOC          181  SIGLFGYLAARSLGQPFERLMEQQVFPALGLEQTHLDVPEAALAQYAQGYGKDDRPLRVG
PDC-5UUH98459.1_806PaOC          181  SIGLFGYLAARSLGQPFERLMEQQVFPALGLEQTHLDVPEAALAQYAQGYGKDDRPLRVG
PDC-5UVH93148.1_837PaOC          181  SIGLFGYLAARSLGQPFERLMEQQVFPALGLEQTHLDVPEAALAQYAQGYGKDDRPLRVG
PDC-5UWU62523.1_171PaOC          181  SIGLFGYLAARSLGQPFERLMEQQVFPALGLEQTHLDVPEAALAQYAQGYGKDDRPLRVG
PDC-5WDZ01346.1_910PaOC          181  SIGLFGYLAARSLGQPFERLMEQQVFPALGLEQTHLDVPEAALAQYAQGYGKDDRPLRVG
PDC-5WEO42644.1_846PaOC          181  SIGLFGYLAARSLGQPFERLMEQQVFPALGLEQTHLDVPEAALAQYAQGYGKDDRPLRVG
PDC-5WGW99708.1_849PaOC          181  SIGLFGYLAARSLGQPFERLMEQQVFPALGLEQTHLDVPEAALAQYAQGYGKDDRPLRVG
PDC-5WJM42992.1_848PaOC          181  SIGLFGYLAARSLGQPFERLMEQQVFPALGLEQTHLDVPEAALAQYAQGYGKDDRPLRVG
PDC-60QWY08308.1_854PaBCP        181  SIGLFGYLAARSLGQPFERLMEQQVFPALGLEQTHLDVPEAALAQYAQGYGKDDRPLRVG
PDC-60QZV50082.1_838PaBCP        181  SIGLFGYLAARSLGQPFERLMEQQVFPALGLEQTHLDVPEAALAQYAQGYGKDDRPLRVG
PDC-60QWY08308.1_854PaBOC        181  SIGLFGYLAARSLGQPFERLMEQQVFPALGLEQTHLDVPEAALAQYAQGYGKDDRPLRVG
PDC-60QZV50082.1_838PaBOC        181  SIGLFGYLAARSLGQPFERLMEQQVFPALGLEQTHLDVPEAALAQYAQGYGKDDRPLRVG
PDC-60QPZ60674.1_902PaOC         181  SIGLFGYLAARSLGQPFERLMEQQVFPALGLEQTHLDVPEAALAQYAQGYGKDDRPLRVG
PDC-60WAJ92260.1_847PaOC         181  SIGLFGYLAARSLGQPFERLMEQQVFPALGLEQTHLDVPEAALAQYAQGYGKDDRPLRVG
PDC-60WAJ98065.1_847PaOC         181  SIGLFGYLAARSLGQPFERLMEQQVFPALGLEQTHLDVPEAALAQYAQGYGKDDRPLRVG
PDC-15UNL10022.1_837PaBCP        181  SIGLFGYLAARSLGQPFERLMEQQVFPALGLEQTHLDVPEAALAQYAQGYGKDDRPLRVG
PDC-15UNL10022.1_837PaBOC        181  SIGLFGYLAARSLGQPFERLMEQQVFPALGLEQTHLDVPEAALAQYAQGYGKDDRPLRVG
PDC-15AOT38867.1_3370PaOC        181  SIGLFGYLAARSLGQPFERLMEQQVFPALGLEQTHLDVPEAALAQYAQGYGKDDRPLRVG
PDC-15ALZ27705.1_859PaOC         181  SIGLFGYLAARSLGQPFERLMEQQVFPALGLEQTHLDVPEAALAQYAQGYGKDDRPLRVG
PDC-15AXC19915.1_1529PaOC        181  SIGLFGYLAARSLGQPFERLMEQQVFPALGLEQTHLDVPEAALAQYAQGYGKDDRPLRVG
PDC-15UTP69334.1_895PaOC         181  SIGLFGYLAARSLGQPFERLMEQQVFPALGLEQTHLDVPEAALAQYAQGYGKDDRPLRVG
PDC-15UNK91447.1_837PaOC         181  SIGLFGYLAARSLGQPFERLMEQQVFPALGLEQTHLDVPEAALAQYAQGYGKDDRPLRVG
PDC-15UNL03855.1_837PaOC         181  SIGLFGYLAARSLGQPFERLMEQQVFPALGLEQTHLDVPEAALAQYAQGYGKDDRPLRVG
PDC-28UYM61272.1_6539PaOC        181  SIGLFGYLAARSLGQPFERLMEQQLFPALGLEQTHLDVPEAALAQYAQGYGKDDRPLRVG
PDC-22QPZ72804.1_848PaOC         181  SIGLFGYLAARSLGQPFERLMEQQLFPALGLEQTHLDVPEAALAQYAQGYGKDDRPLRVG
PDC-22QQM08170.1_4816PaOC        181  SIGLFGYLAARSLGQPFERLMEQQLFPALGLEQTHLDVPEAALAQYAQGYGKDDRPLRVG
PDC-97QKR35301.1_4456PaOC        181  SIGLFGYLAARSLGQPFERLMEQQVFPALGLEQTHLDVPEAALAQYAQGYGKDDRPLRAG
PDC-51WGV68048.1_897PaOC         181  SIGLFGYLAARSLGQPFERLMEQQVFPALGLEQTHLDVPEAALAQYAQGYGKDDRPLRAG
PDC-1HW06_09835_867PaOC          181  SIGLFGYLAARSLGQPFERLMEQQVFPALGLEQTHLDVPEAALAQYAQGYGKDDRPLRVG
PDC-151QQV49515.1_805PaOC        181  SIGLFGYLAARSLGQPFERSMERQLFPALGLEHTFIRVPTAQQGLYAQGYGKDDHPLRVG
consensus>70                          SIgLFGYLAARSLGQPFeRlMEqq.FPALGLEqThldVPeAalaqYAQGYGkddrplrvg
                                 acc
```

PDC-374QKR78821.1_828PaOC α8 β7 α9 η7 α10 β8 TT β9 β10

PDC-374QKR78821.1_828PaOC	241	PGPLDAEGYGVKTSAADLLRFVDANLHPERLDRPWAQALDATHRGYYKVGDMTQGLGWEA
PDC-331WCI44612.1_917PaOC	232	NGPLDAEGYGVKTSAADLLRFVDANLHPERLDRPWAQALDATHRGYYKVGDMTQGLGWEA
PDC-8HV97_01835_992PaOC	241	PGPLDAEGYGVKTSAADLLRFVDANLHPERLDRPWAQALDATHRGYYKVGDMTQGLGWEA
PDC-8CLH63_27535_5433PaOC	241	PGPLDAEGYGVKTSAADLLRFVDANLHPERLDRPWAQALDATHRGYYKVGDMTQGLGWEA
PDC-31HW08_20870_899PaOC	241	PGPLDAEGYGVKTSAADLLRFVDANLHPERLDRPWAQALDATHRGYYKVGDMTQGLGWEA
PDC-3CPZ93_04765_942PaBCP	241	PGPLDAEGYGVKTSAADLLRFVDANLHPERLDRPWAQALDATHRGYYKVGDMTQGLGWEA
PDC-3CPZ93_04765_942PaBOC	241	PGPLDAEGYGVKTSAADLLRFVDANLHPERLDRPWAQALDATHRGYYKVGDMTQGLGWEA
PDC-3CD796_04405_870PaOC	241	PGPLDAEGYGVKTSAADLLRFVDANLHPERLDRPWAQALDATHRGYYKVGDMTQGLGWEA
PDC-1HV98_23795_933PaOC	241	PGPLDAEGYGVKTSAADLLRFVDANLHPERLDRPWAQALDATHRGYYKVGDMTQGLGWEA
PDC-103HV95_30355_911PaOC	241	PGPLDAEGYGVKTSAADLLRFVDANLHPERLDRPWAQALDATHRGYYKVGDMTQGLGWEA
PDC-5HW02_17620_927PaOC	241	PGPLDAEGYGVKTSAADLLRFVDANLHPERLDRPWAQALDATHRGYYKVGDMTQGLGWEA
PDC-5HW01_11705_873PaOC	241	PGPLDAEGYGVKTSAADLLRFVDANLHPERLDRPWAQALDATHRGYYKVGDMTQGLGWEA
PDC-5CD800_33195_6567PaOC	241	PGPLDAEGYGVKTSAADLLRFVDANLHPERLDRPWAQALDATHRGYYKVGDMTQGLGWEA
PDC-5CD799_31455_6219PaOC	241	PGPLDAEGYGVKTSAADLLRFVDANLHPERLDRPWAQALDATHRGYYKVGDMTQGLGWEA
PDC-46CEK59_30555_6032PaOC	241	PGPLDAEGYGVKTSAADLLRFVDANLHPERLDRPWAQALDATHRGYYKVGDMTQGLGWEA
PDC-66QTQ98808.1_855PaOC	241	PGPLDAEGYGVKTSAADLLRFVDANLHPERLDKPWAQALDATHRGYYKVGDMTQGLGWEA
PDC-364QYE79191.1_1969PaOC	241	PGPLDAEGYGVKTSAADLLRFVDANLHPERLDKPWAQALDATHRGYYKVGDMTQGLGWEA
PDC-122QYN07359.1_5685PaOC	241	PGPLDAEGYGVKTSAADLLRFVDANLHPERLDRPWAQALDATHRGYYKVGDMTQGLGWEA
PDC-100QKR60216.1_898PaOC	241	PGPLDAEGYGVKTSAADLLRFVDANLHPERLDRPWAQALDATHRGYYKVGDMTQGLGWEA
PDC-34WBM27707.1_865PaBCP	241	PGPLDAEGYGVKTSAADLLRFVDANLHPERLDRPWAQALDATHRGYYKVGDMTQGLGWEA
PDC-34WGX11792.1_865PaBCP	241	PGPLDAEGYGVKTSAADLLRFVDANLHPERLDRPWAQALDATHRGYYKVGDMTQGLGWEA
PDC-34WBM27707.1_865PaBOC	241	PGPLDAEGYGVKTSAADLLRFVDANLHPERLDRPWAQALDATHRGYYKVGDMTQGLGWEA
PDC-34WGX11792.1_865PaBOC	241	PGPLDAEGYGVKTSAADLLRFVDANLHPERLDRPWAQALDATHRGYYKVGDMTQGLGWEA
PDC-34ALY72428.1_939PaOC	241	PGPLDAEGYGVKTSAADLLRFVDANLHPERLDRPWAQALDATHRGYYKVGDMTQGLGWEA
PDC-34ASM83699.1_885PaOC	241	PGPLDAEGYGVKTSAADLLRFVDANLHPERLDRPWAQALDATHRGYYKVGDMTQGLGWEA
PDC-34AYW64445.1_883PaOC	241	PGPLDAEGYGVKTSAADLLRFVDANLHPERLDRPWAQALDATHRGYYKVGDMTQGLGWEA
PDC-34UTR27428.1_860PaOC	241	PGPLDAEGYGVKTSAADLLRFVDANLHPERLDRPWAQALDATHRGYYKVGDMTQGLGWEA
PDC-34UOD92021.1_934PaOC	241	PGPLDAEGYGVKTSAADLLRFVDANLHPERLDRPWAQALDATHRGYYKVGDMTQGLGWEA
PDC-34WIV39806.1_860PaOC	241	PGPLDAEGYGVKTSAADLLRFVDANLHPERLDRPWAQALDATHRGYYKVGDMTQGLGWEA
PDC-264QKR42793.1_833PaOC	241	PGPLDAEGYGVKTSAADLLRFVDANLHPERLDRPWAQALDATHRGYYKVGDMTQGLGWEA
PDC-167QIZ28250.1_4314PaOC	241	PGPLDAEGYGVKTSAADLLRFVDANLHPERLDRQWAQALDATHRGYYKVGDMTQGLGWEA
PDC-167UTQ27575.1_842PaOC	241	PGPLDAEGYGVKTSAADLLRFVDANLHPERLDRQWAQALDATHRGYYKVGDMTQGLGWEA
PDC-6QZV30905.1_832PaBCP	241	PGPLDAEGYGVKTSAADLLRFVDANLHPERLDRPWAQALDATHRGYYKVGDMTQGLGWEA
PDC-6QZV30905.1_832PaBOC	241	PGPLDAEGYGVKTSAADLLRFVDANLHPERLDRPWAQALDATHRGYYKVGDMTQGLGWEA
PDC-37WAJ85675.1_843PaBCP	241	PGPLDAEGYGVKTSAADLLRFVDANLHPERLDRPWAQALDATHRGYYKVGDMTQGLGWEA
PDC-37WGX56158.1_855PaBCP	241	PGPLDAEGYGVKTSAADLLRFVDANLHPERLDRPWAQALDATHRGYYKVGDMTQGLGWEA
PDC-37WAJ85675.1_843PaBOC	241	PGPLDAEGYGVKTSAADLLRFVDANLHPERLDRPWAQALDATHRGYYKVGDMTQGLGWEA
PDC-37WGX56158.1_855PaBOC	241	PGPLDAEGYGVKTSAADLLRFVDANLHPERLDRPWAQALDATHRGYYKVGDMTQGLGWEA
PDC-37ANA71197.1_2757.PaOC	241	PGPLDAEGYGVKTSAADLLRFVDANLHPERLDRPWAQALDATHRGYYKVGDMTQGLGWEA
PDC-37WGX14507.1_3195PaOC	241	PGPLDAEGYGVKTSAADLLRFVDANLHPERLDRPWAQALDATHRGYYKVGDMTQGLGWEA
PDC-46AUA93832.1_897PaOC	241	PGPLDAEGYGVKTSAADLLRFVDANLHPERLDRPWAQALDATHRGYYKVGDMTQGLGWEA
PDC-105QGP95004.1_877PaBCP	241	PGPLDAEGYGVKTSAADLLRFVDANLHPERLDRPWAQALDATHRGYYKVGDMTQGLGWEA
PDC-105QGP95004.1_877PaBOC	241	PGPLDAEGYGVKTSAADLLRFVDANLHPERLDRPWAQALDATHRGYYKVGDMTQGLGWEA
PDC-12AVN43882.1_2061.PaOC	241	PGPLDAEGYGVKTSAADLLRFVDANLHPERLDRPWAQALDATHRGYYKVGDMTQGLGWEA
PDC-12QNE63041.1_859PaOC	241	PGPLDAEGYGVKTSAADLLRFVDANLHPERLDRPWAQALDATHRGYYKVGDMTQGLGWEA
PDC-12WGW93864.1_852PaOC	241	PGPLDAEGYGVKTSAADLLRFVDANLHPERLDRPWAQALDATHRGYYKVGDMTQGLGWEA
PDC-12WGX05587.1_852PaOC	241	PGPLDAEGYGVKTSAADLLRFVDANLHPERLDRPWAQALDATHRGYYKVGDMTQGLGWEA
PDC-11QSA85555.1_5581PaBCP	241	PGPLDAEGYGVKTSAADLLRFVDANLHPERLDRPWAQALDATHRGYYKVGDMTQGLGWEA
PDC-11QSA91467.1_5367PaBCP	241	PGPLDAEGYGVKTSAADLLRFVDANLHPERLDRPWAQALDATHRGYYKVGDMTQGLGWEA
PDC-11QSA85555.1_5581PaBOC	241	PGPLDAEGYGVKTSAADLLRFVDANLHPERLDRPWAQALDATHRGYYKVGDMTQGLGWEA
PDC-11QSA91467.1_5367PaBOC	241	PGPLDAEGYGVKTSAADLLRFVDANLHPERLDRPWAQALDATHRGYYKVGDMTQGLGWEA
PDC-11AYQ83203.1_3491PaOC	241	PGPLDAEGYGVKTSAADLLRFVDANLHPERLDRPWAQALDATHRGYYKVGDMTQGLGWEA
PDC-11AYR15208.1_5205PaOC	241	PGPLDAEGYGVKTSAADLLRFVDANLHPERLDRPWAQALDATHRGYYKVGDMTQGLGWEA
PDC-11AZN02324.1_4787PaOC	241	PGPLDAEGYGVKTSAADLLRFVDANLHPERLDRPWAQALDATHRGYYKVGDMTQGLGWEA
PDC-11AZN04123.1_203PaOC	241	PGPLDAEGYGVKTSAADLLRFVDANLHPERLDRPWAQALDATHRGYYKVGDMTQGLGWEA
PDC-11AZN17071.1_5847PaOC	241	PGPLDAEGYGVKTSAADLLRFVDANLHPERLDRPWAQALDATHRGYYKVGDMTQGLGWEA
PDC-11AZN48510.1_4981PaOC	241	PGPLDAEGYGVKTSAADLLRFVDANLHPERLDRPWAQALDATHRGYYKVGDMTQGLGWEA
PDC-11AZN60564.1_5101PaOC	241	PGPLDAEGYGVKTSAADLLRFVDANLHPERLDRPWAQALDATHRGYYKVGDMTQGLGWEA
PDC-11AZN55779.1_6545PaOC	241	PGPLDAEGYGVKTSAADLLRFVDANLHPERLDRPWAQALDATHRGYYKVGDMTQGLGWEA
PDC-11AYF68870.1_744PaOC	241	PGPLDAEGYGVKTSAADLLRFVDANLHPERLDRPWAQALDATHRGYYKVGDMTQGLGWEA
PDC-11QUS21307.1_2433PaOC	241	PGPLDAEGYGVKTSAADLLRFVDANLHPERLDRPWAQALDATHRGYYKVGDMTQGLGWEA
PDC-11UVH87183.1_982PaOC	241	PGPLDAEGYGVKTSAADLLRFVDANLHPERLDRPWAQALDATHRGYYKVGDMTQGLGWEA
PDC-16QCC64126.1_958PaBCP	241	PGPLDAEGYGVKTSAADLLRFVDANLHPERLDRPWAQALDATHRGYYKVGDMTQGLGWEA
PDC-16UBI70916.1_933PaBCP	241	PGPLDAEGYGVKTSAADLLRFVDANLHPERLDRPWAQALDATHRGYYKVGDMTQGLGWEA
PDC-16UTQ84596.1_855PaBCP	241	PGPLDAEGYGVKTSAADLLRFVDANLHPERLDRPWAQALDATHRGYYKVGDMTQGLGWEA
PDC-16QCC64126.1_958PaBOC	241	PGPLDAEGYGVKTSAADLLRFVDANLHPERLDRPWAQALDATHRGYYKVGDMTQGLGWEA
PDC-16UBI70916.1_933PaBOC	241	PGPLDAEGYGVKTSAADLLRFVDANLHPERLDRPWAQALDATHRGYYKVGDMTQGLGWEA
PDC-16UTQ84596.1_855PaBOC	241	PGPLDAEGYGVKTSAADLLRFVDANLHPERLDRPWAQALDATHRGYYKVGDMTQGLGWEA
PDC-16ALS12949.1_2977.PaOC	241	PGPLDAEGYGVKTSAADLLRFVDANLHPERLDRPWAQALDATHRGYYKVGDMTQGLGWEA
PDC-16AYW58201.1_855PaOC	241	PGPLDAEGYGVKTSAADLLRFVDANLHPERLDRPWAQALDATHRGYYKVGDMTQGLGWEA
PDC-16QDR00398.1_890PaOC	241	PGPLDAEGYGVKTSAADLLRFVDANLHPERLDRPWAQALDATHRGYYKVGDMTQGLGWEA
PDC-16QGJ31628.1_895PaOC	241	PGPLDAEGYGVKTSAADLLRFVDANLHPERLDRPWAQALDATHRGYYKVGDMTQGLGWEA
PDC-16QKG34934.1_1840PaOC	241	PGPLDAEGYGVKTSAADLLRFVDANLHPERLDRPWAQALDATHRGYYKVGDMTQGLGWEA
PDC-16QOQ64874.1_122PaOC	241	PGPLDAEGYGVKTSAADLLRFVDANLHPERLDRPWAQALDATHRGYYKVGDMTQGLGWEA
PDC-16QVQ73157.1_860PaOC	241	PGPLDAEGYGVKTSAADLLRFVDANLHPERLDRPWAQALDATHRGYYKVGDMTQGLGWEA
PDC-16UOC64480.1_827PaOC	241	PGPLDAEGYGVKTSAADLLRFVDANLHPERLDRPWAQALDATHRGYYKVGDMTQGLGWEA
PDC-16UOC70564.1_826PaOC	241	PGPLDAEGYGVKTSAADLLRFVDANLHPERLDRPWAQALDATHRGYYKVGDMTQGLGWEA
PDC-16UOC76649.1_827PaOC	241	PGPLDAEGYGVKTSAADLLRFVDANLHPERLDRPWAQALDATHRGYYKVGDMTQGLGWEA
PDC-16UOC82740.1_827PaOC	241	PGPLDAEGYGVKTSAADLLRFVDANLHPERLDRPWAQALDATHRGYYKVGDMTQGLGWEA
PDC-16UOC88825.1_827PaOC	241	PGPLDAEGYGVKTSAADLLRFVDANLHPERLDRPWAQALDATHRGYYKVGDMTQGLGWEA
PDC-16UOE68701.1_827PaOC	241	PGPLDAEGYGVKTSAADLLRFVDANLHPERLDRPWAQALDATHRGYYKVGDMTQGLGWEA
PDC-16WDC65088.1_844PaOC	241	PGPLDAEGYGVKTSAADLLRFVDANLHPERLDRPWAQALDATHRGYYKVGDMTQGLGWEA
PDC-16WGW70130.1_845PaOC	241	PGPLDAEGYGVKTSAADLLRFVDANLHPERLDRPWAQALDATHRGYYKVGDMTQGLGWEA
PDC-16WGW76010.1_845PaOC	241	PGPLDAEGYGVKTSAADLLRFVDANLHPERLDRPWAQALDATHRGYYKVGDMTQGLGWEA
PDC-16WGW81892.1_845PaOC	241	PGPLDAEGYGVKTSAADLLRFVDANLHPERLDRPWAQALDATHRGYYKVGDMTQGLGWEA
PDC-16WGX43443.1_806PaOC	241	PGPLDAEGYGVKTSAADLLRFVDANLHPERLDRPWAQALDATHRGYYKVGDMTQGLGWEA
PDC-16WJM37210.1_871PaOC	241	PGPLDAEGYGVKTSAADLLRFVDANLHPERLDRPWAQALDATHRGYYKVGDMTQGLGWEA
PDC-59UQZ18314.1_902PaOC	241	PGPLDAEGYGVKTSAADLLRFVDANLHPERLDRPWAQALDATHRGYYKVGDMTQGLGWEA
PDC-142WGW57525.1_830PaBCP	241	PGPLDAEGYGVKTSAADLLRFVDANLHPERLDRPWAQALDATHRGYYKVGDMTQGLGWEA
PDC-142WGW32210.1_830PaBCP	241	PGPLDAEGYGVKTSAADLLRFVDANLHPERLDRPWAQALDATHRGYYKVGDMTQGLGWEA
PDC-142WGW44776.1_830PaBCP	241	PGPLDAEGYGVKTSAADLLRFVDANLHPERLDRPWAQALDATHRGYYKVGDMTQGLGWEA
PDC-142WGW57525.1_830PaBOC	241	PGPLDAEGYGVKTSAADLLRFVDANLHPERLDRPWAQALDATHRGYYKVGDMTQGLGWEA
PDC-142WGW32210.1_830PaBOC	241	PGPLDAEGYGVKTSAADLLRFVDANLHPERLDRPWAQALDATHRGYYKVGDMTQGLGWEA
PDC-142WGW44776.1_830PaBOC	241	PGPLDAEGYGVKTSAADLLRFVDANLHPERLDRPWAQALDATHRGYYKVGDMTQGLGWEA
PDC-142AON05138.1_825PaOC	241	PGPLDAEGYGVKTSAADLLRFVDANLHPERLDRPWAQALDATHRGYYKVGDMTQGLGWEA
PDC-142AON11127.1_825PaOC	241	PGPLDAEGYGVKTSAADLLRFVDANLHPERLDRPWAQALDATHRGYYKVGDMTQGLGWEA
PDC-142AON23122.1_825PaOC	241	PGPLDAEGYGVKTSAADLLRFVDANLHPERLDRPWAQALDATHRGYYKVGDMTQGLGWEA
PDC-142AON29110.1_825PaOC	241	PGPLDAEGYGVKTSAADLLRFVDANLHPERLDRPWAQALDATHRGYYKVGDMTQGLGWEA
PDC-142AON41099.1_825PaOC	241	PGPLDAEGYGVKTSAADLLRFVDANLHPERLDRPWAQALDATHRGYYKVGDMTQGLGWEA
PDC-142AON47112.1_824PaOC	241	PGPLDAEGYGVKTSAADLLRFVDANLHPERLDRPWAQALDATHRGYYKVGDMTQGLGWEA
PDC-142AON17115.1_824PaOC	241	PGPLDAEGYGVKTSAADLLRFVDANLHPERLDRPWAQALDATHRGYYKVGDMTQGLGWEA
PDC-142AON35116.1_825PaOC	241	PGPLDAEGYGVKTSAADLLRFVDANLHPERLDRPWAQALDATHRGYYKVGDMTQGLGWEA
PDC-19aWGW51434.1_1207PaBCP	241	PGPLDAEGYGVKTSAADLLRFVDANLHPERLDRPWAQALDATHRGYYKVGDMTQGLGWEA

```
PDC-19aWGW37666.1_6817PaBCP    241 PGPLDAEGYGVKTSAADLLRFVDANLHPERLDRPWAQALDATHRGYYKVGDMTQGLGWEA
PDC-19aWGW59864.1_3660PaBCP    241 PGPLDAEGYGVKTSAADLLRFVDANLHPERLDRPWAQALDATHRGYYKVGDMTQGLGWEA
PDC-19aWGX49838.1_877PaBCP     241 PGPLDAEGYGVKTSAADLLRFVDANLHPERLDRPWAQALDATHRGYYKVGDMTQGLGWEA
PDC-19aWGX24308.1_877PaBCP     241 PGPLDAEGYGVKTSAADLLRFVDANLHPERLDRPWAQALDATHRGYYKVGDMTQGLGWEA
PDC-19aWGX35552.1_5487PaBCP    241 PGPLDAEGYGVKTSAADLLRFVDANLHPERLDRPWAQALDATHRGYYKVGDMTQGLGWEA
PDC-19aWKA37694.1_2446PaBCP    241 PGPLDAEGYGVKTSAADLLRFVDANLHPERLDRPWAQALDATHRGYYKVGDMTQGLGWEA
PDC-19aWGW51434.1_1207PaBOC    241 PGPLDAEGYGVKTSAADLLRFVDANLHPERLDRPWAQALDATHRGYYKVGDMTQGLGWEA
PDC-19aWGW37666.1_6817PaBOC    241 PGPLDAEGYGVKTSAADLLRFVDANLHPERLDRPWAQALDATHRGYYKVGDMTQGLGWEA
PDC-19aWGW59864.1_3660PaBOC    241 PGPLDAEGYGVKTSAADLLRFVDANLHPERLDRPWAQALDATHRGYYKVGDMTQGLGWEA
PDC-19aWGX49838.1_877PaBOC     241 PGPLDAEGYGVKTSAADLLRFVDANLHPERLDRPWAQALDATHRGYYKVGDMTQGLGWEA
PDC-19aWGX24308.1_877PaBOC     241 PGPLDAEGYGVKTSAADLLRFVDANLHPERLDRPWAQALDATHRGYYKVGDMTQGLGWEA
PDC-19aWGX35552.1_5487PaBOC    241 PGPLDAEGYGVKTSAADLLRFVDANLHPERLDRPWAQALDATHRGYYKVGDMTQGLGWEA
PDC-19aWKA37694.1_2446PaBOC    241 PGPLDAEGYGVKTSAADLLRFVDANLHPERLDRPWAQALDATHRGYYKVGDMTQGLGWEA
PDC-19aAPB63370.1_616PaOC      241 PGPLDAEGYGVKTSAADLLRFVDANLHPERLDRPWAQALDATHRGYYKVGDMTQGLGWEA
PDC-19aARI89529.1_870PaOC      241 PGPLDAEGYGVKTSAADLLRFVDANLHPERLDRPWAQALDATHRGYYKVGDMTQGLGWEA
PDC-19aARI95964.1_871PaOC      241 PGPLDAEGYGVKTSAADLLRFVDANLHPERLDRPWAQALDATHRGYYKVGDMTQGLGWEA
PDC-19aASA13594.1_977PaOC      241 PGPLDAEGYGVKTSAADLLRFVDANLHPERLDRPWAQALDATHRGYYKVGDMTQGLGWEA
PDC-19aASA27558.1_962PaOC      241 PGPLDAEGYGVKTSAADLLRFVDANLHPERLDRPWAQALDATHRGYYKVGDMTQGLGWEA
PDC-19aASD01901.1_971PaOC      241 PGPLDAEGYGVKTSAADLLRFVDANLHPERLDRPWAQALDATHRGYYKVGDMTQGLGWEA
PDC-19aUAC92160.1_908PaOC      241 PGPLDAEGYGVKTSAADLLRFVDANLHPERLDRPWAQALDATHRGYYKVGDMTQGLGWEA
PDC-19aUAC98839.1_942PaOC      241 PGPLDAEGYGVKTSAADLLRFVDANLHPERLDRPWAQALDATHRGYYKVGDMTQGLGWEA
PDC-19aUTQ33604.1_830PaOC      241 PGPLDAEGYGVKTSAADLLRFVDANLHPERLDRPWAQALDATHRGYYKVGDMTQGLGWEA
PDC-19aUOO58611.1_830PaOC      241 PGPLDAEGYGVKTSAADLLRFVDANLHPERLDRPWAQALDATHRGYYKVGDMTQGLGWEA
PDC-19aWDA50910.1_883PaOC      241 PGPLDAEGYGVKTSAADLLRFVDANLHPERLDRPWAQALDATHRGYYKVGDMTQGLGWEA
PDC-19aWGX30960.1_941PaOC      241 PGPLDAEGYGVKTSAADLLRFVDANLHPERLDRPWAQALDATHRGYYKVGDMTQGLGWEA
PDC-19aWGX61448.1_5603PaOC     241 PGPLDAEGYGVKTSAADLLRFVDANLHPERLDRPWAQALDATHRGYYKVGDMTQGLGWEA
PDC-19aWGX82118.1_941PaOC      241 PGPLDAEGYGVKTSAADLLRFVDANLHPERLDRPWAQALDATHRGYYKVGDMTQGLGWEA
PDC-19aWIO49783.1_187PaOC      241 PGPLDAEGYGVKTSAADLLRFVDANLHPERLDRPWAQALDATHRGYYKVGDMTQGLGWEA
PDC-24QZV43725.1_941PaBCP     241 PGPLDAEGYGVKTSAADLLRFVDANLHPERLDRPWAQALDATHRGYYKVGDMTQGLGWEA
PDC-24QZV43725.1_941PaBOC     241 PGPLDAEGYGVKTSAADLLRFVDANLHPERLDRPWAQALDATHRGYYKVGDMTQGLGWEA
PDC-24QDR06751.1_860PaOC      241 PGPLDAEGYGVKTSAADLLRFVDANLHPERLDRPWAQALDATHRGYYKVGDMTQGLGWEA
PDC-24UGR36796.1_3412PaOC     241 PGPLDAEGYGVKTSAADLLRFVDANLHPERLDRPWAQALDATHRGYYKVGDMTQGLGWEA
PDC-24UTQ39921.1_833PaOC      241 PGPLDAEGYGVKTSAADLLRFVDANLHPERLDRPWAQALDATHRGYYKVGDMTQGLGWEA
PDC-137QKE94864.1_852PaOC     241 PGPLDAEGYGVKTSAADLLRFVDANLHPERLDRPWAQALDATHRGYYKVGDMTQGLGWEA
PDC-147UGR30133.1_1950PaOC    241 PGPLDAEGYGVKTSAADLLRFVDANLHPERLDRPWAQALDATHRGYYKVGDMTQGLGWEA
PDC-8AYY41152.1_5352PaBCP     241 PGPLDAEGYGVKTSAADLLRFVDANLHPERLDRPWAQALDATHRGYYKVGDMTQGLGWEA
PDC-8QOE14337.1_211PaBCP      241 PGPLDAEGYGVKTSAADLLRFVDANLHPERLDRPWAQALDATHRGYYKVGDMTQGLGWEA
PDC-8QPN15577.1_911PaBCP      241 PGPLDAEGYGVKTSAADLLRFVDANLHPERLDRPWAQALDATHRGYYKVGDMTQGLGWEA
PDC-8QPN23993.1_908PaBCP      241 PGPLDAEGYGVKTSAADLLRFVDANLHPERLDRPWAQALDATHRGYYKVGDMTQGLGWEA
PDC-8QUE64702.1_909PaBCP      241 PGPLDAEGYGVKTSAADLLRFVDANLHPERLDRPWAQALDATHRGYYKVGDMTQGLGWEA
PDC-8QYA88773.1_2249PaBCP     241 PGPLDAEGYGVKTSAADLLRFVDANLHPERLDRPWAQALDATHRGYYKVGDMTQGLGWEA
PDC-8QYF01720.1_5249PaBCP     241 PGPLDAEGYGVKTSAADLLRFVDANLHPERLDRPWAQALDATHRGYYKVGDMTQGLGWEA
PDC-8QZU99504.1_908PaBCP      241 PGPLDAEGYGVKTSAADLLRFVDANLHPERLDRPWAQALDATHRGYYKVGDMTQGLGWEA
PDC-8QZV05727.1_908PaBCP      241 PGPLDAEGYGVKTSAADLLRFVDANLHPERLDRPWAQALDATHRGYYKVGDMTQGLGWEA
PDC-8QZV12028.1_910PaBCP      241 PGPLDAEGYGVKTSAADLLRFVDANLHPERLDRPWAQALDATHRGYYKVGDMTQGLGWEA
PDC-8QZV18112.1_863PaBCP      241 PGPLDAEGYGVKTSAADLLRFVDANLHPERLDRPWAQALDATHRGYYKVGDMTQGLGWEA
PDC-8QZV24387.1_912PaBCP      241 PGPLDAEGYGVKTSAADLLRFVDANLHPERLDRPWAQALDATHRGYYKVGDMTQGLGWEA
PDC-8QZV68851.1_908PaBCP      241 PGPLDAEGYGVKTSAADLLRFVDANLHPERLDRPWAQALDATHRGYYKVGDMTQGLGWEA
PDC-8QZV81338.1_870PaBCP      241 PGPLDAEGYGVKTSAADLLRFVDANLHPERLDRPWAQALDATHRGYYKVGDMTQGLGWEA
PDC-8QZV87769.1_908PaBCP      241 PGPLDAEGYGVKTSAADLLRFVDANLHPERLDRPWAQALDATHRGYYKVGDMTQGLGWEA
PDC-8QZV94048.1_908PaBCP      241 PGPLDAEGYGVKTSAADLLRFVDANLHPERLDRPWAQALDATHRGYYKVGDMTQGLGWEA
PDC-8UXH53497.1_877PaBCP      241 PGPLDAEGYGVKTSAADLLRFVDANLHPERLDRPWAQALDATHRGYYKVGDMTQGLGWEA
PDC-8UXH66521.1_877PaBCP      241 PGPLDAEGYGVKTSAADLLRFVDANLHPERLDRPWAQALDATHRGYYKVGDMTQGLGWEA
PDC-8UXH70664.1_4987PaBCP     241 PGPLDAEGYGVKTSAADLLRFVDANLHPERLDRPWAQALDATHRGYYKVGDMTQGLGWEA
PDC-8UUW81479.1_5273PaBCP     241 PGPLDAEGYGVKTSAADLLRFVDANLHPERLDRPWAQALDATHRGYYKVGDMTQGLGWEA
PDC-8WDM07130.1_910PaBCP      241 PGPLDAEGYGVKTSAADLLRFVDANLHPERLDRPWAQALDATHRGYYKVGDMTQGLGWEA
PDC-8WHM62936.1_909PaBCP      241 PGPLDAEGYGVKTSAADLLRFVDANLHPERLDRPWAQALDATHRGYYKVGDMTQGLGWEA
PDC-8WHM69354.1_909PaBCP      241 PGPLDAEGYGVKTSAADLLRFVDANLHPERLDRPWAQALDATHRGYYKVGDMTQGLGWEA
PDC-8WHM75768.1_909PaBCP      241 PGPLDAEGYGVKTSAADLLRFVDANLHPERLDRPWAQALDATHRGYYKVGDMTQGLGWEA
PDC-8WHM84263.1_909PaBCP      241 PGPLDAEGYGVKTSAADLLRFVDANLHPERLDRPWAQALDATHRGYYKVGDMTQGLGWEA
PDC-8AYY41152.1_5352PaBOC     241 PGPLDAEGYGVKTSAADLLRFVDANLHPERLDRPWAQALDATHRGYYKVGDMTQGLGWEA
PDC-8QOE14337.1_211PaBOC      241 PGPLDAEGYGVKTSAADLLRFVDANLHPERLDRPWAQALDATHRGYYKVGDMTQGLGWEA
PDC-8QPN15577.1_911PaBOC      241 PGPLDAEGYGVKTSAADLLRFVDANLHPERLDRPWAQALDATHRGYYKVGDMTQGLGWEA
PDC-8QPN23993.1_908PaBOC      241 PGPLDAEGYGVKTSAADLLRFVDANLHPERLDRPWAQALDATHRGYYKVGDMTQGLGWEA
PDC-8QUE64702.1_909PaBOC      241 PGPLDAEGYGVKTSAADLLRFVDANLHPERLDRPWAQALDATHRGYYKVGDMTQGLGWEA
PDC-8QYA88773.1_2249PaBOC     241 PGPLDAEGYGVKTSAADLLRFVDANLHPERLDRPWAQALDATHRGYYKVGDMTQGLGWEA
PDC-8QYF01720.1_5249PaBOC     241 PGPLDAEGYGVKTSAADLLRFVDANLHPERLDRPWAQALDATHRGYYKVGDMTQGLGWEA
PDC-8QZU99504.1_908PaBOC      241 PGPLDAEGYGVKTSAADLLRFVDANLHPERLDRPWAQALDATHRGYYKVGDMTQGLGWEA
PDC-8QZV05727.1_908PaBOC      241 PGPLDAEGYGVKTSAADLLRFVDANLHPERLDRPWAQALDATHRGYYKVGDMTQGLGWEA
PDC-8QZV12028.1_910PaBOC      241 PGPLDAEGYGVKTSAADLLRFVDANLHPERLDRPWAQALDATHRGYYKVGDMTQGLGWEA
PDC-8QZV18112.1_863PaBOC      241 PGPLDAEGYGVKTSAADLLRFVDANLHPERLDRPWAQALDATHRGYYKVGDMTQGLGWEA
PDC-8QZV24387.1_912PaBOC      241 PGPLDAEGYGVKTSAADLLRFVDANLHPERLDRPWAQALDATHRGYYKVGDMTQGLGWEA
PDC-8QZV68851.1_908PaBOC      241 PGPLDAEGYGVKTSAADLLRFVDANLHPERLDRPWAQALDATHRGYYKVGDMTQGLGWEA
PDC-8QZV81338.1_870PaBOC      241 PGPLDAEGYGVKTSAADLLRFVDANLHPERLDRPWAQALDATHRGYYKVGDMTQGLGWEA
PDC-8QZV87769.1_908PaBOC      241 PGPLDAEGYGVKTSAADLLRFVDANLHPERLDRPWAQALDATHRGYYKVGDMTQGLGWEA
PDC-8QZV94048.1_908PaBOC      241 PGPLDAEGYGVKTSAADLLRFVDANLHPERLDRPWAQALDATHRGYYKVGDMTQGLGWEA
PDC-8UXH53497.1_877PaBOC      241 PGPLDAEGYGVKTSAADLLRFVDANLHPERLDRPWAQALDATHRGYYKVGDMTQGLGWEA
PDC-8UXH66521.1_877PaBOC      241 PGPLDAEGYGVKTSAADLLRFVDANLHPERLDRPWAQALDATHRGYYKVGDMTQGLGWEA
PDC-8UXH70664.1_4987PaBOC     241 PGPLDAEGYGVKTSAADLLRFVDANLHPERLDRPWAQALDATHRGYYKVGDMTQGLGWEA
PDC-8UUW81479.1_5273PaBOC     241 PGPLDAEGYGVKTSAADLLRFVDANLHPERLDRPWAQALDATHRGYYKVGDMTQGLGWEA
PDC-8WDM07130.1_910PaBOC      241 PGPLDAEGYGVKTSAADLLRFVDANLHPERLDRPWAQALDATHRGYYKVGDMTQGLGWEA
PDC-8WHM62936.1_909PaBOC      241 PGPLDAEGYGVKTSAADLLRFVDANLHPERLDRPWAQALDATHRGYYKVGDMTQGLGWEA
PDC-8WHM69354.1_909PaBOC      241 PGPLDAEGYGVKTSAADLLRFVDANLHPERLDRPWAQALDATHRGYYKVGDMTQGLGWEA
PDC-8WHM75768.1_909PaBOC      241 PGPLDAEGYGVKTSAADLLRFVDANLHPERLDRPWAQALDATHRGYYKVGDMTQGLGWEA
PDC-8WHM84263.1_909PaBOC      241 PGPLDAEGYGVKTSAADLLRFVDANLHPERLDRPWAQALDATHRGYYKVGDMTQGLGWEA
PDC-8AMA35328.1_919.PaOC      241 PGPLDAEGYGVKTSAADLLRFVDANLHPERLDRPWAQALDATHRGYYKVGDMTQGLGWEA
PDC-8ANI06966.1_119PaOC       241 PGPLDAEGYGVKTSAADLLRFVDANLHPERLDRPWAQALDATHRGYYKVGDMTQGLGWEA
PDC-8AYW70752.1_952PaOC       241 PGPLDAEGYGVKTSAADLLRFVDANLHPERLDRPWAQALDATHRGYYKVGDMTQGLGWEA
PDC-8AYZ76972.1_2272PaOC      241 PGPLDAEGYGVKTSAADLLRFVDANLHPERLDRPWAQALDATHRGYYKVGDMTQGLGWEA
PDC-8QKP85289.1_778PaOC       241 PGPLDAEGYGVKTSAADLLRFVDANLHPERLDRPWAQALDATHRGYYKVGDMTQGLGWEA
PDC-8QKR30573.1_5806PaOC      241 PGPLDAEGYGVKTSAADLLRFVDANLHPERLDRPWAQALDATHRGYYKVGDMTQGLGWEA
PDC-8QLF19879.1_939PaOC       241 PGPLDAEGYGVKTSAADLLRFVDANLHPERLDRPWAQALDATHRGYYKVGDMTQGLGWEA
PDC-8QYE59452.1_5107PaOC      241 PGPLDAEGYGVKTSAADLLRFVDANLHPERLDRPWAQALDATHRGYYKVGDMTQGLGWEA
PDC-8QZV98104.1_4956PaOC      241 PGPLDAEGYGVKTSAADLLRFVDANLHPERLDRPWAQALDATHRGYYKVGDMTQGLGWEA
PDC-8UFK73541.1_1068PaOC      241 PGPLDAEGYGVKTSAADLLRFVDANLHPERLDRPWAQALDATHRGYYKVGDMTQGLGWEA
PDC-8UGR33975.2_368PaOC       241 PGPLDAEGYGVKTSAADLLRFVDANLHPERLDRPWAQALDATHRGYYKVGDMTQGLGWEA
PDC-8UTN26696.1_862PaOC       241 PGPLDAEGYGVKTSAADLLRFVDANLHPERLDRPWAQALDATHRGYYKVGDMTQGLGWEA
PDC-8UTQ91314.1_910PaOC       241 PGPLDAEGYGVKTSAADLLRFVDANLHPERLDRPWAQALDATHRGYYKVGDMTQGLGWEA
PDC-8UNK85299.1_868PaOC       241 PGPLDAEGYGVKTSAADLLRFVDANLHPERLDRPWAQALDATHRGYYKVGDMTQGLGWEA
PDC-8UNK97662.1_866PaOC       241 PGPLDAEGYGVKTSAADLLRFVDANLHPERLDRPWAQALDATHRGYYKVGDMTQGLGWEA
PDC-8USI83494.1_833PaOC       241 PGPLDAEGYGVKTSAADLLRFVDANLHPERLDRPWAQALDATHRGYYKVGDMTQGLGWEA
PDC-8WAO16415.1_5521PaOC      241 PGPLDAEGYGVKTSAADLLRFVDANLHPERLDRPWAQALDATHRGYYKVGDMTQGLGWEA
PDC-98QZD62144.1_4346PaBCP    241 PGPLDAEGYGVKTSAADLLRFVDANLHPERLDRPWAQALDATHRGYYKVGDMTQGLGWEA
```

PDC-98QZH48390.1_4214PaBCP	241	PGPLDAEGYGVKTSAADLLRFVDANLHPERLDRPWAQALDATHRGYYKVGDMTQGLGWEA
PDC-98QZD62144.1_4346PaBOC	241	PGPLDAEGYGVKTSAADLLRFVDANLHPERLDRPWAQALDATHRGYYKVGDMTQGLGWEA
PDC-98QZH48390.1_4214PaBOC	241	PGPLDAEGYGVKTSAADLLRFVDANLHPERLDRPWAQALDATHRGYYKVGDMTQGLGWEA
PDC-98ALP56563.1_1203.PaOC	241	PGPLDAEGYGVKTSAADLLRFVDANLHPERLDRPWAQALDATHRGYYKVGDMTQGLGWEA
PDC-98UTL97623.1_830PaOC	241	PGPLDAEGYGVKTSAADLLRFVDANLHPERLDRPWAQALDATHRGYYKVGDMTQGLGWEA
PDC-457UTN40061.1_863PaOC	241	PGPLDAEGYGVKTSAADLLRFVDANLHPERLDRPWAQALDATHRGYYKVGDMTQGLGWEA
PDC-23UXI89283.1_830PaOC	241	PGPLDAEGYGVKTSAADLLRFVDANLHPERLDRPWAQALDATHRGYYKVGDMTQGLGWEA
PDC-245AUA75408.1_866PaOC	241	PGPLDAEGYGVKTSAADLLRFVDANLHPERLDRPWAQALDATHRGYYKVGDMTQGLGWEA
PDC-31QZW06655.1_840PaBCP	241	PGPLDAEGYGVKTSAADLLRFVDANLHPERLDRPWAQALDATHRGYYKVGDMTQGLGWEA
PDC-31QZW06655.1_840PaBOC	241	PGPLDAEGYGVKTSAADLLRFVDANLHPERLDRPWAQALDATHRGYYKVGDMTQGLGWEA
PDC-31AXN27248.1_931PaOC	241	PGPLDAEGYGVKTSAADLLRFVDANLHPERLDRPWAQALDATHRGYYKVGDMTQGLGWEA
PDC-31QBC07904.1_3021PaOC	241	PGPLDAEGYGVKTSAADLLRFVDANLHPERLDRPWAQALDATHRGYYKVGDMTQGLGWEA
PDC-31UFM90275.1_905PaOC	241	PGPLDAEGYGVKTSAADLLRFVDANLHPERLDRPWAQALDATHRGYYKVGDMTQGLGWEA
PDC-31UFM98870.1_905PaOC	241	PGPLDAEGYGVKTSAADLLRFVDANLHPERLDRPWAQALDATHRGYYKVGDMTQGLGWEA
PDC-31UNT25139.1_901PaOC	241	PGPLDAEGYGVKTSAADLLRFVDANLHPERLDRPWAQALDATHRGYYKVGDMTQGLGWEA
PDC-31UWJ96607.1_4267PaOC	241	PGPLDAEGYGVKTSAADLLRFVDANLHPERLDRPWAQALDATHRGYYKVGDMTQGLGWEA
PDC-31WGX88300.1_823PaOC	241	PGPLDAEGYGVKTSAADLLRFVDANLHPERLDRPWAQALDATHRGYYKVGDMTQGLGWEA
PDC-31WHM08550.1_859PaOC	241	PGPLDAEGYGVKTSAADLLRFVDANLHPERLDRPWAQALDATHRGYYKVGDMTQGLGWEA
PDC-39AYK21377.1_872PaOC	241	PGPLDAEGYGVKTSAADLLRFVDANLHPERLDRPWAQALDATHRGYYKVGDMTQGLGWEA
PDC-39UJC21946.1_837PaOC	241	PGPLDAEGYGVKTSAADLLRFVDANLHPERLDRPWAQALDATHRGYYKVGDMTQGLGWEA
PDC-30UPL39337.1_928PaBCP	241	PGPLDAEGYGVKTSAADLLRFVDANLHPERLDRPWAQALDATHRGYYKVGDMTQGLGWEA
PDC-30UPL39337.1_928PaBOC	241	PGPLDAEGYGVKTSAADLLRFVDANLHPERLDRPWAQALDATHRGYYKVGDMTQGLGWEA
PDC-30UHL95932.1_5029PaOC	241	PGPLDAEGYGVKTSAADLLRFVDANLHPERLDRPWAQALDATHRGYYKVGDMTQGLGWEA
PDC-30UML99445.1_838PaOC	241	PGPLDAEGYGVKTSAADLLRFVDANLHPERLDRPWAQALDATHRGYYKVGDMTQGLGWEA
PDC-80WAJ79202.1_935PaOC	241	PGPLDAGGYGVKTSAADLLRFVDANLHPERLDRPWAQALDATHRGYYKVGDMTQGLGWEA
8SDL_1|Chain	241	PGPLDAEGYGVKTSAADLLRFVDANLHPERLDRPWAQALDATHRGYYKVGDMTQGLGWEA
PDC-3UTN33167.1_589PaBCP	241	PGPLDAEGYGVKTSAADLLRFVDANLHPERLDRPWAQALDATHRGYYKVGDMTQGLGWEA
PDC-3WCI38158.1_984PaBCP	241	PGPLDAEGYGVKTSAADLLRFVDANLHPERLDRPWAQALDATHRGYYKVGDMTQGLGWEA
PDC-3WCI50909.1_867PaBCP	241	PGPLDAEGYGVKTSAADLLRFVDANLHPERLDRPWAQALDATHRGYYKVGDMTQGLGWEA
PDC-3WCI90621.1_961PaBCP	241	PGPLDAEGYGVKTSAADLLRFVDANLHPERLDRPWAQALDATHRGYYKVGDMTQGLGWEA
PDC-3WEO48793.1_832PaBCP	241	PGPLDAEGYGVKTSAADLLRFVDANLHPERLDRPWAQALDATHRGYYKVGDMTQGLGWEA
PDC-3CAI9907424.1_19963]PaBCP	241	PGPLDAEGYGVKTSAADLLRFVDANLHPERLDRPWAQALDATHRGYYKVGDMTQGLGWEA
PDC-3UTN33167.1_589PaBOC	241	PGPLDAEGYGVKTSAADLLRFVDANLHPERLDRPWAQALDATHRGYYKVGDMTQGLGWEA
PDC-3WCI38158.1_984PaBOC	241	PGPLDAEGYGVKTSAADLLRFVDANLHPERLDRPWAQALDATHRGYYKVGDMTQGLGWEA
PDC-3WCI50909.1_867PaBOC	241	PGPLDAEGYGVKTSAADLLRFVDANLHPERLDRPWAQALDATHRGYYKVGDMTQGLGWEA
PDC-3WCI90621.1_961PaBOC	241	PGPLDAEGYGVKTSAADLLRFVDANLHPERLDRPWAQALDATHRGYYKVGDMTQGLGWEA
PDC-3WEO48793.1_832PaBOC	241	PGPLDAEGYGVKTSAADLLRFVDANLHPERLDRPWAQALDATHRGYYKVGDMTQGLGWEA
PDC-3CAI9907424.1_19963]PaBOC	241	PGPLDAEGYGVKTSAADLLRFVDANLHPERLDRPWAQALDATHRGYYKVGDMTQGLGWEA
PDC-3AHA17954.1_839.PaOC	241	PGPLDAEGYGVKTSAADLLRFVDANLHPERLDRPWAQALDATHRGYYKVGDMTQGLGWEA
PDC-3ALE46683.1_840.PaOC	241	PGPLDAEGYGVKTSAADLLRFVDANLHPERLDRPWAQALDATHRGYYKVGDMTQGLGWEA
PDC-3ARC78082.1_863.PaOC	241	PGPLDAEGYGVKTSAADLLRFVDANLHPERLDRPWAQALDATHRGYYKVGDMTQGLGWEA
PDC-3ARG53386.1_5885PaOC	241	PGPLDAEGYGVKTSAADLLRFVDANLHPERLDRPWAQALDATHRGYYKVGDMTQGLGWEA
PDC-3WAC79950.1_4482PaOC	241	PGPLDAEGYGVKTSAADLLRFVDANLHPERLDRPWAQALDATHRGYYKVGDMTQGLGWEA
PDC-3WAE22012.1_4863PaOC	241	PGPLDAEGYGVKTSAADLLRFVDANLHPERLDRPWAQALDATHRGYYKVGDMTQGLGWEA
PDC-3WAE29433.1_777PaOC	241	PGPLDAEGYGVKTSAADLLRFVDANLHPERLDRPWAQALDATHRGYYKVGDMTQGLGWEA
PDC-3WAW42145.1_847PaOC	241	PGPLDAEGYGVKTSAADLLRFVDANLHPERLDRPWAQALDATHRGYYKVGDMTQGLGWEA
PDC-3WCI57477.1_911PaOC	241	PGPLDAEGYGVKTSAADLLRFVDANLHPERLDRPWAQALDATHRGYYKVGDMTQGLGWEA
PDC-3WCI77402.1_900PaOC	241	PGPLDAEGYGVKTSAADLLRFVDANLHPERLDRPWAQALDATHRGYYKVGDMTQGLGWEA
PDC-3WCI64002.1_913PaOC	241	PGPLDAEGYGVKTSAADLLRFVDANLHPERLDRPWAQALDATHRGYYKVGDMTQGLGWEA
PDC-3WCI83994.1_905PaOC	241	PGPLDAEGYGVKTSAADLLRFVDANLHPERLDRPWAQALDATHRGYYKVGDMTQGLGWEA
PDC-3WJM48814.1_842PaOC	241	PGPLDAEGYGVKTSAADLLRFVDANLHPERLDRPWAQALDATHRGYYKVGDMTQGLGWEA
PDC-374ANP60834.1_3741PaBCP	241	PGPLDAEGYGVKTSAADLLRFVDANLHPERLDRPWAQALDATHRGYYKVGDMTQGLGWEA
PDC-374AYZ86971.1_5950PaBCP	241	PGPLDAEGYGVKTSAADLLRFVDANLHPERLDRPWAQALDATHRGYYKVGDMTQGLGWEA
PDC-374QZV75115.1_843PaBCP	241	PGPLDAEGYGVKTSAADLLRFVDANLHPERLDRPWAQALDATHRGYYKVGDMTQGLGWEA
PDC-374QZW12711.1_843PaBCP	241	PGPLDAEGYGVKTSAADLLRFVDANLHPERLDRPWAQALDATHRGYYKVGDMTQGLGWEA
PDC-374UEG06685.1_907PaBCP	241	PGPLDAEGYGVKTSAADLLRFVDANLHPERLDRPWAQALDATHRGYYKVGDMTQGLGWEA
PDC-374UGW95802.1_984PaBCP	241	PGPLDAEGYGVKTSAADLLRFVDANLHPERLDRPWAQALDATHRGYYKVGDMTQGLGWEA
PDC-374WGW26222.1_839PaBCP	241	PGPLDAEGYGVKTSAADLLRFVDANLHPERLDRPWAQALDATHRGYYKVGDMTQGLGWEA
PDC-374ANP60834.1_3741PaBOC	241	PGPLDAEGYGVKTSAADLLRFVDANLHPERLDRPWAQALDATHRGYYKVGDMTQGLGWEA
PDC-374AYZ86971.1_5950PaBOC	241	PGPLDAEGYGVKTSAADLLRFVDANLHPERLDRPWAQALDATHRGYYKVGDMTQGLGWEA
PDC-374QZV75115.1_843PaBOC	241	PGPLDAEGYGVKTSAADLLRFVDANLHPERLDRPWAQALDATHRGYYKVGDMTQGLGWEA
PDC-374QZW12711.1_843PaBOC	241	PGPLDAEGYGVKTSAADLLRFVDANLHPERLDRPWAQALDATHRGYYKVGDMTQGLGWEA
PDC-374UEG06685.1_907PaBOC	241	PGPLDAEGYGVKTSAADLLRFVDANLHPERLDRPWAQALDATHRGYYKVGDMTQGLGWEA
PDC-374UGW95802.1_984PaBOC	241	PGPLDAEGYGVKTSAADLLRFVDANLHPERLDRPWAQALDATHRGYYKVGDMTQGLGWEA
PDC-374WGW26222.1_839PaBOC	241	PGPLDAEGYGVKTSAADLLRFVDANLHPERLDRPWAQALDATHRGYYKVGDMTQGLGWEA
PDC-374AOP56366.1_851PaOC	241	PGPLDAEGYGVKTSAADLLRFVDANLHPERLDRPWAQALDATHRGYYKVGDMTQGLGWEA
PDC-374AVE31445.1_873.PaOC	241	PGPLDAEGYGVKTSAADLLRFVDANLHPERLDRPWAQALDATHRGYYKVGDMTQGLGWEA
PDC-374QAS71510.1_873PaOC	241	PGPLDAEGYGVKTSAADLLRFVDANLHPERLDRPWAQALDATHRGYYKVGDMTQGLGWEA
PDC-374QDD38027.1_4463PaOC	241	PGPLDAEGYGVKTSAADLLRFVDANLHPERLDRPWAQALDATHRGYYKVGDMTQGLGWEA
PDC-374QKF05339.1_5830PaOC	241	PGPLDAEGYGVKTSAADLLRFVDANLHPERLDRPWAQALDATHRGYYKVGDMTQGLGWEA
PDC-374QKK83983.1_853PaOC	241	PGPLDAEGYGVKTSAADLLRFVDANLHPERLDRPWAQALDATHRGYYKVGDMTQGLGWEA
PDC-374QKS41466.1_4199PaOC	241	PGPLDAEGYGVKTSAADLLRFVDANLHPERLDRPWAQALDATHRGYYKVGDMTQGLGWEA
PDC-374QPZ80284.1_873PaOC	241	PGPLDAEGYGVKTSAADLLRFVDANLHPERLDRPWAQALDATHRGYYKVGDMTQGLGWEA
PDC-374QQW07466.1_922PaOC	241	PGPLDAEGYGVKTSAADLLRFVDANLHPERLDRPWAQALDATHRGYYKVGDMTQGLGWEA
PDC-374QZU93305.1_843PaOC	241	PGPLDAEGYGVKTSAADLLRFVDANLHPERLDRPWAQALDATHRGYYKVGDMTQGLGWEA
PDC-374UAC85653.1_934PaOC	241	PGPLDAEGYGVKTSAADLLRFVDANLHPERLDRPWAQALDATHRGYYKVGDMTQGLGWEA
PDC-374UGR20250.1_3485PaOC	241	PGPLDAEGYGVKTSAADLLRFVDANLHPERLDRPWAQALDATHRGYYKVGDMTQGLGWEA
PDC-374UIU12727.1_851PaOC	241	PGPLDAEGYGVKTSAADLLRFVDANLHPERLDRPWAQALDATHRGYYKVGDMTQGLGWEA
PDC-374UJC03215.1_956PaOC	241	PGPLDAEGYGVKTSAADLLRFVDANLHPERLDRPWAQALDATHRGYYKVGDMTQGLGWEA
PDC-374UJC09425.1_959PaOC	241	PGPLDAEGYGVKTSAADLLRFVDANLHPERLDRPWAQALDATHRGYYKVGDMTQGLGWEA
PDC-374UJF57976.1_829PaOC	241	PGPLDAEGYGVKTSAADLLRFVDANLHPERLDRPWAQALDATHRGYYKVGDMTQGLGWEA
PDC-374UJF63613.1_829PaOC	241	PGPLDAEGYGVKTSAADLLRFVDANLHPERLDRPWAQALDATHRGYYKVGDMTQGLGWEA
PDC-374UJF68041.1_5361PaOC	241	PGPLDAEGYGVKTSAADLLRFVDANLHPERLDRPWAQALDATHRGYYKVGDMTQGLGWEA
PDC-374UJF74874.1_829PaOC	241	PGPLDAEGYGVKTSAADLLRFVDANLHPERLDRPWAQALDATHRGYYKVGDMTQGLGWEA
PDC-374UJF80517.1_829PaOC	241	PGPLDAEGYGVKTSAADLLRFVDANLHPERLDRPWAQALDATHRGYYKVGDMTQGLGWEA
PDC-374UJF86151.1_829PaOC	241	PGPLDAEGYGVKTSAADLLRFVDANLHPERLDRPWAQALDATHRGYYKVGDMTQGLGWEA
PDC-374UJF90590.1_5359PaOC	241	PGPLDAEGYGVKTSAADLLRFVDANLHPERLDRPWAQALDATHRGYYKVGDMTQGLGWEA
PDC-374UJF97432.1_830PaOC	241	PGPLDAEGYGVKTSAADLLRFVDANLHPERLDRPWAQALDATHRGYYKVGDMTQGLGWEA
PDC-374UJG01877.1_5359PaOC	241	PGPLDAEGYGVKTSAADLLRFVDANLHPERLDRPWAQALDATHRGYYKVGDMTQGLGWEA
PDC-374UJG08704.1_829PaOC	241	PGPLDAEGYGVKTSAADLLRFVDANLHPERLDRPWAQALDATHRGYYKVGDMTQGLGWEA
PDC-374UJG14426.1_829PaOC	241	PGPLDAEGYGVKTSAADLLRFVDANLHPERLDRPWAQALDATHRGYYKVGDMTQGLGWEA
PDC-374UJG20129.1_829PaOC	241	PGPLDAEGYGVKTSAADLLRFVDANLHPERLDRPWAQALDATHRGYYKVGDMTQGLGWEA
PDC-374UJG25828.1_829PaOC	241	PGPLDAEGYGVKTSAADLLRFVDANLHPERLDRPWAQALDATHRGYYKVGDMTQGLGWEA
PDC-374UJG31693.1_884PaOC	241	PGPLDAEGYGVKTSAADLLRFVDANLHPERLDRPWAQALDATHRGYYKVGDMTQGLGWEA
PDC-374UJG36239.1_5358PaOC	241	PGPLDAEGYGVKTSAADLLRFVDANLHPERLDRPWAQALDATHRGYYKVGDMTQGLGWEA
PDC-374UGR51117.1_68PaOC	241	PGPLDAEGYGVKTSAADLLRFVDANLHPERLDRPWAQALDATHRGYYKVGDMTQGLGWEA
PDC-374UON75698.1_5570PaOC	241	PGPLDAEGYGVKTSAADLLRFVDANLHPERLDRPWAQALDATHRGYYKVGDMTQGLGWEA
PDC-374UPG04116.1_3174PaOC	241	PGPLDAEGYGVKTSAADLLRFVDANLHPERLDRPWAQALDATHRGYYKVGDMTQGLGWEA
PDC-374UPZ07602.1_2096PaOC	241	PGPLDAEGYGVKTSAADLLRFVDANLHPERLDRPWAQALDATHRGYYKVGDMTQGLGWEA
PDC-374URM55875.1_5937PaOC	241	PGPLDAEGYGVKTSAADLLRFVDANLHPERLDRPWAQALDATHRGYYKVGDMTQGLGWEA
PDC-374USV13808.1_3922PaOC	241	PGPLDAEGYGVKTSAADLLRFVDANLHPERLDRPWAQALDATHRGYYKVGDMTQGLGWEA
PDC-374USV19196.1_4087PaOC	241	PGPLDAEGYGVKTSAADLLRFVDANLHPERLDRPWAQALDATHRGYYKVGDMTQGLGWEA
PDC-374USX84542.1_851PaOC	241	PGPLDAEGYGVKTSAADLLRFVDANLHPERLDRPWAQALDATHRGYYKVGDMTQGLGWEA

PDC-374USX78872.1_851PaOC	241	PGPLDAEGYGVKTSAADLLRFVDANLHPERLDRPWAQALDATHRGYYKVGDMTQGLGWEA
PDC-374UUJ85605.1_1747PaOC	241	PGPLDAEGYGVKTSAADLLRFVDANLHPERLDRPWAQALDATHRGYYKVGDMTQGLGWEA
PDC-374UVS71982.1_4220PaOC	241	PGPLDAEGYGVKTSAADLLRFVDANLHPERLDRPWAQALDATHRGYYKVGDMTQGLGWEA
PDC-374UWI85046.1_4210PaOC	241	PGPLDAEGYGVKTSAADLLRFVDANLHPERLDRPWAQALDATHRGYYKVGDMTQGLGWEA
PDC-374UWI92968.1_851PaOC	241	PGPLDAEGYGVKTSAADLLRFVDANLHPERLDRPWAQALDATHRGYYKVGDMTQGLGWEA
PDC-374UXA22714.1_4203PaOC	241	PGPLDAEGYGVKTSAADLLRFVDANLHPERLDRPWAQALDATHRGYYKVGDMTQGLGWEA
PDC-374UXA30656.1_926PaOC	241	PGPLDAEGYGVKTSAADLLRFVDANLHPERLDRPWAQALDATHRGYYKVGDMTQGLGWEA
PDC-374UXA44988.1_926PaOC	241	PGPLDAEGYGVKTSAADLLRFVDANLHPERLDRPWAQALDATHRGYYKVGDMTQGLGWEA
PDC-374UXN39470.1_4222PaOC	241	PGPLDAEGYGVKTSAADLLRFVDANLHPERLDRPWAQALDATHRGYYKVGDMTQGLGWEA
PDC-374UZA87382.1_4980PaOC	241	PGPLDAEGYGVKTSAADLLRFVDANLHPERLDRPWAQALDATHRGYYKVGDMTQGLGWEA
PDC-374WBM66401.1_846PaOC	241	PGPLDAEGYGVKTSAADLLRFVDANLHPERLDRPWAQALDATHRGYYKVGDMTQGLGWEA
PDC-374WGJ75339.1_925PaOC	241	PGPLDAEGYGVKTSAADLLRFVDANLHPERLDRPWAQALDATHRGYYKVGDMTQGLGWEA
PDC-374WGW87865.1_837PaOC	241	PGPLDAEGYGVKTSAADLLRFVDANLHPERLDRPWAQALDATHRGYYKVGDMTQGLGWEA
PDC-103QES80695.1_910PaOC	241	PGPLDAEGYGVKTSAADLLRFVDANLHPERLDRPWAQALDATHRGYYKVGDMTQGLGWEA
PDC-103QPV53905.1_6224PaOC	241	PGPLDAEGYGVKTSAADLLRFVDANLHPERLDRPWAQALDATHRGYYKVGDMTQGLGWEA
PDC-35AVR81260.1_945.PaOC	241	PGPLDAEGYGVKTSAADLLRFVDANLHPERLDRPWAQALDATHRGYYKVGDMTQGLGWEA
PDC-35QJA35930.1_977PaOC	241	PGPLDAEGYGVKTSAADLLRFVDANLHPERLDRPWAQALDATHRGYYKVGDMTQGLGWEA
PDC-35AYZ46521.1_3210PaOC	241	PGPLDAEGYGVKTSAADLLRFVDANLHPERLDRPWAQALDATHRGYYKVGDMTQGLGWEA
PDC-35AYZ59004.1_3976PaOC	241	PGPLDAEGYGVKTSAADLLRFVDANLHPERLDRPWAQALDATHRGYYKVGDMTQGLGWEA
PDC-35QDR21260.1_3098PaOC	241	PGPLDAEGYGVKTSAADLLRFVDANLHPERLDRPWAQALDATHRGYYKVGDMTQGLGWEA
PDC-35QEK40749.1_862PaOC	241	PGPLDAEGYGVKTSAADLLRFVDANLHPERLDRPWAQALDATHRGYYKVGDMTQGLGWEA
PDC-35AUB00033.3_866PaOC	241	PGPLDAEGYGVKTSAADLLRFVDANLHPERLDRPWAQALDATHRGYYKVGDMTQGLGWEA
PDC-35QPP34775.1_1311PaOC	241	PGPLDAEGYGVKTSAADLLRFVDANLHPERLDRPWAQALDATHRGYYKVGDMTQGLGWEA
PDC-35QLF38648.1_891PaOC	241	PGPLDAEGYGVKTSAADLLRFVDANLHPERLDRPWAQALDATHRGYYKVGDMTQGLGWEA
PDC-35QLF14474.1_2055PaOC	241	PGPLDAEGYGVKTSAADLLRFVDANLHPERLDRPWAQALDATHRGYYKVGDMTQGLGWEA
PDC-35QQV60864.1_5728PaOC	241	PGPLDAEGYGVKTSAADLLRFVDANLHPERLDRPWAQALDATHRGYYKVGDMTQGLGWEA
PDC-35QQW01132.1_862PaOC	241	PGPLDAEGYGVKTSAADLLRFVDANLHPERLDRPWAQALDATHRGYYKVGDMTQGLGWEA
PDC-35QQW17992.1_5232PaOC	241	PGPLDAEGYGVKTSAADLLRFVDANLHPERLDRPWAQALDATHRGYYKVGDMTQGLGWEA
PDC-35QQW23330.1_862PaOC	241	PGPLDAEGYGVKTSAADLLRFVDANLHPERLDRPWAQALDATHRGYYKVGDMTQGLGWEA
PDC-35QZE42332.1_862PaOC	241	PGPLDAEGYGVKTSAADLLRFVDANLHPERLDRPWAQALDATHRGYYKVGDMTQGLGWEA
PDC-35UFM83790.1_826PaOC	241	PGPLDAEGYGVKTSAADLLRFVDANLHPERLDRPWAQALDATHRGYYKVGDMTQGLGWEA
PDC-35UJC15714.1_892PaOC	241	PGPLDAEGYGVKTSAADLLRFVDANLHPERLDRPWAQALDATHRGYYKVGDMTQGLGWEA
PDC-35UTR21121.1_931PaOC	241	PGPLDAEGYGVKTSAADLLRFVDANLHPERLDRPWAQALDATHRGYYKVGDMTQGLGWEA
PDC-35UUP09555.1_853PaOC	241	PGPLDAEGYGVKTSAADLLRFVDANLHPERLDRPWAQALDATHRGYYKVGDMTQGLGWEA
PDC-35UWG56199.1_885PaOC	241	PGPLDAEGYGVKTSAADLLRFVDANLHPERLDRPWAQALDATHRGYYKVGDMTQGLGWEA
PDC-43UNT30829.1_824PaOC	241	PGPLDAEGYGVKTSAADLLRFVDANLHPERLDRPWAQALDATHRGYYKVGDMTQGLGWEA
PDC-45ALZ89573.1_855.PaOC	241	PGPLDAEGYGVKTSAADLLRFVDANLHPERLDRPWAQALDATHRGYYKVGDMTQGLGWEA
PDC-45QKL11315.1_849PaOC	241	PGPLDAEGYGVKTSAADLLRFVDANLHPERLDRPWAQALDATHRGYYKVGDMTQGLGWEA
PDC-45QQV94504.1_850PaOC	241	PGPLDAEGYGVKTSAADLLRFVDANLHPERLDRPWAQALDATHRGYYKVGDMTQGLGWEA
PDC-162UJB98759.1_2776PaOC	241	PGPLDAEGYGVKTSAADLLRFVDANLHPERLDRTWAQALDATHRGYYKVGDMTQGLGWEA
PDC-123QYE90948.1_3103PaOC	241	PGPLDAEGYGVKTSAADLLRFVDANLHPERLDRPWAQALDATHRGYYKVGDMTQGLGWEA
PDC-5UXH60990.1_2046PaBCP	241	PGPLDAEGYGVKTSAADLLRFVDANLHPERLDRPWAQALDATHRGYYKVGDMTQGLGWEA
PDC-5WBA37364.1_905PaBCP	241	PGPLDAEGYGVKTSAADLLRFVDANLHPERLDRPWAQALDATHRGYYKVGDMTQGLGWEA
PDC-5WGJ68312.1_842PaBCP	241	PGPLDAEGYGVKTSAADLLRFVDANLHPERLDRPWAQALDATHRGYYKVGDMTQGLGWEA
PDC-5WGT14845.1_882PaBCP	241	PGPLDAEGYGVKTSAADLLRFVDANLHPERLDRPWAQALDATHRGYYKVGDMTQGLGWEA
PDC-5WGX69730.1_872PaBCP	241	PGPLDAEGYGVKTSAADLLRFVDANLHPERLDRPWAQALDATHRGYYKVGDMTQGLGWEA
PDC-5CAI9893238.1_8405]PaBCP	241	PGPLDAEGYGVKTSAADLLRFVDANLHPERLDRPWAQALDATHRGYYKVGDMTQGLGWEA
PDC-5CAI9905252.1_60335]PaBCP	241	PGPLDAEGYGVKTSAADLLRFVDANLHPERLDRPWAQALDATHRGYYKVGDMTQGLGWEA
PDC-5UXH60990.1_2046PaBOC	241	PGPLDAEGYGVKTSAADLLRFVDANLHPERLDRPWAQALDATHRGYYKVGDMTQGLGWEA
PDC-5WBA37364.1_905PaBOC	241	PGPLDAEGYGVKTSAADLLRFVDANLHPERLDRPWAQALDATHRGYYKVGDMTQGLGWEA
PDC-5WGJ68312.1_842PaBOC	241	PGPLDAEGYGVKTSAADLLRFVDANLHPERLDRPWAQALDATHRGYYKVGDMTQGLGWEA
PDC-5WGT14845.1_882PaBOC	241	PGPLDAEGYGVKTSAADLLRFVDANLHPERLDRPWAQALDATHRGYYKVGDMTQGLGWEA
PDC-5WGX69730.1_872PaBOC	241	PGPLDAEGYGVKTSAADLLRFVDANLHPERLDRPWAQALDATHRGYYKVGDMTQGLGWEA
PDC-5CAI9893238.1_8405]PaBOC	241	PGPLDAEGYGVKTSAADLLRFVDANLHPERLDRPWAQALDATHRGYYKVGDMTQGLGWEA
PDC-5CAI9905252.1_60335]PaBOC	241	PGPLDAEGYGVKTSAADLLRFVDANLHPERLDRPWAQALDATHRGYYKVGDMTQGLGWEA
PDC-5ALU48309.1_2211.PaOC	241	PGPLDAEGYGVKTSAADLLRFVDANLHPERLDRPWAQALDATHRGYYKVGDMTQGLGWEA
PDC-5AMX89317.1_4085.PaOC	241	PGPLDAEGYGVKTSAADLLRFVDANLHPERLDRPWAQALDATHRGYYKVGDMTQGLGWEA
PDC-5ATH01452.1_827PaOC	241	PGPLDAEGYGVKTSAADLLRFVDANLHPERLDRPWAQALDATHRGYYKVGDMTQGLGWEA
PDC-5UTN13111.1_830PaOC	241	PGPLDAEGYGVKTSAADLLRFVDANLHPERLDRPWAQALDATHRGYYKVGDMTQGLGWEA
PDC-5UUH88148.1_851PaOC	241	PGPLDAEGYGVKTSAADLLRFVDANLHPERLDRPWAQALDATHRGYYKVGDMTQGLGWEA
PDC-5UUH98459.1_806PaOC	241	PGPLDAEGYGVKTSAADLLRFVDANLHPERLDRPWAQALDATHRGYYKVGDMTQGLGWEA
PDC-5UVH93148.1_837PaOC	241	PGPLDAEGYGVKTSAADLLRFVDANLHPERLDRPWAQALDATHRGYYKVGDMTQGLGWEA
PDC-5UWU62523.1_171PaOC	241	PGPLDAEGYGVKTSAADLLRFVDANLHPERLDRPWAQALDATHRGYYKVGDMTQGLGWEA
PDC-5WDZ01346.1_910PaOC	241	PGPLDAEGYGVKTSAADLLRFVDANLHPERLDRPWAQALDATHRGYYKVGDMTQGLGWEA
PDC-5WEO42644.1_846PaOC	241	PGPLDAEGYGVKTSAADLLRFVDANLHPERLDRPWAQALDATHRGYYKVGDMTQGLGWEA
PDC-5WGW99708.1_849PaOC	241	PGPLDAEGYGVKTSAADLLRFVDANLHPERLDRPWAQALDATHRGYYKVGDMTQGLGWEA
PDC-5WJM42992.1_848PaOC	241	PGPLDAEGYGVKTSAADLLRFVDANLHPERLDRPWAQALDATHRGYYKVGDMTQGLGWEA
PDC-60QWY08308.1_854PaBCP	241	PGPLDAEGYGVKTSAADLLRFVDANLHPERLDRPWAQALDATHRGYYKVGDMTQGLGWEA
PDC-60QZV50082.1_838PaBCP	241	PGPLDAEGYGVKTSAADLLRFVDANLHPERLDRPWAQALDATHRGYYKVGDMTQGLGWEA
PDC-60QWY08308.1_854PaBOC	241	PGPLDAEGYGVKTSAADLLRFVDANLHPERLDRPWAQALDATHRGYYKVGDMTQGLGWEA
PDC-60QZV50082.1_838PaBOC	241	PGPLDAEGYGVKTSAADLLRFVDANLHPERLDRPWAQALDATHRGYYKVGDMTQGLGWEA
PDC-60QPZ60674.1_902PaOC	241	PGPLDAEGYGVKTSAADLLRFVDANLHPERLDRPWAQALDATHRGYYKVGDMTQGLGWEA
PDC-60WAJ92260.1_847PaOC	241	PGPLDAEGYGVKTSAADLLRFVDANLHPERLDRPWAQALDATHRGYYKVGDMTQGLGWEA
PDC-60WAJ98065.1_847PaOC	241	PGPLDAEGYGVKTSAADLLRFVDANLHPERLDRPWAQALDATHRGYYKVGDMTQGLGWEA
PDC-15UNL10022.1_837PaBCP	241	PGPLDAEGYGVKTSAADLLRFVDANLHPERLDRLWAQALDATHRGYYKVGDMTQGLGWEA
PDC-15UNL10022.1_837PaBOC	241	PGPLDAEGYGVKTSAADLLRFVDANLHPERLDRLWAQALDATHRGYYKVGDMTQGLGWEA
PDC-15AOT38867.1_3370PaOC	241	PGPLDAEGYGVKTSAADLLRFVDANLHPERLDRLWAQALDATHRGYYKVGDMTQGLGWEA
PDC-15ALZ27705.1_859PaOC	241	PGPLDAEGYGVKTSAADLLRFVDANLHPERLDRLWAQALDATHRGYYKVGDMTQGLGWEA
PDC-15AXC19915.1_1529PaOC	241	PGPLDAEGYGVKTSAADLLRFVDANLHPERLDRLWAQALDATHRGYYKVGDMTQGLGWEA
PDC-15UTP69334.1_895PaOC	241	PGPLDAEGYGVKTSAADLLRFVDANLHPERLDRLWAQALDATHRGYYKVGDMTQGLGWEA
PDC-15UNK91447.1_837PaOC	241	PGPLDAEGYGVKTSAADLLRFVDANLHPERLDRLWAQALDATHRGYYKVGDMTQGLGWEA
PDC-15UNL03855.1_837PaOC	241	PGPLDAEGYGVKTSAADLLRFVDANLHPERLDRLWAQALDATHRGYYKVGDMTQGLGWEA
PDC-28UYM61272.1_6539PaOC	241	PGPLDAEGYGVKTSAADLLRFVDANLHPERLDRPWAQALDATHRGYYKVGDMTQGLGWEA
PDC-22QPZ72804.1_848PaOC	241	PGPLDAEGYGVKTSAADLLRFVDANLHPERLDRPWAQALDATHRGYYKVGDMTQGLGWEA
PDC-22QQM08170.1_4816PaOC	241	PGPLDAEGYGVKTSAADLLRFVDANLHPERLDRPWAQALDATHRGYYKVGDMTQGLGWEA
PDC-97QKR35301.1_4456PaOC	241	PGPLDAEGYGVKTSAADLLRFVDANLHPERLDRPWAQALDATHRGYYKVGDMTQGLGWEA
PDC-51WGV68048.1_897PaOC	241	PGPLDAEGYGVKTSAADLLRFVDANLHPERLDRPWAQALDATHRGYYKVGDMTQGLGWEA
PDC-1HW06_09835_867PaOC	241	PGPLDAEGYGVKTSAADLLRFVDANLHPERLDRPWAQALDATHRGYYKVGDMTQGLGWEA
PDC-151QQV49515.1_805PaOC	241	PGPLDAEAYGLKSSAADLLRFVEANLHPERLEKPWAQALDATHRGYYKVGDMTQGLGWEA
consensus>70		pGPLDAegYGvKtSAADLLRFV#ANLHPERL#rpWAQALDATHRGYYKVGDMTQGLGWEA
acc		

PDC-374QKR78821.1_828PaOC — α11 α12 β11 β12 β13 TT β14 η8

```
PDC-374QKR78821.1_828PaOC      301 YDWPISLKRLQAGNSTPMALQPHRIARLPAPQALEGQRLLNKTGSTNGFGAYVAFVPGRD
PDC-331WCI44612.1_917PaOC      292 YDWPISLKRLQAGNSTPMALQPHRIARLPAPQALEGQRLLNKTGSTNGFGAYVAFVPGRD
PDC-8HV97_01835_992PaOC        301 YDWPISLKRLQAGNSTPMALQPHRIARLPAPQALEGQRLLNKTGSTNGFGAYVAFVPGRD
PDC-8CLH63_27535_5433PaOC      301 YDWPISLKRLQAGNSTPMALQPHRIARLPAPQALEGQRLLNKTGSTNGFGAYVAFVPGRD
PDC-31HW08_20870_899PaOC       301 YDWPISLKRLQAGNSTPMALQPHRIARLPAPQALEGQRLLNKTGSTNGFGAYVAFVPGRD
PDC-3CPZ93_04765_942PaBCP      301 YDWPISLKRLQAGNSTPMALQPHRIARLPAPQALEGQRLLNKTGSTNGFGAYVAFVPGRD
PDC-3CPZ93_04765_942PaBOC      301 YDWPISLKRLQAGNSTPMALQPHRIARLPAPQALEGQRLLNKTGSTNGFGAYVAFVPGRD
PDC-3CD796_04405_870PaOC       301 YDWPISLKRLQAGNSTPMALQPHRIARLPAPQALEGQRLLNKTGSTNGFGAYVAFVPGRD
PDC-1HV98_23795_933PaOC        301 YDWPISLKRLQAGNSTPMALQPHRIARLPAPQALEGQRLLNKTGSTNGFGAYVAFVPGRD
PDC-103HV95_30355_911PaOC      301 YDWPISLKRLQAGNSTPMALQPHRIARLPAPQALEGQRLLNKTGSTNGFGAYVAFVPGRD
PDC-5HW02_17620_927PaOC        301 YDWPISLKRLQAGNSTPMALQPHRIARLPAPQALEGQRLLNKTGSTNGFGAYVAFVPGRD
PDC-5HW01_11705_873PaOC        301 YDWPISLKRLQAGNSTPMALQPHRIARLPAPQALEGQRLLNKTGSTNGFGAYVAFVPGRD
PDC-5CD800_33195_6567PaOC      301 YDWPISLKRLQAGNSTPMALQPHRIARLPAPQALEGQRLLNKTGSTNGFGAYVAFVPGRD
PDC-5CD799_31455_6219PaOC      301 YDWPISLKRLQAGNSTPMALQPHRIARLPAPQALEGQRLLNKTGSTNGFGAYVAFVPGRD
PDC-46CEK59_30555_6032PaOC     301 YDWPISLKRLQAGNSTPMALQPHRIARLPAPQALEGQRLLNKTGSTNGFGAYVAFVPGRD
PDC-66QTQ98808.1_855PaOC       301 YDWPISLKRLQAGNSTPMALQPHRIARLPAPQALEGQRLLNKTGSTNGFGAYVAFIPGRD
PDC-364QYE79191.1_1969PaOC     301 YDWPISLKRLQAGNSTPMALQPHRIARLPAPQALEGQRLLNKTGSTNGFGAYVAFIPGRD
PDC-122QYN07359.1_5685PaOC     301 YDWPISLKRMQAGNSTPMALQPHRIARLPAPQALEGQRLLNKTGSTNGFGAYVAFVPGRD
PDC-100QKR60216.1_898PaOC      301 YDWPITLKRLQAGNSTPMALQPHRIARLPAPQALEGQRLLNKTGSTNGFGAYVAFVPGRD
PDC-34WBM27707.1_865PaBCP      301 YDWPISLKRLQAGNSTPMALQPHRIARLPAPQALEGQRLLNKTGSTNGFGAYVAFVPGRD
PDC-34WGX11792.1_865PaBCP      301 YDWPISLKRLQAGNSTPMALQPHRIARLPAPQALEGQRLLNKTGSTNGFGAYVAFVPGRD
PDC-34WBM27707.1_865PaBOC      301 YDWPISLKRLQAGNSTPMALQPHRIARLPAPQALEGQRLLNKTGSTNGFGAYVAFVPGRD
PDC-34WGX11792.1_865PaBOC      301 YDWPISLKRLQAGNSTPMALQPHRIARLPAPQALEGQRLLNKTGSTNGFGAYVAFVPGRD
PDC-34ALY72428.1_939PaOC       301 YDWPISLKRLQAGNSTPMALQPHRIARLPAPQALEGQRLLNKTGSTNGFGAYVAFVPGRD
PDC-34ASM83699.1_885PaOC       301 YDWPISLKRLQAGNSTPMALQPHRIARLPAPQALEGQRLLNKTGSTNGFGAYVAFVPGRD
PDC-34AYW64445.1_883PaOC       301 YDWPISLKRLQAGNSTPMALQPHRIARLPAPQALEGQRLLNKTGSTNGFGAYVAFVPGRD
PDC-34UTR27428.1_860PaOC       301 YDWPISLKRLQAGNSTPMALQPHRIARLPAPQALEGQRLLNKTGSTNGFGAYVAFVPGRD
PDC-34UOD92021.1_934PaOC       301 YDWPISLKRLQAGNSTPMALQPHRIARLPAPQALEGQRLLNKTGSTNGFGAYVAFVPGRD
PDC-34WIV39806.1_860PaOC       301 YDWPISLKRLQAGNSTPMALQPHRIARLPAPQALEGQRLLNKTGSTNGFGAYVAFVPGRD
PDC-264QKR42793.1_833PaOC      301 YDWPISLKRLQAGNSTPMALQPHRIARLPAPQALEGQRLLNKTGSTNGFGAYVAFVPGRD
PDC-167QIZ28250.1_4314PaOC     301 YDWPIALKRLQAGNSTPMALQPHRVARLPAPQALEGQRLLNKTGSTNGFGAYVAFIPGRD
PDC-167UTQ27575.1_842PaOC      301 YDWPIALKRLQAGNSTPMALQPHRVARLPAPQALEGQRLLNKTGSTNGFGAYVAFIPGRD
PDC-6QZV30905.1_832PaBCP       301 YDWPISLKRLQAGNSTPMALQPHRIARLPAPQALEGQRLLNKTGSTNGFGAYVAFVPGRD
PDC-6QZV30905.1_832PaBOC       301 YDWPISLKRLQAGNSTPMALQPHRIARLPAPQALEGQRLLNKTGSTNGFGAYVAFVPGRD
PDC-37WAJ85675.1_843PaBCP      301 YDWPISLKRLQAGNSTPMALQPHRIARLPAPQALEGQRLLNKTGSTNGFGAYVAFIPGRD
PDC-37WGX56158.1_855PaBCP      301 YDWPISLKRLQAGNSTPMALQPHRIARLPAPQALEGQRLLNKTGSTNGFGAYVAFIPGRD
PDC-37WAJ85675.1_843PaBOC      301 YDWPISLKRLQAGNSTPMALQPHRIARLPAPQALEGQRLLNKTGSTNGFGAYVAFIPGRD
PDC-37WGX56158.1_855PaBOC      301 YDWPISLKRLQAGNSTPMALQPHRIARLPAPQALEGQRLLNKTGSTNGFGAYVAFIPGRD
PDC-37ANA71197.1_2757.PaOC     301 YDWPISLKRLQAGNSTPMALQPHRIARLPAPQALEGQRLLNKTGSTNGFGAYVAFIPGRD
PDC-37WGX14507.1_3195PaOC      301 YDWPISLKRLQAGNSTPMALQPHRIARLPAPQALEGQRLLNKTGSTNGFGAYVAFIPGRD
PDC-46AUA93832.1_897PaOC       301 YDWPISLKRLQAGNSTPMALQPHRIARLPAPQALEGQRLLNKTGSTNGFGAYVAFVPGRD
PDC-105QGP95004.1_877PaBCP     301 YDWPISLKRLQAGNSTPMALQPHRIARLPAPQALEGQRLLNKTGSTNGFGAYVAFIPGRD
PDC-105QGP95004.1_877PaBOC     301 YDWPISLKRLQAGNSTPMALQPHRIARLPAPQALEGQRLLNKTGSTNGFGAYVAFIPGRD
PDC-12AVN43882.1_2061.PaOC     301 YDWPISLKRLQAGNSTPMALQPHRIARLPAPQALEGQRLLNKTGSTNGFGAYVAFVPGRD
PDC-12QNE63041.1_859PaOC       301 YDWPISLKRLQAGNSTPMALQPHRIARLPAPQALEGQRLLNKTGSTNGFGAYVAFVPGRD
PDC-12WGW93864.1_852PaOC       301 YDWPISLKRLQAGNSTPMALQPHRIARLPAPQALEGQRLLNKTGSTNGFGAYVAFVPGRD
PDC-12WGX05587.1_852PaOC       301 YDWPISLKRLQAGNSTPMALQPHRIARLPAPQALEGQRLLNKTGSTNGFGAYVAFVPGRD
PDC-11QSA85555.1_5581PaBCP     301 YDWPISLKRLQAGNSTPMALQPHRIARLPAPQALEGQRLLNKTGSTNGFGAYVAFVPGRD
PDC-11QSA91467.1_5367PaBCP     301 YDWPISLKRLQAGNSTPMALQPHRIARLPAPQALEGQRLLNKTGSTNGFGAYVAFVPGRD
PDC-11QSA85555.1_5581PaBOC     301 YDWPISLKRLQAGNSTPMALQPHRIARLPAPQALEGQRLLNKTGSTNGFGAYVAFVPGRD
PDC-11QSA91467.1_5367PaBOC     301 YDWPISLKRLQAGNSTPMALQPHRIARLPAPQALEGQRLLNKTGSTNGFGAYVAFVPGRD
PDC-11AYQ83203.1_3491PaOC      301 YDWPISLKRLQAGNSTPMALQPHRIARLPAPQALEGQRLLNKTGSTNGFGAYVAFVPGRD
PDC-11AYR15208.1_5205PaOC      301 YDWPISLKRLQAGNSTPMALQPHRIARLPAPQALEGQRLLNKTGSTNGFGAYVAFVPGRD
PDC-11AZN02324.1_4787PaOC      301 YDWPISLKRLQAGNSTPMALQPHRIARLPAPQALEGQRLLNKTGSTNGFGAYVAFVPGRD
PDC-11AZN04123.1_203PaOC       301 YDWPISLKRLQAGNSTPMALQPHRIARLPAPQALEGQRLLNKTGSTNGFGAYVAFVPGRD
PDC-11AZN17071.1_5847PaOC      301 YDWPISLKRLQAGNSTPMALQPHRIARLPAPQALEGQRLLNKTGSTNGFGAYVAFVPGRD
PDC-11AZN48510.1_4981PaOC      301 YDWPISLKRLQAGNSTPMALQPHRIARLPAPQALEGQRLLNKTGSTNGFGAYVAFVPGRD
PDC-11AZN60564.1_5101PaOC      301 YDWPISLKRLQAGNSTPMALQPHRIARLPAPQALEGQRLLNKTGSTNGFGAYVAFVPGRD
PDC-11AZN55779.1_6545PaOC      301 YDWPISLKRLQAGNSTPMALQPHRIARLPAPQALEGQRLLNKTGSTNGFGAYVAFVPGRD
PDC-11AYF68870.1_744PaOC       301 YDWPISLKRLQAGNSTPMALQPHRIARLPAPQALEGQRLLNKTGSTNGFGAYVAFVPGRD
PDC-11QUS21307.1_2433PaOC      301 YDWPISLKRLQAGNSTPMALQPHRIARLPAPQALEGQRLLNKTGSTNGFGAYVAFVPGRD
PDC-11UVH87183.1_982PaOC       301 YDWPISLKRLQAGNSTPMALQPHRIARLPAPQALEGQRLLNKTGSTNGFGAYVAFVPGRD
PDC-16QCC64126.1_958PaBCP      301 YDWPISLKRLQAGNSTPMALQPHRIARLPAPQALEGQRLLNKTGSTNGFGAYVAFVPGRD
PDC-16UBI70916.1_933PaBCP      301 YDWPISLKRLQAGNSTPMALQPHRIARLPAPQALEGQRLLNKTGSTNGFGAYVAFVPGRD
PDC-16UTQ84596.1_855PaBCP      301 YDWPISLKRLQAGNSTPMALQPHRIARLPAPQALEGQRLLNKTGSTNGFGAYVAFVPGRD
PDC-16QCC64126.1_958PaBOC      301 YDWPISLKRLQAGNSTPMALQPHRIARLPAPQALEGQRLLNKTGSTNGFGAYVAFVPGRD
PDC-16UBI70916.1_933PaBOC      301 YDWPISLKRLQAGNSTPMALQPHRIARLPAPQALEGQRLLNKTGSTNGFGAYVAFVPGRD
PDC-16UTQ84596.1_855PaBOC      301 YDWPISLKRLQAGNSTPMALQPHRIARLPAPQALEGQRLLNKTGSTNGFGAYVAFVPGRD
PDC-16ALS12949.1_2977.PaOC     301 YDWPISLKRLQAGNSTPMALQPHRIARLPAPQALEGQRLLNKTGSTNGFGAYVAFVPGRD
PDC-16AYW58201.1_855PaOC       301 YDWPISLKRLQAGNSTPMALQPHRIARLPAPQALEGQRLLNKTGSTNGFGAYVAFVPGRD
PDC-16QDR00398.1_890PaOC       301 YDWPISLKRLQAGNSTPMALQPHRIARLPAPQALEGQRLLNKTGSTNGFGAYVAFVPGRD
PDC-16QGJ31628.1_895PaOC       301 YDWPISLKRLQAGNSTPMALQPHRIARLPAPQALEGQRLLNKTGSTNGFGAYVAFVPGRD
PDC-16QKG34934.1_1840PaOC      301 YDWPISLKRLQAGNSTPMALQPHRIARLPAPQALEGQRLLNKTGSTNGFGAYVAFVPGRD
PDC-16QOQ64874.1_122PaOC       301 YDWPISLKRLQAGNSTPMALQPHRIARLPAPQALEGQRLLNKTGSTNGFGAYVAFVPGRD
PDC-16QVQ73157.1_860PaOC       301 YDWPISLKRLQAGNSTPMALQPHRIARLPAPQALEGQRLLNKTGSTNGFGAYVAFVPGRD
PDC-16UOC64480.1_827PaOC       301 YDWPISLKRLQAGNSTPMALQPHRIARLPAPQALEGQRLLNKTGSTNGFGAYVAFVPGRD
PDC-16UOC70564.1_826PaOC       301 YDWPISLKRLQAGNSTPMALQPHRIARLPAPQALEGQRLLNKTGSTNGFGAYVAFVPGRD
PDC-16UOC76649.1_827PaOC       301 YDWPISLKRLQAGNSTPMALQPHRIARLPAPQALEGQRLLNKTGSTNGFGAYVAFVPGRD
PDC-16UOC82740.1_827PaOC       301 YDWPISLKRLQAGNSTPMALQPHRIARLPAPQALEGQRLLNKTGSTNGFGAYVAFVPGRD
PDC-16UOC88825.1_827PaOC       301 YDWPISLKRLQAGNSTPMALQPHRIARLPAPQALEGQRLLNKTGSTNGFGAYVAFVPGRD
PDC-16UOE68701.1_827PaOC       301 YDWPISLKRLQAGNSTPMALQPHRIARLPAPQALEGQRLLNKTGSTNGFGAYVAFVPGRD
PDC-16WDC65088.1_844PaOC       301 YDWPISLKRLQAGNSTPMALQPHRIARLPAPQALEGQRLLNKTGSTNGFGAYVAFVPGRD
PDC-16WGW70130.1_845PaOC       301 YDWPISLKRLQAGNSTPMALQPHRIARLPAPQALEGQRLLNKTGSTNGFGAYVAFVPGRD
PDC-16WGW76010.1_845PaOC       301 YDWPISLKRLQAGNSTPMALQPHRIARLPAPQALEGQRLLNKTGSTNGFGAYVAFVPGRD
PDC-16WGW81892.1_845PaOC       301 YDWPISLKRLQAGNSTPMALQPHRIARLPAPQALEGQRLLNKTGSTNGFGAYVAFVPGRD
PDC-16WGX43443.1_806PaOC       301 YDWPISLKRLQAGNSTPMALQPHRIARLPAPQALEGQRLLNKTGSTNGFGAYVAFVPGRD
PDC-16WJM37210.1_871PaOC       301 YDWPISLKRLQAGNSTPMALQPHRIARLPAPQALEGQRLLNKTGSTNGFGAYVAFVPGRD
PDC-59UQZ18314.1_902PaOC       301 YDWPITLKRLQAGNSTPMALQPHRIARLPAPQALEGQRLLNKTGSTNGFGAYVAFVPGRD
PDC-142WGW57525.1_830PaBCP     301 YDWPISLKRLQAGNSTPMALQPHRIARLPAPQALEGQRLLNKTGSTNGFGAYVAFVPGRD
PDC-142WGW32210.1_830PaBCP     301 YDWPISLKRLQAGNSTPMALQPHRIARLPAPQALEGQRLLNKTGSTNGFGAYVAFVPGRD
PDC-142WGW44776.1_830PaBCP     301 YDWPISLKRLQAGNSTPMALQPHRIARLPAPQALEGQRLLNKTGSTNGFGAYVAFVPGRD
PDC-142WGW57525.1_830PaBOC     301 YDWPISLKRLQAGNSTPMALQPHRIARLPAPQALEGQRLLNKTGSTNGFGAYVAFVPGRD
PDC-142WGW32210.1_830PaBOC     301 YDWPISLKRLQAGNSTPMALQPHRIARLPAPQALEGQRLLNKTGSTNGFGAYVAFVPGRD
PDC-142WGW44776.1_830PaBOC     301 YDWPISLKRLQAGNSTPMALQPHRIARLPAPQALEGQRLLNKTGSTNGFGAYVAFVPGRD
PDC-142AON05138.1_825PaOC      301 YDWPISLKRLQAGNSTPMALQPHRIARLPAPQALEGQRLLNKTGSTNGFGAYVAFVPGRD
PDC-142AON11127.1_825PaOC      301 YDWPISLKRLQAGNSTPMALQPHRIARLPAPQALEGQRLLNKTGSTNGFGAYVAFVPGRD
PDC-142AON23122.1_825PaOC      301 YDWPISLKRLQAGNSTPMALQPHRIARLPAPQALEGQRLLNKTGSTNGFGAYVAFVPGRD
PDC-142AON29110.1_825PaOC      301 YDWPISLKRLQAGNSTPMALQPHRIARLPAPQALEGQRLLNKTGSTNGFGAYVAFVPGRD
PDC-142AON41099.1_825PaOC      301 YDWPISLKRLQAGNSTPMALQPHRIARLPAPQALEGQRLLNKTGSTNGFGAYVAFVPGRD
PDC-142AON47112.1_824PaOC      301 YDWPISLKRLQAGNSTPMALQPHRIARLPAPQALEGQRLLNKTGSTNGFGAYVAFVPGRD
PDC-142AON17115.1_824PaOC      301 YDWPISLKRLQAGNSTPMALQPHRIARLPAPQALEGQRLLNKTGSTNGFGAYVAFVPGRD
PDC-142AON35116.1_825PaOC      301 YDWPISLKRLQAGNSTPMALQPHRIARLPAPQALEGQRLLNKTGSTNGFGAYVAFVPGRD
PDC-19aWGW51434.1_1207PaBCP    301 YDWPISLKRLQAGNSTPMALQPHRIARLPAPQALEGQRLLNKTGSTNGFGAYVAFIPGRD
```

```
PDC-19aWGW37666.1_6817PaBCP     301  YDWPISLKRLQAGNSTPMALQPHRIARLPAPQALEGQRLLNKTGSTNGFGAYVAFIPGRD
PDC-19aWGW59864.1_3660PaBCP     301  YDWPISLKRLQAGNSTPMALQPHRIARLPAPQALEGQRLLNKTGSTNGFGAYVAFIPGRD
PDC-19aWGX49838.1_877PaBCP      301  YDWPISLKRLQAGNSTPMALQPHRIARLPAPQALEGQRLLNKTGSTNGFGAYVAFIPGRD
PDC-19aWGX24308.1_877PaBCP      301  YDWPISLKRLQAGNSTPMALQPHRIARLPAPQALEGQRLLNKTGSTNGFGAYVAFIPGRD
PDC-19aWGX35552.1_5487PaBCP     301  YDWPISLKRLQAGNSTPMALQPHRIARLPAPQALEGQRLLNKTGSTNGFGAYVAFIPGRD
PDC-19aWKA37694.1_2446PaBCP     301  YDWPISLKRLQAGNSTPMALQPHRIARLPAPQALEGQRLLNKTGSTNGFGAYVAFIPGRD
PDC-19aWGW51434.1_1207PaBOC     301  YDWPISLKRLQAGNSTPMALQPHRIARLPAPQALEGQRLLNKTGSTNGFGAYVAFIPGRD
PDC-19aWGW37666.1_6817PaBOC     301  YDWPISLKRLQAGNSTPMALQPHRIARLPAPQALEGQRLLNKTGSTNGFGAYVAFIPGRD
PDC-19aWGW59864.1_3660PaBOC     301  YDWPISLKRLQAGNSTPMALQPHRIARLPAPQALEGQRLLNKTGSTNGFGAYVAFIPGRD
PDC-19aWGX49838.1_877PaBOC      301  YDWPISLKRLQAGNSTPMALQPHRIARLPAPQALEGQRLLNKTGSTNGFGAYVAFIPGRD
PDC-19aWGX24308.1_877PaBOC      301  YDWPISLKRLQAGNSTPMALQPHRIARLPAPQALEGQRLLNKTGSTNGFGAYVAFIPGRD
PDC-19aWGX35552.1_5487PaBOC     301  YDWPISLKRLQAGNSTPMALQPHRIARLPAPQALEGQRLLNKTGSTNGFGAYVAFIPGRD
PDC-19aWKA37694.1_2446PaBOC     301  YDWPISLKRLQAGNSTPMALQPHRIARLPAPQALEGQRLLNKTGSTNGFGAYVAFIPGRD
PDC-19aAPB63370.1_616PaOC       301  YDWPISLKRLQAGNSTPMALQPHRIARLPAPQALEGQRLLNKTGSTNGFGAYVAFIPGRD
PDC-19aARI89529.1_870PaOC       301  YDWPISLKRLQAGNSTPMALQPHRIARLPAPQALEGQRLLNKTGSTNGFGAYVAFIPGRD
PDC-19aARI95964.1_871PaOC       301  YDWPISLKRLQAGNSTPMALQPHRIARLPAPQALEGQRLLNKTGSTNGFGAYVAFIPGRD
PDC-19aASA13594.1_977PaOC       301  YDWPISLKRLQAGNSTPMALQPHRIARLPAPQALEGQRLLNKTGSTNGFGAYVAFIPGRD
PDC-19aASA27558.1_962PaOC       301  YDWPISLKRLQAGNSTPMALQPHRIARLPAPQALEGQRLLNKTGSTNGFGAYVAFIPGRD
PDC-19aASD01901.1_971PaOC       301  YDWPISLKRLQAGNSTPMALQPHRIARLPAPQALEGQRLLNKTGSTNGFGAYVAFIPGRD
PDC-19aUAC92160.1_908PaOC       301  YDWPISLKRLQAGNSTPMALQPHRIARLPAPQALEGQRLLNKTGSTNGFGAYVAFIPGRD
PDC-19aUAC98839.1_942PaOC       301  YDWPISLKRLQAGNSTPMALQPHRIARLPAPQALEGQRLLNKTGSTNGFGAYVAFIPGRD
PDC-19aUTQ33604.1_830PaOC       301  YDWPISLKRLQAGNSTPMALQPHRIARLPAPQALEGQRLLNKTGSTNGFGAYVAFIPGRD
PDC-19aUOO58611.1_830PaOC       301  YDWPISLKRLQAGNSTPMALQPHRIARLPAPQALEGQRLLNKTGSTNGFGAYVAFIPGRD
PDC-19aWDA50910.1_883PaOC       301  YDWPISLKRLQAGNSTPMALQPHRIARLPAPQALEGQRLLNKTGSTNGFGAYVAFIPGRD
PDC-19aWGX30960.1_941PaOC       301  YDWPISLKRLQAGNSTPMALQPHRIARLPAPQALEGQRLLNKTGSTNGFGAYVAFIPGRD
PDC-19aWGX61448.1_5603PaOC      301  YDWPISLKRLQAGNSTPMALQPHRIARLPAPQALEGQRLLNKTGSTNGFGAYVAFIPGRD
PDC-19aWGX82118.1_941PaOC       301  YDWPISLKRLQAGNSTPMALQPHRIARLPAPQALEGQRLLNKTGSTNGFGAYVAFIPGRD
PDC-19aWIO49783.1_187PaOC       301  YDWPISLKRLQAGNSTPMALQPHRIARLPAPQALEGQRLLNKTGSTNGFGAYVAFIPGRD
PDC-24QZV43725.1_941PaBCP       301  YDWPISLKRLQAGNSTPMALQPHRIARLPAPQALEGQRLLNKTGSTNGFGAYVAFVPGRD
PDC-24QZV43725.1_941PaBOC       301  YDWPISLKRLQAGNSTPMALQPHRIARLPAPQALEGQRLLNKTGSTNGFGAYVAFVPGRD
PDC-24QDR06751.1_860PaOC        301  YDWPISLKRLQAGNSTPMALQPHRIARLPAPQALEGQRLLNKTGSTNGFGAYVAFVPGRD
PDC-24UGR36796.1_3412PaOC       301  YDWPISLKRLQAGNSTPMALQPHRIARLPAPQALEGQRLLNKTGSTNGFGAYVAFVPGRD
PDC-24UTQ39921.1_833PaOC        301  YDWPISLKRLQAGNSTPMALQPHRIARLPAPQALEGQRLLNKTGSTNGFGAYVAFVPGRD
PDC-137QKE94864.1_852PaOC      301  YDWPISLKRLQAGNSTPMALQPHRIARLPAPQALEGQRLLNKTGSTNGFGAYVAFVPGRD
PDC-147UGR30133.1_1950PaOC     301  YDWPISLKRLQAGNSTPMALQPHRIARLPAPQALEGQRLLNKTGSTNGFGAYVAFVPGRD
PDC-8AYY41152.1_5352PaBCP      301  YDWPISLKRLQAGNSTPMALQPHRIARLPAPQALEGQRLLNKTGSTNGFGAYVAFVPGRD
PDC-8QOE14337.1_211PaBCP       301  YDWPISLKRLQAGNSTPMALQPHRIARLPAPQALEGQRLLNKTGSTNGFGAYVAFVPGRD
PDC-8QPN15577.1_911PaBCP       301  YDWPISLKRLQAGNSTPMALQPHRIARLPAPQALEGQRLLNKTGSTNGFGAYVAFVPGRD
PDC-8QPN23993.1_908PaBCP       301  YDWPISLKRLQAGNSTPMALQPHRIARLPAPQALEGQRLLNKTGSTNGFGAYVAFVPGRD
PDC-8QUE64702.1_909PaBCP       301  YDWPISLKRLQAGNSTPMALQPHRIARLPAPQALEGQRLLNKTGSTNGFGAYVAFVPGRD
PDC-8QYA88773.1_2249PaBCP      301  YDWPISLKRLQAGNSTPMALQPHRIARLPAPQALEGQRLLNKTGSTNGFGAYVAFVPGRD
PDC-8QYF01720.1_5249PaBCP      301  YDWPISLKRLQAGNSTPMALQPHRIARLPAPQALEGQRLLNKTGSTNGFGAYVAFVPGRD
PDC-8QZU99504.1_908PaBCP       301  YDWPISLKRLQAGNSTPMALQPHRIARLPAPQALEGQRLLNKTGSTNGFGAYVAFVPGRD
PDC-8QZV05727.1_908PaBCP       301  YDWPISLKRLQAGNSTPMALQPHRIARLPAPQALEGQRLLNKTGSTNGFGAYVAFVPGRD
PDC-8QZV12028.1_910PaBCP       301  YDWPISLKRLQAGNSTPMALQPHRIARLPAPQALEGQRLLNKTGSTNGFGAYVAFVPGRD
PDC-8QZV18112.1_863PaBCP       301  YDWPISLKRLQAGNSTPMALQPHRIARLPAPQALEGQRLLNKTGSTNGFGAYVAFVPGRD
PDC-8QZV24387.1_912PaBCP       301  YDWPISLKRLQAGNSTPMALQPHRIARLPAPQALEGQRLLNKTGSTNGFGAYVAFVPGRD
PDC-8QZV68851.1_908PaBCP       301  YDWPISLKRLQAGNSTPMALQPHRIARLPAPQALEGQRLLNKTGSTNGFGAYVAFVPGRD
PDC-8QZV81338.1_870PaBCP       301  YDWPISLKRLQAGNSTPMALQPHRIARLPAPQALEGQRLLNKTGSTNGFGAYVAFVPGRD
PDC-8QZV87769.1_908PaBCP       301  YDWPISLKRLQAGNSTPMALQPHRIARLPAPQALEGQRLLNKTGSTNGFGAYVAFVPGRD
PDC-8QZV94048.1_908PaBCP       301  YDWPISLKRLQAGNSTPMALQPHRIARLPAPQALEGQRLLNKTGSTNGFGAYVAFVPGRD
PDC-8UXH53497.1_877PaBCP       301  YDWPISLKRLQAGNSTPMALQPHRIARLPAPQALEGQRLLNKTGSTNGFGAYVAFVPGRD
PDC-8UXH66521.1_877PaBCP       301  YDWPISLKRLQAGNSTPMALQPHRIARLPAPQALEGQRLLNKTGSTNGFGAYVAFVPGRD
PDC-8UXH70664.1_4987PaBCP      301  YDWPISLKRLQAGNSTPMALQPHRIARLPAPQALEGQRLLNKTGSTNGFGAYVAFVPGRD
PDC-8UUW81479.1_5273PaBCP      301  YDWPISLKRLQAGNSTPMALQPHRIARLPAPQALEGQRLLNKTGSTNGFGAYVAFVPGRD
PDC-8WDM07130.1_910PaBCP       301  YDWPISLKRLQAGNSTPMALQPHRIARLPAPQALEGQRLLNKTGSTNGFGAYVAFVPGRD
PDC-8WHM62936.1_909PaBCP       301  YDWPISLKRLQAGNSTPMALQPHRIARLPAPQALEGQRLLNKTGSTNGFGAYVAFVPGRD
PDC-8WHM69354.1_909PaBCP       301  YDWPISLKRLQAGNSTPMALQPHRIARLPAPQALEGQRLLNKTGSTNGFGAYVAFVPGRD
PDC-8WHM75768.1_909PaBCP       301  YDWPISLKRLQAGNSTPMALQPHRIARLPAPQALEGQRLLNKTGSTNGFGAYVAFVPGRD
PDC-8WHM84263.1_909PaBCP       301  YDWPISLKRLQAGNSTPMALQPHRIARLPAPQALEGQRLLNKTGSTNGFGAYVAFVPGRD
PDC-8AYY41152.1_5352PaBOC      301  YDWPISLKRLQAGNSTPMALQPHRIARLPAPQALEGQRLLNKTGSTNGFGAYVAFVPGRD
PDC-8QOE14337.1_211PaBOC       301  YDWPISLKRLQAGNSTPMALQPHRIARLPAPQALEGQRLLNKTGSTNGFGAYVAFVPGRD
PDC-8QPN15577.1_911PaBOC       301  YDWPISLKRLQAGNSTPMALQPHRIARLPAPQALEGQRLLNKTGSTNGFGAYVAFVPGRD
PDC-8QPN23993.1_908PaBOC       301  YDWPISLKRLQAGNSTPMALQPHRIARLPAPQALEGQRLLNKTGSTNGFGAYVAFVPGRD
PDC-8QUE64702.1_909PaBOC       301  YDWPISLKRLQAGNSTPMALQPHRIARLPAPQALEGQRLLNKTGSTNGFGAYVAFVPGRD
PDC-8QYA88773.1_2249PaBOC      301  YDWPISLKRLQAGNSTPMALQPHRIARLPAPQALEGQRLLNKTGSTNGFGAYVAFVPGRD
PDC-8QYF01720.1_5249PaBOC      301  YDWPISLKRLQAGNSTPMALQPHRIARLPAPQALEGQRLLNKTGSTNGFGAYVAFVPGRD
PDC-8QZU99504.1_908PaBOC       301  YDWPISLKRLQAGNSTPMALQPHRIARLPAPQALEGQRLLNKTGSTNGFGAYVAFVPGRD
PDC-8QZV05727.1_908PaBOC       301  YDWPISLKRLQAGNSTPMALQPHRIARLPAPQALEGQRLLNKTGSTNGFGAYVAFVPGRD
PDC-8QZV12028.1_910PaBOC       301  YDWPISLKRLQAGNSTPMALQPHRIARLPAPQALEGQRLLNKTGSTNGFGAYVAFVPGRD
PDC-8QZV18112.1_863PaBOC       301  YDWPISLKRLQAGNSTPMALQPHRIARLPAPQALEGQRLLNKTGSTNGFGAYVAFVPGRD
PDC-8QZV24387.1_912PaBOC       301  YDWPISLKRLQAGNSTPMALQPHRIARLPAPQALEGQRLLNKTGSTNGFGAYVAFVPGRD
PDC-8QZV68851.1_908PaBOC       301  YDWPISLKRLQAGNSTPMALQPHRIARLPAPQALEGQRLLNKTGSTNGFGAYVAFVPGRD
PDC-8QZV81338.1_870PaBOC       301  YDWPISLKRLQAGNSTPMALQPHRIARLPAPQALEGQRLLNKTGSTNGFGAYVAFVPGRD
PDC-8QZV87769.1_908PaBOC       301  YDWPISLKRLQAGNSTPMALQPHRIARLPAPQALEGQRLLNKTGSTNGFGAYVAFVPGRD
PDC-8QZV94048.1_908PaBOC       301  YDWPISLKRLQAGNSTPMALQPHRIARLPAPQALEGQRLLNKTGSTNGFGAYVAFVPGRD
PDC-8UXH53497.1_877PaBOC       301  YDWPISLKRLQAGNSTPMALQPHRIARLPAPQALEGQRLLNKTGSTNGFGAYVAFVPGRD
PDC-8UXH66521.1_877PaBOC       301  YDWPISLKRLQAGNSTPMALQPHRIARLPAPQALEGQRLLNKTGSTNGFGAYVAFVPGRD
PDC-8UXH70664.1_4987PaBOC      301  YDWPISLKRLQAGNSTPMALQPHRIARLPAPQALEGQRLLNKTGSTNGFGAYVAFVPGRD
PDC-8UUW81479.1_5273PaBOC      301  YDWPISLKRLQAGNSTPMALQPHRIARLPAPQALEGQRLLNKTGSTNGFGAYVAFVPGRD
PDC-8WDM07130.1_910PaBOC       301  YDWPISLKRLQAGNSTPMALQPHRIARLPAPQALEGQRLLNKTGSTNGFGAYVAFVPGRD
PDC-8WHM62936.1_909PaBOC       301  YDWPISLKRLQAGNSTPMALQPHRIARLPAPQALEGQRLLNKTGSTNGFGAYVAFVPGRD
PDC-8WHM69354.1_909PaBOC       301  YDWPISLKRLQAGNSTPMALQPHRIARLPAPQALEGQRLLNKTGSTNGFGAYVAFVPGRD
PDC-8WHM75768.1_909PaBOC       301  YDWPISLKRLQAGNSTPMALQPHRIARLPAPQALEGQRLLNKTGSTNGFGAYVAFVPGRD
PDC-8WHM84263.1_909PaBOC       301  YDWPISLKRLQAGNSTPMALQPHRIARLPAPQALEGQRLLNKTGSTNGFGAYVAFVPGRD
PDC-8AMA35328.1_919.PaOC       301  YDWPISLKRLQAGNSTPMALQPHRIARLPAPQALEGQRLLNKTGSTNGFGAYVAFVPGRD
PDC-8ANI06966.1_119PaOC        301  YDWPISLKRLQAGNSTPMALQPHRIARLPAPQALEGQRLLNKTGSTNGFGAYVAFVPGRD
PDC-8AYW70752.1_952PaOC        301  YDWPISLKRLQAGNSTPMALQPHRIARLPAPQALEGQRLLNKTGSTNGFGAYVAFVPGRD
PDC-8AYZ76972.1_2272PaOC       301  YDWPISLKRLQAGNSTPMALQPHRIARLPAPQALEGQRLLNKTGSTNGFGAYVAFVPGRD
PDC-8QKP85289.1_778PaOC        301  YDWPISLKRLQAGNSTPMALQPHRIARLPAPQALEGQRLLNKTGSTNGFGAYVAFVPGRD
PDC-8QKR30573.1_5806PaOC       301  YDWPISLKRLQAGNSTPMALQPHRIARLPAPQALEGQRLLNKTGSTNGFGAYVAFVPGRD
PDC-8QLF19879.1_939PaOC        301  YDWPISLKRLQAGNSTPMALQPHRIARLPAPQALEGQRLLNKTGSTNGFGAYVAFVPGRD
PDC-8QYE59452.1_5107PaOC       301  YDWPISLKRLQAGNSTPMALQPHRIARLPAPQALEGQRLLNKTGSTNGFGAYVAFVPGRD
PDC-8QZV98104.1_4956PaOC       301  YDWPISLKRLQAGNSTPMALQPHRIARLPAPQALEGQRLLNKTGSTNGFGAYVAFVPGRD
PDC-8UFK73541.1_1068PaOC       301  YDWPISLKRLQAGNSTPMALQPHRIARLPAPQALEGQRLLNKTGSTNGFGAYVAFVPGRD
PDC-8UGR33975.2_368PaOC        301  YDWPISLKRLQAGNSTPMALQPHRIARLPAPQALEGQRLLNKTGSTNGFGAYVAFVPGRD
PDC-8UTN26696.1_862PaOC        301  YDWPISLKRLQAGNSTPMALQPHRIARLPAPQALEGQRLLNKTGSTNGFGAYVAFVPGRD
PDC-8UTQ91314.1_910PaOC        301  YDWPISLKRLQAGNSTPMALQPHRIARLPAPQALEGQRLLNKTGSTNGFGAYVAFVPGRD
PDC-8UNK85299.1_868PaOC        301  YDWPISLKRLQAGNSTPMALQPHRIARLPAPQALEGQRLLNKTGSTNGFGAYVAFVPGRD
PDC-8UNK97662.1_866PaOC        301  YDWPISLKRLQAGNSTPMALQPHRIARLPAPQALEGQRLLNKTGSTNGFGAYVAFVPGRD
PDC-8USI83494.1_833PaOC        301  YDWPISLKRLQAGNSTPMALQPHRIARLPAPQALEGQRLLNKTGSTNGFGAYVAFVPGRD
PDC-8WAO16415.1_5521PaOC       301  YDWPISLKRLQAGNSTPMALQPHRIARLPAPQALEGQRLLNKTGSTNGFGAYVAFVPGRD
PDC-98QZD62144.1_4346PaBCP     301  YDWPISLKRLQAGNSTPMALQPHRIARLPAPQALEGQRLLNKTGSTNGFGAYVAFVPGRD
```

```
PDC-98QZH48390.1_4214PaBCP      301  YDWPISLKRLQAGNSTPMALQPHRIARLPAPQALEGQRLLNKTGSTNGFGAYVAFVPGRD
PDC-98QZD62144.1_4346PaBOC      301  YDWPISLKRLQAGNSTPMALQPHRIARLPAPQALEGQRLLNKTGSTNGFGAYVAFVPGRD
PDC-98QZH48390.1_4214PaBOC      301  YDWPISLKRLQAGNSTPMALQPHRIARLPAPQALEGQRLLNKTGSTNGFGAYVAFVPGRD
PDC-98ALP56563.1_1203.PaOC      301  YDWPISLKRLQAGNSTPMALQPHRIARLPAPQALEGQRLLNKTGSTNGFGAYVAFVPGRD
PDC-98UTL97623.1_830PaOC        301  YDWPISLKRLQAGNSTPMALQPHRIARLPAPQALEGQRLLNKTGSTNGFGAYVAFVPGRD
PDC-457UTN40061.1_863PaOC       301  YDWPISLKRLQAGNSTPMALQPHRIARLPAPQALEGQRLLNKTGSTNGFGAYVAFVPGRD
PDC-23UXI89283.1_830PaOC        301  YDWPITLKRLQAGNSTPMALQPHRIARLPAPQALEGQRLLNKTGSTNGFGAYVAFVPGRD
PDC-245AUA75408.1_866PaOC       301  YDWPISLKRLQAGNSTPMALQPHRIARLPAPQALEGQRLLNKTGSTNGFGAYVAFVPGRD
PDC-31QZW06655.1_840PaBCP       301  YDWPISLKRLQAGNSTPMALQPHRIARLPAPQALEGQRLLNKTGSTNGFGAYVAFVPGRD
PDC-31QZW06655.1_840PaBOC       301  YDWPISLKRLQAGNSTPMALQPHRIARLPAPQALEGQRLLNKTGSTNGFGAYVAFVPGRD
PDC-31AXN27248.1_931PaOC        301  YDWPISLKRLQAGNSTPMALQPHRIARLPAPQALEGQRLLNKTGSTNGFGAYVAFVPGRD
PDC-31QBC07904.1_3021PaOC       301  YDWPISLKRLQAGNSTPMALQPHRIARLPAPQALEGQRLLNKTGSTNGFGAYVAFVPGRD
PDC-31UFM90275.1_905PaOC        301  YDWPISLKRLQAGNSTPMALQPHRIARLPAPQALEGQRLLNKTGSTNGFGAYVAFVPGRD
PDC-31UFM98870.1_905PaOC        301  YDWPISLKRLQAGNSTPMALQPHRIARLPAPQALEGQRLLNKTGSTNGFGAYVAFVPGRD
PDC-31UNT25139.1_901PaOC        301  YDWPISLKRLQAGNSTPMALQPHRIARLPAPQALEGQRLLNKTGSTNGFGAYVAFVPGRD
PDC-31UWJ96607.1_4267PaOC       301  YDWPISLKRLQAGNSTPMALQPHRIARLPAPQALEGQRLLNKTGSTNGFGAYVAFVPGRD
PDC-31WGX88300.1_823PaOC        301  YDWPISLKRLQAGNSTPMALQPHRIARLPAPQALEGQRLLNKTGSTNGFGAYVAFVPGRD
PDC-31WHM08550.1_859PaOC        301  YDWPISLKRLQAGNSTPMALQPHRIARLPAPQALEGQRLLNKTGSTNGFGAYVAFVPGRD
PDC-39AYK21377.1_872PaOC        301  YDWPISLKRLQAGNSTPMALQPHRIARLPAPQALEGQRLLNKTGSTNGFGAYVAFVPGRD
PDC-39UJC21946.1_837PaOC        301  YDWPISLKRLQAGNSTPMALQPHRIARLPAPQALEGQRLLNKTGSTNGFGAYVAFVPGRD
PDC-30UPL39337.1_928PaBCP       301  YDWPISLKRLQAGNSTPMALQPHRIARLPAPQALEGQRLLNKTGSTNGFGAYVAFVPGRD
PDC-30UPL39337.1_928PaBOC       301  YDWPISLKRLQAGNSTPMALQPHRIARLPAPQALEGQRLLNKTGSTNGFGAYVAFVPGRD
PDC-30UHL95932.1_5029PaOC       301  YDWPISLKRLQAGNSTPMALQPHRIARLPAPQALEGQRLLNKTGSTNGFGAYVAFVPGRD
PDC-30UML99445.1_838PaOC        301  YDWPISLKRLQAGNSTPMALQPHRIARLPAPQALEGQRLLNKTGSTNGFGAYVAFVPGRD
PDC-80WAJ79202.1_935PaOC        301  YDWPISLKRLQAGNSTPMALQPHRIARLPAPQALEGQRLLNKTGSTNGFGAYVAFVPGRD
8SDL_1|Chain                    301  YDWPISLKRLQAGNSTPMALQPHRIARLPAPQALEGQRLLNKTGSTNGFGAYVAFVPGRD
PDC-3UTN33167.1_589PaBCP        301  YDWPISLKRLQAGNSTPMALQPHRIARLPAPQALEGQRLLNKTGSTNGFGAYVAFVPGRD
PDC-3WCI38158.1_984PaBCP        301  YDWPISLKRLQAGNSTPMALQPHRIARLPAPQALEGQRLLNKTGSTNGFGAYVAFVPGRD
PDC-3WCI50909.1_867PaBCP        301  YDWPISLKRLQAGNSTPMALQPHRIARLPAPQALEGQRLLNKTGSTNGFGAYVAFVPGRD
PDC-3WCI90621.1_961PaBCP        301  YDWPISLKRLQAGNSTPMALQPHRIARLPAPQALEGQRLLNKTGSTNGFGAYVAFVPGRD
PDC-3WEO48793.1_832PaBCP        301  YDWPISLKRLQAGNSTPMALQPHRIARLPAPQALEGQRLLNKTGSTNGFGAYVAFVPGRD
PDC-3CAI9907424.1_19963]PaBCP   301  YDWPISLKRLQAGNSTPMALQPHRIARLPAPQALEGQRLLNKTGSTNGFGAYVAFVPGRD
PDC-3UTN33167.1_589PaBOC        301  YDWPISLKRLQAGNSTPMALQPHRIARLPAPQALEGQRLLNKTGSTNGFGAYVAFVPGRD
PDC-3WCI38158.1_984PaBOC        301  YDWPISLKRLQAGNSTPMALQPHRIARLPAPQALEGQRLLNKTGSTNGFGAYVAFVPGRD
PDC-3WCI50909.1_867PaBOC        301  YDWPISLKRLQAGNSTPMALQPHRIARLPAPQALEGQRLLNKTGSTNGFGAYVAFVPGRD
PDC-3WCI90621.1_961PaBOC        301  YDWPISLKRLQAGNSTPMALQPHRIARLPAPQALEGQRLLNKTGSTNGFGAYVAFVPGRD
PDC-3WEO48793.1_832PaBOC        301  YDWPISLKRLQAGNSTPMALQPHRIARLPAPQALEGQRLLNKTGSTNGFGAYVAFVPGRD
PDC-3CAI9907424.1_19963]PaBOC   301  YDWPISLKRLQAGNSTPMALQPHRIARLPAPQALEGQRLLNKTGSTNGFGAYVAFVPGRD
PDC-3AHA17954.1_839.PaOC        301  YDWPISLKRLQAGNSTPMALQPHRIARLPAPQALEGQRLLNKTGSTNGFGAYVAFVPGRD
PDC-3ALE46683.1_840.PaOC        301  YDWPISLKRLQAGNSTPMALQPHRIARLPAPQALEGQRLLNKTGSTNGFGAYVAFVPGRD
PDC-3ARC78082.1_863.PaOC        301  YDWPISLKRLQAGNSTPMALQPHRIARLPAPQALEGQRLLNKTGSTNGFGAYVAFVPGRD
PDC-3ARG53386.1_5885PaOC        301  YDWPISLKRLQAGNSTPMALQPHRIARLPAPQALEGQRLLNKTGSTNGFGAYVAFVPGRD
PDC-3WAC79950.1_4482PaOC        301  YDWPISLKRLQAGNSTPMALQPHRIARLPAPQALEGQRLLNKTGSTNGFGAYVAFVPGRD
PDC-3WAE22012.1_4863PaOC        301  YDWPISLKRLQAGNSTPMALQPHRIARLPAPQALEGQRLLNKTGSTNGFGAYVAFVPGRD
PDC-3WAE29433.1_777PaOC         301  YDWPISLKRLQAGNSTPMALQPHRIARLPAPQALEGQRLLNKTGSTNGFGAYVAFVPGRD
PDC-3WAW42145.1_847PaOC         301  YDWPISLKRLQAGNSTPMALQPHRIARLPAPQALEGQRLLNKTGSTNGFGAYVAFVPGRD
PDC-3WCI57477.1_911PaOC         301  YDWPISLKRLQAGNSTPMALQPHRIARLPAPQALEGQRLLNKTGSTNGFGAYVAFVPGRD
PDC-3WCI77402.1_900PaOC         301  YDWPISLKRLQAGNSTPMALQPHRIARLPAPQALEGQRLLNKTGSTNGFGAYVAFVPGRD
PDC-3WCI64002.1_913PaOC         301  YDWPISLKRLQAGNSTPMALQPHRIARLPAPQALEGQRLLNKTGSTNGFGAYVAFVPGRD
PDC-3WCI83994.1_905PaOC         301  YDWPISLKRLQAGNSTPMALQPHRIARLPAPQALEGQRLLNKTGSTNGFGAYVAFVPGRD
PDC-3WJM48814.1_842PaOC         301  YDWPISLKRLQAGNSTPMALQPHRIARLPAPQALEGQRLLNKTGSTNGFGAYVAFVPGRD
PDC-374ANP60834.1_3741PaBCP     301  YDWPISLKRLQAGNSTPMALQPHRIARLPAPQALEGQRLLNKTGSTNGFGAYVAFVPGRD
PDC-374AYZ86971.1_5950PaBCP     301  YDWPISLKRLQAGNSTPMALQPHRIARLPAPQALEGQRLLNKTGSTNGFGAYVAFVPGRD
PDC-374QZV75115.1_843PaBCP      301  YDWPISLKRLQAGNSTPMALQPHRIARLPAPQALEGQRLLNKTGSTNGFGAYVAFVPGRD
PDC-374QZW12711.1_843PaBCP      301  YDWPISLKRLQAGNSTPMALQPHRIARLPAPQALEGQRLLNKTGSTNGFGAYVAFVPGRD
PDC-374UEG06685.1_907PaBCP      301  YDWPISLKRLQAGNSTPMALQPHRIARLPAPQALEGQRLLNKTGSTNGFGAYVAFVPGRD
PDC-374UGW95802.1_984PaBCP      301  YDWPISLKRLQAGNSTPMALQPHRIARLPAPQALEGQRLLNKTGSTNGFGAYVAFVPGRD
PDC-374WGW26222.1_839PaBCP      301  YDWPISLKRLQAGNSTPMALQPHRIARLPAPQALEGQRLLNKTGSTNGFGAYVAFVPGRD
PDC-374ANP60834.1_3741PaBOC     301  YDWPISLKRLQAGNSTPMALQPHRIARLPAPQALEGQRLLNKTGSTNGFGAYVAFVPGRD
PDC-374AYZ86971.1_5950PaBOC     301  YDWPISLKRLQAGNSTPMALQPHRIARLPAPQALEGQRLLNKTGSTNGFGAYVAFVPGRD
PDC-374QZV75115.1_843PaBOC      301  YDWPISLKRLQAGNSTPMALQPHRIARLPAPQALEGQRLLNKTGSTNGFGAYVAFVPGRD
PDC-374QZW12711.1_843PaBOC      301  YDWPISLKRLQAGNSTPMALQPHRIARLPAPQALEGQRLLNKTGSTNGFGAYVAFVPGRD
PDC-374UEG06685.1_907PaBOC      301  YDWPISLKRLQAGNSTPMALQPHRIARLPAPQALEGQRLLNKTGSTNGFGAYVAFVPGRD
PDC-374UGW95802.1_984PaBOC      301  YDWPISLKRLQAGNSTPMALQPHRIARLPAPQALEGQRLLNKTGSTNGFGAYVAFVPGRD
PDC-374WGW26222.1_839PaBOC      301  YDWPISLKRLQAGNSTPMALQPHRIARLPAPQALEGQRLLNKTGSTNGFGAYVAFVPGRD
PDC-374AOP56366.1_851PaOC       301  YDWPISLKRLQAGNSTPMALQPHRIARLPAPQALEGQRLLNKTGSTNGFGAYVAFVPGRD
PDC-374AVE31445.1_873.PaOC      301  YDWPISLKRLQAGNSTPMALQPHRIARLPAPQALEGQRLLNKTGSTNGFGAYVAFVPGRD
PDC-374QAS71510.1_873PaOC       301  YDWPISLKRLQAGNSTPMALQPHRIARLPAPQALEGQRLLNKTGSTNGFGAYVAFVPGRD
PDC-374QDD38027.1_4463PaOC      301  YDWPISLKRLQAGNSTPMALQPHRIARLPAPQALEGQRLLNKTGSTNGFGAYVAFVPGRD
PDC-374QKF05339.1_5830PaOC      301  YDWPISLKRLQAGNSTPMALQPHRIARLPAPQALEGQRLLNKTGSTNGFGAYVAFVPGRD
PDC-374QKK83983.1_853PaOC       301  YDWPISLKRLQAGNSTPMALQPHRIARLPAPQALEGQRLLNKTGSTNGFGAYVAFVPGRD
PDC-374QKS41466.1_4199PaOC      301  YDWPISLKRLQAGNSTPMALQPHRIARLPAPQALEGQRLLNKTGSTNGFGAYVAFVPGRD
PDC-374QPZ80284.1_873PaOC       301  YDWPISLKRLQAGNSTPMALQPHRIARLPAPQALEGQRLLNKTGSTNGFGAYVAFVPGRD
PDC-374QQW07466.1_922PaOC       301  YDWPISLKRLQAGNSTPMALQPHRIARLPAPQALEGQRLLNKTGSTNGFGAYVAFVPGRD
PDC-374QZU93305.1_843PaOC       301  YDWPISLKRLQAGNSTPMALQPHRIARLPAPQALEGQRLLNKTGSTNGFGAYVAFVPGRD
PDC-374UAC85653.1_934PaOC       301  YDWPISLKRLQAGNSTPMALQPHRIARLPAPQALEGQRLLNKTGSTNGFGAYVAFVPGRD
PDC-374UGR20250.1_3485PaOC      301  YDWPISLKRLQAGNSTPMALQPHRIARLPAPQALEGQRLLNKTGSTNGFGAYVAFVPGRD
PDC-374UIU12727.1_851PaOC       301  YDWPISLKRLQAGNSTPMALQPHRIARLPAPQALEGQRLLNKTGSTNGFGAYVAFVPGRD
PDC-374UJC03215.1_956PaOC       301  YDWPISLKRLQAGNSTPMALQPHRIARLPAPQALEGQRLLNKTGSTNGFGAYVAFVPGRD
PDC-374UJC09425.1_959PaOC       301  YDWPISLKRLQAGNSTPMALQPHRIARLPAPQALEGQRLLNKTGSTNGFGAYVAFVPGRD
PDC-374UJF57976.1_829PaOC       301  YDWPISLKRLQAGNSTPMALQPHRIARLPAPQALEGQRLLNKTGSTNGFGAYVAFVPGRD
PDC-374UJF63613.1_829PaOC       301  YDWPISLKRLQAGNSTPMALQPHRIARLPAPQALEGQRLLNKTGSTNGFGAYVAFVPGRD
PDC-374UJF68041.1_5361PaOC      301  YDWPISLKRLQAGNSTPMALQPHRIARLPAPQALEGQRLLNKTGSTNGFGAYVAFVPGRD
PDC-374UJF74874.1_829PaOC       301  YDWPISLKRLQAGNSTPMALQPHRIARLPAPQALEGQRLLNKTGSTNGFGAYVAFVPGRD
PDC-374UJF80517.1_829PaOC       301  YDWPISLKRLQAGNSTPMALQPHRIARLPAPQALEGQRLLNKTGSTNGFGAYVAFVPGRD
PDC-374UJF86151.1_829PaOC       301  YDWPISLKRLQAGNSTPMALQPHRIARLPAPQALEGQRLLNKTGSTNGFGAYVAFVPGRD
PDC-374UJF90590.1_5359PaOC      301  YDWPISLKRLQAGNSTPMALQPHRIARLPAPQALEGQRLLNKTGSTNGFGAYVAFVPGRD
PDC-374UJF97432.1_830PaOC       301  YDWPISLKRLQAGNSTPMALQPHRIARLPAPQALEGQRLLNKTGSTNGFGAYVAFVPGRD
PDC-374UJG01877.1_5359PaOC      301  YDWPISLKRLQAGNSTPMALQPHRIARLPAPQALEGQRLLNKTGSTNGFGAYVAFVPGRD
PDC-374UJG08704.1_829PaOC       301  YDWPISLKRLQAGNSTPMALQPHRIARLPAPQALEGQRLLNKTGSTNGFGAYVAFVPGRD
PDC-374UJG14426.1_829PaOC       301  YDWPISLKRLQAGNSTPMALQPHRIARLPAPQALEGQRLLNKTGSTNGFGAYVAFVPGRD
PDC-374UJG20129.1_829PaOC       301  YDWPISLKRLQAGNSTPMALQPHRIARLPAPQALEGQRLLNKTGSTNGFGAYVAFVPGRD
PDC-374UJG25828.1_829PaOC       301  YDWPISLKRLQAGNSTPMALQPHRIARLPAPQALEGQRLLNKTGSTNGFGAYVAFVPGRD
PDC-374UJG31693.1_884PaOC       301  YDWPISLKRLQAGNSTPMALQPHRIARLPAPQALEGQRLLNKTGSTNGFGAYVAFVPGRD
PDC-374UJG36239.1_5358PaOC      301  YDWPISLKRLQAGNSTPMALQPHRIARLPAPQALEGQRLLNKTGSTNGFGAYVAFVPGRD
PDC-374UGR51117.1_68PaOC        301  YDWPISLKRLQAGNSTPMALQPHRIARLPAPQALEGQRLLNKTGSTNGFGAYVAFVPGRD
PDC-374UON75698.1_5570PaOC      301  YDWPISLKRLQAGNSTPMALQPHRIARLPAPQALEGQRLLNKTGSTNGFGAYVAFVPGRD
PDC-374UPG04116.1_3174PaOC      301  YDWPISLKRLQAGNSTPMALQPHRIARLPAPQALEGQRLLNKTGSTNGFGAYVAFVPGRD
PDC-374UPZ07602.1_2096PaOC      301  YDWPISLKRLQAGNSTPMALQPHRIARLPAPQALEGQRLLNKTGSTNGFGAYVAFVPGRD
PDC-374URM55875.1_5937PaOC      301  YDWPISLKRLQAGNSTPMALQPHRIARLPAPQALEGQRLLNKTGSTNGFGAYVAFVPGRD
PDC-374USV13808.1_3922PaOC      301  YDWPISLKRLQAGNSTPMALQPHRIARLPAPQALEGQRLLNKTGSTNGFGAYVAFVPGRD
PDC-374USV19196.1_4087PaOC      301  YDWPISLKRLQAGNSTPMALQPHRIARLPAPQALEGQRLLNKTGSTNGFGAYVAFVPGRD
PDC-374USX84542.1_851PaOC       301  YDWPISLKRLQAGNSTPMALQPHRIARLPAPQALEGQRLLNKTGSTNGFGAYVAFVPGRD
```

```
PDC-374USX78872.1_851PaOC        301  YDWPISLKRLQAGNSTPMALQPHRIARLPAPQALEGQRLLNKTGSTNGFGAYVAFVPGRD
PDC-374UUJ85605.1_1747PaOC       301  YDWPISLKRLQAGNSTPMALQPHRIARLPAPQALEGQRLLNKTGSTNGFGAYVAFVPGRD
PDC-374UVS71982.1_4220PaOC       301  YDWPISLKRLQAGNSTPMALQPHRIARLPAPQALEGQRLLNKTGSTNGFGAYVAFVPGRD
PDC-374UWI85046.1_4210PaOC       301  YDWPISLKRLQAGNSTPMALQPHRIARLPAPQALEGQRLLNKTGSTNGFGAYVAFVPGRD
PDC-374UWI92968.1_851PaOC        301  YDWPISLKRLQAGNSTPMALQPHRIARLPAPQALEGQRLLNKTGSTNGFGAYVAFVPGRD
PDC-374UXA22714.1_4203PaOC       301  YDWPISLKRLQAGNSTPMALQPHRIARLPAPQALEGQRLLNKTGSTNGFGAYVAFVPGRD
PDC-374UXA30656.1_926PaOC        301  YDWPISLKRLQAGNSTPMALQPHRIARLPAPQALEGQRLLNKTGSTNGFGAYVAFVPGRD
PDC-374UXA44988.1_926PaOC        301  YDWPISLKRLQAGNSTPMALQPHRIARLPAPQALEGQRLLNKTGSTNGFGAYVAFVPGRD
PDC-374UXN39470.1_4222PaOC       301  YDWPISLKRLQAGNSTPMALQPHRIARLPAPQALEGQRLLNKTGSTNGFGAYVAFVPGRD
PDC-374UZA87382.1_4980PaOC       301  YDWPISLKRLQAGNSTPMALQPHRIARLPAPQALEGQRLLNKTGSTNGFGAYVAFVPGRD
PDC-374WBM66401.1_846PaOC        301  YDWPISLKRLQAGNSTPMALQPHRIARLPAPQALEGQRLLNKTGSTNGFGAYVAFVPGRD
PDC-374WGJ75339.1_925PaOC        301  YDWPISLKRLQAGNSTPMALQPHRIARLPAPQALEGQRLLNKTGSTNGFGAYVAFVPGRD
PDC-374WGW87865.1_837PaOC        301  YDWPISLKRLQAGNSTPMALQPHRIARLPAPQALEGQRLLNKTGSTNGFGAYVAFVPGRD
PDC-103QES80695.1_910PaOC        301  YDWPISLKRLQAGNSTPMALQPHRIARLPAPQALEGQRLLNKTGSTNGFGAYVAFVPGRD
PDC-103QPV53905.1_6224PaOC       301  YDWPISLKRLQAGNSTPMALQPHRIARLPAPQALEGQRLLNKTGSTNGFGAYVAFVPGRD
PDC-35AVR81260.1_945.PaOC        301  YDWPISLKRLQAGNSTPMALQPHRIARLPAPQALEGQRLLNKTGSTNGFGAYVAFVPGRD
PDC-35QJA35930.1_977PaOC         301  YDWPISLKRLQAGNSTPMALQPHRIARLPAPQALEGQRLLNKTGSTNGFGAYVAFVPGRD
PDC-35AYZ46521.1_3210PaOC        301  YDWPISLKRLQAGNSTPMALQPHRIARLPAPQALEGQRLLNKTGSTNGFGAYVAFVPGRD
PDC-35AYZ59004.1_3976PaOC        301  YDWPISLKRLQAGNSTPMALQPHRIARLPAPQALEGQRLLNKTGSTNGFGAYVAFVPGRD
PDC-35QDR21260.1_3098PaOC        301  YDWPISLKRLQAGNSTPMALQPHRIARLPAPQALEGQRLLNKTGSTNGFGAYVAFVPGRD
PDC-35QEK40749.1_862PaOC         301  YDWPISLKRLQAGNSTPMALQPHRIARLPAPQALEGQRLLNKTGSTNGFGAYVAFVPGRD
PDC-35AUB00033.3_866PaOC         301  YDWPISLKRLQAGNSTPMALQPHRIARLPAPQALEGQRLLNKTGSTNGFGAYVAFVPGRD
PDC-35QPP34775.1_1311PaOC        301  YDWPISLKRLQAGNSTPMALQPHRIARLPAPQALEGQRLLNKTGSTNGFGAYVAFVPGRD
PDC-35QLF38648.1_891PaOC         301  YDWPISLKRLQAGNSTPMALQPHRIARLPAPQALEGQRLLNKTGSTNGFGAYVAFVPGRD
PDC-35QLF14474.1_2055PaOC        301  YDWPISLKRLQAGNSTPMALQPHRIARLPAPQALEGQRLLNKTGSTNGFGAYVAFVPGRD
PDC-35QQV60864.1_5728PaOC        301  YDWPISLKRLQAGNSTPMALQPHRIARLPAPQALEGQRLLNKTGSTNGFGAYVAFVPGRD
PDC-35QQW01132.1_862PaOC         301  YDWPISLKRLQAGNSTPMALQPHRIARLPAPQALEGQRLLNKTGSTNGFGAYVAFVPGRD
PDC-35QQW17992.1_5232PaOC        301  YDWPISLKRLQAGNSTPMALQPHRIARLPAPQALEGQRLLNKTGSTNGFGAYVAFVPGRD
PDC-35QQW23330.1_862PaOC         301  YDWPISLKRLQAGNSTPMALQPHRIARLPAPQALEGQRLLNKTGSTNGFGAYVAFVPGRD
PDC-35QZE42332.1_862PaOC         301  YDWPISLKRLQAGNSTPMALQPHRIARLPAPQALEGQRLLNKTGSTNGFGAYVAFVPGRD
PDC-35UFM83790.1_826PaOC         301  YDWPISLKRLQAGNSTPMALQPHRIARLPAPQALEGQRLLNKTGSTNGFGAYVAFVPGRD
PDC-35UJC15714.1_892PaOC         301  YDWPISLKRLQAGNSTPMALQPHRIARLPAPQALEGQRLLNKTGSTNGFGAYVAFVPGRD
PDC-35UTR21121.1_931PaOC         301  YDWPISLKRLQAGNSTPMALQPHRIARLPAPQALEGQRLLNKTGSTNGFGAYVAFVPGRD
PDC-35UUP09555.1_853PaOC         301  YDWPISLKRLQAGNSTPMALQPHRIARLPAPQALEGQRLLNKTGSTNGFGAYVAFVPGRD
PDC-35UWG56199.1_885PaOC         301  YDWPISLKRLQAGNSTPMALQPHRIARLPAPQALEGQRLLNKTGSTNGFGAYVAFVPGRD
PDC-43UNT30829.1_824PaOC         301  YDWPISLKRLQAGNSTPMALQPHRIARLPVPQALEGQRLLNKTGSTNGFGAYVAFVPGRD
PDC-45ALZ89573.1_855.PaOC        301  YDWPISLKRLQAGNSTPMALQPHRIARLPAPQALEGQRLLNKTGSTNGFGAYVAFVPGRD
PDC-45QKL11315.1_849PaOC         301  YDWPISLKRLQAGNSTPMALQPHRIARLPAPQALEGQRLLNKTGSTNGFGAYVAFVPGRD
PDC-45QQV94504.1_850PaOC         301  YDWPISLKRLQAGNSTPMALQPHRIARLPAPQALEGQRLLNKTGSTNGFGAYVAFVPGRD
PDC-162UJB98759.1_2776PaOC       301  YDWPISLKRLQAGNSTPMALQPHRIARLPAPQALEGQRLLNKTGSTNGFGAYVAFVPGRD
PDC-123QYE90948.1_3103PaOC       301  YDWPISLKRLQAGNSTPMALQPHRIARLPAPQALEGQRLLNKTGSTNGFGAYVAFVPGRD
PDC-5UXH60990.1_2046PaBCP        301  YDWPISLKRLQAGNSTPMALQPHRIARLPAPQALEGQRLLNKTGSTNGFGAYVAFVPGRD
PDC-5WBA37364.1_905PaBCP         301  YDWPISLKRLQAGNSTPMALQPHRIARLPAPQALEGQRLLNKTGSTNGFGAYVAFVPGRD
PDC-5WGJ68312.1_842PaBCP         301  YDWPISLKRLQAGNSTPMALQPHRIARLPAPQALEGQRLLNKTGSTNGFGAYVAFVPGRD
PDC-5WGT14845.1_882PaBCP         301  YDWPISLKRLQAGNSTPMALQPHRIARLPAPQALEGQRLLNKTGSTNGFGAYVAFVPGRD
PDC-5WGX69730.1_872PaBCP         301  YDWPISLKRLQAGNSTPMALQPHRIARLPAPQALEGQRLLNKTGSTNGFGAYVAFVPGRD
PDC-5CAI9893238.1_8405]PaBCP    301  YDWPISLKRLQAGNSTPMALQPHRIARLPAPQALEGQRLLNKTGSTNGFGAYVAFVPGRD
PDC-5CAI9905252.1_60335]PaBCP   301  YDWPISLKRLQAGNSTPMALQPHRIARLPAPQALEGQRLLNKTGSTNGFGAYVAFVPGRD
PDC-5UXH60990.1_2046PaBOC        301  YDWPISLKRLQAGNSTPMALQPHRIARLPAPQALEGQRLLNKTGSTNGFGAYVAFVPGRD
PDC-5WBA37364.1_905PaBOC         301  YDWPISLKRLQAGNSTPMALQPHRIARLPAPQALEGQRLLNKTGSTNGFGAYVAFVPGRD
PDC-5WGJ68312.1_842PaBOC         301  YDWPISLKRLQAGNSTPMALQPHRIARLPAPQALEGQRLLNKTGSTNGFGAYVAFVPGRD
PDC-5WGT14845.1_882PaBOC         301  YDWPISLKRLQAGNSTPMALQPHRIARLPAPQALEGQRLLNKTGSTNGFGAYVAFVPGRD
PDC-5WGX69730.1_872PaBOC         301  YDWPISLKRLQAGNSTPMALQPHRIARLPAPQALEGQRLLNKTGSTNGFGAYVAFVPGRD
PDC-5CAI9893238.1_8405]PaBOC    301  YDWPISLKRLQAGNSTPMALQPHRIARLPAPQALEGQRLLNKTGSTNGFGAYVAFVPGRD
PDC-5CAI9905252.1_60335]PaBOC   301  YDWPISLKRLQAGNSTPMALQPHRIARLPAPQALEGQRLLNKTGSTNGFGAYVAFVPGRD
PDC-5ALU48309.1_2211.PaOC       301  YDWPISLKRLQAGNSTPMALQPHRIARLPAPQALEGQRLLNKTGSTNGFGAYVAFVPGRD
PDC-5AMX89317.1_4085.PaOC       301  YDWPISLKRLQAGNSTPMALQPHRIARLPAPQALEGQRLLNKTGSTNGFGAYVAFVPGRD
PDC-5ATH01452.1_827PaOC         301  YDWPISLKRLQAGNSTPMALQPHRIARLPAPQALEGQRLLNKTGSTNGFGAYVAFVPGRD
PDC-5UTN13111.1_830PaOC         301  YDWPISLKRLQAGNSTPMALQPHRIARLPAPQALEGQRLLNKTGSTNGFGAYVAFVPGRD
PDC-5UUH88148.1_851PaOC         301  YDWPISLKRLQAGNSTPMALQPHRIARLPAPQALEGQRLLNKTGSTNGFGAYVAFVPGRD
PDC-5UUH98459.1_806PaOC         301  YDWPISLKRLQAGNSTPMALQPHRIARLPAPQALEGQRLLNKTGSTNGFGAYVAFVPGRD
PDC-5UVH93148.1_837PaOC         301  YDWPISLKRLQAGNSTPMALQPHRIARLPAPQALEGQRLLNKTGSTNGFGAYVAFVPGRD
PDC-5UWU62523.1_171PaOC         301  YDWPISLKRLQAGNSTPMALQPHRIARLPAPQALEGQRLLNKTGSTNGFGAYVAFVPGRD
PDC-5WDZ01346.1_910PaOC         301  YDWPISLKRLQAGNSTPMALQPHRIARLPAPQALEGQRLLNKTGSTNGFGAYVAFVPGRD
PDC-5WEO42644.1_846PaOC         301  YDWPISLKRLQAGNSTPMALQPHRIARLPAPQALEGQRLLNKTGSTNGFGAYVAFVPGRD
PDC-5WGW99708.1_849PaOC         301  YDWPISLKRLQAGNSTPMALQPHRIARLPAPQALEGQRLLNKTGSTNGFGAYVAFVPGRD
PDC-5WJM42992.1_848PaOC         301  YDWPISLKRLQAGNSTPMALQPHRIARLPAPQALEGQRLLNKTGSTNGFGAYVAFVPGRD
PDC-60QWY08308.1_854PaBCP       301  YDWPISLKRLQAGNSTPMALQPHRIARLPAPQALEGQRLLNKTGSTNGFGAYVAFVPGRD
PDC-60QZV50082.1_838PaBCP       301  YDWPISLKRLQAGNSTPMALQPHRIARLPAPQALEGQRLLNKTGSTNGFGAYVAFVPGRD
PDC-60QWY08308.1_854PaBOC       301  YDWPISLKRLQAGNSTPMALQPHRIARLPAPQALEGQRLLNKTGSTNGFGAYVAFVPGRD
PDC-60QZV50082.1_838PaBOC       301  YDWPISLKRLQAGNSTPMALQPHRIARLPAPQALEGQRLLNKTGSTNGFGAYVAFVPGRD
PDC-60QPZ60674.1_902PaOC        301  YDWPISLKRLQAGNSTPMALQPHRIARLPAPQALEGQRLLNKTGSTNGFGAYVAFVPGRD
PDC-60WAJ92260.1_847PaOC        301  YDWPISLKRLQAGNSTPMALQPHRIARLPAPQALEGQRLLNKTGSTNGFGAYVAFVPGRD
PDC-60WAJ98065.1_847PaOC        301  YDWPISLKRLQAGNSTPMALQPHRIARLPAPQALEGQRLLNKTGSTNGFGAYVAFVPGRD
PDC-15UNL10022.1_837PaBCP       301  YDWPISLKRLQAGNSTPMALQPHRIARLPAPQALEGQRLLNKTGSTNGFGAYVAFVPGRD
PDC-15UNL10022.1_837PaBOC       301  YDWPISLKRLQAGNSTPMALQPHRIARLPAPQALEGQRLLNKTGSTNGFGAYVAFVPGRD
PDC-15AOT38867.1_3370PaOC       301  YDWPISLKRLQAGNSTPMALQPHRIARLPAPQALEGQRLLNKTGSTNGFGAYVAFVPGRD
PDC-15ALZ27705.1_859PaOC        301  YDWPISLKRLQAGNSTPMALQPHRIARLPAPQALEGQRLLNKTGSTNGFGAYVAFVPGRD
PDC-15AXC19915.1_1529PaOC       301  YDWPISLKRLQAGNSTPMALQPHRIARLPAPQALEGQRLLNKTGSTNGFGAYVAFVPGRD
PDC-15UTP69334.1_895PaOC        301  YDWPISLKRLQAGNSTPMALQPHRIARLPAPQALEGQRLLNKTGSTNGFGAYVAFVPGRD
PDC-15UNK91447.1_837PaOC        301  YDWPISLKRLQAGNSTPMALQPHRIARLPAPQALEGQRLLNKTGSTNGFGAYVAFVPGRD
PDC-15UNL03855.1_837PaOC        301  YDWPISLKRLQAGNSTPMALQPHRIARLPAPQALEGQRLLNKTGSTNGFGAYVAFVPGRD
PDC-28UYM61272.1_6539PaOC       301  YDWPISLKRLQAGNSTPMALQPHRIARLPAPQALEGQRLLNKTGSTNGFGAYVAFVPGRD
PDC-22QPZ72804.1_848PaOC        301  YDWPISLKRLQAGNSTPMALQPHRIARLPAPQALEGQRLLNKTGSTNGFGAYVAFIPGRD
PDC-22QQM08170.1_4816PaOC       301  YDWPISLKRLQAGNSTPMALQPHRIARLPAPQALEGQRLLNKTGSTNGFGAYVAFIPGRD
PDC-97QKR35301.1_4456PaOC       301  YDWPISLKRLQAGNSTPMALQPHRIARLPAPQALEGQRLLNKTGSTNGFGAYVAFVPGRD
PDC-51WGV68048.1_897PaOC        301  YDWPISLKRLQAGNSTPMALQPHRIARLPAPQALEGQRLLNKTGSTNGFGAYVAFVPGRD
PDC-1HW06_09835_867PaOC         301  YDWPISLKRLQAGNSTPMALQPHRIARLPAPQALEGQRLLNKTGSTNGFGAYVAFVPGRD
PDC-151QQV49515.1_805PaOC       301  YDWPIDLKRLQAGNSAPMALQAHKVARLPAPQALDGQRLLNKTGSTNGFGAYLAFIPGRD
consensus>70                          YDWPIsLKR$QAGNStPMALQpHr!ARLPaPQAL#GQRLLNKTGSTNGFGAYvAF!PGRD
                                acc
```

```
                                      β15          α13
PDC-374QKR78821.1_828PaOC

PDC-374QKR78821.1_828PaOC      361  LGLVILANRNYPNAERVKIAYAILSGLEQQGKVPLKRAR...EGDG.........A
PDC-331WCI44612.1_917PaOC      352  LGLVILANRNYPNAERVKIAYAILSGLEQQGKVPLKR...................
PDC-8HV97_01835_992PaOC        361  LGLVILANRNYPNAERVKIAYAILSGLEQQGKVPLKR.SALARATE.........R
PDC-8CLH63_27535_5433PaOC      361  LGLVILANRNYPNAERVKIAYAILSGLEQQGKVPLKR.SALARATE.........R
PDC-31HW08_20870_899PaOC       361  LGLVILANRNYPNAERVKIAYAILSGLEQQGKVPLKR.SALARATE.........R
PDC-3CPZ93_04765_942PaBCP      361  LGLVILANRNYPNAERVKIAYAILSGLEQQGKVPLKR.SALARATE.........R
PDC-3CPZ93_04765_942PaBOC      361  LGLVILANRNYPNAERVKIAYAILSGLEQQGKVPLKR.SALARATE.........R
PDC-3CD796_04405_870PaOC       361  LGLVILANRNYPNAERVKIAYAILSGLEQQGKVPLKR.SALARATE.........R
PDC-1HV98_23795_933PaOC        361  LGLVILANRNYPNAERVKIAYAILSGLEQQGKVPLKR.SALARATE.........R
PDC-103HV95_30355_911PaOC      361  LGLVILANRNYPNAERVKIAYAILSGLEQQGKVPLKR.SALARATE.........R
PDC-5HW02_17620_927PaOC        361  LGLVILANRNYPNAERVKIAYAILSGLEQQGKVPLKR.SALARATE.........R
PDC-5HW01_11705_873PaOC        361  LGLVILANRNYPNAERVKIAYAILSGLEQQGKVPLKR.SALARATE.........R
PDC-5CD800_33195_6567PaOC      361  LGLVILANRNYPNAERVKIAYAILSGLEQQGKVPLKR.SALARATE.........R
PDC-5CD799_31455_6219PaOC      361  LGLVILANRNYPNAERVKIAYAILSGLEQQGKVPLKR.SALARATE.........R
PDC-46CEK59_30555_6032PaOC     361  LGLVILANRNYPNAERVKIAYAILSGLEQQAKVPLKR.SALARATE.........R
PDC-66QTQ98808.1_855PaOC       361  LGLVILANRNYPNAERVKIAYAILSGLEQQAKVPLKR...................
PDC-364QYE79191.1_1969PaOC     361  LGLVILANRNYPNAERVKIAYAILSGLEQQAKVPLKR...................
PDC-122QYN07359.1_5685PaOC     361  LGLVILANRNYPNAERVKIAYAILSGLEQQGKVPLKR...................
PDC-100QKR60216.1_898PaOC      361  LGLVILANRNYPNAERVKIAYAILSGLEQQAKVPLKR...................
PDC-34WBM27707.1_865PaBCP      361  LGLVILANRNYPNAERVKIAYAILSGLEQQAKVPLKR...................
PDC-34WGX11792.1_865PaBCP      361  LGLVILANRNYPNAERVKIAYAILSGLEQQAKVPLKR...................
PDC-34WBM27707.1_865PaBOC      361  LGLVILANRNYPNAERVKIAYAILSGLEQQAKVPLKR...................
PDC-34WGX11792.1_865PaBOC      361  LGLVILANRNYPNAERVKIAYAILSGLEQQAKVPLKR...................
PDC-34ALY72428.1_939PaOC       361  LGLVILANRNYPNAERVKIAYAILSGLEQQAKVPLKR...................
PDC-34ASM83699.1_885PaOC       361  LGLVILANRNYPNAERVKIAYAILSGLEQQAKVPLKR...................
PDC-34AYW64445.1_883PaOC       361  LGLVILANRNYPNAERVKIAYAILSGLEQQAKVPLKR...................
PDC-34UTR27428.1_860PaOC       361  LGLVILANRNYPNAERVKIAYAILSGLEQQAKVPLKR...................
PDC-34UOD92021.1_934PaOC       361  LGLVILANRNYPNAERVKIAYAILSGLEQQAKVPLKR...................
PDC-34WIV39806.1_860PaOC       361  LGLVILANRNYPNAERVKIAYAILSGLEQQAKVPLKR...................
PDC-264QKR42793.1_833PaOC      361  LGLVILANRNYPNAERVKIAYAILSGLEQQGKVPLKR...................
PDC-167QIZ28250.1_4314PaOC     361  LGLVILANRNYPNAERVKIAYAILSGLEQQAKVPLAR...................
PDC-167UTQ27575.1_842PaOC      361  LGLVILANRNYPNAERVKIAYAILSGLEQQAKVPLAR...................
PDC-6QZV30905.1_832PaBCP       361  LGLVILANRNYPNAERVKIAYAILSGLEQQGKVPLKR...................
PDC-6QZV30905.1_832PaBOC       361  LGLVILANRNYPNAERVKIAYAILSGLEQQGKVPLKR...................
PDC-37WAJ85675.1_843PaBCP      361  LGLVILANRNYPNAERVKIAYAILSGLEQQAKVPLKR...................
PDC-37WGX56158.1_855PaBCP      361  LGLVILANRNYPNAERVKIAYAILSGLEQQAKVPLKR...................
PDC-37WAJ85675.1_843PaBOC      361  LGLVILANRNYPNAERVKIAYAILSGLEQQAKVPLKR...................
PDC-37WGX56158.1_855PaBOC      361  LGLVILANRNYPNAERVKIAYAILSGLEQQAKVPLKR...................
PDC-37ANA71197.1_2757.PaOC     361  LGLVILANRNYPNAERVKIAYAILSGLEQQAKVPLKR...................
PDC-37WGX14507.1_3195PaOC      361  LGLVILANRNYPNAERVKIAYAILSGLEQQAKVPLKR...................
PDC-46AUA93832.1_897PaOC       361  LGLVILANRNYPNAERVKIAYAILSGLEQQAKVPLKR...................
PDC-105QGP95004.1_877PaBCP     361  LGLVILANRNYPNAERVKIAYAILSGLEQQAKVPLKR...................
PDC-105QGP95004.1_877PaBOC     361  LGLVILANRNYPNAERVKIAYAILSGLEQQAKVPLKR...................
PDC-12AVN43882.1_2061.PaOC     361  LGLVILANRNYPNAERVKIAYAILSGLEQQAKVPLKR...................
PDC-12QNE63041.1_859PaOC       361  LGLVILANRNYPNAERVKIAYAILSGLEQQAKVPLKR...................
PDC-12WGW93864.1_852PaOC       361  LGLVILANRNYPNAERVKIAYAILSGLEQQAKVPLKR...................
PDC-12WGX05587.1_852PaOC       361  LGLVILANRNYPNAERVKIAYAILSGLEQQAKVPLKR...................
PDC-11QSA85555.1_5581PaBCP     361  LGLVILANRNYPNAERVKIAYAILSGLEQQAKVPLKR...................
PDC-11QSA91467.1_5367PaBCP     361  LGLVILANRNYPNAERVKIAYAILSGLEQQAKVPLKR...................
PDC-11QSA85555.1_5581PaBOC     361  LGLVILANRNYPNAERVKIAYAILSGLEQQAKVPLKR...................
PDC-11QSA91467.1_5367PaBOC     361  LGLVILANRNYPNAERVKIAYAILSGLEQQAKVPLKR...................
PDC-11AYQ83203.1_3491PaOC      361  LGLVILANRNYPNAERVKIAYAILSGLEQQAKVPLKR...................
PDC-11AYR15208.1_5205PaOC      361  LGLVILANRNYPNAERVKIAYAILSGLEQQAKVPLKR...................
PDC-11AZN02324.1_4787PaOC      361  LGLVILANRNYPNAERVKIAYAILSGLEQQAKVPLKR...................
PDC-11AZN04123.1_203PaOC       361  LGLVILANRNYPNAERVKIAYAILSGLEQQAKVPLKR...................
PDC-11AZN17071.1_5847PaOC      361  LGLVILANRNYPNAERVKIAYAILSGLEQQAKVPLKR...................
PDC-11AZN48510.1_4981PaOC      361  LGLVILANRNYPNAERVKIAYAILSGLEQQAKVPLKR...................
PDC-11AZN60564.1_5101PaOC      361  LGLVILANRNYPNAERVKIAYAILSGLEQQAKVPLKR...................
PDC-11AZN55779.1_6545PaOC      361  LGLVILANRNYPNAERVKIAYAILSGLEQQAKVPLKR...................
PDC-11AYF68870.1_744PaOC       361  LGLVILANRNYPNAERVKIAYAILSGLEQQAKVPLKR...................
PDC-11QUS21307.1_2433PaOC      361  LGLVILANRNYPNAERVKIAYAILSGLEQQAKVPLKR...................
PDC-11UVH87183.1_982PaOC       361  LGLVILANRNYPNAERVKIAYAILSGLEQQAKVPLKR...................
PDC-16QCC64126.1_958PaBCP      361  LGLVILANRNYPNAERVKIAYAILSGLEQQAKVPLKR...................
PDC-16UBI70916.1_933PaBCP      361  LGLVILANRNYPNAERVKIAYAILSGLEQQAKVPLKR...................
PDC-16UTQ84596.1_855PaBCP      361  LGLVILANRNYPNAERVKIAYAILSGLEQQAKVPLKR...................
PDC-16QCC64126.1_958PaBOC      361  LGLVILANRNYPNAERVKIAYAILSGLEQQAKVPLKR...................
PDC-16UBI70916.1_933PaBOC      361  LGLVILANRNYPNAERVKIAYAILSGLEQQAKVPLKR...................
PDC-16UTQ84596.1_855PaBOC      361  LGLVILANRNYPNAERVKIAYAILSGLEQQAKVPLKR...................
PDC-16ALS12949.1_2977.PaOC     361  LGLVILANRNYPNAERVKIAYAILSGLEQQAKVPLKR...................
PDC-16AYW58201.1_855PaOC       361  LGLVILANRNYPNAERVKIAYAILSGLEQQAKVPLKR...................
PDC-16QDR00398.1_890PaOC       361  LGLVILANRNYPNAERVKIAYAILSGLEQQAKVPLKR...................
PDC-16QGJ31628.1_895PaOC       361  LGLVILANRNYPNAERVKIAYAILSGLEQQAKVPLKR...................
PDC-16QKG34934.1_1840PaOC      361  LGLVILANRNYPNAERVKIAYAILSGLEQQAKVPLKR...................
PDC-16QOQ64874.1_122PaOC       361  LGLVILANRNYPNAERVKIAYAILSGLEQQAKVPLKR...................
PDC-16QVQ73157.1_860PaOC       361  LGLVILANRNYPNAERVKIAYAILSGLEQQAKVPLKR...................
PDC-16UOC64480.1_827PaOC       361  LGLVILANRNYPNAERVKIAYAILSGLEQQAKVPLKR...................
PDC-16UOC70564.1_826PaOC       361  LGLVILANRNYPNAERVKIAYAILSGLEQQAKVPLKR...................
PDC-16UOC76649.1_827PaOC       361  LGLVILANRNYPNAERVKIAYAILSGLEQQAKVPLKR...................
PDC-16UOC82740.1_827PaOC       361  LGLVILANRNYPNAERVKIAYAILSGLEQQAKVPLKR...................
PDC-16UOC88825.1_827PaOC       361  LGLVILANRNYPNAERVKIAYAILSGLEQQAKVPLKR...................
PDC-16UOE68701.1_827PaOC       361  LGLVILANRNYPNAERVKIAYAILSGLEQQAKVPLKR...................
PDC-16WDC65088.1_844PaOC       361  LGLVILANRNYPNAERVKIAYAILSGLEQQAKVPLKR...................
PDC-16WGW70130.1_845PaOC       361  LGLVILANRNYPNAERVKIAYAILSGLEQQAKVPLKR...................
PDC-16WGW76010.1_845PaOC       361  LGLVILANRNYPNAERVKIAYAILSGLEQQAKVPLKR...................
PDC-16WGW81892.1_845PaOC       361  LGLVILANRNYPNAERVKIAYAILSGLEQQAKVPLKR...................
PDC-16WGX43443.1_806PaOC       361  LGLVILANRNYPNAERVKIAYAILSGLEQQAKVPLKR...................
PDC-16WJM37210.1_871PaOC       361  LGLVILANRNYPNAERVKIAYAILSGLEQQAKVPLKR...................
PDC-59UQZ18314.1_902PaOC       361  LGLVILANRNYPNAERVKIAYAILSGLEQQAKVPLKR...................
PDC-142WGW57525.1_830PaBCP     361  LGLVILANRNYPNAERVKIAYAILSGLEQQAKVPLKR...................
PDC-142WGW32210.1_830PaBCP     361  LGLVILANRNYPNAERVKIAYAILSGLEQQAKVPLKR...................
PDC-142WGW44776.1_830PaBCP     361  LGLVILANRNYPNAERVKIAYAILSGLEQQAKVPLKR...................
PDC-142WGW57525.1_830PaBOC     361  LGLVILANRNYPNAERVKIAYAILSGLEQQAKVPLKR...................
PDC-142WGW32210.1_830PaBOC     361  LGLVILANRNYPNAERVKIAYAILSGLEQQAKVPLKR...................
PDC-142WGW44776.1_830PaBOC     361  LGLVILANRNYPNAERVKIAYAILSGLEQQAKVPLKR...................
PDC-142AON05138.1_825PaOC      361  LGLVILANRNYPNAERVKIAYAILSGLEQQAKVPLKR...................
PDC-142AON11127.1_825PaOC      361  LGLVILANRNYPNAERVKIAYAILSGLEQQAKVPLKR...................
PDC-142AON23122.1_825PaOC      361  LGLVILANRNYPNAERVKIAYAILSGLEQQAKVPLKR...................
PDC-142AON29110.1_825PaOC      361  LGLVILANRNYPNAERVKIAYAILSGLEQQAKVPLKR...................
PDC-142AON41099.1_825PaOC      361  LGLVILANRNYPNAERVKIAYAILSGLEQQAKVPLKR...................
PDC-142AON47112.1_824PaOC      361  LGLVILANRNYPNAERVKIAYAILSGLEQQAKVPLKR...................
PDC-142AON17115.1_824PaOC      361  LGLVILANRNYPNAERVKIAYAILSGLEQQAKVPLKR...................
PDC-142AON35116.1_825PaOC      361  LGLVILANRNYPNAERVKIAYAILSGLEQQAKVPLKR...................
PDC-19aWGW51434.1_1207PaBCP    361  LGLVILANRNYPNAERVKIAYAILSGLEQQAKVPLKR...................
```

```
PDC-19aWGW37666.1_6817PaBCP    361  LGLVILANRNYPNAERVKIAYAILSGLEQQAKVPLKR.......................
PDC-19aWGW59864.1_3660PaBCP    361  LGLVILANRNYPNAERVKIAYAILSGLEQQAKVPLKR.......................
PDC-19aWGX49838.1_877PaBCP     361  LGLVILANRNYPNAERVKIAYAILSGLEQQAKVPLKR.......................
PDC-19aWGX24308.1_877PaBCP     361  LGLVILANRNYPNAERVKIAYAILSGLEQQAKVPLKR.......................
PDC-19aWGX35552.1_5487PaBCP    361  LGLVILANRNYPNAERVKIAYAILSGLEQQAKVPLKR.......................
PDC-19aWKA37694.1_2446PaBCP    361  LGLVILANRNYPNAERVKIAYAILSGLEQQAKVPLKR.......................
PDC-19aWGW51434.1_1207PaBOC    361  LGLVILANRNYPNAERVKIAYAILSGLEQQAKVPLKR.......................
PDC-19aWGW37666.1_6817PaBOC    361  LGLVILANRNYPNAERVKIAYAILSGLEQQAKVPLKR.......................
PDC-19aWGW59864.1_3660PaBOC    361  LGLVILANRNYPNAERVKIAYAILSGLEQQAKVPLKR.......................
PDC-19aWGX49838.1_877PaBOC     361  LGLVILANRNYPNAERVKIAYAILSGLEQQAKVPLKR.......................
PDC-19aWGX24308.1_877PaBOC     361  LGLVILANRNYPNAERVKIAYAILSGLEQQAKVPLKR.......................
PDC-19aWGX35552.1_5487PaBOC    361  LGLVILANRNYPNAERVKIAYAILSGLEQQAKVPLKR.......................
PDC-19aWKA37694.1_2446PaBOC    361  LGLVILANRNYPNAERVKIAYAILSGLEQQAKVPLKR.......................
PDC-19aAPB63370.1_616PaOC      361  LGLVILANRNYPNAERVKIAYAILSGLEQQAKVPLKR.......................
PDC-19aARI89529.1_870PaOC      361  LGLVILANRNYPNAERVKIAYAILSGLEQQAKVPLKR.......................
PDC-19aARI95964.1_871PaOC      361  LGLVILANRNYPNAERVKIAYAILSGLEQQAKVPLKR.......................
PDC-19aASA13594.1_977PaOC      361  LGLVILANRNYPNAERVKIAYAILSGLEQQAKVPLKR.......................
PDC-19aASA27558.1_962PaOC      361  LGLVILANRNYPNAERVKIAYAILSGLEQQAKVPLKR.......................
PDC-19aASD01901.1_971PaOC      361  LGLVILANRNYPNAERVKIAYAILSGLEQQAKVPLKR.......................
PDC-19aUAC92160.1_908PaOC      361  LGLVILANRNYPNAERVKIAYAILSGLEQQAKVPLKR.......................
PDC-19aUAC98839.1_942PaOC      361  LGLVILANRNYPNAERVKIAYAILSGLEQQAKVPLKR.......................
PDC-19aUTQ33604.1_830PaOC      361  LGLVILANRNYPNAERVKIAYAILSGLEQQAKVPLKR.......................
PDC-19aUOO58611.1_830PaOC      361  LGLVILANRNYPNAERVKIAYAILSGLEQQAKVPLKR.......................
PDC-19aWDA50910.1_883PaOC      361  LGLVILANRNYPNAERVKIAYAILSGLEQQAKVPLKR.......................
PDC-19aWGX30960.1_941PaOC      361  LGLVILANRNYPNAERVKIAYAILSGLEQQAKVPLKR.......................
PDC-19aWGX61448.1_5603PaOC     361  LGLVILANRNYPNAERVKIAYAILSGLEQQAKVPLKR.......................
PDC-19aWGX82118.1_941PaOC      361  LGLVILANRNYPNAERVKIAYAILSGLEQQAKVPLKR.......................
PDC-19aWIO49783.1_187PaOC      361  LGLVILANRNYPNAERVKIAYAILSGLEQQAKVPLKR.......................
PDC-24QZV43725.1_941PaBCP      361  LGLVILANRNYPNAERVKIAYAILSGLEQQAKVPLKR.......................
PDC-24QZV43725.1_941PaBOC      361  LGLVILANRNYPNAERVKIAYAILSGLEQQAKVPLKR.......................
PDC-24QDR06751.1_860PaOC       361  LGLVILANRNYPNAERVKIAYAILSGLEQQAKVPLKR.......................
PDC-24UGR36796.1_3412PaOC      361  LGLVILANRNYPNAERVKIAYAILSGLEQQAKVPLKR.......................
PDC-24UTQ39921.1_833PaOC       361  LGLVILANRNYPNAERVKIAYAILSGLEQQAKVPLKR.......................
PDC-137QKE94864.1_852PaOC      361  LGLVILANRNYPNAERVKIAYAILSGLEQQAKVPLKR.......................
PDC-147UGR30133.1_1950PaOC     361  LGLVILANRNYPNAERVKIAYAILSGLEQQGKVPLKR.......................
PDC-8AYY41152.1_5352PaBCP      361  LGLVILANRNYPNAERVKIAYAILSGLEQQGKVPLKR.......................
PDC-8QOE14337.1_211PaBCP       361  LGLVILANRNYPNAERVKIAYAILSGLEQQGKVPLKR.......................
PDC-8QPN15577.1_911PaBCP       361  LGLVILANRNYPNAERVKIAYAILSGLEQQGKVPLKR.......................
PDC-8QPN23993.1_908PaBCP       361  LGLVILANRNYPNAERVKIAYAILSGLEQQGKVPLKR.......................
PDC-8QUE64702.1_909PaBCP       361  LGLVILANRNYPNAERVKIAYAILSGLEQQGKVPLKR.......................
PDC-8QYA88773.1_2249PaBCP      361  LGLVILANRNYPNAERVKIAYAILSGLEQQGKVPLKR.......................
PDC-8QYF01720.1_5249PaBCP      361  LGLVILANRNYPNAERVKIAYAILSGLEQQGKVPLKR.......................
PDC-8QZU99504.1_908PaBCP       361  LGLVILANRNYPNAERVKIAYAILSGLEQQGKVPLKR.......................
PDC-8QZV05727.1_908PaBCP       361  LGLVILANRNYPNAERVKIAYAILSGLEQQGKVPLKR.......................
PDC-8QZV12028.1_910PaBCP       361  LGLVILANRNYPNAERVKIAYAILSGLEQQGKVPLKR.......................
PDC-8QZV18112.1_863PaBCP       361  LGLVILANRNYPNAERVKIAYAILSGLEQQGKVPLKR.......................
PDC-8QZV24387.1_912PaBCP       361  LGLVILANRNYPNAERVKIAYAILSGLEQQGKVPLKR.......................
PDC-8QZV68851.1_908PaBCP       361  LGLVILANRNYPNAERVKIAYAILSGLEQQGKVPLKR.......................
PDC-8QZV81338.1_870PaBCP       361  LGLVILANRNYPNAERVKIAYAILSGLEQQGKVPLKR.......................
PDC-8QZV87769.1_908PaBCP       361  LGLVILANRNYPNAERVKIAYAILSGLEQQGKVPLKR.......................
PDC-8QZV94048.1_908PaBCP       361  LGLVILANRNYPNAERVKIAYAILSGLEQQGKVPLKR.......................
PDC-8UXH53497.1_877PaBCP       361  LGLVILANRNYPNAERVKIAYAILSGLEQQGKVPLKR.......................
PDC-8UXH66521.1_877PaBCP       361  LGLVILANRNYPNAERVKIAYAILSGLEQQGKVPLKR.......................
PDC-8UXH70664.1_4987PaBCP      361  LGLVILANRNYPNAERVKIAYAILSGLEQQGKVPLKR.......................
PDC-8UUW81479.1_5273PaBCP      361  LGLVILANRNYPNAERVKIAYAILSGLEQQGKVPLKR.......................
PDC-8WDM07130.1_910PaBCP       361  LGLVILANRNYPNAERVKIAYAILSGLEQQGKVPLKR.......................
PDC-8WHM62936.1_909PaBCP       361  LGLVILANRNYPNAERVKIAYAILSGLEQQGKVPLKR.......................
PDC-8WHM69354.1_909PaBCP       361  LGLVILANRNYPNAERVKIAYAILSGLEQQGKVPLKR.......................
PDC-8WHM75768.1_909PaBCP       361  LGLVILANRNYPNAERVKIAYAILSGLEQQGKVPLKR.......................
PDC-8WHM84263.1_909PaBCP       361  LGLVILANRNYPNAERVKIAYAILSGLEQQGKVPLKR.......................
PDC-8AYY41152.1_5352PaBOC      361  LGLVILANRNYPNAERVKIAYAILSGLEQQGKVPLKR.......................
PDC-8QOE14337.1_211PaBOC       361  LGLVILANRNYPNAERVKIAYAILSGLEQQGKVPLKR.......................
PDC-8QPN15577.1_911PaBOC       361  LGLVILANRNYPNAERVKIAYAILSGLEQQGKVPLKR.......................
PDC-8QPN23993.1_908PaBOC       361  LGLVILANRNYPNAERVKIAYAILSGLEQQGKVPLKR.......................
PDC-8QUE64702.1_909PaBOC       361  LGLVILANRNYPNAERVKIAYAILSGLEQQGKVPLKR.......................
PDC-8QYA88773.1_2249PaBOC      361  LGLVILANRNYPNAERVKIAYAILSGLEQQGKVPLKR.......................
PDC-8QYF01720.1_5249PaBOC      361  LGLVILANRNYPNAERVKIAYAILSGLEQQGKVPLKR.......................
PDC-8QZU99504.1_908PaBOC       361  LGLVILANRNYPNAERVKIAYAILSGLEQQGKVPLKR.......................
PDC-8QZV05727.1_908PaBOC       361  LGLVILANRNYPNAERVKIAYAILSGLEQQGKVPLKR.......................
PDC-8QZV12028.1_910PaBOC       361  LGLVILANRNYPNAERVKIAYAILSGLEQQGKVPLKR.......................
PDC-8QZV18112.1_863PaBOC       361  LGLVILANRNYPNAERVKIAYAILSGLEQQGKVPLKR.......................
PDC-8QZV24387.1_912PaBOC       361  LGLVILANRNYPNAERVKIAYAILSGLEQQGKVPLKR.......................
PDC-8QZV68851.1_908PaBOC       361  LGLVILANRNYPNAERVKIAYAILSGLEQQGKVPLKR.......................
PDC-8QZV81338.1_870PaBOC       361  LGLVILANRNYPNAERVKIAYAILSGLEQQGKVPLKR.......................
PDC-8QZV87769.1_908PaBOC       361  LGLVILANRNYPNAERVKIAYAILSGLEQQGKVPLKR.......................
PDC-8QZV94048.1_908PaBOC       361  LGLVILANRNYPNAERVKIAYAILSGLEQQGKVPLKR.......................
PDC-8UXH53497.1_877PaBOC       361  LGLVILANRNYPNAERVKIAYAILSGLEQQGKVPLKR.......................
PDC-8UXH66521.1_877PaBOC       361  LGLVILANRNYPNAERVKIAYAILSGLEQQGKVPLKR.......................
PDC-8UXH70664.1_4987PaBOC      361  LGLVILANRNYPNAERVKIAYAILSGLEQQGKVPLKR.......................
PDC-8UUW81479.1_5273PaBOC      361  LGLVILANRNYPNAERVKIAYAILSGLEQQGKVPLKR.......................
PDC-8WDM07130.1_910PaBOC       361  LGLVILANRNYPNAERVKIAYAILSGLEQQGKVPLKR.......................
PDC-8WHM62936.1_909PaBOC       361  LGLVILANRNYPNAERVKIAYAILSGLEQQGKVPLKR.......................
PDC-8WHM69354.1_909PaBOC       361  LGLVILANRNYPNAERVKIAYAILSGLEQQGKVPLKR.......................
PDC-8WHM75768.1_909PaBOC       361  LGLVILANRNYPNAERVKIAYAILSGLEQQGKVPLKR.......................
PDC-8WHM84263.1_909PaBOC       361  LGLVILANRNYPNAERVKIAYAILSGLEQQGKVPLKR.......................
PDC-8AMA35328.1_919.PaOC      361  LGLVILANRNYPNAERVKIAYAILSGLEQQGKVPLKR.......................
PDC-8ANI06966.1_119PaOC       361  LGLVILANRNYPNAERVKIAYAILSGLEQQGKVPLKR.......................
PDC-8AYW70752.1_952PaOC       361  LGLVILANRNYPNAERVKIAYAILSGLEQQGKVPLKR.......................
PDC-8AYZ76972.1_2272PaOC      361  LGLVILANRNYPNAERVKIAYAILSGLEQQGKVPLKR.......................
PDC-8QKP85289.1_778PaOC       361  LGLVILANRNYPNAERVKIAYAILSGLEQQGKVPLKR.......................
PDC-8QKR30573.1_5806PaOC      361  LGLVILANRNYPNAERVKIAYAILSGLEQQGKVPLKR.......................
PDC-8QLF19879.1_939PaOC       361  LGLVILANRNYPNAERVKIAYAILSGLEQQGKVPLKR.......................
PDC-8QYE59452.1_5107PaOC      361  LGLVILANRNYPNAERVKIAYAILSGLEQQGKVPLKR.......................
PDC-8QZV98104.1_4956PaOC      361  LGLVILANRNYPNAERVKIAYAILSGLEQQGKVPLKR.......................
PDC-8UFK73541.1_1068PaOC      361  LGLVILANRNYPNAERVKIAYAILSGLEQQGKVPLKR.......................
PDC-8UGR33975.2_368PaOC       361  LGLVILANRNYPNAERVKIAYAILSGLEQQGKVPLKR.......................
PDC-8UTN26696.1_862PaOC       361  LGLVILANRNYPNAERVKIAYAILSGLEQQGKVPLKR.......................
PDC-8UTQ91314.1_910PaOC       361  LGLVILANRNYPNAERVKIAYAILSGLEQQGKVPLKR.......................
PDC-8UNK85299.1_868PaOC       361  LGLVILANRNYPNAERVKIAYAILSGLEQQGKVPLKR.......................
PDC-8UNK97662.1_866PaOC       361  LGLVILANRNYPNAERVKIAYAILSGLEQQGKVPLKR.......................
PDC-8USI83494.1_833PaOC       361  LGLVILANRNYPNAERVKIAYAILSGLEQQGKVPLKR.......................
PDC-8WAO16415.1_5521PaOC      361  LGLVILANRNYPNAERVKIAYAILSGLEQQGKVPLKR.......................
PDC-98QZD62144.1_4346PaBCP    361  LGLVILANRNYPNAERVKIAYAILSGLEQQGKVPLKR.......................
```

```
PDC-98QZH48390.1_4214PaBCP     361  LGLVILANRNYPNAERVKIAYAILSGLEQQGKVPLKR...................
PDC-98QZD62144.1_4346PaBOC     361  LGLVILANRNYPNAERVKIAYAILSGLEQQGKVPLKR...................
PDC-98QZH48390.1_4214PaBOC     361  LGLVILANRNYPNAERVKIAYAILSGLEQQGKVPLKR...................
PDC-98ALP56563.1_1203.PaOC     361  LGLVILANRNYPNAERVKIAYAILSGLEQQGKVPLKR...................
PDC-98UTL97623.1_830PaOC       361  LGLVILANRNYPNAERVKIAYAILSGLEQQGKVPLKR...................
PDC-457UTN40061.1_863PaOC      361  LGLVILANRNYPNAERVKIAYAILSGLEQQANVPLKR...................
PDC-23UXI89283.1_830PaOC       361  LGLVILANRNYPNAERVKIAYAILSGLEQQAKVPLKR...................
PDC-245AUA75408.1_866PaOC      361  LGLVILANRNYPNAERVKIAYAILSGLEQQAKVPLKR...................
PDC-31QZW06655.1_840PaBCP      361  LGLVILANRNYPNAERVKIAYAILSGLEQQGKVPLKR...................
PDC-31QZW06655.1_840PaBOC      361  LGLVILANRNYPNAERVKIAYAILSGLEQQGKVPLKR...................
PDC-31AXN27248.1_931PaOC       361  LGLVILANRNYPNAERVKIAYAILSGLEQQGKVPLKR...................
PDC-31QBC07904.1_3021PaOC      361  LGLVILANRNYPNAERVKIAYAILSGLEQQGKVPLKR...................
PDC-31UFM90275.1_905PaOC       361  LGLVILANRNYPNAERVKIAYAILSGLEQQGKVPLKR...................
PDC-31UFM98870.1_905PaOC       361  LGLVILANRNYPNAERVKIAYAILSGLEQQGKVPLKR...................
PDC-31UNT25139.1_901PaOC       361  LGLVILANRNYPNAERVKIAYAILSGLEQQGKVPLKR...................
PDC-31UWJ96607.1_4267PaOC      361  LGLVILANRNYPNAERVKIAYAILSGLEQQGKVPLKR...................
PDC-31WGX88300.1_823PaOC       361  LGLVILANRNYPNAERVKIAYAILSGLEQQGKVPLKR...................
PDC-31WHM08550.1_859PaOC       361  LGLVILANRNYPNAERVKIAYAILSGLEQQGKVPLKR...................
PDC-39AYK21377.1_872PaOC       361  LGLVILANRNYPNAERVKIAYAILSGLEQQAKVPLKR...................
PDC-39UJC21946.1_837PaOC       361  LGLVILANRNYPNAERVKIAYAILSGLEQQAKVPLKR...................
PDC-30UPL39337.1_928PaBCP      361  LGLVILANRNYPNAERVKIAYAILSGLEQQPKVPLKR...................
PDC-30UPL39337.1_928PaBOC      361  LGLVILANRNYPNAERVKIAYAILSGLEQQPKVPLKR...................
PDC-30UHL95932.1_5029PaOC      361  LGLVILANRNYPNAERVKIAYAILSGLEQQPKVPLKR...................
PDC-30UML99445.1_838PaOC       361  LGLVILANRNYPNAERVKIAYAILSGLEQQPKVPLKR...................
PDC-80WAJ79202.1_935PaOC       361  LGLVILANRNYPNAERVKIAYAILSGLEQQGKVPLKR...................
8SDL_1|Chain                   361  LGLVILANRNYPNAERVKIAYAILSGLEQQGKVPLKR...................
PDC-3UTN33167.1_589PaBCP       361  LGLVILANRNYPNAERVKIAYAILSGLEQQGKVPLKR...................
PDC-3WCI38158.1_984PaBCP       361  LGLVILANRNYPNAERVKIAYAILSGLEQQGKVPLKR...................
PDC-3WCI50909.1_867PaBCP       361  LGLVILANRNYPNAERVKIAYAILSGLEQQGKVPLKR...................
PDC-3WCI90621.1_961PaBCP       361  LGLVILANRNYPNAERVKIAYAILSGLEQQGKVPLKR...................
PDC-3WEO48793.1_832PaBCP       361  LGLVILANRNYPNAERVKIAYAILSGLEQQGKVPLKR...................
PDC-3CAI9907424.1_19963]PaBCP  361  LGLVILANRNYPNAERVKIAYAILSGLEQQGKVPLKR...................
PDC-3UTN33167.1_589PaBOC       361  LGLVILANRNYPNAERVKIAYAILSGLEQQGKVPLKR...................
PDC-3WCI38158.1_984PaBOC       361  LGLVILANRNYPNAERVKIAYAILSGLEQQGKVPLKR...................
PDC-3WCI50909.1_867PaBOC       361  LGLVILANRNYPNAERVKIAYAILSGLEQQGKVPLKR...................
PDC-3WCI90621.1_961PaBOC       361  LGLVILANRNYPNAERVKIAYAILSGLEQQGKVPLKR...................
PDC-3WEO48793.1_832PaBOC       361  LGLVILANRNYPNAERVKIAYAILSGLEQQGKVPLKR...................
PDC-3CAI9907424.1_19963]PaBOC  361  LGLVILANRNYPNAERVKIAYAILSGLEQQGKVPLKR...................
PDC-3AHA17954.1_839.PaOC       361  LGLVILANRNYPNAERVKIAYAILSGLEQQGKVPLKR...................
PDC-3ALE46683.1_840.PaOC       361  LGLVILANRNYPNAERVKIAYAILSGLEQQGKVPLKR...................
PDC-3ARC78082.1_863.PaOC       361  LGLVILANRNYPNAERVKIAYAILSGLEQQGKVPLKR...................
PDC-3ARG53386.1_5885PaOC       361  LGLVILANRNYPNAERVKIAYAILSGLEQQGKVPLKR...................
PDC-3WAC79950.1_4482PaOC       361  LGLVILANRNYPNAERVKIAYAILSGLEQQGKVPLKR...................
PDC-3WAE22012.1_4863PaOC       361  LGLVILANRNYPNAERVKIAYAILSGLEQQGKVPLKR...................
PDC-3WAE29433.1_777PaOC        361  LGLVILANRNYPNAERVKIAYAILSGLEQQGKVPLKR...................
PDC-3WAW42145.1_847PaOC        361  LGLVILANRNYPNAERVKIAYAILSGLEQQGKVPLKR...................
PDC-3WCI57477.1_911PaOC        361  LGLVILANRNYPNAERVKIAYAILSGLEQQGKVPLKR...................
PDC-3WCI77402.1_900PaOC        361  LGLVILANRNYPNAERVKIAYAILSGLEQQGKVPLKR...................
PDC-3WCI64002.1_913PaOC        361  LGLVILANRNYPNAERVKIAYAILSGLEQQGKVPLKR...................
PDC-3WCI83994.1_905PaOC        361  LGLVILANRNYPNAERVKIAYAILSGLEQQGKVPLKR...................
PDC-3WJM48814.1_842PaOC        361  LGLVILANRNYPNAERVKIAYAILSGLEQQGKVPLKR...................
PDC-374ANP60834.1_3741PaBCP    361  LGLVILANRNYPNAERVKIAYAILSGLEQQGKVPLKR...................
PDC-374AYZ86971.1_5950PaBCP    361  LGLVILANRNYPNAERVKIAYAILSGLEQQGKVPLKR...................
PDC-374QZV75115.1_843PaBCP     361  LGLVILANRNYPNAERVKIAYAILSGLEQQGKVPLKR...................
PDC-374QZW12711.1_843PaBCP     361  LGLVILANRNYPNAERVKIAYAILSGLEQQGKVPLKR...................
PDC-374UEG06685.1_907PaBCP     361  LGLVILANRNYPNAERVKIAYAILSGLEQQGKVPLKR...................
PDC-374UGW95802.1_984PaBCP     361  LGLVILANRNYPNAERVKIAYAILSGLEQQGKVPLKR...................
PDC-374WGW26222.1_839PaBCP     361  LGLVILANRNYPNAERVKIAYAILSGLEQQGKVPLKR...................
PDC-374ANP60834.1_3741PaBOC    361  LGLVILANRNYPNAERVKIAYAILSGLEQQGKVPLKR...................
PDC-374AYZ86971.1_5950PaBOC    361  LGLVILANRNYPNAERVKIAYAILSGLEQQGKVPLKR...................
PDC-374QZV75115.1_843PaBOC     361  LGLVILANRNYPNAERVKIAYAILSGLEQQGKVPLKR...................
PDC-374QZW12711.1_843PaBOC     361  LGLVILANRNYPNAERVKIAYAILSGLEQQGKVPLKR...................
PDC-374UEG06685.1_907PaBOC     361  LGLVILANRNYPNAERVKIAYAILSGLEQQGKVPLKR...................
PDC-374UGW95802.1_984PaBOC     361  LGLVILANRNYPNAERVKIAYAILSGLEQQGKVPLKR...................
PDC-374WGW26222.1_839PaBOC     361  LGLVILANRNYPNAERVKIAYAILSGLEQQGKVPLKR...................
PDC-374AOP56366.1_851PaOC      361  LGLVILANRNYPNAERVKIAYAILSGLEQQGKVPLKR...................
PDC-374AVE31445.1_873.PaOC     361  LGLVILANRNYPNAERVKIAYAILSGLEQQGKVPLKR...................
PDC-374QAS71510.1_873PaOC      361  LGLVILANRNYPNAERVKIAYAILSGLEQQGKVPLKR...................
PDC-374QDD38027.1_4463PaOC     361  LGLVILANRNYPNAERVKIAYAILSGLEQQGKVPLKR...................
PDC-374QKF05339.1_5830PaOC     361  LGLVILANRNYPNAERVKIAYAILSGLEQQGKVPLKR...................
PDC-374QKK83983.1_853PaOC      361  LGLVILANRNYPNAERVKIAYAILSGLEQQGKVPLKR...................
PDC-374QKS41466.1_4199PaOC     361  LGLVILANRNYPNAERVKIAYAILSGLEQQGKVPLKR...................
PDC-374QPZ80284.1_873PaOC      361  LGLVILANRNYPNAERVKIAYAILSGLEQQGKVPLKR...................
PDC-374QQW07466.1_922PaOC      361  LGLVILANRNYPNAERVKIAYAILSGLEQQGKVPLKR...................
PDC-374QZU93305.1_843PaOC      361  LGLVILANRNYPNAERVKIAYAILSGLEQQGKVPLKR...................
PDC-374UAC85653.1_934PaOC      361  LGLVILANRNYPNAERVKIAYAILSGLEQQGKVPLKR...................
PDC-374UGR20250.1_3485PaOC     361  LGLVILANRNYPNAERVKIAYAILSGLEQQGKVPLKR...................
PDC-374UIU12727.1_851PaOC      361  LGLVILANRNYPNAERVKIAYAILSGLEQQGKVPLKR...................
PDC-374UJC03215.1_956PaOC      361  LGLVILANRNYPNAERVKIAYAILSGLEQQGKVPLKR...................
PDC-374UJC09425.1_959PaOC      361  LGLVILANRNYPNAERVKIAYAILSGLEQQGKVPLKR...................
PDC-374UJF57976.1_829PaOC      361  LGLVILANRNYPNAERVKIAYAILSGLEQQGKVPLKR...................
PDC-374UJF63613.1_829PaOC      361  LGLVILANRNYPNAERVKIAYAILSGLEQQGKVPLKR...................
PDC-374UJF68041.1_5361PaOC     361  LGLVILANRNYPNAERVKIAYAILSGLEQQGKVPLKR...................
PDC-374UJF74874.1_829PaOC      361  LGLVILANRNYPNAERVKIAYAILSGLEQQGKVPLKR...................
PDC-374UJF80517.1_829PaOC      361  LGLVILANRNYPNAERVKIAYAILSGLEQQGKVPLKR...................
PDC-374UJF86151.1_829PaOC      361  LGLVILANRNYPNAERVKIAYAILSGLEQQGKVPLKR...................
PDC-374UJF90590.1_5359PaOC     361  LGLVILANRNYPNAERVKIAYAILSGLEQQGKVPLKR...................
PDC-374UJF97432.1_830PaOC      361  LGLVILANRNYPNAERVKIAYAILSGLEQQGKVPLKR...................
PDC-374UJG01877.1_5359PaOC     361  LGLVILANRNYPNAERVKIAYAILSGLEQQGKVPLKR...................
PDC-374UJG08704.1_829PaOC      361  LGLVILANRNYPNAERVKIAYAILSGLEQQGKVPLKR...................
PDC-374UJG14426.1_829PaOC      361  LGLVILANRNYPNAERVKIAYAILSGLEQQGKVPLKR...................
PDC-374UJG20129.1_829PaOC      361  LGLVILANRNYPNAERVKIAYAILSGLEQQGKVPLKR...................
PDC-374UJG25828.1_829PaOC      361  LGLVILANRNYPNAERVKIAYAILSGLEQQGKVPLKR...................
PDC-374UJG31693.1_884PaOC      361  LGLVILANRNYPNAERVKIAYAILSGLEQQGKVPLKR...................
PDC-374UJG36239.1_5358PaOC     361  LGLVILANRNYPNAERVKIAYAILSGLEQQGKVPLKR...................
PDC-374UGR51117.1_68PaOC       361  LGLVILANRNYPNAERVKIAYAILSGLEQQGKVPLKR...................
PDC-374UON75698.1_5570PaOC     361  LGLVILANRNYPNAERVKIAYAILSGLEQQGKVPLKR...................
PDC-374UPG04116.1_3174PaOC     361  LGLVILANRNYPNAERVKIAYAILSGLEQQGKVPLKR...................
PDC-374UPZ07602.1_2096PaOC     361  LGLVILANRNYPNAERVKIAYAILSGLEQQGKVPLKR...................
PDC-374URM55875.1_5937PaOC     361  LGLVILANRNYPNAERVKIAYAILSGLEQQGKVPLKR...................
PDC-374USV13808.1_3922PaOC     361  LGLVILANRNYPNAERVKIAYAILSGLEQQGKVPLKR...................
PDC-374USV19196.1_4087PaOC     361  LGLVILANRNYPNAERVKIAYAILSGLEQQGKVPLKR...................
PDC-374USX84542.1_851PaOC      361  LGLVILANRNYPNAERVKIAYAILSGLEQQGKVPLKR...................
```

```
PDC-374USX78872.1_851PaOC        361  LGLVILANRNYPNAERVKIAYAILSGLEQQGKVPLKR..................
PDC-374UUJ85605.1_1747PaOC       361  LGLVILANRNYPNAERVKIAYAILSGLEQQGKVPLKR..................
PDC-374UVS71982.1_4220PaOC       361  LGLVILANRNYPNAERVKIAYAILSGLEQQGKVPLKR..................
PDC-374UWI85046.1_4210PaOC       361  LGLVILANRNYPNAERVKIAYAILSGLEQQGKVPLKR..................
PDC-374UWI92968.1_851PaOC        361  LGLVILANRNYPNAERVKIAYAILSGLEQQGKVPLKR..................
PDC-374UXA22714.1_4203PaOC       361  LGLVILANRNYPNAERVKIAYAILSGLEQQGKVPLKR..................
PDC-374UXA30656.1_926PaOC        361  LGLVILANRNYPNAERVKIAYAILSGLEQQGKVPLKR..................
PDC-374UXA44988.1_926PaOC        361  LGLVILANRNYPNAERVKIAYAILSGLEQQGKVPLKR..................
PDC-374UXN39470.1_4222PaOC       361  LGLVILANRNYPNAERVKIAYAILSGLEQQGKVPLKR..................
PDC-374UZA87382.1_4980PaOC       361  LGLVILANRNYPNAERVKIAYAILSGLEQQGKVPLKR..................
PDC-374WBM66401.1_846PaOC        361  LGLVILANRNYPNAERVKIAYAILSGLEQQGKVPLKR..................
PDC-374WGJ75339.1_925PaOC        361  LGLVILANRNYPNAERVKIAYAILSGLEQQGKVPLKR..................
PDC-374WGW87865.1_837PaOC        361  LGLVILANRNYPNAERVKIAYAILSGLEQQGKVPLKR..................
PDC-103QES80695.1_910PaOC        361  LGLVILANRNYPNAERVKIAYAILSGLEQQGKVPLKR..................
PDC-103QPV53905.1_6224PaOC       361  LGLVILANRNYPNAERVKIAYAILSGLEQQGKVPLKR..................
PDC-35AVR81260.1_945.PaOC        361  LGLVILANRNYPNAERVKIAYAILSGLEQQAKVPLKR..................
PDC-35QJA35930.1_977PaOC         361  LGLVILANRNYPNAERVKIAYAILSGLEQQAKVPLKR..................
PDC-35AYZ46521.1_3210PaOC        361  LGLVILANRNYPNAERVKIAYAILSGLEQQAKVPLKR..................
PDC-35AYZ59004.1_3976PaOC        361  LGLVILANRNYPNAERVKIAYAILSGLEQQAKVPLKR..................
PDC-35QDR21260.1_3098PaOC        361  LGLVILANRNYPNAERVKIAYAILSGLEQQAKVPLKR..................
PDC-35QEK40749.1_862PaOC         361  LGLVILANRNYPNAERVKIAYAILSGLEQQAKVPLKR..................
PDC-35AUB00033.3_866PaOC         361  LGLVILANRNYPNAERVKIAYAILSGLEQQAKVPLKR..................
PDC-35QPP34775.1_1311PaOC        361  LGLVILANRNYPNAERVKIAYAILSGLEQQAKVPLKR..................
PDC-35QLF38648.1_891PaOC         361  LGLVILANRNYPNAERVKIAYAILSGLEQQAKVPLKR..................
PDC-35QLF14474.1_2055PaOC        361  LGLVILANRNYPNAERVKIAYAILSGLEQQAKVPLKR..................
PDC-35QQV60864.1_5728PaOC        361  LGLVILANRNYPNAERVKIAYAILSGLEQQAKVPLKR..................
PDC-35QQW01132.1_862PaOC         361  LGLVILANRNYPNAERVKIAYAILSGLEQQAKVPLKR..................
PDC-35QQW17992.1_5232PaOC        361  LGLVILANRNYPNAERVKIAYAILSGLEQQAKVPLKR..................
PDC-35QQW23330.1_862PaOC         361  LGLVILANRNYPNAERVKIAYAILSGLEQQAKVPLKR..................
PDC-35QZE42332.1_862PaOC         361  LGLVILANRNYPNAERVKIAYAILSGLEQQAKVPLKR..................
PDC-35UFM83790.1_826PaOC         361  LGLVILANRNYPNAERVKIAYAILSGLEQQAKVPLKR..................
PDC-35UJC15714.1_892PaOC         361  LGLVILANRNYPNAERVKIAYAILSGLEQQAKVPLKR..................
PDC-35UTR21121.1_931PaOC         361  LGLVILANRNYPNAERVKIAYAILSGLEQQAKVPLKR..................
PDC-35UUP09555.1_853PaOC         361  LGLVILANRNYPNAERVKIAYAILSGLEQQAKVPLKR..................
PDC-35UWG56199.1_885PaOC         361  LGLVILANRNYPNAERVKIAYAILSGLEQQAKVPLKR..................
PDC-43UNT30829.1_824PaOC         361  LGLVILANRNYPNAERVKIAYAILSGLEQQGKVPLKR..................
PDC-45ALZ89573.1_855.PaOC        361  LGLVILANRNYPNAERVKIAYAILSGLEQQGKVPLKR..................
PDC-45QKL11315.1_849PaOC         361  LGLVILANRNYPNAERVKIAYAILSGLEQQGKVPLKR..................
PDC-45QQV94504.1_850PaOC         361  LGLVILANRNYPNAERVKIAYAILSGLEQQGKVPLKR..................
PDC-162UJB98759.1_2776PaOC       361  LGLVILANRNYPNAERVKIAYAILSGLEQQGKVPLKR..................
PDC-123QYE90948.1_3103PaOC       361  LGLVILANRNYPNAERVKIAYAILSGLEQQGKVPLKR..................
PDC-5UXH60990.1_2046PaBCP        361  LGLVILANRNYPNAERVKIAYAILSGLEQQGKVPLKR..................
PDC-5WBA37364.1_905PaBCP         361  LGLVILANRNYPNAERVKIAYAILSGLEQQGKVPLKR..................
PDC-5WGJ68312.1_842PaBCP         361  LGLVILANRNYPNAERVKIAYAILSGLEQQGKVPLKR..................
PDC-5WGT14845.1_882PaBCP         361  LGLVILANRNYPNAERVKIAYAILSGLEQQGKVPLKR..................
PDC-5WGX69730.1_872PaBCP         361  LGLVILANRNYPNAERVKIAYAILSGLEQQGKVPLKR..................
PDC-5CAI9893238.1_8405]PaBCP     361  LGLVILANRNYPNAERVKIAYAILSGLEQQGKVPLKR..................
PDC-5CAI9905252.1_60335]PaBCP    361  LGLVILANRNYPNAERVKIAYAILSGLEQQGKVPLKR..................
PDC-5UXH60990.1_2046PaBOC        361  LGLVILANRNYPNAERVKIAYAILSGLEQQGKVPLKR..................
PDC-5WBA37364.1_905PaBOC         361  LGLVILANRNYPNAERVKIAYAILSGLEQQGKVPLKR..................
PDC-5WGJ68312.1_842PaBOC         361  LGLVILANRNYPNAERVKIAYAILSGLEQQGKVPLKR..................
PDC-5WGT14845.1_882PaBOC         361  LGLVILANRNYPNAERVKIAYAILSGLEQQGKVPLKR..................
PDC-5WGX69730.1_872PaBOC         361  LGLVILANRNYPNAERVKIAYAILSGLEQQGKVPLKR..................
PDC-5CAI9893238.1_8405]PaBOC     361  LGLVILANRNYPNAERVKIAYAILSGLEQQGKVPLKR..................
PDC-5CAI9905252.1_60335]PaBOC    361  LGLVILANRNYPNAERVKIAYAILSGLEQQGKVPLKR..................
PDC-5ALU48309.1_2211.PaOC        361  LGLVILANRNYPNAERVKIAYAILSGLEQQGKVPLKR..................
PDC-5AMX89317.1_4085.PaOC        361  LGLVILANRNYPNAERVKIAYAILSGLEQQGKVPLKR..................
PDC-5ATH01452.1_827PaOC          361  LGLVILANRNYPNAERVKIAYAILSGLEQQGKVPLKR..................
PDC-5UTN13111.1_830PaOC          361  LGLVILANRNYPNAERVKIAYAILSGLEQQGKVPLKR..................
PDC-5UUH88148.1_851PaOC          361  LGLVILANRNYPNAERVKIAYAILSGLEQQGKVPLKR..................
PDC-5UUH98459.1_806PaOC          361  LGLVILANRNYPNAERVKIAYAILSGLEQQGKVPLKR..................
PDC-5UVH93148.1_837PaOC          361  LGLVILANRNYPNAERVKIAYAILSGLEQQGKVPLKR..................
PDC-5UWU62523.1_171PaOC          361  LGLVILANRNYPNAERVKIAYAILSGLEQQGKVPLKR..................
PDC-5WDZ01346.1_910PaOC          361  LGLVILANRNYPNAERVKIAYAILSGLEQQGKVPLKR..................
PDC-5WEO42644.1_846PaOC          361  LGLVILANRNYPNAERVKIAYAILSGLEQQGKVPLKR..................
PDC-5WGW99708.1_849PaOC          361  LGLVILANRNYPNAERVKIAYAILSGLEQQGKVPLKR..................
PDC-5WJM42992.1_848PaOC          361  LGLVILANRNYPNAERVKIAYAILSGLEQQGKVPLKR..................
PDC-60QWY08308.1_854PaBCP        361  LGLVVLANRNYPNAERVKIAYAILSGLEQQGKVPLKR..................
PDC-60QZV50082.1_838PaBCP        361  LGLVVLANRNYPNAERVKIAYAILSGLEQQGKVPLKR..................
PDC-60QWY08308.1_854PaBOC        361  LGLVVLANRNYPNAERVKIAYAILSGLEQQGKVPLKR..................
PDC-60QZV50082.1_838PaBOC        361  LGLVVLANRNYPNAERVKIAYAILSGLEQQGKVPLKR..................
PDC-60QPZ60674.1_902PaOC         361  LGLVVLANRNYPNAERVKIAYAILSGLEQQGKVPLKR..................
PDC-60WAJ92260.1_847PaOC         361  LGLVVLANRNYPNAERVKIAYAILSGLEQQGKVPLKR..................
PDC-60WAJ98065.1_847PaOC         361  LGLVVLANRNYPNAERVKIAYAILSGLEQQGKVPLKR..................
PDC-15UNL10022.1_837PaBCP        361  LGLVILANRNYPNAERVKIAYAILSGLEQQGKVPLKR..................
PDC-15UNL10022.1_837PaBOC        361  LGLVILANRNYPNAERVKIAYAILSGLEQQGKVPLKR..................
PDC-15AOT38867.1_3370PaOC        361  LGLVILANRNYPNAERVKIAYAILSGLEQQGKVPLKR..................
PDC-15ALZ27705.1_859PaOC         361  LGLVILANRNYPNAERVKIAYAILSGLEQQGKVPLKR..................
PDC-15AXC19915.1_1529PaOC        361  LGLVILANRNYPNAERVKIAYAILSGLEQQGKVPLKR..................
PDC-15UTP69334.1_895PaOC         361  LGLVILANRNYPNAERVKIAYAILSGLEQQGKVPLKR..................
PDC-15UNK91447.1_837PaOC         361  LGLVILANRNYPNAERVKIAYAILSGLEQQGKVPLKR..................
PDC-15UNL03855.1_837PaOC         361  LGLVILANRNYPNAERVKIAYAILSGLEQQGKVPLKR..................
PDC-28UYM61272.1_6539PaOC        361  LGLVILANRNYPNAERVKIAYAILSGLEQQAKVPLKR..................
PDC-22QPZ72804.1_848PaOC         361  LGLVILANRNYPNAERVKIAYAILSGLEQQAKVPLKR..................
PDC-22QQM08170.1_4816PaOC        361  LGLVILANRNYPNAERVKIAYAILSGLEQQAKVPLKR..................
PDC-97QKR35301.1_4456PaOC        361  LGLVILANRNYPNAERVKIAYAILSGLEQQGKVPLKR..................
PDC-51WGV68048.1_897PaOC         361  LGLVILANRNYPNAERVKIAYAILSGLEQQGKVPLKR..................
PDC-1HW06_09835_867PaOC          361  LGLVILANRNYPNAERVKIAYAILSGLEQQGKVPLKR.SALARATERAARDAGPGY
PDC-151QQV49515.1_805PaOC        361  VGLVILANRNYPNAERVKIAYAILSGLEQQAKVPLVR..................
consensus>70                          lGLV!LANRNYPNAERVKIAYAILSGLEQQ.kVPLkR..................
                         acc
```
