# Supplementary material for: AmpC β-lactamases: A key to antibiotic resistance in ESKAPE pathogens
Source: Cell Surf. 2025 Sep 22;14:100154. doi: 10.1016/j.tcsw.2025.100154 (PMC12528871; doi:10.1016/j.tcsw.2025.100154)
Supplement: Supplementary Table 1 — Origin (Natural/Acquired) of Ambler Class C Beta-lactamases. [file mmc6.docx]

**Table S1:** Origin (Natural/Acquired) of Ambler Class C Beta-lactamases

| **Ambler class C beta-lactamases** | **GenPeptID** | **Natural (N) or Acquired (A)** |
| --- | --- | --- |
| **ACC** |  |  |
| ACC-1 | CDI45198 | A |
| ACC-1a | AAF86692 | N (Hafnia alvei) |
| ACC-1b | AAF86694 | N (Hafnia alvei) |
| ACC-1c | AAF86698 | N (Hafnia alvei) |
| ACC-1d | EFV39045 | N (Hafnia alvei) |
| ACC-2 | AAF86691 | N (Hafnia alvei) |
| ACC-4 | AIT76084 | A |
| ACC-6 | --- | #N/A |
| ACC-7 | ATJ25947 | N (Hafnia alvei N11-01820) |
| ACC-8 | QNG62072 | A |
| ACC-P1 | AAF86697 | N (Hafnia alvei HA-7) |
| ACC-P2 | CCK86740 | N (Hafnia alvei) |
| ACC-P3 | AAF86695 | N (Hafnia alvei HA-5) |
| **ACT** |  |  |
| ACT-9 | --- | #N/A |
| ACT-1 | AAC45086 | A |
| ACT-2 | CAJ28994 | N (Enterobacter asburiae) |
| ACT-3 | ABL67017 | A |
| ACT-4 | ABZ81086 | N (Enterobacter asburiae) |
| ACT-5 | ACJ05689 | A |
| ACT-6 | ACJ05686 | A |
| ACT-7 | ACJ05688 | A |
| ACT-8 | CBI75448 | A |
| ACT-10 | AEV91214 | A |
| ACT-12 | AFU25650 | N (Enterobacter cloacae) |
| ACT-13 | CCK86741 | N (Enterobacter asburiae) |
| ACT-14 | AFU25647 | N (Enterobacter cloacae) |
| ACT-15 | AFU25653 | N (Enterobacter cloacae) |
| ACT-16 | AAQ73498 | N (Enterobacter cloacae) |
| ACT-17 | KTQ57147 | A |
| ACT-18 | AHM76777 | N (Enterobacter cloacae) |
| ACT-19 | AHM76779 | N (Enterobacter cloacae) |
| ACT-20 | AHA80105 | N (Enterobacter hormaechei) |
| ACT-21 | AHA80106 | N (Enterobacter hormaechei) |
| ACT-22 | AHM76774 | N (Enterobacter cloacae) |
| ACT-23 | AGU38146 | N (Enterobacter cloacae) |
| ACT-24 | AHL39336 | N (Enterobacter cloacae) |
| ACT-25 | AHL39338 | N (Enterobacter cloacae) |
| ACT-27 | AHL39340 | N (Enterobacter cloacae) |
| ACT-28 | AHL39333 | N (Enterobacter cloacae) |
| ACT-29 | AIT76085 | N (Enterobacter asburiae) |
| ACT-30 | AIT76086 | N (Enterobacter cloacae) |
| ACT-31 | AIT76096 | N (Enterobacter cloacae) |
| ACT-32 | AIT76088 | N (Enterobacter cloacae) |
| ACT-33 | AIT76087 | N (Enterobacter cloacae) |
| ACT-34 | CDP41983 | N (Enterobacter cloacae) |
| ACT-35 | BAP68758 | N (Enterobacter cloacae) |
| ACT-36 | AJG06170 | N (Enterobacter cloacae) |
| ACT-37 | AJG06172 | N (Enterobacter cloacae) |
| ACT-38 | AKS43590 | A |
| ACT-39 | AMP42993 | N (Enterobacter cloacae) |
| ACT-40 | ANE37563 | N (Enterobacter cloacae) |
| ACT-41 | ANE37564 | N (Enterobacter cloacae) |
| ACT-42 | AIG20026 | N (Enterobacter cloacae) |
| ACT-43 | ANE37566 | N (Enterobacter cloacae) |
| ACT-44 | AIG20040 | N (Enterobacter cloacae) |
| ACT-45 | ABF29680 | A and N (Enterobacter cloacae) |
| ACT-46 | ANE37570 | N (Enterobacter cloacae) |
| ACT-47 | ANE37574 | N (Enterobacter hormaechei) |
| ACT-48 | ANE37562 | N (Enterobacter asburiae) |
| ACT-49 | AIG20030 | N (Enterobacter cloacae) |
| ACT-50 | ANE37572 | N (Enterobacter cloacae complex sp. 35699) |
| ACT-51 | ANE37573 | N (Enterobacter kobei) |
| ACT-52 | AIX56560 | N (Enterobacter cloacae) |
| ACT-53 | ASP63120 | N (Enterobacter kobei) |
| ACT-54 | ATJ25948 | N (Enterobacter cloacae N11-01141) |
| ACT-55 | AIG20016 | N (Enterobacter cloacae) |
| ACT-56 | AIG20020 | N (Enterobacter cloacae) |
| ACT-57 | AWY87653 | N (Enterobacter asburiae) |
| ACT-58 | AWY87654 | N (Enterobacter cloacae complex sp.) |
| ACT-59 | AWY87655 | N (Enterobacter hormaechei subsp. oharae) |
| ACT-60 | AWY87656 | N (Enterobacter hormaechei subsp. oharae) |
| ACT-61 | AWY87658 | N (Enterobacter hormaechei subsp. oharae) |
| ACT-62 | AWY87659 | N (Enterobacter cloacae complex sp.) |
| ACT-63 | AIG20033 | N (Enterobacter cloacae) |
| ACT-64 | AWY87661 | N (Enterobacter kobei) |
| ACT-65 | AIG20018 | N (Enterobacter cloacae) |
| ACT-66 | QBF38561 | N (Enterobacter hormaechei subsp. hoffmannii) |
| ACT-67 | AMJ35012 | N (uncultured bacterium) |
| ACT-68 | QBG64270 | N (Enterobacter asburiae) |
| ACT-69 | KLW38111 | N (Enterobacter sp. MGH119) |
| ACT-70 | KLQ84547 | N (Enterobacter hormaechei subsp. steigerwaltii) |
| ACT-72 | KJX22324 | N (Enterobacter hormaechei subsp. xiangfangensis) |
| ACT-73 | AIG20051 | N (Enterobacter cloacae 5705) |
| ACT-74 | KTJ29119 | N (Enterobacter hormaechei subsp. steigerwaltii SMART_448) |
| ACT-75 | KJM62872 | N (Enterobacter hormaechei subsp. xiangfangensis 39373) |
| ACT-76 | RUO01826 | N (Enterobacter bugandensis WCHEB045359) |
| ACT-77 | KZP65494 | N (Enterobacter cloacae complex sp. GN04787) |
| ACT-78 | QHD57400 | N (Enterobacter cloacae) |
| ACT-79 | QHD57401 | N (Enterobacter cloacae) |
| ACT-80 | QHD57402 | N (Enterobacter cloacae) |
| ACT-81 | QHD57403 | N (Enterobacter cloacae) |
| ACT-82 | QHD57404 | N (Enterobacter cloacae) |
| ACT-83 | QIC04101 | N (Enterobacter hormaechei) |
| ACT-84 | QIH98458 | A and N (Enterobacter hormaechei) |
| ACT-85 | MRN75760 | N (Enterobacter hormaechei) |
| ACT-86 | MPV43777 | N (Enterobacter hormaechei) |
| ACT-87 | AYL04658 | N (Enterobacter kobei) |
| ACT-88 | QTV32571 | N (Enterobacter ludwigii) |
| ACT-89 | CAA30257 | N (Enterobacter cloacae P99) |
| ACT-90 | AIG20025 | N (Enterobacter cloacae 4358) |
| ACT-91 | UEG31054 | N (Enterobacter cloacae) |
| ACT-92 | UEG31055 | N (Enterobacter cloacae) |
| ACT-93 | ALL15984 | N (Enterobacter sp. E20) |
| ACT-94 | MCI9499071 | N (Enterobacter hormaechei subsp. steigerwaltii) |
| ACT-95 | BBV69349 | N (Enterobacter kobei) |
| ACT-96 | UHK14141 | N (Enterobacter cloacae complex RIVM_C008762) |
| ACT-97 | UHK14144 | N (Enterobacter cloacae complex RIVM_C008935) |
| ACT-98 | MCE1260927 | N (Enterobacter kobei) |
| ACT-99 | MBG0579301 | N (Enterobacter kobei) |
| ACT-100 | UHK14157 | N (Enterobacter cloacae complex RIVM_C015464) |
| ACT-101 | HBL7320680 | N (Enterobacter kobei) |
| ACT-102 | BBV79841 | N (Enterobacter kobei) |
| ACT-103 | SAF49753 | N (Enterobacter kobei) |
| ACT-104 | UHK14164 | N (Enterobacter cloacae complex) |
| ACT-105 | UMO60338 | N (Enterobacter asburiae) |
| ACT-106 | EHD3702606 | N (Enterobacter hormaechei) |
| ACT-107 | UNN26045 | N (Enterobacter huaxiensis) |
| ACT-108 | EHD4407246 | N (Enterobacter hormaechei) |
| ACT-109 | CZV56298 | N (Enterobacter ludwigii) |
| ACT-110 | BDT38917 | N (Enterobacter kobei) |
| ACT-112 | WEG44939 | N (Enterobacter ludwigii) |
| ACT-113 | WEG44940 | N (Enterobacter cloacae) |
| ACT-114 | WP_180255442 | N (Enterobacter hormaechei) |
| ACT-115 | AXQ35263 | N (Enterobacter hormaechei) |
| ACT-116 | AOL14308 | N (Enterobacter sp. HK169) |
| ACT-117 | WEY36195 | N (Enterobacter cloacae) |
| ACT-118 | WEY36196 | N (Enterobacter cloacae) |
| ACT-119 | WEY36198 | N (Enterobacter cloacae) |
| ACT-120 | WLO97150 | N (Enterobacter hormaechei subsp. hormaechei) |
| ACT-121 | WLO97151 | N (Enterobacter hormaechei subsp. hormaechei) |
| ACT-122 | HAV1779570 | N (Enterobacter hormaechei subsp. steigerwaltii) |
| ACT-123 | HBL5329718 | N (Enterobacter hormaechei) |
| ACT-124 | WLO97154 | N (Enterobacter hormaechei subsp. steigerwaltii) |
| ACT-C190 | BAA07922 | N (Enterobacter cloacae GC1) |
| ACT-C191 | CAC08444 | N (Enterobacter cloacae CHE) |
| ACT-C192 | AAL05857 | N (Enterobacter cloacae K9973) |
| ACT-C194 | CAA30879 | N (Enterobacter cloacae MHN1) |
| ACT-C195 | BAA32077 | N (Enterobacter cloacae GN747) |
| ACT-C196 | AAL05855 | N (Enterobacter cloacae K99230) |
| ACT-C197 | CAC08446 | N (Enterobacter cloacae OUDhyp) |
| ACT-C198 | AAM11666 | N (Enterobacter cancerogenus) |
| **ADC** |  |  |
| ADC-1 | AAR00515 | N (Acinetobacter baumannii) |
| ADC-2 | ENU77769 | A and N (Acinetobacter baumannii) |
| ADC-3 | AAO59456 | N (Acinetobacter baumannii) |
| ADC-4 | AAO59457 | N (Acinetobacter baumannii) |
| ADC-5 | AIS07280 | N (Acinetobacter baumannii) |
| ADC-6 | AAR13676 | N (Acinetobacter baumannii) |
| ADC-7 | AAT70411 | N (Acinetobacter baumannii) |
| ADC-8 | CAL25116 | N (Acinetobacter baylyi) |
| ADC-10 | ABI18382 | N (Acinetobacter baumannii) |
| ADC-11 | AFA35105 | N (Acinetobacter baumannii) |
| ADC-12 | CAK95249 | N (Acinetobacter pittii) |
| ADC-13 | CAK95248 | N (Acinetobacter pittii) |
| ADC-14 | CAK95247 | N (Acinetobacter pittii) |
| ADC-15 | CAK95246 | N (Acinetobacter pittii) |
| ADC-16 | CAK95245 | N (Acinetobacter pittii) |
| ADC-17 | CAK95244 | N (Acinetobacter pittii) |
| ADC-18 | KCY52905 | N (Acinetobacter baumannii) |
| ADC-19 | CAK95242 | N (Acinetobacter pittii) |
| ADC-20 | CAK95241 | N (Acinetobacter pittii) |
| ADC-21 | CAK95240 | N (Acinetobacter pittii) |
| ADC-22 | CAK95239 | N (Acinetobacter pittii) |
| ADC-23 | CAK95238 | N (Acinetobacter pittii) |
| ADC-24 | CAK95237 | N (Acinetobacter baumannii) |
| ADC-25 | ABK34773 | N (Acinetobacter baumannii) |
| ADC-26 | ADG46043 | N (Acinetobacter baumannii) |
| ADC-29 | ACC66195 | N (Acinetobacter baumannii) |
| ADC-30 | ABU63976 | N (Acinetobacter baumannii) |
| ADC-31 | ADX04315 | N (Acinetobacter baumannii) |
| ADC-32 | KMV27182 | N (Acinetobacter baumannii) |
| ADC-33 | ABV21800 | N (Acinetobacter baumannii) |
| ADC-38 | ACC95873 | N (Acinetobacter baumannii) |
| ADC-39 | ACC95874 | N (Acinetobacter baumannii) |
| ADC-41 | ACN62070 | N (Acinetobacter pittii) |
| ADC-42 | ACN62071 | N (Acinetobacter pittii) |
| ADC-43 | ACN62072 | N (Acinetobacter pittii) |
| ADC-44 | ACN62073 | N (Acinetobacter pittii) |
| ADC-50 | ADG46038 | N (Acinetobacter baumannii) |
| ADC-51 | ADG46040 | N (Acinetobacter baumannii) |
| ADC-52 | ADG46042 | N (Acinetobacter baumannii) |
| ADC-53 | ADG46044 | N (Acinetobacter baumannii) |
| ADC-54 | ADK35761 | N (Acinetobacter baumannii) |
| ADC-56 | AEG47700 | N (Acinetobacter baumannii) |
| ADC-57 | ADO51072 | N (Acinetobacter baumannii) |
| ADC-58 | AFG25594 | N (Acinetobacter baumannii) |
| ADC-59 | AFG25595 | N (Acinetobacter baumannii) |
| ADC-60 | AFH53180 | N (Acinetobacter baumannii) |
| ADC-61 | AFI56570 | N (Acinetobacter baumannii) |
| ADC-62 | AFK24475 | N (Acinetobacter baumannii) |
| ADC-63 | AFM80040 | N (Acinetobacter baumannii) |
| ADC-64 | --- | #N/A |
| ADC-65 | AFP73417 | N (Acinetobacter baumannii) |
| ADC-66 | AFP73418 | N (Acinetobacter baumannii) |
| ADC-67 | ABQ45322 | N (Acinetobacter baumannii) |
| ADC-68 | AGL39360 | N (Acinetobacter baumannii) |
| ADC-69 | AGV39975 | N (Acinetobacter baumannii) |
| ADC-70 | AIL90389 | N (Acinetobacter calcoaceticus) |
| ADC-73 | AIA51109 | N (Acinetobacter baumannii) |
| ADC-74 | ALA14809 | N (Acinetobacter baumannii) |
| ADC-75 | ALA14810 | N (Acinetobacter baumannii) |
| ADC-76 | AKQ29837 | N (Acinetobacter baumannii) |
| ADC-77 | ALA14812 | N (Acinetobacter baumannii) |
| ADC-78 | ALA14813 | N (Acinetobacter baumannii) |
| ADC-79 | ALA14814 | N (Acinetobacter baumannii) |
| ADC-80 | AJB66322 | N (Acinetobacter baumannii) |
| ADC-81 | AFH74976 | N (Acinetobacter baumannii) |
| ADC-82 | SBS21217 | N (Acinetobacter baumannii) |
| ADC-83 | ANW47146 | N (Acinetobacter sp. MRSN7700) |
| ADC-84 | ANW47149 | N (Acinetobacter baumannii) |
| ADC-85 | ANW47142 | N (Acinetobacter sp. MRSN7616) |
| ADC-86 | ANW47143 | N (Acinetobacter sp. MRSN7664) |
| ADC-87 | ANW47154 | N (Acinetobacter baumannii) |
| ADC-88 | ANW47135 | N (Acinetobacter baumannii) |
| ADC-89 | ANW47136 | N (Acinetobacter baumannii) |
| ADC-90 | ANW47147 | N (Acinetobacter baumannii) |
| ADC-91 | ANW47132 | N (Acinetobacter baumannii) |
| ADC-92 | ANW47134 | N (Acinetobacter baumannii) |
| ADC-93 | ANW47145 | N (Acinetobacter baumannii) |
| ADC-94 | ANW47137 | N (Acinetobacter baumannii) |
| ADC-95 | ANW47153 | N (Acinetobacter baumannii) |
| ADC-96 | ANW47150 | N (Acinetobacter baumannii) |
| ADC-97 | ANW47139 | N (Acinetobacter baumannii) |
| ADC-98 | ANW47138 | N (Acinetobacter baumannii) |
| ADC-99 | ANW47140 | N (Acinetobacter baumannii) |
| ADC-100 | ANW47141 | N (Acinetobacter baumannii) |
| ADC-101 | ANW47133 | N (Acinetobacter baumannii) |
| ADC-102 | ANW47148 | N (Acinetobacter baumannii) |
| ADC-103 | ANW69905 | N (Acinetobacter baumannii) |
| ADC-104 | ANW69906 | N (Acinetobacter baumannii) |
| ADC-105 | ANW69907 | N (Acinetobacter baumannii) |
| ADC-106 | ANW69909 | N (Acinetobacter baumannii) |
| ADC-107 | ANW69912 | N (Acinetobacter baumannii) |
| ADC-109 | AAV32519 | N (Acinetobacter baumannii) |
| ADC-110 | ABO38124 | N (Acinetobacter baumannii) |
| ADC-112 | ABV21801 | N (Acinetobacter baumannii) |
| ADC-113 | ABV21802 | N (Acinetobacter baumannii) |
| ADC-114 | ETY67158 | N (Acinetobacter baumannii MDR_MMC4) |
| ADC-115 | AFU38919 | N (Acinetobacter baumannii TYTH-1) |
| ADC-116 | WP_017816757 | N (Acinetobacter baumannii AB4A3) |
| ADC-117 | ELW88222 | N (Acinetobacter baumannii AA-014) |
| ADC-119 | ENU51112 | N (Acinetobacter baumannii NIPH 1669) |
| ADC-120 | EKP48471 | N (Acinetobacter baumannii Naval-82) |
| ADC-121 | ENW36647 | N (Acinetobacter baumannii NIPH 201) |
| ADC-122 | ENW46489 | N (Acinetobacter baumannii NIPH 329) |
| ADC-123 | ENV30802 | N (Acinetobacter baumannii NIPH 60) |
| ADC-125 | ENW51227 | N (Acinetobacter baumannii NIPH 67) |
| ADC-127 | ENW00696 | N (Acinetobacter calcoaceticus DSM 30006 = CIP 81.8) |
| ADC-128 | ENU07956 | N (Acinetobacter calcoaceticus NIPH 13) |
| ADC-129 | ENV92309 | N (Acinetobacter calcoaceticus ANC 3680) |
| ADC-130 | CDG74687 | N (Acinetobacter nosocomialis 28F) |
| ADC-131 | ENU48760 | N (Acinetobacter nosocomialis NIPH 2119) |
| ADC-132 | ENW11417 | N (Acinetobacter pittii ANC 3678) |
| ADC-133 | ENU43147 | N (Acinetobacter seifertii) |
| ADC-134 | ENX43770 | N (Acinetobacter sp. NIPH 542) |
| ADC-135 | ENV03983 | N (Acinetobacter sp. NIPH 817) |
| ADC-136 | EOQ64883 | N (Acinetobacter calcoaceticus ANC 3811) |
| ADC-137 | EOQ71234 | N (Acinetobacter pittii ANC 4050) |
| ADC-138 | EOQ73533 | N (Acinetobacter lactucae) |
| ADC-139 | EXS60093 | N (Acinetobacter baumannii 1406589) |
| ADC-140 | EYS55294 | N (Acinetobacter baumannii 16553_10) |
| ADC-141 | EXD64655 | N (Acinetobacter baumannii 58452) |
| ADC-143 | OTL99021 | N (Acinetobacter baumannii) |
| ADC-144 | OVK75103 | N (Acinetobacter baumannii) |
| ADC-145 | KHY08585 | N (Acinetobacter baumannii) |
| ADC-146 | KHV30477 | N (Acinetobacter baumannii) |
| ADC-147 | KJC71195 | N (Acinetobacter baumannii) |
| ADC-148 | AJB47604 | N (Acinetobacter nosocomialis) |
| ADC-149 | ADY82440 | N (Acinetobacter pittii PHEA-2) |
| ADC-150 | AKT73351 | N (Acinetobacter pittii) |
| ADC-151 | AMX20227 | N (Acinetobacter pittii) |
| ADC-152 | AQV05407 | N (Acinetobacter baumannii AB2RED09) |
| ADC-153 | ARO72334 | N (Acinetobacter baumannii MRY12-1052) |
| ADC-154 | ART46239 | N (Acinetobacter baumannii JX101) |
| ADC-155 | ARX71325 | N (Acinetobacter baumannii A369) |
| ADC-156 | ARX71326 | N (Acinetobacter baumannii B30) |
| ADC-157 | ARX71327 | N (Acinetobacter baumannii 13-LS066) |
| ADC-158 | EXE89933 | N (Acinetobacter baumannii 532279) |
| ADC-159 | ATP60652 | N (Acinetobacter baumannii H120) |
| ADC-160 | ATP60653 | N (Acinetobacter baumannii P997) |
| ADC-162 | APQ84581 | N (Acinetobacter baumannii CMC-CR-MDR-Ab4) |
| ADC-163 | AUD40657 | N (Acinetobacter baumannii N8) |
| ADC-164 | AUD40660 | N (Acinetobacter baumannii JX214) |
| ADC-165 | OJK08626 | N (Acinetobacter baumannii RB-1399) |
| ADC-166 | AUD40663 | N (Acinetobacter baumannii B7) |
| ADC-167 | AUD40664 | N (Acinetobacter baumannii T10) |
| ADC-168 | AUD40665 | N (Acinetobacter baumannii T16) |
| ADC-169 | AUG98343 | N (Acinetobacter baumannii SH27) |
| ADC-170 | AUI41018 | N (Acinetobacter baumannii 17A1832) |
| ADC-171 | KCY26530 | N (Acinetobacter baumannii 1042969-1197) |
| ADC-172 | EXD57031 | N (Acinetobacter baumannii 653020) |
| ADC-173 | OBL86128 | N (Acinetobacter baumannii ABUH777) |
| ADC-174 | AXC08546 | N (Acinetobacter baumannii) |
| ADC-175 | AXC08547 | N (Acinetobacter baumannii) |
| ADC-176 | ACJ39441 | N (Acinetobacter baumannii AB0057) |
| ADC-177 | AXC08549 | N (Acinetobacter baumannii) |
| ADC-178 | AXC08550 | N (Acinetobacter baumannii) |
| ADC-179 | AXC08551 | N (Acinetobacter baumannii) |
| ADC-180 | AXC08552 | N (Acinetobacter baumannii) |
| ADC-181 | AZK35803 | N (Acinetobacter baumannii) |
| ADC-182 | AYY88259 | N (Acinetobacter baumannii) |
| ADC-183 | AZK35805 | N (Acinetobacter baumannii) |
| ADC-184 | AGC09439 | N (Acinetobacter baumannii) |
| ADC-185 | QBC36171 | N (Acinetobacter baumannii) |
| ADC-186 | AXB15474 | N (Acinetobacter baumannii) |
| ADC-187 | QBC36173 | N (Acinetobacter baumannii) |
| ADC-188 | QBC36175 | N (Acinetobacter baumannii) |
| ADC-189 | QBC36176 | N (Acinetobacter baumannii) |
| ADC-190 | QBC36177 | N (Acinetobacter baumannii) |
| ADC-191 | EMT97706 | N (Acinetobacter baumannii ABNIH6) |
| ADC-192 | AOA49613 | N (Acinetobacter baumannii) |
| ADC-193 | ADI89941 | N (Acinetobacter oleivorans DR1) |
| ADC-194 | QBX33277 | N (Acinetobacter calcoaceticus AC_2117) |
| ADC-195 | QCF61855 | N (Acinetobacter baumannii) |
| ADC-196 | QDY98381 | N (Acinetobacter baumannii) |
| ADC-197 | QEJ73994 | N (Acinetobacter baumannii 12A079) |
| ADC-198 | QEJ73996 | N (Acinetobacter baumannii 18A2080) |
| ADC-199 | QEJ73997 | N (Acinetobacter baumannii 18A2117) |
| ADC-200 | QEJ73998 | N (Acinetobacter baumannii 19A2601) |
| ADC-201 | QEJ73999 | N (Acinetobacter pittii 16A1504) |
| ADC-202 | QEJ74000 | N (Acinetobacter pittii 18A2277) |
| ADC-203 | QEJ80825 | N (Acinetobacter baumannii 17A1625) |
| ADC-204 | QEJ80826 | N (Acinetobacter baumannii 17A1960) |
| ADC-205 | QEJ80827 | N (Acinetobacter calcoaceticus 13A419) |
| ADC-206 | QEJ80828 | N (Acinetobacter pittii 16A1546) |
| ADC-207 | QEJ80829 | N (Acinetobacter pittii 16A1532) |
| ADC-208 | QEJ80830 | N (Acinetobacter pittii 13A363) |
| ADC-209 | QEJ80831 | N (Acinetobacter pittii 18A2303) |
| ADC-210 | QEJ80832 | N (Acinetobacter pittii 18A2411) |
| ADC-211 | QEJ73995 | N (Acinetobacter baumannii 12A229) |
| ADC-212 | OTN05897 | N (Acinetobacter baumannii PR383) |
| ADC-213 | OTR53589 | N (Acinetobacter baumannii ARLG1845) |
| ADC-214 | OTR85897 | N (Acinetobacter baumannii ARLG1858) |
| ADC-215 | OTT53070 | N (Acinetobacter baumannii ARLG1896) |
| ADC-216 | OTT57830 | N (Acinetobacter baumannii ARLG1898) |
| ADC-217 | OTT60833 | N (Acinetobacter baumannii ARLG1900) |
| ADC-218 | OTU52329 | N (Acinetobacter baumannii ARLG1864) |
| ADC-219 | OTU79690 | N (Acinetobacter baumannii ARLG1882) |
| ADC-220 | OVN99777 | N (Acinetobacter baumannii ARLG-1777) |
| ADC-221 | QGI88785 | N (Acinetobacter pittii) |
| ADC-222 | EXC36858 | N (Acinetobacter baumannii 951631) |
| ADC-223 | OBR19538 | N (Acinetobacter baumannii) |
| ADC-224 | OBE30875 | N (Acinetobacter baumannii) |
| ADC-225 | QGX04220 | N (Acinetobacter baumannii) |
| ADC-226 | EXA77464 | N (Acinetobacter baumannii 1202252) |
| ADC-227 | QGX04222 | N (Acinetobacter baumannii) |
| ADC-228 | QGX04223 | N (Acinetobacter baumannii) |
| ADC-229 | QGX04224 | N (Acinetobacter baumannii) |
| ADC-230 | QGX04225 | N (Acinetobacter baumannii) |
| ADC-231 | QGX04227 | N (Acinetobacter baumannii) |
| ADC-232 | OBA29495 | N (Acinetobacter baumannii) |
| ADC-233 | EXC72393 | N (Acinetobacter baumannii 1042969-1265) |
| ADC-234 | KAF0597708 | N (Acinetobacter baumannii) |
| ADC-235 | KAF0620133 | N (Acinetobacter baumannii) |
| ADC-236 | QLB34755 | N (Acinetobacter baumannii) |
| ADC-237 | OTK49191 | N (Acinetobacter baumannii PR310) |
| ADC-238 | EGY7227092 | N (Acinetobacter baumannii) |
| ADC-239 | EXE72034 | N (Acinetobacter sp. 1566109) |
| ADC-240 | ALJ87177 | N (Acinetobacter baumannii) |
| ADC-241 | EGY6368838 | N (Acinetobacter baumannii) |
| ADC-242 | EGY6653416 | N (Acinetobacter baumannii) |
| ADC-243 | HAV4197092 | N (Acinetobacter baumannii) |
| ADC-244 | ETQ71923 | N (Acinetobacter baumannii UH5107) |
| ADC-245 | EXE91732 | N (Acinetobacter sp. 1578804) |
| ADC-246 | ETR00468 | N (Acinetobacter baumannii UH6507) |
| ADC-247 | AYY53548 | N (Acinetobacter baumannii) |
| ADC-248 | ERH68455 | N (Acinetobacter baumannii EGD-HP18) |
| ADC-249 | EGY6094271 | N (Acinetobacter baumannii) |
| ADC-250 | OTS26706 | N (Acinetobacter baumannii) |
| ADC-251 | OTT95091 | N (Acinetobacter baumannii) |
| ADC-252 | OTU20214 | N (Acinetobacter pittii) |
| ADC-253 | OTU44844 | N (Acinetobacter pittii) |
| ADC-254 | EXE40908 | N (Acinetobacter baumannii 1546444) |
| ADC-255 | ERL68177 | N (Acinetobacter nosocomialis M2) |
| ADC-256 | HAV3701000 | N (Acinetobacter baumannii) |
| ADC-257 | MBV6578165 | N (Acinetobacter baumannii) |
| ADC-258 | HAV4519842 | N (Acinetobacter baumannii) |
| ADC-259 | KKZ48792 | N (Acinetobacter baumannii) |
| ADC-260 | UCZ39549 | N (Acinetobacter baumannii) |
| ADC-261 | UGW32408 | N (Acinetobacter baumannii) |
| ADC-262 | MCA4302018 | N (Acinetobacter baumannii) |
| ADC-263 | EGY2544112 | N (Acinetobacter baumannii) |
| ADC-264 | UHO07591 | N (Acinetobacter calcoaceticus) |
| ADC-265 | OTL17060 | N (Acinetobacter pittii) |
| ADC-266 | UOU25744 | N (Acinetobacter nosocomialis) |
| ADC-267 | UTQ48801 | N (Acinetobacter baumannii) |
| ADC-268 | EHM8006321 | N (Acinetobacter baumannii) |
| ADC-269 | EXR49175 | N (Acinetobacter baumannii 1391434) |
| ADC-270 | MBJ9492029 | N (Acinetobacter baumannii) |
| ADC-271 | HCA5308359 | N (Acinetobacter baumannii) |
| ADC-272 | UTS94217 | N (Acinetobacter baumannii) |
| ADC-273 | UTS94218 | N (Acinetobacter baumannii) |
| ADC-274 | UTS94219 | N (Acinetobacter baumannii) |
| ADC-275 | UTS94220 | N (Acinetobacter baumannii) |
| ADC-276 | UTS94221 | N (Acinetobacter baumannii) |
| ADC-277 | UTS94222 | N (Acinetobacter baumannii) |
| ADC-278 | MBJ9474042 | N (Acinetobacter baumannii) |
| ADC-279 | MCQ1094612 | N (Acinetobacter baumannii) |
| ADC-280 | EIZ7533467 | N (Acinetobacter baumannii) |
| ADC-281 | UTS94249 | N (Acinetobacter lactucae) |
| ADC-282 | UTS94250 | N (Acinetobacter pittii) |
| ADC-283 | UUM03672 | N (Acinetobacter baumannii) |
| ADC-284 | RDM51267 | N (Acinetobacter baumannii) |
| ADC-285 | UVU92353 | N (Acinetobacter pittii) |
| ADC-286 | UZF98454 | N (Acinetobacter pittii) |
| ADC-287 | OCY30567 | N (Acinetobacter pittii) |
| ADC-288 | PPC02561 | N (Acinetobacter pittii) |
| ADC-289 | SSV40494 | N (Acinetobacter nosocomialis) |
| ADC-290 | UZQ18805 | N (Acinetobacter baumannii) |
| ADC-291 | AGH36247 | N (Acinetobacter baumannii D1279779) |
| ADC-292 | WP_274858813 | N (Acinetobacter pittii) |
| ADC-293 | WEG44937 | N (Acinetobacter baumannii) |
| ADC-294 | WEG44938 | N (Acinetobacter baumannii) |
| ADC-295 | EKU38981 | N (Acinetobacter sp. WC-141) |
| ADC-302 | EKB37862 | N (Acinetobacter baumannii Ab33333) |
| ADC-303 | EHU1703587 | N (Acinetobacter baumannii) |
| ADC-304 | EJE7261073 | N (Acinetobacter baumannii) |
| ADC-305 | WP_272267218 | N (Acinetobacter baumannii) |
| ADC-306 | WP_272372047 | N (Acinetobacter baumannii) |
| ADC-307 | WP_272247217 | N (Acinetobacter baumannii) |
| ADC-308 | MBK4745117 | N (Acinetobacter baumannii) |
| ADC-309 | WP_272326044 | N (Acinetobacter baumannii) |
| ADC-310 | EXF21814 | N (Acinetobacter baumannii 1417041) |
| ADC-311 | NUF89511 | N (Acinetobacter baumannii) |
| ADC-312 | EII7756836 | N (Acinetobacter baumannii) |
| ADC-313 | WP_272333251 | N (Acinetobacter baumannii) |
| ADC-314 | WP_272290549 | N (Acinetobacter baumannii) |
| ADC-315 | WP_031380135 | N (Acinetobacter baumannii) |
| ADC-316 | WP_272273772 | N (Acinetobacter baumannii) |
| ADC-317 | WP_272389475 | N (Acinetobacter baumannii) |
| ADC-318 | WP_272311194 | N (Acinetobacter baumannii) |
| ADC-319 | WP_272284684 | N (Acinetobacter baumannii) |
| ADC-320 | WP_272236549 | N (Acinetobacter baumannii) |
| ADC-321 | WP_262093836 | N (Acinetobacter baumannii) |
| ADC-322 | WP_272291658 | N (Acinetobacter baumannii) |
| ADC-323 | WP_272359117 | N (Acinetobacter baumannii) |
| ADC-324 | WP_272276659 | N (Acinetobacter baumannii) |
| ADC-325 | MCT9383172 | N (Acinetobacter baumannii) |
| ADC-326 | WP_272315880 | N (Acinetobacter baumannii) |
| ADC-327 | EIJ9033612 | N (Acinetobacter baumannii) |
| ADC-328 | AOM85301 | N (Acinetobacter baumannii) |
| ADC-329 | WP_272312953 | N (Acinetobacter baumannii) |
| ADC-330 | WP_272357959 | N (Acinetobacter baumannii) |
| ADC-331 | WP_272305010 | N (Acinetobacter baumannii) |
| ADC-332 | WP_272289597 | N (Acinetobacter baumannii) |
| ADC-333 | WP_272321894 | N (Acinetobacter baumannii) |
| ADC-334 | ELW79232 | N (Acinetobacter sp. OIFC021) |
| ADC-335 | WIU89417 | N (Acinetobacter seifertii) |
| ADC-336 | EGY5284444 | N (Acinetobacter baumannii) |
| ADC-337 | HCW4295294 | N (Acinetobacter baumannii) |
| ADC-338 | MDH2594334 | N (Acinetobacter baumannii) |
| ADC-339 | HAV4519810 | N (Acinetobacter baumannii) |
| ADC-340 | WLF01976 | N (Acinetobacter baumannii) |
| ADC-341 | WLF01977 | N (Acinetobacter baumannii) |
| ADC-342 | MCA4182138 | N (Acinetobacter baumannii) |
| ADC-343 | BER91151 | N (Acinetobacter pittii) |
| ADC-344 | HCT1752637 | N (Acinetobacter baumannii) |
| ADC-345 | MDY7271281 | N (Acinetobacter baumannii) |
| ADC-346 | BEV74606 | N (Acinetobacter baumannii) |
| ADC-347 | HDX5930622 | N (Acinetobacter baumannii) |
| ADC-348 | PCM90145 | N (Acinetobacter baumannii) |
| ADC-349 | HDV0602813 | N (Acinetobacter baumannii) |
| ADC-350 | ARG16312 | N (Acinetobacter nosocomialis) |
| ADC-351 | WVW91702 | N (Acinetobacter pittii) |
| ADC-352 | WVW91703 | N (Acinetobacter pittii) |
| ADC-353 | WVW91704 | N (Acinetobacter sp.) |
| ADC-P1 | AGV39975 | N (Acinetobacter baumannii) |
| ADC-71 | ODL99984 | N (Acinetobacter pittii TCM292) |
| **CMH** |  |  |
| CMH-1 | AFI56422 | N (*Enterobacter cloacae) |
| CMH-2 | AKC92684 | A |
| CMH-3 | ABF29657 | N (*Enterobacter cloacae TR1102) |
| CMH-4 | CUI73838 | N (Enterobacter cloacae) |
| CMH-5 | KKY86602 | N (Enterobacter cloacae B2) |
| CMH-6 | QGQ32907 | N (Enterobacter cloacae) |
| CMH-7 | UBJ91318 | N (Enterobacter cloacae) |
| CMH-8 | BDS51132 | N (Enterobacter cloacae subsp. cloacae) |
| CMH-9 | AIG20038 | N (Enterobacter cloacae 4494) |
| CMH-10 | HCC8045472 | N (Enterobacter cloacae) |
| CMH-11 | WFP34008 | N (Enterobacter cloacae) |
| CMH-12 | WFP34010 | N (Enterobacter cloacae) |
| CMH-13 | HAT7731846 | N (Enterobacter cloacae) |
| CMH-T1 | MUI29200 | N (Enterobacter cloacae ENT385) |
| **CMY** |  |  |
| CMY-1 | CAA63264 | A |
| CMY-2 | AIM49619 | A |
| CMY-2b | AAB80855 | A |
| CMY-3 | --- | #N/A |
| CMY-4 | KSY23356 | A |
| CMY-5 | CAB50867 | A |
| CMY-6 | AEN71420 | A |
| CMY-7 | AAZ99136 | A |
| CMY-8 | BAD89188 | A |
| CMY-8b | AAZ03413 | A |
| CMY-9 | BAB72158 | A |
| CMY-10 | AAK59969 | A |
| CMY-11 | AAK31370 | A |
| CMY-12 | CAA76382 | A |
| CMY-13 | EEH94734 | A |
| CMY-14 | ACC60954 | A |
| CMY-15 | CAD88477 | A |
| CMY-16 | AGO62642 | A |
| CMY-17 | AAS13399 | A |
| CMY-18 | AAU95778 | A |
| CMY-19 | BAE48233 | A |
| CMY-20 | AAX58682 | A |
| CMY-21 | AAZ66866 | A |
| CMY-22 | ABB72431 | A |
| CMY-23 | ABD94117 | A |
| CMY-24 | ABN69070 | A |
| CMY-25 | EHL84218 | A |
| CMY-26 | BAF56185 | A |
| CMY-27 | ACA30421 | A |
| CMY-28 | ABQ51091 | A |
| CMY-29 | ABS12248 | A |
| CMY-30 | KRR92472 | A |
| CMY-31 | ABR18736 | A |
| CMY-32 | ACA97846 | A |
| CMY-33 | ACA97847 | A |
| CMY-34 | ABN51006 | N (Citrobacter freundii) |
| CMY-35 | ABN51007 | N (Citrobacter freundii) |
| CMY-36 | AAB46884 | A |
| CMY-37 | BAF36388 | N (Citrobacter freundii) |
| CMY-38 | CAP60699 | A |
| CMY-39 | BAF95726 | N (Citrobacter freundii) |
| CMY-40 | ACA30422 | A |
| CMY-41 | BAG14343 | N (Citrobacter freundii) |
| CMY-42 | ADM21467 | A |
| CMY-43 | ACJ05361 | A |
| CMY-44 | ACJ65711 | A |
| CMY-45 | CBB16411 | A |
| CMY-46 | CBH19182 | A |
| CMY-47 | ADH82410 | N (Citrobacter freundii) |
| CMY-48 | ADP02979 | N (Citrobacter freundii) |
| CMY-49 | ACV32310 | N (Citrobacter freundii) |
| CMY-50 | CBI75447 | A |
| CMY-51 | AFK73431 | A |
| CMY-53 | ADQ38362 | A |
| CMY-54 | ADK55604 | A |
| CMY-55 | ADK55605 | A |
| CMY-56 | ADT91162 | A |
| CMY-57 | ADP37961 | A |
| CMY-58 | ADY19254 | A |
| CMY-59 | WP_063859852 | A |
| CMY-60 | AEM97672 | A |
| CMY-61 | AEM97673 | A |
| CMY-62 | AEM97674 | A |
| CMY-63 | AET07387 | N (Citrobacter freundii) |
| CMY-64 | ADW84690 | A |
| CMY-65 | ABO09820 | N (Citrobacter freundii) |
| CMY-66 | AEZ49849 | A |
| CMY-67 | AFK08541 | N (Citrobacter freundii) |
| CMY-68 | AEZ49855 | N (Citrobacter freundii) |
| CMY-69 | AGE45504 | A |
| CMY-70 | KKC62625 | A |
| CMY-71 | AFK08538 | N (Citrobacter freundii) |
| CMY-72 | AAK32688 | N (Citrobacter freundii) |
| CMY-73 | ACU00152 | N (Citrobacter freundii) |
| CMY-74 | AFU25632 | N (Citrobacter freundii) |
| CMY-75 | AAK32687 | N (Citrobacter freundii) |
| CMY-76 | AFK73437 | N (Citrobacter freundii) |
| CMY-77 | AFU25644 | N (Citrobacter freundii) |
| CMY-78 | AFK73443 | N (Citrobacter freundii) |
| CMY-79 | AFK73446 | N (Citrobacter freundii) |
| CMY-80 | AFK73449 | N (Citrobacter freundii) |
| CMY-81 | AFK73452 | N (Citrobacter freundii) |
| CMY-82 | AHL39324 | N (Citrobacter freundii) |
| CMY-83 | AFU25638 | N (Citrobacter freundii) |
| CMY-84 | AFK73455 | N (Citrobacter freundii) |
| CMY-85 | AHL39322 | N (Citrobacter freundii) |
| CMY-86 | AHL39327 | N (Citrobacter freundii) |
| CMY-87 | BAL63057 | A |
| CMY-89 | CCK86742 | N (Citrobacter freundii) |
| CMY-90 | CCK86743 | N (Citrobacter freundii) |
| CMY-93 | AHM76768 | N (Citrobacter freundii) |
| CMY-94 | AGC54798 | A |
| CMY-95 | AGC54799 | A |
| CMY-96 | AFZ85212 | A |
| CMY-97 | AFZ85213 | A |
| CMY-99 | AGU59995 | A |
| CMY-100 | AHA80101 | N (Citrobacter freundii) |
| CMY-101 | EOQ33708 | A |
| CMY-102 | AHA80103 | A |
| CMY-103 | AHA80104 | N (Citrobacter freundii) |
| CMY-104 | AGR82311 | N (Citrobacter freundii) |
| CMY-105 | AHL39330 | N (Citrobacter freundii) |
| CMY-106 | AJH76980 | N (Citrobacter freundii) |
| CMY-107 | ADD17018 | A |
| CMY-108 | AGZ20169 | A |
| CMY-109 | CAG34070 | N (Citrobacter freundii) |
| CMY-110 | BAO05497 | A |
| CMY-111 | AHW47897 | A |
| CMY-112 | AIT76090 | N (Citrobacter freundii) |
| CMY-113 | AIT76089 | N (Citrobacter freundii) |
| CMY-114 | AIT76099 | N (Citrobacter freundii) |
| CMY-115 | AIT76092 | N (Citrobacter freundii) |
| CMY-116 | AIT76093 | N (Citrobacter freundii) |
| CMY-117 | AIT76097 | N (Citrobacter freundii) |
| CMY-118 | AIT76091 | N (Citrobacter freundii) |
| CMY-119 | AIT76098 | N (Citrobacter freundii) |
| CMY-121 | AIZ48988 | A |
| CMY-122 | AKO62859 | N (Citrobacter freundii) |
| CMY-124 | AKO62861 | N (Citrobacter freundii) |
| CMY-125 | AKO62862 | N (Citrobacter freundii) |
| CMY-127 | AKO62864 | N (Citrobacter freundii) |
| CMY-128 | AKO62865 | N (Citrobacter freundii) |
| CMY-129 | AKO62866 | N (Citrobacter freundii) |
| CMY-130 | AKI06385 | A |
| CMY-131 | AKG51729 | A |
| CMY-132 | AKZ20822 | A |
| CMY-133 | AKZ20821 | A |
| CMY-134 | AKA60779 | A |
| CMY-135 | AKP17985 | N (Citrobacter freundii) |
| CMY-136 | AVR61040 | A |
| CMY-137 | WP_053390271 | N (Citrobacter freundii WCHCF65) |
| CMY-138 | ALM96710 | A |
| CMY-139 | AMK49571 | A |
| CMY-140 | ANJ78051 | A |
| CMY-141 | ANS56705 | A |
| CMY-142 | AOS70335 | A |
| CMY-143 | AOX21805 | A |
| CMY-144 | APC23883 | N (Citrobacter freundii) |
| CMY-145 | APD79118 | A |
| CMY-146 | APD79117 | A |
| CMY-147 | AQM40187 | A |
| CMY-148 | AQS23583 | A |
| CMY-149 | AQS23584 | A |
| CMY-150 | ARQ85811 | N (Citrobacter freundii) |
| CMY-151 | ARE65229 | N (Citrobacter freundii D36-1) |
| CMY-152 | ARM19732 | N (Citrobacter freundii CWH001) |
| CMY-153 | ARQ85812 | A |
| CMY-154 | ARX70574 | A |
| CMY-155 | ALF62843 | A |
| CMY-156 | ATD84840 | A |
| CMY-157 | ASW32315 | N (Citrobacter sp.) |
| CMY-158 | ATD84841 | A |
| CMY-159 | ATD84842 | A |
| CMY-160 | AWI33313 | A |
| CMY-161 | AVP74338 | A |
| CMY-162 | AWR93229 | A |
| CMY-163 | AWU66456 | A |
| CMY-164 | QCT85446 | A |
| CMY-165 | QDM39443 | A |
| CMY-166 | QDM39447 | A |
| CMY-167 | QDY98371 | A |
| CMY-168 | QFR38193 | A |
| CMY-169 | QFR38194 | A |
| CMY-170 | QFR38195 | A |
| CMY-171 | QHD25689 | A |
| CMY-172 | MTZ79300 | A |
| CMY-174 | QQL36767 | A |
| CMY-175 | QVO43829 | N (Citrobacter freundii) |
| CMY-176 | MBJ9337561 | N (Citrobacter freundii) |
| CMY-177 | UBL87560 | A |
| CMY-178 | UDF87831 | A |
| CMY-179 | MBC6501781 | N (Citrobacter freundii) |
| CMY-180 | AYL78128 | N (Citrobacter freundii) |
| CMY-181 | EGT0516627 | N (Citrobacter freundii) |
| CMY-182 | AUV24482 | N (Citrobacter freundii complex sp. CFNIH3) |
| CMY-183 | AUV45551 | N (Citrobacter freundii complex sp. CFNIH9) |
| CMY-184 | WAK12379 | A |
| CMY-185 | WCB91330 | A |
| CMY-186 | MDI2051974 | A |
| CMY-188 | WNH41907 | N (Citrobacter freundii) |
| CMY-189 | BAA02494 | N (Citrobacter freundii GN346) |
| CMY-190 | WPR17911 | A |
| CMY-98 | AGH70380 | N (Citrobacter freundii) |
| **DHA** |  |  |
| DHA-1 | AEP68014 | A |
| DHA-2 | AAG36927 | A |
| DHA-3 | AAR87489 | A |
| DHA-4 | KGZ25601 | N (Morganella morganii) |
| DHA-5 | AEL22919 | N (Morganella morganii) |
| DHA-6 | ADT91161 | A |
| DHA-7 | ADQ00385 | A |
| DHA-9 | AHL39320 | N (Morganella morganii) |
| DHA-10 | AJO16043 | N (Morganella morganii) |
| DHA-12 | CDL68900 | A |
| DHA-13 | AIT76108 | N (Morganella morganii) |
| DHA-14 | ABF29635 | A |
| DHA-15 | AIT76106 | A |
| DHA-16 | AIT76105 | N (Morganella morganii) |
| DHA-17 | AIT76103 | N (Morganella morganii) |
| DHA-18 | AIT76094 | N (Morganella morganii) |
| DHA-19 | AIT76102 | N (Morganella morganii) |
| DHA-20 | AIT76101 | A |
| DHA-21 | AIT76100 | N (Morganella morganii) |
| DHA-22 | AIT76109 | A |
| DHA-23 | AMY61251 | A |
| DHA-24 | AMM39807 | A |
| DHA-25 | AQM40192 | A |
| DHA-26 | AVP74341 | A |
| DHA-27 | AVP74340 | A |
| DHA-28 | AZB74599 | N (Morganella morganii) |
| DHA-29 | QDY98373 | A |
| DHA-30 | BEK76843 | N (Morganella morganii subsp. sibonii) |
| DHA-31 | HDU8654191 | N (Morganella morganii subsp. morganii) |
| DHA-33 | ELX0465764 | A |
| **MIR** |  |  |
| MIR-1 | AAD22636 | A |
| MIR-2 | AAO42602 | N (Enterobacter cloacae) |
| MIR-3 | ANE37558 | N (Enterobacter asburiae) |
| MIR-4 | ABN69112 | A |
| MIR-5 | AJO16037 | A |
| MIR-6 | AFJ79785 | A |
| MIR-7 | ANE37557 | N (Enterobacter asburiae) |
| MIR-9 | AIT76113 | N (Enterobacter asburiae) |
| MIR-10 | AIT76111 | N (Enterobacter asburiae) |
| MIR-11 | AIT76112 | N (Enterobacter cloacae) |
| MIR-12 | AIT76116 | N (Enterobacter cloacae) |
| MIR-13 | AIT76115 | N (Enterobacter asburiae) |
| MIR-14 | AIT76117 | N (Enterobacter asburiae) |
| MIR-15 | KZQ92369 | N (Enterobacter cloacae complex 'Hoffmann cluster IV') |
| MIR-16 | AIT76114 | N (Enterobacter asburiae) |
| MIR-17 | CEA29752 | N (Enterobacter cloacae) |
| MIR-18 | CEF57506 | N (Enterobacter sp. ET82) |
| MIR-19 | ANE37559 | N (Enterobacter asburiae) |
| MIR-20 | ANE37560 | N (Enterobacter asburiae) |
| MIR-21 | ANE37561 | N (Enterobacter asburiae) |
| MIR-22 | ATJ25949 | N (Enterobacter cloacae N12-00349) |
| MIR-23 | AIG20028 | N (Enterobacter cloacae 4405) |
| MIR-24 | AIG20031 | N (Enterobacter cloacae 4444) |
| MIR-25 | CAE6213286 | N (Enterobacter cloacae) |
| **PDC** |  |  |
| PDC-1 | AAM08945 | N (Pseudomonas aeruginosa) |
| PDC-2 | ACQ82806 | N (Pseudomonas aeruginosa) |
| PDC-3 | ACQ82808 | N (Pseudomonas aeruginosa) |
| PDC-4 | ACQ82809 | N (Pseudomonas aeruginosa) |
| PDC-5 | AAM08943 | N (Pseudomonas aeruginosa) |
| PDC-6 | ACQ82811 | N (Pseudomonas aeruginosa) |
| PDC-7 | ACQ82812 | N (Pseudomonas aeruginosa) |
| PDC-8 | ACQ82813 | N (Pseudomonas aeruginosa) |
| PDC-9 | ACQ82814 | N (Pseudomonas aeruginosa) |
| PDC-10 | ACQ82815 | N (Pseudomonas aeruginosa) |
| PDC-11 | ACX31161 | N (Pseudomonas aeruginosa) |
| PDC-12 | ACX31162 | N (Pseudomonas aeruginosa) |
| PDC-13 | ACX31163 | N (Pseudomonas aeruginosa) |
| PDC-14 | ACX31164 | N (Pseudomonas aeruginosa) |
| PDC-15 | ACX31165 | N (Pseudomonas aeruginosa) |
| PDC-16 | ACX31166 | N (Pseudomonas aeruginosa) |
| PDC-17 | ACX31167 | N (Pseudomonas aeruginosa) |
| PDC-18 | ADC96714 | N (Pseudomonas aeruginosa) |
| PDC-19a | CBZ41769 | N (Pseudomonas aeruginosa) |
| PDC-19b | AEM44530 | N (Pseudomonas aeruginosa) |
| PDC-20 | AEM44531 | N (Pseudomonas aeruginosa) |
| PDC-21a | CBZ41771 | N (Pseudomonas aeruginosa) |
| PDC-21b | AEM44532 | N (Pseudomonas aeruginosa) |
| PDC-22 | CBZ41772 | N (Pseudomonas aeruginosa) |
| PDC-23 | CBZ41773 | N (Pseudomonas aeruginosa) |
| PDC-24 | AEM44535 | N (Pseudomonas aeruginosa) |
| PDC-25 | AEM44536 | N (Pseudomonas aeruginosa) |
| PDC-26 | AEM44537 | N (Pseudomonas aeruginosa) |
| PDC-28 | AEM44533 | N (Pseudomonas aeruginosa) |
| PDC-30 | AIG19969 | N (Pseudomonas aeruginosa) |
| PDC-31 | AAM08942 | N (Pseudomonas aeruginosa) |
| PDC-32 | BAE46546 | N (Pseudomonas aeruginosa) |
| PDC-33 | AMR55737 | N (Pseudomonas aeruginosa) |
| PDC-34 | SAJ27912 | #N/A |
| PDC-35 | AIG19972 | N (Pseudomonas aeruginosa) |
| PDC-36 | AIG19973 | N (Pseudomonas aeruginosa) |
| PDC-37 | AIG19974 | N (Pseudomonas aeruginosa) |
| PDC-38 | AIG19975 | N (Pseudomonas aeruginosa) |
| PDC-39 | AAM08410 | N (Pseudomonas aeruginosa) |
| PDC-40 | AIG19977 | N (Pseudomonas aeruginosa) |
| PDC-41 | AIG19978 | N (Pseudomonas aeruginosa) |
| PDC-42 | AIG19979 | N (Pseudomonas aeruginosa) |
| PDC-43 | AIG19980 | N (Pseudomonas aeruginosa) |
| PDC-44 | AIG19981 | N (Pseudomonas aeruginosa) |
| PDC-45 | AIG19982 | N (Pseudomonas aeruginosa) |
| PDC-46 | AIG19983 | N (Pseudomonas aeruginosa) |
| PDC-47 | AIG19984 | N (Pseudomonas aeruginosa) |
| PDC-48 | AIG19985 | N (Pseudomonas aeruginosa) |
| PDC-49 | AIG19986 | N (Pseudomonas aeruginosa) |
| PDC-50 | AIG19987 | N (Pseudomonas aeruginosa) |
| PDC-51 | AIG19988 | N (Pseudomonas aeruginosa) |
| PDC-52 | AIG19989 | N (Pseudomonas aeruginosa) |
| PDC-53 | AIG19990 | N (Pseudomonas aeruginosa) |
| PDC-54 | AIG19991 | N (Pseudomonas aeruginosa) |
| PDC-55 | AIG19992 | N (Pseudomonas aeruginosa) |
| PDC-56 | AIG19993 | N (Pseudomonas aeruginosa) |
| PDC-57 | AIG19994 | N (Pseudomonas aeruginosa) |
| PDC-58 | AIG19995 | N (Pseudomonas aeruginosa) |
| PDC-59 | AIG19996 | N (Pseudomonas aeruginosa) |
| PDC-60 | AIG19997 | N (Pseudomonas aeruginosa) |
| PDC-61 | AIG19998 | N (Pseudomonas aeruginosa) |
| PDC-62 | AIG19999 | N (Pseudomonas aeruginosa) |
| PDC-63 | AIG20000 | N (Pseudomonas aeruginosa) |
| PDC-64 | AIG20001 | N (Pseudomonas aeruginosa) |
| PDC-65 | AIG20002 | N (Pseudomonas aeruginosa) |
| PDC-66 | AIG20003 | N (Pseudomonas aeruginosa) |
| PDC-67 | AIG20004 | N (Pseudomonas aeruginosa) |
| PDC-68 | AIG20005 | N (Pseudomonas aeruginosa) |
| PDC-69 | AIG20006 | N (Pseudomonas aeruginosa) |
| PDC-70 | AIG20007 | N (Pseudomonas aeruginosa) |
| PDC-71 | AIG20008 | N (Pseudomonas aeruginosa) |
| PDC-72 | AIG20009 | N (Pseudomonas aeruginosa) |
| PDC-74 | WP_040184286 | N (Pseudomonas aeruginosa) |
| PDC-75 | AKR18015 | N (Pseudomonas aeruginosa PDC-75) |
| PDC-76 | AKR18016 | N (Pseudomonas aeruginosa PDC-76) |
| PDC-78 | AKR18018 | N (Pseudomonas aeruginosa PDC-78) |
| PDC-80 | ANJ78206 | N (Pseudomonas aeruginosa) |
| PDC-81 | AKR18021 | N (Pseudomonas aeruginosa PDC-81) |
| PDC-82 | AKR18022 | N (Pseudomonas aeruginosa PDC-82) |
| PDC-85 | AKR18025 | N (Pseudomonas aeruginosa PDC-85) |
| PDC-86 | KSN86152 | N (Pseudomonas aeruginosa) |
| PDC-87 | AKR18027 | #N/A |
| PDC-88 | AKR18028 | N (Pseudomonas aeruginosa PDC-88) |
| PDC-89 | AKR18029 | N (Pseudomonas aeruginosa PDC-89) |
| PDC-90 | AKR18030 | N (Pseudomonas aeruginosa PDC-90) |
| PDC-91 | AKR18031 | N (Pseudomonas aeruginosa PDC-91) |
| PDC-92 | AKR18032 | #N/A |
| PDC-94 | AMR55736 | N (Pseudomonas aeruginosa) |
| PDC-95 | AMR55738 | N (Pseudomonas aeruginosa) |
| PDC-96 | AMR55739 | N (Pseudomonas aeruginosa) |
| PDC-97 | AMR55740 | N (Pseudomonas aeruginosa) |
| PDC-98 | ALP56563 | N (Pseudomonas aeruginosa) |
| PDC-99 | ANB78223 | N (Pseudomonas aeruginosa) |
| PDC-100 | ANB78224 | N (Pseudomonas aeruginosa) |
| PDC-101 | ANB78225 | N (Pseudomonas aeruginosa) |
| PDC-102 | ANB78226 | N (Pseudomonas aeruginosa) |
| PDC-103 | ALY87174 | #N/A |
| PDC-104 | ANB78228 | N (Pseudomonas aeruginosa) |
| PDC-105 | ANB78229 | N (Pseudomonas aeruginosa) |
| PDC-106 | ANB78230 | N (Pseudomonas aeruginosa) |
| PDC-107 | ANB78231 | N (Pseudomonas aeruginosa) |
| PDC-108 | ANB78232 | N (Pseudomonas aeruginosa) |
| PDC-109 | ANB78233 | N (Pseudomonas aeruginosa) |
| PDC-110 | ANB78234 | N (Pseudomonas aeruginosa) |
| PDC-111 | ANB78235 | N (Pseudomonas aeruginosa) |
| PDC-112 | ANB78236 | N (Pseudomonas aeruginosa) |
| PDC-113 | ANB78237 | N (Pseudomonas aeruginosa) |
| PDC-114 | AND46540 | N (Pseudomonas aeruginosa 1206834) |
| PDC-115 | AND46541 | N (Pseudomonas aeruginosa 1216382) |
| PDC-116 | AND46542 | N (Pseudomonas aeruginosa 1209087) |
| PDC-117 | AND46543 | N (Pseudomonas aeruginosa 1261878) |
| PDC-118 | AND46544 | N (Pseudomonas aeruginosa 1216427) |
| PDC-119 | AND46545 | N (Pseudomonas aeruginosa 1207698) |
| PDC-120 | AND46546 | N (Pseudomonas aeruginosa 1299217) |
| PDC-121 | AND46547 | N (Pseudomonas aeruginosa 1221511) |
| PDC-122 | AND46549 | N (Pseudomonas aeruginosa 1218214) |
| PDC-123 | AND46550 | N (Pseudomonas aeruginosa 1229772) |
| PDC-124 | AND46551 | N (Pseudomonas aeruginosa 1318019) |
| PDC-125 | AND46552 | N (Pseudomonas aeruginosa) |
| PDC-126 | ANF28160 | N (Pseudomonas aeruginosa) |
| PDC-127 | ANF28161 | N (Pseudomonas aeruginosa) |
| PDC-128 | ANF28162 | N (Pseudomonas aeruginosa) |
| PDC-129 | ANF28163 | N (Pseudomonas aeruginosa) |
| PDC-130 | ANF28164 | N (Pseudomonas aeruginosa) |
| PDC-131 | ANF28165 | N (Pseudomonas aeruginosa) |
| PDC-132 | ANF28166 | N (Pseudomonas aeruginosa) |
| PDC-133 | ANF28167 | N (Pseudomonas aeruginosa) |
| PDC-134 | ANF28168 | N (Pseudomonas aeruginosa) |
| PDC-135 | ANF28169 | N (Pseudomonas aeruginosa) |
| PDC-136 | ANF28170 | N (Pseudomonas aeruginosa) |
| PDC-137 | ANF28171 | N (Pseudomonas aeruginosa) |
| PDC-138 | ANF28172 | N (Pseudomonas aeruginosa) |
| PDC-139 | ANF28173 | N (Pseudomonas aeruginosa) |
| PDC-140 | ANF28174 | N (Pseudomonas aeruginosa) |
| PDC-141 | ANF28175 | N (Pseudomonas aeruginosa) |
| PDC-142 | ANH22236 | N (Pseudomonas aeruginosa) |
| PDC-143 | ANH22237 | N (Pseudomonas aeruginosa) |
| PDC-144 | ANH22238 | N (Pseudomonas aeruginosa) |
| PDC-145 | ANH22239 | N (Pseudomonas aeruginosa) |
| PDC-146 | ANH22240 | N (Pseudomonas aeruginosa) |
| PDC-147 | ANH22241 | #N/A |
| PDC-148 | ANH22242 | N (Pseudomonas aeruginosa) |
| PDC-149 | ANH22243 | N (Pseudomonas aeruginosa) |
| PDC-150 | ANH22244 | N (Pseudomonas aeruginosa) |
| PDC-151 | ANH22245 | N (Pseudomonas aeruginosa) |
| PDC-152 | ANH22246 | N (Pseudomonas aeruginosa) |
| PDC-153 | ANH22247 | N (Pseudomonas aeruginosa) |
| PDC-154 | ANH22248 | N (Pseudomonas aeruginosa) |
| PDC-155 | ANH22249 | N (Pseudomonas aeruginosa) |
| PDC-156 | ANH22250 | N (Pseudomonas aeruginosa) |
| PDC-157 | ANH22251 | N (Pseudomonas aeruginosa) |
| PDC-158 | ANH22252 | N (Pseudomonas aeruginosa) |
| PDC-159 | ANH22253 | N (Pseudomonas aeruginosa) |
| PDC-160 | ANH22254 | N (Pseudomonas aeruginosa) |
| PDC-161 | ANH22255 | N (Pseudomonas aeruginosa) |
| PDC-162 | ANH22256 | N (Pseudomonas aeruginosa) |
| PDC-163 | ANH22257 | N (Pseudomonas aeruginosa) |
| PDC-164 | ANH22258 | N (Pseudomonas aeruginosa) |
| PDC-165 | ANH22259 | N (Pseudomonas aeruginosa) |
| PDC-166 | ANJ78204 | N (Pseudomonas aeruginosa) |
| PDC-167 | ANJ78205 | N (Pseudomonas aeruginosa) |
| PDC-168 | ANM44753 | N (Pseudomonas aeruginosa) |
| PDC-169 | ANM44754 | N (Pseudomonas aeruginosa) |
| PDC-170 | ANM44755 | N (Pseudomonas aeruginosa) |
| PDC-171 | ANM44756 | N (Pseudomonas aeruginosa) |
| PDC-172 | AOD62557 | N (Pseudomonas aeruginosa) |
| PDC-173 | APA44944 | N (Pseudomonas aeruginosa 1514789) |
| PDC-174 | OFB91124 | N (Pseudomonas aeruginosa) |
| PDC-175 | AQK20857 | N (Pseudomonas aeruginosa 1408698) |
| PDC-176 | AQK20858 | N (Pseudomonas aeruginosa 1408743) |
| PDC-177 | AQS23581 | N (Pseudomonas aeruginosa 1375084) |
| PDC-178 | AQS23582 | N (Pseudomonas aeruginosa 1404308) |
| PDC-179 | ARQ87956 | N (Pseudomonas aeruginosa 1370284) |
| PDC-180 | ARQ87957 | N (Pseudomonas aeruginosa 1370907) |
| PDC-181 | ARQ87958 | N (Pseudomonas aeruginosa 1371066) |
| PDC-182 | ARQ87959 | N (Pseudomonas aeruginosa 1373168) |
| PDC-183 | ARQ87960 | N (Pseudomonas aeruginosa 1373238) |
| PDC-184 | ARQ87961 | N (Pseudomonas aeruginosa 1373245) |
| PDC-185 | ARQ87962 | N (Pseudomonas aeruginosa 1377834) |
| PDC-186 | ARQ87963 | N (Pseudomonas aeruginosa 1378016) |
| PDC-187 | ARQ87964 | N (Pseudomonas aeruginosa 1378233) |
| PDC-188 | ARQ87965 | N (Pseudomonas aeruginosa 1385970) |
| PDC-189 | ARQ87966 | N (Pseudomonas aeruginosa 1386780) |
| PDC-190 | ARQ87967 | N (Pseudomonas aeruginosa 1387568) |
| PDC-191 | ARQ87968 | N (Pseudomonas aeruginosa 1387596) |
| PDC-192 | ARQ87969 | N (Pseudomonas aeruginosa 1389730) |
| PDC-193 | ARQ87970 | N (Pseudomonas aeruginosa 1390131) |
| PDC-194 | ARQ87971 | N (Pseudomonas aeruginosa 1393916) |
| PDC-195 | AHH52937 | N (Pseudomonas aeruginosa YL84) |
| PDC-196 | ARQ87973 | N (Pseudomonas aeruginosa 1396243) |
| PDC-197 | ARQ87974 | N (Pseudomonas aeruginosa 1404015) |
| PDC-198 | ARQ87975 | N (Pseudomonas aeruginosa 1404043) |
| PDC-199 | ARQ87976 | N (Pseudomonas aeruginosa 1410839) |
| PDC-200 | ARQ87977 | N (Pseudomonas aeruginosa 1469471) |
| PDC-201 | ARQ87978 | N (Pseudomonas aeruginosa 1470730) |
| PDC-202 | ARQ87979 | N (Pseudomonas aeruginosa 1475818) |
| PDC-203 | ARQ87980 | N (Pseudomonas aeruginosa 1494116) |
| PDC-204 | ARQ87981 | N (Pseudomonas aeruginosa 1377165) |
| PDC-205 | ARQ87982 | N (Pseudomonas aeruginosa 1377184) |
| PDC-206 | ARQ87983 | N (Pseudomonas aeruginosa 1377203) |
| PDC-207 | ARQ87984 | N (Pseudomonas aeruginosa 1385291) |
| PDC-208 | ARQ87985 | N (Pseudomonas aeruginosa 1398076) |
| PDC-209 | ARQ87988 | N (Pseudomonas aeruginosa 1450331) |
| PDC-210 | ARQ87990 | N (Pseudomonas aeruginosa 1499711) |
| PDC-211 | ARX71249 | N (Pseudomonas aeruginosa 1372443) |
| PDC-212 | ARX71250 | N (Pseudomonas aeruginosa 1372713) |
| PDC-213 | ARX71252 | N (Pseudomonas aeruginosa 1379657) |
| PDC-214 | ARX71253 | N (Pseudomonas aeruginosa 1384924) |
| PDC-215 | ARX71254 | N (Pseudomonas aeruginosa 1388960) |
| PDC-216 | ARX71255 | N (Pseudomonas aeruginosa 1395788) |
| PDC-217 | ARX71256 | N (Pseudomonas aeruginosa 1399675) |
| PDC-218 | ARX71257 | N (Pseudomonas aeruginosa 1437939) |
| PDC-219 | ARX71258 | N (Pseudomonas aeruginosa 1518073) |
| PDC-220 | ARX71251 | N (Pseudomonas aeruginosa 1375420) |
| PDC-221 | ATB18531 | N (Pseudomonas aeruginosa) |
| PDC-222 | ATB18532 | N (Pseudomonas aeruginosa) |
| PDC-223 | ATB18533 | N (Pseudomonas aeruginosa) |
| PDC-224 | ATZ76920 | N (Pseudomonas aeruginosa 131561) |
| PDC-225 | ATZ76921 | N (Pseudomonas aeruginosa 142175) |
| PDC-226 | ATZ76922 | N (Pseudomonas aeruginosa 142543) |
| PDC-227 | AUT06964 | N (Pseudomonas aeruginosa) |
| PDC-228 | AUT06965 | N (Pseudomonas aeruginosa) |
| PDC-229 | AUT06966 | N (Pseudomonas aeruginosa) |
| PDC-230 | AUT06967 | N (Pseudomonas aeruginosa) |
| PDC-231 | AUT06968 | N (Pseudomonas aeruginosa) |
| PDC-232 | AUT06969 | N (Pseudomonas aeruginosa) |
| PDC-233 | AUT06970 | N (Pseudomonas aeruginosa) |
| PDC-234 | AUT06971 | N (Pseudomonas aeruginosa) |
| PDC-235 | AUT06972 | N (Pseudomonas aeruginosa) |
| PDC-236 | AUT06973 | N (Pseudomonas aeruginosa) |
| PDC-237 | AUT06974 | N (Pseudomonas aeruginosa) |
| PDC-238 | AUT06975 | N (Pseudomonas aeruginosa) |
| PDC-239 | AUT06976 | N (Pseudomonas aeruginosa) |
| PDC-240 | AUT06977 | N (Pseudomonas aeruginosa) |
| PDC-241 | AUT06978 | N (Pseudomonas aeruginosa) |
| PDC-242 | AUT06979 | N (Pseudomonas aeruginosa) |
| PDC-243 | AUT06980 | N (Pseudomonas aeruginosa) |
| PDC-244 | AUT06982 | N (Pseudomonas aeruginosa) |
| PDC-245 | AUA75408 | N (Pseudomonas aeruginosa) |
| PDC-246 | AUT06984 | N (Pseudomonas aeruginosa) |
| PDC-247 | AUT06985 | N (Pseudomonas aeruginosa) |
| PDC-248 | AUT06986 | N (Pseudomonas aeruginosa) |
| PDC-249 | AUT06987 | N (Pseudomonas sp.) |
| PDC-250 | AWI33278 | N (Pseudomonas aeruginosa) |
| PDC-251 | AWI33279 | N (Pseudomonas aeruginosa) |
| PDC-252 | AWI33280 | N (Pseudomonas aeruginosa) |
| PDC-253 | AWI33281 | N (Pseudomonas aeruginosa) |
| PDC-254 | AWI33282 | N (Pseudomonas aeruginosa) |
| PDC-255 | AWI33283 | N (Pseudomonas aeruginosa) |
| PDC-256 | AWI33284 | N (Pseudomonas aeruginosa) |
| PDC-257 | AWI33285 | N (Pseudomonas aeruginosa) |
| PDC-258 | AWI33286 | N (Pseudomonas aeruginosa) |
| PDC-259 | AWI33287 | N (Pseudomonas aeruginosa) |
| PDC-260 | AWI33288 | N (Pseudomonas aeruginosa) |
| PDC-261 | AWI33289 | N (Pseudomonas aeruginosa) |
| PDC-262 | AWI33290 | N (Pseudomonas aeruginosa) |
| PDC-263 | AWI33291 | N (Pseudomonas aeruginosa) |
| PDC-264 | AWI33292 | N (Pseudomonas aeruginosa) |
| PDC-265 | AWI33293 | N (Pseudomonas aeruginosa) |
| PDC-266 | AWI33294 | N (Pseudomonas aeruginosa) |
| PDC-267 | AWI33295 | N (Pseudomonas aeruginosa) |
| PDC-268 | AWI33296 | N (Pseudomonas aeruginosa) |
| PDC-270 | AWI33298 | N (Pseudomonas aeruginosa) |
| PDC-271 | AWI33299 | N (Pseudomonas aeruginosa) |
| PDC-272 | AWI33300 | N (Pseudomonas aeruginosa) |
| PDC-273 | AWI33301 | N (Pseudomonas aeruginosa) |
| PDC-274 | AWI33302 | N (Pseudomonas aeruginosa) |
| PDC-275 | AWU66402 | N (Pseudomonas aeruginosa) |
| PDC-276 | AWU66411 | N (Pseudomonas aeruginosa) |
| PDC-277 | AWU66412 | N (Pseudomonas aeruginosa) |
| PDC-278 | AWU66413 | N (Pseudomonas aeruginosa) |
| PDC-279 | AWU66414 | N (Pseudomonas aeruginosa) |
| PDC-280 | AWU66415 | N (Pseudomonas aeruginosa) |
| PDC-281 | AWU66416 | N (Pseudomonas aeruginosa) |
| PDC-282 | AWU66417 | N (Pseudomonas aeruginosa) |
| PDC-283 | AWU66418 | N (Pseudomonas aeruginosa) |
| PDC-284 | AWU66419 | N (Pseudomonas aeruginosa) |
| PDC-285 | AWU66420 | N (Pseudomonas aeruginosa) |
| PDC-286 | ARI03796 | N (Pseudomonas aeruginosa PAK) |
| PDC-287 | AWU66421 | N (Pseudomonas aeruginosa) |
| PDC-288 | AWU66422 | N (Pseudomonas aeruginosa) |
| PDC-289 | AWU66423 | N (Pseudomonas aeruginosa) |
| PDC-290 | AWU66424 | N (Pseudomonas aeruginosa) |
| PDC-291 | AWU66426 | N (Pseudomonas aeruginosa) |
| PDC-292 | AWU66427 | N (Pseudomonas aeruginosa) |
| PDC-293 | AWU66428 | N (Pseudomonas aeruginosa) |
| PDC-294 | AWU66404 | N (Pseudomonas aeruginosa) |
| PDC-295 | AWU66405 | N (Pseudomonas aeruginosa) |
| PDC-296 | AWU66406 | N (Pseudomonas aeruginosa) |
| PDC-297 | AWU66407 | N (Pseudomonas aeruginosa) |
| PDC-298 | AWU66408 | N (Pseudomonas aeruginosa) |
| PDC-299 | AWU66409 | N (Pseudomonas aeruginosa) |
| PDC-300 | AWU66410 | N (Pseudomonas aeruginosa) |
| PDC-301 | AWU66458 | N (Pseudomonas aeruginosa) |
| PDC-302 | AWU66459 | N (Pseudomonas aeruginosa) |
| PDC-303 | AWU66460 | N (Pseudomonas aeruginosa) |
| PDC-304 | AXG63951 | N (Pseudomonas aeruginosa) |
| PDC-305 | AXH79881 | N (Pseudomonas aeruginosa) |
| PDC-306 | AXH79882 | N (Pseudomonas aeruginosa) |
| PDC-307 | AXQ11876 | N (Pseudomonas aeruginosa) |
| PDC-308 | AXQ11877 | N (Pseudomonas aeruginosa) |
| PDC-309 | AXQ11878 | N (Pseudomonas aeruginosa) |
| PDC-310 | AXQ11879 | N (Pseudomonas aeruginosa) |
| PDC-311 | AXQ11880 | N (Pseudomonas aeruginosa) |
| PDC-312 | AXQ11881 | N (Pseudomonas aeruginosa) |
| PDC-313 | AXQ11882 | N (Pseudomonas aeruginosa) |
| PDC-314 | AXQ11883 | N (Pseudomonas aeruginosa) |
| PDC-315 | AYF58375 | N (Pseudomonas aeruginosa) |
| PDC-316 | AYF58376 | N (Pseudomonas aeruginosa) |
| PDC-317 | AZB74598 | N (Pseudomonas aeruginosa) |
| PDC-318 | QCO43782 | N (Pseudomonas aeruginosa 186036) |
| PDC-319 | QCO43784 | N (Pseudomonas aeruginosa 186058) |
| PDC-320 | QCO43785 | N (Pseudomonas aeruginosa 186144) |
| PDC-321 | QCO43786 | N (Pseudomonas aeruginosa 186193) |
| PDC-322 | QCO43787 | N (Pseudomonas aeruginosa 186203) |
| PDC-323 | QCO43788 | N (Pseudomonas aeruginosa 196330) |
| PDC-324 | QCO43790 | N (Pseudomonas aeruginosa 196430) |
| PDC-325 | QCO43791 | N (Pseudomonas aeruginosa 196540) |
| PDC-326 | QCO43792 | N (Pseudomonas aeruginosa 196566) |
| PDC-327 | QCO43793 | N (Pseudomonas aeruginosa 196571) |
| PDC-328 | QCO43794 | N (Pseudomonas aeruginosa 196615) |
| PDC-329 | QCO43795 | N (Pseudomonas aeruginosa 196638) |
| PDC-330 | QCO43796 | N (Pseudomonas aeruginosa 196642) |
| PDC-331 | QCO43789 | N (Pseudomonas aeruginosa 196422) |
| PDC-332 | QCO43783 | N (Pseudomonas aeruginosa 186152) |
| PDC-333 | QCU71418 | N (Pseudomonas aeruginosa) |
| PDC-334 | QDC28521 | N (Pseudomonas aeruginosa) |
| PDC-335 | QDC28522 | N (Pseudomonas aeruginosa) |
| PDC-336 | QDC28523 | N (Pseudomonas aeruginosa) |
| PDC-337 | QDM39444 | N (Pseudomonas aeruginosa) |
| PDC-338 | QDM39445 | N (Pseudomonas aeruginosa) |
| PDC-339 | QDM39446 | N (Pseudomonas aeruginosa) |
| PDC-340 | QDM39448 | N (Pseudomonas aeruginosa) |
| PDC-341 | QDO71658 | N (Pseudomonas aeruginosa) |
| PDC-342 | QDO71659 | N (Pseudomonas aeruginosa) |
| PDC-343 | QDO71660 | N (Pseudomonas aeruginosa) |
| PDC-344 | QDO71661 | N (Pseudomonas aeruginosa) |
| PDC-345 | QDO71662 | N (Pseudomonas aeruginosa) |
| PDC-346 | QDO71663 | N (Pseudomonas aeruginosa) |
| PDC-347 | QDO71664 | N (Pseudomonas aeruginosa) |
| PDC-348 | QDO71665 | N (Pseudomonas aeruginosa) |
| PDC-349 | QDO71666 | N (Pseudomonas aeruginosa) |
| PDC-350 | QDO71667 | N (Pseudomonas aeruginosa) |
| PDC-351 | QDO71668 | N (Pseudomonas aeruginosa) |
| PDC-352 | QDO71669 | N (Pseudomonas aeruginosa) |
| PDC-353 | QDO71670 | N (Pseudomonas aeruginosa) |
| PDC-354 | QDO71671 | N (Pseudomonas aeruginosa) |
| PDC-355 | QDO71672 | N (Pseudomonas aeruginosa) |
| PDC-356 | QDO71674 | N (Pseudomonas aeruginosa) |
| PDC-357 | QDO71675 | N (Pseudomonas aeruginosa) |
| PDC-358 | QDO71676 | N (Pseudomonas aeruginosa) |
| PDC-359 | QDO71678 | N (Pseudomonas aeruginosa) |
| PDC-360 | QDO71679 | N (Pseudomonas aeruginosa) |
| PDC-361 | ETV31772 | N (Pseudomonas aeruginosa BWHPSA043) |
| PDC-362 | QDO71681 | N (Pseudomonas aeruginosa) |
| PDC-363 | QDO71682 | N (Pseudomonas aeruginosa) |
| PDC-364 | QDO71683 | N (Pseudomonas aeruginosa) |
| PDC-365 | QDO71684 | N (Pseudomonas aeruginosa) |
| PDC-366 | QDO71685 | N (Pseudomonas aeruginosa) |
| PDC-367 | QDO71686 | N (Pseudomonas aeruginosa) |
| PDC-368 | QDO71687 | N (Pseudomonas aeruginosa) |
| PDC-369 | QDO71688 | N (Pseudomonas aeruginosa) |
| PDC-370 | QDO71689 | N (Pseudomonas aeruginosa) |
| PDC-371 | QDO71690 | N (Pseudomonas aeruginosa) |
| PDC-372 | QDO71692 | N (Pseudomonas aeruginosa) |
| PDC-373 | QDO71693 | N (Pseudomonas aeruginosa) |
| PDC-374 | ENH92143 | N (Pseudomonas aeruginosa PA45) |
| PDC-375 | QDY98377 | N (Pseudomonas aeruginosa) |
| PDC-376 | QDY98378 | N (Pseudomonas aeruginosa) |
| PDC-377 | QDY98379 | N (Pseudomonas aeruginosa) |
| PDC-378 | QDY98380 | N (Pseudomonas aeruginosa) |
| PDC-379 | QDY98374 | N (Pseudomonas aeruginosa) |
| PDC-380 | QDY98375 | N (Pseudomonas aeruginosa) |
| PDC-381 | QDY98376 | N (Pseudomonas aeruginosa) |
| PDC-382 | QDY98383 | N (Pseudomonas aeruginosa) |
| PDC-383 | QED08958 | N (Pseudomonas aeruginosa) |
| PDC-384 | QEJ74002 | N (Pseudomonas aeruginosa 196911) |
| PDC-385 | QEM40510 | N (Pseudomonas aeruginosa 196379) |
| PDC-386 | QEQ92511 | N (Pseudomonas aeruginosa 196986) |
| PDC-387 | QEV89918 | N (Pseudomonas aeruginosa 197016) |
| PDC-388 | QFC98036 | N (Pseudomonas aeruginosa PAE2850) |
| PDC-389 | QHI08148 | N (Pseudomonas aeruginosa) |
| PDC-390 | QHI08149 | N (Pseudomonas aeruginosa) |
| PDC-391 | QHI08150 | N (Pseudomonas aeruginosa) |
| PDC-392 | QHI08151 | N (Pseudomonas aeruginosa) |
| PDC-393 | QHN71477 | N (Pseudomonas aeruginosa) |
| PDC-394 | QHT72951 | N (Pseudomonas aeruginosa) |
| PDC-395 | QHT72952 | N (Pseudomonas aeruginosa) |
| PDC-396 | QHT72953 | N (Pseudomonas aeruginosa) |
| PDC-397 | QIC04083 | N (Pseudomonas aeruginosa) |
| PDC-398 | QIM14680 | N (Pseudomonas aeruginosa) |
| PDC-399 | QIM14681 | N (Pseudomonas aeruginosa) |
| PDC-400 | QIM14682 | N (Pseudomonas aeruginosa) |
| PDC-401 | QIM14684 | N (Pseudomonas aeruginosa) |
| PDC-402 | QIM14685 | N (Pseudomonas aeruginosa) |
| PDC-403 | QIM14686 | N (Pseudomonas aeruginosa) |
| PDC-404 | QIM14687 | N (Pseudomonas aeruginosa) |
| PDC-405 | QIM14688 | N (Pseudomonas aeruginosa) |
| PDC-406 | QIM14689 | N (Pseudomonas aeruginosa) |
| PDC-407 | QIM14690 | N (Pseudomonas aeruginosa) |
| PDC-408 | QIM14691 | N (Pseudomonas aeruginosa) |
| PDC-409 | QIM14692 | N (Pseudomonas aeruginosa) |
| PDC-410 | QIM14693 | N (Pseudomonas aeruginosa) |
| PDC-411 | QIM14694 | N (Pseudomonas aeruginosa) |
| PDC-412 | QIM14695 | N (Pseudomonas aeruginosa) |
| PDC-413 | QIM14696 | N (Pseudomonas aeruginosa) |
| PDC-414 | QIM14697 | N (Pseudomonas aeruginosa) |
| PDC-415 | QIM14698 | N (Pseudomonas aeruginosa) |
| PDC-416 | QIM14699 | N (Pseudomonas aeruginosa) |
| PDC-417 | QIM14700 | N (Pseudomonas aeruginosa) |
| PDC-418 | QIM14683 | N (Pseudomonas aeruginosa) |
| PDC-419 | QIM14703 | N (Pseudomonas aeruginosa) |
| PDC-420 | QIM14704 | N (Pseudomonas aeruginosa) |
| PDC-421 | QIM59617 | N (Pseudomonas aeruginosa) |
| PDC-422 | QKF95719 | N (Pseudomonas aeruginosa) |
| PDC-423 | QKF95720 | N (Pseudomonas aeruginosa) |
| PDC-424 | QKF95721 | N (Pseudomonas aeruginosa) |
| PDC-425 | QKF95722 | N (Pseudomonas aeruginosa) |
| PDC-426 | QKF95723 | N (Pseudomonas aeruginosa) |
| PDC-427 | QKF95724 | N (Pseudomonas aeruginosa) |
| PDC-428 | QKF95725 | N (Pseudomonas aeruginosa) |
| PDC-429 | QKF95726 | N (Pseudomonas aeruginosa) |
| PDC-430 | QKF95727 | N (Pseudomonas aeruginosa) |
| PDC-431 | ERY76913 | N (Pseudomonas aeruginosa BWHPSA016) |
| PDC-432 | MBA4958488 | N (Pseudomonas aeruginosa) |
| PDC-433 | OXR79059 | N (Pseudomonas aeruginosa) |
| PDC-434 | QKO79895 | N (Pseudomonas aeruginosa) |
| PDC-435 | QKO79896 | N (Pseudomonas aeruginosa) |
| PDC-436 | QKO79897 | N (Pseudomonas aeruginosa) |
| PDC-437 | QKO79898 | N (Pseudomonas aeruginosa) |
| PDC-438 | QKO79899 | N (Pseudomonas aeruginosa) |
| PDC-439 | QKO79900 | N (Pseudomonas aeruginosa) |
| PDC-440 | QKO79901 | N (Pseudomonas aeruginosa) |
| PDC-441 | QKO79902 | N (Pseudomonas aeruginosa) |
| PDC-442 | QKO79903 | N (Pseudomonas aeruginosa) |
| PDC-443 | QKO79904 | N (Pseudomonas aeruginosa) |
| PDC-444 | QKO79905 | N (Pseudomonas aeruginosa) |
| PDC-445 | QKO79906 | N (Pseudomonas aeruginosa) |
| PDC-446 | QKO79907 | N (Pseudomonas aeruginosa) |
| PDC-447 | QKO79908 | N (Pseudomonas aeruginosa) |
| PDC-448 | QKO79890 | N (Pseudomonas aeruginosa) |
| PDC-449 | QKO79891 | N (Pseudomonas aeruginosa) |
| PDC-450 | QKO79892 | N (Pseudomonas aeruginosa) |
| PDC-451 | QKO79893 | N (Pseudomonas aeruginosa) |
| PDC-452 | QKO79894 | N (Pseudomonas aeruginosa) |
| PDC-453 | QLE10678 | N (Pseudomonas aeruginosa) |
| PDC-454 | NRS76295 | N (Pseudomonas aeruginosa) |
| PDC-455 | KSR48412 | N (Pseudomonas aeruginosa) |
| PDC-456 | QNG62043 | N (Pseudomonas aeruginosa) |
| PDC-457 | OZO28236 | N (Pseudomonas aeruginosa) |
| PDC-458 | QPI70489 | N (Pseudomonas aeruginosa) |
| PDC-459 | QPI70490 | N (Pseudomonas aeruginosa) |
| PDC-460 | QPI70491 | N (Pseudomonas aeruginosa) |
| PDC-461 | QPI70492 | N (Pseudomonas aeruginosa) |
| PDC-462 | QPI70493 | N (Pseudomonas aeruginosa) |
| PDC-463 | QPI70494 | N (Pseudomonas aeruginosa) |
| PDC-464 | QPI70495 | N (Pseudomonas aeruginosa) |
| PDC-465 | QPI70496 | N (Pseudomonas aeruginosa) |
| PDC-466 | QPI70497 | N (Pseudomonas aeruginosa) |
| PDC-467 | QQK53881 | N (Pseudomonas aeruginosa) |
| PDC-468 | QQK53882 | N (Pseudomonas aeruginosa) |
| PDC-469 | QQK53883 | N (Pseudomonas aeruginosa) |
| PDC-470 | QQK53884 | N (Pseudomonas aeruginosa) |
| PDC-471 | QQK53885 | N (Pseudomonas aeruginosa) |
| PDC-472 | QRN78587 | N (Pseudomonas aeruginosa) |
| PDC-473 | QRV13255 | N (Pseudomonas aeruginosa) |
| PDC-474 | QRV13256 | N (Pseudomonas aeruginosa) |
| PDC-475 | QRV13257 | N (Pseudomonas aeruginosa) |
| PDC-476 | QRV13258 | N (Pseudomonas aeruginosa) |
| PDC-477 | QSG71713 | N (Pseudomonas aeruginosa) |
| PDC-478 | QSG71714 | N (Pseudomonas aeruginosa) |
| PDC-479 | MBI8474107 | N (Pseudomonas aeruginosa) |
| PDC-480 | MBG6899535 | N (Pseudomonas aeruginosa) |
| PDC-481 | QTG68653 | N (Pseudomonas aeruginosa) |
| PDC-482 | QUR41145 | N (Pseudomonas aeruginosa) |
| PDC-483 | QUR41146 | N (Pseudomonas aeruginosa) |
| PDC-484 | QWW93428 | N (Pseudomonas aeruginosa) |
| PDC-485 | QWW93429 | N (Pseudomonas aeruginosa) |
| PDC-486 | QWW93430 | N (Pseudomonas aeruginosa) |
| PDC-487 | QWW93431 | N (Pseudomonas aeruginosa) |
| PDC-488 | QWW93432 | N (Pseudomonas aeruginosa) |
| PDC-489 | QYZ75852 | N (Pseudomonas aeruginosa) |
| PDC-490 | QYZ75853 | N (Pseudomonas aeruginosa) |
| PDC-491 | QYZ75854 | N (Pseudomonas aeruginosa) |
| PDC-492 | QYZ75855 | N (Pseudomonas aeruginosa) |
| PDC-493 | QYZ75856 | N (Pseudomonas aeruginosa) |
| PDC-494 | QYZ75857 | N (Pseudomonas aeruginosa) |
| PDC-495 | QYZ75858 | N (Pseudomonas aeruginosa) |
| PDC-496 | QYZ75859 | N (Pseudomonas aeruginosa) |
| PDC-497 | UAX43329 | N (Pseudomonas aeruginosa) |
| PDC-498 | UGN25765 | N (Pseudomonas aeruginosa 219052) |
| PDC-499 | UGN25766 | N (Pseudomonas aeruginosa 219062) |
| PDC-500 | UGN25767 | N (Pseudomonas aeruginosa 219246) |
| PDC-501 | UGW32402 | N (Pseudomonas aeruginosa V-3-1) |
| PDC-502 | UGW32403 | N (Pseudomonas aeruginosa VI-4-2) |
| PDC-503 | UGW32404 | N (Pseudomonas aeruginosa VIII-4-2) |
| PDC-504 | UGW32405 | N (Pseudomonas aeruginosa VIII-4-3) |
| PDC-505 | UGW32406 | N (Pseudomonas aeruginosa XII-1-2) |
| PDC-506 | UGW32407 | N (Pseudomonas aeruginosa IX-3-3) |
| PDC-507 | UHO07588 | N (Pseudomonas aeruginosa 219331) |
| PDC-508 | UHO07593 | N (Pseudomonas aeruginosa CFD_2012_09) |
| PDC-509 | UHO07595 | N (Pseudomonas aeruginosa CFD_2012_47) |
| PDC-510 | UKA98423 | N (Pseudomonas aeruginosa 229474) |
| PDC-511 | UKA98424 | N (Pseudomonas aeruginosa 229481) |
| PDC-512 | ULU82598 | N (Pseudomonas aeruginosa) |
| PDC-513 | ULU82665 | N (Pseudomonas aeruginosa) |
| PDC-514 | UMO60339 | N (Pseudomonas aeruginosa) |
| PDC-515 | UMO60340 | N (Pseudomonas aeruginosa) |
| PDC-516 | UMO60341 | N (Pseudomonas aeruginosa) |
| PDC-517 | UNZ81761 | N (Pseudomonas aeruginosa) |
| PDC-518 | UOU25743 | N (Pseudomonas aeruginosa) |
| PDC-519 | URY98700 | N (Pseudomonas aeruginosa) |
| PDC-520 | UTQ48800 | N (Pseudomonas aeruginosa) |
| PDC-521 | UTS94203 | N (Pseudomonas aeruginosa) |
| PDC-522 | UTS94204 | N (Pseudomonas aeruginosa) |
| PDC-523 | UTS94205 | N (Pseudomonas aeruginosa) |
| PDC-524 | UTS94206 | N (Pseudomonas aeruginosa) |
| PDC-525 | UTS94207 | N (Pseudomonas aeruginosa) |
| PDC-526 | HBO2746427 | N (Pseudomonas aeruginosa) |
| PDC-527 | UTS94209 | N (Pseudomonas aeruginosa) |
| PDC-528 | TJZ01032 | N (Pseudomonas aeruginosa) |
| PDC-529 | RPW62925 | N (Pseudomonas aeruginosa) |
| PDC-530 | UUM03671 | N (Pseudomonas aeruginosa) |
| PDC-531 | UVB72393 | N (Pseudomonas aeruginosa) |
| PDC-532 | UWQ12886 | N (Pseudomonas aeruginosa) |
| PDC-533 | UWQ12887 | N (Pseudomonas aeruginosa) |
| PDC-534 | UZQ18791 | N (Pseudomonas aeruginosa) |
| PDC-535 | RTV77445 | N (Pseudomonas aeruginosa) |
| PDC-536 | UZQ18793 | N (Pseudomonas aeruginosa) |
| PDC-537 | MBG7037483 | N (Pseudomonas aeruginosa) |
| PDC-538 | WAW84756 | N (Pseudomonas aeruginosa) |
| PDC-539 | WAW84757 | N (Pseudomonas aeruginosa) |
| PDC-540 | WDE35084 | N (Pseudomonas aeruginosa) |
| PDC-541 | WDE35085 | N (Pseudomonas aeruginosa) |
| PDC-542 | WDE35086 | N (Pseudomonas aeruginosa) |
| PDC-543 | WEG44941 | N (Pseudomonas aeruginosa) |
| PDC-544 | WEG44942 | N (Pseudomonas aeruginosa) |
| PDC-545 | WEG44943 | N (Pseudomonas aeruginosa) |
| PDC-546 | HCF4506154 | N (Pseudomonas aeruginosa) |
| PDC-547 | WP_275802705 | N (Pseudomonas aeruginosa) |
| PDC-548 | WFG63533 | N (Pseudomonas aeruginosa) |
| PDC-549 | WFG63534 | N (Pseudomonas aeruginosa) |
| PDC-550 | WFG63535 | N (Pseudomonas aeruginosa) |
| PDC-551 | WFG63536 | N (Pseudomonas aeruginosa) |
| PDC-552 | WFG63537 | N (Pseudomonas aeruginosa) |
| PDC-553 | WJR95504 | N (Pseudomonas aeruginosa) |
| PDC-554 | WKB14826 | N (Pseudomonas aeruginosa) |
| PDC-555 | WKB14827 | N (Pseudomonas aeruginosa) |
| PDC-556 | WKB14828 | N (Pseudomonas aeruginosa) |
| PDC-557 | WKB14829 | N (Pseudomonas aeruginosa) |
| PDC-558 | HCF9371628 | N (Pseudomonas aeruginosa) |
| PDC-559 | WKB14831 | N (Pseudomonas aeruginosa) |
| PDC-560 | MCO1749962 | N (Pseudomonas aeruginosa) |
| PDC-561 | WKB14833 | N (Pseudomonas aeruginosa) |
| PDC-562 | WKB14834 | N (Pseudomonas aeruginosa) |
| PDC-563 | WKB14835 | N (Pseudomonas aeruginosa) |
| PDC-564 | HCE9298344 | N (Pseudomonas aeruginosa) |
| PDC-565 | HCE6103944 | N (Pseudomonas aeruginosa) |
| PDC-566 | WLF01985 | N (Pseudomonas aeruginosa) |
| PDC-567 | WLF01986 | N (Pseudomonas aeruginosa) |
| PDC-568 | WLF01987 | N (Pseudomonas aeruginosa) |
| PDC-569 | WLF01988 | N (Pseudomonas aeruginosa) |
| PDC-570 | WLF01989 | N (Pseudomonas aeruginosa) |
| PDC-571 | WLF01990 | N (Pseudomonas aeruginosa) |
| PDC-572 | RCH01810 | N (Pseudomonas aeruginosa) |
| PDC-573 | WLF01992 | N (Pseudomonas aeruginosa) |
| PDC-574 | WLF01993 | N (Pseudomonas aeruginosa) |
| PDC-575 | WMI45072 | N (Pseudomonas aeruginosa) |
| PDC-576 | MBO8413357 | N (Pseudomonas aeruginosa) |
| PDC-577 | WPB15230 | N (Pseudomonas aeruginosa) |
| PDC-578 | WPG58515 | N (Pseudomonas aeruginosa) |
| PDC-579 | WPG58516 | N (Pseudomonas aeruginosa) |
| PDC-580 | WPG58517 | N (Pseudomonas aeruginosa) |
| PDC-581 | WPG58519 | N (Pseudomonas aeruginosa) |
| PDC-582 | HCW0152309 | N (Pseudomonas aeruginosa) |
| PDC-583 | HCW1020513 | N (Pseudomonas aeruginosa) |
| PDC-584 | HCW1026566 | N (Pseudomonas aeruginosa) |
| PDC-585 | WVW91591 | N (Pseudomonas aeruginosa) |
| PDC-586 | WVW91686 | N (Pseudomonas aeruginosa) |
| PDC-587 | HCF6216454 | N (Pseudomonas aeruginosa) |
| PDC-588 | WVW91688 | N (Pseudomonas aeruginosa) |
| PDC-589 | WVW91689 | N (Pseudomonas aeruginosa) |
| PDC-590 | WVW91690 | N (Pseudomonas aeruginosa) |
| PDC-591 | WVW91691 | N (Pseudomonas aeruginosa) |
| PDC-592 | HDY6346959 | N (Pseudomonas aeruginosa) |
| **PIB** |  |  |
| PIB-1 | AAG08927 | N (Pseudomonas aeruginosa PAO1) |
