# Supplementary material for: AmpC β-lactamases: A key to antibiotic resistance in ESKAPE pathogens
Source: Cell Surf. 2025 Sep 22;14:100154. doi: 10.1016/j.tcsw.2025.100154 (PMC12528871; doi:10.1016/j.tcsw.2025.100154)
Supplement: Supplementary Table 2 — Distribution of AmpC enzyme groups in ESKAPE pathogens. [file mmc7.docx]

**Table S2:** Distribution of AmpC enzyme groups in ESKAPE pathogens.

| **Organism** | **Enzyme groups of AmpC found in chromosome where both chromosome and plasmid are genetic material** | **Enzyme groups of AmpC found in plasmid where both chromosome and plasmid are genetic material** | **Enzyme groups of AmpC found in chromosome where only chromosome is genetic material** |
| --- | --- | --- | --- |
| *E. faecium* | 0 | 0 | 0 |
| *S. aureus* | 0 | 0 | 0 |
| *K. pneumoniae* | DHA-1  CMY-4  CMY-6 | DHA-1  CMY-4  DHA-7  CMY-6  CMY-16  CMY-2  DHA-15  CMY-172  CMY-174 | DHA-1 |
|  |  |  |  |
|  |  |  |  |
|  |  |  |  |
|  |  |  |  |
|  |  |  |  |
|  |  |  |  |
|  |  |  |  |
|  |  |  |  |
| *A. baumannii* | ADC-33  ADC-176  ADC-115  ADC-291  ADC-240  ADC-162  ADC-152  ADC-154  ADC-155  ADC-156  ADC-163  ADC-166  ADC-169  ADC-186  ADC-175  ADC-247  ADC-182  ADC-181  ADC-263  ADC-249  ADC-238  ADC-268  ADC-117  ADC-191  ADC-248  ADC-222  ADC-158  ADC-269  ADC-82  ADC-99  ADC-87  ADC-106  ADC-25  ADC-30  ADC-50  ADC-52  ADC-26  ADC-31  ADC-56  ADC-11  ADC-1  ADC-6  ADC-81  ADC-18  ADC-73  ADC-5  ADC-80  ADC-76  ADC-32  ADC-74  ADC-79  ADC-243  ADC-347  ADC-165  ADC-212  ADC-214  ADC-217  ADC-185  ADC-199  ADC-203  ADC-236  ADC-184  ADC-328  ADC-75 | ADC-33  ADC-176  ADC-115  ADC-291  ADC-240  ADC-162  ADC-152  ADC-154  ADC-155  ADC-156  ADC-163  ADC-166  ADC-169  ADC-186  ADC-175  ADC-247  ADC-182  ADC-181  ADC-263  ADC-249  ADC-238  ADC-268  ADC-117  ADC-191  ADC-248  ADC-222  ADC-158  ADC-269  ADC-82  ADC-99  ADC-87  ADC-106  ADC-25  ADC-30  ADC-50  ADC-52  ADC-26  ADC-31  ADC-56  ADC-11  ADC-1  ADC-6  ADC-81  ADC-18  ADC-73  ADC-5  ADC-80  ADC-76  ADC-32  ADC-74  ADC-79  ADC-243  ADC-347  ADC-165  ADC-212  ADC-214  ADC-217  ADC-185  ADC-199  ADC-203  ADC-236 | ADC-33  ADC-176  ADC-115  ADC-184  ADC-291  ADC-328  ADC-162  ADC-152  ADC-155  ADC-156  ADC-247  ADC-191  ADC-222  ADC-158  ADC-82  ADC-25  ADC-30  ADC-52  ADC-26  ADC-31  ADC-11  ADC-73  ADC-5  ADC-80  ADC-76  ADC-75  ADC-217  ADC-199  ADC-203 |
|  |  |  |  |
|  |  |  |  |
|  |  |  |  |
|  |  |  |  |
|  |  |  |  |
|  |  |  |  |
|  |  |  |  |
|  |  |  |  |
|  |  |  |  |
|  |  |  |  |
|  |  |  |  |
|  |  |  |  |
|  |  |  |  |
|  |  |  |  |
|  |  |  |  |
|  |  |  |  |
|  |  |  |  |
|  |  |  |  |
|  |  |  |  |
|  |  |  |  |
|  |  |  |  |
|  |  |  |  |
|  |  |  |  |
|  |  |  |  |
|  |  |  |  |
|  |  |  |  |
|  |  |  |  |
|  |  |  |  |
|  |  |  |  |
|  |  |  |  |
|  |  |  |  |
|  |  |  |  |
|  |  |  |  |
|  |  |  |  |
|  |  |  |  |
|  |  |  |  |
|  |  |  |  |
|  |  |  |  |
|  |  |  |  |
|  |  |  |  |
|  |  |  |  |
|  |  |  |  |
|  |  |  |  |
|  |  |  |  |
|  |  |  |  |
|  |  |  |  |
|  |  |  |  |
|  |  |  |  |
|  |  |  |  |
|  |  |  |  |
|  |  |  |  |
|  |  |  |  |
|  |  |  |  |
|  |  |  |  |
|  |  |  |  |
|  |  |  |  |
|  |  |  |  |
|  |  |  |  |
|  |  |  |  |
|  |  |  |  |
|  |  |  |  |
|  |  |  |  |
|  |  |  |  |
| *P. aeruginosa* | PDC-374  PDC-34  PDC-105  PDC-142  PDC-31  PDC-5  PDC-3  PDC-6  PDC-8  PDC-11  PDC-15  PDC-16  PDC-19a  PDC-24  PDC-30  PDC-37  PDC-60  PDC-98 | PDC-374  PDC-34  PDC-105  PDC-142  PDC-31  PDC-5  PDC-3  PDC-6  PDC-8  PDC-11  PDC-15  PDC-16  PDC-19a  PDC-24  PDC-30  PDC-37  PDC-60  PDC-98  CMY-2 | PIB-1  PDC-122  PDC-123  PDC-245  PDC-264  PDC-374  PDC-97  PDC-34  PDC-100  PDC-137  PDC-142  PDC-147  PDC-151  PDC-162  PDC-167  PDC-80  PDC-39  PDC-31  PDC-5  PDC-1  PDC-3  PDC-8  PDC-11  PDC-12  PDC-15  PDC-16  PDC-19a  PDC-22  PDC-23  PDC-28  PDC-24  PDC-30  PDC-35  PDC-37  PDC-43  PDC-45  PDC-46  PDC-51  PDC-59  PDC-60  PDC-66  PDC-98  PDC-103  PDC-457  PDC-331  PDC-364 |
|  |  |  |  |
|  |  |  |  |
|  |  |  |  |
|  |  |  |  |
|  |  |  |  |
|  |  |  |  |
|  |  |  |  |
|  |  |  |  |
|  |  |  |  |
|  |  |  |  |
|  |  |  |  |
|  |  |  |  |
|  |  |  |  |
|  |  |  |  |
|  |  |  |  |
|  |  |  |  |
|  |  |  |  |
|  |  |  |  |
|  |  |  |  |
|  |  |  |  |
|  |  |  |  |
|  |  |  |  |
|  |  |  |  |
|  |  |  |  |
|  |  |  |  |
|  |  |  |  |
|  |  |  |  |
|  |  |  |  |
|  |  |  |  |
|  |  |  |  |
|  |  |  |  |
|  |  |  |  |
|  |  |  |  |
|  |  |  |  |
|  |  |  |  |
|  |  |  |  |
|  |  |  |  |
|  |  |  |  |
|  |  |  |  |
|  |  |  |  |
|  |  |  |  |
|  |  |  |  |
|  |  |  |  |
|  |  |  |  |
|  |  |  |  |
| *Enterobacter spp.* | ACT-1  ACT-55  ACT-65  ACT-56  ACT-90  MIR-23  CMH-9  ACT-67  ACT-53  MIR-22  ACT-57  ACT-64  ACT-115  ACT-87  ACT-95  ACT-102  ACT-89  CMH-4  MIR-15  MIR-3  MIR-19  MIR-20  MIR-21  ACT-40  ACT-46  ACT-51  ACT-3  ACT-4  ACT-6  ACT-9  ACT-16  ACT-23  ACT-28  ACT-24  ACT-25  ACT-27  ACT-44  MIR-14  ACT-52  MIR-17  ACT-37  MIR-5  CMH-3  ACT-45  ACT-17  ACT-75  ACT-74  ACT-77  ACT-99  ACT-84  CMH-7  DHA-1  ACC-1 | ACT-1  ACT-55  ACT-65  ACT-56  ACT-90  MIR-23  CMH-9  ACT-67  ACT-53  MIR-22  ACT-57  ACT-64  ACT-115  ACT-87  ACT-95  ACT-102  ACT-89  CMH-4  MIR-15  MIR-3  MIR-19  MIR-20  MIR-21  ACT-40  ACT-46  ACT-51  ACT-3  ACT-4  ACT-6  ACT-9  ACT-16  ACT-23  ACT-28  ACT-24  ACT-25  ACT-27  ACT-44  MIR-14  ACT-52  MIR-17  ACT-37  MIR-5  CMH-3  ACT-45  ACT-17  ACT-75  ACT-74  ACT-77  ACT-99  ACT-84  CMH-7 | ACT-C198  ACT-55  ACT-65  ACT-56  ACT-93  ACT-C197  MIR-7  ACT-9  ACT-25  MIR-10  ACT-17  ACT-74  ACT-103 |
|  |  |  |  |
|  |  |  |  |
|  |  |  |  |
|  |  |  |  |
|  |  |  |  |
|  |  |  |  |
|  |  |  |  |
|  |  |  |  |
|  |  |  |  |
|  |  |  |  |
|  |  |  |  |
|  |  |  |  |
|  |  |  |  |
|  |  |  |  |
|  |  |  |  |
|  |  |  |  |
|  |  |  |  |
|  |  |  |  |
|  |  |  |  |
|  |  |  |  |
|  |  |  |  |
|  |  |  |  |
|  |  |  |  |
|  |  |  |  |
|  |  |  |  |
|  |  |  |  |
|  |  |  |  |
|  |  |  |  |
|  |  |  |  |
|  |  |  |  |
|  |  |  |  |
|  |  |  |  |
|  |  |  |  |
|  |  |  |  |
|  |  |  |  |
|  |  |  |  |
|  |  |  |  |
|  |  |  |  |
|  |  |  |  |
|  |  |  |  |
|  |  |  |  |
|  |  |  |  |
|  |  |  |  |
|  |  |  |  |
|  |  |  |  |
|  |  |  |  |
|  |  |  |  |
|  |  |  |  |
|  |  |  |  |
|  |  |  |  |
|  |  |  |  |
|  |  |  |  |
