# Supplementary material for: AmpC β-lactamases: A key to antibiotic resistance in ESKAPE pathogens
Source: Cell Surf. 2025 Sep 22;14:100154. doi: 10.1016/j.tcsw.2025.100154 (PMC12528871; doi:10.1016/j.tcsw.2025.100154)
Supplement: Supplementary Table 3 — List of variations within each enzyme group by comparing amino acid positions where substitution occurs. [file mmc8.docx]

**Table S3:** List of variations within each enzyme group by comparing amino acid positions where substitution occurs.

| **S. No.** | **Enzyme Group** | **Organism & Gene Location (s)** | **Number of sequences** | **Variations** |
| --- | --- | --- | --- | --- |
| 1 | CMY | *K. pneumoniae* (B: chromosome,  B: plasmid, O: chromosome), *P. aeruginosa* (B: Plasmid) | 54 | A171S, R221L, R221W, N366I, W374R |
| 2 | DHA | *K. pneumoniae* (B: plasmid, B: chromosome, O: chromosome)  *Enterobacter spp* (B: chromosome) | 135 | D165Y |
| 3 | ADC | *A. baumannii* (B: chromosome, B: plasmid, O: chromosome) | 755 | R2Q, L11F, S12P, S18N, G24D, D29E, I32V, E45D, M52I, V56I, M65I, S80R, S81I, T82S, T94N, A95G, G99A, N103A, D110K, T112R, T112K, E119V, P124S, D126N, G139S, Q144K, Q144R, K150Q, D152E, F158L, K163Q, P164T, S167A, S167P, Y171S, R172Q, G183R, K184E, V185I, V185F, A187G, A187S, N191K, K192Q, F194L, D195S, V197A, K200E, A205D, A205P, G207H, H210N, H210Y, P216A, M220I, A224D, F225Y, F225V, V236A, N237T, P238R, D242G, P254A, A255E, G257S, S267K, S267T, H270Y, H270N, L273F, N274T, P279S, A280K, A280T, D281N, N287S, R293H, R293F, V296E, V296I, V296L, N297G, S309A, A312T, T313P, T313M, N321S, T333S, Y344F, T351N, T351S, G352R, T355S, V357I, A386T, N389D, N389S, K393E |
| 4 | ACC | *Enterobacter spp* (B: chromosome) | 13 | Not found |
| 5 | ACT | *Enterobacter spp* (B: chromosome) | 377 | M36K, K37T, K37R, S39F, S39L, S39T, L40V, L40F, C41F, C42S, L45M, G47S, G47A, I48L, I48V, I48T, C50G, S51A, A52V, A55G, A55S, A55T, P56S, V57L, V57M, Q61E, A63S, E64K, E64D, V65M, A67K, A67E, N68Q, N68R, T69N, V70I, K75I, S78G, S78A, S78V, V79I, Q89E, K91H, K91Q, P92S, Y95F, K99E, V102T, I102V, A103T, A104K, N105S, N105K, K106T, P107S, I119V, G125A, I132V, D140G, D140N, P142A, T144I, T144A, R145K, Q149D, Q149E, Q149A, R159S, M160L, D177E, E178N, E178D, V179I, T180K, T180A, N182D, N182P, A183T, S184A, R187N, N191D, N191H, N191Y, N191T, T200A, A207T, A207S, K218Q, P223R, P223N, P223G, P223S, Y224F, Q226R, A227T, M228I, T230K, T230E, L233F, P235R, K237R, K237A, D239N, D239H, N244Q, N244D, A248S, A248E, E249D, E250A, A251T, A251Q, H252Y, W255R, A262T, V263I, V263T, R264H, M269T, A272T, A272G, Q273E, T279S, V281I, V281A, Q282E, Q282K, M284I, A285S, N286S, V288L, M289K, A290V, A293N, A293V, A293K, E295D, E295A, N296A, N296K, N296R, N296S, V297L, A298E, A298Q, A298S, A298P, D299N, D299A, A300N, A300S, A300T, S301T, K303G, K303R, Q304K, Q304E, I306L, A307S, A307T, I316V, S318A, E333D, A334G, A334S, N335Q, N335K, V337I, V338I, E339D, E339G, S343N, V345I, P349A, P351L, V352A, A353R, A353V, N356S, A359V, K363E, K363N, T370M, Q387E, Q387N, I388L, I388V, K396T, S397N, H409Q, H409R, E412S, E412N, E412D, E412K |
| 6 | CMH | *Enterobacter spp* (B: chromosome,  B: plasmid, O: chromosome) | 16 | E25K, G123S, D144N, A149V, P217S, H218N, P264S, Q265P, A300V, D378E, A379T |
| 7 | MIR | *Enterobacter spp* (B: chromosome,  B: plasmid, O: chromosome) | 40 | A15T, S17A, K26T, M40I, N41K, I103V, A104S, A110T, R125H, D128E, T148N, V198I, K203N, D205N, A214T, E225D, K248Q, V254L, I255M, A256V, Q264H, A265D, P266S, K269R, Q270K, R281C, G305E, V318A, A319R, P327L, K329N |
| 8 | PDC | *P. aeruginosa* (B: chromosome,  B: plasmid, O: chromosome) | 397 | D3H, T4A, R5T, R5G, F6I, P7L, P7S, C8N, I12L, L18F, F19L, T21A, P23S, I25F, G27D, G27T, D32E, A36T, A55T, S59T, L71V, G77A, R79Q, R79K, P82L, T94S, A97V, A105T, R114S, Q117L, Q117R, A136G, F147L, S150V, Q155R, Q155P, A156V, A156T, A170T, Q174H, L176Q, L176R, G183D, E198K, L200S, L200I, Q203R, Q204R, V205L, Q213H, H215F, L216I, D217R, E220T, A222Q, L223Q, A224G, Q225L, K232N, R235H, V239A, E247G, G248A, V251L, T253S, D263E, D272E, R273K, P274T, P274Q, P274L, S306A, S306T, S306D, L310M, T316A, P322A, R324K, I325V, A330V, E335D, V353L, V356I, L361V, I365V, G391P, G391A, K392N, K396V, K396A |
| 9 | PIB | *P. aeruginosa* (O: chromosome) | 3 | Not found |
