# Supplementary material for: AmpC β-lactamases: A key to antibiotic resistance in ESKAPE pathogens
Source: Cell Surf. 2025 Sep 22;14:100154. doi: 10.1016/j.tcsw.2025.100154 (PMC12528871; doi:10.1016/j.tcsw.2025.100154)
Supplement: Supplementary Table 4 — Variant Analysis of Conserved Motifs in Class C β-Lactamases Using PROVEAN. [file mmc9.docx]

**Table S4:** Variant Analysis of Conserved Motifs in Class C β-Lactamases Using PROVEAN

| **Motif** | **Variant** | **PROVEAN score** | **Prediction (cutoff= -2.5)** |
| --- | --- | --- | --- |
| **ACT** |  |  |  |
| **S** | S84V | -5.792 | Deleterious |
| **X** | I85A | -3.228 | Deleterious |
| **X** | S86V | -5.793 | Deleterious |
| **K** | K87I | -7.723 | Deleterious |
| **Y** | Y170G | -9.551 | Deleterious |
| **X** | A171L | -3.393 | Deleterious |
| **N** | N172P | -7.605 | Deleterious |
| **K** | K335N | -4.664 | Deleterious |
| **T** | T336M | -5.598 | Deleterious |
| **G** | G337V | -8.397 | Deleterious |
| **PDC** |  |  |  |
| **K** | K342A | -6 | Deleterious |
| **T** | T343Y | -7 | Deleterious |
| **G** | G344V | -8.939 | Deleterious |
| **ADC** |  |  |  |
| **S** | S88D | -3.989 | Deleterious |
|  | S88G | -3.989 | Deleterious |
| **X** | V89E | -5.984 | Deleterious |
|  | V89N | -6.723 | Deleterious |
| **X** | S90L | -5.984 | Deleterious |
|  | S90V | -5.984 | Deleterious |
| **K** | K91A | -5.984 | Deleterious |
|  | K91Q | -3.989 | Deleterious |
| **Y** | Y174F | -3.996 | Deleterious |
|  | Y174P | -9.991 | Deleterious |
| **X** | S175G | -3.984 | Deleterious |
|  | S175I | -5.978 | Deleterious |
| **N** | N176R | -5.995 | Deleterious |
|  | N176Y | -7.993 | Deleterious |
| **K** | K336H | -5.989 | Deleterious |
|  | K336M | -5.989 | Deleterious |
|  | K336V | -6.955 | Deleterious |
| **T** | T337K | -5.989 | Deleterious |
|  | T337Y | -6.987 | Deleterious |
| **G** | G338H | -7.986 | Deleterious |
|  | G338M | -8.984 | Deleterious |
|  | G338T | -7.986 | Deleterious |
| **CMY** |  |  |  |
| **S** | S84V | -5.792 | Deleterious |
| **X** | V85S | -5.626 | Deleterious |
| **X** | S86K | -3.862 | Deleterious |
| **K** | K87T | -5.792 | Deleterious |
| **Y** | Y170A | -8.597 | Deleterious |
| **N** | N172S | -4.753 | Deleterious |
| **K** | K335T | -5.596 | Deleterious |
| **T** | T336G | -6.531 | Deleterious |
| **G** | G337S | -5.598 | Deleterious |
