# Supplementary material for: AmpC β-lactamases: A key to antibiotic resistance in ESKAPE pathogens
Source: Cell Surf. 2025 Sep 22;14:100154. doi: 10.1016/j.tcsw.2025.100154 (PMC12528871; doi:10.1016/j.tcsw.2025.100154)

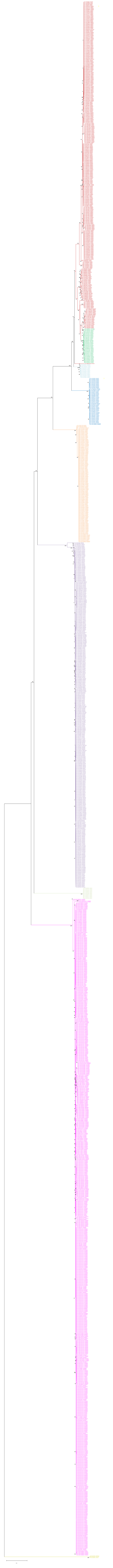

Supplement: Supplementary Figure S2 — Neighbor-Joining Phylogram of 1790 AmpC Beta-Lactamase Sequences. This tree illustrates the detailed evolutionary relationships and diversity among all 1790 AmpC sequences from Acinetobacter baumannii, Enterobacter spp., Klebsiella pneumoniae, and Pseudomonas aeruginosa. The branches are color-coded according to the pathogen, revealing distinct clades and bootstrap values. [file mmc2.pdf]
